# Global profiling reveals common and distinct N6-methyladenosine (m6A) regulation of innate immune responses during bacterial and viral infections

**DOI:** 10.1038/s41419-022-04681-4

**Published:** 2022-03-14

**Authors:** Jian Feng, Teng Zhang, Océane Sorel, Wen Meng, Xinquan Zhang, Zhao Lai, Weiming Yuan, Yidong Chen, Yufei Huang, Shou-Jiang Gao

**Affiliations:** 1 Cancer Virology Program, UPMC Hillman Cancer Center, University of Pittsburgh, Pittsburgh, PA USA; 2 Department of Microbiology and Molecular Genetics, University of Pittsburgh, Pittsburgh, PA USA; 3 Greehey Children’s Cancer Research Institute, University of Texas Health Science Center at San Antonio, San Antonio, TX USA; 4 Department of Molecular Microbiology and Immunology, Keck School of Medicine, University of Southern California, Los Angeles, CA USA; 5 Department of Molecular Medicine, University of Texas Health San Antonio, San Antonio, TX USA; 6 Department of Populational Health Science, University of Texas Health San Antonio, San Antonio, TX USA; 7 Department of Medicine, University of Pittsburgh, Pittsburgh, PA USA

**Keywords:** RNA modification, Bacterial infection, Viral infection, Infection

## Abstract

*N*^6^-methyladenosine (m^6^A) is a dynamic post-transcriptional RNA modification influencing all aspects of mRNA biology. While m^6^A modifications during numerous viral infections have been described, the role of m^6^A in innate immune response remains unclear. Here, we examined cellular m^6^A epitranscriptomes during infections of *Pseudomonas aeruginosa* and herpes simplex virus type 1 (HSV-1), and lipopolysaccharide (LPS) stimulation to identify m^6^A-regulated innate immune response genes. We showed that a significant portion of cellular genes including many innate immune response genes underwent m^6^A modifications in 5'UTR and 3'UTR. We identified common and distinct m^6^A-modified genes under different stimulating conditions. Significantly, the expression of a subset of innate immune response genes was positively correlated with m^6^A level. Importantly, we identified genes that had significant enrichments of m^6^A peaks during *P. aeruginosa* infection following knockdown of m^6^A “eraser” ALKBH5, confirming the regulation of these genes by m^6^A and ALKBH5. Among them, we confirmed the association of m^6^A modification with gene expression in immune response genes TNFAIP3, IFIT1, IFIT2 and IFIH1. Taken together, our results revealed the vital role of m^6^A in regulating innate immunity against bacterial and viral infections. These works also provided rich resources for the scientific community.

## Introduction

Innate immunity, the first line of defense against infection, relies on pattern recognition receptors (PRRs) to sense pathogens by recognizing pathogen-associated molecular patterns (PAMPs) [1, 2]. PRRs react with specific PAMPs to activate different signaling pathways, leading to distinct anti-pathogen responses [3] including interferon (IFN)-stimulated genes (ISGs) [4].

Discovered in 1970s [5], *N*^6^-methyladenosine (m^6^A) methylation is the most abundant internal modification occurring in RNAs of most eukaryotes [6]. Regulation by m^6^A is dynamic and reversible, which is executed by methyltransferases (“writers”) [7, 8], demethylases (“erasers”) including alkB homolog 5 (ALKBH5) and fat mass and obesity associated (FTO) [9, 10], m^6^A-binding proteins (“readers”) [11–13], and proteins that preferentially bind to mRNAs in the absence of m^6^A (“anti-readers”) [14, 15]. m^6^A modification on RNA not only participates in RNA metabolism but is also involved in diverse biological functions [9, 10]. Almost every aspect of RNA biology is affected by m^6^A modification, including structure, maturation, stability, splicing, export, translation and decay [16]. The advent of m^6^A sequencing (m^6^A-seq) has given rise to the field of epitranscriptomics [17].

The interaction between virus and host is regulated by m^6^A [18]. m^6^A modifications are present in genomes of RNA viruses and transcripts of DNA viruses, which regulate viral gene expression and replication [19–22]. Furthermore, m^6^A epitranscriptomes in host cells are altered upon viral infections resulting in either enhanced infection or host resistance [23].

m^6^A regulates innate immunity upon viral infection. During vesicular stomatitis virus infection, RNA helicase DDX46 recruits ALKBH5 to erase m^6^A from MAVS, TRAF3 and TRAF6 transcripts [24], increasing their nuclear retention and resulting in severe attenuation of antiviral type I IFN response [24]. During herpes simplex virus-1 (HSV-1) infection, hnRNPA2B1 blocks FTO access to cGAS, IFI16 and STING transcripts, thereby increasing methylation level and nuclear export, resulting in a stronger antiviral response [25]. Upon viral infection, host cells impair ALKBH5 enzymatic activity to increase m^6^A methylation on α-ketoglutarate dehydrogenase (OGDH) mRNA resulting in reduced mRNA stability, protein expression, and decreased production of metabolite itaconate required for viral replication [26]. Consequently, ALKBH5-deficient mice display an innate immunity-independent resistance to viral challenge [26].

In contrast to viral infection, there is few study on the role of m^6^A during bacterial infection. In this study, we systematically compared cellular m^6^A epitranscriptomes during bacterial and viral infections, and identified common and unique m^6^A-regulated innate immune response genes, providing further insights into the biological roles of m^6^A modifications.

## Results

### m^6^A epitranscriptome in bacterial infection

Macrophages are robust effector cells of innate immunity and have a central role in host defense against bacterial infection [27]. We infected mouse macrophage RAW264.7 cells with Gram-negative bacteria *P. aeruginosa* for 0, 2, 4 and 6 h. Cluster analysis of input and m^6^A-immunoprecipitation (m^6^A-IP) samples from two independent experiments showed grouping by time points, indicating experimental consistency (Supplementary Fig. 1). Analysis with ExomePeak [28] revealed >3000 upregulated or downregulated genes at any time points (Supplementary Fig. 2A). However, there were substantially fewer differential m^6^A methylated genes (DMs) with 524, 449 and 410 hypermethylated genes, and 1159, 1191 and 885 hypomethylated genes at 2, 4 and 6 h, respectively (Supplementary Fig. 2B), suggesting regulation of only some differentially expressed genes by m^6^A, and that there were about 2-fold more hypomethylated than hypermethylated genes.

**Fig. 1:**
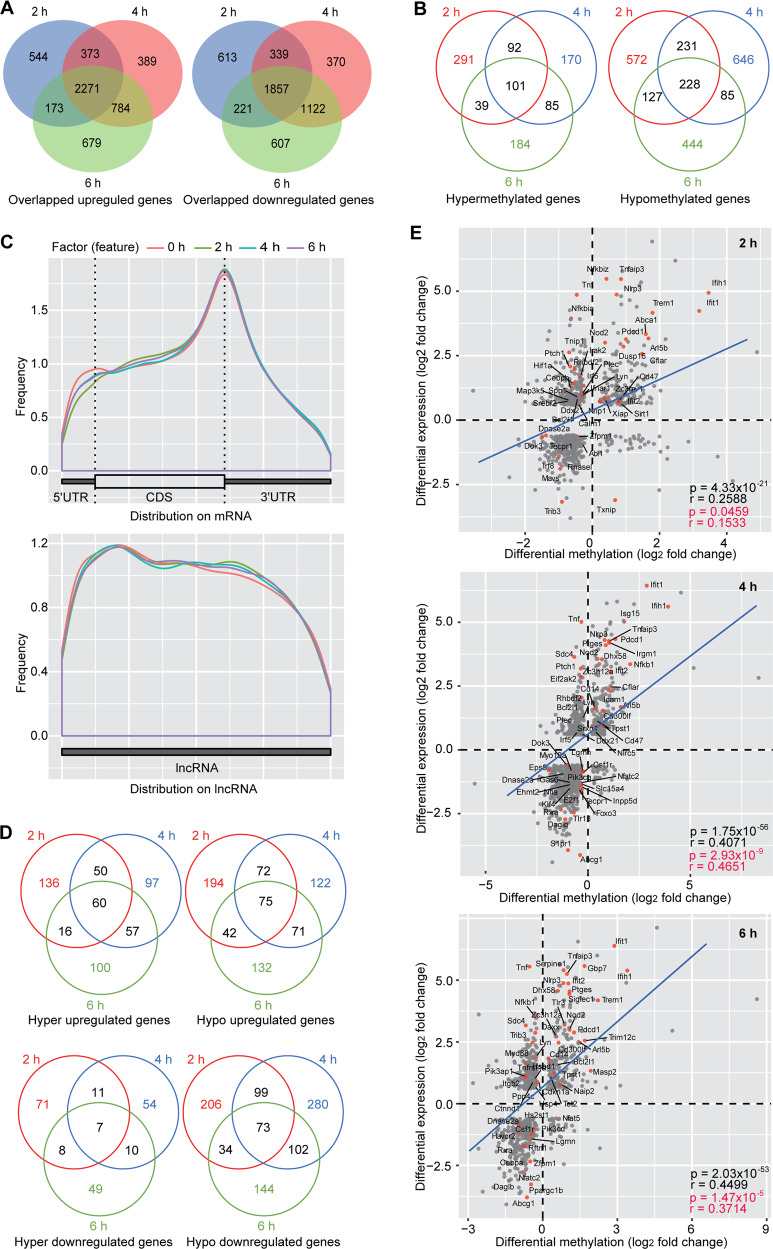
*P. aeruginosa* infection alters cellular transcriptome and m^6^A epitranscriptome. Overlapped differentially expressed (**A**) and methylated (**B**) genes following *P. aeruginosa* infection for 2, 4 and 6 h. **C** Overall m^6^A distribution on cellular mRNA and lncRNA at different time points following *P. aeruginosa* infection. **D** Overlapped differentially expressed genes with hypermethylation and hypomethylation following *P. aeruginosa* infection for 2, 4 and 6 h. **E** Correlation of mRNA expression level and m^6^A level for differentially expressed and methylated genes at 2, 4 and 6 h *P. aeruginosa* post-infection. The regression lines are marked in “blue”. Innate immune related genes are marked with “red dots”.

We identified 2271 (43.56%) and 1857 (36.21%) upregulated and downregulated genes at all time points, respectively, indicating consistency of most altered genes (Fig. 1A). However, only 101 (10.50%) and 228 (9.78%) genes were consistently hypermethylated and hypomethylated, respectively, at all the time points, indicating more dynamic m^6^A alterations during bacterial infection (Fig. 1B).

Analysis of m^6^A transcript distribution revealed more m^6^A losses at 5'UTR and more m^6^A gains at 3'UTR on mRNA following *P. aeruginosa* infection (Fig. 1C). There were also more m^6^A losses at 5' end and more m^6^A gains from the middle region to 3' end on lncRNA (Fig. 1C).

To identify m^6^A-regulated differentially expressed genes (DEs), we overlapped DEs with differentially methylated genes (DMs). There were 60 and 75 upregulated genes that were differentially hypermethylated and hypomethylated, respectively, and 7 and 73 downregulated genes that were differentially hypermethylated and hypomethylated, respectively, at all the time points (Fig. 1D). Clearly, there were fewer hypermethylated downregulated genes than all other groups.

Gene Set Enrichment Analysis revealed that among DEs, numerous immune response pathways including Immune system and Cytokine signaling in the immune system were highly enriched, and that these pathways were upregulated following bacterial infection (Supplementary Fig. 3A and Supplementary Table 1). Among DMs, these two pathways were also enriched besides several other immune response pathways (Supplementary Fig. 3B and Supplementary Table 2). Among DEs that were also DMs (DEDMs), several immune response pathways including numerous Toll-like receptor (TLR) pathways were enriched (Supplementary Fig. 3C and Supplementary Table 3). Particularly, the major innate immune response TLR4 pathway in bacterial infection was enriched.

Examination of DEDMs revealed high degree of positive correlation at all time points between levels of DEs and levels of differential methylation (*r* = 0.2588, *p* = 4.33E−21 at 2 h; *r* = 0.4071, *p* = 1.75E−56 at 4 h; and *r* = 0.4499, *p* = 2.03E−53 at 6 h in Fig. 1E). This trend was also present for immune response genes (*r* = 0.1533, *p* = 0.0459 at 2 h; *r* = 0.4651, *p* = 2.93E−09 at 4 h; *r* = 0.3714, *p* = 1.47E−05 at 6 h in Fig. 1E). These results indicated that m^6^A might have a critical role in innate immune response to bacterial infection.

### m^6^A epitranscriptome following LPS treatment

Since the TLR4 pathway is primarily activated by LPS in Gram-negative bacteria [29], we examined m^6^A epitranscriptome following treatment of RAW264.7 cells with LPS for 6 h, which induced maximal innate immune response.

**Fig. 2:**
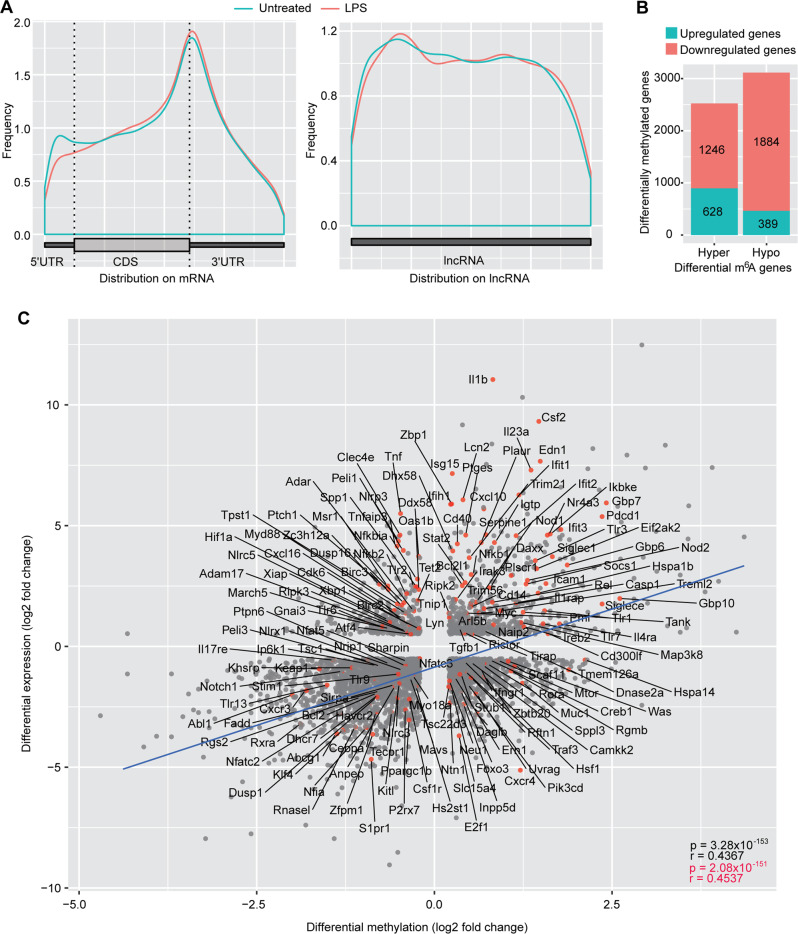
LPS treatment alters cellular transcriptome and m^6^A epitranscriptome. **A** Overall m^6^A distribution on cellular mRNA and lncRNA following LPS treatment for 6 h. **B** Number of differentially expressed genes with hypermethylation and hypomethylation following LPS treatment for 6 h. **C** Correlation of mRNA expression level and m^6^A level for differentially expressed and methylated genes following LPS treatment. The regression lines are marked in “blue”. Innate immune related genes are marked with “red dots”.

We identified 5874 upregulated and 2446 downregulated genes (Supplementary Fig. 4), and 3157 hypermethylated and 3226 hypomethylated genes (Supplementary Fig. 4). Similar to bacterial infection, there were more m^6^A losses at 5'UTR and more m^6^A gains at 3'UTR on mRNA (Fig. 2A). There were also more m^6^A losses at 5' end and more m^6^A gains at 3' end on lncRNA (Fig. 2A). Among the upregulated genes, there were more hypermethylated than hypomethylated genes (628 vs 329). In contrast, among the downregulated genes, there were fewer hypermethylated than hypomethylated genes (1246 vs 1884) (Fig. 2B).

As expected, among DEs, numerous immune response pathways were enriched, and upregulated by LPS treatment (Supplementary Fig. 5A and Supplementary Table 4). Among DMs, numerous TLR4-related pathways were highly enriched (Supplementary Fig. 5B and Supplementary Table 5). Among DEDMs, TLR4-related pathways remained enriched (Supplementary Fig. 5C and Supplementary Table 6), suggesting they were regulated by m^6^A. We also observed correlation between levels of DEs and levels of differential methylation (*r* = 0.4367, *p* = 3.28E−153 in Fig. 2C), including immune response genes (*r* = 0.4537, *p* = 2.08E−151 in Fig. 2C).

### HSV-1 infection alters cellular m^6^A epitranscriptome

To identify cellular genes regulated by m^6^A during viral infection, we used a common human pathogen HSV-1 to infect the highly permissive HeLa cells at 5 multiplicities of infection (MOIs) for 5 and 8 h. Over 4000 DEs were identified at both time points (Supplementary Fig. 6A). However, there were fewer DMs with 1339 and 2367 hypermethylated genes, and 729 and 1412 hypomethylated genes at 5 and 8 h, respectively (Supplementary Fig. 6B), suggesting that the expression of close to 50% of genes were directly or indirectly regulated by m^6^A, and there were more hypomethylated than hypermethylated genes.

**Fig. 3:**
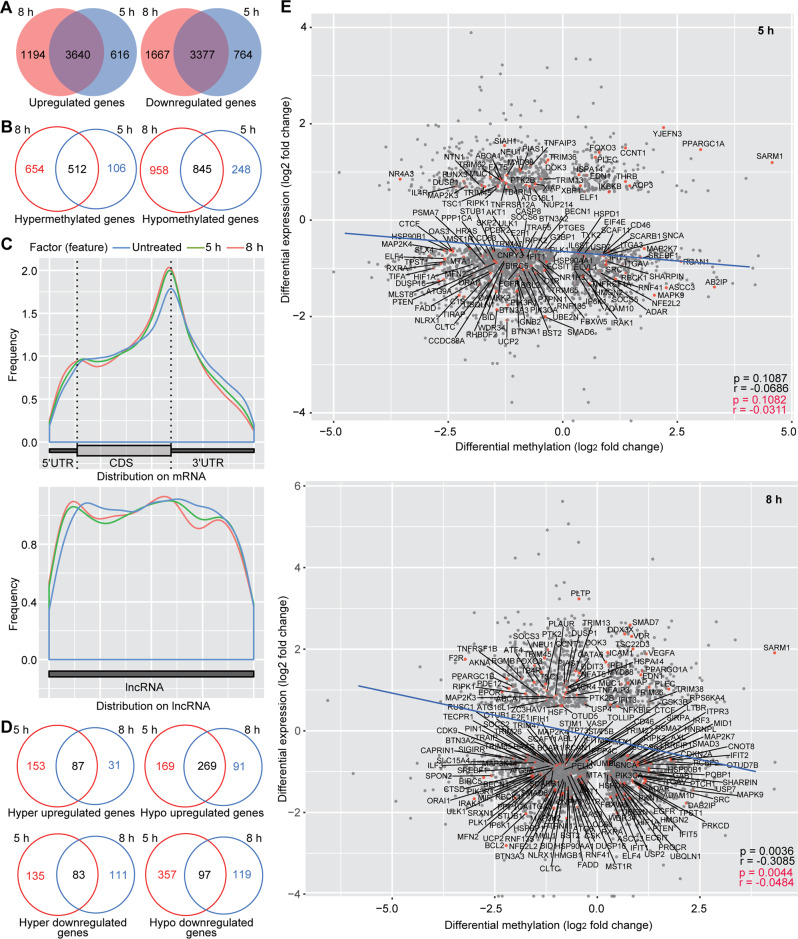
HSV-1 infection alters cellular transcriptome and m^6^A epitranscriptome. Overlapped differentially expressed (**A**) and methylated (**B**) genes following HSV-1 infection for 5 and 8 h. **C** Overall m^6^A distribution on cellular mRNA and lncRNA at different time points following HSV-1 infection. **D** Overlapped differentially expressed genes with hypermethylation and hypomethylation following HSV-1 infection for 5 and 8 h. **E** Correlation of mRNA expression level and m^6^A level for differentially expressed and methylated genes at 5 and 8 h HSV-1 post-infection. The regression lines are marked in “blue”. Innate immune related genes are marked with “red dots”.

We identified 3640 (66.79%) upregulated and 3377 (58.14%) downregulated genes at both 5 and 8 h, respectively, indicating persistent alterations of a large portion of these genes (Fig. 3A). Unlike bacterial infection, as many as 512 (40.25%) and 845 (41.20%) genes were consistently hypermethylated and hypomethylated at 5 and 8 h, respectively, indicating most of the genes were persistently regulated by m^6^A (Fig. 3B). Unlike bacterial infection, we observed m^6^A gains at both 5'UTR and 3'UTR on mRNA (Fig. 3C). m^6^A gains were also observed at 5' end on lncRNA; however, there were more m^6^A losses at 3' end on lncRNA (Fig. 3C). Among the upregulated genes, there were more hypomethylated than hypermethylated genes (269 vs 87) (Fig. 3D). However, among the downregulated genes, there were similar hypomethylated as hypermethylated genes (83 *vs* 97) (Fig. 3D). Interestingly, we did not observe enrichment of any immune response pathways among DEs (Supplementary Fig. 7A and Supplementary Table 7), DMs (Supplementary Fig. 7B and Supplementary Table 8), or DEDMs (Supplementary Fig. 7C and Supplementary Table 9) albeit numerous immune response DEs and DMs were detected. Furthermore, we only observed a negative correlation between levels of DEs and levels of differential methylation at 8 h but not 5 h (*r* = −0.0686, *p* = 0.1087 at 5 h; *r* = −0.3085, *p* = 0.0036 at 8 h in Fig. 3E), including immune response genes (*r* = −0.0311, *p* = 0.1082 at 5 h; *r* = −0.0484, *p* = 0.0044 at 8 h in Fig. 3E). These results suggest regulation of innate immune response by m^6^A at a later time point. It is known that HSV-1 encodes numerous inhibitory proteins of innate immunity [30], which might affect m^6^A’s role during HSV-1 infection.

### Common DEDMs during bacterial and viral infections

**Fig. 4:**
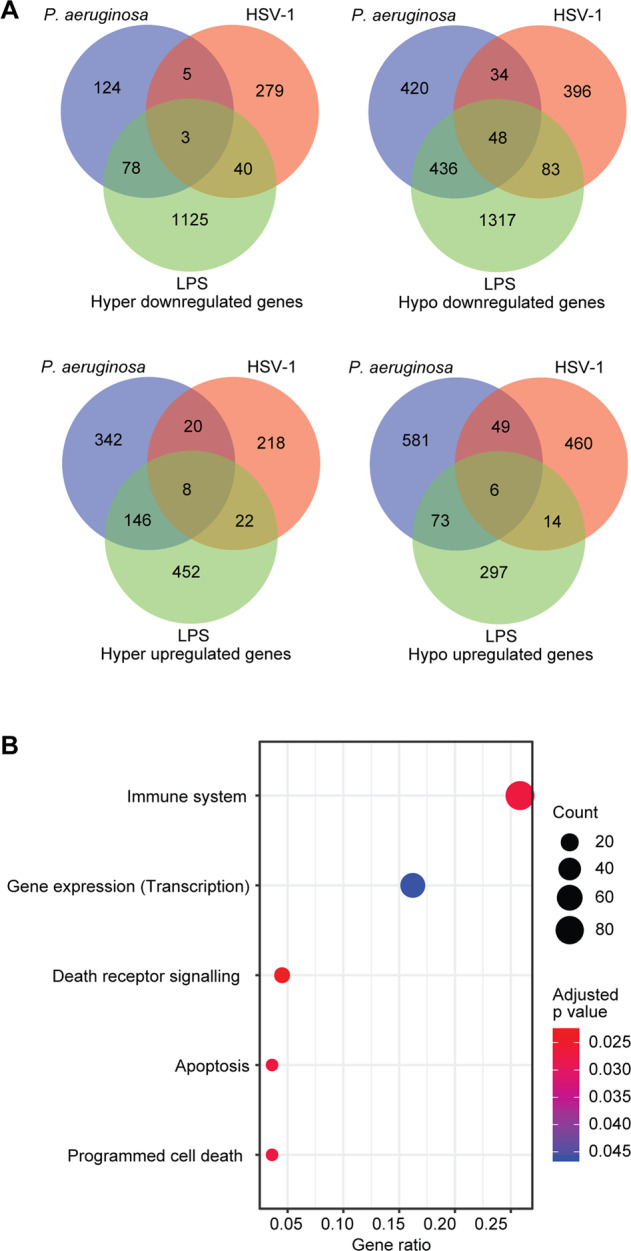
Common and distinct differentially expressed and methylation genes following *P. aeruginosa* and HSV-1 infections, and LPS treatment. **A** Common and distinct differentially expressed and methylated genes following *P. aeruginosa* and HSV-1 infections as well as LPS treatment. **B** Enriched pathways for common differentially expressed and methylated genes following *P. aeruginosa* and HSV-1 infections, or LPS treatment and HSV-1 infection.

We further identified common genes regulated by m^6^A during bacterial and viral infections, and LPS treatment. As expected, there were clearly more common DEDMs between *P. aeruginosa* infection and LPS treatment than between *P. aeruginosa* and HSV-1 infections, or LPS treatment and HSV-1 infection (Fig. 4A, Supplementary Table 10). This could be due to the distinct responses to different stimulations. However, the use of different cells might also contribute to the variations. When considering DEDMs at any time points during bacterial and viral infections, and LPS treatment, we identified 65 common genes (Fig. 4A, Supplementary Table 10). However, we failed to find any enriched pathways for these genes, which could be due to the small gene number. When we analyzed overlapped DEDMs between two conditions including bacterial and HSV-1 infections, and LPS treatment and HSV-1 infection, we obtained 5 enriched pathways including the Immune system pathway (Fig. 4B). Hence, m^6^A might regulate distinct sets of immune response genes between viral and bacterial infections, or between viral infection and LPS treatment.

### ALKBH5 knockdown enhances innate immune responses during bacterial infection

**Fig. 5:**
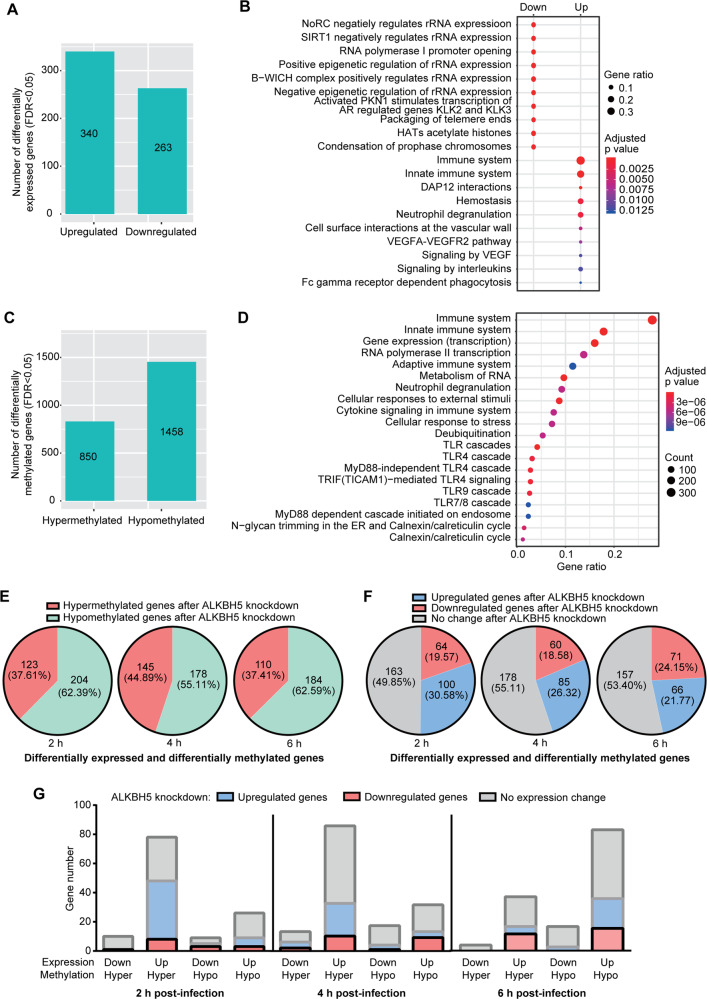
Alterations of cellular transcriptome and m^6^A epitranscriptome during *P. aeruginosa* infection following ALKBH5 knockdown. **A** Numbers of differentially expressed genes during *P. aeruginosa* infection following ALKBH5 knockdown. **B** Enriched pathways of differentially expressed genes during *P. aeruginosa* infection following ALKBH5 knockdown. **C** Numbers of differentially methylated genes during *P. aeruginosa* infection following ALKBH5 knockdown. **D** Enriched pathways of differentially methylated genes during *P. aeruginosa* infection following ALKBH5 knockdown. **E** Hypermethylated and hypomethylated genes following ALKBH5 knockdown among common differentially expressed and methylated genes between *P. aeruginosa* and HSV-1 infections, or *P. aeruginosa* infection and LPS treatment displayed by 2, 4, and 6 h *P. aeruginosa* post-infection. **F** Upregulated and downregulated genes following ALKBH5 knockdown among common differentially expressed and methylated genes between *P. aeruginosa* and HSV-1 infections, or *P. aeruginosa* infection and LPS treatment displayed by 2, 4, and 6 h *P. aeruginosa* post-infection. **G** Upregulated and downregulated genes following ALKBH5 knockdown among common differentially expressed and methylated genes between *P. aeruginosa* and HSV-1 infections, or *P. aeruginosa* infection and LPS treatment further displayed by differentially expressed and methylated genes at 2, 4, and 6 h *P. aeruginosa* post-infection.

To identify genes directly regulated by m^6^A, we performed knockdown of m^6^A writers or erasers. Unfortunately, we were unable to achieve efficient knockdown of METTL3 and METTL14 without causing severe cellular toxicity. However, we achieved efficient ALKBH5 knockdown (Supplementary Fig. 8A) in RAW264.7 cells, which were infected with *P. aeruginosa* for 6 h and subjected to m^6^A profiling (Supplementary Fig. 8B). We identified 340 upregulated and 263 downregulated genes (Fig. 5A and Supplementary Fig. 9A). Among them, numerous immune response pathways were enriched (Fig. 5B), indicating ALKBH5 knockdown enhances immune response, most likely through regulating m^6^A levels on these transcripts. While we identified 850 hypermethylated genes, there were also 1458 hypomethylated genes, which might be regulated by other indirect mechanisms (Fig. 5C and Supplementary Fig. 9B). The fact that there were significantly more DMs than DEs suggested that not all m^6^A alterations were translated into DEs. Importantly, we found that most enriched pathways of DMs were related to immune responses (Fig. 5D), further confirming the regulation of immune response by ALKBH5 during bacterial infection.

### ALKBH5 regulates DEDMs during bacterial and viral infections

Next, we examined DEDMs following ALKBH5 knockdown. Because there were few overlapped genes between viral infection and bacterial infection or LPS treatment, we included genes mutually regulated by bacterial infection and LPS treatment, and those mutually regulated by bacterial and viral infections. We found that, at all time points of bacterial infection, >50% of DEDMs had m^6^A hypomethylation following ALKBH5 knockdown (Fig. 5E), suggesting an indirect role of ALKBH5. Nevertheless, 37.61%, 44.89% and 37.41% of the DEDMs were hypermethylated at 2, 4 and 6 h post-infection, respectively, following ALKBH5 knockdown (Fig. 5E). These genes were likely directly regulated by ALKBH5 and m^6^A. Among DEDMs during viral and bacterial infections, or bacterial infection and LPS treatment, ≥50% of them did not have any expression changes following ALKBH5 knockdown (Fig. 5F). However, 30.58%, 26.32% and 21.77% of these genes were upregulated while 19.57%, 18.58% and 24.15% of them were downregulated following ALKBH5 knockdown when presented by 2, 4 and 6 h following bacterial infection, respectively (Fig. 5F). As expected, most genes impacted by ALKBH5 knockdown were those upregulated during viral and bacterial infections, or bacterial infection and LPS treatment (Fig. 5G). A large portion of the upregulated and hypermethylated genes at 2 and 4 h were further upregulated following ALKBH5 knockdown (Fig. 5G), suggesting an important role of m^6^A hypermethylation for inducing these genes during bacterial and viral infections. Nevertheless, a consistent fraction of the upregulated and hypermethylated genes (15–35%) at all the time points during bacterial infection were indeed downregulated following ALKBH5 knockdown (Fig. 5G), suggesting a negative regulatory role of m^6^A hypermethylation for these genes. Similarly, a fraction of the upregulated and hypomethylated genes (12–24%) at all the time points were also downregulated following ALKBH5 knockdown (Fig. 5G), suggesting a role of m^6^A hypomethylation in inducing these genes. However, a subset of the upregulated and hypomethylated genes (15–25%) at all the time points were upregulated following ALKBH5 knockdown (Fig. 5G), suggesting a negative regulatory role of m^6^A hypomethylation in these genes. Of interest, upregulated and hypomethylated genes were mostly impacted by ALKBH5 knockdown at a later time point of infection (6 h) (Fig. 5G). Together, these results revealed the complex dynamics and function of m^6^A during bacterial and viral infections.

### Confirmation of m^6^A-regulated immune response genes

**Fig. 6:**
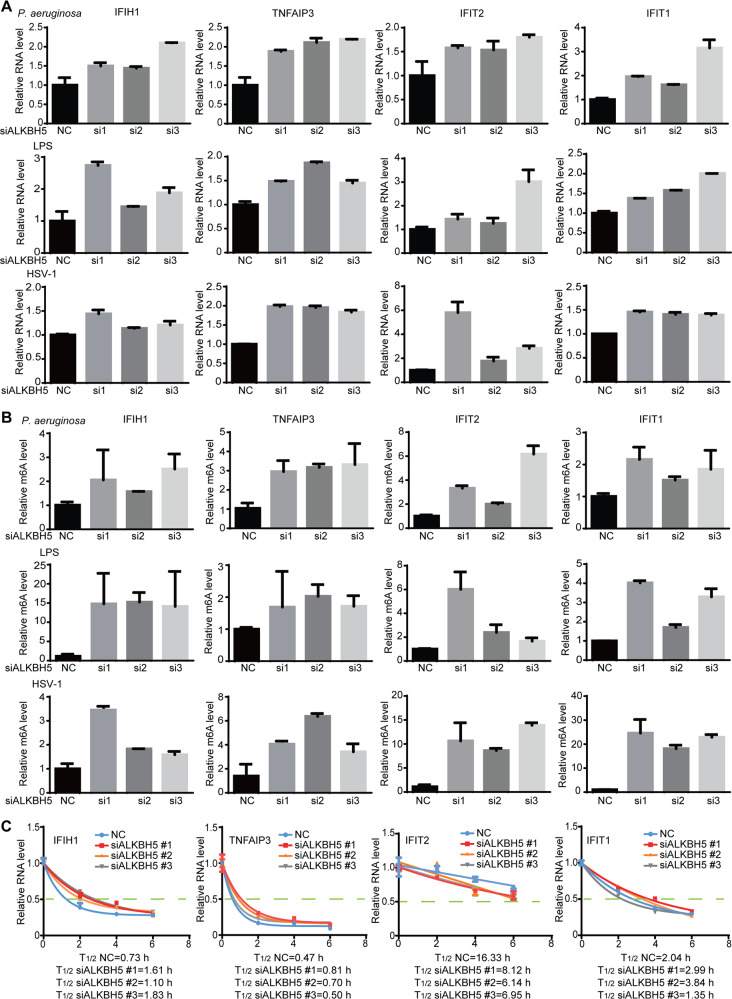
Alterations of expression and m^6^A modification of innate immune response genes TNFAIP3, IFIH1, IFIT1 and IFIT2 during *P. aeruginosa* or HSV-1 infection, or LPS treatment following ALKBH5 knockdown. **A** Alterations of expression levels of TNFAIP3, IFIH1, IFIT1 and IFIT2 genes examined by RT-qPCR after infection with *P. aeruginosa* or HSV-1, or LPS treatment for 2 h following ALKBH5 knockdown. **B** Alterations of m^6^A levels of TNFAIP3, IFIH1, IFIT1 and IFIT2 genes examined by RT-qPCR after infection with *P. aeruginosa* or HSV-1, or LPS treatment for 2 h following ALKBH5 knockdown. **C** Alterations of half-lives of TNFAIP3, IFIH1, IFIT1 and IFIT2 transcripts during *P. aeruginosa* infection following ALKBH5 knockdown. RAW264.7 cells with ALKBH5 knockdown were infected with *P. aeruginosa* or HSV-1, or treated with LPS for 2 h, in the presence of 10 μg/mL actinomycin D, and collected for RT-qPCR at the indicated time points.

Among DEDMs, those with hypermethylation and upregulation were the most common genes regulated by ALKBH5 during bacterial and viral infections (Fig. 5G). We identified a set of these immune response genes, including IFIH1, TNFAIP3, IFIT1 and IFIT2. ALKBH5 knockdown further increased the expression of these genes during *P. aeruginosa* or HSV*-*1 infection, or LPS treatment in RAW264.7 cells (Fig. 6A and Supplementary Fig. 10). We detected increased m^6^A levels of these genes following ALKBH5 knockdown (Fig. 6B). Hence, IFIH1, TNFAIP3, IFIT1 and IFIT2 were the common immune response genes regulated by m^6^A during bacterial or viral infection, or LPS treatment.

We determined the mechanism mediating m^6^A regulation of IFIH1, TNFAIP3, IFIT1 and IFIT2. Examination of transcript splicing pattern and nuclear export did not reveal any consistent alteration. However, RNA stability of IFIH1, TNFAIP3 and IFIT2 transcripts was altered following ALKBH5 knockdown with IFIH1 and TNFAIP3 transcripts having significant extended half-lives while IFIT2 transcript having a significant shorter half-life (Fig. 6C). No consistent change of half-life was observed for IFIT1 following ALKBH5 knockdown. These results confirmed the regulation of IFIH1, TNFAIP3, IFIT1 and IFIT2 by ALKBH5 and m^6^A with distinct mechanisms during both bacterial and viral infections.

## Discussion

We have analyzed m^6^A epitranscriptomes following *P. aeruginosa* and HSV-1 infections as well as LPS treatment. Firstly, we have observed more DEs than DMs in all three conditions, indicating that m^6^A only regulates a fraction of DEs. Secondly, there are almost 4 times more DMs than DEs during bacterial infection following ALKBH5 knockdown, indicating that m^6^A alterations do not always lead to differential gene expression. Thirdly, there are more altered m^6^A peaks in 5'UTR and 3'UTR than other regions of the transcripts. Hypomethylation predominantly occurs in 5'UTR and hypermethylation predominantly occurs in 3'UTR during bacterial infection and LPS treatment. Similar observations were previously reported in heatshock and invasive malignant pleomorphic adenoma [31–33]. It is known that 5'UTR regulates mRNA translation and 3'UTR mediates mRNA stability [34, 35]. Interestingly, m^6^A hypermethylation occurs in both 5'UTR and 3'UTR during HSV-1 infection. It is likely that m^6^A alterations in 5'UTR and 3'UTR might mediate important cellular responses during bacterial and viral infections.

There are large numbers of overlapping DEDMs between *P. aeruginosa* infection and LPS treatment as expected. LPS is the major PAMP of Gram-negative bacteria such as *P. aeruginosa*, inducing robust innate immune response. Indeed, both DEs and DMs are significantly enriched in pathways related to innate immune responses following *P. aeruginosa* infection and LPS treatment. Significantly, we have found that the levels of DEs is positively correlated with differential methylation levels. A similar correlation is also present for immune response genes alone. Hence, m^6^A might function to promote gene expression, particularly rapidly induced immune response genes. However, it is also possible that m^6^A might function as a feedback mechanism to control the expression of these genes. Indeed, among these genes, we have identified both upregulated and downregulated genes following ALKBH5 knockdown (Fig. 5G), suggesting the existence of both mechanisms. m^6^A is known to mediate mRNA degradation through reader proteins YTHDF2, YTHDF3 and YTHDC2 [12, 13, 36, 37]. The mechanism of m^6^A positive regulation of gene expression remains to be elucidated. Regardless of the mechanism involved, m^6^A is likely essential for regulating cellular response including immune response to bacterial infection and LPS treatment.

In contrast, we have found few overlapped DEDMs between HSV-1 infection and *P. aeruginosa* infection or LPS treatment. We have also failed to detect a correlation between levels of DEs and differential methylation levels following HSV-1 infection albeit there is a weak negative correlation for immune response genes. It is possible that HSV-1 infection might trigger a distinct cellular response including an immune response. Indeed, HSV-1 encodes numerous inhibitory genes of innate immune response [30]. It remains possible that HSV-1 genes might directly interfere with m^6^A-related complexes to upsurge m^6^A functions.

To confirm genes directly regulated by m^6^A during *P. aeruginosa* infection, we have performed ALKBH5 knockdown. Among DEs caused by ALKBH5 knockdown, over one-third are hypermethylated. However, two-third of them are hypomethylated, which are likely regulated by other unknown mechanisms. Among DEDMs caused by ALKBH5 knockdown, pathways related to immune response genes are significantly enriched during *P. aeruginosa* infection, confirming m^6^A’s important role in regulating the expression of these genes.

By focusing on overlapped DEDMs between bacterial and viral infections, or bacterial infection and LPS treatment, we have identified sets of hypermethylated and hypomethylated genes that are either upregulated or downregulated following ALKBH5 knockdown. However, we have found that over 50% of these genes are both hypermethylated and upregulated, confirming the important positive regulatory role of m^6^A in the expression of these genes. We have confirmed the hypermethylation and upregulation of numerous innate immune response genes including IFIH1, TNFAIP3, IFIT1 and IFIT2 following ALKBH5 knockdown during bacterial and viral infections as well as LPS treatment, indicating that these genes might be universally regulated by m^6^A during bacterial and viral infections. While the reduced IFIT2 transcript stability is expected following ALKBH5 knockdown and an increase in m^6^A level, we have also found increased stability of TNFAIP3 and IFIH1 transcripts. It would be interesting to further investigate how the increased m^6^A modifications and ALKBH5 knockdown stabilize these transcripts. The mechanism of m^6^A enhancement and ALKBH5 reduction of IFIT1 expression is also unclear.

In summary, we have mapped m^6^A epitranscriptomes during bacterial and viral infections, and LPS stimulation, and identified m^6^A- and ALKBH5-regulated common and distinct innate immune response genes including a set of genes with positive correlation of expression with m^6^A level. These results reveal the important roles of m^6^A and ALKBH5 in innate immune response, and provide rich resources for the scientific community.

## Materials and methods

### Bacteria, virus and cells

*P. aeruginosa* were purchased from ATCC; Herpes simplex virus type 1 (HSV-1, F strain) was a gift from Dr. David Johnson (Oregon Health Science University, Portland, OR); RAW 264.7 and HeLa cells were purchased from ATCC and cultured following the instructions provided by ATCC.

### Infection and LPS treatment

RAW264.7 cells at 4 × 10^5^ cells per mL were infected with *P. aeruginosa* at 10^7^ per mL and harvested at the indicated time point. RAW264.7 cells at 4 × 10^5^ cells per mL were treated with LPS at 1 μg/mL and harvested at 6 h post-treatment. HeLa cells were infected with HSV-1 at 5 MOIs and harvested at 5 or 8 h post-infection.

### m^6^A-immunoprecipitation (m^6^A-IP)

Isolation of m^6^A-containing fragments was performed as previously described with minor modifications [38]. Briefly, total RNA was extracted from cells using TRI Reagent (T9424-200ML, Sigma-Aldrich). The total RNA was fragmented using an RNA fragmentation kit (AM8740, ThermoFisher). Successful fragmentation of RNA with sizes close to 100 nucleotides was validated using a bioanalyzer (2100 bioanalyzer instrument, Agilent). Anti-m^6^A antibody (10 µg) (202-003, Synaptic Systems) was incubated with 30 µl slurry of Pierce Protein A Agarose beads (20365, ThermoFisher) by rotating in 250 µl PBS at 4 °C for 3 h. The beads were washed three times in cold PBS followed by one wash in an IP buffer containing 10 mM Tris-HCl at pH 7.4, 150 mM NaCl and 1% Igepal CA-630 (I8896-50ML, Sigma-Aldrich). To isolate the m^6^A-containing fragments, 120 µg of fragmented total RNA was added to the antibody-bound beads in 250 µl IP buffer supplemented with RNasin Plus RNase inhibitor (PRN2615, Promega), and the mixture was incubated at 4 °C for 2 h. The beads were washed seven times with 1 ml IP buffer and eluted with 100 µl IP buffer supplemented with 6.67 mM of m^6^A salt (M2780, Sigma-Aldrich) for 1 h at 4 °C. A second elution was carried out and the eluates were pooled together before purification with ethanol 70% precipitation.

### m^6^A-seq

Purified eluate and input samples were used to generate complementary DNA libraries for m^6^A-seq. Approximately 25 ng was used for library preparation for RNA sequencing (RNA-seq) using the KAPA Stranded RNA-Seq Kit with RiboErase (HMR) sample preparation guide (KAPA Biosystems) according to the instructions of the manufacturer with two modifications. First, the elute-frag-prime stage was done at 85 °C for 2 min to allow annealing without causing fragmentation. RNA was reverse transcribed into first-strand cDNA using reverse transcriptase and random primers. This was followed by the second strand cDNA synthesis using DNA Polymerase I and RNase H. The cDNA fragments then went through an end repair process with the addition of a single ‘A’ base followed by ligation of adapters. The products were then purified and enriched by PCR amplification for ten cycles to generate the final RNA-seq library. Secondly, beads/DNA ratio of 1:3 instead of 1:1 was used to preserve smaller fragments during the adapter ligation double beads clean-up step. cDNA libraries were quantified and pooled for cBot amplification and subsequent sequencing on an Illumina HiSeq 3000 platform 50 bp single read sequencing module. After the sequencing run, demultiplexing with Bcl2fastq2 was employed to generate a fastq file for each sample.

### m^6^A-seq data analysis

m^6^A-seq data analysis was performed as previously described [38]. Reads of the IP/Input samples were aligned to the corresponding genomes using Tophat2 Aligner v2.0.6, which implicitly calls Bowtie2 with default options. Following this, peak calling and differential m^6^A/m methylation analyses were performed using the exomePeak R/Bioconductor package, a software specifically designed for m^6^A analysis. The peak calling is based on the Przyborowski and Wilenski method, which compares the means of two Poisson distributions (*c*-test), which computes the methylation enrichment of normalized IP reads over normalized input reads (or mRNA abundance) as the test statistics. Only the loci that show significant methylation enrichment are determined as peak regions. For analysis of differential m^6^A peaks, the fold changes of methylation enrichments between two conditions are calculated and a rescaled hypergeometric test is applied to determine the significance of differential fold enrichment. The output of exomePeak includes the loci of the (differential) m^6^A/m peaks, the gene symbol of the transcripts to which peaks localize, the detection P values, false discovery rates (FDR) and methylation enrichment (for peak calling) or enrichment fold-change (for differential m6A analysis). For both m^6^A peak calling and differential peak discovery, an FDR threshold of 0.05 is used. m^6^A peaks were annotated with identifiers such as Gene Symbol and RefSeq ID as well as regional overlapping status (5'UTR/coding DNA sequence/3'UTR). Regional distribution of m^6^A was plotted using the Guitar package. Motif analysis of m^6^A peaks was performed using the MEME package.

### Clustering analysis

To investigate the methylation behavior of genes across samples, the highest fold enrichment among all peaks within a gene was considered as the methylation fold enrichment of the gene. Hierarchical clustering with Euclidean distance was used to group similarly methylated genes according to samples.

### Gene expression analysis

The expression of transcripts and isoforms and differential expression levels were calculated using cufflinks and cuffdiff, respectively, based on the input samples. To avoid underestimating the expression of genes, we used reads mapped to the cellular genome to calculate the fragments per kilobase of transcript per million mapped reads (FPKM). All the other bioinformatics analyses were performed on Matlab, R or Perl.

### siRNA knockdown

siRNA silencing was performed as previously described [38]. Briefly, RAW264.7 cells were transfected with 2.5 pmol of each siRNA per well in a 12-well plate using Lipofectamine RNAi Max (13778150, ThermoFisher) according to manufacturer’s instructions. Two days after transfection, the cells were monitored for knockdown efficiency of the target gene by RT-qPCR and Western-blotting, and infected by indicated pathogen for the indicated time. siRNAs purchased from Sigma-Aldrich are as follows:

ALKBH5 si1: SASI_Mm01_00106232;

ALKBH5 si2: SASI_Mm02_00344968;

ALKBH5 si3: SASI_Mm01_00106233; and

si Negative Control (siNC): Sigma siRNA Universal Negative Control #1 (SIC001-10NMOL).

### RNA stability assay

For RNA stability assays, actinomycin D (10 μg/ml) (A9415-2MG, Sigma-Aldrich) was added to inhibit transcription. RNA isolated at 0, 2, 4 and 6 h after actinomycin D treatment. Total RNA was isolated using Trizol, quantified by RT-qPCR.

### RT-qPCR for gene expression and MeRIP-qPCR for m^6^A-seq validation

RT-qPCR for gene expression and MeRIP-qPCR for m^6^A-seq validation was carried out as previously described [38]. Total RNA was isolated with TRI Reagent (T9424-200ML, Sigma-Aldrich) according to the manufacturer’s protocol. Reverse transcription was performed with 1 µg of total RNA using Maxima H Minus First Strand cDNA Synthesis Kit (Cat.# K1652, ThermoFisher). Quantitative PCR was done using SsoAdvanced Universal SYBR Green Supermix (1725271, Bio-Rad) and analyzed with real-time PCR machine (CFX Connect Real-Time PCR System, Bio-Rad). Relative gene expression levels were obtained by normalizing the cycle threshold (CT) values to yield 2^-ΔΔCt^ values. For validation of m^6^A-seq, eluted or input mRNA was subjected to RT-qPCR. Fold enrichment was calculated by calculating the 2^-ΔCt^ of eluate relative to the input sample. The primers used for gene expression are: 5'AAGAGCTGCTTGCACCACAC3' (forward) and 5'GTGCCACAATATGCCTGCAA3' (reverse) for IFIT1; 5'ATCTCCACCTACGGCTGACT3' (forward) and 5'TTACTGCATGGGGAAGCGAG3' (reverse) for IFIT2; 5'ACATCATGCACTGCCGGTAA3' (forward) and 5'CACAGCCATGACCATCAGGT3' (reverse) for TNFAIP3; 5'CCAGGGTCCAAATTCGCCTT3' (forward) and 5'GTACCCGGCATGGGAAGATG3' (reverse) for IFIH1; and 5'CCCTGAAGTACCCCATTGAA3' (forward) and 5'GGGGTGTTGAAGGTCTCAAA3' (reverse) for β-actin.

### Western-blotting

Protein samples were lysed in Laemmli buffer, separated by SDS-PAGE and transferred to a nitrocellulose membrane [39]. The membrane was blocked with 5% milk and then incubated with primary antibody to GAPDH (5174 s, CST) or ALKBH5 (HPA007196, Sigma) overnight at 4 °C. The membrane was washed with TBS-Tween and probed with a secondary antibody conjugated to horseradish peroxidase (HRP). After further washing with TBS-T, the blot was visualized with SuperSignal™ West Femto Maximum Sensitivity Substrate (34096, Thermo) and imaged on a ChemiDoc™ MP Imaging System (12003154, Bio-Rad).

### RNA-seq data

All RNA-seq data are available are available at GenBank accession#: GSE196272 at https://www.ncbi.nlm.nih.gov/geo/query/acc.cgi?acc=GSE196272

## Supplementary information

Supplemental Information

Original Western-blots

Checklist reporting form

## Data Availability

All data needed to evaluate the conclusions in the paper are present in the paper. Additional data related to this paper may be requested from the corresponding author.
