# Supplementary figures and images for: Global profiling reveals common and distinct N6-methyladenosine (m6A) regulation of innate immune responses during bacterial and viral infections
Source: Cell Death Dis. 2022 Mar 14;13(3):234. doi: 10.1038/s41419-022-04681-4 (PMC8921188; doi:10.1038/s41419-022-04681-4)

Supplementary Figure 8

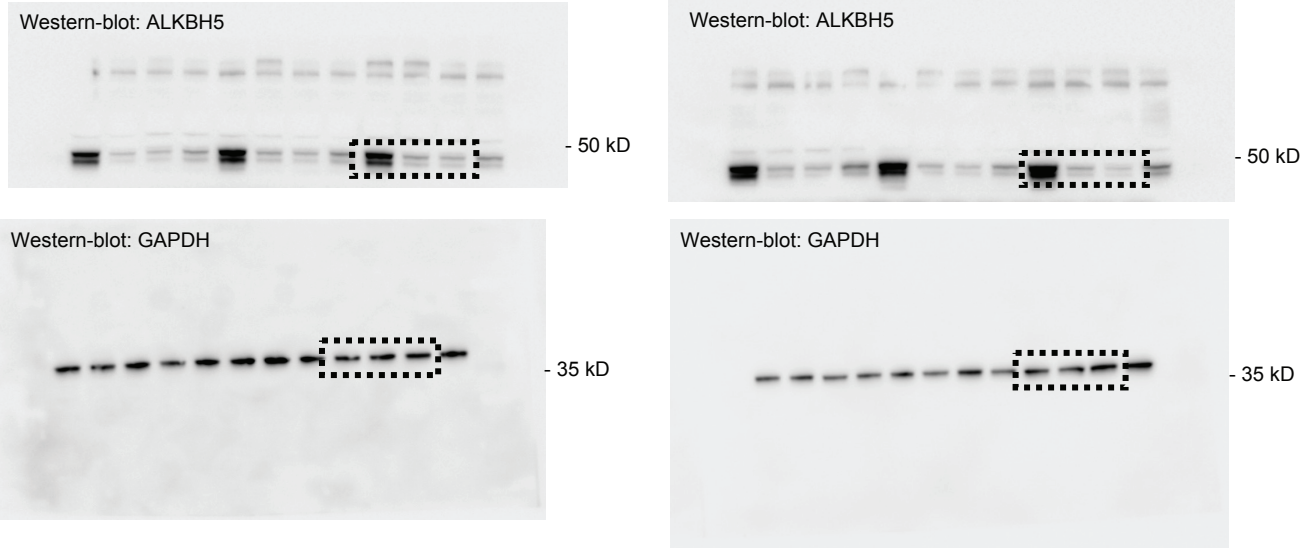

Supplementary Figure 10

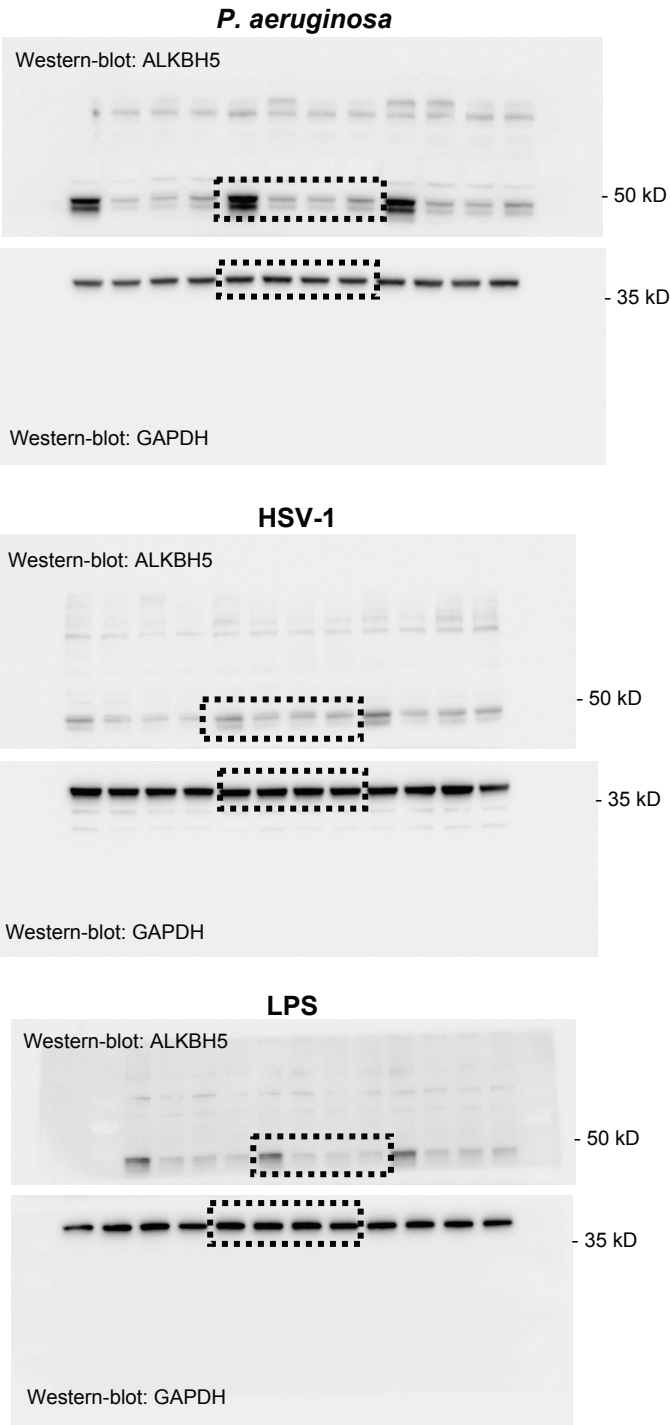

Supplement: Supplementary file 2 — Original Western-blots [file 41419_2022_4681_MOESM2_ESM.pdf]
