# Supplementary material for: Global profiling reveals common and distinct N6-methyladenosine (m6A) regulation of innate immune responses during bacterial and viral infections
Source: Cell Death Dis. 2022 Mar 14;13(3):234. doi: 10.1038/s41419-022-04681-4 (PMC8921188; doi:10.1038/s41419-022-04681-4)
Supplement: Supplementary file 1 — Supplemental Information [file 41419_2022_4681_MOESM1_ESM.pdf]

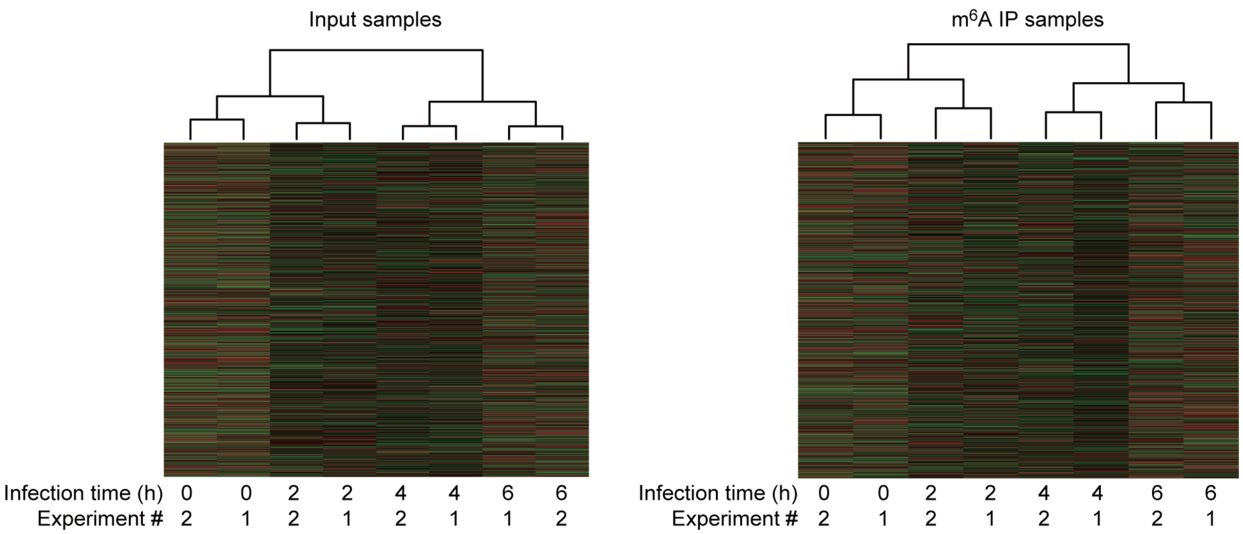

Supplementary Fig. 1 Clustering analysis of RNAseq results of input and m^{6}A-IP samples of cells infected with *P. aeruginosa* for 2, 4 and 6 h from two independent experiments.

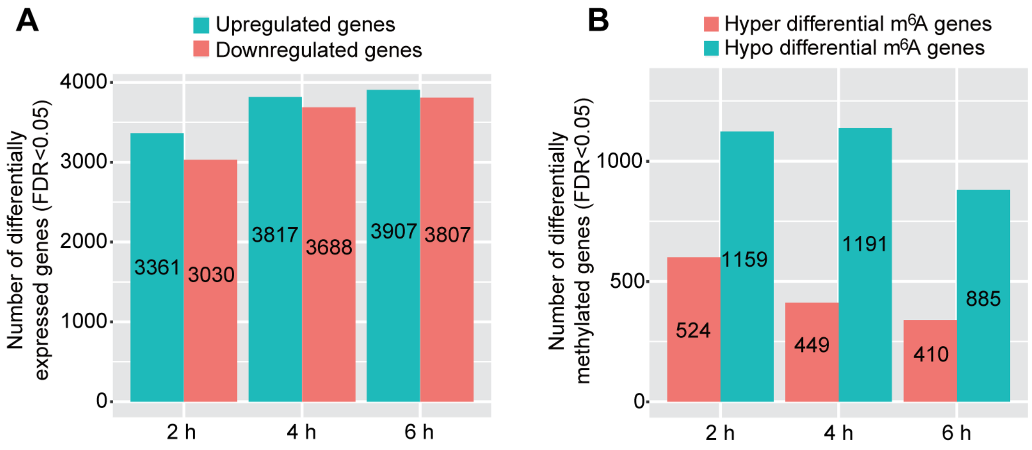

Supplementary Fig. 2 Analysis of transcriptomes and epitranscriptomes of cells infected with *P. aeruginosa.* A Numbers of upregulated and downregulated genes in cells infected with *P. aeruginosa* for 2, 4, and 6 h. **B** Numbers of hypermethylated and hypomethylated genes in cells infected with *P. aeruginosa* for 2, 4, and 6 h.

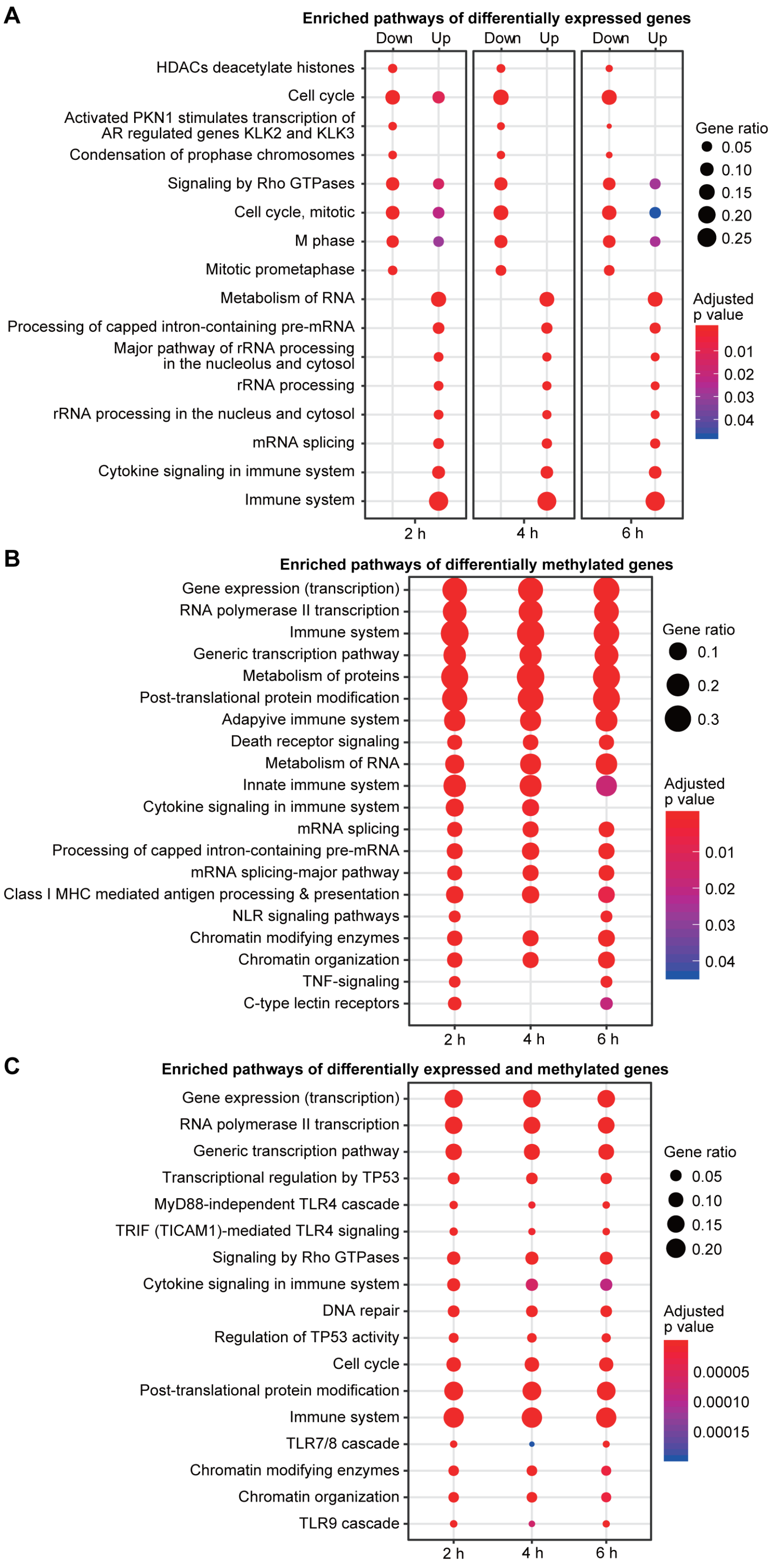

A
Enriched pathways of differentially expressed genes
Down
Up
HDACs deacetylate histones
Cell cycle
Activated PKN1 stimulates transcription of AR regulated genes KLK2 and KLK3
Condensation of prophase chromosomes
Signaling by Rho GTPases
Cell cycle, mitotic
M phase
Mitotic prometaphase
Metabolism of RNA
Processing of capped intron-containing pre-mRNA
Major pathway of rRNA processing in the nucleolus and cytosol
rRNA processing
rRNA processing in the nucleus and cytosol
mRNA splicing
Cytokine signaling in immune system
Immune system
2 h
4 h
6 h
Gene ratio
0.05
0.10
0.15
0.20
0.25
Adjusted p value
0.01
0.02
0.03
0.04
B
Enriched pathways of differentially methylated genes
Gene expression (transcription)
RNA polymerase II transcription
Immune system
Generic transcription pathway
Metabolism of proteins
Post-translational protein modification
Adapyive immune system
Death receptor signaling
Metabolism of RNA
Innate immune system
Cytokine signaling in immune system
mRNA splicing
Processing of capped intron-containing pre-mRNA
mRNA splicing-major pathway
Class I MHC mediated antigen processing & presentation
NLR signaling pathways
Chromatin modifying enzymes
Chromatin organization
TNF-signaling
C-type lectin receptors
2 h
4 h
6 h
Gene ratio
0.1
0.2
0.3
Adjusted p value
0.01
0.02
0.03
0.04
C
Enriched pathways of differentially expressed and methylated genes
Gene expression (transcription)
RNA polymerase II transcription
Generic transcription pathway
Transcriptional regulation by TP53
MyD88-independent TLR4 cascade
TRIF (TICAM1)-mediated TLR4 signaling
Signaling by Rho GTPases
Cytokine signaling in immune system
DNA repair
Regulation of TP53 activity
Cell cycle
Post-translational protein modification
Immune system
TLR7/8 cascade
Chromatin modifying enzymes
Chromatin organization
TLR9 cascade
2 h
4 h
6 h
Gene ratio
0.05
0.10
0.15
0.20
Adjusted p value
0.00005
0.00010
0.00015

Supplementary Fig. 3 Pathway enrichment analysis of differentially expressed and methylated genes following *P. aeruginosa* infection. A Pathways enriched for differentially expressed genes following *P. aeruginosa* infection for 2, 4 and 6 h. **B** Pathways enriched for differentially m^6A methylated genes following *P. aeruginosa* infection for 2, 4 and 6 h. **C** Pathways enriched for genes with both differential expression and differential m^6A methylation following *P. aeruginosa* infection for 2, 4 and 6 h.

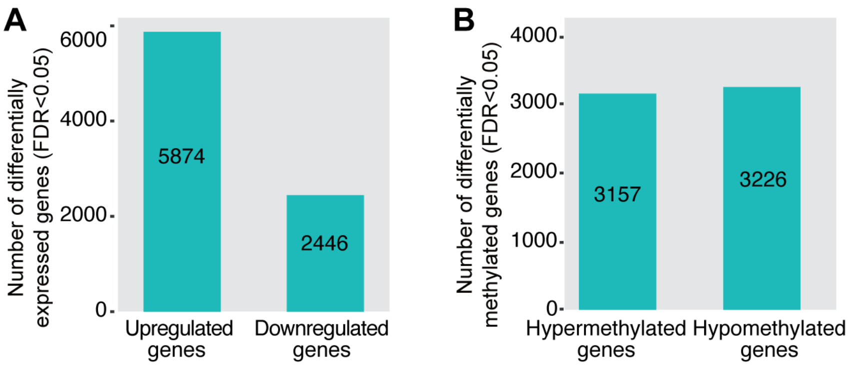

Supplementary Fig. 4 Analysis of transcriptomes and epitranscriptomes of LPS treated cells. **A** Numbers of upregulated and downregulated genes in cells treated with LPS for 6 h. **B** Numbers of hypermethylated and hypomethylated genes in cells treated with LPS for 6 h.

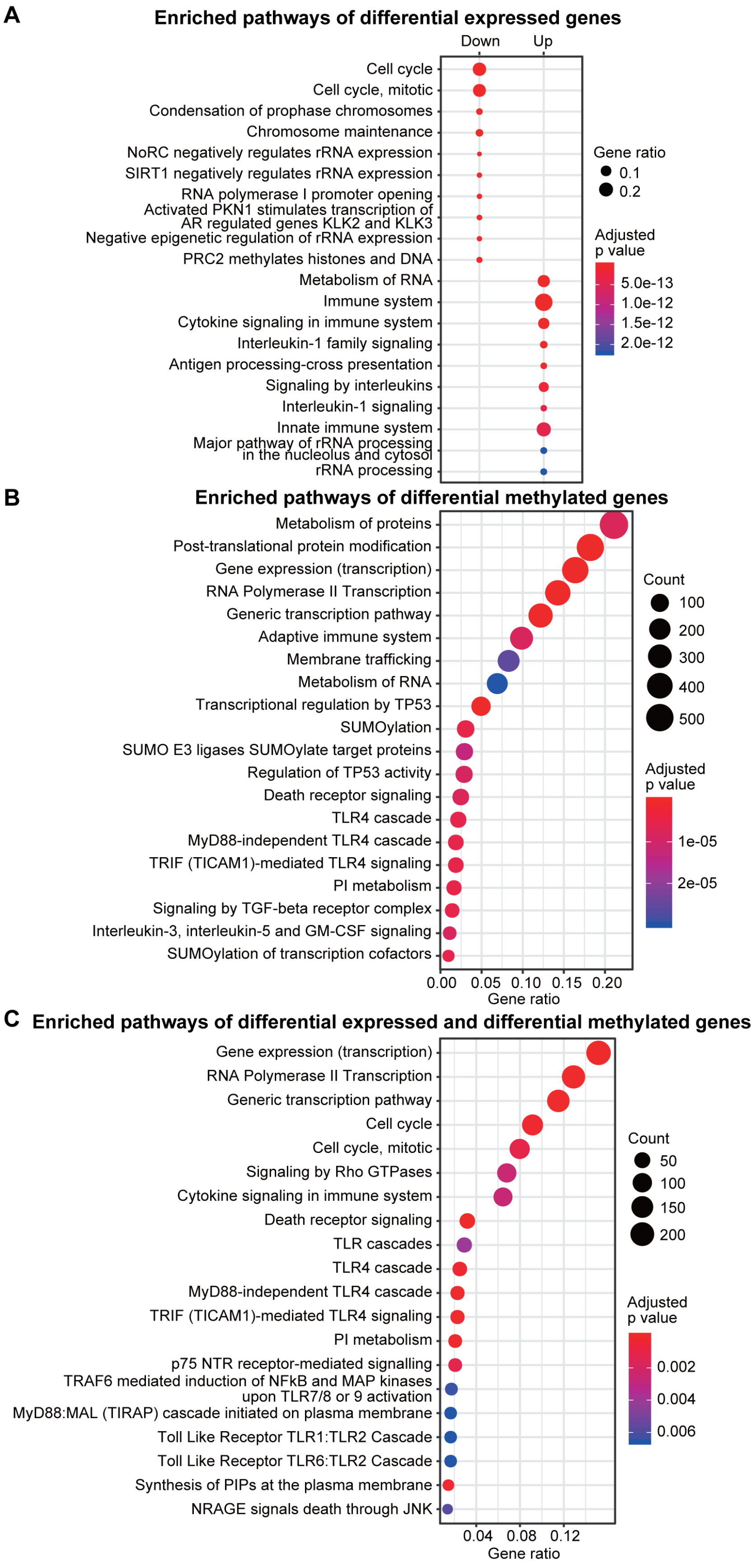

A
Enriched pathways of differential expressed genes
Down
Up
Cell cycle
Cell cycle, mitotic
Condensation of prophase chromosomes
Chromosome maintenance
NoRC negatively regulates rRNA expression
SIRT1 negatively regulates rRNA expression
RNA polymerase I promoter opening
Activated PKN1 stimulates transcription of AR regulated genes KLK2 and KLK3
Negative epigenetic regulation of rRNA expression
PRC2 methylates histones and DNA
Metabolism of RNA
Immune system
Cytokine signaling in immune system
Interleukin-1 family signaling
Antigen processing-cross presentation
Signaling by interleukins
Interleukin-1 signaling
Innate immune system
Major pathway of rRNA processing in the nucleolus and cytosol
rRNA processing
Gene ratio
0.1
0.2
Adjusted p value
5.0e-13
1.0e-12
1.5e-12
2.0e-12
B
Enriched pathways of differential methylated genes
Metabolism of proteins
Post-translational protein modification
Gene expression (transcription)
RNA Polymerase II Transcription
Generic transcription pathway
Adaptive immune system
Membrane trafficking
Metabolism of RNA
Transcriptional regulation by TP53
SUMOylation
SUMO E3 ligases SUMOylate target proteins
Regulation of TP53 activity
Death receptor signaling
TLR4 cascade
MyD88-independent TLR4 cascade
TRIF (TICAM1)-mediated TLR4 signaling
PI metabolism
Signaling by TGF-beta receptor complex
Interleukin-3, interleukin-5 and GM-CSF signaling
SUMOylation of transcription cofactors
0.00 0.05 0.10 0.15 0.20
Gene ratio
Count
100
200
300
400
500
Adjusted p value
1e-05
2e-05
C
Enriched pathways of differential expressed and differential methylated genes
Gene expression (transcription)
RNA Polymerase II Transcription
Generic transcription pathway
Cell cycle
Cell cycle, mitotic
Signaling by Rho GTPases
Cytokine signaling in immune system
Death receptor signaling
TLR cascades
TLR4 cascade
MyD88-independent TLR4 cascade
TRIF (TICAM1)-mediated TLR4 signaling
PI metabolism
p75 NTR receptor-mediated signalling
TRAF6 mediated induction of NFkB and MAP kinases upon TLR7/8 or 9 activation
MyD88:MAL (TIRAP) cascade initiated on plasma membrane
Toll Like Receptor TLR1:TLR2 Cascade
Toll Like Receptor TLR6:TLR2 Cascade
Synthesis of PIPs at the plasma membrane
NRAGE signals death through JNK
0.04 0.08 0.12
Gene ratio
Count
50
100
150
200
Adjusted p value
0.002
0.004
0.006

Supplementary Fig. 5 Pathway enrichment analysis of differentially expressed and methylated genes following LPS treatment. A Pathways enriched for differentially expressed genes following LPS treatment for 6 h. **B,** Pathways enriched for differentially m^6A methylated genes following LPS treatment for 6 h. **C** Pathways enriched for genes with both differential expression and differential m^6A methylation following LPS treatment for 6 h.

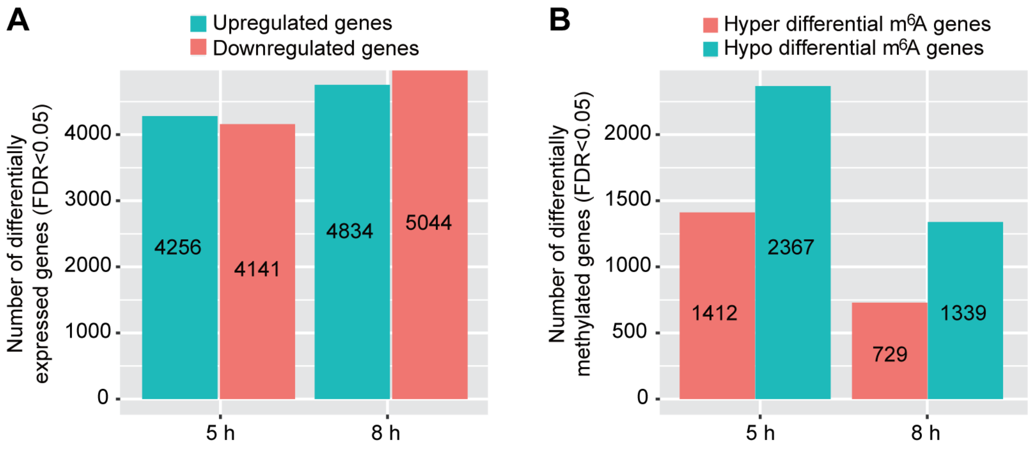

Supplementary Fig. 6 Analysis of transcriptomes and epitranscriptomes of cells infected with HSV-1. A Numbers of upregulated and downregulated genes in cells infected with HSV-1 for 5 and 8 h. **B** Numbers of hypermethylated and hypomethylated genes in cells infected with HSV-1 for 5 and 8 h.

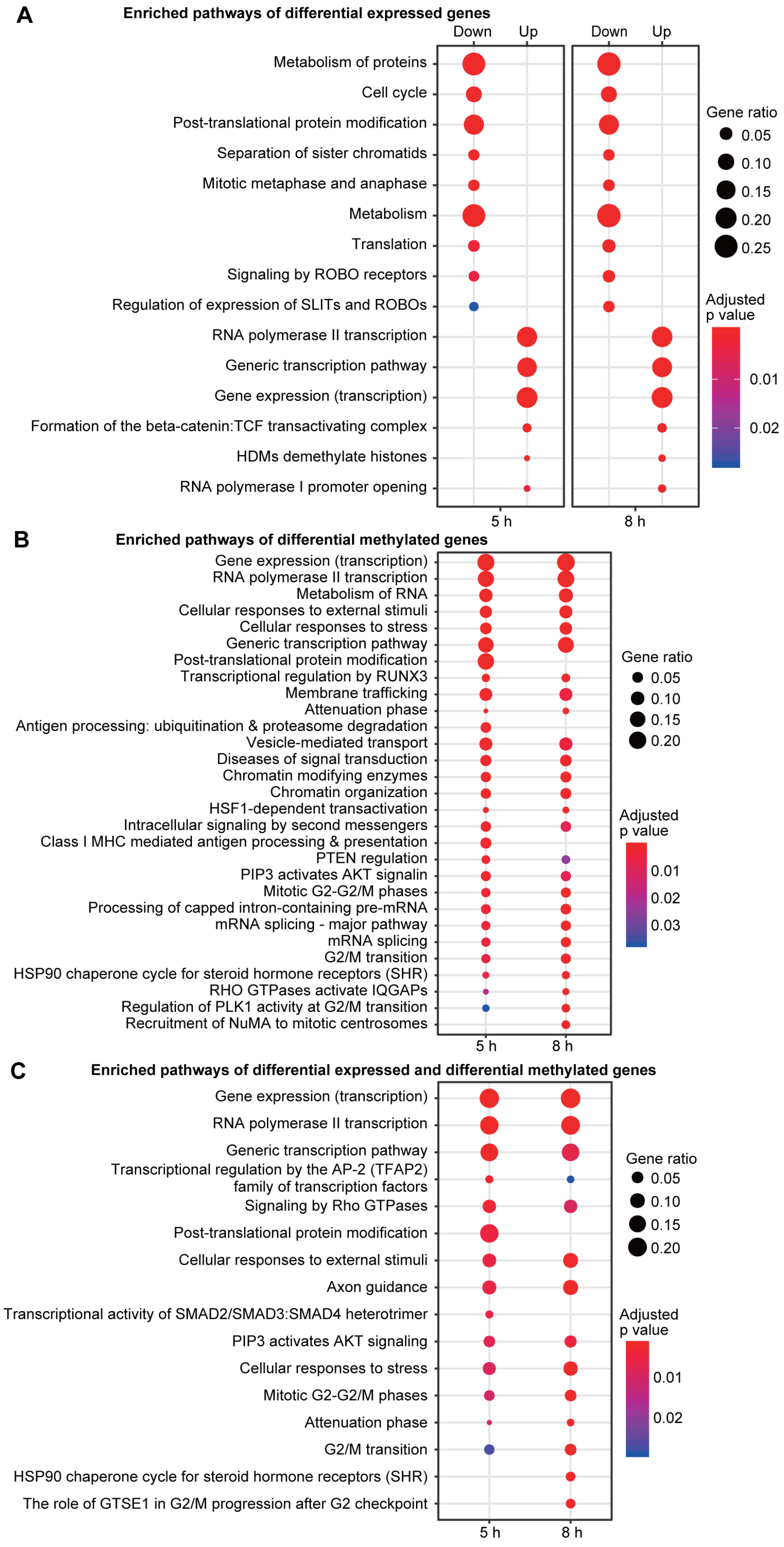

A
Enriched pathways of differential expressed genes
Down
Up
Down
Up
Metabolism of proteins
Cell cycle
Post-translational protein modification
Separation of sister chromatids
Mitotic metaphase and anaphase
Metabolism
Translation
Signaling by ROBO receptors
Regulation of expression of SLITs and ROBOs
RNA polymerase II transcription
Generic transcription pathway
Gene expression (transcription)
Formation of the beta-catenin:TCF transactivating complex
HDMs demethylate histones
RNA polymerase I promoter opening
5 h
8 h
Gene ratio
0.05
0.10
0.15
0.20
0.25
Adjusted p value
0.01
0.02
B
Enriched pathways of differential methylated genes
Gene expression (transcription)
RNA polymerase II transcription
Metabolism of RNA
Cellular responses to external stimuli
Cellular responses to stress
Generic transcription pathway
Post-translational protein modification
Transcriptional regulation by RUNX3
Membrane trafficking
Attenuation phase
Antigen processing: ubiquitination & proteasome degradation
Vesicle-mediated transport
Diseases of signal transduction
Chromatin modifying enzymes
Chromatin organization
HSF1-dependent transactivation
Intracellular signaling by second messengers
Class I MHC mediated antigen processing & presentation
PTEN regulation
PIP3 activates AKT signalin
Mitotic G2-G2/M phases
Processing of capped intron-containing pre-mRNA
mRNA splicing - major pathway
mRNA splicing
G2/M transition
HSP90 chaperone cycle for steroid hormone receptors (SHR)
RHO GTPases activate IQGAPs
Regulation of PLK1 activity at G2/M transition
Recruitment of NuMA to mitotic centrosomes
5 h
8 h
Gene ratio
0.05
0.10
0.15
0.20
Adjusted p value
0.01
0.02
0.03
C
Enriched pathways of differential expressed and differential methylated genes
Gene expression (transcription)
RNA polymerase II transcription
Generic transcription pathway
Transcriptional regulation by the AP-2 (TFAP2) family of transcription factors
Signaling by Rho GTPases
Post-translational protein modification
Cellular responses to external stimuli
Axon guidance
Transcriptional activity of SMAD2/SMAD3:SMAD4 heterotrimer
PIP3 activates AKT signaling
Cellular responses to stress
Mitotic G2-G2/M phases
Attenuation phase
G2/M transition
HSP90 chaperone cycle for steroid hormone receptors (SHR)
The role of GTSE1 in G2/M progression after G2 checkpoint
5 h
8 h
Gene ratio
0.05
0.10
0.15
0.20
Adjusted p value
0.01
0.02

Supplementary Fig. 7 Pathway enrichment analysis of differentially expressed and methylated genes following HSV-1 infection. **A** Pathways enriched for differentially expressed genes following HSV-1 infection for 5 and 8 h. **B** Pathways enriched for differentially m^6A methylated genes following HSV-1 infection for 5 and 8 h. **C** Pathways enriched for genes with both differential expression and differential m^6A methylation following HSV-1 infection for 5 and 8 h.

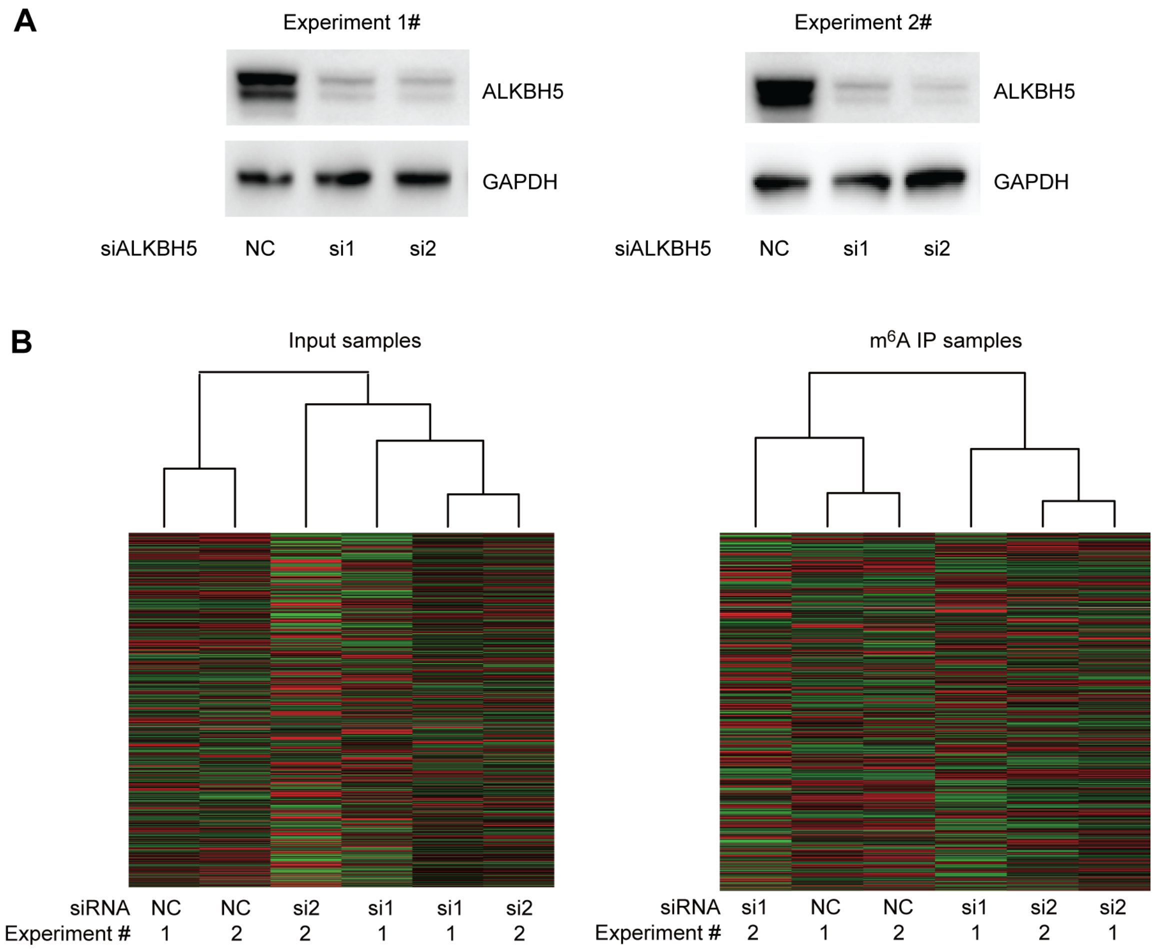

Supplementary Fig. 8 Clustering analysis of RNAseq results of input and m^{6}A-IP samples of ALKBH5 knockdown cells infected with *P. aeruginosa* from two independent experiments. A ALKBH5 knockdown efficiencies in two independent experiments examined by Western-blotting. **B** Clustering analysis of RNAseq results of input and m^{6}A-IP samples of ALKBH5 knockdown cells infected with *P. aeruginosa* for 6 h from two independent experiments.

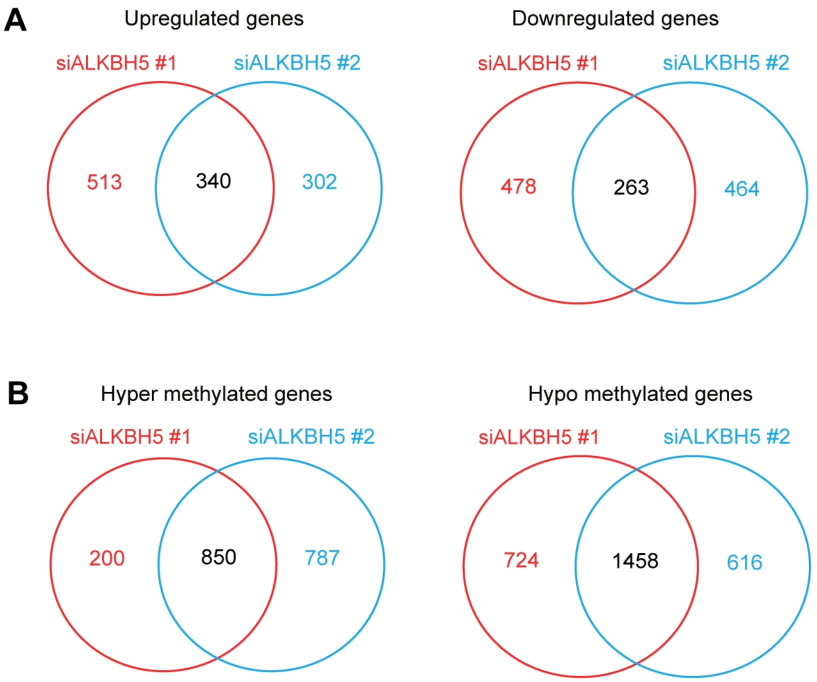

Supplementary Fig. 9 Analysis of gene expression and m^{6}A modifications of *P. aeruginosa* infected cells with ALKBH5 knockdown with two siRNAs. A Numbers of upregulated and downregulated genes in ALKBH5 knockdown cells infected with *P. aeruginosa* for 6 h. **B** Numbers of hypermethylated and hypomethylated genes in ALKBH5 knockdown cells infected with *P. aeruginosa* for 6 h.

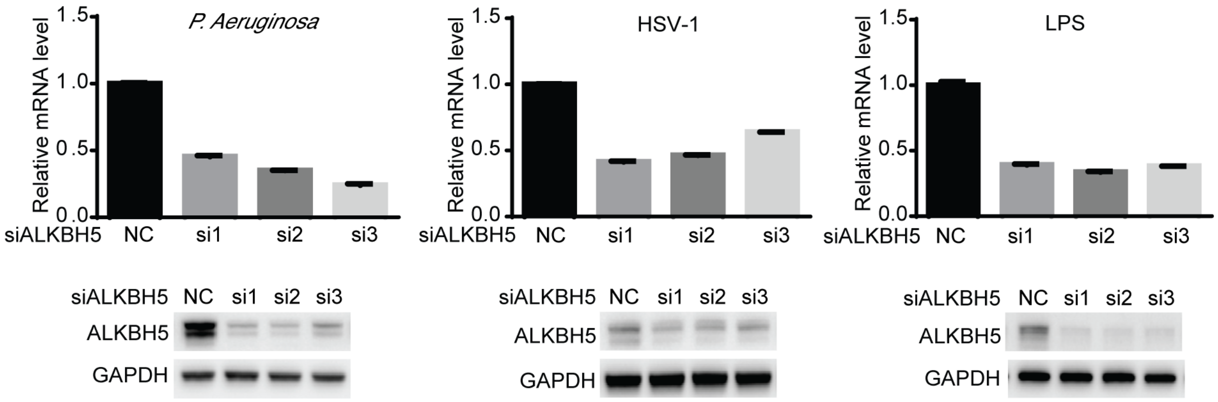

Fig. S10 Efficiencies of ALKBH5 knockdown in RAW264.7 cells infected with *P. aeruginosa* or HSV-1, or treated with LPS.

Table S1 Differentially expressed genes following *P. aeruginosa* infection

Up-regulated genes following 2 h *P. aeruginosa* infection

Metabolism of RNA

gene_name	baseMean	log2FoldChange	p-value
Noc4l	855.80	0.49	5.07E-05
Sf3b3	9646.91	0.36	3.68E-09
Csnk1d	3033.24	0.29	1.55E-04
Cnot6	2130.67	0.29	4.23E-03
Tsr1	1116.88	0.82	3.97E-13
Utp15	1312.69	0.80	4.62E-14
Sf3b4	1757.74	0.95	2.82E-24
Thoc5	1155.63	0.50	4.13E-06
Srsf9	1400.29	0.37	3.82E-04
Cstf2	1258.38	0.69	9.23E-11
Srsf1	5189.04	1.06	1.20E-42
Pwp2	1280.32	1.20	9.08E-30
Tgs1	1144.68	0.52	1.12E-05
Apobec1	3177.15	0.36	6.48E-06
Aqr	2565.57	0.47	1.11E-08
Hnrnpd	3628.18	0.94	1.01E-31
Bop1	2245.75	0.69	3.21E-15
Cdk7	629.21	0.51	3.86E-04
Dhx15	5131.97	0.98	1.49E-43
Ddx5	12469.88	1.27	7.99E-93
Ddx6	4995.87	0.56	1.06E-15
Dhx9	5561.16	0.17	2.15E-02
Ercc3	937.38	0.42	9.14E-04
Fbl	826.92	0.38	2.44E-03
Emg1	1344.19	0.54	1.06E-07
Gtf2h1	2709.73	0.79	1.52E-21
Gtf2h4	391.34	0.48	6.77E-03
Hnrnpc	4344.64	0.68	5.05E-17
Hnrnpk	5505.33	0.79	2.84E-20
Hspa1b	445.90	0.88	6.45E-08
Elavl1	2796.21	0.70	1.82E-17
Rps2	3955.80	0.28	2.00E-04
Psmb9	149.93	0.58	2.68E-02
Psmb8	1263.83	0.93	2.69E-18
Magoh	607.34	0.57	1.79E-04
Mapkapk2	19163.37	1.87	1.05E-193
Psmd7	2221.96	0.97	6.74E-29
Ncl	37904.91	1.03	3.76E-75
Pdcd11	4423.52	0.48	4.40E-09

Papola	6721.71	0.80	3.62E-34
Cnot7	1382.23	1.37	1.07E-33
Ppp2ca	3481.38	0.73	2.47E-20
Nup88	1507.82	0.45	4.15E-06
Psma2	1307.08	0.66	2.73E-10
Psma3	318.87	1.33	2.37E-12
Psmb10	503.81	1.43	3.06E-18
Psmb4	2641.44	0.71	5.96E-17
Psmb5	733.35	0.50	1.81E-04
Psmb6	2414.62	1.16	2.24E-40
Psmb7	2059.92	0.63	1.11E-11
Psmc1	2078.70	0.72	5.03E-16
Psmc2	1905.33	0.70	5.26E-13
Psmc5	1770.72	0.73	2.81E-10
Psme1	897.17	0.79	7.42E-10
Psme2	212.05	1.56	8.07E-12
Psme3	3341.08	0.84	4.21E-20
Ptbp1	6220.62	0.89	7.25E-39
Eif4a3	1713.45	0.29	2.01E-03
Hspa1a	218.57	0.74	1.05E-03
Ranbp2	8567.97	0.95	4.06E-34
Utp14b	671.07	1.24	1.25E-19
Upf1	3547.07	0.25	9.09E-04
Rnps1	1258.06	0.66	4.88E-09
Polr2c	353.29	0.95	1.15E-07
Rps18	1027.99	0.32	1.65E-02
Rps7	3527.83	0.23	3.20E-03
Sf3a2	1414.93	0.48	2.70E-06
Srsf2	4465.48	1.28	1.85E-53
Srsf3	2645.57	1.31	2.82E-39
Tra2b	1799.20	1.56	4.20E-39
Slbp	687.47	0.38	4.27E-03
Smn1	618.72	0.59	2.48E-05
Eftud2	3203.96	0.37	1.39E-06
Snrnp70	7458.86	0.25	1.90E-04
Snrpb	3112.08	0.32	3.58E-04
Snrpd1	647.36	0.38	8.61E-03
Snu13	2044.42	0.98	2.70E-24
Eif4g1	19458.44	0.60	7.65E-25
Gtf2h3	396.91	0.49	3.30E-03
Tbl3	999.25	0.72	4.44E-10
Bms1	2798.07	0.62	7.75E-15
Tut7	2579.20	0.86	1.06E-22

Diexf	1252.22	0.32	2.69E-03
Gemin5	1308.29	0.47	3.99E-06
Utp6	1303.70	0.63	3.43E-09
Utp18	1093.75	0.52	9.28E-06
Psmd2	4476.34	0.40	1.59E-08
Utp4	2059.62	0.89	4.90E-23
Heatr1	3675.59	0.73	3.21E-22
Nup153	3933.48	0.32	1.30E-05
Ttc37	1152.29	0.31	8.02E-03
Psmd3	2894.68	1.23	5.68E-52
Zrsr2	904.60	0.65	4.76E-08
U2af2	4665.07	0.72	4.46E-18
Ubc	287025.79	0.41	1.53E-11
Dcaf13	1197.64	1.01	1.62E-20
Wbp4	746.15	0.48	4.80E-04
Srsf7	2301.14	1.26	2.06E-48
Thoc1	899.19	0.74	8.18E-10
Wdr36	2008.27	1.00	2.63E-23
Patl1	2575.92	0.68	2.30E-16
Ybx1	7873.51	0.29	1.73E-05
Ywhaz	10307.09	1.43	5.40E-80
Smg7	4150.58	1.36	4.40E-75
Nup188	2572.05	0.63	3.78E-13
Tnks1bp1	635.65	0.54	1.21E-04
Cstf3	981.58	0.68	7.59E-09
Hnrnpa3	6419.85	0.85	1.09E-25
Smg5	2213.55	0.48	3.17E-08
Prpf38a	747.09	0.92	7.05E-12
Bud31	602.07	1.24	1.15E-15
Cnot3	1378.15	0.24	2.27E-02
Fus	5757.16	1.31	4.30E-76
Edc4	1903.61	0.23	2.08E-02
Gtf2h2	329.81	0.51	6.44E-03
Pcbp1	7853.64	0.30	2.94E-06
Psmc4	2470.73	0.28	8.84E-04
Xrn1	2391.74	0.75	1.67E-16
Xrn2	3817.08	1.00	2.17E-30
Psma1	1718.45	0.47	1.36E-06
Psma4	1203.96	0.54	5.09E-07
Psma5	435.57	0.80	1.98E-06
Psma6	2553.03	1.13	1.23E-35
Psmb2	1614.36	0.65	1.36E-11
Rps15a	3590.19	0.22	7.93E-03

Cpsf7	2073.24	0.50	2.33E-08
Nup54	805.86	0.83	6.78E-11
Wdr3	2773.51	0.29	3.20E-04
Tex10	1102.22	0.53	1.22E-06
Nup98	6176.36	1.00	1.54E-51
Txnl4a	2369.11	0.30	8.41E-04
Rrp9	987.19	1.40	1.27E-33
Imp4	1568.56	0.65	8.24E-11
Usp39	1164.78	0.32	3.90E-03
Gnl3	3384.80	0.54	2.21E-12
Snrnp200	5713.36	0.39	1.37E-08
Thoc2	3105.26	0.46	3.43E-07
Edc3	703.75	0.33	1.23E-02
Ltv1	1132.93	1.36	5.51E-34
Thoc6	414.57	0.69	7.11E-05
Lsm4	850.92	0.48	1.86E-04
Exosc10	2071.46	0.88	7.15E-21
Pdcd7	373.16	0.87	8.49E-07
Cpsf2	2726.53	1.07	3.81E-39
Ppp2r1a	4892.59	0.54	5.63E-15
Hnrnpu	21298.31	0.56	4.89E-22
Cnot11	817.13	0.30	1.66E-02
Plrg1	1232.43	0.57	5.62E-08
Nxf1	2440.76	0.55	3.62E-10
Hnrnpa2b1	12966.43	0.53	2.06E-13
Cnot4	1206.49	0.43	8.25E-05
Ddx39b	5495.58	0.48	2.11E-12
Rpp30	166.54	0.67	1.01E-02
Ywhab	3822.62	0.68	1.31E-20
Nop58	3819.50	1.46	1.08E-58
Ppie	366.99	0.57	2.40E-03
Prpf40a	3297.12	0.67	3.67E-15
Ddx21	9247.56	0.87	5.05E-40
Hnrnph2	2145.64	0.92	5.12E-16
Mettl3	418.05	0.82	6.88E-07
Adar	6373.73	2.55	1.81E-261
Hbs1l	1477.49	0.67	1.15E-11
Psmd8	8034.09	1.06	2.78E-54
Wdr12	832.08	0.70	2.88E-08
Isy1	764.41	0.75	1.07E-07
Hnrnph1	3473.28	0.42	1.42E-07
Nup160	1708.86	1.00	2.41E-22
Rcl1	799.78	0.72	1.19E-08

Psmd14	1865.95	1.17	1.45E-32
Wbp11	1760.84	0.27	6.59E-03
Rbm8a	1149.12	0.58	9.89E-08
Wtap	1835.27	0.58	2.58E-10
Dhx38	3112.05	1.14	1.07E-38
Pes1	1723.62	0.35	4.06E-04
Sf3b6	823.90	0.74	4.99E-09
Sarnp	193.67	0.74	2.60E-03
Pno1	948.69	1.05	8.34E-18
Wdr61	994.02	0.32	9.62E-03
Snw1	1901.38	0.41	5.74E-06
Exosc3	421.09	1.05	2.20E-10
Psmd6	1226.03	0.56	1.67E-07
Rps23	3912.42	0.31	1.08E-04
Chtop	3036.63	0.52	2.91E-10
Exosc1	846.78	0.58	2.80E-06
Snrnp40	882.33	0.52	1.65E-05
Snrnp27	587.31	0.41	4.29E-03
Ccnh	536.43	0.46	1.82E-03
Riok3	1597.07	1.10	9.69E-29
Psmd12	2034.72	1.04	3.31E-26
Rpp14	452.55	0.43	1.19E-02
Psmc6	1881.68	0.93	5.52E-24
Nop56	4541.66	0.54	6.00E-14
Zcrb1	656.94	0.96	3.20E-10
Utp11	902.67	0.29	1.47E-02
Lsm1	603.86	0.40	5.97E-03
Rnpc3	566.46	1.26	6.95E-17
Cwc27	818.21	0.40	2.37E-03
Sf3a1	2738.48	0.35	3.76E-05
Cwc25	654.30	0.44	2.82E-03
Ccar1	3102.03	0.88	4.01E-30
Polr2g	740.76	0.45	1.69E-03
Ddx47	1083.77	0.45	1.31E-04
Rnmt	1941.08	0.66	6.42E-12
U2surp	3532.65	0.41	2.88E-07
Puf60	2002.40	0.76	1.59E-17
Mphosph10	1739.22	0.62	1.92E-08
Srsf6	4785.08	0.65	4.65E-20
Rps13	1304.12	0.33	1.86E-03
Ddx39	1894.26	0.89	5.40E-21
Mphosph6	337.01	1.49	1.69E-15
Gtf2f2	603.03	0.63	1.09E-05

Nol11	1029.73	0.77	1.90E-10
Snrpa1	972.63	0.56	1.45E-06
Prpf31	2699.17	0.65	2.53E-15
Ebna1bp2	1265.87	0.95	1.27E-19
Psmd11	1598.13	0.97	4.92E-22
Srsf11	3448.22	0.53	4.24E-10
Polr2d	294.66	1.06	8.36E-08
Nup37	341.28	0.70	1.28E-04
Fyttd1	1033.71	0.67	1.22E-08
A1cf	12.86	3.28	1.16E-05
Cd2bp2	1764.96	0.32	7.12E-04
Psmd1	3836.24	1.21	5.88E-60
Zc3h11a	5295.21	0.76	1.38E-26
Dcp2	1738.76	1.58	4.80E-63
Utp20	4188.67	0.88	1.65E-33
Prpf3	1417.09	0.83	6.47E-15
Riok1	706.25	0.83	6.31E-10
Nupl1	1837.54	0.87	2.09E-17
Ppp2r2a	2064.71	1.15	2.71E-38
Ddx42	1884.35	0.48	1.29E-07
Cnot2	1267.16	0.59	3.02E-08
Seh1l	1401.07	0.88	1.93E-17
Mtrex	1671.10	0.30	6.71E-03
Wdr43	3527.29	0.93	7.50E-35
Utp14a	1949.76	0.72	6.94E-15
Dis3	799.85	0.38	2.16E-03
Thoc3	663.20	0.41	2.49E-03
Wdr75	1519.51	0.73	1.19E-11
Fcf1	440.15	0.56	5.06E-04
Poldip3	2940.44	0.40	3.67E-07
Wdr33	2103.28	0.29	1.15E-03
Ddx23	2983.92	0.20	1.26E-02
Pcf11	3945.90	1.47	1.51E-86
Rrp7a	1169.58	0.76	4.21E-12
Nop14	2063.36	1.19	1.23E-35
Rps25	4461.05	0.16	2.64E-02
Dcp1a	1307.25	0.72	4.60E-12
Smndc1	1521.51	0.97	5.37E-23
Hnrnpm	5031.22	0.80	9.70E-30
Rbm17	1226.72	0.35	2.99E-03
Hnrnpa0	4036.97	0.80	3.86E-23
Cwc22	1872.46	0.46	4.30E-07
Senp3	2366.44	0.22	7.22E-03

Sf3b1	8686.33	0.77	1.81E-31
Rbm5	2521.49	0.25	2.80E-03
Srrt	3562.01	0.30	6.53E-05
Gtf2f1	1718.09	0.82	2.75E-17
Hnrnpf	14104.41	0.77	4.26E-35

Processing of capped intron-containing pre-mRNA

gene_name	baseMean	log2FoldChange	p-value
Sf3b3	9646.91	0.36	3.68E-09
Sf3b4	1757.74	0.95	2.82E-24
Thoc5	1155.63	0.50	4.13E-06
Srsf9	1400.29	0.37	3.82E-04
Cstf2	1258.38	0.69	9.23E-11
Srsf1	5189.04	1.06	1.20E-42
Aqr	2565.57	0.47	1.11E-08
Hnrnpd	3628.18	0.94	1.01E-31
Dhx15	5131.97	0.98	1.49E-43
Ddx5	12469.88	1.27	7.99E-93
Dhx9	5561.16	0.17	2.15E-02
Hnrnpc	4344.64	0.68	5.05E-17
Hnrnpk	5505.33	0.79	2.84E-20
Elavl1	2796.21	0.70	1.82E-17
Magoh	607.34	0.57	1.79E-04
Papola	6721.71	0.80	3.62E-34
Nup88	1507.82	0.45	4.15E-06
Ptbp1	6220.62	0.89	7.25E-39
Eif4a3	1713.45	0.29	2.01E-03
Ranbp2	8567.97	0.95	4.06E-34
Rnps1	1258.06	0.66	4.88E-09
Polr2c	353.29	0.95	1.15E-07
Sf3a2	1414.93	0.48	2.70E-06
Srsf2	4465.48	1.28	1.85E-53
Srsf3	2645.57	1.31	2.82E-39
Tra2b	1799.20	1.56	4.20E-39
Eftud2	3203.96	0.37	1.39E-06
Snrnp70	7458.86	0.25	1.90E-04
Snrpb	3112.08	0.32	3.58E-04
Snrpd1	647.36	0.38	8.61E-03
Snu13	2044.42	0.98	2.70E-24
Nup153	3933.48	0.32	1.30E-05
Zrsr2	904.60	0.65	4.76E-08
U2af2	4665.07	0.72	4.46E-18
Wbp4	746.15	0.48	4.80E-04
Srsf7	2301.14	1.26	2.06E-48

Thoc1	899.19	0.74	8.18E-10
Ybx1	7873.51	0.29	1.73E-05
Nup188	2572.05	0.63	3.78E-13
Cstf3	981.58	0.68	7.59E-09
Hnrnpa3	6419.85	0.85	1.09E-25
Prpf38a	747.09	0.92	7.05E-12
Bud31	602.07	1.24	1.15E-15
Fus	5757.16	1.31	4.30E-76
Pcbp1	7853.64	0.30	2.94E-06
Cpsf7	2073.24	0.50	2.33E-08
Nup54	805.86	0.83	6.78E-11
Nup98	6176.36	1.00	1.54E-51
Txnl4a	2369.11	0.30	8.41E-04
Usp39	1164.78	0.32	3.90E-03
Snrnp200	5713.36	0.39	1.37E-08
Thoc2	3105.26	0.46	3.43E-07
Thoc6	414.57	0.69	7.11E-05
Lsm4	850.92	0.48	1.86E-04
Pdcd7	373.16	0.87	8.49E-07
Cpsf2	2726.53	1.07	3.81E-39
Hnrnpu	21298.31	0.56	4.89E-22
Plrg1	1232.43	0.57	5.62E-08
Nxf1	2440.76	0.55	3.62E-10
Hnrnpa2b1	12966.43	0.53	2.06E-13
Ddx39b	5495.58	0.48	2.11E-12
Ppie	366.99	0.57	2.40E-03
Prpf40a	3297.12	0.67	3.67E-15
Hnrnph2	2145.64	0.92	5.12E-16
Mettl3	418.05	0.82	6.88E-07
Isy1	764.41	0.75	1.07E-07
Hnrnph1	3473.28	0.42	1.42E-07
Nup160	1708.86	1.00	2.41E-22
Wbp11	1760.84	0.27	6.59E-03
Rbm8a	1149.12	0.58	9.89E-08
Wtap	1835.27	0.58	2.58E-10
Dhx38	3112.05	1.14	1.07E-38
Sf3b6	823.90	0.74	4.99E-09
Sarnp	193.67	0.74	2.60E-03
Snw1	1901.38	0.41	5.74E-06
Chtop	3036.63	0.52	2.91E-10
Snrnp40	882.33	0.52	1.65E-05
Snrnp27	587.31	0.41	4.29E-03
Zcrb1	656.94	0.96	3.20E-10

Rnpc3	566.46	1.26	6.95E-17
Cwc27	818.21	0.40	2.37E-03
Sf3a1	2738.48	0.35	3.76E-05
Cwc25	654.30	0.44	2.82E-03
Ccar1	3102.03	0.88	4.01E-30
Polr2g	740.76	0.45	1.69E-03
U2surp	3532.65	0.41	2.88E-07
Puf60	2002.40	0.76	1.59E-17
Srsf6	4785.08	0.65	4.65E-20
Ddx39	1894.26	0.89	5.40E-21
Gtf2f2	603.03	0.63	1.09E-05
Snrpa1	972.63	0.56	1.45E-06
Prpf31	2699.17	0.65	2.53E-15
Srsf11	3448.22	0.53	4.24E-10
Polr2d	294.66	1.06	8.36E-08
Nup37	341.28	0.70	1.28E-04
Fyttd1	1033.71	0.67	1.22E-08
Cd2bp2	1764.96	0.32	7.12E-04
Zc3h11a	5295.21	0.76	1.38E-26
Prpf3	1417.09	0.83	6.47E-15
Nupl1	1837.54	0.87	2.09E-17
Ddx42	1884.35	0.48	1.29E-07
Seh1l	1401.07	0.88	1.93E-17
Thoc3	663.20	0.41	2.49E-03
Poldip3	2940.44	0.40	3.67E-07
Wdr33	2103.28	0.29	1.15E-03
Ddx23	2983.92	0.20	1.26E-02
Pcf11	3945.90	1.47	1.51E-86
Smndc1	1521.51	0.97	5.37E-23
Hnrnpm	5031.22	0.80	9.70E-30
Rbm17	1226.72	0.35	2.99E-03
Hnrnpa0	4036.97	0.80	3.86E-23
Cwc22	1872.46	0.46	4.30E-07
Sf3b1	8686.33	0.77	1.81E-31
Rbm5	2521.49	0.25	2.80E-03
Srrt	3562.01	0.30	6.53E-05
Gtf2f1	1718.09	0.82	2.75E-17
Hnrnpf	14104.41	0.77	4.26E-35

Major pathway of rRNA processing in the nucleolus and cytosol

gene_name	baseMean	log2FoldChange	p-value
Noc4l	855.80	0.49	5.07E-05
Csnk1d	3033.24	0.29	1.55E-04
Tsr1	1116.88	0.82	3.97E-13

Utp15	1312.69	0.80	4.62E-14
Pwp2	1280.32	1.20	9.08E-30
Bop1	2245.75	0.69	3.21E-15
Fbl	826.92	0.38	2.44E-03
Emg1	1344.19	0.54	1.06E-07
Rps2	3955.80	0.28	2.00E-04
Ncl	37904.91	1.03	3.76E-75
Pdcd11	4423.52	0.48	4.40E-09
Utp14b	671.07	1.24	1.25E-19
Rps18	1027.99	0.32	1.65E-02
Rps7	3527.83	0.23	3.20E-03
Snu13	2044.42	0.98	2.70E-24
Tbl3	999.25	0.72	4.44E-10
Bms1	2798.07	0.62	7.75E-15
Diexf	1252.22	0.32	2.69E-03
Utp6	1303.70	0.63	3.43E-09
Utp18	1093.75	0.52	9.28E-06
Utp4	2059.62	0.89	4.90E-23
Heatr1	3675.59	0.73	3.21E-22
Dcaf13	1197.64	1.01	1.62E-20
Wdr36	2008.27	1.00	2.63E-23
Xrn2	3817.08	1.00	2.17E-30
Rps15a	3590.19	0.22	7.93E-03
Wdr3	2773.51	0.29	3.20E-04
Tex10	1102.22	0.53	1.22E-06
Rrp9	987.19	1.40	1.27E-33
Imp4	1568.56	0.65	8.24E-11
Gnl3	3384.80	0.54	2.21E-12
Ltv1	1132.93	1.36	5.51E-34
Exosc10	2071.46	0.88	7.15E-21
Rpp30	166.54	0.67	1.01E-02
Nop58	3819.50	1.46	1.08E-58
Ddx21	9247.56	0.87	5.05E-40
Wdr12	832.08	0.70	2.88E-08
Rcl1	799.78	0.72	1.19E-08
Pes1	1723.62	0.35	4.06E-04
Pno1	948.69	1.05	8.34E-18
Exosc3	421.09	1.05	2.20E-10
Rps23	3912.42	0.31	1.08E-04
Exosc1	846.78	0.58	2.80E-06
Riok3	1597.07	1.10	9.69E-29
Rpp14	452.55	0.43	1.19E-02
Nop56	4541.66	0.54	6.00E-14

Utp11	902.67	0.29	1.47E-02
Ddx47	1083.77	0.45	1.31E-04
Mphosph10	1739.22	0.62	1.92E-08
Rps13	1304.12	0.33	1.86E-03
Mphosph6	337.01	1.49	1.69E-15
Nol11	1029.73	0.77	1.90E-10
Ebna1bp2	1265.87	0.95	1.27E-19
Utp20	4188.67	0.88	1.65E-33
Riok1	706.25	0.83	6.31E-10
Mtrex	1671.10	0.30	6.71E-03
Wdr43	3527.29	0.93	7.50E-35
Utp14a	1949.76	0.72	6.94E-15
Wdr75	1519.51	0.73	1.19E-11
Fcf1	440.15	0.56	5.06E-04
Rrp7a	1169.58	0.76	4.21E-12
Nop14	2063.36	1.19	1.23E-35
Rps25	4461.05	0.16	2.64E-02
Senp3	2366.44	0.22	7.22E-03

rRNA processing

gene_name	baseMean	log2FoldChange	p-value
Noc4l	855.80	0.49	5.07E-05
Csnk1d	3033.24	0.29	1.55E-04
Tsr1	1116.88	0.82	3.97E-13
Utp15	1312.69	0.80	4.62E-14
Pwp2	1280.32	1.20	9.08E-30
Bop1	2245.75	0.69	3.21E-15
Fbl	826.92	0.38	2.44E-03
Emg1	1344.19	0.54	1.06E-07
Rps2	3955.80	0.28	2.00E-04
Ncl	37904.91	1.03	3.76E-75
Pdcd11	4423.52	0.48	4.40E-09
Utp14b	671.07	1.24	1.25E-19
Rps18	1027.99	0.32	1.65E-02
Rps7	3527.83	0.23	3.20E-03
Snu13	2044.42	0.98	2.70E-24
Tbl3	999.25	0.72	4.44E-10
Bms1	2798.07	0.62	7.75E-15
Diexf	1252.22	0.32	2.69E-03
Utp6	1303.70	0.63	3.43E-09
Utp18	1093.75	0.52	9.28E-06
Utp4	2059.62	0.89	4.90E-23
Heatr1	3675.59	0.73	3.21E-22
Dcaf13	1197.64	1.01	1.62E-20

Wdr36	2008.27	1.00	2.63E-23
Xrn2	3817.08	1.00	2.17E-30
Rps15a	3590.19	0.22	7.93E-03
Wdr3	2773.51	0.29	3.20E-04
Tex10	1102.22	0.53	1.22E-06
Rrp9	987.19	1.40	1.27E-33
Imp4	1568.56	0.65	8.24E-11
Gnl3	3384.80	0.54	2.21E-12
Ltv1	1132.93	1.36	5.51E-34
Exosc10	2071.46	0.88	7.15E-21
Rpp30	166.54	0.67	1.01E-02
Nop58	3819.50	1.46	1.08E-58
Ddx21	9247.56	0.87	5.05E-40
Wdr12	832.08	0.70	2.88E-08
Rcl1	799.78	0.72	1.19E-08
Pes1	1723.62	0.35	4.06E-04
Pno1	948.69	1.05	8.34E-18
Exosc3	421.09	1.05	2.20E-10
Rps23	3912.42	0.31	1.08E-04
Exosc1	846.78	0.58	2.80E-06
Riok3	1597.07	1.10	9.69E-29
Rpp14	452.55	0.43	1.19E-02
Nop56	4541.66	0.54	6.00E-14
Utp11	902.67	0.29	1.47E-02
Ddx47	1083.77	0.45	1.31E-04
Mphosph10	1739.22	0.62	1.92E-08
Rps13	1304.12	0.33	1.86E-03
Mphosph6	337.01	1.49	1.69E-15
Nol11	1029.73	0.77	1.90E-10
Ebna1bp2	1265.87	0.95	1.27E-19
Utp20	4188.67	0.88	1.65E-33
Riok1	706.25	0.83	6.31E-10
Mtrex	1671.10	0.30	6.71E-03
Wdr43	3527.29	0.93	7.50E-35
Utp14a	1949.76	0.72	6.94E-15
Wdr75	1519.51	0.73	1.19E-11
Fcf1	440.15	0.56	5.06E-04
Rrp7a	1169.58	0.76	4.21E-12
Nop14	2063.36	1.19	1.23E-35
Rps25	4461.05	0.16	2.64E-02
Senp3	2366.44	0.22	7.22E-03

rRNA processing in the nucleus and cytosol

gene_name	baseMean	log2FoldChange	p-value

Noc4l	855.80	0.49	5.07E-05
Csnk1d	3033.24	0.29	1.55E-04
Tsr1	1116.88	0.82	3.97E-13
Utp15	1312.69	0.80	4.62E-14
Pwp2	1280.32	1.20	9.08E-30
Bop1	2245.75	0.69	3.21E-15
Fbl	826.92	0.38	2.44E-03
Emg1	1344.19	0.54	1.06E-07
Rps2	3955.80	0.28	2.00E-04
Ncl	37904.91	1.03	3.76E-75
Pdcd11	4423.52	0.48	4.40E-09
Utp14b	671.07	1.24	1.25E-19
Rps18	1027.99	0.32	1.65E-02
Rps7	3527.83	0.23	3.20E-03
Snu13	2044.42	0.98	2.70E-24
Tbl3	999.25	0.72	4.44E-10
Bms1	2798.07	0.62	7.75E-15
Diexf	1252.22	0.32	2.69E-03
Utp6	1303.70	0.63	3.43E-09
Utp18	1093.75	0.52	9.28E-06
Utp4	2059.62	0.89	4.90E-23
Heatr1	3675.59	0.73	3.21E-22
Dcaf13	1197.64	1.01	1.62E-20
Wdr36	2008.27	1.00	2.63E-23
Xrn2	3817.08	1.00	2.17E-30
Rps15a	3590.19	0.22	7.93E-03
Wdr3	2773.51	0.29	3.20E-04
Tex10	1102.22	0.53	1.22E-06
Rrp9	987.19	1.40	1.27E-33
Imp4	1568.56	0.65	8.24E-11
Gnl3	3384.80	0.54	2.21E-12
Ltv1	1132.93	1.36	5.51E-34
Exosc10	2071.46	0.88	7.15E-21
Rpp30	166.54	0.67	1.01E-02
Nop58	3819.50	1.46	1.08E-58
Ddx21	9247.56	0.87	5.05E-40
Wdr12	832.08	0.70	2.88E-08
Rcl1	799.78	0.72	1.19E-08
Pes1	1723.62	0.35	4.06E-04
Pno1	948.69	1.05	8.34E-18
Exosc3	421.09	1.05	2.20E-10
Rps23	3912.42	0.31	1.08E-04
Exosc1	846.78	0.58	2.80E-06

Riok3	1597.07	1.10	9.69E-29
Rpp14	452.55	0.43	1.19E-02
Nop56	4541.66	0.54	6.00E-14
Utp11	902.67	0.29	1.47E-02
Ddx47	1083.77	0.45	1.31E-04
Mphosph10	1739.22	0.62	1.92E-08
Rps13	1304.12	0.33	1.86E-03
Mphosph6	337.01	1.49	1.69E-15
Nol11	1029.73	0.77	1.90E-10
Ebna1bp2	1265.87	0.95	1.27E-19
Utp20	4188.67	0.88	1.65E-33
Riok1	706.25	0.83	6.31E-10
Mtrex	1671.10	0.30	6.71E-03
Wdr43	3527.29	0.93	7.50E-35
Utp14a	1949.76	0.72	6.94E-15
Wdr75	1519.51	0.73	1.19E-11
Fcf1	440.15	0.56	5.06E-04
Rrp7a	1169.58	0.76	4.21E-12
Nop14	2063.36	1.19	1.23E-35
Rps25	4461.05	0.16	2.64E-02
Senp3	2366.44	0.22	7.22E-03

mRNA splicing

gene_name	baseMean	log2FoldChange	p-value
Sf3b3	9646.91	0.36	3.68E-09
Sf3b4	1757.74	0.95	2.82E-24
Srsf9	1400.29	0.37	3.82E-04
Cstf2	1258.38	0.69	9.23E-11
Srsf1	5189.04	1.06	1.20E-42
Aqr	2565.57	0.47	1.11E-08
Hnrnpd	3628.18	0.94	1.01E-31
Dhx15	5131.97	0.98	1.49E-43
Ddx5	12469.88	1.27	7.99E-93
Dhx9	5561.16	0.17	2.15E-02
Hnrnpc	4344.64	0.68	5.05E-17
Hnrnpk	5505.33	0.79	2.84E-20
Elavl1	2796.21	0.70	1.82E-17
Magoh	607.34	0.57	1.79E-04
Papola	6721.71	0.80	3.62E-34
Ptbp1	6220.62	0.89	7.25E-39
Eif4a3	1713.45	0.29	2.01E-03
Rnps1	1258.06	0.66	4.88E-09
Polr2c	353.29	0.95	1.15E-07
Sf3a2	1414.93	0.48	2.70E-06

Srsf2	4465.48	1.28	1.85E-53
Srsf3	2645.57	1.31	2.82E-39
Tra2b	1799.20	1.56	4.20E-39
Eftud2	3203.96	0.37	1.39E-06
Snrnp70	7458.86	0.25	1.90E-04
Snrpb	3112.08	0.32	3.58E-04
Snrpd1	647.36	0.38	8.61E-03
Snu13	2044.42	0.98	2.70E-24
Zrsr2	904.60	0.65	4.76E-08
U2af2	4665.07	0.72	4.46E-18
Wbp4	746.15	0.48	4.80E-04
Srsf7	2301.14	1.26	2.06E-48
Ybx1	7873.51	0.29	1.73E-05
Cstf3	981.58	0.68	7.59E-09
Hnrnpa3	6419.85	0.85	1.09E-25
Prpf38a	747.09	0.92	7.05E-12
Bud31	602.07	1.24	1.15E-15
Fus	5757.16	1.31	4.30E-76
Pcbp1	7853.64	0.30	2.94E-06
Cpsf7	2073.24	0.50	2.33E-08
Txnl4a	2369.11	0.30	8.41E-04
Usp39	1164.78	0.32	3.90E-03
Snrnp200	5713.36	0.39	1.37E-08
Lsm4	850.92	0.48	1.86E-04
Pdcd7	373.16	0.87	8.49E-07
Cpsf2	2726.53	1.07	3.81E-39
Hnrnpu	21298.31	0.56	4.89E-22
Plrg1	1232.43	0.57	5.62E-08
Hnrnpa2b1	12966.43	0.53	2.06E-13
Ppie	366.99	0.57	2.40E-03
Prpf40a	3297.12	0.67	3.67E-15
Hnrnph2	2145.64	0.92	5.12E-16
Isy1	764.41	0.75	1.07E-07
Hnrnph1	3473.28	0.42	1.42E-07
Wbp11	1760.84	0.27	6.59E-03
Rbm8a	1149.12	0.58	9.89E-08
Dhx38	3112.05	1.14	1.07E-38
Sf3b6	823.90	0.74	4.99E-09
Snw1	1901.38	0.41	5.74E-06
Snrnp40	882.33	0.52	1.65E-05
Snrnp27	587.31	0.41	4.29E-03
Zcrb1	656.94	0.96	3.20E-10
Rnpc3	566.46	1.26	6.95E-17

Cwc27	818.21	0.40	2.37E-03
Sf3a1	2738.48	0.35	3.76E-05
Cwc25	654.30	0.44	2.82E-03
Ccar1	3102.03	0.88	4.01E-30
Polr2g	740.76	0.45	1.69E-03
U2surp	3532.65	0.41	2.88E-07
Puf60	2002.40	0.76	1.59E-17
Srsf6	4785.08	0.65	4.65E-20
Gtf2f2	603.03	0.63	1.09E-05
Snrpa1	972.63	0.56	1.45E-06
Prpf31	2699.17	0.65	2.53E-15
Srsf11	3448.22	0.53	4.24E-10
Polr2d	294.66	1.06	8.36E-08
Cd2bp2	1764.96	0.32	7.12E-04
Prpf3	1417.09	0.83	6.47E-15
Ddx42	1884.35	0.48	1.29E-07
Wdr33	2103.28	0.29	1.15E-03
Ddx23	2983.92	0.20	1.26E-02
Pcf11	3945.90	1.47	1.51E-86
Smndc1	1521.51	0.97	5.37E-23
Hnrnpm	5031.22	0.80	9.70E-30
Rbm17	1226.72	0.35	2.99E-03
Hnrnpa0	4036.97	0.80	3.86E-23
Cwc22	1872.46	0.46	4.30E-07
Sf3b1	8686.33	0.77	1.81E-31
Rbm5	2521.49	0.25	2.80E-03
Srrt	3562.01	0.30	6.53E-05
Gtf2f1	1718.09	0.82	2.75E-17
Hnrnpf	14104.41	0.77	4.26E-35

Cytokine signaling in immune system

gene_name	baseMean	log2FoldChange	p-value
Isg15	1768.06	5.80	1.06E-29
Fbxw11	2393.98	0.49	7.29E-09
Rapgef1	3500.81	0.76	4.38E-24
Camk2d	787.23	2.46	4.65E-64
Irak2	2157.52	1.87	3.11E-85
Txlna	2418.28	0.46	2.54E-08
Birc3	1268.54	1.98	2.04E-72
App	6082.22	0.18	9.59E-03
Atf1	997.98	0.88	3.70E-14
Atf2	1245.48	0.71	5.58E-11
Canx	17707.20	0.68	1.19E-31
Casp1	421.10	0.47	4.55E-03

Chuk	1801.62	0.84	3.72E-20
Socs3	2693.34	2.52	1.83E-177
Socs1	34.35	3.82	2.11E-09
Crk	2237.50	0.91	3.71E-24
Crkl	1194.88	0.36	1.12E-03
Csf1	772.08	5.10	1.00E-164
Csf2	137.94	6.81	1.49E-13
Csf2ra	2371.27	1.05	1.81E-29
Csf2rb	2178.62	1.03	7.81E-33
Csf2rb2	1276.72	0.95	4.49E-19
Csf3	12968.04	10.09	0.00E+00
Dhx9	5561.16	0.17	2.15E-02
Hck	2225.09	2.78	2.13E-163
Ifnar1	1295.45	0.36	5.02E-04
Ifnb1	3.00	1.25	2.29E-02
Ikbkb	1973.97	0.36	7.70E-05
Il10	52.90	5.01	2.66E-10
Il10ra	919.28	0.68	7.69E-08
Il10rb	1186.35	0.95	6.08E-19
Il12rb2	100.94	0.77	1.37E-02
Il13ra2	4.78	1.80	3.48E-03
Il16	1176.57	0.27	2.09E-02
Il17ra	2041.41	0.21	2.29E-02
Il18	95.56	0.83	9.40E-03
Il1a	8860.86	6.99	1.72E-35
Il1b	3010.48	1.83	5.05E-07
Il1rap	318.62	1.47	1.12E-14
Il1rn	842.80	6.71	8.94E-134
Il2rg	1922.11	0.79	3.90E-17
Il6	105.78	6.01	6.70E-14
Il7	8.59	2.41	2.41E-03
Il7r	151.14	1.94	1.10E-11
Irf9	1645.00	2.95	1.30E-163
Jak1	3981.25	0.51	8.58E-10
Jak2	2409.58	2.47	1.30E-157
Jun	5350.55	1.74	1.79E-127
Lck	222.73	2.65	2.85E-27
Lif	3364.66	2.03	1.23E-05
Psmb9	149.93	0.58	2.68E-02
Psmb8	1263.83	0.93	2.69E-18
Lta	46.18	2.22	1.06E-06
Ltb	869.78	1.14	1.47E-15
Ltbr	839.48	0.43	5.15E-04

Il1rl1	401.61	1.33	3.28E-15
Lyn	7091.21	1.89	1.38E-172
Mapkapk2	19163.37	1.87	1.05E-193
Psmd7	2221.96	0.97	6.74E-29
Mx2	1754.59	8.82	3.72E-109
Myd88	2055.05	1.64	1.93E-75
Nfkb1	11298.39	3.56	0.00E+00
Nfkb2	5526.89	2.74	0.00E+00
Nfkbia	6463.17	3.18	0.00E+00
Nfkbib	1719.58	2.22	4.57E-92
Sqstm1	31840.37	1.78	1.14E-195
Osm	1661.73	5.61	0.00E+00
P4hb	18103.33	1.31	1.73E-108
Ppp2ca	3481.38	0.73	2.47E-20
Ppp2cb	827.68	0.67	1.47E-07
Eif2ak2	4747.58	3.44	0.00E+00
Psma2	1307.08	0.66	2.73E-10
Psma3	318.87	1.33	2.37E-12
Psmb10	503.81	1.43	3.06E-18
Psmb4	2641.44	0.71	5.96E-17
Psmb5	733.35	0.50	1.81E-04
Psmb6	2414.62	1.16	2.24E-40
Psmb7	2059.92	0.63	1.11E-11
Psmc1	2078.70	0.72	5.03E-16
Psmc2	1905.33	0.70	5.26E-13
Psmc5	1770.72	0.73	2.81E-10
Psme1	897.17	0.79	7.42E-10
Psme2	212.05	1.56	8.07E-12
Psme3	3341.08	0.84	4.21E-20
Eif4a3	1713.45	0.29	2.01E-03
Ptpn11	2343.35	0.49	1.57E-08
Ptpn2	898.52	1.62	4.51E-39
Ripk2	553.98	1.29	1.35E-17
Rela	2281.64	0.95	6.17E-27
Relb	1042.85	2.40	8.58E-69
Rps6ka2	1672.14	3.82	1.01E-249
Shc1	2224.69	0.51	7.55E-08
Smarca4	6769.63	0.36	3.95E-07
Sos2	1952.80	0.68	5.86E-14
Stat1	4763.25	3.39	0.00E+00
Stat2	2949.54	3.58	0.00E+00
Stat3	2888.45	1.88	7.48E-121
Stat6	4409.97	0.43	1.69E-09

Eif4g1	19458.44	0.60	7.65E-25
Stx3	1917.32	0.68	1.30E-13
Syk	3280.07	0.73	2.49E-19
Il20rb	42.09	1.39	1.99E-03
Trim25	10006.03	1.39	8.59E-110
Psmd2	4476.34	0.40	1.59E-08
Tnf	40207.52	5.55	0.00E+00
Tnfrsf1a	1765.15	1.17	1.59E-33
Tnfrsf1b	15391.36	4.39	0.00E+00
Cd40	475.70	5.70	5.65E-100
Tnfrsf8	66.07	3.71	2.92E-15
Tnfrsf9	53.95	2.47	3.15E-08
Tnfsf9	1729.10	1.22	2.55E-38
Hsp90b1	17647.54	1.36	3.90E-117
Traf2	484.31	0.85	3.90E-08
Traf6	1073.55	0.48	2.03E-05
Psmd3	2894.68	1.23	5.68E-52
Ubc	287025.79	0.41	1.53E-11
Ube2e1	292.37	0.71	5.51E-04
Uba3	502.86	0.76	1.89E-06
Sumo1	923.35	0.70	1.91E-07
Vamp2	480.08	0.59	3.97E-03
Ywhaz	10307.09	1.43	5.40E-80
Ddx58	6391.48	4.61	0.00E+00
Eif4g3	6610.43	0.48	3.66E-11
Arih1	4072.46	0.38	5.70E-07
Psmc4	2470.73	0.28	8.84E-04
Abce1	5087.36	0.74	8.32E-23
Usp18	3979.83	5.75	0.00E+00
Il27	643.66	5.44	2.64E-135
Map2k3	1277.77	0.41	7.66E-05
Map2k4	843.84	0.46	2.58E-04
Map2k7	1448.20	0.42	1.54E-04
Map3k8	518.68	0.87	1.82E-08
Mapk1	2764.54	0.25	1.68E-03
Mapk8	584.53	0.77	1.29E-07
Psma1	1718.45	0.47	1.36E-06
Psma4	1203.96	0.54	5.09E-07
Psma5	435.57	0.80	1.98E-06
Psma6	2553.03	1.13	1.23E-35
Psmb2	1614.36	0.65	1.36E-11
Cul1	1659.18	0.45	7.85E-06
Eif4e2	1905.38	0.60	3.19E-10

Tnfrsf12a	247.34	2.17	3.57E-23
Flnb	12394.46	2.65	0.00E+00
Dusp4	4613.41	2.60	8.31E-246
Ebi3	681.34	1.34	1.61E-21
Ppp2r1a	4892.59	0.54	5.63E-15
Il1f6	26.25	4.39	7.64E-08
Tollip	2027.15	1.28	1.33E-45
Rbx1	444.38	0.65	1.91E-04
Tbk1	2801.53	1.60	1.99E-78
Ube2l6	1184.04	2.49	9.19E-11
Psmd8	8034.09	1.06	2.78E-54
Crlf2	420.96	1.00	1.42E-09
Psmd14	1865.95	1.17	1.45E-32
Psmd6	1226.03	0.56	1.67E-07
Psmd12	2034.72	1.04	3.31E-26
Psmc6	1881.68	0.93	5.52E-24
Peli1	2682.63	3.66	1.30E-301
Tab2	3293.21	0.83	5.61E-23
Psmd11	1598.13	0.97	4.92E-22
Psmd1	3836.24	1.21	5.88E-60
Ppp2r1b	851.01	0.75	6.38E-09
Irak3	1070.83	2.09	1.01E-51
Uba7	1966.31	2.02	5.14E-88
Pik3cb	1863.58	0.81	5.39E-19
Il33	19.98	3.97	5.49E-07
Il23a	118.75	5.36	6.94E-26
Brwd1	3896.91	0.66	1.95E-16

Immune system

gene_name	baseMean	log2FoldChange	p-value
Dnajc3	1942.00	1.38	3.00E-51
Isg15	1768.06	5.80	1.06E-29
Gm11127	1870.57	0.42	4.23E-06
Ube3c	1571.08	0.37	1.34E-04
Fbxw11	2393.98	0.49	7.29E-09
Acly	6460.08	0.66	3.11E-24
Txndc5	3348.81	0.88	2.29E-31
Nckap1l	7972.59	0.46	6.46E-13
Fbxo4	177.41	0.61	1.42E-02
Rapgef1	3500.81	0.76	4.38E-24
Camk2d	787.23	2.46	4.65E-64
Olr1	41.83	5.10	2.25E-09
Atp6v1h	2274.15	1.33	9.28E-50
Irak2	2157.52	1.87	3.11E-85

Txlna	2418.28	0.46	2.54E-08
Sec13	1844.71	0.32	6.37E-04
Abi1	3324.36	1.14	5.44E-43
Atp6v0b	2942.24	0.68	9.23E-14
Actb	133616.46	2.06	0.00E+00
Actg1	34610.51	3.65	0.00E+00
Adam8	4912.70	0.61	6.46E-17
Aldoa	26212.64	0.23	4.59E-05
Tirap	527.99	0.48	1.48E-03
Ampd3	1825.67	1.68	6.83E-66
Ap1g1	2080.00	0.38	1.24E-05
Ap1m1	1929.19	0.38	9.98E-05
Ap2m1	1820.99	0.24	1.23E-02
Birc3	1268.54	1.98	2.04E-72
App	6082.22	0.18	9.59E-03
Aprt	2534.49	0.55	2.71E-10
Rhoa	5803.62	0.39	7.28E-09
Arpc1b	6195.33	0.53	9.08E-15
Atf1	997.98	0.88	3.70E-14
Atf2	1245.48	0.71	5.58E-11
Atp6v1b2	3816.23	0.23	4.08E-03
Atp6v0d1	2153.89	0.25	4.27E-03
Atp6v1e1	1912.71	1.42	7.19E-50
Atp6v0e	1823.33	0.42	1.89E-04
Atp6v0a1	1630.36	1.20	4.23E-36
Atp7a	2927.59	1.10	4.27E-33
B2m	15480.45	0.70	1.47E-33
Bcl10	1441.56	1.71	1.04E-51
Bcl2l1	2311.84	1.58	5.68E-74
C3	1156.67	0.43	1.47E-04
C3ar1	777.73	0.78	4.46E-07
C5ar1	4240.80	4.25	0.00E+00
Cab39	3479.56	0.25	4.84E-03
Anxa2	7593.37	0.66	8.31E-25
Calm1	9977.50	1.10	3.40E-72
Calr	21103.36	0.91	5.34E-45
Canx	17707.20	0.68	1.19E-31
Cap1	13114.73	0.55	4.90E-21
Capza2	2942.54	1.21	1.45E-42
Capzb	4370.06	0.33	2.71E-06
Casp1	421.10	0.47	4.55E-03
Casp4	731.04	2.99	1.28E-90
Casp8	1119.56	0.98	2.43E-17

Ctnnb1	11838.12	1.17	1.42E-73
Cct2	8980.72	0.51	2.10E-16
Cct8	6271.26	0.73	5.08E-21
Cd14	18433.99	1.79	1.13E-190
Cd44	18442.13	3.24	0.00E+00
Cd53	6572.97	1.21	3.51E-43
Cd80	984.41	0.62	2.32E-07
Cd86	65.37	1.73	7.05E-06
Cdc42	6133.43	0.56	3.67E-11
Chuk	1801.62	0.84	3.72E-20
Socs3	2693.34	2.52	1.83E-177
Socs1	34.35	3.82	2.11E-09
Clu	1169.61	0.29	8.38E-03
Ccr2	161.31	1.22	2.60E-06
Crebbp	4902.19	0.49	1.02E-09
Crk	2237.50	0.91	3.71E-24
Crkl	1194.88	0.36	1.12E-03
Csf1	772.08	5.10	1.00E-164
Csf2	137.94	6.81	1.49E-13
Csf2ra	2371.27	1.05	1.81E-29
Csf2rb	2178.62	1.03	7.81E-33
Csf2rb2	1276.72	0.95	4.49E-19
Csf3	12968.04	10.09	0.00E+00
Csnk2b	1990.97	0.33	2.89E-04
Dnajc5	2610.86	0.63	9.81E-15
Cstb	2413.33	1.05	4.62E-32
Ctsb	51966.03	0.40	9.83E-14
Ctsc	735.33	0.70	2.73E-07
Ctsl	20054.77	1.13	3.51E-91
Cyba	3614.66	0.35	4.57E-06
Cybb	30589.95	2.27	1.02E-304
Ddost	2433.70	0.51	7.98E-09
Ddx3x	10031.26	1.08	7.22E-54
Dhx9	5561.16	0.17	2.15E-02
Degs1	2862.01	1.36	6.93E-40
Diaph1	3139.04	0.61	8.05E-13
Dync1i2	2216.65	0.43	1.58E-06
Stom	7478.75	0.58	4.75E-17
Ube2j2	651.95	0.62	5.21E-05
Ptk2	2491.37	0.74	1.24E-17
Fcer1g	4391.26	0.74	3.37E-16
Fcgr2b	3824.90	2.19	1.04E-174
Fgr	1435.70	2.31	9.82E-103

Fpr1	37.12	4.40	1.88E-08
Tlr3	193.18	3.20	3.59E-32
Lilr4b	19538.91	2.11	1.94E-246
Lilrb4a	26318.58	2.18	4.00E-301
Cxcl1	7.83	2.02	5.62E-03
Pdia3	11970.92	1.02	1.95E-53
Hspa5	47533.63	2.54	0.00E+00
Trip12	12808.39	1.46	4.97E-121
Hck	2225.09	2.78	2.13E-163
Hspa1b	445.90	0.88	6.45E-08
Hsp90ab1	48516.80	0.75	1.84E-28
Hsp90aa1	12397.88	1.40	6.17E-110
Icam1	2741.08	1.60	1.17E-85
Ifnar1	1295.45	0.36	5.02E-04
Ifnb1	3.00	1.25	2.29E-02
Ikbkb	1973.97	0.36	7.70E-05
Il10	52.90	5.01	2.66E-10
Il10ra	919.28	0.68	7.69E-08
Il10rb	1186.35	0.95	6.08E-19
Il12rb2	100.94	0.77	1.37E-02
Il13ra2	4.78	1.80	3.48E-03
Il16	1176.57	0.27	2.09E-02
Il17ra	2041.41	0.21	2.29E-02
Il18	95.56	0.83	9.40E-03
Il1a	8860.86	6.99	1.72E-35
Il1b	3010.48	1.83	5.05E-07
Il1rap	318.62	1.47	1.12E-14
Il1rn	842.80	6.71	8.94E-134
Il2rg	1922.11	0.79	3.90E-17
Il6	105.78	6.01	6.70E-14
Il7	8.59	2.41	2.41E-03
Il7r	151.14	1.94	1.10E-11
Kpnb1	9009.07	0.81	3.83E-33
Irf9	1645.00	2.95	1.30E-163
Itgam	9522.85	0.70	2.37E-30
Itgav	3682.65	2.44	6.84E-173
Itgax	44.41	1.72	1.38E-04
Itgb1	4576.19	0.78	3.31E-28
Itgb2	6091.03	0.99	1.85E-44
Cd47	2568.27	1.04	3.75E-34
Jak1	3981.25	0.51	8.58E-10
Jak2	2409.58	2.47	1.30E-157
Jun	5350.55	1.74	1.79E-127

Kif3b	905.55	0.33	5.78E-03
Kif5b	5681.03	0.98	6.28E-38
Lamp2	6310.89	0.28	2.29E-04
Lck	222.73	2.65	2.85E-27
Lcp2	13147.82	2.62	0.00E+00
Lif	3364.66	2.03	1.23E-05
Psmb9	149.93	0.58	2.68E-02
Psmb8	1263.83	0.93	2.69E-18
Lta	46.18	2.22	1.06E-06
Ltb	869.78	1.14	1.47E-15
Ltbr	839.48	0.43	5.15E-04
Znrf1	2207.16	1.64	1.42E-76
Pilrb1	24.29	3.58	5.72E-07
Tlr7	3606.80	0.62	2.96E-12
Il1rl1	401.61	1.33	3.28E-15
Ly96	160.72	1.09	2.90E-05
Lyn	7091.21	1.89	1.38E-172
Mapkapk2	19163.37	1.87	1.05E-193
Masp2	120.95	1.33	3.62E-05
Mif	3682.75	0.53	2.95E-12
Mmp9	283.98	5.55	1.32E-60
Psmd7	2221.96	0.97	6.74E-29
Clec4d	5182.01	3.01	0.00E+00
Mx2	1754.59	8.82	3.72E-109
Myd88	2055.05	1.64	1.93E-75
Myh9	34021.01	0.69	8.31E-28
Myo10	20146.58	2.97	0.00E+00
Ncf1	5921.85	0.19	5.65E-03
Ncf4	2801.03	0.96	5.45E-33
Nck1	466.43	0.99	3.05E-10
Nfkb1	11298.39	3.56	0.00E+00
Nfkb2	5526.89	2.74	0.00E+00
Nfkbia	6463.17	3.18	0.00E+00
Nfkbib	1719.58	2.22	4.57E-92
Nfkbie	552.73	1.09	2.65E-13
Nme2	2709.15	0.33	1.24E-04
Nos2	1298.60	7.52	1.32E-157
Slc11a1	1572.08	1.35	1.65E-39
Nras	3177.60	0.64	6.07E-14
Orm2	57.14	1.74	2.30E-05
Orm3	42.82	2.13	4.48E-06
Sqstm1	31840.37	1.78	1.14E-195
Osm	1661.73	5.61	0.00E+00

P4hb	18103.33	1.31	1.73E-108
Pafah1b2	2611.67	0.57	9.03E-10
Pdcd1	122.73	2.88	9.75E-20
Padi2	210.14	1.11	1.15E-06
Pfkl	4959.96	1.61	1.80E-117
Pgam1	14129.19	1.01	1.26E-42
Pkm	49579.12	0.15	1.12E-02
Plau	1587.81	1.56	1.14E-52
Plaur	8781.40	5.62	0.00E+00
Pld3	2608.10	0.78	4.28E-21
Pa2g4	5103.61	0.44	1.49E-09
Pnp	2366.15	2.67	1.68E-180
Ppp2ca	3481.38	0.73	2.47E-20
Ppp2cb	827.68	0.67	1.47E-07
Ppp3cb	1283.20	0.53	8.57E-06
Eif2ak2	4747.58	3.44	0.00E+00
Psen1	3197.57	0.97	2.04E-36
Psma2	1307.08	0.66	2.73E-10
Psma3	318.87	1.33	2.37E-12
Psmb10	503.81	1.43	3.06E-18
Psmb4	2641.44	0.71	5.96E-17
Psmb5	733.35	0.50	1.81E-04
Psmb6	2414.62	1.16	2.24E-40
Psmb7	2059.92	0.63	1.11E-11
Psmc1	2078.70	0.72	5.03E-16
Psmc2	1905.33	0.70	5.26E-13
Psmc5	1770.72	0.73	2.81E-10
Psme1	897.17	0.79	7.42E-10
Psme2	212.05	1.56	8.07E-12
Psme3	3341.08	0.84	4.21E-20
Ptafr	672.78	2.18	2.41E-53
Eif4a3	1713.45	0.29	2.01E-03
Ptpn11	2343.35	0.49	1.57E-08
Ptpn2	898.52	1.62	4.51E-39
Ptpn22	2073.57	1.46	4.76E-53
Ripk2	553.98	1.29	1.35E-17
Ptprj	15431.10	3.18	0.00E+00
Rab10	3700.67	0.60	1.14E-13
Rab18	1239.01	0.40	1.50E-04
Rab4b	604.66	0.33	2.24E-02
Rab5c	2717.45	0.29	3.67E-04
Rab6a	1668.04	0.85	1.41E-18
Rab7	2022.31	0.56	2.71E-09

Hspa1a	218.57	0.74	1.05E-03
Rbbp6	3952.40	0.32	2.62E-04
Rel	1137.47	2.03	1.07E-69
Rela	2281.64	0.95	6.17E-27
Relb	1042.85	2.40	8.58E-69
Ripk1	1318.15	0.26	1.19E-02
Rlim	6631.10	1.23	3.58E-62
Rnf4	2778.62	0.71	6.88E-18
Rock1	2809.04	0.88	7.32E-24
Rps6ka2	1672.14	3.82	1.01E-249
S100a8	21.70	3.28	4.18E-06
Cxcl2	48373.24	6.83	3.75E-40
Sec22b	933.53	0.59	7.99E-07
Shc1	2224.69	0.51	7.55E-08
Cyfip1	9233.06	1.15	2.70E-67
Siah2	644.99	2.19	3.32E-51
Slpi	10618.25	4.71	0.00E+00
Smarca4	6769.63	0.36	3.95E-07
Siglec1	2756.48	4.44	0.00E+00
Sos2	1952.80	0.68	5.86E-14
Fbxo40	39.68	1.22	7.67E-03
Sptbn2	207.18	0.86	1.72E-04
Dtx4	4358.07	1.49	5.26E-97
Src	766.56	4.82	1.53E-33
Trim21	611.47	3.01	1.31E-83
Stat1	4763.25	3.39	0.00E+00
Stat2	2949.54	3.58	0.00E+00
Stat3	2888.45	1.88	7.48E-121
Stat6	4409.97	0.43	1.69E-09
Eif4g1	19458.44	0.60	7.65E-25
Cblb	452.50	0.95	2.62E-09
Stk10	5362.59	2.00	1.02E-169
Stx3	1917.32	0.68	1.30E-13
Dtx3l	3414.87	2.72	5.63E-220
Surf4	5566.64	1.59	5.44E-85
Syk	3280.07	0.73	2.49E-19
Trim41	1291.03	0.25	1.78E-02
Nhlrc3	436.84	0.57	5.04E-04
Il20rb	42.09	1.39	1.99E-03
Tank	2398.38	2.11	4.74E-131
Tap1	1985.42	2.04	9.37E-105
Tapbp	8779.08	1.94	3.65E-155
Eea1	6710.50	1.82	2.53E-163

Nlrp3	17227.26	4.88	0.00E+00
Trim25	10006.03	1.39	8.59E-110
Cdc27	1938.76	0.50	2.51E-06
Psmd2	4476.34	0.40	1.59E-08
Ube2e2	23.17	2.93	2.66E-06
Sec24c	3566.44	0.58	1.07E-12
Tnf	40207.52	5.55	0.00E+00
Tnfaip3	4575.84	5.25	7.39E-108
Tnfrsf1a	1765.15	1.17	1.59E-33
Tnfrsf1b	15391.36	4.39	0.00E+00
Cd40	475.70	5.70	5.65E-100
Tnfrsf8	66.07	3.71	2.92E-15
Tnfrsf9	53.95	2.47	3.15E-08
Tnfsf9	1729.10	1.22	2.55E-38
Tom1	736.06	0.69	5.21E-07
Tpp2	4269.51	1.18	4.42E-44
Hsp90b1	17647.54	1.36	3.90E-117
Traf2	484.31	0.85	3.90E-08
Traf6	1073.55	0.48	2.03E-05
Psmd3	2894.68	1.23	5.68E-52
Tuba1a	3623.76	0.83	4.79E-28
Tuba1b	10381.77	0.55	2.45E-16
Tuba4a	1072.06	1.03	6.34E-20
Tuba1c	10521.17	0.92	6.34E-46
Txk	46.16	1.30	2.97E-03
Txn1	2863.48	0.43	2.81E-07
Ubc	287025.79	0.41	1.53E-11
Ube2e1	292.37	0.71	5.51E-04
Uba3	502.86	0.76	1.89E-06
Uba1	7125.64	0.60	2.60E-21
Ube2g2	834.60	0.43	1.72E-03
Ube3a	2082.29	0.54	3.08E-08
Sumo1	923.35	0.70	1.91E-07
Vamp2	480.08	0.59	3.97E-03
Vamp3	734.17	0.50	1.32E-04
Vcl	2686.01	1.56	3.28E-79
Vhl	348.17	0.71	9.93E-05
Was	1351.14	0.34	9.36E-04
Pak2	4965.65	1.03	3.54E-50
Enpp4	356.98	3.90	5.02E-70
Ubr2	6163.61	1.00	1.07E-36
Fbxo11	1599.57	0.67	6.41E-11
Ywhaz	10307.09	1.43	5.40E-80

Ppp2r5a	2472.14	1.29	1.64E-50
Tubb4b	1920.27	0.54	6.81E-09
Trib3	2127.18	2.48	4.80E-140
Ddx58	6391.48	4.61	0.00E+00
Eif4g3	6610.43	0.48	3.66E-11
Uba6	1531.25	0.87	1.04E-13
Fbxw8	1975.30	0.26	3.93E-03
Pilra	404.38	4.89	5.72E-88
Clec12a	230.02	0.58	7.69E-03
Dera	330.72	0.64	5.32E-04
Dync1li2	1834.31	0.39	2.28E-05
Dnajc13	4468.80	0.80	4.77E-27
Dync1li1	2414.69	0.74	1.70E-18
Arih1	4072.46	0.38	5.70E-07
Arih2	1062.35	1.05	4.73E-21
Serpina3f	4.62	1.75	4.11E-03
Clec5a	3532.67	1.48	4.65E-86
Fyb	2568.82	0.80	5.64E-17
Impdh1	1824.43	0.89	1.97E-21
Pin1	760.84	0.73	9.02E-08
Psmc4	2470.73	0.28	8.84E-04
Abce1	5087.36	0.74	8.32E-23
Malt1	16381.37	4.32	0.00E+00
Mex3c	749.76	0.55	2.16E-05
Tlr2	6089.24	2.87	0.00E+00
Usp18	3979.83	5.75	0.00E+00
Atp8b4	4944.22	1.07	1.69E-51
Kbtbd8	176.49	1.11	5.94E-06
Tarm1	7.12	2.35	4.65E-04
Cd207	3.12	1.28	2.06E-02
Il27	643.66	5.44	2.64E-135
Ap1s3	494.80	1.59	2.33E-24
Ceacam1	1319.34	1.03	3.18E-22
Ceacam2	363.71	0.76	3.00E-05
Map2k3	1277.77	0.41	7.66E-05
Map2k4	843.84	0.46	2.58E-04
Map2k7	1448.20	0.42	1.54E-04
Map3k8	518.68	0.87	1.82E-08
Mapk1	2764.54	0.25	1.68E-03
Mapk8	584.53	0.77	1.29E-07
Psma1	1718.45	0.47	1.36E-06
Psma4	1203.96	0.54	5.09E-07
Psma5	435.57	0.80	1.98E-06

Psma6	2553.03	1.13	1.23E-35
Psmb2	1614.36	0.65	1.36E-11
Cul3	2422.85	0.68	1.36E-12
Ube2z	1877.63	1.07	1.29E-28
Vcp	3725.70	1.01	9.73E-41
Cul1	1659.18	0.45	7.85E-06
Eif4e2	1905.38	0.60	3.19E-10
Tcirg1	13688.81	1.12	5.54E-74
Tnfrsf12a	247.34	2.17	3.57E-23
Flnb	12394.46	2.65	0.00E+00
Iqgap1	30570.92	0.81	3.66E-44
Rnf19a	659.49	0.93	1.45E-11
Vapa	3650.97	0.80	3.98E-25
Dusp4	4613.41	2.60	8.31E-246
Dcaf1	901.69	1.24	1.83E-24
Rnf182	10.40	2.93	4.82E-05
Ep300	5705.66	0.52	2.04E-13
Treml2	238.09	1.40	9.78E-10
Cxcl3	508.71	6.01	1.07E-24
Aim2	1662.82	0.74	9.02E-15
Trim56	2209.12	1.93	3.89E-107
Ebi3	681.34	1.34	1.61E-21
Icosl	273.70	2.63	5.89E-32
Fbxo15	49.02	4.36	1.87E-12
Nckap1	104.51	1.43	9.36E-06
Ppp2r1a	4892.59	0.54	5.63E-15
Pvr	1371.97	1.59	2.31E-53
Hgsnat	971.04	0.96	8.00E-16
Tax1bp1	2847.50	0.30	5.94E-04
Lair1	126.01	1.63	1.01E-07
Ube2k	1366.71	0.61	1.92E-09
Pla2g6	809.22	0.58	5.41E-06
Sdcbp	9159.84	1.35	8.53E-85
Tuba8	53.23	3.39	2.91E-12
Irf7	5363.58	6.23	0.00E+00
Ywhab	3822.62	0.68	1.31E-20
Il1f6	26.25	4.39	7.64E-08
Tollip	2027.15	1.28	1.33E-45
Pilrb2	24.46	4.04	9.09E-06
Bri3	1305.44	0.50	8.88E-05
Ppie	366.99	0.57	2.40E-03
Rhog	2601.62	0.36	9.49E-06
Ube2j1	1318.46	0.43	4.60E-05

Acpp	4.01	1.57	7.67E-03
Arpc3	2160.84	0.34	6.77E-05
Rbx1	444.38	0.65	1.91E-04
Actr10	955.66	0.57	1.04E-06
Tbk1	2801.53	1.60	1.99E-78
Ikbke	5603.24	3.93	0.00E+00
Ripk3	377.74	0.86	8.68E-07
Ube2d2a	1485.76	0.56	9.28E-07
Lamtor3	459.49	0.55	6.75E-04
Rnf14	1134.05	1.21	4.57E-24
Ube2l6	1184.04	2.49	9.19E-11
Psmd8	8034.09	1.06	2.78E-54
Golga7	1585.68	0.54	1.16E-06
Crlf2	420.96	1.00	1.42E-09
Sh3kbp1	1448.98	1.50	1.17E-49
Trem1	110.24	4.18	2.07E-26
Huwe1	20369.21	0.28	9.40E-06
Psmd14	1865.95	1.17	1.45E-32
Ncstn	1985.10	0.94	7.94E-22
Cd274	1214.38	2.67	3.24E-104
Ctsz	14616.15	0.64	1.05E-27
Asb1	448.93	1.01	3.93E-10
Ube2d3	5144.96	1.49	2.04E-57
Ifitm3	14458.80	1.98	4.74E-177
Atp6v1f	1768.46	0.43	5.39E-06
Atp6v1g2	71.24	1.24	7.18E-04
Smurf2	716.73	0.53	6.47E-05
Atp6v1c1	3713.75	0.38	3.69E-07
Sar1b	560.85	0.47	2.95E-03
Psmd6	1226.03	0.56	1.67E-07
Uba5	1053.56	0.90	4.20E-15
Pgm1	466.28	0.35	2.57E-02
Pnp2	677.34	2.55	2.79E-71
Actr2	9274.62	1.06	5.90E-47
Ube2w	656.23	1.03	5.38E-13
Psmd12	2034.72	1.04	3.31E-26
Magt1	1832.90	0.53	2.52E-08
Psmc6	1881.68	0.93	5.52E-24
Herc6	722.00	3.76	3.58E-121
Cmtm6	913.67	0.73	8.33E-10
Peli1	2682.63	3.66	1.30E-301
Herc4	1810.08	0.64	7.71E-12
Ufl1	713.26	0.64	5.75E-06

Agpat2	224.67	0.56	1.03E-02
Rnf41	1282.50	0.54	2.27E-07
Arpc5	5242.86	0.97	3.07E-38
Ube2f	703.05	1.53	3.70E-29
Eloc	533.31	0.58	1.69E-04
Tubb6	2765.87	2.08	8.24E-141
Sugt1	820.89	0.32	1.17E-02
Npc2	6353.70	0.58	2.84E-18
Arpc4	2517.15	0.56	3.87E-11
Rnaset2b	2151.82	0.37	2.41E-05
Arel1	2066.26	0.54	4.27E-09
Wipf2	1250.59	0.69	1.02E-09
Tab2	3293.21	0.83	5.61E-23
Arl8a	2192.46	0.48	3.22E-07
Psmd11	1598.13	0.97	4.92E-22
Ubr4	18810.72	0.70	1.28E-29
Gsdmd	1390.67	0.46	6.39E-06
Sec31a	4310.99	0.27	3.21E-04
Bst2	2760.02	2.07	8.41E-133
Sec24d	1116.53	2.16	5.23E-72
Tmbim1	1457.58	0.42	4.83E-05
Tmem30a	2107.31	0.38	3.82E-05
Ube2q1	3303.21	0.63	1.30E-15
Psmd1	3836.24	1.21	5.88E-60
Rnf126	1595.14	0.94	2.18E-20
Copb1	3529.92	0.75	5.66E-22
Atp6ap2	1563.80	0.23	1.55E-02
Qpct	183.99	0.77	1.18E-03
Ube2v2	556.31	1.38	4.56E-16
Trim69	15.39	2.03	1.75E-03
Kctd6	163.45	1.16	2.01E-05
Ap2b1	4124.92	0.76	5.34E-26
Fbxo30	482.32	1.19	3.12E-14
Plpp5	552.91	2.07	1.70E-38
Ist1	1536.66	0.68	4.87E-11
Cotl1	3134.58	1.14	1.24E-48
Rap2c	962.42	1.33	4.45E-28
Pgm2	1044.04	1.02	2.85E-16
Dhx36	1373.80	0.30	8.71E-03
Asb6	721.64	1.17	8.16E-19
Pdzd11	492.48	0.60	8.19E-05
Ppp2r1b	851.01	0.75	6.38E-09
Atp6v1d	1327.57	0.38	2.93E-04

Irak3	1070.83	2.09	1.01E-51
Actr3	7031.99	2.26	9.76E-173
Rnf6	1380.91	0.79	4.40E-14
Uba7	1966.31	2.02	5.14E-88
Kcmf1	1131.74	0.50	5.92E-06
Pik3cb	1863.58	0.81	5.39E-19
Rnf19b	1151.26	1.73	8.13E-53
Cul5	1520.76	0.95	7.23E-17
Smurf1	548.64	0.97	3.52E-11
Atp11b	1226.97	0.29	7.41E-03
Erp44	1446.65	0.72	1.03E-12
Faf2	1528.69	0.64	2.39E-10
Mib2	1188.76	0.60	1.07E-07
Arpc2	9397.42	0.70	3.40E-30
Mospd2	720.46	0.61	7.04E-06
Il33	19.98	3.97	5.49E-07
Sec24a	2095.13	0.77	7.99E-19
Ube2s	997.59	1.12	7.93E-20
Rbsn	879.48	0.45	9.30E-04
Mvp	3157.70	0.52	2.36E-10
Rictor	2481.63	0.30	4.30E-04
Wsb1	5914.22	2.20	7.11E-180
Trim39	762.61	0.69	1.54E-07
Rnf34	506.54	0.76	8.35E-07
Erap1	1312.68	0.42	7.58E-05
Gpr84	4194.56	4.39	0.00E+00
Rnf114	1841.06	1.95	3.54E-94
Siglece	70.77	1.75	4.10E-06
Il23a	118.75	5.36	6.94E-26
Pik3ap1	4132.32	1.11	1.16E-47
Nedd4l	167.24	2.10	5.83E-16
Rnf111	2699.26	0.71	4.19E-17
Brwd1	3896.91	0.66	1.95E-16
Pag1	1112.00	0.59	6.69E-08
Sec24b	2165.53	0.60	7.88E-11

Cell cycle

gene_name	baseMean	log2FoldChange	p-value
Xpo1	4315.50	0.33	1.51E-04
Fbxw11	2442.76	0.31	1.89E-04
Csnk1d	3310.89	0.29	2.38E-04
E2f4	1608.07	0.25	1.14E-02
Pom121	2816.47	0.42	8.21E-08
Sec13	2026.59	0.33	2.68E-04

Akt1	6530.69	0.17	1.03E-02
Akt2	1223.87	0.28	1.54E-02
Bub3	2281.39	0.22	8.94E-03
Cdk1	650.24	0.49	3.99E-04
Cdc42	6651.18	0.52	2.43E-03
Cdc45	320.88	0.54	2.72E-03
Cdk6	2421.17	1.15	6.40E-43
Cdkn1a	9584.58	1.19	1.50E-61
Cenpc1	1108.38	0.95	4.16E-18
Csnk2a1	2739.14	0.34	1.07E-03
Csnk2a2	1852.13	0.61	3.76E-10
Dyrk1a	1997.02	0.26	2.87E-03
E2f5	245.34	0.94	4.95E-06
Mapre1	2711.22	0.77	2.13E-19
Emd	1201.25	0.89	3.35E-17
H3f3a	3062.64	0.31	9.70E-04
H3f3b	13024.05	2.26	7.30E-234
Hsp90ab1	44375.41	0.31	3.40E-06
Hsp90aa1	10065.04	0.77	3.95E-32
Hus1	509.17	0.43	5.11E-03
Jak2	2939.45	2.64	9.83E-198
Lmna	12670.08	0.61	4.66E-18
Lyn	4881.06	0.97	8.99E-42
Mcm3	2369.25	0.29	5.16E-04
Mcm4	1560.71	0.30	1.88E-03
Mdm2	6812.40	0.59	2.50E-14
Psmd7	1797.82	0.25	7.09E-03
Ppp1r12a	2467.89	0.43	1.18E-07
Pafah1b1	3937.45	0.60	1.36E-13
Pcm1	2963.89	0.52	1.48E-09
Pcna	1782.34	0.54	1.49E-08
Ppp1cb	2803.09	0.57	4.14E-09
Ppp2ca	3861.66	0.76	1.80E-24
Ppp2cb	894.18	0.64	1.16E-07
Psma2	1265.41	0.36	5.61E-04
Psma3	311.93	1.08	1.47E-08
Psmb10	385.36	0.66	1.56E-04
Psmb4	2558.42	0.41	9.76E-07
Psmb6	2047.96	0.60	2.26E-11
Psmb7	2063.85	0.41	1.99E-06
Psmc1	1873.28	0.24	6.32E-03
Psmc3	2412.33	0.22	9.63E-03
Psmc5	1630.90	0.32	2.14E-03

Psme2	165.00	0.84	3.60E-04
Psme3	3480.39	0.73	3.53E-16
Rab1a	3339.56	0.38	1.86E-06
Ranbp2	8243.35	0.66	5.52E-17
Src	395.80	3.93	2.73E-70
Stag1	1322.24	0.26	1.80E-02
Plk4	768.10	0.53	9.16E-05
Skp1a	1493.43	0.35	1.02E-03
Phlda1	2619.07	6.92	0.00E+00
Taok1	4463.51	0.34	2.11E-06
Cdc27	1986.47	0.33	3.45E-04
Nup153	4584.08	0.48	1.02E-11
Wapl	4590.04	0.30	2.88E-05
Trp53	3271.76	0.32	3.03E-04
Psmd3	2276.28	0.49	9.24E-09
Tuba1a	4351.91	1.03	1.14E-41
Tuba1b	12760.58	0.82	6.56E-31
Tuba1c	13384.08	1.24	2.61E-77
Tubb2a	190.11	0.59	8.57E-03
Ube2e1	311.78	0.64	9.31E-04
Sumo1	895.15	0.40	2.02E-03
Rbbp8	929.05	1.41	1.41E-32
Ywhae	7950.57	0.43	9.43E-12
Ywhag	11250.33	0.94	2.55E-54
Ywhah	4022.35	0.93	2.84E-33
Ywhaq	2585.26	0.23	8.93E-03
Ywhaz	8921.66	0.94	3.15E-33
Ahctf1	3307.55	0.97	8.63E-37
Ppp2r5a	1992.64	0.62	1.17E-11
Tubb4b	2164.10	0.61	1.91E-11
Atrip	416.99	0.74	5.11E-05
Ofd1	293.58	0.62	1.00E-03
Dkc1	2057.51	0.39	2.99E-05
Rbbp7	1813.74	0.50	5.28E-08
Tubgcp3	1260.49	0.51	2.50E-06
Cop1	740.11	0.67	1.27E-07
Psma4	1240.89	0.39	1.57E-04
Psma6	2160.66	0.55	1.03E-08
Psmb2	1548.68	0.33	9.68E-04
Pkmyt1	299.40	0.52	5.62E-03
Nup54	896.43	0.86	4.39E-13
Cul1	1713.32	0.30	1.97E-03
Nup98	6034.35	0.75	2.40E-29

Nbn	380.66	0.80	1.91E-06
Cep63	487.62	0.88	5.04E-09
Cenpt	237.49	0.49	1.43E-02
Lemd3	799.22	0.51	2.61E-05
Cep135	542.93	0.75	1.08E-06
Hdac1	367.02	1.51	1.33E-17
Ppp2r1a	4939.40	0.34	1.18E-06
Zwint	1107.45	0.92	3.52E-16
Dctn3	732.97	0.34	8.79E-03
Diaph2	1794.25	0.40	1.11E-05
Ywhab	3505.61	0.24	1.19E-03
Fkbpl	327.52	0.89	6.87E-07
Rbx1	470.69	0.57	5.93E-04
Ruvbl1	393.06	0.49	2.53E-03
Rps27	352.78	0.45	9.78E-03
Psmd8	7208.76	0.62	1.16E-19
Rnf8	429.35	0.54	5.64E-04
Nup160	1577.74	0.61	5.43E-10
Rab2a	1071.23	0.50	5.85E-06
Psmd14	1572.91	0.58	7.40E-09
Lpin2	1528.16	0.57	1.72E-08
Blzf1	719.38	0.96	2.63E-13
Psmd6	1214.72	0.31	3.95E-03
Pole4	390.65	0.61	3.97E-04
Psmd12	1776.51	0.52	6.74E-08
Psmc6	1658.49	0.43	2.31E-05
Tubb6	3227.78	2.18	1.55E-167
Kmt5a	2630.23	0.21	9.55E-03
Psmd11	1553.21	0.70	1.38E-12
Cdc16	1044.05	0.31	5.76E-03
Gorasp2	2148.19	0.56	9.01E-11
Psmd1	3167.88	0.58	3.74E-14
Ube2v2	430.15	0.63	8.93E-05
Nupl1	1710.01	0.49	1.35E-06
Ppp2r2a	2039.04	0.92	9.93E-25
Seh1l	1233.77	0.37	1.27E-03
Ndc1	856.57	0.32	1.13E-02
Ppp2r1b	869.94	0.59	2.33E-06
Tubb2b	80.00	0.81	9.20E-03
Fgfr1op	729.26	0.54	2.45E-05
Kat5	1153.60	0.76	7.18E-13
Ndel1	1531.31	1.05	5.71E-28

Signaling by Rho GTPases

gene_name	baseMean	log2FoldChange	p-value
Rhoq	2115.79	0.40	4.37E-06
Nckap1l	7972.59	0.46	6.46E-13
Arap3	4480.58	1.63	1.04E-115
Pkn2	1416.94	0.43	5.33E-05
Abr	14624.14	1.51	1.86E-136
Sec13	1844.71	0.32	6.37E-04
Abi1	3324.36	1.14	5.44E-43
Actb	133616.46	2.06	0.00E+00
Actg1	34610.51	3.65	0.00E+00
Rhoa	5803.62	0.39	7.28E-09
Rhob	233.87	2.93	4.53E-35
Rhoc	3227.86	2.54	4.41E-184
Arpc1b	6195.33	0.53	9.08E-15
Bub3	2149.21	0.30	6.50E-04
Calm1	9977.50	1.10	3.40E-72
Ctnna1	2899.71	0.44	4.75E-08
Ctnnb1	11838.12	1.17	1.42E-73
Cdc42	6133.43	0.56	3.67E-11
Arhgap31	959.50	0.39	7.38E-04
Cenpc1	782.12	0.34	8.63E-03
Cyba	3614.66	0.35	4.57E-06
Cybb	30589.95	2.27	1.02E-304
Diaph1	3139.04	0.61	8.05E-13
Dync1i2	2216.65	0.43	1.58E-06
Mapre1	2401.52	0.70	2.35E-16
Ptk2	2491.37	0.74	1.24E-17
Gdi1	1001.33	0.35	2.45E-03
Gna13	4000.71	0.58	1.25E-14
H3f3b	10133.99	1.98	1.66E-171
Kif5b	5681.03	0.98	6.28E-38
Ktn1	1656.97	0.77	4.88E-14
Arhgef2	6362.55	1.30	2.14E-77
Myh9	34021.01	0.69	8.31E-28
Myl6	1706.51	0.92	2.72E-17
Ppp1r12a	2326.61	0.51	2.98E-08
Ncf1	5921.85	0.19	5.65E-03
Ncf4	2801.03	0.96	5.45E-33
Nck1	466.43	0.99	3.05E-10
Pafah1b1	4263.35	0.99	5.97E-36
Pfn1	11719.64	1.28	1.32E-94
Ppp1cb	2678.67	0.68	6.65E-14
Ppp2ca	3481.38	0.73	2.47E-20

Ppp2cb	827.68	0.67	1.47E-07
Flna	35843.34	2.13	7.65E-277
Arhgdia	15232.02	0.65	1.58E-27
Ranbp2	8567.97	0.95	4.06E-34
Rock1	2809.04	0.88	7.32E-24
Rock2	3688.93	0.18	1.66E-02
Cyfip1	9233.06	1.15	2.70E-67
Sos2	1952.80	0.68	5.86E-14
Src	766.56	4.82	1.53E-33
Srf	1464.28	1.16	5.47E-29
Daam1	1343.79	1.92	1.27E-74
Taok1	5063.66	0.85	2.61E-30
Tuba1a	3623.76	0.83	4.79E-28
Tuba1b	10381.77	0.55	2.45E-16
Tuba4a	1072.06	1.03	6.34E-20
Tuba1c	10521.17	0.92	6.34E-46
Trio	3749.21	0.20	7.08E-03
Was	1351.14	0.34	9.36E-04
Pak2	4965.65	1.03	3.54E-50
Nox3	3.51	1.38	1.44E-02
Fam13b	1879.14	1.19	1.25E-31
Ywhae	8183.76	0.72	4.69E-30
Ywhag	15299.78	1.74	3.59E-200
Ywhah	3341.46	0.72	1.93E-18
Ywhaz	10307.09	1.43	5.40E-80
Arhgap30	4061.45	0.30	5.89E-05
Ahctf1	2524.29	0.57	5.56E-12
Ppp2r5a	2472.14	1.29	1.64E-50
Tubb4b	1920.27	0.54	6.81E-09
Arhgap1	1431.32	0.27	8.04E-03
Arhgap25	872.52	0.44	2.66E-04
Dync1li2	1834.31	0.39	2.28E-05
Dync1li1	2414.69	0.74	1.70E-18
Mapk1	2764.54	0.25	1.68E-03
Zw10	1288.21	0.42	6.83E-05
Nup98	6176.36	1.00	1.54E-51
Iqgap1	30570.92	0.81	3.66E-44
Hist1h4c	1908.26	0.36	6.03E-05
Hist1h4n	2597.28	0.22	1.00E-02
Hist4h4	806.80	0.30	2.36E-02
Cenpt	222.74	0.56	1.20E-02
Nckap1	104.51	1.43	9.36E-06
Ppp2r1a	4892.59	0.54	5.63E-15

Zwint	889.49	0.63	9.40E-07
Tuba8	53.23	3.39	2.91E-12
Diaph2	3006.01	1.69	7.86E-99
Arhgef7	9283.42	1.45	2.92E-111
Ywhab	3822.62	0.68	1.31E-20
Rhog	2601.62	0.36	9.49E-06
Arpc3	2160.84	0.34	6.77E-05
Fmnl1	7649.96	0.44	8.85E-11
Nup160	1708.86	1.00	2.41E-22
Actr2	9274.62	1.06	5.90E-47
Arpc5	5242.86	0.97	3.07E-38
Tubb6	2765.87	2.08	8.24E-141
Arpc4	2517.15	0.56	3.87E-11
Wipf2	1250.59	0.69	1.02E-09
Rhou	20.42	1.78	2.45E-03
Arhgef12	2417.12	1.33	1.80E-52
Arap1	11279.08	1.39	1.33E-110
Nup37	341.28	0.70	1.28E-04
Arhgef3	923.25	2.94	2.98E-105
Noxo1	78.06	0.95	7.79E-03
Seh1l	1401.07	0.88	1.93E-17
Ppp2r1b	851.01	0.75	6.38E-09
Actr3	7031.99	2.26	9.76E-173
Akap13	11797.23	1.72	2.12E-131
Arpc2	9397.42	0.70	3.40E-30
Arhgap10	1463.92	0.74	1.36E-12
Ndel1	1679.25	1.43	4.12E-51
Hist2h4	1175.42	0.34	1.57E-03
Kdm1a	1597.81	0.37	1.88E-04

Cell cycle, mitotic

gene_name	baseMean	log2FoldChange	p-value
Fbxw11	2393.98	0.49	7.29E-09
Csnk1d	3033.24	0.29	1.55E-04
E2f4	1664.48	0.56	1.24E-08
Sec13	1844.71	0.32	6.37E-04
Bub3	2149.21	0.30	6.50E-04
Ccnd2	1640.87	0.79	1.98E-13
Cdc42	6133.43	0.56	3.67E-11
Cdk6	1668.85	0.53	3.11E-08
Cdk7	629.21	0.51	3.86E-04
Cdkn1a	7558.25	0.87	2.53E-35
Cenpc1	782.12	0.34	8.63E-03
Csnk2a1	2655.30	0.49	3.25E-06

Csnk2a2	1812.81	0.77	1.24E-16
Csnk2b	1990.97	0.33	2.89E-04
Dync1i2	2216.65	0.43	1.58E-06
E2f5	185.41	0.53	2.44E-02
Mapre1	2401.52	0.70	2.35E-16
Emd	1314.63	1.28	1.71E-32
H3f3b	10133.99	1.98	1.66E-171
Hsp90ab1	48516.80	0.75	1.84E-28
Hsp90aa1	12397.88	1.40	6.17E-110
Jak2	2409.58	2.47	1.30E-157
Psmb9	149.93	0.58	2.68E-02
Psmb8	1263.83	0.93	2.69E-18
Lyn	7091.21	1.89	1.38E-172
Psmd7	2221.96	0.97	6.74E-29
Ppp1r12a	2326.61	0.51	2.98E-08
Orc2	797.75	0.45	3.74E-04
Pafah1b1	4263.35	0.99	5.97E-36
Pcna	1457.33	0.26	1.12E-02
Pola2	1908.34	0.64	1.33E-12
Ppp1cb	2678.67	0.68	6.65E-14
Ppp2ca	3481.38	0.73	2.47E-20
Ppp2cb	827.68	0.67	1.47E-07
Nup88	1507.82	0.45	4.15E-06
Psma2	1307.08	0.66	2.73E-10
Psma3	318.87	1.33	2.37E-12
Psmb10	503.81	1.43	3.06E-18
Psmb4	2641.44	0.71	5.96E-17
Psmb5	733.35	0.50	1.81E-04
Psmb6	2414.62	1.16	2.24E-40
Psmb7	2059.92	0.63	1.11E-11
Psmc1	2078.70	0.72	5.03E-16
Psmc2	1905.33	0.70	5.26E-13
Psmc5	1770.72	0.73	2.81E-10
Psme1	897.17	0.79	7.42E-10
Psme2	212.05	1.56	8.07E-12
Psme3	3341.08	0.84	4.21E-20
Rab1a	3818.30	0.91	5.09E-31
Ranbp2	8567.97	0.95	4.06E-34
Src	766.56	4.82	1.53E-33
Stag1	1313.85	0.47	2.43E-05
Stag2	3652.52	0.67	1.63E-16
Plk4	703.60	0.55	3.61E-05
Phlda1	837.63	5.51	9.13E-174

Taok1	5063.66	0.85	2.61E-30
Cdc27	1938.76	0.50	2.51E-06
Psmd2	4476.34	0.40	1.59E-08
Nup153	3933.48	0.32	1.30E-05
Wapl	4913.70	0.69	1.07E-22
Trp53	3154.33	0.45	5.50E-07
Psmd3	2894.68	1.23	5.68E-52
Tuba1a	3623.76	0.83	4.79E-28
Tuba1b	10381.77	0.55	2.45E-16
Tuba4a	1072.06	1.03	6.34E-20
Tuba1c	10521.17	0.92	6.34E-46
Ubc	287025.79	0.41	1.53E-11
Ube2e1	292.37	0.71	5.51E-04
Ywhae	8183.76	0.72	4.69E-30
Ywhag	15299.78	1.74	3.59E-200
Ahctf1	2524.29	0.57	5.56E-12
Ppp2r5a	2472.14	1.29	1.64E-50
Tubb4b	1920.27	0.54	6.81E-09
Nup188	2572.05	0.63	3.78E-13
Dync1li2	1834.31	0.39	2.28E-05
Dync1li1	2414.69	0.74	1.70E-18
Ofd1	282.96	0.75	1.72E-04
Psmc4	2470.73	0.28	8.84E-04
Tubgcp3	1058.46	0.30	1.27E-02
Mapk1	2764.54	0.25	1.68E-03
Orc4	559.14	0.68	6.68E-06
Psma1	1718.45	0.47	1.36E-06
Psma4	1203.96	0.54	5.09E-07
Psma5	435.57	0.80	1.98E-06
Psma6	2553.03	1.13	1.23E-35
Psmb2	1614.36	0.65	1.36E-11
Nup54	805.86	0.83	6.78E-11
Zw10	1288.21	0.42	6.83E-05
Cul1	1659.18	0.45	7.85E-06
Nup98	6176.36	1.00	1.54E-51
Cep63	453.69	0.93	1.28E-08
Hist1h4c	1908.26	0.36	6.03E-05
Hist1h4n	2597.28	0.22	1.00E-02
Hist4h4	806.80	0.30	2.36E-02
Cenpt	222.74	0.56	1.20E-02
Cep135	437.60	0.46	6.42E-03
Cnep1r1	408.67	0.52	1.75E-03
Hdac1	311.65	1.39	9.54E-13

Orc3	1273.43	0.30	5.54E-03
Ppp2r1a	4892.59	0.54	5.63E-15
Zwint	889.49	0.63	9.40E-07
Anapc16	372.61	0.67	2.13E-04
Tuba8	53.23	3.39	2.91E-12
Diaph2	3006.01	1.69	7.86E-99
Ensa	2436.85	0.72	1.29E-17
Rbx1	444.38	0.65	1.91E-04
Psmd8	8034.09	1.06	2.78E-54
Nup160	1708.86	1.00	2.41E-22
Rab2a	904.04	0.31	1.80E-02
Psmd14	1865.95	1.17	1.45E-32
Blzf1	715.80	1.15	8.17E-18
Psmd6	1226.03	0.56	1.67E-07
Ccnh	536.43	0.46	1.82E-03
Pole4	500.90	1.35	2.37E-15
Psmd12	2034.72	1.04	3.31E-26
Psmc6	1881.68	0.93	5.52E-24
Tubb6	2765.87	2.08	8.24E-141
Kmt5a	2697.46	0.51	3.10E-10
Psmd11	1598.13	0.97	4.92E-22
Nup37	341.28	0.70	1.28E-04
Gorasp2	2383.26	1.00	8.42E-32
Psmd1	3836.24	1.21	5.88E-60
Optn	39.84	1.53	9.44E-04
Nupl1	1837.54	0.87	2.09E-17
Ppp2r2a	2064.71	1.15	2.71E-38
Seh1l	1401.07	0.88	1.93E-17
Ppme1	1481.80	0.99	1.12E-22
Ppp2r1b	851.01	0.75	6.38E-09
Esco1	763.79	0.52	1.59E-04
Ndel1	1679.25	1.43	4.12E-51
Hist2h4	1175.42	0.34	1.57E-03

M phase

gene_name	baseMean	log2FoldChange	p-value
Csnk1d	3033.24	0.29	1.55E-04
Sec13	1844.71	0.32	6.37E-04
Bub3	2149.21	0.30	6.50E-04
Cdc42	6133.43	0.56	3.67E-11
Cenpc1	782.12	0.34	8.63E-03
Csnk2a1	2655.30	0.49	3.25E-06
Csnk2a2	1812.81	0.77	1.24E-16
Csnk2b	1990.97	0.33	2.89E-04

Dync1i2	2216.65	0.43	1.58E-06
Mapre1	2401.52	0.70	2.35E-16
Emd	1314.63	1.28	1.71E-32
H3f3b	10133.99	1.98	1.66E-171
Hsp90aa1	12397.88	1.40	6.17E-110
Psmb9	149.93	0.58	2.68E-02
Psmb8	1263.83	0.93	2.69E-18
Psmd7	2221.96	0.97	6.74E-29
Pafah1b1	4263.35	0.99	5.97E-36
Ppp2ca	3481.38	0.73	2.47E-20
Ppp2cb	827.68	0.67	1.47E-07
Nup88	1507.82	0.45	4.15E-06
Psma2	1307.08	0.66	2.73E-10
Psma3	318.87	1.33	2.37E-12
Psmb10	503.81	1.43	3.06E-18
Psmb4	2641.44	0.71	5.96E-17
Psmb5	733.35	0.50	1.81E-04
Psmb6	2414.62	1.16	2.24E-40
Psmb7	2059.92	0.63	1.11E-11
Psmc1	2078.70	0.72	5.03E-16
Psmc2	1905.33	0.70	5.26E-13
Psmc5	1770.72	0.73	2.81E-10
Psme1	897.17	0.79	7.42E-10
Psme2	212.05	1.56	8.07E-12
Psme3	3341.08	0.84	4.21E-20
Rab1a	3818.30	0.91	5.09E-31
Ranbp2	8567.97	0.95	4.06E-34
Stag1	1313.85	0.47	2.43E-05
Stag2	3652.52	0.67	1.63E-16
Plk4	703.60	0.55	3.61E-05
Taok1	5063.66	0.85	2.61E-30
Cdc27	1938.76	0.50	2.51E-06
Psmd2	4476.34	0.40	1.59E-08
Nup153	3933.48	0.32	1.30E-05
Wapl	4913.70	0.69	1.07E-22
Psmd3	2894.68	1.23	5.68E-52
Tuba1a	3623.76	0.83	4.79E-28
Tuba1b	10381.77	0.55	2.45E-16
Tuba4a	1072.06	1.03	6.34E-20
Tuba1c	10521.17	0.92	6.34E-46
Ubc	287025.79	0.41	1.53E-11
Ube2e1	292.37	0.71	5.51E-04
Ywhae	8183.76	0.72	4.69E-30

Ywhag	15299.78	1.74	3.59E-200
Ahctf1	2524.29	0.57	5.56E-12
Ppp2r5a	2472.14	1.29	1.64E-50
Tubb4b	1920.27	0.54	6.81E-09
Nup188	2572.05	0.63	3.78E-13
Dync1li2	1834.31	0.39	2.28E-05
Dync1li1	2414.69	0.74	1.70E-18
Ofd1	282.96	0.75	1.72E-04
Psmc4	2470.73	0.28	8.84E-04
Tubgcp3	1058.46	0.30	1.27E-02
Mapk1	2764.54	0.25	1.68E-03
Psma1	1718.45	0.47	1.36E-06
Psma4	1203.96	0.54	5.09E-07
Psma5	435.57	0.80	1.98E-06
Psma6	2553.03	1.13	1.23E-35
Psmb2	1614.36	0.65	1.36E-11
Nup54	805.86	0.83	6.78E-11
Zw10	1288.21	0.42	6.83E-05
Nup98	6176.36	1.00	1.54E-51
Cep63	453.69	0.93	1.28E-08
Hist1h4c	1908.26	0.36	6.03E-05
Hist1h4n	2597.28	0.22	1.00E-02
Hist4h4	806.80	0.30	2.36E-02
Cenpt	222.74	0.56	1.20E-02
Cep135	437.60	0.46	6.42E-03
Cnep1r1	408.67	0.52	1.75E-03
Ppp2r1a	4892.59	0.54	5.63E-15
Zwint	889.49	0.63	9.40E-07
Anapc16	372.61	0.67	2.13E-04
Tuba8	53.23	3.39	2.91E-12
Diaph2	3006.01	1.69	7.86E-99
Ensa	2436.85	0.72	1.29E-17
Psmd8	8034.09	1.06	2.78E-54
Nup160	1708.86	1.00	2.41E-22
Rab2a	904.04	0.31	1.80E-02
Psmd14	1865.95	1.17	1.45E-32
Blzf1	715.80	1.15	8.17E-18
Psmd6	1226.03	0.56	1.67E-07
Psmd12	2034.72	1.04	3.31E-26
Psmc6	1881.68	0.93	5.52E-24
Tubb6	2765.87	2.08	8.24E-141
Kmt5a	2697.46	0.51	3.10E-10
Psmd11	1598.13	0.97	4.92E-22

Nup37	341.28	0.70	1.28E-04
Gorasp2	2383.26	1.00	8.42E-32
Psmd1	3836.24	1.21	5.88E-60
Nupl1	1837.54	0.87	2.09E-17
Seh1l	1401.07	0.88	1.93E-17
Ppp2r1b	851.01	0.75	6.38E-09
Ndel1	1679.25	1.43	4.12E-51
Hist2h4	1175.42	0.34	1.57E-03

Up-regulated genes following 4 h *P. aeruginosa* infection

Metabolism of RNA

gene_name	baseMean	log2FoldChange	p-value
Noc4l	985.00	0.54	8.74E-07
Sf3b3	10469.20	0.26	8.29E-06
Csnk1d	3644.41	0.45	2.03E-11
Cnot6	2355.81	0.24	5.93E-03
Tsr1	1249.06	0.80	9.31E-13
Utp15	1486.04	0.80	1.65E-16
Sf3b4	1954.24	0.91	1.31E-27
Pom121	2701.83	0.24	1.45E-03
Srsf9	1574.35	0.36	1.24E-04
Cstf2	1316.75	0.51	1.03E-06
Srsf1	5769.91	1.03	4.17E-51
Pwp2	1349.06	1.06	1.13E-25
Tgs1	1611.83	1.02	4.18E-25
Aqr	2651.77	0.25	9.35E-04
Hnrnpd	3939.23	0.86	2.48E-31
Bop1	2265.35	0.42	1.66E-07
Cdk7	876.75	0.99	1.07E-16
Dhx15	5227.94	0.75	5.63E-32
Ddx5	14016.10	1.26	5.13E-126
Ddx6	6882.50	1.02	1.64E-66
Ercc3	1037.65	0.37	1.13E-03
Fbl	967.01	0.47	4.17E-05
Emg1	1414.86	0.37	7.93E-05
Hnrnpc	4561.55	0.51	5.11E-13
Hnrnpk	6459.99	0.88	2.52E-44
Hspa1b	772.16	1.73	3.43E-39
Elavl1	3094.62	0.65	1.51E-19
Rps2	4305.82	0.19	4.06E-03
Psmb8	1166.16	0.47	9.47E-06
Magoh	683.62	0.57	1.38E-05
Mapkapk2	23577.27	2.03	5.57E-288
Psmd7	2212.34	0.69	1.50E-17

Ncl	38692.94	0.81	6.76E-60
Pdcd11	5067.15	0.52	4.13E-16
Prkcd	4039.36	0.37	5.16E-08
Papola	6068.95	0.26	2.23E-05
Cnot7	1542.16	1.34	3.54E-38
Ppp2ca	4029.10	0.79	4.97E-27
Psma2	1343.58	0.43	1.64E-05
Psma3	354.28	1.30	5.59E-14
Psmb10	507.80	1.20	6.78E-14
Psmb4	2685.91	0.46	3.95E-09
Psmb5	792.75	0.39	1.22E-03
Psmb6	2313.64	0.81	3.56E-23
Psmb7	2149.34	0.44	6.54E-08
Psmc1	2058.07	0.41	9.64E-07
Psmc5	1791.53	0.48	4.54E-07
Psme1	931.08	0.60	5.28E-07
Psme2	245.19	1.59	8.80E-14
Psme3	3722.23	0.81	3.69E-23
Ptbp1	6182.74	0.60	2.07E-22
Casc3	1408.44	0.34	3.61E-04
Hspa1a	388.47	1.65	3.79E-18
Ranbp2	9575.43	0.93	7.90E-36
Utp14b	622.70	0.82	1.14E-09
Upf1	3865.56	0.16	1.96E-02
Rnps1	1332.28	0.51	5.55E-07
Polr2c	339.37	0.59	7.48E-04
Rps18	1203.32	0.41	6.78E-04
Rps7	3939.20	0.21	3.27E-03
Sf3a2	1694.45	0.62	4.85E-12
Srsf2	4773.91	1.17	1.34E-68
Srsf3	3279.26	1.50	3.90E-76
Srsf5	4850.11	0.76	1.53E-33
Tra2b	1680.87	1.18	8.83E-26
Smn1	726.49	0.68	9.48E-08
Snrpd1	869.79	0.79	6.95E-11
Snu13	2209.83	0.88	1.89E-23
Eif4g1	20270.33	0.41	1.43E-13
Tbl3	1072.45	0.60	1.52E-08
Bms1	2951.39	0.46	3.05E-10
Tut7	2479.75	0.48	1.07E-07
Diexf	1447.13	0.38	6.69E-05
Gemin5	1333.65	0.21	2.71E-02
Utp6	1531.88	0.72	3.92E-14

Utp18	1314.38	0.67	1.81E-10
Utp4	2200.51	0.77	2.47E-20
Heatr1	3779.20	0.51	2.28E-12
Nup153	4326.68	0.25	1.09E-04
Ttc37	1271.13	0.25	2.00E-02
Psmd3	2519.29	0.66	2.45E-17
Zrsr2	1016.59	0.64	3.25E-09
U2af2	4756.99	0.48	2.66E-09
Dcaf13	1425.76	1.13	1.52E-30
Wbp4	888.78	0.61	6.65E-08
Srsf7	2571.25	1.24	6.37E-58
Thoc1	1032.15	0.78	2.42E-12
Wdr36	2263.62	0.99	1.44E-29
Patl1	2509.51	0.32	3.88E-05
Ywhaz	10649.44	1.26	8.92E-116
Smg7	4065.57	1.07	3.08E-50
Rpp38	229.57	0.56	6.93E-03
Hnrnpa3	7031.24	0.79	2.73E-27
Smg5	2476.05	0.46	1.89E-09
Prpf38a	790.31	0.78	4.53E-10
Bud31	537.24	0.73	2.50E-06
Cnot3	1591.43	0.30	1.58E-03
Fus	6240.10	1.23	3.63E-78
Edc4	2422.34	0.54	2.68E-10
Gtf2h2	389.93	0.62	1.71E-04
Pcbp1	8736.22	0.26	2.72E-06
Xrn1	2504.93	0.57	2.51E-11
Xrn2	3914.68	0.79	1.26E-23
Psma1	1808.96	0.29	1.49E-03
Psma4	1348.83	0.52	2.61E-07
Psma5	425.84	0.47	3.21E-03
Psma6	2635.79	0.94	1.38E-29
Psmb2	1633.25	0.39	1.87E-05
Rps15a	4320.57	0.39	4.42E-07
Cpsf7	2517.61	0.68	1.10E-17
Nup54	924.41	0.86	6.08E-14
Wdr3	3095.92	0.26	2.24E-04
Tex10	1221.08	0.49	3.36E-06
Nup98	6176.41	0.73	2.56E-34
Txnl4a	2714.25	0.34	1.46E-05
Lsm2	597.42	0.39	3.99E-03
Rrp9	1099.64	1.38	3.00E-37
Imp4	1633.35	0.46	8.80E-07

Gnl3	3828.80	0.55	1.49E-15
Snrnp200	5967.51	0.19	2.45E-03
Thoc2	3262.88	0.27	3.87E-04
Ltv1	1037.90	0.93	8.15E-18
Thoc6	427.78	0.48	6.25E-03
Lsm4	910.76	0.35	4.38E-03
Exosc10	2170.13	0.72	1.56E-15
Pdcd7	417.26	0.85	1.06E-07
Cpsf2	2668.51	0.76	8.19E-23
Ppp2r1a	4925.40	0.25	6.13E-05
Srrm1	6026.02	0.32	4.58E-07
Hnrnpu	23734.98	0.53	1.60E-23
Krr1	1196.26	0.29	3.56E-03
Cnot11	1005.57	0.51	3.61E-06
Plrg1	1274.91	0.36	2.42E-04
Nxf1	2586.77	0.40	3.84E-07
Hnrnpa2b1	13935.85	0.41	2.27E-09
Bysl	1282.73	0.34	1.26E-03
Cnot4	1349.77	0.41	5.38E-05
Ddx39b	5576.43	0.21	6.19E-04
Rpp30	199.70	0.80	5.46E-04
Ywhab	3750.78	0.34	4.19E-07
Nop58	3976.73	1.30	2.86E-55
Prpf40a	3695.16	0.65	1.37E-17
Ddx21	9345.51	0.62	2.26E-27
Hnrnph2	2165.43	0.67	3.02E-10
Mettl3	456.55	0.74	6.08E-06
Adar	5765.54	2.17	9.94E-230
Hbs1l	1461.31	0.35	1.83E-04
Psmd8	7369.33	0.60	2.32E-22
C1d	377.48	0.80	1.46E-06
Wdr12	908.73	0.62	4.83E-08
Isy1	806.28	0.59	1.33E-06
Hnrnph1	3814.23	0.36	1.15E-06
Nup160	1672.91	0.68	2.30E-13
Rcl1	1041.48	1.04	1.77E-20
Psmd14	1783.24	0.81	6.08E-18
Rbm8a	1248.88	0.49	8.88E-07
Wtap	2378.53	0.90	1.16E-29
Dhx38	2677.93	0.54	1.78E-12
Utp3	1713.65	0.22	1.99E-02
Sf3b6	860.71	0.56	2.84E-06
Sarnp	213.36	0.68	3.98E-03

Pno1	922.37	0.72	2.05E-09
Snw1	1992.63	0.22	6.97E-03
Exosc3	452.17	0.94	1.01E-09
Psmd6	1368.56	0.53	3.68E-08
Rps23	4375.83	0.29	1.53E-05
Rps21	1789.99	0.20	2.53E-02
Chtop	3499.28	0.57	1.18E-14
Exosc1	978.74	0.64	6.74E-09
Snrnp40	1011.37	0.56	1.86E-07
Snrnp27	680.08	0.47	1.90E-04
Rbm22	1414.75	0.45	2.81E-06
Tmem223	52.62	0.94	2.63E-02
Riok3	1561.16	0.79	5.35E-17
Psmd12	2035.76	0.77	2.75E-18
Psmc6	1783.81	0.53	1.50E-09
Nop56	5493.35	0.71	5.23E-29
Zcrb1	725.32	0.91	1.01E-12
Rnpc3	696.72	1.43	1.03E-28
Cwc27	895.57	0.32	1.27E-02
Sf3a1	3147.41	0.39	6.80E-08
Cwc25	750.55	0.47	2.43E-04
Ccar1	2962.48	0.49	2.02E-11
Polr2g	816.51	0.39	8.80E-04
Ddx47	1166.16	0.34	1.45E-03
Snrnp48	909.91	0.34	7.11E-03
Rnmt	1940.88	0.36	2.28E-05
Rps27l	1216.40	0.36	1.12E-03
U2surp	3972.91	0.40	3.64E-08
Puf60	2194.11	0.69	9.09E-18
Mphosph10	1832.72	0.45	4.96E-06
Srsf6	5807.88	0.81	2.29E-39
Ddx39	1900.22	0.62	1.82E-13
Mphosph6	416.06	1.65	7.18E-24
Gtf2f2	680.81	0.63	2.32E-06
Nol11	1268.10	0.96	4.16E-22
Snrpa1	1048.08	0.45	2.98E-05
Prpf31	2662.25	0.32	3.90E-05
Ebna1bp2	1292.07	0.73	1.26E-13
Psmd11	1660.14	0.78	2.26E-16
Srsf11	3608.26	0.34	1.17E-05
Polr2d	298.19	0.82	2.25E-05
Nup37	363.19	0.56	8.64E-04
Fyttd1	1137.06	0.61	9.51E-09

Prpf4	774.07	0.50	6.40E-05
Psmd1	3764.89	0.91	5.37E-41
Zc3h11a	5375.55	0.51	8.08E-15
Dcp2	2118.65	1.73	2.31E-94
Utp20	4193.18	0.60	7.27E-18
Prpf3	1564.26	0.78	2.35E-17
Riok1	791.75	0.81	2.64E-11
Nupl1	2061.16	0.86	9.51E-21
Ppp2r2a	2058.86	0.88	1.75E-26
Ddx42	2212.03	0.58	8.47E-13
Cnot2	1391.52	0.53	2.12E-07
Seh1l	1620.64	0.94	4.50E-25
Mtrex	1996.99	0.44	7.12E-06
Wdr43	4018.39	0.96	1.96E-46
Dis3	1120.28	0.89	3.89E-16
Thoc3	749.74	0.42	7.04E-04
Wdr75	1799.72	0.84	1.30E-18
Fcf1	493.77	0.55	1.74E-04
Poldip3	3631.68	0.62	9.46E-20
Nol9	1885.40	0.34	9.09E-05
Wdr33	2275.36	0.18	2.66E-02
Ddx23	3324.21	0.17	2.29E-02
Pcf11	3523.37	0.99	1.12E-46
Rrp7a	1302.16	0.73	4.73E-13
Sf3a3	1425.41	0.25	9.83E-03
Nop14	1764.72	0.58	1.28E-09
Dcp1a	1598.48	0.90	1.37E-23
Srrm2	38062.99	0.37	2.31E-12
Smndc1	1665.75	0.90	6.25E-24
Lsm8	387.62	0.40	1.44E-02
Hnrnpm	4706.48	0.35	1.42E-06
Hnrnpa0	4499.69	0.77	1.79E-26
Ddx52	1086.70	0.40	3.13E-04
Lsm6	510.04	0.46	1.79E-03
Cwc22	2074.60	0.42	3.36E-07
Senp3	2684.48	0.24	1.42E-03
Sf3b1	9836.42	0.78	2.84E-41
Cstf2t	1679.29	0.37	2.18E-04
Rbm5	3071.62	0.45	1.19E-09
Gtf2f1	1690.01	0.50	3.74E-08
Hnrnpf	15129.33	0.65	2.39E-35
Clp1	323.88	0.45	1.66E-02

Processing of capped intron-containing pre-mRNA

gene_name	baseMean	log2FoldChange	p-value
Sf3b3	10469.20	0.26	8.29E-06
Sf3b4	1954.24	0.91	1.31E-27
Pom121	2701.83	0.24	1.45E-03
Srsf9	1574.35	0.36	1.24E-04
Cstf2	1316.75	0.51	1.03E-06
Srsf1	5769.91	1.03	4.17E-51
Aqr	2651.77	0.25	9.35E-04
Hnrnpd	3939.23	0.86	2.48E-31
Dhx15	5227.94	0.75	5.63E-32
Ddx5	14016.10	1.26	5.13E-126
Hnrnpc	4561.55	0.51	5.11E-13
Hnrnpk	6459.99	0.88	2.52E-44
Elavl1	3094.62	0.65	1.51E-19
Magoh	683.62	0.57	1.38E-05
Papola	6068.95	0.26	2.23E-05
Ptbp1	6182.74	0.60	2.07E-22
Casc3	1408.44	0.34	3.61E-04
Ranbp2	9575.43	0.93	7.90E-36
Rnps1	1332.28	0.51	5.55E-07
Polr2c	339.37	0.59	7.48E-04
Sf3a2	1694.45	0.62	4.85E-12
Srsf2	4773.91	1.17	1.34E-68
Srsf3	3279.26	1.50	3.90E-76
Srsf5	4850.11	0.76	1.53E-33
Tra2b	1680.87	1.18	8.83E-26
Snrpd1	869.79	0.79	6.95E-11
Snu13	2209.83	0.88	1.89E-23
Nup153	4326.68	0.25	1.09E-04
Zrsr2	1016.59	0.64	3.25E-09
U2af2	4756.99	0.48	2.66E-09
Wbp4	888.78	0.61	6.65E-08
Srsf7	2571.25	1.24	6.37E-58
Thoc1	1032.15	0.78	2.42E-12
Hnrnpa3	7031.24	0.79	2.73E-27
Prpf38a	790.31	0.78	4.53E-10
Bud31	537.24	0.73	2.50E-06
Fus	6240.10	1.23	3.63E-78
Pcbp1	8736.22	0.26	2.72E-06
Cpsf7	2517.61	0.68	1.10E-17
Nup54	924.41	0.86	6.08E-14
Nup98	6176.41	0.73	2.56E-34
Txnl4a	2714.25	0.34	1.46E-05

Lsm2	597.42	0.39	3.99E-03
Snrnp200	5967.51	0.19	2.45E-03
Thoc2	3262.88	0.27	3.87E-04
Thoc6	427.78	0.48	6.25E-03
Lsm4	910.76	0.35	4.38E-03
Pdcd7	417.26	0.85	1.06E-07
Cpsf2	2668.51	0.76	8.19E-23
Srrm1	6026.02	0.32	4.58E-07
Hnrnpu	23734.98	0.53	1.60E-23
Plrg1	1274.91	0.36	2.42E-04
Nxf1	2586.77	0.40	3.84E-07
Hnrnpa2b1	13935.85	0.41	2.27E-09
Ddx39b	5576.43	0.21	6.19E-04
Prpf40a	3695.16	0.65	1.37E-17
Hnrnph2	2165.43	0.67	3.02E-10
Mettl3	456.55	0.74	6.08E-06
Isy1	806.28	0.59	1.33E-06
Hnrnph1	3814.23	0.36	1.15E-06
Nup160	1672.91	0.68	2.30E-13
Rbm8a	1248.88	0.49	8.88E-07
Wtap	2378.53	0.90	1.16E-29
Dhx38	2677.93	0.54	1.78E-12
Sf3b6	860.71	0.56	2.84E-06
Sarnp	213.36	0.68	3.98E-03
Snw1	1992.63	0.22	6.97E-03
Chtop	3499.28	0.57	1.18E-14
Snrnp40	1011.37	0.56	1.86E-07
Snrnp27	680.08	0.47	1.90E-04
Rbm22	1414.75	0.45	2.81E-06
Tmem223	52.62	0.94	2.63E-02
Zcrb1	725.32	0.91	1.01E-12
Rnpc3	696.72	1.43	1.03E-28
Cwc27	895.57	0.32	1.27E-02
Sf3a1	3147.41	0.39	6.80E-08
Cwc25	750.55	0.47	2.43E-04
Ccar1	2962.48	0.49	2.02E-11
Polr2g	816.51	0.39	8.80E-04
Snrnp48	909.91	0.34	7.11E-03
U2surp	3972.91	0.40	3.64E-08
Puf60	2194.11	0.69	9.09E-18
Srsf6	5807.88	0.81	2.29E-39
Ddx39	1900.22	0.62	1.82E-13
Gtf2f2	680.81	0.63	2.32E-06

Snrpa1	1048.08	0.45	2.98E-05
Prpf31	2662.25	0.32	3.90E-05
Srsf11	3608.26	0.34	1.17E-05
Polr2d	298.19	0.82	2.25E-05
Nup37	363.19	0.56	8.64E-04
Fyttd1	1137.06	0.61	9.51E-09
Prpf4	774.07	0.50	6.40E-05
Zc3h11a	5375.55	0.51	8.08E-15
Prpf3	1564.26	0.78	2.35E-17
Nupl1	2061.16	0.86	9.51E-21
Ddx42	2212.03	0.58	8.47E-13
Seh1l	1620.64	0.94	4.50E-25
Thoc3	749.74	0.42	7.04E-04
Poldip3	3631.68	0.62	9.46E-20
Wdr33	2275.36	0.18	2.66E-02
Ddx23	3324.21	0.17	2.29E-02
Pcf11	3523.37	0.99	1.12E-46
Sf3a3	1425.41	0.25	9.83E-03
Srrm2	38062.99	0.37	2.31E-12
Smndc1	1665.75	0.90	6.25E-24
Lsm8	387.62	0.40	1.44E-02
Hnrnpm	4706.48	0.35	1.42E-06
Hnrnpa0	4499.69	0.77	1.79E-26
Lsm6	510.04	0.46	1.79E-03
Cwc22	2074.60	0.42	3.36E-07
Sf3b1	9836.42	0.78	2.84E-41
Cstf2t	1679.29	0.37	2.18E-04
Rbm5	3071.62	0.45	1.19E-09
Gtf2f1	1690.01	0.50	3.74E-08
Hnrnpf	15129.33	0.65	2.39E-35
Clp1	323.88	0.45	1.66E-02

Major pathway of rRNA processing in the nucleolus and cytosol

gene_name	baseMean	log2FoldChange	p-value
Noc4l	985.00	0.54	8.74E-07
Csnk1d	3644.41	0.45	2.03E-11
Tsr1	1249.06	0.80	9.31E-13
Utp15	1486.04	0.80	1.65E-16
Pwp2	1349.06	1.06	1.13E-25
Bop1	2265.35	0.42	1.66E-07
Fbl	967.01	0.47	4.17E-05
Emg1	1414.86	0.37	7.93E-05
Rps2	4305.82	0.19	4.06E-03
Ncl	38692.94	0.81	6.76E-60

Pdcd11	5067.15	0.52	4.13E-16
Utp14b	622.70	0.82	1.14E-09
Rps18	1203.32	0.41	6.78E-04
Rps7	3939.20	0.21	3.27E-03
Snu13	2209.83	0.88	1.89E-23
Tbl3	1072.45	0.60	1.52E-08
Bms1	2951.39	0.46	3.05E-10
Diexf	1447.13	0.38	6.69E-05
Utp6	1531.88	0.72	3.92E-14
Utp18	1314.38	0.67	1.81E-10
Utp4	2200.51	0.77	2.47E-20
Heatr1	3779.20	0.51	2.28E-12
Dcaf13	1425.76	1.13	1.52E-30
Wdr36	2263.62	0.99	1.44E-29
Rpp38	229.57	0.56	6.93E-03
Xrn2	3914.68	0.79	1.26E-23
Rps15a	4320.57	0.39	4.42E-07
Wdr3	3095.92	0.26	2.24E-04
Tex10	1221.08	0.49	3.36E-06
Rrp9	1099.64	1.38	3.00E-37
Imp4	1633.35	0.46	8.80E-07
Gnl3	3828.80	0.55	1.49E-15
Ltv1	1037.90	0.93	8.15E-18
Exosc10	2170.13	0.72	1.56E-15
Krr1	1196.26	0.29	3.56E-03
Bysl	1282.73	0.34	1.26E-03
Rpp30	199.70	0.80	5.46E-04
Nop58	3976.73	1.30	2.86E-55
Ddx21	9345.51	0.62	2.26E-27
C1d	377.48	0.80	1.46E-06
Wdr12	908.73	0.62	4.83E-08
Rcl1	1041.48	1.04	1.77E-20
Utp3	1713.65	0.22	1.99E-02
Pno1	922.37	0.72	2.05E-09
Exosc3	452.17	0.94	1.01E-09
Rps23	4375.83	0.29	1.53E-05
Rps21	1789.99	0.20	2.53E-02
Exosc1	978.74	0.64	6.74E-09
Riok3	1561.16	0.79	5.35E-17
Nop56	5493.35	0.71	5.23E-29
Ddx47	1166.16	0.34	1.45E-03
Rps27l	1216.40	0.36	1.12E-03
Mphosph10	1832.72	0.45	4.96E-06

Mphosph6	416.06	1.65	7.18E-24
Nol11	1268.10	0.96	4.16E-22
Ebna1bp2	1292.07	0.73	1.26E-13
Utp20	4193.18	0.60	7.27E-18
Riok1	791.75	0.81	2.64E-11
Mtrex	1996.99	0.44	7.12E-06
Wdr43	4018.39	0.96	1.96E-46
Wdr75	1799.72	0.84	1.30E-18
Fcf1	493.77	0.55	1.74E-04
Nol9	1885.40	0.34	9.09E-05
Rrp7a	1302.16	0.73	4.73E-13
Nop14	1764.72	0.58	1.28E-09
Ddx52	1086.70	0.40	3.13E-04
Senp3	2684.48	0.24	1.42E-03

rRNA processing

gene_name	baseMean	log2FoldChange	p-value
Noc4l	985.00	0.54	8.74E-07
Csnk1d	3644.41	0.45	2.03E-11
Tsr1	1249.06	0.80	9.31E-13
Utp15	1486.04	0.80	1.65E-16
Pwp2	1349.06	1.06	1.13E-25
Bop1	2265.35	0.42	1.66E-07
Fbl	967.01	0.47	4.17E-05
Emg1	1414.86	0.37	7.93E-05
Rps2	4305.82	0.19	4.06E-03
Ncl	38692.94	0.81	6.76E-60
Pdcd11	5067.15	0.52	4.13E-16
Utp14b	622.70	0.82	1.14E-09
Rps18	1203.32	0.41	6.78E-04
Rps7	3939.20	0.21	3.27E-03
Snu13	2209.83	0.88	1.89E-23
Tbl3	1072.45	0.60	1.52E-08
Bms1	2951.39	0.46	3.05E-10
Diexf	1447.13	0.38	6.69E-05
Utp6	1531.88	0.72	3.92E-14
Utp18	1314.38	0.67	1.81E-10
Utp4	2200.51	0.77	2.47E-20
Heatr1	3779.20	0.51	2.28E-12
Dcaf13	1425.76	1.13	1.52E-30
Wdr36	2263.62	0.99	1.44E-29
Rpp38	229.57	0.56	6.93E-03
Xrn2	3914.68	0.79	1.26E-23
Rps15a	4320.57	0.39	4.42E-07

Wdr3	3095.92	0.26	2.24E-04
Tex10	1221.08	0.49	3.36E-06
Rrp9	1099.64	1.38	3.00E-37
Imp4	1633.35	0.46	8.80E-07
Gnl3	3828.80	0.55	1.49E-15
Ltv1	1037.90	0.93	8.15E-18
Exosc10	2170.13	0.72	1.56E-15
Krr1	1196.26	0.29	3.56E-03
Bysl	1282.73	0.34	1.26E-03
Rpp30	199.70	0.80	5.46E-04
Nop58	3976.73	1.30	2.86E-55
Ddx21	9345.51	0.62	2.26E-27
C1d	377.48	0.80	1.46E-06
Wdr12	908.73	0.62	4.83E-08
Rcl1	1041.48	1.04	1.77E-20
Utp3	1713.65	0.22	1.99E-02
Pno1	922.37	0.72	2.05E-09
Exosc3	452.17	0.94	1.01E-09
Rps23	4375.83	0.29	1.53E-05
Rps21	1789.99	0.20	2.53E-02
Exosc1	978.74	0.64	6.74E-09
Riok3	1561.16	0.79	5.35E-17
Nop56	5493.35	0.71	5.23E-29
Ddx47	1166.16	0.34	1.45E-03
Rps27l	1216.40	0.36	1.12E-03
Mphosph10	1832.72	0.45	4.96E-06
Mphosph6	416.06	1.65	7.18E-24
Nol11	1268.10	0.96	4.16E-22
Ebna1bp2	1292.07	0.73	1.26E-13
Utp20	4193.18	0.60	7.27E-18
Riok1	791.75	0.81	2.64E-11
Mtrex	1996.99	0.44	7.12E-06
Wdr43	4018.39	0.96	1.96E-46
Wdr75	1799.72	0.84	1.30E-18
Fcf1	493.77	0.55	1.74E-04
Nol9	1885.40	0.34	9.09E-05
Rrp7a	1302.16	0.73	4.73E-13
Nop14	1764.72	0.58	1.28E-09
Ddx52	1086.70	0.40	3.13E-04
Senp3	2684.48	0.24	1.42E-03

rRNA processing in the nucleus and cytosol

gene_name	baseMean	log2FoldChange	p-value
Noc4l	985.00	0.54	8.74E-07

Csnk1d	3644.41	0.45	2.03E-11
Tsr1	1249.06	0.80	9.31E-13
Utp15	1486.04	0.80	1.65E-16
Pwp2	1349.06	1.06	1.13E-25
Bop1	2265.35	0.42	1.66E-07
Fbl	967.01	0.47	4.17E-05
Emg1	1414.86	0.37	7.93E-05
Rps2	4305.82	0.19	4.06E-03
Ncl	38692.94	0.81	6.76E-60
Pdcd11	5067.15	0.52	4.13E-16
Utp14b	622.70	0.82	1.14E-09
Rps18	1203.32	0.41	6.78E-04
Rps7	3939.20	0.21	3.27E-03
Snu13	2209.83	0.88	1.89E-23
Tbl3	1072.45	0.60	1.52E-08
Bms1	2951.39	0.46	3.05E-10
Diexf	1447.13	0.38	6.69E-05
Utp6	1531.88	0.72	3.92E-14
Utp18	1314.38	0.67	1.81E-10
Utp4	2200.51	0.77	2.47E-20
Heatr1	3779.20	0.51	2.28E-12
Dcaf13	1425.76	1.13	1.52E-30
Wdr36	2263.62	0.99	1.44E-29
Rpp38	229.57	0.56	6.93E-03
Xrn2	3914.68	0.79	1.26E-23
Rps15a	4320.57	0.39	4.42E-07
Wdr3	3095.92	0.26	2.24E-04
Tex10	1221.08	0.49	3.36E-06
Rrp9	1099.64	1.38	3.00E-37
Imp4	1633.35	0.46	8.80E-07
Gnl3	3828.80	0.55	1.49E-15
Ltv1	1037.90	0.93	8.15E-18
Exosc10	2170.13	0.72	1.56E-15
Krr1	1196.26	0.29	3.56E-03
Bysl	1282.73	0.34	1.26E-03
Rpp30	199.70	0.80	5.46E-04
Nop58	3976.73	1.30	2.86E-55
Ddx21	9345.51	0.62	2.26E-27
C1d	377.48	0.80	1.46E-06
Wdr12	908.73	0.62	4.83E-08
Rcl1	1041.48	1.04	1.77E-20
Utp3	1713.65	0.22	1.99E-02
Pno1	922.37	0.72	2.05E-09

Exosc3	452.17	0.94	1.01E-09
Rps23	4375.83	0.29	1.53E-05
Rps21	1789.99	0.20	2.53E-02
Exosc1	978.74	0.64	6.74E-09
Riok3	1561.16	0.79	5.35E-17
Nop56	5493.35	0.71	5.23E-29
Ddx47	1166.16	0.34	1.45E-03
Rps27l	1216.40	0.36	1.12E-03
Mphosph10	1832.72	0.45	4.96E-06
Mphosph6	416.06	1.65	7.18E-24
Nol11	1268.10	0.96	4.16E-22
Ebna1bp2	1292.07	0.73	1.26E-13
Utp20	4193.18	0.60	7.27E-18
Riok1	791.75	0.81	2.64E-11
Mtrex	1996.99	0.44	7.12E-06
Wdr43	4018.39	0.96	1.96E-46
Wdr75	1799.72	0.84	1.30E-18
Fcf1	493.77	0.55	1.74E-04
Nol9	1885.40	0.34	9.09E-05
Rrp7a	1302.16	0.73	4.73E-13
Nop14	1764.72	0.58	1.28E-09
Ddx52	1086.70	0.40	3.13E-04
Senp3	2684.48	0.24	1.42E-03

mRNA splicing

gene_name	baseMean	log2FoldChange	p-value
Sf3b3	10469.20	0.26	8.29E-06
Sf3b4	1954.24	0.91	1.31E-27
Srsf9	1574.35	0.36	1.24E-04
Cstf2	1316.75	0.51	1.03E-06
Srsf1	5769.91	1.03	4.17E-51
Aqr	2651.77	0.25	9.35E-04
Hnrnpd	3939.23	0.86	2.48E-31
Dhx15	5227.94	0.75	5.63E-32
Ddx5	14016.10	1.26	5.13E-126
Hnrnpc	4561.55	0.51	5.11E-13
Hnrnpk	6459.99	0.88	2.52E-44
Elavl1	3094.62	0.65	1.51E-19
Magoh	683.62	0.57	1.38E-05
Papola	6068.95	0.26	2.23E-05
Ptbp1	6182.74	0.60	2.07E-22
Rnps1	1332.28	0.51	5.55E-07
Polr2c	339.37	0.59	7.48E-04
Sf3a2	1694.45	0.62	4.85E-12

Srsf2	4773.91	1.17	1.34E-68
Srsf3	3279.26	1.50	3.90E-76
Srsf5	4850.11	0.76	1.53E-33
Tra2b	1680.87	1.18	8.83E-26
Snrpd1	869.79	0.79	6.95E-11
Snu13	2209.83	0.88	1.89E-23
Zrsr2	1016.59	0.64	3.25E-09
U2af2	4756.99	0.48	2.66E-09
Wbp4	888.78	0.61	6.65E-08
Srsf7	2571.25	1.24	6.37E-58
Hnrnpa3	7031.24	0.79	2.73E-27
Prpf38a	790.31	0.78	4.53E-10
Bud31	537.24	0.73	2.50E-06
Fus	6240.10	1.23	3.63E-78
Pcbp1	8736.22	0.26	2.72E-06
Cpsf7	2517.61	0.68	1.10E-17
Txnl4a	2714.25	0.34	1.46E-05
Lsm2	597.42	0.39	3.99E-03
Snrnp200	5967.51	0.19	2.45E-03
Lsm4	910.76	0.35	4.38E-03
Pdcd7	417.26	0.85	1.06E-07
Cpsf2	2668.51	0.76	8.19E-23
Srrm1	6026.02	0.32	4.58E-07
Hnrnpu	23734.98	0.53	1.60E-23
Plrg1	1274.91	0.36	2.42E-04
Hnrnpa2b1	13935.85	0.41	2.27E-09
Prpf40a	3695.16	0.65	1.37E-17
Hnrnph2	2165.43	0.67	3.02E-10
Isy1	806.28	0.59	1.33E-06
Hnrnph1	3814.23	0.36	1.15E-06
Rbm8a	1248.88	0.49	8.88E-07
Dhx38	2677.93	0.54	1.78E-12
Sf3b6	860.71	0.56	2.84E-06
Snw1	1992.63	0.22	6.97E-03
Snrnp40	1011.37	0.56	1.86E-07
Snrnp27	680.08	0.47	1.90E-04
Rbm22	1414.75	0.45	2.81E-06
Zcrb1	725.32	0.91	1.01E-12
Rnpc3	696.72	1.43	1.03E-28
Cwc27	895.57	0.32	1.27E-02
Sf3a1	3147.41	0.39	6.80E-08
Cwc25	750.55	0.47	2.43E-04
Ccar1	2962.48	0.49	2.02E-11

Polr2g	816.51	0.39	8.80E-04
Snrnp48	909.91	0.34	7.11E-03
U2surp	3972.91	0.40	3.64E-08
Puf60	2194.11	0.69	9.09E-18
Srsf6	5807.88	0.81	2.29E-39
Gtf2f2	680.81	0.63	2.32E-06
Snrpa1	1048.08	0.45	2.98E-05
Prpf31	2662.25	0.32	3.90E-05
Srsf11	3608.26	0.34	1.17E-05
Polr2d	298.19	0.82	2.25E-05
Prpf4	774.07	0.50	6.40E-05
Prpf3	1564.26	0.78	2.35E-17
Ddx42	2212.03	0.58	8.47E-13
Wdr33	2275.36	0.18	2.66E-02
Ddx23	3324.21	0.17	2.29E-02
Pcf11	3523.37	0.99	1.12E-46
Sf3a3	1425.41	0.25	9.83E-03
Srrm2	38062.99	0.37	2.31E-12
Smndc1	1665.75	0.90	6.25E-24
Lsm8	387.62	0.40	1.44E-02
Hnrnpm	4706.48	0.35	1.42E-06
Hnrnpa0	4499.69	0.77	1.79E-26
Lsm6	510.04	0.46	1.79E-03
Cwc22	2074.60	0.42	3.36E-07
Sf3b1	9836.42	0.78	2.84E-41
Cstf2t	1679.29	0.37	2.18E-04
Rbm5	3071.62	0.45	1.19E-09
Gtf2f1	1690.01	0.50	3.74E-08
Hnrnpf	15129.33	0.65	2.39E-35
Clp1	323.88	0.45	1.66E-02

Cytokine signaling in immune system

gene_name	baseMean	log2FoldChange	p-value
Isg15	1432.98	5.02	1.78E-25
Fbxw11	2398.21	0.19	2.22E-02
Rapgef1	3386.68	0.39	1.61E-08
Camk2d	823.31	2.32	3.44E-68
Irak2	2726.79	2.07	2.66E-157
Txlna	2762.00	0.50	3.56E-11
Rps6ka3	910.95	0.37	1.14E-03
Birc3	1320.98	1.83	9.96E-74
Birc2	502.26	0.50	6.93E-04
Atf1	1007.76	0.62	1.03E-08
Atf2	1405.88	0.72	4.12E-13

Canx	17884.31	0.41	2.06E-16
Chuk	1790.79	0.55	2.94E-10
Socs3	5943.11	3.60	0.00E+00
Socs1	89.14	4.90	4.09E-18
Crk	2298.37	0.69	8.78E-16
Csf1	596.78	4.52	1.88E-126
Csf2	49.21	4.79	1.09E-09
Csf2ra	2096.70	0.49	1.11E-08
Csf2rb	2723.02	1.24	2.66E-59
Csf2rb2	1719.87	1.32	2.85E-46
Csf3	3475.28	7.99	0.00E+00
Hck	1723.75	2.14	5.07E-99
Ifnar1	1706.06	0.73	5.98E-17
Ifnb1	10.84	2.51	4.46E-05
Ifngr2	1989.91	1.12	2.34E-37
Ikbkb	2162.19	0.29	6.84E-04
Il10	126.22	5.80	1.74E-14
Il10ra	926.88	0.41	4.48E-04
Il10rb	1288.67	0.87	9.57E-17
Il11	9.36	1.31	2.59E-02
Il13ra2	3.27	1.05	1.83E-02
Il18rap	192.07	0.75	1.07E-03
Il1a	3683.94	5.28	9.75E-26
Il1b	1374.86	1.31	1.92E-05
Il1rap	338.48	1.35	2.65E-14
Il1rn	335.29	5.12	7.85E-72
Il2rg	1875.14	0.44	5.79E-07
Il4ra	210.77	0.73	6.90E-04
Il6	31.79	3.76	1.29E-07
Il7	18.16	3.07	7.38E-05
Inppl1	4329.26	0.42	3.90E-10
Irf9	1634.79	2.74	3.38E-159
Jak2	3749.03	3.00	0.00E+00
Lck	117.96	1.29	7.30E-06
Lif	4539.36	1.51	5.82E-06
Psmb8	1166.16	0.47	9.47E-06
Lta	55.45	2.26	3.41E-08
Ltb	1311.99	1.71	2.66E-48
Il1rl1	455.23	1.34	8.94E-18
Lyn	5624.43	1.21	1.23E-81
Mapkapk2	23577.27	2.03	5.57E-288
Psmd7	2212.34	0.69	1.50E-17
Mx2	1826.21	8.54	7.48E-108

Myd88	1782.22	1.11	4.79E-37
Nfkb1	11205.73	3.36	0.00E+00
Nfkb2	6124.64	2.71	0.00E+00
Nfkbia	8480.12	3.42	0.00E+00
Nfkbib	1507.45	1.77	1.77E-59
Sqstm1	26165.69	1.15	2.21E-103
Osm	376.58	3.15	4.51E-61
P4hb	14386.54	0.54	1.14E-23
Prkcd	4039.36	0.37	5.16E-08
Ppm1b	1414.39	0.46	1.45E-06
Ppp2ca	4029.10	0.79	4.97E-27
Ppp2cb	949.78	0.71	6.49E-10
Eif2ak2	3738.08	2.87	1.83E-273
Psma2	1343.58	0.43	1.64E-05
Psma3	354.28	1.30	5.59E-14
Psmb10	507.80	1.20	6.78E-14
Psmb4	2685.91	0.46	3.95E-09
Psmb5	792.75	0.39	1.22E-03
Psmb6	2313.64	0.81	3.56E-23
Psmb7	2149.34	0.44	6.54E-08
Psmc1	2058.07	0.41	9.64E-07
Psmc5	1791.53	0.48	4.54E-07
Psme1	931.08	0.60	5.28E-07
Psme2	245.19	1.59	8.80E-14
Psme3	3722.23	0.81	3.69E-23
Ptpn11	2610.47	0.46	2.13E-09
Ptpn2	897.97	1.39	5.09E-33
Ripk2	1050.74	2.25	1.08E-88
Rela	2241.88	0.63	9.49E-14
Relb	1364.51	2.64	1.72E-115
Rps6ka2	1117.71	2.99	3.12E-141
Shc1	2230.17	0.21	1.97E-02
Stat1	3591.65	2.74	1.29E-275
Stat2	2583.75	3.18	3.33E-298
Stat3	2109.65	1.02	1.62E-35
Eif4g1	20270.33	0.41	1.43E-13
Syk	2952.75	0.17	2.26E-02
Sdc1	293.82	0.85	6.03E-06
Il20rb	53.92	1.59	5.98E-05
Trim25	10908.76	1.32	1.63E-128
Tnf	31684.83	5.02	0.00E+00
Tnfrsf11a	1704.68	1.43	7.81E-55
Tnfrsf1a	1470.95	0.49	4.32E-07

Tnfrsf1b	13713.56	4.03	0.00E+00
Cd40	286.92	4.71	9.87E-64
Tnfrsf8	62.79	3.34	6.20E-14
Tnfrsf9	43.86	1.87	2.43E-05
Tnfsf9	1376.33	0.44	4.13E-06
Hsp90b1	16689.00	0.99	8.82E-75
Traf2	494.13	0.62	2.45E-05
Traf3	1918.31	0.80	1.73E-20
Traf6	1161.70	0.37	3.64E-04
Psmd3	2519.29	0.66	2.45E-17
Tnfrsf4	51.83	1.09	6.97E-03
Uba3	498.54	0.46	3.05E-03
Sumo1	923.57	0.41	6.49E-04
Vamp2	523.25	0.50	7.68E-03
Ywhaz	10649.44	1.26	8.92E-116
Ddx58	3482.95	3.50	0.00E+00
Eif4g3	6741.81	0.23	3.25E-04
Arih1	4638.60	0.40	2.01E-09
Abce1	5823.64	0.77	2.36E-30
Usp18	1864.33	4.43	0.00E+00
Il27	753.26	5.45	5.87E-150
Map2k3	1883.24	1.03	2.83E-33
Map2k4	1100.27	0.80	1.76E-13
Map2k7	1742.96	0.58	4.96E-10
Mapk8	719.85	0.96	3.18E-14
Psma1	1808.96	0.29	1.49E-03
Psma4	1348.83	0.52	2.61E-07
Psma5	425.84	0.47	3.21E-03
Psma6	2635.79	0.94	1.38E-29
Psmb2	1633.25	0.39	1.87E-05
Cul1	1852.60	0.42	3.60E-06
Eif4e2	2204.82	0.67	1.54E-16
Tnfrsf12a	334.17	2.47	2.04E-38
Flnb	12409.73	2.45	0.00E+00
Dusp4	3935.54	2.12	2.66E-178
Ebi3	561.10	0.66	1.02E-05
Ppp2r1a	4925.40	0.25	6.13E-05
Il1f6	8.82	2.20	1.62E-04
Tollip	1731.05	0.68	1.81E-14
Rbx1	524.19	0.75	1.21E-06
Tbk1	2574.73	1.20	7.16E-47
Ube2l6	699.16	1.54	9.91E-26
Psmd8	7369.33	0.60	2.32E-22

Crlf2	387.39	0.53	1.20E-03
Psmd14	1783.24	0.81	6.08E-18
Psmd6	1368.56	0.53	3.68E-08
Tab3	481.53	0.55	6.23E-04
Psmd12	2035.76	0.77	2.75E-18
Psmc6	1783.81	0.53	1.50E-09
Peli1	5233.02	4.50	0.00E+00
Tab2	4033.92	1.01	7.46E-51
Psmd11	1660.14	0.78	2.26E-16
Psmd1	3764.89	0.91	5.37E-41
Ppp2r1b	1020.61	0.89	1.83E-14
Irak3	761.39	1.22	1.67E-17
Uba7	1824.65	1.66	2.82E-72
Pik3cb	2125.76	0.84	3.69E-24
Il33	55.99	4.98	4.04E-10
Il23a	57.93	3.91	1.84E-14
Brwd1	5124.60	1.00	4.50E-51

Immune system

gene_name	baseMean	log2FoldChange	p-value
Dnajc3	2311.11	1.48	3.00E-76
Isg15	1432.98	5.02	1.78E-25
Gm11127	1941.75	0.21	1.33E-02
Ube3c	1857.66	0.48	1.51E-08
Fbxw11	2398.21	0.19	2.22E-02
Acly	6527.30	0.39	7.32E-11
Txndc5	3507.52	0.72	4.87E-25
Nckap1l	8739.57	0.39	2.31E-12
Ticam1	914.42	0.97	1.26E-16
Rapgef1	3386.68	0.39	1.61E-08
Camk2d	823.31	2.32	3.44E-68
Olr1	100.72	5.81	4.27E-12
Atp6v1h	1802.57	0.56	7.88E-10
Irak2	2726.79	2.07	2.66E-157
Ube2q2	634.20	0.51	2.16E-04
Txlna	2762.00	0.50	3.56E-11
Sec13	2056.76	0.29	7.41E-04
Rps6ka3	910.95	0.37	1.14E-03
Abi1	4045.84	1.29	6.96E-81
Atp6v0b	2954.57	0.40	1.85E-06
Actb	116082.06	1.58	5.81E-277
Actg1	32427.81	3.35	0.00E+00
Adam8	6457.73	0.96	7.30E-55
Tirap	631.43	0.62	8.31E-06

Ampd3	1480.63	1.02	7.37E-26
Ap1g1	2229.76	0.25	1.73E-03
Birc3	1320.98	1.83	9.96E-74
Birc2	502.26	0.50	6.93E-04
Aprt	2711.83	0.42	1.32E-06
Rhoa	6230.04	0.26	2.16E-05
Arpc1b	6538.41	0.36	1.20E-09
Atf1	1007.76	0.62	1.03E-08
Atf2	1405.88	0.72	4.12E-13
Atp6v1e1	1673.71	0.89	2.02E-22
Atp6v0a1	1247.71	0.31	1.95E-03
B2m	15401.94	0.39	1.40E-14
Bcl10	1318.64	1.31	1.23E-38
Bcl2l1	2356.97	1.38	1.99E-66
C3ar1	892.25	0.81	1.04E-09
C5ar1	4499.20	4.15	0.00E+00
Anxa2	8338.34	0.60	9.96E-27
Calm1	9928.68	0.83	9.66E-53
Calr	21548.07	0.68	4.60E-32
Canx	17884.31	0.41	2.06E-16
Cap1	12768.72	0.17	1.01E-03
Capza2	3167.35	1.11	6.94E-50
Capzb	4670.07	0.19	2.78E-03
Casp4	437.42	1.91	1.19E-29
Ctnnb1	13389.72	1.18	3.16E-93
Cct2	9380.02	0.32	9.55E-09
Cct8	6258.67	0.43	1.29E-09
Cd14	19074.00	1.63	2.00E-198
Cd36	3.05	0.94	2.66E-02
Cd44	20296.38	3.20	0.00E+00
Cd53	5762.86	0.66	2.64E-20
Cd80	990.25	0.34	2.41E-03
Cdc42	6612.47	0.45	1.54E-10
Chuk	1790.79	0.55	2.94E-10
Socs3	5943.11	3.60	0.00E+00
Socs1	89.14	4.90	4.09E-18
Clu	1401.57	0.45	4.35E-06
Cxcr2	35.74	1.24	6.06E-03
Crebbp	5605.45	0.52	3.11E-14
Crk	2298.37	0.69	8.78E-16
Csf1	596.78	4.52	1.88E-126
Csf2	49.21	4.79	1.09E-09
Csf2ra	2096.70	0.49	1.11E-08

Csf2rb	2723.02	1.24	2.66E-59
Csf2rb2	1719.87	1.32	2.85E-46
Csf3	3475.28	7.99	0.00E+00
Dnajc5	2657.29	0.38	5.71E-07
Cstb	2298.86	0.68	9.51E-16
Ctsb	53983.50	0.18	1.43E-04
Ctsl	19117.40	0.76	4.69E-53
Cyba	4100.94	0.37	1.62E-07
Cybb	24601.70	1.66	3.17E-243
Ddost	2500.92	0.27	4.77E-04
Ddx3x	11678.21	1.15	1.25E-81
Degs1	2836.36	1.09	2.35E-38
Diaph1	3479.75	0.56	1.26E-13
Ube2j2	783.00	0.77	1.91E-10
Ptk2	2269.90	0.21	9.11E-03
Fcer1g	4383.13	0.45	2.44E-07
Fcgr2b	1794.72	0.41	5.73E-06
Fgr	1606.98	2.29	8.00E-122
Fpr1	26.64	3.46	7.16E-07
Tlr3	95.43	1.79	2.97E-08
Lilr4b	17967.36	1.74	2.87E-210
Lilrb4a	24602.76	1.84	2.59E-283
Spsb2	224.27	0.51	2.72E-02
Pdia3	12240.29	0.80	5.88E-43
Hspa5	47115.62	2.32	0.00E+00
Trip12	11465.03	0.98	9.23E-62
Hck	1723.75	2.14	5.07E-99
Hspa1b	772.16	1.73	3.43E-39
Hsp90ab1	45993.82	0.33	5.76E-10
Hsp90aa1	11290.43	0.96	6.38E-60
Icam1	4683.28	2.35	3.93E-270
Ifnar1	1706.06	0.73	5.98E-17
Ifnb1	10.84	2.51	4.46E-05
Ifngr2	1989.91	1.12	2.34E-37
Ikbkb	2162.19	0.29	6.84E-04
Il10	126.22	5.80	1.74E-14
Il10ra	926.88	0.41	4.48E-04
Il10rb	1288.67	0.87	9.57E-17
Il11	9.36	1.31	2.59E-02
Il13ra2	3.27	1.05	1.83E-02
Il18rap	192.07	0.75	1.07E-03
Il1a	3683.94	5.28	9.75E-26
Il1b	1374.86	1.31	1.92E-05

Il1rap	338.48	1.35	2.65E-14
Il1rn	335.29	5.12	7.85E-72
Il2rg	1875.14	0.44	5.79E-07
Il4ra	210.77	0.73	6.90E-04
Il6	31.79	3.76	1.29E-07
Il7	18.16	3.07	7.38E-05
Kpnb1	9445.05	0.64	1.95E-25
Inppl1	4329.26	0.42	3.90E-10
Irf9	1634.79	2.74	3.38E-159
Itch	1862.58	0.28	1.72E-03
Itgav	6809.08	3.25	0.00E+00
Itgax	37.28	1.15	1.11E-02
Itgb1	4964.31	0.69	3.29E-27
Itgb2	6134.33	0.73	2.71E-27
Cd47	2754.47	0.93	2.02E-28
Jak2	3749.03	3.00	0.00E+00
Kif3b	1075.95	0.47	8.35E-06
Kif5b	5722.11	0.73	2.40E-25
Kras	770.65	0.31	1.61E-02
Lck	117.96	1.29	7.30E-06
Lcp2	12544.78	2.34	0.00E+00
Lif	4539.36	1.51	5.82E-06
Psmb8	1166.16	0.47	9.47E-06
Lta	55.45	2.26	3.41E-08
Ltb	1311.99	1.71	2.66E-48
Znrf1	2017.56	1.23	5.69E-47
Pilrb1	20.16	2.90	5.94E-06
Il1rl1	455.23	1.34	8.94E-18
Lyn	5624.43	1.21	1.23E-81
Mapkapk2	23577.27	2.03	5.57E-288
Masp2	123.87	1.13	1.65E-04
Mmp9	146.48	4.25	3.30E-31
Psmd7	2212.34	0.69	1.50E-17
Clec4d	3895.75	2.33	3.98E-236
Mx2	1826.21	8.54	7.48E-108
Myd88	1782.22	1.11	4.79E-37
Myh9	34213.32	0.42	7.47E-14
Myo10	22371.53	2.95	0.00E+00
Ncf4	2723.02	0.62	5.12E-17
Nck1	549.95	1.08	1.81E-14
Nfkb1	11205.73	3.36	0.00E+00
Nfkb2	6124.64	2.71	0.00E+00
Nfkbia	8480.12	3.42	0.00E+00

Nfkbib	1507.45	1.77	1.77E-59
Nfkbie	1011.89	2.02	1.45E-68
Nme2	2968.97	0.26	8.38E-04
Nos2	604.11	6.17	5.94E-104
Slc11a1	1381.46	0.82	4.08E-16
Nras	3726.93	0.73	1.32E-25
Orm2	47.67	1.16	5.25E-03
Sqstm1	26165.69	1.15	2.21E-103
Osm	376.58	3.15	4.51E-61
P4hb	14386.54	0.54	1.14E-23
Pafah1b2	2822.80	0.46	7.76E-10
Pdcd1	357.04	4.34	7.44E-77
Padi2	190.90	0.64	4.98E-03
Pfkl	3533.21	0.64	4.73E-19
Pgam1	12380.68	0.42	1.29E-13
Prkcd	4039.36	0.37	5.16E-08
Plau	1516.08	1.23	3.22E-36
Plaur	5295.59	4.69	0.00E+00
Pld3	2543.13	0.43	1.70E-08
Pa2g4	5453.73	0.30	2.82E-06
Pnp	1435.43	1.57	2.48E-55
Ppm1b	1414.39	0.46	1.45E-06
Ppp2ca	4029.10	0.79	4.97E-27
Ppp2cb	949.78	0.71	6.49E-10
Ppp3cb	1353.68	0.36	2.87E-04
Ppp3r1	1370.92	0.28	4.83E-03
Eif2ak2	3738.08	2.87	1.83E-273
Psen1	2975.54	0.53	2.59E-13
Psma2	1343.58	0.43	1.64E-05
Psma3	354.28	1.30	5.59E-14
Psmb10	507.80	1.20	6.78E-14
Psmb4	2685.91	0.46	3.95E-09
Psmb5	792.75	0.39	1.22E-03
Psmb6	2313.64	0.81	3.56E-23
Psmb7	2149.34	0.44	6.54E-08
Psmc1	2058.07	0.41	9.64E-07
Psmc5	1791.53	0.48	4.54E-07
Psme1	931.08	0.60	5.28E-07
Psme2	245.19	1.59	8.80E-14
Psme3	3722.23	0.81	3.69E-23
Pstpip1	1870.05	0.26	2.05E-03
Ptafr	619.40	1.81	8.05E-39
Ptpn11	2610.47	0.46	2.13E-09

Ptpn2	897.97	1.39	5.09E-33
Ptpn22	2649.87	1.70	5.15E-90
Ripk2	1050.74	2.25	1.08E-88
Ptprj	23325.96	3.64	0.00E+00
Rab10	4114.99	0.57	2.98E-17
Rab18	1346.58	0.30	1.45E-03
Rab4b	678.24	0.32	1.65E-02
Rab6a	1604.89	0.47	1.92E-07
Hspa1a	388.47	1.65	3.79E-18
Rbbp6	4561.56	0.38	9.37E-08
Rel	1229.61	1.95	4.04E-79
Rela	2241.88	0.63	9.49E-14
Relb	1364.51	2.64	1.72E-115
Rlim	7235.75	1.16	2.65E-89
Rnf4	3282.04	0.82	2.45E-29
Rock1	2907.60	0.68	1.10E-16
Rps6ka2	1117.71	2.99	3.12E-141
S100a8	11.62	1.95	1.42E-03
Cxcl2	18374.11	5.18	4.24E-27
Sec22b	1026.99	0.53	3.89E-06
Sec23a	784.48	0.33	1.89E-02
Shc1	2230.17	0.21	1.97E-02
Cyfip1	9404.13	0.93	4.06E-58
Siah2	494.97	1.47	7.09E-23
Slpi	7807.47	4.06	0.00E+00
Siglec1	969.99	2.60	5.83E-102
Fbxo40	88.49	2.38	3.59E-12
Sptbn2	244.85	0.96	2.01E-06
Dtx4	4430.94	1.29	1.14E-85
Src	904.47	4.70	1.56E-33
Trim21	804.40	3.25	4.69E-121
Stat1	3591.65	2.74	1.29E-275
Stat2	2583.75	3.18	3.33E-298
Stat3	2109.65	1.02	1.62E-35
Eif4g1	20270.33	0.41	1.43E-13
Stk10	4708.95	1.53	2.78E-116
Dtx3l	3564.55	2.59	2.06E-218
Surf4	4842.95	1.06	2.80E-51
Syk	2952.75	0.17	2.26E-02
Sdc1	293.82	0.85	6.03E-06
Trim41	1491.57	0.31	7.91E-04
Il20rb	53.92	1.59	5.98E-05
Tank	1585.64	1.09	5.01E-33

Tap1	1623.32	1.44	2.04E-52
Tapbp	7626.23	1.45	1.32E-91
Eea1	5917.31	1.34	2.15E-95
Nlrp3	13267.17	4.31	0.00E+00
Trim25	10908.76	1.32	1.63E-128
Cdc27	2076.28	0.37	1.12E-05
Ube2e2	21.03	2.43	1.93E-05
Sec24c	4078.83	0.61	1.08E-19
Tnf	31684.83	5.02	0.00E+00
Tnfaip3	2693.21	4.26	1.22E-74
Tnfrsf11a	1704.68	1.43	7.81E-55
Tnfrsf1a	1470.95	0.49	4.32E-07
Tnfrsf1b	13713.56	4.03	0.00E+00
Cd40	286.92	4.71	9.87E-64
Tnfrsf8	62.79	3.34	6.20E-14
Tnfrsf9	43.86	1.87	2.43E-05
Tnfsf9	1376.33	0.44	4.13E-06
Tpp2	4088.83	0.83	3.56E-27
Hsp90b1	16689.00	0.99	8.82E-75
Traf2	494.13	0.62	2.45E-05
Traf3	1918.31	0.80	1.73E-20
Traf6	1161.70	0.37	3.64E-04
Psmd3	2519.29	0.66	2.45E-17
Tuba1a	4024.74	0.80	1.18E-33
Tuba1b	11495.93	0.51	3.93E-20
Tuba4a	1100.42	0.83	6.37E-15
Tuba1c	12137.73	0.97	1.43E-72
Tnfrsf4	51.83	1.09	6.97E-03
Txk	47.09	1.06	1.44E-02
Txn1	3253.18	0.45	2.70E-09
Uba3	498.54	0.46	3.05E-03
Uba1	7218.95	0.34	3.69E-09
Ube3a	2408.34	0.60	1.09E-11
Sumo1	923.57	0.41	6.49E-04
Vamp2	523.25	0.50	7.68E-03
Vamp3	926.60	0.76	1.91E-10
Vcl	3528.11	1.84	4.92E-150
Vhl	344.49	0.40	2.24E-02
Naprt	38.97	1.14	8.85E-03
Pak2	5014.82	0.79	5.24E-35
Enpp4	271.85	3.26	3.14E-46
Fbxo11	1859.35	0.74	2.36E-15
Ywhaz	10649.44	1.26	8.92E-116

Ppp2r5a	2539.12	1.10	3.46E-42
Tubb4b	2256.56	0.64	2.55E-15
Ddx58	3482.95	3.50	0.00E+00
Lrrc41	1998.67	0.36	2.85E-05
Eif4g3	6741.81	0.23	3.25E-04
Uba6	2162.76	1.34	1.91E-35
Pilra	136.80	2.98	1.17E-22
Dync1li2	1922.49	0.20	1.90E-02
Dnajc13	4236.22	0.39	2.93E-07
Arih1	4638.60	0.40	2.01E-09
Arih2	1013.40	0.68	4.41E-10
Clec5a	4209.60	1.59	2.46E-126
Fyb	3120.52	0.96	1.87E-32
Rhof	984.16	0.85	1.12E-12
Impdh1	1822.15	0.61	2.40E-12
Impdh2	568.27	0.35	1.37E-02
Pin1	866.77	0.75	3.32E-08
Lnpep	6140.15	0.33	3.20E-08
Abce1	5823.64	0.77	2.36E-30
Malt1	18062.33	4.23	1.26E-182
Mex3c	1174.02	1.27	1.20E-33
Tlr2	8206.48	3.16	0.00E+00
Usp18	1864.33	4.43	0.00E+00
Kbtbd8	246.18	1.52	1.46E-13
Tarm1	3.72	1.15	1.17E-02
Il27	753.26	5.45	5.87E-150
Ap1s3	565.02	1.61	8.91E-30
Ceacam1	1087.79	0.29	6.24E-03
Map2k3	1883.24	1.03	2.83E-33
Map2k4	1100.27	0.80	1.76E-13
Map2k7	1742.96	0.58	4.96E-10
Mapk8	719.85	0.96	3.18E-14
Psma1	1808.96	0.29	1.49E-03
Psma4	1348.83	0.52	2.61E-07
Psma5	425.84	0.47	3.21E-03
Psma6	2635.79	0.94	1.38E-29
Psmb2	1633.25	0.39	1.87E-05
Cul3	3034.01	0.92	1.82E-23
Ube2z	2008.06	0.96	5.66E-24
Vcp	3732.59	0.75	3.28E-27
Cul1	1852.60	0.42	3.60E-06
Eif4e2	2204.82	0.67	1.54E-16
Tcirg1	14980.70	1.05	4.39E-84

Tnfrsf12a	334.17	2.47	2.04E-38
Trim36	174.67	1.88	1.11E-14
Flnb	12409.73	2.45	0.00E+00
Iqgap1	33872.62	0.77	1.28E-51
Rnf19a	618.39	0.51	1.67E-04
Vapa	3732.13	0.57	5.74E-17
Dusp4	3935.54	2.12	2.66E-178
Dcaf1	778.46	0.66	4.68E-08
Ep300	6318.53	0.47	6.90E-15
Cxcl3	187.76	4.43	1.16E-16
Trim56	2124.73	1.63	3.54E-77
Ebi3	561.10	0.66	1.02E-05
Icosl	460.58	3.27	3.64E-68
Fbxo15	134.68	5.49	8.16E-23
Fbxl3	1317.42	0.35	5.47E-04
Nckap1	117.22	1.41	6.68E-07
Ppp2r1a	4925.40	0.25	6.13E-05
Pvr	979.89	0.62	1.51E-08
Lair1	102.62	0.98	1.88E-03
Ube2k	1499.75	0.54	4.30E-09
Sdcbp	7129.11	0.54	2.52E-18
Tuba8	56.40	3.19	1.43E-12
Irf7	2342.76	4.83	0.00E+00
Ywhab	3750.78	0.34	4.19E-07
Il1f6	8.82	2.20	1.62E-04
Tollip	1731.05	0.68	1.81E-14
Pilrb2	16.94	2.90	1.25E-04
Rhog	2884.52	0.32	3.22E-05
Ube2j1	1584.85	0.58	3.19E-10
Acpp	3.20	1.02	1.99E-02
Arpc3	2362.81	0.26	8.59E-04
Rbx1	524.19	0.75	1.21E-06
Actr10	1024.53	0.44	4.14E-05
Tbk1	2574.73	1.20	7.16E-47
Ikbke	6330.50	3.93	0.00E+00
Rapgef4	3.83	1.18	1.03E-02
Ube2d2a	1726.61	0.63	4.27E-11
Rnf14	1139.00	0.97	3.96E-19
Ube2l6	699.16	1.54	9.91E-26
Psmd8	7369.33	0.60	2.32E-22
Golga7	1687.49	0.40	3.36E-05
Rnf25	583.48	0.43	3.05E-03
Crlf2	387.39	0.53	1.20E-03

Sh3kbp1	1510.80	1.35	4.24E-46
Trem1	41.65	2.31	8.69E-07
Huwe1	22015.85	0.17	3.85E-03
Psmd14	1783.24	0.81	6.08E-18
Ncstn	1855.41	0.51	2.17E-07
Cd274	785.82	1.69	5.04E-37
Ctsz	14201.40	0.27	1.45E-07
Asb1	441.88	0.71	5.04E-06
Ube2d3	5385.36	1.34	3.13E-73
Ifitm3	11307.25	1.28	1.40E-92
Atp6v1g2	113.35	1.86	3.82E-10
Smurf2	859.27	0.67	1.37E-08
Sar1b	664.88	0.59	2.84E-05
Psmd6	1368.56	0.53	3.68E-08
Cdc26	453.30	0.47	2.39E-03
Uba5	1084.72	0.70	9.13E-11
Pnp2	401.47	1.39	5.10E-17
Actr2	9597.83	0.88	1.13E-47
Tab3	481.53	0.55	6.23E-04
Ube2w	591.47	0.52	1.55E-04
Pycard	176.92	0.86	3.13E-04
Psmd12	2035.76	0.77	2.75E-18
Magt1	1864.67	0.26	5.92E-03
Psmc6	1783.81	0.53	1.50E-09
Ube2g1	644.63	0.68	1.38E-06
Herc6	499.36	2.97	2.03E-72
Cmtm6	1051.74	0.78	9.28E-13
Peli1	5233.02	4.50	0.00E+00
Herc4	2291.86	0.91	2.16E-28
Ufl1	774.56	0.55	3.54E-05
Agpat2	284.65	0.82	2.64E-05
Arpc5	5770.91	0.91	1.13E-51
Ube2f	873.56	1.71	1.27E-47
Eloc	592.07	0.54	1.49E-04
Tubb6	2758.06	1.85	1.41E-127
Npc2	6120.67	0.17	4.00E-03
Arpc4	2883.34	0.60	6.90E-16
Rnaset2b	2342.28	0.27	5.08E-04
Fbxl15	114.91	0.91	1.42E-03
Arel1	2038.09	0.20	1.44E-02
Wipf2	1390.92	0.65	1.59E-09
Tab2	4033.92	1.01	7.46E-51
Arl8a	2357.04	0.36	3.37E-05

Psmd11	1660.14	0.78	2.26E-16
Ubr4	16958.60	0.14	1.65E-02
Gsdmd	1473.78	0.30	1.75E-03
Bst2	1892.43	1.11	4.37E-37
Cdk13	2118.84	0.24	6.09E-03
Sec24d	953.25	1.66	6.02E-45
Cdc16	1073.30	0.30	5.06E-03
Tmem30a	2317.42	0.32	7.90E-05
Ube2q1	3283.66	0.32	7.96E-06
Psmd1	3764.89	0.91	5.37E-41
Rnf126	1414.97	0.38	1.63E-04
Copb1	3575.32	0.50	1.07E-12
Qpct	202.58	0.71	2.00E-03
Ube2v2	581.64	1.22	5.54E-18
Cul2	899.10	0.30	1.02E-02
Ap2b1	3824.28	0.28	3.15E-05
Fbxo30	420.04	0.62	9.18E-05
Cand1	3607.24	0.56	1.72E-14
Plpp5	508.34	1.69	4.36E-24
Ist1	1516.71	0.35	4.80E-04
Fbxo22	636.99	0.36	6.65E-03
Cotl1	2942.79	0.73	7.01E-24
Rap2c	1098.69	1.35	1.98E-35
Dhx36	1596.47	0.38	3.58E-04
Asb6	1203.59	1.92	2.11E-75
Wasl	640.09	0.37	5.03E-03
Glipr1	1320.20	0.31	1.29E-03
Ppp2r1b	1020.61	0.89	1.83E-14
Irak3	761.39	1.22	1.67E-17
Herc3	624.61	0.30	2.38E-02
Actr3	6232.00	1.83	6.19E-198
Armc8	867.03	0.69	5.98E-09
Rnf6	1668.34	0.94	3.95E-25
Uba7	1824.65	1.66	2.82E-72
Kcmf1	1252.94	0.45	8.15E-06
Pik3cb	2125.76	0.84	3.69E-24
Rnf19b	1332.82	1.78	3.95E-69
Cul5	1768.64	1.02	1.17E-31
Smurf1	662.74	1.11	1.15E-17
Erp44	1462.87	0.46	1.33E-06
Faf2	1506.92	0.31	9.22E-04
Arpc2	9171.46	0.35	2.55E-10
Il33	55.99	4.98	4.04E-10

Sec24a	2299.58	0.71	1.59E-18
Ube2s	959.26	0.77	2.80E-12
Rbsn	944.75	0.32	7.12E-03
Icam4	50.67	1.48	3.05E-04
Mvp	3183.03	0.24	1.56E-03
Rictor	3641.80	0.93	2.19E-40
Wsb1	4274.13	1.37	3.79E-93
Trim39	850.41	0.66	2.35E-08
Rnf34	580.93	0.79	1.24E-08
Ifitm2	3518.39	0.34	1.58E-04
Erap1	1464.97	0.39	2.65E-05
Gpr84	5172.90	4.52	0.00E+00
Rnf114	1713.64	1.59	1.17E-69
Siglece	71.03	1.51	3.11E-05
Il23a	57.93	3.91	1.84E-14
Pik3ap1	4688.36	1.12	2.92E-58
Rnf111	2926.60	0.62	7.22E-16
Brwd1	5124.60	1.00	4.50E-51
Sec24b	2593.29	0.74	1.00E-19

Up-regulated genes following 6 h *P. aeruginosa* infection

Metabolism of RNA

gene_name	baseMean	log2FoldChange	p-value
Noc4l	855.80	0.49	5.07E-05
Sf3b3	9646.91	0.36	3.68E-09
Csnk1d	3033.24	0.29	1.55E-04
Cnot6	2130.67	0.29	4.23E-03
Tsr1	1116.88	0.82	3.97E-13
Utp15	1312.69	0.80	4.62E-14
Sf3b4	1757.74	0.95	2.82E-24
Thoc5	1155.63	0.50	4.13E-06
Srsf9	1400.29	0.37	3.82E-04
Cstf2	1258.38	0.69	9.23E-11
Srsf1	5189.04	1.06	1.20E-42
Pwp2	1280.32	1.20	9.08E-30
Tgs1	1144.68	0.52	1.12E-05
Apobec1	3177.15	0.36	6.48E-06
Aqr	2565.57	0.47	1.11E-08
Hnrnpd	3628.18	0.94	1.01E-31
Bop1	2245.75	0.69	3.21E-15
Cdk7	629.21	0.51	3.86E-04
Dhx15	5131.97	0.98	1.49E-43
Ddx5	12469.88	1.27	7.99E-93
Ddx6	4995.87	0.56	1.06E-15

Dhx9	5561.16	0.17	2.15E-02
Ercc3	937.38	0.42	9.14E-04
Fbl	826.92	0.38	2.44E-03
Emg1	1344.19	0.54	1.06E-07
Gtf2h1	2709.73	0.79	1.52E-21
Gtf2h4	391.34	0.48	6.77E-03
Hnrnpc	4344.64	0.68	5.05E-17
Hnrnpk	5505.33	0.79	2.84E-20
Hspa1b	445.90	0.88	6.45E-08
Elavl1	2796.21	0.70	1.82E-17
Rps2	3955.80	0.28	2.00E-04
Psmb9	149.93	0.58	2.68E-02
Psmb8	1263.83	0.93	2.69E-18
Magoh	607.34	0.57	1.79E-04
Mapkapk2	19163.37	1.87	1.05E-193
Psmd7	2221.96	0.97	6.74E-29
Ncl	37904.91	1.03	3.76E-75
Pdcd11	4423.52	0.48	4.40E-09
Papola	6721.71	0.80	3.62E-34
Cnot7	1382.23	1.37	1.07E-33
Ppp2ca	3481.38	0.73	2.47E-20
Nup88	1507.82	0.45	4.15E-06
Psma2	1307.08	0.66	2.73E-10
Psma3	318.87	1.33	2.37E-12
Psmb10	503.81	1.43	3.06E-18
Psmb4	2641.44	0.71	5.96E-17
Psmb5	733.35	0.50	1.81E-04
Psmb6	2414.62	1.16	2.24E-40
Psmb7	2059.92	0.63	1.11E-11
Psmc1	2078.70	0.72	5.03E-16
Psmc2	1905.33	0.70	5.26E-13
Psmc5	1770.72	0.73	2.81E-10
Psme1	897.17	0.79	7.42E-10
Psme2	212.05	1.56	8.07E-12
Psme3	3341.08	0.84	4.21E-20
Ptbp1	6220.62	0.89	7.25E-39
Eif4a3	1713.45	0.29	2.01E-03
Hspa1a	218.57	0.74	1.05E-03
Ranbp2	8567.97	0.95	4.06E-34
Utp14b	671.07	1.24	1.25E-19
Upf1	3547.07	0.25	9.09E-04
Rnps1	1258.06	0.66	4.88E-09
Polr2c	353.29	0.95	1.15E-07

Rps18	1027.99	0.32	1.65E-02
Rps7	3527.83	0.23	3.20E-03
Sf3a2	1414.93	0.48	2.70E-06
Srsf2	4465.48	1.28	1.85E-53
Srsf3	2645.57	1.31	2.82E-39
Tra2b	1799.20	1.56	4.20E-39
Slbp	687.47	0.38	4.27E-03
Smn1	618.72	0.59	2.48E-05
Eftud2	3203.96	0.37	1.39E-06
Snrnp70	7458.86	0.25	1.90E-04
Snrpb	3112.08	0.32	3.58E-04
Snrpd1	647.36	0.38	8.61E-03
Snu13	2044.42	0.98	2.70E-24
Eif4g1	19458.44	0.60	7.65E-25
Gtf2h3	396.91	0.49	3.30E-03
Tbl3	999.25	0.72	4.44E-10
Bms1	2798.07	0.62	7.75E-15
Tut7	2579.20	0.86	1.06E-22
Diexf	1252.22	0.32	2.69E-03
Gemin5	1308.29	0.47	3.99E-06
Utp6	1303.70	0.63	3.43E-09
Utp18	1093.75	0.52	9.28E-06
Psmd2	4476.34	0.40	1.59E-08
Utp4	2059.62	0.89	4.90E-23
Heatr1	3675.59	0.73	3.21E-22
Nup153	3933.48	0.32	1.30E-05
Ttc37	1152.29	0.31	8.02E-03
Psmd3	2894.68	1.23	5.68E-52
Zrsr2	904.60	0.65	4.76E-08
U2af2	4665.07	0.72	4.46E-18
Ubc	287025.79	0.41	1.53E-11
Dcaf13	1197.64	1.01	1.62E-20
Wbp4	746.15	0.48	4.80E-04
Srsf7	2301.14	1.26	2.06E-48
Thoc1	899.19	0.74	8.18E-10
Wdr36	2008.27	1.00	2.63E-23
Patl1	2575.92	0.68	2.30E-16
Ybx1	7873.51	0.29	1.73E-05
Ywhaz	10307.09	1.43	5.40E-80
Smg7	4150.58	1.36	4.40E-75
Nup188	2572.05	0.63	3.78E-13
Tnks1bp1	635.65	0.54	1.21E-04
Cstf3	981.58	0.68	7.59E-09

Hnrnpa3	6419.85	0.85	1.09E-25
Smg5	2213.55	0.48	3.17E-08
Prpf38a	747.09	0.92	7.05E-12
Bud31	602.07	1.24	1.15E-15
Cnot3	1378.15	0.24	2.27E-02
Fus	5757.16	1.31	4.30E-76
Edc4	1903.61	0.23	2.08E-02
Gtf2h2	329.81	0.51	6.44E-03
Pcbp1	7853.64	0.30	2.94E-06
Psmc4	2470.73	0.28	8.84E-04
Xrn1	2391.74	0.75	1.67E-16
Xrn2	3817.08	1.00	2.17E-30
Psma1	1718.45	0.47	1.36E-06
Psma4	1203.96	0.54	5.09E-07
Psma5	435.57	0.80	1.98E-06
Psma6	2553.03	1.13	1.23E-35
Psmb2	1614.36	0.65	1.36E-11
Rps15a	3590.19	0.22	7.93E-03
Cpsf7	2073.24	0.50	2.33E-08
Nup54	805.86	0.83	6.78E-11
Wdr3	2773.51	0.29	3.20E-04
Tex10	1102.22	0.53	1.22E-06
Nup98	6176.36	1.00	1.54E-51
Txnl4a	2369.11	0.30	8.41E-04
Rrp9	987.19	1.40	1.27E-33
Imp4	1568.56	0.65	8.24E-11
Usp39	1164.78	0.32	3.90E-03
Gnl3	3384.80	0.54	2.21E-12
Snrnp200	5713.36	0.39	1.37E-08
Thoc2	3105.26	0.46	3.43E-07
Edc3	703.75	0.33	1.23E-02
Ltv1	1132.93	1.36	5.51E-34
Thoc6	414.57	0.69	7.11E-05
Lsm4	850.92	0.48	1.86E-04
Exosc10	2071.46	0.88	7.15E-21
Pdcd7	373.16	0.87	8.49E-07
Cpsf2	2726.53	1.07	3.81E-39
Ppp2r1a	4892.59	0.54	5.63E-15
Hnrnpu	21298.31	0.56	4.89E-22
Cnot11	817.13	0.30	1.66E-02
Plrg1	1232.43	0.57	5.62E-08
Nxf1	2440.76	0.55	3.62E-10
Hnrnpa2b1	12966.43	0.53	2.06E-13

Cnot4	1206.49	0.43	8.25E-05
Ddx39b	5495.58	0.48	2.11E-12
Rpp30	166.54	0.67	1.01E-02
Ywhab	3822.62	0.68	1.31E-20
Nop58	3819.50	1.46	1.08E-58
Ppie	366.99	0.57	2.40E-03
Prpf40a	3297.12	0.67	3.67E-15
Ddx21	9247.56	0.87	5.05E-40
Hnrnph2	2145.64	0.92	5.12E-16
Mettl3	418.05	0.82	6.88E-07
Adar	6373.73	2.55	1.81E-261
Hbs1l	1477.49	0.67	1.15E-11
Psmd8	8034.09	1.06	2.78E-54
Wdr12	832.08	0.70	2.88E-08
Isy1	764.41	0.75	1.07E-07
Hnrnph1	3473.28	0.42	1.42E-07
Nup160	1708.86	1.00	2.41E-22
Rcl1	799.78	0.72	1.19E-08
Psmd14	1865.95	1.17	1.45E-32
Wbp11	1760.84	0.27	6.59E-03
Rbm8a	1149.12	0.58	9.89E-08
Wtap	1835.27	0.58	2.58E-10
Dhx38	3112.05	1.14	1.07E-38
Pes1	1723.62	0.35	4.06E-04
Sf3b6	823.90	0.74	4.99E-09
Sarnp	193.67	0.74	2.60E-03
Pno1	948.69	1.05	8.34E-18
Wdr61	994.02	0.32	9.62E-03
Snw1	1901.38	0.41	5.74E-06
Exosc3	421.09	1.05	2.20E-10
Psmd6	1226.03	0.56	1.67E-07
Rps23	3912.42	0.31	1.08E-04
Chtop	3036.63	0.52	2.91E-10
Exosc1	846.78	0.58	2.80E-06
Snrnp40	882.33	0.52	1.65E-05
Snrnp27	587.31	0.41	4.29E-03
Ccnh	536.43	0.46	1.82E-03
Riok3	1597.07	1.10	9.69E-29
Psmd12	2034.72	1.04	3.31E-26
Rpp14	452.55	0.43	1.19E-02
Psmc6	1881.68	0.93	5.52E-24
Nop56	4541.66	0.54	6.00E-14
Zcrb1	656.94	0.96	3.20E-10

Utp11	902.67	0.29	1.47E-02
Lsm1	603.86	0.40	5.97E-03
Rnpc3	566.46	1.26	6.95E-17
Cwc27	818.21	0.40	2.37E-03
Sf3a1	2738.48	0.35	3.76E-05
Cwc25	654.30	0.44	2.82E-03
Ccar1	3102.03	0.88	4.01E-30
Polr2g	740.76	0.45	1.69E-03
Ddx47	1083.77	0.45	1.31E-04
Rnmt	1941.08	0.66	6.42E-12
U2surp	3532.65	0.41	2.88E-07
Puf60	2002.40	0.76	1.59E-17
Mphosph10	1739.22	0.62	1.92E-08
Srsf6	4785.08	0.65	4.65E-20
Rps13	1304.12	0.33	1.86E-03
Ddx39	1894.26	0.89	5.40E-21
Mphosph6	337.01	1.49	1.69E-15
Gtf2f2	603.03	0.63	1.09E-05
Nol11	1029.73	0.77	1.90E-10
Snrpa1	972.63	0.56	1.45E-06
Prpf31	2699.17	0.65	2.53E-15
Ebna1bp2	1265.87	0.95	1.27E-19
Psmd11	1598.13	0.97	4.92E-22
Srsf11	3448.22	0.53	4.24E-10
Polr2d	294.66	1.06	8.36E-08
Nup37	341.28	0.70	1.28E-04
Fyttd1	1033.71	0.67	1.22E-08
A1cf	12.86	3.28	1.16E-05
Cd2bp2	1764.96	0.32	7.12E-04
Psmd1	3836.24	1.21	5.88E-60
Zc3h11a	5295.21	0.76	1.38E-26
Dcp2	1738.76	1.58	4.80E-63
Utp20	4188.67	0.88	1.65E-33
Prpf3	1417.09	0.83	6.47E-15
Riok1	706.25	0.83	6.31E-10
Nupl1	1837.54	0.87	2.09E-17
Ppp2r2a	2064.71	1.15	2.71E-38
Ddx42	1884.35	0.48	1.29E-07
Cnot2	1267.16	0.59	3.02E-08
Seh1l	1401.07	0.88	1.93E-17
Mtrex	1671.10	0.30	6.71E-03
Wdr43	3527.29	0.93	7.50E-35
Utp14a	1949.76	0.72	6.94E-15

Dis3	799.85	0.38	2.16E-03
Thoc3	663.20	0.41	2.49E-03
Wdr75	1519.51	0.73	1.19E-11
Fcf1	440.15	0.56	5.06E-04
Poldip3	2940.44	0.40	3.67E-07
Wdr33	2103.28	0.29	1.15E-03
Ddx23	2983.92	0.20	1.26E-02
Pcf11	3945.90	1.47	1.51E-86
Rrp7a	1169.58	0.76	4.21E-12
Nop14	2063.36	1.19	1.23E-35
Rps25	4461.05	0.16	2.64E-02
Dcp1a	1307.25	0.72	4.60E-12
Smndc1	1521.51	0.97	5.37E-23
Hnrnpm	5031.22	0.80	9.70E-30
Rbm17	1226.72	0.35	2.99E-03
Hnrnpa0	4036.97	0.80	3.86E-23
Cwc22	1872.46	0.46	4.30E-07
Senp3	2366.44	0.22	7.22E-03
Sf3b1	8686.33	0.77	1.81E-31
Rbm5	2521.49	0.25	2.80E-03
Srrt	3562.01	0.30	6.53E-05
Gtf2f1	1718.09	0.82	2.75E-17
Hnrnpf	14104.41	0.77	4.26E-35

Processing of capped intron-containing pre-mRNA

gene_name	baseMean	log2FoldChange	p-value
Sf3b3	9646.91	0.36	3.68E-09
Sf3b4	1757.74	0.95	2.82E-24
Thoc5	1155.63	0.50	4.13E-06
Srsf9	1400.29	0.37	3.82E-04
Cstf2	1258.38	0.69	9.23E-11
Srsf1	5189.04	1.06	1.20E-42
Aqr	2565.57	0.47	1.11E-08
Hnrnpd	3628.18	0.94	1.01E-31
Dhx15	5131.97	0.98	1.49E-43
Ddx5	12469.88	1.27	7.99E-93
Dhx9	5561.16	0.17	2.15E-02
Hnrnpc	4344.64	0.68	5.05E-17
Hnrnpk	5505.33	0.79	2.84E-20
Elavl1	2796.21	0.70	1.82E-17
Magoh	607.34	0.57	1.79E-04
Papola	6721.71	0.80	3.62E-34
Nup88	1507.82	0.45	4.15E-06
Ptbp1	6220.62	0.89	7.25E-39

Eif4a3	1713.45	0.29	2.01E-03
Ranbp2	8567.97	0.95	4.06E-34
Rnps1	1258.06	0.66	4.88E-09
Polr2c	353.29	0.95	1.15E-07
Sf3a2	1414.93	0.48	2.70E-06
Srsf2	4465.48	1.28	1.85E-53
Srsf3	2645.57	1.31	2.82E-39
Tra2b	1799.20	1.56	4.20E-39
Eftud2	3203.96	0.37	1.39E-06
Snrnp70	7458.86	0.25	1.90E-04
Snrpb	3112.08	0.32	3.58E-04
Snrpd1	647.36	0.38	8.61E-03
Snu13	2044.42	0.98	2.70E-24
Nup153	3933.48	0.32	1.30E-05
Zrsr2	904.60	0.65	4.76E-08
U2af2	4665.07	0.72	4.46E-18
Wbp4	746.15	0.48	4.80E-04
Srsf7	2301.14	1.26	2.06E-48
Thoc1	899.19	0.74	8.18E-10
Ybx1	7873.51	0.29	1.73E-05
Nup188	2572.05	0.63	3.78E-13
Cstf3	981.58	0.68	7.59E-09
Hnrnpa3	6419.85	0.85	1.09E-25
Prpf38a	747.09	0.92	7.05E-12
Bud31	602.07	1.24	1.15E-15
Fus	5757.16	1.31	4.30E-76
Pcbp1	7853.64	0.30	2.94E-06
Cpsf7	2073.24	0.50	2.33E-08
Nup54	805.86	0.83	6.78E-11
Nup98	6176.36	1.00	1.54E-51
Txnl4a	2369.11	0.30	8.41E-04
Usp39	1164.78	0.32	3.90E-03
Snrnp200	5713.36	0.39	1.37E-08
Thoc2	3105.26	0.46	3.43E-07
Thoc6	414.57	0.69	7.11E-05
Lsm4	850.92	0.48	1.86E-04
Pdcd7	373.16	0.87	8.49E-07
Cpsf2	2726.53	1.07	3.81E-39
Hnrnpu	21298.31	0.56	4.89E-22
Plrg1	1232.43	0.57	5.62E-08
Nxf1	2440.76	0.55	3.62E-10
Hnrnpa2b1	12966.43	0.53	2.06E-13
Ddx39b	5495.58	0.48	2.11E-12

Ppie	366.99	0.57	2.40E-03
Prpf40a	3297.12	0.67	3.67E-15
Hnrnph2	2145.64	0.92	5.12E-16
Mettl3	418.05	0.82	6.88E-07
Isy1	764.41	0.75	1.07E-07
Hnrnph1	3473.28	0.42	1.42E-07
Nup160	1708.86	1.00	2.41E-22
Wbp11	1760.84	0.27	6.59E-03
Rbm8a	1149.12	0.58	9.89E-08
Wtap	1835.27	0.58	2.58E-10
Dhx38	3112.05	1.14	1.07E-38
Sf3b6	823.90	0.74	4.99E-09
Sarnp	193.67	0.74	2.60E-03
Snw1	1901.38	0.41	5.74E-06
Chtop	3036.63	0.52	2.91E-10
Snrnp40	882.33	0.52	1.65E-05
Snrnp27	587.31	0.41	4.29E-03
Zcrb1	656.94	0.96	3.20E-10
Rnpc3	566.46	1.26	6.95E-17
Cwc27	818.21	0.40	2.37E-03
Sf3a1	2738.48	0.35	3.76E-05
Cwc25	654.30	0.44	2.82E-03
Ccar1	3102.03	0.88	4.01E-30
Polr2g	740.76	0.45	1.69E-03
U2surp	3532.65	0.41	2.88E-07
Puf60	2002.40	0.76	1.59E-17
Srsf6	4785.08	0.65	4.65E-20
Ddx39	1894.26	0.89	5.40E-21
Gtf2f2	603.03	0.63	1.09E-05
Snrpa1	972.63	0.56	1.45E-06
Prpf31	2699.17	0.65	2.53E-15
Srsf11	3448.22	0.53	4.24E-10
Polr2d	294.66	1.06	8.36E-08
Nup37	341.28	0.70	1.28E-04
Fyttd1	1033.71	0.67	1.22E-08
Cd2bp2	1764.96	0.32	7.12E-04
Zc3h11a	5295.21	0.76	1.38E-26
Prpf3	1417.09	0.83	6.47E-15
Nupl1	1837.54	0.87	2.09E-17
Ddx42	1884.35	0.48	1.29E-07
Seh1l	1401.07	0.88	1.93E-17
Thoc3	663.20	0.41	2.49E-03
Poldip3	2940.44	0.40	3.67E-07

Wdr33	2103.28	0.29	1.15E-03
Ddx23	2983.92	0.20	1.26E-02
Pcf11	3945.90	1.47	1.51E-86
Smndc1	1521.51	0.97	5.37E-23
Hnrnpm	5031.22	0.80	9.70E-30
Rbm17	1226.72	0.35	2.99E-03
Hnrnpa0	4036.97	0.80	3.86E-23
Cwc22	1872.46	0.46	4.30E-07
Sf3b1	8686.33	0.77	1.81E-31
Rbm5	2521.49	0.25	2.80E-03
Srrt	3562.01	0.30	6.53E-05
Gtf2f1	1718.09	0.82	2.75E-17
Hnrnpf	14104.41	0.77	4.26E-35

Major pathway of rRNA processing in the nucleolus and cytosol

gene_name	baseMean	log2FoldChange	p-value
Noc4l	855.80	0.49	5.07E-05
Csnk1d	3033.24	0.29	1.55E-04
Tsr1	1116.88	0.82	3.97E-13
Utp15	1312.69	0.80	4.62E-14
Pwp2	1280.32	1.20	9.08E-30
Bop1	2245.75	0.69	3.21E-15
Fbl	826.92	0.38	2.44E-03
Emg1	1344.19	0.54	1.06E-07
Rps2	3955.80	0.28	2.00E-04
Ncl	37904.91	1.03	3.76E-75
Pdcd11	4423.52	0.48	4.40E-09
Utp14b	671.07	1.24	1.25E-19
Rps18	1027.99	0.32	1.65E-02
Rps7	3527.83	0.23	3.20E-03
Snu13	2044.42	0.98	2.70E-24
Tbl3	999.25	0.72	4.44E-10
Bms1	2798.07	0.62	7.75E-15
Diexf	1252.22	0.32	2.69E-03
Utp6	1303.70	0.63	3.43E-09
Utp18	1093.75	0.52	9.28E-06
Utp4	2059.62	0.89	4.90E-23
Heatr1	3675.59	0.73	3.21E-22
Dcaf13	1197.64	1.01	1.62E-20
Wdr36	2008.27	1.00	2.63E-23
Xrn2	3817.08	1.00	2.17E-30
Rps15a	3590.19	0.22	7.93E-03
Wdr3	2773.51	0.29	3.20E-04
Tex10	1102.22	0.53	1.22E-06

Rrp9	987.19	1.40	1.27E-33
Imp4	1568.56	0.65	8.24E-11
Gnl3	3384.80	0.54	2.21E-12
Ltv1	1132.93	1.36	5.51E-34
Exosc10	2071.46	0.88	7.15E-21
Rpp30	166.54	0.67	1.01E-02
Nop58	3819.50	1.46	1.08E-58
Ddx21	9247.56	0.87	5.05E-40
Wdr12	832.08	0.70	2.88E-08
Rcl1	799.78	0.72	1.19E-08
Pes1	1723.62	0.35	4.06E-04
Pno1	948.69	1.05	8.34E-18
Exosc3	421.09	1.05	2.20E-10
Rps23	3912.42	0.31	1.08E-04
Exosc1	846.78	0.58	2.80E-06
Riok3	1597.07	1.10	9.69E-29
Rpp14	452.55	0.43	1.19E-02
Nop56	4541.66	0.54	6.00E-14
Utp11	902.67	0.29	1.47E-02
Ddx47	1083.77	0.45	1.31E-04
Mphosph10	1739.22	0.62	1.92E-08
Rps13	1304.12	0.33	1.86E-03
Mphosph6	337.01	1.49	1.69E-15
Nol11	1029.73	0.77	1.90E-10
Ebna1bp2	1265.87	0.95	1.27E-19
Utp20	4188.67	0.88	1.65E-33
Riok1	706.25	0.83	6.31E-10
Mtrex	1671.10	0.30	6.71E-03
Wdr43	3527.29	0.93	7.50E-35
Utp14a	1949.76	0.72	6.94E-15
Wdr75	1519.51	0.73	1.19E-11
Fcf1	440.15	0.56	5.06E-04
Rrp7a	1169.58	0.76	4.21E-12
Nop14	2063.36	1.19	1.23E-35
Rps25	4461.05	0.16	2.64E-02
Senp3	2366.44	0.22	7.22E-03

rRNA processing

gene_name	baseMean	log2FoldChange	p-value
Noc4l	855.80	0.49	5.07E-05
Csnk1d	3033.24	0.29	1.55E-04
Tsr1	1116.88	0.82	3.97E-13
Utp15	1312.69	0.80	4.62E-14
Pwp2	1280.32	1.20	9.08E-30

Bop1	2245.75	0.69	3.21E-15
Fbl	826.92	0.38	2.44E-03
Emg1	1344.19	0.54	1.06E-07
Rps2	3955.80	0.28	2.00E-04
Ncl	37904.91	1.03	3.76E-75
Pdcd11	4423.52	0.48	4.40E-09
Utp14b	671.07	1.24	1.25E-19
Rps18	1027.99	0.32	1.65E-02
Rps7	3527.83	0.23	3.20E-03
Snu13	2044.42	0.98	2.70E-24
Tbl3	999.25	0.72	4.44E-10
Bms1	2798.07	0.62	7.75E-15
Diexf	1252.22	0.32	2.69E-03
Utp6	1303.70	0.63	3.43E-09
Utp18	1093.75	0.52	9.28E-06
Utp4	2059.62	0.89	4.90E-23
Heatr1	3675.59	0.73	3.21E-22
Dcaf13	1197.64	1.01	1.62E-20
Wdr36	2008.27	1.00	2.63E-23
Xrn2	3817.08	1.00	2.17E-30
Rps15a	3590.19	0.22	7.93E-03
Wdr3	2773.51	0.29	3.20E-04
Tex10	1102.22	0.53	1.22E-06
Rrp9	987.19	1.40	1.27E-33
Imp4	1568.56	0.65	8.24E-11
Gnl3	3384.80	0.54	2.21E-12
Ltv1	1132.93	1.36	5.51E-34
Exosc10	2071.46	0.88	7.15E-21
Rpp30	166.54	0.67	1.01E-02
Nop58	3819.50	1.46	1.08E-58
Ddx21	9247.56	0.87	5.05E-40
Wdr12	832.08	0.70	2.88E-08
Rcl1	799.78	0.72	1.19E-08
Pes1	1723.62	0.35	4.06E-04
Pno1	948.69	1.05	8.34E-18
Exosc3	421.09	1.05	2.20E-10
Rps23	3912.42	0.31	1.08E-04
Exosc1	846.78	0.58	2.80E-06
Riok3	1597.07	1.10	9.69E-29
Rpp14	452.55	0.43	1.19E-02
Nop56	4541.66	0.54	6.00E-14
Utp11	902.67	0.29	1.47E-02
Ddx47	1083.77	0.45	1.31E-04

Mphosph10	1739.22	0.62	1.92E-08
Rps13	1304.12	0.33	1.86E-03
Mphosph6	337.01	1.49	1.69E-15
Nol11	1029.73	0.77	1.90E-10
Ebna1bp2	1265.87	0.95	1.27E-19
Utp20	4188.67	0.88	1.65E-33
Riok1	706.25	0.83	6.31E-10
Mtrex	1671.10	0.30	6.71E-03
Wdr43	3527.29	0.93	7.50E-35
Utp14a	1949.76	0.72	6.94E-15
Wdr75	1519.51	0.73	1.19E-11
Fcf1	440.15	0.56	5.06E-04
Rrp7a	1169.58	0.76	4.21E-12
Nop14	2063.36	1.19	1.23E-35
Rps25	4461.05	0.16	2.64E-02
Senp3	2366.44	0.22	7.22E-03

rRNA processing in the nucleus and cytosol

gene_name	baseMean	log2FoldChange	p-value
Noc4l	855.80	0.49	5.07E-05
Csnk1d	3033.24	0.29	1.55E-04
Tsr1	1116.88	0.82	3.97E-13
Utp15	1312.69	0.80	4.62E-14
Pwp2	1280.32	1.20	9.08E-30
Bop1	2245.75	0.69	3.21E-15
Fbl	826.92	0.38	2.44E-03
Emg1	1344.19	0.54	1.06E-07
Rps2	3955.80	0.28	2.00E-04
Ncl	37904.91	1.03	3.76E-75
Pdcd11	4423.52	0.48	4.40E-09
Utp14b	671.07	1.24	1.25E-19
Rps18	1027.99	0.32	1.65E-02
Rps7	3527.83	0.23	3.20E-03
Snu13	2044.42	0.98	2.70E-24
Tbl3	999.25	0.72	4.44E-10
Bms1	2798.07	0.62	7.75E-15
Diexf	1252.22	0.32	2.69E-03
Utp6	1303.70	0.63	3.43E-09
Utp18	1093.75	0.52	9.28E-06
Utp4	2059.62	0.89	4.90E-23
Heatr1	3675.59	0.73	3.21E-22
Dcaf13	1197.64	1.01	1.62E-20
Wdr36	2008.27	1.00	2.63E-23
Xrn2	3817.08	1.00	2.17E-30

Rps15a	3590.19	0.22	7.93E-03
Wdr3	2773.51	0.29	3.20E-04
Tex10	1102.22	0.53	1.22E-06
Rrp9	987.19	1.40	1.27E-33
Imp4	1568.56	0.65	8.24E-11
Gnl3	3384.80	0.54	2.21E-12
Ltv1	1132.93	1.36	5.51E-34
Exosc10	2071.46	0.88	7.15E-21
Rpp30	166.54	0.67	1.01E-02
Nop58	3819.50	1.46	1.08E-58
Ddx21	9247.56	0.87	5.05E-40
Wdr12	832.08	0.70	2.88E-08
Rcl1	799.78	0.72	1.19E-08
Pes1	1723.62	0.35	4.06E-04
Pno1	948.69	1.05	8.34E-18
Exosc3	421.09	1.05	2.20E-10
Rps23	3912.42	0.31	1.08E-04
Exosc1	846.78	0.58	2.80E-06
Riok3	1597.07	1.10	9.69E-29
Rpp14	452.55	0.43	1.19E-02
Nop56	4541.66	0.54	6.00E-14
Utp11	902.67	0.29	1.47E-02
Ddx47	1083.77	0.45	1.31E-04
Mphosph10	1739.22	0.62	1.92E-08
Rps13	1304.12	0.33	1.86E-03
Mphosph6	337.01	1.49	1.69E-15
Nol11	1029.73	0.77	1.90E-10
Ebna1bp2	1265.87	0.95	1.27E-19
Utp20	4188.67	0.88	1.65E-33
Riok1	706.25	0.83	6.31E-10
Mtrex	1671.10	0.30	6.71E-03
Wdr43	3527.29	0.93	7.50E-35
Utp14a	1949.76	0.72	6.94E-15
Wdr75	1519.51	0.73	1.19E-11
Fcf1	440.15	0.56	5.06E-04
Rrp7a	1169.58	0.76	4.21E-12
Nop14	2063.36	1.19	1.23E-35
Rps25	4461.05	0.16	2.64E-02
Senp3	2366.44	0.22	7.22E-03

mRNA splicing

gene_name	baseMean	log2FoldChange	p-value
Sf3b3	9646.91	0.36	3.68E-09
Sf3b4	1757.74	0.95	2.82E-24

Srsf9	1400.29	0.37	3.82E-04
Cstf2	1258.38	0.69	9.23E-11
Srsf1	5189.04	1.06	1.20E-42
Aqr	2565.57	0.47	1.11E-08
Hnrnpd	3628.18	0.94	1.01E-31
Dhx15	5131.97	0.98	1.49E-43
Ddx5	12469.88	1.27	7.99E-93
Dhx9	5561.16	0.17	2.15E-02
Hnrnpc	4344.64	0.68	5.05E-17
Hnrnpk	5505.33	0.79	2.84E-20
Elavl1	2796.21	0.70	1.82E-17
Magoh	607.34	0.57	1.79E-04
Papola	6721.71	0.80	3.62E-34
Ptbp1	6220.62	0.89	7.25E-39
Eif4a3	1713.45	0.29	2.01E-03
Rnps1	1258.06	0.66	4.88E-09
Polr2c	353.29	0.95	1.15E-07
Sf3a2	1414.93	0.48	2.70E-06
Srsf2	4465.48	1.28	1.85E-53
Srsf3	2645.57	1.31	2.82E-39
Tra2b	1799.20	1.56	4.20E-39
Eftud2	3203.96	0.37	1.39E-06
Snrnp70	7458.86	0.25	1.90E-04
Snrpb	3112.08	0.32	3.58E-04
Snrpd1	647.36	0.38	8.61E-03
Snu13	2044.42	0.98	2.70E-24
Zrsr2	904.60	0.65	4.76E-08
U2af2	4665.07	0.72	4.46E-18
Wbp4	746.15	0.48	4.80E-04
Srsf7	2301.14	1.26	2.06E-48
Ybx1	7873.51	0.29	1.73E-05
Cstf3	981.58	0.68	7.59E-09
Hnrnpa3	6419.85	0.85	1.09E-25
Prpf38a	747.09	0.92	7.05E-12
Bud31	602.07	1.24	1.15E-15
Fus	5757.16	1.31	4.30E-76
Pcbp1	7853.64	0.30	2.94E-06
Cpsf7	2073.24	0.50	2.33E-08
Txnl4a	2369.11	0.30	8.41E-04
Usp39	1164.78	0.32	3.90E-03
Snrnp200	5713.36	0.39	1.37E-08
Lsm4	850.92	0.48	1.86E-04
Pdcd7	373.16	0.87	8.49E-07

Cpsf2	2726.53	1.07	3.81E-39
Hnrnpu	21298.31	0.56	4.89E-22
Plrg1	1232.43	0.57	5.62E-08
Hnrnpa2b1	12966.43	0.53	2.06E-13
Ppie	366.99	0.57	2.40E-03
Prpf40a	3297.12	0.67	3.67E-15
Hnrnph2	2145.64	0.92	5.12E-16
Isy1	764.41	0.75	1.07E-07
Hnrnph1	3473.28	0.42	1.42E-07
Wbp11	1760.84	0.27	6.59E-03
Rbm8a	1149.12	0.58	9.89E-08
Dhx38	3112.05	1.14	1.07E-38
Sf3b6	823.90	0.74	4.99E-09
Snw1	1901.38	0.41	5.74E-06
Snrnp40	882.33	0.52	1.65E-05
Snrnp27	587.31	0.41	4.29E-03
Zcrb1	656.94	0.96	3.20E-10
Rnpc3	566.46	1.26	6.95E-17
Cwc27	818.21	0.40	2.37E-03
Sf3a1	2738.48	0.35	3.76E-05
Cwc25	654.30	0.44	2.82E-03
Ccar1	3102.03	0.88	4.01E-30
Polr2g	740.76	0.45	1.69E-03
U2surp	3532.65	0.41	2.88E-07
Puf60	2002.40	0.76	1.59E-17
Srsf6	4785.08	0.65	4.65E-20
Gtf2f2	603.03	0.63	1.09E-05
Snrpa1	972.63	0.56	1.45E-06
Prpf31	2699.17	0.65	2.53E-15
Srsf11	3448.22	0.53	4.24E-10
Polr2d	294.66	1.06	8.36E-08
Cd2bp2	1764.96	0.32	7.12E-04
Prpf3	1417.09	0.83	6.47E-15
Ddx42	1884.35	0.48	1.29E-07
Wdr33	2103.28	0.29	1.15E-03
Ddx23	2983.92	0.20	1.26E-02
Pcf11	3945.90	1.47	1.51E-86
Smndc1	1521.51	0.97	5.37E-23
Hnrnpm	5031.22	0.80	9.70E-30
Rbm17	1226.72	0.35	2.99E-03
Hnrnpa0	4036.97	0.80	3.86E-23
Cwc22	1872.46	0.46	4.30E-07
Sf3b1	8686.33	0.77	1.81E-31

Rbm5	2521.49	0.25	2.80E-03
Srrt	3562.01	0.30	6.53E-05
Gtf2f1	1718.09	0.82	2.75E-17
Hnrnpf	14104.41	0.77	4.26E-35

Cytokine signaling in immune system

gene_name	baseMean	log2FoldChange	p-value
Isg15	1768.06	5.80	1.06E-29
Fbxw11	2393.98	0.49	7.29E-09
Rapgef1	3500.81	0.76	4.38E-24
Camk2d	787.23	2.46	4.65E-64
Irak2	2157.52	1.87	3.11E-85
Txlna	2418.28	0.46	2.54E-08
Birc3	1268.54	1.98	2.04E-72
App	6082.22	0.18	9.59E-03
Atf1	997.98	0.88	3.70E-14
Atf2	1245.48	0.71	5.58E-11
Canx	17707.20	0.68	1.19E-31
Casp1	421.10	0.47	4.55E-03
Chuk	1801.62	0.84	3.72E-20
Socs3	2693.34	2.52	1.83E-177
Socs1	34.35	3.82	2.11E-09
Crk	2237.50	0.91	3.71E-24
Crkl	1194.88	0.36	1.12E-03
Csf1	772.08	5.10	1.00E-164
Csf2	137.94	6.81	1.49E-13
Csf2ra	2371.27	1.05	1.81E-29
Csf2rb	2178.62	1.03	7.81E-33
Csf2rb2	1276.72	0.95	4.49E-19
Csf3	12968.04	10.09	0.00E+00
Dhx9	5561.16	0.17	2.15E-02
Hck	2225.09	2.78	2.13E-163
Ifnar1	1295.45	0.36	5.02E-04
Ifnb1	3.00	1.25	2.29E-02
Ikbkb	1973.97	0.36	7.70E-05
Il10	52.90	5.01	2.66E-10
Il10ra	919.28	0.68	7.69E-08
Il10rb	1186.35	0.95	6.08E-19
Il12rb2	100.94	0.77	1.37E-02
Il13ra2	4.78	1.80	3.48E-03
Il16	1176.57	0.27	2.09E-02
Il17ra	2041.41	0.21	2.29E-02
Il18	95.56	0.83	9.40E-03
Il1a	8860.86	6.99	1.72E-35

Il1b	3010.48	1.83	5.05E-07
Il1rap	318.62	1.47	1.12E-14
Il1rn	842.80	6.71	8.94E-134
Il2rg	1922.11	0.79	3.90E-17
Il6	105.78	6.01	6.70E-14
Il7	8.59	2.41	2.41E-03
Il7r	151.14	1.94	1.10E-11
Irf9	1645.00	2.95	1.30E-163
Jak1	3981.25	0.51	8.58E-10
Jak2	2409.58	2.47	1.30E-157
Jun	5350.55	1.74	1.79E-127
Lck	222.73	2.65	2.85E-27
Lif	3364.66	2.03	1.23E-05
Psmb9	149.93	0.58	2.68E-02
Psmb8	1263.83	0.93	2.69E-18
Lta	46.18	2.22	1.06E-06
Ltb	869.78	1.14	1.47E-15
Ltbr	839.48	0.43	5.15E-04
Il1rl1	401.61	1.33	3.28E-15
Lyn	7091.21	1.89	1.38E-172
Mapkapk2	19163.37	1.87	1.05E-193
Psmd7	2221.96	0.97	6.74E-29
Mx2	1754.59	8.82	3.72E-109
Myd88	2055.05	1.64	1.93E-75
Nfkb1	11298.39	3.56	0.00E+00
Nfkb2	5526.89	2.74	0.00E+00
Nfkbia	6463.17	3.18	0.00E+00
Nfkbib	1719.58	2.22	4.57E-92
Sqstm1	31840.37	1.78	1.14E-195
Osm	1661.73	5.61	0.00E+00
P4hb	18103.33	1.31	1.73E-108
Ppp2ca	3481.38	0.73	2.47E-20
Ppp2cb	827.68	0.67	1.47E-07
Eif2ak2	4747.58	3.44	0.00E+00
Psma2	1307.08	0.66	2.73E-10
Psma3	318.87	1.33	2.37E-12
Psmb10	503.81	1.43	3.06E-18
Psmb4	2641.44	0.71	5.96E-17
Psmb5	733.35	0.50	1.81E-04
Psmb6	2414.62	1.16	2.24E-40
Psmb7	2059.92	0.63	1.11E-11
Psmc1	2078.70	0.72	5.03E-16
Psmc2	1905.33	0.70	5.26E-13

Psmc5	1770.72	0.73	2.81E-10
Psme1	897.17	0.79	7.42E-10
Psme2	212.05	1.56	8.07E-12
Psme3	3341.08	0.84	4.21E-20
Eif4a3	1713.45	0.29	2.01E-03
Ptpn11	2343.35	0.49	1.57E-08
Ptpn2	898.52	1.62	4.51E-39
Ripk2	553.98	1.29	1.35E-17
Rela	2281.64	0.95	6.17E-27
Relb	1042.85	2.40	8.58E-69
Rps6ka2	1672.14	3.82	1.01E-249
Shc1	2224.69	0.51	7.55E-08
Smarca4	6769.63	0.36	3.95E-07
Sos2	1952.80	0.68	5.86E-14
Stat1	4763.25	3.39	0.00E+00
Stat2	2949.54	3.58	0.00E+00
Stat3	2888.45	1.88	7.48E-121
Stat6	4409.97	0.43	1.69E-09
Eif4g1	19458.44	0.60	7.65E-25
Stx3	1917.32	0.68	1.30E-13
Syk	3280.07	0.73	2.49E-19
Il20rb	42.09	1.39	1.99E-03
Trim25	10006.03	1.39	8.59E-110
Psmd2	4476.34	0.40	1.59E-08
Tnf	40207.52	5.55	0.00E+00
Tnfrsf1a	1765.15	1.17	1.59E-33
Tnfrsf1b	15391.36	4.39	0.00E+00
Cd40	475.70	5.70	5.65E-100
Tnfrsf8	66.07	3.71	2.92E-15
Tnfrsf9	53.95	2.47	3.15E-08
Tnfsf9	1729.10	1.22	2.55E-38
Hsp90b1	17647.54	1.36	3.90E-117
Traf2	484.31	0.85	3.90E-08
Traf6	1073.55	0.48	2.03E-05
Psmd3	2894.68	1.23	5.68E-52
Ubc	287025.79	0.41	1.53E-11
Ube2e1	292.37	0.71	5.51E-04
Uba3	502.86	0.76	1.89E-06
Sumo1	923.35	0.70	1.91E-07
Vamp2	480.08	0.59	3.97E-03
Ywhaz	10307.09	1.43	5.40E-80
Ddx58	6391.48	4.61	0.00E+00
Eif4g3	6610.43	0.48	3.66E-11

Arih1	4072.46	0.38	5.70E-07
Psmc4	2470.73	0.28	8.84E-04
Abce1	5087.36	0.74	8.32E-23
Usp18	3979.83	5.75	0.00E+00
Il27	643.66	5.44	2.64E-135
Map2k3	1277.77	0.41	7.66E-05
Map2k4	843.84	0.46	2.58E-04
Map2k7	1448.20	0.42	1.54E-04
Map3k8	518.68	0.87	1.82E-08
Mapk1	2764.54	0.25	1.68E-03
Mapk8	584.53	0.77	1.29E-07
Psma1	1718.45	0.47	1.36E-06
Psma4	1203.96	0.54	5.09E-07
Psma5	435.57	0.80	1.98E-06
Psma6	2553.03	1.13	1.23E-35
Psmb2	1614.36	0.65	1.36E-11
Cul1	1659.18	0.45	7.85E-06
Eif4e2	1905.38	0.60	3.19E-10
Tnfrsf12a	247.34	2.17	3.57E-23
Flnb	12394.46	2.65	0.00E+00
Dusp4	4613.41	2.60	8.31E-246
Ebi3	681.34	1.34	1.61E-21
Ppp2r1a	4892.59	0.54	5.63E-15
Il1f6	26.25	4.39	7.64E-08
Tollip	2027.15	1.28	1.33E-45
Rbx1	444.38	0.65	1.91E-04
Tbk1	2801.53	1.60	1.99E-78
Ube2l6	1184.04	2.49	9.19E-11
Psmd8	8034.09	1.06	2.78E-54
Crlf2	420.96	1.00	1.42E-09
Psmd14	1865.95	1.17	1.45E-32
Psmd6	1226.03	0.56	1.67E-07
Psmd12	2034.72	1.04	3.31E-26
Psmc6	1881.68	0.93	5.52E-24
Peli1	2682.63	3.66	1.30E-301
Tab2	3293.21	0.83	5.61E-23
Psmd11	1598.13	0.97	4.92E-22
Psmd1	3836.24	1.21	5.88E-60
Ppp2r1b	851.01	0.75	6.38E-09
Irak3	1070.83	2.09	1.01E-51
Uba7	1966.31	2.02	5.14E-88
Pik3cb	1863.58	0.81	5.39E-19
Il33	19.98	3.97	5.49E-07

Il23a	118.75	5.36	6.94E-26
Brwd1	3896.91	0.66	1.95E-16

Immune system

gene_name	baseMean	log2FoldChange	p-value
Dnajc3	1942.00	1.38	3.00E-51
Isg15	1768.06	5.80	1.06E-29
Gm11127	1870.57	0.42	4.23E-06
Ube3c	1571.08	0.37	1.34E-04
Fbxw11	2393.98	0.49	7.29E-09
Acly	6460.08	0.66	3.11E-24
Txndc5	3348.81	0.88	2.29E-31
Nckap1l	7972.59	0.46	6.46E-13
Fbxo4	177.41	0.61	1.42E-02
Rapgef1	3500.81	0.76	4.38E-24
Camk2d	787.23	2.46	4.65E-64
Olr1	41.83	5.10	2.25E-09
Atp6v1h	2274.15	1.33	9.28E-50
Irak2	2157.52	1.87	3.11E-85
Txlna	2418.28	0.46	2.54E-08
Sec13	1844.71	0.32	6.37E-04
Abi1	3324.36	1.14	5.44E-43
Atp6v0b	2942.24	0.68	9.23E-14
Actb	133616.46	2.06	0.00E+00
Actg1	34610.51	3.65	0.00E+00
Adam8	4912.70	0.61	6.46E-17
Aldoa	26212.64	0.23	4.59E-05
Tirap	527.99	0.48	1.48E-03
Ampd3	1825.67	1.68	6.83E-66
Ap1g1	2080.00	0.38	1.24E-05
Ap1m1	1929.19	0.38	9.98E-05
Ap2m1	1820.99	0.24	1.23E-02
Birc3	1268.54	1.98	2.04E-72
App	6082.22	0.18	9.59E-03
Aprt	2534.49	0.55	2.71E-10
Rhoa	5803.62	0.39	7.28E-09
Arpc1b	6195.33	0.53	9.08E-15
Atf1	997.98	0.88	3.70E-14
Atf2	1245.48	0.71	5.58E-11
Atp6v1b2	3816.23	0.23	4.08E-03
Atp6v0d1	2153.89	0.25	4.27E-03
Atp6v1e1	1912.71	1.42	7.19E-50
Atp6v0e	1823.33	0.42	1.89E-04
Atp6v0a1	1630.36	1.20	4.23E-36

Atp7a	2927.59	1.10	4.27E-33
B2m	15480.45	0.70	1.47E-33
Bcl10	1441.56	1.71	1.04E-51
Bcl2l1	2311.84	1.58	5.68E-74
C3	1156.67	0.43	1.47E-04
C3ar1	777.73	0.78	4.46E-07
C5ar1	4240.80	4.25	0.00E+00
Cab39	3479.56	0.25	4.84E-03
Anxa2	7593.37	0.66	8.31E-25
Calm1	9977.50	1.10	3.40E-72
Calr	21103.36	0.91	5.34E-45
Canx	17707.20	0.68	1.19E-31
Cap1	13114.73	0.55	4.90E-21
Capza2	2942.54	1.21	1.45E-42
Capzb	4370.06	0.33	2.71E-06
Casp1	421.10	0.47	4.55E-03
Casp4	731.04	2.99	1.28E-90
Casp8	1119.56	0.98	2.43E-17
Ctnnb1	11838.12	1.17	1.42E-73
Cct2	8980.72	0.51	2.10E-16
Cct8	6271.26	0.73	5.08E-21
Cd14	18433.99	1.79	1.13E-190
Cd44	18442.13	3.24	0.00E+00
Cd53	6572.97	1.21	3.51E-43
Cd80	984.41	0.62	2.32E-07
Cd86	65.37	1.73	7.05E-06
Cdc42	6133.43	0.56	3.67E-11
Chuk	1801.62	0.84	3.72E-20
Socs3	2693.34	2.52	1.83E-177
Socs1	34.35	3.82	2.11E-09
Clu	1169.61	0.29	8.38E-03
Ccr2	161.31	1.22	2.60E-06
Crebbp	4902.19	0.49	1.02E-09
Crk	2237.50	0.91	3.71E-24
Crkl	1194.88	0.36	1.12E-03
Csf1	772.08	5.10	1.00E-164
Csf2	137.94	6.81	1.49E-13
Csf2ra	2371.27	1.05	1.81E-29
Csf2rb	2178.62	1.03	7.81E-33
Csf2rb2	1276.72	0.95	4.49E-19
Csf3	12968.04	10.09	0.00E+00
Csnk2b	1990.97	0.33	2.89E-04
Dnajc5	2610.86	0.63	9.81E-15

Cstb	2413.33	1.05	4.62E-32
Ctsb	51966.03	0.40	9.83E-14
Ctsc	735.33	0.70	2.73E-07
Ctsl	20054.77	1.13	3.51E-91
Cyba	3614.66	0.35	4.57E-06
Cybb	30589.95	2.27	1.02E-304
Ddost	2433.70	0.51	7.98E-09
Ddx3x	10031.26	1.08	7.22E-54
Dhx9	5561.16	0.17	2.15E-02
Degs1	2862.01	1.36	6.93E-40
Diaph1	3139.04	0.61	8.05E-13
Dync1i2	2216.65	0.43	1.58E-06
Stom	7478.75	0.58	4.75E-17
Ube2j2	651.95	0.62	5.21E-05
Ptk2	2491.37	0.74	1.24E-17
Fcer1g	4391.26	0.74	3.37E-16
Fcgr2b	3824.90	2.19	1.04E-174
Fgr	1435.70	2.31	9.82E-103
Fpr1	37.12	4.40	1.88E-08
Tlr3	193.18	3.20	3.59E-32
Lilr4b	19538.91	2.11	1.94E-246
Lilrb4a	26318.58	2.18	4.00E-301
Cxcl1	7.83	2.02	5.62E-03
Pdia3	11970.92	1.02	1.95E-53
Hspa5	47533.63	2.54	0.00E+00
Trip12	12808.39	1.46	4.97E-121
Hck	2225.09	2.78	2.13E-163
Hspa1b	445.90	0.88	6.45E-08
Hsp90ab1	48516.80	0.75	1.84E-28
Hsp90aa1	12397.88	1.40	6.17E-110
Icam1	2741.08	1.60	1.17E-85
Ifnar1	1295.45	0.36	5.02E-04
Ifnb1	3.00	1.25	2.29E-02
Ikbkb	1973.97	0.36	7.70E-05
Il10	52.90	5.01	2.66E-10
Il10ra	919.28	0.68	7.69E-08
Il10rb	1186.35	0.95	6.08E-19
Il12rb2	100.94	0.77	1.37E-02
Il13ra2	4.78	1.80	3.48E-03
Il16	1176.57	0.27	2.09E-02
Il17ra	2041.41	0.21	2.29E-02
Il18	95.56	0.83	9.40E-03
Il1a	8860.86	6.99	1.72E-35

Il1b	3010.48	1.83	5.05E-07
Il1rap	318.62	1.47	1.12E-14
Il1rn	842.80	6.71	8.94E-134
Il2rg	1922.11	0.79	3.90E-17
Il6	105.78	6.01	6.70E-14
Il7	8.59	2.41	2.41E-03
Il7r	151.14	1.94	1.10E-11
Kpnb1	9009.07	0.81	3.83E-33
Irf9	1645.00	2.95	1.30E-163
Itgam	9522.85	0.70	2.37E-30
Itgav	3682.65	2.44	6.84E-173
Itgax	44.41	1.72	1.38E-04
Itgb1	4576.19	0.78	3.31E-28
Itgb2	6091.03	0.99	1.85E-44
Cd47	2568.27	1.04	3.75E-34
Jak1	3981.25	0.51	8.58E-10
Jak2	2409.58	2.47	1.30E-157
Jun	5350.55	1.74	1.79E-127
Kif3b	905.55	0.33	5.78E-03
Kif5b	5681.03	0.98	6.28E-38
Lamp2	6310.89	0.28	2.29E-04
Lck	222.73	2.65	2.85E-27
Lcp2	13147.82	2.62	0.00E+00
Lif	3364.66	2.03	1.23E-05
Psmb9	149.93	0.58	2.68E-02
Psmb8	1263.83	0.93	2.69E-18
Lta	46.18	2.22	1.06E-06
Ltb	869.78	1.14	1.47E-15
Ltbr	839.48	0.43	5.15E-04
Znrf1	2207.16	1.64	1.42E-76
Pilrb1	24.29	3.58	5.72E-07
Tlr7	3606.80	0.62	2.96E-12
Il1rl1	401.61	1.33	3.28E-15
Ly96	160.72	1.09	2.90E-05
Lyn	7091.21	1.89	1.38E-172
Mapkapk2	19163.37	1.87	1.05E-193
Masp2	120.95	1.33	3.62E-05
Mif	3682.75	0.53	2.95E-12
Mmp9	283.98	5.55	1.32E-60
Psmd7	2221.96	0.97	6.74E-29
Clec4d	5182.01	3.01	0.00E+00
Mx2	1754.59	8.82	3.72E-109
Myd88	2055.05	1.64	1.93E-75

Myh9	34021.01	0.69	8.31E-28
Myo10	20146.58	2.97	0.00E+00
Ncf1	5921.85	0.19	5.65E-03
Ncf4	2801.03	0.96	5.45E-33
Nck1	466.43	0.99	3.05E-10
Nfkb1	11298.39	3.56	0.00E+00
Nfkb2	5526.89	2.74	0.00E+00
Nfkbia	6463.17	3.18	0.00E+00
Nfkbib	1719.58	2.22	4.57E-92
Nfkbie	552.73	1.09	2.65E-13
Nme2	2709.15	0.33	1.24E-04
Nos2	1298.60	7.52	1.32E-157
Slc11a1	1572.08	1.35	1.65E-39
Nras	3177.60	0.64	6.07E-14
Orm2	57.14	1.74	2.30E-05
Orm3	42.82	2.13	4.48E-06
Sqstm1	31840.37	1.78	1.14E-195
Osm	1661.73	5.61	0.00E+00
P4hb	18103.33	1.31	1.73E-108
Pafah1b2	2611.67	0.57	9.03E-10
Pdcd1	122.73	2.88	9.75E-20
Padi2	210.14	1.11	1.15E-06
Pfkl	4959.96	1.61	1.80E-117
Pgam1	14129.19	1.01	1.26E-42
Pkm	49579.12	0.15	1.12E-02
Plau	1587.81	1.56	1.14E-52
Plaur	8781.40	5.62	0.00E+00
Pld3	2608.10	0.78	4.28E-21
Pa2g4	5103.61	0.44	1.49E-09
Pnp	2366.15	2.67	1.68E-180
Ppp2ca	3481.38	0.73	2.47E-20
Ppp2cb	827.68	0.67	1.47E-07
Ppp3cb	1283.20	0.53	8.57E-06
Eif2ak2	4747.58	3.44	0.00E+00
Psen1	3197.57	0.97	2.04E-36
Psma2	1307.08	0.66	2.73E-10
Psma3	318.87	1.33	2.37E-12
Psmb10	503.81	1.43	3.06E-18
Psmb4	2641.44	0.71	5.96E-17
Psmb5	733.35	0.50	1.81E-04
Psmb6	2414.62	1.16	2.24E-40
Psmb7	2059.92	0.63	1.11E-11
Psmc1	2078.70	0.72	5.03E-16

Psmc2	1905.33	0.70	5.26E-13
Psmc5	1770.72	0.73	2.81E-10
Psme1	897.17	0.79	7.42E-10
Psme2	212.05	1.56	8.07E-12
Psme3	3341.08	0.84	4.21E-20
Ptafr	672.78	2.18	2.41E-53
Eif4a3	1713.45	0.29	2.01E-03
Ptpn11	2343.35	0.49	1.57E-08
Ptpn2	898.52	1.62	4.51E-39
Ptpn22	2073.57	1.46	4.76E-53
Ripk2	553.98	1.29	1.35E-17
Ptprj	15431.10	3.18	0.00E+00
Rab10	3700.67	0.60	1.14E-13
Rab18	1239.01	0.40	1.50E-04
Rab4b	604.66	0.33	2.24E-02
Rab5c	2717.45	0.29	3.67E-04
Rab6a	1668.04	0.85	1.41E-18
Rab7	2022.31	0.56	2.71E-09
Hspa1a	218.57	0.74	1.05E-03
Rbbp6	3952.40	0.32	2.62E-04
Rel	1137.47	2.03	1.07E-69
Rela	2281.64	0.95	6.17E-27
Relb	1042.85	2.40	8.58E-69
Ripk1	1318.15	0.26	1.19E-02
Rlim	6631.10	1.23	3.58E-62
Rnf4	2778.62	0.71	6.88E-18
Rock1	2809.04	0.88	7.32E-24
Rps6ka2	1672.14	3.82	1.01E-249
S100a8	21.70	3.28	4.18E-06
Cxcl2	48373.24	6.83	3.75E-40
Sec22b	933.53	0.59	7.99E-07
Shc1	2224.69	0.51	7.55E-08
Cyfip1	9233.06	1.15	2.70E-67
Siah2	644.99	2.19	3.32E-51
Slpi	10618.25	4.71	0.00E+00
Smarca4	6769.63	0.36	3.95E-07
Siglec1	2756.48	4.44	0.00E+00
Sos2	1952.80	0.68	5.86E-14
Fbxo40	39.68	1.22	7.67E-03
Sptbn2	207.18	0.86	1.72E-04
Dtx4	4358.07	1.49	5.26E-97
Src	766.56	4.82	1.53E-33
Trim21	611.47	3.01	1.31E-83

Stat1	4763.25	3.39	0.00E+00
Stat2	2949.54	3.58	0.00E+00
Stat3	2888.45	1.88	7.48E-121
Stat6	4409.97	0.43	1.69E-09
Eif4g1	19458.44	0.60	7.65E-25
Cblb	452.50	0.95	2.62E-09
Stk10	5362.59	2.00	1.02E-169
Stx3	1917.32	0.68	1.30E-13
Dtx3l	3414.87	2.72	5.63E-220
Surf4	5566.64	1.59	5.44E-85
Syk	3280.07	0.73	2.49E-19
Trim41	1291.03	0.25	1.78E-02
Nhlrc3	436.84	0.57	5.04E-04
Il20rb	42.09	1.39	1.99E-03
Tank	2398.38	2.11	4.74E-131
Tap1	1985.42	2.04	9.37E-105
Tapbp	8779.08	1.94	3.65E-155
Eea1	6710.50	1.82	2.53E-163
Nlrp3	17227.26	4.88	0.00E+00
Trim25	10006.03	1.39	8.59E-110
Cdc27	1938.76	0.50	2.51E-06
Psmd2	4476.34	0.40	1.59E-08
Ube2e2	23.17	2.93	2.66E-06
Sec24c	3566.44	0.58	1.07E-12
Tnf	40207.52	5.55	0.00E+00
Tnfaip3	4575.84	5.25	7.39E-108
Tnfrsf1a	1765.15	1.17	1.59E-33
Tnfrsf1b	15391.36	4.39	0.00E+00
Cd40	475.70	5.70	5.65E-100
Tnfrsf8	66.07	3.71	2.92E-15
Tnfrsf9	53.95	2.47	3.15E-08
Tnfsf9	1729.10	1.22	2.55E-38
Tom1	736.06	0.69	5.21E-07
Tpp2	4269.51	1.18	4.42E-44
Hsp90b1	17647.54	1.36	3.90E-117
Traf2	484.31	0.85	3.90E-08
Traf6	1073.55	0.48	2.03E-05
Psmd3	2894.68	1.23	5.68E-52
Tuba1a	3623.76	0.83	4.79E-28
Tuba1b	10381.77	0.55	2.45E-16
Tuba4a	1072.06	1.03	6.34E-20
Tuba1c	10521.17	0.92	6.34E-46
Txk	46.16	1.30	2.97E-03

Txn1	2863.48	0.43	2.81E-07
Ubc	287025.79	0.41	1.53E-11
Ube2e1	292.37	0.71	5.51E-04
Uba3	502.86	0.76	1.89E-06
Uba1	7125.64	0.60	2.60E-21
Ube2g2	834.60	0.43	1.72E-03
Ube3a	2082.29	0.54	3.08E-08
Sumo1	923.35	0.70	1.91E-07
Vamp2	480.08	0.59	3.97E-03
Vamp3	734.17	0.50	1.32E-04
Vcl	2686.01	1.56	3.28E-79
Vhl	348.17	0.71	9.93E-05
Was	1351.14	0.34	9.36E-04
Pak2	4965.65	1.03	3.54E-50
Enpp4	356.98	3.90	5.02E-70
Ubr2	6163.61	1.00	1.07E-36
Fbxo11	1599.57	0.67	6.41E-11
Ywhaz	10307.09	1.43	5.40E-80
Ppp2r5a	2472.14	1.29	1.64E-50
Tubb4b	1920.27	0.54	6.81E-09
Trib3	2127.18	2.48	4.80E-140
Ddx58	6391.48	4.61	0.00E+00
Eif4g3	6610.43	0.48	3.66E-11
Uba6	1531.25	0.87	1.04E-13
Fbxw8	1975.30	0.26	3.93E-03
Pilra	404.38	4.89	5.72E-88
Clec12a	230.02	0.58	7.69E-03
Dera	330.72	0.64	5.32E-04
Dync1li2	1834.31	0.39	2.28E-05
Dnajc13	4468.80	0.80	4.77E-27
Dync1li1	2414.69	0.74	1.70E-18
Arih1	4072.46	0.38	5.70E-07
Arih2	1062.35	1.05	4.73E-21
Serpina3f	4.62	1.75	4.11E-03
Clec5a	3532.67	1.48	4.65E-86
Fyb	2568.82	0.80	5.64E-17
Impdh1	1824.43	0.89	1.97E-21
Pin1	760.84	0.73	9.02E-08
Psmc4	2470.73	0.28	8.84E-04
Abce1	5087.36	0.74	8.32E-23
Malt1	16381.37	4.32	0.00E+00
Mex3c	749.76	0.55	2.16E-05
Tlr2	6089.24	2.87	0.00E+00

Usp18	3979.83	5.75	0.00E+00
Atp8b4	4944.22	1.07	1.69E-51
Kbtbd8	176.49	1.11	5.94E-06
Tarm1	7.12	2.35	4.65E-04
Cd207	3.12	1.28	2.06E-02
Il27	643.66	5.44	2.64E-135
Ap1s3	494.80	1.59	2.33E-24
Ceacam1	1319.34	1.03	3.18E-22
Ceacam2	363.71	0.76	3.00E-05
Map2k3	1277.77	0.41	7.66E-05
Map2k4	843.84	0.46	2.58E-04
Map2k7	1448.20	0.42	1.54E-04
Map3k8	518.68	0.87	1.82E-08
Mapk1	2764.54	0.25	1.68E-03
Mapk8	584.53	0.77	1.29E-07
Psma1	1718.45	0.47	1.36E-06
Psma4	1203.96	0.54	5.09E-07
Psma5	435.57	0.80	1.98E-06
Psma6	2553.03	1.13	1.23E-35
Psmb2	1614.36	0.65	1.36E-11
Cul3	2422.85	0.68	1.36E-12
Ube2z	1877.63	1.07	1.29E-28
Vcp	3725.70	1.01	9.73E-41
Cul1	1659.18	0.45	7.85E-06
Eif4e2	1905.38	0.60	3.19E-10
Tcirg1	13688.81	1.12	5.54E-74
Tnfrsf12a	247.34	2.17	3.57E-23
Flnb	12394.46	2.65	0.00E+00
Iqgap1	30570.92	0.81	3.66E-44
Rnf19a	659.49	0.93	1.45E-11
Vapa	3650.97	0.80	3.98E-25
Dusp4	4613.41	2.60	8.31E-246
Dcaf1	901.69	1.24	1.83E-24
Rnf182	10.40	2.93	4.82E-05
Ep300	5705.66	0.52	2.04E-13
Treml2	238.09	1.40	9.78E-10
Cxcl3	508.71	6.01	1.07E-24
Aim2	1662.82	0.74	9.02E-15
Trim56	2209.12	1.93	3.89E-107
Ebi3	681.34	1.34	1.61E-21
Icosl	273.70	2.63	5.89E-32
Fbxo15	49.02	4.36	1.87E-12
Nckap1	104.51	1.43	9.36E-06

Ppp2r1a	4892.59	0.54	5.63E-15
Pvr	1371.97	1.59	2.31E-53
Hgsnat	971.04	0.96	8.00E-16
Tax1bp1	2847.50	0.30	5.94E-04
Lair1	126.01	1.63	1.01E-07
Ube2k	1366.71	0.61	1.92E-09
Pla2g6	809.22	0.58	5.41E-06
Sdcbp	9159.84	1.35	8.53E-85
Tuba8	53.23	3.39	2.91E-12
Irf7	5363.58	6.23	0.00E+00
Ywhab	3822.62	0.68	1.31E-20
Il1f6	26.25	4.39	7.64E-08
Tollip	2027.15	1.28	1.33E-45
Pilrb2	24.46	4.04	9.09E-06
Bri3	1305.44	0.50	8.88E-05
Ppie	366.99	0.57	2.40E-03
Rhog	2601.62	0.36	9.49E-06
Ube2j1	1318.46	0.43	4.60E-05
Acpp	4.01	1.57	7.67E-03
Arpc3	2160.84	0.34	6.77E-05
Rbx1	444.38	0.65	1.91E-04
Actr10	955.66	0.57	1.04E-06
Tbk1	2801.53	1.60	1.99E-78
Ikbke	5603.24	3.93	0.00E+00
Ripk3	377.74	0.86	8.68E-07
Ube2d2a	1485.76	0.56	9.28E-07
Lamtor3	459.49	0.55	6.75E-04
Rnf14	1134.05	1.21	4.57E-24
Ube2l6	1184.04	2.49	9.19E-11
Psmd8	8034.09	1.06	2.78E-54
Golga7	1585.68	0.54	1.16E-06
Crlf2	420.96	1.00	1.42E-09
Sh3kbp1	1448.98	1.50	1.17E-49
Trem1	110.24	4.18	2.07E-26
Huwe1	20369.21	0.28	9.40E-06
Psmd14	1865.95	1.17	1.45E-32
Ncstn	1985.10	0.94	7.94E-22
Cd274	1214.38	2.67	3.24E-104
Ctsz	14616.15	0.64	1.05E-27
Asb1	448.93	1.01	3.93E-10
Ube2d3	5144.96	1.49	2.04E-57
Ifitm3	14458.80	1.98	4.74E-177
Atp6v1f	1768.46	0.43	5.39E-06

Atp6v1g2	71.24	1.24	7.18E-04
Smurf2	716.73	0.53	6.47E-05
Atp6v1c1	3713.75	0.38	3.69E-07
Sar1b	560.85	0.47	2.95E-03
Psmd6	1226.03	0.56	1.67E-07
Uba5	1053.56	0.90	4.20E-15
Pgm1	466.28	0.35	2.57E-02
Pnp2	677.34	2.55	2.79E-71
Actr2	9274.62	1.06	5.90E-47
Ube2w	656.23	1.03	5.38E-13
Psmd12	2034.72	1.04	3.31E-26
Magt1	1832.90	0.53	2.52E-08
Psmc6	1881.68	0.93	5.52E-24
Herc6	722.00	3.76	3.58E-121
Cmtm6	913.67	0.73	8.33E-10
Peli1	2682.63	3.66	1.30E-301
Herc4	1810.08	0.64	7.71E-12
Ufl1	713.26	0.64	5.75E-06
Agpat2	224.67	0.56	1.03E-02
Rnf41	1282.50	0.54	2.27E-07
Arpc5	5242.86	0.97	3.07E-38
Ube2f	703.05	1.53	3.70E-29
Eloc	533.31	0.58	1.69E-04
Tubb6	2765.87	2.08	8.24E-141
Sugt1	820.89	0.32	1.17E-02
Npc2	6353.70	0.58	2.84E-18
Arpc4	2517.15	0.56	3.87E-11
Rnaset2b	2151.82	0.37	2.41E-05
Arel1	2066.26	0.54	4.27E-09
Wipf2	1250.59	0.69	1.02E-09
Tab2	3293.21	0.83	5.61E-23
Arl8a	2192.46	0.48	3.22E-07
Psmd11	1598.13	0.97	4.92E-22
Ubr4	18810.72	0.70	1.28E-29
Gsdmd	1390.67	0.46	6.39E-06
Sec31a	4310.99	0.27	3.21E-04
Bst2	2760.02	2.07	8.41E-133
Sec24d	1116.53	2.16	5.23E-72
Tmbim1	1457.58	0.42	4.83E-05
Tmem30a	2107.31	0.38	3.82E-05
Ube2q1	3303.21	0.63	1.30E-15
Psmd1	3836.24	1.21	5.88E-60
Rnf126	1595.14	0.94	2.18E-20

Copb1	3529.92	0.75	5.66E-22
Atp6ap2	1563.80	0.23	1.55E-02
Qpct	183.99	0.77	1.18E-03
Ube2v2	556.31	1.38	4.56E-16
Trim69	15.39	2.03	1.75E-03
Kctd6	163.45	1.16	2.01E-05
Ap2b1	4124.92	0.76	5.34E-26
Fbxo30	482.32	1.19	3.12E-14
Plpp5	552.91	2.07	1.70E-38
Ist1	1536.66	0.68	4.87E-11
Cotl1	3134.58	1.14	1.24E-48
Rap2c	962.42	1.33	4.45E-28
Pgm2	1044.04	1.02	2.85E-16
Dhx36	1373.80	0.30	8.71E-03
Asb6	721.64	1.17	8.16E-19
Pdzd11	492.48	0.60	8.19E-05
Ppp2r1b	851.01	0.75	6.38E-09
Atp6v1d	1327.57	0.38	2.93E-04
Irak3	1070.83	2.09	1.01E-51
Actr3	7031.99	2.26	9.76E-173
Rnf6	1380.91	0.79	4.40E-14
Uba7	1966.31	2.02	5.14E-88
Kcmf1	1131.74	0.50	5.92E-06
Pik3cb	1863.58	0.81	5.39E-19
Rnf19b	1151.26	1.73	8.13E-53
Cul5	1520.76	0.95	7.23E-17
Smurf1	548.64	0.97	3.52E-11
Atp11b	1226.97	0.29	7.41E-03
Erp44	1446.65	0.72	1.03E-12
Faf2	1528.69	0.64	2.39E-10
Mib2	1188.76	0.60	1.07E-07
Arpc2	9397.42	0.70	3.40E-30
Mospd2	720.46	0.61	7.04E-06
Il33	19.98	3.97	5.49E-07
Sec24a	2095.13	0.77	7.99E-19
Ube2s	997.59	1.12	7.93E-20
Rbsn	879.48	0.45	9.30E-04
Mvp	3157.70	0.52	2.36E-10
Rictor	2481.63	0.30	4.30E-04
Wsb1	5914.22	2.20	7.11E-180
Trim39	762.61	0.69	1.54E-07
Rnf34	506.54	0.76	8.35E-07
Erap1	1312.68	0.42	7.58E-05

Gpr84	4194.56	4.39	0.00E+00
Rnf114	1841.06	1.95	3.54E-94
Siglece	70.77	1.75	4.10E-06
Il23a	118.75	5.36	6.94E-26
Pik3ap1	4132.32	1.11	1.16E-47
Nedd4l	167.24	2.10	5.83E-16
Rnf111	2699.26	0.71	4.19E-17
Brwd1	3896.91	0.66	1.95E-16
Pag1	1112.00	0.59	6.69E-08
Sec24b	2165.53	0.60	7.88E-11

Down-regulated genes following 2 h *P. aeruginosa* infection

HDACs deacetylate histones

gene_name	baseMean	log2FoldChange	p-value
Hist1h4m	1831.21	-0.47	3.44E-06
Chd4	16112.90	-0.18	7.98E-03
Mta1	2379.88	-0.27	4.84E-03
Hist2h2aa1	20324.25	-0.40	2.53E-11
Hmg20b	1066.24	-0.36	1.99E-03
Hdac10	349.19	-0.45	1.20E-02
Phf21a	821.47	-1.08	5.15E-19
Ncor1	7475.92	-0.24	4.45E-04
Chd3	3146.03	-0.37	6.13E-06
Rcor1	1082.97	-0.41	3.62E-04
Gatad2b	2066.97	-0.35	3.33E-04
Arid4a	1293.27	-1.20	9.83E-31
Hist1h3f	1457.12	-0.42	2.42E-05
Hist1h3c	1746.00	-0.51	7.95E-07
Hist1h3d	5418.51	-0.81	1.41E-26
Hist1h3b	2284.47	-0.66	4.39E-13
Hist1h3e	1629.35	-0.32	6.87E-04
Hist1h3h	2396.56	-0.77	2.16E-18
Hist1h3i	2479.14	-0.66	3.97E-15
Hist2h3b	2997.68	-0.71	2.97E-16
Hist1h4f	1696.61	-0.42	5.92E-05
Hist1h4i	882.11	-0.29	1.52E-02
Hist1h4j	1541.68	-0.54	6.08E-08
Hist1h4k	2149.28	-0.26	3.46E-03
Hist1h4n	2286.46	-0.42	1.85E-05
Hist1h2ac	2480.62	-0.83	2.75E-19
Hist1h2ae	2648.97	-0.89	1.35E-24
Hist1h2ag	2842.52	-0.84	1.23E-23
Hist1h2an	1727.60	-0.81	9.86E-18

Hist1h2ab	3537.08	-0.88	1.13E-26
Hist2h2ac	1896.67	-0.42	4.92E-06
Hist1h2ba	238.32	-0.54	1.34E-02
Hist1h2bb	2069.22	-0.67	3.56E-14
Hist1h2be	2008.04	-0.36	2.20E-04
Hist1h2bg	2501.46	-0.37	1.92E-05
Hist1h2bk	2093.94	-0.58	2.04E-08
Hist1h2bm	737.43	-1.15	3.27E-17
Hist2h2bb	2202.09	-0.36	8.71E-04
Hist2h2be	144.00	-1.38	9.98E-08
Hist1h2ai	3028.82	-0.84	9.88E-24
Hist4h4	644.09	-0.65	1.26E-06
Hist1h4b	1892.34	-0.27	3.34E-03
Hist1h3a	1002.91	-0.55	1.80E-06
Hist1h2ao	6811.26	-0.76	3.65E-27
Hist1h2bc	2735.64	-0.29	2.42E-03
Hist1h4h	2584.34	-0.44	9.08E-07
Hdac8	150.73	-1.11	1.09E-05
Hist2h3c2	5631.19	-0.54	1.11E-12
Hist2h4	846.38	-1.00	5.99E-16
Hist1h3g	1069.95	-0.80	2.48E-13

Cell cycle

gene_name	baseMean	log2FoldChange	p-value
Hist1h4m	1831.21	-0.47	3.44E-06
Akap9	5187.31	-0.21	1.03E-02
Pot1a	234.00	-0.67	1.29E-03
Numa1	6009.58	-0.53	3.03E-14
Tubg1	596.60	-0.40	3.61E-03
Espl1	831.17	-0.54	8.28E-06
Cenpf	1111.25	-0.35	1.65E-03
Abl1	1567.85	-0.93	8.69E-21
Atm	2399.30	-0.72	4.06E-15
Cep131	264.13	-1.28	9.51E-10
Blm	624.65	-0.51	1.68E-04
Brca1	559.52	-1.26	5.68E-16
Btrc	414.71	-0.73	6.36E-06
Bub1b	1291.06	-0.30	3.67E-03
Ccnd3	906.92	-0.68	9.52E-09
Cdc25b	811.11	-1.12	1.32E-16
Cdc7	158.70	-1.09	2.01E-05
Cdk2	563.07	-0.47	2.34E-03
Cdkn1b	534.01	-1.00	9.83E-12
Cdkn2c	185.66	-0.82	2.88E-04

Cdkn2d	398.54	-1.07	3.81E-10
Dync1h1	26493.49	-0.30	2.68E-05
Foxm1	1008.93	-0.41	3.09E-04
Lpin1	737.90	-0.84	9.95E-11
Herc2	8491.86	-0.31	1.07E-05
Hist2h2aa1	20324.25	-0.40	2.53E-11
Incenp	3188.91	-0.37	2.38E-06
Cep250	2525.49	-0.23	5.26E-03
Mad1l1	666.07	-0.31	1.63E-02
Mdm4	2189.99	-0.26	2.19E-03
Rab8a	1545.72	-0.26	6.35E-03
Mre11a	780.93	-0.39	4.30E-03
Orc1	202.30	-0.66	2.42E-03
Plk1	356.33	-1.00	8.75E-09
Pold1	1179.39	-0.41	2.03E-04
Rad1	208.27	-0.84	1.34E-04
Rad50	1405.90	-0.57	1.70E-07
Rad9a	275.94	-1.21	6.53E-10
Rangap1	1930.76	-0.68	2.58E-14
Rb1	1033.15	-0.38	5.44E-04
Rbl1	808.65	-0.96	6.76E-12
Rbl2	1130.95	-0.70	1.65E-10
Rfc1	1723.38	-0.54	4.33E-09
Rpa2	347.06	-0.66	1.89E-04
Cep78	462.70	-0.66	1.82E-05
Aurkb	517.33	-0.35	1.50E-02
Aurka	300.53	-0.93	8.26E-07
Tfdp2	877.59	-0.76	2.78E-10
Cep164	1332.49	-0.31	2.92E-03
Ncaph	578.45	-0.43	2.43E-03
Terf2	624.64	-0.59	7.21E-05
Tert	33.72	-1.80	5.89E-05
Mis18bp1	522.45	-0.55	3.27E-04
Ppp2r5d	1072.77	-0.41	1.64E-04
Lin52	112.90	-0.64	2.04E-02
Nek9	3158.96	-0.42	1.21E-07
Cenpj	202.03	-1.15	5.92E-07
Tmpo	2457.75	-0.54	7.72E-11
Top3a	351.42	-0.93	5.00E-07
Tubb4a	164.15	-0.98	4.23E-05
Ubc	223950.46	-0.58	1.29E-13
Wee1	386.02	-0.79	1.09E-05
Aaas	1093.06	-0.38	1.33E-03

Lemd2	738.79	-0.49	8.25E-05
Ppp2r5b	404.09	-0.38	1.88E-02
Kif18a	238.38	-0.52	1.34E-02
Phf20	1478.55	-1.20	1.02E-33
Cenpe	989.80	-0.37	1.33E-03
Fbxl18	381.41	-0.81	1.97E-06
Tubgcp5	632.59	-0.43	1.35E-03
Dync1li2	1547.75	-0.35	3.00E-04
Topbp1	3165.04	-0.19	1.75E-02
Brip1	333.44	-0.67	2.44E-04
Akt3	1565.42	-0.32	7.44E-04
Cdc6	242.59	-1.05	3.33E-07
Mdc1	2329.18	-0.68	4.62E-14
E2f2	484.56	-1.48	1.52E-21
Mcph1	766.27	-0.42	1.60E-03
Hist1h3f	1457.12	-0.42	2.42E-05
Orc5	393.42	-1.17	2.80E-12
Exo1	214.12	-0.99	6.06E-06
Cntrl	1855.39	-0.34	2.96E-04
Ppp2r5c	2940.28	-0.33	3.90E-05
Ppp2r5e	1189.28	-0.31	3.31E-03
Zw10	1015.13	-0.53	3.77E-06
Clspn	563.76	-0.67	2.43E-06
Csnk1e	1629.74	-0.46	2.94E-06
Skp2	450.01	-1.34	3.42E-13
Zfp385a	1065.82	-0.86	5.84E-13
Gtse1	1151.18	-0.50	4.05E-06
Hist1h3c	1746.00	-0.51	7.95E-07
Hist1h3d	5418.51	-0.81	1.41E-26
Hist1h3b	2284.47	-0.66	4.39E-13
Hist1h3e	1629.35	-0.32	6.87E-04
Hist1h3h	2396.56	-0.77	2.16E-18
Hist1h3i	2479.14	-0.66	3.97E-15
Hist2h3b	2997.68	-0.71	2.97E-16
Hist1h4f	1696.61	-0.42	5.92E-05
Hist1h4i	882.11	-0.29	1.52E-02
Hist1h4j	1541.68	-0.54	6.08E-08
Hist1h4k	2149.28	-0.26	3.46E-03
Hist1h4n	2286.46	-0.42	1.85E-05
Hist1h2ac	2480.62	-0.83	2.75E-19
Hist1h2ae	2648.97	-0.89	1.35E-24
Hist1h2ag	2842.52	-0.84	1.23E-23
Hist1h2an	1727.60	-0.81	9.86E-18

Hist1h2ab	3537.08	-0.88	1.13E-26
Hist2h2ac	1896.67	-0.42	4.92E-06
Hist1h2ba	238.32	-0.54	1.34E-02
Hist1h2bb	2069.22	-0.67	3.56E-14
Hist1h2be	2008.04	-0.36	2.20E-04
Hist1h2bg	2501.46	-0.37	1.92E-05
Hist1h2bk	2093.94	-0.58	2.04E-08
Hist1h2bm	737.43	-1.15	3.27E-17
Hist2h2be	144.00	-1.38	9.98E-08
Hist1h2ai	3028.82	-0.84	9.88E-24
Hist4h4	644.09	-0.65	1.26E-06
Hist1h4b	1892.34	-0.27	3.34E-03
Dna2	496.90	-1.29	2.54E-16
Tubgcp6	902.10	-0.90	2.56E-14
Hist1h3a	1002.91	-0.55	1.80E-06
Nsl1	168.53	-1.19	8.35E-07
Chek2	275.22	-1.46	2.32E-13
Cdca8	542.92	-0.60	2.73E-05
Cenpo	292.94	-0.61	1.07E-03
Cdc23	599.23	-0.39	1.02E-02
Tuba8	3.63	-0.75	1.74E-02
Actr1a	2783.62	-0.31	1.89E-04
Nup210	1458.15	-0.31	1.81E-03
Sfn	45.52	-1.41	4.52E-04
Anapc7	617.63	-0.47	6.46E-04
Fzr1	1046.32	-0.52	9.42E-06
Clip1	3421.34	-0.39	7.29E-06
Gmnn	314.99	-0.66	4.33E-04
Nek7	1472.02	-0.30	2.04E-03
Ska2	93.85	-1.68	9.81E-08
Hist1h2ao	6811.26	-0.76	3.65E-27
Mastl	173.01	-1.26	3.92E-06
Fbxo5	129.15	-1.91	2.89E-11
Cdt1	2036.68	-0.21	1.53E-02
Nde1	892.94	-0.81	9.61E-11
Cdca5	227.62	-1.10	4.96E-07
Zwilch	208.74	-0.52	1.91E-02
Hist1h2bc	2735.64	-0.29	2.42E-03
Dynll2	1014.42	-0.46	4.04E-05
Ncapd2	1552.13	-0.51	2.71E-07
Ssna1	268.94	-0.53	8.09E-03
Sgo2a	208.29	-0.82	3.25E-04
Hist1h4h	2584.34	-0.44	9.08E-07

Mcm10	427.09	-0.44	9.66E-03
Rnf168	493.64	-0.53	4.52E-04
Hdac8	150.73	-1.11	1.09E-05
Spdl1	466.33	-0.64	4.22E-04
Abraxas1	226.21	-0.53	1.09E-02
Cep192	1543.85	-0.49	4.11E-06
Esco2	210.00	-1.25	3.93E-07
Sgo1	128.61	-2.22	1.47E-14
Pot1b	169.66	-1.03	5.56E-05
Kif2c	197.14	-0.67	3.75E-03
Cep57	508.14	-0.49	1.02E-03
Rmi1	370.07	-0.67	7.48E-05
Gorasp1	258.91	-1.16	4.48E-08
Mau2	1436.80	-0.43	1.62E-05
Haus8	357.22	-0.53	2.06E-03
Sdccag8	409.32	-1.03	1.70E-10
Bora	274.28	-0.61	1.90E-03
Ninl	258.00	-0.51	9.83E-03
Ncapd3	1140.99	-0.25	2.22E-02
Gins3	332.56	-2.51	4.03E-36
Sfi1	4544.49	-0.96	6.77E-42
Hist2h3c2	5631.19	-0.54	1.11E-12
Hist2h4	846.38	-1.00	5.99E-16
Hist1h3g	1069.95	-0.80	2.48E-13
Golga2	1354.34	-0.24	1.69E-02

Activated PKN stimulates transcription of AR regulated genes KLK2 and KLK3

gene_name	baseMean	log2FoldChange	p-value
Hist1h4m	1831.21	-0.47	3.44E-06
Hist2h2aa1	20324.25	-0.40	2.53E-11
Ncoa2	1907.01	-0.47	1.45E-07
Hist1h3f	1457.12	-0.42	2.42E-05
Hist1h3c	1746.00	-0.51	7.95E-07
Hist1h3d	5418.51	-0.81	1.41E-26
Hist1h3b	2284.47	-0.66	4.39E-13
Hist1h3e	1629.35	-0.32	6.87E-04
Hist1h3h	2396.56	-0.77	2.16E-18
Hist1h3i	2479.14	-0.66	3.97E-15
Hist2h3b	2997.68	-0.71	2.97E-16
Hist1h4f	1696.61	-0.42	5.92E-05
Hist1h4i	882.11	-0.29	1.52E-02
Hist1h4j	1541.68	-0.54	6.08E-08
Hist1h4k	2149.28	-0.26	3.46E-03
Hist1h4n	2286.46	-0.42	1.85E-05

Hist1h2ac	2480.62	-0.83	2.75E-19
Hist1h2ae	2648.97	-0.89	1.35E-24
Hist1h2ag	2842.52	-0.84	1.23E-23
Hist1h2an	1727.60	-0.81	9.86E-18
Hist1h2ab	3537.08	-0.88	1.13E-26
Hist2h2ac	1896.67	-0.42	4.92E-06
Hist1h2ba	238.32	-0.54	1.34E-02
Hist1h2bb	2069.22	-0.67	3.56E-14
Hist1h2be	2008.04	-0.36	2.20E-04
Hist1h2bg	2501.46	-0.37	1.92E-05
Hist1h2bk	2093.94	-0.58	2.04E-08
Hist1h2bm	737.43	-1.15	3.27E-17
Hist2h2be	144.00	-1.38	9.98E-08
Hist1h2ai	3028.82	-0.84	9.88E-24
Hist4h4	644.09	-0.65	1.26E-06
Pkn1	2174.25	-0.58	2.14E-11
Hist1h4b	1892.34	-0.27	3.34E-03
Hist1h3a	1002.91	-0.55	1.80E-06
Hist1h2ao	6811.26	-0.76	3.65E-27
Hist1h2bc	2735.64	-0.29	2.42E-03
Hist1h4h	2584.34	-0.44	9.08E-07
Kdm4c	565.96	-0.34	1.96E-02
Hist2h3c2	5631.19	-0.54	1.11E-12
Hist2h4	846.38	-1.00	5.99E-16
Hist1h3g	1069.95	-0.80	2.48E-13

Condensation of prophase chromosomes

gene_name	baseMean	log2FoldChange	p-value
Hist1h4m	1831.21	-0.47	3.44E-06
Hist2h2aa1	20324.25	-0.40	2.53E-11
Plk1	356.33	-1.00	8.75E-09
Rb1	1033.15	-0.38	5.44E-04
Mcph1	766.27	-0.42	1.60E-03
Hist1h3f	1457.12	-0.42	2.42E-05
Hist1h3c	1746.00	-0.51	7.95E-07
Hist1h3d	5418.51	-0.81	1.41E-26
Hist1h3b	2284.47	-0.66	4.39E-13
Hist1h3e	1629.35	-0.32	6.87E-04
Hist1h3h	2396.56	-0.77	2.16E-18
Hist1h3i	2479.14	-0.66	3.97E-15
Hist2h3b	2997.68	-0.71	2.97E-16
Hist1h4f	1696.61	-0.42	5.92E-05
Hist1h4i	882.11	-0.29	1.52E-02
Hist1h4j	1541.68	-0.54	6.08E-08

Hist1h4k	2149.28	-0.26	3.46E-03
Hist1h4n	2286.46	-0.42	1.85E-05
Hist1h2ac	2480.62	-0.83	2.75E-19
Hist1h2ae	2648.97	-0.89	1.35E-24
Hist1h2ag	2842.52	-0.84	1.23E-23
Hist1h2an	1727.60	-0.81	9.86E-18
Hist1h2ab	3537.08	-0.88	1.13E-26
Hist2h2ac	1896.67	-0.42	4.92E-06
Hist1h2ba	238.32	-0.54	1.34E-02
Hist1h2bb	2069.22	-0.67	3.56E-14
Hist1h2be	2008.04	-0.36	2.20E-04
Hist1h2bg	2501.46	-0.37	1.92E-05
Hist1h2bk	2093.94	-0.58	2.04E-08
Hist1h2bm	737.43	-1.15	3.27E-17
Hist2h2be	144.00	-1.38	9.98E-08
Hist1h2ai	3028.82	-0.84	9.88E-24
Hist4h4	644.09	-0.65	1.26E-06
Hist1h4b	1892.34	-0.27	3.34E-03
Hist1h3a	1002.91	-0.55	1.80E-06
Hist1h2ao	6811.26	-0.76	3.65E-27
Hist1h2bc	2735.64	-0.29	2.42E-03
Hist1h4h	2584.34	-0.44	9.08E-07
Ncapd3	1140.99	-0.25	2.22E-02
Hist2h3c2	5631.19	-0.54	1.11E-12
Hist2h4	846.38	-1.00	5.99E-16
Hist1h3g	1069.95	-0.80	2.48E-13

Signaling by Rho GTPases

gene_name	baseMean	log2FoldChange	p-value
Hist1h4m	1831.21	-0.47	3.44E-06
Arhgef18	348.33	-1.10	2.06E-10
Cenpf	1111.25	-0.35	1.65E-03
Baiap2	998.84	-0.36	1.12E-03
Abl1	1567.85	-0.93	8.69E-21
Rhod	124.89	-2.27	6.25E-14
Btk	1134.47	-0.39	6.00E-04
Bub1b	1291.06	-0.30	3.67E-03
Cit	527.61	-0.62	3.22E-05
Dync1h1	26493.49	-0.30	2.68E-05
Dvl2	546.87	-0.70	1.74E-06
Ect2	351.88	-0.76	1.48E-05
Hist2h2aa1	20324.25	-0.40	2.53E-11
Incenp	3188.91	-0.37	2.38E-06
Inpp5b	703.58	-1.19	9.22E-16

Itsn1	1165.38	-0.89	1.93E-16
Kif5a	1071.71	-1.69	1.38E-46
Klc1	2626.82	-0.49	2.51E-09
Klc2	841.80	-0.29	2.14E-02
Limk1	193.93	-0.64	7.57E-03
Rac3	34.23	-1.70	8.19E-05
Mad1l1	666.07	-0.31	1.63E-02
Mcf2l	803.09	-0.98	1.36E-15
Men1	925.91	-1.28	2.08E-25
Ncoa2	1907.01	-0.47	1.45E-07
Pdpk1	1995.94	-0.26	3.68E-03
Pfn2	92.71	-2.62	6.57E-14
Pik3r2	1441.63	-0.73	1.85E-11
Plk1	356.33	-1.00	8.75E-09
Rangap1	1930.76	-0.68	2.58E-14
Ralbp1	1912.55	-0.29	9.84E-04
Rtkn	92.63	-1.52	8.90E-07
Sos1	1146.88	-0.45	4.82E-05
Aurkb	517.33	-0.35	1.50E-02
Depdc7	67.41	-0.87	1.18E-02
Arhgef11	2043.72	-0.71	4.59E-15
Rhot2	805.46	-0.67	1.38E-07
Wipf1	2554.64	-1.15	4.99E-40
Arhgap9	1013.34	-0.39	4.21E-04
Ppp2r5d	1072.77	-0.41	1.64E-04
Tubb4a	164.15	-0.98	4.23E-05
Vav1	3053.17	-0.33	9.34E-05
Arhgap39	703.62	-1.40	1.31E-26
Mkl1	2189.78	-1.02	1.10E-28
Fgd4	1297.95	-2.43	3.83E-90
Pik3c3	990.01	-0.42	3.16E-04
Fam13b	1070.51	-0.48	5.50E-05
Ppp2r5b	404.09	-0.38	1.88E-02
Arhgap30	3711.94	-0.21	7.29E-03
Arhgef4	195.25	-1.01	7.29E-06
Kif18a	238.38	-0.52	1.34E-02
Cenpe	989.80	-0.37	1.33E-03
Arhgef39	65.63	-1.85	4.19E-07
Arhgap25	566.93	-1.29	2.83E-19
Arhgap33	68.84	-1.06	3.02E-03
Prc1	979.48	-0.39	9.43E-04
Arhgef10	478.93	-1.21	3.33E-15
Dync1li2	1547.75	-0.35	3.00E-04

Stard8	234.77	-1.12	6.93E-08
Arhgap22	542.24	-0.36	1.18E-02
Rhobtb2	263.53	-1.85	4.61E-19
Srgap3	746.10	-0.62	9.75E-07
Hist1h3f	1457.12	-0.42	2.42E-05
Fgd2	199.80	-1.19	3.82E-07
Arhgef40	670.28	-1.04	1.74E-13
Ppp2r5c	2940.28	-0.33	3.90E-05
Ppp2r5e	1189.28	-0.31	3.31E-03
Racgap1	954.33	-0.42	3.80E-04
Zw10	1015.13	-0.53	3.77E-06
Plekhg5	220.40	-1.18	3.72E-07
Myo9a	1213.23	-0.25	1.98E-02
Prex1	2401.72	-0.30	5.12E-04
Fgd3	2172.50	-0.26	2.88E-03
Hist1h3c	1746.00	-0.51	7.95E-07
Hist1h3d	5418.51	-0.81	1.41E-26
Hist1h3b	2284.47	-0.66	4.39E-13
Hist1h3e	1629.35	-0.32	6.87E-04
Hist1h3h	2396.56	-0.77	2.16E-18
Hist1h3i	2479.14	-0.66	3.97E-15
Hist2h3b	2997.68	-0.71	2.97E-16
Hist1h4f	1696.61	-0.42	5.92E-05
Hist1h4i	882.11	-0.29	1.52E-02
Hist1h4j	1541.68	-0.54	6.08E-08
Hist1h4k	2149.28	-0.26	3.46E-03
Hist1h4n	2286.46	-0.42	1.85E-05
Hist1h2ac	2480.62	-0.83	2.75E-19
Hist1h2ae	2648.97	-0.89	1.35E-24
Hist1h2ag	2842.52	-0.84	1.23E-23
Hist1h2an	1727.60	-0.81	9.86E-18
Hist1h2ab	3537.08	-0.88	1.13E-26
Hist2h2ac	1896.67	-0.42	4.92E-06
Hist1h2ba	238.32	-0.54	1.34E-02
Hist1h2bb	2069.22	-0.67	3.56E-14
Hist1h2be	2008.04	-0.36	2.20E-04
Hist1h2bg	2501.46	-0.37	1.92E-05
Hist1h2bk	2093.94	-0.58	2.04E-08
Hist1h2bm	737.43	-1.15	3.27E-17
Hist2h2be	144.00	-1.38	9.98E-08
Hist1h2ai	3028.82	-0.84	9.88E-24
Scai	231.02	-0.72	1.11E-03
Hist4h4	644.09	-0.65	1.26E-06

Pkn1	2174.25	-0.58	2.14E-11
Hist1h4b	1892.34	-0.27	3.34E-03
Hist1h3a	1002.91	-0.55	1.80E-06
Obscn	40.62	-1.40	5.26E-04
Nsl1	168.53	-1.19	8.35E-07
Cdca8	542.92	-0.60	2.73E-05
Cenpo	292.94	-0.61	1.07E-03
Tuba8	3.63	-0.75	1.74E-02
Arhgap27	734.73	-1.54	7.91E-32
Sfn	45.52	-1.41	4.52E-04
Clip1	3421.34	-0.39	7.29E-06
Ska2	93.85	-1.68	9.81E-08
Hist1h2ao	6811.26	-0.76	3.65E-27
Nde1	892.94	-0.81	9.61E-11
Zwilch	208.74	-0.52	1.91E-02
Hist1h2bc	2735.64	-0.29	2.42E-03
Dynll2	1014.42	-0.46	4.04E-05
Sgo2a	208.29	-0.82	3.25E-04
Hist1h4h	2584.34	-0.44	9.08E-07
Arap1	6267.91	-0.25	6.70E-04
Spdl1	466.33	-0.64	4.22E-04
Arhgap17	1814.88	-1.44	5.00E-53
Arhgap45	5992.14	-0.99	3.24E-46
Arhgap19	47.71	-1.37	6.23E-04
Arhgap26	194.36	-0.62	4.93E-03
Sgo1	128.61	-2.22	1.47E-14
Arhgef10l	2292.36	-0.44	6.60E-07
Kif2c	197.14	-0.67	3.75E-03
Arhgap18	583.37	-0.33	2.37E-02
Klc4	1051.45	-0.70	3.54E-08
Arhgap12	642.75	-1.42	2.39E-23
Kdm4c	565.96	-0.34	1.96E-02
Cyfip2	175.64	-0.62	1.08E-02
Hist2h3c2	5631.19	-0.54	1.11E-12
Hist2h4	846.38	-1.00	5.99E-16
Hist1h3g	1069.95	-0.80	2.48E-13

Cell cycle, mitotic

gene_name	baseMean	log2FoldChange	p-value
Hist1h4m	1831.21	-0.47	3.44E-06
Akap9	5187.31	-0.21	1.03E-02
Numa1	6009.58	-0.53	3.03E-14
Tubg1	596.60	-0.40	3.61E-03
Espl1	831.17	-0.54	8.28E-06

Cenpf	1111.25	-0.35	1.65E-03
Abl1	1567.85	-0.93	8.69E-21
Cep131	264.13	-1.28	9.51E-10
Btrc	414.71	-0.73	6.36E-06
Bub1b	1291.06	-0.30	3.67E-03
Ccnd3	906.92	-0.68	9.52E-09
Cdc25b	811.11	-1.12	1.32E-16
Cdc7	158.70	-1.09	2.01E-05
Cdk2	563.07	-0.47	2.34E-03
Cdkn1b	534.01	-1.00	9.83E-12
Cdkn2c	185.66	-0.82	2.88E-04
Cdkn2d	398.54	-1.07	3.81E-10
Dync1h1	26493.49	-0.30	2.68E-05
Foxm1	1008.93	-0.41	3.09E-04
Lpin1	737.90	-0.84	9.95E-11
Hist2h2aa1	20324.25	-0.40	2.53E-11
Incenp	3188.91	-0.37	2.38E-06
Cep250	2525.49	-0.23	5.26E-03
Mad1l1	666.07	-0.31	1.63E-02
Rab8a	1545.72	-0.26	6.35E-03
Orc1	202.30	-0.66	2.42E-03
Plk1	356.33	-1.00	8.75E-09
Pold1	1179.39	-0.41	2.03E-04
Rangap1	1930.76	-0.68	2.58E-14
Rb1	1033.15	-0.38	5.44E-04
Rbl1	808.65	-0.96	6.76E-12
Rbl2	1130.95	-0.70	1.65E-10
Rfc1	1723.38	-0.54	4.33E-09
Rpa2	347.06	-0.66	1.89E-04
Cep78	462.70	-0.66	1.82E-05
Aurkb	517.33	-0.35	1.50E-02
Aurka	300.53	-0.93	8.26E-07
Tfdp2	877.59	-0.76	2.78E-10
Cep164	1332.49	-0.31	2.92E-03
Ncaph	578.45	-0.43	2.43E-03
Ppp2r5d	1072.77	-0.41	1.64E-04
Lin52	112.90	-0.64	2.04E-02
Nek9	3158.96	-0.42	1.21E-07
Cenpj	202.03	-1.15	5.92E-07
Tmpo	2457.75	-0.54	7.72E-11
Tubb4a	164.15	-0.98	4.23E-05
Ubc	223950.46	-0.58	1.29E-13
Wee1	386.02	-0.79	1.09E-05

Aaas	1093.06	-0.38	1.33E-03
Lemd2	738.79	-0.49	8.25E-05
Ppp2r5b	404.09	-0.38	1.88E-02
Kif18a	238.38	-0.52	1.34E-02
Cenpe	989.80	-0.37	1.33E-03
Fbxl18	381.41	-0.81	1.97E-06
Tubgcp5	632.59	-0.43	1.35E-03
Dync1li2	1547.75	-0.35	3.00E-04
Akt3	1565.42	-0.32	7.44E-04
Cdc6	242.59	-1.05	3.33E-07
E2f2	484.56	-1.48	1.52E-21
Mcph1	766.27	-0.42	1.60E-03
Hist1h3f	1457.12	-0.42	2.42E-05
Orc5	393.42	-1.17	2.80E-12
Cntrl	1855.39	-0.34	2.96E-04
Ppp2r5c	2940.28	-0.33	3.90E-05
Ppp2r5e	1189.28	-0.31	3.31E-03
Zw10	1015.13	-0.53	3.77E-06
Csnk1e	1629.74	-0.46	2.94E-06
Skp2	450.01	-1.34	3.42E-13
Gtse1	1151.18	-0.50	4.05E-06
Hist1h3c	1746.00	-0.51	7.95E-07
Hist1h3d	5418.51	-0.81	1.41E-26
Hist1h3b	2284.47	-0.66	4.39E-13
Hist1h3e	1629.35	-0.32	6.87E-04
Hist1h3h	2396.56	-0.77	2.16E-18
Hist1h3i	2479.14	-0.66	3.97E-15
Hist2h3b	2997.68	-0.71	2.97E-16
Hist1h4f	1696.61	-0.42	5.92E-05
Hist1h4i	882.11	-0.29	1.52E-02
Hist1h4j	1541.68	-0.54	6.08E-08
Hist1h4k	2149.28	-0.26	3.46E-03
Hist1h4n	2286.46	-0.42	1.85E-05
Hist1h2ac	2480.62	-0.83	2.75E-19
Hist1h2ae	2648.97	-0.89	1.35E-24
Hist1h2ag	2842.52	-0.84	1.23E-23
Hist1h2an	1727.60	-0.81	9.86E-18
Hist1h2ab	3537.08	-0.88	1.13E-26
Hist2h2ac	1896.67	-0.42	4.92E-06
Hist1h2ba	238.32	-0.54	1.34E-02
Hist1h2bb	2069.22	-0.67	3.56E-14
Hist1h2be	2008.04	-0.36	2.20E-04
Hist1h2bg	2501.46	-0.37	1.92E-05

Hist1h2bk	2093.94	-0.58	2.04E-08
Hist1h2bm	737.43	-1.15	3.27E-17
Hist2h2be	144.00	-1.38	9.98E-08
Hist1h2ai	3028.82	-0.84	9.88E-24
Hist4h4	644.09	-0.65	1.26E-06
Hist1h4b	1892.34	-0.27	3.34E-03
Dna2	496.90	-1.29	2.54E-16
Tubgcp6	902.10	-0.90	2.56E-14
Hist1h3a	1002.91	-0.55	1.80E-06
Nsl1	168.53	-1.19	8.35E-07
Cdca8	542.92	-0.60	2.73E-05
Cenpo	292.94	-0.61	1.07E-03
Cdc23	599.23	-0.39	1.02E-02
Tuba8	3.63	-0.75	1.74E-02
Actr1a	2783.62	-0.31	1.89E-04
Nup210	1458.15	-0.31	1.81E-03
Anapc7	617.63	-0.47	6.46E-04
Fzr1	1046.32	-0.52	9.42E-06
Clip1	3421.34	-0.39	7.29E-06
Gmnn	314.99	-0.66	4.33E-04
Nek7	1472.02	-0.30	2.04E-03
Ska2	93.85	-1.68	9.81E-08
Hist1h2ao	6811.26	-0.76	3.65E-27
Mastl	173.01	-1.26	3.92E-06
Fbxo5	129.15	-1.91	2.89E-11
Cdt1	2036.68	-0.21	1.53E-02
Nde1	892.94	-0.81	9.61E-11
Cdca5	227.62	-1.10	4.96E-07
Zwilch	208.74	-0.52	1.91E-02
Hist1h2bc	2735.64	-0.29	2.42E-03
Dynll2	1014.42	-0.46	4.04E-05
Ncapd2	1552.13	-0.51	2.71E-07
Ssna1	268.94	-0.53	8.09E-03
Sgo2a	208.29	-0.82	3.25E-04
Hist1h4h	2584.34	-0.44	9.08E-07
Mcm10	427.09	-0.44	9.66E-03
Hdac8	150.73	-1.11	1.09E-05
Spdl1	466.33	-0.64	4.22E-04
Cep192	1543.85	-0.49	4.11E-06
Esco2	210.00	-1.25	3.93E-07
Sgo1	128.61	-2.22	1.47E-14
Kif2c	197.14	-0.67	3.75E-03
Cep57	508.14	-0.49	1.02E-03

Gorasp1	258.91	-1.16	4.48E-08
Mau2	1436.80	-0.43	1.62E-05
Haus8	357.22	-0.53	2.06E-03
Sdccag8	409.32	-1.03	1.70E-10
Bora	274.28	-0.61	1.90E-03
Ninl	258.00	-0.51	9.83E-03
Ncapd3	1140.99	-0.25	2.22E-02
Gins3	332.56	-2.51	4.03E-36
Sfi1	4544.49	-0.96	6.77E-42
Hist2h3c2	5631.19	-0.54	1.11E-12
Hist2h4	846.38	-1.00	5.99E-16
Hist1h3g	1069.95	-0.80	2.48E-13
Golga2	1354.34	-0.24	1.69E-02

M phase

gene_name	baseMean	log2FoldChange	p-value
Hist1h4m	1831.21	-0.47	3.44E-06
Akap9	5187.31	-0.21	1.03E-02
Numa1	6009.58	-0.53	3.03E-14
Tubg1	596.60	-0.40	3.61E-03
Espl1	831.17	-0.54	8.28E-06
Cenpf	1111.25	-0.35	1.65E-03
Cep131	264.13	-1.28	9.51E-10
Bub1b	1291.06	-0.30	3.67E-03
Dync1h1	26493.49	-0.30	2.68E-05
Lpin1	737.90	-0.84	9.95E-11
Hist2h2aa1	20324.25	-0.40	2.53E-11
Incenp	3188.91	-0.37	2.38E-06
Cep250	2525.49	-0.23	5.26E-03
Mad1l1	666.07	-0.31	1.63E-02
Plk1	356.33	-1.00	8.75E-09
Rangap1	1930.76	-0.68	2.58E-14
Rb1	1033.15	-0.38	5.44E-04
Cep78	462.70	-0.66	1.82E-05
Aurkb	517.33	-0.35	1.50E-02
Cep164	1332.49	-0.31	2.92E-03
Ncaph	578.45	-0.43	2.43E-03
Ppp2r5d	1072.77	-0.41	1.64E-04
Nek9	3158.96	-0.42	1.21E-07
Cenpj	202.03	-1.15	5.92E-07
Tmpo	2457.75	-0.54	7.72E-11
Tubb4a	164.15	-0.98	4.23E-05
Ubc	223950.46	-0.58	1.29E-13
Aaas	1093.06	-0.38	1.33E-03

Lemd2	738.79	-0.49	8.25E-05
Ppp2r5b	404.09	-0.38	1.88E-02
Kif18a	238.38	-0.52	1.34E-02
Cenpe	989.80	-0.37	1.33E-03
Tubgcp5	632.59	-0.43	1.35E-03
Dync1li2	1547.75	-0.35	3.00E-04
Mcph1	766.27	-0.42	1.60E-03
Hist1h3f	1457.12	-0.42	2.42E-05
Cntrl	1855.39	-0.34	2.96E-04
Ppp2r5c	2940.28	-0.33	3.90E-05
Ppp2r5e	1189.28	-0.31	3.31E-03
Zw10	1015.13	-0.53	3.77E-06
Csnk1e	1629.74	-0.46	2.94E-06
Hist1h3c	1746.00	-0.51	7.95E-07
Hist1h3d	5418.51	-0.81	1.41E-26
Hist1h3b	2284.47	-0.66	4.39E-13
Hist1h3e	1629.35	-0.32	6.87E-04
Hist1h3h	2396.56	-0.77	2.16E-18
Hist1h3i	2479.14	-0.66	3.97E-15
Hist2h3b	2997.68	-0.71	2.97E-16
Hist1h4f	1696.61	-0.42	5.92E-05
Hist1h4i	882.11	-0.29	1.52E-02
Hist1h4j	1541.68	-0.54	6.08E-08
Hist1h4k	2149.28	-0.26	3.46E-03
Hist1h4n	2286.46	-0.42	1.85E-05
Hist1h2ac	2480.62	-0.83	2.75E-19
Hist1h2ae	2648.97	-0.89	1.35E-24
Hist1h2ag	2842.52	-0.84	1.23E-23
Hist1h2an	1727.60	-0.81	9.86E-18
Hist1h2ab	3537.08	-0.88	1.13E-26
Hist2h2ac	1896.67	-0.42	4.92E-06
Hist1h2ba	238.32	-0.54	1.34E-02
Hist1h2bb	2069.22	-0.67	3.56E-14
Hist1h2be	2008.04	-0.36	2.20E-04
Hist1h2bg	2501.46	-0.37	1.92E-05
Hist1h2bk	2093.94	-0.58	2.04E-08
Hist1h2bm	737.43	-1.15	3.27E-17
Hist2h2be	144.00	-1.38	9.98E-08
Hist1h2ai	3028.82	-0.84	9.88E-24
Hist4h4	644.09	-0.65	1.26E-06
Hist1h4b	1892.34	-0.27	3.34E-03
Tubgcp6	902.10	-0.90	2.56E-14
Hist1h3a	1002.91	-0.55	1.80E-06

Nsl1	168.53	-1.19	8.35E-07
Cdca8	542.92	-0.60	2.73E-05
Cenpo	292.94	-0.61	1.07E-03
Cdc23	599.23	-0.39	1.02E-02
Tuba8	3.63	-0.75	1.74E-02
Actr1a	2783.62	-0.31	1.89E-04
Nup210	1458.15	-0.31	1.81E-03
Anapc7	617.63	-0.47	6.46E-04
Clip1	3421.34	-0.39	7.29E-06
Nek7	1472.02	-0.30	2.04E-03
Ska2	93.85	-1.68	9.81E-08
Hist1h2ao	6811.26	-0.76	3.65E-27
Mastl	173.01	-1.26	3.92E-06
Fbxo5	129.15	-1.91	2.89E-11
Nde1	892.94	-0.81	9.61E-11
Cdca5	227.62	-1.10	4.96E-07
Zwilch	208.74	-0.52	1.91E-02
Hist1h2bc	2735.64	-0.29	2.42E-03
Dynll2	1014.42	-0.46	4.04E-05
Ncapd2	1552.13	-0.51	2.71E-07
Ssna1	268.94	-0.53	8.09E-03
Sgo2a	208.29	-0.82	3.25E-04
Hist1h4h	2584.34	-0.44	9.08E-07
Hdac8	150.73	-1.11	1.09E-05
Spdl1	466.33	-0.64	4.22E-04
Cep192	1543.85	-0.49	4.11E-06
Sgo1	128.61	-2.22	1.47E-14
Kif2c	197.14	-0.67	3.75E-03
Cep57	508.14	-0.49	1.02E-03
Gorasp1	258.91	-1.16	4.48E-08
Mau2	1436.80	-0.43	1.62E-05
Haus8	357.22	-0.53	2.06E-03
Sdccag8	409.32	-1.03	1.70E-10
Ninl	258.00	-0.51	9.83E-03
Ncapd3	1140.99	-0.25	2.22E-02
Sfi1	4544.49	-0.96	6.77E-42
Hist2h3c2	5631.19	-0.54	1.11E-12
Hist2h4	846.38	-1.00	5.99E-16
Hist1h3g	1069.95	-0.80	2.48E-13
Golga2	1354.34	-0.24	1.69E-02

Mitotic prometaphase

gene_name	baseMean	log2FoldChange	p-value
Akap9	5187.31	-0.21	1.03E-02

Numa1	6009.58	-0.53	3.03E-14
Tubg1	596.60	-0.40	3.61E-03
Cenpf	1111.25	-0.35	1.65E-03
Cep131	264.13	-1.28	9.51E-10
Bub1b	1291.06	-0.30	3.67E-03
Dync1h1	26493.49	-0.30	2.68E-05
Incenp	3188.91	-0.37	2.38E-06
Cep250	2525.49	-0.23	5.26E-03
Mad1l1	666.07	-0.31	1.63E-02
Plk1	356.33	-1.00	8.75E-09
Rangap1	1930.76	-0.68	2.58E-14
Cep78	462.70	-0.66	1.82E-05
Aurkb	517.33	-0.35	1.50E-02
Cep164	1332.49	-0.31	2.92E-03
Ncaph	578.45	-0.43	2.43E-03
Ppp2r5d	1072.77	-0.41	1.64E-04
Cenpj	202.03	-1.15	5.92E-07
Tubb4a	164.15	-0.98	4.23E-05
Ppp2r5b	404.09	-0.38	1.88E-02
Kif18a	238.38	-0.52	1.34E-02
Cenpe	989.80	-0.37	1.33E-03
Tubgcp5	632.59	-0.43	1.35E-03
Dync1li2	1547.75	-0.35	3.00E-04
Cntrl	1855.39	-0.34	2.96E-04
Ppp2r5c	2940.28	-0.33	3.90E-05
Ppp2r5e	1189.28	-0.31	3.31E-03
Zw10	1015.13	-0.53	3.77E-06
Csnk1e	1629.74	-0.46	2.94E-06
Tubgcp6	902.10	-0.90	2.56E-14
Nsl1	168.53	-1.19	8.35E-07
Cdca8	542.92	-0.60	2.73E-05
Cenpo	292.94	-0.61	1.07E-03
Tuba8	3.63	-0.75	1.74E-02
Actr1a	2783.62	-0.31	1.89E-04
Clip1	3421.34	-0.39	7.29E-06
Ska2	93.85	-1.68	9.81E-08
Nde1	892.94	-0.81	9.61E-11
Cdca5	227.62	-1.10	4.96E-07
Zwilch	208.74	-0.52	1.91E-02
Dynll2	1014.42	-0.46	4.04E-05
Ncapd2	1552.13	-0.51	2.71E-07
Ssna1	268.94	-0.53	8.09E-03
Sgo2a	208.29	-0.82	3.25E-04

Hdac8	150.73	-1.11	1.09E-05
Spdl1	466.33	-0.64	4.22E-04
Cep192	1543.85	-0.49	4.11E-06
Sgo1	128.61	-2.22	1.47E-14
Kif2c	197.14	-0.67	3.75E-03
Cep57	508.14	-0.49	1.02E-03
Haus8	357.22	-0.53	2.06E-03
Sdccag8	409.32	-1.03	1.70E-10
Ninl	258.00	-0.51	9.83E-03
Sfi1	4544.49	-0.96	6.77E-42

Down-regulated genes following 4 h *P. aeruginosa* infection

HDACs deacetylate histones

gene_name	baseMean	log2FoldChange	p-value
Hist1h4m	1895.98	-0.46	1.44E-07
Chd4	15211.33	-0.46	3.74E-15
Mta1	2243.93	-0.57	2.62E-10
Mta3	717.21	-0.51	8.09E-05
Hist2h2aa1	20176.52	-0.53	2.08E-25
Hmg20b	939.02	-0.94	1.22E-15
Hdac10	339.46	-0.67	1.44E-04
Phf21a	765.14	-1.61	7.28E-34
Ncor1	7778.27	-0.22	2.38E-04
Ncor2	4955.79	-0.46	1.09E-10
Tbl1x	1504.01	-0.27	4.75E-03
Chd3	2833.60	-0.87	1.63E-30
Gatad2b	2015.70	-0.54	2.80E-09
Arid4a	1315.34	-1.27	3.03E-37
Hist1h3f	1363.45	-0.77	2.82E-15
Hist1h3c	1565.78	-1.06	1.94E-29
Hist1h3d	5161.95	-1.15	7.13E-67
Hist1h3b	2087.56	-1.17	3.98E-40
Hist1h3e	1469.33	-0.80	2.15E-17
Hist1h3h	2193.34	-1.30	1.93E-52
Hist1h3i	2266.74	-1.17	2.12E-43
Hist2h3b	2719.24	-1.26	1.53E-44
Hist1h4d	2684.89	-0.22	4.83E-03
Hist1h4f	1522.67	-0.94	2.25E-20
Hist1h4i	829.50	-0.61	3.24E-07
Hist1h4j	1441.05	-0.92	2.37E-21
Hist1h4k	1905.91	-0.78	1.36E-18
Hist1h4n	2367.62	-0.41	3.73E-07
Hist1h2ac	2453.61	-1.01	4.40E-38
Hist1h2ae	2624.68	-1.06	1.03E-41

Hist1h2ag	2722.66	-1.17	1.60E-51
Hist1h2an	1621.93	-1.22	1.30E-38
Hist1h2ab	3440.49	-1.14	1.02E-49
Hist2h2ac	1890.34	-0.54	1.78E-10
Hist1h2ba	229.34	-0.80	1.13E-03
Hist1h2bb	2089.34	-0.75	1.92E-19
Hist1h2be	2122.33	-0.28	4.48E-03
Hist1h2bg	2604.45	-0.34	5.84E-05
Hist1h2bk	2107.81	-0.67	6.47E-13
Hist1h2bm	887.41	-0.54	3.26E-06
Hist2h2bb	2245.72	-0.40	1.72E-06
Hist2h2be	130.69	-2.23	1.66E-14
Hist1h2ai	2965.60	-1.07	1.44E-44
Hist4h4	663.03	-0.67	4.23E-07
Hist1h4a	2002.28	-0.71	4.53E-17
Hist1h4b	1730.88	-0.68	7.49E-15
Hist1h3a	890.74	-1.15	1.28E-20
Hist2h2ab	259.65	-0.45	2.19E-02
Hist1h2ao	6653.04	-0.98	1.92E-56
Hist1h4h	2448.10	-0.75	9.01E-22
Hdac8	166.97	-0.85	5.20E-04
Suds3	1230.88	-0.63	5.59E-10
Tbl1xr1	1857.05	-0.32	1.74E-04
Arid4b	1313.52	-0.34	1.23E-03
Hist2h3c2	5169.81	-0.99	3.37E-43
Hist2h4	873.72	-1.00	8.48E-17
Hist1h3g	963.61	-1.41	1.92E-30

Cell cycle

gene_name	baseMean	log2FoldChange	p-value
Hist1h4m	1895.98	-0.46	1.44E-07
Pds5b	1250.00	-0.45	2.03E-05
Akap9	4914.28	-0.49	1.16E-11
Pot1a	258.92	-0.44	2.40E-02
Ccp110	479.85	-0.56	1.80E-04
Numa1	5094.99	-1.36	4.96E-91
Cenpi	110.18	-1.02	2.08E-03
Psme4	2271.55	-0.32	6.39E-05
Xpo1	3698.37	-0.18	1.01E-02
Tubg1	527.80	-0.97	1.67E-11
Tubg2	52.07	-1.35	8.59E-04
Espl1	655.14	-1.78	4.94E-38
Rfc4	280.56	-0.58	3.01E-03
Nsd2	1540.74	-0.44	3.75E-06

Babam2	657.09	-0.30	1.89E-02
Cenpf	896.17	-1.33	2.39E-27
Rcc2	10642.06	-0.45	7.59E-13
Gins4	365.98	-0.88	9.60E-07
Abl1	1575.77	-1.04	2.98E-26
Birc5	276.15	-1.30	1.05E-10
Atm	2310.03	-1.00	2.37E-33
Cep131	235.70	-2.19	5.37E-21
Bard1	234.54	-1.18	1.60E-08
Blm	522.51	-1.39	6.23E-21
Brca1	481.49	-2.47	1.19E-48
Btrc	473.00	-0.38	1.13E-02
Bub1	467.77	-0.89	1.57E-08
Bub1b	1040.27	-1.26	8.58E-29
Ccna2	484.19	-0.99	1.28E-10
Ccnd1	6753.54	-0.58	3.00E-22
Ccnd3	767.30	-1.58	2.57E-33
Ccne1	182.00	-1.60	2.45E-09
Cdc25b	701.67	-2.16	5.68E-52
Cdc7	165.79	-1.06	3.71E-05
Cdk2	485.38	-1.18	2.32E-12
Cdk4	3775.19	-0.25	2.46E-04
Cdkn1b	495.81	-1.52	8.43E-23
Cdkn2c	152.20	-2.01	4.71E-14
Cdkn2d	409.44	-1.11	3.28E-11
Cenpa	319.25	-0.47	7.85E-03
Smc3	2571.96	-0.42	1.27E-07
Dctn1	2732.14	-0.54	1.53E-11
Dync1h1	26338.96	-0.42	1.02E-12
Dyrk1a	1749.90	-0.20	2.19E-02
E2f1	321.73	-1.60	1.34E-17
Fen1	1209.77	-0.74	7.21E-13
Smc2	1355.94	-0.43	9.94E-06
Foxm1	796.63	-1.53	1.17E-35
Lpin1	565.55	-2.66	1.07E-63
Herc2	8121.72	-0.57	1.79E-18
Hist2h2aa1	20176.52	-0.53	2.08E-25
H2afx	2057.30	-0.42	7.98E-07
Hmmr	425.93	-1.12	1.86E-03
Incenp	2833.13	-0.92	4.05E-35
Cep250	2577.38	-0.27	1.29E-03
Lig1	1117.42	-1.03	1.24E-20
Lmnb1	1171.37	-0.55	3.21E-07

Mad1l1	587.01	-0.88	1.79E-10
Nme7	299.86	-0.43	2.03E-02
Mcm3	1752.59	-0.75	2.26E-17
Mcm2	1445.72	-1.52	5.07E-55
Mcm4	1262.06	-0.41	6.08E-05
Mcm5	1695.68	-1.02	6.50E-26
Mcm6	1747.12	-1.17	3.27E-37
Mcm7	932.03	-0.85	1.81E-13
Mdm4	2222.75	-0.31	9.18E-05
Rab8a	1525.76	-0.40	1.26E-05
Mre11a	815.10	-0.36	2.57E-03
Nek2	343.20	-1.19	9.28E-09
Nudc	1497.61	-0.34	1.29E-03
Odf2	1301.20	-0.31	1.97E-03
Pcnt	1842.88	-0.85	2.07E-22
Prkaca	1266.36	-0.54	3.03E-06
Plk1	363.52	-1.06	6.04E-09
Pola1	735.42	-1.09	3.17E-18
Pold1	918.74	-1.62	3.66E-38
Pole	813.29	-1.39	2.18E-29
Pole2	149.82	-0.72	1.01E-02
Prim1	244.66	-1.22	4.62E-08
Prim2	367.41	-0.77	5.41E-06
Psmd4	3242.96	-0.20	6.51E-03
Kif20a	408.39	-0.64	1.05E-04
Rad50	1401.39	-0.69	2.89E-10
Rad9a	332.61	-0.60	8.42E-04
Rangap1	1965.01	-0.73	4.17E-15
Rb1	856.10	-1.24	4.05E-25
Rbl1	747.41	-1.50	2.31E-29
Rbl2	1001.28	-1.37	5.34E-31
Rfc1	1564.04	-1.05	6.99E-26
Rfc2	512.60	-1.03	3.64E-12
Rpa2	327.43	-1.02	4.12E-08
Cep78	397.50	-1.47	7.23E-18
Kntc1	578.93	-1.16	7.72E-16
Aurkb	400.41	-1.55	3.98E-20
Aurka	316.16	-0.86	3.72E-06
Tfdp2	725.25	-1.87	7.49E-45
Cdk5rap2	1615.96	-0.41	3.86E-06
Cep164	1056.36	-1.36	4.21E-33
Ncaph	448.17	-1.68	2.94E-22
Tert	37.88	-1.57	6.57E-04

Mis18bp1	475.01	-1.04	3.58E-11
Ppp2r5d	1023.84	-0.69	6.20E-10
Nek9	3176.29	-0.51	1.27E-12
Tfdp1	1489.70	-0.74	2.05E-14
Haus4	157.56	-2.39	4.32E-17
Tmpo	2152.94	-1.21	1.04E-42
Top3a	389.73	-0.67	1.22E-04
Tubb4a	178.59	-0.79	9.71E-04
Tubb5	13443.86	-0.17	1.54E-03
Wee1	325.19	-1.79	2.88E-18
Aaas	927.32	-1.13	5.85E-20
Wrn	386.67	-0.66	6.16E-05
Lemd2	773.16	-0.45	2.93E-04
Ppp2r5b	375.21	-0.76	7.88E-06
Nup214	3309.15	-0.23	1.41E-03
Kif18a	198.90	-1.40	3.14E-09
Phf20	1818.19	-0.50	5.29E-09
Cdc14a	252.22	-0.54	9.19E-03
Cenpe	884.52	-0.90	1.14E-12
Haus6	437.55	-0.54	5.94E-04
Lin54	581.69	-0.86	9.40E-10
Fbxl18	427.97	-0.52	1.61E-03
H2afj	1326.92	-0.59	1.21E-08
Topbp1	2641.22	-0.95	2.71E-32
Ercc6l	162.82	-1.12	5.07E-06
Brip1	265.04	-1.96	3.08E-21
Akt3	1443.23	-0.72	4.66E-14
Cdc6	239.14	-1.27	1.18E-08
Mdc1	2013.00	-1.46	8.78E-49
Smc1a	2539.64	-0.47	1.32E-09
E2f2	483.16	-1.69	9.06E-26
Mcph1	787.11	-0.44	7.18E-04
Hist1h3f	1363.45	-0.77	2.82E-15
Mapk3	2594.23	-0.53	3.01E-10
Orc5	432.78	-0.90	1.36E-08
Cenph	117.24	-0.81	6.60E-03
Exo1	187.40	-1.86	2.26E-14
Cntrl	1734.50	-0.69	1.05E-13
Ppp2r5c	2652.41	-0.81	1.01E-25
Zw10	1016.94	-0.64	6.45E-09
Clspn	415.81	-2.58	3.68E-46
Dbf4	572.35	-0.51	3.81E-04
Gins2	102.97	-0.95	1.55E-03

Csnk1e	1367.78	-1.30	1.53E-34
Skp2	534.02	-0.75	2.29E-06
Zfp385a	997.20	-1.31	9.92E-31
Gtse1	910.70	-1.68	3.21E-41
Lcmt1	367.30	-0.39	2.41E-02
Hist1h3c	1565.78	-1.06	1.94E-29
Hist1h3d	5161.95	-1.15	7.13E-67
Hist1h3b	2087.56	-1.17	3.98E-40
Hist1h3e	1469.33	-0.80	2.15E-17
Hist1h3h	2193.34	-1.30	1.93E-52
Hist1h3i	2266.74	-1.17	2.12E-43
Hist2h3b	2719.24	-1.26	1.53E-44
Hist1h4d	2684.89	-0.22	4.83E-03
Hist1h4f	1522.67	-0.94	2.25E-20
Hist1h4i	829.50	-0.61	3.24E-07
Hist1h4j	1441.05	-0.92	2.37E-21
Hist1h4k	1905.91	-0.78	1.36E-18
Hist1h4n	2367.62	-0.41	3.73E-07
Hist1h2ac	2453.61	-1.01	4.40E-38
Hist1h2ae	2624.68	-1.06	1.03E-41
Hist1h2ag	2722.66	-1.17	1.60E-51
Hist1h2an	1621.93	-1.22	1.30E-38
Hist1h2ab	3440.49	-1.14	1.02E-49
Hist2h2ac	1890.34	-0.54	1.78E-10
Hist1h2ba	229.34	-0.80	1.13E-03
Hist1h2bb	2089.34	-0.75	1.92E-19
Hist1h2be	2122.33	-0.28	4.48E-03
Hist1h2bg	2604.45	-0.34	5.84E-05
Hist1h2bk	2107.81	-0.67	6.47E-13
Hist1h2bm	887.41	-0.54	3.26E-06
Hist2h2be	130.69	-2.23	1.66E-14
Hist1h2ai	2965.60	-1.07	1.44E-44
Hist4h4	663.03	-0.67	4.23E-07
Hist1h4a	2002.28	-0.71	4.53E-17
Hist1h4b	1730.88	-0.68	7.49E-15
Dna2	458.70	-1.96	1.06E-31
Tubgcp6	907.13	-1.01	2.46E-18
Hist1h3a	890.74	-1.15	1.28E-20
Nsl1	175.84	-1.17	1.22E-06
Nup85	821.33	-0.51	1.72E-05
Acd	466.09	-0.68	1.01E-05
Chek2	230.85	-3.24	4.00E-38
Cdca8	481.29	-1.23	8.32E-16

Cenpo	321.36	-0.41	2.51E-02
Ncaph2	1448.25	-0.52	4.13E-08
Actr1a	2965.34	-0.21	5.18E-03
Ncapg	445.19	-0.80	1.05E-06
Nup210	1246.12	-1.01	1.46E-23
Sfn	53.88	-0.95	1.47E-02
Mad2l1	288.01	-0.87	4.13E-06
Fzr1	1112.64	-0.42	1.09E-04
Clip1	2941.90	-1.09	1.35E-49
Gmnn	251.16	-1.91	1.56E-18
Anapc5	2992.22	-0.27	5.91E-04
Nek7	1495.37	-0.35	2.06E-04
Nek6	957.31	-0.63	5.68E-08
Cenpk	78.70	-1.13	3.27E-03
Lpin2	952.86	-0.98	7.21E-17
Ska2	120.26	-0.76	9.16E-03
Anapc11	613.95	-0.45	7.85E-04
Haus2	541.62	-0.47	4.17E-03
Spc25	204.77	-1.18	1.66E-07
Ska1	58.94	-2.28	2.65E-08
Hist1h2ao	6653.04	-0.98	1.92E-56
Cenpm	100.40	-1.18	2.45E-04
Mcm8	100.15	-1.16	1.53E-04
Dsn1	170.29	-0.71	4.88E-03
Nuf2	324.28	-0.75	2.74E-05
Pmf1	425.82	-0.59	7.42E-04
Ndc80	211.34	-1.32	7.69E-09
Mastl	158.83	-1.98	8.78E-13
Fbxo5	140.31	-1.65	2.28E-09
Cdt1	1811.19	-0.72	3.68E-16
Nde1	918.83	-0.83	4.27E-13
Cdca5	195.79	-2.18	8.04E-19
Pold3	735.33	-0.52	3.44E-05
Zwilch	192.21	-0.95	6.32E-05
Dynll2	992.76	-0.65	3.80E-09
Babam1	1359.70	-0.22	2.50E-02
Rpa1	1177.93	-0.78	3.02E-14
Ncapd2	1291.48	-1.42	3.20E-44
Sgo2a	227.33	-0.63	4.28E-03
Rfc3	205.73	-0.67	2.39E-03
Gins1	102.61	-1.44	2.13E-06
Hist1h4h	2448.10	-0.75	9.01E-22
Dctn2	2085.49	-0.38	3.62E-06

Cenps	37.43	-1.27	5.42E-03
Mcm10	353.30	-1.34	7.27E-13
Smc4	1485.96	-0.59	9.21E-08
Rnf168	522.63	-0.44	3.69E-03
Hdac8	166.97	-0.85	5.20E-04
Spdl1	386.58	-1.65	1.14E-21
Oip5	46.33	-1.47	4.52E-04
Cep192	1433.98	-0.88	3.38E-17
Nup93	1686.90	-0.48	6.18E-07
Kif23	653.32	-1.20	3.71E-19
Cenpu	60.33	-1.49	1.31E-04
Haus5	140.71	-1.26	2.50E-06
Esco2	180.20	-2.53	5.31E-23
Mzt2	164.76	-0.88	3.87E-04
Tpx2	828.64	-0.84	1.23E-11
Cenpn	183.78	-1.60	2.93E-11
Sgo1	159.07	-1.20	2.93E-06
Kif2c	174.63	-1.31	4.30E-07
Cep57	510.32	-0.59	5.21E-05
Cep72	43.16	-1.96	1.19E-05
Gorasp1	314.16	-0.54	3.53E-03
Ckap5	2860.41	-0.31	2.81E-04
Ncapg2	875.62	-0.96	4.66E-16
Haus8	295.01	-1.49	6.06E-15
Clasp2	1657.85	-0.31	4.17E-04
Sdccag8	398.55	-1.31	5.36E-15
H2afv	213.26	-1.00	5.19E-06
Bora	278.12	-0.68	4.63E-04
Ninl	218.49	-1.32	1.01E-09
Ncapd3	898.26	-1.30	3.90E-29
Gins3	337.00	-2.74	1.17E-43
Sfi1	5681.69	-0.26	4.11E-05
Hist2h3c2	5169.81	-0.99	3.37E-43
Hist2h4	873.72	-1.00	8.48E-17
Hist1h3g	963.61	-1.41	1.92E-30
Anapc2	1840.61	-0.37	7.70E-05

Activated PKN stimulates transcription of AR regulated genes KLK2 and KLK3

gene_name	baseMean	log2FoldChange	p-value
Hist1h4m	1895.98	-0.46	1.44E-07
Hist2h2aa1	20176.52	-0.53	2.08E-25
H2afx	2057.30	-0.42	7.98E-07
Ncoa2	1849.47	-0.69	8.95E-15
H2afj	1326.92	-0.59	1.21E-08

Hist1h3f	1363.45	-0.77	2.82E-15
Hist1h3c	1565.78	-1.06	1.94E-29
Hist1h3d	5161.95	-1.15	7.13E-67
Hist1h3b	2087.56	-1.17	3.98E-40
Hist1h3e	1469.33	-0.80	2.15E-17
Hist1h3h	2193.34	-1.30	1.93E-52
Hist1h3i	2266.74	-1.17	2.12E-43
Hist2h3b	2719.24	-1.26	1.53E-44
Hist1h4d	2684.89	-0.22	4.83E-03
Hist1h4f	1522.67	-0.94	2.25E-20
Hist1h4i	829.50	-0.61	3.24E-07
Hist1h4j	1441.05	-0.92	2.37E-21
Hist1h4k	1905.91	-0.78	1.36E-18
Hist1h4n	2367.62	-0.41	3.73E-07
Hist1h2ac	2453.61	-1.01	4.40E-38
Hist1h2ae	2624.68	-1.06	1.03E-41
Hist1h2ag	2722.66	-1.17	1.60E-51
Hist1h2an	1621.93	-1.22	1.30E-38
Hist1h2ab	3440.49	-1.14	1.02E-49
Hist2h2ac	1890.34	-0.54	1.78E-10
Hist1h2ba	229.34	-0.80	1.13E-03
Hist1h2bb	2089.34	-0.75	1.92E-19
Hist1h2be	2122.33	-0.28	4.48E-03
Hist1h2bg	2604.45	-0.34	5.84E-05
Hist1h2bk	2107.81	-0.67	6.47E-13
Hist1h2bm	887.41	-0.54	3.26E-06
Hist2h2be	130.69	-2.23	1.66E-14
Hist1h2ai	2965.60	-1.07	1.44E-44
Hist4h4	663.03	-0.67	4.23E-07
Pkn1	2039.22	-0.95	1.86E-29
Hist1h4a	2002.28	-0.71	4.53E-17
Hist1h4b	1730.88	-0.68	7.49E-15
Hist1h3a	890.74	-1.15	1.28E-20
Hist1h2ao	6653.04	-0.98	1.92E-56
Hist1h4h	2448.10	-0.75	9.01E-22
H2afv	213.26	-1.00	5.19E-06
Hist2h3c2	5169.81	-0.99	3.37E-43
Hist2h4	873.72	-1.00	8.48E-17
Hist1h3g	963.61	-1.41	1.92E-30

Condensation of prophase chromosomes

gene_name	baseMean	log2FoldChange	p-value
Hist1h4m	1895.98	-0.46	1.44E-07
Smc2	1355.94	-0.43	9.94E-06

Hist2h2aa1	20176.52	-0.53	2.08E-25
H2afx	2057.30	-0.42	7.98E-07
Plk1	363.52	-1.06	6.04E-09
Rb1	856.10	-1.24	4.05E-25
H2afj	1326.92	-0.59	1.21E-08
Mcph1	787.11	-0.44	7.18E-04
Hist1h3f	1363.45	-0.77	2.82E-15
Hist1h3c	1565.78	-1.06	1.94E-29
Hist1h3d	5161.95	-1.15	7.13E-67
Hist1h3b	2087.56	-1.17	3.98E-40
Hist1h3e	1469.33	-0.80	2.15E-17
Hist1h3h	2193.34	-1.30	1.93E-52
Hist1h3i	2266.74	-1.17	2.12E-43
Hist2h3b	2719.24	-1.26	1.53E-44
Hist1h4d	2684.89	-0.22	4.83E-03
Hist1h4f	1522.67	-0.94	2.25E-20
Hist1h4i	829.50	-0.61	3.24E-07
Hist1h4j	1441.05	-0.92	2.37E-21
Hist1h4k	1905.91	-0.78	1.36E-18
Hist1h4n	2367.62	-0.41	3.73E-07
Hist1h2ac	2453.61	-1.01	4.40E-38
Hist1h2ae	2624.68	-1.06	1.03E-41
Hist1h2ag	2722.66	-1.17	1.60E-51
Hist1h2an	1621.93	-1.22	1.30E-38
Hist1h2ab	3440.49	-1.14	1.02E-49
Hist2h2ac	1890.34	-0.54	1.78E-10
Hist1h2ba	229.34	-0.80	1.13E-03
Hist1h2bb	2089.34	-0.75	1.92E-19
Hist1h2be	2122.33	-0.28	4.48E-03
Hist1h2bg	2604.45	-0.34	5.84E-05
Hist1h2bk	2107.81	-0.67	6.47E-13
Hist1h2bm	887.41	-0.54	3.26E-06
Hist2h2be	130.69	-2.23	1.66E-14
Hist1h2ai	2965.60	-1.07	1.44E-44
Hist4h4	663.03	-0.67	4.23E-07
Hist1h4a	2002.28	-0.71	4.53E-17
Hist1h4b	1730.88	-0.68	7.49E-15
Hist1h3a	890.74	-1.15	1.28E-20
Ncaph2	1448.25	-0.52	4.13E-08
Hist1h2ao	6653.04	-0.98	1.92E-56
Hist1h4h	2448.10	-0.75	9.01E-22
Smc4	1485.96	-0.59	9.21E-08
Ncapg2	875.62	-0.96	4.66E-16

H2afv	213.26	-1.00	5.19E-06
Ncapd3	898.26	-1.30	3.90E-29
Hist2h3c2	5169.81	-0.99	3.37E-43
Hist2h4	873.72	-1.00	8.48E-17
Hist1h3g	963.61	-1.41	1.92E-30

Signaling by Rho GTPases

gene_name	baseMean	log2FoldChange	p-value
Hist1h4m	1895.98	-0.46	1.44E-07
Arhgef18	429.88	-0.43	5.76E-03
Cenpi	110.18	-1.02	2.08E-03
Xpo1	3698.37	-0.18	1.01E-02
Cenpf	896.17	-1.33	2.39E-27
Baiap2	812.40	-1.31	1.22E-24
Rcc2	10642.06	-0.45	7.59E-13
Abl1	1575.77	-1.04	2.98E-26
Birc5	276.15	-1.30	1.05E-10
Rhod	121.27	-3.03	1.84E-19
Arhgdib	5940.12	-0.43	3.56E-11
Bub1	467.77	-0.89	1.57E-08
Bub1b	1040.27	-1.26	8.58E-29
Cenpa	319.25	-0.47	7.85E-03
Cit	428.58	-1.73	3.41E-26
Dlg4	206.16	-0.53	2.15E-02
Dync1h1	26338.96	-0.42	1.02E-12
Ect2	325.75	-1.23	1.97E-11
Hist2h2aa1	20176.52	-0.53	2.08E-25
H2afx	2057.30	-0.42	7.98E-07
Incenp	2833.13	-0.92	4.05E-35
Inpp5b	775.17	-0.91	4.81E-12
Itsn1	1195.98	-0.92	3.93E-18
Kif5a	1051.66	-2.03	2.17E-68
Klc1	2456.21	-0.86	1.91E-27
Arhgef1	5731.37	-0.21	1.39E-03
Limk1	188.14	-0.89	2.21E-04
Mad1l1	587.01	-0.88	1.79E-10
Mcf2l	999.47	-0.30	6.04E-03
Men1	1253.80	-0.24	1.65E-02
Ncf2	2656.26	-0.38	6.07E-07
Ncoa2	1849.47	-0.69	8.95E-15
Nf2	3098.49	-0.68	1.18E-19
Nudc	1497.61	-0.34	1.29E-03
Pak1	966.44	-0.95	1.42E-15
Pik3r2	1193.11	-1.81	4.59E-65

Plk1	363.52	-1.06	6.04E-09
Rac2	7972.15	-0.42	4.93E-11
Rangap1	1965.01	-0.73	4.17E-15
Ralbp1	1638.45	-0.97	3.61E-25
Rtkn	87.82	-2.12	1.80E-09
Kntc1	578.93	-1.16	7.72E-16
Aurkb	400.41	-1.55	3.98E-20
Depdc7	67.30	-1.04	5.16E-03
Arhgef11	1920.72	-1.10	5.53E-34
Rhot2	864.24	-0.53	8.69E-06
Wipf1	2652.46	-1.12	4.75E-49
Arhgap9	894.50	-0.98	3.11E-17
Ppp2r5d	1023.84	-0.69	6.20E-10
Depdc1b	30.64	-1.32	5.79E-03
Tubb4a	178.59	-0.79	9.71E-04
Vav1	2863.84	-0.66	8.14E-18
Arhgap39	863.26	-0.65	1.70E-08
Mkl1	2059.72	-1.47	1.21E-58
Fgd4	1508.33	-1.58	1.75E-51
Pik3c3	955.77	-0.66	6.27E-09
Ppp2r5b	375.21	-0.76	7.88E-06
Arhgef4	209.69	-0.87	3.88E-04
Kif18a	198.90	-1.40	3.14E-09
Arhgap11a	953.55	-0.44	1.03E-04
Cenpe	884.52	-0.90	1.14E-12
Arhgef39	68.38	-1.93	4.20E-07
Arhgap24	233.22	-1.60	1.69E-12
Arhgap25	574.29	-1.39	1.33E-21
H2afj	1326.92	-0.59	1.21E-08
Arhgap33	60.84	-1.92	2.38E-06
Prc1	837.86	-1.11	3.51E-20
Arhgef10	521.19	-0.98	8.49E-10
Stard8	253.26	-0.95	3.67E-06
Ercc6l	162.82	-1.12	5.07E-06
Wasf2	4406.40	-0.17	8.44E-03
Rhobtb2	318.16	-1.05	2.34E-08
Srgap3	816.91	-0.41	4.61E-04
Hist1h3f	1363.45	-0.77	2.82E-15
Pkn3	153.69	-0.67	1.31E-02
Fgd2	163.03	-2.30	7.22E-06
Mapk3	2594.23	-0.53	3.01E-10
Arhgef40	566.81	-2.19	2.38E-43
Cenph	117.24	-0.81	6.60E-03

Ppp2r5c	2652.41	-0.81	1.01E-25
Racgap1	723.30	-1.80	3.52E-34
Zw10	1016.94	-0.64	6.45E-09
Plekhg5	175.59	-3.19	9.88E-29
Prex1	1907.77	-1.33	1.32E-51
Fgd3	1879.14	-0.89	8.50E-24
Hist1h3c	1565.78	-1.06	1.94E-29
Hist1h3d	5161.95	-1.15	7.13E-67
Hist1h3b	2087.56	-1.17	3.98E-40
Hist1h3e	1469.33	-0.80	2.15E-17
Hist1h3h	2193.34	-1.30	1.93E-52
Hist1h3i	2266.74	-1.17	2.12E-43
Hist2h3b	2719.24	-1.26	1.53E-44
Hist1h4d	2684.89	-0.22	4.83E-03
Hist1h4f	1522.67	-0.94	2.25E-20
Hist1h4i	829.50	-0.61	3.24E-07
Hist1h4j	1441.05	-0.92	2.37E-21
Hist1h4k	1905.91	-0.78	1.36E-18
Hist1h4n	2367.62	-0.41	3.73E-07
Hist1h2ac	2453.61	-1.01	4.40E-38
Hist1h2ae	2624.68	-1.06	1.03E-41
Hist1h2ag	2722.66	-1.17	1.60E-51
Hist1h2an	1621.93	-1.22	1.30E-38
Hist1h2ab	3440.49	-1.14	1.02E-49
Hist2h2ac	1890.34	-0.54	1.78E-10
Hist1h2ba	229.34	-0.80	1.13E-03
Hist1h2bb	2089.34	-0.75	1.92E-19
Hist1h2be	2122.33	-0.28	4.48E-03
Hist1h2bg	2604.45	-0.34	5.84E-05
Hist1h2bk	2107.81	-0.67	6.47E-13
Hist1h2bm	887.41	-0.54	3.26E-06
Hist2h2be	130.69	-2.23	1.66E-14
Hist1h2ai	2965.60	-1.07	1.44E-44
Hist4h4	663.03	-0.67	4.23E-07
Pkn1	2039.22	-0.95	1.86E-29
Hist1h4a	2002.28	-0.71	4.53E-17
Hist1h4b	1730.88	-0.68	7.49E-15
Hist1h3a	890.74	-1.15	1.28E-20
Obscn	43.53	-1.35	1.49E-03
Kif14	331.68	-1.55	1.06E-16
Nsl1	175.84	-1.17	1.22E-06
Iqgap3	552.43	-1.27	2.23E-17
Nup85	821.33	-0.51	1.72E-05

Cdca8	481.29	-1.23	8.32E-16
Cenpo	321.36	-0.41	2.51E-02
Arhgap27	727.76	-1.80	1.18E-42
Sfn	53.88	-0.95	1.47E-02
Mad2l1	288.01	-0.87	4.13E-06
Clip1	2941.90	-1.09	1.35E-49
Vav3	867.92	-0.39	6.70E-04
Cenpk	78.70	-1.13	3.27E-03
Ska2	120.26	-0.76	9.16E-03
Spc25	204.77	-1.18	1.66E-07
Ska1	58.94	-2.28	2.65E-08
Hist1h2ao	6653.04	-0.98	1.92E-56
Cenpm	100.40	-1.18	2.45E-04
Dsn1	170.29	-0.71	4.88E-03
Nuf2	324.28	-0.75	2.74E-05
Pmf1	425.82	-0.59	7.42E-04
Ndc80	211.34	-1.32	7.69E-09
Nde1	918.83	-0.83	4.27E-13
Myl12b	2628.22	-0.27	9.43E-04
Zwilch	192.21	-0.95	6.32E-05
Dynll2	992.76	-0.65	3.80E-09
Sgo2a	227.33	-0.63	4.28E-03
Hist1h4h	2448.10	-0.75	9.01E-22
Cenps	37.43	-1.27	5.42E-03
Spdl1	386.58	-1.65	1.14E-21
Arhgap17	1846.87	-1.52	2.26E-66
Arhgap45	5248.23	-1.86	2.11E-177
Arhgap19	46.82	-1.77	4.16E-05
Arhgap26	170.56	-1.30	1.09E-07
Cenpu	60.33	-1.49	1.31E-04
Cenpn	183.78	-1.60	2.93E-11
Sgo1	159.07	-1.20	2.93E-06
Arhgef10l	2435.74	-0.34	3.08E-05
Arhgef6	4065.90	-1.10	1.88E-55
Kif2c	174.63	-1.31	4.30E-07
Arhgap18	450.41	-1.53	3.09E-20
Klc4	835.65	-2.02	3.44E-47
Arhgap12	790.71	-0.66	1.07E-07
Pik3r4	1019.12	-0.38	5.40E-04
Ckap5	2860.41	-0.31	2.81E-04
Arhgap15	203.51	-1.15	1.84E-06
Tax1bp3	291.64	-0.45	2.16E-02
Clasp2	1657.85	-0.31	4.17E-04

Cyfip2	145.63	-1.60	8.25E-09
H2afv	213.26	-1.00	5.19E-06
Gmip	2988.02	-0.24	2.15E-03
Hist2h3c2	5169.81	-0.99	3.37E-43
Hist2h4	873.72	-1.00	8.48E-17
Hist1h3g	963.61	-1.41	1.92E-30

Cell cycle, mitotic

gene_name	baseMean	log2FoldChange	p-value
Hist1h4m	1895.98	-0.46	1.44E-07
Pds5b	1250.00	-0.45	2.03E-05
Akap9	4914.28	-0.49	1.16E-11
Ccp110	479.85	-0.56	1.80E-04
Numa1	5094.99	-1.36	4.96E-91
Cenpi	110.18	-1.02	2.08E-03
Psme4	2271.55	-0.32	6.39E-05
Xpo1	3698.37	-0.18	1.01E-02
Tubg1	527.80	-0.97	1.67E-11
Tubg2	52.07	-1.35	8.59E-04
Espl1	655.14	-1.78	4.94E-38
Rfc4	280.56	-0.58	3.01E-03
Cenpf	896.17	-1.33	2.39E-27
Rcc2	10642.06	-0.45	7.59E-13
Gins4	365.98	-0.88	9.60E-07
Abl1	1575.77	-1.04	2.98E-26
Birc5	276.15	-1.30	1.05E-10
Cep131	235.70	-2.19	5.37E-21
Btrc	473.00	-0.38	1.13E-02
Bub1	467.77	-0.89	1.57E-08
Bub1b	1040.27	-1.26	8.58E-29
Ccna2	484.19	-0.99	1.28E-10
Ccnd1	6753.54	-0.58	3.00E-22
Ccnd3	767.30	-1.58	2.57E-33
Ccne1	182.00	-1.60	2.45E-09
Cdc25b	701.67	-2.16	5.68E-52
Cdc7	165.79	-1.06	3.71E-05
Cdk2	485.38	-1.18	2.32E-12
Cdk4	3775.19	-0.25	2.46E-04
Cdkn1b	495.81	-1.52	8.43E-23
Cdkn2c	152.20	-2.01	4.71E-14
Cdkn2d	409.44	-1.11	3.28E-11
Cenpa	319.25	-0.47	7.85E-03
Smc3	2571.96	-0.42	1.27E-07
Dctn1	2732.14	-0.54	1.53E-11

Dync1h1	26338.96	-0.42	1.02E-12
Dyrk1a	1749.90	-0.20	2.19E-02
E2f1	321.73	-1.60	1.34E-17
Fen1	1209.77	-0.74	7.21E-13
Smc2	1355.94	-0.43	9.94E-06
Foxm1	796.63	-1.53	1.17E-35
Lpin1	565.55	-2.66	1.07E-63
Hist2h2aa1	20176.52	-0.53	2.08E-25
H2afx	2057.30	-0.42	7.98E-07
Hmmr	425.93	-1.12	1.86E-03
Incenp	2833.13	-0.92	4.05E-35
Cep250	2577.38	-0.27	1.29E-03
Lig1	1117.42	-1.03	1.24E-20
Lmnb1	1171.37	-0.55	3.21E-07
Mad1l1	587.01	-0.88	1.79E-10
Nme7	299.86	-0.43	2.03E-02
Mcm3	1752.59	-0.75	2.26E-17
Mcm2	1445.72	-1.52	5.07E-55
Mcm4	1262.06	-0.41	6.08E-05
Mcm5	1695.68	-1.02	6.50E-26
Mcm6	1747.12	-1.17	3.27E-37
Mcm7	932.03	-0.85	1.81E-13
Rab8a	1525.76	-0.40	1.26E-05
Nek2	343.20	-1.19	9.28E-09
Nudc	1497.61	-0.34	1.29E-03
Odf2	1301.20	-0.31	1.97E-03
Pcnt	1842.88	-0.85	2.07E-22
Prkaca	1266.36	-0.54	3.03E-06
Plk1	363.52	-1.06	6.04E-09
Pola1	735.42	-1.09	3.17E-18
Pold1	918.74	-1.62	3.66E-38
Pole	813.29	-1.39	2.18E-29
Pole2	149.82	-0.72	1.01E-02
Prim1	244.66	-1.22	4.62E-08
Prim2	367.41	-0.77	5.41E-06
Psmd4	3242.96	-0.20	6.51E-03
Kif20a	408.39	-0.64	1.05E-04
Rangap1	1965.01	-0.73	4.17E-15
Rb1	856.10	-1.24	4.05E-25
Rbl1	747.41	-1.50	2.31E-29
Rbl2	1001.28	-1.37	5.34E-31
Rfc1	1564.04	-1.05	6.99E-26
Rfc2	512.60	-1.03	3.64E-12

Rpa2	327.43	-1.02	4.12E-08
Cep78	397.50	-1.47	7.23E-18
Kntc1	578.93	-1.16	7.72E-16
Aurkb	400.41	-1.55	3.98E-20
Aurka	316.16	-0.86	3.72E-06
Tfdp2	725.25	-1.87	7.49E-45
Cdk5rap2	1615.96	-0.41	3.86E-06
Cep164	1056.36	-1.36	4.21E-33
Ncaph	448.17	-1.68	2.94E-22
Ppp2r5d	1023.84	-0.69	6.20E-10
Nek9	3176.29	-0.51	1.27E-12
Tfdp1	1489.70	-0.74	2.05E-14
Haus4	157.56	-2.39	4.32E-17
Tmpo	2152.94	-1.21	1.04E-42
Tubb4a	178.59	-0.79	9.71E-04
Tubb5	13443.86	-0.17	1.54E-03
Wee1	325.19	-1.79	2.88E-18
Aaas	927.32	-1.13	5.85E-20
Lemd2	773.16	-0.45	2.93E-04
Ppp2r5b	375.21	-0.76	7.88E-06
Nup214	3309.15	-0.23	1.41E-03
Kif18a	198.90	-1.40	3.14E-09
Cdc14a	252.22	-0.54	9.19E-03
Cenpe	884.52	-0.90	1.14E-12
Haus6	437.55	-0.54	5.94E-04
Lin54	581.69	-0.86	9.40E-10
Fbxl18	427.97	-0.52	1.61E-03
H2afj	1326.92	-0.59	1.21E-08
Ercc6l	162.82	-1.12	5.07E-06
Akt3	1443.23	-0.72	4.66E-14
Cdc6	239.14	-1.27	1.18E-08
Smc1a	2539.64	-0.47	1.32E-09
E2f2	483.16	-1.69	9.06E-26
Mcph1	787.11	-0.44	7.18E-04
Hist1h3f	1363.45	-0.77	2.82E-15
Mapk3	2594.23	-0.53	3.01E-10
Orc5	432.78	-0.90	1.36E-08
Cenph	117.24	-0.81	6.60E-03
Cntrl	1734.50	-0.69	1.05E-13
Ppp2r5c	2652.41	-0.81	1.01E-25
Zw10	1016.94	-0.64	6.45E-09
Dbf4	572.35	-0.51	3.81E-04
Gins2	102.97	-0.95	1.55E-03

Csnk1e	1367.78	-1.30	1.53E-34
Skp2	534.02	-0.75	2.29E-06
Gtse1	910.70	-1.68	3.21E-41
Lcmt1	367.30	-0.39	2.41E-02
Hist1h3c	1565.78	-1.06	1.94E-29
Hist1h3d	5161.95	-1.15	7.13E-67
Hist1h3b	2087.56	-1.17	3.98E-40
Hist1h3e	1469.33	-0.80	2.15E-17
Hist1h3h	2193.34	-1.30	1.93E-52
Hist1h3i	2266.74	-1.17	2.12E-43
Hist2h3b	2719.24	-1.26	1.53E-44
Hist1h4d	2684.89	-0.22	4.83E-03
Hist1h4f	1522.67	-0.94	2.25E-20
Hist1h4i	829.50	-0.61	3.24E-07
Hist1h4j	1441.05	-0.92	2.37E-21
Hist1h4k	1905.91	-0.78	1.36E-18
Hist1h4n	2367.62	-0.41	3.73E-07
Hist1h2ac	2453.61	-1.01	4.40E-38
Hist1h2ae	2624.68	-1.06	1.03E-41
Hist1h2ag	2722.66	-1.17	1.60E-51
Hist1h2an	1621.93	-1.22	1.30E-38
Hist1h2ab	3440.49	-1.14	1.02E-49
Hist2h2ac	1890.34	-0.54	1.78E-10
Hist1h2ba	229.34	-0.80	1.13E-03
Hist1h2bb	2089.34	-0.75	1.92E-19
Hist1h2be	2122.33	-0.28	4.48E-03
Hist1h2bg	2604.45	-0.34	5.84E-05
Hist1h2bk	2107.81	-0.67	6.47E-13
Hist1h2bm	887.41	-0.54	3.26E-06
Hist2h2be	130.69	-2.23	1.66E-14
Hist1h2ai	2965.60	-1.07	1.44E-44
Hist4h4	663.03	-0.67	4.23E-07
Hist1h4a	2002.28	-0.71	4.53E-17
Hist1h4b	1730.88	-0.68	7.49E-15
Dna2	458.70	-1.96	1.06E-31
Tubgcp6	907.13	-1.01	2.46E-18
Hist1h3a	890.74	-1.15	1.28E-20
Nsl1	175.84	-1.17	1.22E-06
Nup85	821.33	-0.51	1.72E-05
Cdca8	481.29	-1.23	8.32E-16
Cenpo	321.36	-0.41	2.51E-02
Ncaph2	1448.25	-0.52	4.13E-08
Actr1a	2965.34	-0.21	5.18E-03

Ncapg	445.19	-0.80	1.05E-06
Nup210	1246.12	-1.01	1.46E-23
Mad2l1	288.01	-0.87	4.13E-06
Fzr1	1112.64	-0.42	1.09E-04
Clip1	2941.90	-1.09	1.35E-49
Gmnn	251.16	-1.91	1.56E-18
Anapc5	2992.22	-0.27	5.91E-04
Nek7	1495.37	-0.35	2.06E-04
Nek6	957.31	-0.63	5.68E-08
Cenpk	78.70	-1.13	3.27E-03
Lpin2	952.86	-0.98	7.21E-17
Ska2	120.26	-0.76	9.16E-03
Anapc11	613.95	-0.45	7.85E-04
Haus2	541.62	-0.47	4.17E-03
Spc25	204.77	-1.18	1.66E-07
Ska1	58.94	-2.28	2.65E-08
Hist1h2ao	6653.04	-0.98	1.92E-56
Cenpm	100.40	-1.18	2.45E-04
Mcm8	100.15	-1.16	1.53E-04
Dsn1	170.29	-0.71	4.88E-03
Nuf2	324.28	-0.75	2.74E-05
Pmf1	425.82	-0.59	7.42E-04
Ndc80	211.34	-1.32	7.69E-09
Mastl	158.83	-1.98	8.78E-13
Fbxo5	140.31	-1.65	2.28E-09
Cdt1	1811.19	-0.72	3.68E-16
Nde1	918.83	-0.83	4.27E-13
Cdca5	195.79	-2.18	8.04E-19
Pold3	735.33	-0.52	3.44E-05
Zwilch	192.21	-0.95	6.32E-05
Dynll2	992.76	-0.65	3.80E-09
Rpa1	1177.93	-0.78	3.02E-14
Ncapd2	1291.48	-1.42	3.20E-44
Sgo2a	227.33	-0.63	4.28E-03
Rfc3	205.73	-0.67	2.39E-03
Gins1	102.61	-1.44	2.13E-06
Hist1h4h	2448.10	-0.75	9.01E-22
Dctn2	2085.49	-0.38	3.62E-06
Cenps	37.43	-1.27	5.42E-03
Mcm10	353.30	-1.34	7.27E-13
Smc4	1485.96	-0.59	9.21E-08
Hdac8	166.97	-0.85	5.20E-04
Spdl1	386.58	-1.65	1.14E-21

Cep192	1433.98	-0.88	3.38E-17
Nup93	1686.90	-0.48	6.18E-07
Kif23	653.32	-1.20	3.71E-19
Cenpu	60.33	-1.49	1.31E-04
Haus5	140.71	-1.26	2.50E-06
Esco2	180.20	-2.53	5.31E-23
Mzt2	164.76	-0.88	3.87E-04
Tpx2	828.64	-0.84	1.23E-11
Cenpn	183.78	-1.60	2.93E-11
Sgo1	159.07	-1.20	2.93E-06
Kif2c	174.63	-1.31	4.30E-07
Cep57	510.32	-0.59	5.21E-05
Cep72	43.16	-1.96	1.19E-05
Gorasp1	314.16	-0.54	3.53E-03
Ckap5	2860.41	-0.31	2.81E-04
Ncapg2	875.62	-0.96	4.66E-16
Haus8	295.01	-1.49	6.06E-15
Clasp2	1657.85	-0.31	4.17E-04
Sdccag8	398.55	-1.31	5.36E-15
H2afv	213.26	-1.00	5.19E-06
Bora	278.12	-0.68	4.63E-04
Ninl	218.49	-1.32	1.01E-09
Ncapd3	898.26	-1.30	3.90E-29
Gins3	337.00	-2.74	1.17E-43
Sfi1	5681.69	-0.26	4.11E-05
Hist2h3c2	5169.81	-0.99	3.37E-43
Hist2h4	873.72	-1.00	8.48E-17
Hist1h3g	963.61	-1.41	1.92E-30
Anapc2	1840.61	-0.37	7.70E-05

M phase

gene_name	baseMean	log2FoldChange	p-value
Hist1h4m	1895.98	-0.46	1.44E-07
Pds5b	1250.00	-0.45	2.03E-05
Akap9	4914.28	-0.49	1.16E-11
Ccp110	479.85	-0.56	1.80E-04
Numa1	5094.99	-1.36	4.96E-91
Cenpi	110.18	-1.02	2.08E-03
Psme4	2271.55	-0.32	6.39E-05
Xpo1	3698.37	-0.18	1.01E-02
Tubg1	527.80	-0.97	1.67E-11
Tubg2	52.07	-1.35	8.59E-04
Espl1	655.14	-1.78	4.94E-38
Cenpf	896.17	-1.33	2.39E-27

Rcc2	10642.06	-0.45	7.59E-13
Birc5	276.15	-1.30	1.05E-10
Cep131	235.70	-2.19	5.37E-21
Bub1	467.77	-0.89	1.57E-08
Bub1b	1040.27	-1.26	8.58E-29
Cenpa	319.25	-0.47	7.85E-03
Smc3	2571.96	-0.42	1.27E-07
Dctn1	2732.14	-0.54	1.53E-11
Dync1h1	26338.96	-0.42	1.02E-12
Smc2	1355.94	-0.43	9.94E-06
Lpin1	565.55	-2.66	1.07E-63
Hist2h2aa1	20176.52	-0.53	2.08E-25
H2afx	2057.30	-0.42	7.98E-07
Incenp	2833.13	-0.92	4.05E-35
Cep250	2577.38	-0.27	1.29E-03
Lmnb1	1171.37	-0.55	3.21E-07
Mad1l1	587.01	-0.88	1.79E-10
Nme7	299.86	-0.43	2.03E-02
Nek2	343.20	-1.19	9.28E-09
Nudc	1497.61	-0.34	1.29E-03
Odf2	1301.20	-0.31	1.97E-03
Pcnt	1842.88	-0.85	2.07E-22
Prkaca	1266.36	-0.54	3.03E-06
Plk1	363.52	-1.06	6.04E-09
Psmd4	3242.96	-0.20	6.51E-03
Kif20a	408.39	-0.64	1.05E-04
Rangap1	1965.01	-0.73	4.17E-15
Rb1	856.10	-1.24	4.05E-25
Cep78	397.50	-1.47	7.23E-18
Kntc1	578.93	-1.16	7.72E-16
Aurkb	400.41	-1.55	3.98E-20
Cdk5rap2	1615.96	-0.41	3.86E-06
Cep164	1056.36	-1.36	4.21E-33
Ncaph	448.17	-1.68	2.94E-22
Ppp2r5d	1023.84	-0.69	6.20E-10
Nek9	3176.29	-0.51	1.27E-12
Haus4	157.56	-2.39	4.32E-17
Tmpo	2152.94	-1.21	1.04E-42
Tubb4a	178.59	-0.79	9.71E-04
Tubb5	13443.86	-0.17	1.54E-03
Aaas	927.32	-1.13	5.85E-20
Lemd2	773.16	-0.45	2.93E-04
Ppp2r5b	375.21	-0.76	7.88E-06

Nup214	3309.15	-0.23	1.41E-03
Kif18a	198.90	-1.40	3.14E-09
Cenpe	884.52	-0.90	1.14E-12
Haus6	437.55	-0.54	5.94E-04
H2afj	1326.92	-0.59	1.21E-08
Ercc6l	162.82	-1.12	5.07E-06
Smc1a	2539.64	-0.47	1.32E-09
Mcph1	787.11	-0.44	7.18E-04
Hist1h3f	1363.45	-0.77	2.82E-15
Mapk3	2594.23	-0.53	3.01E-10
Cenph	117.24	-0.81	6.60E-03
Cntrl	1734.50	-0.69	1.05E-13
Ppp2r5c	2652.41	-0.81	1.01E-25
Zw10	1016.94	-0.64	6.45E-09
Csnk1e	1367.78	-1.30	1.53E-34
Hist1h3c	1565.78	-1.06	1.94E-29
Hist1h3d	5161.95	-1.15	7.13E-67
Hist1h3b	2087.56	-1.17	3.98E-40
Hist1h3e	1469.33	-0.80	2.15E-17
Hist1h3h	2193.34	-1.30	1.93E-52
Hist1h3i	2266.74	-1.17	2.12E-43
Hist2h3b	2719.24	-1.26	1.53E-44
Hist1h4d	2684.89	-0.22	4.83E-03
Hist1h4f	1522.67	-0.94	2.25E-20
Hist1h4i	829.50	-0.61	3.24E-07
Hist1h4j	1441.05	-0.92	2.37E-21
Hist1h4k	1905.91	-0.78	1.36E-18
Hist1h4n	2367.62	-0.41	3.73E-07
Hist1h2ac	2453.61	-1.01	4.40E-38
Hist1h2ae	2624.68	-1.06	1.03E-41
Hist1h2ag	2722.66	-1.17	1.60E-51
Hist1h2an	1621.93	-1.22	1.30E-38
Hist1h2ab	3440.49	-1.14	1.02E-49
Hist2h2ac	1890.34	-0.54	1.78E-10
Hist1h2ba	229.34	-0.80	1.13E-03
Hist1h2bb	2089.34	-0.75	1.92E-19
Hist1h2be	2122.33	-0.28	4.48E-03
Hist1h2bg	2604.45	-0.34	5.84E-05
Hist1h2bk	2107.81	-0.67	6.47E-13
Hist1h2bm	887.41	-0.54	3.26E-06
Hist2h2be	130.69	-2.23	1.66E-14
Hist1h2ai	2965.60	-1.07	1.44E-44
Hist4h4	663.03	-0.67	4.23E-07

Hist1h4a	2002.28	-0.71	4.53E-17
Hist1h4b	1730.88	-0.68	7.49E-15
Tubgcp6	907.13	-1.01	2.46E-18
Hist1h3a	890.74	-1.15	1.28E-20
Nsl1	175.84	-1.17	1.22E-06
Nup85	821.33	-0.51	1.72E-05
Cdca8	481.29	-1.23	8.32E-16
Cenpo	321.36	-0.41	2.51E-02
Ncaph2	1448.25	-0.52	4.13E-08
Actr1a	2965.34	-0.21	5.18E-03
Ncapg	445.19	-0.80	1.05E-06
Nup210	1246.12	-1.01	1.46E-23
Mad2l1	288.01	-0.87	4.13E-06
Clip1	2941.90	-1.09	1.35E-49
Anapc5	2992.22	-0.27	5.91E-04
Nek7	1495.37	-0.35	2.06E-04
Nek6	957.31	-0.63	5.68E-08
Cenpk	78.70	-1.13	3.27E-03
Lpin2	952.86	-0.98	7.21E-17
Ska2	120.26	-0.76	9.16E-03
Anapc11	613.95	-0.45	7.85E-04
Haus2	541.62	-0.47	4.17E-03
Spc25	204.77	-1.18	1.66E-07
Ska1	58.94	-2.28	2.65E-08
Hist1h2ao	6653.04	-0.98	1.92E-56
Cenpm	100.40	-1.18	2.45E-04
Dsn1	170.29	-0.71	4.88E-03
Nuf2	324.28	-0.75	2.74E-05
Pmf1	425.82	-0.59	7.42E-04
Ndc80	211.34	-1.32	7.69E-09
Mastl	158.83	-1.98	8.78E-13
Fbxo5	140.31	-1.65	2.28E-09
Nde1	918.83	-0.83	4.27E-13
Cdca5	195.79	-2.18	8.04E-19
Zwilch	192.21	-0.95	6.32E-05
Dynll2	992.76	-0.65	3.80E-09
Ncapd2	1291.48	-1.42	3.20E-44
Sgo2a	227.33	-0.63	4.28E-03
Hist1h4h	2448.10	-0.75	9.01E-22
Dctn2	2085.49	-0.38	3.62E-06
Cenps	37.43	-1.27	5.42E-03
Smc4	1485.96	-0.59	9.21E-08
Hdac8	166.97	-0.85	5.20E-04

Spdl1	386.58	-1.65	1.14E-21
Cep192	1433.98	-0.88	3.38E-17
Nup93	1686.90	-0.48	6.18E-07
Kif23	653.32	-1.20	3.71E-19
Cenpu	60.33	-1.49	1.31E-04
Haus5	140.71	-1.26	2.50E-06
Mzt2	164.76	-0.88	3.87E-04
Cenpn	183.78	-1.60	2.93E-11
Sgo1	159.07	-1.20	2.93E-06
Kif2c	174.63	-1.31	4.30E-07
Cep57	510.32	-0.59	5.21E-05
Cep72	43.16	-1.96	1.19E-05
Gorasp1	314.16	-0.54	3.53E-03
Ckap5	2860.41	-0.31	2.81E-04
Ncapg2	875.62	-0.96	4.66E-16
Haus8	295.01	-1.49	6.06E-15
Clasp2	1657.85	-0.31	4.17E-04
Sdccag8	398.55	-1.31	5.36E-15
H2afv	213.26	-1.00	5.19E-06
Ninl	218.49	-1.32	1.01E-09
Ncapd3	898.26	-1.30	3.90E-29
Sfi1	5681.69	-0.26	4.11E-05
Hist2h3c2	5169.81	-0.99	3.37E-43
Hist2h4	873.72	-1.00	8.48E-17
Hist1h3g	963.61	-1.41	1.92E-30
Anapc2	1840.61	-0.37	7.70E-05

Mitotic prometaphase

gene_name	baseMean	log2FoldChange	p-value
Pds5b	1250.00	-0.45	2.03E-05
Akap9	4914.28	-0.49	1.16E-11
Ccp110	479.85	-0.56	1.80E-04
Numa1	5094.99	-1.36	4.96E-91
Cenpi	110.18	-1.02	2.08E-03
Xpo1	3698.37	-0.18	1.01E-02
Tubg1	527.80	-0.97	1.67E-11
Tubg2	52.07	-1.35	8.59E-04
Cenpf	896.17	-1.33	2.39E-27
Rcc2	10642.06	-0.45	7.59E-13
Birc5	276.15	-1.30	1.05E-10
Cep131	235.70	-2.19	5.37E-21
Bub1	467.77	-0.89	1.57E-08
Bub1b	1040.27	-1.26	8.58E-29
Cenpa	319.25	-0.47	7.85E-03

Smc3	2571.96	-0.42	1.27E-07
Dctn1	2732.14	-0.54	1.53E-11
Dync1h1	26338.96	-0.42	1.02E-12
Smc2	1355.94	-0.43	9.94E-06
Incenp	2833.13	-0.92	4.05E-35
Cep250	2577.38	-0.27	1.29E-03
Mad1l1	587.01	-0.88	1.79E-10
Nme7	299.86	-0.43	2.03E-02
Nek2	343.20	-1.19	9.28E-09
Nudc	1497.61	-0.34	1.29E-03
Odf2	1301.20	-0.31	1.97E-03
Pcnt	1842.88	-0.85	2.07E-22
Prkaca	1266.36	-0.54	3.03E-06
Plk1	363.52	-1.06	6.04E-09
Rangap1	1965.01	-0.73	4.17E-15
Cep78	397.50	-1.47	7.23E-18
Kntc1	578.93	-1.16	7.72E-16
Aurkb	400.41	-1.55	3.98E-20
Cdk5rap2	1615.96	-0.41	3.86E-06
Cep164	1056.36	-1.36	4.21E-33
Ncaph	448.17	-1.68	2.94E-22
Ppp2r5d	1023.84	-0.69	6.20E-10
Haus4	157.56	-2.39	4.32E-17
Tubb4a	178.59	-0.79	9.71E-04
Tubb5	13443.86	-0.17	1.54E-03
Ppp2r5b	375.21	-0.76	7.88E-06
Kif18a	198.90	-1.40	3.14E-09
Cenpe	884.52	-0.90	1.14E-12
Haus6	437.55	-0.54	5.94E-04
Ercc6l	162.82	-1.12	5.07E-06
Smc1a	2539.64	-0.47	1.32E-09
Cenph	117.24	-0.81	6.60E-03
Cntrl	1734.50	-0.69	1.05E-13
Ppp2r5c	2652.41	-0.81	1.01E-25
Zw10	1016.94	-0.64	6.45E-09
Csnk1e	1367.78	-1.30	1.53E-34
Tubgcp6	907.13	-1.01	2.46E-18
Nsl1	175.84	-1.17	1.22E-06
Nup85	821.33	-0.51	1.72E-05
Cdca8	481.29	-1.23	8.32E-16
Cenpo	321.36	-0.41	2.51E-02
Actr1a	2965.34	-0.21	5.18E-03
Ncapg	445.19	-0.80	1.05E-06

Mad2l1	288.01	-0.87	4.13E-06
Clip1	2941.90	-1.09	1.35E-49
Cenpk	78.70	-1.13	3.27E-03
Ska2	120.26	-0.76	9.16E-03
Haus2	541.62	-0.47	4.17E-03
Spc25	204.77	-1.18	1.66E-07
Ska1	58.94	-2.28	2.65E-08
Cenpm	100.40	-1.18	2.45E-04
Dsn1	170.29	-0.71	4.88E-03
Nuf2	324.28	-0.75	2.74E-05
Pmf1	425.82	-0.59	7.42E-04
Ndc80	211.34	-1.32	7.69E-09
Nde1	918.83	-0.83	4.27E-13
Cdca5	195.79	-2.18	8.04E-19
Zwilch	192.21	-0.95	6.32E-05
Dynll2	992.76	-0.65	3.80E-09
Ncapd2	1291.48	-1.42	3.20E-44
Sgo2a	227.33	-0.63	4.28E-03
Dctn2	2085.49	-0.38	3.62E-06
Cenps	37.43	-1.27	5.42E-03
Smc4	1485.96	-0.59	9.21E-08
Hdac8	166.97	-0.85	5.20E-04
Spdl1	386.58	-1.65	1.14E-21
Cep192	1433.98	-0.88	3.38E-17
Cenpu	60.33	-1.49	1.31E-04
Haus5	140.71	-1.26	2.50E-06
Mzt2	164.76	-0.88	3.87E-04
Cenpn	183.78	-1.60	2.93E-11
Sgo1	159.07	-1.20	2.93E-06
Kif2c	174.63	-1.31	4.30E-07
Cep57	510.32	-0.59	5.21E-05
Cep72	43.16	-1.96	1.19E-05
Ckap5	2860.41	-0.31	2.81E-04
Haus8	295.01	-1.49	6.06E-15
Clasp2	1657.85	-0.31	4.17E-04
Sdccag8	398.55	-1.31	5.36E-15
Ninl	218.49	-1.32	1.01E-09
Sfi1	5681.69	-0.26	4.11E-05

Down-regulated genes following 6 h *P. aeruginosa* infection

HDACs deacetylate histones

gene_name	baseMean	log2FoldChange	p-value
Chd4	12992.05	-0.59	3.30E-18
Mta1	2055.43	-0.46	3.15E-06

Mta3	595.07	-0.75	3.70E-07
Hmg20b	748.47	-1.45	3.43E-23
Hdac10	268.28	-1.13	5.09E-08
Phf21a	740.31	-1.16	8.12E-18
Chd3	2373.44	-1.11	3.10E-38
Gatad2b	1717.16	-0.68	5.63E-13
Arid4a	1176.70	-1.23	3.79E-28
Hist1h3c	1693.67	-0.31	8.60E-04
Hist1h3d	5403.83	-0.49	3.38E-11
Hist1h3b	2233.70	-0.43	6.21E-06
Hist1h3h	2424.17	-0.41	5.02E-06
Hist1h3i	2512.38	-0.31	7.92E-04
Hist2h3b	2811.02	-0.62	2.36E-12
Hist1h4f	1543.29	-0.44	1.42E-04
Hist1h4j	1477.67	-0.39	1.50E-04
Hist1h2ac	2598.46	-0.35	2.88E-05
Hist1h2ae	2711.63	-0.47	1.99E-08
Hist1h2ag	2783.21	-0.59	7.82E-13
Hist1h2an	1678.41	-0.58	2.03E-09
Hist1h2ab	3493.11	-0.59	6.17E-13
Hist1h2bb	2057.12	-0.38	2.36E-05
Hist1h2bk	2094.33	-0.28	3.22E-03
Hist2h2be	115.74	-2.26	7.65E-12
Hist1h2ai	3058.64	-0.48	1.98E-09
Hist1h3a	946.46	-0.45	2.35E-04
Sap30l	172.00	-0.97	3.04E-04
Hist2h2ab	219.14	-0.63	1.00E-02
Hist1h2ao	6618.69	-0.54	6.28E-16
Hist1h4h	2419.90	-0.36	1.40E-05
Hdac8	152.64	-0.74	5.72E-03
Suds3	1050.18	-0.78	1.42E-11
Tbl1xr1	1347.30	-1.08	1.45E-24
Arid4b	1182.53	-0.30	2.31E-02
Hist2h3c2	5455.55	-0.35	3.37E-06
Hist1h3g	1066.67	-0.49	2.10E-05

Cell cycle

gene_name	baseMean	log2FoldChange	p-value
Pds5b	1062.67	-0.60	4.86E-07
Akap9	4543.13	-0.35	1.01E-04
Numa1	4544.60	-1.33	5.99E-70
Cenpi	99.13	-0.98	2.51E-03
Xpo1	2809.89	-0.71	5.99E-17
Tubg1	499.07	-0.72	3.22E-06

Espl1	548.80	-2.19	1.37E-42
Rfc4	205.84	-1.36	7.81E-09
Nsd2	1239.69	-0.79	1.98E-13
Pom121	2049.09	-0.21	1.91E-02
Cenpf	806.53	-1.26	4.28E-18
Rcc2	9296.26	-0.50	1.99E-13
Gins4	289.23	-1.40	3.64E-11
Abl1	1398.58	-1.04	1.42E-22
Birc5	243.39	-1.34	2.50E-09
Atm	2252.71	-0.62	3.85E-12
Cep131	212.51	-2.06	2.76E-15
Bard1	186.15	-1.78	5.58E-11
Blm	474.27	-1.28	9.91E-15
Brca1	434.67	-2.32	1.84E-35
Btrc	362.31	-0.92	3.84E-07
Bub1	448.05	-0.59	3.94E-04
Bub1b	1044.14	-0.73	1.32E-10
Ccna2	411.45	-1.18	1.26E-11
Ccnb2	243.50	-1.20	4.39E-08
Ccnd1	4927.97	-1.42	8.62E-89
Ccnd3	658.97	-1.78	1.64E-35
Ccne1	150.09	-2.10	1.91E-13
Ccne2	87.24	-1.41	5.03E-05
Cdc25a	504.92	-0.53	7.44E-04
Cdc25b	684.37	-1.54	4.13E-25
Cdc7	154.41	-0.87	1.68E-03
Cdk2	409.01	-1.44	6.91E-15
Cdk4	3409.11	-0.20	9.46E-03
Cdkn1b	464.92	-1.23	9.79E-14
Cdkn2c	126.13	-2.57	1.63E-15
Cdkn2d	369.42	-1.03	8.41E-09
Cenpa	249.75	-0.94	7.85E-06
Smc3	2240.69	-0.48	5.32E-07
Dctn1	2434.36	-0.52	9.31E-08
E2f1	280.38	-1.71	1.78E-15
Fen1	1058.45	-0.79	4.58E-12
Smc2	1230.27	-0.36	8.36E-04
Foxm1	688.41	-1.69	5.99E-33
Lpin1	514.81	-2.41	4.02E-46
Herc2	7281.01	-0.53	2.56E-13
Hmmr	375.21	-1.26	1.14E-11
Incenp	2669.79	-0.68	4.78E-16
Lig1	843.69	-1.90	5.97E-47

Lmnb1	1011.19	-0.65	1.66E-08
Nup155	1875.43	-0.42	4.09E-06
Mad1l1	514.60	-0.93	1.57E-09
Mcm3	1529.60	-0.81	2.73E-16
Mcm2	1218.11	-1.83	2.70E-59
Mcm4	973.76	-0.92	1.04E-14
Mcm5	1443.48	-1.20	2.63E-27
Mcm6	1347.69	-1.95	1.72E-71
Mcm7	682.19	-1.81	2.19E-37
Mre11a	692.93	-0.50	2.29E-04
Nek2	309.22	-1.12	8.50E-08
Odf2	1114.99	-0.43	9.24E-04
Pcnt	1669.09	-0.77	1.70E-13
Prkaca	1045.87	-0.81	1.52E-09
Plk1	341.13	-0.83	7.69E-06
Pola1	637.29	-1.20	5.48E-17
Pold1	770.62	-1.98	7.88E-48
Pole	633.63	-2.21	2.75E-49
Pole2	123.30	-1.01	8.32E-04
Prim1	180.86	-2.37	7.21E-18
Prim2	298.42	-1.14	6.40E-09
Kif20a	369.85	-0.56	1.46E-03
Rad50	1286.16	-0.57	3.32E-07
Rangap1	1883.69	-0.45	9.36E-07
Rb1	669.96	-1.95	2.99E-31
Rbl1	715.39	-1.12	1.41E-16
Rbl2	838.79	-1.69	7.49E-38
Rfc1	1418.08	-0.95	2.05E-18
Rfc2	429.81	-1.28	4.42E-14
Rpa2	267.25	-1.42	1.02E-10
Cep78	352.17	-1.47	6.30E-15
Kntc1	505.54	-1.23	4.65E-15
Aurkb	326.09	-2.10	4.89E-26
Aurka	308.88	-0.50	2.18E-02
Tfdp2	602.58	-2.37	3.05E-50
Cdk5rap2	1429.45	-0.42	1.13E-04
Cep164	889.63	-1.64	7.13E-37
Ncaph	396.92	-1.68	2.10E-17
Terf1	330.39	-0.48	1.06E-02
Tert	31.38	-2.01	2.26E-04
Mis18bp1	401.02	-1.27	6.38E-13
Ppp2r5d	858.57	-0.91	7.80E-13
Nek9	2821.86	-0.51	1.17E-09

Tfdp1	1232.59	-1.02	2.68E-20
Haus4	133.53	-2.88	1.62E-17
Tmpo	1849.13	-1.36	2.29E-38
Tubb5	11692.54	-0.23	2.19E-04
Vrk1	611.15	-0.42	4.55E-03
Wee1	283.74	-1.89	1.83E-17
Aaas	776.39	-1.41	3.88E-26
Wrn	309.77	-1.07	3.05E-08
Ppp2r5b	365.12	-0.43	1.69E-02
Nup214	2804.45	-0.37	4.47E-06
Kif18a	159.49	-2.02	2.23E-13
Phf20	1623.79	-0.48	6.26E-07
Cdc14a	183.56	-1.34	5.55E-07
Cenpe	838.80	-0.64	8.27E-07
Haus6	385.78	-0.57	9.86E-04
Haus3	278.84	-0.60	4.37E-03
Lin54	587.51	-0.39	7.26E-03
Fbxl18	304.51	-1.44	3.97E-13
H2afj	1096.77	-0.86	3.67E-13
Tubgcp5	585.92	-0.39	7.23E-03
Nup133	447.74	-0.37	2.72E-02
Topbp1	2132.89	-1.39	4.76E-48
Ercc6l	161.12	-0.69	6.98E-03
Brip1	299.09	-0.75	1.28E-04
Akt3	1232.26	-0.86	6.61E-16
Cdc6	201.32	-1.55	1.34E-10
Mdc1	1804.62	-1.41	2.23E-39
Smc1a	2207.10	-0.54	3.86E-10
E2f2	410.95	-1.96	2.89E-27
Mcph1	718.54	-0.34	1.51E-02
Mapk3	2178.59	-0.73	1.28E-15
Cenph	84.50	-1.82	3.02E-07
Pkmyt1	181.72	-0.64	1.28E-02
Exo1	168.13	-1.78	4.84E-11
Cntrl	1539.62	-0.68	8.23E-10
Ppp2r5c	2449.23	-0.66	1.51E-14
Clspn	359.18	-2.88	1.02E-44
Gins2	81.52	-1.45	9.77E-05
Csnk1e	1187.48	-1.41	1.97E-34
Skp2	416.10	-1.31	4.62E-12
Zfp385a	805.09	-1.84	1.45E-42
Gtse1	768.55	-2.01	1.69E-48
Lcmt1	288.53	-0.82	6.20E-05

Hist1h3c	1693.67	-0.31	8.60E-04
Hist1h3d	5403.83	-0.49	3.38E-11
Hist1h3b	2233.70	-0.43	6.21E-06
Hist1h3h	2424.17	-0.41	5.02E-06
Hist1h3i	2512.38	-0.31	7.92E-04
Hist2h3b	2811.02	-0.62	2.36E-12
Hist1h4f	1543.29	-0.44	1.42E-04
Hist1h4j	1477.67	-0.39	1.50E-04
Hist1h2ac	2598.46	-0.35	2.88E-05
Hist1h2ae	2711.63	-0.47	1.99E-08
Hist1h2ag	2783.21	-0.59	7.82E-13
Hist1h2an	1678.41	-0.58	2.03E-09
Hist1h2ab	3493.11	-0.59	6.17E-13
Hist1h2bb	2057.12	-0.38	2.36E-05
Hist1h2bk	2094.33	-0.28	3.22E-03
Hist2h2be	115.74	-2.26	7.65E-12
Hist1h2ai	3058.64	-0.48	1.98E-09
Dna2	391.80	-2.25	3.25E-33
Tubgcp6	778.76	-1.16	2.45E-18
Ppp1r12b	319.06	-0.48	1.14E-02
Hist1h3a	946.46	-0.45	2.35E-04
Nsl1	152.77	-1.27	2.89E-06
Nup85	706.18	-0.62	1.19E-05
Acd	427.06	-0.57	7.04E-04
Chek2	208.41	-2.99	1.91E-27
Tubgcp4	661.14	-0.33	1.47E-02
Cdca8	462.85	-0.88	4.11E-08
Cenpo	278.42	-0.48	1.75E-02
Cdc23	532.09	-0.49	9.37E-04
Ncaph2	1215.37	-0.72	2.96E-10
Actr1a	2566.92	-0.28	7.12E-04
Ncapg	378.43	-0.98	1.27E-07
Nup210	1052.97	-1.23	1.41E-24
Sfn	40.75	-1.70	6.86E-04
Mad2l1	252.57	-0.91	1.46E-05
Fzr1	969.41	-0.48	5.82E-05
Clip1	2750.78	-0.86	2.36E-22
Gmnn	213.38	-2.24	2.07E-19
Rnf8	268.04	-0.54	8.58E-03
Anapc5	2441.09	-0.55	1.33E-10
Nek7	1183.56	-0.75	3.67E-12
Nek6	845.71	-0.64	2.91E-06
Cenpk	64.35	-1.58	1.28E-04

Lpin2	840.18	-1.00	5.27E-15
Anapc11	507.59	-0.70	5.40E-06
Cenpp	45.82	-1.13	1.19E-02
Spc25	172.15	-1.45	1.18E-08
Ska1	52.97	-2.18	2.04E-06
Hist1h2ao	6618.69	-0.54	6.28E-16
Cenpm	79.46	-1.80	2.57E-06
Mcm8	92.59	-0.99	2.86E-03
Dsn1	136.90	-1.11	2.14E-04
Nuf2	249.28	-1.38	2.95E-10
Pmf1	361.85	-0.74	9.98E-05
Ndc80	181.63	-1.49	2.69E-09
Mastl	147.63	-1.69	8.81E-09
Fbxo5	123.67	-1.70	3.00E-08
Cdt1	1749.09	-0.41	8.83E-06
Nde1	743.66	-1.22	4.88E-20
Spc24	101.42	-0.75	1.90E-02
Cdca5	180.97	-1.88	3.35E-12
Zwilch	151.30	-1.49	6.44E-07
Dynll2	845.90	-0.80	7.59E-10
Rpa1	909.44	-1.41	9.10E-30
Ncapd2	1140.31	-1.45	1.43E-34
Ssna1	244.96	-0.55	1.23E-02
Sgo2a	161.35	-1.61	4.42E-09
Gins1	88.93	-1.57	4.68E-06
Hist1h4h	2419.90	-0.36	1.40E-05
Cenps	30.16	-1.79	7.79E-04
Mcm10	377.03	-0.57	1.01E-03
Smc4	1394.29	-0.40	8.85E-04
Rnf168	442.81	-0.60	3.18E-04
Hdac8	152.64	-0.74	5.72E-03
Spdl1	380.25	-1.12	2.25E-10
Oip5	38.80	-1.82	1.89E-04
Cep192	1240.75	-0.98	2.30E-17
Ankle2	1177.66	-0.25	1.81E-02
Nup93	1595.75	-0.27	1.18E-02
Kif23	669.59	-0.61	9.56E-06
Cenpu	44.95	-2.76	1.03E-07
Haus5	110.53	-1.96	1.72E-09
Esco2	159.61	-2.56	2.81E-18
Mzt2	138.63	-1.10	8.96E-05
Tpx2	844.28	-0.34	7.13E-03
Cenpn	165.21	-1.53	3.99E-09

Sgo1	134.49	-1.45	3.22E-07
Rhno1	392.68	-0.40	1.97E-02
Pot1b	145.59	-1.36	6.18E-07
Psma8	250.65	-0.57	1.15E-02
Kif2c	160.23	-1.16	1.41E-05
Tubgcp2	607.43	-0.37	1.85E-02
Cep57	449.60	-0.62	2.77E-04
Cep72	43.22	-1.33	4.12E-03
Gorasp1	285.15	-0.46	2.72E-02
Mau2	1354.92	-0.33	3.67E-03
Lin37	145.24	-0.91	6.67E-04
Ckap5	2597.66	-0.23	1.09E-02
Ncapg2	792.22	-0.88	1.14E-11
Haus8	290.58	-0.99	8.79E-07
Clasp2	1416.31	-0.44	2.02E-05
Clasp1	1781.71	-0.35	5.61E-04
Sdccag8	349.94	-1.37	1.68E-13
H2afv	158.52	-1.91	6.35E-11
Bora	206.20	-1.46	7.58E-10
Ninl	166.59	-2.27	2.30E-16
Ncapd3	763.86	-1.52	1.61E-30
Gins3	307.42	-2.47	5.04E-31
Sfi1	5006.80	-0.28	4.11E-05
Hist2h3c2	5455.55	-0.35	3.37E-06
Hist1h3g	1066.67	-0.49	2.10E-05
Cep152	434.25	-0.76	2.07E-05
Anapc2	1479.82	-0.71	2.03E-12

Activated PKN stimulates transcription of AR regulated genes KLK2 and KLK3

gene_name	baseMean	log2FoldChange	p-value
Ncoa2	1396.56	-1.40	3.49E-39
H2afj	1096.77	-0.86	3.67E-13
Hist1h3c	1693.67	-0.31	8.60E-04
Hist1h3d	5403.83	-0.49	3.38E-11
Hist1h3b	2233.70	-0.43	6.21E-06
Hist1h3h	2424.17	-0.41	5.02E-06
Hist1h3i	2512.38	-0.31	7.92E-04
Hist2h3b	2811.02	-0.62	2.36E-12
Hist1h4f	1543.29	-0.44	1.42E-04
Hist1h4j	1477.67	-0.39	1.50E-04
Hist1h2ac	2598.46	-0.35	2.88E-05
Hist1h2ae	2711.63	-0.47	1.99E-08
Hist1h2ag	2783.21	-0.59	7.82E-13
Hist1h2an	1678.41	-0.58	2.03E-09

Hist1h2ab	3493.11	-0.59	6.17E-13
Hist1h2bb	2057.12	-0.38	2.36E-05
Hist1h2bk	2094.33	-0.28	3.22E-03
Hist2h2be	115.74	-2.26	7.65E-12
Hist1h2ai	3058.64	-0.48	1.98E-09
Pkn1	1857.70	-0.84	1.27E-18
Hist1h3a	946.46	-0.45	2.35E-04
Hist1h2ao	6618.69	-0.54	6.28E-16
Hist1h4h	2419.90	-0.36	1.40E-05
Kdm4c	495.81	-0.49	2.59E-03
H2afv	158.52	-1.91	6.35E-11
Hist2h3c2	5455.55	-0.35	3.37E-06
Hist1h3g	1066.67	-0.49	2.10E-05

Condensation of prophase chromosomes

gene_name	baseMean	log2FoldChange	p-value
Smc2	1230.27	-0.36	8.36E-04
Plk1	341.13	-0.83	7.69E-06
Rb1	669.96	-1.95	2.99E-31
H2afj	1096.77	-0.86	3.67E-13
Mcph1	718.54	-0.34	1.51E-02
Hist1h3c	1693.67	-0.31	8.60E-04
Hist1h3d	5403.83	-0.49	3.38E-11
Hist1h3b	2233.70	-0.43	6.21E-06
Hist1h3h	2424.17	-0.41	5.02E-06
Hist1h3i	2512.38	-0.31	7.92E-04
Hist2h3b	2811.02	-0.62	2.36E-12
Hist1h4f	1543.29	-0.44	1.42E-04
Hist1h4j	1477.67	-0.39	1.50E-04
Hist1h2ac	2598.46	-0.35	2.88E-05
Hist1h2ae	2711.63	-0.47	1.99E-08
Hist1h2ag	2783.21	-0.59	7.82E-13
Hist1h2an	1678.41	-0.58	2.03E-09
Hist1h2ab	3493.11	-0.59	6.17E-13
Hist1h2bb	2057.12	-0.38	2.36E-05
Hist1h2bk	2094.33	-0.28	3.22E-03
Hist2h2be	115.74	-2.26	7.65E-12
Hist1h2ai	3058.64	-0.48	1.98E-09
Hist1h3a	946.46	-0.45	2.35E-04
Ncaph2	1215.37	-0.72	2.96E-10
Hist1h2ao	6618.69	-0.54	6.28E-16
Hist1h4h	2419.90	-0.36	1.40E-05
Smc4	1394.29	-0.40	8.85E-04
Ncapg2	792.22	-0.88	1.14E-11

H2afv	158.52	-1.91	6.35E-11
Ncapd3	763.86	-1.52	1.61E-30
Hist2h3c2	5455.55	-0.35	3.37E-06
Hist1h3g	1066.67	-0.49	2.10E-05

Signaling by Rho GTPases

gene_name	baseMean	log2FoldChange	p-value
Arhgef18	355.86	-0.67	1.67E-04
Cenpi	99.13	-0.98	2.51E-03
Xpo1	2809.89	-0.71	5.99E-17
Cenpf	806.53	-1.26	4.28E-18
Baiap2	711.81	-1.37	6.04E-24
Rcc2	9296.26	-0.50	1.99E-13
Abl1	1398.58	-1.04	1.42E-22
Birc5	243.39	-1.34	2.50E-09
Rhod	106.90	-3.15	1.15E-16
Arhgap5	1673.44	-0.38	2.47E-04
Arhgdib	4799.43	-0.76	7.42E-25
Bub1	448.05	-0.59	3.94E-04
Bub1b	1044.14	-0.73	1.32E-10
Calm3	2874.78	-0.30	5.60E-03
Cenpa	249.75	-0.94	7.85E-06
Cit	370.92	-1.89	1.83E-24
Dlg4	166.25	-0.87	1.04E-03
Ect2	312.86	-0.87	3.37E-05
Incenp	2669.79	-0.68	4.78E-16
Itsn1	1248.19	-0.33	2.63E-03
Kif5a	866.41	-2.67	4.91E-61
Klc1	2175.62	-0.87	3.34E-22
Limk1	143.64	-1.57	5.19E-07
Rac3	36.40	-1.15	2.57E-02
Mad1l1	514.60	-0.93	1.57E-09
Mcf2l	743.25	-0.94	3.84E-11
Men1	999.77	-0.59	4.82E-07
Ncoa2	1396.56	-1.40	3.49E-39
Nf2	2556.63	-0.97	4.30E-28
Pak1	740.84	-1.68	6.29E-31
Pfn2	119.85	-0.75	1.01E-02
Pik3r2	1042.94	-1.90	1.00E-56
Plk1	341.13	-0.83	7.69E-06
Rac2	7161.69	-0.38	1.11E-07
Rangap1	1883.69	-0.45	9.36E-07
Ralbp1	1504.56	-0.83	1.61E-16
Rtkn	76.70	-2.28	4.36E-09

Sos1	1034.88	-0.50	1.22E-05
Kntc1	505.54	-1.23	4.65E-15
Aurkb	326.09	-2.10	4.89E-26
Arhgef11	1853.96	-0.74	1.56E-15
Rhot2	779.39	-0.47	2.42E-04
Wipf1	2826.26	-0.41	3.72E-07
Arhgap9	868.42	-0.62	6.10E-07
Ppp2r5d	858.57	-0.91	7.80E-13
Vav1	2902.95	-0.20	1.46E-02
Vav2	159.38	-0.67	1.19E-02
Arhgap39	655.88	-1.31	6.11E-17
Mkl1	2043.07	-0.94	1.88E-23
Fgd4	1579.77	-0.80	1.66E-13
Pik3c3	793.17	-0.92	1.62E-11
Ppp2r5b	365.12	-0.43	1.69E-02
Arhgef4	170.45	-1.25	8.41E-07
Kif18a	159.49	-2.02	2.23E-13
Cenpe	838.80	-0.64	8.27E-07
Arhgef39	65.35	-1.52	1.75E-04
Arhgap24	177.12	-2.83	3.99E-23
H2afj	1096.77	-0.86	3.67E-13
Arhgap35	1742.78	-0.26	1.58E-02
Arhgap33	46.60	-3.51	1.54E-09
Prc1	796.24	-0.82	1.82E-10
Arhgef10	495.05	-0.71	5.15E-06
Nup133	447.74	-0.37	2.72E-02
Ercc6l	161.12	-0.69	6.98E-03
Arhgap22	367.15	-1.64	1.22E-17
Rhobtb2	288.45	-0.96	5.14E-06
Srgap3	520.17	-1.94	4.06E-33
Fgd2	139.80	-3.40	1.43E-22
Mapk3	2178.59	-0.73	1.28E-15
Arhgef40	462.03	-3.07	7.03E-48
Cenph	84.50	-1.82	3.02E-07
Ppp2r5c	2449.23	-0.66	1.51E-14
Racgap1	659.20	-1.64	2.03E-29
Plekhg5	149.00	-2.83	1.13E-05
Prex1	1841.37	-0.94	3.57E-19
Fgd3	1437.36	-1.60	3.41E-39
Hist1h3c	1693.67	-0.31	8.60E-04
Hist1h3d	5403.83	-0.49	3.38E-11
Hist1h3b	2233.70	-0.43	6.21E-06
Hist1h3h	2424.17	-0.41	5.02E-06

Hist1h3i	2512.38	-0.31	7.92E-04
Hist2h3b	2811.02	-0.62	2.36E-12
Hist1h4f	1543.29	-0.44	1.42E-04
Hist1h4j	1477.67	-0.39	1.50E-04
Hist1h2ac	2598.46	-0.35	2.88E-05
Hist1h2ae	2711.63	-0.47	1.99E-08
Hist1h2ag	2783.21	-0.59	7.82E-13
Hist1h2an	1678.41	-0.58	2.03E-09
Hist1h2ab	3493.11	-0.59	6.17E-13
Hist1h2bb	2057.12	-0.38	2.36E-05
Hist1h2bk	2094.33	-0.28	3.22E-03
Hist2h2be	115.74	-2.26	7.65E-12
Hist1h2ai	3058.64	-0.48	1.98E-09
Scai	203.42	-0.89	1.69E-04
Pkn1	1857.70	-0.84	1.27E-18
Ppp1r12b	319.06	-0.48	1.14E-02
Hist1h3a	946.46	-0.45	2.35E-04
Obscn	40.41	-1.18	1.22E-02
Kif14	293.92	-1.56	2.21E-14
Nsl1	152.77	-1.27	2.89E-06
Iqgap3	405.46	-2.53	7.95E-37
Nup85	706.18	-0.62	1.19E-05
Cdca8	462.85	-0.88	4.11E-08
Rhpn2	9.36	-1.76	1.40E-02
Cenpo	278.42	-0.48	1.75E-02
Arhgap27	723.51	-1.18	2.68E-14
Sfn	40.75	-1.70	6.86E-04
Mad2l1	252.57	-0.91	1.46E-05
Clip1	2750.78	-0.86	2.36E-22
Vav3	614.88	-1.29	3.82E-19
Cenpk	64.35	-1.58	1.28E-04
Cenpp	45.82	-1.13	1.19E-02
Spc25	172.15	-1.45	1.18E-08
Ska1	52.97	-2.18	2.04E-06
Hist1h2ao	6618.69	-0.54	6.28E-16
Cenpm	79.46	-1.80	2.57E-06
Dsn1	136.90	-1.11	2.14E-04
Nuf2	249.28	-1.38	2.95E-10
Pmf1	361.85	-0.74	9.98E-05
Ndc80	181.63	-1.49	2.69E-09
Nde1	743.66	-1.22	4.88E-20
Spc24	101.42	-0.75	1.90E-02
Myl12b	2150.75	-0.54	1.30E-08

Zwilch	151.30	-1.49	6.44E-07
Dynll2	845.90	-0.80	7.59E-10
Sgo2a	161.35	-1.61	4.42E-09
Hist1h4h	2419.90	-0.36	1.40E-05
Cenps	30.16	-1.79	7.79E-04
Spdl1	380.25	-1.12	2.25E-10
Arhgap17	2095.71	-0.46	1.73E-07
Arhgap45	4746.15	-1.73	7.52E-115
Arhgap19	41.44	-1.82	1.51E-04
Arhgap26	144.90	-1.52	1.28E-07
Arhgap21	1737.21	-0.26	1.00E-02
Cenpu	44.95	-2.76	1.03E-07
Cenpn	165.21	-1.53	3.99E-09
Sgo1	134.49	-1.45	3.22E-07
Arhgef10l	1988.61	-0.62	4.20E-11
Arhgef6	3325.98	-1.50	9.31E-83
Kif2c	160.23	-1.16	1.41E-05
Arhgap18	332.71	-3.02	1.94E-42
Klc4	694.51	-2.57	6.62E-57
Arhgap12	740.87	-0.46	5.34E-04
Ckap5	2597.66	-0.23	1.09E-02
Arhgap15	145.85	-2.50	5.51E-16
Clasp2	1416.31	-0.44	2.02E-05
Clasp1	1781.71	-0.35	5.61E-04
Kdm4c	495.81	-0.49	2.59E-03
Cyfip2	133.40	-1.43	1.61E-06
H2afv	158.52	-1.91	6.35E-11
Hist2h3c2	5455.55	-0.35	3.37E-06
Hist1h3g	1066.67	-0.49	2.10E-05

Cell cycle, mitotic

gene_name	baseMean	log2FoldChange	p-value
Pds5b	1062.67	-0.60	4.86E-07
Akap9	4543.13	-0.35	1.01E-04
Numa1	4544.60	-1.33	5.99E-70
Cenpi	99.13	-0.98	2.51E-03
Xpo1	2809.89	-0.71	5.99E-17
Tubg1	499.07	-0.72	3.22E-06
Espl1	548.80	-2.19	1.37E-42
Rfc4	205.84	-1.36	7.81E-09
Pom121	2049.09	-0.21	1.91E-02
Cenpf	806.53	-1.26	4.28E-18
Rcc2	9296.26	-0.50	1.99E-13
Gins4	289.23	-1.40	3.64E-11

Abl1	1398.58	-1.04	1.42E-22
Birc5	243.39	-1.34	2.50E-09
Cep131	212.51	-2.06	2.76E-15
Btrc	362.31	-0.92	3.84E-07
Bub1	448.05	-0.59	3.94E-04
Bub1b	1044.14	-0.73	1.32E-10
Ccna2	411.45	-1.18	1.26E-11
Ccnb2	243.50	-1.20	4.39E-08
Ccnd1	4927.97	-1.42	8.62E-89
Ccnd3	658.97	-1.78	1.64E-35
Ccne1	150.09	-2.10	1.91E-13
Ccne2	87.24	-1.41	5.03E-05
Cdc25a	504.92	-0.53	7.44E-04
Cdc25b	684.37	-1.54	4.13E-25
Cdc7	154.41	-0.87	1.68E-03
Cdk2	409.01	-1.44	6.91E-15
Cdk4	3409.11	-0.20	9.46E-03
Cdkn1b	464.92	-1.23	9.79E-14
Cdkn2c	126.13	-2.57	1.63E-15
Cdkn2d	369.42	-1.03	8.41E-09
Cenpa	249.75	-0.94	7.85E-06
Smc3	2240.69	-0.48	5.32E-07
Dctn1	2434.36	-0.52	9.31E-08
E2f1	280.38	-1.71	1.78E-15
Fen1	1058.45	-0.79	4.58E-12
Smc2	1230.27	-0.36	8.36E-04
Foxm1	688.41	-1.69	5.99E-33
Lpin1	514.81	-2.41	4.02E-46
Hmmr	375.21	-1.26	1.14E-11
Incenp	2669.79	-0.68	4.78E-16
Lig1	843.69	-1.90	5.97E-47
Lmnb1	1011.19	-0.65	1.66E-08
Nup155	1875.43	-0.42	4.09E-06
Mad1l1	514.60	-0.93	1.57E-09
Mcm3	1529.60	-0.81	2.73E-16
Mcm2	1218.11	-1.83	2.70E-59
Mcm4	973.76	-0.92	1.04E-14
Mcm5	1443.48	-1.20	2.63E-27
Mcm6	1347.69	-1.95	1.72E-71
Mcm7	682.19	-1.81	2.19E-37
Nek2	309.22	-1.12	8.50E-08
Odf2	1114.99	-0.43	9.24E-04
Pcnt	1669.09	-0.77	1.70E-13

Prkaca	1045.87	-0.81	1.52E-09
Plk1	341.13	-0.83	7.69E-06
Pola1	637.29	-1.20	5.48E-17
Pold1	770.62	-1.98	7.88E-48
Pole	633.63	-2.21	2.75E-49
Pole2	123.30	-1.01	8.32E-04
Prim1	180.86	-2.37	7.21E-18
Prim2	298.42	-1.14	6.40E-09
Kif20a	369.85	-0.56	1.46E-03
Rangap1	1883.69	-0.45	9.36E-07
Rb1	669.96	-1.95	2.99E-31
Rbl1	715.39	-1.12	1.41E-16
Rbl2	838.79	-1.69	7.49E-38
Rfc1	1418.08	-0.95	2.05E-18
Rfc2	429.81	-1.28	4.42E-14
Rpa2	267.25	-1.42	1.02E-10
Cep78	352.17	-1.47	6.30E-15
Kntc1	505.54	-1.23	4.65E-15
Aurkb	326.09	-2.10	4.89E-26
Aurka	308.88	-0.50	2.18E-02
Tfdp2	602.58	-2.37	3.05E-50
Cdk5rap2	1429.45	-0.42	1.13E-04
Cep164	889.63	-1.64	7.13E-37
Ncaph	396.92	-1.68	2.10E-17
Ppp2r5d	858.57	-0.91	7.80E-13
Nek9	2821.86	-0.51	1.17E-09
Tfdp1	1232.59	-1.02	2.68E-20
Haus4	133.53	-2.88	1.62E-17
Tmpo	1849.13	-1.36	2.29E-38
Tubb5	11692.54	-0.23	2.19E-04
Vrk1	611.15	-0.42	4.55E-03
Wee1	283.74	-1.89	1.83E-17
Aaas	776.39	-1.41	3.88E-26
Ppp2r5b	365.12	-0.43	1.69E-02
Nup214	2804.45	-0.37	4.47E-06
Kif18a	159.49	-2.02	2.23E-13
Cdc14a	183.56	-1.34	5.55E-07
Cenpe	838.80	-0.64	8.27E-07
Haus6	385.78	-0.57	9.86E-04
Haus3	278.84	-0.60	4.37E-03
Lin54	587.51	-0.39	7.26E-03
Fbxl18	304.51	-1.44	3.97E-13
H2afj	1096.77	-0.86	3.67E-13

Tubgcp5	585.92	-0.39	7.23E-03
Nup133	447.74	-0.37	2.72E-02
Ercc6l	161.12	-0.69	6.98E-03
Akt3	1232.26	-0.86	6.61E-16
Cdc6	201.32	-1.55	1.34E-10
Smc1a	2207.10	-0.54	3.86E-10
E2f2	410.95	-1.96	2.89E-27
Mcph1	718.54	-0.34	1.51E-02
Mapk3	2178.59	-0.73	1.28E-15
Cenph	84.50	-1.82	3.02E-07
Pkmyt1	181.72	-0.64	1.28E-02
Cntrl	1539.62	-0.68	8.23E-10
Ppp2r5c	2449.23	-0.66	1.51E-14
Gins2	81.52	-1.45	9.77E-05
Csnk1e	1187.48	-1.41	1.97E-34
Skp2	416.10	-1.31	4.62E-12
Gtse1	768.55	-2.01	1.69E-48
Lcmt1	288.53	-0.82	6.20E-05
Hist1h3c	1693.67	-0.31	8.60E-04
Hist1h3d	5403.83	-0.49	3.38E-11
Hist1h3b	2233.70	-0.43	6.21E-06
Hist1h3h	2424.17	-0.41	5.02E-06
Hist1h3i	2512.38	-0.31	7.92E-04
Hist2h3b	2811.02	-0.62	2.36E-12
Hist1h4f	1543.29	-0.44	1.42E-04
Hist1h4j	1477.67	-0.39	1.50E-04
Hist1h2ac	2598.46	-0.35	2.88E-05
Hist1h2ae	2711.63	-0.47	1.99E-08
Hist1h2ag	2783.21	-0.59	7.82E-13
Hist1h2an	1678.41	-0.58	2.03E-09
Hist1h2ab	3493.11	-0.59	6.17E-13
Hist1h2bb	2057.12	-0.38	2.36E-05
Hist1h2bk	2094.33	-0.28	3.22E-03
Hist2h2be	115.74	-2.26	7.65E-12
Hist1h2ai	3058.64	-0.48	1.98E-09
Dna2	391.80	-2.25	3.25E-33
Tubgcp6	778.76	-1.16	2.45E-18
Ppp1r12b	319.06	-0.48	1.14E-02
Hist1h3a	946.46	-0.45	2.35E-04
Nsl1	152.77	-1.27	2.89E-06
Nup85	706.18	-0.62	1.19E-05
Tubgcp4	661.14	-0.33	1.47E-02
Cdca8	462.85	-0.88	4.11E-08

Cenpo	278.42	-0.48	1.75E-02
Cdc23	532.09	-0.49	9.37E-04
Ncaph2	1215.37	-0.72	2.96E-10
Actr1a	2566.92	-0.28	7.12E-04
Ncapg	378.43	-0.98	1.27E-07
Nup210	1052.97	-1.23	1.41E-24
Mad2l1	252.57	-0.91	1.46E-05
Fzr1	969.41	-0.48	5.82E-05
Clip1	2750.78	-0.86	2.36E-22
Gmnn	213.38	-2.24	2.07E-19
Anapc5	2441.09	-0.55	1.33E-10
Nek7	1183.56	-0.75	3.67E-12
Nek6	845.71	-0.64	2.91E-06
Cenpk	64.35	-1.58	1.28E-04
Lpin2	840.18	-1.00	5.27E-15
Anapc11	507.59	-0.70	5.40E-06
Cenpp	45.82	-1.13	1.19E-02
Spc25	172.15	-1.45	1.18E-08
Ska1	52.97	-2.18	2.04E-06
Hist1h2ao	6618.69	-0.54	6.28E-16
Cenpm	79.46	-1.80	2.57E-06
Mcm8	92.59	-0.99	2.86E-03
Dsn1	136.90	-1.11	2.14E-04
Nuf2	249.28	-1.38	2.95E-10
Pmf1	361.85	-0.74	9.98E-05
Ndc80	181.63	-1.49	2.69E-09
Mastl	147.63	-1.69	8.81E-09
Fbxo5	123.67	-1.70	3.00E-08
Cdt1	1749.09	-0.41	8.83E-06
Nde1	743.66	-1.22	4.88E-20
Spc24	101.42	-0.75	1.90E-02
Cdca5	180.97	-1.88	3.35E-12
Zwilch	151.30	-1.49	6.44E-07
Dynll2	845.90	-0.80	7.59E-10
Rpa1	909.44	-1.41	9.10E-30
Ncapd2	1140.31	-1.45	1.43E-34
Ssna1	244.96	-0.55	1.23E-02
Sgo2a	161.35	-1.61	4.42E-09
Gins1	88.93	-1.57	4.68E-06
Hist1h4h	2419.90	-0.36	1.40E-05
Cenps	30.16	-1.79	7.79E-04
Mcm10	377.03	-0.57	1.01E-03
Smc4	1394.29	-0.40	8.85E-04

Hdac8	152.64	-0.74	5.72E-03
Spdl1	380.25	-1.12	2.25E-10
Cep192	1240.75	-0.98	2.30E-17
Ankle2	1177.66	-0.25	1.81E-02
Nup93	1595.75	-0.27	1.18E-02
Kif23	669.59	-0.61	9.56E-06
Cenpu	44.95	-2.76	1.03E-07
Haus5	110.53	-1.96	1.72E-09
Esco2	159.61	-2.56	2.81E-18
Mzt2	138.63	-1.10	8.96E-05
Tpx2	844.28	-0.34	7.13E-03
Cenpn	165.21	-1.53	3.99E-09
Sgo1	134.49	-1.45	3.22E-07
Psma8	250.65	-0.57	1.15E-02
Kif2c	160.23	-1.16	1.41E-05
Tubgcp2	607.43	-0.37	1.85E-02
Cep57	449.60	-0.62	2.77E-04
Cep72	43.22	-1.33	4.12E-03
Gorasp1	285.15	-0.46	2.72E-02
Mau2	1354.92	-0.33	3.67E-03
Lin37	145.24	-0.91	6.67E-04
Ckap5	2597.66	-0.23	1.09E-02
Ncapg2	792.22	-0.88	1.14E-11
Haus8	290.58	-0.99	8.79E-07
Clasp2	1416.31	-0.44	2.02E-05
Clasp1	1781.71	-0.35	5.61E-04
Sdccag8	349.94	-1.37	1.68E-13
H2afv	158.52	-1.91	6.35E-11
Bora	206.20	-1.46	7.58E-10
Ninl	166.59	-2.27	2.30E-16
Ncapd3	763.86	-1.52	1.61E-30
Gins3	307.42	-2.47	5.04E-31
Sfi1	5006.80	-0.28	4.11E-05
Hist2h3c2	5455.55	-0.35	3.37E-06
Hist1h3g	1066.67	-0.49	2.10E-05
Cep152	434.25	-0.76	2.07E-05
Anapc2	1479.82	-0.71	2.03E-12

M phase

gene_name	baseMean	log2FoldChange	p-value
Pds5b	1062.67	-0.60	4.86E-07
Akap9	4543.13	-0.35	1.01E-04
Numa1	4544.60	-1.33	5.99E-70
Cenpi	99.13	-0.98	2.51E-03

Xpo1	2809.89	-0.71	5.99E-17
Tubg1	499.07	-0.72	3.22E-06
Espl1	548.80	-2.19	1.37E-42
Pom121	2049.09	-0.21	1.91E-02
Cenpf	806.53	-1.26	4.28E-18
Rcc2	9296.26	-0.50	1.99E-13
Birc5	243.39	-1.34	2.50E-09
Cep131	212.51	-2.06	2.76E-15
Bub1	448.05	-0.59	3.94E-04
Bub1b	1044.14	-0.73	1.32E-10
Ccnb2	243.50	-1.20	4.39E-08
Cenpa	249.75	-0.94	7.85E-06
Smc3	2240.69	-0.48	5.32E-07
Dctn1	2434.36	-0.52	9.31E-08
Smc2	1230.27	-0.36	8.36E-04
Lpin1	514.81	-2.41	4.02E-46
Incenp	2669.79	-0.68	4.78E-16
Lmnb1	1011.19	-0.65	1.66E-08
Nup155	1875.43	-0.42	4.09E-06
Mad1l1	514.60	-0.93	1.57E-09
Nek2	309.22	-1.12	8.50E-08
Odf2	1114.99	-0.43	9.24E-04
Pcnt	1669.09	-0.77	1.70E-13
Prkaca	1045.87	-0.81	1.52E-09
Plk1	341.13	-0.83	7.69E-06
Kif20a	369.85	-0.56	1.46E-03
Rangap1	1883.69	-0.45	9.36E-07
Rb1	669.96	-1.95	2.99E-31
Cep78	352.17	-1.47	6.30E-15
Kntc1	505.54	-1.23	4.65E-15
Aurkb	326.09	-2.10	4.89E-26
Cdk5rap2	1429.45	-0.42	1.13E-04
Cep164	889.63	-1.64	7.13E-37
Ncaph	396.92	-1.68	2.10E-17
Ppp2r5d	858.57	-0.91	7.80E-13
Nek9	2821.86	-0.51	1.17E-09
Haus4	133.53	-2.88	1.62E-17
Tmpo	1849.13	-1.36	2.29E-38
Tubb5	11692.54	-0.23	2.19E-04
Vrk1	611.15	-0.42	4.55E-03
Aaas	776.39	-1.41	3.88E-26
Ppp2r5b	365.12	-0.43	1.69E-02
Nup214	2804.45	-0.37	4.47E-06

Kif18a	159.49	-2.02	2.23E-13
Cenpe	838.80	-0.64	8.27E-07
Haus6	385.78	-0.57	9.86E-04
Haus3	278.84	-0.60	4.37E-03
H2afj	1096.77	-0.86	3.67E-13
Tubgcp5	585.92	-0.39	7.23E-03
Nup133	447.74	-0.37	2.72E-02
Ercc6l	161.12	-0.69	6.98E-03
Smc1a	2207.10	-0.54	3.86E-10
Mcph1	718.54	-0.34	1.51E-02
Mapk3	2178.59	-0.73	1.28E-15
Cenph	84.50	-1.82	3.02E-07
Cntrl	1539.62	-0.68	8.23E-10
Ppp2r5c	2449.23	-0.66	1.51E-14
Csnk1e	1187.48	-1.41	1.97E-34
Hist1h3c	1693.67	-0.31	8.60E-04
Hist1h3d	5403.83	-0.49	3.38E-11
Hist1h3b	2233.70	-0.43	6.21E-06
Hist1h3h	2424.17	-0.41	5.02E-06
Hist1h3i	2512.38	-0.31	7.92E-04
Hist2h3b	2811.02	-0.62	2.36E-12
Hist1h4f	1543.29	-0.44	1.42E-04
Hist1h4j	1477.67	-0.39	1.50E-04
Hist1h2ac	2598.46	-0.35	2.88E-05
Hist1h2ae	2711.63	-0.47	1.99E-08
Hist1h2ag	2783.21	-0.59	7.82E-13
Hist1h2an	1678.41	-0.58	2.03E-09
Hist1h2ab	3493.11	-0.59	6.17E-13
Hist1h2bb	2057.12	-0.38	2.36E-05
Hist1h2bk	2094.33	-0.28	3.22E-03
Hist2h2be	115.74	-2.26	7.65E-12
Hist1h2ai	3058.64	-0.48	1.98E-09
Tubgcp6	778.76	-1.16	2.45E-18
Hist1h3a	946.46	-0.45	2.35E-04
Nsl1	152.77	-1.27	2.89E-06
Nup85	706.18	-0.62	1.19E-05
Tubgcp4	661.14	-0.33	1.47E-02
Cdca8	462.85	-0.88	4.11E-08
Cenpo	278.42	-0.48	1.75E-02
Cdc23	532.09	-0.49	9.37E-04
Ncaph2	1215.37	-0.72	2.96E-10
Actr1a	2566.92	-0.28	7.12E-04
Ncapg	378.43	-0.98	1.27E-07

Nup210	1052.97	-1.23	1.41E-24
Mad2l1	252.57	-0.91	1.46E-05
Clip1	2750.78	-0.86	2.36E-22
Anapc5	2441.09	-0.55	1.33E-10
Nek7	1183.56	-0.75	3.67E-12
Nek6	845.71	-0.64	2.91E-06
Cenpk	64.35	-1.58	1.28E-04
Lpin2	840.18	-1.00	5.27E-15
Anapc11	507.59	-0.70	5.40E-06
Cenpp	45.82	-1.13	1.19E-02
Spc25	172.15	-1.45	1.18E-08
Ska1	52.97	-2.18	2.04E-06
Hist1h2ao	6618.69	-0.54	6.28E-16
Cenpm	79.46	-1.80	2.57E-06
Dsn1	136.90	-1.11	2.14E-04
Nuf2	249.28	-1.38	2.95E-10
Pmf1	361.85	-0.74	9.98E-05
Ndc80	181.63	-1.49	2.69E-09
Mastl	147.63	-1.69	8.81E-09
Fbxo5	123.67	-1.70	3.00E-08
Nde1	743.66	-1.22	4.88E-20
Spc24	101.42	-0.75	1.90E-02
Cdca5	180.97	-1.88	3.35E-12
Zwilch	151.30	-1.49	6.44E-07
Dynll2	845.90	-0.80	7.59E-10
Ncapd2	1140.31	-1.45	1.43E-34
Ssna1	244.96	-0.55	1.23E-02
Sgo2a	161.35	-1.61	4.42E-09
Hist1h4h	2419.90	-0.36	1.40E-05
Cenps	30.16	-1.79	7.79E-04
Smc4	1394.29	-0.40	8.85E-04
Hdac8	152.64	-0.74	5.72E-03
Spdl1	380.25	-1.12	2.25E-10
Cep192	1240.75	-0.98	2.30E-17
Ankle2	1177.66	-0.25	1.81E-02
Nup93	1595.75	-0.27	1.18E-02
Kif23	669.59	-0.61	9.56E-06
Cenpu	44.95	-2.76	1.03E-07
Haus5	110.53	-1.96	1.72E-09
Mzt2	138.63	-1.10	8.96E-05
Cenpn	165.21	-1.53	3.99E-09
Sgo1	134.49	-1.45	3.22E-07
Psma8	250.65	-0.57	1.15E-02

Kif2c	160.23	-1.16	1.41E-05
Tubgcp2	607.43	-0.37	1.85E-02
Cep57	449.60	-0.62	2.77E-04
Cep72	43.22	-1.33	4.12E-03
Gorasp1	285.15	-0.46	2.72E-02
Mau2	1354.92	-0.33	3.67E-03
Ckap5	2597.66	-0.23	1.09E-02
Ncapg2	792.22	-0.88	1.14E-11
Haus8	290.58	-0.99	8.79E-07
Clasp2	1416.31	-0.44	2.02E-05
Clasp1	1781.71	-0.35	5.61E-04
Sdccag8	349.94	-1.37	1.68E-13
H2afv	158.52	-1.91	6.35E-11
Ninl	166.59	-2.27	2.30E-16
Ncapd3	763.86	-1.52	1.61E-30
Sfi1	5006.80	-0.28	4.11E-05
Hist2h3c2	5455.55	-0.35	3.37E-06
Hist1h3g	1066.67	-0.49	2.10E-05
Cep152	434.25	-0.76	2.07E-05
Anapc2	1479.82	-0.71	2.03E-12

Mitotic prometaphase

gene_name	baseMean	log2FoldChange	p-value
Pds5b	1062.67	-0.60	4.86E-07
Akap9	4543.13	-0.35	1.01E-04
Numa1	4544.60	-1.33	5.99E-70
Cenpi	99.13	-0.98	2.51E-03
Xpo1	2809.89	-0.71	5.99E-17
Tubg1	499.07	-0.72	3.22E-06
Cenpf	806.53	-1.26	4.28E-18
Rcc2	9296.26	-0.50	1.99E-13
Birc5	243.39	-1.34	2.50E-09
Cep131	212.51	-2.06	2.76E-15
Bub1	448.05	-0.59	3.94E-04
Bub1b	1044.14	-0.73	1.32E-10
Ccnb2	243.50	-1.20	4.39E-08
Cenpa	249.75	-0.94	7.85E-06
Smc3	2240.69	-0.48	5.32E-07
Dctn1	2434.36	-0.52	9.31E-08
Smc2	1230.27	-0.36	8.36E-04
Incenp	2669.79	-0.68	4.78E-16
Mad1l1	514.60	-0.93	1.57E-09
Nek2	309.22	-1.12	8.50E-08
Odf2	1114.99	-0.43	9.24E-04

Pcnt	1669.09	-0.77	1.70E-13
Prkaca	1045.87	-0.81	1.52E-09
Plk1	341.13	-0.83	7.69E-06
Rangap1	1883.69	-0.45	9.36E-07
Cep78	352.17	-1.47	6.30E-15
Kntc1	505.54	-1.23	4.65E-15
Aurkb	326.09	-2.10	4.89E-26
Cdk5rap2	1429.45	-0.42	1.13E-04
Cep164	889.63	-1.64	7.13E-37
Ncaph	396.92	-1.68	2.10E-17
Ppp2r5d	858.57	-0.91	7.80E-13
Haus4	133.53	-2.88	1.62E-17
Tubb5	11692.54	-0.23	2.19E-04
Ppp2r5b	365.12	-0.43	1.69E-02
Kif18a	159.49	-2.02	2.23E-13
Cenpe	838.80	-0.64	8.27E-07
Haus6	385.78	-0.57	9.86E-04
Haus3	278.84	-0.60	4.37E-03
Tubgcp5	585.92	-0.39	7.23E-03
Nup133	447.74	-0.37	2.72E-02
Ercc6l	161.12	-0.69	6.98E-03
Smc1a	2207.10	-0.54	3.86E-10
Cenph	84.50	-1.82	3.02E-07
Cntrl	1539.62	-0.68	8.23E-10
Ppp2r5c	2449.23	-0.66	1.51E-14
Csnk1e	1187.48	-1.41	1.97E-34
Tubgcp6	778.76	-1.16	2.45E-18
Nsl1	152.77	-1.27	2.89E-06
Nup85	706.18	-0.62	1.19E-05
Tubgcp4	661.14	-0.33	1.47E-02
Cdca8	462.85	-0.88	4.11E-08
Cenpo	278.42	-0.48	1.75E-02
Actr1a	2566.92	-0.28	7.12E-04
Ncapg	378.43	-0.98	1.27E-07
Mad2l1	252.57	-0.91	1.46E-05
Clip1	2750.78	-0.86	2.36E-22
Cenpk	64.35	-1.58	1.28E-04
Cenpp	45.82	-1.13	1.19E-02
Spc25	172.15	-1.45	1.18E-08
Ska1	52.97	-2.18	2.04E-06
Cenpm	79.46	-1.80	2.57E-06
Dsn1	136.90	-1.11	2.14E-04
Nuf2	249.28	-1.38	2.95E-10

Pmf1	361.85	-0.74	9.98E-05
Ndc80	181.63	-1.49	2.69E-09
Nde1	743.66	-1.22	4.88E-20
Spc24	101.42	-0.75	1.90E-02
Cdca5	180.97	-1.88	3.35E-12
Zwilch	151.30	-1.49	6.44E-07
Dynll2	845.90	-0.80	7.59E-10
Ncapd2	1140.31	-1.45	1.43E-34
Ssna1	244.96	-0.55	1.23E-02
Sgo2a	161.35	-1.61	4.42E-09
Cenps	30.16	-1.79	7.79E-04
Smc4	1394.29	-0.40	8.85E-04
Hdac8	152.64	-0.74	5.72E-03
Spdl1	380.25	-1.12	2.25E-10
Cep192	1240.75	-0.98	2.30E-17
Cenpu	44.95	-2.76	1.03E-07
Haus5	110.53	-1.96	1.72E-09
Mzt2	138.63	-1.10	8.96E-05
Cenpn	165.21	-1.53	3.99E-09
Sgo1	134.49	-1.45	3.22E-07
Kif2c	160.23	-1.16	1.41E-05
Tubgcp2	607.43	-0.37	1.85E-02
Cep57	449.60	-0.62	2.77E-04
Cep72	43.22	-1.33	4.12E-03
Ckap5	2597.66	-0.23	1.09E-02
Haus8	290.58	-0.99	8.79E-07
Clasp2	1416.31	-0.44	2.02E-05
Clasp1	1781.71	-0.35	5.61E-04
Sdccag8	349.94	-1.37	1.68E-13
Ninl	166.59	-2.27	2.30E-16
Sfi1	5006.80	-0.28	4.11E-05
Cep152	434.25	-0.76	2.07E-05

Table S2 Differentially methylated genes following *P. aeruginosa* infection

Differentially methylated genes following 2 h *P. aeruginosa* infection

Gene expression (transcription)

gene_name	chr	chromStart	chromEnd	diff.lg.p	diff.log2.fc
Cnot11	chr1	39592727	39593027	-0.49	-0.09
Cnot11	chr1	39603302	39603662	-1.63	-0.30
Cnot9	chr1	74575438	74575798	-2.06	-0.28
Psmd1	chr1	87961193	87961313	-0.67	0.21
Ing5	chr1	95714871	95715861	-2.79	-0.31
Ccnt2	chr1	129670740	129670861	-1.38	0.49
Ccnt2	chr1	129698762	129700591	-0.54	0.03
Rbbp5	chr1	134394590	134400982	-1.17	0.15
Rbbp5	chr1	134401131	134401312	-0.34	-0.03
Kdm5b	chr1	136514466	136514617	-0.68	0.28
Kdm5b	chr1	136521310	136521760	-2.33	0.45
Kdm5b	chr1	136525288	136527418	-0.37	-0.03
Kdm5b	chr1	136527777	136528227	-1.23	0.27
Nr1i3	chr1	173148035	173148485	-1.67	-0.41
Nr1i3	chr1	173148844	173149234	-0.72	-0.23
Exo1	chr1	177829710	177831526	-1.63	-0.39
Exo1	chr1	177838863	177839044	-0.20	0.03
Exo1	chr1	177839162	177839433	-0.27	0.29
Parp1	chr1	182499105	182503843	-1.60	-0.27
Trp53bp2	chr1	184371133	184371894	-0.41	-0.09
Trp53bp2	chr1	184376642	184379234	-0.81	0.12
Trp53bp2	chr1	184389061	184392388	-0.44	-0.06
Ints7	chr1	193443646	193445390	-0.54	-0.07
Gls	chr1	52244565	52244656	-2.44	0.47
Sf3b1	chr1	55084201	55084322	-0.54	-0.12
Sumo1	chr1	59696455	59696989	-1.11	-0.09
Bard1	chr1	71121331	71122020	-0.53	0.08
Zc3h11a	chr1	135516447	135517433	-0.47	-0.02
Zc3h11a	chr1	135517641	135518733	-1.03	0.06
Zc3h11a	chr1	135521310	135523811	-2.56	0.12
Btg2	chr1	135971471	135971532	-0.90	0.83
Btg2	chr1	135973595	135975732	-0.89	-0.05
Rnf2	chr1	153318408	153318528	-0.89	0.28
Pou2f1	chr1	167805604	167806174	-0.35	0.03
Tfb2m	chr1	181476069	181476398	-0.78	-0.18
Smyd2	chr1	191704578	191706000	-1.27	0.17
Bmi1	chr2	18605667	18606468	-0.89	0.08
Rxra	chr2	27597444	27604173	-0.47	-0.11
Rxra	chr2	27617342	27618510	-1.22	-0.15

Tsc1	chr2	28531258	28532060	-2.14	0.31
Tsc1	chr2	28541313	28542943	-1.55	-0.18
Tsc1	chr2	28546177	28546268	-0.26	0.00
Ttf1	chr2	28915782	28922656	-0.49	0.04
Ttf1	chr2	28941735	28942005	-1.00	-0.33
Abl1	chr2	31544075	31551320	-0.40	0.02
Abl1	chr2	31652573	31658159	-1.65	-0.10
Abl1	chr2	31658367	31658428	-1.12	-0.61
Abl1	chr2	31659237	31659477	-0.56	0.58
Nup214	chr2	31888785	31893653	-1.32	0.09
Mapkap1	chr2	34479093	34479423	-1.30	0.22
Psmc3	chr2	90894439	90895182	-1.38	-0.10
Cstf3	chr2	104504891	104505372	-2.37	0.28
Rtf1	chr2	119558206	119559195	-0.92	-0.10
Rtf1	chr2	119560243	119560484	-1.04	0.33
Mga	chr2	119698286	119698347	-0.18	0.00
Mga	chr2	119767083	119771919	-0.49	0.05
Mga	chr2	119787692	119790793	-2.58	-0.17
Polr1b	chr2	128950988	128952033	-0.87	-0.08
Polr3f	chr2	144362909	144364972	-2.00	0.21
Itch	chr2	154959216	154959307	-0.25	0.00
Itch	chr2	155050132	155050373	-0.80	-0.16
Phf20	chr2	156092990	156093201	-1.93	0.33
Phf20	chr2	156113573	156113634	-1.16	0.44
Tgif2	chr2	156679040	156679219	-0.58	0.85
Tgif2	chr2	156679307	156679813	-0.75	-0.28
Src	chr2	157295226	157296471	-0.85	0.32
Rprd1b	chr2	157900721	157901141	-1.50	-0.24
Trp53rkb	chr2	166619352	166619563	-0.37	0.13
Trp53rkb	chr2	166620915	166621755	-2.59	-0.52
Cstf1	chr2	172201351	172201608	-2.58	0.38
Rae1	chr2	172839488	172841203	-2.88	-0.32
Polr3k	chr2	181599124	181600679	-0.81	0.23
Gata3	chr2	9779567	9784722	-1.35	0.40
Gata3	chr2	9784840	9796310	-0.60	0.24
Taf3	chr2	9837317	9964072	-0.79	0.05
Pip4k2a	chr2	18765497	18769115	-1.74	0.11
Ehmt1	chr2	24646742	24647852	-1.08	-0.13
Ehmt1	chr2	24733063	24775080	-2.58	0.23
Snapc4	chr2	26218284	26222767	-0.95	-0.09
Snapc4	chr2	26223896	26225464	-1.39	0.24
Notch1	chr2	26314049	26314319	-1.37	1.42
Notch1	chr2	26315036	26315097	-0.53	-1.83

Notch1	chr2	26315275	26315486	-0.62	0.91
Notch1	chr2	26316053	26316413	-0.28	0.10
Notch1	chr2	26323937	26324117	-0.52	0.80
Notch1	chr2	26336689	26337202	-0.49	1.03
Notch1	chr2	26339865	26340015	-0.40	0.61
Gtf3c5	chr2	28421841	28423224	-1.34	0.19
Gtf3c5	chr2	28438382	28438502	-1.35	-0.46
Gtf3c4	chr2	28679915	28680574	-3.04	-0.40
Gtf3c4	chr2	28680723	28683020	-3.63	-0.29
Gtf3c4	chr2	28686074	28690190	-3.59	-0.23
Gtf3c4	chr2	28690308	28692035	-3.64	0.32
Gtf3c4	chr2	28695490	28695880	-0.65	-0.15
Psmd5	chr2	34707993	34709794	-1.37	-0.17
Psmd5	chr2	34722532	34726482	-1.60	-0.40
Psmb7	chr2	38498946	38499426	-1.35	-0.19
Nr6a1	chr2	38781663	38781753	-0.49	-0.29
Nr4a2	chr2	56960175	56961102	-1.17	-0.25
Nr4a2	chr2	56964099	56965537	-0.49	-0.07
Nr4a2	chr2	56967204	56967355	-0.52	0.68
Clp1	chr2	84563548	84566299	-2.19	-0.20
Zfp661	chr2	127402341	127403565	-2.72	0.50
Snrpb	chr2	130002885	130005100	-2.05	-0.18
Pcna	chr2	132078555	132078916	-3.65	-0.19
Psmf1	chr2	151543111	151543232	-0.83	-0.47
Psmf1	chr2	151543649	151544068	-0.81	-0.19
Zfp334	chr2	165203294	165203715	-1.00	0.46
Zfp334	chr2	165205211	165206201	-0.49	0.09
Zfp334	chr2	165206829	165207339	-2.14	0.67
Trp53rka	chr2	165315878	165318814	-0.78	-0.11
Taf4	chr2	179647358	179656708	-0.54	-0.05
Skil	chr3	30994042	30997382	-1.57	0.11
Gatad2b	chr3	90127990	90145879	-0.41	-0.02
Gatad2b	chr3	90155787	90155878	-3.11	-0.35
Rorc	chr3	94201354	94201475	-0.30	1.58
Hist2h2be	chr3	96025043	96025490	-1.83	0.52
Hist2h2be	chr3	96026738	96027215	-1.30	-0.39
Rbm8a	chr3	96433850	96433910	-0.37	0.11
Notch2	chr3	97935343	97941284	-2.25	-0.13
Trim33	chr3	103148255	103148495	-1.27	0.15
Trim33	chr3	103156073	103158203	-1.33	0.10
Trim33	chr3	103158830	103159400	-0.30	0.00
Ints12	chr3	132771889	132772487	-0.74	-0.08
Ube2d3	chr3	135101656	135102228	-3.24	-0.27

Gtf2b	chr3	142428210	142434431	-1.34	-0.30
Gtf2b	chr3	142446182	142446450	-0.48	0.05
Phc3	chr3	30805887	30813151	-0.57	-0.08
Phc3	chr3	30864703	30868337	-0.42	0.12
Elf2	chr3	51059646	51060809	-1.08	-0.08
Elf2	chr3	51061575	51064964	-0.90	-0.11
Maml3	chr3	51491863	51496200	-1.51	0.17
Maml3	chr3	51501603	51600338	-0.59	-0.42
Maml3	chr3	51907609	51907880	-3.24	0.71
Maml3	chr3	51907939	51908209	-2.93	0.70
Maml3	chr3	51908358	51908838	-1.96	0.46
Lamtor2	chr3	88353798	88356849	-1.88	-0.11
Ints3	chr3	90195369	90196223	-1.47	-0.15
Ints3	chr3	90237228	90237558	-1.87	-0.39
Chtop	chr3	90303653	90304012	-2.00	-0.11
Chtop	chr3	90313185	90313365	-2.41	-0.21
Psmd4	chr3	94836626	94836924	-1.79	-0.12
Psmd4	chr3	94839816	94846467	-2.16	-0.13
Rprd2	chr3	95567509	95570116	-2.33	-0.11
Rprd2	chr3	95576734	95577124	-2.87	0.55
Hist2h3c2	chr3	96042454	96042544	-0.57	0.69
Hist2h2aa1	chr3	96044071	96044216	-1.04	1.17
Prmt6	chr3	110050616	110051517	-1.67	-0.58
Prmt6	chr3	110052356	110053916	-3.07	-0.18
Srsf11	chr3	157674772	157675838	-1.15	-0.06
Rngtt	chr4	33587224	33587763	-1.62	0.30
Rngtt	chr4	33588270	33588390	-0.93	0.89
Polr1e	chr4	45044855	45045245	-0.36	0.05
Polr1e	chr4	45045964	45046084	-1.09	-1.30
Nr4a3	chr4	48058191	48058281	-0.24	-0.14
Nr4a3	chr4	48061990	48065027	-1.94	-0.50
Nr4a3	chr4	48083931	48096224	-1.06	-0.81
Zfp189	chr4	49542890	49543460	-2.62	0.62
Rraga	chr4	86221606	86222293	-0.53	0.03
Ndc1	chr4	107083754	107086943	-0.44	0.04
Tal1	chr4	114737352	114741762	-1.59	-0.29
Tal1	chr4	114741970	114742061	-0.45	-0.22
Med8	chr4	118087440	118087618	-2.77	0.38
Scmh1	chr4	120201875	120202085	-2.07	0.58
Rragc	chr4	123597654	123598314	-1.34	0.21
Rragc	chr4	123612802	123613821	-0.41	-0.01
Rragc	chr4	123614089	123614180	-1.02	0.20
Lsm10	chr4	125773896	125775828	-1.43	-0.17

Phc2	chr4	128404910	128405061	-1.05	-0.71
Rpa2	chr4	132333796	132334035	-1.34	-0.32
Nr0b2	chr4	133112127	133112363	-0.61	0.57
Zfp46	chr4	135846237	135848631	-0.51	-0.05
Mtor	chr4	147822690	147826440	-1.12	-0.33
Mtor	chr4	147930392	147931764	-0.80	0.10
Ssu72	chr4	155078923	155079163	-2.43	1.17
Ssu72	chr4	155079462	155089762	-0.81	-0.07
Mllt3	chr4	87419218	87419819	-0.46	0.07
Mllt3	chr4	87486705	87487246	-0.69	0.10
Zfp691	chr4	118842660	118845711	-0.35	0.03
Zfp69	chr4	120602801	120603909	-2.55	0.49
Ago1	chr4	126116411	126116652	-0.93	0.28
Ago4	chr4	126169807	126170316	-2.25	1.07
Ago4	chr4	126170375	126170526	-2.15	1.17
Sfn	chr4	133156530	133158083	-0.66	0.16
Srrm1	chr4	134878175	134878385	-0.88	-0.09
Srrm1	chr4	134902739	134909069	-1.22	0.14
Eloa	chr4	135561252	135561580	-1.14	0.19
Eloa	chr4	135565701	135577564	-2.32	0.10
Ski	chr4	154529765	154533533	-2.17	0.10
Ski	chr4	154533945	154596644	-1.11	-0.08
Cdk6	chr5	3519406	3520884	-0.47	0.04
Mterf1b	chr5	4192366	4196581	-0.29	-0.06
Kmt2e	chr5	22940246	22940516	-2.82	0.51
Kmt2e	chr5	23002909	23005273	-0.76	0.07
Kmt2e	chr5	23007381	23008999	-1.16	0.08
Nupl2	chr5	23687756	23687817	-0.27	0.02
Nupl2	chr5	23688026	23689736	-0.62	0.07
Ywhah	chr5	33361494	33361614	-0.40	0.10
Ywhah	chr5	33369269	33370047	-0.64	0.02
Rbpj	chr5	54044845	54045146	-0.26	0.54
Mtf2	chr5	108494760	108498287	-1.05	-0.22
Mtf2	chr5	108498346	108498406	-0.39	0.12
Mtf2	chr5	108535653	108536103	-1.66	-0.22
Zfp605	chr5	110556631	110557140	-1.63	0.56
Zfp605	chr5	110557318	110558156	-0.32	-0.03
Triap1	chr5	115791255	115793471	-0.71	-0.07
Rnf34	chr5	123273869	123314504	-1.72	0.21
Psmd9	chr5	123678228	123684616	-1.07	0.18
Kmt5a	chr5	124911176	124911327	-0.89	-0.12
Gtf2h3	chr5	125046222	125046313	-0.93	-0.48
Zfp664	chr5	125343194	125366362	-1.24	-0.09

Zfp664	chr5	125366721	125367081	-0.31	0.01
Crcp	chr5	130505175	130513761	-0.96	0.20
Crcp	chr5	130535663	130536293	-0.36	-0.02
Rfc2	chr5	135073840	135074198	-0.38	-0.03
Baz1b	chr5	135663192	135663612	-3.23	-0.46
Baz1b	chr5	135718322	135721070	-1.09	-0.07
Zkscan1	chr5	138526311	138534046	-0.73	0.23
Zkscan1	chr5	138541914	138542485	-0.51	0.05
Zkscan1	chr5	138542723	138543984	-1.79	0.16
Zkscan1	chr5	138544222	138545962	-0.77	-0.07
Zfp12	chr5	144005811	144007881	-0.65	-0.08
Zfp12	chr5	144007940	144008390	-0.69	-0.23
Zkscan5	chr5	145981691	145982470	-2.39	0.63
Zfp655	chr5	145992583	145994463	-2.43	-0.45
Zfp655	chr5	146004460	146006138	-2.25	0.12
Cdk8	chr5	147042885	147071118	-2.04	-0.41
Smarcd3	chr5	24100691	24101019	-0.21	0.00
Prkag2	chr5	24605830	24606400	-1.25	0.18
Kmt2c	chr5	24790108	24790979	-0.87	0.09
Slbp	chr5	33992485	33994955	-0.80	0.14
Nup54	chr5	92845130	92846543	-1.56	0.19
Prkab1	chr5	116463747	116468029	-0.50	-0.04
Prkab1	chr5	116474109	116474378	-0.66	-0.12
Ptpn11	chr5	121581827	121582307	-2.13	0.25
Ptpn11	chr5	121583292	121587519	-0.51	-0.03
Ncor2	chr5	125514394	125514836	-0.46	-0.06
Ncor2	chr5	125538274	125548306	-0.47	0.04
Ncor2	chr5	125659463	125659584	-0.26	-0.17
Zfp11	chr5	130162143	130162980	-1.02	0.38
Zfp11	chr5	130164055	130166739	-0.47	0.31
Pom121	chr5	135853620	135857198	-0.30	0.00
Pom121	chr5	135857496	135857795	-0.92	0.12
Pom121	chr5	135869997	135870416	-0.68	-0.16
Zfp113	chr5	138585511	138586171	-1.24	-0.27
Zfp113	chr5	138586380	138586650	-1.50	0.58
Ints1	chr5	140251541	140251602	-0.37	0.17
Smurf1	chr5	145637693	145638023	-0.74	-0.29
Smurf1	chr5	145638352	145638682	-1.09	-0.34
Smurf1	chr5	145639310	145639491	-0.37	0.13
Zkscan14	chr5	145956021	145962515	-3.26	-0.45
Rfc3	chr5	152445398	152445668	-1.50	0.34
Nup205	chr6	35127615	35133895	-0.29	0.01
Zfp398	chr6	47785869	47790711	-0.90	0.31

Zfp398	chr6	47815946	47817117	-0.67	-0.11
Zfp282	chr6	47827703	47830376	-0.29	0.00
Zfp282	chr6	47854827	47855966	-3.17	-0.33
Zfp282	chr6	47856175	47856835	-1.42	-0.27
Zfp212	chr6	47870625	47876800	-0.60	0.18
Zfp212	chr6	47879122	47881565	-1.03	-0.20
Zfp775	chr6	48555624	48570026	-1.58	-0.60
Zfp775	chr6	48570354	48570923	-1.34	-0.72
Polr1a	chr6	71928026	71930161	-1.86	-0.16
Polr1a	chr6	71934658	71934809	-0.38	0.29
Gata2	chr6	88154683	88155799	-3.13	-0.61
Nr2c2	chr6	92114256	92117902	-2.91	-0.32
Brpf1	chr6	113257160	113269353	-0.42	0.01
Chd4	chr6	125079888	125080399	-1.62	-0.07
Cdkn1b	chr6	134870898	134872390	-2.89	0.28
Gm8994	chr6	136278992	136279111	-0.18	0.47
Gm8994	chr6	136279792	136279971	-0.26	0.19
H2afj	chr6	136756828	136756889	-0.45	0.13
H2afj	chr6	136757307	136757577	-1.31	-0.28
Aebp2	chr6	140571543	140572383	-1.35	-0.28
Aebp2	chr6	140572622	140573517	-2.75	-0.28
Aebp2	chr6	140602621	140602921	-1.72	0.28
Hipk2	chr6	38647868	38666025	-2.58	-0.11
Hipk2	chr6	38697991	38787047	-0.54	-0.02
Hipk2	chr6	38825859	38826040	-2.58	-0.39
Zfp786	chr6	47769651	47770337	-0.42	-0.30
Zfp786	chr6	47770485	47771170	-0.67	0.66
Zfp786	chr6	47771318	47771676	-0.36	-0.40
Zfp746	chr6	48013500	48016503	-1.38	-0.15
Zfp746	chr6	48033553	48036532	-0.55	-0.18
Gadd45a	chr6	66985178	66987371	-2.48	-0.22
Nup210	chr6	90963060	90963720	-3.45	-0.38
Rybp	chr6	100182274	100183082	-1.03	0.42
Zfp248	chr6	118378764	118380402	-3.16	0.57
Phc1	chr6	122273438	122273618	-1.06	0.49
Phc1	chr6	122275560	122275830	-0.30	-0.03
Tigar	chr6	127037498	127038307	-0.73	0.09
Ccnd2	chr6	127079556	127080245	-1.36	-0.18
Rhno1	chr6	128307315	128309210	-1.80	-0.15
Kras	chr6	145168783	145169023	-0.29	0.00
Zfp667	chr7	6256313	6256524	-1.53	1.14
Zfp667	chr7	6256792	6256942	-0.28	-0.11
Zfp667	chr7	6257719	6257870	-0.76	0.76

Zfp78	chr7	6331042	6331103	-0.19	0.59
Zfp78	chr7	6331550	6332298	-1.80	0.98
Zfp28	chr7	6344966	6347349	-0.99	0.23
Zfp110	chr7	13434054	13434502	-0.38	-0.03
Zfp324	chr7	13555672	13556391	-2.57	-0.89
Zfp324	chr7	13556928	13557676	-0.54	-0.23
Zfp446	chr7	13563376	13563437	-0.26	-0.09
Zfp446	chr7	13564395	13564546	-0.27	-0.02
Zfp446	chr7	13567226	13568306	-0.59	-0.12
Zfp446	chr7	13568485	13569026	-1.73	-0.53
Ppp1r13l	chr7	19956867	19960636	-1.70	-0.27
Ercc2	chr7	19979441	19981041	-2.11	-0.19
Zfp180	chr7	24882912	24891576	-1.89	-0.12
Zfp112	chr7	24910123	24910454	-0.36	-0.31
Zfp112	chr7	24910962	24911533	-0.46	0.24
Zfp112	chr7	24912371	24912551	-1.18	1.63
Zfp235	chr7	24921520	24921581	-0.55	1.10
Zfp235	chr7	24925681	24925772	-0.67	0.72
Zfp235	chr7	24926760	24927241	-1.64	-0.63
Zfp235	chr7	24927899	24928200	-0.30	-0.12
Akt2	chr7	28422202	28423428	-1.06	0.15
Paf1	chr7	29178014	29178074	-0.35	0.09
Paf1	chr7	29181953	29184402	-2.55	-0.12
Zfp30	chr7	30577491	30577582	-2.02	2.09
Zfp30	chr7	30577790	30578810	-2.01	0.66
Zfp790	chr7	30613525	30616012	-0.65	-0.06
Zfp420	chr7	30645086	30661309	-0.62	-0.06
Zfp382	chr7	30916575	30919873	-2.17	-0.19
Polr2i	chr7	31017332	31017772	-0.29	0.00
Zfp658	chr7	50827988	50828378	-0.42	0.12
Zfp719	chr7	50835075	50835135	-1.05	0.74
Zfp719	chr7	50841731	50846748	-2.68	0.18
Zfp819	chr7	50873172	50873292	-0.50	3.17
Gtf2h1	chr7	54051493	54051554	-1.82	-0.48
Zfp710	chr7	87169699	87227267	-2.58	-0.11
Zfp710	chr7	87231444	87231625	-0.38	0.06
Zfp710	chr7	87233516	87236264	-1.55	0.11
Lamtor1	chr7	109054380	109058211	-1.32	-0.28
Ctr9	chr7	118197379	118199651	-2.59	0.24
Setd1a	chr7	134920932	134920993	-1.26	1.23
Setd1a	chr7	134927635	134930705	-0.77	0.04
Setd1a	chr7	134931750	134931998	-1.29	-0.20
Zik1	chr7	11075804	11075955	-0.39	-0.19

Zfp551	chr7	13000735	13000946	-0.79	1.17
Cd3eap	chr7	19943033	19943304	-0.29	0.00
Zfp94	chr7	25087170	25088874	-0.51	-0.11
Pou2f2	chr7	25901224	25917479	-3.35	1.22
Supt5	chr7	29116415	29116476	-0.45	0.06
Psmd8	chr7	29965393	29965573	-1.95	-0.13
Zfp940	chr7	30629168	30629499	-0.28	0.07
Zfp940	chr7	30629558	30629679	-0.68	0.98
Zfp940	chr7	30630277	30630518	-0.33	-0.19
Zfp940	chr7	30630847	30631058	-1.56	1.10
Zfp27	chr7	30678475	30680419	-0.34	0.01
Zfp27	chr7	30680747	30683850	-0.38	-0.03
Zfp14	chr7	30823226	30823406	-0.45	0.71
Zfp14	chr7	30823704	30823854	-0.93	1.66
Zfp14	chr7	30823972	30824152	-0.75	1.91
Zfp566	chr7	30862474	30863454	-2.54	0.50
Kmt2b	chr7	31368475	31373655	-3.36	-0.13
Ccne1	chr7	38883150	38883388	-1.47	-0.46
Zfp715	chr7	50552967	50556619	-3.08	0.17
Nr1h2	chr7	51806700	51806791	-0.86	0.19
Zfp473	chr7	51987480	51988021	-1.22	1.42
Zfp473	chr7	51988559	51988710	-0.18	-0.04
Zfp473	chr7	51989249	51989760	-1.53	1.19
Med25	chr7	52132313	52133597	-1.95	-0.23
Bax	chr7	52722059	52722238	-0.83	0.22
E2f8	chr7	56126083	56127247	-1.54	-0.22
Blm	chr7	87647232	87647711	-0.92	-0.15
Blm	chr7	87654407	87659523	-2.35	0.28
Eed	chr7	97125526	97129068	-0.79	-0.09
Eed	chr7	97129246	97129456	-2.79	0.48
Pcf11	chr7	99801691	99808521	-0.95	-0.05
Nup98	chr7	109268003	109269291	-1.15	-0.06
Rrp8	chr7	112881232	112881890	-0.31	0.01
Rrp8	chr7	112882533	112882833	-0.32	-0.01
Rrp8	chr7	112883248	112885029	-0.41	0.04
Gtf3c1	chr7	132784557	132785247	-0.68	-0.07
Gtf3c1	chr7	132786109	132787873	-2.16	-0.19
Gtf3c1	chr7	132790192	132791129	-1.43	0.15
Gtf3c1	chr7	132850991	132851202	-1.20	0.24
Zfp747	chr7	134516408	134519504	-2.59	0.25
9130019O22Rik	chr7	134525922	134526311	-0.65	0.64
9130019O22Rik	chr7	134526817	134529144	-1.48	0.21
E430018J23Rik	chr7	134534349	134536898	-0.64	-0.15

E430018J23Rik	chr7	134536956	134537135	-0.28	-0.18
Zfp764	chr7	134547867	134549152	-2.70	-0.38
Zfp764	chr7	134550156	134550306	-0.31	-0.13
Zfp688	chr7	134562479	134563593	-0.33	-0.02
Zfp688	chr7	134565458	134565548	-0.42	-0.46
Zfp689	chr7	134587294	134588642	-1.03	-0.25
Zfp689	chr7	134592252	134592433	-0.97	0.50
Zfp668	chr7	135009321	135009472	-0.34	-0.12
Pidd1	chr7	148624413	148625004	-2.27	-0.57
Pidd1	chr7	148628991	148629907	-3.39	-1.06
Polr2l	chr7	148659279	148661052	-0.40	0.16
Ccnd1	chr7	152118441	152118622	-0.78	-0.05
Ccnd1	chr7	152125529	152125830	-0.84	0.07
Snapc2	chr8	4255341	4256190	-0.53	-0.07
Tfdp1	chr8	13377166	13377345	-1.90	-0.26
Tfdp1	chr8	13377582	13377702	-2.49	-0.30
Kat6a	chr8	24040729	24043035	-1.41	0.13
Kat6a	chr8	24046098	24051460	-0.39	-0.01
Ppp2cb	chr8	34710092	34710330	-0.74	-0.58
Gtf2e2	chr8	34886483	34887084	-2.84	0.31
Nr2c2ap	chr8	72655231	72655498	-2.58	0.60
Nr2c2ap	chr8	72657115	72657470	-1.15	-0.17
Ell	chr8	73063573	73102761	-1.80	0.39
Ell	chr8	73105671	73115739	-2.29	0.17
Sin3b	chr8	75247186	75248032	-0.43	0.05
Sin3b	chr8	75273726	75273996	-2.25	0.63
Sin3b	chr8	75280901	75281383	-2.17	-0.52
Nr3c2	chr8	79432284	79432583	-1.74	2.06
Nr3c2	chr8	79433507	79433836	-1.15	2.46
Polr2c	chr8	97387425	97387634	-0.99	-0.15
Cbfb	chr8	107694603	107694841	-1.27	-0.20
Zfp90	chr8	108948628	108949645	-2.85	0.58
Zfp1	chr8	114190002	114194792	-0.82	0.10
Banp	chr8	124474462	124474523	-0.29	0.19
Banp	chr8	124549891	124550071	-0.27	0.02
Banp	chr8	124550280	124550791	-1.64	0.85
Banp	chr8	124550969	124551120	-0.31	-0.26
Banp	chr8	124551299	124552918	-1.18	0.43
Zfpm1	chr8	124806220	124856010	-0.73	0.11
Zfpm1	chr8	124858912	124861147	-3.23	0.22
Ash2l	chr8	26950536	26958082	-3.54	-0.43
Brf2	chr8	28234303	28236503	-0.29	0.00
Brf2	chr8	28238897	28239104	-0.46	0.12

Wrn	chr8	34346284	34346854	-0.31	-0.02
Cnot7	chr8	41592761	41593001	-1.13	0.18
Ing2	chr8	48753850	48754631	-3.25	-0.30
Gatad2a	chr8	72459689	72459840	-1.48	-0.16
Gatad2a	chr8	72497999	72498150	-0.82	-0.30
Gatad2a	chr8	72519948	72520248	-0.90	-0.33
Med26	chr8	75019172	75072090	-1.92	-0.15
Smarca5	chr8	83263088	83263358	-0.80	-0.17
Psmb10	chr8	108461631	108461997	-1.25	-0.38
Dhx38	chr8	112071923	112073753	-2.72	0.29
Taf1c	chr8	122121770	122123330	-0.32	0.01
Maml2	chr9	13510263	13511312	-0.79	0.20
Pin1	chr9	20456573	20459918	-2.86	-0.44
Smarca4	chr9	21505619	21507475	-1.36	0.14
Zfp202	chr9	40014929	40015928	-0.32	0.03
Zfp202	chr9	40018505	40020086	-3.02	0.31
Usp2	chr9	43883576	43884236	-2.87	2.50
Usp2	chr9	43902032	43902332	-0.41	-0.10
H2afx	chr9	44143446	44144156	-2.11	-0.11
Ppp2r1b	chr9	50687990	50688710	-0.88	-0.11
Ppp2r1b	chr9	50701016	50701705	-0.62	0.10
Psma4	chr9	54805658	54805748	-1.39	0.21
Sin3a	chr9	56973115	56975576	-0.47	0.04
Snapc5	chr9	64027103	64030261	-2.64	0.30
Ints14	chr9	64809641	64809731	-1.87	0.71
Ints14	chr9	64833706	64834423	-1.41	0.15
Rora	chr9	69226655	69226835	-0.29	0.06
Ice2	chr9	69263440	69263950	-2.00	0.32
Ice2	chr9	69280013	69280223	-0.38	0.10
Atr	chr9	95765519	95765729	-0.56	0.14
Zkscan7	chr9	122797636	122797935	-0.27	-0.02
Zkscan7	chr9	122803837	122804973	-0.35	0.05
Med17	chr9	15066624	15066715	-0.36	-0.07
Zfp558	chr9	18260716	18261406	-1.01	0.57
Zfp426	chr9	20273262	20273323	-0.75	2.21
Zfp426	chr9	20274940	20276201	-1.15	0.20
Zfp266	chr9	20304149	20306501	-1.23	-0.14
Dnmt1	chr9	20734577	20736587	-0.40	-0.05
Kmt2a	chr9	44615605	44617182	-1.02	0.14
Kmt2a	chr9	44650695	44650756	-3.48	0.90
Atm	chr9	53247473	53248264	-1.08	0.14
Pml	chr9	58067532	58068881	-1.38	-0.21
Pml	chr9	58077807	58077868	-0.49	0.22

Pml	chr9	58080966	58094982	-3.67	-0.47
Pml	chr9	58097382	58097563	-0.75	-0.30
Smad3	chr9	63605680	63605801	-0.63	0.71
Smad3	chr9	63605729	63605891	-0.25	0.23
Rnf111	chr9	70274585	70277390	-1.13	-0.14
Tcf12	chr9	71693887	71694367	-2.25	0.19
Tcf12	chr9	71958831	71958922	-0.41	-0.08
Tcf12	chr9	71959385	71959626	-1.17	-0.25
Atrip	chr9	108960445	108961754	-0.40	-0.01
Atrip	chr9	108967670	108968495	-0.58	0.09
Cnot10	chr9	114541840	114549228	-0.65	0.12
Sgk1	chr10	21718603	21718843	-0.48	0.25
Sesn1	chr10	41530409	41607263	-1.97	-0.34
Sesn1	chr10	41627317	41627765	-1.28	-0.38
Dna2	chr10	62436065	62436156	-0.32	-0.08
Stk11	chr10	79579091	79588239	-2.17	0.17
Stk11	chr10	79592650	79593215	-0.48	-0.05
Txnrd1	chr10	82296755	82323149	-0.66	-0.19
Polr3b	chr10	84188455	84188965	-1.04	-0.18
E2f7	chr10	110216069	110216279	-0.34	0.10
Smarcc2	chr10	127920231	127921148	-2.34	0.28
Smarcc2	chr10	127924513	127924663	-1.10	-0.30
Smarcc2	chr10	127926093	127926303	-3.40	0.46
Gtf3c6	chr10	39969066	39969564	-0.76	0.13
Ddit4	chr10	59412599	59412718	-2.71	0.89
Ddit4	chr10	59412895	59414518	-0.91	-0.09
Sirt1	chr10	62801454	62801783	-1.02	-0.51
Nrbf2	chr10	66729853	66730867	-0.88	0.10
Ube2d1	chr10	70718055	70718592	-2.86	0.34
Smarcb1	chr10	75359661	75359780	-2.54	-0.51
Polrmt	chr10	79201153	79202890	-1.52	-0.21
Polrmt	chr10	79206001	79209326	-3.36	-0.39
Polr2e	chr10	79498845	79498935	-0.48	-0.07
Polr2e	chr10	79501286	79502404	-0.85	-0.12
Tcf3	chr10	79872386	79873344	-0.38	0.02
Cradd	chr10	94637889	94638400	-0.76	0.21
Cradd	chr10	94785366	94786140	-0.40	0.11
Cnot2	chr10	115922216	115922606	-1.24	0.23
Cnot2	chr10	115922874	115923115	-0.75	0.16
Yeats4	chr10	116652435	116652884	-0.66	0.08
Mdm2	chr10	117126799	117129688	-0.41	0.01
Dyrk2	chr10	118296404	118305870	-2.04	-0.14
Pip4k2c	chr10	126635286	126635944	-0.82	0.09

Pip4k2c	chr10	126636701	126637297	-0.51	-0.06
Cdk2	chr10	128135891	128136071	-0.28	0.00
Thoc5	chr11	4799846	4799907	-2.86	-0.63
Psme4	chr11	30671774	30672135	-0.53	-0.17
Slu7	chr11	43247232	43251629	-0.59	0.07
Slu7	chr11	43258883	43259812	-2.51	-0.21
Med7	chr11	46250448	46250688	-0.94	-0.27
Ppp2ca	chr11	51935449	51935836	-0.93	0.05
Aff4	chr11	53164268	53186578	-2.63	-0.12
Aff4	chr11	53225500	53229162	-0.71	0.07
Cnot8	chr11	57930958	57931228	-0.69	-0.18
Zkscan6	chr11	65636352	65642412	-0.30	0.00
Trp53	chr11	69393860	69394608	-1.25	-0.24
Suz12	chr11	79843530	79845978	-3.16	0.17
Psmd11	chr11	80285574	80285635	-1.26	-0.32
Taf15	chr11	83286609	83286700	-0.52	-0.08
Taf15	chr11	83318239	83318300	-0.54	0.18
Ppm1d	chr11	85124755	85125383	-3.11	-0.49
Ppm1d	chr11	85140408	85140529	-0.38	0.08
Supt4a	chr11	87557029	87557119	-0.49	0.20
Srsf1	chr11	87861172	87861322	-3.40	-0.49
Cdk12	chr11	98064618	98065548	-1.86	0.10
Cdk12	chr11	98071707	98072546	-1.51	0.09
Cdk12	chr11	98104823	98107067	-2.98	0.25
Psmd3	chr11	98556993	98557203	-2.02	-0.21
Thra	chr11	98603186	98617477	-0.48	0.09
Thra	chr11	98625080	98626348	-0.46	0.05
Thra	chr11	98626407	98629662	-3.23	0.78
Casc3	chr11	98683710	98689970	-1.75	-0.13
Rara	chr11	98799009	98808938	-0.63	0.23
Rara	chr11	98808997	98822389	-1.01	-0.17
Psme3	chr11	101177564	101178336	-0.77	0.09
Psme3	chr11	101182100	101183258	-2.86	-0.26
Tnrc6c	chr11	117621813	117622474	-0.67	-0.13
Tnrc6c	chr11	117623163	117623793	-2.52	0.53
Cbx2	chr11	118884462	118890969	-0.81	0.14
Rptor	chr11	119464338	119464399	-0.33	0.10
Rptor	chr11	119759737	119760755	-0.62	-0.06
Hus1	chr11	8896711	8896861	-2.14	0.69
Lsm11	chr11	45747027	45747476	-0.85	-0.20
Maml1	chr11	50069912	50070450	-1.28	0.23
Maml1	chr11	50071106	50074568	-0.75	0.08
Maml1	chr11	50076832	50105838	-0.45	0.03

Zfp354c	chr11	50628213	50628304	-0.22	0.78
Rad50	chr11	53463650	53465769	-0.89	-0.12
Zkscan17	chr11	59299022	59299412	-2.57	0.35
Zkscan17	chr11	59300338	59306167	-3.63	-0.20
Zkscan17	chr11	59319692	59319933	-0.44	-0.13
Top3a	chr11	60553560	60555499	-0.76	-0.16
Top3a	chr11	60555827	60557475	-2.70	-0.42
Ncor1	chr11	62147216	62147367	-0.76	0.12
Ncor1	chr11	62182894	62186997	-0.89	-0.06
Ncor1	chr11	62196726	62198338	-2.00	-0.18
Zfp287	chr11	62527173	62527774	-0.67	0.24
Zfp287	chr11	62528133	62528373	-0.69	0.38
Zfp287	chr11	62528582	62528763	-0.92	0.76
Zfp287	chr11	62541813	62542474	-2.41	0.87
Rpa1	chr11	75153888	75161885	-1.45	-0.24
Supt6	chr11	78047070	78059205	-0.45	-0.04
Brip1	chr11	85874744	85878314	-0.63	-0.10
Med13	chr11	86084314	86084555	-1.19	0.21
Med13	chr11	86100031	86100272	-1.64	0.21
Med13	chr11	86104608	86114187	-0.85	-0.06
Med13	chr11	86132926	86141950	-0.38	0.01
Pcgf2	chr11	97551180	97551629	-0.96	-0.18
Pcgf2	chr11	97553081	97553393	-0.41	0.07
Pip4k2b	chr11	97578204	97584269	-2.59	-0.11
Med1	chr11	98014158	98015054	-0.30	0.00
Med1	chr11	98015143	98015684	-1.90	0.21
Med1	chr11	98016282	98017363	-1.46	0.10
Med1	chr11	98017662	98017873	-0.56	-0.08
Med1	chr11	98018052	98018173	-0.35	0.03
Med24	chr11	98590278	98590655	-0.71	-0.34
Nr1d1	chr11	98629308	98629667	-1.72	0.43
Nr1d1	chr11	98631693	98632540	-0.68	-0.11
Nr1d1	chr11	98633166	98636556	-0.43	0.04
Kat2a	chr11	100573480	100573601	-0.54	-0.16
Cavin1	chr11	100819819	100820120	-0.23	-0.05
Cavin1	chr11	100820569	100824165	-0.38	1.32
Brca1	chr11	101384754	101385384	-1.00	0.21
Brca1	chr11	101385443	101386611	-0.56	0.06
Brca1	chr11	101386909	101387689	-2.16	0.30
Ubtf	chr11	102178946	102180320	-0.70	-0.29
Smarcd2	chr11	106133897	106134286	-1.61	-0.41
Smurf2	chr11	106683743	106684358	-1.06	-0.24
Smurf2	chr11	106781579	106781849	-2.81	-0.99

Cbx4	chr11	118941759	118947461	-1.94	0.14
Dnmt3a	chr12	3806582	3866198	-1.98	-0.23
Dnmt3a	chr12	3906804	3908006	-2.27	-0.13
E2f6	chr12	16817770	16817920	-1.01	-0.50
E2f6	chr12	16832231	16832859	-1.53	-0.19
Twistnb	chr12	34121850	34123173	-2.18	0.15
Psma6	chr12	56499811	56499900	-1.08	-0.18
Ppm1a	chr12	73862227	73885470	-1.52	-0.08
Snapc1	chr12	75065516	75068947	-1.48	-0.34
Psmc1	chr12	101348377	101351316	-0.70	-0.11
Cpsf2	chr12	103214183	103214333	-1.55	0.42
Cpsf2	chr12	103228209	103228270	-1.83	0.39
Cpsf2	chr12	103240910	103242024	-2.01	0.13
Papola	chr12	107022971	107023122	-1.31	-0.13
Papola	chr12	107072945	107075506	-0.44	-0.02
Ccnk	chr12	109418007	109424620	-0.57	0.10
Zfp839	chr12	112088488	112099099	-0.78	-0.14
Zfp839	chr12	112106479	112107344	-1.45	-0.27
Ywhaq	chr12	21422717	21423076	-0.98	-0.15
Max	chr12	78038822	78039718	-1.87	-0.08
Max	chr12	78063025	78063205	-3.17	-0.43
Snw1	chr12	88791126	88791810	-2.89	-0.19
Gtf2a1	chr12	92797800	92806028	-2.10	0.15
Dicer1	chr12	105929639	105930368	-2.10	0.29
Dicer1	chr12	105932689	105935476	-0.73	0.05
Ppp1r13b	chr12	113066968	113067568	-0.50	0.08
Ppp1r13b	chr12	113070791	113073499	-0.42	-0.03
Ppp1r13b	chr12	113146145	113146266	-0.59	1.21
Brf1	chr12	114198252	114199483	-1.02	-0.14
Brf1	chr12	114200643	114200824	-0.66	0.14
Zkscan4	chr13	21576234	21576865	-0.23	-0.05
Hist1h2bg	chr13	23663268	23663474	-3.01	-0.19
Hist1h3d	chr13	23667608	23667786	-1.85	-0.32
Hist1h2bc	chr13	23776067	23776213	-0.42	-0.04
Prelid1	chr13	55423415	55423591	-2.87	-0.24
Rmi1	chr13	58503698	58503988	-1.45	-0.24
Rmi1	chr13	58508365	58511252	-0.33	-0.01
Zfp369	chr13	65397640	65398417	-0.30	0.01
Med10	chr13	69954496	69954853	-1.60	-0.13
Ell2	chr13	75844961	75845171	-1.61	-0.54
Taf9	chr13	101435858	101436128	-3.16	0.35
Slc38a9	chr13	113528179	113528690	-3.31	1.16
Cdk13	chr13	17809653	17811597	-3.39	0.17

Cdk13	chr13	17858328	17896931	-2.82	-0.14
Zkscan3	chr13	21479290	21480454	-1.16	0.12
Zkscan3	chr13	21488062	21494295	-2.56	-0.43
Zkscan8	chr13	21611911	21613722	-1.42	-0.21
Zkscan8	chr13	21617156	21618818	-1.05	0.27
Hist1h3i	chr13	21875180	21875266	-1.25	-0.25
Hist1h3c	chr13	23837329	23837390	-0.45	-0.10
Nup153	chr13	46776258	46776499	-0.69	-0.10
Nup153	chr13	46782010	46792068	-2.74	0.10
Dek	chr13	47201073	47201589	-1.38	0.23
Thoc3	chr13	54561240	54561569	-1.64	-0.23
Ctsl	chr13	64470393	64471614	-1.97	-0.09
Zfp729a	chr13	67722397	67722577	-0.51	0.13
Zfp738	chr13	67770082	67770412	-1.52	0.55
Zfp738	chr13	67771278	67771818	-1.27	0.47
Ice1	chr13	70741154	70747297	-0.57	0.02
Ice1	chr13	70747386	70749417	-0.98	0.16
Nr2f1	chr13	78328775	78329046	-0.77	-2.34
Polr3g	chr13	81814362	81815082	-2.64	0.47
Jmy	chr13	94204723	94212614	-2.91	0.23
Jmy	chr13	94242583	94269644	-0.74	-0.07
Bdp1	chr13	100830436	100834338	-2.40	0.13
Bdp1	chr13	100873605	100873965	-0.90	-0.14
Rad17	chr13	101387477	101389058	-1.90	-0.31
Ercc6	chr14	33326706	33332187	-1.23	-0.18
Psmc6	chr14	45949498	45950514	-0.58	0.08
Ints9	chr14	65658302	65658483	-0.76	-0.21
Med4	chr14	73909855	73911003	-0.58	-0.16
Med4	chr14	73917729	73918293	-1.55	-0.20
Elf1	chr14	79881389	79936053	-0.32	-0.02
Elf1	chr14	79973114	79980817	-1.47	-0.10
Rarb	chr14	17264515	17264636	-0.46	-0.29
Rarb	chr14	17407447	17451267	-0.56	0.29
Nr1d2	chr14	19044403	19047125	-2.39	0.77
Nr1d2	chr14	19047304	19049180	-1.70	0.18
Polr3a	chr14	25267945	25269986	-1.03	-0.18
Supt16	chr14	52803669	52816914	-2.67	-0.36
Prmt5	chr14	55126018	55126553	-1.21	-0.15
Rabggta	chr14	56340490	56340807	-2.27	0.59
Cenpj	chr14	57145657	57148592	-0.36	0.08
Cenpj	chr14	57170788	57171625	-1.48	0.34
Cenpj	chr14	57171774	57172312	-0.36	0.05
Polr3d	chr14	70842783	70843023	-0.52	-0.09

Gtf2f2	chr14	76297012	76306651	-0.54	-0.05
Prkaa1	chr15	5093860	5110677	-1.10	-0.23
Prkaa1	chr15	5128795	5129184	-0.60	-0.09
Rictor	chr15	6745928	6747254	-2.42	0.20
Polr2k	chr15	36103771	36103949	-0.31	-0.05
Zfp707	chr15	75804010	75804189	-0.95	0.66
Zfp707	chr15	75804871	75806149	-1.08	-0.19
Tnrc6b	chr15	80709323	80711723	-2.66	0.12
Tnrc6b	chr15	80759640	80760240	-0.96	0.17
Rbx1	chr15	81305941	81306090	-0.83	-0.28
Ep300	chr15	81416643	81438767	-1.05	0.06
Ep300	chr15	81479273	81481819	-0.51	0.02
L3mbtl2	chr15	81516741	81518556	-1.73	-0.15
Nup50	chr15	84757805	84765852	-1.08	-0.07
Nup50	chr15	84773033	84773303	-0.62	0.12
Arid2	chr15	96117982	96118043	-0.41	-0.14
Arid2	chr15	96199968	96201115	-1.06	0.08
Arid2	chr15	96201684	96209296	-1.71	0.10
Arid2	chr15	96223066	96232775	-2.27	0.36
Zfp740	chr15	102034138	102035505	-2.75	0.24
Zfp740	chr15	102039630	102043880	-0.82	0.06
Sp1	chr15	102238241	102256174	-1.81	0.06
Sp1	chr15	102261200	102261651	-3.30	-0.23
Skp2	chr15	9043329	9043540	-0.80	0.27
Skp2	chr15	9069207	9070167	-0.68	-0.14
Ywhaz	chr15	36723405	36723964	-0.95	0.41
Zfp706	chr15	36936977	36937157	-2.07	-0.55
Taf2	chr15	54847403	54848094	-0.81	-0.13
Taf2	chr15	54903436	54903617	-0.44	-0.08
Atad2	chr15	57928856	57930279	-1.16	0.12
Atad2	chr15	57966487	57966637	-1.54	-0.46
Ago2	chr15	72936518	72937656	-0.67	-0.02
Cbx6	chr15	79658159	79664703	-1.02	0.09
Polr3h	chr15	81718807	81718927	-0.22	-0.25
Polr3h	chr15	81719674	81745484	-0.49	0.09
Polr3h	chr15	81746470	81746920	-2.14	-0.30
Polr3h	chr15	81755685	81756135	-3.21	0.63
Poldip3	chr15	82958053	82959641	-2.39	-0.13
Poldip3	chr15	82968734	82968795	-0.70	0.11
Brd1	chr15	88543110	88543809	-2.54	0.27
Mapk11	chr15	88973270	88973361	-1.33	1.55
Mapk11	chr15	88973776	88973926	-0.54	0.57
Yaf2	chr15	93115848	93167336	-0.40	0.02

Vdr	chr15	97687093	97687333	-0.24	0.68
Zfp385a	chr15	103144534	103144595	-0.30	-0.02
Zfp385a	chr15	103144653	103144774	-0.37	-0.05
Zfp385a	chr15	103144892	103145221	-0.36	0.02
Zfp385a	chr15	103150806	103170487	-0.85	0.15
Zfp263	chr16	3744098	3745116	-0.66	-0.07
Zfp263	chr16	3748158	3750040	-0.43	0.02
Rrn3	chr16	13813314	13813525	-1.79	-0.30
Psmd2	chr16	20651724	20652324	-3.25	-0.17
Ncbp2	chr16	31957240	31957450	-2.58	-0.37
Fyttd1	chr16	32877869	32884216	-0.32	-0.02
Fyttd1	chr16	32902586	32905812	-0.49	-0.07
Zfp597	chr16	3872223	3872374	-0.32	1.03
Crebbp	chr16	4154961	4213404	-1.70	-0.10
Gtf2e1	chr16	37510570	37510961	-2.88	-0.45
Gtf2e1	chr16	37511589	37522849	-0.57	0.08
Gtf2e1	chr16	37523028	37539825	-0.41	0.04
Arid1b	chr17	5332607	5339263	-2.33	-0.16
Zfp160	chr17	21178540	21179198	-2.36	1.43
Rnps1	chr17	24551619	24552074	-1.68	-0.36
Ppard	chr17	28369728	28432453	-1.72	-0.27
Mapk14	chr17	28828286	28828675	-2.21	-0.37
Brpf3	chr17	28938130	28938251	-0.19	0.10
Brpf3	chr17	28954078	28960704	-1.08	0.19
Brpf3	chr17	28973638	28973848	-0.68	0.35
Psmb8	chr17	34335288	34336214	-1.01	-0.16
Ehmt2	chr17	35042089	35042587	-2.96	-0.29
Ehmt2	chr17	35049712	35050992	-3.05	-0.21
Ddx39b	chr17	35378690	35378917	-2.25	-0.37
Ddx39b	chr17	35386162	35386790	-0.42	-0.02
Ddx39b	chr17	35566011	35566729	-0.95	0.28
Kat2b	chr17	53706385	53706446	-0.22	0.15
Zfp213	chr17	23694268	23694714	-1.32	-0.70
Zfp213	chr17	23694921	23696378	-0.39	0.24
Thoc6	chr17	23810618	23810831	-0.44	0.09
Pdpk1	chr17	24215583	24216033	-2.93	0.32
Ube2i	chr17	25397995	25398776	-0.81	-0.11
Stub1	chr17	25967580	25967909	-2.17	-0.16
Stub1	chr17	25969947	25970306	-0.38	0.08
Taf11	chr17	28038342	28038672	-1.67	-0.25
Taf11	chr17	28042348	28045158	-0.73	0.11
Ring1	chr17	34158155	34160310	-1.41	-0.14
Ring1	chr17	34160369	34161265	-0.31	-0.04

Brd2	chr17	34259120	34259211	-1.42	0.77
Runx2	chr17	44744988	44745109	-0.62	0.24
Runx2	chr17	44872609	44873298	-2.06	-0.37
Taf8	chr17	47625297	47626945	-1.85	-0.17
Taf8	chr17	47638265	47639236	-2.04	-0.48
Gtf2f1	chr17	57143401	57143559	-0.53	-0.10
Gtf2f1	chr17	57143961	57144203	-0.51	-0.06
Tgif1	chr17	71199117	71202842	-1.83	-0.13
Srsf7	chr17	80606178	80606645	-0.76	-0.19
Thoc1	chr18	9993386	9993507	-0.34	0.02
Rbbp8	chr18	11880308	11881165	-0.34	-0.01
Taf4b	chr18	14965941	14971957	-1.59	0.33
Polr2d	chr18	31955847	31956355	-3.30	0.43
Wdr33	chr18	32067351	32067412	-0.44	0.52
Iws1	chr18	32227387	32243000	-1.99	-0.09
Ercc3	chr18	32399984	32400372	-0.36	0.04
Prelid3a	chr18	67639251	67639430	-1.04	0.59
Rnmt	chr18	68460038	68465632	-1.16	0.13
Smad7	chr18	75527018	75527734	-0.56	0.14
Smad7	chr18	75528210	75530550	-2.26	0.57
Smad2	chr18	76422187	76422338	-0.39	0.04
Smad2	chr18	76462139	76464727	-0.86	-0.12
Epc1	chr18	6436991	6440114	-0.66	0.09
Epc1	chr18	6448973	6450113	-2.33	0.40
Epc1	chr18	6490659	6515906	-1.33	-0.35
Kctd1	chr18	15220309	15220877	-0.44	0.38
Kctd1	chr18	15221623	15221804	-0.15	-0.06
Kctd1	chr18	15221952	15222043	-0.14	0.32
Nr3c1	chr18	39588392	39649457	-3.45	0.21
Smad4	chr18	73799858	73800008	-0.66	-0.12
Smad4	chr18	73863185	73863395	-3.16	-1.68
Ctdp1	chr18	80605356	80630912	-3.52	0.36
Ctdp1	chr18	80642798	80647906	-3.16	-0.17
Men1	chr19	6335008	6336781	-2.54	-0.27
Men1	chr19	6338873	6340864	-1.37	-0.10
Ints5	chr19	8967536	8971662	-1.15	-0.08
Ints5	chr19	8971870	8972350	-0.64	-0.09
Smarca2	chr19	26705264	26705505	-0.28	0.00
Cstf2t	chr19	31157419	31159177	-0.63	-0.03
Pcgf5	chr19	36484266	36486670	-2.56	0.48
Pcgf5	chr19	36519495	36530396	-0.85	-0.17
Taf5	chr19	47142775	47149532	-1.43	0.33
Taf5	chr19	47156787	47157401	-0.49	0.09

Rad9a	chr19	4195437	4196286	-0.91	-0.11
Rbm14	chr19	4743957	4744526	-1.85	-0.59
Kat5	chr19	5603013	5603692	-1.67	-0.15
Kat5	chr19	5609321	5609621	-0.39	-0.05
Kat5	chr19	5609680	5610034	-0.36	-0.10
Prdx5	chr19	6983955	6984135	-1.81	-0.18
Esrra	chr19	6996237	6996298	-0.31	0.14
Polr2g	chr19	8872986	8873047	-0.27	-0.01
Ldb1	chr19	46107090	46108545	-0.69	0.04
Ldb1	chr19	46119462	46119522	-0.53	-0.37
Pcgf6	chr19	47108916	47109007	-0.64	-0.23
Med12	chrX	98488039	98488190	-2.90	0.51
Taf1	chrX	98794675	98794915	-0.42	0.05
Cstf2	chrX	130609104	130609225	-1.51	0.37
Rragb	chrX	149574589	149575911	-0.52	0.09
Suv39h1	chrX	7647858	7649530	-0.54	0.06
G6pdx	chrX	71655299	71655479	-0.51	-0.03
Rad9b	chr5	122803966	122804026	-0.22	0.19

RNA polymerase II transcription

gene_name	chr	chromStart	chromEnd	diff.lg.p	diff.log2.fc
Cnot11	chr1	39592727	39593027	-0.49	-0.09
Cnot11	chr1	39603302	39603662	-1.63	-0.30
Cnot9	chr1	74575438	74575798	-2.06	-0.28
Psmd1	chr1	87961193	87961313	-0.67	0.21
Ing5	chr1	95714871	95715861	-2.79	-0.31
Ccnt2	chr1	129670740	129670861	-1.38	0.49
Ccnt2	chr1	129698762	129700591	-0.54	0.03
Rbbp5	chr1	134394590	134400982	-1.17	0.15
Rbbp5	chr1	134401131	134401312	-0.34	-0.03
Kdm5b	chr1	136514466	136514617	-0.68	0.28
Kdm5b	chr1	136521310	136521760	-2.33	0.45
Kdm5b	chr1	136525288	136527418	-0.37	-0.03
Kdm5b	chr1	136527777	136528227	-1.23	0.27
Nr1i3	chr1	173148035	173148485	-1.67	-0.41
Nr1i3	chr1	173148844	173149234	-0.72	-0.23
Exo1	chr1	177829710	177831526	-1.63	-0.39
Exo1	chr1	177838863	177839044	-0.20	0.03
Exo1	chr1	177839162	177839433	-0.27	0.29
Parp1	chr1	182499105	182503843	-1.60	-0.27
Trp53bp2	chr1	184371133	184371894	-0.41	-0.09
Trp53bp2	chr1	184376642	184379234	-0.81	0.12
Trp53bp2	chr1	184389061	184392388	-0.44	-0.06
Ints7	chr1	193443646	193445390	-0.54	-0.07

Gls	chr1	52244565	52244656	-2.44	0.47
Sumo1	chr1	59696455	59696989	-1.11	-0.09
Bard1	chr1	71121331	71122020	-0.53	0.08
Zc3h11a	chr1	135516447	135517433	-0.47	-0.02
Zc3h11a	chr1	135517641	135518733	-1.03	0.06
Zc3h11a	chr1	135521310	135523811	-2.56	0.12
Btg2	chr1	135971471	135971532	-0.90	0.83
Btg2	chr1	135973595	135975732	-0.89	-0.05
Rnf2	chr1	153318408	153318528	-0.89	0.28
Pou2f1	chr1	167805604	167806174	-0.35	0.03
Smyd2	chr1	191704578	191706000	-1.27	0.17
Bmi1	chr2	18605667	18606468	-0.89	0.08
Rxra	chr2	27597444	27604173	-0.47	-0.11
Rxra	chr2	27617342	27618510	-1.22	-0.15
Tsc1	chr2	28531258	28532060	-2.14	0.31
Tsc1	chr2	28541313	28542943	-1.55	-0.18
Tsc1	chr2	28546177	28546268	-0.26	0.00
Abl1	chr2	31544075	31551320	-0.40	0.02
Abl1	chr2	31652573	31658159	-1.65	-0.10
Abl1	chr2	31658367	31658428	-1.12	-0.61
Abl1	chr2	31659237	31659477	-0.56	0.58
Mapkap1	chr2	34479093	34479423	-1.30	0.22
Psmc3	chr2	90894439	90895182	-1.38	-0.10
Cstf3	chr2	104504891	104505372	-2.37	0.28
Rtf1	chr2	119558206	119559195	-0.92	-0.10
Rtf1	chr2	119560243	119560484	-1.04	0.33
Mga	chr2	119698286	119698347	-0.18	0.00
Mga	chr2	119767083	119771919	-0.49	0.05
Mga	chr2	119787692	119790793	-2.58	-0.17
Itch	chr2	154959216	154959307	-0.25	0.00
Itch	chr2	155050132	155050373	-0.80	-0.16
Phf20	chr2	156092990	156093201	-1.93	0.33
Phf20	chr2	156113573	156113634	-1.16	0.44
Tgif2	chr2	156679040	156679219	-0.58	0.85
Tgif2	chr2	156679307	156679813	-0.75	-0.28
Src	chr2	157295226	157296471	-0.85	0.32
Rprd1b	chr2	157900721	157901141	-1.50	-0.24
Trp53rkb	chr2	166619352	166619563	-0.37	0.13
Trp53rkb	chr2	166620915	166621755	-2.59	-0.52
Cstf1	chr2	172201351	172201608	-2.58	0.38
Gata3	chr2	9779567	9784722	-1.35	0.40
Gata3	chr2	9784840	9796310	-0.60	0.24
Taf3	chr2	9837317	9964072	-0.79	0.05

Pip4k2a	chr2	18765497	18769115	-1.74	0.11
Ehmt1	chr2	24646742	24647852	-1.08	-0.13
Ehmt1	chr2	24733063	24775080	-2.58	0.23
Snapc4	chr2	26218284	26222767	-0.95	-0.09
Snapc4	chr2	26223896	26225464	-1.39	0.24
Notch1	chr2	26314049	26314319	-1.37	1.42
Notch1	chr2	26315036	26315097	-0.53	-1.83
Notch1	chr2	26315275	26315486	-0.62	0.91
Notch1	chr2	26316053	26316413	-0.28	0.10
Notch1	chr2	26323937	26324117	-0.52	0.80
Notch1	chr2	26336689	26337202	-0.49	1.03
Notch1	chr2	26339865	26340015	-0.40	0.61
Psmd5	chr2	34707993	34709794	-1.37	-0.17
Psmd5	chr2	34722532	34726482	-1.60	-0.40
Psmb7	chr2	38498946	38499426	-1.35	-0.19
Nr6a1	chr2	38781663	38781753	-0.49	-0.29
Nr4a2	chr2	56960175	56961102	-1.17	-0.25
Nr4a2	chr2	56964099	56965537	-0.49	-0.07
Nr4a2	chr2	56967204	56967355	-0.52	0.68
Clp1	chr2	84563548	84566299	-2.19	-0.20
Zfp661	chr2	127402341	127403565	-2.72	0.50
Snrpb	chr2	130002885	130005100	-2.05	-0.18
Pcna	chr2	132078555	132078916	-3.65	-0.19
Psmf1	chr2	151543111	151543232	-0.83	-0.47
Psmf1	chr2	151543649	151544068	-0.81	-0.19
Zfp334	chr2	165203294	165203715	-1.00	0.46
Zfp334	chr2	165205211	165206201	-0.49	0.09
Zfp334	chr2	165206829	165207339	-2.14	0.67
Trp53rka	chr2	165315878	165318814	-0.78	-0.11
Taf4	chr2	179647358	179656708	-0.54	-0.05
Skil	chr3	30994042	30997382	-1.57	0.11
Gatad2b	chr3	90127990	90145879	-0.41	-0.02
Gatad2b	chr3	90155787	90155878	-3.11	-0.35
Rorc	chr3	94201354	94201475	-0.30	1.58
Hist2h2be	chr3	96025043	96025490	-1.83	0.52
Hist2h2be	chr3	96026738	96027215	-1.30	-0.39
Rbm8a	chr3	96433850	96433910	-0.37	0.11
Notch2	chr3	97935343	97941284	-2.25	-0.13
Trim33	chr3	103148255	103148495	-1.27	0.15
Trim33	chr3	103156073	103158203	-1.33	0.10
Trim33	chr3	103158830	103159400	-0.30	0.00
Ints12	chr3	132771889	132772487	-0.74	-0.08
Ube2d3	chr3	135101656	135102228	-3.24	-0.27

Gtf2b	chr3	142428210	142434431	-1.34	-0.30
Gtf2b	chr3	142446182	142446450	-0.48	0.05
Phc3	chr3	30805887	30813151	-0.57	-0.08
Phc3	chr3	30864703	30868337	-0.42	0.12
Elf2	chr3	51059646	51060809	-1.08	-0.08
Elf2	chr3	51061575	51064964	-0.90	-0.11
Maml3	chr3	51491863	51496200	-1.51	0.17
Maml3	chr3	51501603	51600338	-0.59	-0.42
Maml3	chr3	51907609	51907880	-3.24	0.71
Maml3	chr3	51907939	51908209	-2.93	0.70
Maml3	chr3	51908358	51908838	-1.96	0.46
Lamtor2	chr3	88353798	88356849	-1.88	-0.11
Ints3	chr3	90195369	90196223	-1.47	-0.15
Ints3	chr3	90237228	90237558	-1.87	-0.39
Chtop	chr3	90303653	90304012	-2.00	-0.11
Chtop	chr3	90313185	90313365	-2.41	-0.21
Psmd4	chr3	94836626	94836924	-1.79	-0.12
Psmd4	chr3	94839816	94846467	-2.16	-0.13
Rprd2	chr3	95567509	95570116	-2.33	-0.11
Rprd2	chr3	95576734	95577124	-2.87	0.55
Hist2h3c2	chr3	96042454	96042544	-0.57	0.69
Hist2h2aa1	chr3	96044071	96044216	-1.04	1.17
Prmt6	chr3	110050616	110051517	-1.67	-0.58
Prmt6	chr3	110052356	110053916	-3.07	-0.18
Srsf11	chr3	157674772	157675838	-1.15	-0.06
Rngtt	chr4	33587224	33587763	-1.62	0.30
Rngtt	chr4	33588270	33588390	-0.93	0.89
Nr4a3	chr4	48058191	48058281	-0.24	-0.14
Nr4a3	chr4	48061990	48065027	-1.94	-0.50
Nr4a3	chr4	48083931	48096224	-1.06	-0.81
Zfp189	chr4	49542890	49543460	-2.62	0.62
Rraga	chr4	86221606	86222293	-0.53	0.03
Tal1	chr4	114737352	114741762	-1.59	-0.29
Tal1	chr4	114741970	114742061	-0.45	-0.22
Med8	chr4	118087440	118087618	-2.77	0.38
Scmh1	chr4	120201875	120202085	-2.07	0.58
Rragc	chr4	123597654	123598314	-1.34	0.21
Rragc	chr4	123612802	123613821	-0.41	-0.01
Rragc	chr4	123614089	123614180	-1.02	0.20
Lsm10	chr4	125773896	125775828	-1.43	-0.17
Phc2	chr4	128404910	128405061	-1.05	-0.71
Rpa2	chr4	132333796	132334035	-1.34	-0.32
Nr0b2	chr4	133112127	133112363	-0.61	0.57

Zfp46	chr4	135846237	135848631	-0.51	-0.05
Mtor	chr4	147822690	147826440	-1.12	-0.33
Mtor	chr4	147930392	147931764	-0.80	0.10
Ssu72	chr4	155078923	155079163	-2.43	1.17
Ssu72	chr4	155079462	155089762	-0.81	-0.07
Mllt3	chr4	87419218	87419819	-0.46	0.07
Mllt3	chr4	87486705	87487246	-0.69	0.10
Zfp691	chr4	118842660	118845711	-0.35	0.03
Zfp69	chr4	120602801	120603909	-2.55	0.49
Sfn	chr4	133156530	133158083	-0.66	0.16
Srrm1	chr4	134878175	134878385	-0.88	-0.09
Srrm1	chr4	134902739	134909069	-1.22	0.14
Eloa	chr4	135561252	135561580	-1.14	0.19
Eloa	chr4	135565701	135577564	-2.32	0.10
Ski	chr4	154529765	154533533	-2.17	0.10
Ski	chr4	154533945	154596644	-1.11	-0.08
Cdk6	chr5	3519406	3520884	-0.47	0.04
Kmt2e	chr5	22940246	22940516	-2.82	0.51
Kmt2e	chr5	23002909	23005273	-0.76	0.07
Kmt2e	chr5	23007381	23008999	-1.16	0.08
Ywhah	chr5	33361494	33361614	-0.40	0.10
Ywhah	chr5	33369269	33370047	-0.64	0.02
Rbpj	chr5	54044845	54045146	-0.26	0.54
Zfp605	chr5	110556631	110557140	-1.63	0.56
Zfp605	chr5	110557318	110558156	-0.32	-0.03
Triap1	chr5	115791255	115793471	-0.71	-0.07
Rnf34	chr5	123273869	123314504	-1.72	0.21
Psmd9	chr5	123678228	123684616	-1.07	0.18
Kmt5a	chr5	124911176	124911327	-0.89	-0.12
Gtf2h3	chr5	125046222	125046313	-0.93	-0.48
Zfp664	chr5	125343194	125366362	-1.24	-0.09
Zfp664	chr5	125366721	125367081	-0.31	0.01
Rfc2	chr5	135073840	135074198	-0.38	-0.03
Zkscan1	chr5	138526311	138534046	-0.73	0.23
Zkscan1	chr5	138541914	138542485	-0.51	0.05
Zkscan1	chr5	138542723	138543984	-1.79	0.16
Zkscan1	chr5	138544222	138545962	-0.77	-0.07
Zfp12	chr5	144005811	144007881	-0.65	-0.08
Zfp12	chr5	144007940	144008390	-0.69	-0.23
Zkscan5	chr5	145981691	145982470	-2.39	0.63
Zfp655	chr5	145992583	145994463	-2.43	-0.45
Zfp655	chr5	146004460	146006138	-2.25	0.12
Cdk8	chr5	147042885	147071118	-2.04	-0.41

Smarcd3	chr5	24100691	24101019	-0.21	0.00
Prkag2	chr5	24605830	24606400	-1.25	0.18
Kmt2c	chr5	24790108	24790979	-0.87	0.09
Slbp	chr5	33992485	33994955	-0.80	0.14
Prkab1	chr5	116463747	116468029	-0.50	-0.04
Prkab1	chr5	116474109	116474378	-0.66	-0.12
Ptpn11	chr5	121581827	121582307	-2.13	0.25
Ptpn11	chr5	121583292	121587519	-0.51	-0.03
Ncor2	chr5	125514394	125514836	-0.46	-0.06
Ncor2	chr5	125538274	125548306	-0.47	0.04
Ncor2	chr5	125659463	125659584	-0.26	-0.17
Zfp11	chr5	130162143	130162980	-1.02	0.38
Zfp11	chr5	130164055	130166739	-0.47	0.31
Zfp113	chr5	138585511	138586171	-1.24	-0.27
Zfp113	chr5	138586380	138586650	-1.50	0.58
Ints1	chr5	140251541	140251602	-0.37	0.17
Smurf1	chr5	145637693	145638023	-0.74	-0.29
Smurf1	chr5	145638352	145638682	-1.09	-0.34
Smurf1	chr5	145639310	145639491	-0.37	0.13
Zkscan14	chr5	145956021	145962515	-3.26	-0.45
Rfc3	chr5	152445398	152445668	-1.50	0.34
Zfp398	chr6	47785869	47790711	-0.90	0.31
Zfp398	chr6	47815946	47817117	-0.67	-0.11
Zfp282	chr6	47827703	47830376	-0.29	0.00
Zfp282	chr6	47854827	47855966	-3.17	-0.33
Zfp282	chr6	47856175	47856835	-1.42	-0.27
Zfp212	chr6	47870625	47876800	-0.60	0.18
Zfp212	chr6	47879122	47881565	-1.03	-0.20
Zfp775	chr6	48555624	48570026	-1.58	-0.60
Zfp775	chr6	48570354	48570923	-1.34	-0.72
Gata2	chr6	88154683	88155799	-3.13	-0.61
Nr2c2	chr6	92114256	92117902	-2.91	-0.32
Brpf1	chr6	113257160	113269353	-0.42	0.01
Chd4	chr6	125079888	125080399	-1.62	-0.07
Cdkn1b	chr6	134870898	134872390	-2.89	0.28
Gm8994	chr6	136278992	136279111	-0.18	0.47
Gm8994	chr6	136279792	136279971	-0.26	0.19
H2afj	chr6	136756828	136756889	-0.45	0.13
H2afj	chr6	136757307	136757577	-1.31	-0.28
Hipk2	chr6	38647868	38666025	-2.58	-0.11
Hipk2	chr6	38697991	38787047	-0.54	-0.02
Hipk2	chr6	38825859	38826040	-2.58	-0.39
Zfp786	chr6	47769651	47770337	-0.42	-0.30

Zfp786	chr6	47770485	47771170	-0.67	0.66
Zfp786	chr6	47771318	47771676	-0.36	-0.40
Zfp746	chr6	48013500	48016503	-1.38	-0.15
Zfp746	chr6	48033553	48036532	-0.55	-0.18
Gadd45a	chr6	66985178	66987371	-2.48	-0.22
Rybp	chr6	100182274	100183082	-1.03	0.42
Zfp248	chr6	118378764	118380402	-3.16	0.57
Phc1	chr6	122273438	122273618	-1.06	0.49
Phc1	chr6	122275560	122275830	-0.30	-0.03
Tigar	chr6	127037498	127038307	-0.73	0.09
Ccnd2	chr6	127079556	127080245	-1.36	-0.18
Rhno1	chr6	128307315	128309210	-1.80	-0.15
Kras	chr6	145168783	145169023	-0.29	0.00
Zfp667	chr7	6256313	6256524	-1.53	1.14
Zfp667	chr7	6256792	6256942	-0.28	-0.11
Zfp667	chr7	6257719	6257870	-0.76	0.76
Zfp78	chr7	6331042	6331103	-0.19	0.59
Zfp78	chr7	6331550	6332298	-1.80	0.98
Zfp28	chr7	6344966	6347349	-0.99	0.23
Zfp110	chr7	13434054	13434502	-0.38	-0.03
Zfp324	chr7	13555672	13556391	-2.57	-0.89
Zfp324	chr7	13556928	13557676	-0.54	-0.23
Zfp446	chr7	13563376	13563437	-0.26	-0.09
Zfp446	chr7	13564395	13564546	-0.27	-0.02
Zfp446	chr7	13567226	13568306	-0.59	-0.12
Zfp446	chr7	13568485	13569026	-1.73	-0.53
Ppp1r13l	chr7	19956867	19960636	-1.70	-0.27
Ercc2	chr7	19979441	19981041	-2.11	-0.19
Zfp180	chr7	24882912	24891576	-1.89	-0.12
Zfp112	chr7	24910123	24910454	-0.36	-0.31
Zfp112	chr7	24910962	24911533	-0.46	0.24
Zfp112	chr7	24912371	24912551	-1.18	1.63
Zfp235	chr7	24921520	24921581	-0.55	1.10
Zfp235	chr7	24925681	24925772	-0.67	0.72
Zfp235	chr7	24926760	24927241	-1.64	-0.63
Zfp235	chr7	24927899	24928200	-0.30	-0.12
Akt2	chr7	28422202	28423428	-1.06	0.15
Paf1	chr7	29178014	29178074	-0.35	0.09
Paf1	chr7	29181953	29184402	-2.55	-0.12
Zfp30	chr7	30577491	30577582	-2.02	2.09
Zfp30	chr7	30577790	30578810	-2.01	0.66
Zfp790	chr7	30613525	30616012	-0.65	-0.06
Zfp420	chr7	30645086	30661309	-0.62	-0.06

Zfp382	chr7	30916575	30919873	-2.17	-0.19
Polr2i	chr7	31017332	31017772	-0.29	0.00
Zfp658	chr7	50827988	50828378	-0.42	0.12
Zfp719	chr7	50835075	50835135	-1.05	0.74
Zfp719	chr7	50841731	50846748	-2.68	0.18
Zfp819	chr7	50873172	50873292	-0.50	3.17
Gtf2h1	chr7	54051493	54051554	-1.82	-0.48
Zfp710	chr7	87169699	87227267	-2.58	-0.11
Zfp710	chr7	87231444	87231625	-0.38	0.06
Zfp710	chr7	87233516	87236264	-1.55	0.11
Lamtor1	chr7	109054380	109058211	-1.32	-0.28
Ctr9	chr7	118197379	118199651	-2.59	0.24
Setd1a	chr7	134920932	134920993	-1.26	1.23
Setd1a	chr7	134927635	134930705	-0.77	0.04
Setd1a	chr7	134931750	134931998	-1.29	-0.20
Zik1	chr7	11075804	11075955	-0.39	-0.19
Zfp551	chr7	13000735	13000946	-0.79	1.17
Zfp94	chr7	25087170	25088874	-0.51	-0.11
Pou2f2	chr7	25901224	25917479	-3.35	1.22
Supt5	chr7	29116415	29116476	-0.45	0.06
Psmd8	chr7	29965393	29965573	-1.95	-0.13
Zfp940	chr7	30629168	30629499	-0.28	0.07
Zfp940	chr7	30629558	30629679	-0.68	0.98
Zfp940	chr7	30630277	30630518	-0.33	-0.19
Zfp940	chr7	30630847	30631058	-1.56	1.10
Zfp27	chr7	30678475	30680419	-0.34	0.01
Zfp27	chr7	30680747	30683850	-0.38	-0.03
Zfp14	chr7	30823226	30823406	-0.45	0.71
Zfp14	chr7	30823704	30823854	-0.93	1.66
Zfp14	chr7	30823972	30824152	-0.75	1.91
Zfp566	chr7	30862474	30863454	-2.54	0.50
Kmt2b	chr7	31368475	31373655	-3.36	-0.13
Ccne1	chr7	38883150	38883388	-1.47	-0.46
Zfp715	chr7	50552967	50556619	-3.08	0.17
Nr1h2	chr7	51806700	51806791	-0.86	0.19
Zfp473	chr7	51987480	51988021	-1.22	1.42
Zfp473	chr7	51988559	51988710	-0.18	-0.04
Zfp473	chr7	51989249	51989760	-1.53	1.19
Med25	chr7	52132313	52133597	-1.95	-0.23
Bax	chr7	52722059	52722238	-0.83	0.22
E2f8	chr7	56126083	56127247	-1.54	-0.22
Blm	chr7	87647232	87647711	-0.92	-0.15
Blm	chr7	87654407	87659523	-2.35	0.28

Eed	chr7	97125526	97129068	-0.79	-0.09
Eed	chr7	97129246	97129456	-2.79	0.48
Pcf11	chr7	99801691	99808521	-0.95	-0.05
Zfp747	chr7	134516408	134519504	-2.59	0.25
9130019O22Rik	chr7	134525922	134526311	-0.65	0.64
9130019O22Rik	chr7	134526817	134529144	-1.48	0.21
E430018J23Rik	chr7	134534349	134536898	-0.64	-0.15
E430018J23Rik	chr7	134536956	134537135	-0.28	-0.18
Zfp764	chr7	134547867	134549152	-2.70	-0.38
Zfp764	chr7	134550156	134550306	-0.31	-0.13
Zfp688	chr7	134562479	134563593	-0.33	-0.02
Zfp688	chr7	134565458	134565548	-0.42	-0.46
Zfp689	chr7	134587294	134588642	-1.03	-0.25
Zfp689	chr7	134592252	134592433	-0.97	0.50
Zfp668	chr7	135009321	135009472	-0.34	-0.12
Pidd1	chr7	148624413	148625004	-2.27	-0.57
Pidd1	chr7	148628991	148629907	-3.39	-1.06
Polr2l	chr7	148659279	148661052	-0.40	0.16
Ccnd1	chr7	152118441	152118622	-0.78	-0.05
Ccnd1	chr7	152125529	152125830	-0.84	0.07
Snapc2	chr8	4255341	4256190	-0.53	-0.07
Tfdp1	chr8	13377166	13377345	-1.90	-0.26
Tfdp1	chr8	13377582	13377702	-2.49	-0.30
Kat6a	chr8	24040729	24043035	-1.41	0.13
Kat6a	chr8	24046098	24051460	-0.39	-0.01
Ppp2cb	chr8	34710092	34710330	-0.74	-0.58
Gtf2e2	chr8	34886483	34887084	-2.84	0.31
Nr2c2ap	chr8	72655231	72655498	-2.58	0.60
Nr2c2ap	chr8	72657115	72657470	-1.15	-0.17
Ell	chr8	73063573	73102761	-1.80	0.39
Ell	chr8	73105671	73115739	-2.29	0.17
Sin3b	chr8	75247186	75248032	-0.43	0.05
Sin3b	chr8	75273726	75273996	-2.25	0.63
Sin3b	chr8	75280901	75281383	-2.17	-0.52
Nr3c2	chr8	79432284	79432583	-1.74	2.06
Nr3c2	chr8	79433507	79433836	-1.15	2.46
Polr2c	chr8	97387425	97387634	-0.99	-0.15
Cbfb	chr8	107694603	107694841	-1.27	-0.20
Zfp90	chr8	108948628	108949645	-2.85	0.58
Zfp1	chr8	114190002	114194792	-0.82	0.10
Banp	chr8	124474462	124474523	-0.29	0.19
Banp	chr8	124549891	124550071	-0.27	0.02
Banp	chr8	124550280	124550791	-1.64	0.85

Banp	chr8	124550969	124551120	-0.31	-0.26
Banp	chr8	124551299	124552918	-1.18	0.43
Zfpm1	chr8	124806220	124856010	-0.73	0.11
Zfpm1	chr8	124858912	124861147	-3.23	0.22
Ash2l	chr8	26950536	26958082	-3.54	-0.43
Wrn	chr8	34346284	34346854	-0.31	-0.02
Cnot7	chr8	41592761	41593001	-1.13	0.18
Ing2	chr8	48753850	48754631	-3.25	-0.30
Gatad2a	chr8	72459689	72459840	-1.48	-0.16
Gatad2a	chr8	72497999	72498150	-0.82	-0.30
Gatad2a	chr8	72519948	72520248	-0.90	-0.33
Med26	chr8	75019172	75072090	-1.92	-0.15
Psmb10	chr8	108461631	108461997	-1.25	-0.38
Dhx38	chr8	112071923	112073753	-2.72	0.29
Maml2	chr9	13510263	13511312	-0.79	0.20
Pin1	chr9	20456573	20459918	-2.86	-0.44
Smarca4	chr9	21505619	21507475	-1.36	0.14
Zfp202	chr9	40014929	40015928	-0.32	0.03
Zfp202	chr9	40018505	40020086	-3.02	0.31
Usp2	chr9	43883576	43884236	-2.87	2.50
Usp2	chr9	43902032	43902332	-0.41	-0.10
H2afx	chr9	44143446	44144156	-2.11	-0.11
Ppp2r1b	chr9	50687990	50688710	-0.88	-0.11
Ppp2r1b	chr9	50701016	50701705	-0.62	0.10
Psma4	chr9	54805658	54805748	-1.39	0.21
Sin3a	chr9	56973115	56975576	-0.47	0.04
Snapc5	chr9	64027103	64030261	-2.64	0.30
Ints14	chr9	64809641	64809731	-1.87	0.71
Ints14	chr9	64833706	64834423	-1.41	0.15
Rora	chr9	69226655	69226835	-0.29	0.06
Ice2	chr9	69263440	69263950	-2.00	0.32
Ice2	chr9	69280013	69280223	-0.38	0.10
Atr	chr9	95765519	95765729	-0.56	0.14
Zkscan7	chr9	122797636	122797935	-0.27	-0.02
Zkscan7	chr9	122803837	122804973	-0.35	0.05
Med17	chr9	15066624	15066715	-0.36	-0.07
Zfp558	chr9	18260716	18261406	-1.01	0.57
Zfp426	chr9	20273262	20273323	-0.75	2.21
Zfp426	chr9	20274940	20276201	-1.15	0.20
Zfp266	chr9	20304149	20306501	-1.23	-0.14
Kmt2a	chr9	44615605	44617182	-1.02	0.14
Kmt2a	chr9	44650695	44650756	-3.48	0.90
Atm	chr9	53247473	53248264	-1.08	0.14

Pml	chr9	58067532	58068881	-1.38	-0.21
Pml	chr9	58077807	58077868	-0.49	0.22
Pml	chr9	58080966	58094982	-3.67	-0.47
Pml	chr9	58097382	58097563	-0.75	-0.30
Smad3	chr9	63605680	63605801	-0.63	0.71
Smad3	chr9	63605729	63605891	-0.25	0.23
Rnf111	chr9	70274585	70277390	-1.13	-0.14
Tcf12	chr9	71693887	71694367	-2.25	0.19
Tcf12	chr9	71958831	71958922	-0.41	-0.08
Tcf12	chr9	71959385	71959626	-1.17	-0.25
Atrip	chr9	108960445	108961754	-0.40	-0.01
Atrip	chr9	108967670	108968495	-0.58	0.09
Cnot10	chr9	114541840	114549228	-0.65	0.12
Sgk1	chr10	21718603	21718843	-0.48	0.25
Sesn1	chr10	41530409	41607263	-1.97	-0.34
Sesn1	chr10	41627317	41627765	-1.28	-0.38
Dna2	chr10	62436065	62436156	-0.32	-0.08
Stk11	chr10	79579091	79588239	-2.17	0.17
Stk11	chr10	79592650	79593215	-0.48	-0.05
Txnrd1	chr10	82296755	82323149	-0.66	-0.19
E2f7	chr10	110216069	110216279	-0.34	0.10
Smarcc2	chr10	127920231	127921148	-2.34	0.28
Smarcc2	chr10	127924513	127924663	-1.10	-0.30
Smarcc2	chr10	127926093	127926303	-3.40	0.46
Ddit4	chr10	59412599	59412718	-2.71	0.89
Ddit4	chr10	59412895	59414518	-0.91	-0.09
Nrbf2	chr10	66729853	66730867	-0.88	0.10
Ube2d1	chr10	70718055	70718592	-2.86	0.34
Smarcb1	chr10	75359661	75359780	-2.54	-0.51
Polr2e	chr10	79498845	79498935	-0.48	-0.07
Polr2e	chr10	79501286	79502404	-0.85	-0.12
Tcf3	chr10	79872386	79873344	-0.38	0.02
Cradd	chr10	94637889	94638400	-0.76	0.21
Cradd	chr10	94785366	94786140	-0.40	0.11
Cnot2	chr10	115922216	115922606	-1.24	0.23
Cnot2	chr10	115922874	115923115	-0.75	0.16
Yeats4	chr10	116652435	116652884	-0.66	0.08
Mdm2	chr10	117126799	117129688	-0.41	0.01
Dyrk2	chr10	118296404	118305870	-2.04	-0.14
Pip4k2c	chr10	126635286	126635944	-0.82	0.09
Pip4k2c	chr10	126636701	126637297	-0.51	-0.06
Cdk2	chr10	128135891	128136071	-0.28	0.00
Thoc5	chr11	4799846	4799907	-2.86	-0.63

Psme4	chr11	30671774	30672135	-0.53	-0.17
Slu7	chr11	43247232	43251629	-0.59	0.07
Slu7	chr11	43258883	43259812	-2.51	-0.21
Med7	chr11	46250448	46250688	-0.94	-0.27
Ppp2ca	chr11	51935449	51935836	-0.93	0.05
Aff4	chr11	53164268	53186578	-2.63	-0.12
Aff4	chr11	53225500	53229162	-0.71	0.07
Cnot8	chr11	57930958	57931228	-0.69	-0.18
Zkscan6	chr11	65636352	65642412	-0.30	0.00
Trp53	chr11	69393860	69394608	-1.25	-0.24
Suz12	chr11	79843530	79845978	-3.16	0.17
Psmd11	chr11	80285574	80285635	-1.26	-0.32
Taf15	chr11	83286609	83286700	-0.52	-0.08
Taf15	chr11	83318239	83318300	-0.54	0.18
Ppm1d	chr11	85124755	85125383	-3.11	-0.49
Ppm1d	chr11	85140408	85140529	-0.38	0.08
Supt4a	chr11	87557029	87557119	-0.49	0.20
Srsf1	chr11	87861172	87861322	-3.40	-0.49
Cdk12	chr11	98064618	98065548	-1.86	0.10
Cdk12	chr11	98071707	98072546	-1.51	0.09
Cdk12	chr11	98104823	98107067	-2.98	0.25
Psmd3	chr11	98556993	98557203	-2.02	-0.21
Thra	chr11	98603186	98617477	-0.48	0.09
Thra	chr11	98625080	98626348	-0.46	0.05
Thra	chr11	98626407	98629662	-3.23	0.78
Casc3	chr11	98683710	98689970	-1.75	-0.13
Rara	chr11	98799009	98808938	-0.63	0.23
Rara	chr11	98808997	98822389	-1.01	-0.17
Psme3	chr11	101177564	101178336	-0.77	0.09
Psme3	chr11	101182100	101183258	-2.86	-0.26
Cbx2	chr11	118884462	118890969	-0.81	0.14
Rptor	chr11	119464338	119464399	-0.33	0.10
Rptor	chr11	119759737	119760755	-0.62	-0.06
Hus1	chr11	8896711	8896861	-2.14	0.69
Lsm11	chr11	45747027	45747476	-0.85	-0.20
Maml1	chr11	50069912	50070450	-1.28	0.23
Maml1	chr11	50071106	50074568	-0.75	0.08
Maml1	chr11	50076832	50105838	-0.45	0.03
Zfp354c	chr11	50628213	50628304	-0.22	0.78
Rad50	chr11	53463650	53465769	-0.89	-0.12
Zkscan17	chr11	59299022	59299412	-2.57	0.35
Zkscan17	chr11	59300338	59306167	-3.63	-0.20
Zkscan17	chr11	59319692	59319933	-0.44	-0.13

Top3a	chr11	60553560	60555499	-0.76	-0.16
Top3a	chr11	60555827	60557475	-2.70	-0.42
Ncor1	chr11	62147216	62147367	-0.76	0.12
Ncor1	chr11	62182894	62186997	-0.89	-0.06
Ncor1	chr11	62196726	62198338	-2.00	-0.18
Zfp287	chr11	62527173	62527774	-0.67	0.24
Zfp287	chr11	62528133	62528373	-0.69	0.38
Zfp287	chr11	62528582	62528763	-0.92	0.76
Zfp287	chr11	62541813	62542474	-2.41	0.87
Rpa1	chr11	75153888	75161885	-1.45	-0.24
Supt6	chr11	78047070	78059205	-0.45	-0.04
Brip1	chr11	85874744	85878314	-0.63	-0.10
Med13	chr11	86084314	86084555	-1.19	0.21
Med13	chr11	86100031	86100272	-1.64	0.21
Med13	chr11	86104608	86114187	-0.85	-0.06
Med13	chr11	86132926	86141950	-0.38	0.01
Pcgf2	chr11	97551180	97551629	-0.96	-0.18
Pcgf2	chr11	97553081	97553393	-0.41	0.07
Pip4k2b	chr11	97578204	97584269	-2.59	-0.11
Med1	chr11	98014158	98015054	-0.30	0.00
Med1	chr11	98015143	98015684	-1.90	0.21
Med1	chr11	98016282	98017363	-1.46	0.10
Med1	chr11	98017662	98017873	-0.56	-0.08
Med1	chr11	98018052	98018173	-0.35	0.03
Med24	chr11	98590278	98590655	-0.71	-0.34
Nr1d1	chr11	98629308	98629667	-1.72	0.43
Nr1d1	chr11	98631693	98632540	-0.68	-0.11
Nr1d1	chr11	98633166	98636556	-0.43	0.04
Kat2a	chr11	100573480	100573601	-0.54	-0.16
Brca1	chr11	101384754	101385384	-1.00	0.21
Brca1	chr11	101385443	101386611	-0.56	0.06
Brca1	chr11	101386909	101387689	-2.16	0.30
Smarcd2	chr11	106133897	106134286	-1.61	-0.41
Smurf2	chr11	106683743	106684358	-1.06	-0.24
Smurf2	chr11	106781579	106781849	-2.81	-0.99
Cbx4	chr11	118941759	118947461	-1.94	0.14
E2f6	chr12	16817770	16817920	-1.01	-0.50
E2f6	chr12	16832231	16832859	-1.53	-0.19
Psma6	chr12	56499811	56499900	-1.08	-0.18
Ppm1a	chr12	73862227	73885470	-1.52	-0.08
Snapc1	chr12	75065516	75068947	-1.48	-0.34
Psmc1	chr12	101348377	101351316	-0.70	-0.11
Cpsf2	chr12	103214183	103214333	-1.55	0.42

Cpsf2	chr12	103228209	103228270	-1.83	0.39
Cpsf2	chr12	103240910	103242024	-2.01	0.13
Papola	chr12	107022971	107023122	-1.31	-0.13
Papola	chr12	107072945	107075506	-0.44	-0.02
Ccnk	chr12	109418007	109424620	-0.57	0.10
Zfp839	chr12	112088488	112099099	-0.78	-0.14
Zfp839	chr12	112106479	112107344	-1.45	-0.27
Ywhaq	chr12	21422717	21423076	-0.98	-0.15
Max	chr12	78038822	78039718	-1.87	-0.08
Max	chr12	78063025	78063205	-3.17	-0.43
Snw1	chr12	88791126	88791810	-2.89	-0.19
Gtf2a1	chr12	92797800	92806028	-2.10	0.15
Ppp1r13b	chr12	113066968	113067568	-0.50	0.08
Ppp1r13b	chr12	113070791	113073499	-0.42	-0.03
Ppp1r13b	chr12	113146145	113146266	-0.59	1.21
Zkscan4	chr13	21576234	21576865	-0.23	-0.05
Hist1h2bg	chr13	23663268	23663474	-3.01	-0.19
Hist1h3d	chr13	23667608	23667786	-1.85	-0.32
Hist1h2bc	chr13	23776067	23776213	-0.42	-0.04
Prelid1	chr13	55423415	55423591	-2.87	-0.24
Rmi1	chr13	58503698	58503988	-1.45	-0.24
Rmi1	chr13	58508365	58511252	-0.33	-0.01
Zfp369	chr13	65397640	65398417	-0.30	0.01
Med10	chr13	69954496	69954853	-1.60	-0.13
Ell2	chr13	75844961	75845171	-1.61	-0.54
Taf9	chr13	101435858	101436128	-3.16	0.35
Slc38a9	chr13	113528179	113528690	-3.31	1.16
Cdk13	chr13	17809653	17811597	-3.39	0.17
Cdk13	chr13	17858328	17896931	-2.82	-0.14
Zkscan3	chr13	21479290	21480454	-1.16	0.12
Zkscan3	chr13	21488062	21494295	-2.56	-0.43
Zkscan8	chr13	21611911	21613722	-1.42	-0.21
Zkscan8	chr13	21617156	21618818	-1.05	0.27
Hist1h3i	chr13	21875180	21875266	-1.25	-0.25
Hist1h3c	chr13	23837329	23837390	-0.45	-0.10
Dek	chr13	47201073	47201589	-1.38	0.23
Thoc3	chr13	54561240	54561569	-1.64	-0.23
Ctsl	chr13	64470393	64471614	-1.97	-0.09
Zfp729a	chr13	67722397	67722577	-0.51	0.13
Zfp738	chr13	67770082	67770412	-1.52	0.55
Zfp738	chr13	67771278	67771818	-1.27	0.47
Ice1	chr13	70741154	70747297	-0.57	0.02
Ice1	chr13	70747386	70749417	-0.98	0.16

Nr2f1	chr13	78328775	78329046	-0.77	-2.34
Jmy	chr13	94204723	94212614	-2.91	0.23
Jmy	chr13	94242583	94269644	-0.74	-0.07
Rad17	chr13	101387477	101389058	-1.90	-0.31
Psmc6	chr14	45949498	45950514	-0.58	0.08
Ints9	chr14	65658302	65658483	-0.76	-0.21
Med4	chr14	73909855	73911003	-0.58	-0.16
Med4	chr14	73917729	73918293	-1.55	-0.20
Elf1	chr14	79881389	79936053	-0.32	-0.02
Elf1	chr14	79973114	79980817	-1.47	-0.10
Rarb	chr14	17264515	17264636	-0.46	-0.29
Rarb	chr14	17407447	17451267	-0.56	0.29
Nr1d2	chr14	19044403	19047125	-2.39	0.77
Nr1d2	chr14	19047304	19049180	-1.70	0.18
Supt16	chr14	52803669	52816914	-2.67	-0.36
Prmt5	chr14	55126018	55126553	-1.21	-0.15
Rabggta	chr14	56340490	56340807	-2.27	0.59
Cenpj	chr14	57145657	57148592	-0.36	0.08
Cenpj	chr14	57170788	57171625	-1.48	0.34
Cenpj	chr14	57171774	57172312	-0.36	0.05
Gtf2f2	chr14	76297012	76306651	-0.54	-0.05
Prkaa1	chr15	5093860	5110677	-1.10	-0.23
Prkaa1	chr15	5128795	5129184	-0.60	-0.09
Rictor	chr15	6745928	6747254	-2.42	0.20
Polr2k	chr15	36103771	36103949	-0.31	-0.05
Zfp707	chr15	75804010	75804189	-0.95	0.66
Zfp707	chr15	75804871	75806149	-1.08	-0.19
Rbx1	chr15	81305941	81306090	-0.83	-0.28
Ep300	chr15	81416643	81438767	-1.05	0.06
Ep300	chr15	81479273	81481819	-0.51	0.02
L3mbtl2	chr15	81516741	81518556	-1.73	-0.15
Arid2	chr15	96117982	96118043	-0.41	-0.14
Arid2	chr15	96199968	96201115	-1.06	0.08
Arid2	chr15	96201684	96209296	-1.71	0.10
Arid2	chr15	96223066	96232775	-2.27	0.36
Zfp740	chr15	102034138	102035505	-2.75	0.24
Zfp740	chr15	102039630	102043880	-0.82	0.06
Sp1	chr15	102238241	102256174	-1.81	0.06
Sp1	chr15	102261200	102261651	-3.30	-0.23
Skp2	chr15	9043329	9043540	-0.80	0.27
Skp2	chr15	9069207	9070167	-0.68	-0.14
Ywhaz	chr15	36723405	36723964	-0.95	0.41
Zfp706	chr15	36936977	36937157	-2.07	-0.55

Taf2	chr15	54847403	54848094	-0.81	-0.13
Taf2	chr15	54903436	54903617	-0.44	-0.08
Atad2	chr15	57928856	57930279	-1.16	0.12
Atad2	chr15	57966487	57966637	-1.54	-0.46
Cbx6	chr15	79658159	79664703	-1.02	0.09
Poldip3	chr15	82958053	82959641	-2.39	-0.13
Poldip3	chr15	82968734	82968795	-0.70	0.11
Brd1	chr15	88543110	88543809	-2.54	0.27
Mapk11	chr15	88973270	88973361	-1.33	1.55
Mapk11	chr15	88973776	88973926	-0.54	0.57
Yaf2	chr15	93115848	93167336	-0.40	0.02
Vdr	chr15	97687093	97687333	-0.24	0.68
Zfp385a	chr15	103144534	103144595	-0.30	-0.02
Zfp385a	chr15	103144653	103144774	-0.37	-0.05
Zfp385a	chr15	103144892	103145221	-0.36	0.02
Zfp385a	chr15	103150806	103170487	-0.85	0.15
Zfp263	chr16	3744098	3745116	-0.66	-0.07
Zfp263	chr16	3748158	3750040	-0.43	0.02
Psmd2	chr16	20651724	20652324	-3.25	-0.17
Ncbp2	chr16	31957240	31957450	-2.58	-0.37
Fyttd1	chr16	32877869	32884216	-0.32	-0.02
Fyttd1	chr16	32902586	32905812	-0.49	-0.07
Zfp597	chr16	3872223	3872374	-0.32	1.03
Crebbp	chr16	4154961	4213404	-1.70	-0.10
Gtf2e1	chr16	37510570	37510961	-2.88	-0.45
Gtf2e1	chr16	37511589	37522849	-0.57	0.08
Gtf2e1	chr16	37523028	37539825	-0.41	0.04
Arid1b	chr17	5332607	5339263	-2.33	-0.16
Zfp160	chr17	21178540	21179198	-2.36	1.43
Rnps1	chr17	24551619	24552074	-1.68	-0.36
Ppard	chr17	28369728	28432453	-1.72	-0.27
Mapk14	chr17	28828286	28828675	-2.21	-0.37
Brpf3	chr17	28938130	28938251	-0.19	0.10
Brpf3	chr17	28954078	28960704	-1.08	0.19
Brpf3	chr17	28973638	28973848	-0.68	0.35
Psmb8	chr17	34335288	34336214	-1.01	-0.16
Ehmt2	chr17	35042089	35042587	-2.96	-0.29
Ehmt2	chr17	35049712	35050992	-3.05	-0.21
Ddx39b	chr17	35378690	35378917	-2.25	-0.37
Ddx39b	chr17	35386162	35386790	-0.42	-0.02
Ddx39b	chr17	35566011	35566729	-0.95	0.28
Kat2b	chr17	53706385	53706446	-0.22	0.15
Zfp213	chr17	23694268	23694714	-1.32	-0.70

Zfp213	chr17	23694921	23696378	-0.39	0.24
Thoc6	chr17	23810618	23810831	-0.44	0.09
Pdpk1	chr17	24215583	24216033	-2.93	0.32
Ube2i	chr17	25397995	25398776	-0.81	-0.11
Stub1	chr17	25967580	25967909	-2.17	-0.16
Stub1	chr17	25969947	25970306	-0.38	0.08
Taf11	chr17	28038342	28038672	-1.67	-0.25
Taf11	chr17	28042348	28045158	-0.73	0.11
Ring1	chr17	34158155	34160310	-1.41	-0.14
Ring1	chr17	34160369	34161265	-0.31	-0.04
Brd2	chr17	34259120	34259211	-1.42	0.77
Runx2	chr17	44744988	44745109	-0.62	0.24
Runx2	chr17	44872609	44873298	-2.06	-0.37
Taf8	chr17	47625297	47626945	-1.85	-0.17
Taf8	chr17	47638265	47639236	-2.04	-0.48
Gtf2f1	chr17	57143401	57143559	-0.53	-0.10
Gtf2f1	chr17	57143961	57144203	-0.51	-0.06
Tgif1	chr17	71199117	71202842	-1.83	-0.13
Srsf7	chr17	80606178	80606645	-0.76	-0.19
Thoc1	chr18	9993386	9993507	-0.34	0.02
Rbbp8	chr18	11880308	11881165	-0.34	-0.01
Taf4b	chr18	14965941	14971957	-1.59	0.33
Polr2d	chr18	31955847	31956355	-3.30	0.43
Wdr33	chr18	32067351	32067412	-0.44	0.52
Iws1	chr18	32227387	32243000	-1.99	-0.09
Ercc3	chr18	32399984	32400372	-0.36	0.04
Prelid3a	chr18	67639251	67639430	-1.04	0.59
Rnmt	chr18	68460038	68465632	-1.16	0.13
Smad7	chr18	75527018	75527734	-0.56	0.14
Smad7	chr18	75528210	75530550	-2.26	0.57
Smad2	chr18	76422187	76422338	-0.39	0.04
Smad2	chr18	76462139	76464727	-0.86	-0.12
Epc1	chr18	6436991	6440114	-0.66	0.09
Epc1	chr18	6448973	6450113	-2.33	0.40
Epc1	chr18	6490659	6515906	-1.33	-0.35
Kctd1	chr18	15220309	15220877	-0.44	0.38
Kctd1	chr18	15221623	15221804	-0.15	-0.06
Kctd1	chr18	15221952	15222043	-0.14	0.32
Nr3c1	chr18	39588392	39649457	-3.45	0.21
Smad4	chr18	73799858	73800008	-0.66	-0.12
Smad4	chr18	73863185	73863395	-3.16	-1.68
Ctdp1	chr18	80605356	80630912	-3.52	0.36
Ctdp1	chr18	80642798	80647906	-3.16	-0.17

Men1	chr19	6335008	6336781	-2.54	-0.27
Men1	chr19	6338873	6340864	-1.37	-0.10
Ints5	chr19	8967536	8971662	-1.15	-0.08
Ints5	chr19	8971870	8972350	-0.64	-0.09
Smarca2	chr19	26705264	26705505	-0.28	0.00
Cstf2t	chr19	31157419	31159177	-0.63	-0.03
Pcgf5	chr19	36484266	36486670	-2.56	0.48
Pcgf5	chr19	36519495	36530396	-0.85	-0.17
Taf5	chr19	47142775	47149532	-1.43	0.33
Taf5	chr19	47156787	47157401	-0.49	0.09
Rad9a	chr19	4195437	4196286	-0.91	-0.11
Rbm14	chr19	4743957	4744526	-1.85	-0.59
Kat5	chr19	5603013	5603692	-1.67	-0.15
Kat5	chr19	5609321	5609621	-0.39	-0.05
Kat5	chr19	5609680	5610034	-0.36	-0.10
Prdx5	chr19	6983955	6984135	-1.81	-0.18
Esrra	chr19	6996237	6996298	-0.31	0.14
Polr2g	chr19	8872986	8873047	-0.27	-0.01
Ldb1	chr19	46107090	46108545	-0.69	0.04
Ldb1	chr19	46119462	46119522	-0.53	-0.37
Pcgf6	chr19	47108916	47109007	-0.64	-0.23
Med12	chrX	98488039	98488190	-2.90	0.51
Taf1	chrX	98794675	98794915	-0.42	0.05
Cstf2	chrX	130609104	130609225	-1.51	0.37
Rragb	chrX	149574589	149575911	-0.52	0.09
G6pdx	chrX	71655299	71655479	-0.51	-0.03
Rad9b	chr5	122803966	122804026	-0.22	0.19

Immune system

gene_name	chr	chromStart	chromEnd	diff.lg.p	diff.log2.fc
Il1r1	chr1	40370384	40370505	-0.22	1.00
Il1rl1	chr1	40518643	40519182	-0.86	-0.23
Il1rl1	chr1	40521392	40521602	-0.79	0.31
Il18rap	chr1	40605544	40606142	-0.34	-0.04
Stat1	chr1	52216218	52216729	-0.42	0.05
Casp8	chr1	58890568	58903481	-0.47	0.03
Abi2	chr1	60527596	60534214	-0.37	0.02
Cd28	chr1	60826544	60826935	-0.44	-0.25
Cd28	chr1	60827743	60828044	-0.54	0.44
Creb1	chr1	64622786	64644320	-3.22	0.24
Slc11a1	chr1	74421776	74422412	-1.15	-0.25
Slc11a1	chr1	74432056	74432625	-0.60	0.06
Psmd1	chr1	87961193	87961313	-0.67	0.21
Eif4e2	chr1	89134456	89135775	-0.30	0.00

Inpp5d	chr1	89516886	89517096	-2.34	-0.38
Inpp5d	chr1	89614406	89616844	-1.74	-0.09
Ube2f	chr1	93182063	93182512	-2.14	-0.16
Asb1	chr1	93437351	93443741	-2.00	0.50
Asb1	chr1	93448832	93448983	-0.89	0.38
Asb1	chr1	93451373	93451764	-0.41	0.07
Tnfrsf11a	chr1	107721612	107741653	-2.53	-0.18
Arl8a	chr1	137043410	137043590	-2.90	-0.45
Ncf2	chr1	154654924	154658055	-0.49	0.03
Rnasel	chr1	155600002	155602337	-0.35	-0.01
Rnasel	chr1	155610184	155610334	-0.28	0.03
Creg1	chr1	167693910	167694001	-1.58	-0.40
Cd247	chr1	167791873	167791964	-0.45	2.42
Fcgr4	chr1	172959267	172959892	-3.04	0.51
Tmem63a	chr1	182904309	182904638	-0.97	0.21
Hecw2	chr1	53869734	53883756	-1.12	2.77
Hecw2	chr1	53982435	53983513	-0.26	-0.03
Sumo1	chr1	59696455	59696989	-1.11	-0.09
Tmbim1	chr1	74335150	74335301	-0.54	-0.09
Tmbim1	chr1	74341896	74350790	-2.54	-0.44
Tuba4a	chr1	75211756	75211936	-0.82	-0.43
Cul3	chr1	80336885	80337005	-1.62	-0.38
Trip12	chr1	84721087	84721998	-1.08	0.10
Trip12	chr1	84782695	84836815	-1.84	0.07
Pdcd1	chr1	95937963	95949130	-1.13	-1.44
Bcl2	chr1	108438354	108439674	-0.39	0.03
Bcl2	chr1	108439763	108608408	-1.52	0.53
Bcl2	chr1	108608737	108610867	-0.32	0.01
Ptpn4	chr1	121555598	121572143	-1.58	0.18
Ptpn4	chr1	121733527	121733648	-0.29	-0.05
Actr3	chr1	127331893	127332265	-1.67	-0.16
Ikbke	chr1	133151208	133153534	-0.51	-0.04
Ikbke	chr1	133173083	133173488	-3.28	-0.78
Dhx9	chr1	155303276	155303397	-0.99	0.27
Qsox1	chr1	157626418	157628411	-1.52	-0.23
Klhl20	chr1	163019047	163019258	-1.39	-0.43
Klhl20	chr1	163039327	163060580	-0.32	0.01
Fcgr2b	chr1	172890688	172891194	-2.46	0.13
Fcgr3	chr1	172981418	172981627	-0.40	0.03
Ncstn	chr1	173996440	173996917	-1.85	-0.17
Degs1	chr1	184206857	184208760	-0.92	0.08
Ppp2r5a	chr1	193176577	193176817	-2.17	0.22
Prkcq	chr2	11221863	11221954	-0.17	-0.59

Il15ra	chr2	11654930	11655499	-1.57	0.54
Commd3	chr2	18596461	18597638	-0.89	0.08
Il1rn	chr2	24205097	24205277	-0.26	0.00
Anapc2	chr2	25128463	25128941	-0.88	-0.09
Fbxw5	chr2	25358791	25359805	-1.19	0.14
Fbxw5	chr2	25360013	25360452	-1.24	-0.22
Fbxw5	chr2	25360840	25360960	-3.19	1.04
Rapgef1	chr2	29475329	29475420	-0.29	-0.07
Rapgef1	chr2	29557821	29558001	-0.99	-0.14
Abl1	chr2	31544075	31551320	-0.40	0.02
Abl1	chr2	31652573	31658159	-1.65	-0.10
Abl1	chr2	31658367	31658428	-1.12	-0.61
Abl1	chr2	31659237	31659477	-0.56	0.58
Ptges2	chr2	32257756	32257994	-1.02	-0.29
Mapkap1	chr2	34479093	34479423	-1.30	0.22
Hspa5	chr2	34630750	34631930	-2.17	-0.03
Tank	chr2	61416642	61431225	-1.19	-0.33
Tank	chr2	61482412	61484260	-0.36	-0.03
Tank	chr2	61484409	61488438	-0.62	0.04
Tank	chr2	61491449	61491629	-0.49	0.06
Klhl41	chr2	69508654	69508893	-1.32	0.66
Dync1i2	chr2	71049792	71049883	-0.39	0.11
Itgav	chr2	83643447	83643658	-1.94	-0.36
Itgav	chr2	83643837	83644077	-1.39	0.28
Psmc3	chr2	90894439	90895182	-1.38	-0.10
Traf6	chr2	101531587	101537929	-3.55	-0.22
Kif18a	chr2	109173461	109173702	-2.70	0.61
B2m	chr2	121973422	121973632	-1.88	-0.08
Trim69	chr2	121993421	121993481	-0.14	0.42
Trim69	chr2	122004286	122004614	-0.57	-1.61
Sirpa	chr2	129418574	129418963	-0.30	0.00
Pygb	chr2	150654298	150656555	-1.01	0.16
Hck	chr2	152934322	152934473	-1.33	0.47
Hck	chr2	152976937	152977028	-0.61	-0.13
Kif3b	chr2	153141953	153146041	-2.25	0.20
Kif3b	chr2	153155920	153156639	-0.36	0.02
Kif3b	chr2	153158285	153158915	-0.93	0.19
Itch	chr2	154959216	154959307	-0.25	0.00
Itch	chr2	155050132	155050373	-0.80	-0.16
Src	chr2	157295226	157296471	-0.85	0.32
Plcg1	chr2	160587522	160588233	-1.55	-0.33
Mmp9	chr2	164779119	164780689	-0.74	0.61
Mmp9	chr2	164780747	164780808	-0.24	0.74

Cd40	chr2	164896297	164896475	-0.35	0.15
Cd40	chr2	164896740	164896829	-0.20	0.00
Rnf114	chr2	167318293	167325329	-0.28	-0.05
Ptpn1	chr2	167757856	167758036	-0.92	0.24
Ptpn1	chr2	167800939	167802433	-0.91	0.11
Ptpn1	chr2	167802791	167803629	-1.01	0.16
Dnajc5	chr2	181255239	181255330	-0.27	0.01
Abi1	chr2	22895609	22895730	-1.66	-0.37
Tubb4b	chr2	25077884	25079546	-0.33	0.00
Tubb4b	chr2	25079713	25080192	-0.31	-0.02
Dpp7	chr2	25207809	25207987	-0.69	0.11
Traf2	chr2	25374427	25375145	-1.34	-0.17
Card9	chr2	26212789	26214357	-0.38	0.02
Agpat2	chr2	26449106	26450111	-0.33	-0.03
Surf4	chr2	26782443	26789001	-3.09	-0.35
Vav2	chr2	27282074	27282345	-0.49	-0.25
Lrsam1	chr2	32781993	32782383	-0.65	-0.16
Fbxw2	chr2	34661048	34661869	-2.12	-0.20
Fbxw2	chr2	34662764	34664040	-0.46	-0.08
Fbxw2	chr2	34668436	34678672	-1.12	-0.10
Fbxw2	chr2	34681468	34681725	-0.58	0.19
Psmd5	chr2	34707993	34709794	-1.37	-0.17
Psmd5	chr2	34722532	34726482	-1.60	-0.40
Psmb7	chr2	38498946	38499426	-1.35	-0.19
Wipf1	chr2	73270330	73270480	-3.23	-0.40
Wipf1	chr2	73273024	73278150	-0.91	-0.05
Wipf1	chr2	73282527	73291856	-2.22	0.32
Ptprj	chr2	90273202	90274160	-3.35	-0.26
Ptprj	chr2	90420474	90420774	-0.84	-0.21
Cat	chr2	103325161	103325280	-0.58	-0.15
Atp8b4	chr2	126148354	126148594	-0.43	0.05
Il1a	chr2	129126416	129126536	-0.54	0.73
Il1b	chr2	129195984	129196856	-1.18	3.00
Ubox5	chr2	130416966	130417566	-1.43	-0.36
Ubox5	chr2	130425325	130426405	-0.55	0.08
Siglec1	chr2	130895194	130895494	-1.06	0.66
Siglec1	chr2	130900572	130901960	-0.37	0.08
Siglec1	chr2	130903894	130904407	-0.55	0.28
Siglec1	chr2	130906334	130909140	-0.49	0.17
Siglec1	chr2	130911282	130911343	-0.24	-0.08
Siglec1	chr2	130911962	130912441	-2.24	1.31
Cd93	chr2	148266841	148266931	-0.14	0.32
Psmf1	chr2	151543111	151543232	-0.83	-0.47

Psmf1	chr2	151543649	151544068	-0.81	-0.19
Trib3	chr2	152169646	152169796	-3.17	-0.77
Bcl2l1	chr2	152606972	152607272	-1.10	0.18
Bcl2l1	chr2	152656208	152657418	-1.01	-0.36
Cpne1	chr2	155922158	155937671	-0.37	-0.01
Dsn1	chr2	156820797	156820977	-0.49	0.26
Dsn1	chr2	156821482	156821990	-2.26	-0.39
Elmo2	chr2	165115147	165116717	-2.45	-0.22
Elmo2	chr2	165117554	165119793	-0.34	0.05
Nfatc2	chr2	168301939	168303199	-2.11	-0.21
Nfatc2	chr2	168304128	168330396	-0.75	0.06
Nfatc2	chr2	168396287	168427005	-2.25	-0.19
Fabp5	chr3	10012692	10015564	-0.91	0.09
Pld1	chr3	28029255	28031356	-0.46	0.06
Pld1	chr3	28031505	28031985	-0.33	-0.03
Pik3ca	chr3	32335072	32335402	-0.84	0.17
Pik3ca	chr3	32360681	32362022	-0.52	0.05
Rap2b	chr3	61168428	61170467	-0.56	0.02
Rap2b	chr3	61171065	61171426	-2.42	0.51
Ctso	chr3	81736537	81736628	-0.27	0.06
Ctso	chr3	81756174	81758915	-1.63	0.24
Fbxw7	chr3	84619498	84694070	-1.92	0.38
Fbxw7	chr3	84694129	84756226	-2.05	0.20
Fbxw7	chr3	84780140	84781955	-0.37	0.02
Shc1	chr3	89222472	89226252	-2.21	-0.23
Shc1	chr3	89232244	89232484	-0.63	-0.10
Ube2q1	chr3	89577530	89577890	-3.20	-0.36
Ube2q1	chr3	89585705	89587919	-0.89	-0.04
Ilf2	chr3	90291521	90291852	-0.76	-0.09
S100a8	chr3	90472992	90473549	-0.69	2.04
Them4	chr3	94135467	94135976	-0.96	0.20
Ctss	chr3	95330707	95333452	-1.18	-0.07
Rnf115	chr3	96592580	96593978	-1.30	-0.15
Sec22b	chr3	97725140	97725438	-2.99	-0.26
Nras	chr3	102868375	102868495	-0.73	-0.10
Vav3	chr3	109143630	109143900	-2.32	-1.00
Cenpe	chr3	134910323	134910564	-0.59	-0.09
Cenpe	chr3	134933684	134933745	-0.39	0.11
Cenpe	chr3	134936264	134936504	-0.40	0.06
Ube2d3	chr3	135101656	135102228	-3.24	-0.27
Manba	chr3	135230647	135233888	-3.27	-0.25
Bcl10	chr3	145587232	145587592	-0.60	0.07
Bcl10	chr3	145588099	145593615	-2.28	0.24

Il7	chr3	7573754	7573815	-0.33	-2.32
Pag1	chr3	9689310	9689401	-0.90	-0.46
Pag1	chr3	9690420	9691081	-1.53	-0.30
Gm9733	chr3	15320566	15320714	-0.28	-0.19
Nhlrc3	chr3	53255917	53257492	-3.45	-0.39
Siah2	chr3	58495620	58495891	-1.37	0.55
Dhx36	chr3	62274511	62275295	-0.47	-0.07
Tlr2	chr3	83640193	83642691	-0.41	-0.01
Lamtor2	chr3	88353798	88356849	-1.88	-0.11
Il6ra	chr3	89673245	89675305	-2.03	-0.13
Psmd4	chr3	94836626	94836924	-1.79	-0.12
Psmd4	chr3	94839816	94846467	-2.16	-0.13
Hist2h3c2	chr3	96042454	96042544	-0.57	0.69
Cd101	chr3	100797451	100797692	-0.26	-0.05
Csf1	chr3	107544354	107544444	-0.83	0.84
Csf1	chr3	107544832	107548597	-0.29	-0.01
Csf1	chr3	107548745	107550706	-1.24	-1.52
Csf1	chr3	107551063	107553281	-0.37	0.05
Sec24b	chr3	129686102	129687101	-0.83	-0.07
Nfkb1	chr3	135248217	135252299	-2.46	-0.20
Nfkb1	chr3	135354240	135354361	-0.32	0.21
Dapp1	chr3	137595527	137598550	-2.31	-0.57
Prkacb	chr3	146394723	146394993	-1.39	-0.21
Lyn	chr4	3605267	3605506	-3.28	-0.20
Fbxl4	chr4	22303713	22313319	-1.70	0.24
Fbxl4	chr4	22354425	22354486	-0.94	0.57
Map3k7	chr4	32051081	32051232	-2.61	-1.03
Map3k7	chr4	32109961	32110261	-0.43	0.08
Ube2j1	chr4	33136832	33136892	-1.32	-0.32
Ube2r2	chr4	41137777	41138136	-1.17	0.13
Atp6v1g1	chr4	63205828	63209679	-1.23	0.19
Trim32	chr4	65274645	65276139	-0.35	0.01
Pgm2	chr4	99602055	99602234	-1.25	-0.43
Pgm2	chr4	99656703	99659542	-1.56	-0.30
Lrrc41	chr4	115761466	115762183	-1.09	-0.08
Lrrc41	chr4	115768961	115769260	-0.52	0.05
Csf3r	chr4	125720761	125721238	-3.13	-0.56
Ptafr	chr4	132120011	132136781	-0.37	-0.01
Fgr	chr4	132530039	132542068	-1.56	0.56
Fuca1	chr4	135495027	135495355	-0.80	0.11
Eif4g3	chr4	137676266	137682738	-0.34	0.00
Eif4g3	chr4	137702245	137707514	-0.39	-0.05
Eif4g3	chr4	137727076	137727166	-0.70	-0.10

Eif4g3	chr4	137762176	137762386	-2.48	0.37
Ddost	chr4	137868046	137868376	-0.67	-0.07
Capzb	chr4	138748845	138794946	-0.34	-0.01
Casp9	chr4	141349886	141352694	-2.10	-0.34
Casp9	chr4	141368153	141369672	-0.94	-0.31
Casp9	chr4	141369821	141369911	-0.41	-0.59
Fbxo2	chr4	147538344	147538434	-0.25	-0.78
Mtor	chr4	147822690	147826440	-1.12	-0.33
Mtor	chr4	147930392	147931764	-0.80	0.10
Tnfrsf9	chr4	150302149	150307203	-1.72	0.87
Klhl21	chr4	151383089	151389959	-0.56	0.05
Klhl21	chr4	151390287	151390408	-0.53	0.22
Ripk2	chr4	16050729	16051621	-2.11	-0.28
Ufl1	chr4	25177588	25177709	-0.34	0.05
Ddx58	chr4	40152603	40155517	-1.64	0.25
B4galt1	chr4	40753786	40754830	-0.76	-0.05
B4galt1	chr4	40770567	40801001	-1.03	0.10
Vcp	chr4	43013259	43013349	-0.48	-0.16
Fbxo10	chr4	45048105	45048374	-0.47	0.54
Fbxo10	chr4	45048433	45048494	-0.73	1.50
Fbxo10	chr4	45048553	45048793	-0.74	0.68
Fbxo10	chr4	45071323	45071922	-0.48	0.30
Cdc26	chr4	62055609	62055998	-2.00	0.59
Alad	chr4	62170203	62171204	-0.65	-0.14
Ifnb1	chr4	88167987	88168343	-0.39	1.73
Ifnb1	chr4	88168549	88168698	-0.33	1.45
Jun	chr4	94716322	94718913	-1.41	-0.04
Jak1	chr4	100825596	100826046	-0.48	0.04
Kif2c	chr4	116832539	116832928	-1.47	-0.33
Rnf220	chr4	116958526	116968779	-3.32	0.35
Cdc20	chr4	118109887	118109948	-0.55	-0.37
Lck	chr4	129225797	129226067	-0.39	0.10
Txlna	chr4	129311785	129317754	-0.43	-0.02
Cda	chr4	137894592	137894712	-1.83	1.47
Fbxo6	chr4	147520063	147520213	-1.03	0.25
Pik3cd	chr4	149023545	149025880	-2.62	-0.17
Spsb1	chr4	149271827	149281337	-1.02	0.57
Mib2	chr4	155028580	155028761	-0.30	-0.09
Atad3a	chr4	155114748	155115286	-1.57	-0.13
Ube3c	chr5	29895781	29895962	-1.72	-1.14
Ube3c	chr5	30001057	30001268	-0.46	0.07
Bst1	chr5	44210131	44210281	-0.56	-0.24
Anapc4	chr5	53257704	53257973	-2.14	-0.29

Klhl5	chr5	65558211	65558721	-1.61	0.23
Cxcl3	chr5	91215188	91215429	-0.26	-0.14
Stbd1	chr5	93034080	93034170	-0.19	-0.81
Ube3b	chr5	114830734	114830795	-0.28	0.61
Ube3b	chr5	114869593	114869952	-0.38	0.03
Fbxo21	chr5	118426838	118426928	-0.57	0.34
Fbxo21	chr5	118452063	118458648	-0.32	0.01
Hvcn1	chr5	122690267	122692306	-0.59	-0.07
Anapc7	chr5	122891328	122891538	-0.52	-0.09
Anapc7	chr5	122893875	122894502	-3.47	-0.38
P2rx7	chr5	123131027	123132855	-0.67	0.05
P2rx7	chr5	123133154	123133964	-0.37	-0.02
Rnf34	chr5	123273869	123314504	-1.72	0.21
Psmd9	chr5	123678228	123684616	-1.07	0.18
Atp6v0a2	chr5	125200361	125202517	-3.02	0.33
Kctd7	chr5	130628247	130628815	-1.33	-0.62
Tnip2	chr5	34839221	34839491	-0.36	0.04
Fbxl5	chr5	44142150	44149098	-2.30	-0.48
Fbxl5	chr5	44149396	44151973	-0.42	-0.02
Tlr1	chr5	65317328	65317538	-0.30	0.07
Tlr1	chr5	65318076	65318466	-0.27	-0.01
Tlr6	chr5	65344333	65346799	-3.02	0.30
Atp8a1	chr5	68013273	68013813	-0.67	0.11
Tec	chr5	73147021	73148633	-1.95	-0.24
Tec	chr5	73214842	73259687	-1.72	0.36
Rchy1	chr5	92391795	92392064	-0.36	0.05
Sec31a	chr5	100790877	100791058	-0.38	0.02
Mlec	chr5	115597834	115600228	-0.64	-0.05
Fbxw8	chr5	118515497	118515797	-0.35	-0.06
Ptpn11	chr5	121581827	121582307	-2.13	0.25
Ptpn11	chr5	121583292	121587519	-0.51	-0.03
Rhof	chr5	123553648	123553977	-2.60	1.51
Rhof	chr5	123567408	123567856	-1.12	0.40
Rhof	chr5	123568123	123582429	-2.11	-0.12
Slc15a4	chr5	128076540	128080902	-0.77	-0.08
Ncf1	chr5	134705434	134705495	-1.15	-0.28
Trim56	chr5	137587155	137588025	-0.51	-0.04
Pilra	chr5	138263299	138263390	-0.40	1.81
Pilra	chr5	138264796	138272396	-1.16	1.30
Pilra	chr5	138276577	138276758	-2.35	1.83
Pilrb2	chr5	138312135	138312373	-0.55	2.87
Card11	chr5	141348952	141350390	-3.03	-0.50
Fbxl18	chr5	143633766	143634186	-0.39	0.07

Fbxl18	chr5	143640382	143644224	-0.93	-0.38
Fbxl18	chr5	143650503	143656917	-0.38	-0.27
Smurf1	chr5	145637693	145638023	-0.74	-0.29
Smurf1	chr5	145638352	145638682	-1.09	-0.34
Smurf1	chr5	145639310	145639491	-0.37	0.13
Rnf6	chr5	147021822	147022181	-2.92	0.33
Znrf2	chr6	54838508	54838629	-0.37	-0.02
Herc6	chr6	57445639	57531182	-0.37	-0.17
Dctn1	chr6	83140226	83141067	-0.66	-0.06
Kbtbd8	chr6	95071961	95077327	-2.16	0.32
Arpc4	chr6	113328136	113330743	-0.80	-0.14
Il17re	chr6	113420086	113420177	-0.14	0.22
Vhl	chr6	113578147	113579978	-1.16	0.21
Irak2	chr6	113588490	113588581	-0.26	0.00
Fbxl14	chr6	119429685	119431451	-2.73	-0.15
Il17ra	chr6	120413214	120413455	-0.36	-0.04
Usp18	chr6	121195982	121196071	-0.23	-0.06
Clec4d	chr6	123212183	123212244	-1.46	0.42
Gm8994	chr6	136278992	136279111	-0.18	0.47
Gm8994	chr6	136279792	136279971	-0.26	0.19
Lrmp	chr6	145070431	145070492	-1.05	0.44
Lrmp	chr6	145086263	145086444	-1.03	0.32
Lrmp	chr6	145123118	145123179	-0.82	-0.20
Klhl42	chr6	147039984	147056970	-0.73	-0.10
Impdh1	chr6	29159242	29166081	-0.29	0.00
Ube2h	chr6	30163827	30164395	-0.64	0.08
Ube2h	chr6	30164514	30164664	-1.04	0.40
Ube2h	chr6	30164783	30164993	-0.55	0.22
Nod1	chr6	54874621	54874682	-0.58	2.04
Nod1	chr6	54893198	54893646	-1.73	2.07
Nod1	chr6	54894093	54894451	-0.13	0.08
Nod1	chr6	54894629	54894929	-0.30	0.33
Vamp8	chr6	72335214	72335506	-0.31	0.00
Vamp8	chr6	72338374	72340661	-0.33	-0.02
Kcmf1	chr6	72849172	72849233	-0.46	-0.84
Kcmf1	chr6	72849822	72849973	-0.55	-0.62
Rbsn	chr6	92139105	92140395	-0.37	0.02
Raf1	chr6	115568620	115569280	-2.59	-0.22
Alox5	chr6	116410986	116411196	-0.57	-0.13
Atp6v1e1	chr6	120768397	120772703	-0.46	-0.04
Slc2a3	chr6	122679681	122681413	-0.60	-0.09
C1s1	chr6	124480928	124481078	-0.13	0.19
C1s2	chr6	124575479	124575809	-0.34	1.58

Ptpn6	chr6	124683075	124688519	-0.61	-0.20
Olr1	chr6	129436944	129437814	-0.53	2.03
Kras	chr6	145168783	145169023	-0.29	0.00
Itpr2	chr6	146060001	146060981	-1.65	0.22
Ap2s1	chr7	17323792	17328621	-1.81	-0.21
Plaur	chr7	25257191	25257252	-0.42	-0.17
Akt2	chr7	28422202	28423428	-1.06	0.15
Tyrobp	chr7	31198806	31198896	-0.38	-0.01
Irf3	chr7	52253029	52253209	-0.82	-0.23
Irf3	chr7	52256298	52257801	-3.00	-0.31
Irf3	chr7	52257979	52258069	-0.96	-1.07
Cyfip1	chr7	63097490	63097551	-0.47	0.63
Cyfip1	chr7	63097610	63127288	-0.74	0.10
Herc2	chr7	63485102	63486928	-1.77	0.13
Ube3a	chr7	66484119	66496353	-2.58	0.28
Klhl25	chr7	82993342	83012197	-1.00	0.11
Klhl25	chr7	83012375	83018299	-0.87	-0.23
Klhl25	chr7	83018716	83018926	-0.35	-0.11
Ctsc	chr7	95437215	95437515	-0.81	0.31
Ctsc	chr7	95458037	95458487	-2.34	0.25
Prcp	chr7	100081654	100082343	-0.74	-0.10
Ndufc2	chr7	104548571	104548661	-1.22	-0.24
Lamtor1	chr7	109054380	109058211	-1.32	-0.28
Ampd3	chr7	117954502	117954682	-3.30	0.62
Dctn5	chr7	129276554	129277689	-0.53	0.10
Dctn5	chr7	129291791	129292440	-1.28	0.09
Rbbp6	chr7	130114077	130114438	-1.49	-0.14
Rbbp6	chr7	130133665	130133786	-0.82	-0.15
Rbbp6	chr7	130140585	130140795	-0.65	0.08
Il4ra	chr7	132718698	132720561	-0.42	-0.05
Il4ra	chr7	132720860	132721370	-2.99	0.66
Il21r	chr7	132776065	132776545	-0.24	0.08
Fbxl19	chr7	134890288	134891744	-2.81	-0.80
Fbxl19	chr7	134911014	134911877	-0.34	-0.02
Stx4a	chr7	134985321	134985978	-0.91	-0.11
Ap2a2	chr7	148748108	148748169	-1.07	-0.80
Tarm1	chr7	3497555	3497675	-0.39	2.12
Lair1	chr7	3960490	3960699	-1.39	1.24
Lair1	chr7	3961115	3961205	-1.34	-1.04
Il11	chr7	4725025	4727615	-1.07	0.44
Il11	chr7	4727931	4729743	-0.36	0.10
C5ar2	chr7	16821937	16822178	-0.32	-0.19
C5ar2	chr7	16822506	16823255	-0.32	0.07

Calm3	chr7	17502039	17502309	-1.49	0.09
Klc3	chr7	19979788	19981213	-1.51	-0.23
Relb	chr7	20191570	20192229	-0.55	-0.06
Nectin2	chr7	20302441	20302980	-0.95	0.42
Nectin2	chr7	20306842	20310128	-0.64	-0.15
Nectin2	chr7	20334652	20334892	-0.56	0.22
Pvr	chr7	20490268	20494550	-0.49	0.06
Ceacam1	chr7	26247739	26248608	-3.25	0.28
Ceacam1	chr7	26256924	26258919	-0.89	0.24
Ceacam2	chr7	26302704	26302765	-2.15	3.98
Psmd8	chr7	29965393	29965573	-1.95	-0.13
Cd22	chr7	31662583	31662704	-0.12	0.00
Siglece	chr7	50914788	50914849	-0.21	-0.02
Flt3l	chr7	52386558	52386918	-3.47	0.88
Asb7	chr7	73792504	73823980	-0.99	-0.14
Asb7	chr7	73834117	73834387	-0.57	0.21
Det1	chr7	85972688	85972809	-1.48	-0.65
Det1	chr7	85988116	85988866	-0.31	-0.01
Anpep	chr7	86966748	86966927	-1.32	0.20
Anpep	chr7	86986085	86993080	-2.81	-0.11
Iqgap1	chr7	87858508	87858659	-0.78	-0.04
Inppl1	chr7	108971355	108974609	-1.09	-0.05
Inppl1	chr7	108985423	108986411	-0.97	-0.21
Inppl1	chr7	108986619	108986740	-1.28	1.00
Rhog	chr7	109387636	109398632	-1.64	-0.05
Trim21	chr7	109707270	109708500	-0.61	0.13
Sh2b1	chr7	133610925	133611549	-0.93	-0.18
Sh2b1	chr7	133617103	133618554	-2.01	-0.54
Aldoa	chr7	133943840	133943961	-0.40	-0.30
Kif22	chr7	134179351	134185934	-1.40	-0.39
Adam8	chr7	147177314	147178387	-0.69	-0.11
Ifitm2	chr7	148141740	148141860	-0.90	0.06
Hras	chr7	148376639	148378177	-1.50	-0.13
Hras	chr7	148378835	148379754	-0.27	-0.01
Hras	chr7	148379812	148379903	-0.43	0.54
Irf7	chr7	148451539	148451718	-1.08	0.31
Fadd	chr7	151765572	151768281	-2.06	-0.21
Map2k7	chr8	4245576	4246251	-1.83	-0.18
Map2k7	chr8	4246579	4246760	-0.57	-0.12
Tnfsf13b	chr8	10035462	10035612	-0.36	0.13
Atp11a	chr8	12757015	12757255	-0.70	-0.56
Atp11a	chr8	12868009	12868130	-0.61	0.20
Cdc16	chr8	13779233	13781763	-0.76	0.08

Plpp5	chr8	26834611	26834970	-2.39	-0.33
Ppp2cb	chr8	34710092	34710330	-0.74	-0.58
Sh3rf1	chr8	63704632	63808042	-0.71	0.21
Sh3rf1	chr8	63863616	63863797	-0.45	-0.26
Sh3rf1	chr8	63865414	63873071	-0.33	-0.05
Myo9b	chr8	73796612	73839062	-1.76	-0.10
Myo9b	chr8	73857637	73857698	-0.67	0.13
Myo9b	chr8	73880502	73884611	-1.08	-0.05
Man2b1	chr8	87607167	87608319	-0.31	0.01
Cyld	chr8	91220956	91226218	-0.51	0.31
Fbxl8	chr8	107791922	107793078	-0.54	-0.13
Nfatc3	chr8	108602571	108603618	-0.98	0.07
Nfatc3	chr8	108632435	108652166	-2.54	0.17
Ap1g1	chr8	112382745	112384686	-0.52	0.03
Znrf1	chr8	114060539	114062246	-2.99	0.21
Znrf1	chr8	114146249	114147028	-0.58	0.04
Gan	chr8	119682094	119707502	-2.38	0.66
Plcg2	chr8	120022190	120028105	-0.40	0.04
Tubb3	chr8	125944514	125944901	-0.18	-0.07
Itgb1	chr8	131209643	131209853	-0.70	-0.08
Ikbkb	chr8	23770546	23770997	-1.89	-0.25
Golga7	chr8	24352846	24353057	-1.27	0.16
Hgsnat	chr8	27054960	27055615	-0.42	0.03
Hgsnat	chr8	27083350	27087156	-1.41	-0.31
Dctn6	chr8	35169406	35171565	-0.59	0.15
Lonrf1	chr8	37280137	37282962	-0.64	0.13
Lonrf1	chr8	37311699	37312090	-1.27	-0.69
Tlr3	chr8	46482457	46483076	-0.93	0.75
Tlr3	chr8	46483195	46483675	-0.31	-0.08
Tlr3	chr8	46483734	46484485	-0.49	0.27
Klhl2	chr8	67219421	67219870	-3.53	-0.50
Pik3r2	chr8	73292079	73292558	-1.14	-0.16
Bst2	chr8	74061276	74061336	-0.33	-0.04
Adgre5	chr8	86247268	86247687	-1.48	-0.25
Calr	chr8	87366315	87366923	-2.76	-0.06
Calr	chr8	87369960	87370800	-0.94	0.04
Siah1a	chr8	89248756	89269816	-1.08	-0.12
Dync1li2	chr8	106944120	106944451	-1.40	-0.19
Psmb10	chr8	108461631	108461997	-1.25	-0.38
Hp	chr8	112099087	112099834	-0.43	-0.11
Ist1	chr8	112195789	112196600	-2.35	0.15
Il34	chr8	113266054	113266576	-0.63	1.89
Il34	chr8	113279275	113279364	-0.18	0.74

Cotl1	chr8	122364299	122364479	-0.72	0.10
Fbxo31	chr8	124075878	124076237	-3.34	0.63
Galns	chr8	125102225	125103030	-0.47	-0.05
Galns	chr8	125135207	125135327	-1.24	0.83
Casp1	chr9	5298664	5299458	-2.63	0.43
Pin1	chr9	20456573	20459918	-2.86	-0.44
Icam1	chr9	20820403	20823729	-0.66	-0.08
Icam1	chr9	20824027	20824596	-1.12	0.25
Icam1	chr9	20831708	20833180	-0.81	0.04
Icam5	chr9	20838239	20839240	-1.67	1.05
Icam5	chr9	20840977	20842091	-0.59	0.20
Slc44a2	chr9	21156935	21159352	-1.16	0.07
Dnm2	chr9	21310134	21311379	-1.31	0.09
Smarca4	chr9	21505619	21507475	-1.36	0.14
Ppp2r1b	chr9	50687990	50688710	-0.88	-0.11
Ppp2r1b	chr9	50701016	50701705	-0.62	0.10
Psma4	chr9	54805658	54805748	-1.39	0.21
Fbxo22	chr9	55066270	55071672	-2.03	0.17
Fbxo22	chr9	55071880	55072031	-0.93	-0.28
Pstpip1	chr9	55937782	55937962	-0.42	0.08
Adam10	chr9	70526807	70526988	-1.21	0.24
Adam10	chr9	70626056	70626297	-2.69	0.34
Rab27a	chr9	72892701	72908786	-0.51	-0.51
Rab27a	chr9	72943089	72943840	-1.02	-0.28
Myo5a	chr9	74919012	74919373	-0.83	-0.18
Myo5a	chr9	75071223	75071314	-0.37	0.07
Anapc13	chr9	102528625	102536370	-0.48	0.06
Pik3r4	chr9	105545594	105545714	-0.79	-0.43
Pik3r4	chr9	105546492	105551208	-2.70	0.26
Pik3r4	chr9	105552647	105556389	-1.18	0.18
Pik3r4	chr9	105589445	105589955	-0.50	-0.07
Dusp7	chr9	106270962	106271201	-2.97	-1.90
Dcaf1	chr9	106738991	106740852	-3.06	0.55
Dcaf1	chr9	106760607	106761940	-3.49	0.37
Nckipsd	chr9	108714735	108714915	-1.02	-0.43
Nckipsd	chr9	108720039	108720159	-1.23	0.66
Glb1	chr9	114310242	114326114	-2.08	0.44
Glb1	chr9	114368770	114373569	-0.38	-0.02
Glb1	chr9	114374377	114383495	-3.66	0.26
Dync1li1	chr9	114632656	114632895	-3.05	0.38
Dync1li1	chr9	114632812	114633160	-1.11	0.11
Ccr2	chr9	124021097	124021397	-0.49	-0.23
Birc2	chr9	7818584	7818943	-0.91	0.25

Birc2	chr9	7834955	7835255	-3.06	0.75
Birc3	chr9	7848939	7850928	-0.49	0.05
Birc3	chr9	7860311	7872961	-0.93	0.08
Panx1	chr9	14810228	14814484	-1.07	-0.15
Fbxl12	chr9	20448850	20449181	-0.31	-0.05
Keap1	chr9	21034503	21038410	-0.91	0.07
Keap1	chr9	21041585	21042276	-1.22	-0.20
Keap1	chr9	21043025	21043776	-0.79	-0.29
Rab3d	chr9	21714741	21715072	-1.15	-0.35
Tirap	chr9	34994095	34994305	-0.61	0.25
Tirap	chr9	34994573	34996823	-0.33	-0.01
Crtam	chr9	40781146	40781679	-0.68	1.01
Cbl	chr9	44041829	44042099	-2.62	-0.55
Nlrx1	chr9	44060795	44061604	-1.41	-0.32
Nlrx1	chr9	44064475	44064566	-0.30	0.09
Nlrx1	chr9	44070436	44072329	-0.30	0.00
Ube4a	chr9	44731568	44732078	-2.10	0.26
Ube4a	chr9	44756010	44756191	-0.52	-0.14
Il10ra	chr9	45062219	45062579	-0.35	-0.04
Il10ra	chr9	45063175	45068459	-1.09	0.10
Cul5	chr9	53425530	53426100	-1.13	0.12
Pias1	chr9	62729583	62729943	-0.64	0.13
Rnf111	chr9	70274585	70277390	-1.13	-0.14
Fbxo9	chr9	77943491	77943611	-0.30	0.14
Tmem30a	chr9	79617076	79617495	-1.99	0.21
Pik3cb	chr9	98939628	98941447	-3.03	0.22
Armc8	chr9	99380819	99381090	-0.40	-0.09
Il20rb	chr9	100358465	100358556	-0.38	-1.51
Il20rb	chr9	100358644	100359660	-0.54	0.27
Apeh	chr9	107987744	107987924	-0.34	-0.05
Arih2	chr9	108507307	108507428	-0.63	0.11
Arih2	chr9	108522434	108547287	-1.35	0.23
Myd88	chr9	119248949	119249158	-0.84	-0.19
Fuca2	chr10	13223007	13225667	-0.83	-0.15
Fuca2	chr10	13234515	13235470	-0.57	-0.03
Ifngr1	chr10	19329011	19329761	-1.01	-0.10
Frk	chr10	34203714	34203804	-0.35	-0.86
Frk	chr10	34327775	34328313	-0.54	-2.10
Fyn	chr10	39089604	39089964	-1.17	-0.23
Fyn	chr10	39271197	39284162	-3.65	-0.24
Itgb2	chr10	76993092	77005145	-1.85	-0.24
Ube2g2	chr10	77107280	77107637	-0.93	-0.11
Ube2g2	chr10	77107992	77108141	-1.22	0.25

Icosl	chr10	77536609	77536670	-0.38	-0.28
Eef2	chr10	80644273	80645254	-0.46	0.00
Fbxo7	chr10	85487309	85487369	-0.37	0.10
Eea1	chr10	95488768	95489708	-1.82	-0.15
Eea1	chr10	95502273	95503004	-2.06	-0.20
Eea1	chr10	95504733	95504884	-0.95	-0.17
Cep290	chr10	100034744	100035631	-1.47	0.52
Gns	chr10	120802145	120802355	-0.43	0.05
Gns	chr10	120832206	120832746	-0.42	-0.01
Gns	chr10	120834001	120834301	-3.63	0.23
Arhgap9	chr10	126759938	126761407	-0.45	0.12
Stat6	chr10	127080041	127080222	-0.81	-0.13
Stat2	chr10	127727910	127728767	-0.32	-0.02
Tab2	chr10	7625983	7626463	-1.95	0.18
Tab2	chr10	7626671	7644544	-2.25	0.07
Tnfaip3	chr10	18721581	18725137	-2.68	-0.19
Rnf217	chr10	31254643	31329531	-1.58	-0.29
Ube2d1	chr10	70718055	70718592	-2.86	0.34
Trpm2	chr10	77372593	77372804	-0.23	-0.16
Pfkl	chr10	77472360	77472511	-0.36	0.05
Rnf126	chr10	79221259	79223642	-1.89	-0.11
Rnf126	chr10	79229537	79229686	-1.44	-0.51
Fzr1	chr10	80829682	80830187	-1.59	0.17
Ckap4	chr10	83990001	83991399	-2.44	-0.11
Ckap4	chr10	83996216	83996633	-1.45	-0.31
Glipr1	chr10	111434202	111434262	-0.61	0.14
Cand1	chr10	118647900	118648919	-0.55	0.03
Irak3	chr10	119579518	119580238	-0.55	-0.07
Kif5a	chr10	126700029	126700180	-1.18	-0.45
Il23a	chr10	127733549	127733905	-0.33	-0.14
Ap1b1	chr11	4847942	4887051	-1.74	0.41
Dbnl	chr11	5688485	5691845	-3.19	-0.45
Peli1	chr11	20991326	20991507	-0.82	-0.25
Psme4	chr11	30671774	30672135	-0.53	-0.17
Stk10	chr11	32504170	32512777	-2.97	-0.29
Fbxw11	chr11	32644536	32644986	-0.65	0.06
Mapk9	chr11	49660312	49660462	-0.47	0.14
Rnf130	chr11	49933551	49933881	-0.41	0.38
Ppp2ca	chr11	51935449	51935836	-0.93	0.05
Kif3a	chr11	53380880	53380941	-1.76	-1.08
Gm2a	chr11	54923031	54923270	-0.59	0.07
Trim11	chr11	58791617	58791916	-0.34	-0.02
Trim11	chr11	58802336	58802515	-1.26	0.40

Trim11	chr11	58802842	58804960	-1.02	0.09
2310033P09Rik	chr11	59021980	59023882	-0.98	0.12
Nlrp3	chr11	59379889	59380129	-0.34	0.02
Map2k3	chr11	60765468	60766245	-1.76	-0.11
Vamp2	chr11	68902059	68902715	-0.32	0.03
Pld2	chr11	70371013	70371612	-1.44	-0.29
Ube2g1	chr11	72420792	72421062	-2.86	-0.55
Ube2g1	chr11	72497025	72497295	-0.29	0.00
Crk	chr11	75518934	75519324	-0.73	0.09
Nos2	chr11	78734360	78735737	-0.82	1.81
Psmd11	chr11	80285574	80285635	-1.26	-0.32
Mpo	chr11	87610892	87611072	-0.77	4.32
Trim25	chr11	88860716	88874870	-1.69	-0.06
Trim25	chr11	88877033	88878370	-0.70	-0.03
Psmd3	chr11	98556993	98557203	-2.02	-0.21
Stat5a	chr11	100721039	100722748	-0.67	-0.27
Atp6v0a1	chr11	100923774	100924043	-2.93	-0.36
Psme3	chr11	101177564	101178336	-0.77	0.09
Psme3	chr11	101182100	101183258	-2.86	-0.26
Baiap2	chr11	119862824	119863004	-2.19	0.86
Baiap2	chr11	119867797	119868096	-2.45	0.41
Nf2	chr11	4748990	4749290	-0.77	-0.14
Il9r	chr11	32090755	32090815	-0.27	-1.58
Dock2	chr11	34127042	34128632	-0.56	-0.03
Dock2	chr11	34546949	34597325	-0.57	0.05
Cyfip2	chr11	46050850	46051031	-0.19	0.09
Cyfip2	chr11	46051359	46051599	-1.21	2.74
Trim41	chr11	48625890	48630474	-2.56	-0.19
Trim41	chr11	48630623	48630893	-2.44	0.54
Sec24a	chr11	51556955	51570200	-1.50	-0.11
Csf2	chr11	54060859	54061185	-0.38	-1.06
Atox1	chr11	55264107	55274640	-1.41	-0.17
Mapk7	chr11	61306963	61307471	-0.67	0.13
Nlrp1b	chr11	70966603	70969571	-1.97	-0.37
Nlrp1b	chr11	70995162	70995342	-0.91	-0.46
Nlrp1b	chr11	70996028	70996268	-0.26	0.01
Wsb1	chr11	79053599	79053898	-2.67	-0.17
Ube2z	chr11	95911464	95911704	-2.06	-0.26
Kpnb1	chr11	97049055	97049206	-3.64	-0.54
Fbxl20	chr11	97944196	97944257	-0.53	0.44
Ormdl3	chr11	98443291	98443381	-1.34	0.71
Ormdl3	chr11	98443469	98443619	-1.65	0.66
Ormdl3	chr11	98443767	98444472	-0.72	-0.16

Klhl11	chr11	100323925	100325737	-2.89	0.19
Klhl11	chr11	100333857	100334096	-0.43	-0.10
Rab5c	chr11	100581475	100599444	-1.66	-0.43
Ghdc	chr11	100627734	100627853	-1.86	0.92
Ghdc	chr11	100630436	100630763	-2.36	0.63
Dusp3	chr11	101835527	101835885	-1.10	-0.08
Dusp3	chr11	101835944	101836213	-2.85	-0.24
Map3k14	chr11	103081077	103082247	-0.72	-0.12
Map3k14	chr11	103100286	103101085	-0.44	0.06
Map3k14	chr11	103103466	103128715	-0.61	-0.18
Cdc27	chr11	104366205	104368561	-0.85	-0.06
Cd79b	chr11	106172774	106172954	-0.59	3.17
Smurf2	chr11	106683743	106684358	-1.06	-0.24
Smurf2	chr11	106781579	106781849	-2.81	-0.99
Cd300c2	chr11	114862804	114862865	-0.52	-0.36
Cd300e	chr11	114914519	114914670	-1.08	1.43
Grb2	chr11	115505517	115505906	-2.19	0.26
Grb2	chr11	115506561	115508033	-2.47	0.12
Ube2o	chr11	116399633	116399902	-0.30	0.01
Ube2o	chr11	116400140	116404120	-2.64	-0.17
Ube2o	chr11	116405875	116406025	-0.84	0.21
Ube2o	chr11	116442611	116442731	-0.48	-0.68
Tmc6	chr11	117627298	117628154	-0.39	-0.04
Socs3	chr11	117830559	117830620	-0.38	0.48
Cant1	chr11	118268378	118271504	-0.34	-0.01
Cant1	chr11	118272131	118279176	-1.88	-0.18
Cant1	chr11	118280220	118280281	-0.29	0.08
P4hb	chr11	120422393	120423503	-0.94	-0.04
Kif3c	chr12	3365310	3367494	-1.39	-0.14
Kif3c	chr12	3403233	3403504	-0.69	0.15
Kif3c	chr12	3405476	3405926	-1.66	0.23
Sdc1	chr12	8778201	8778442	-1.77	-0.68
Sdc1	chr12	8798515	8799355	-0.52	-0.08
Psma6	chr12	56499811	56499900	-1.08	-0.18
Lrr1	chr12	70275442	70276002	-1.46	-0.50
Psmc1	chr12	101348377	101351316	-0.70	-0.11
Dync1h1	chr12	111839634	111839815	-1.16	-0.25
Dync1h1	chr12	111855934	111857506	-0.62	-0.02
Dync1h1	chr12	111904592	111905154	-2.05	0.14
Traf3	chr12	112499706	112500694	-0.68	-0.07
Klc1	chr12	112997059	113010604	-0.37	-0.02
Pld4	chr12	113998865	113999998	-1.43	-0.19
Pld4	chr12	114000207	114001663	-0.42	0.03

Sos2	chr12	70685584	70689696	-1.15	-0.08
Sos2	chr12	70782629	70782809	-1.89	-1.62
Ppp2r5e	chr12	76594419	76616288	-3.57	0.50
Ppp2r5e	chr12	76616467	76697187	-3.21	0.22
Atp6v1d	chr12	79962474	79962535	-1.42	-0.42
Npc2	chr12	86097901	86101679	-1.04	0.04
Arel1	chr12	86259725	86261648	-0.82	0.05
Arel1	chr12	86311686	86311836	-0.96	0.38
Rps6ka5	chr12	101789211	101791305	-1.35	-0.27
Elmo1	chr13	20692056	20697406	-0.46	0.03
Hist1h3d	chr13	23667608	23667786	-1.85	-0.32
Ripk1	chr13	34119764	34125274	-2.04	-0.13
Ripk1	chr13	34125393	34126112	-1.53	-0.25
Dsp	chr13	38287718	38287838	-0.16	-0.42
Mylip	chr13	45503815	45506325	-3.05	0.42
Rnf144b	chr13	47338305	47340700	-0.89	-0.14
Rnf144b	chr13	47342821	47343181	-1.25	0.41
Syk	chr13	52678805	52706692	-1.23	-0.09
Syk	chr13	52727039	52733575	-0.39	-0.02
Syk	chr13	52737300	52738635	-0.88	-0.13
Syk	chr13	52738964	52742902	-3.69	-0.15
Syk	chr13	52743800	52743951	-0.57	-0.11
Faf2	chr13	54723144	54730781	-1.07	0.44
Faf2	chr13	54757693	54763778	-2.34	-0.16
Lpcat1	chr13	73651326	73651986	-1.03	-0.22
Erap1	chr13	74777319	74783985	-2.40	0.26
Arsb	chr13	94710635	94710756	-0.54	-0.12
Il6st	chr13	113254277	113255963	-0.34	-0.03
Il6st	chr13	113294105	113296021	-1.16	0.06
Il6st	chr13	113296618	113296769	-0.32	-0.02
Cdk13	chr13	17809653	17811597	-3.39	0.17
Cdk13	chr13	17858328	17896931	-2.82	-0.14
Hist1h3i	chr13	21875180	21875266	-1.25	-0.25
Hist1h3c	chr13	23837329	23837390	-0.45	-0.10
Serpinb1a	chr13	32934139	32934200	-0.26	1.00
Tubb2b	chr13	34219319	34219498	-1.13	2.05
Tubb2b	chr13	34220029	34220783	-1.25	-0.78
Txndc5	chr13	38592141	38592559	-3.04	0.23
Txndc5	chr13	38593006	38595325	-2.44	-0.12
Txndc5	chr13	38620243	38620304	-0.28	0.00
Hk3	chr13	55107346	55107674	-1.66	-0.13
Hk3	chr13	55122215	55122665	-0.41	-0.06
Rab24	chr13	55421264	55421501	-2.29	-0.29

Dok3	chr13	55628759	55629086	-1.35	-0.15
Ddx41	chr13	55637366	55637930	-0.30	0.01
Ctsl	chr13	64470393	64471614	-1.97	-0.09
Scamp1	chr13	94973830	94978130	-0.32	0.01
Pik3r1	chr13	102453956	102456264	-1.75	-0.18
Pik3r1	chr13	102478932	102525656	-1.06	0.31
Pik3r1	chr13	102527214	102538172	-0.33	-0.01
Map3k1	chr13	112545535	112546521	-0.47	-0.03
Map3k1	chr13	112566079	112599191	-0.72	-0.15
Flnb	chr14	8737140	8739780	-2.54	0.14
Flnb	chr14	8782901	8784071	-0.91	-0.06
Kctd6	chr14	9052713	9055605	-1.86	-0.26
Nkiras1	chr14	19111014	19112745	-0.48	0.11
Sec24c	chr14	21512398	21513205	-0.75	-0.07
Sec24c	chr14	21513563	21514072	-1.48	-0.13
Vcl	chr14	21850890	21851250	-2.10	0.22
Psmc6	chr14	45949498	45950514	-0.58	0.08
Pnp	chr14	51571126	51571721	-1.77	0.17
Irf9	chr14	56222821	56223430	-2.26	0.85
Irf9	chr14	56223549	56224030	-0.68	-0.24
Irf9	chr14	56227759	56228328	-3.64	0.43
Clu	chr14	66587437	66592220	-0.31	0.01
Clu	chr14	66594451	66594600	-0.64	0.12
Kbtbd7	chr14	79827126	79828716	-1.70	0.29
Ppp3cb	chr14	21365614	21365735	-1.14	-0.58
Camk2g	chr14	21554813	21555948	-0.30	0.00
Prkcd	chr14	31408808	31411699	-0.55	0.03
Prkcd	chr14	31411787	31411937	-0.29	-0.01
Tspan14	chr14	41720841	41721322	-0.75	-0.14
Ptk2b	chr14	66772123	66773020	-1.61	-0.10
Ptk2b	chr14	66899768	66899829	-0.51	0.21
Fbxl3	chr14	103482090	103488631	-0.81	-0.05
Fbxl3	chr14	103494436	103498143	-0.54	-0.05
Fbxl3	chr14	103498202	103498726	-1.82	-0.43
Fyb	chr15	6529870	6569669	-1.33	-0.07
Fyb	chr15	6584856	6590489	-0.34	-0.01
Rictor	chr15	6745928	6747254	-2.42	0.20
Myo10	chr15	25743096	25743426	-2.54	-0.31
Ncf4	chr15	78075240	78075447	-0.37	-0.03
Ncf4	chr15	78091438	78092951	-2.32	-0.15
Csf2rb	chr15	78156427	78166798	-0.34	0.02
Syngr1	chr15	79946545	79946695	-0.52	0.21
Rbx1	chr15	81305941	81306090	-0.83	-0.28

Ep300	chr15	81416643	81438767	-1.05	0.06
Ep300	chr15	81479273	81481819	-0.51	0.02
Xrcc6	chr15	81870184	81870514	-0.80	0.13
Ano6	chr15	95621273	95694678	-0.36	-0.03
Ano6	chr15	95802935	95805002	-0.88	-0.04
Tuba1c	chr15	98867757	98867967	-0.36	0.01
Atf1	chr15	100084825	100090754	-3.22	0.22
Mettl7a1	chr15	100143493	100143732	-0.42	0.13
Nckap1l	chr15	103328031	103328301	-2.08	-0.18
Skp2	chr15	9043329	9043540	-0.80	0.27
Skp2	chr15	9069207	9070167	-0.68	-0.14
Il7r	chr15	9437501	9438102	-0.75	0.21
Rnf19a	chr15	36170946	36173866	-0.40	0.03
Ywhaz	chr15	36723405	36723964	-0.95	0.41
Fbxo32	chr15	58011872	58012083	-0.36	-0.16
Fbxo32	chr15	58012410	58013069	-1.46	-0.50
Fbxo32	chr15	58046223	58046433	-2.16	-1.12
Ptk2	chr15	73035594	73039802	-0.41	0.02
Ptk2	chr15	73190599	73190660	-0.64	0.47
Dgat1	chr15	76342068	76342248	-0.53	0.16
Csf2rb2	chr15	78114588	78116449	-1.22	-0.15
Pla2g6	chr15	79116776	79117224	-0.88	-0.17
Nfam1	chr15	82828225	82828854	-2.38	-0.27
Nfam1	chr15	82829422	82831880	-1.58	-0.14
Mapk12	chr15	88961133	88961374	-0.45	2.32
Mapk11	chr15	88973270	88973361	-1.33	1.55
Mapk11	chr15	88973776	88973926	-0.54	0.57
Arsa	chr15	89303025	89303891	-1.14	0.17
Arsa	chr15	89305958	89306615	-3.10	-0.44
Col2a1	chr15	97806360	97806570	-1.38	2.81
Asb8	chr15	97965939	97971774	-0.39	0.02
Racgap1	chr15	99470224	99482052	-1.10	-0.20
Itgb7	chr15	102046603	102047016	-0.40	-0.02
Itgb7	chr15	102057938	102062366	-1.68	0.23
Gpr84	chr15	103138665	103140869	-0.35	-0.01
Hmox2	chr16	4764613	4765838	-2.38	-0.15
Mgrn1	chr16	4936923	4937342	-0.36	0.02
Mapk1	chr16	17038671	17038912	-0.29	0.00
Snap29	chr16	17427202	17428502	-0.76	0.12
Crkl	chr16	17452109	17484582	-0.30	0.00
Klhl22	chr16	17771956	17777358	-1.71	-0.20
Klhl22	chr16	17778345	17778466	-0.64	0.80
Ap2m1	chr16	20535576	20537801	-0.37	-0.06

Psmd2	chr16	20651724	20652324	-3.25	-0.17
Eif4g1	chr16	20679306	20680085	-1.42	0.04
Il1rap	chr16	26701407	26701468	-0.26	0.35
Il1rap	chr16	26714958	26715289	-1.23	0.49
Itgb5	chr16	33829750	33830049	-3.12	-1.09
Itgb5	chr16	33919922	33920251	-0.33	-0.01
Itgb5	chr16	33947843	33949275	-0.39	-0.02
Cd80	chr16	38473975	38479185	-1.67	-0.16
Cd80	chr16	38486416	38486895	-3.14	-0.36
Cd80	chr16	38494596	38494776	-0.67	0.24
Cd200r1	chr16	44794312	44794552	-0.73	-0.27
Cd200r4	chr16	44811897	44820870	-1.68	-0.99
Cd200r2	chr16	44915270	44915894	-0.26	-0.01
Cblb	chr16	52166229	52166380	-0.94	0.44
Cblb	chr16	52204565	52205105	-0.38	-0.05
Il10rb	chr16	91425363	91425662	-1.49	-0.16
Ifnar1	chr16	91506780	91506871	-0.57	0.18
Ifnar1	chr16	91507110	91507680	-1.67	0.21
Ifngr2	chr16	91563142	91563652	-1.48	-0.10
Crebbp	chr16	4154961	4213404	-1.70	-0.10
Socs1	chr16	10783960	10784764	-0.35	0.15
Socs1	chr16	10784822	10784882	-0.42	-1.26
Socs1	chr16	10784970	10785540	-0.17	-0.27
Cpped1	chr16	11804530	11827393	-0.72	0.06
Cpped1	chr16	11828288	11884362	-3.35	0.51
Cpped1	chr16	11887807	11909516	-2.19	-0.53
Pak2	chr16	32019862	32021909	-1.43	0.09
Dtx3l	chr16	35929143	35932046	-2.43	0.35
Dtx3l	chr16	35932375	35933483	-0.33	0.01
Cd86	chr16	36604193	36604552	-0.46	-0.35
Cd86	chr16	36620796	36620917	-0.87	1.18
Atp6v1a	chr16	44116916	44139132	-0.90	0.12
App	chr16	85025115	85043863	-0.75	-0.03
App	chr16	85043922	85056713	-2.06	-0.14
App	chr16	85120877	85173892	-2.41	-0.23
Ltn1	chr16	87415807	87416587	-2.10	0.18
Cct8	chr16	87496053	87496114	-0.58	0.12
Brwd1	chr16	96223575	96225491	-0.35	0.01
Brwd1	chr16	96226300	96226615	-0.52	-1.11
Brwd1	chr16	96229480	96229661	-1.19	0.48
Rps6ka2	chr17	7374463	7374584	-0.75	1.07
Rps6ka2	chr17	7507034	7507424	-0.64	-0.40
Itpr3	chr17	27258538	27258808	-0.49	0.07

Mapk14	chr17	28828286	28828675	-2.21	-0.37
H2-DMa	chr17	34272202	34272620	-1.64	0.61
Tap1	chr17	34324500	34325281	-0.31	0.01
Tap1	chr17	34333539	34333870	-2.43	0.51
Psmb8	chr17	34335288	34336214	-1.01	-0.16
Tap2	chr17	34341423	34342567	-0.60	-0.10
Tap2	chr17	34352342	34353206	-0.31	-0.01
H2-Ob	chr17	34378095	34378156	-0.21	1.00
Neu1	chr17	35071427	35072869	-3.32	-0.17
H2-D1	chr17	35403787	35404234	-3.70	-0.07
H2-Q6	chr17	35565949	35566720	-0.96	0.29
H2-Q7	chr17	35576925	35577015	-0.56	-1.32
H2-Q10	chr17	35607880	35610039	-0.32	0.07
Nfkbie	chr17	45692664	45695502	-1.11	-0.14
Nfkbie	chr17	45697103	45697389	-0.61	-0.25
Nfkbie	chr17	45697447	45700117	-0.90	-0.09
Cul7	chr17	46787586	46787707	-0.19	0.03
Cul7	chr17	46788456	46789468	-0.30	-0.09
Trem1	chr17	48371921	48385139	-2.64	1.06
Treml2	chr17	48438863	48439774	-1.94	2.67
Treml2	chr17	48449100	48449610	-0.28	0.02
Ebi3	chr17	56095665	56096445	-1.01	-0.13
Ypel5	chr17	73198138	73198199	-1.90	0.70
Ypel5	chr17	73198557	73198888	-0.34	0.02
Ppm1b	chr17	85357641	85364400	-0.47	0.19
Ppm1b	chr17	85393024	85402617	-0.51	0.03
Prkce	chr17	86567124	86568262	-1.54	-0.27
Prkce	chr17	87054027	87055374	-0.46	0.07
Socs5	chr17	87507287	87507348	-0.71	-0.42
Socs5	chr17	87520106	87534893	-3.68	0.22
Dynlt1f	chr17	6306369	6306549	-0.33	-0.13
Igf2r	chr17	12876409	12876980	-1.19	-0.09
Igf2r	chr17	12962421	12962572	-0.60	0.17
Fpr1	chr17	18013552	18013905	-0.29	0.82
Fpr1	chr17	18014257	18014522	-0.62	3.00
Pdpk1	chr17	24215583	24216033	-2.93	0.32
Atp6v0c	chr17	24306196	24306374	-0.33	0.02
Ccnf	chr17	24360773	24360864	-1.19	-0.44
Stub1	chr17	25967580	25967909	-2.17	-0.16
Stub1	chr17	25969947	25970306	-0.38	0.08
H2-K1	chr17	34136362	34136423	-0.41	0.19
Hspa1b	chr17	35093851	35095346	-0.55	0.06
Hspa1b	chr17	35095554	35096183	-2.02	0.44

Hspa1a	chr17	35107225	35107435	-1.72	-4.26
H2-T23	chr17	36169099	36248130	-0.77	-0.09
Trim39	chr17	36397225	36398520	-1.76	0.23
Trim39	chr17	36400794	36404080	-1.08	-0.35
Enpp4	chr17	44234667	44234818	-0.51	0.64
Enpp4	chr17	44235056	44235147	-0.58	-1.68
Enpp4	chr17	44236285	44239353	-3.17	0.82
Klc4	chr17	46781224	46782971	-0.41	0.07
Ppp2r5d	chr17	46820028	46820862	-1.04	-0.11
Ppp2r5d	chr17	46837029	46841951	-0.31	0.02
Ubr2	chr17	47065869	47067399	-0.68	-0.05
Ubr2	chr17	47147240	47147481	-0.50	-0.08
Tnfsf14	chr17	57329333	57330291	-0.25	0.10
Pja2	chr17	64658035	64660528	-0.54	-0.04
Vapa	chr17	65962685	65962865	-2.27	-0.24
Rab31	chr17	66001065	66001632	-0.47	0.02
Rab31	chr17	66003418	66017287	-0.77	0.04
Rab31	chr17	66122031	66122092	-0.23	-0.05
Xdh	chr17	74238633	74238694	-0.24	-0.06
Eif2ak2	chr17	79252515	79254306	-1.48	0.13
Eif2ak2	chr17	79277576	79281912	-0.88	0.23
Sos1	chr17	80797041	80799097	-0.34	-0.01
Sos1	chr17	80832979	80834320	-1.17	0.18
Sos1	chr17	80879313	80879554	-0.43	-0.33
Calm2	chr17	87835011	87846216	-3.10	-0.20
Fbxo11	chr17	88390945	88391656	-0.52	0.03
Colec12	chr18	9707645	9840283	-0.84	-0.07
Colec12	chr18	9848284	9859890	-1.40	-0.04
Ube2d2a	chr18	35931242	35931392	-1.92	-0.45
Ube2d2a	chr18	35965360	35965570	-1.78	0.47
Rnf14	chr18	38467720	38467990	-0.45	0.07
Dctn4	chr18	60686409	60686590	-0.35	0.21
Dctn4	chr18	60716345	60716496	-0.75	-0.18
Camk2a	chr18	61144487	61145325	-0.34	-0.05
Csf1r	chr18	61265471	61269328	-0.53	0.02
Csf1r	chr18	61290403	61290793	-1.71	-0.05
Tubb6	chr18	67550384	67552330	-3.31	-0.30
Mex3c	chr18	73732718	73749293	-0.62	-0.08
Map3k8	chr18	4348929	4352951	-0.67	0.15
Rock1	chr18	10181251	10181790	-2.15	-0.22
Cdc23	chr18	34792931	34793291	-2.74	-0.56
Cdc23	chr18	34793470	34793621	-0.73	-0.23
Tmem173	chr18	35893749	35894288	-1.56	-0.19

Trim36	chr18	46326929	46328816	-0.84	0.34
Ticam2	chr18	46718782	46719052	-0.29	0.01
Nfatc1	chr18	80803872	80832687	-3.26	-0.24
Nfatc1	chr18	80846262	80846653	-1.93	0.30
Nfatc1	chr18	80879052	80904894	-0.33	-0.01
Unc93b1	chr19	3935215	3935335	-0.82	-0.10
Sptbn2	chr19	4744084	4745130	-1.43	-0.66
Rela	chr19	5637578	5637639	-0.26	0.03
Jak2	chr19	29385493	29386732	-1.43	0.23
Cd274	chr19	29441927	29460289	-0.50	0.04
Pdcd1lg2	chr19	29545031	29545330	-0.38	0.49
Pgam1	chr19	41986449	41990281	-2.13	-0.60
Btrc	chr19	45604207	45604268	-0.27	-0.42
Nfkb2	chr19	46379346	46380320	-1.80	0.46
Fbxl15	chr19	46403619	46404936	-0.68	0.11
Tcirg1	chr19	3906829	3907103	-1.92	-0.32
Aldh3b1	chr19	3913726	3913875	-2.12	-0.31
Tbc1d10c	chr19	4184415	4184833	-1.55	1.33
Peli3	chr19	4932182	4932600	-0.82	2.08
Klc2	chr19	5117426	5118348	-2.22	-0.46
Sipa1	chr19	5651184	5651544	-1.77	0.18
Sipa1	chr19	5652777	5652838	-0.60	-0.18
Sipa1	chr19	5653976	5655148	-1.43	-0.12
Sipa1	chr19	5659486	5662646	-2.21	-0.13
Ppp2r5b	chr19	6227915	6228302	-0.46	0.08
Stx3	chr19	11893622	11893893	-0.92	0.35
Dtx4	chr19	12543910	12544151	-1.35	-0.17
Chuk	chr19	44181846	44181937	-0.41	-0.11
Fbxw4	chr19	45652836	45653256	-0.43	-0.03
Actr1a	chr19	46452405	46452913	-1.68	-0.11
Ddx3x	chrX	12858147	12861347	-0.80	0.06
Il13ra1	chrX	33695583	33709519	-2.22	0.35
Ube2a	chrX	34414360	34414479	-0.33	-0.03
Ikbkg	chrX	71675074	71678148	-0.70	-0.30
Tab3	chrX	82860462	82860673	-2.95	0.81
Tab3	chrX	82875864	82876014	-3.15	1.28
Kif4	chrX	97921923	97922431	-2.34	-0.35
Ap1s2	chrX	160347091	160347182	-0.27	0.01
Was	chrX	7667496	7667617	-0.32	0.03
Cfp	chrX	20508559	20508650	-1.66	0.59
Rap2c	chrX	48359709	48370808	-2.32	0.21
Irak1	chrX	71260386	71261911	-0.47	0.03
Dnase1l1	chrX	71522609	71526996	-1.08	0.25

Eda2r	chrX	94533994	94534384	-0.94	0.65
Pja1	chrX	96661669	96661909	-3.01	0.59
Pja1	chrX	96662087	96663849	-0.78	0.06
Magt1	chrX	103207027	103207148	-2.30	0.50
Gla	chrX	131135394	131135544	-0.33	0.04
Siah1b	chrX	160509579	160510081	-0.42	0.07
Tlr8	chrX	163680663	163681289	-0.60	0.40
Tlr8	chrX	163681408	163681796	-2.38	1.46
Tlr8	chrX	163681884	163682362	-1.04	0.86
Tlr7	chrX	163745697	163746354	-3.29	0.14
Tnfrsf1b	chr4	144836594	144836774	-2.39	-0.60
Stim1	chr7	109416755	109416875	-0.47	-0.21
Dynll2	chr11	87794645	87799923	-0.49	0.03
Diaph1	chr18	38094885	38095065	-0.51	-0.12

Generic transcription pathway

gene_name	chr	chromStart	chromEnd	diff.lg.p	diff.log2.fc
Cnot11	chr1	39592727	39593027	-0.49	-0.09
Cnot11	chr1	39603302	39603662	-1.63	-0.30
Cnot9	chr1	74575438	74575798	-2.06	-0.28
Psmd1	chr1	87961193	87961313	-0.67	0.21
Ing5	chr1	95714871	95715861	-2.79	-0.31
Ccnt2	chr1	129670740	129670861	-1.38	0.49
Ccnt2	chr1	129698762	129700591	-0.54	0.03
Rbbp5	chr1	134394590	134400982	-1.17	0.15
Rbbp5	chr1	134401131	134401312	-0.34	-0.03
Kdm5b	chr1	136514466	136514617	-0.68	0.28
Kdm5b	chr1	136521310	136521760	-2.33	0.45
Kdm5b	chr1	136525288	136527418	-0.37	-0.03
Kdm5b	chr1	136527777	136528227	-1.23	0.27
Nr1i3	chr1	173148035	173148485	-1.67	-0.41
Nr1i3	chr1	173148844	173149234	-0.72	-0.23
Exo1	chr1	177829710	177831526	-1.63	-0.39
Exo1	chr1	177838863	177839044	-0.20	0.03
Exo1	chr1	177839162	177839433	-0.27	0.29
Parp1	chr1	182499105	182503843	-1.60	-0.27
Trp53bp2	chr1	184371133	184371894	-0.41	-0.09
Trp53bp2	chr1	184376642	184379234	-0.81	0.12
Trp53bp2	chr1	184389061	184392388	-0.44	-0.06
Gls	chr1	52244565	52244656	-2.44	0.47
Sumo1	chr1	59696455	59696989	-1.11	-0.09
Bard1	chr1	71121331	71122020	-0.53	0.08
Btg2	chr1	135971471	135971532	-0.90	0.83
Btg2	chr1	135973595	135975732	-0.89	-0.05

Rnf2	chr1	153318408	153318528	-0.89	0.28
Smyd2	chr1	191704578	191706000	-1.27	0.17
Bmi1	chr2	18605667	18606468	-0.89	0.08
Rxra	chr2	27597444	27604173	-0.47	-0.11
Rxra	chr2	27617342	27618510	-1.22	-0.15
Tsc1	chr2	28531258	28532060	-2.14	0.31
Tsc1	chr2	28541313	28542943	-1.55	-0.18
Tsc1	chr2	28546177	28546268	-0.26	0.00
Abl1	chr2	31544075	31551320	-0.40	0.02
Abl1	chr2	31652573	31658159	-1.65	-0.10
Abl1	chr2	31658367	31658428	-1.12	-0.61
Abl1	chr2	31659237	31659477	-0.56	0.58
Mapkap1	chr2	34479093	34479423	-1.30	0.22
Psmc3	chr2	90894439	90895182	-1.38	-0.10
Mga	chr2	119698286	119698347	-0.18	0.00
Mga	chr2	119767083	119771919	-0.49	0.05
Mga	chr2	119787692	119790793	-2.58	-0.17
Itch	chr2	154959216	154959307	-0.25	0.00
Itch	chr2	155050132	155050373	-0.80	-0.16
Phf20	chr2	156092990	156093201	-1.93	0.33
Phf20	chr2	156113573	156113634	-1.16	0.44
Tgif2	chr2	156679040	156679219	-0.58	0.85
Tgif2	chr2	156679307	156679813	-0.75	-0.28
Src	chr2	157295226	157296471	-0.85	0.32
Trp53rkb	chr2	166619352	166619563	-0.37	0.13
Trp53rkb	chr2	166620915	166621755	-2.59	-0.52
Gata3	chr2	9779567	9784722	-1.35	0.40
Gata3	chr2	9784840	9796310	-0.60	0.24
Taf3	chr2	9837317	9964072	-0.79	0.05
Pip4k2a	chr2	18765497	18769115	-1.74	0.11
Ehmt1	chr2	24646742	24647852	-1.08	-0.13
Ehmt1	chr2	24733063	24775080	-2.58	0.23
Notch1	chr2	26314049	26314319	-1.37	1.42
Notch1	chr2	26315036	26315097	-0.53	-1.83
Notch1	chr2	26315275	26315486	-0.62	0.91
Notch1	chr2	26316053	26316413	-0.28	0.10
Notch1	chr2	26323937	26324117	-0.52	0.80
Notch1	chr2	26336689	26337202	-0.49	1.03
Notch1	chr2	26339865	26340015	-0.40	0.61
Psmd5	chr2	34707993	34709794	-1.37	-0.17
Psmd5	chr2	34722532	34726482	-1.60	-0.40
Psmb7	chr2	38498946	38499426	-1.35	-0.19
Nr6a1	chr2	38781663	38781753	-0.49	-0.29

Nr4a2	chr2	56960175	56961102	-1.17	-0.25
Nr4a2	chr2	56964099	56965537	-0.49	-0.07
Nr4a2	chr2	56967204	56967355	-0.52	0.68
Zfp661	chr2	127402341	127403565	-2.72	0.50
Pcna	chr2	132078555	132078916	-3.65	-0.19
Psmf1	chr2	151543111	151543232	-0.83	-0.47
Psmf1	chr2	151543649	151544068	-0.81	-0.19
Zfp334	chr2	165203294	165203715	-1.00	0.46
Zfp334	chr2	165205211	165206201	-0.49	0.09
Zfp334	chr2	165206829	165207339	-2.14	0.67
Trp53rka	chr2	165315878	165318814	-0.78	-0.11
Taf4	chr2	179647358	179656708	-0.54	-0.05
Skil	chr3	30994042	30997382	-1.57	0.11
Gatad2b	chr3	90127990	90145879	-0.41	-0.02
Gatad2b	chr3	90155787	90155878	-3.11	-0.35
Rorc	chr3	94201354	94201475	-0.30	1.58
Hist2h2be	chr3	96025043	96025490	-1.83	0.52
Hist2h2be	chr3	96026738	96027215	-1.30	-0.39
Notch2	chr3	97935343	97941284	-2.25	-0.13
Trim33	chr3	103148255	103148495	-1.27	0.15
Trim33	chr3	103156073	103158203	-1.33	0.10
Trim33	chr3	103158830	103159400	-0.30	0.00
Ube2d3	chr3	135101656	135102228	-3.24	-0.27
Phc3	chr3	30805887	30813151	-0.57	-0.08
Phc3	chr3	30864703	30868337	-0.42	0.12
Elf2	chr3	51059646	51060809	-1.08	-0.08
Elf2	chr3	51061575	51064964	-0.90	-0.11
Maml3	chr3	51491863	51496200	-1.51	0.17
Maml3	chr3	51501603	51600338	-0.59	-0.42
Maml3	chr3	51907609	51907880	-3.24	0.71
Maml3	chr3	51907939	51908209	-2.93	0.70
Maml3	chr3	51908358	51908838	-1.96	0.46
Lamtor2	chr3	88353798	88356849	-1.88	-0.11
Psmd4	chr3	94836626	94836924	-1.79	-0.12
Psmd4	chr3	94839816	94846467	-2.16	-0.13
Hist2h3c2	chr3	96042454	96042544	-0.57	0.69
Hist2h2aa1	chr3	96044071	96044216	-1.04	1.17
Prmt6	chr3	110050616	110051517	-1.67	-0.58
Prmt6	chr3	110052356	110053916	-3.07	-0.18
Nr4a3	chr4	48058191	48058281	-0.24	-0.14
Nr4a3	chr4	48061990	48065027	-1.94	-0.50
Nr4a3	chr4	48083931	48096224	-1.06	-0.81
Zfp189	chr4	49542890	49543460	-2.62	0.62

Rraga	chr4	86221606	86222293	-0.53	0.03
Tal1	chr4	114737352	114741762	-1.59	-0.29
Tal1	chr4	114741970	114742061	-0.45	-0.22
Med8	chr4	118087440	118087618	-2.77	0.38
Scmh1	chr4	120201875	120202085	-2.07	0.58
Rragc	chr4	123597654	123598314	-1.34	0.21
Rragc	chr4	123612802	123613821	-0.41	-0.01
Rragc	chr4	123614089	123614180	-1.02	0.20
Phc2	chr4	128404910	128405061	-1.05	-0.71
Rpa2	chr4	132333796	132334035	-1.34	-0.32
Nr0b2	chr4	133112127	133112363	-0.61	0.57
Zfp46	chr4	135846237	135848631	-0.51	-0.05
Mtor	chr4	147822690	147826440	-1.12	-0.33
Mtor	chr4	147930392	147931764	-0.80	0.10
Zfp691	chr4	118842660	118845711	-0.35	0.03
Zfp69	chr4	120602801	120603909	-2.55	0.49
Sfn	chr4	133156530	133158083	-0.66	0.16
Eloa	chr4	135561252	135561580	-1.14	0.19
Eloa	chr4	135565701	135577564	-2.32	0.10
Ski	chr4	154529765	154533533	-2.17	0.10
Ski	chr4	154533945	154596644	-1.11	-0.08
Cdk6	chr5	3519406	3520884	-0.47	0.04
Kmt2e	chr5	22940246	22940516	-2.82	0.51
Kmt2e	chr5	23002909	23005273	-0.76	0.07
Kmt2e	chr5	23007381	23008999	-1.16	0.08
Ywhah	chr5	33361494	33361614	-0.40	0.10
Ywhah	chr5	33369269	33370047	-0.64	0.02
Rbpj	chr5	54044845	54045146	-0.26	0.54
Zfp605	chr5	110556631	110557140	-1.63	0.56
Zfp605	chr5	110557318	110558156	-0.32	-0.03
Triap1	chr5	115791255	115793471	-0.71	-0.07
Rnf34	chr5	123273869	123314504	-1.72	0.21
Psmd9	chr5	123678228	123684616	-1.07	0.18
Kmt5a	chr5	124911176	124911327	-0.89	-0.12
Gtf2h3	chr5	125046222	125046313	-0.93	-0.48
Zfp664	chr5	125343194	125366362	-1.24	-0.09
Zfp664	chr5	125366721	125367081	-0.31	0.01
Rfc2	chr5	135073840	135074198	-0.38	-0.03
Zkscan1	chr5	138526311	138534046	-0.73	0.23
Zkscan1	chr5	138541914	138542485	-0.51	0.05
Zkscan1	chr5	138542723	138543984	-1.79	0.16
Zkscan1	chr5	138544222	138545962	-0.77	-0.07
Zfp12	chr5	144005811	144007881	-0.65	-0.08

Zfp12	chr5	144007940	144008390	-0.69	-0.23
Zkscan5	chr5	145981691	145982470	-2.39	0.63
Zfp655	chr5	145992583	145994463	-2.43	-0.45
Zfp655	chr5	146004460	146006138	-2.25	0.12
Cdk8	chr5	147042885	147071118	-2.04	-0.41
Smarcd3	chr5	24100691	24101019	-0.21	0.00
Prkag2	chr5	24605830	24606400	-1.25	0.18
Kmt2c	chr5	24790108	24790979	-0.87	0.09
Prkab1	chr5	116463747	116468029	-0.50	-0.04
Prkab1	chr5	116474109	116474378	-0.66	-0.12
Ptpn11	chr5	121581827	121582307	-2.13	0.25
Ptpn11	chr5	121583292	121587519	-0.51	-0.03
Ncor2	chr5	125514394	125514836	-0.46	-0.06
Ncor2	chr5	125538274	125548306	-0.47	0.04
Ncor2	chr5	125659463	125659584	-0.26	-0.17
Zfp11	chr5	130162143	130162980	-1.02	0.38
Zfp11	chr5	130164055	130166739	-0.47	0.31
Zfp113	chr5	138585511	138586171	-1.24	-0.27
Zfp113	chr5	138586380	138586650	-1.50	0.58
Smurf1	chr5	145637693	145638023	-0.74	-0.29
Smurf1	chr5	145638352	145638682	-1.09	-0.34
Smurf1	chr5	145639310	145639491	-0.37	0.13
Zkscan14	chr5	145956021	145962515	-3.26	-0.45
Rfc3	chr5	152445398	152445668	-1.50	0.34
Zfp398	chr6	47785869	47790711	-0.90	0.31
Zfp398	chr6	47815946	47817117	-0.67	-0.11
Zfp282	chr6	47827703	47830376	-0.29	0.00
Zfp282	chr6	47854827	47855966	-3.17	-0.33
Zfp282	chr6	47856175	47856835	-1.42	-0.27
Zfp212	chr6	47870625	47876800	-0.60	0.18
Zfp212	chr6	47879122	47881565	-1.03	-0.20
Zfp775	chr6	48555624	48570026	-1.58	-0.60
Zfp775	chr6	48570354	48570923	-1.34	-0.72
Gata2	chr6	88154683	88155799	-3.13	-0.61
Nr2c2	chr6	92114256	92117902	-2.91	-0.32
Brpf1	chr6	113257160	113269353	-0.42	0.01
Chd4	chr6	125079888	125080399	-1.62	-0.07
Cdkn1b	chr6	134870898	134872390	-2.89	0.28
H2afj	chr6	136756828	136756889	-0.45	0.13
H2afj	chr6	136757307	136757577	-1.31	-0.28
Hipk2	chr6	38647868	38666025	-2.58	-0.11
Hipk2	chr6	38697991	38787047	-0.54	-0.02
Hipk2	chr6	38825859	38826040	-2.58	-0.39

Zfp786	chr6	47769651	47770337	-0.42	-0.30
Zfp786	chr6	47770485	47771170	-0.67	0.66
Zfp786	chr6	47771318	47771676	-0.36	-0.40
Zfp746	chr6	48013500	48016503	-1.38	-0.15
Zfp746	chr6	48033553	48036532	-0.55	-0.18
Gadd45a	chr6	66985178	66987371	-2.48	-0.22
Rybp	chr6	100182274	100183082	-1.03	0.42
Zfp248	chr6	118378764	118380402	-3.16	0.57
Phc1	chr6	122273438	122273618	-1.06	0.49
Phc1	chr6	122275560	122275830	-0.30	-0.03
Tigar	chr6	127037498	127038307	-0.73	0.09
Ccnd2	chr6	127079556	127080245	-1.36	-0.18
Rhno1	chr6	128307315	128309210	-1.80	-0.15
Kras	chr6	145168783	145169023	-0.29	0.00
Zfp667	chr7	6256313	6256524	-1.53	1.14
Zfp667	chr7	6256792	6256942	-0.28	-0.11
Zfp667	chr7	6257719	6257870	-0.76	0.76
Zfp78	chr7	6331042	6331103	-0.19	0.59
Zfp78	chr7	6331550	6332298	-1.80	0.98
Zfp28	chr7	6344966	6347349	-0.99	0.23
Zfp110	chr7	13434054	13434502	-0.38	-0.03
Zfp324	chr7	13555672	13556391	-2.57	-0.89
Zfp324	chr7	13556928	13557676	-0.54	-0.23
Zfp446	chr7	13563376	13563437	-0.26	-0.09
Zfp446	chr7	13564395	13564546	-0.27	-0.02
Zfp446	chr7	13567226	13568306	-0.59	-0.12
Zfp446	chr7	13568485	13569026	-1.73	-0.53
Ppp1r13l	chr7	19956867	19960636	-1.70	-0.27
Ercc2	chr7	19979441	19981041	-2.11	-0.19
Zfp180	chr7	24882912	24891576	-1.89	-0.12
Zfp112	chr7	24910123	24910454	-0.36	-0.31
Zfp112	chr7	24910962	24911533	-0.46	0.24
Zfp112	chr7	24912371	24912551	-1.18	1.63
Zfp235	chr7	24921520	24921581	-0.55	1.10
Zfp235	chr7	24925681	24925772	-0.67	0.72
Zfp235	chr7	24926760	24927241	-1.64	-0.63
Zfp235	chr7	24927899	24928200	-0.30	-0.12
Akt2	chr7	28422202	28423428	-1.06	0.15
Zfp30	chr7	30577491	30577582	-2.02	2.09
Zfp30	chr7	30577790	30578810	-2.01	0.66
Zfp790	chr7	30613525	30616012	-0.65	-0.06
Zfp420	chr7	30645086	30661309	-0.62	-0.06
Zfp382	chr7	30916575	30919873	-2.17	-0.19

Polr2i	chr7	31017332	31017772	-0.29	0.00
Zfp658	chr7	50827988	50828378	-0.42	0.12
Zfp719	chr7	50835075	50835135	-1.05	0.74
Zfp719	chr7	50841731	50846748	-2.68	0.18
Zfp819	chr7	50873172	50873292	-0.50	3.17
Gtf2h1	chr7	54051493	54051554	-1.82	-0.48
Zfp710	chr7	87169699	87227267	-2.58	-0.11
Zfp710	chr7	87231444	87231625	-0.38	0.06
Zfp710	chr7	87233516	87236264	-1.55	0.11
Lamtor1	chr7	109054380	109058211	-1.32	-0.28
Setd1a	chr7	134920932	134920993	-1.26	1.23
Setd1a	chr7	134927635	134930705	-0.77	0.04
Setd1a	chr7	134931750	134931998	-1.29	-0.20
Zik1	chr7	11075804	11075955	-0.39	-0.19
Zfp551	chr7	13000735	13000946	-0.79	1.17
Zfp94	chr7	25087170	25088874	-0.51	-0.11
Supt5	chr7	29116415	29116476	-0.45	0.06
Psmd8	chr7	29965393	29965573	-1.95	-0.13
Zfp940	chr7	30629168	30629499	-0.28	0.07
Zfp940	chr7	30629558	30629679	-0.68	0.98
Zfp940	chr7	30630277	30630518	-0.33	-0.19
Zfp940	chr7	30630847	30631058	-1.56	1.10
Zfp27	chr7	30678475	30680419	-0.34	0.01
Zfp27	chr7	30680747	30683850	-0.38	-0.03
Zfp14	chr7	30823226	30823406	-0.45	0.71
Zfp14	chr7	30823704	30823854	-0.93	1.66
Zfp14	chr7	30823972	30824152	-0.75	1.91
Zfp566	chr7	30862474	30863454	-2.54	0.50
Kmt2b	chr7	31368475	31373655	-3.36	-0.13
Ccne1	chr7	38883150	38883388	-1.47	-0.46
Zfp715	chr7	50552967	50556619	-3.08	0.17
Nr1h2	chr7	51806700	51806791	-0.86	0.19
Zfp473	chr7	51987480	51988021	-1.22	1.42
Zfp473	chr7	51988559	51988710	-0.18	-0.04
Zfp473	chr7	51989249	51989760	-1.53	1.19
Med25	chr7	52132313	52133597	-1.95	-0.23
Bax	chr7	52722059	52722238	-0.83	0.22
E2f8	chr7	56126083	56127247	-1.54	-0.22
Blm	chr7	87647232	87647711	-0.92	-0.15
Blm	chr7	87654407	87659523	-2.35	0.28
Eed	chr7	97125526	97129068	-0.79	-0.09
Eed	chr7	97129246	97129456	-2.79	0.48
Zfp747	chr7	134516408	134519504	-2.59	0.25

9130019O22Rik	chr7	134525922	134526311	-0.65	0.64
9130019O22Rik	chr7	134526817	134529144	-1.48	0.21
E430018J23Rik	chr7	134534349	134536898	-0.64	-0.15
E430018J23Rik	chr7	134536956	134537135	-0.28	-0.18
Zfp764	chr7	134547867	134549152	-2.70	-0.38
Zfp764	chr7	134550156	134550306	-0.31	-0.13
Zfp688	chr7	134562479	134563593	-0.33	-0.02
Zfp688	chr7	134565458	134565548	-0.42	-0.46
Zfp689	chr7	134587294	134588642	-1.03	-0.25
Zfp689	chr7	134592252	134592433	-0.97	0.50
Zfp668	chr7	135009321	135009472	-0.34	-0.12
Pidd1	chr7	148624413	148625004	-2.27	-0.57
Pidd1	chr7	148628991	148629907	-3.39	-1.06
Polr2l	chr7	148659279	148661052	-0.40	0.16
Ccnd1	chr7	152118441	152118622	-0.78	-0.05
Ccnd1	chr7	152125529	152125830	-0.84	0.07
Tfdp1	chr8	13377166	13377345	-1.90	-0.26
Tfdp1	chr8	13377582	13377702	-2.49	-0.30
Kat6a	chr8	24040729	24043035	-1.41	0.13
Kat6a	chr8	24046098	24051460	-0.39	-0.01
Ppp2cb	chr8	34710092	34710330	-0.74	-0.58
Nr2c2ap	chr8	72655231	72655498	-2.58	0.60
Nr2c2ap	chr8	72657115	72657470	-1.15	-0.17
Ell	chr8	73063573	73102761	-1.80	0.39
Ell	chr8	73105671	73115739	-2.29	0.17
Sin3b	chr8	75247186	75248032	-0.43	0.05
Sin3b	chr8	75273726	75273996	-2.25	0.63
Sin3b	chr8	75280901	75281383	-2.17	-0.52
Nr3c2	chr8	79432284	79432583	-1.74	2.06
Nr3c2	chr8	79433507	79433836	-1.15	2.46
Polr2c	chr8	97387425	97387634	-0.99	-0.15
Cbfb	chr8	107694603	107694841	-1.27	-0.20
Zfp90	chr8	108948628	108949645	-2.85	0.58
Zfp1	chr8	114190002	114194792	-0.82	0.10
Banp	chr8	124474462	124474523	-0.29	0.19
Banp	chr8	124549891	124550071	-0.27	0.02
Banp	chr8	124550280	124550791	-1.64	0.85
Banp	chr8	124550969	124551120	-0.31	-0.26
Banp	chr8	124551299	124552918	-1.18	0.43
Zfpm1	chr8	124806220	124856010	-0.73	0.11
Zfpm1	chr8	124858912	124861147	-3.23	0.22
Ash2l	chr8	26950536	26958082	-3.54	-0.43
Wrn	chr8	34346284	34346854	-0.31	-0.02

Cnot7	chr8	41592761	41593001	-1.13	0.18
Ing2	chr8	48753850	48754631	-3.25	-0.30
Gatad2a	chr8	72459689	72459840	-1.48	-0.16
Gatad2a	chr8	72497999	72498150	-0.82	-0.30
Gatad2a	chr8	72519948	72520248	-0.90	-0.33
Med26	chr8	75019172	75072090	-1.92	-0.15
Psmb10	chr8	108461631	108461997	-1.25	-0.38
Maml2	chr9	13510263	13511312	-0.79	0.20
Pin1	chr9	20456573	20459918	-2.86	-0.44
Smarca4	chr9	21505619	21507475	-1.36	0.14
Zfp202	chr9	40014929	40015928	-0.32	0.03
Zfp202	chr9	40018505	40020086	-3.02	0.31
Usp2	chr9	43883576	43884236	-2.87	2.50
Usp2	chr9	43902032	43902332	-0.41	-0.10
H2afx	chr9	44143446	44144156	-2.11	-0.11
Ppp2r1b	chr9	50687990	50688710	-0.88	-0.11
Ppp2r1b	chr9	50701016	50701705	-0.62	0.10
Psma4	chr9	54805658	54805748	-1.39	0.21
Sin3a	chr9	56973115	56975576	-0.47	0.04
Rora	chr9	69226655	69226835	-0.29	0.06
Atr	chr9	95765519	95765729	-0.56	0.14
Zkscan7	chr9	122797636	122797935	-0.27	-0.02
Zkscan7	chr9	122803837	122804973	-0.35	0.05
Med17	chr9	15066624	15066715	-0.36	-0.07
Zfp558	chr9	18260716	18261406	-1.01	0.57
Zfp426	chr9	20273262	20273323	-0.75	2.21
Zfp426	chr9	20274940	20276201	-1.15	0.20
Zfp266	chr9	20304149	20306501	-1.23	-0.14
Kmt2a	chr9	44615605	44617182	-1.02	0.14
Kmt2a	chr9	44650695	44650756	-3.48	0.90
Atm	chr9	53247473	53248264	-1.08	0.14
Pml	chr9	58067532	58068881	-1.38	-0.21
Pml	chr9	58077807	58077868	-0.49	0.22
Pml	chr9	58080966	58094982	-3.67	-0.47
Pml	chr9	58097382	58097563	-0.75	-0.30
Smad3	chr9	63605680	63605801	-0.63	0.71
Smad3	chr9	63605729	63605891	-0.25	0.23
Rnf111	chr9	70274585	70277390	-1.13	-0.14
Tcf12	chr9	71693887	71694367	-2.25	0.19
Tcf12	chr9	71958831	71958922	-0.41	-0.08
Tcf12	chr9	71959385	71959626	-1.17	-0.25
Atrip	chr9	108960445	108961754	-0.40	-0.01
Atrip	chr9	108967670	108968495	-0.58	0.09

Cnot10	chr9	114541840	114549228	-0.65	0.12
Sgk1	chr10	21718603	21718843	-0.48	0.25
Sesn1	chr10	41530409	41607263	-1.97	-0.34
Sesn1	chr10	41627317	41627765	-1.28	-0.38
Dna2	chr10	62436065	62436156	-0.32	-0.08
Stk11	chr10	79579091	79588239	-2.17	0.17
Stk11	chr10	79592650	79593215	-0.48	-0.05
Txnrd1	chr10	82296755	82323149	-0.66	-0.19
E2f7	chr10	110216069	110216279	-0.34	0.10
Smarcc2	chr10	127920231	127921148	-2.34	0.28
Smarcc2	chr10	127924513	127924663	-1.10	-0.30
Smarcc2	chr10	127926093	127926303	-3.40	0.46
Ddit4	chr10	59412599	59412718	-2.71	0.89
Ddit4	chr10	59412895	59414518	-0.91	-0.09
Nrbf2	chr10	66729853	66730867	-0.88	0.10
Ube2d1	chr10	70718055	70718592	-2.86	0.34
Smarcb1	chr10	75359661	75359780	-2.54	-0.51
Polr2e	chr10	79498845	79498935	-0.48	-0.07
Polr2e	chr10	79501286	79502404	-0.85	-0.12
Tcf3	chr10	79872386	79873344	-0.38	0.02
Cradd	chr10	94637889	94638400	-0.76	0.21
Cradd	chr10	94785366	94786140	-0.40	0.11
Cnot2	chr10	115922216	115922606	-1.24	0.23
Cnot2	chr10	115922874	115923115	-0.75	0.16
Yeats4	chr10	116652435	116652884	-0.66	0.08
Mdm2	chr10	117126799	117129688	-0.41	0.01
Dyrk2	chr10	118296404	118305870	-2.04	-0.14
Pip4k2c	chr10	126635286	126635944	-0.82	0.09
Pip4k2c	chr10	126636701	126637297	-0.51	-0.06
Cdk2	chr10	128135891	128136071	-0.28	0.00
Psme4	chr11	30671774	30672135	-0.53	-0.17
Med7	chr11	46250448	46250688	-0.94	-0.27
Ppp2ca	chr11	51935449	51935836	-0.93	0.05
Cnot8	chr11	57930958	57931228	-0.69	-0.18
Zkscan6	chr11	65636352	65642412	-0.30	0.00
Trp53	chr11	69393860	69394608	-1.25	-0.24
Suz12	chr11	79843530	79845978	-3.16	0.17
Psmd11	chr11	80285574	80285635	-1.26	-0.32
Taf15	chr11	83286609	83286700	-0.52	-0.08
Taf15	chr11	83318239	83318300	-0.54	0.18
Ppm1d	chr11	85124755	85125383	-3.11	-0.49
Ppm1d	chr11	85140408	85140529	-0.38	0.08
Supt4a	chr11	87557029	87557119	-0.49	0.20

Cdk12	chr11	98064618	98065548	-1.86	0.10
Cdk12	chr11	98071707	98072546	-1.51	0.09
Cdk12	chr11	98104823	98107067	-2.98	0.25
Psmd3	chr11	98556993	98557203	-2.02	-0.21
Thra	chr11	98603186	98617477	-0.48	0.09
Thra	chr11	98625080	98626348	-0.46	0.05
Thra	chr11	98626407	98629662	-3.23	0.78
Rara	chr11	98799009	98808938	-0.63	0.23
Rara	chr11	98808997	98822389	-1.01	-0.17
Psme3	chr11	101177564	101178336	-0.77	0.09
Psme3	chr11	101182100	101183258	-2.86	-0.26
Cbx2	chr11	118884462	118890969	-0.81	0.14
Rptor	chr11	119464338	119464399	-0.33	0.10
Rptor	chr11	119759737	119760755	-0.62	-0.06
Hus1	chr11	8896711	8896861	-2.14	0.69
Maml1	chr11	50069912	50070450	-1.28	0.23
Maml1	chr11	50071106	50074568	-0.75	0.08
Maml1	chr11	50076832	50105838	-0.45	0.03
Zfp354c	chr11	50628213	50628304	-0.22	0.78
Rad50	chr11	53463650	53465769	-0.89	-0.12
Zkscan17	chr11	59299022	59299412	-2.57	0.35
Zkscan17	chr11	59300338	59306167	-3.63	-0.20
Zkscan17	chr11	59319692	59319933	-0.44	-0.13
Top3a	chr11	60553560	60555499	-0.76	-0.16
Top3a	chr11	60555827	60557475	-2.70	-0.42
Ncor1	chr11	62147216	62147367	-0.76	0.12
Ncor1	chr11	62182894	62186997	-0.89	-0.06
Ncor1	chr11	62196726	62198338	-2.00	-0.18
Zfp287	chr11	62527173	62527774	-0.67	0.24
Zfp287	chr11	62528133	62528373	-0.69	0.38
Zfp287	chr11	62528582	62528763	-0.92	0.76
Zfp287	chr11	62541813	62542474	-2.41	0.87
Rpa1	chr11	75153888	75161885	-1.45	-0.24
Brip1	chr11	85874744	85878314	-0.63	-0.10
Med13	chr11	86084314	86084555	-1.19	0.21
Med13	chr11	86100031	86100272	-1.64	0.21
Med13	chr11	86104608	86114187	-0.85	-0.06
Med13	chr11	86132926	86141950	-0.38	0.01
Pcgf2	chr11	97551180	97551629	-0.96	-0.18
Pcgf2	chr11	97553081	97553393	-0.41	0.07
Pip4k2b	chr11	97578204	97584269	-2.59	-0.11
Med1	chr11	98014158	98015054	-0.30	0.00
Med1	chr11	98015143	98015684	-1.90	0.21

Med1	chr11	98016282	98017363	-1.46	0.10
Med1	chr11	98017662	98017873	-0.56	-0.08
Med1	chr11	98018052	98018173	-0.35	0.03
Med24	chr11	98590278	98590655	-0.71	-0.34
Nr1d1	chr11	98629308	98629667	-1.72	0.43
Nr1d1	chr11	98631693	98632540	-0.68	-0.11
Nr1d1	chr11	98633166	98636556	-0.43	0.04
Kat2a	chr11	100573480	100573601	-0.54	-0.16
Brca1	chr11	101384754	101385384	-1.00	0.21
Brca1	chr11	101385443	101386611	-0.56	0.06
Brca1	chr11	101386909	101387689	-2.16	0.30
Smarcd2	chr11	106133897	106134286	-1.61	-0.41
Smurf2	chr11	106683743	106684358	-1.06	-0.24
Smurf2	chr11	106781579	106781849	-2.81	-0.99
Cbx4	chr11	118941759	118947461	-1.94	0.14
E2f6	chr12	16817770	16817920	-1.01	-0.50
E2f6	chr12	16832231	16832859	-1.53	-0.19
Psma6	chr12	56499811	56499900	-1.08	-0.18
Ppm1a	chr12	73862227	73885470	-1.52	-0.08
Psmc1	chr12	101348377	101351316	-0.70	-0.11
Ccnk	chr12	109418007	109424620	-0.57	0.10
Zfp839	chr12	112088488	112099099	-0.78	-0.14
Zfp839	chr12	112106479	112107344	-1.45	-0.27
Ywhaq	chr12	21422717	21423076	-0.98	-0.15
Max	chr12	78038822	78039718	-1.87	-0.08
Max	chr12	78063025	78063205	-3.17	-0.43
Snw1	chr12	88791126	88791810	-2.89	-0.19
Ppp1r13b	chr12	113066968	113067568	-0.50	0.08
Ppp1r13b	chr12	113070791	113073499	-0.42	-0.03
Ppp1r13b	chr12	113146145	113146266	-0.59	1.21
Zkscan4	chr13	21576234	21576865	-0.23	-0.05
Hist1h2bg	chr13	23663268	23663474	-3.01	-0.19
Hist1h3d	chr13	23667608	23667786	-1.85	-0.32
Hist1h2bc	chr13	23776067	23776213	-0.42	-0.04
Prelid1	chr13	55423415	55423591	-2.87	-0.24
Rmi1	chr13	58503698	58503988	-1.45	-0.24
Rmi1	chr13	58508365	58511252	-0.33	-0.01
Zfp369	chr13	65397640	65398417	-0.30	0.01
Med10	chr13	69954496	69954853	-1.60	-0.13
Taf9	chr13	101435858	101436128	-3.16	0.35
Slc38a9	chr13	113528179	113528690	-3.31	1.16
Cdk13	chr13	17809653	17811597	-3.39	0.17
Cdk13	chr13	17858328	17896931	-2.82	-0.14

Zkscan3	chr13	21479290	21480454	-1.16	0.12
Zkscan3	chr13	21488062	21494295	-2.56	-0.43
Zkscan8	chr13	21611911	21613722	-1.42	-0.21
Zkscan8	chr13	21617156	21618818	-1.05	0.27
Hist1h3i	chr13	21875180	21875266	-1.25	-0.25
Hist1h3c	chr13	23837329	23837390	-0.45	-0.10
Dek	chr13	47201073	47201589	-1.38	0.23
Ctsl	chr13	64470393	64471614	-1.97	-0.09
Zfp729a	chr13	67722397	67722577	-0.51	0.13
Zfp738	chr13	67770082	67770412	-1.52	0.55
Zfp738	chr13	67771278	67771818	-1.27	0.47
Nr2f1	chr13	78328775	78329046	-0.77	-2.34
Jmy	chr13	94204723	94212614	-2.91	0.23
Jmy	chr13	94242583	94269644	-0.74	-0.07
Rad17	chr13	101387477	101389058	-1.90	-0.31
Psmc6	chr14	45949498	45950514	-0.58	0.08
Med4	chr14	73909855	73911003	-0.58	-0.16
Med4	chr14	73917729	73918293	-1.55	-0.20
Elf1	chr14	79881389	79936053	-0.32	-0.02
Elf1	chr14	79973114	79980817	-1.47	-0.10
Rarb	chr14	17264515	17264636	-0.46	-0.29
Rarb	chr14	17407447	17451267	-0.56	0.29
Nr1d2	chr14	19044403	19047125	-2.39	0.77
Nr1d2	chr14	19047304	19049180	-1.70	0.18
Supt16	chr14	52803669	52816914	-2.67	-0.36
Prmt5	chr14	55126018	55126553	-1.21	-0.15
Rabggta	chr14	56340490	56340807	-2.27	0.59
Cenpj	chr14	57145657	57148592	-0.36	0.08
Cenpj	chr14	57170788	57171625	-1.48	0.34
Cenpj	chr14	57171774	57172312	-0.36	0.05
Gtf2f2	chr14	76297012	76306651	-0.54	-0.05
Prkaa1	chr15	5093860	5110677	-1.10	-0.23
Prkaa1	chr15	5128795	5129184	-0.60	-0.09
Rictor	chr15	6745928	6747254	-2.42	0.20
Polr2k	chr15	36103771	36103949	-0.31	-0.05
Zfp707	chr15	75804010	75804189	-0.95	0.66
Zfp707	chr15	75804871	75806149	-1.08	-0.19
Rbx1	chr15	81305941	81306090	-0.83	-0.28
Ep300	chr15	81416643	81438767	-1.05	0.06
Ep300	chr15	81479273	81481819	-0.51	0.02
L3mbtl2	chr15	81516741	81518556	-1.73	-0.15
Arid2	chr15	96117982	96118043	-0.41	-0.14
Arid2	chr15	96199968	96201115	-1.06	0.08

Arid2	chr15	96201684	96209296	-1.71	0.10
Arid2	chr15	96223066	96232775	-2.27	0.36
Zfp740	chr15	102034138	102035505	-2.75	0.24
Zfp740	chr15	102039630	102043880	-0.82	0.06
Sp1	chr15	102238241	102256174	-1.81	0.06
Sp1	chr15	102261200	102261651	-3.30	-0.23
Skp2	chr15	9043329	9043540	-0.80	0.27
Skp2	chr15	9069207	9070167	-0.68	-0.14
Ywhaz	chr15	36723405	36723964	-0.95	0.41
Zfp706	chr15	36936977	36937157	-2.07	-0.55
Taf2	chr15	54847403	54848094	-0.81	-0.13
Taf2	chr15	54903436	54903617	-0.44	-0.08
Atad2	chr15	57928856	57930279	-1.16	0.12
Atad2	chr15	57966487	57966637	-1.54	-0.46
Cbx6	chr15	79658159	79664703	-1.02	0.09
Brd1	chr15	88543110	88543809	-2.54	0.27
Mapk11	chr15	88973270	88973361	-1.33	1.55
Mapk11	chr15	88973776	88973926	-0.54	0.57
Yaf2	chr15	93115848	93167336	-0.40	0.02
Vdr	chr15	97687093	97687333	-0.24	0.68
Zfp385a	chr15	103144534	103144595	-0.30	-0.02
Zfp385a	chr15	103144653	103144774	-0.37	-0.05
Zfp385a	chr15	103144892	103145221	-0.36	0.02
Zfp385a	chr15	103150806	103170487	-0.85	0.15
Zfp263	chr16	3744098	3745116	-0.66	-0.07
Zfp263	chr16	3748158	3750040	-0.43	0.02
Psmd2	chr16	20651724	20652324	-3.25	-0.17
Zfp597	chr16	3872223	3872374	-0.32	1.03
Crebbp	chr16	4154961	4213404	-1.70	-0.10
Arid1b	chr17	5332607	5339263	-2.33	-0.16
Zfp160	chr17	21178540	21179198	-2.36	1.43
Ppard	chr17	28369728	28432453	-1.72	-0.27
Mapk14	chr17	28828286	28828675	-2.21	-0.37
Brpf3	chr17	28938130	28938251	-0.19	0.10
Brpf3	chr17	28954078	28960704	-1.08	0.19
Brpf3	chr17	28973638	28973848	-0.68	0.35
Psmb8	chr17	34335288	34336214	-1.01	-0.16
Ehmt2	chr17	35042089	35042587	-2.96	-0.29
Ehmt2	chr17	35049712	35050992	-3.05	-0.21
Kat2b	chr17	53706385	53706446	-0.22	0.15
Zfp213	chr17	23694268	23694714	-1.32	-0.70
Zfp213	chr17	23694921	23696378	-0.39	0.24
Pdpk1	chr17	24215583	24216033	-2.93	0.32

Ube2i	chr17	25397995	25398776	-0.81	-0.11
Stub1	chr17	25967580	25967909	-2.17	-0.16
Stub1	chr17	25969947	25970306	-0.38	0.08
Taf11	chr17	28038342	28038672	-1.67	-0.25
Taf11	chr17	28042348	28045158	-0.73	0.11
Ring1	chr17	34158155	34160310	-1.41	-0.14
Ring1	chr17	34160369	34161265	-0.31	-0.04
Brd2	chr17	34259120	34259211	-1.42	0.77
Runx2	chr17	44744988	44745109	-0.62	0.24
Runx2	chr17	44872609	44873298	-2.06	-0.37
Gtf2f1	chr17	57143401	57143559	-0.53	-0.10
Gtf2f1	chr17	57143961	57144203	-0.51	-0.06
Tgif1	chr17	71199117	71202842	-1.83	-0.13
Rbbp8	chr18	11880308	11881165	-0.34	-0.01
Taf4b	chr18	14965941	14971957	-1.59	0.33
Polr2d	chr18	31955847	31956355	-3.30	0.43
Ercc3	chr18	32399984	32400372	-0.36	0.04
Prelid3a	chr18	67639251	67639430	-1.04	0.59
Smad7	chr18	75527018	75527734	-0.56	0.14
Smad7	chr18	75528210	75530550	-2.26	0.57
Smad2	chr18	76422187	76422338	-0.39	0.04
Smad2	chr18	76462139	76464727	-0.86	-0.12
Epc1	chr18	6436991	6440114	-0.66	0.09
Epc1	chr18	6448973	6450113	-2.33	0.40
Epc1	chr18	6490659	6515906	-1.33	-0.35
Kctd1	chr18	15220309	15220877	-0.44	0.38
Kctd1	chr18	15221623	15221804	-0.15	-0.06
Kctd1	chr18	15221952	15222043	-0.14	0.32
Nr3c1	chr18	39588392	39649457	-3.45	0.21
Smad4	chr18	73799858	73800008	-0.66	-0.12
Smad4	chr18	73863185	73863395	-3.16	-1.68
Ctdp1	chr18	80605356	80630912	-3.52	0.36
Ctdp1	chr18	80642798	80647906	-3.16	-0.17
Men1	chr19	6335008	6336781	-2.54	-0.27
Men1	chr19	6338873	6340864	-1.37	-0.10
Smarca2	chr19	26705264	26705505	-0.28	0.00
Pcgf5	chr19	36484266	36486670	-2.56	0.48
Pcgf5	chr19	36519495	36530396	-0.85	-0.17
Taf5	chr19	47142775	47149532	-1.43	0.33
Taf5	chr19	47156787	47157401	-0.49	0.09
Rad9a	chr19	4195437	4196286	-0.91	-0.11
Rbm14	chr19	4743957	4744526	-1.85	-0.59
Kat5	chr19	5603013	5603692	-1.67	-0.15

Kat5	chr19	5609321	5609621	-0.39	-0.05
Kat5	chr19	5609680	5610034	-0.36	-0.10
Prdx5	chr19	6983955	6984135	-1.81	-0.18
Esrra	chr19	6996237	6996298	-0.31	0.14
Polr2g	chr19	8872986	8873047	-0.27	-0.01
Ldb1	chr19	46107090	46108545	-0.69	0.04
Ldb1	chr19	46119462	46119522	-0.53	-0.37
Pcgf6	chr19	47108916	47109007	-0.64	-0.23
Med12	chrX	98488039	98488190	-2.90	0.51
Taf1	chrX	98794675	98794915	-0.42	0.05
Rragb	chrX	149574589	149575911	-0.52	0.09
G6pdx	chrX	71655299	71655479	-0.51	-0.03
Rad9b	chr5	122803966	122804026	-0.22	0.19

Metabolism of proteins

gene_name	chr	chromStart	chromEnd	diff.lg.p	diff.log2.fc
Rab23	chr1	33795210	33796982	-1.68	-0.29
Ttll4	chr1	74708327	74727946	-2.60	0.14
Ttll4	chr1	74744068	74744368	-2.07	-0.43
Mrpl44	chr1	79772771	79774975	-2.49	0.26
Mrpl44	chr1	79776125	79777632	-1.57	0.31
Sp100	chr1	87605814	87606023	-0.86	0.43
Psmd1	chr1	87961193	87961313	-0.67	0.21
Cops8	chr1	92508845	92509678	-0.59	-0.04
Ube2f	chr1	93182063	93182512	-2.14	-0.16
Asb1	chr1	93437351	93443741	-2.00	0.50
Asb1	chr1	93448832	93448983	-0.89	0.38
Asb1	chr1	93451373	93451764	-0.41	0.07
Mgat5	chr1	129203315	129203555	-1.22	0.30
Mgat5	chr1	129376047	129379549	-0.34	-0.01
Yod1	chr1	132613903	132614024	-0.26	-0.33
Yod1	chr1	132614351	132616601	-0.85	0.10
Rab7b	chr1	133585271	133594407	-0.60	0.14
Rab7b	chr1	133602280	133610067	-1.33	-0.11
Rab29	chr1	133763853	133763943	-0.50	-0.39
Rbbp5	chr1	134394590	134400982	-1.17	0.15
Rbbp5	chr1	134401131	134401312	-0.34	-0.03
Ube2t	chr1	136859153	136864304	-1.69	-0.60
Edem3	chr1	153658769	153666557	-0.61	0.05
F5	chr1	166120421	166122204	-1.15	2.81
F5	chr1	166122382	166122503	-0.93	3.42
F5	chr1	166123102	166123193	-0.44	1.85
F5	chr1	166124151	166124452	-2.11	3.30
Fcgr4	chr1	172959267	172959892	-3.04	0.51

Adamts4	chr1	173189096	173189157	-0.26	-0.91
Adamts4	chr1	173189215	173189276	-0.16	-0.42
B4galt3	chr1	173206252	173206818	-0.59	0.06
Dcaf8	chr1	174078235	174078505	-0.95	-0.24
Dcaf8	chr1	174124786	174125087	-1.13	-0.19
Pigm	chr1	174306661	174308397	-2.87	0.16
Parp1	chr1	182499105	182503843	-1.60	-0.27
Mrpl15	chr1	4766437	4766766	-0.92	-0.11
Cops5	chr1	10027799	10027979	-1.87	0.21
Uggt1	chr1	36200615	36203523	-0.76	-0.05
Uggt1	chr1	36300936	36301147	-3.25	-0.47
Mgat4a	chr1	37501120	37501720	-2.66	0.34
Chst10	chr1	38920717	38922995	-0.68	0.03
Satb2	chr1	56852675	56854020	-0.34	0.01
Satb2	chr1	56928415	56948033	-1.33	0.25
Satb2	chr1	57026707	57028178	-0.42	-0.07
Sumo1	chr1	59696455	59696989	-1.11	-0.09
Ino80d	chr1	63121070	63139998	-2.26	-0.15
Bard1	chr1	71121331	71122020	-0.53	0.08
Usp37	chr1	74485767	74485918	-0.64	0.18
Tuba4a	chr1	75211756	75211936	-0.82	-0.43
Cul3	chr1	80336885	80337005	-1.62	-0.38
Pign	chr1	107560192	107560253	-0.80	0.36
Ikbke	chr1	133151208	133153534	-0.51	-0.04
Ikbke	chr1	133173083	133173488	-3.28	-0.78
Rnf2	chr1	153318408	153318528	-0.89	0.28
Lamc1	chr1	155080624	155081688	-1.33	-0.18
Lamc1	chr1	155179317	155179856	-2.16	-0.21
Qsox1	chr1	157626418	157628411	-1.52	-0.23
Klhl20	chr1	163019047	163019258	-1.39	-0.43
Klhl20	chr1	163039327	163060580	-0.32	0.01
Dcaf6	chr1	167281032	167281331	-1.63	0.37
Atf6	chr1	172639771	172677240	-0.86	0.07
Usp21	chr1	173216496	173218092	-3.29	-0.45
Dtl	chr1	193363068	193363969	-0.39	0.07
Dtl	chr1	193364536	193365046	-0.52	-0.08
Kin	chr2	10013347	10014328	-0.81	0.10
Stam	chr2	14067864	14068462	-3.00	0.37
Commd3	chr2	18596461	18597638	-0.89	0.08
Bmi1	chr2	18605667	18606468	-0.89	0.08
Fbxw5	chr2	25358791	25359805	-1.19	0.14
Fbxw5	chr2	25360013	25360452	-1.24	-0.22
Fbxw5	chr2	25360840	25360960	-3.19	1.04

Adamts13	chr2	26829055	26829510	-0.47	0.39
Rxra	chr2	27597444	27604173	-0.47	-0.11
Rxra	chr2	27617342	27618510	-1.22	-0.15
Mrps2	chr2	28324898	28325708	-2.61	-0.16
Ttf1	chr2	28915782	28922656	-0.49	0.04
Ttf1	chr2	28941735	28942005	-1.00	-0.33
Dolpp1	chr2	30252653	30255005	-0.54	-0.11
Usp20	chr2	30863351	30865900	-0.51	-0.13
Nup214	chr2	31888785	31893653	-1.32	0.09
Pomt1	chr2	32109927	32110226	-1.75	-0.42
Golga2	chr2	32158521	32159772	-1.15	-0.16
Golga2	chr2	32161974	32162244	-1.46	0.29
Dpm2	chr2	32427908	32429091	-1.97	-0.18
St6galnac4	chr2	32442607	32443050	-0.76	0.31
St6galnac4	chr2	32449589	32449950	-1.02	-0.18
St6galnac4	chr2	32451265	32451326	-0.40	0.15
St6galnac4	chr2	32452522	32453780	-3.42	-0.26
Mrrf	chr2	36044722	36045173	-0.70	0.13
Mbd5	chr2	49101163	49101373	-2.40	0.76
Mbd5	chr2	49111717	49113872	-3.69	0.28
Mbd5	chr2	49134326	49172128	-0.55	0.13
Klhl41	chr2	69508654	69508893	-1.32	0.66
Dync1i2	chr2	71049792	71049883	-0.39	0.11
Psmc3	chr2	90894439	90895182	-1.38	-0.10
Traf6	chr2	101531587	101537929	-3.55	-0.22
Rtf1	chr2	119558206	119559195	-0.92	-0.10
Rtf1	chr2	119560243	119560484	-1.04	0.33
Usp8	chr2	126563777	126568417	-0.85	0.06
Prnd	chr2	131735693	131776618	-0.69	0.31
Tbc1d20	chr2	152119607	152127928	-0.69	-0.11
Tbc1d20	chr2	152137106	152137759	-1.14	-0.14
Asxl1	chr2	153222520	153228367	-3.49	-0.09
Actr5	chr2	158450648	158451096	-3.11	-0.77
Actr5	chr2	158462488	158464947	-1.71	-0.32
Top1	chr2	160471632	160471962	-0.61	-0.07
Rae1	chr2	172839488	172841203	-2.88	-0.32
Rab22a	chr2	173521497	173526997	-1.35	-0.15
Adrm1	chr2	179906292	179906906	-2.93	-0.47
Gata3	chr2	9779567	9784722	-1.35	0.40
Gata3	chr2	9784840	9796310	-0.60	0.24
Mrpl41	chr2	24829604	24830588	-0.48	0.04
Tubb4b	chr2	25077884	25079546	-0.33	0.00
Tubb4b	chr2	25079713	25080192	-0.31	-0.02

Traf2	chr2	25374427	25375145	-1.34	-0.17
Sec16a	chr2	26265370	26265521	-1.26	-0.31
Sec16a	chr2	26277747	26279943	-0.76	0.12
Sec16a	chr2	26294102	26294703	-3.27	0.24
Sec16a	chr2	26295062	26300736	-2.69	0.11
Ntng2	chr2	29063096	29103560	-0.32	-0.05
Dolk	chr2	30139748	30140407	-0.33	-0.02
Dolk	chr2	30140646	30141844	-1.09	-0.17
Fbxw2	chr2	34661048	34661869	-2.12	-0.20
Fbxw2	chr2	34662764	34664040	-0.46	-0.08
Fbxw2	chr2	34668436	34678672	-1.12	-0.10
Fbxw2	chr2	34681468	34681725	-0.58	0.19
Psmd5	chr2	34707993	34709794	-1.37	-0.17
Psmd5	chr2	34722532	34726482	-1.60	-0.40
Pdcl	chr2	37214762	37214852	-2.83	2.09
Psmb7	chr2	38498946	38499426	-1.35	-0.19
Stam2	chr2	52579981	52595233	-0.55	0.15
Stam2	chr2	52601122	52601183	-0.31	-0.06
Sp3	chr2	72776048	72779137	-2.88	0.20
Slc35c1	chr2	92293966	92294832	-2.30	-0.24
Slc35c1	chr2	92298872	92299619	-0.47	0.04
Slc35c1	chr2	92299947	92300675	-0.27	-0.42
Rcn1	chr2	105227977	105229285	-0.79	-0.11
Rcn1	chr2	105239148	105239446	-0.74	0.18
Ino80	chr2	119200037	119200278	-1.19	0.27
Ino80	chr2	119303244	119303365	-0.39	-0.13
Trp53bp1	chr2	121030697	121033825	-0.66	0.11
Trp53bp1	chr2	121034169	121046975	-1.32	-0.15
Trp53bp1	chr2	121061313	121069657	-0.32	0.01
Pcna	chr2	132078555	132078916	-3.65	-0.19
Psmf1	chr2	151543111	151543232	-0.83	-0.47
Psmf1	chr2	151543649	151544068	-0.81	-0.19
Eif2s2	chr2	154698460	154698521	-1.85	0.55
Edem2	chr2	155527795	155531394	-0.45	0.03
Edem2	chr2	155555031	155555181	-0.49	0.12
Neurl2	chr2	164656229	164658296	-2.89	-0.44
Neurl2	chr2	164658917	164659037	-3.40	1.18
B4galt5	chr2	167126418	167130545	-2.25	-0.10
Cp	chr3	19892644	19892765	-0.56	0.22
Sec62	chr3	30692214	30699082	-1.16	0.36
Sec62	chr3	30711269	30718098	-1.92	0.12
Gfm1	chr3	67278519	67278960	-1.74	0.24
Fbxw7	chr3	84619498	84694070	-1.92	0.38

Fbxw7	chr3	84694129	84756226	-2.05	0.20
Fbxw7	chr3	84780140	84781955	-0.37	0.02
Rab13	chr3	90028667	90029826	-0.92	0.28
Mrpl9	chr3	94251762	94252630	-2.45	-0.16
Otud7b	chr3	95956546	95960193	-2.15	-0.13
Hist2h2be	chr3	96025043	96025490	-1.83	0.52
Hist2h2be	chr3	96026738	96027215	-1.30	-0.39
Sec22b	chr3	97725140	97725438	-2.99	-0.26
Dph5	chr3	115631611	115631971	-1.70	-0.32
Ube2d3	chr3	135101656	135102228	-3.24	-0.27
Bcl10	chr3	145587232	145587592	-0.60	0.07
Bcl10	chr3	145588099	145593615	-2.28	0.24
Usp33	chr3	152029782	152033363	-0.30	0.00
Pigk	chr3	152448391	152449139	-0.87	0.13
Pigk	chr3	152449825	152450513	-2.48	0.40
Pigk	chr3	152451588	152451857	-2.02	0.56
Pex2	chr3	5561142	5563166	-2.95	-0.27
Phc3	chr3	30805887	30813151	-0.57	-0.08
Phc3	chr3	30864703	30868337	-0.42	0.12
Cog6	chr3	52798099	52798220	-0.15	-0.29
Commd2	chr3	57450303	57450573	-2.39	-0.46
Siah2	chr3	58495620	58495891	-1.37	0.55
Dap3	chr3	88725620	88725979	-0.29	-0.02
Dap3	chr3	88726336	88726815	-0.38	-0.08
Dap3	chr3	88727202	88727531	-0.56	-0.18
Dap3	chr3	88753815	88753905	-0.32	-0.04
Psmd4	chr3	94836626	94836924	-1.79	-0.12
Psmd4	chr3	94839816	94846467	-2.16	-0.13
Hist2h2aa1	chr3	96044071	96044216	-1.04	1.17
Man1a2	chr3	100371071	100382434	-1.39	-0.12
Man1a2	chr3	100460030	100489396	-1.61	-0.09
Csf1	chr3	107544354	107544444	-0.83	0.84
Csf1	chr3	107544832	107548597	-0.29	-0.01
Csf1	chr3	107548745	107550706	-1.24	-1.52
Csf1	chr3	107551063	107553281	-0.37	0.05
Gnai3	chr3	107911702	107912208	-1.11	-0.08
Rwdd3	chr3	120859334	120862148	-1.36	0.36
Ank2	chr3	126625873	126626864	-0.77	0.39
Sec24b	chr3	129686102	129687101	-0.83	-0.07
Cyr61	chr3	145310530	145310591	-0.46	2.58
Fpgt	chr3	154749553	154753118	-0.61	0.07
Fbxl4	chr4	22303713	22313319	-1.70	0.24
Fbxl4	chr4	22354425	22354486	-0.94	0.57

Map3k7	chr4	32051081	32051232	-2.61	-1.03
Map3k7	chr4	32109961	32110261	-0.43	0.08
Ube2r2	chr4	41137777	41138136	-1.17	0.13
Creb3	chr4	43575505	43575715	-0.86	-0.18
Creb3	chr4	43579692	43579932	-1.01	0.27
Dcaf10	chr4	45355032	45355512	-0.76	-0.17
Dcaf10	chr4	45385860	45387298	-0.73	-0.11
Nans	chr4	46515563	46516280	-0.44	-0.04
Tgfbr1	chr4	47419877	47423717	-1.19	0.29
Stx17	chr4	48197791	48198211	-0.64	0.17
Rad23b	chr4	55398385	55402936	-0.95	0.07
Rraga	chr4	86221606	86222293	-0.53	0.03
Usp24	chr4	105988817	105989117	-1.06	-0.35
Usp24	chr4	106111387	106111867	-0.45	-0.03
Usp24	chr4	106112884	106113842	-2.89	0.24
Ndc1	chr4	107083754	107086943	-0.44	0.04
Lrrc41	chr4	115761466	115762183	-1.09	-0.08
Lrrc41	chr4	115768961	115769260	-0.52	0.05
Scmh1	chr4	120201875	120202085	-2.07	0.58
Trappc3	chr4	125939648	125949968	-0.77	-0.13
Trappc3	chr4	125951250	125952918	-1.12	-0.09
Clspn	chr4	126237164	126239019	-0.31	-0.01
Clspn	chr4	126243223	126249609	-0.34	-0.02
Clspn	chr4	126270038	126270788	-1.23	-0.26
Phc2	chr4	128404910	128405061	-1.05	-0.71
Ptp4a2	chr4	129497464	129516451	-0.43	-0.02
Fuca1	chr4	135495027	135495355	-0.80	0.11
Usp48	chr4	137211154	137213554	-1.84	0.15
Mul1	chr4	137996388	137996837	-0.29	0.01
Capzb	chr4	138748845	138794946	-0.34	-0.01
Fbxo2	chr4	147538344	147538434	-0.25	-0.78
Klhl21	chr4	151383089	151389959	-0.56	0.05
Klhl21	chr4	151390287	151390408	-0.53	0.22
Icmt	chr4	151674797	151677845	-1.75	-0.20
Gnb1	chr4	154933138	154933258	-0.58	-0.09
Mxra8	chr4	155217437	155217527	-0.27	-0.15
Ripk2	chr4	16050729	16051621	-2.11	-0.28
Manea	chr4	26254667	26273799	-0.95	0.09
Ddx58	chr4	40152603	40155517	-1.64	0.25
B4galt1	chr4	40753786	40754830	-0.76	-0.05
B4galt1	chr4	40770567	40801001	-1.03	0.10
Vcp	chr4	43013259	43013349	-0.48	-0.16
Gne	chr4	44047845	44048266	-1.53	-0.50

Gne	chr4	44049104	44049255	-0.80	0.70
Gne	chr4	44068129	44079675	-0.31	0.01
Fbxo10	chr4	45048105	45048374	-0.47	0.54
Fbxo10	chr4	45048433	45048494	-0.73	1.50
Fbxo10	chr4	45048553	45048793	-0.74	0.68
Fbxo10	chr4	45071323	45071922	-0.48	0.30
Alg2	chr4	47482973	47483543	-1.01	0.16
Mrpl50	chr4	49526386	49527305	-1.35	0.13
Mysm1	chr4	94631563	94631714	-1.83	1.14
Mysm1	chr4	94631833	94632492	-3.38	0.30
Dph2	chr4	117562115	117562206	-0.41	0.14
Dph2	chr4	117562463	117563268	-1.81	-0.28
Dph2	chr4	117564417	117564628	-0.78	-0.27
St3gal3	chr4	117604757	117612708	-0.45	-0.04
Cdc20	chr4	118109887	118109948	-0.55	-0.37
Rab42	chr4	131858314	131858373	-0.31	-1.81
Wdtc1	chr4	132849782	132850290	-2.36	-0.35
Pigv	chr4	133218200	133220602	-2.57	-0.58
Pigv	chr4	133228472	133228562	-0.59	0.47
Dhdds	chr4	133526767	133536084	-0.36	-0.02
Man1c1	chr4	134119221	134119701	-0.62	-0.06
Otud3	chr4	138451293	138453499	-0.59	0.08
Fbxo6	chr4	147520063	147520213	-1.03	0.25
Pex14	chr4	148335210	148335898	-1.74	-0.17
Pex14	chr4	148358178	148473921	-0.61	0.08
Spsb1	chr4	149271827	149281337	-1.02	0.57
Nupl2	chr5	23687756	23687817	-0.27	0.02
Nupl2	chr5	23688026	23689736	-0.62	0.07
Nub1	chr5	24191722	24195229	-0.45	-0.13
Nub1	chr5	24216014	24216194	-0.28	0.00
Galnt11	chr5	24771083	24771350	-1.41	0.32
Bst1	chr5	44210131	44210281	-0.56	-0.24
Klhl5	chr5	65558211	65558721	-1.61	0.23
Dcun1d4	chr5	73950743	73951343	-1.40	0.48
Exoc1	chr5	76998801	76999310	-0.36	0.03
Uso1	chr5	92630175	92631402	-0.50	0.03
Pigg	chr5	108776899	108777289	-0.58	-0.18
Usp30	chr5	114572302	114572933	-2.44	-0.39
Rab35	chr5	116082025	116082085	-0.40	0.30
Wsb2	chr5	117827434	117828121	-0.36	0.02
Fbxo21	chr5	118426838	118426928	-0.57	0.34
Fbxo21	chr5	118452063	118458648	-0.32	0.01
Psmd9	chr5	123678228	123684616	-1.07	0.18

B3gnt4	chr5	123961238	123961328	-0.20	-0.03
Mrps17	chr5	130223908	130224417	-2.55	0.24
Sumf2	chr5	130338811	130339081	-0.89	-0.45
Kctd7	chr5	130628247	130628815	-1.33	-0.62
Vgf	chr5	137506960	137507972	-0.59	-0.23
Vgf	chr5	137508209	137508864	-0.79	-0.47
Ache	chr5	137731479	137733258	-3.51	0.99
Ache	chr5	137735362	137735572	-0.35	-0.11
Cops6	chr5	138605218	138605278	-0.72	-0.47
Fam20c	chr5	139231034	139232111	-1.21	-0.07
Fam20c	chr5	139284048	139286017	-3.54	-0.23
Foxk1	chr5	142931509	142934800	-1.90	-0.10
Kdelr2	chr5	144165498	144165648	-0.43	-0.06
Trrap	chr5	145550403	145551932	-0.83	-0.16
Preb	chr5	31254099	31254400	-0.89	0.35
Preb	chr5	31255028	31255718	-1.40	0.29
Preb	chr5	31256227	31256528	-0.48	-0.11
Preb	chr5	31257006	31257247	-0.73	0.25
Preb	chr5	31257366	31258308	-0.47	-0.02
Preb	chr5	31262373	31262704	-2.91	-0.42
Tnip2	chr5	34839221	34839491	-0.36	0.04
Tada2b	chr5	36818228	36826755	-0.36	-0.01
Wfs1	chr5	37358091	37358361	-0.55	0.38
Wfs1	chr5	37358629	37358750	-0.90	-1.02
Wfs1	chr5	37358929	37359708	-0.31	0.02
Fbxl5	chr5	44142150	44149098	-2.30	-0.48
Fbxl5	chr5	44149396	44151973	-0.42	-0.02
Commd8	chr5	72550637	72551057	-0.88	0.12
Nup54	chr5	92845130	92846543	-1.56	0.19
Sec31a	chr5	100790877	100791058	-0.38	0.02
Abraxas1	chr5	101234952	101235460	-0.91	-0.18
Mlec	chr5	115597834	115600228	-0.64	-0.05
Suds3	chr5	117542584	117542913	-1.68	-0.19
Suds3	chr5	117558367	117565972	-1.81	-0.18
Fbxw8	chr5	118515497	118515797	-0.35	-0.06
Eif2b1	chr5	125020669	125021791	-0.56	0.05
Pom121	chr5	135853620	135857198	-0.30	0.00
Pom121	chr5	135857496	135857795	-0.92	0.12
Pom121	chr5	135869997	135870416	-0.68	-0.16
Fbxl18	chr5	143633766	143634186	-0.39	0.07
Fbxl18	chr5	143640382	143644224	-0.93	-0.38
Fbxl18	chr5	143650503	143656917	-0.38	-0.27
Usp42	chr5	144471642	144477010	-1.74	-0.15

Usp42	chr5	144477457	144478068	-0.31	0.01
Usp12	chr5	147548927	147550725	-1.46	-0.24
Calu	chr6	29298148	29306560	-1.10	0.22
Calu	chr6	29322584	29324851	-1.44	0.09
Exoc4	chr6	33199149	33215941	-0.31	-0.02
Exoc4	chr6	33871536	33922362	-2.77	0.27
Nup205	chr6	35127615	35133895	-0.29	0.01
Rab19	chr6	39339614	39340319	-0.89	-0.29
Tnip3	chr6	65564764	65582093	-2.86	0.24
Gng12	chr6	66846480	66846903	-0.52	-1.18
Eif2ak3	chr6	70840206	70843273	-1.06	-0.17
Eif2ak3	chr6	70843391	70850228	-1.89	0.62
Eif2ak3	chr6	70854128	70854726	-1.25	-0.22
Rnf103	chr6	71444126	71444217	-0.35	-0.24
St3gal5	chr6	72092172	72097116	-0.95	0.10
Dctn1	chr6	83140226	83141067	-0.66	-0.06
Cct7	chr6	85402007	85402187	-2.76	-0.42
Ruvbl1	chr6	88415416	88415624	-0.60	0.20
Kbtbd8	chr6	95071961	95077327	-2.16	0.32
Mitf	chr6	97969644	97970094	-1.02	0.16
Edem1	chr6	108805488	108805668	-2.85	-0.15
Ttll3	chr6	113357294	113359347	-3.58	0.53
Ttll3	chr6	113362865	113364505	-1.18	-0.20
Vhl	chr6	113578147	113579978	-1.16	0.21
Fbxl14	chr6	119429685	119431451	-2.73	-0.15
Rad52	chr6	119865354	119869071	-1.84	0.25
Rad52	chr6	119870928	119871139	-1.97	0.40
Rad52	chr6	119871288	119872841	-0.64	0.11
Usp18	chr6	121195982	121196071	-0.23	-0.06
Mrpl51	chr6	125143277	125143546	-0.91	0.10
Cmas	chr6	142705235	142705504	-3.29	-0.63
Mrps35	chr6	147019097	147019424	-0.70	0.10
Klhl42	chr6	147039984	147056970	-0.73	-0.10
Etfbkmt	chr6	149090148	149092915	-0.36	-0.07
Ube2h	chr6	30163827	30164395	-0.64	0.08
Ube2h	chr6	30164514	30164664	-1.04	0.40
Ube2h	chr6	30164783	30164993	-0.55	0.22
Hipk2	chr6	38647868	38666025	-2.58	-0.11
Hipk2	chr6	38697991	38787047	-0.54	-0.02
Hipk2	chr6	38825859	38826040	-2.58	-0.39
Nod1	chr6	54874621	54874682	-0.58	2.04
Nod1	chr6	54893198	54893646	-1.73	2.07
Nod1	chr6	54894093	54894451	-0.13	0.08

Nod1	chr6	54894629	54894929	-0.30	0.33
Ptcd3	chr6	71830841	71831536	-3.09	-0.31
Rnf181	chr6	72310209	72310594	-0.35	-0.02
Tgoln1	chr6	72561733	72566785	-0.76	-0.03
Mrpl19	chr6	81911638	81912330	-0.34	0.01
Ino80b	chr6	83074670	83075023	-0.62	0.15
Rab43	chr6	87739684	87739804	-0.33	-0.04
Rab43	chr6	87761443	87761773	-0.72	0.19
Nup210	chr6	90963060	90963720	-3.45	-0.38
Xpc	chr6	91439866	91440135	-3.04	-0.81
Xpc	chr6	91448100	91450008	-0.70	-0.11
Xpc	chr6	91460626	91465852	-1.01	0.46
Mrps25	chr6	92124773	92125723	-2.44	-0.25
Sumf1	chr6	108058060	108058479	-0.75	0.10
Sumf1	chr6	108135307	108135577	-1.12	-0.19
Rad18	chr6	112577031	112577691	-0.71	0.12
Tada3	chr6	113322347	113324488	-0.52	0.12
Phc1	chr6	122273438	122273618	-1.06	0.49
Phc1	chr6	122275560	122275830	-0.30	-0.03
Pex5	chr6	124347220	124347340	-0.93	-0.27
Usp5	chr6	124776424	124779465	-2.08	-0.36
Trappc6a	chr7	20094077	20099806	-2.85	-0.29
Plaur	chr7	25257191	25257252	-0.42	-0.17
B3gnt8	chr7	26415160	26415551	-1.21	-0.16
Paf1	chr7	29178014	29178074	-0.35	0.09
Paf1	chr7	29181953	29184402	-2.55	-0.12
Etfb	chr7	50699512	50707736	-0.77	-0.18
Josd2	chr7	51723425	51723690	-0.32	-0.03
Napsa	chr7	51827904	51828025	-0.93	-0.27
Hrc	chr7	52590698	52591209	-0.35	-0.26
Hrc	chr7	52591478	52591689	-0.77	1.06
Prmt3	chr7	57112887	57113008	-3.64	-0.59
Herc2	chr7	63485102	63486928	-1.77	0.13
Klhl25	chr7	82993342	83012197	-1.00	0.11
Klhl25	chr7	83012375	83018299	-0.87	-0.23
Klhl25	chr7	83018716	83018926	-0.35	-0.11
Mrps11	chr7	85936782	85937754	-0.94	-0.16
Ctsc	chr7	95437215	95437515	-0.81	0.31
Ctsc	chr7	95458037	95458487	-2.34	0.25
Rab30	chr7	99984250	99984520	-0.26	-0.26
Alg8	chr7	104540160	104540578	-1.38	-0.28
Arrb1	chr7	106747059	106749813	-0.34	0.01
Arrb1	chr7	106752442	106752711	-1.98	-0.22

Ctr9	chr7	118197379	118199651	-2.59	0.24
Usp47	chr7	119234949	119245370	-2.45	-0.14
Usp47	chr7	119252192	119254002	-0.85	-0.09
Spon1	chr7	121183472	121183922	-0.88	0.69
Ccp110	chr7	125864970	125865479	-0.53	0.09
Ccp110	chr7	125865867	125868001	-0.53	0.07
Dctn5	chr7	129276554	129277689	-0.53	0.10
Dctn5	chr7	129291791	129292440	-1.28	0.09
Rnf40	chr7	134739594	134740336	-0.78	-0.07
Fbxl19	chr7	134890288	134891744	-2.81	-0.80
Fbxl19	chr7	134911014	134911877	-0.34	-0.02
Sec23ip	chr7	135888443	135894042	-3.19	-0.20
Abraxas2	chr7	140072616	140072677	-0.71	0.38
Abraxas2	chr7	140072975	140075508	-0.35	-0.01
Zranb1	chr7	140122884	140158368	-0.34	-0.01
Zranb1	chr7	140174515	140175634	-0.43	-0.06
Zranb1	chr7	140175516	140176023	-0.87	0.14
Pnpla2	chr7	148641086	148641236	-1.29	-0.28
Pnpla2	chr7	148645626	148646045	-2.50	0.26
Tfpt	chr7	3581338	3581486	-0.93	0.33
Sae1	chr7	16912835	16913105	-2.59	-0.21
Calm3	chr7	17502039	17502309	-1.49	0.09
Apoe	chr7	20282098	20282920	-1.74	0.36
Apoe	chr7	20284425	20284485	-1.41	1.26
Ceacam1	chr7	26247739	26248608	-3.25	0.28
Ceacam1	chr7	26256924	26258919	-0.89	0.24
Ceacam2	chr7	26302704	26302765	-2.15	3.98
Sptbn4	chr7	28149267	28149418	-0.46	1.81
Sptbn4	chr7	28189431	28189552	-0.18	0.68
Mrps12	chr7	29524778	29525105	-1.79	-0.30
Psmd8	chr7	29965393	29965573	-1.95	-0.13
Nr1h2	chr7	51806700	51806791	-0.86	0.19
Nucb1	chr7	52758551	52762906	-3.25	-0.31
Nucb1	chr7	52765032	52765778	-1.96	-0.30
Spaca4	chr7	52980506	52980713	-0.34	-0.66
Spaca4	chr7	52981037	52981186	-0.62	0.98
Nsmce3	chr7	72016624	72017837	-3.57	-0.23
Asb7	chr7	73792504	73823980	-0.99	-0.14
Asb7	chr7	73834117	73834387	-0.57	0.21
Mrpl46	chr7	85920226	85920609	-2.43	-0.31
Mfge8	chr7	86278653	86279192	-0.71	-0.06
Mfge8	chr7	86293825	86293916	-3.60	0.84
Man2a2	chr7	87496117	87497911	-1.70	-0.08

Man2a2	chr7	87515719	87516012	-0.86	-0.26
Blm	chr7	87647232	87647711	-0.92	-0.15
Blm	chr7	87654407	87659523	-2.35	0.28
B3gnt6	chr7	105342961	105343081	-0.22	-1.00
Neu3	chr7	106961618	106962782	-2.63	0.57
Nup98	chr7	109268003	109269291	-1.15	-0.06
Usp17ld	chr7	110398629	110398868	-0.37	0.82
Cog7	chr7	129066679	129066858	-1.01	-0.30
Cog7	chr7	129121227	129125207	-0.66	-0.12
Ino80e	chr7	134005440	134005501	-0.47	0.44
Nsmce4a	chr7	137690685	137690865	-1.03	-0.54
Bet1l	chr7	148039282	148042282	-3.67	-0.23
Trappc5	chr8	3676506	3679785	-3.15	-0.23
Trappc5	chr8	3680083	3680622	-2.24	-0.42
F7	chr8	13034759	13035479	-1.53	-0.88
Proz	chr8	13073522	13073612	-0.41	0.46
Cul4a	chr8	13146533	13146713	-1.15	-0.20
Cul4a	chr8	13146862	13147072	-0.43	-0.06
Thsd1	chr8	23368810	23369377	-1.19	-1.93
Thsd1	chr8	23369584	23370091	-2.31	3.62
Ank1	chr8	24242612	24243062	-0.39	0.32
Cope	chr8	72826683	72826773	-0.78	-0.16
Fkbp8	chr8	73051641	73051859	-0.44	-0.08
Babam1	chr8	73920753	73921677	-1.65	-0.19
Mrpl34	chr8	73988824	73989652	-0.31	0.00
Dda1	chr8	73998687	73999406	-1.12	-0.08
Dda1	chr8	73999764	73999855	-0.87	0.21
Rab8a	chr8	74704051	74704703	-0.83	-0.06
Nr3c2	chr8	79432284	79432583	-1.74	2.06
Nr3c2	chr8	79433507	79433836	-1.15	2.46
Cyld	chr8	91220956	91226218	-0.51	0.31
Mmp2	chr8	95376513	95376812	-0.34	-0.10
Mmp2	chr8	95377020	95377319	-0.63	0.31
Fbxl8	chr8	107791922	107793078	-0.54	-0.13
Cog4	chr8	113370709	113373788	-1.39	-0.37
Cog4	chr8	113405393	113406134	-0.62	-0.09
St3gal2	chr8	113444003	113491411	-1.01	-0.11
St3gal2	chr8	113493490	113494575	-0.52	0.08
St3gal2	chr8	113494634	113495202	-0.68	0.13
Gan	chr8	119682094	119707502	-2.38	0.66
Usp10	chr8	122460483	122471049	-0.74	0.04
Usp10	chr8	122480587	122481247	-0.85	0.09
Trappc2l	chr8	125135525	125137014	-0.39	-0.08

Tubb3	chr8	125944514	125944901	-0.18	-0.07
Rab4a	chr8	126358896	126359073	-1.59	1.17
Galnt2	chr8	126864828	126868362	-0.38	0.02
Dcun1d2	chr8	13256291	13257907	-0.46	-0.03
Gas6	chr8	13465552	13465851	-0.30	0.01
Vdac3	chr8	23687812	23688050	-3.03	0.38
Pomk	chr8	27091432	27091522	-0.36	-0.49
Pomk	chr8	27092801	27100129	-0.87	-0.09
Dctn6	chr8	35169406	35171565	-0.59	0.15
Ing2	chr8	48753850	48754631	-3.25	-0.30
Galnt7	chr8	60062627	60131852	-1.46	-0.07
Klhl2	chr8	67219421	67219870	-3.53	-0.50
Ano8	chr8	74000216	74000456	-0.50	-0.36
Ano8	chr8	74000514	74000695	-0.32	-0.16
Ano8	chr8	74003128	74005028	-1.87	-0.28
B3gnt3	chr8	74215973	74217291	-0.31	-0.01
B3gnt3	chr8	74217408	74227314	-0.61	-0.20
Rad23a	chr8	87358550	87358761	-1.01	-0.39
Calr	chr8	87366315	87366923	-2.76	-0.06
Calr	chr8	87369960	87370800	-0.94	0.04
Amfr	chr8	96496474	96497162	-1.29	-0.10
Dync1li2	chr8	106944120	106944451	-1.40	-0.19
B3gnt9	chr8	107777610	107779021	-2.43	0.56
Psmb10	chr8	108461631	108461997	-1.25	-0.38
Cog8	chr8	109570548	109573005	-1.46	-0.14
Cog8	chr8	109576126	109580248	-1.04	0.10
Mbtps1	chr8	122032081	122032770	-0.67	0.05
Mbtps1	chr8	122070114	122082661	-3.15	0.21
Fbxo31	chr8	124075878	124076237	-3.34	0.63
Exoc8	chr8	127418756	127418936	-0.37	-0.09
Exoc8	chr8	127419235	127421605	-0.57	-0.05
Mrpl4	chr9	20812982	20813281	-0.84	-0.08
Pigyl	chr9	21961289	21962798	-1.13	0.18
Opcml	chr9	28210755	28211775	-0.41	-0.29
Opcml	chr9	28729304	28729904	-0.49	0.42
Opcml	chr9	28730113	28730774	-1.60	0.95
Nfrkb	chr9	31218903	31226648	-1.88	0.21
Usp2	chr9	43883576	43884236	-2.87	2.50
Usp2	chr9	43902032	43902332	-0.41	-0.10
Dpagt1	chr9	44141205	44141504	-0.33	-0.02
Apoa1	chr9	46037892	46038519	-0.60	2.20
Usp28	chr9	48849572	48849783	-2.16	0.69
Alg9	chr9	50651063	50651633	-0.38	-0.03

Psma4	chr9	54805658	54805748	-1.39	0.21
Fbxo22	chr9	55066270	55071672	-2.03	0.17
Fbxo22	chr9	55071880	55072031	-0.93	-0.28
Sin3a	chr9	56973115	56975576	-0.47	0.04
Rora	chr9	69226655	69226835	-0.29	0.06
Adam10	chr9	70526807	70526988	-1.21	0.24
Adam10	chr9	70626056	70626297	-2.69	0.34
Rab27a	chr9	72892701	72908786	-0.51	-0.51
Rab27a	chr9	72943089	72943840	-1.02	-0.28
Cish	chr9	107199256	107202294	-2.04	3.67
Usp4	chr9	108293381	108294561	-0.32	0.00
Usp19	chr9	108393096	108395299	-0.76	-0.07
Cdc25a	chr9	109778082	109778888	-0.45	0.07
Cdc25a	chr9	109794596	109795525	-1.51	0.22
Glb1	chr9	114310242	114326114	-2.08	0.44
Glb1	chr9	114368770	114373569	-0.38	-0.02
Glb1	chr9	114374377	114383495	-3.66	0.26
Dync1li1	chr9	114632656	114632895	-3.05	0.38
Dync1li1	chr9	114632812	114633160	-1.11	0.11
Wdr48	chr9	119833344	119835070	-2.12	0.15
Birc2	chr9	7818584	7818943	-0.91	0.25
Birc2	chr9	7834955	7835255	-3.06	0.75
Birc3	chr9	7848939	7850928	-0.49	0.05
Birc3	chr9	7860311	7872961	-0.93	0.08
Fbxl12	chr9	20448850	20449181	-0.31	-0.05
Dnmt1	chr9	20734577	20736587	-0.40	-0.05
Keap1	chr9	21034503	21038410	-0.91	0.07
Keap1	chr9	21041585	21042276	-1.22	-0.20
Keap1	chr9	21043025	21043776	-0.79	-0.29
Rab3d	chr9	21714741	21715072	-1.15	-0.35
Aplp2	chr9	30957469	30957679	-0.71	-0.07
Aplp2	chr9	30985646	31019400	-0.55	-0.11
Arcn1	chr9	44551783	44551994	-2.61	0.22
Arcn1	chr9	44568237	44575891	-0.66	0.09
Cul5	chr9	53425530	53426100	-1.13	0.12
Rab39	chr9	53494560	53494769	-0.63	1.81
Pml	chr9	58067532	58068881	-1.38	-0.21
Pml	chr9	58077807	58077868	-0.49	0.22
Pml	chr9	58080966	58094982	-3.67	-0.47
Pml	chr9	58097382	58097563	-0.75	-0.30
Senp8	chr9	59584121	59585492	-0.49	0.08
Pias1	chr9	62729583	62729943	-0.64	0.13
Smad3	chr9	63605680	63605801	-0.63	0.71

Smad3	chr9	63605729	63605891	-0.25	0.23
Rab11a	chr9	64585502	64585563	-1.24	0.53
Usp3	chr9	66366103	66367920	-0.95	0.19
Usp3	chr9	66440935	66441025	-0.40	0.79
Usp3	chr9	66441083	66441320	-0.53	0.45
Rab8b	chr9	66715068	66767482	-1.09	0.21
Pigb	chr9	72863505	72865261	-0.80	0.10
Fbxo9	chr9	77943491	77943611	-0.30	0.14
Pgm3	chr9	86447667	86448117	-1.42	-0.31
Mrps22	chr9	98501918	98502098	-2.94	-0.55
Apeh	chr9	107987744	107987924	-0.34	-0.05
Dag1	chr9	108166098	108166165	-0.32	0.40
Gorasp1	chr9	119834790	119835059	-2.78	0.60
Sec22c	chr9	121593476	121593866	-0.39	-0.09
Mtrf1l	chr10	4532471	4533493	-0.46	-0.10
Shprh	chr10	10869257	10871973	-1.10	-0.20
Shprh	chr10	10882742	10884564	-0.46	-0.06
Fuca2	chr10	13223007	13225667	-0.83	-0.15
Fuca2	chr10	13234515	13235470	-0.57	-0.03
Sec63	chr10	42548759	42548969	-0.48	0.04
Nus1	chr10	52137442	52137742	-3.64	-0.33
Nus1	chr10	52153241	52156613	-0.44	0.03
Adora2a	chr10	74795923	74796222	-2.47	5.11
Adora2a	chr10	74796429	74796728	-0.17	-0.23
Adora2a	chr10	74797234	74797295	-0.42	2.81
Pofut2	chr10	76731353	76732331	-0.45	0.02
Sumo3	chr10	77068978	77069512	-0.81	0.18
Ube2g2	chr10	77107280	77107637	-0.93	-0.11
Ube2g2	chr10	77107992	77108141	-1.22	0.25
Eef2	chr10	80644273	80645254	-0.46	0.00
Dohh	chr10	80853321	80854179	-2.96	-0.64
Eid3	chr10	82329548	82330052	-0.30	0.00
Eid3	chr10	82330555	82330674	-1.43	0.91
Fbxo7	chr10	85487309	85487369	-0.37	0.10
Galnt4	chr10	98570828	98571068	-2.11	0.75
Galnt4	chr10	98571366	98572174	-0.95	0.19
Galnt4	chr10	98572412	98573250	-1.58	0.31
Ccdc59	chr10	105278534	105284446	-2.55	0.17
Cpm	chr10	117120810	117120871	-0.51	1.00
Pcmt1	chr10	7350508	7350747	-0.29	0.00
Pcmt1	chr10	7383084	7383144	-0.27	0.09
Tnfaip3	chr10	18721581	18725137	-2.68	-0.19
Rnf146	chr10	29064221	29064491	-0.63	0.08

Man1a	chr10	53750519	53795572	-0.83	-0.31
Ube2d1	chr10	70718055	70718592	-2.86	0.34
Trappc10	chr10	77650129	77651540	-0.42	-0.03
Pias4	chr10	80616715	80617300	-0.65	0.09
Pias4	chr10	80619974	80625923	-1.83	-0.26
Pias4	chr10	80626491	80630465	-0.33	-0.01
Mrpl54	chr10	80727466	80729671	-0.85	-0.07
Ckap4	chr10	83990001	83991399	-2.44	-0.11
Ckap4	chr10	83996216	83996633	-1.45	-0.31
Rab21	chr10	114727724	114727964	-0.78	-0.07
Mdm2	chr10	117126799	117129688	-0.41	0.01
Cand1	chr10	118647900	118648919	-0.55	0.03
Usp15	chr10	122550299	122558549	-0.93	0.08
Os9	chr10	126531314	126532125	-0.37	-0.04
Os9	chr10	126532244	126535544	-0.37	0.01
Os9	chr10	126535886	126536678	-1.25	0.17
Os9	chr10	126557594	126558216	-2.29	-0.30
Mbd6	chr10	126719161	126719461	-1.03	-0.13
Inhbe	chr10	126786931	126787169	-0.98	1.78
Ykt6	chr11	5855760	5859321	-0.36	-0.03
Usp34	chr11	23206894	23233439	-0.62	-0.12
Usp34	chr11	23263833	23264511	-0.87	0.06
Usp34	chr11	23388308	23389213	-0.34	-0.01
Psme4	chr11	30671774	30672135	-0.53	-0.17
Fbxw11	chr11	32644536	32644986	-0.65	0.06
Mgat1	chr11	49057752	49075721	-2.51	-0.17
Galnt10	chr11	57597286	57598644	-0.31	-0.02
Galnt10	chr11	57599150	57599241	-0.83	0.79
Hist3h2a	chr11	58768691	58768899	-0.65	0.26
Nlrp3	chr11	59379889	59380129	-0.34	0.02
Pigl	chr11	62271961	62272172	-1.69	0.58
Trp53	chr11	69393860	69394608	-1.25	-0.24
Ube2g1	chr11	72420792	72421062	-2.86	-0.55
Ube2g1	chr11	72497025	72497295	-0.29	0.00
Pigs	chr11	78141923	78142297	-0.34	-0.05
Pigs	chr11	78155437	78156158	-1.60	0.14
Suz12	chr11	79843530	79845978	-3.16	0.17
Psmd11	chr11	80285574	80285635	-1.26	-0.32
Ptrh2	chr11	86497514	86503722	-3.45	-0.19
Mrps23	chr11	88024206	88024950	-0.30	0.00
Mrpl27	chr11	94521138	94521226	-2.77	0.49
Gngt2	chr11	95703608	95705158	-0.47	-0.13
Mrpl10	chr11	96909612	96910026	-1.05	-0.12

Psmd3	chr11	98556993	98557203	-2.02	-0.21
Thra	chr11	98603186	98617477	-0.48	0.09
Thra	chr11	98625080	98626348	-0.46	0.05
Thra	chr11	98626407	98629662	-3.23	0.78
Rara	chr11	98799009	98808938	-0.63	0.23
Rara	chr11	98808997	98822389	-1.01	-0.17
Psme3	chr11	101177564	101178336	-0.77	0.09
Psme3	chr11	101182100	101183258	-2.86	-0.26
Dcaf7	chr11	105898185	105898515	-1.00	0.13
Dcaf7	chr11	105916210	105916330	-1.28	-0.23
Dcaf7	chr11	105919829	105920099	-0.29	0.00
Arsg	chr11	109346860	109378636	-0.29	0.00
Arsg	chr11	109433808	109434167	-0.34	0.02
Cog1	chr11	113517362	113518437	-0.45	-0.04
Sphk1	chr11	116393284	116393761	-0.64	3.81
Engase	chr11	118348441	118349432	-0.62	-0.17
Cbx2	chr11	118884462	118890969	-0.81	0.14
Hgs	chr11	120344969	120345268	-1.28	-0.22
Gps1	chr11	120648738	120649262	-0.86	-0.36
Gps1	chr11	120649939	120650416	-1.35	-0.22
Foxk2	chr11	121161028	121168992	-2.31	-0.14
Fn3k	chr11	121303052	121303292	-0.44	-2.25
Fn3k	chr11	121303589	121303799	-0.35	-1.32
Mrps24	chr11	5604429	5604637	-0.48	-0.05
Commd1	chr11	22799727	22800056	-0.70	0.07
Commd1	chr11	22856647	22878367	-0.34	0.03
Commd1	chr11	22878962	22882284	-1.57	-0.40
Pex13	chr11	23548957	23549226	-1.26	0.32
Pex13	chr11	23551042	23555931	-0.71	0.12
Sptbn1	chr11	30017714	30018324	-0.65	0.10
Sptbn1	chr11	30036124	30038367	-0.57	0.04
Sptbn1	chr11	30038786	30040543	-1.61	-0.15
Sptbn1	chr11	30119730	30168025	-0.37	0.07
Stc2	chr11	31259979	31260460	-1.02	-0.51
0610009B22Rik	chr11	51499092	51499534	-0.38	0.03
Sec24a	chr11	51556955	51570200	-1.50	-0.11
Derl2	chr11	70823390	70826702	-1.47	-0.13
Derl2	chr11	70832716	70832777	-0.27	0.01
Hic1	chr11	74978874	74979025	-0.69	1.22
Hic1	chr11	74979084	74979444	-0.39	-0.83
Hic1	chr11	74980641	74980732	-0.16	0.49
Rpa1	chr11	75153888	75161885	-1.45	-0.24
Gosr1	chr11	76543144	76550896	-0.53	-0.05

Eral1	chr11	77887262	77887988	-1.78	-0.25
Wsb1	chr11	79053599	79053898	-2.67	-0.17
Pex12	chr11	83111041	83111132	-0.98	-0.53
Pex12	chr11	83111280	83111913	-1.08	0.33
Pigw	chr11	84691181	84691984	-1.18	-0.17
Ube2z	chr11	95911464	95911704	-2.06	-0.26
Pcgf2	chr11	97551180	97551629	-0.96	-0.18
Pcgf2	chr11	97553081	97553393	-0.41	0.07
Fbxl20	chr11	97944196	97944257	-0.53	0.44
Klhl11	chr11	100323925	100325737	-2.89	0.19
Klhl11	chr11	100333857	100334096	-0.43	-0.10
Kat2a	chr11	100573480	100573601	-0.54	-0.16
Rab5c	chr11	100581475	100599444	-1.66	-0.43
Becn1	chr11	101163065	101163581	-0.94	-0.14
Brca1	chr11	101384754	101385384	-1.00	0.21
Brca1	chr11	101385443	101386611	-0.56	0.06
Brca1	chr11	101386909	101387689	-2.16	0.30
Nsf	chr11	103684322	103685071	-0.70	0.10
Nsf	chr11	103792113	103815310	-0.32	0.08
Ern1	chr11	106259052	106261558	-0.38	-0.02
Smurf2	chr11	106683743	106684358	-1.06	-0.24
Smurf2	chr11	106781579	106781849	-2.81	-0.99
Mrpl38	chr11	115993130	115993341	-0.72	0.12
Mrpl38	chr11	115999521	115999702	-0.67	0.30
Exoc7	chr11	116149909	116150478	-1.47	-0.41
Socs3	chr11	117830559	117830620	-0.38	0.48
Cbx4	chr11	118941759	118947461	-1.94	0.14
P4hb	chr11	120422393	120423503	-0.94	-0.04
Csnk1d	chr11	120819778	120820048	-2.61	-0.30
Csnk1d	chr11	120823779	120824109	-0.34	0.01
Csnk1d	chr11	120824767	120826288	-1.85	-0.13
Csnk1d	chr11	120852257	120852467	-0.47	-0.06
B3gntl1	chr11	121478301	121480951	-0.40	-0.03
Asxl2	chr12	3426883	3442534	-0.44	0.04
Asxl2	chr12	3496366	3497026	-3.15	0.17
Asxl2	chr12	3497115	3502535	-2.99	0.09
Smc6	chr12	11292342	11292522	-2.81	0.91
Smc6	chr12	11324583	11325004	-0.43	0.03
Cog5	chr12	32339733	32339824	-0.31	0.05
Psma6	chr12	56499811	56499900	-1.08	-0.18
Mia2	chr12	60208992	60209821	-1.76	0.98
Lrr1	chr12	70275442	70276002	-1.46	-0.50
Mgat2	chr12	70285182	70287153	-1.15	-0.05

Mgat2	chr12	70285144	70287136	-1.06	-0.04
Hif1a	chr12	75043067	75043308	-3.26	-0.17
Fut8	chr12	78432987	78466016	-0.68	-0.18
Fut8	chr12	78576163	78576944	-2.51	0.44
Dcaf4	chr12	84882463	84882673	-0.76	0.30
Eif2b2	chr12	86560462	86560886	-1.39	-0.12
Eif2b2	chr12	86567073	86567489	-0.83	0.10
Ttll5	chr12	87165652	87165773	-0.27	-0.03
Ttll5	chr12	87259905	87263926	-0.29	-0.01
Psmc1	chr12	101348377	101351316	-0.70	-0.11
Otub2	chr12	104641523	104643067	-0.48	0.06
Yy1	chr12	110031520	110044712	-3.35	-0.22
Dync1h1	chr12	111839634	111839815	-1.16	-0.25
Dync1h1	chr12	111855934	111857506	-0.62	-0.02
Dync1h1	chr12	111904592	111905154	-2.05	0.14
Wdr20	chr12	112017684	112032696	-0.40	0.02
Traf3	chr12	112499706	112500694	-0.68	-0.07
Eif5	chr12	112776341	112777093	-2.43	0.20
Ncoa1	chr12	4247570	4248468	-0.70	-0.10
Ncoa1	chr12	4248766	4252834	-0.39	-0.03
Ncoa1	chr12	4267709	4267949	-1.13	0.26
Ncoa1	chr12	4294980	4296117	-2.77	0.24
Trappc6b	chr12	60162299	60162448	-1.32	-0.37
Sptb	chr12	77714000	77714181	-0.13	-0.12
Rab15	chr12	77899366	77899456	-0.30	0.81
Pigh	chr12	80183212	80183482	-3.23	-0.81
Pigh	chr12	80190135	80190373	-2.29	1.30
Dcaf5	chr12	81437908	81438298	-0.47	-0.08
Dcaf5	chr12	81439313	81449328	-1.43	-0.10
Dcaf5	chr12	81537288	81537379	-1.00	0.54
Tmed10	chr12	86683962	86685208	-0.48	0.02
Tmed10	chr12	86715456	86715667	-0.68	-0.06
Pomt2	chr12	88449791	88451254	-1.85	-0.29
Pomt2	chr12	88488462	88488612	-0.26	-0.11
Sel1l	chr12	93046846	93048492	-0.47	-0.01
Sel1l	chr12	93080231	93087597	-2.13	-0.33
Atxn3	chr12	103157140	103157589	-0.64	-0.11
Ankrd9	chr12	112214221	112214281	-0.40	-2.32
Ankrd9	chr12	112214550	112215299	-1.40	-0.62
Ankrd9	chr12	112215597	112215718	-0.58	0.58
Ero1lb	chr13	12658149	12658389	-0.64	-0.17
Ero1lb	chr13	12698367	12699321	-3.28	0.99
B3galnt2	chr13	14090802	14091131	-3.29	-0.44

Inhba	chr13	16109320	16109440	-0.27	1.32
Inhba	chr13	16109499	16118119	-0.42	2.81
Trim27	chr13	21283975	21284366	-3.06	-0.20
Hist1h2bg	chr13	23663268	23663474	-3.01	-0.19
Hist1h2bc	chr13	23776067	23776213	-0.42	-0.04
Gpld1	chr13	25078887	25082252	-0.77	-0.16
Ripk1	chr13	34119764	34125274	-2.04	-0.13
Ripk1	chr13	34125393	34126112	-1.53	-0.25
2010111I01Rik	chr13	63116345	63134452	-0.51	0.06
2010111I01Rik	chr13	63400931	63403592	-0.68	-0.05
Mrpl36	chr13	73469131	73469277	-0.62	0.10
Arsb	chr13	94710635	94710756	-0.54	-0.12
Gfm2	chr13	97944981	97945927	-0.39	0.04
Mrps27	chr13	100184740	100185516	-1.87	-0.23
Ercc8	chr13	108948961	108949051	-1.87	-0.97
Gmds	chr13	31911574	31911785	-1.42	-0.36
Tubb2b	chr13	34219319	34219498	-1.13	2.05
Tubb2b	chr13	34220029	34220783	-1.25	-0.78
Nup153	chr13	46776258	46776499	-0.69	-0.10
Nup153	chr13	46782010	46792068	-2.74	0.10
Uimc1	chr13	55129866	55132327	-0.74	0.14
Uimc1	chr13	55176900	55177348	-1.67	-0.22
Rab24	chr13	55421264	55421501	-2.29	-0.29
Lman2	chr13	55447467	55447708	-1.84	-0.28
Lman2	chr13	55448036	55448336	-2.00	-0.16
Hnrnpk	chr13	58501349	58504674	-2.32	-0.21
Golm1	chr13	59736356	59736923	-0.80	-0.12
Golm1	chr13	59738410	59741528	-0.61	-0.10
Slc30a5	chr13	101572662	101573353	-0.88	-0.08
Kctd6	chr14	9052713	9055605	-1.86	-0.26
Atxn7	chr14	14932473	14936644	-2.02	-0.16
Sec24c	chr14	21512398	21513205	-0.75	-0.07
Sec24c	chr14	21513563	21514072	-1.48	-0.13
Actr8	chr14	30804011	30804814	-1.45	-0.43
Bap1	chr14	32070621	32070742	-0.87	0.20
Bap1	chr14	32072009	32072637	-2.29	-0.24
Psmc6	chr14	45949498	45950514	-0.58	0.08
Oxa1l	chr14	54988005	54988365	-0.86	0.10
Dcaf11	chr14	56178865	56178955	-0.66	-0.41
Dcaf11	chr14	56188485	56188902	-1.96	-0.28
Kbtbd7	chr14	79827126	79828716	-1.70	0.29
Gng2	chr14	20694828	20796262	-3.69	0.37
Spcs1	chr14	31813189	31813397	-0.78	0.07

Ankrd28	chr14	32514639	32518497	-1.72	-0.14
Dph3	chr14	32893866	32894315	-2.21	0.62
Exoc5	chr14	49633558	49633949	-1.77	0.18
Exoc5	chr14	49686221	49686282	-0.31	0.11
Nedd8	chr14	56290685	56290743	-0.55	-0.12
Rabggta	chr14	56340490	56340807	-2.27	0.59
Cog3	chr14	76102935	76102996	-0.58	0.26
Cog3	chr14	76103085	76106953	-2.15	-0.24
Commd6	chr14	102033311	102039300	-0.34	0.01
Fbxl3	chr14	103482090	103488631	-0.81	-0.05
Fbxl3	chr14	103494436	103498143	-0.54	-0.05
Fbxl3	chr14	103498202	103498726	-1.82	-0.43
Sema5a	chr15	32616573	32620160	-0.68	-0.09
Dcaf13	chr15	38944419	38950257	-0.48	0.07
Rnf139	chr15	58732325	58732416	-1.18	0.88
Ly6e	chr15	74786018	74786078	-1.11	0.25
Commd5	chr15	76730370	76731727	-1.84	-0.21
Apol9b	chr15	77566398	77566605	-1.11	2.04
Csf2rb	chr15	78156427	78166798	-0.34	0.02
Mgat3	chr15	80042031	80042182	-0.17	0.59
Rbx1	chr15	81305941	81306090	-0.83	-0.28
Ep300	chr15	81416643	81438767	-1.05	0.06
Ep300	chr15	81479273	81481819	-0.51	0.02
L3mbtl2	chr15	81516741	81518556	-1.73	-0.15
Nup50	chr15	84757805	84765852	-1.08	-0.07
Nup50	chr15	84773033	84773303	-0.62	0.12
Tuba1c	chr15	98867757	98867967	-0.36	0.01
Eif4b	chr15	101925104	101925897	-1.32	-0.05
Skp2	chr15	9043329	9043540	-0.80	0.27
Skp2	chr15	9069207	9070167	-0.68	-0.14
Rad21	chr15	51794834	51794954	-0.36	-0.02
Rad21	chr15	51798246	51799392	-0.87	-0.13
Mrpl13	chr15	55386902	55388867	-0.35	0.05
Fbxo32	chr15	58011872	58012083	-0.36	-0.16
Fbxo32	chr15	58012410	58013069	-1.46	-0.50
Fbxo32	chr15	58046223	58046433	-2.16	-1.12
St3gal1	chr15	66936954	66938364	-1.56	-0.14
St3gal1	chr15	66944037	67005237	-0.40	-0.02
Apol9a	chr15	77234337	77234723	-0.99	-0.99
Apol9a	chr15	77234811	77235109	-0.44	-0.50
Apol10b	chr15	77415802	77416129	-0.27	1.22
Apol8	chr15	77579072	77579283	-1.84	3.24
Csf2rb2	chr15	78114588	78116449	-1.22	-0.15

Ddx17	chr15	79359562	79359623	-3.23	-0.45
Ddx17	chr15	79376901	79377021	-1.72	-0.27
Josd1	chr15	79506286	79506585	-0.42	-0.06
Josd1	chr15	79517855	79518272	-1.01	0.24
Rangap1	chr15	81535546	81535636	-0.97	-0.19
Rangap1	chr15	81559691	81560349	-0.88	-0.22
Pmm1	chr15	81781535	81781891	-2.07	0.31
Ttll12	chr15	83406959	83407229	-1.63	-0.34
Alg12	chr15	88635911	88636421	-0.42	-0.05
Arsa	chr15	89303025	89303891	-1.14	0.17
Arsa	chr15	89305958	89306615	-3.10	-0.44
Vdr	chr15	97687093	97687333	-0.24	0.68
Senp1	chr15	97872854	97873303	-0.94	-0.13
Asb8	chr15	97965939	97971774	-0.39	0.02
Galnt6	chr15	100522228	100522647	-0.52	-0.09
Galnt6	chr15	100544605	100550892	-0.77	-0.08
Alg1	chr16	5244791	5245001	-0.28	0.00
Klhl22	chr16	17771956	17777358	-1.71	-0.20
Klhl22	chr16	17778345	17778466	-0.64	0.80
Psmd2	chr16	20651724	20652324	-3.25	-0.17
Eif4g1	chr16	20679306	20680085	-1.42	0.04
Senp2	chr16	22035915	22036096	-0.98	-0.61
Senp2	chr16	22046889	22047129	-1.33	-0.22
St6gal1	chr16	23321194	23321733	-0.65	0.14
Pigz	chr16	31944722	31944783	-0.24	-1.22
Rnf168	chr16	32277636	32282431	-0.66	0.10
Rnf168	chr16	32298648	32299428	-1.18	0.18
Golgb1	chr16	36914905	36915206	-2.70	0.34
Golgb1	chr16	36932031	36933171	-0.77	-0.08
Tomm70a	chr16	57152724	57153054	-1.60	-0.13
Usp25	chr16	77081892	77084046	-0.60	0.12
Usp25	chr16	77115246	77116156	-2.54	0.20
Usp16	chr16	87477080	87480005	-2.02	-0.15
Mrps6	chr16	92112030	92112325	-0.37	0.01
Eef2kmt	chr16	5244783	5245380	-1.09	0.16
Mrpl40	chr16	18872286	18872667	-1.80	-0.21
Senp5	chr16	31963352	31965869	-0.38	-0.03
Senp5	chr16	31989077	31990547	-0.59	0.03
Sec22a	chr16	35313599	35318950	-0.91	-0.16
Nrip1	chr16	76291196	76295213	-2.02	0.11
Nrip1	chr16	76352759	76374042	-1.51	-0.61
App	chr16	85025115	85043863	-0.75	-0.03
App	chr16	85043922	85056713	-2.06	-0.14

App	chr16	85120877	85173892	-2.41	-0.23
Cct8	chr16	87496053	87496114	-0.58	0.12
Tulp4	chr17	6232220	6232851	-2.79	-0.26
Tulp4	chr17	6232910	6233480	-1.17	-0.24
Tulp4	chr17	6234139	6236560	-2.06	0.41
Tcp1	chr17	13117452	13117603	-0.95	-0.24
Abca3	chr17	24546305	24547146	-0.49	0.04
Spsb3	chr17	25023707	25024470	-2.46	-0.59
Spsb3	chr17	25024809	25029092	-1.63	-0.14
Mrps34	chr17	25032064	25033497	-3.15	-0.20
Axin1	chr17	26275840	26310679	-1.19	0.10
Axin1	chr17	26319507	26325117	-3.16	-0.30
Axin1	chr17	26330938	26332096	-0.33	-0.02
Adamts10	chr17	33665942	33666153	-0.99	-0.46
Adamts10	chr17	33667079	33667439	-1.19	-0.43
Adamts10	chr17	33690397	33690697	-3.34	-0.81
Psmb8	chr17	34335288	34336214	-1.01	-0.16
Neu1	chr17	35071427	35072869	-3.32	-0.17
Mdc1	chr17	35983321	35985582	-1.83	-0.14
Mdc1	chr17	35987436	35987527	-3.30	0.71
Mdc1	chr17	35987825	35988082	-1.08	0.26
Mdc1	chr17	35989399	35989938	-0.34	0.01
Mdc1	chr17	35994492	35994583	-0.46	0.09
Mrpl14	chr17	45834998	45835444	-0.76	-0.08
Mrps18a	chr17	46247952	46262241	-0.79	-0.09
Mrpl2	chr17	46783196	46783346	-1.97	-0.40
Cul7	chr17	46787586	46787707	-0.19	0.03
Cul7	chr17	46788456	46789468	-0.30	-0.09
Mrps10	chr17	47505835	47506196	-2.01	-0.75
Rab5a	chr17	53618648	53623279	-1.12	0.12
Rab5a	chr17	53639752	53646076	-0.68	-0.06
Kat2b	chr17	53706385	53706446	-0.22	0.15
Fem1a	chr17	56400758	56401238	-0.39	0.05
Fem1a	chr17	56401745	56402702	-0.37	0.03
Safb	chr17	56724404	56724555	-1.20	0.29
Safb	chr17	56733308	56735128	-1.28	-0.14
Safb	chr17	56737716	56737897	-1.70	0.51
Safb	chr17	56738105	56738676	-2.46	-0.21
Man2a1	chr17	64950988	64974652	-0.82	-0.11
Slc30a6	chr17	74822435	74823062	-0.37	0.03
Socs5	chr17	87507287	87507348	-0.71	-0.42
Socs5	chr17	87520106	87534893	-3.68	0.22
Mrpl18	chr17	13104220	13104580	-3.52	0.31

Ccnf	chr17	24360773	24360864	-1.19	-0.44
Ube2i	chr17	25397995	25398776	-0.81	-0.11
Rab40c	chr17	26020165	26020794	-1.48	0.23
Pigq	chr17	26064506	26064597	-0.31	-0.01
Pigq	chr17	26073565	26073656	-0.30	0.10
Pigq	chr17	26073775	26078847	-1.47	-0.13
Rab11b	chr17	33883909	33884149	-2.85	-0.29
Rab11b	chr17	33884297	33884597	-1.25	0.23
Rab11b	chr17	33897340	33897431	-1.34	0.73
Ring1	chr17	34158155	34160310	-1.41	-0.14
Ring1	chr17	34160369	34161265	-0.31	-0.04
Ly6g6d	chr17	35208292	35208667	-1.07	2.51
Cul9	chr17	46680184	46683217	-0.34	0.04
Rab31	chr17	66001065	66001632	-0.47	0.02
Rab31	chr17	66003418	66017287	-0.77	0.04
Rab31	chr17	66122031	66122092	-0.23	-0.05
Rab12	chr17	66844211	66844991	-0.97	0.09
Pigf	chr17	87396598	87396896	-3.10	0.22
Mcfd2	chr17	87654440	87654561	-0.80	0.23
Mcfd2	chr17	87654739	87656518	-1.65	0.15
Calm2	chr17	87835011	87846216	-3.10	-0.20
Fbxo11	chr17	88390945	88391656	-0.52	0.03
Wac	chr18	7869438	7869558	-1.82	-0.66
Wac	chr18	7905960	7910820	-1.76	0.12
Galnt1	chr18	24438485	24443794	-2.19	-0.18
Apc	chr18	34455759	34458292	-1.70	0.44
Ube2d2a	chr18	35931242	35931392	-1.92	-0.45
Ube2d2a	chr18	35965360	35965570	-1.78	0.47
Commd10	chr18	47246724	47246904	-0.46	0.06
Dctn4	chr18	60686409	60686590	-0.35	0.21
Dctn4	chr18	60716345	60716496	-0.75	-0.18
Napg	chr18	63154672	63156813	-3.08	-0.29
Sec11c	chr18	65960231	65960321	-0.85	-0.18
Tubb6	chr18	67550384	67552330	-3.31	-0.30
Mbd1	chr18	74437069	74438854	-1.40	-0.21
Mbd1	chr18	74439929	74440708	-2.55	-0.31
Mbd1	chr18	74441560	74442218	-3.23	-0.32
Smad7	chr18	75527018	75527734	-0.56	0.14
Smad7	chr18	75528210	75530550	-2.26	0.57
Smad2	chr18	76422187	76422338	-0.39	0.04
Smad2	chr18	76462139	76464727	-0.86	-0.12
Pias2	chr18	77304096	77304536	-2.25	0.61
Pias2	chr18	77304625	77336416	-0.40	-0.02

Pias2	chr18	77388818	77391933	-0.34	0.01
Usp14	chr18	10030026	10030087	-1.17	-0.30
B4galt6	chr18	20846332	20847761	-1.60	-0.24
Ino80c	chr18	24263918	24264068	-1.24	-0.33
Ino80c	chr18	24264545	24270229	-0.93	0.13
Etf1	chr18	35064409	35064620	-3.56	-0.17
Etf1	chr18	35073803	35091657	-2.12	-0.15
Nr3c1	chr18	39588392	39649457	-3.45	0.21
Fem1c	chr18	46664557	46685565	-0.41	-0.01
Tmed7	chr18	46747763	46748242	-0.50	-0.03
Lman1	chr18	66140572	66140992	-1.89	0.18
Lman1	chr18	66151175	66151326	-1.49	-0.18
Lman1	chr18	66162078	66162289	-0.76	0.11
Smad4	chr18	73799858	73800008	-0.66	-0.12
Smad4	chr18	73863185	73863395	-3.16	-1.68
Socs6	chr18	89038427	89056448	-0.39	-0.02
Sptbn2	chr19	4744084	4745130	-1.43	-0.66
B4gat1	chr19	5041278	5046482	-2.34	-0.20
Rela	chr19	5637578	5637639	-0.26	0.03
Fau	chr19	6057887	6057975	-1.20	0.60
Men1	chr19	6335008	6336781	-2.54	-0.27
Men1	chr19	6338873	6340864	-1.37	-0.10
Trmt112	chr19	6984277	6984947	-1.86	-0.15
Stx5a	chr19	8829416	8830132	-0.49	0.04
Ubxn1	chr19	8945985	8946226	-0.36	0.03
Mrpl16	chr19	11848755	11849170	-0.34	-0.01
Uhrf2	chr19	30105002	30105212	-1.71	-0.42
Uhrf2	chr19	30113733	30131060	-3.08	0.29
Stambpl1	chr19	34266759	34267030	-1.66	-0.25
Stambpl1	chr19	34307271	34308667	-1.01	0.18
Exoc6	chr19	37757411	37757650	-0.93	0.17
Btrc	chr19	45604207	45604268	-0.27	-0.42
Gbf1	chr19	46227047	46227258	-0.79	0.19
Gbf1	chr19	46360279	46360430	-0.70	0.15
Nfkb2	chr19	46379346	46380320	-1.80	0.46
Fbxl15	chr19	46403619	46404936	-0.68	0.11
Adra2a	chr19	54122063	54122273	-0.62	2.97
Adra2a	chr19	54122362	54122423	-0.48	2.70
Adra2a	chr19	54122065	54122275	-0.62	2.97
1700020D05Rik	chr19	5502888	5503128	-0.65	0.38
1700020D05Rik	chr19	5503217	5503398	-0.29	0.08
1700020D05Rik	chr19	5503487	5503787	-0.38	0.25
Incenp	chr19	9947145	9947295	-0.39	0.02

Incenp	chr19	9949324	9952191	-1.89	0.14
Incenp	chr19	9958207	9968481	-3.67	-0.11
Incenp	chr19	9969946	9974023	-0.62	0.11
Gcnt1	chr19	17403142	17407708	-3.03	0.20
Smc5	chr19	23348276	23348367	-0.83	-0.31
Fbxw4	chr19	45652836	45653256	-0.43	-0.03
Actr1a	chr19	46452405	46452913	-1.68	-0.11
Eif3a	chr19	60837737	60844695	-1.12	0.02
Eif3a	chr19	60844784	60846963	-2.75	0.09
Usp11	chrX	20281154	20281215	-0.29	-0.12
Ube2a	chrX	34414360	34414479	-0.33	-0.03
Ikbkg	chrX	71675074	71678148	-0.70	-0.30
Ogt	chrX	98874640	98878285	-0.71	0.04
Ogt	chrX	98878792	98878973	-1.90	-0.42
Ogt	chrX	98879151	98879242	-0.52	-0.19
Piga	chrX	160866941	160869822	-1.00	0.37
Piga	chrX	160870267	160870507	-2.83	1.12
Cfp	chrX	20508559	20508650	-1.66	0.59
C1galt1c1	chrX	35984136	35988290	-0.62	-0.06
Hcfc1	chrX	71194680	71196000	-1.71	-0.08
Hcfc1	chrX	71211533	71211654	-0.84	0.13
Mbtps2	chrX	153988770	153989011	-0.74	0.15
Mbtps2	chrX	154006202	154006563	-0.37	-0.07
Yy2	chrX	154005692	154006559	-0.47	0.07
Yy2	chrX	154005705	154006545	-0.50	0.08
Napb	chr2	148538436	148558156	-2.45	0.80
Tnks	chr8	36028504	36028744	-1.36	-0.36
Dynll2	chr11	87794645	87799923	-0.49	0.03

Post translational protein modification

gene_name	chr	chromStart	chromEnd	diff.lg.p	diff.log2.fc
Rab23	chr1	33795210	33796982	-1.68	-0.29
Ttll4	chr1	74708327	74727946	-2.60	0.14
Ttll4	chr1	74744068	74744368	-2.07	-0.43
Sp100	chr1	87605814	87606023	-0.86	0.43
Psmd1	chr1	87961193	87961313	-0.67	0.21
Cops8	chr1	92508845	92509678	-0.59	-0.04
Ube2f	chr1	93182063	93182512	-2.14	-0.16
Asb1	chr1	93437351	93443741	-2.00	0.50
Asb1	chr1	93448832	93448983	-0.89	0.38
Asb1	chr1	93451373	93451764	-0.41	0.07
Mgat5	chr1	129203315	129203555	-1.22	0.30
Mgat5	chr1	129376047	129379549	-0.34	-0.01
Yod1	chr1	132613903	132614024	-0.26	-0.33

Yod1	chr1	132614351	132616601	-0.85	0.10
Rab7b	chr1	133585271	133594407	-0.60	0.14
Rab7b	chr1	133602280	133610067	-1.33	-0.11
Rab29	chr1	133763853	133763943	-0.50	-0.39
Rbbp5	chr1	134394590	134400982	-1.17	0.15
Rbbp5	chr1	134401131	134401312	-0.34	-0.03
Ube2t	chr1	136859153	136864304	-1.69	-0.60
Edem3	chr1	153658769	153666557	-0.61	0.05
F5	chr1	166120421	166122204	-1.15	2.81
F5	chr1	166122382	166122503	-0.93	3.42
F5	chr1	166123102	166123193	-0.44	1.85
F5	chr1	166124151	166124452	-2.11	3.30
Fcgr4	chr1	172959267	172959892	-3.04	0.51
Adamts4	chr1	173189096	173189157	-0.26	-0.91
Adamts4	chr1	173189215	173189276	-0.16	-0.42
B4galt3	chr1	173206252	173206818	-0.59	0.06
Dcaf8	chr1	174078235	174078505	-0.95	-0.24
Dcaf8	chr1	174124786	174125087	-1.13	-0.19
Pigm	chr1	174306661	174308397	-2.87	0.16
Parp1	chr1	182499105	182503843	-1.60	-0.27
Cops5	chr1	10027799	10027979	-1.87	0.21
Uggt1	chr1	36200615	36203523	-0.76	-0.05
Uggt1	chr1	36300936	36301147	-3.25	-0.47
Mgat4a	chr1	37501120	37501720	-2.66	0.34
Chst10	chr1	38920717	38922995	-0.68	0.03
Satb2	chr1	56852675	56854020	-0.34	0.01
Satb2	chr1	56928415	56948033	-1.33	0.25
Satb2	chr1	57026707	57028178	-0.42	-0.07
Sumo1	chr1	59696455	59696989	-1.11	-0.09
Ino80d	chr1	63121070	63139998	-2.26	-0.15
Bard1	chr1	71121331	71122020	-0.53	0.08
Usp37	chr1	74485767	74485918	-0.64	0.18
Tuba4a	chr1	75211756	75211936	-0.82	-0.43
Cul3	chr1	80336885	80337005	-1.62	-0.38
Pign	chr1	107560192	107560253	-0.80	0.36
Ikbke	chr1	133151208	133153534	-0.51	-0.04
Ikbke	chr1	133173083	133173488	-3.28	-0.78
Rnf2	chr1	153318408	153318528	-0.89	0.28
Lamc1	chr1	155080624	155081688	-1.33	-0.18
Lamc1	chr1	155179317	155179856	-2.16	-0.21
Qsox1	chr1	157626418	157628411	-1.52	-0.23
Klhl20	chr1	163019047	163019258	-1.39	-0.43
Klhl20	chr1	163039327	163060580	-0.32	0.01

Dcaf6	chr1	167281032	167281331	-1.63	0.37
Usp21	chr1	173216496	173218092	-3.29	-0.45
Dtl	chr1	193363068	193363969	-0.39	0.07
Dtl	chr1	193364536	193365046	-0.52	-0.08
Kin	chr2	10013347	10014328	-0.81	0.10
Stam	chr2	14067864	14068462	-3.00	0.37
Commd3	chr2	18596461	18597638	-0.89	0.08
Bmi1	chr2	18605667	18606468	-0.89	0.08
Fbxw5	chr2	25358791	25359805	-1.19	0.14
Fbxw5	chr2	25360013	25360452	-1.24	-0.22
Fbxw5	chr2	25360840	25360960	-3.19	1.04
Adamts13	chr2	26829055	26829510	-0.47	0.39
Rxra	chr2	27597444	27604173	-0.47	-0.11
Rxra	chr2	27617342	27618510	-1.22	-0.15
Dolpp1	chr2	30252653	30255005	-0.54	-0.11
Usp20	chr2	30863351	30865900	-0.51	-0.13
Nup214	chr2	31888785	31893653	-1.32	0.09
Pomt1	chr2	32109927	32110226	-1.75	-0.42
Golga2	chr2	32158521	32159772	-1.15	-0.16
Golga2	chr2	32161974	32162244	-1.46	0.29
Dpm2	chr2	32427908	32429091	-1.97	-0.18
St6galnac4	chr2	32442607	32443050	-0.76	0.31
St6galnac4	chr2	32449589	32449950	-1.02	-0.18
St6galnac4	chr2	32451265	32451326	-0.40	0.15
St6galnac4	chr2	32452522	32453780	-3.42	-0.26
Mbd5	chr2	49101163	49101373	-2.40	0.76
Mbd5	chr2	49111717	49113872	-3.69	0.28
Mbd5	chr2	49134326	49172128	-0.55	0.13
Klhl41	chr2	69508654	69508893	-1.32	0.66
Dync1i2	chr2	71049792	71049883	-0.39	0.11
Psmc3	chr2	90894439	90895182	-1.38	-0.10
Traf6	chr2	101531587	101537929	-3.55	-0.22
Rtf1	chr2	119558206	119559195	-0.92	-0.10
Rtf1	chr2	119560243	119560484	-1.04	0.33
Usp8	chr2	126563777	126568417	-0.85	0.06
Prnd	chr2	131735693	131776618	-0.69	0.31
Tbc1d20	chr2	152119607	152127928	-0.69	-0.11
Tbc1d20	chr2	152137106	152137759	-1.14	-0.14
Asxl1	chr2	153222520	153228367	-3.49	-0.09
Actr5	chr2	158450648	158451096	-3.11	-0.77
Actr5	chr2	158462488	158464947	-1.71	-0.32
Top1	chr2	160471632	160471962	-0.61	-0.07
Rae1	chr2	172839488	172841203	-2.88	-0.32

Rab22a	chr2	173521497	173526997	-1.35	-0.15
Adrm1	chr2	179906292	179906906	-2.93	-0.47
Gata3	chr2	9779567	9784722	-1.35	0.40
Gata3	chr2	9784840	9796310	-0.60	0.24
Tubb4b	chr2	25077884	25079546	-0.33	0.00
Tubb4b	chr2	25079713	25080192	-0.31	-0.02
Traf2	chr2	25374427	25375145	-1.34	-0.17
Sec16a	chr2	26265370	26265521	-1.26	-0.31
Sec16a	chr2	26277747	26279943	-0.76	0.12
Sec16a	chr2	26294102	26294703	-3.27	0.24
Sec16a	chr2	26295062	26300736	-2.69	0.11
Ntng2	chr2	29063096	29103560	-0.32	-0.05
Dolk	chr2	30139748	30140407	-0.33	-0.02
Dolk	chr2	30140646	30141844	-1.09	-0.17
Fbxw2	chr2	34661048	34661869	-2.12	-0.20
Fbxw2	chr2	34662764	34664040	-0.46	-0.08
Fbxw2	chr2	34668436	34678672	-1.12	-0.10
Fbxw2	chr2	34681468	34681725	-0.58	0.19
Psmd5	chr2	34707993	34709794	-1.37	-0.17
Psmd5	chr2	34722532	34726482	-1.60	-0.40
Psmb7	chr2	38498946	38499426	-1.35	-0.19
Stam2	chr2	52579981	52595233	-0.55	0.15
Stam2	chr2	52601122	52601183	-0.31	-0.06
Sp3	chr2	72776048	72779137	-2.88	0.20
Slc35c1	chr2	92293966	92294832	-2.30	-0.24
Slc35c1	chr2	92298872	92299619	-0.47	0.04
Slc35c1	chr2	92299947	92300675	-0.27	-0.42
Rcn1	chr2	105227977	105229285	-0.79	-0.11
Rcn1	chr2	105239148	105239446	-0.74	0.18
Ino80	chr2	119200037	119200278	-1.19	0.27
Ino80	chr2	119303244	119303365	-0.39	-0.13
Trp53bp1	chr2	121030697	121033825	-0.66	0.11
Trp53bp1	chr2	121034169	121046975	-1.32	-0.15
Trp53bp1	chr2	121061313	121069657	-0.32	0.01
Pcna	chr2	132078555	132078916	-3.65	-0.19
Psmf1	chr2	151543111	151543232	-0.83	-0.47
Psmf1	chr2	151543649	151544068	-0.81	-0.19
Edem2	chr2	155527795	155531394	-0.45	0.03
Edem2	chr2	155555031	155555181	-0.49	0.12
Neurl2	chr2	164656229	164658296	-2.89	-0.44
Neurl2	chr2	164658917	164659037	-3.40	1.18
B4galt5	chr2	167126418	167130545	-2.25	-0.10
Cp	chr3	19892644	19892765	-0.56	0.22

Fbxw7	chr3	84619498	84694070	-1.92	0.38
Fbxw7	chr3	84694129	84756226	-2.05	0.20
Fbxw7	chr3	84780140	84781955	-0.37	0.02
Rab13	chr3	90028667	90029826	-0.92	0.28
Otud7b	chr3	95956546	95960193	-2.15	-0.13
Hist2h2be	chr3	96025043	96025490	-1.83	0.52
Hist2h2be	chr3	96026738	96027215	-1.30	-0.39
Sec22b	chr3	97725140	97725438	-2.99	-0.26
Dph5	chr3	115631611	115631971	-1.70	-0.32
Ube2d3	chr3	135101656	135102228	-3.24	-0.27
Bcl10	chr3	145587232	145587592	-0.60	0.07
Bcl10	chr3	145588099	145593615	-2.28	0.24
Usp33	chr3	152029782	152033363	-0.30	0.00
Pigk	chr3	152448391	152449139	-0.87	0.13
Pigk	chr3	152449825	152450513	-2.48	0.40
Pigk	chr3	152451588	152451857	-2.02	0.56
Pex2	chr3	5561142	5563166	-2.95	-0.27
Phc3	chr3	30805887	30813151	-0.57	-0.08
Phc3	chr3	30864703	30868337	-0.42	0.12
Cog6	chr3	52798099	52798220	-0.15	-0.29
Commd2	chr3	57450303	57450573	-2.39	-0.46
Siah2	chr3	58495620	58495891	-1.37	0.55
Psmd4	chr3	94836626	94836924	-1.79	-0.12
Psmd4	chr3	94839816	94846467	-2.16	-0.13
Hist2h2aa1	chr3	96044071	96044216	-1.04	1.17
Man1a2	chr3	100371071	100382434	-1.39	-0.12
Man1a2	chr3	100460030	100489396	-1.61	-0.09
Csf1	chr3	107544354	107544444	-0.83	0.84
Csf1	chr3	107544832	107548597	-0.29	-0.01
Csf1	chr3	107548745	107550706	-1.24	-1.52
Csf1	chr3	107551063	107553281	-0.37	0.05
Rwdd3	chr3	120859334	120862148	-1.36	0.36
Ank2	chr3	126625873	126626864	-0.77	0.39
Sec24b	chr3	129686102	129687101	-0.83	-0.07
Cyr61	chr3	145310530	145310591	-0.46	2.58
Fpgt	chr3	154749553	154753118	-0.61	0.07
Fbxl4	chr4	22303713	22313319	-1.70	0.24
Fbxl4	chr4	22354425	22354486	-0.94	0.57
Map3k7	chr4	32051081	32051232	-2.61	-1.03
Map3k7	chr4	32109961	32110261	-0.43	0.08
Ube2r2	chr4	41137777	41138136	-1.17	0.13
Dcaf10	chr4	45355032	45355512	-0.76	-0.17
Dcaf10	chr4	45385860	45387298	-0.73	-0.11

Nans	chr4	46515563	46516280	-0.44	-0.04
Tgfbr1	chr4	47419877	47423717	-1.19	0.29
Stx17	chr4	48197791	48198211	-0.64	0.17
Rad23b	chr4	55398385	55402936	-0.95	0.07
Rraga	chr4	86221606	86222293	-0.53	0.03
Usp24	chr4	105988817	105989117	-1.06	-0.35
Usp24	chr4	106111387	106111867	-0.45	-0.03
Usp24	chr4	106112884	106113842	-2.89	0.24
Ndc1	chr4	107083754	107086943	-0.44	0.04
Lrrc41	chr4	115761466	115762183	-1.09	-0.08
Lrrc41	chr4	115768961	115769260	-0.52	0.05
Scmh1	chr4	120201875	120202085	-2.07	0.58
Trappc3	chr4	125939648	125949968	-0.77	-0.13
Trappc3	chr4	125951250	125952918	-1.12	-0.09
Clspn	chr4	126237164	126239019	-0.31	-0.01
Clspn	chr4	126243223	126249609	-0.34	-0.02
Clspn	chr4	126270038	126270788	-1.23	-0.26
Phc2	chr4	128404910	128405061	-1.05	-0.71
Ptp4a2	chr4	129497464	129516451	-0.43	-0.02
Fuca1	chr4	135495027	135495355	-0.80	0.11
Usp48	chr4	137211154	137213554	-1.84	0.15
Mul1	chr4	137996388	137996837	-0.29	0.01
Capzb	chr4	138748845	138794946	-0.34	-0.01
Fbxo2	chr4	147538344	147538434	-0.25	-0.78
Klhl21	chr4	151383089	151389959	-0.56	0.05
Klhl21	chr4	151390287	151390408	-0.53	0.22
Icmt	chr4	151674797	151677845	-1.75	-0.20
Mxra8	chr4	155217437	155217527	-0.27	-0.15
Ripk2	chr4	16050729	16051621	-2.11	-0.28
Manea	chr4	26254667	26273799	-0.95	0.09
Ddx58	chr4	40152603	40155517	-1.64	0.25
B4galt1	chr4	40753786	40754830	-0.76	-0.05
B4galt1	chr4	40770567	40801001	-1.03	0.10
Vcp	chr4	43013259	43013349	-0.48	-0.16
Gne	chr4	44047845	44048266	-1.53	-0.50
Gne	chr4	44049104	44049255	-0.80	0.70
Gne	chr4	44068129	44079675	-0.31	0.01
Fbxo10	chr4	45048105	45048374	-0.47	0.54
Fbxo10	chr4	45048433	45048494	-0.73	1.50
Fbxo10	chr4	45048553	45048793	-0.74	0.68
Fbxo10	chr4	45071323	45071922	-0.48	0.30
Alg2	chr4	47482973	47483543	-1.01	0.16
Mysm1	chr4	94631563	94631714	-1.83	1.14

Mysm1	chr4	94631833	94632492	-3.38	0.30
Dph2	chr4	117562115	117562206	-0.41	0.14
Dph2	chr4	117562463	117563268	-1.81	-0.28
Dph2	chr4	117564417	117564628	-0.78	-0.27
St3gal3	chr4	117604757	117612708	-0.45	-0.04
Cdc20	chr4	118109887	118109948	-0.55	-0.37
Rab42	chr4	131858314	131858373	-0.31	-1.81
Wdtc1	chr4	132849782	132850290	-2.36	-0.35
Pigv	chr4	133218200	133220602	-2.57	-0.58
Pigv	chr4	133228472	133228562	-0.59	0.47
Dhdds	chr4	133526767	133536084	-0.36	-0.02
Man1c1	chr4	134119221	134119701	-0.62	-0.06
Otud3	chr4	138451293	138453499	-0.59	0.08
Fbxo6	chr4	147520063	147520213	-1.03	0.25
Pex14	chr4	148335210	148335898	-1.74	-0.17
Pex14	chr4	148358178	148473921	-0.61	0.08
Spsb1	chr4	149271827	149281337	-1.02	0.57
Nupl2	chr5	23687756	23687817	-0.27	0.02
Nupl2	chr5	23688026	23689736	-0.62	0.07
Nub1	chr5	24191722	24195229	-0.45	-0.13
Nub1	chr5	24216014	24216194	-0.28	0.00
Galnt11	chr5	24771083	24771350	-1.41	0.32
Bst1	chr5	44210131	44210281	-0.56	-0.24
Klhl5	chr5	65558211	65558721	-1.61	0.23
Dcun1d4	chr5	73950743	73951343	-1.40	0.48
Uso1	chr5	92630175	92631402	-0.50	0.03
Pigg	chr5	108776899	108777289	-0.58	-0.18
Usp30	chr5	114572302	114572933	-2.44	-0.39
Rab35	chr5	116082025	116082085	-0.40	0.30
Wsb2	chr5	117827434	117828121	-0.36	0.02
Fbxo21	chr5	118426838	118426928	-0.57	0.34
Fbxo21	chr5	118452063	118458648	-0.32	0.01
Psmd9	chr5	123678228	123684616	-1.07	0.18
B3gnt4	chr5	123961238	123961328	-0.20	-0.03
Sumf2	chr5	130338811	130339081	-0.89	-0.45
Kctd7	chr5	130628247	130628815	-1.33	-0.62
Vgf	chr5	137506960	137507972	-0.59	-0.23
Vgf	chr5	137508209	137508864	-0.79	-0.47
Cops6	chr5	138605218	138605278	-0.72	-0.47
Fam20c	chr5	139231034	139232111	-1.21	-0.07
Fam20c	chr5	139284048	139286017	-3.54	-0.23
Foxk1	chr5	142931509	142934800	-1.90	-0.10
Kdelr2	chr5	144165498	144165648	-0.43	-0.06

Trrap	chr5	145550403	145551932	-0.83	-0.16
Preb	chr5	31254099	31254400	-0.89	0.35
Preb	chr5	31255028	31255718	-1.40	0.29
Preb	chr5	31256227	31256528	-0.48	-0.11
Preb	chr5	31257006	31257247	-0.73	0.25
Preb	chr5	31257366	31258308	-0.47	-0.02
Preb	chr5	31262373	31262704	-2.91	-0.42
Tnip2	chr5	34839221	34839491	-0.36	0.04
Tada2b	chr5	36818228	36826755	-0.36	-0.01
Wfs1	chr5	37358091	37358361	-0.55	0.38
Wfs1	chr5	37358629	37358750	-0.90	-1.02
Wfs1	chr5	37358929	37359708	-0.31	0.02
Fbxl5	chr5	44142150	44149098	-2.30	-0.48
Fbxl5	chr5	44149396	44151973	-0.42	-0.02
Commd8	chr5	72550637	72551057	-0.88	0.12
Nup54	chr5	92845130	92846543	-1.56	0.19
Sec31a	chr5	100790877	100791058	-0.38	0.02
Abraxas1	chr5	101234952	101235460	-0.91	-0.18
Mlec	chr5	115597834	115600228	-0.64	-0.05
Suds3	chr5	117542584	117542913	-1.68	-0.19
Suds3	chr5	117558367	117565972	-1.81	-0.18
Fbxw8	chr5	118515497	118515797	-0.35	-0.06
Pom121	chr5	135853620	135857198	-0.30	0.00
Pom121	chr5	135857496	135857795	-0.92	0.12
Pom121	chr5	135869997	135870416	-0.68	-0.16
Fbxl18	chr5	143633766	143634186	-0.39	0.07
Fbxl18	chr5	143640382	143644224	-0.93	-0.38
Fbxl18	chr5	143650503	143656917	-0.38	-0.27
Usp42	chr5	144471642	144477010	-1.74	-0.15
Usp42	chr5	144477457	144478068	-0.31	0.01
Usp12	chr5	147548927	147550725	-1.46	-0.24
Calu	chr6	29298148	29306560	-1.10	0.22
Calu	chr6	29322584	29324851	-1.44	0.09
Nup205	chr6	35127615	35133895	-0.29	0.01
Rab19	chr6	39339614	39340319	-0.89	-0.29
Tnip3	chr6	65564764	65582093	-2.86	0.24
Rnf103	chr6	71444126	71444217	-0.35	-0.24
St3gal5	chr6	72092172	72097116	-0.95	0.10
Dctn1	chr6	83140226	83141067	-0.66	-0.06
Ruvbl1	chr6	88415416	88415624	-0.60	0.20
Kbtbd8	chr6	95071961	95077327	-2.16	0.32
Mitf	chr6	97969644	97970094	-1.02	0.16
Edem1	chr6	108805488	108805668	-2.85	-0.15

Ttll3	chr6	113357294	113359347	-3.58	0.53
Ttll3	chr6	113362865	113364505	-1.18	-0.20
Vhl	chr6	113578147	113579978	-1.16	0.21
Fbxl14	chr6	119429685	119431451	-2.73	-0.15
Rad52	chr6	119865354	119869071	-1.84	0.25
Rad52	chr6	119870928	119871139	-1.97	0.40
Rad52	chr6	119871288	119872841	-0.64	0.11
Usp18	chr6	121195982	121196071	-0.23	-0.06
Cmas	chr6	142705235	142705504	-3.29	-0.63
Klhl42	chr6	147039984	147056970	-0.73	-0.10
Etfbkmt	chr6	149090148	149092915	-0.36	-0.07
Ube2h	chr6	30163827	30164395	-0.64	0.08
Ube2h	chr6	30164514	30164664	-1.04	0.40
Ube2h	chr6	30164783	30164993	-0.55	0.22
Hipk2	chr6	38647868	38666025	-2.58	-0.11
Hipk2	chr6	38697991	38787047	-0.54	-0.02
Hipk2	chr6	38825859	38826040	-2.58	-0.39
Nod1	chr6	54874621	54874682	-0.58	2.04
Nod1	chr6	54893198	54893646	-1.73	2.07
Nod1	chr6	54894093	54894451	-0.13	0.08
Nod1	chr6	54894629	54894929	-0.30	0.33
Rnf181	chr6	72310209	72310594	-0.35	-0.02
Tgoln1	chr6	72561733	72566785	-0.76	-0.03
Ino80b	chr6	83074670	83075023	-0.62	0.15
Rab43	chr6	87739684	87739804	-0.33	-0.04
Rab43	chr6	87761443	87761773	-0.72	0.19
Nup210	chr6	90963060	90963720	-3.45	-0.38
Xpc	chr6	91439866	91440135	-3.04	-0.81
Xpc	chr6	91448100	91450008	-0.70	-0.11
Xpc	chr6	91460626	91465852	-1.01	0.46
Sumf1	chr6	108058060	108058479	-0.75	0.10
Sumf1	chr6	108135307	108135577	-1.12	-0.19
Rad18	chr6	112577031	112577691	-0.71	0.12
Tada3	chr6	113322347	113324488	-0.52	0.12
Phc1	chr6	122273438	122273618	-1.06	0.49
Phc1	chr6	122275560	122275830	-0.30	-0.03
Pex5	chr6	124347220	124347340	-0.93	-0.27
Usp5	chr6	124776424	124779465	-2.08	-0.36
Trappc6a	chr7	20094077	20099806	-2.85	-0.29
Plaur	chr7	25257191	25257252	-0.42	-0.17
B3gnt8	chr7	26415160	26415551	-1.21	-0.16
Paf1	chr7	29178014	29178074	-0.35	0.09
Paf1	chr7	29181953	29184402	-2.55	-0.12

Etfb	chr7	50699512	50707736	-0.77	-0.18
Josd2	chr7	51723425	51723690	-0.32	-0.03
Hrc	chr7	52590698	52591209	-0.35	-0.26
Hrc	chr7	52591478	52591689	-0.77	1.06
Prmt3	chr7	57112887	57113008	-3.64	-0.59
Herc2	chr7	63485102	63486928	-1.77	0.13
Klhl25	chr7	82993342	83012197	-1.00	0.11
Klhl25	chr7	83012375	83018299	-0.87	-0.23
Klhl25	chr7	83018716	83018926	-0.35	-0.11
Ctsc	chr7	95437215	95437515	-0.81	0.31
Ctsc	chr7	95458037	95458487	-2.34	0.25
Rab30	chr7	99984250	99984520	-0.26	-0.26
Alg8	chr7	104540160	104540578	-1.38	-0.28
Arrb1	chr7	106747059	106749813	-0.34	0.01
Arrb1	chr7	106752442	106752711	-1.98	-0.22
Ctr9	chr7	118197379	118199651	-2.59	0.24
Usp47	chr7	119234949	119245370	-2.45	-0.14
Usp47	chr7	119252192	119254002	-0.85	-0.09
Spon1	chr7	121183472	121183922	-0.88	0.69
Ccp110	chr7	125864970	125865479	-0.53	0.09
Ccp110	chr7	125865867	125868001	-0.53	0.07
Dctn5	chr7	129276554	129277689	-0.53	0.10
Dctn5	chr7	129291791	129292440	-1.28	0.09
Rnf40	chr7	134739594	134740336	-0.78	-0.07
Fbxl19	chr7	134890288	134891744	-2.81	-0.80
Fbxl19	chr7	134911014	134911877	-0.34	-0.02
Sec23ip	chr7	135888443	135894042	-3.19	-0.20
Abraxas2	chr7	140072616	140072677	-0.71	0.38
Abraxas2	chr7	140072975	140075508	-0.35	-0.01
Zranb1	chr7	140122884	140158368	-0.34	-0.01
Zranb1	chr7	140174515	140175634	-0.43	-0.06
Zranb1	chr7	140175516	140176023	-0.87	0.14
Pnpla2	chr7	148641086	148641236	-1.29	-0.28
Pnpla2	chr7	148645626	148646045	-2.50	0.26
Tfpt	chr7	3581338	3581486	-0.93	0.33
Sae1	chr7	16912835	16913105	-2.59	-0.21
Calm3	chr7	17502039	17502309	-1.49	0.09
Apoe	chr7	20282098	20282920	-1.74	0.36
Apoe	chr7	20284425	20284485	-1.41	1.26
Ceacam1	chr7	26247739	26248608	-3.25	0.28
Ceacam1	chr7	26256924	26258919	-0.89	0.24
Ceacam2	chr7	26302704	26302765	-2.15	3.98
Sptbn4	chr7	28149267	28149418	-0.46	1.81

Sptbn4	chr7	28189431	28189552	-0.18	0.68
Psmd8	chr7	29965393	29965573	-1.95	-0.13
Nr1h2	chr7	51806700	51806791	-0.86	0.19
Nucb1	chr7	52758551	52762906	-3.25	-0.31
Nucb1	chr7	52765032	52765778	-1.96	-0.30
Spaca4	chr7	52980506	52980713	-0.34	-0.66
Spaca4	chr7	52981037	52981186	-0.62	0.98
Nsmce3	chr7	72016624	72017837	-3.57	-0.23
Asb7	chr7	73792504	73823980	-0.99	-0.14
Asb7	chr7	73834117	73834387	-0.57	0.21
Mfge8	chr7	86278653	86279192	-0.71	-0.06
Mfge8	chr7	86293825	86293916	-3.60	0.84
Man2a2	chr7	87496117	87497911	-1.70	-0.08
Man2a2	chr7	87515719	87516012	-0.86	-0.26
Blm	chr7	87647232	87647711	-0.92	-0.15
Blm	chr7	87654407	87659523	-2.35	0.28
B3gnt6	chr7	105342961	105343081	-0.22	-1.00
Neu3	chr7	106961618	106962782	-2.63	0.57
Nup98	chr7	109268003	109269291	-1.15	-0.06
Usp17ld	chr7	110398629	110398868	-0.37	0.82
Cog7	chr7	129066679	129066858	-1.01	-0.30
Cog7	chr7	129121227	129125207	-0.66	-0.12
Ino80e	chr7	134005440	134005501	-0.47	0.44
Nsmce4a	chr7	137690685	137690865	-1.03	-0.54
Bet1l	chr7	148039282	148042282	-3.67	-0.23
Trappc5	chr8	3676506	3679785	-3.15	-0.23
Trappc5	chr8	3680083	3680622	-2.24	-0.42
F7	chr8	13034759	13035479	-1.53	-0.88
Proz	chr8	13073522	13073612	-0.41	0.46
Cul4a	chr8	13146533	13146713	-1.15	-0.20
Cul4a	chr8	13146862	13147072	-0.43	-0.06
Thsd1	chr8	23368810	23369377	-1.19	-1.93
Thsd1	chr8	23369584	23370091	-2.31	3.62
Ank1	chr8	24242612	24243062	-0.39	0.32
Cope	chr8	72826683	72826773	-0.78	-0.16
Fkbp8	chr8	73051641	73051859	-0.44	-0.08
Babam1	chr8	73920753	73921677	-1.65	-0.19
Dda1	chr8	73998687	73999406	-1.12	-0.08
Dda1	chr8	73999764	73999855	-0.87	0.21
Rab8a	chr8	74704051	74704703	-0.83	-0.06
Nr3c2	chr8	79432284	79432583	-1.74	2.06
Nr3c2	chr8	79433507	79433836	-1.15	2.46
Cyld	chr8	91220956	91226218	-0.51	0.31

Fbxl8	chr8	107791922	107793078	-0.54	-0.13
Cog4	chr8	113370709	113373788	-1.39	-0.37
Cog4	chr8	113405393	113406134	-0.62	-0.09
St3gal2	chr8	113444003	113491411	-1.01	-0.11
St3gal2	chr8	113493490	113494575	-0.52	0.08
St3gal2	chr8	113494634	113495202	-0.68	0.13
Gan	chr8	119682094	119707502	-2.38	0.66
Usp10	chr8	122460483	122471049	-0.74	0.04
Usp10	chr8	122480587	122481247	-0.85	0.09
Trappc2l	chr8	125135525	125137014	-0.39	-0.08
Tubb3	chr8	125944514	125944901	-0.18	-0.07
Rab4a	chr8	126358896	126359073	-1.59	1.17
Galnt2	chr8	126864828	126868362	-0.38	0.02
Dcun1d2	chr8	13256291	13257907	-0.46	-0.03
Gas6	chr8	13465552	13465851	-0.30	0.01
Vdac3	chr8	23687812	23688050	-3.03	0.38
Pomk	chr8	27091432	27091522	-0.36	-0.49
Pomk	chr8	27092801	27100129	-0.87	-0.09
Dctn6	chr8	35169406	35171565	-0.59	0.15
Ing2	chr8	48753850	48754631	-3.25	-0.30
Galnt7	chr8	60062627	60131852	-1.46	-0.07
Klhl2	chr8	67219421	67219870	-3.53	-0.50
Ano8	chr8	74000216	74000456	-0.50	-0.36
Ano8	chr8	74000514	74000695	-0.32	-0.16
Ano8	chr8	74003128	74005028	-1.87	-0.28
B3gnt3	chr8	74215973	74217291	-0.31	-0.01
B3gnt3	chr8	74217408	74227314	-0.61	-0.20
Rad23a	chr8	87358550	87358761	-1.01	-0.39
Calr	chr8	87366315	87366923	-2.76	-0.06
Calr	chr8	87369960	87370800	-0.94	0.04
Amfr	chr8	96496474	96497162	-1.29	-0.10
Dync1li2	chr8	106944120	106944451	-1.40	-0.19
B3gnt9	chr8	107777610	107779021	-2.43	0.56
Psmb10	chr8	108461631	108461997	-1.25	-0.38
Cog8	chr8	109570548	109573005	-1.46	-0.14
Cog8	chr8	109576126	109580248	-1.04	0.10
Mbtps1	chr8	122032081	122032770	-0.67	0.05
Mbtps1	chr8	122070114	122082661	-3.15	0.21
Fbxo31	chr8	124075878	124076237	-3.34	0.63
Pigyl	chr9	21961289	21962798	-1.13	0.18
Opcml	chr9	28210755	28211775	-0.41	-0.29
Opcml	chr9	28729304	28729904	-0.49	0.42
Opcml	chr9	28730113	28730774	-1.60	0.95

Nfrkb	chr9	31218903	31226648	-1.88	0.21
Usp2	chr9	43883576	43884236	-2.87	2.50
Usp2	chr9	43902032	43902332	-0.41	-0.10
Dpagt1	chr9	44141205	44141504	-0.33	-0.02
Apoa1	chr9	46037892	46038519	-0.60	2.20
Usp28	chr9	48849572	48849783	-2.16	0.69
Alg9	chr9	50651063	50651633	-0.38	-0.03
Psma4	chr9	54805658	54805748	-1.39	0.21
Fbxo22	chr9	55066270	55071672	-2.03	0.17
Fbxo22	chr9	55071880	55072031	-0.93	-0.28
Sin3a	chr9	56973115	56975576	-0.47	0.04
Rora	chr9	69226655	69226835	-0.29	0.06
Adam10	chr9	70526807	70526988	-1.21	0.24
Adam10	chr9	70626056	70626297	-2.69	0.34
Rab27a	chr9	72892701	72908786	-0.51	-0.51
Rab27a	chr9	72943089	72943840	-1.02	-0.28
Cish	chr9	107199256	107202294	-2.04	3.67
Usp4	chr9	108293381	108294561	-0.32	0.00
Usp19	chr9	108393096	108395299	-0.76	-0.07
Cdc25a	chr9	109778082	109778888	-0.45	0.07
Cdc25a	chr9	109794596	109795525	-1.51	0.22
Glb1	chr9	114310242	114326114	-2.08	0.44
Glb1	chr9	114368770	114373569	-0.38	-0.02
Glb1	chr9	114374377	114383495	-3.66	0.26
Dync1li1	chr9	114632656	114632895	-3.05	0.38
Dync1li1	chr9	114632812	114633160	-1.11	0.11
Wdr48	chr9	119833344	119835070	-2.12	0.15
Birc2	chr9	7818584	7818943	-0.91	0.25
Birc2	chr9	7834955	7835255	-3.06	0.75
Birc3	chr9	7848939	7850928	-0.49	0.05
Birc3	chr9	7860311	7872961	-0.93	0.08
Fbxl12	chr9	20448850	20449181	-0.31	-0.05
Dnmt1	chr9	20734577	20736587	-0.40	-0.05
Keap1	chr9	21034503	21038410	-0.91	0.07
Keap1	chr9	21041585	21042276	-1.22	-0.20
Keap1	chr9	21043025	21043776	-0.79	-0.29
Rab3d	chr9	21714741	21715072	-1.15	-0.35
Aplp2	chr9	30957469	30957679	-0.71	-0.07
Aplp2	chr9	30985646	31019400	-0.55	-0.11
Arcn1	chr9	44551783	44551994	-2.61	0.22
Arcn1	chr9	44568237	44575891	-0.66	0.09
Cul5	chr9	53425530	53426100	-1.13	0.12
Rab39	chr9	53494560	53494769	-0.63	1.81

Pml	chr9	58067532	58068881	-1.38	-0.21
Pml	chr9	58077807	58077868	-0.49	0.22
Pml	chr9	58080966	58094982	-3.67	-0.47
Pml	chr9	58097382	58097563	-0.75	-0.30
Senp8	chr9	59584121	59585492	-0.49	0.08
Pias1	chr9	62729583	62729943	-0.64	0.13
Smad3	chr9	63605680	63605801	-0.63	0.71
Smad3	chr9	63605729	63605891	-0.25	0.23
Rab11a	chr9	64585502	64585563	-1.24	0.53
Usp3	chr9	66366103	66367920	-0.95	0.19
Usp3	chr9	66440935	66441025	-0.40	0.79
Usp3	chr9	66441083	66441320	-0.53	0.45
Rab8b	chr9	66715068	66767482	-1.09	0.21
Pigb	chr9	72863505	72865261	-0.80	0.10
Fbxo9	chr9	77943491	77943611	-0.30	0.14
Pgm3	chr9	86447667	86448117	-1.42	-0.31
Dag1	chr9	108166098	108166165	-0.32	0.40
Gorasp1	chr9	119834790	119835059	-2.78	0.60
Sec22c	chr9	121593476	121593866	-0.39	-0.09
Shprh	chr10	10869257	10871973	-1.10	-0.20
Shprh	chr10	10882742	10884564	-0.46	-0.06
Fuca2	chr10	13223007	13225667	-0.83	-0.15
Fuca2	chr10	13234515	13235470	-0.57	-0.03
Nus1	chr10	52137442	52137742	-3.64	-0.33
Nus1	chr10	52153241	52156613	-0.44	0.03
Pofut2	chr10	76731353	76732331	-0.45	0.02
Sumo3	chr10	77068978	77069512	-0.81	0.18
Ube2g2	chr10	77107280	77107637	-0.93	-0.11
Ube2g2	chr10	77107992	77108141	-1.22	0.25
Eef2	chr10	80644273	80645254	-0.46	0.00
Dohh	chr10	80853321	80854179	-2.96	-0.64
Eid3	chr10	82329548	82330052	-0.30	0.00
Eid3	chr10	82330555	82330674	-1.43	0.91
Fbxo7	chr10	85487309	85487369	-0.37	0.10
Galnt4	chr10	98570828	98571068	-2.11	0.75
Galnt4	chr10	98571366	98572174	-0.95	0.19
Galnt4	chr10	98572412	98573250	-1.58	0.31
Cpm	chr10	117120810	117120871	-0.51	1.00
Tnfaip3	chr10	18721581	18725137	-2.68	-0.19
Rnf146	chr10	29064221	29064491	-0.63	0.08
Man1a	chr10	53750519	53795572	-0.83	-0.31
Ube2d1	chr10	70718055	70718592	-2.86	0.34
Trappc10	chr10	77650129	77651540	-0.42	-0.03

Pias4	chr10	80616715	80617300	-0.65	0.09
Pias4	chr10	80619974	80625923	-1.83	-0.26
Pias4	chr10	80626491	80630465	-0.33	-0.01
Ckap4	chr10	83990001	83991399	-2.44	-0.11
Ckap4	chr10	83996216	83996633	-1.45	-0.31
Rab21	chr10	114727724	114727964	-0.78	-0.07
Mdm2	chr10	117126799	117129688	-0.41	0.01
Cand1	chr10	118647900	118648919	-0.55	0.03
Usp15	chr10	122550299	122558549	-0.93	0.08
Os9	chr10	126531314	126532125	-0.37	-0.04
Os9	chr10	126532244	126535544	-0.37	0.01
Os9	chr10	126535886	126536678	-1.25	0.17
Os9	chr10	126557594	126558216	-2.29	-0.30
Mbd6	chr10	126719161	126719461	-1.03	-0.13
Ykt6	chr11	5855760	5859321	-0.36	-0.03
Usp34	chr11	23206894	23233439	-0.62	-0.12
Usp34	chr11	23263833	23264511	-0.87	0.06
Usp34	chr11	23388308	23389213	-0.34	-0.01
Psme4	chr11	30671774	30672135	-0.53	-0.17
Fbxw11	chr11	32644536	32644986	-0.65	0.06
Mgat1	chr11	49057752	49075721	-2.51	-0.17
Galnt10	chr11	57597286	57598644	-0.31	-0.02
Galnt10	chr11	57599150	57599241	-0.83	0.79
Hist3h2a	chr11	58768691	58768899	-0.65	0.26
Nlrp3	chr11	59379889	59380129	-0.34	0.02
Pigl	chr11	62271961	62272172	-1.69	0.58
Trp53	chr11	69393860	69394608	-1.25	-0.24
Ube2g1	chr11	72420792	72421062	-2.86	-0.55
Ube2g1	chr11	72497025	72497295	-0.29	0.00
Pigs	chr11	78141923	78142297	-0.34	-0.05
Pigs	chr11	78155437	78156158	-1.60	0.14
Suz12	chr11	79843530	79845978	-3.16	0.17
Psmd11	chr11	80285574	80285635	-1.26	-0.32
Ptrh2	chr11	86497514	86503722	-3.45	-0.19
Psmd3	chr11	98556993	98557203	-2.02	-0.21
Thra	chr11	98603186	98617477	-0.48	0.09
Thra	chr11	98625080	98626348	-0.46	0.05
Thra	chr11	98626407	98629662	-3.23	0.78
Rara	chr11	98799009	98808938	-0.63	0.23
Rara	chr11	98808997	98822389	-1.01	-0.17
Psme3	chr11	101177564	101178336	-0.77	0.09
Psme3	chr11	101182100	101183258	-2.86	-0.26
Dcaf7	chr11	105898185	105898515	-1.00	0.13

Dcaf7	chr11	105916210	105916330	-1.28	-0.23
Dcaf7	chr11	105919829	105920099	-0.29	0.00
Arsg	chr11	109346860	109378636	-0.29	0.00
Arsg	chr11	109433808	109434167	-0.34	0.02
Cog1	chr11	113517362	113518437	-0.45	-0.04
Engase	chr11	118348441	118349432	-0.62	-0.17
Cbx2	chr11	118884462	118890969	-0.81	0.14
Hgs	chr11	120344969	120345268	-1.28	-0.22
Gps1	chr11	120648738	120649262	-0.86	-0.36
Gps1	chr11	120649939	120650416	-1.35	-0.22
Foxk2	chr11	121161028	121168992	-2.31	-0.14
Fn3k	chr11	121303052	121303292	-0.44	-2.25
Fn3k	chr11	121303589	121303799	-0.35	-1.32
Commd1	chr11	22799727	22800056	-0.70	0.07
Commd1	chr11	22856647	22878367	-0.34	0.03
Commd1	chr11	22878962	22882284	-1.57	-0.40
Pex13	chr11	23548957	23549226	-1.26	0.32
Pex13	chr11	23551042	23555931	-0.71	0.12
Sptbn1	chr11	30017714	30018324	-0.65	0.10
Sptbn1	chr11	30036124	30038367	-0.57	0.04
Sptbn1	chr11	30038786	30040543	-1.61	-0.15
Sptbn1	chr11	30119730	30168025	-0.37	0.07
Stc2	chr11	31259979	31260460	-1.02	-0.51
0610009B22Rik	chr11	51499092	51499534	-0.38	0.03
Sec24a	chr11	51556955	51570200	-1.50	-0.11
Derl2	chr11	70823390	70826702	-1.47	-0.13
Derl2	chr11	70832716	70832777	-0.27	0.01
Hic1	chr11	74978874	74979025	-0.69	1.22
Hic1	chr11	74979084	74979444	-0.39	-0.83
Hic1	chr11	74980641	74980732	-0.16	0.49
Rpa1	chr11	75153888	75161885	-1.45	-0.24
Gosr1	chr11	76543144	76550896	-0.53	-0.05
Wsb1	chr11	79053599	79053898	-2.67	-0.17
Pex12	chr11	83111041	83111132	-0.98	-0.53
Pex12	chr11	83111280	83111913	-1.08	0.33
Pigw	chr11	84691181	84691984	-1.18	-0.17
Ube2z	chr11	95911464	95911704	-2.06	-0.26
Pcgf2	chr11	97551180	97551629	-0.96	-0.18
Pcgf2	chr11	97553081	97553393	-0.41	0.07
Fbxl20	chr11	97944196	97944257	-0.53	0.44
Klhl11	chr11	100323925	100325737	-2.89	0.19
Klhl11	chr11	100333857	100334096	-0.43	-0.10
Kat2a	chr11	100573480	100573601	-0.54	-0.16

Rab5c	chr11	100581475	100599444	-1.66	-0.43
Becn1	chr11	101163065	101163581	-0.94	-0.14
Brca1	chr11	101384754	101385384	-1.00	0.21
Brca1	chr11	101385443	101386611	-0.56	0.06
Brca1	chr11	101386909	101387689	-2.16	0.30
Nsf	chr11	103684322	103685071	-0.70	0.10
Nsf	chr11	103792113	103815310	-0.32	0.08
Smurf2	chr11	106683743	106684358	-1.06	-0.24
Smurf2	chr11	106781579	106781849	-2.81	-0.99
Socs3	chr11	117830559	117830620	-0.38	0.48
Cbx4	chr11	118941759	118947461	-1.94	0.14
P4hb	chr11	120422393	120423503	-0.94	-0.04
Csnk1d	chr11	120819778	120820048	-2.61	-0.30
Csnk1d	chr11	120823779	120824109	-0.34	0.01
Csnk1d	chr11	120824767	120826288	-1.85	-0.13
Csnk1d	chr11	120852257	120852467	-0.47	-0.06
B3gntl1	chr11	121478301	121480951	-0.40	-0.03
Asxl2	chr12	3426883	3442534	-0.44	0.04
Asxl2	chr12	3496366	3497026	-3.15	0.17
Asxl2	chr12	3497115	3502535	-2.99	0.09
Smc6	chr12	11292342	11292522	-2.81	0.91
Smc6	chr12	11324583	11325004	-0.43	0.03
Cog5	chr12	32339733	32339824	-0.31	0.05
Psma6	chr12	56499811	56499900	-1.08	-0.18
Mia2	chr12	60208992	60209821	-1.76	0.98
Lrr1	chr12	70275442	70276002	-1.46	-0.50
Mgat2	chr12	70285182	70287153	-1.15	-0.05
Mgat2	chr12	70285144	70287136	-1.06	-0.04
Hif1a	chr12	75043067	75043308	-3.26	-0.17
Fut8	chr12	78432987	78466016	-0.68	-0.18
Fut8	chr12	78576163	78576944	-2.51	0.44
Dcaf4	chr12	84882463	84882673	-0.76	0.30
Ttll5	chr12	87165652	87165773	-0.27	-0.03
Ttll5	chr12	87259905	87263926	-0.29	-0.01
Psmc1	chr12	101348377	101351316	-0.70	-0.11
Otub2	chr12	104641523	104643067	-0.48	0.06
Yy1	chr12	110031520	110044712	-3.35	-0.22
Dync1h1	chr12	111839634	111839815	-1.16	-0.25
Dync1h1	chr12	111855934	111857506	-0.62	-0.02
Dync1h1	chr12	111904592	111905154	-2.05	0.14
Wdr20	chr12	112017684	112032696	-0.40	0.02
Traf3	chr12	112499706	112500694	-0.68	-0.07
Ncoa1	chr12	4247570	4248468	-0.70	-0.10

Ncoa1	chr12	4248766	4252834	-0.39	-0.03
Ncoa1	chr12	4267709	4267949	-1.13	0.26
Ncoa1	chr12	4294980	4296117	-2.77	0.24
Trappc6b	chr12	60162299	60162448	-1.32	-0.37
Sptb	chr12	77714000	77714181	-0.13	-0.12
Rab15	chr12	77899366	77899456	-0.30	0.81
Pigh	chr12	80183212	80183482	-3.23	-0.81
Pigh	chr12	80190135	80190373	-2.29	1.30
Dcaf5	chr12	81437908	81438298	-0.47	-0.08
Dcaf5	chr12	81439313	81449328	-1.43	-0.10
Dcaf5	chr12	81537288	81537379	-1.00	0.54
Tmed10	chr12	86683962	86685208	-0.48	0.02
Tmed10	chr12	86715456	86715667	-0.68	-0.06
Pomt2	chr12	88449791	88451254	-1.85	-0.29
Pomt2	chr12	88488462	88488612	-0.26	-0.11
Sel1l	chr12	93046846	93048492	-0.47	-0.01
Sel1l	chr12	93080231	93087597	-2.13	-0.33
Atxn3	chr12	103157140	103157589	-0.64	-0.11
Ankrd9	chr12	112214221	112214281	-0.40	-2.32
Ankrd9	chr12	112214550	112215299	-1.40	-0.62
Ankrd9	chr12	112215597	112215718	-0.58	0.58
B3galnt2	chr13	14090802	14091131	-3.29	-0.44
Trim27	chr13	21283975	21284366	-3.06	-0.20
Hist1h2bg	chr13	23663268	23663474	-3.01	-0.19
Hist1h2bc	chr13	23776067	23776213	-0.42	-0.04
Gpld1	chr13	25078887	25082252	-0.77	-0.16
Ripk1	chr13	34119764	34125274	-2.04	-0.13
Ripk1	chr13	34125393	34126112	-1.53	-0.25
Arsb	chr13	94710635	94710756	-0.54	-0.12
Ercc8	chr13	108948961	108949051	-1.87	-0.97
Gmds	chr13	31911574	31911785	-1.42	-0.36
Tubb2b	chr13	34219319	34219498	-1.13	2.05
Tubb2b	chr13	34220029	34220783	-1.25	-0.78
Nup153	chr13	46776258	46776499	-0.69	-0.10
Nup153	chr13	46782010	46792068	-2.74	0.10
Uimc1	chr13	55129866	55132327	-0.74	0.14
Uimc1	chr13	55176900	55177348	-1.67	-0.22
Rab24	chr13	55421264	55421501	-2.29	-0.29
Lman2	chr13	55447467	55447708	-1.84	-0.28
Lman2	chr13	55448036	55448336	-2.00	-0.16
Hnrnpk	chr13	58501349	58504674	-2.32	-0.21
Golm1	chr13	59736356	59736923	-0.80	-0.12
Golm1	chr13	59738410	59741528	-0.61	-0.10

Kctd6	chr14	9052713	9055605	-1.86	-0.26
Atxn7	chr14	14932473	14936644	-2.02	-0.16
Sec24c	chr14	21512398	21513205	-0.75	-0.07
Sec24c	chr14	21513563	21514072	-1.48	-0.13
Actr8	chr14	30804011	30804814	-1.45	-0.43
Bap1	chr14	32070621	32070742	-0.87	0.20
Bap1	chr14	32072009	32072637	-2.29	-0.24
Psmc6	chr14	45949498	45950514	-0.58	0.08
Dcaf11	chr14	56178865	56178955	-0.66	-0.41
Dcaf11	chr14	56188485	56188902	-1.96	-0.28
Kbtbd7	chr14	79827126	79828716	-1.70	0.29
Ankrd28	chr14	32514639	32518497	-1.72	-0.14
Dph3	chr14	32893866	32894315	-2.21	0.62
Nedd8	chr14	56290685	56290743	-0.55	-0.12
Rabggta	chr14	56340490	56340807	-2.27	0.59
Cog3	chr14	76102935	76102996	-0.58	0.26
Cog3	chr14	76103085	76106953	-2.15	-0.24
Commd6	chr14	102033311	102039300	-0.34	0.01
Fbxl3	chr14	103482090	103488631	-0.81	-0.05
Fbxl3	chr14	103494436	103498143	-0.54	-0.05
Fbxl3	chr14	103498202	103498726	-1.82	-0.43
Sema5a	chr15	32616573	32620160	-0.68	-0.09
Dcaf13	chr15	38944419	38950257	-0.48	0.07
Rnf139	chr15	58732325	58732416	-1.18	0.88
Ly6e	chr15	74786018	74786078	-1.11	0.25
Commd5	chr15	76730370	76731727	-1.84	-0.21
Apol9b	chr15	77566398	77566605	-1.11	2.04
Mgat3	chr15	80042031	80042182	-0.17	0.59
Rbx1	chr15	81305941	81306090	-0.83	-0.28
Ep300	chr15	81416643	81438767	-1.05	0.06
Ep300	chr15	81479273	81481819	-0.51	0.02
L3mbtl2	chr15	81516741	81518556	-1.73	-0.15
Nup50	chr15	84757805	84765852	-1.08	-0.07
Nup50	chr15	84773033	84773303	-0.62	0.12
Tuba1c	chr15	98867757	98867967	-0.36	0.01
Skp2	chr15	9043329	9043540	-0.80	0.27
Skp2	chr15	9069207	9070167	-0.68	-0.14
Rad21	chr15	51794834	51794954	-0.36	-0.02
Rad21	chr15	51798246	51799392	-0.87	-0.13
Fbxo32	chr15	58011872	58012083	-0.36	-0.16
Fbxo32	chr15	58012410	58013069	-1.46	-0.50
Fbxo32	chr15	58046223	58046433	-2.16	-1.12
St3gal1	chr15	66936954	66938364	-1.56	-0.14

St3gal1	chr15	66944037	67005237	-0.40	-0.02
Apol9a	chr15	77234337	77234723	-0.99	-0.99
Apol9a	chr15	77234811	77235109	-0.44	-0.50
Apol10b	chr15	77415802	77416129	-0.27	1.22
Apol8	chr15	77579072	77579283	-1.84	3.24
Ddx17	chr15	79359562	79359623	-3.23	-0.45
Ddx17	chr15	79376901	79377021	-1.72	-0.27
Josd1	chr15	79506286	79506585	-0.42	-0.06
Josd1	chr15	79517855	79518272	-1.01	0.24
Rangap1	chr15	81535546	81535636	-0.97	-0.19
Rangap1	chr15	81559691	81560349	-0.88	-0.22
Pmm1	chr15	81781535	81781891	-2.07	0.31
Ttll12	chr15	83406959	83407229	-1.63	-0.34
Alg12	chr15	88635911	88636421	-0.42	-0.05
Arsa	chr15	89303025	89303891	-1.14	0.17
Arsa	chr15	89305958	89306615	-3.10	-0.44
Vdr	chr15	97687093	97687333	-0.24	0.68
Senp1	chr15	97872854	97873303	-0.94	-0.13
Asb8	chr15	97965939	97971774	-0.39	0.02
Galnt6	chr15	100522228	100522647	-0.52	-0.09
Galnt6	chr15	100544605	100550892	-0.77	-0.08
Alg1	chr16	5244791	5245001	-0.28	0.00
Klhl22	chr16	17771956	17777358	-1.71	-0.20
Klhl22	chr16	17778345	17778466	-0.64	0.80
Psmd2	chr16	20651724	20652324	-3.25	-0.17
Senp2	chr16	22035915	22036096	-0.98	-0.61
Senp2	chr16	22046889	22047129	-1.33	-0.22
St6gal1	chr16	23321194	23321733	-0.65	0.14
Pigz	chr16	31944722	31944783	-0.24	-1.22
Rnf168	chr16	32277636	32282431	-0.66	0.10
Rnf168	chr16	32298648	32299428	-1.18	0.18
Golgb1	chr16	36914905	36915206	-2.70	0.34
Golgb1	chr16	36932031	36933171	-0.77	-0.08
Tomm70a	chr16	57152724	57153054	-1.60	-0.13
Usp25	chr16	77081892	77084046	-0.60	0.12
Usp25	chr16	77115246	77116156	-2.54	0.20
Usp16	chr16	87477080	87480005	-2.02	-0.15
Eef2kmt	chr16	5244783	5245380	-1.09	0.16
Senp5	chr16	31963352	31965869	-0.38	-0.03
Senp5	chr16	31989077	31990547	-0.59	0.03
Sec22a	chr16	35313599	35318950	-0.91	-0.16
Nrip1	chr16	76291196	76295213	-2.02	0.11
Nrip1	chr16	76352759	76374042	-1.51	-0.61

App	chr16	85025115	85043863	-0.75	-0.03
App	chr16	85043922	85056713	-2.06	-0.14
App	chr16	85120877	85173892	-2.41	-0.23
Tulp4	chr17	6232220	6232851	-2.79	-0.26
Tulp4	chr17	6232910	6233480	-1.17	-0.24
Tulp4	chr17	6234139	6236560	-2.06	0.41
Spsb3	chr17	25023707	25024470	-2.46	-0.59
Spsb3	chr17	25024809	25029092	-1.63	-0.14
Axin1	chr17	26275840	26310679	-1.19	0.10
Axin1	chr17	26319507	26325117	-3.16	-0.30
Axin1	chr17	26330938	26332096	-0.33	-0.02
Adamts10	chr17	33665942	33666153	-0.99	-0.46
Adamts10	chr17	33667079	33667439	-1.19	-0.43
Adamts10	chr17	33690397	33690697	-3.34	-0.81
Psmb8	chr17	34335288	34336214	-1.01	-0.16
Neu1	chr17	35071427	35072869	-3.32	-0.17
Mdc1	chr17	35983321	35985582	-1.83	-0.14
Mdc1	chr17	35987436	35987527	-3.30	0.71
Mdc1	chr17	35987825	35988082	-1.08	0.26
Mdc1	chr17	35989399	35989938	-0.34	0.01
Mdc1	chr17	35994492	35994583	-0.46	0.09
Cul7	chr17	46787586	46787707	-0.19	0.03
Cul7	chr17	46788456	46789468	-0.30	-0.09
Rab5a	chr17	53618648	53623279	-1.12	0.12
Rab5a	chr17	53639752	53646076	-0.68	-0.06
Kat2b	chr17	53706385	53706446	-0.22	0.15
Fem1a	chr17	56400758	56401238	-0.39	0.05
Fem1a	chr17	56401745	56402702	-0.37	0.03
Safb	chr17	56724404	56724555	-1.20	0.29
Safb	chr17	56733308	56735128	-1.28	-0.14
Safb	chr17	56737716	56737897	-1.70	0.51
Safb	chr17	56738105	56738676	-2.46	-0.21
Man2a1	chr17	64950988	64974652	-0.82	-0.11
Socs5	chr17	87507287	87507348	-0.71	-0.42
Socs5	chr17	87520106	87534893	-3.68	0.22
Ccnf	chr17	24360773	24360864	-1.19	-0.44
Ube2i	chr17	25397995	25398776	-0.81	-0.11
Rab40c	chr17	26020165	26020794	-1.48	0.23
Pigq	chr17	26064506	26064597	-0.31	-0.01
Pigq	chr17	26073565	26073656	-0.30	0.10
Pigq	chr17	26073775	26078847	-1.47	-0.13
Rab11b	chr17	33883909	33884149	-2.85	-0.29
Rab11b	chr17	33884297	33884597	-1.25	0.23

Rab11b	chr17	33897340	33897431	-1.34	0.73
Ring1	chr17	34158155	34160310	-1.41	-0.14
Ring1	chr17	34160369	34161265	-0.31	-0.04
Ly6g6d	chr17	35208292	35208667	-1.07	2.51
Cul9	chr17	46680184	46683217	-0.34	0.04
Rab31	chr17	66001065	66001632	-0.47	0.02
Rab31	chr17	66003418	66017287	-0.77	0.04
Rab31	chr17	66122031	66122092	-0.23	-0.05
Rab12	chr17	66844211	66844991	-0.97	0.09
Pigf	chr17	87396598	87396896	-3.10	0.22
Mcfd2	chr17	87654440	87654561	-0.80	0.23
Mcfd2	chr17	87654739	87656518	-1.65	0.15
Calm2	chr17	87835011	87846216	-3.10	-0.20
Fbxo11	chr17	88390945	88391656	-0.52	0.03
Wac	chr18	7869438	7869558	-1.82	-0.66
Wac	chr18	7905960	7910820	-1.76	0.12
Galnt1	chr18	24438485	24443794	-2.19	-0.18
Apc	chr18	34455759	34458292	-1.70	0.44
Ube2d2a	chr18	35931242	35931392	-1.92	-0.45
Ube2d2a	chr18	35965360	35965570	-1.78	0.47
Commd10	chr18	47246724	47246904	-0.46	0.06
Dctn4	chr18	60686409	60686590	-0.35	0.21
Dctn4	chr18	60716345	60716496	-0.75	-0.18
Napg	chr18	63154672	63156813	-3.08	-0.29
Tubb6	chr18	67550384	67552330	-3.31	-0.30
Mbd1	chr18	74437069	74438854	-1.40	-0.21
Mbd1	chr18	74439929	74440708	-2.55	-0.31
Mbd1	chr18	74441560	74442218	-3.23	-0.32
Smad7	chr18	75527018	75527734	-0.56	0.14
Smad7	chr18	75528210	75530550	-2.26	0.57
Smad2	chr18	76422187	76422338	-0.39	0.04
Smad2	chr18	76462139	76464727	-0.86	-0.12
Pias2	chr18	77304096	77304536	-2.25	0.61
Pias2	chr18	77304625	77336416	-0.40	-0.02
Pias2	chr18	77388818	77391933	-0.34	0.01
Usp14	chr18	10030026	10030087	-1.17	-0.30
B4galt6	chr18	20846332	20847761	-1.60	-0.24
Ino80c	chr18	24263918	24264068	-1.24	-0.33
Ino80c	chr18	24264545	24270229	-0.93	0.13
Nr3c1	chr18	39588392	39649457	-3.45	0.21
Fem1c	chr18	46664557	46685565	-0.41	-0.01
Tmed7	chr18	46747763	46748242	-0.50	-0.03
Lman1	chr18	66140572	66140992	-1.89	0.18

Lman1	chr18	66151175	66151326	-1.49	-0.18
Lman1	chr18	66162078	66162289	-0.76	0.11
Smad4	chr18	73799858	73800008	-0.66	-0.12
Smad4	chr18	73863185	73863395	-3.16	-1.68
Socs6	chr18	89038427	89056448	-0.39	-0.02
Sptbn2	chr19	4744084	4745130	-1.43	-0.66
B4gat1	chr19	5041278	5046482	-2.34	-0.20
Rela	chr19	5637578	5637639	-0.26	0.03
Men1	chr19	6335008	6336781	-2.54	-0.27
Men1	chr19	6338873	6340864	-1.37	-0.10
Stx5a	chr19	8829416	8830132	-0.49	0.04
Ubxn1	chr19	8945985	8946226	-0.36	0.03
Uhrf2	chr19	30105002	30105212	-1.71	-0.42
Uhrf2	chr19	30113733	30131060	-3.08	0.29
Stambpl1	chr19	34266759	34267030	-1.66	-0.25
Stambpl1	chr19	34307271	34308667	-1.01	0.18
Btrc	chr19	45604207	45604268	-0.27	-0.42
Gbf1	chr19	46227047	46227258	-0.79	0.19
Gbf1	chr19	46360279	46360430	-0.70	0.15
Nfkb2	chr19	46379346	46380320	-1.80	0.46
Fbxl15	chr19	46403619	46404936	-0.68	0.11
1700020D05Rik	chr19	5502888	5503128	-0.65	0.38
1700020D05Rik	chr19	5503217	5503398	-0.29	0.08
1700020D05Rik	chr19	5503487	5503787	-0.38	0.25
Incenp	chr19	9947145	9947295	-0.39	0.02
Incenp	chr19	9949324	9952191	-1.89	0.14
Incenp	chr19	9958207	9968481	-3.67	-0.11
Incenp	chr19	9969946	9974023	-0.62	0.11
Gcnt1	chr19	17403142	17407708	-3.03	0.20
Smc5	chr19	23348276	23348367	-0.83	-0.31
Fbxw4	chr19	45652836	45653256	-0.43	-0.03
Actr1a	chr19	46452405	46452913	-1.68	-0.11
Usp11	chrX	20281154	20281215	-0.29	-0.12
Ube2a	chrX	34414360	34414479	-0.33	-0.03
Ikbkg	chrX	71675074	71678148	-0.70	-0.30
Ogt	chrX	98874640	98878285	-0.71	0.04
Ogt	chrX	98878792	98878973	-1.90	-0.42
Ogt	chrX	98879151	98879242	-0.52	-0.19
Piga	chrX	160866941	160869822	-1.00	0.37
Piga	chrX	160870267	160870507	-2.83	1.12
Cfp	chrX	20508559	20508650	-1.66	0.59
C1galt1c1	chrX	35984136	35988290	-0.62	-0.06
Hcfc1	chrX	71194680	71196000	-1.71	-0.08

Hcfc1	chrX	71211533	71211654	-0.84	0.13
Napb	chr2	148538436	148558156	-2.45	0.80
Tnks	chr8	36028504	36028744	-1.36	-0.36
Dynll2	chr11	87794645	87799923	-0.49	0.03

Adaptive immune system

gene_name	chr	chromStart	chromEnd	diff.lg.p	diff.log2.fc
Cd28	chr1	60826544	60826935	-0.44	-0.25
Cd28	chr1	60827743	60828044	-0.54	0.44
Psmd1	chr1	87961193	87961313	-0.67	0.21
Inpp5d	chr1	89516886	89517096	-2.34	-0.38
Inpp5d	chr1	89614406	89616844	-1.74	-0.09
Ube2f	chr1	93182063	93182512	-2.14	-0.16
Asb1	chr1	93437351	93443741	-2.00	0.50
Asb1	chr1	93448832	93448983	-0.89	0.38
Asb1	chr1	93451373	93451764	-0.41	0.07
Ncf2	chr1	154654924	154658055	-0.49	0.03
Cd247	chr1	167791873	167791964	-0.45	2.42
Hecw2	chr1	53869734	53883756	-1.12	2.77
Hecw2	chr1	53982435	53983513	-0.26	-0.03
Tuba4a	chr1	75211756	75211936	-0.82	-0.43
Cul3	chr1	80336885	80337005	-1.62	-0.38
Trip12	chr1	84721087	84721998	-1.08	0.10
Trip12	chr1	84782695	84836815	-1.84	0.07
Pdcd1	chr1	95937963	95949130	-1.13	-1.44
Klhl20	chr1	163019047	163019258	-1.39	-0.43
Klhl20	chr1	163039327	163060580	-0.32	0.01
Ppp2r5a	chr1	193176577	193176817	-2.17	0.22
Prkcq	chr2	11221863	11221954	-0.17	-0.59
Anapc2	chr2	25128463	25128941	-0.88	-0.09
Fbxw5	chr2	25358791	25359805	-1.19	0.14
Fbxw5	chr2	25360013	25360452	-1.24	-0.22
Fbxw5	chr2	25360840	25360960	-3.19	1.04
Mapkap1	chr2	34479093	34479423	-1.30	0.22
Hspa5	chr2	34630750	34631930	-2.17	-0.03
Klhl41	chr2	69508654	69508893	-1.32	0.66
Dync1i2	chr2	71049792	71049883	-0.39	0.11
Itgav	chr2	83643447	83643658	-1.94	-0.36
Itgav	chr2	83643837	83644077	-1.39	0.28
Psmc3	chr2	90894439	90895182	-1.38	-0.10
Traf6	chr2	101531587	101537929	-3.55	-0.22
Kif18a	chr2	109173461	109173702	-2.70	0.61
B2m	chr2	121973422	121973632	-1.88	-0.08
Trim69	chr2	121993421	121993481	-0.14	0.42

Trim69	chr2	122004286	122004614	-0.57	-1.61
Kif3b	chr2	153141953	153146041	-2.25	0.20
Kif3b	chr2	153155920	153156639	-0.36	0.02
Kif3b	chr2	153158285	153158915	-0.93	0.19
Itch	chr2	154959216	154959307	-0.25	0.00
Itch	chr2	155050132	155050373	-0.80	-0.16
Src	chr2	157295226	157296471	-0.85	0.32
Plcg1	chr2	160587522	160588233	-1.55	-0.33
Cd40	chr2	164896297	164896475	-0.35	0.15
Cd40	chr2	164896740	164896829	-0.20	0.00
Rnf114	chr2	167318293	167325329	-0.28	-0.05
Tubb4b	chr2	25077884	25079546	-0.33	0.00
Tubb4b	chr2	25079713	25080192	-0.31	-0.02
Lrsam1	chr2	32781993	32782383	-0.65	-0.16
Fbxw2	chr2	34661048	34661869	-2.12	-0.20
Fbxw2	chr2	34662764	34664040	-0.46	-0.08
Fbxw2	chr2	34668436	34678672	-1.12	-0.10
Fbxw2	chr2	34681468	34681725	-0.58	0.19
Psmd5	chr2	34707993	34709794	-1.37	-0.17
Psmd5	chr2	34722532	34726482	-1.60	-0.40
Psmb7	chr2	38498946	38499426	-1.35	-0.19
Ptprj	chr2	90273202	90274160	-3.35	-0.26
Ptprj	chr2	90420474	90420774	-0.84	-0.21
Ubox5	chr2	130416966	130417566	-1.43	-0.36
Ubox5	chr2	130425325	130426405	-0.55	0.08
Siglec1	chr2	130895194	130895494	-1.06	0.66
Siglec1	chr2	130900572	130901960	-0.37	0.08
Siglec1	chr2	130903894	130904407	-0.55	0.28
Siglec1	chr2	130906334	130909140	-0.49	0.17
Siglec1	chr2	130911282	130911343	-0.24	-0.08
Siglec1	chr2	130911962	130912441	-2.24	1.31
Psmf1	chr2	151543111	151543232	-0.83	-0.47
Psmf1	chr2	151543649	151544068	-0.81	-0.19
Trib3	chr2	152169646	152169796	-3.17	-0.77
Nfatc2	chr2	168301939	168303199	-2.11	-0.21
Nfatc2	chr2	168304128	168330396	-0.75	0.06
Nfatc2	chr2	168396287	168427005	-2.25	-0.19
Pik3ca	chr3	32335072	32335402	-0.84	0.17
Pik3ca	chr3	32360681	32362022	-0.52	0.05
Ctso	chr3	81736537	81736628	-0.27	0.06
Ctso	chr3	81756174	81758915	-1.63	0.24
Fbxw7	chr3	84619498	84694070	-1.92	0.38
Fbxw7	chr3	84694129	84756226	-2.05	0.20

Fbxw7	chr3	84780140	84781955	-0.37	0.02
Ube2q1	chr3	89577530	89577890	-3.20	-0.36
Ube2q1	chr3	89585705	89587919	-0.89	-0.04
Them4	chr3	94135467	94135976	-0.96	0.20
Ctss	chr3	95330707	95333452	-1.18	-0.07
Rnf115	chr3	96592580	96593978	-1.30	-0.15
Sec22b	chr3	97725140	97725438	-2.99	-0.26
Nras	chr3	102868375	102868495	-0.73	-0.10
Cenpe	chr3	134910323	134910564	-0.59	-0.09
Cenpe	chr3	134933684	134933745	-0.39	0.11
Cenpe	chr3	134936264	134936504	-0.40	0.06
Ube2d3	chr3	135101656	135102228	-3.24	-0.27
Bcl10	chr3	145587232	145587592	-0.60	0.07
Bcl10	chr3	145588099	145593615	-2.28	0.24
Pag1	chr3	9689310	9689401	-0.90	-0.46
Pag1	chr3	9690420	9691081	-1.53	-0.30
Siah2	chr3	58495620	58495891	-1.37	0.55
Psmd4	chr3	94836626	94836924	-1.79	-0.12
Psmd4	chr3	94839816	94846467	-2.16	-0.13
Cd101	chr3	100797451	100797692	-0.26	-0.05
Sec24b	chr3	129686102	129687101	-0.83	-0.07
Nfkb1	chr3	135248217	135252299	-2.46	-0.20
Nfkb1	chr3	135354240	135354361	-0.32	0.21
Dapp1	chr3	137595527	137598550	-2.31	-0.57
Prkacb	chr3	146394723	146394993	-1.39	-0.21
Lyn	chr4	3605267	3605506	-3.28	-0.20
Fbxl4	chr4	22303713	22313319	-1.70	0.24
Fbxl4	chr4	22354425	22354486	-0.94	0.57
Map3k7	chr4	32051081	32051232	-2.61	-1.03
Map3k7	chr4	32109961	32110261	-0.43	0.08
Ube2j1	chr4	33136832	33136892	-1.32	-0.32
Ube2r2	chr4	41137777	41138136	-1.17	0.13
Trim32	chr4	65274645	65276139	-0.35	0.01
Lrrc41	chr4	115761466	115762183	-1.09	-0.08
Lrrc41	chr4	115768961	115769260	-0.52	0.05
Capzb	chr4	138748845	138794946	-0.34	-0.01
Fbxo2	chr4	147538344	147538434	-0.25	-0.78
Mtor	chr4	147822690	147826440	-1.12	-0.33
Mtor	chr4	147930392	147931764	-0.80	0.10
Klhl21	chr4	151383089	151389959	-0.56	0.05
Klhl21	chr4	151390287	151390408	-0.53	0.22
Ripk2	chr4	16050729	16051621	-2.11	-0.28
Ufl1	chr4	25177588	25177709	-0.34	0.05

Fbxo10	chr4	45048105	45048374	-0.47	0.54
Fbxo10	chr4	45048433	45048494	-0.73	1.50
Fbxo10	chr4	45048553	45048793	-0.74	0.68
Fbxo10	chr4	45071323	45071922	-0.48	0.30
Cdc26	chr4	62055609	62055998	-2.00	0.59
Kif2c	chr4	116832539	116832928	-1.47	-0.33
Rnf220	chr4	116958526	116968779	-3.32	0.35
Cdc20	chr4	118109887	118109948	-0.55	-0.37
Lck	chr4	129225797	129226067	-0.39	0.10
Fbxo6	chr4	147520063	147520213	-1.03	0.25
Pik3cd	chr4	149023545	149025880	-2.62	-0.17
Spsb1	chr4	149271827	149281337	-1.02	0.57
Mib2	chr4	155028580	155028761	-0.30	-0.09
Ube3c	chr5	29895781	29895962	-1.72	-1.14
Ube3c	chr5	30001057	30001268	-0.46	0.07
Anapc4	chr5	53257704	53257973	-2.14	-0.29
Klhl5	chr5	65558211	65558721	-1.61	0.23
Ube3b	chr5	114830734	114830795	-0.28	0.61
Ube3b	chr5	114869593	114869952	-0.38	0.03
Fbxo21	chr5	118426838	118426928	-0.57	0.34
Fbxo21	chr5	118452063	118458648	-0.32	0.01
Anapc7	chr5	122891328	122891538	-0.52	-0.09
Anapc7	chr5	122893875	122894502	-3.47	-0.38
Rnf34	chr5	123273869	123314504	-1.72	0.21
Psmd9	chr5	123678228	123684616	-1.07	0.18
Kctd7	chr5	130628247	130628815	-1.33	-0.62
Fbxl5	chr5	44142150	44149098	-2.30	-0.48
Fbxl5	chr5	44149396	44151973	-0.42	-0.02
Rchy1	chr5	92391795	92392064	-0.36	0.05
Sec31a	chr5	100790877	100791058	-0.38	0.02
Fbxw8	chr5	118515497	118515797	-0.35	-0.06
Ptpn11	chr5	121581827	121582307	-2.13	0.25
Ptpn11	chr5	121583292	121587519	-0.51	-0.03
Ncf1	chr5	134705434	134705495	-1.15	-0.28
Pilra	chr5	138263299	138263390	-0.40	1.81
Pilra	chr5	138264796	138272396	-1.16	1.30
Pilra	chr5	138276577	138276758	-2.35	1.83
Pilrb2	chr5	138312135	138312373	-0.55	2.87
Card11	chr5	141348952	141350390	-3.03	-0.50
Fbxl18	chr5	143633766	143634186	-0.39	0.07
Fbxl18	chr5	143640382	143644224	-0.93	-0.38
Fbxl18	chr5	143650503	143656917	-0.38	-0.27
Smurf1	chr5	145637693	145638023	-0.74	-0.29

Smurf1	chr5	145638352	145638682	-1.09	-0.34
Smurf1	chr5	145639310	145639491	-0.37	0.13
Rnf6	chr5	147021822	147022181	-2.92	0.33
Znrf2	chr6	54838508	54838629	-0.37	-0.02
Herc6	chr6	57445639	57531182	-0.37	-0.17
Dctn1	chr6	83140226	83141067	-0.66	-0.06
Kbtbd8	chr6	95071961	95077327	-2.16	0.32
Vhl	chr6	113578147	113579978	-1.16	0.21
Fbxl14	chr6	119429685	119431451	-2.73	-0.15
Klhl42	chr6	147039984	147056970	-0.73	-0.10
Ube2h	chr6	30163827	30164395	-0.64	0.08
Ube2h	chr6	30164514	30164664	-1.04	0.40
Ube2h	chr6	30164783	30164993	-0.55	0.22
Vamp8	chr6	72335214	72335506	-0.31	0.00
Vamp8	chr6	72338374	72340661	-0.33	-0.02
Raf1	chr6	115568620	115569280	-2.59	-0.22
Ptpn6	chr6	124683075	124688519	-0.61	-0.20
Kras	chr6	145168783	145169023	-0.29	0.00
Itpr2	chr6	146060001	146060981	-1.65	0.22
Ap2s1	chr7	17323792	17328621	-1.81	-0.21
Akt2	chr7	28422202	28423428	-1.06	0.15
Herc2	chr7	63485102	63486928	-1.77	0.13
Ube3a	chr7	66484119	66496353	-2.58	0.28
Klhl25	chr7	82993342	83012197	-1.00	0.11
Klhl25	chr7	83012375	83018299	-0.87	-0.23
Klhl25	chr7	83018716	83018926	-0.35	-0.11
Ctsc	chr7	95437215	95437515	-0.81	0.31
Ctsc	chr7	95458037	95458487	-2.34	0.25
Dctn5	chr7	129276554	129277689	-0.53	0.10
Dctn5	chr7	129291791	129292440	-1.28	0.09
Rbbp6	chr7	130114077	130114438	-1.49	-0.14
Rbbp6	chr7	130133665	130133786	-0.82	-0.15
Rbbp6	chr7	130140585	130140795	-0.65	0.08
Fbxl19	chr7	134890288	134891744	-2.81	-0.80
Fbxl19	chr7	134911014	134911877	-0.34	-0.02
Stx4a	chr7	134985321	134985978	-0.91	-0.11
Ap2a2	chr7	148748108	148748169	-1.07	-0.80
Lair1	chr7	3960490	3960699	-1.39	1.24
Lair1	chr7	3961115	3961205	-1.34	-1.04
Calm3	chr7	17502039	17502309	-1.49	0.09
Klc3	chr7	19979788	19981213	-1.51	-0.23
Nectin2	chr7	20302441	20302980	-0.95	0.42
Nectin2	chr7	20306842	20310128	-0.64	-0.15

Nectin2	chr7	20334652	20334892	-0.56	0.22
Pvr	chr7	20490268	20494550	-0.49	0.06
Psmd8	chr7	29965393	29965573	-1.95	-0.13
Cd22	chr7	31662583	31662704	-0.12	0.00
Siglece	chr7	50914788	50914849	-0.21	-0.02
Asb7	chr7	73792504	73823980	-0.99	-0.14
Asb7	chr7	73834117	73834387	-0.57	0.21
Det1	chr7	85972688	85972809	-1.48	-0.65
Det1	chr7	85988116	85988866	-0.31	-0.01
Trim21	chr7	109707270	109708500	-0.61	0.13
Kif22	chr7	134179351	134185934	-1.40	-0.39
Ifitm2	chr7	148141740	148141860	-0.90	0.06
Hras	chr7	148376639	148378177	-1.50	-0.13
Hras	chr7	148378835	148379754	-0.27	-0.01
Hras	chr7	148379812	148379903	-0.43	0.54
Cdc16	chr8	13779233	13781763	-0.76	0.08
Ppp2cb	chr8	34710092	34710330	-0.74	-0.58
Sh3rf1	chr8	63704632	63808042	-0.71	0.21
Sh3rf1	chr8	63863616	63863797	-0.45	-0.26
Sh3rf1	chr8	63865414	63873071	-0.33	-0.05
Fbxl8	chr8	107791922	107793078	-0.54	-0.13
Nfatc3	chr8	108602571	108603618	-0.98	0.07
Nfatc3	chr8	108632435	108652166	-2.54	0.17
Ap1g1	chr8	112382745	112384686	-0.52	0.03
Znrf1	chr8	114060539	114062246	-2.99	0.21
Znrf1	chr8	114146249	114147028	-0.58	0.04
Gan	chr8	119682094	119707502	-2.38	0.66
Plcg2	chr8	120022190	120028105	-0.40	0.04
Tubb3	chr8	125944514	125944901	-0.18	-0.07
Itgb1	chr8	131209643	131209853	-0.70	-0.08
Ikbkb	chr8	23770546	23770997	-1.89	-0.25
Dctn6	chr8	35169406	35171565	-0.59	0.15
Lonrf1	chr8	37280137	37282962	-0.64	0.13
Lonrf1	chr8	37311699	37312090	-1.27	-0.69
Klhl2	chr8	67219421	67219870	-3.53	-0.50
Pik3r2	chr8	73292079	73292558	-1.14	-0.16
Calr	chr8	87366315	87366923	-2.76	-0.06
Calr	chr8	87369960	87370800	-0.94	0.04
Siah1a	chr8	89248756	89269816	-1.08	-0.12
Dync1li2	chr8	106944120	106944451	-1.40	-0.19
Psmb10	chr8	108461631	108461997	-1.25	-0.38
Fbxo31	chr8	124075878	124076237	-3.34	0.63
Icam1	chr9	20820403	20823729	-0.66	-0.08

Icam1	chr9	20824027	20824596	-1.12	0.25
Icam1	chr9	20831708	20833180	-0.81	0.04
Icam5	chr9	20838239	20839240	-1.67	1.05
Icam5	chr9	20840977	20842091	-0.59	0.20
Dnm2	chr9	21310134	21311379	-1.31	0.09
Ppp2r1b	chr9	50687990	50688710	-0.88	-0.11
Ppp2r1b	chr9	50701016	50701705	-0.62	0.10
Psma4	chr9	54805658	54805748	-1.39	0.21
Fbxo22	chr9	55066270	55071672	-2.03	0.17
Fbxo22	chr9	55071880	55072031	-0.93	-0.28
Anapc13	chr9	102528625	102536370	-0.48	0.06
Dcaf1	chr9	106738991	106740852	-3.06	0.55
Dcaf1	chr9	106760607	106761940	-3.49	0.37
Dync1li1	chr9	114632656	114632895	-3.05	0.38
Dync1li1	chr9	114632812	114633160	-1.11	0.11
Fbxl12	chr9	20448850	20449181	-0.31	-0.05
Keap1	chr9	21034503	21038410	-0.91	0.07
Keap1	chr9	21041585	21042276	-1.22	-0.20
Keap1	chr9	21043025	21043776	-0.79	-0.29
Crtam	chr9	40781146	40781679	-0.68	1.01
Ube4a	chr9	44731568	44732078	-2.10	0.26
Ube4a	chr9	44756010	44756191	-0.52	-0.14
Cul5	chr9	53425530	53426100	-1.13	0.12
Rnf111	chr9	70274585	70277390	-1.13	-0.14
Fbxo9	chr9	77943491	77943611	-0.30	0.14
Pik3cb	chr9	98939628	98941447	-3.03	0.22
Arih2	chr9	108507307	108507428	-0.63	0.11
Arih2	chr9	108522434	108547287	-1.35	0.23
Fyn	chr10	39089604	39089964	-1.17	-0.23
Fyn	chr10	39271197	39284162	-3.65	-0.24
Itgb2	chr10	76993092	77005145	-1.85	-0.24
Ube2g2	chr10	77107280	77107637	-0.93	-0.11
Ube2g2	chr10	77107992	77108141	-1.22	0.25
Icosl	chr10	77536609	77536670	-0.38	-0.28
Fbxo7	chr10	85487309	85487369	-0.37	0.10
Tab2	chr10	7625983	7626463	-1.95	0.18
Tab2	chr10	7626671	7644544	-2.25	0.07
Rnf217	chr10	31254643	31329531	-1.58	-0.29
Ube2d1	chr10	70718055	70718592	-2.86	0.34
Rnf126	chr10	79221259	79223642	-1.89	-0.11
Rnf126	chr10	79229537	79229686	-1.44	-0.51
Fzr1	chr10	80829682	80830187	-1.59	0.17
Kif5a	chr10	126700029	126700180	-1.18	-0.45

Ap1b1	chr11	4847942	4887051	-1.74	0.41
Psme4	chr11	30671774	30672135	-0.53	-0.17
Fbxw11	chr11	32644536	32644986	-0.65	0.06
Rnf130	chr11	49933551	49933881	-0.41	0.38
Ppp2ca	chr11	51935449	51935836	-0.93	0.05
Kif3a	chr11	53380880	53380941	-1.76	-1.08
Trim11	chr11	58791617	58791916	-0.34	-0.02
Trim11	chr11	58802336	58802515	-1.26	0.40
Trim11	chr11	58802842	58804960	-1.02	0.09
Ube2g1	chr11	72420792	72421062	-2.86	-0.55
Ube2g1	chr11	72497025	72497295	-0.29	0.00
Psmd11	chr11	80285574	80285635	-1.26	-0.32
Psmd3	chr11	98556993	98557203	-2.02	-0.21
Psme3	chr11	101177564	101178336	-0.77	0.09
Psme3	chr11	101182100	101183258	-2.86	-0.26
Trim41	chr11	48625890	48630474	-2.56	-0.19
Trim41	chr11	48630623	48630893	-2.44	0.54
Sec24a	chr11	51556955	51570200	-1.50	-0.11
Wsb1	chr11	79053599	79053898	-2.67	-0.17
Ube2z	chr11	95911464	95911704	-2.06	-0.26
Fbxl20	chr11	97944196	97944257	-0.53	0.44
Klhl11	chr11	100323925	100325737	-2.89	0.19
Klhl11	chr11	100333857	100334096	-0.43	-0.10
Map3k14	chr11	103081077	103082247	-0.72	-0.12
Map3k14	chr11	103100286	103101085	-0.44	0.06
Map3k14	chr11	103103466	103128715	-0.61	-0.18
Cdc27	chr11	104366205	104368561	-0.85	-0.06
Cd79b	chr11	106172774	106172954	-0.59	3.17
Smurf2	chr11	106683743	106684358	-1.06	-0.24
Smurf2	chr11	106781579	106781849	-2.81	-0.99
Cd300c2	chr11	114862804	114862865	-0.52	-0.36
Cd300e	chr11	114914519	114914670	-1.08	1.43
Grb2	chr11	115505517	115505906	-2.19	0.26
Grb2	chr11	115506561	115508033	-2.47	0.12
Ube2o	chr11	116399633	116399902	-0.30	0.01
Ube2o	chr11	116400140	116404120	-2.64	-0.17
Ube2o	chr11	116405875	116406025	-0.84	0.21
Ube2o	chr11	116442611	116442731	-0.48	-0.68
Socs3	chr11	117830559	117830620	-0.38	0.48
Kif3c	chr12	3365310	3367494	-1.39	-0.14
Kif3c	chr12	3403233	3403504	-0.69	0.15
Kif3c	chr12	3405476	3405926	-1.66	0.23
Psma6	chr12	56499811	56499900	-1.08	-0.18

Lrr1	chr12	70275442	70276002	-1.46	-0.50
Psmc1	chr12	101348377	101351316	-0.70	-0.11
Dync1h1	chr12	111839634	111839815	-1.16	-0.25
Dync1h1	chr12	111855934	111857506	-0.62	-0.02
Dync1h1	chr12	111904592	111905154	-2.05	0.14
Klc1	chr12	112997059	113010604	-0.37	-0.02
Ppp2r5e	chr12	76594419	76616288	-3.57	0.50
Ppp2r5e	chr12	76616467	76697187	-3.21	0.22
Arel1	chr12	86259725	86261648	-0.82	0.05
Arel1	chr12	86311686	86311836	-0.96	0.38
Mylip	chr13	45503815	45506325	-3.05	0.42
Rnf144b	chr13	47338305	47340700	-0.89	-0.14
Rnf144b	chr13	47342821	47343181	-1.25	0.41
Syk	chr13	52678805	52706692	-1.23	-0.09
Syk	chr13	52727039	52733575	-0.39	-0.02
Syk	chr13	52737300	52738635	-0.88	-0.13
Syk	chr13	52738964	52742902	-3.69	-0.15
Syk	chr13	52743800	52743951	-0.57	-0.11
Erap1	chr13	74777319	74783985	-2.40	0.26
Tubb2b	chr13	34219319	34219498	-1.13	2.05
Tubb2b	chr13	34220029	34220783	-1.25	-0.78
Ctsl	chr13	64470393	64471614	-1.97	-0.09
Pik3r1	chr13	102453956	102456264	-1.75	-0.18
Pik3r1	chr13	102478932	102525656	-1.06	0.31
Pik3r1	chr13	102527214	102538172	-0.33	-0.01
Kctd6	chr14	9052713	9055605	-1.86	-0.26
Sec24c	chr14	21512398	21513205	-0.75	-0.07
Sec24c	chr14	21513563	21514072	-1.48	-0.13
Psmc6	chr14	45949498	45950514	-0.58	0.08
Kbtbd7	chr14	79827126	79828716	-1.70	0.29
Ppp3cb	chr14	21365614	21365735	-1.14	-0.58
Fbxl3	chr14	103482090	103488631	-0.81	-0.05
Fbxl3	chr14	103494436	103498143	-0.54	-0.05
Fbxl3	chr14	103498202	103498726	-1.82	-0.43
Fyb	chr15	6529870	6569669	-1.33	-0.07
Fyb	chr15	6584856	6590489	-0.34	-0.01
Rictor	chr15	6745928	6747254	-2.42	0.20
Ncf4	chr15	78075240	78075447	-0.37	-0.03
Ncf4	chr15	78091438	78092951	-2.32	-0.15
Rbx1	chr15	81305941	81306090	-0.83	-0.28
Tuba1c	chr15	98867757	98867967	-0.36	0.01
Skp2	chr15	9043329	9043540	-0.80	0.27
Skp2	chr15	9069207	9070167	-0.68	-0.14

Rnf19a	chr15	36170946	36173866	-0.40	0.03
Ywhaz	chr15	36723405	36723964	-0.95	0.41
Fbxo32	chr15	58011872	58012083	-0.36	-0.16
Fbxo32	chr15	58012410	58013069	-1.46	-0.50
Fbxo32	chr15	58046223	58046433	-2.16	-1.12
Col2a1	chr15	97806360	97806570	-1.38	2.81
Asb8	chr15	97965939	97971774	-0.39	0.02
Racgap1	chr15	99470224	99482052	-1.10	-0.20
Itgb7	chr15	102046603	102047016	-0.40	-0.02
Itgb7	chr15	102057938	102062366	-1.68	0.23
Mgrn1	chr16	4936923	4937342	-0.36	0.02
Klhl22	chr16	17771956	17777358	-1.71	-0.20
Klhl22	chr16	17778345	17778466	-0.64	0.80
Ap2m1	chr16	20535576	20537801	-0.37	-0.06
Psmd2	chr16	20651724	20652324	-3.25	-0.17
Itgb5	chr16	33829750	33830049	-3.12	-1.09
Itgb5	chr16	33919922	33920251	-0.33	-0.01
Itgb5	chr16	33947843	33949275	-0.39	-0.02
Cd80	chr16	38473975	38479185	-1.67	-0.16
Cd80	chr16	38486416	38486895	-3.14	-0.36
Cd80	chr16	38494596	38494776	-0.67	0.24
Cd200r1	chr16	44794312	44794552	-0.73	-0.27
Cd200r4	chr16	44811897	44820870	-1.68	-0.99
Cd200r2	chr16	44915270	44915894	-0.26	-0.01
Cblb	chr16	52166229	52166380	-0.94	0.44
Cblb	chr16	52204565	52205105	-0.38	-0.05
Socs1	chr16	10783960	10784764	-0.35	0.15
Socs1	chr16	10784822	10784882	-0.42	-1.26
Socs1	chr16	10784970	10785540	-0.17	-0.27
Pak2	chr16	32019862	32021909	-1.43	0.09
Dtx3l	chr16	35929143	35932046	-2.43	0.35
Dtx3l	chr16	35932375	35933483	-0.33	0.01
Cd86	chr16	36604193	36604552	-0.46	-0.35
Cd86	chr16	36620796	36620917	-0.87	1.18
Ltn1	chr16	87415807	87416587	-2.10	0.18
Itpr3	chr17	27258538	27258808	-0.49	0.07
H2-DMa	chr17	34272202	34272620	-1.64	0.61
Tap1	chr17	34324500	34325281	-0.31	0.01
Tap1	chr17	34333539	34333870	-2.43	0.51
Psmb8	chr17	34335288	34336214	-1.01	-0.16
Tap2	chr17	34341423	34342567	-0.60	-0.10
Tap2	chr17	34352342	34353206	-0.31	-0.01
H2-Ob	chr17	34378095	34378156	-0.21	1.00

H2-D1	chr17	35403787	35404234	-3.70	-0.07
H2-Q6	chr17	35565949	35566720	-0.96	0.29
H2-Q7	chr17	35576925	35577015	-0.56	-1.32
H2-Q10	chr17	35607880	35610039	-0.32	0.07
Nfkbie	chr17	45692664	45695502	-1.11	-0.14
Nfkbie	chr17	45697103	45697389	-0.61	-0.25
Nfkbie	chr17	45697447	45700117	-0.90	-0.09
Cul7	chr17	46787586	46787707	-0.19	0.03
Cul7	chr17	46788456	46789468	-0.30	-0.09
Trem1	chr17	48371921	48385139	-2.64	1.06
Treml2	chr17	48438863	48439774	-1.94	2.67
Treml2	chr17	48449100	48449610	-0.28	0.02
Pdpk1	chr17	24215583	24216033	-2.93	0.32
Ccnf	chr17	24360773	24360864	-1.19	-0.44
Stub1	chr17	25967580	25967909	-2.17	-0.16
Stub1	chr17	25969947	25970306	-0.38	0.08
H2-K1	chr17	34136362	34136423	-0.41	0.19
H2-T23	chr17	36169099	36248130	-0.77	-0.09
Trim39	chr17	36397225	36398520	-1.76	0.23
Trim39	chr17	36400794	36404080	-1.08	-0.35
Klc4	chr17	46781224	46782971	-0.41	0.07
Ppp2r5d	chr17	46820028	46820862	-1.04	-0.11
Ppp2r5d	chr17	46837029	46841951	-0.31	0.02
Ubr2	chr17	47065869	47067399	-0.68	-0.05
Ubr2	chr17	47147240	47147481	-0.50	-0.08
Pja2	chr17	64658035	64660528	-0.54	-0.04
Xdh	chr17	74238633	74238694	-0.24	-0.06
Sos1	chr17	80797041	80799097	-0.34	-0.01
Sos1	chr17	80832979	80834320	-1.17	0.18
Sos1	chr17	80879313	80879554	-0.43	-0.33
Calm2	chr17	87835011	87846216	-3.10	-0.20
Fbxo11	chr17	88390945	88391656	-0.52	0.03
Colec12	chr18	9707645	9840283	-0.84	-0.07
Colec12	chr18	9848284	9859890	-1.40	-0.04
Ube2d2a	chr18	35931242	35931392	-1.92	-0.45
Ube2d2a	chr18	35965360	35965570	-1.78	0.47
Rnf14	chr18	38467720	38467990	-0.45	0.07
Dctn4	chr18	60686409	60686590	-0.35	0.21
Dctn4	chr18	60716345	60716496	-0.75	-0.18
Tubb6	chr18	67550384	67552330	-3.31	-0.30
Mex3c	chr18	73732718	73749293	-0.62	-0.08
Map3k8	chr18	4348929	4352951	-0.67	0.15
Cdc23	chr18	34792931	34793291	-2.74	-0.56

Cdc23	chr18	34793470	34793621	-0.73	-0.23
Trim36	chr18	46326929	46328816	-0.84	0.34
Nfatc1	chr18	80803872	80832687	-3.26	-0.24
Nfatc1	chr18	80846262	80846653	-1.93	0.30
Nfatc1	chr18	80879052	80904894	-0.33	-0.01
Sptbn2	chr19	4744084	4745130	-1.43	-0.66
Rela	chr19	5637578	5637639	-0.26	0.03
Cd274	chr19	29441927	29460289	-0.50	0.04
Pdcd1lg2	chr19	29545031	29545330	-0.38	0.49
Btrc	chr19	45604207	45604268	-0.27	-0.42
Fbxl15	chr19	46403619	46404936	-0.68	0.11
Klc2	chr19	5117426	5118348	-2.22	-0.46
Sipa1	chr19	5651184	5651544	-1.77	0.18
Sipa1	chr19	5652777	5652838	-0.60	-0.18
Sipa1	chr19	5653976	5655148	-1.43	-0.12
Sipa1	chr19	5659486	5662646	-2.21	-0.13
Ppp2r5b	chr19	6227915	6228302	-0.46	0.08
Chuk	chr19	44181846	44181937	-0.41	-0.11
Fbxw4	chr19	45652836	45653256	-0.43	-0.03
Actr1a	chr19	46452405	46452913	-1.68	-0.11
Ube2a	chrX	34414360	34414479	-0.33	-0.03
Ikbkg	chrX	71675074	71678148	-0.70	-0.30
Kif4	chrX	97921923	97922431	-2.34	-0.35
Ap1s2	chrX	160347091	160347182	-0.27	0.01
Was	chrX	7667496	7667617	-0.32	0.03
Pja1	chrX	96661669	96661909	-3.01	0.59
Pja1	chrX	96662087	96663849	-0.78	0.06
Siah1b	chrX	160509579	160510081	-0.42	0.07
Stim1	chr7	109416755	109416875	-0.47	-0.21
Dynll2	chr11	87794645	87799923	-0.49	0.03

Death receptor signaling

gene_name	chr	chromStart	chromEnd	diff.lg.p	diff.log2.fc
Arhgef4	chr1	34868581	34868971	-0.43	0.12
Cflar	chr1	58779883	58783419	-2.72	0.60
Casp8	chr1	58890568	58903481	-0.47	0.03
Usp21	chr1	173216496	173218092	-3.29	-0.45
Ncstn	chr1	173996440	173996917	-1.85	-0.17
Psen2	chr1	182157552	182159085	-2.06	0.25
Psen2	chr1	182175838	182176005	-0.47	-0.20
Traf6	chr2	101531587	101537929	-3.55	-0.22
Traf2	chr2	25374427	25375145	-1.34	-0.17
Vav2	chr2	27282074	27282345	-0.49	-0.25
Traf1	chr2	34803327	34804015	-1.17	-0.19

Madd	chr2	90977546	90977756	-0.38	-0.07
Madd	chr2	91018180	91018480	-0.45	-0.08
Prex1	chr2	166392533	166394859	-1.51	-0.12
Prkci	chr3	30949234	30949475	-1.03	0.27
Arhgef11	chr3	87536684	87539298	-0.86	0.09
Arhgef11	chr3	87541566	87541835	-0.37	0.03
Arhgef2	chr3	88447296	88447775	-2.17	-0.29
Otud7b	chr3	95956546	95960193	-2.15	-0.13
Vav3	chr3	109143630	109143900	-2.32	-1.00
Nfkb1	chr3	135248217	135252299	-2.46	-0.20
Nfkb1	chr3	135354240	135354361	-0.32	0.21
Map3k7	chr4	32051081	32051232	-2.61	-1.03
Map3k7	chr4	32109961	32110261	-0.43	0.08
Plekhg5	chr4	151487918	151488669	-0.44	0.10
Plekhg5	chr4	151488938	151489269	-0.44	0.21
Nsmaf	chr4	6381298	6381418	-0.26	-0.16
Ripk2	chr4	16050729	16051621	-2.11	-0.28
Plekhg2	chr7	29144712	29147677	-2.02	0.33
Plekhg2	chr7	29155655	29156014	-0.66	0.72
Plekhg2	chr7	29156784	29157353	-1.64	-0.63
Arhgef17	chr7	108021528	108022039	-0.33	0.13
Arhgef17	chr7	108077255	108078935	-1.29	1.05
Arhgef17	chr7	108079084	108079355	-0.30	1.00
Fadd	chr7	151765572	151768281	-2.06	-0.21
Arhgef18	chr8	3452169	3453370	-0.29	-0.01
Arhgef18	chr8	3454865	3455284	-1.14	-0.39
Arhgef7	chr8	11831499	11833540	-3.31	-0.15
Mcf2l	chr8	12963213	12984716	-1.11	-0.34
Mcf2l	chr8	13019010	13019580	-1.02	-0.25
Cyld	chr8	91220956	91226218	-0.51	0.31
Ikbkb	chr8	23770546	23770997	-1.89	-0.25
Bag4	chr8	26877798	26878878	-2.46	0.26
Tradd	chr8	107782474	107783441	-0.33	0.01
Tradd	chr8	107783843	107788494	-1.18	0.34
Smpd3	chr8	108779174	108779539	-0.33	-1.29
Usp2	chr9	43883576	43884236	-2.87	2.50
Usp2	chr9	43902032	43902332	-0.41	-0.10
Usp4	chr9	108293381	108294561	-0.32	0.00
Birc2	chr9	7818584	7818943	-0.91	0.25
Birc2	chr9	7834955	7835255	-3.06	0.75
Birc3	chr9	7848939	7850928	-0.49	0.05
Birc3	chr9	7860311	7872961	-0.93	0.08
Arhgef12	chr9	42780101	42782733	-1.74	0.34

Arhgef12	chr9	42850752	42913801	-0.30	0.01
Aph1b	chr9	66626669	66627088	-1.17	0.50
Aph1c	chr9	66666695	66667266	-1.28	0.20
Myd88	chr9	119248949	119249158	-0.84	-0.19
Sppl2b	chr10	80329626	80329897	-0.88	0.59
Tab2	chr10	7625983	7626463	-1.95	0.18
Tab2	chr10	7626671	7644544	-2.25	0.07
Tnfaip3	chr10	18721581	18725137	-2.68	-0.19
Rtn4	chr11	29606743	29607283	-0.34	0.01
Rtn4	chr11	29607402	29607523	-0.92	-0.16
Rtn4	chr11	29607881	29608301	-1.69	0.16
Obscn	chr11	58814511	58814661	-0.15	0.07
Obscn	chr11	58826304	58826575	-0.26	0.74
Obscn	chr11	58826873	58827144	-0.85	4.21
Abr	chr11	76307868	76307958	-0.39	-0.11
Abr	chr11	76308047	76308378	-1.64	0.47
Sos2	chr12	70685584	70689696	-1.15	-0.08
Sos2	chr12	70782629	70782809	-1.89	-1.62
Ripk1	chr13	34119764	34125274	-2.04	-0.13
Ripk1	chr13	34125393	34126112	-1.53	-0.25
Net1	chr13	3883211	3883659	-0.79	0.14
Net1	chr13	3916774	3917197	-0.55	0.55
Fgd3	chr13	49358478	49360128	-1.14	0.09
Fgd3	chr13	49391715	49393309	-1.44	0.14
Arhgef3	chr14	28213521	28215536	-0.57	0.07
Arhgef40	chr14	52604567	52607129	-0.57	-0.35
Arhgef40	chr14	52608357	52609817	-1.15	-0.15
Arhgef40	chr14	52616039	52617444	-0.67	-0.12
Tnfrsf10b	chr14	70180378	70182455	-2.58	0.84
Trio	chr15	27661541	27663364	-0.93	0.09
Trio	chr15	27673747	27673928	-0.94	0.23
Trio	chr15	27677706	27678216	-2.69	0.33
Trio	chr15	27830982	27834917	-0.75	0.09
Sharpin	chr15	76180512	76181540	-0.37	-0.02
Fgd4	chr16	16422549	16422879	-1.88	-0.35
Fgd4	chr16	16475223	16482703	-0.35	0.04
Fgd4	chr16	16484139	16487285	-0.60	0.08
Tiam1	chr16	89789210	89791963	-1.13	0.32
Tiam2	chr17	3518167	3518737	-0.78	0.26
Tiam2	chr17	3519066	3519397	-0.95	0.43
Sos1	chr17	80797041	80799097	-0.34	-0.01
Sos1	chr17	80832979	80834320	-1.17	0.18
Sos1	chr17	80879313	80879554	-0.43	-0.33

Rela	chr19	5637578	5637639	-0.26	0.03
Bad	chr19	7016906	7025164	-0.30	-0.01
Chuk	chr19	44181846	44181937	-0.41	-0.11
Ikbkg	chrX	71675074	71678148	-0.70	-0.30
Tab3	chrX	82860462	82860673	-2.95	0.81
Tab3	chrX	82875864	82876014	-3.15	1.28
Irak1	chrX	71260386	71261911	-0.47	0.03

Metabolism of RNA

gene_name	chr	chromStart	chromEnd	diff.lg.p	diff.log2.fc
Imp4	chr1	34496703	34496967	-0.34	0.02
Cnot11	chr1	39592727	39593027	-0.49	-0.09
Cnot11	chr1	39603302	39603662	-1.63	-0.30
Cnot9	chr1	74575438	74575798	-2.06	-0.28
Utp14b	chr1	78654429	78655125	-1.34	-0.25
Psmd1	chr1	87961193	87961313	-0.67	0.21
Sf3b1	chr1	55084201	55084322	-0.54	-0.12
Ncl	chr1	88254778	88256030	-1.35	-0.07
Zc3h11a	chr1	135516447	135517433	-0.47	-0.02
Zc3h11a	chr1	135517641	135518733	-1.03	0.06
Zc3h11a	chr1	135521310	135523811	-2.56	0.12
Smg7	chr1	154685051	154685410	-0.32	0.01
Smg7	chr1	154686097	154687255	-0.57	-0.06
Smg7	chr1	154695731	154696031	-0.58	-0.06
Dhx9	chr1	155303276	155303397	-0.99	0.27
Hnrnpu	chr1	180259373	180260299	-0.30	0.00
Diexf	chr1	194933444	194933955	-0.38	0.03
Diexf	chr1	194944545	194948237	-3.49	0.29
Diexf	chr1	194954627	194955692	-0.29	0.02
Exosc2	chr2	31526256	31526345	-0.50	0.16
Exosc2	chr2	31536119	31536355	-0.38	-0.04
Nup214	chr2	31888785	31893653	-1.32	0.09
Psmc3	chr2	90894439	90895182	-1.38	-0.10
Cstf3	chr2	104504891	104505372	-2.37	0.28
Nop56	chr2	130100147	130100207	-0.28	0.02
Nop56	chr2	130104634	130105019	-2.46	-0.17
Ctnnbl1	chr2	157563136	157600315	-3.07	-0.25
Cstf1	chr2	172201351	172201608	-2.58	0.38
Rae1	chr2	172839488	172841203	-2.88	-0.32
Rpp38	chr2	3246220	3249900	-0.50	-0.05
Rbm17	chr2	11522350	11524826	-1.32	-0.27
Psmd5	chr2	34707993	34709794	-1.37	-0.17
Psmd5	chr2	34722532	34726482	-1.60	-0.40
Psmb7	chr2	38498946	38499426	-1.35	-0.19

Prpf40a	chr2	53049040	53050262	-0.84	0.13
Cwc22	chr2	77733949	77741886	-0.89	-0.07
Clp1	chr2	84563548	84566299	-2.19	-0.20
Snrpb	chr2	130002885	130005100	-2.05	-0.18
Psmf1	chr2	151543111	151543232	-0.83	-0.47
Psmf1	chr2	151543649	151544068	-0.81	-0.19
Smg5	chr3	88140181	88145781	-0.99	-0.23
Smg5	chr3	88154740	88155542	-2.74	0.16
Smg5	chr3	88165183	88165423	-0.60	-0.10
Adar	chr3	89518943	89540188	-1.84	-0.10
Adar	chr3	89554602	89555315	-3.64	-0.32
Rbm8a	chr3	96433850	96433910	-0.37	0.11
Bcas2	chr3	102982305	102982454	-2.42	-0.20
Prcc	chr3	87662854	87664130	-0.35	-0.02
Chtop	chr3	90303653	90304012	-2.00	-0.11
Chtop	chr3	90313185	90313365	-2.41	-0.21
Psmd4	chr3	94836626	94836924	-1.79	-0.12
Psmd4	chr3	94839816	94846467	-2.16	-0.13
Wdr3	chr3	99942341	99942760	-2.13	0.17
Ddx20	chr3	105481379	105482396	-0.68	-0.06
Rnpc3	chr3	113332946	113333067	-0.48	-0.23
Srsf11	chr3	157674772	157675838	-1.15	-0.06
Tgs1	chr4	3542039	3542219	-2.76	-0.54
Rngtt	chr4	33587224	33587763	-1.62	0.30
Rngtt	chr4	33588270	33588390	-0.93	0.89
Prpf4	chr4	62083697	62085481	-1.56	-0.17
Ndc1	chr4	107083754	107086943	-0.44	0.04
Tut4	chr4	108185594	108190039	-0.69	-0.08
Ebna1bp2	chr4	118293433	118293583	-2.58	-0.41
Lsm10	chr4	125773896	125775828	-1.43	-0.17
Dnajc8	chr4	132108975	132109154	-0.58	-0.05
Syf2	chr4	134486894	134490962	-2.04	-0.13
Syf2	chr4	134493303	134493452	-1.42	-0.35
Hnrnpr	chr4	135895368	135895996	-0.58	0.05
Exosc10	chr4	147932535	147933683	-0.30	0.01
Nol9	chr4	151431914	151434061	-2.41	-0.13
Nol6	chr4	41070603	41071312	-1.27	0.26
Exosc3	chr4	45333265	45333475	-2.15	-0.38
Tex10	chr4	48481183	48482144	-1.89	0.14
Ppih	chr4	118979838	118983372	-1.31	0.29
Srrm1	chr4	134878175	134878385	-0.88	-0.09
Srrm1	chr4	134902739	134909069	-1.22	0.14
Nupl2	chr5	23687756	23687817	-0.27	0.02

Nupl2	chr5	23688026	23689736	-0.62	0.07
Utp3	chr5	88983507	88985078	-2.13	0.07
Psmd9	chr5	123678228	123684616	-1.07	0.18
Snrnp35	chr5	124933163	124941013	-1.28	0.15
Gtf2h3	chr5	125046222	125046313	-0.93	-0.48
Bud31	chr5	145908765	145908943	-2.11	-0.28
Pan3	chr5	148242155	148242515	-0.49	-0.14
Pan3	chr5	148357233	148359090	-0.83	-0.06
Slbp	chr5	33992485	33994955	-0.80	0.14
Nop14	chr5	35000599	35002797	-0.49	-0.04
Nup54	chr5	92845130	92846543	-1.56	0.19
Dhx37	chr5	125894862	125895712	-3.65	-0.32
Pom121	chr5	135853620	135857198	-0.30	0.00
Pom121	chr5	135857496	135857795	-0.92	0.12
Pom121	chr5	135869997	135870416	-0.68	-0.16
Nup205	chr6	35127615	35133895	-0.29	0.01
Hnrnpf	chr6	117850506	117856962	-2.04	-0.29
Hnrnpf	chr6	117873756	117874265	-1.34	-0.10
Ddx47	chr6	134971525	134973794	-0.44	-0.02
Gm8994	chr6	136278992	136279111	-0.18	0.47
Gm8994	chr6	136279792	136279971	-0.26	0.19
Hnrnpa2b1	chr6	51419743	51419893	-1.91	-0.18
Usp39	chr6	72268758	72269207	-1.04	0.14
Pcbp1	chr6	86474877	86476159	-0.78	-0.01
Isy1	chr6	87769010	87771528	-0.31	-0.01
Nup210	chr6	90963060	90963720	-3.45	-0.38
Bms1	chr6	118333518	118334272	-1.98	-0.17
Bms1	chr6	118348132	118355287	-2.43	-0.14
Apobec1	chr6	122528643	122531627	-0.79	-0.05
Wbp11	chr6	136762473	136765520	-1.76	-0.10
Prpf31	chr7	3581586	3582460	-1.60	0.15
Snrpd2	chr7	19735186	19736653	-1.83	-0.27
Ercc2	chr7	19979441	19981041	-2.11	-0.19
Polr2i	chr7	31017332	31017772	-0.29	0.00
Gtf2h1	chr7	54051493	54051554	-1.82	-0.48
Snrpa1	chr7	73205399	73205549	-1.71	-0.33
Gemin7	chr7	20150297	20158692	-2.65	0.16
Supt5	chr7	29116415	29116476	-0.45	0.06
Psmd8	chr7	29965393	29965573	-1.95	-0.13
Zfp473	chr7	51987480	51988021	-1.22	1.42
Zfp473	chr7	51988559	51988710	-0.18	-0.04
Zfp473	chr7	51989249	51989760	-1.53	1.19
Pcf11	chr7	99801691	99808521	-0.95	-0.05

Nup98	chr7	109268003	109269291	-1.15	-0.06
Polr2l	chr7	148659279	148661052	-0.40	0.16
Sugp1	chr8	72566920	72572439	-0.33	-0.05
Polr2c	chr8	97387425	97387634	-0.99	-0.15
Exosc6	chr8	113580238	113581499	-0.47	-0.02
Elavl1	chr8	4289159	4289430	-1.24	-0.17
Cnot7	chr8	41592761	41593001	-1.13	0.18
Ddx49	chr8	72816972	72817479	-2.30	-0.29
Upf1	chr8	72855420	72856900	-0.33	-0.01
Psmb10	chr8	108461631	108461997	-1.25	-0.38
Nob1	chr8	109936418	109936954	-0.42	-0.02
Dhx38	chr8	112071923	112073753	-2.72	0.29
Sf3b3	chr8	113334571	113334842	-1.95	0.11
Mphosph6	chr8	120316041	120316218	-1.00	0.21
Psma4	chr9	54805658	54805748	-1.39	0.21
Imp3	chr9	56785306	56786205	-1.77	-0.08
Snupn	chr9	56830587	56830946	-0.45	0.07
Edc3	chr9	57563979	57575316	-0.39	0.03
Edc3	chr9	57596029	57596537	-1.30	-0.20
Xrn1	chr9	95953058	95953238	-1.76	0.33
Rrp9	chr9	106379639	106379729	-1.26	0.64
Cnot10	chr9	114541840	114549228	-0.65	0.12
Ppil4	chr10	7534486	7541105	-0.34	0.01
Hbs1l	chr10	21087565	21088546	-0.77	-0.09
Wdr18	chr10	79422896	79423107	-0.85	-0.15
Sf3a2	chr10	80261479	80263960	-0.73	-0.20
Sf3a2	chr10	80266830	80266980	-0.97	-0.18
Pan2	chr10	127740450	127741373	-1.33	-0.44
Pan2	chr10	127747269	127747789	-0.41	-0.06
Ccar1	chr10	62208074	62209776	-1.59	-0.14
Adarb1	chr10	76757152	76758488	-0.28	0.00
Pwp2	chr10	77633953	77636188	-0.47	-0.03
Polr2e	chr10	79498845	79498935	-0.48	-0.07
Polr2e	chr10	79501286	79502404	-0.85	-0.12
Utp20	chr10	88209351	88209771	-0.43	0.03
Cnot2	chr10	115922216	115922606	-1.24	0.23
Cnot2	chr10	115922874	115923115	-0.75	0.16
Pes1	chr11	3878878	3878938	-0.44	-0.06
Sf3a1	chr11	4080296	4080657	-3.20	-0.37
Zmat5	chr11	4604673	4628589	-1.56	-0.46
Thoc5	chr11	4799846	4799907	-2.86	-0.63
Xpo1	chr11	23156040	23156101	-0.70	0.34
Psme4	chr11	30671774	30672135	-0.53	-0.17

Slu7	chr11	43247232	43251629	-0.59	0.07
Slu7	chr11	43258883	43259812	-2.51	-0.21
Ppp2ca	chr11	51935449	51935836	-0.93	0.05
Cnot8	chr11	57930958	57931228	-0.69	-0.18
Tsr1	chr11	74711581	74711952	-1.34	-0.25
Smg6	chr11	74739373	74744156	-2.46	0.17
Psmd11	chr11	80285574	80285635	-1.26	-0.32
Ddx52	chr11	83773028	83775842	-2.44	0.26
Ddx52	chr11	83776110	83776349	-0.51	0.12
Srsf1	chr11	87861172	87861322	-3.40	-0.49
Psmd3	chr11	98556993	98557203	-2.02	-0.21
Casc3	chr11	98683710	98689970	-1.75	-0.13
Psme3	chr11	101177564	101178336	-0.77	0.09
Psme3	chr11	101182100	101183258	-2.86	-0.26
Pno1	chr11	17111326	17111592	-0.47	-0.04
Lsm11	chr11	45747027	45747476	-0.85	-0.20
Gemin5	chr11	57933831	57934041	-0.75	0.49
Gemin5	chr11	57934607	57938883	-1.26	-0.14
Senp3	chr11	69492510	69495389	-1.27	-0.06
Pelp1	chr11	70206442	70208529	-2.16	-0.13
Gemin4	chr11	76024102	76025477	-3.09	-0.31
Gemin4	chr11	76025925	76027390	-0.34	-0.01
Utp6	chr11	79748406	79749481	-1.83	-0.13
Cwc25	chr11	97608042	97614001	-2.20	-0.20
Nol11	chr11	107028524	107029031	-1.12	0.16
Nol11	chr11	107050456	107050606	-1.12	-0.29
Prkca	chr11	107797729	107797880	-2.84	2.75
Csnk1d	chr11	120819778	120820048	-2.61	-0.30
Csnk1d	chr11	120823779	120824109	-0.34	0.01
Csnk1d	chr11	120824767	120826288	-1.85	-0.13
Csnk1d	chr11	120852257	120852467	-0.47	-0.06
Psma6	chr12	56499811	56499900	-1.08	-0.18
Psmc1	chr12	101348377	101351316	-0.70	-0.11
Cpsf2	chr12	103214183	103214333	-1.55	0.42
Cpsf2	chr12	103228209	103228270	-1.83	0.39
Cpsf2	chr12	103240910	103242024	-2.01	0.13
Papola	chr12	107022971	107023122	-1.31	-0.13
Papola	chr12	107072945	107075506	-0.44	-0.02
Zfp36l1	chr12	81209730	81210030	-0.70	-0.13
Zfp36l1	chr12	81210476	81214000	-2.20	-0.17
Snw1	chr12	88791126	88791810	-2.89	-0.19
Riok1	chr13	38152674	38153063	-2.08	0.35
Snrnp48	chr13	38312637	38313246	-0.58	-0.09

Ddx46	chr13	55741151	55751787	-0.70	-0.04
Smn1	chr13	100894809	100896580	-2.84	-0.58
Nup153	chr13	46776258	46776499	-0.69	-0.10
Nup153	chr13	46782010	46792068	-2.74	0.10
Thoc3	chr13	54561240	54561569	-1.64	-0.23
Hnrnpk	chr13	58501349	58504674	-2.32	-0.21
Tut7	chr13	59873478	59883679	-1.72	0.15
Tut7	chr13	59921505	59921566	-0.33	0.05
Tut7	chr13	59924328	59924508	-0.25	0.02
Utp15	chr13	99018475	99020783	-1.18	-0.09
Ppwd1	chr13	105018743	105018924	-1.46	0.46
Rpp14	chr14	8922487	8922934	-2.35	-0.26
Dcp1a	chr14	31332485	31332636	-1.01	0.22
Dcp1a	chr14	31336008	31337367	-1.18	-0.13
Dcp1a	chr14	31339462	31339853	-2.08	0.39
Psmc6	chr14	45949498	45950514	-0.58	0.08
Prkcd	chr14	31408808	31411699	-0.55	0.03
Prkcd	chr14	31411787	31411937	-0.29	-0.01
Gnl3	chr14	31830581	31832216	-0.32	0.00
Ppp2r2a	chr14	67634507	67635434	-0.31	0.00
Gtf2f2	chr14	76297012	76306651	-0.54	-0.05
Wbp4	chr14	79861569	79863783	-2.10	-0.28
Dis3	chr14	99476449	99476539	-0.45	0.21
Polr2k	chr15	36103771	36103949	-0.31	-0.05
Dcaf13	chr15	38944419	38950257	-0.48	0.07
Exosc4	chr15	76157856	76159491	-1.97	-0.40
Nup50	chr15	84757805	84765852	-1.08	-0.07
Nup50	chr15	84773033	84773303	-0.62	0.12
Eif4b	chr15	101925104	101925897	-1.32	-0.05
Ywhaz	chr15	36723405	36723964	-0.95	0.41
Puf60	chr15	75900611	75900909	-0.96	-0.09
Bop1	chr15	76305252	76307699	-1.75	-0.22
Csnk1e	chr15	79248761	79248941	-0.78	0.21
Csnk1e	chr15	79249120	79251327	-0.68	-0.06
Csnk1e	chr15	79252916	79254661	-1.40	-0.13
Csnk1e	chr15	79272227	79272487	-1.09	-0.88
Poldip3	chr15	82958053	82959641	-2.39	-0.13
Poldip3	chr15	82968734	82968795	-0.70	0.11
Ddx23	chr15	98488768	98493320	-1.34	-0.17
Psmd2	chr16	20651724	20652324	-3.25	-0.17
Eif4g1	chr16	20679306	20680085	-1.42	0.04
Ncbp2	chr16	31957240	31957450	-2.58	-0.37
Fyttd1	chr16	32877869	32884216	-0.32	-0.02

Fyttd1	chr16	32902586	32905812	-0.49	-0.07
Srrm2	chr17	23948991	23949082	-0.71	-0.24
Srrm2	chr17	23952780	23953201	-1.82	-0.06
Srrm2	chr17	23957516	23958116	-1.42	0.05
Rnps1	chr17	24551619	24552074	-1.68	-0.36
Wdr46	chr17	34077667	34077894	-0.91	-0.17
Psmb8	chr17	34335288	34336214	-1.01	-0.16
Ddx39b	chr17	35378690	35378917	-2.25	-0.37
Ddx39b	chr17	35386162	35386790	-0.42	-0.02
Ddx39b	chr17	35566011	35566729	-0.95	0.28
Wdr43	chr17	71965558	71965768	-0.58	0.10
Wdr43	chr17	72006872	72007292	-0.52	-0.03
Wdr43	chr17	72008010	72008371	-0.32	0.01
Gemin6	chr17	80625009	80627484	-1.78	-0.35
Wtap	chr17	13167327	13168316	-1.35	0.15
Thoc6	chr17	23810618	23810831	-0.44	0.09
Tbl3	chr17	24843061	24844598	-0.53	-0.11
Hnrnpm	chr17	33814217	33822403	-1.25	-0.08
Hspa1b	chr17	35093851	35095346	-0.55	0.06
Hspa1b	chr17	35095554	35096183	-2.02	0.44
Hspa1a	chr17	35107225	35107435	-1.72	-4.26
Rpp21	chr17	36394428	36394791	-2.67	-0.51
Cdc5l	chr17	45528987	45530123	-0.34	-0.01
Cdc5l	chr17	45564979	45570507	-0.86	-0.36
Rrp36	chr17	46804404	46807115	-1.11	-0.13
Rrp36	chr17	46809705	46811204	-0.48	0.10
Bysl	chr17	47748141	47748202	-0.31	-0.06
Gtf2f1	chr17	57143401	57143559	-0.53	-0.10
Gtf2f1	chr17	57143961	57144203	-0.51	-0.06
Srsf7	chr17	80606178	80606645	-0.76	-0.19
Thoc1	chr18	9993386	9993507	-0.34	0.02
Riok3	chr18	12313852	12314001	-0.31	0.01
Polr2d	chr18	31955847	31956355	-3.30	0.43
Wdr33	chr18	32067351	32067412	-0.44	0.52
Ercc3	chr18	32399984	32400372	-0.36	0.04
Dcp2	chr18	44572199	44578164	-2.22	0.24
Rnmt	chr18	68460038	68465632	-1.16	0.13
Txnl4a	chr18	80403987	80404903	-0.90	-0.14
Txnl4a	chr18	80415452	80419362	-1.31	-0.12
Txnl4a	chr18	80421065	80421873	-1.19	-0.41
Fau	chr19	6057887	6057975	-1.20	0.60
Prpf19	chr19	10969781	10969902	-0.43	-0.06
Rcl1	chr19	29217639	29218299	-0.94	-0.09

Cstf2t	chr19	31157419	31159177	-0.63	-0.03
Pdcd11	chr19	47165255	47167320	-1.04	-0.28
Pdcd11	chr19	47186632	47191796	-1.37	-0.10
Pdcd11	chr19	47203370	47203700	-2.30	-0.49
Sart1	chr19	5381212	5381668	-1.18	-0.21
Polr2g	chr19	8872986	8873047	-0.27	-0.01
Smndc1	chr19	53454959	53455050	-0.45	0.05
Gpkow	chrX	7285300	7285480	-1.43	0.37
Utp14a	chrX	45616799	45620147	-1.64	0.29
Cstf2	chrX	130609104	130609225	-1.51	0.37
Gemin8	chrX	162618285	162627552	-0.28	0.01
Zrsr2	chrX	160374633	160377244	-2.24	0.48
Zrsr2	chrX	160396327	160396568	-1.16	-0.25

Innate immune system

gene_name	chr	chromStart	chromEnd	diff.lg.p	diff.log2.fc
Casp8	chr1	58890568	58903481	-0.47	0.03
Abi2	chr1	60527596	60534214	-0.37	0.02
Creb1	chr1	64622786	64644320	-3.22	0.24
Slc11a1	chr1	74421776	74422412	-1.15	-0.25
Slc11a1	chr1	74432056	74432625	-0.60	0.06
Psmd1	chr1	87961193	87961313	-0.67	0.21
Arl8a	chr1	137043410	137043590	-2.90	-0.45
Ncf2	chr1	154654924	154658055	-0.49	0.03
Creg1	chr1	167693910	167694001	-1.58	-0.40
Cd247	chr1	167791873	167791964	-0.45	2.42
Fcgr4	chr1	172959267	172959892	-3.04	0.51
Tmem63a	chr1	182904309	182904638	-0.97	0.21
Tmbim1	chr1	74335150	74335301	-0.54	-0.09
Tmbim1	chr1	74341896	74350790	-2.54	-0.44
Bcl2	chr1	108438354	108439674	-0.39	0.03
Bcl2	chr1	108439763	108608408	-1.52	0.53
Bcl2	chr1	108608737	108610867	-0.32	0.01
Ptpn4	chr1	121555598	121572143	-1.58	0.18
Ptpn4	chr1	121733527	121733648	-0.29	-0.05
Actr3	chr1	127331893	127332265	-1.67	-0.16
Ikbke	chr1	133151208	133153534	-0.51	-0.04
Ikbke	chr1	133173083	133173488	-3.28	-0.78
Dhx9	chr1	155303276	155303397	-0.99	0.27
Qsox1	chr1	157626418	157628411	-1.52	-0.23
Fcgr2b	chr1	172890688	172891194	-2.46	0.13
Fcgr3	chr1	172981418	172981627	-0.40	0.03
Ncstn	chr1	173996440	173996917	-1.85	-0.17
Degs1	chr1	184206857	184208760	-0.92	0.08

Prkcq	chr2	11221863	11221954	-0.17	-0.59
Commd3	chr2	18596461	18597638	-0.89	0.08
Abl1	chr2	31544075	31551320	-0.40	0.02
Abl1	chr2	31652573	31658159	-1.65	-0.10
Abl1	chr2	31658367	31658428	-1.12	-0.61
Abl1	chr2	31659237	31659477	-0.56	0.58
Ptges2	chr2	32257756	32257994	-1.02	-0.29
Tank	chr2	61416642	61431225	-1.19	-0.33
Tank	chr2	61482412	61484260	-0.36	-0.03
Tank	chr2	61484409	61488438	-0.62	0.04
Tank	chr2	61491449	61491629	-0.49	0.06
Itgav	chr2	83643447	83643658	-1.94	-0.36
Itgav	chr2	83643837	83644077	-1.39	0.28
Psmc3	chr2	90894439	90895182	-1.38	-0.10
Traf6	chr2	101531587	101537929	-3.55	-0.22
B2m	chr2	121973422	121973632	-1.88	-0.08
Sirpa	chr2	129418574	129418963	-0.30	0.00
Pygb	chr2	150654298	150656555	-1.01	0.16
Hck	chr2	152934322	152934473	-1.33	0.47
Hck	chr2	152976937	152977028	-0.61	-0.13
Itch	chr2	154959216	154959307	-0.25	0.00
Itch	chr2	155050132	155050373	-0.80	-0.16
Src	chr2	157295226	157296471	-0.85	0.32
Plcg1	chr2	160587522	160588233	-1.55	-0.33
Mmp9	chr2	164779119	164780689	-0.74	0.61
Mmp9	chr2	164780747	164780808	-0.24	0.74
Dnajc5	chr2	181255239	181255330	-0.27	0.01
Abi1	chr2	22895609	22895730	-1.66	-0.37
Tubb4b	chr2	25077884	25079546	-0.33	0.00
Tubb4b	chr2	25079713	25080192	-0.31	-0.02
Dpp7	chr2	25207809	25207987	-0.69	0.11
Card9	chr2	26212789	26214357	-0.38	0.02
Agpat2	chr2	26449106	26450111	-0.33	-0.03
Surf4	chr2	26782443	26789001	-3.09	-0.35
Vav2	chr2	27282074	27282345	-0.49	-0.25
Psmd5	chr2	34707993	34709794	-1.37	-0.17
Psmd5	chr2	34722532	34726482	-1.60	-0.40
Psmb7	chr2	38498946	38499426	-1.35	-0.19
Wipf1	chr2	73270330	73270480	-3.23	-0.40
Wipf1	chr2	73273024	73278150	-0.91	-0.05
Wipf1	chr2	73282527	73291856	-2.22	0.32
Ptprj	chr2	90273202	90274160	-3.35	-0.26
Ptprj	chr2	90420474	90420774	-0.84	-0.21

Cat	chr2	103325161	103325280	-0.58	-0.15
Atp8b4	chr2	126148354	126148594	-0.43	0.05
Il1b	chr2	129195984	129196856	-1.18	3.00
Cd93	chr2	148266841	148266931	-0.14	0.32
Psmf1	chr2	151543111	151543232	-0.83	-0.47
Psmf1	chr2	151543649	151544068	-0.81	-0.19
Bcl2l1	chr2	152606972	152607272	-1.10	0.18
Bcl2l1	chr2	152656208	152657418	-1.01	-0.36
Cpne1	chr2	155922158	155937671	-0.37	-0.01
Dsn1	chr2	156820797	156820977	-0.49	0.26
Dsn1	chr2	156821482	156821990	-2.26	-0.39
Elmo2	chr2	165115147	165116717	-2.45	-0.22
Elmo2	chr2	165117554	165119793	-0.34	0.05
Nfatc2	chr2	168301939	168303199	-2.11	-0.21
Nfatc2	chr2	168304128	168330396	-0.75	0.06
Nfatc2	chr2	168396287	168427005	-2.25	-0.19
Fabp5	chr3	10012692	10015564	-0.91	0.09
Pld1	chr3	28029255	28031356	-0.46	0.06
Pld1	chr3	28031505	28031985	-0.33	-0.03
Pik3ca	chr3	32335072	32335402	-0.84	0.17
Pik3ca	chr3	32360681	32362022	-0.52	0.05
Rap2b	chr3	61168428	61170467	-0.56	0.02
Rap2b	chr3	61171065	61171426	-2.42	0.51
Shc1	chr3	89222472	89226252	-2.21	-0.23
Shc1	chr3	89232244	89232484	-0.63	-0.10
Ilf2	chr3	90291521	90291852	-0.76	-0.09
S100a8	chr3	90472992	90473549	-0.69	2.04
Ctss	chr3	95330707	95333452	-1.18	-0.07
Nras	chr3	102868375	102868495	-0.73	-0.10
Vav3	chr3	109143630	109143900	-2.32	-1.00
Ube2d3	chr3	135101656	135102228	-3.24	-0.27
Manba	chr3	135230647	135233888	-3.27	-0.25
Bcl10	chr3	145587232	145587592	-0.60	0.07
Bcl10	chr3	145588099	145593615	-2.28	0.24
Gm9733	chr3	15320566	15320714	-0.28	-0.19
Nhlrc3	chr3	53255917	53257492	-3.45	-0.39
Dhx36	chr3	62274511	62275295	-0.47	-0.07
Tlr2	chr3	83640193	83642691	-0.41	-0.01
Lamtor2	chr3	88353798	88356849	-1.88	-0.11
Psmd4	chr3	94836626	94836924	-1.79	-0.12
Psmd4	chr3	94839816	94846467	-2.16	-0.13
Nfkb1	chr3	135248217	135252299	-2.46	-0.20
Nfkb1	chr3	135354240	135354361	-0.32	0.21

Prkacb	chr3	146394723	146394993	-1.39	-0.21
Lyn	chr4	3605267	3605506	-3.28	-0.20
Map3k7	chr4	32051081	32051232	-2.61	-1.03
Map3k7	chr4	32109961	32110261	-0.43	0.08
Atp6v1g1	chr4	63205828	63209679	-1.23	0.19
Trim32	chr4	65274645	65276139	-0.35	0.01
Pgm2	chr4	99602055	99602234	-1.25	-0.43
Pgm2	chr4	99656703	99659542	-1.56	-0.30
Ptafr	chr4	132120011	132136781	-0.37	-0.01
Fgr	chr4	132530039	132542068	-1.56	0.56
Fuca1	chr4	135495027	135495355	-0.80	0.11
Ddost	chr4	137868046	137868376	-0.67	-0.07
Casp9	chr4	141349886	141352694	-2.10	-0.34
Casp9	chr4	141368153	141369672	-0.94	-0.31
Casp9	chr4	141369821	141369911	-0.41	-0.59
Ripk2	chr4	16050729	16051621	-2.11	-0.28
Ddx58	chr4	40152603	40155517	-1.64	0.25
B4galt1	chr4	40753786	40754830	-0.76	-0.05
B4galt1	chr4	40770567	40801001	-1.03	0.10
Vcp	chr4	43013259	43013349	-0.48	-0.16
Alad	chr4	62170203	62171204	-0.65	-0.14
Jun	chr4	94716322	94718913	-1.41	-0.04
Lck	chr4	129225797	129226067	-0.39	0.10
Cda	chr4	137894592	137894712	-1.83	1.47
Atad3a	chr4	155114748	155115286	-1.57	-0.13
Bst1	chr5	44210131	44210281	-0.56	-0.24
Cxcl3	chr5	91215188	91215429	-0.26	-0.14
Stbd1	chr5	93034080	93034170	-0.19	-0.81
Hvcn1	chr5	122690267	122692306	-0.59	-0.07
P2rx7	chr5	123131027	123132855	-0.67	0.05
P2rx7	chr5	123133154	123133964	-0.37	-0.02
Psmd9	chr5	123678228	123684616	-1.07	0.18
Atp6v0a2	chr5	125200361	125202517	-3.02	0.33
Tnip2	chr5	34839221	34839491	-0.36	0.04
Tlr1	chr5	65317328	65317538	-0.30	0.07
Tlr1	chr5	65318076	65318466	-0.27	-0.01
Tlr6	chr5	65344333	65346799	-3.02	0.30
Atp8a1	chr5	68013273	68013813	-0.67	0.11
Tec	chr5	73147021	73148633	-1.95	-0.24
Tec	chr5	73214842	73259687	-1.72	0.36
Mlec	chr5	115597834	115600228	-0.64	-0.05
Ptpn11	chr5	121581827	121582307	-2.13	0.25
Ptpn11	chr5	121583292	121587519	-0.51	-0.03

Rhof	chr5	123553648	123553977	-2.60	1.51
Rhof	chr5	123567408	123567856	-1.12	0.40
Rhof	chr5	123568123	123582429	-2.11	-0.12
Slc15a4	chr5	128076540	128080902	-0.77	-0.08
Ncf1	chr5	134705434	134705495	-1.15	-0.28
Trim56	chr5	137587155	137588025	-0.51	-0.04
Card11	chr5	141348952	141350390	-3.03	-0.50
Arpc4	chr6	113328136	113330743	-0.80	-0.14
Irak2	chr6	113588490	113588581	-0.26	0.00
Clec4d	chr6	123212183	123212244	-1.46	0.42
Lrmp	chr6	145070431	145070492	-1.05	0.44
Lrmp	chr6	145086263	145086444	-1.03	0.32
Lrmp	chr6	145123118	145123179	-0.82	-0.20
Impdh1	chr6	29159242	29166081	-0.29	0.00
Nod1	chr6	54874621	54874682	-0.58	2.04
Nod1	chr6	54893198	54893646	-1.73	2.07
Nod1	chr6	54894093	54894451	-0.13	0.08
Nod1	chr6	54894629	54894929	-0.30	0.33
Vamp8	chr6	72335214	72335506	-0.31	0.00
Vamp8	chr6	72338374	72340661	-0.33	-0.02
Kcmf1	chr6	72849172	72849233	-0.46	-0.84
Kcmf1	chr6	72849822	72849973	-0.55	-0.62
Rbsn	chr6	92139105	92140395	-0.37	0.02
Raf1	chr6	115568620	115569280	-2.59	-0.22
Alox5	chr6	116410986	116411196	-0.57	-0.13
Atp6v1e1	chr6	120768397	120772703	-0.46	-0.04
Slc2a3	chr6	122679681	122681413	-0.60	-0.09
C1s1	chr6	124480928	124481078	-0.13	0.19
C1s2	chr6	124575479	124575809	-0.34	1.58
Ptpn6	chr6	124683075	124688519	-0.61	-0.20
Olr1	chr6	129436944	129437814	-0.53	2.03
Kras	chr6	145168783	145169023	-0.29	0.00
Plaur	chr7	25257191	25257252	-0.42	-0.17
Tyrobp	chr7	31198806	31198896	-0.38	-0.01
Irf3	chr7	52253029	52253209	-0.82	-0.23
Irf3	chr7	52256298	52257801	-3.00	-0.31
Irf3	chr7	52257979	52258069	-0.96	-1.07
Cyfip1	chr7	63097490	63097551	-0.47	0.63
Cyfip1	chr7	63097610	63127288	-0.74	0.10
Ctsc	chr7	95437215	95437515	-0.81	0.31
Ctsc	chr7	95458037	95458487	-2.34	0.25
Prcp	chr7	100081654	100082343	-0.74	-0.10
Ndufc2	chr7	104548571	104548661	-1.22	-0.24

Lamtor1	chr7	109054380	109058211	-1.32	-0.28
Ampd3	chr7	117954502	117954682	-3.30	0.62
Ap2a2	chr7	148748108	148748169	-1.07	-0.80
Tarm1	chr7	3497555	3497675	-0.39	2.12
Lair1	chr7	3960490	3960699	-1.39	1.24
Lair1	chr7	3961115	3961205	-1.34	-1.04
C5ar2	chr7	16821937	16822178	-0.32	-0.19
C5ar2	chr7	16822506	16823255	-0.32	0.07
Calm3	chr7	17502039	17502309	-1.49	0.09
Relb	chr7	20191570	20192229	-0.55	-0.06
Ceacam1	chr7	26247739	26248608	-3.25	0.28
Ceacam1	chr7	26256924	26258919	-0.89	0.24
Ceacam2	chr7	26302704	26302765	-2.15	3.98
Psmd8	chr7	29965393	29965573	-1.95	-0.13
Siglece	chr7	50914788	50914849	-0.21	-0.02
Anpep	chr7	86966748	86966927	-1.32	0.20
Anpep	chr7	86986085	86993080	-2.81	-0.11
Iqgap1	chr7	87858508	87858659	-0.78	-0.04
Rhog	chr7	109387636	109398632	-1.64	-0.05
Trim21	chr7	109707270	109708500	-0.61	0.13
Aldoa	chr7	133943840	133943961	-0.40	-0.30
Adam8	chr7	147177314	147178387	-0.69	-0.11
Hras	chr7	148376639	148378177	-1.50	-0.13
Hras	chr7	148378835	148379754	-0.27	-0.01
Hras	chr7	148379812	148379903	-0.43	0.54
Irf7	chr7	148451539	148451718	-1.08	0.31
Fadd	chr7	151765572	151768281	-2.06	-0.21
Map2k7	chr8	4245576	4246251	-1.83	-0.18
Map2k7	chr8	4246579	4246760	-0.57	-0.12
Atp11a	chr8	12757015	12757255	-0.70	-0.56
Atp11a	chr8	12868009	12868130	-0.61	0.20
Plpp5	chr8	26834611	26834970	-2.39	-0.33
Ppp2cb	chr8	34710092	34710330	-0.74	-0.58
Myo9b	chr8	73796612	73839062	-1.76	-0.10
Myo9b	chr8	73857637	73857698	-0.67	0.13
Myo9b	chr8	73880502	73884611	-1.08	-0.05
Man2b1	chr8	87607167	87608319	-0.31	0.01
Cyld	chr8	91220956	91226218	-0.51	0.31
Nfatc3	chr8	108602571	108603618	-0.98	0.07
Nfatc3	chr8	108632435	108652166	-2.54	0.17
Plcg2	chr8	120022190	120028105	-0.40	0.04
Ikbkb	chr8	23770546	23770997	-1.89	-0.25
Golga7	chr8	24352846	24353057	-1.27	0.16

Hgsnat	chr8	27054960	27055615	-0.42	0.03
Hgsnat	chr8	27083350	27087156	-1.41	-0.31
Tlr3	chr8	46482457	46483076	-0.93	0.75
Tlr3	chr8	46483195	46483675	-0.31	-0.08
Tlr3	chr8	46483734	46484485	-0.49	0.27
Pik3r2	chr8	73292079	73292558	-1.14	-0.16
Bst2	chr8	74061276	74061336	-0.33	-0.04
Adgre5	chr8	86247268	86247687	-1.48	-0.25
Psmb10	chr8	108461631	108461997	-1.25	-0.38
Hp	chr8	112099087	112099834	-0.43	-0.11
Ist1	chr8	112195789	112196600	-2.35	0.15
Cotl1	chr8	122364299	122364479	-0.72	0.10
Galns	chr8	125102225	125103030	-0.47	-0.05
Galns	chr8	125135207	125135327	-1.24	0.83
Casp1	chr9	5298664	5299458	-2.63	0.43
Pin1	chr9	20456573	20459918	-2.86	-0.44
Slc44a2	chr9	21156935	21159352	-1.16	0.07
Dnm2	chr9	21310134	21311379	-1.31	0.09
Ppp2r1b	chr9	50687990	50688710	-0.88	-0.11
Ppp2r1b	chr9	50701016	50701705	-0.62	0.10
Psma4	chr9	54805658	54805748	-1.39	0.21
Pstpip1	chr9	55937782	55937962	-0.42	0.08
Adam10	chr9	70526807	70526988	-1.21	0.24
Adam10	chr9	70626056	70626297	-2.69	0.34
Rab27a	chr9	72892701	72908786	-0.51	-0.51
Rab27a	chr9	72943089	72943840	-1.02	-0.28
Myo5a	chr9	74919012	74919373	-0.83	-0.18
Myo5a	chr9	75071223	75071314	-0.37	0.07
Pik3r4	chr9	105545594	105545714	-0.79	-0.43
Pik3r4	chr9	105546492	105551208	-2.70	0.26
Pik3r4	chr9	105552647	105556389	-1.18	0.18
Pik3r4	chr9	105589445	105589955	-0.50	-0.07
Dusp7	chr9	106270962	106271201	-2.97	-1.90
Nckipsd	chr9	108714735	108714915	-1.02	-0.43
Nckipsd	chr9	108720039	108720159	-1.23	0.66
Glb1	chr9	114310242	114326114	-2.08	0.44
Glb1	chr9	114368770	114373569	-0.38	-0.02
Glb1	chr9	114374377	114383495	-3.66	0.26
Dync1li1	chr9	114632656	114632895	-3.05	0.38
Dync1li1	chr9	114632812	114633160	-1.11	0.11
Ccr2	chr9	124021097	124021397	-0.49	-0.23
Birc2	chr9	7818584	7818943	-0.91	0.25
Birc2	chr9	7834955	7835255	-3.06	0.75

Birc3	chr9	7848939	7850928	-0.49	0.05
Birc3	chr9	7860311	7872961	-0.93	0.08
Panx1	chr9	14810228	14814484	-1.07	-0.15
Rab3d	chr9	21714741	21715072	-1.15	-0.35
Tirap	chr9	34994095	34994305	-0.61	0.25
Tirap	chr9	34994573	34996823	-0.33	-0.01
Nlrx1	chr9	44060795	44061604	-1.41	-0.32
Nlrx1	chr9	44064475	44064566	-0.30	0.09
Nlrx1	chr9	44070436	44072329	-0.30	0.00
Tmem30a	chr9	79617076	79617495	-1.99	0.21
Pik3cb	chr9	98939628	98941447	-3.03	0.22
Armc8	chr9	99380819	99381090	-0.40	-0.09
Apeh	chr9	107987744	107987924	-0.34	-0.05
Myd88	chr9	119248949	119249158	-0.84	-0.19
Fuca2	chr10	13223007	13225667	-0.83	-0.15
Fuca2	chr10	13234515	13235470	-0.57	-0.03
Frk	chr10	34203714	34203804	-0.35	-0.86
Frk	chr10	34327775	34328313	-0.54	-2.10
Fyn	chr10	39089604	39089964	-1.17	-0.23
Fyn	chr10	39271197	39284162	-3.65	-0.24
Itgb2	chr10	76993092	77005145	-1.85	-0.24
Eef2	chr10	80644273	80645254	-0.46	0.00
Eea1	chr10	95488768	95489708	-1.82	-0.15
Eea1	chr10	95502273	95503004	-2.06	-0.20
Eea1	chr10	95504733	95504884	-0.95	-0.17
Cep290	chr10	100034744	100035631	-1.47	0.52
Gns	chr10	120802145	120802355	-0.43	0.05
Gns	chr10	120832206	120832746	-0.42	-0.01
Gns	chr10	120834001	120834301	-3.63	0.23
Arhgap9	chr10	126759938	126761407	-0.45	0.12
Stat6	chr10	127080041	127080222	-0.81	-0.13
Tab2	chr10	7625983	7626463	-1.95	0.18
Tab2	chr10	7626671	7644544	-2.25	0.07
Tnfaip3	chr10	18721581	18725137	-2.68	-0.19
Ube2d1	chr10	70718055	70718592	-2.86	0.34
Trpm2	chr10	77372593	77372804	-0.23	-0.16
Pfkl	chr10	77472360	77472511	-0.36	0.05
Ckap4	chr10	83990001	83991399	-2.44	-0.11
Ckap4	chr10	83996216	83996633	-1.45	-0.31
Glipr1	chr10	111434202	111434262	-0.61	0.14
Cand1	chr10	118647900	118648919	-0.55	0.03
Dbnl	chr11	5688485	5691845	-3.19	-0.45
Peli1	chr11	20991326	20991507	-0.82	-0.25

Psme4	chr11	30671774	30672135	-0.53	-0.17
Stk10	chr11	32504170	32512777	-2.97	-0.29
Fbxw11	chr11	32644536	32644986	-0.65	0.06
Mapk9	chr11	49660312	49660462	-0.47	0.14
Ppp2ca	chr11	51935449	51935836	-0.93	0.05
Gm2a	chr11	54923031	54923270	-0.59	0.07
2310033P09 Rik	chr11	59021980	59023882	-0.98	0.12
Nlrp3	chr11	59379889	59380129	-0.34	0.02
Map2k3	chr11	60765468	60766245	-1.76	-0.11
Pld2	chr11	70371013	70371612	-1.44	-0.29
Crk	chr11	75518934	75519324	-0.73	0.09
Nos2	chr11	78734360	78735737	-0.82	1.81
Psmd11	chr11	80285574	80285635	-1.26	-0.32
Mpo	chr11	87610892	87611072	-0.77	4.32
Psmd3	chr11	98556993	98557203	-2.02	-0.21
Atp6v0a1	chr11	100923774	100924043	-2.93	-0.36
Psme3	chr11	101177564	101178336	-0.77	0.09
Psme3	chr11	101182100	101183258	-2.86	-0.26
Baiap2	chr11	119862824	119863004	-2.19	0.86
Baiap2	chr11	119867797	119868096	-2.45	0.41
Nf2	chr11	4748990	4749290	-0.77	-0.14
Dock2	chr11	34127042	34128632	-0.56	-0.03
Dock2	chr11	34546949	34597325	-0.57	0.05
Cyfip2	chr11	46050850	46051031	-0.19	0.09
Cyfip2	chr11	46051359	46051599	-1.21	2.74
Atox1	chr11	55264107	55274640	-1.41	-0.17
Mapk7	chr11	61306963	61307471	-0.67	0.13
Nlrp1b	chr11	70966603	70969571	-1.97	-0.37
Nlrp1b	chr11	70995162	70995342	-0.91	-0.46
Nlrp1b	chr11	70996028	70996268	-0.26	0.01
Kpnb1	chr11	97049055	97049206	-3.64	-0.54
Ormdl3	chr11	98443291	98443381	-1.34	0.71
Ormdl3	chr11	98443469	98443619	-1.65	0.66
Ormdl3	chr11	98443767	98444472	-0.72	-0.16
Rab5c	chr11	100581475	100599444	-1.66	-0.43
Ghdc	chr11	100627734	100627853	-1.86	0.92
Ghdc	chr11	100630436	100630763	-2.36	0.63
Dusp3	chr11	101835527	101835885	-1.10	-0.08
Dusp3	chr11	101835944	101836213	-2.85	-0.24
Map3k14	chr11	103081077	103082247	-0.72	-0.12
Map3k14	chr11	103100286	103101085	-0.44	0.06
Map3k14	chr11	103103466	103128715	-0.61	-0.18

Cd300c2	chr11	114862804	114862865	-0.52	-0.36
Cd300e	chr11	114914519	114914670	-1.08	1.43
Grb2	chr11	115505517	115505906	-2.19	0.26
Grb2	chr11	115506561	115508033	-2.47	0.12
Tmc6	chr11	117627298	117628154	-0.39	-0.04
Cant1	chr11	118268378	118271504	-0.34	-0.01
Cant1	chr11	118272131	118279176	-1.88	-0.18
Cant1	chr11	118280220	118280281	-0.29	0.08
Psma6	chr12	56499811	56499900	-1.08	-0.18
Psmc1	chr12	101348377	101351316	-0.70	-0.11
Dync1h1	chr12	111839634	111839815	-1.16	-0.25
Dync1h1	chr12	111855934	111857506	-0.62	-0.02
Dync1h1	chr12	111904592	111905154	-2.05	0.14
Traf3	chr12	112499706	112500694	-0.68	-0.07
Pld4	chr12	113998865	113999998	-1.43	-0.19
Pld4	chr12	114000207	114001663	-0.42	0.03
Atp6v1d	chr12	79962474	79962535	-1.42	-0.42
Npc2	chr12	86097901	86101679	-1.04	0.04
Rps6ka5	chr12	101789211	101791305	-1.35	-0.27
Elmo1	chr13	20692056	20697406	-0.46	0.03
Ripk1	chr13	34119764	34125274	-2.04	-0.13
Ripk1	chr13	34125393	34126112	-1.53	-0.25
Dsp	chr13	38287718	38287838	-0.16	-0.42
Syk	chr13	52678805	52706692	-1.23	-0.09
Syk	chr13	52727039	52733575	-0.39	-0.02
Syk	chr13	52737300	52738635	-0.88	-0.13
Syk	chr13	52738964	52742902	-3.69	-0.15
Syk	chr13	52743800	52743951	-0.57	-0.11
Faf2	chr13	54723144	54730781	-1.07	0.44
Faf2	chr13	54757693	54763778	-2.34	-0.16
Lpcat1	chr13	73651326	73651986	-1.03	-0.22
Arsb	chr13	94710635	94710756	-0.54	-0.12
Cdk13	chr13	17809653	17811597	-3.39	0.17
Cdk13	chr13	17858328	17896931	-2.82	-0.14
Serpinb1a	chr13	32934139	32934200	-0.26	1.00
Txndc5	chr13	38592141	38592559	-3.04	0.23
Txndc5	chr13	38593006	38595325	-2.44	-0.12
Txndc5	chr13	38620243	38620304	-0.28	0.00
Hk3	chr13	55107346	55107674	-1.66	-0.13
Hk3	chr13	55122215	55122665	-0.41	-0.06
Rab24	chr13	55421264	55421501	-2.29	-0.29
Dok3	chr13	55628759	55629086	-1.35	-0.15
Ddx41	chr13	55637366	55637930	-0.30	0.01

Ctsl	chr13	64470393	64471614	-1.97	-0.09
Scamp1	chr13	94973830	94978130	-0.32	0.01
Pik3r1	chr13	102453956	102456264	-1.75	-0.18
Pik3r1	chr13	102478932	102525656	-1.06	0.31
Pik3r1	chr13	102527214	102538172	-0.33	-0.01
Map3k1	chr13	112545535	112546521	-0.47	-0.03
Map3k1	chr13	112566079	112599191	-0.72	-0.15
Nkiras1	chr14	19111014	19112745	-0.48	0.11
Vcl	chr14	21850890	21851250	-2.10	0.22
Psmc6	chr14	45949498	45950514	-0.58	0.08
Pnp	chr14	51571126	51571721	-1.77	0.17
Clu	chr14	66587437	66592220	-0.31	0.01
Clu	chr14	66594451	66594600	-0.64	0.12
Ppp3cb	chr14	21365614	21365735	-1.14	-0.58
Prkcd	chr14	31408808	31411699	-0.55	0.03
Prkcd	chr14	31411787	31411937	-0.29	-0.01
Tspan14	chr14	41720841	41721322	-0.75	-0.14
Myo10	chr15	25743096	25743426	-2.54	-0.31
Ncf4	chr15	78075240	78075447	-0.37	-0.03
Ncf4	chr15	78091438	78092951	-2.32	-0.15
Syngr1	chr15	79946545	79946695	-0.52	0.21
Ep300	chr15	81416643	81438767	-1.05	0.06
Ep300	chr15	81479273	81481819	-0.51	0.02
Xrcc6	chr15	81870184	81870514	-0.80	0.13
Ano6	chr15	95621273	95694678	-0.36	-0.03
Ano6	chr15	95802935	95805002	-0.88	-0.04
Atf1	chr15	100084825	100090754	-3.22	0.22
Mettl7a1	chr15	100143493	100143732	-0.42	0.13
Nckap1l	chr15	103328031	103328301	-2.08	-0.18
Ptk2	chr15	73035594	73039802	-0.41	0.02
Ptk2	chr15	73190599	73190660	-0.64	0.47
Dgat1	chr15	76342068	76342248	-0.53	0.16
Pla2g6	chr15	79116776	79117224	-0.88	-0.17
Nfam1	chr15	82828225	82828854	-2.38	-0.27
Nfam1	chr15	82829422	82831880	-1.58	-0.14
Mapk12	chr15	88961133	88961374	-0.45	2.32
Mapk11	chr15	88973270	88973361	-1.33	1.55
Mapk11	chr15	88973776	88973926	-0.54	0.57
Arsa	chr15	89303025	89303891	-1.14	0.17
Arsa	chr15	89305958	89306615	-3.10	-0.44
Gpr84	chr15	103138665	103140869	-0.35	-0.01
Hmox2	chr16	4764613	4765838	-2.38	-0.15
Mapk1	chr16	17038671	17038912	-0.29	0.00

Snap29	chr16	17427202	17428502	-0.76	0.12
Psmd2	chr16	20651724	20652324	-3.25	-0.17
Crebbp	chr16	4154961	4213404	-1.70	-0.10
Cpped1	chr16	11804530	11827393	-0.72	0.06
Cpped1	chr16	11828288	11884362	-3.35	0.51
Cpped1	chr16	11887807	11909516	-2.19	-0.53
Pak2	chr16	32019862	32021909	-1.43	0.09
Atp6v1a	chr16	44116916	44139132	-0.90	0.12
App	chr16	85025115	85043863	-0.75	-0.03
App	chr16	85043922	85056713	-2.06	-0.14
App	chr16	85120877	85173892	-2.41	-0.23
Cct8	chr16	87496053	87496114	-0.58	0.12
Rps6ka2	chr17	7374463	7374584	-0.75	1.07
Rps6ka2	chr17	7507034	7507424	-0.64	-0.40
Mapk14	chr17	28828286	28828675	-2.21	-0.37
Psmb8	chr17	34335288	34336214	-1.01	-0.16
Neu1	chr17	35071427	35072869	-3.32	-0.17
H2-D1	chr17	35403787	35404234	-3.70	-0.07
H2-Q6	chr17	35565949	35566720	-0.96	0.29
H2-Q7	chr17	35576925	35577015	-0.56	-1.32
H2-Q10	chr17	35607880	35610039	-0.32	0.07
Trem1	chr17	48371921	48385139	-2.64	1.06
Ypel5	chr17	73198138	73198199	-1.90	0.70
Ypel5	chr17	73198557	73198888	-0.34	0.02
Prkce	chr17	86567124	86568262	-1.54	-0.27
Prkce	chr17	87054027	87055374	-0.46	0.07
Dynlt1f	chr17	6306369	6306549	-0.33	-0.13
Igf2r	chr17	12876409	12876980	-1.19	-0.09
Igf2r	chr17	12962421	12962572	-0.60	0.17
Fpr1	chr17	18013552	18013905	-0.29	0.82
Fpr1	chr17	18014257	18014522	-0.62	3.00
Pdpk1	chr17	24215583	24216033	-2.93	0.32
Atp6v0c	chr17	24306196	24306374	-0.33	0.02
H2-K1	chr17	34136362	34136423	-0.41	0.19
Hspa1b	chr17	35093851	35095346	-0.55	0.06
Hspa1b	chr17	35095554	35096183	-2.02	0.44
Hspa1a	chr17	35107225	35107435	-1.72	-4.26
H2-T23	chr17	36169099	36248130	-0.77	-0.09
Enpp4	chr17	44234667	44234818	-0.51	0.64
Enpp4	chr17	44235056	44235147	-0.58	-1.68
Enpp4	chr17	44236285	44239353	-3.17	0.82
Ppp2r5d	chr17	46820028	46820862	-1.04	-0.11
Ppp2r5d	chr17	46837029	46841951	-0.31	0.02

Vapa	chr17	65962685	65962865	-2.27	-0.24
Rab31	chr17	66001065	66001632	-0.47	0.02
Rab31	chr17	66003418	66017287	-0.77	0.04
Rab31	chr17	66122031	66122092	-0.23	-0.05
Sos1	chr17	80797041	80799097	-0.34	-0.01
Sos1	chr17	80832979	80834320	-1.17	0.18
Sos1	chr17	80879313	80879554	-0.43	-0.33
Calm2	chr17	87835011	87846216	-3.10	-0.20
Ube2d2a	chr18	35931242	35931392	-1.92	-0.45
Ube2d2a	chr18	35965360	35965570	-1.78	0.47
Map3k8	chr18	4348929	4352951	-0.67	0.15
Rock1	chr18	10181251	10181790	-2.15	-0.22
Tmem173	chr18	35893749	35894288	-1.56	-0.19
Ticam2	chr18	46718782	46719052	-0.29	0.01
Nfatc1	chr18	80803872	80832687	-3.26	-0.24
Nfatc1	chr18	80846262	80846653	-1.93	0.30
Nfatc1	chr18	80879052	80904894	-0.33	-0.01
Unc93b1	chr19	3935215	3935335	-0.82	-0.10
Rela	chr19	5637578	5637639	-0.26	0.03
Pgam1	chr19	41986449	41990281	-2.13	-0.60
Btrc	chr19	45604207	45604268	-0.27	-0.42
Nfkb2	chr19	46379346	46380320	-1.80	0.46
Tcirg1	chr19	3906829	3907103	-1.92	-0.32
Aldh3b1	chr19	3913726	3913875	-2.12	-0.31
Tbc1d10c	chr19	4184415	4184833	-1.55	1.33
Peli3	chr19	4932182	4932600	-0.82	2.08
Dtx4	chr19	12543910	12544151	-1.35	-0.17
Chuk	chr19	44181846	44181937	-0.41	-0.11
Ddx3x	chrX	12858147	12861347	-0.80	0.06
Ikbkg	chrX	71675074	71678148	-0.70	-0.30
Tab3	chrX	82860462	82860673	-2.95	0.81
Tab3	chrX	82875864	82876014	-3.15	1.28
Was	chrX	7667496	7667617	-0.32	0.03
Cfp	chrX	20508559	20508650	-1.66	0.59
Rap2c	chrX	48359709	48370808	-2.32	0.21
Irak1	chrX	71260386	71261911	-0.47	0.03
Dnase1l1	chrX	71522609	71526996	-1.08	0.25
Magt1	chrX	103207027	103207148	-2.30	0.50
Gla	chrX	131135394	131135544	-0.33	0.04
Tlr8	chrX	163680663	163681289	-0.60	0.40
Tlr8	chrX	163681408	163681796	-2.38	1.46
Tlr8	chrX	163681884	163682362	-1.04	0.86
Tlr7	chrX	163745697	163746354	-3.29	0.14

Tnfrsf1b	chr4	144836594	144836774	-2.39	-0.60
Diaph1	chr18	38094885	38095065	-0.51	-0.12

Cytokine signaling in immune system

gene_name	chr	chromStart	chromEnd	diff.lg.p	diff.log2.fc
Il1r1	chr1	40370384	40370505	-0.22	1.00
Il1rl1	chr1	40518643	40519182	-0.86	-0.23
Il1rl1	chr1	40521392	40521602	-0.79	0.31
Il18rap	chr1	40605544	40606142	-0.34	-0.04
Stat1	chr1	52216218	52216729	-0.42	0.05
Creb1	chr1	64622786	64644320	-3.22	0.24
Psmd1	chr1	87961193	87961313	-0.67	0.21
Eif4e2	chr1	89134456	89135775	-0.30	0.00
Inpp5d	chr1	89516886	89517096	-2.34	-0.38
Inpp5d	chr1	89614406	89616844	-1.74	-0.09
Tnfrsf11a	chr1	107721612	107741653	-2.53	-0.18
Rnasel	chr1	155600002	155602337	-0.35	-0.01
Rnasel	chr1	155610184	155610334	-0.28	0.03
Sumo1	chr1	59696455	59696989	-1.11	-0.09
Dhx9	chr1	155303276	155303397	-0.99	0.27
Il15ra	chr2	11654930	11655499	-1.57	0.54
Il1rn	chr2	24205097	24205277	-0.26	0.00
Rapgef1	chr2	29475329	29475420	-0.29	-0.07
Rapgef1	chr2	29557821	29558001	-0.99	-0.14
Psmc3	chr2	90894439	90895182	-1.38	-0.10
Traf6	chr2	101531587	101537929	-3.55	-0.22
Hck	chr2	152934322	152934473	-1.33	0.47
Hck	chr2	152976937	152977028	-0.61	-0.13
Plcg1	chr2	160587522	160588233	-1.55	-0.33
Cd40	chr2	164896297	164896475	-0.35	0.15
Cd40	chr2	164896740	164896829	-0.20	0.00
Ptpn1	chr2	167757856	167758036	-0.92	0.24
Ptpn1	chr2	167800939	167802433	-0.91	0.11
Ptpn1	chr2	167802791	167803629	-1.01	0.16
Traf2	chr2	25374427	25375145	-1.34	-0.17
Psmd5	chr2	34707993	34709794	-1.37	-0.17
Psmd5	chr2	34722532	34726482	-1.60	-0.40
Psmb7	chr2	38498946	38499426	-1.35	-0.19
Il1a	chr2	129126416	129126536	-0.54	0.73
Il1b	chr2	129195984	129196856	-1.18	3.00
Psmf1	chr2	151543111	151543232	-0.83	-0.47
Psmf1	chr2	151543649	151544068	-0.81	-0.19
Pik3ca	chr3	32335072	32335402	-0.84	0.17
Pik3ca	chr3	32360681	32362022	-0.52	0.05

Shc1	chr3	89222472	89226252	-2.21	-0.23
Shc1	chr3	89232244	89232484	-0.63	-0.10
Il7	chr3	7573754	7573815	-0.33	-2.32
Il6ra	chr3	89673245	89675305	-2.03	-0.13
Psmd4	chr3	94836626	94836924	-1.79	-0.12
Psmd4	chr3	94839816	94846467	-2.16	-0.13
Hist2h3c2	chr3	96042454	96042544	-0.57	0.69
Csf1	chr3	107544354	107544444	-0.83	0.84
Csf1	chr3	107544832	107548597	-0.29	-0.01
Csf1	chr3	107548745	107550706	-1.24	-1.52
Csf1	chr3	107551063	107553281	-0.37	0.05
Nfkb1	chr3	135248217	135252299	-2.46	-0.20
Nfkb1	chr3	135354240	135354361	-0.32	0.21
Lyn	chr4	3605267	3605506	-3.28	-0.20
Map3k7	chr4	32051081	32051232	-2.61	-1.03
Map3k7	chr4	32109961	32110261	-0.43	0.08
Csf3r	chr4	125720761	125721238	-3.13	-0.56
Eif4g3	chr4	137676266	137682738	-0.34	0.00
Eif4g3	chr4	137702245	137707514	-0.39	-0.05
Eif4g3	chr4	137727076	137727166	-0.70	-0.10
Eif4g3	chr4	137762176	137762386	-2.48	0.37
Tnfrsf9	chr4	150302149	150307203	-1.72	0.87
Ripk2	chr4	16050729	16051621	-2.11	-0.28
Ddx58	chr4	40152603	40155517	-1.64	0.25
Ifnb1	chr4	88167987	88168343	-0.39	1.73
Ifnb1	chr4	88168549	88168698	-0.33	1.45
Jun	chr4	94716322	94718913	-1.41	-0.04
Jak1	chr4	100825596	100826046	-0.48	0.04
Lck	chr4	129225797	129226067	-0.39	0.10
Txlna	chr4	129311785	129317754	-0.43	-0.02
Pik3cd	chr4	149023545	149025880	-2.62	-0.17
Psmd9	chr5	123678228	123684616	-1.07	0.18
Tnip2	chr5	34839221	34839491	-0.36	0.04
Tec	chr5	73147021	73148633	-1.95	-0.24
Tec	chr5	73214842	73259687	-1.72	0.36
Ptpn11	chr5	121581827	121582307	-2.13	0.25
Ptpn11	chr5	121583292	121587519	-0.51	-0.03
Il17re	chr6	113420086	113420177	-0.14	0.22
Irak2	chr6	113588490	113588581	-0.26	0.00
Il17ra	chr6	120413214	120413455	-0.36	-0.04
Usp18	chr6	121195982	121196071	-0.23	-0.06
Gm8994	chr6	136278992	136279111	-0.18	0.47
Gm8994	chr6	136279792	136279971	-0.26	0.19

Nod1	chr6	54874621	54874682	-0.58	2.04
Nod1	chr6	54893198	54893646	-1.73	2.07
Nod1	chr6	54894093	54894451	-0.13	0.08
Nod1	chr6	54894629	54894929	-0.30	0.33
Ptpn6	chr6	124683075	124688519	-0.61	-0.20
Irf3	chr7	52253029	52253209	-0.82	-0.23
Irf3	chr7	52256298	52257801	-3.00	-0.31
Irf3	chr7	52257979	52258069	-0.96	-1.07
Il4ra	chr7	132718698	132720561	-0.42	-0.05
Il4ra	chr7	132720860	132721370	-2.99	0.66
Il21r	chr7	132776065	132776545	-0.24	0.08
Stx4a	chr7	134985321	134985978	-0.91	-0.11
Il11	chr7	4725025	4727615	-1.07	0.44
Il11	chr7	4727931	4729743	-0.36	0.10
Relb	chr7	20191570	20192229	-0.55	-0.06
Psmd8	chr7	29965393	29965573	-1.95	-0.13
Flt3l	chr7	52386558	52386918	-3.47	0.88
Inppl1	chr7	108971355	108974609	-1.09	-0.05
Inppl1	chr7	108985423	108986411	-0.97	-0.21
Inppl1	chr7	108986619	108986740	-1.28	1.00
Sh2b1	chr7	133610925	133611549	-0.93	-0.18
Sh2b1	chr7	133617103	133618554	-2.01	-0.54
Map2k7	chr8	4245576	4246251	-1.83	-0.18
Map2k7	chr8	4246579	4246760	-0.57	-0.12
Tnfsf13b	chr8	10035462	10035612	-0.36	0.13
Ppp2cb	chr8	34710092	34710330	-0.74	-0.58
Ikbkb	chr8	23770546	23770997	-1.89	-0.25
Pik3r2	chr8	73292079	73292558	-1.14	-0.16
Psmb10	chr8	108461631	108461997	-1.25	-0.38
Il34	chr8	113266054	113266576	-0.63	1.89
Il34	chr8	113279275	113279364	-0.18	0.74
Casp1	chr9	5298664	5299458	-2.63	0.43
Smarca4	chr9	21505619	21507475	-1.36	0.14
Ppp2r1b	chr9	50687990	50688710	-0.88	-0.11
Ppp2r1b	chr9	50701016	50701705	-0.62	0.10
Psma4	chr9	54805658	54805748	-1.39	0.21
Dusp7	chr9	106270962	106271201	-2.97	-1.90
Birc2	chr9	7818584	7818943	-0.91	0.25
Birc2	chr9	7834955	7835255	-3.06	0.75
Birc3	chr9	7848939	7850928	-0.49	0.05
Birc3	chr9	7860311	7872961	-0.93	0.08
Cbl	chr9	44041829	44042099	-2.62	-0.55
Il10ra	chr9	45062219	45062579	-0.35	-0.04

Il10ra	chr9	45063175	45068459	-1.09	0.10
Pias1	chr9	62729583	62729943	-0.64	0.13
Pik3cb	chr9	98939628	98941447	-3.03	0.22
Il20rb	chr9	100358465	100358556	-0.38	-1.51
Il20rb	chr9	100358644	100359660	-0.54	0.27
Myd88	chr9	119248949	119249158	-0.84	-0.19
Ifngr1	chr10	19329011	19329761	-1.01	-0.10
Fyn	chr10	39089604	39089964	-1.17	-0.23
Fyn	chr10	39271197	39284162	-3.65	-0.24
Stat6	chr10	127080041	127080222	-0.81	-0.13
Stat2	chr10	127727910	127728767	-0.32	-0.02
Tab2	chr10	7625983	7626463	-1.95	0.18
Tab2	chr10	7626671	7644544	-2.25	0.07
Irak3	chr10	119579518	119580238	-0.55	-0.07
Il23a	chr10	127733549	127733905	-0.33	-0.14
Peli1	chr11	20991326	20991507	-0.82	-0.25
Psme4	chr11	30671774	30672135	-0.53	-0.17
Fbxw11	chr11	32644536	32644986	-0.65	0.06
Mapk9	chr11	49660312	49660462	-0.47	0.14
Ppp2ca	chr11	51935449	51935836	-0.93	0.05
Map2k3	chr11	60765468	60766245	-1.76	-0.11
Vamp2	chr11	68902059	68902715	-0.32	0.03
Crk	chr11	75518934	75519324	-0.73	0.09
Psmd11	chr11	80285574	80285635	-1.26	-0.32
Trim25	chr11	88860716	88874870	-1.69	-0.06
Trim25	chr11	88877033	88878370	-0.70	-0.03
Psmd3	chr11	98556993	98557203	-2.02	-0.21
Stat5a	chr11	100721039	100722748	-0.67	-0.27
Psme3	chr11	101177564	101178336	-0.77	0.09
Psme3	chr11	101182100	101183258	-2.86	-0.26
Il9r	chr11	32090755	32090815	-0.27	-1.58
Csf2	chr11	54060859	54061185	-0.38	-1.06
Mapk7	chr11	61306963	61307471	-0.67	0.13
Dusp3	chr11	101835527	101835885	-1.10	-0.08
Dusp3	chr11	101835944	101836213	-2.85	-0.24
Map3k14	chr11	103081077	103082247	-0.72	-0.12
Map3k14	chr11	103100286	103101085	-0.44	0.06
Map3k14	chr11	103103466	103128715	-0.61	-0.18
Grb2	chr11	115505517	115505906	-2.19	0.26
Grb2	chr11	115506561	115508033	-2.47	0.12
Socs3	chr11	117830559	117830620	-0.38	0.48
P4hb	chr11	120422393	120423503	-0.94	-0.04
Sdc1	chr12	8778201	8778442	-1.77	-0.68

Sdc1	chr12	8798515	8799355	-0.52	-0.08
Psma6	chr12	56499811	56499900	-1.08	-0.18
Psmc1	chr12	101348377	101351316	-0.70	-0.11
Traf3	chr12	112499706	112500694	-0.68	-0.07
Sos2	chr12	70685584	70689696	-1.15	-0.08
Sos2	chr12	70782629	70782809	-1.89	-1.62
Rps6ka5	chr12	101789211	101791305	-1.35	-0.27
Hist1h3d	chr13	23667608	23667786	-1.85	-0.32
Syk	chr13	52678805	52706692	-1.23	-0.09
Syk	chr13	52727039	52733575	-0.39	-0.02
Syk	chr13	52737300	52738635	-0.88	-0.13
Syk	chr13	52738964	52742902	-3.69	-0.15
Syk	chr13	52743800	52743951	-0.57	-0.11
Il6st	chr13	113254277	113255963	-0.34	-0.03
Il6st	chr13	113294105	113296021	-1.16	0.06
Il6st	chr13	113296618	113296769	-0.32	-0.02
Hist1h3i	chr13	21875180	21875266	-1.25	-0.25
Hist1h3c	chr13	23837329	23837390	-0.45	-0.10
Pik3r1	chr13	102453956	102456264	-1.75	-0.18
Pik3r1	chr13	102478932	102525656	-1.06	0.31
Pik3r1	chr13	102527214	102538172	-0.33	-0.01
Flnb	chr14	8737140	8739780	-2.54	0.14
Flnb	chr14	8782901	8784071	-0.91	-0.06
Nkiras1	chr14	19111014	19112745	-0.48	0.11
Psmc6	chr14	45949498	45950514	-0.58	0.08
Irf9	chr14	56222821	56223430	-2.26	0.85
Irf9	chr14	56223549	56224030	-0.68	-0.24
Irf9	chr14	56227759	56228328	-3.64	0.43
Camk2g	chr14	21554813	21555948	-0.30	0.00
Prkcd	chr14	31408808	31411699	-0.55	0.03
Prkcd	chr14	31411787	31411937	-0.29	-0.01
Ptk2b	chr14	66772123	66773020	-1.61	-0.10
Ptk2b	chr14	66899768	66899829	-0.51	0.21
Csf2rb	chr15	78156427	78166798	-0.34	0.02
Rbx1	chr15	81305941	81306090	-0.83	-0.28
Atf1	chr15	100084825	100090754	-3.22	0.22
Il7r	chr15	9437501	9438102	-0.75	0.21
Ywhaz	chr15	36723405	36723964	-0.95	0.41
Csf2rb2	chr15	78114588	78116449	-1.22	-0.15
Mapk11	chr15	88973270	88973361	-1.33	1.55
Mapk11	chr15	88973776	88973926	-0.54	0.57
Mapk1	chr16	17038671	17038912	-0.29	0.00
Crkl	chr16	17452109	17484582	-0.30	0.00

Psmd2	chr16	20651724	20652324	-3.25	-0.17
Eif4g1	chr16	20679306	20680085	-1.42	0.04
Il1rap	chr16	26701407	26701468	-0.26	0.35
Il1rap	chr16	26714958	26715289	-1.23	0.49
Il10rb	chr16	91425363	91425662	-1.49	-0.16
Ifnar1	chr16	91506780	91506871	-0.57	0.18
Ifnar1	chr16	91507110	91507680	-1.67	0.21
Ifngr2	chr16	91563142	91563652	-1.48	-0.10
Socs1	chr16	10783960	10784764	-0.35	0.15
Socs1	chr16	10784822	10784882	-0.42	-1.26
Socs1	chr16	10784970	10785540	-0.17	-0.27
App	chr16	85025115	85043863	-0.75	-0.03
App	chr16	85043922	85056713	-2.06	-0.14
App	chr16	85120877	85173892	-2.41	-0.23
Brwd1	chr16	96223575	96225491	-0.35	0.01
Brwd1	chr16	96226300	96226615	-0.52	-1.11
Brwd1	chr16	96229480	96229661	-1.19	0.48
Rps6ka2	chr17	7374463	7374584	-0.75	1.07
Rps6ka2	chr17	7507034	7507424	-0.64	-0.40
Mapk14	chr17	28828286	28828675	-2.21	-0.37
Psmb8	chr17	34335288	34336214	-1.01	-0.16
Ebi3	chr17	56095665	56096445	-1.01	-0.13
Ppm1b	chr17	85357641	85364400	-0.47	0.19
Ppm1b	chr17	85393024	85402617	-0.51	0.03
Socs5	chr17	87507287	87507348	-0.71	-0.42
Socs5	chr17	87520106	87534893	-3.68	0.22
Ppp2r5d	chr17	46820028	46820862	-1.04	-0.11
Ppp2r5d	chr17	46837029	46841951	-0.31	0.02
Tnfsf14	chr17	57329333	57330291	-0.25	0.10
Eif2ak2	chr17	79252515	79254306	-1.48	0.13
Eif2ak2	chr17	79277576	79281912	-0.88	0.23
Sos1	chr17	80797041	80799097	-0.34	-0.01
Sos1	chr17	80832979	80834320	-1.17	0.18
Sos1	chr17	80879313	80879554	-0.43	-0.33
Camk2a	chr18	61144487	61145325	-0.34	-0.05
Csf1r	chr18	61265471	61269328	-0.53	0.02
Csf1r	chr18	61290403	61290793	-1.71	-0.05
Map3k8	chr18	4348929	4352951	-0.67	0.15
Rela	chr19	5637578	5637639	-0.26	0.03
Jak2	chr19	29385493	29386732	-1.43	0.23
Btrc	chr19	45604207	45604268	-0.27	-0.42
Nfkb2	chr19	46379346	46380320	-1.80	0.46
Peli3	chr19	4932182	4932600	-0.82	2.08

Stx3	chr19	11893622	11893893	-0.92	0.35
Chuk	chr19	44181846	44181937	-0.41	-0.11
Il13ra1	chrX	33695583	33709519	-2.22	0.35
Ikbkg	chrX	71675074	71678148	-0.70	-0.30
Tab3	chrX	82860462	82860673	-2.95	0.81
Tab3	chrX	82875864	82876014	-3.15	1.28
Irak1	chrX	71260386	71261911	-0.47	0.03
Eda2r	chrX	94533994	94534384	-0.94	0.65
Tnfrsf1b	chr4	144836594	144836774	-2.39	-0.60

mRNA splicing

gene_name	chr	chromStart	chromEnd	diff.lg.p	diff.log2.fc
Sf3b1	chr1	55084201	55084322	-0.54	-0.12
Dhx9	chr1	155303276	155303397	-0.99	0.27
Hnrnpu	chr1	180259373	180260299	-0.30	0.00
Cstf3	chr2	104504891	104505372	-2.37	0.28
Ctnnbl1	chr2	157563136	157600315	-3.07	-0.25
Cstf1	chr2	172201351	172201608	-2.58	0.38
Rbm17	chr2	11522350	11524826	-1.32	-0.27
Prpf40a	chr2	53049040	53050262	-0.84	0.13
Cwc22	chr2	77733949	77741886	-0.89	-0.07
Clp1	chr2	84563548	84566299	-2.19	-0.20
Snrpb	chr2	130002885	130005100	-2.05	-0.18
Rbm8a	chr3	96433850	96433910	-0.37	0.11
Bcas2	chr3	102982305	102982454	-2.42	-0.20
Prcc	chr3	87662854	87664130	-0.35	-0.02
Rnpc3	chr3	113332946	113333067	-0.48	-0.23
Srsf11	chr3	157674772	157675838	-1.15	-0.06
Prpf4	chr4	62083697	62085481	-1.56	-0.17
Dnajc8	chr4	132108975	132109154	-0.58	-0.05
Syf2	chr4	134486894	134490962	-2.04	-0.13
Syf2	chr4	134493303	134493452	-1.42	-0.35
Hnrnpr	chr4	135895368	135895996	-0.58	0.05
Ppih	chr4	118979838	118983372	-1.31	0.29
Srrm1	chr4	134878175	134878385	-0.88	-0.09
Srrm1	chr4	134902739	134909069	-1.22	0.14
Snrnp35	chr5	124933163	124941013	-1.28	0.15
Bud31	chr5	145908765	145908943	-2.11	-0.28
Hnrnpf	chr6	117850506	117856962	-2.04	-0.29
Hnrnpf	chr6	117873756	117874265	-1.34	-0.10
Gm8994	chr6	136278992	136279111	-0.18	0.47
Gm8994	chr6	136279792	136279971	-0.26	0.19
Hnrnpa2b1	chr6	51419743	51419893	-1.91	-0.18
Usp39	chr6	72268758	72269207	-1.04	0.14

Pcbp1	chr6	86474877	86476159	-0.78	-0.01
Isy1	chr6	87769010	87771528	-0.31	-0.01
Wbp11	chr6	136762473	136765520	-1.76	-0.10
Prpf31	chr7	3581586	3582460	-1.60	0.15
Snrpd2	chr7	19735186	19736653	-1.83	-0.27
Polr2i	chr7	31017332	31017772	-0.29	0.00
Snrpa1	chr7	73205399	73205549	-1.71	-0.33
Pcf11	chr7	99801691	99808521	-0.95	-0.05
Polr2l	chr7	148659279	148661052	-0.40	0.16
Sugp1	chr8	72566920	72572439	-0.33	-0.05
Polr2c	chr8	97387425	97387634	-0.99	-0.15
Elavl1	chr8	4289159	4289430	-1.24	-0.17
Dhx38	chr8	112071923	112073753	-2.72	0.29
Sf3b3	chr8	113334571	113334842	-1.95	0.11
Ppil4	chr10	7534486	7541105	-0.34	0.01
Sf3a2	chr10	80261479	80263960	-0.73	-0.20
Sf3a2	chr10	80266830	80266980	-0.97	-0.18
Ccar1	chr10	62208074	62209776	-1.59	-0.14
Polr2e	chr10	79498845	79498935	-0.48	-0.07
Polr2e	chr10	79501286	79502404	-0.85	-0.12
Sf3a1	chr11	4080296	4080657	-3.20	-0.37
Zmat5	chr11	4604673	4628589	-1.56	-0.46
Srsf1	chr11	87861172	87861322	-3.40	-0.49
Cwc25	chr11	97608042	97614001	-2.20	-0.20
Cpsf2	chr12	103214183	103214333	-1.55	0.42
Cpsf2	chr12	103228209	103228270	-1.83	0.39
Cpsf2	chr12	103240910	103242024	-2.01	0.13
Papola	chr12	107022971	107023122	-1.31	-0.13
Papola	chr12	107072945	107075506	-0.44	-0.02
Snw1	chr12	88791126	88791810	-2.89	-0.19
Snrnp48	chr13	38312637	38313246	-0.58	-0.09
Ddx46	chr13	55741151	55751787	-0.70	-0.04
Hnrnpk	chr13	58501349	58504674	-2.32	-0.21
Ppwd1	chr13	105018743	105018924	-1.46	0.46
Gtf2f2	chr14	76297012	76306651	-0.54	-0.05
Wbp4	chr14	79861569	79863783	-2.10	-0.28
Polr2k	chr15	36103771	36103949	-0.31	-0.05
Puf60	chr15	75900611	75900909	-0.96	-0.09
Ddx23	chr15	98488768	98493320	-1.34	-0.17
Ncbp2	chr16	31957240	31957450	-2.58	-0.37
Srrm2	chr17	23948991	23949082	-0.71	-0.24
Srrm2	chr17	23952780	23953201	-1.82	-0.06
Srrm2	chr17	23957516	23958116	-1.42	0.05

Rnps1	chr17	24551619	24552074	-1.68	-0.36
Hnrnpm	chr17	33814217	33822403	-1.25	-0.08
Cdc5l	chr17	45528987	45530123	-0.34	-0.01
Cdc5l	chr17	45564979	45570507	-0.86	-0.36
Gtf2f1	chr17	57143401	57143559	-0.53	-0.10
Gtf2f1	chr17	57143961	57144203	-0.51	-0.06
Srsf7	chr17	80606178	80606645	-0.76	-0.19
Polr2d	chr18	31955847	31956355	-3.30	0.43
Wdr33	chr18	32067351	32067412	-0.44	0.52
Txnl4a	chr18	80403987	80404903	-0.90	-0.14
Txnl4a	chr18	80415452	80419362	-1.31	-0.12
Txnl4a	chr18	80421065	80421873	-1.19	-0.41
Prpf19	chr19	10969781	10969902	-0.43	-0.06
Cstf2t	chr19	31157419	31159177	-0.63	-0.03
Sart1	chr19	5381212	5381668	-1.18	-0.21
Polr2g	chr19	8872986	8873047	-0.27	-0.01
Smndc1	chr19	53454959	53455050	-0.45	0.05
Gpkow	chrX	7285300	7285480	-1.43	0.37
Cstf2	chrX	130609104	130609225	-1.51	0.37
Zrsr2	chrX	160374633	160377244	-2.24	0.48
Zrsr2	chrX	160396327	160396568	-1.16	-0.25

Processing of capped intro-containing pre-mRNA

gene_name	chr	chromStart	chromEnd	diff.lg.p	diff.log2.fc
Sf3b1	chr1	55084201	55084322	-0.54	-0.12
Zc3h11a	chr1	135516447	135517433	-0.47	-0.02
Zc3h11a	chr1	135517641	135518733	-1.03	0.06
Zc3h11a	chr1	135521310	135523811	-2.56	0.12
Dhx9	chr1	155303276	155303397	-0.99	0.27
Hnrnpu	chr1	180259373	180260299	-0.30	0.00
Nup214	chr2	31888785	31893653	-1.32	0.09
Cstf3	chr2	104504891	104505372	-2.37	0.28
Ctnnbl1	chr2	157563136	157600315	-3.07	-0.25
Cstf1	chr2	172201351	172201608	-2.58	0.38
Rae1	chr2	172839488	172841203	-2.88	-0.32
Rbm17	chr2	11522350	11524826	-1.32	-0.27
Prpf40a	chr2	53049040	53050262	-0.84	0.13
Cwc22	chr2	77733949	77741886	-0.89	-0.07
Clp1	chr2	84563548	84566299	-2.19	-0.20
Snrpb	chr2	130002885	130005100	-2.05	-0.18
Rbm8a	chr3	96433850	96433910	-0.37	0.11
Bcas2	chr3	102982305	102982454	-2.42	-0.20
Prcc	chr3	87662854	87664130	-0.35	-0.02

Chtop	chr3	90303653	90304012	-2.00	-0.11
Chtop	chr3	90313185	90313365	-2.41	-0.21
Rnpc3	chr3	113332946	113333067	-0.48	-0.23
Srsf11	chr3	157674772	157675838	-1.15	-0.06
Prpf4	chr4	62083697	62085481	-1.56	-0.17
Ndc1	chr4	107083754	107086943	-0.44	0.04
Dnajc8	chr4	132108975	132109154	-0.58	-0.05
Syf2	chr4	134486894	134490962	-2.04	-0.13
Syf2	chr4	134493303	134493452	-1.42	-0.35
Hnrnpr	chr4	135895368	135895996	-0.58	0.05
Ppih	chr4	118979838	118983372	-1.31	0.29
Srrm1	chr4	134878175	134878385	-0.88	-0.09
Srrm1	chr4	134902739	134909069	-1.22	0.14
Nupl2	chr5	23687756	23687817	-0.27	0.02
Nupl2	chr5	23688026	23689736	-0.62	0.07
Snrnp35	chr5	124933163	124941013	-1.28	0.15
Bud31	chr5	145908765	145908943	-2.11	-0.28
Nup54	chr5	92845130	92846543	-1.56	0.19
Pom121	chr5	135853620	135857198	-0.30	0.00
Pom121	chr5	135857496	135857795	-0.92	0.12
Pom121	chr5	135869997	135870416	-0.68	-0.16
Nup205	chr6	35127615	35133895	-0.29	0.01
Hnrnpf	chr6	117850506	117856962	-2.04	-0.29
Hnrnpf	chr6	117873756	117874265	-1.34	-0.10
Gm8994	chr6	136278992	136279111	-0.18	0.47
Gm8994	chr6	136279792	136279971	-0.26	0.19
Hnrnpa2b1	chr6	51419743	51419893	-1.91	-0.18
Usp39	chr6	72268758	72269207	-1.04	0.14
Pcbp1	chr6	86474877	86476159	-0.78	-0.01
Isy1	chr6	87769010	87771528	-0.31	-0.01
Nup210	chr6	90963060	90963720	-3.45	-0.38
Wbp11	chr6	136762473	136765520	-1.76	-0.10
Prpf31	chr7	3581586	3582460	-1.60	0.15
Snrpd2	chr7	19735186	19736653	-1.83	-0.27
Polr2i	chr7	31017332	31017772	-0.29	0.00
Snrpa1	chr7	73205399	73205549	-1.71	-0.33
Pcf11	chr7	99801691	99808521	-0.95	-0.05
Nup98	chr7	109268003	109269291	-1.15	-0.06
Polr2l	chr7	148659279	148661052	-0.40	0.16
Sugp1	chr8	72566920	72572439	-0.33	-0.05
Polr2c	chr8	97387425	97387634	-0.99	-0.15
Elavl1	chr8	4289159	4289430	-1.24	-0.17
Dhx38	chr8	112071923	112073753	-2.72	0.29

Sf3b3	chr8	113334571	113334842	-1.95	0.11
Ppil4	chr10	7534486	7541105	-0.34	0.01
Sf3a2	chr10	80261479	80263960	-0.73	-0.20
Sf3a2	chr10	80266830	80266980	-0.97	-0.18
Ccar1	chr10	62208074	62209776	-1.59	-0.14
Polr2e	chr10	79498845	79498935	-0.48	-0.07
Polr2e	chr10	79501286	79502404	-0.85	-0.12
Sf3a1	chr11	4080296	4080657	-3.20	-0.37
Zmat5	chr11	4604673	4628589	-1.56	-0.46
Thoc5	chr11	4799846	4799907	-2.86	-0.63
Slu7	chr11	43247232	43251629	-0.59	0.07
Slu7	chr11	43258883	43259812	-2.51	-0.21
Srsf1	chr11	87861172	87861322	-3.40	-0.49
Casc3	chr11	98683710	98689970	-1.75	-0.13
Cwc25	chr11	97608042	97614001	-2.20	-0.20
Cpsf2	chr12	103214183	103214333	-1.55	0.42
Cpsf2	chr12	103228209	103228270	-1.83	0.39
Cpsf2	chr12	103240910	103242024	-2.01	0.13
Papola	chr12	107022971	107023122	-1.31	-0.13
Papola	chr12	107072945	107075506	-0.44	-0.02
Snw1	chr12	88791126	88791810	-2.89	-0.19
Snrnp48	chr13	38312637	38313246	-0.58	-0.09
Ddx46	chr13	55741151	55751787	-0.70	-0.04
Nup153	chr13	46776258	46776499	-0.69	-0.10
Nup153	chr13	46782010	46792068	-2.74	0.10
Thoc3	chr13	54561240	54561569	-1.64	-0.23
Hnrnpk	chr13	58501349	58504674	-2.32	-0.21
Ppwd1	chr13	105018743	105018924	-1.46	0.46
Gtf2f2	chr14	76297012	76306651	-0.54	-0.05
Wbp4	chr14	79861569	79863783	-2.10	-0.28
Polr2k	chr15	36103771	36103949	-0.31	-0.05
Nup50	chr15	84757805	84765852	-1.08	-0.07
Nup50	chr15	84773033	84773303	-0.62	0.12
Puf60	chr15	75900611	75900909	-0.96	-0.09
Poldip3	chr15	82958053	82959641	-2.39	-0.13
Poldip3	chr15	82968734	82968795	-0.70	0.11
Ddx23	chr15	98488768	98493320	-1.34	-0.17
Ncbp2	chr16	31957240	31957450	-2.58	-0.37
Fyttd1	chr16	32877869	32884216	-0.32	-0.02
Fyttd1	chr16	32902586	32905812	-0.49	-0.07
Srrm2	chr17	23948991	23949082	-0.71	-0.24
Srrm2	chr17	23952780	23953201	-1.82	-0.06
Srrm2	chr17	23957516	23958116	-1.42	0.05

Rnps1	chr17	24551619	24552074	-1.68	-0.36
Ddx39b	chr17	35378690	35378917	-2.25	-0.37
Ddx39b	chr17	35386162	35386790	-0.42	-0.02
Ddx39b	chr17	35566011	35566729	-0.95	0.28
Wtap	chr17	13167327	13168316	-1.35	0.15
Thoc6	chr17	23810618	23810831	-0.44	0.09
Hnrnpm	chr17	33814217	33822403	-1.25	-0.08
Cdc5l	chr17	45528987	45530123	-0.34	-0.01
Cdc5l	chr17	45564979	45570507	-0.86	-0.36
Gtf2f1	chr17	57143401	57143559	-0.53	-0.10
Gtf2f1	chr17	57143961	57144203	-0.51	-0.06
Srsf7	chr17	80606178	80606645	-0.76	-0.19
Thoc1	chr18	9993386	9993507	-0.34	0.02
Polr2d	chr18	31955847	31956355	-3.30	0.43
Wdr33	chr18	32067351	32067412	-0.44	0.52
Txnl4a	chr18	80403987	80404903	-0.90	-0.14
Txnl4a	chr18	80415452	80419362	-1.31	-0.12
Txnl4a	chr18	80421065	80421873	-1.19	-0.41
Prpf19	chr19	10969781	10969902	-0.43	-0.06
Cstf2t	chr19	31157419	31159177	-0.63	-0.03
Sart1	chr19	5381212	5381668	-1.18	-0.21
Polr2g	chr19	8872986	8873047	-0.27	-0.01
Smndc1	chr19	53454959	53455050	-0.45	0.05
Gpkow	chrX	7285300	7285480	-1.43	0.37
Cstf2	chrX	130609104	130609225	-1.51	0.37
Zrsr2	chrX	160374633	160377244	-2.24	0.48
Zrsr2	chrX	160396327	160396568	-1.16	-0.25

Class I MHC mediated antigen processing & presentation

gene_name	chr	chromStart	chromEnd	diff.lg.p	diff.log2.fc
Psmd1	chr1	87961193	87961313	-0.67	0.21
Ube2f	chr1	93182063	93182512	-2.14	-0.16
Asb1	chr1	93437351	93443741	-2.00	0.50
Asb1	chr1	93448832	93448983	-0.89	0.38
Asb1	chr1	93451373	93451764	-0.41	0.07
Ncf2	chr1	154654924	154658055	-0.49	0.03
Hecw2	chr1	53869734	53883756	-1.12	2.77
Hecw2	chr1	53982435	53983513	-0.26	-0.03
Cul3	chr1	80336885	80337005	-1.62	-0.38
Trip12	chr1	84721087	84721998	-1.08	0.10
Trip12	chr1	84782695	84836815	-1.84	0.07
Klhl20	chr1	163019047	163019258	-1.39	-0.43
Klhl20	chr1	163039327	163060580	-0.32	0.01
Anapc2	chr2	25128463	25128941	-0.88	-0.09

Fbxw5	chr2	25358791	25359805	-1.19	0.14
Fbxw5	chr2	25360013	25360452	-1.24	-0.22
Fbxw5	chr2	25360840	25360960	-3.19	1.04
Hspa5	chr2	34630750	34631930	-2.17	-0.03
Klhl41	chr2	69508654	69508893	-1.32	0.66
Itgav	chr2	83643447	83643658	-1.94	-0.36
Itgav	chr2	83643837	83644077	-1.39	0.28
Psmc3	chr2	90894439	90895182	-1.38	-0.10
B2m	chr2	121973422	121973632	-1.88	-0.08
Trim69	chr2	121993421	121993481	-0.14	0.42
Trim69	chr2	122004286	122004614	-0.57	-1.61
Itch	chr2	154959216	154959307	-0.25	0.00
Itch	chr2	155050132	155050373	-0.80	-0.16
Rnf114	chr2	167318293	167325329	-0.28	-0.05
Lrsam1	chr2	32781993	32782383	-0.65	-0.16
Fbxw2	chr2	34661048	34661869	-2.12	-0.20
Fbxw2	chr2	34662764	34664040	-0.46	-0.08
Fbxw2	chr2	34668436	34678672	-1.12	-0.10
Fbxw2	chr2	34681468	34681725	-0.58	0.19
Psmd5	chr2	34707993	34709794	-1.37	-0.17
Psmd5	chr2	34722532	34726482	-1.60	-0.40
Psmb7	chr2	38498946	38499426	-1.35	-0.19
Ubox5	chr2	130416966	130417566	-1.43	-0.36
Ubox5	chr2	130425325	130426405	-0.55	0.08
Psmf1	chr2	151543111	151543232	-0.83	-0.47
Psmf1	chr2	151543649	151544068	-0.81	-0.19
Fbxw7	chr3	84619498	84694070	-1.92	0.38
Fbxw7	chr3	84694129	84756226	-2.05	0.20
Fbxw7	chr3	84780140	84781955	-0.37	0.02
Ube2q1	chr3	89577530	89577890	-3.20	-0.36
Ube2q1	chr3	89585705	89587919	-0.89	-0.04
Rnf115	chr3	96592580	96593978	-1.30	-0.15
Sec22b	chr3	97725140	97725438	-2.99	-0.26
Ube2d3	chr3	135101656	135102228	-3.24	-0.27
Siah2	chr3	58495620	58495891	-1.37	0.55
Psmd4	chr3	94836626	94836924	-1.79	-0.12
Psmd4	chr3	94839816	94846467	-2.16	-0.13
Sec24b	chr3	129686102	129687101	-0.83	-0.07
Fbxl4	chr4	22303713	22313319	-1.70	0.24
Fbxl4	chr4	22354425	22354486	-0.94	0.57
Ube2j1	chr4	33136832	33136892	-1.32	-0.32
Ube2r2	chr4	41137777	41138136	-1.17	0.13
Trim32	chr4	65274645	65276139	-0.35	0.01

Lrrc41	chr4	115761466	115762183	-1.09	-0.08
Lrrc41	chr4	115768961	115769260	-0.52	0.05
Fbxo2	chr4	147538344	147538434	-0.25	-0.78
Klhl21	chr4	151383089	151389959	-0.56	0.05
Klhl21	chr4	151390287	151390408	-0.53	0.22
Ufl1	chr4	25177588	25177709	-0.34	0.05
Fbxo10	chr4	45048105	45048374	-0.47	0.54
Fbxo10	chr4	45048433	45048494	-0.73	1.50
Fbxo10	chr4	45048553	45048793	-0.74	0.68
Fbxo10	chr4	45071323	45071922	-0.48	0.30
Cdc26	chr4	62055609	62055998	-2.00	0.59
Rnf220	chr4	116958526	116968779	-3.32	0.35
Cdc20	chr4	118109887	118109948	-0.55	-0.37
Fbxo6	chr4	147520063	147520213	-1.03	0.25
Spsb1	chr4	149271827	149281337	-1.02	0.57
Mib2	chr4	155028580	155028761	-0.30	-0.09
Ube3c	chr5	29895781	29895962	-1.72	-1.14
Ube3c	chr5	30001057	30001268	-0.46	0.07
Anapc4	chr5	53257704	53257973	-2.14	-0.29
Klhl5	chr5	65558211	65558721	-1.61	0.23
Ube3b	chr5	114830734	114830795	-0.28	0.61
Ube3b	chr5	114869593	114869952	-0.38	0.03
Fbxo21	chr5	118426838	118426928	-0.57	0.34
Fbxo21	chr5	118452063	118458648	-0.32	0.01
Anapc7	chr5	122891328	122891538	-0.52	-0.09
Anapc7	chr5	122893875	122894502	-3.47	-0.38
Rnf34	chr5	123273869	123314504	-1.72	0.21
Psmd9	chr5	123678228	123684616	-1.07	0.18
Kctd7	chr5	130628247	130628815	-1.33	-0.62
Fbxl5	chr5	44142150	44149098	-2.30	-0.48
Fbxl5	chr5	44149396	44151973	-0.42	-0.02
Rchy1	chr5	92391795	92392064	-0.36	0.05
Sec31a	chr5	100790877	100791058	-0.38	0.02
Fbxw8	chr5	118515497	118515797	-0.35	-0.06
Ncf1	chr5	134705434	134705495	-1.15	-0.28
Fbxl18	chr5	143633766	143634186	-0.39	0.07
Fbxl18	chr5	143640382	143644224	-0.93	-0.38
Fbxl18	chr5	143650503	143656917	-0.38	-0.27
Smurf1	chr5	145637693	145638023	-0.74	-0.29
Smurf1	chr5	145638352	145638682	-1.09	-0.34
Smurf1	chr5	145639310	145639491	-0.37	0.13
Rnf6	chr5	147021822	147022181	-2.92	0.33
Znrf2	chr6	54838508	54838629	-0.37	-0.02

Herc6	chr6	57445639	57531182	-0.37	-0.17
Kbtbd8	chr6	95071961	95077327	-2.16	0.32
Vhl	chr6	113578147	113579978	-1.16	0.21
Fbxl14	chr6	119429685	119431451	-2.73	-0.15
Klhl42	chr6	147039984	147056970	-0.73	-0.10
Ube2h	chr6	30163827	30164395	-0.64	0.08
Ube2h	chr6	30164514	30164664	-1.04	0.40
Ube2h	chr6	30164783	30164993	-0.55	0.22
Vamp8	chr6	72335214	72335506	-0.31	0.00
Vamp8	chr6	72338374	72340661	-0.33	-0.02
Herc2	chr7	63485102	63486928	-1.77	0.13
Ube3a	chr7	66484119	66496353	-2.58	0.28
Klhl25	chr7	82993342	83012197	-1.00	0.11
Klhl25	chr7	83012375	83018299	-0.87	-0.23
Klhl25	chr7	83018716	83018926	-0.35	-0.11
Rbbp6	chr7	130114077	130114438	-1.49	-0.14
Rbbp6	chr7	130133665	130133786	-0.82	-0.15
Rbbp6	chr7	130140585	130140795	-0.65	0.08
Fbxl19	chr7	134890288	134891744	-2.81	-0.80
Fbxl19	chr7	134911014	134911877	-0.34	-0.02
Stx4a	chr7	134985321	134985978	-0.91	-0.11
Psmd8	chr7	29965393	29965573	-1.95	-0.13
Asb7	chr7	73792504	73823980	-0.99	-0.14
Asb7	chr7	73834117	73834387	-0.57	0.21
Det1	chr7	85972688	85972809	-1.48	-0.65
Det1	chr7	85988116	85988866	-0.31	-0.01
Trim21	chr7	109707270	109708500	-0.61	0.13
Cdc16	chr8	13779233	13781763	-0.76	0.08
Sh3rf1	chr8	63704632	63808042	-0.71	0.21
Sh3rf1	chr8	63863616	63863797	-0.45	-0.26
Sh3rf1	chr8	63865414	63873071	-0.33	-0.05
Fbxl8	chr8	107791922	107793078	-0.54	-0.13
Znrf1	chr8	114060539	114062246	-2.99	0.21
Znrf1	chr8	114146249	114147028	-0.58	0.04
Gan	chr8	119682094	119707502	-2.38	0.66
Lonrf1	chr8	37280137	37282962	-0.64	0.13
Lonrf1	chr8	37311699	37312090	-1.27	-0.69
Klhl2	chr8	67219421	67219870	-3.53	-0.50
Calr	chr8	87366315	87366923	-2.76	-0.06
Calr	chr8	87369960	87370800	-0.94	0.04
Siah1a	chr8	89248756	89269816	-1.08	-0.12
Psmb10	chr8	108461631	108461997	-1.25	-0.38
Fbxo31	chr8	124075878	124076237	-3.34	0.63

Psma4	chr9	54805658	54805748	-1.39	0.21
Fbxo22	chr9	55066270	55071672	-2.03	0.17
Fbxo22	chr9	55071880	55072031	-0.93	-0.28
Anapc13	chr9	102528625	102536370	-0.48	0.06
Dcaf1	chr9	106738991	106740852	-3.06	0.55
Dcaf1	chr9	106760607	106761940	-3.49	0.37
Fbxl12	chr9	20448850	20449181	-0.31	-0.05
Keap1	chr9	21034503	21038410	-0.91	0.07
Keap1	chr9	21041585	21042276	-1.22	-0.20
Keap1	chr9	21043025	21043776	-0.79	-0.29
Ube4a	chr9	44731568	44732078	-2.10	0.26
Ube4a	chr9	44756010	44756191	-0.52	-0.14
Cul5	chr9	53425530	53426100	-1.13	0.12
Rnf111	chr9	70274585	70277390	-1.13	-0.14
Fbxo9	chr9	77943491	77943611	-0.30	0.14
Arih2	chr9	108507307	108507428	-0.63	0.11
Arih2	chr9	108522434	108547287	-1.35	0.23
Ube2g2	chr10	77107280	77107637	-0.93	-0.11
Ube2g2	chr10	77107992	77108141	-1.22	0.25
Fbxo7	chr10	85487309	85487369	-0.37	0.10
Rnf217	chr10	31254643	31329531	-1.58	-0.29
Ube2d1	chr10	70718055	70718592	-2.86	0.34
Rnf126	chr10	79221259	79223642	-1.89	-0.11
Rnf126	chr10	79229537	79229686	-1.44	-0.51
Fzr1	chr10	80829682	80830187	-1.59	0.17
Psme4	chr11	30671774	30672135	-0.53	-0.17
Fbxw11	chr11	32644536	32644986	-0.65	0.06
Rnf130	chr11	49933551	49933881	-0.41	0.38
Trim11	chr11	58791617	58791916	-0.34	-0.02
Trim11	chr11	58802336	58802515	-1.26	0.40
Trim11	chr11	58802842	58804960	-1.02	0.09
Ube2g1	chr11	72420792	72421062	-2.86	-0.55
Ube2g1	chr11	72497025	72497295	-0.29	0.00
Psmd11	chr11	80285574	80285635	-1.26	-0.32
Psmd3	chr11	98556993	98557203	-2.02	-0.21
Psme3	chr11	101177564	101178336	-0.77	0.09
Psme3	chr11	101182100	101183258	-2.86	-0.26
Trim41	chr11	48625890	48630474	-2.56	-0.19
Trim41	chr11	48630623	48630893	-2.44	0.54
Sec24a	chr11	51556955	51570200	-1.50	-0.11
Wsb1	chr11	79053599	79053898	-2.67	-0.17
Ube2z	chr11	95911464	95911704	-2.06	-0.26
Fbxl20	chr11	97944196	97944257	-0.53	0.44

Klhl11	chr11	100323925	100325737	-2.89	0.19
Klhl11	chr11	100333857	100334096	-0.43	-0.10
Cdc27	chr11	104366205	104368561	-0.85	-0.06
Smurf2	chr11	106683743	106684358	-1.06	-0.24
Smurf2	chr11	106781579	106781849	-2.81	-0.99
Ube2o	chr11	116399633	116399902	-0.30	0.01
Ube2o	chr11	116400140	116404120	-2.64	-0.17
Ube2o	chr11	116405875	116406025	-0.84	0.21
Ube2o	chr11	116442611	116442731	-0.48	-0.68
Socs3	chr11	117830559	117830620	-0.38	0.48
Psma6	chr12	56499811	56499900	-1.08	-0.18
Lrr1	chr12	70275442	70276002	-1.46	-0.50
Psmc1	chr12	101348377	101351316	-0.70	-0.11
Arel1	chr12	86259725	86261648	-0.82	0.05
Arel1	chr12	86311686	86311836	-0.96	0.38
Mylip	chr13	45503815	45506325	-3.05	0.42
Rnf144b	chr13	47338305	47340700	-0.89	-0.14
Rnf144b	chr13	47342821	47343181	-1.25	0.41
Erap1	chr13	74777319	74783985	-2.40	0.26
Kctd6	chr14	9052713	9055605	-1.86	-0.26
Sec24c	chr14	21512398	21513205	-0.75	-0.07
Sec24c	chr14	21513563	21514072	-1.48	-0.13
Psmc6	chr14	45949498	45950514	-0.58	0.08
Kbtbd7	chr14	79827126	79828716	-1.70	0.29
Fbxl3	chr14	103482090	103488631	-0.81	-0.05
Fbxl3	chr14	103494436	103498143	-0.54	-0.05
Fbxl3	chr14	103498202	103498726	-1.82	-0.43
Ncf4	chr15	78075240	78075447	-0.37	-0.03
Ncf4	chr15	78091438	78092951	-2.32	-0.15
Rbx1	chr15	81305941	81306090	-0.83	-0.28
Skp2	chr15	9043329	9043540	-0.80	0.27
Skp2	chr15	9069207	9070167	-0.68	-0.14
Rnf19a	chr15	36170946	36173866	-0.40	0.03
Fbxo32	chr15	58011872	58012083	-0.36	-0.16
Fbxo32	chr15	58012410	58013069	-1.46	-0.50
Fbxo32	chr15	58046223	58046433	-2.16	-1.12
Asb8	chr15	97965939	97971774	-0.39	0.02
Mgrn1	chr16	4936923	4937342	-0.36	0.02
Klhl22	chr16	17771956	17777358	-1.71	-0.20
Klhl22	chr16	17778345	17778466	-0.64	0.80
Psmd2	chr16	20651724	20652324	-3.25	-0.17
Itgb5	chr16	33829750	33830049	-3.12	-1.09
Itgb5	chr16	33919922	33920251	-0.33	-0.01

Itgb5	chr16	33947843	33949275	-0.39	-0.02
Cblb	chr16	52166229	52166380	-0.94	0.44
Cblb	chr16	52204565	52205105	-0.38	-0.05
Socs1	chr16	10783960	10784764	-0.35	0.15
Socs1	chr16	10784822	10784882	-0.42	-1.26
Socs1	chr16	10784970	10785540	-0.17	-0.27
Dtx3l	chr16	35929143	35932046	-2.43	0.35
Dtx3l	chr16	35932375	35933483	-0.33	0.01
Ltn1	chr16	87415807	87416587	-2.10	0.18
Tap1	chr17	34324500	34325281	-0.31	0.01
Tap1	chr17	34333539	34333870	-2.43	0.51
Psmb8	chr17	34335288	34336214	-1.01	-0.16
Tap2	chr17	34341423	34342567	-0.60	-0.10
Tap2	chr17	34352342	34353206	-0.31	-0.01
H2-D1	chr17	35403787	35404234	-3.70	-0.07
H2-Q6	chr17	35565949	35566720	-0.96	0.29
H2-Q7	chr17	35576925	35577015	-0.56	-1.32
H2-Q10	chr17	35607880	35610039	-0.32	0.07
Cul7	chr17	46787586	46787707	-0.19	0.03
Cul7	chr17	46788456	46789468	-0.30	-0.09
Ccnf	chr17	24360773	24360864	-1.19	-0.44
Stub1	chr17	25967580	25967909	-2.17	-0.16
Stub1	chr17	25969947	25970306	-0.38	0.08
H2-K1	chr17	34136362	34136423	-0.41	0.19
H2-T23	chr17	36169099	36248130	-0.77	-0.09
Trim39	chr17	36397225	36398520	-1.76	0.23
Trim39	chr17	36400794	36404080	-1.08	-0.35
Ubr2	chr17	47065869	47067399	-0.68	-0.05
Ubr2	chr17	47147240	47147481	-0.50	-0.08
Pja2	chr17	64658035	64660528	-0.54	-0.04
Fbxo11	chr17	88390945	88391656	-0.52	0.03
Ube2d2a	chr18	35931242	35931392	-1.92	-0.45
Ube2d2a	chr18	35965360	35965570	-1.78	0.47
Rnf14	chr18	38467720	38467990	-0.45	0.07
Mex3c	chr18	73732718	73749293	-0.62	-0.08
Cdc23	chr18	34792931	34793291	-2.74	-0.56
Cdc23	chr18	34793470	34793621	-0.73	-0.23
Trim36	chr18	46326929	46328816	-0.84	0.34
Btrc	chr19	45604207	45604268	-0.27	-0.42
Fbxl15	chr19	46403619	46404936	-0.68	0.11
Fbxw4	chr19	45652836	45653256	-0.43	-0.03
Ube2a	chrX	34414360	34414479	-0.33	-0.03
Pja1	chrX	96661669	96661909	-3.01	0.59

Pja1	chrX	96662087	96663849	-0.78	0.06
Siah1b	chrX	160509579	160510081	-0.42	0.07

Chromatin modifying enzymes

gene_name	chr	chromStart	chromEnd	diff.lg.p	diff.log2.fc
Ing5	chr1	95714871	95715861	-2.79	-0.31
Rbbp5	chr1	134394590	134400982	-1.17	0.15
Rbbp5	chr1	134401131	134401312	-0.34	-0.03
Kdm5b	chr1	136514466	136514617	-0.68	0.28
Kdm5b	chr1	136521310	136521760	-2.33	0.45
Kdm5b	chr1	136525288	136527418	-0.37	-0.03
Kdm5b	chr1	136527777	136528227	-1.23	0.27
Ncoa2	chr1	13132595	13132776	-0.46	0.11
Ncoa2	chr1	13157056	13167163	-1.03	-0.09
Kansl3	chr1	36392574	36393263	-0.43	-0.03
Smyd3	chr1	181325678	181325888	-0.28	-0.02
Smyd3	chr1	181447984	181448134	-0.55	-0.18
Smyd2	chr1	191704578	191706000	-1.27	0.17
Phf20	chr2	156092990	156093201	-1.93	0.33
Phf20	chr2	156113573	156113634	-1.16	0.44
Suv39h2	chr2	3379850	3382179	-1.47	0.27
Suv39h2	chr2	3392047	3392258	-0.50	-0.25
Ehmt1	chr2	24646742	24647852	-1.08	-0.13
Ehmt1	chr2	24733063	24775080	-2.58	0.23
Tbl1xr1	chr3	22108509	22109805	-2.53	0.23
Jade1	chr3	41408645	41418364	-2.31	-0.21
Ash1l	chr3	88878647	88881260	-0.39	0.02
Gatad2b	chr3	90127990	90145879	-0.41	-0.02
Gatad2b	chr3	90155787	90155878	-3.11	-0.35
Hist2h2be	chr3	96025043	96025490	-1.83	0.52
Hist2h2be	chr3	96026738	96027215	-1.30	-0.39
Setd7	chr3	51354172	51364745	-2.74	0.39
Setdb1	chr3	95127865	95128046	-2.40	0.40
Setdb1	chr3	95130188	95132554	-2.35	0.17
Setdb1	chr3	95141164	95142873	-1.65	0.15
Hist2h3c2	chr3	96042454	96042544	-0.57	0.69
Hist2h2aa1	chr3	96044071	96044216	-1.04	1.17
Prmt6	chr3	110050616	110051517	-1.67	-0.58
Prmt6	chr3	110052356	110053916	-3.07	-0.18
Nfkb1	chr3	135248217	135252299	-2.46	-0.20
Nfkb1	chr3	135354240	135354361	-0.32	0.21
Kdm4a	chr4	117809906	117810206	-0.99	-0.17
Kdm1a	chr4	136106537	136106746	-0.82	0.18
Padi3	chr4	140342091	140342272	-0.87	2.25

Kmt2e	chr5	22940246	22940516	-2.82	0.51
Kmt2e	chr5	23002909	23005273	-0.76	0.07
Kmt2e	chr5	23007381	23008999	-1.16	0.08
Nsd2	chr5	34164323	34188853	-0.53	-0.04
Nsd2	chr5	34197314	34203748	-2.63	-0.16
Rest	chr5	77694578	77697550	-0.95	-0.10
Rest	chr5	77709822	77712154	-0.92	-0.05
Kmt5a	chr5	124911176	124911327	-0.89	-0.12
Smarcd3	chr5	24100691	24101019	-0.21	0.00
Kmt2c	chr5	24790108	24790979	-0.87	0.09
Suds3	chr5	117542584	117542913	-1.68	-0.19
Suds3	chr5	117558367	117565972	-1.81	-0.18
Kdm2b	chr5	123321373	123321823	-0.49	0.10
Kdm2b	chr5	123330401	123330851	-1.94	-0.30
Kdm2b	chr5	123371426	123382872	-2.79	-0.66
Ncor2	chr5	125514394	125514836	-0.46	-0.06
Ncor2	chr5	125538274	125548306	-0.47	0.04
Ncor2	chr5	125659463	125659584	-0.26	-0.17
Brpf1	chr6	113257160	113269353	-0.42	0.01
Kdm5a	chr6	120314116	120314686	-0.31	0.01
Kdm5a	chr6	120369521	120377827	-0.72	0.07
Kdm5a	chr6	120380152	120389497	-1.27	0.10
Chd4	chr6	125079888	125080399	-1.62	-0.07
Atf7ip	chr6	136508513	136509952	-0.34	-0.01
Atf7ip	chr6	136535654	136536194	-2.27	0.23
Atf7ip	chr6	136555030	136555660	-0.29	0.00
H2afj	chr6	136756828	136756889	-0.45	0.13
H2afj	chr6	136757307	136757577	-1.31	-0.28
Aebp2	chr6	140571543	140572383	-1.35	-0.28
Aebp2	chr6	140572622	140573517	-2.75	-0.28
Aebp2	chr6	140602621	140602921	-1.72	0.28
Kdm7a	chr6	39092697	39096881	-0.49	-0.03
Kdm3a	chr6	71539383	71540465	-0.70	0.15
Kdm3a	chr6	71553894	71554104	-1.83	0.48
Kdm3a	chr6	71557565	71562002	-0.92	-0.13
Kmt5c	chr7	4697803	4698966	-2.61	-0.27
Prmt3	chr7	57112887	57113008	-3.64	-0.59
Kdm8	chr7	132595743	132595893	-0.94	0.69
Setd1a	chr7	134920932	134920993	-1.26	1.23
Setd1a	chr7	134927635	134930705	-0.77	0.04
Setd1a	chr7	134931750	134931998	-1.29	-0.20
Kat8	chr7	135056030	135056181	-1.44	-0.62
Kmt2b	chr7	31368475	31373655	-3.36	-0.13

Eed	chr7	97125526	97129068	-0.79	-0.09
Eed	chr7	97129246	97129456	-2.79	0.48
Ccnd1	chr7	152118441	152118622	-0.78	-0.05
Ccnd1	chr7	152125529	152125830	-0.84	0.07
Kat6a	chr8	24040729	24043035	-1.41	0.13
Kat6a	chr8	24046098	24051460	-0.39	-0.01
Nsd3	chr8	26751014	26760027	-1.48	-0.09
Nsd3	chr8	26770213	26773719	-2.35	0.14
Nsd3	chr8	26787194	26787674	-0.37	0.03
Ash2l	chr8	26950536	26958082	-3.54	-0.43
Gatad2a	chr8	72459689	72459840	-1.48	-0.16
Gatad2a	chr8	72497999	72498150	-0.82	-0.30
Gatad2a	chr8	72519948	72520248	-0.90	-0.33
Carm1	chr9	21392340	21393190	-2.42	-0.14
Smarca4	chr9	21505619	21507475	-1.36	0.14
H2afx	chr9	44143446	44144156	-2.11	-0.11
Kdm4d	chr9	14268006	14268096	-0.37	2.17
Kdm4d	chr9	14268363	14268602	-1.25	3.93
Kmt2a	chr9	44615605	44617182	-1.02	0.14
Kmt2a	chr9	44650695	44650756	-3.48	0.90
Dot1l	chr10	80217950	80218131	-1.54	-0.50
Dot1l	chr10	80254516	80254817	-3.01	-0.75
Dot1l	chr10	80255475	80255566	-0.30	-0.05
Smarcc2	chr10	127920231	127921148	-2.34	0.28
Smarcc2	chr10	127924513	127924663	-1.10	-0.30
Smarcc2	chr10	127926093	127926303	-3.40	0.46
Arid5b	chr10	67558669	67564649	-1.89	0.12
Smarcb1	chr10	75359661	75359780	-2.54	-0.51
Hmg20b	chr10	80811711	80811891	-0.44	-0.05
Hmg20b	chr10	80812098	80812308	-2.27	0.42
Hmg20b	chr10	80812873	80813172	-1.33	-0.37
Sap30l	chr11	57615138	57619548	-2.36	-0.43
Sap30l	chr11	57621553	57623760	-1.20	-0.20
Hist3h2a	chr11	58768691	58768899	-0.65	0.26
Suz12	chr11	79843530	79845978	-3.16	0.17
Msl1	chr11	98657082	98662282	-2.35	-0.12
Jade2	chr11	51627973	51629289	-2.12	0.16
Jade2	chr11	51629617	51629797	-0.73	0.23
Jade2	chr11	51629856	51641772	-2.27	-0.13
Jade2	chr11	51668400	51668461	-0.31	0.05
Ncor1	chr11	62147216	62147367	-0.76	0.12
Ncor1	chr11	62182894	62186997	-0.89	-0.06
Ncor1	chr11	62196726	62198338	-2.00	-0.18

Kat7	chr11	95171349	95171530	-1.84	-0.29
Kansl1	chr11	104196220	104196852	-0.42	-0.03
Kansl1	chr11	104285114	104303314	-2.66	0.11
Kansl1	chr11	104303433	104303584	-0.48	-0.17
Smarcd2	chr11	106133897	106134286	-1.61	-0.41
Jmjd6	chr11	116698745	116700329	-0.89	-0.11
Jmjd6	chr11	116702283	116702521	-2.40	0.43
Jmjd6	chr11	116703705	116704733	-0.68	-0.06
Dnmt3a	chr12	3806582	3866198	-1.98	-0.23
Dnmt3a	chr12	3906804	3908006	-2.27	-0.13
Arid4a	chr12	72116983	72117769	-1.20	0.41
Rcor1	chr12	112348059	112350521	-0.33	-0.01
Ncoa1	chr12	4247570	4248468	-0.70	-0.10
Ncoa1	chr12	4248766	4252834	-0.39	-0.03
Ncoa1	chr12	4267709	4267949	-1.13	0.26
Ncoa1	chr12	4294980	4296117	-2.77	0.24
Setd3	chr12	109345414	109346800	-3.71	0.28
Arid4b	chr13	14156058	14156209	-0.85	0.32
Hist1h2bg	chr13	23663268	23663474	-3.01	-0.19
Hist1h3d	chr13	23667608	23667786	-1.85	-0.32
Hist1h2bc	chr13	23776067	23776213	-0.42	-0.04
Hist1h3i	chr13	21875180	21875266	-1.25	-0.25
Hist1h3c	chr13	23837329	23837390	-0.45	-0.10
Phf2	chr13	48898195	48899711	-1.75	-0.24
Phf2	chr13	48911913	48913690	-0.46	0.06
Kat6b	chr14	22320299	22336775	-1.19	0.20
Kat6b	chr14	22481439	22481650	-1.92	0.83
Kat6b	chr14	22483908	22490307	-0.84	0.08
Prmt5	chr14	55126018	55126553	-1.21	-0.15
Ep300	chr15	81416643	81438767	-1.05	0.06
Ep300	chr15	81479273	81481819	-0.51	0.02
Arid2	chr15	96117982	96118043	-0.41	-0.14
Arid2	chr15	96199968	96201115	-1.06	0.08
Arid2	chr15	96201684	96209296	-1.71	0.10
Arid2	chr15	96223066	96232775	-2.27	0.36
Brd1	chr15	88543110	88543809	-2.54	0.27
Kansl2	chr15	98349531	98349829	-0.50	-0.10
Riox2	chr16	59471749	59472290	-2.24	0.66
Riox2	chr16	59475394	59475664	-0.80	0.16
Brwd1	chr16	96223575	96225491	-0.35	0.01
Brwd1	chr16	96226300	96226615	-0.52	-1.11
Brwd1	chr16	96229480	96229661	-1.19	0.48
Arid1b	chr17	5332607	5339263	-2.33	-0.16

Brpf3	chr17	28938130	28938251	-0.19	0.10
Brpf3	chr17	28954078	28960704	-1.08	0.19
Brpf3	chr17	28973638	28973848	-0.68	0.35
Ehmt2	chr17	35042089	35042587	-2.96	-0.29
Ehmt2	chr17	35049712	35050992	-3.05	-0.21
Kdm3b	chr18	34964262	34970049	-2.47	-0.14
Kdm3b	chr18	34997287	34998126	-1.40	-0.14
Hdac3	chr18	38096953	38097223	-2.39	-0.32
Kmt5b	chr19	3767600	3767721	-0.43	0.32
Kmt5b	chr19	3807383	3807444	-0.32	-0.05
Kmt5b	chr19	3808821	3816385	-2.74	-0.16
Brms1	chr19	5040306	5040901	-2.26	-0.29
Brms1	chr19	5041286	5046457	-2.05	-0.19
Rela	chr19	5637578	5637639	-0.26	0.03
Smarca2	chr19	26705264	26705505	-0.28	0.00
Nfkb2	chr19	46379346	46380320	-1.80	0.46
Kdm2a	chr19	4316774	4316954	-0.33	-0.02
Kdm2a	chr19	4318778	4320217	-0.49	0.03
Kdm2a	chr19	4322495	4326851	-1.52	0.12
Kdm2a	chr19	4396332	4396782	-1.56	-0.22
Kdm6a	chrX	17827922	17831812	-3.44	0.29
Kdm6a	chrX	17855854	17856005	-2.03	0.69
Jade3	chrX	20094125	20094634	-1.90	0.45
Ogt	chrX	98874640	98878285	-0.71	0.04
Ogt	chrX	98878792	98878973	-1.90	-0.42
Ogt	chrX	98879151	98879242	-0.52	-0.19
Kdm5c	chrX	148667772	148667893	-0.85	0.26
Kdm5c	chrX	148705250	148708150	-0.85	0.04
Suv39h1	chrX	7647858	7649530	-0.54	0.06
Hcfc1	chrX	71194680	71196000	-1.71	-0.08
Hcfc1	chrX	71211533	71211654	-0.84	0.13
Kdm5d	chrY	277702	278241	-2.00	0.29

Chromatin organization

gene_name	chr	chromStart	chromEnd	diff.lg.p	diff.log2.fc
Ing5	chr1	95714871	95715861	-2.79	-0.31
Rbbp5	chr1	134394590	134400982	-1.17	0.15
Rbbp5	chr1	134401131	134401312	-0.34	-0.03
Kdm5b	chr1	136514466	136514617	-0.68	0.28
Kdm5b	chr1	136521310	136521760	-2.33	0.45
Kdm5b	chr1	136525288	136527418	-0.37	-0.03
Kdm5b	chr1	136527777	136528227	-1.23	0.27
Ncoa2	chr1	13132595	13132776	-0.46	0.11
Ncoa2	chr1	13157056	13167163	-1.03	-0.09

Kansl3	chr1	36392574	36393263	-0.43	-0.03
Smyd3	chr1	181325678	181325888	-0.28	-0.02
Smyd3	chr1	181447984	181448134	-0.55	-0.18
Smyd2	chr1	191704578	191706000	-1.27	0.17
Phf20	chr2	156092990	156093201	-1.93	0.33
Phf20	chr2	156113573	156113634	-1.16	0.44
Suv39h2	chr2	3379850	3382179	-1.47	0.27
Suv39h2	chr2	3392047	3392258	-0.50	-0.25
Ehmt1	chr2	24646742	24647852	-1.08	-0.13
Ehmt1	chr2	24733063	24775080	-2.58	0.23
Tbl1xr1	chr3	22108509	22109805	-2.53	0.23
Jade1	chr3	41408645	41418364	-2.31	-0.21
Ash1l	chr3	88878647	88881260	-0.39	0.02
Gatad2b	chr3	90127990	90145879	-0.41	-0.02
Gatad2b	chr3	90155787	90155878	-3.11	-0.35
Hist2h2be	chr3	96025043	96025490	-1.83	0.52
Hist2h2be	chr3	96026738	96027215	-1.30	-0.39
Setd7	chr3	51354172	51364745	-2.74	0.39
Setdb1	chr3	95127865	95128046	-2.40	0.40
Setdb1	chr3	95130188	95132554	-2.35	0.17
Setdb1	chr3	95141164	95142873	-1.65	0.15
Hist2h3c2	chr3	96042454	96042544	-0.57	0.69
Hist2h2aa1	chr3	96044071	96044216	-1.04	1.17
Prmt6	chr3	110050616	110051517	-1.67	-0.58
Prmt6	chr3	110052356	110053916	-3.07	-0.18
Nfkb1	chr3	135248217	135252299	-2.46	-0.20
Nfkb1	chr3	135354240	135354361	-0.32	0.21
Kdm4a	chr4	117809906	117810206	-0.99	-0.17
Kdm1a	chr4	136106537	136106746	-0.82	0.18
Padi3	chr4	140342091	140342272	-0.87	2.25
Kmt2e	chr5	22940246	22940516	-2.82	0.51
Kmt2e	chr5	23002909	23005273	-0.76	0.07
Kmt2e	chr5	23007381	23008999	-1.16	0.08
Nsd2	chr5	34164323	34188853	-0.53	-0.04
Nsd2	chr5	34197314	34203748	-2.63	-0.16
Rest	chr5	77694578	77697550	-0.95	-0.10
Rest	chr5	77709822	77712154	-0.92	-0.05
Kmt5a	chr5	124911176	124911327	-0.89	-0.12
Smarcd3	chr5	24100691	24101019	-0.21	0.00
Kmt2c	chr5	24790108	24790979	-0.87	0.09
Suds3	chr5	117542584	117542913	-1.68	-0.19
Suds3	chr5	117558367	117565972	-1.81	-0.18
Kdm2b	chr5	123321373	123321823	-0.49	0.10

Kdm2b	chr5	123330401	123330851	-1.94	-0.30
Kdm2b	chr5	123371426	123382872	-2.79	-0.66
Ncor2	chr5	125514394	125514836	-0.46	-0.06
Ncor2	chr5	125538274	125548306	-0.47	0.04
Ncor2	chr5	125659463	125659584	-0.26	-0.17
Brpf1	chr6	113257160	113269353	-0.42	0.01
Kdm5a	chr6	120314116	120314686	-0.31	0.01
Kdm5a	chr6	120369521	120377827	-0.72	0.07
Kdm5a	chr6	120380152	120389497	-1.27	0.10
Chd4	chr6	125079888	125080399	-1.62	-0.07
Atf7ip	chr6	136508513	136509952	-0.34	-0.01
Atf7ip	chr6	136535654	136536194	-2.27	0.23
Atf7ip	chr6	136555030	136555660	-0.29	0.00
H2afj	chr6	136756828	136756889	-0.45	0.13
H2afj	chr6	136757307	136757577	-1.31	-0.28
Aebp2	chr6	140571543	140572383	-1.35	-0.28
Aebp2	chr6	140572622	140573517	-2.75	-0.28
Aebp2	chr6	140602621	140602921	-1.72	0.28
Kdm7a	chr6	39092697	39096881	-0.49	-0.03
Kdm3a	chr6	71539383	71540465	-0.70	0.15
Kdm3a	chr6	71553894	71554104	-1.83	0.48
Kdm3a	chr6	71557565	71562002	-0.92	-0.13
Kmt5c	chr7	4697803	4698966	-2.61	-0.27
Prmt3	chr7	57112887	57113008	-3.64	-0.59
Kdm8	chr7	132595743	132595893	-0.94	0.69
Setd1a	chr7	134920932	134920993	-1.26	1.23
Setd1a	chr7	134927635	134930705	-0.77	0.04
Setd1a	chr7	134931750	134931998	-1.29	-0.20
Kat8	chr7	135056030	135056181	-1.44	-0.62
Kmt2b	chr7	31368475	31373655	-3.36	-0.13
Eed	chr7	97125526	97129068	-0.79	-0.09
Eed	chr7	97129246	97129456	-2.79	0.48
Ccnd1	chr7	152118441	152118622	-0.78	-0.05
Ccnd1	chr7	152125529	152125830	-0.84	0.07
Kat6a	chr8	24040729	24043035	-1.41	0.13
Kat6a	chr8	24046098	24051460	-0.39	-0.01
Nsd3	chr8	26751014	26760027	-1.48	-0.09
Nsd3	chr8	26770213	26773719	-2.35	0.14
Nsd3	chr8	26787194	26787674	-0.37	0.03
Ash2l	chr8	26950536	26958082	-3.54	-0.43
Gatad2a	chr8	72459689	72459840	-1.48	-0.16
Gatad2a	chr8	72497999	72498150	-0.82	-0.30
Gatad2a	chr8	72519948	72520248	-0.90	-0.33

Carm1	chr9	21392340	21393190	-2.42	-0.14
Smarca4	chr9	21505619	21507475	-1.36	0.14
H2afx	chr9	44143446	44144156	-2.11	-0.11
Kdm4d	chr9	14268006	14268096	-0.37	2.17
Kdm4d	chr9	14268363	14268602	-1.25	3.93
Kmt2a	chr9	44615605	44617182	-1.02	0.14
Kmt2a	chr9	44650695	44650756	-3.48	0.90
Dot1l	chr10	80217950	80218131	-1.54	-0.50
Dot1l	chr10	80254516	80254817	-3.01	-0.75
Dot1l	chr10	80255475	80255566	-0.30	-0.05
Smarcc2	chr10	127920231	127921148	-2.34	0.28
Smarcc2	chr10	127924513	127924663	-1.10	-0.30
Smarcc2	chr10	127926093	127926303	-3.40	0.46
Arid5b	chr10	67558669	67564649	-1.89	0.12
Smarcb1	chr10	75359661	75359780	-2.54	-0.51
Hmg20b	chr10	80811711	80811891	-0.44	-0.05
Hmg20b	chr10	80812098	80812308	-2.27	0.42
Hmg20b	chr10	80812873	80813172	-1.33	-0.37
Sap30l	chr11	57615138	57619548	-2.36	-0.43
Sap30l	chr11	57621553	57623760	-1.20	-0.20
Hist3h2a	chr11	58768691	58768899	-0.65	0.26
Suz12	chr11	79843530	79845978	-3.16	0.17
Msl1	chr11	98657082	98662282	-2.35	-0.12
Jade2	chr11	51627973	51629289	-2.12	0.16
Jade2	chr11	51629617	51629797	-0.73	0.23
Jade2	chr11	51629856	51641772	-2.27	-0.13
Jade2	chr11	51668400	51668461	-0.31	0.05
Ncor1	chr11	62147216	62147367	-0.76	0.12
Ncor1	chr11	62182894	62186997	-0.89	-0.06
Ncor1	chr11	62196726	62198338	-2.00	-0.18
Kat7	chr11	95171349	95171530	-1.84	-0.29
Kansl1	chr11	104196220	104196852	-0.42	-0.03
Kansl1	chr11	104285114	104303314	-2.66	0.11
Kansl1	chr11	104303433	104303584	-0.48	-0.17
Smarcd2	chr11	106133897	106134286	-1.61	-0.41
Jmjd6	chr11	116698745	116700329	-0.89	-0.11
Jmjd6	chr11	116702283	116702521	-2.40	0.43
Jmjd6	chr11	116703705	116704733	-0.68	-0.06
Dnmt3a	chr12	3806582	3866198	-1.98	-0.23
Dnmt3a	chr12	3906804	3908006	-2.27	-0.13
Arid4a	chr12	72116983	72117769	-1.20	0.41
Rcor1	chr12	112348059	112350521	-0.33	-0.01
Ncoa1	chr12	4247570	4248468	-0.70	-0.10

Ncoa1	chr12	4248766	4252834	-0.39	-0.03
Ncoa1	chr12	4267709	4267949	-1.13	0.26
Ncoa1	chr12	4294980	4296117	-2.77	0.24
Setd3	chr12	109345414	109346800	-3.71	0.28
Arid4b	chr13	14156058	14156209	-0.85	0.32
Hist1h2bg	chr13	23663268	23663474	-3.01	-0.19
Hist1h3d	chr13	23667608	23667786	-1.85	-0.32
Hist1h2bc	chr13	23776067	23776213	-0.42	-0.04
Hist1h3i	chr13	21875180	21875266	-1.25	-0.25
Hist1h3c	chr13	23837329	23837390	-0.45	-0.10
Phf2	chr13	48898195	48899711	-1.75	-0.24
Phf2	chr13	48911913	48913690	-0.46	0.06
Kat6b	chr14	22320299	22336775	-1.19	0.20
Kat6b	chr14	22481439	22481650	-1.92	0.83
Kat6b	chr14	22483908	22490307	-0.84	0.08
Prmt5	chr14	55126018	55126553	-1.21	-0.15
Ep300	chr15	81416643	81438767	-1.05	0.06
Ep300	chr15	81479273	81481819	-0.51	0.02
Arid2	chr15	96117982	96118043	-0.41	-0.14
Arid2	chr15	96199968	96201115	-1.06	0.08
Arid2	chr15	96201684	96209296	-1.71	0.10
Arid2	chr15	96223066	96232775	-2.27	0.36
Brd1	chr15	88543110	88543809	-2.54	0.27
Kansl2	chr15	98349531	98349829	-0.50	-0.10
Riox2	chr16	59471749	59472290	-2.24	0.66
Riox2	chr16	59475394	59475664	-0.80	0.16
Brwd1	chr16	96223575	96225491	-0.35	0.01
Brwd1	chr16	96226300	96226615	-0.52	-1.11
Brwd1	chr16	96229480	96229661	-1.19	0.48
Arid1b	chr17	5332607	5339263	-2.33	-0.16
Brpf3	chr17	28938130	28938251	-0.19	0.10
Brpf3	chr17	28954078	28960704	-1.08	0.19
Brpf3	chr17	28973638	28973848	-0.68	0.35
Ehmt2	chr17	35042089	35042587	-2.96	-0.29
Ehmt2	chr17	35049712	35050992	-3.05	-0.21
Kdm3b	chr18	34964262	34970049	-2.47	-0.14
Kdm3b	chr18	34997287	34998126	-1.40	-0.14
Hdac3	chr18	38096953	38097223	-2.39	-0.32
Kmt5b	chr19	3767600	3767721	-0.43	0.32
Kmt5b	chr19	3807383	3807444	-0.32	-0.05
Kmt5b	chr19	3808821	3816385	-2.74	-0.16
Brms1	chr19	5040306	5040901	-2.26	-0.29
Brms1	chr19	5041286	5046457	-2.05	-0.19

Rela	chr19	5637578	5637639	-0.26	0.03
Smarca2	chr19	26705264	26705505	-0.28	0.00
Nfkb2	chr19	46379346	46380320	-1.80	0.46
Kdm2a	chr19	4316774	4316954	-0.33	-0.02
Kdm2a	chr19	4318778	4320217	-0.49	0.03
Kdm2a	chr19	4322495	4326851	-1.52	0.12
Kdm2a	chr19	4396332	4396782	-1.56	-0.22
Kdm6a	chrX	17827922	17831812	-3.44	0.29
Kdm6a	chrX	17855854	17856005	-2.03	0.69
Jade3	chrX	20094125	20094634	-1.90	0.45
Ogt	chrX	98874640	98878285	-0.71	0.04
Ogt	chrX	98878792	98878973	-1.90	-0.42
Ogt	chrX	98879151	98879242	-0.52	-0.19
Kdm5c	chrX	148667772	148667893	-0.85	0.26
Kdm5c	chrX	148705250	148708150	-0.85	0.04
Suv39h1	chrX	7647858	7649530	-0.54	0.06
Hcfc1	chrX	71194680	71196000	-1.71	-0.08
Hcfc1	chrX	71211533	71211654	-0.84	0.13
Kdm5d	chrY	277702	278241	-2.00	0.29

TNF-signaling

gene_name	chr	chromStart	chromEnd	diff.lg.p	diff.log2.fc
Casp8	chr1	58890568	58903481	-0.47	0.03
Usp21	chr1	173216496	173218092	-3.29	-0.45
Traf2	chr2	25374427	25375145	-1.34	-0.17
Traf1	chr2	34803327	34804015	-1.17	-0.19
Madd	chr2	90977546	90977756	-0.38	-0.07
Madd	chr2	91018180	91018480	-0.45	-0.08
Otud7b	chr3	95956546	95960193	-2.15	-0.13
Map3k7	chr4	32051081	32051232	-2.61	-1.03
Map3k7	chr4	32109961	32110261	-0.43	0.08
Nsmaf	chr4	6381298	6381418	-0.26	-0.16
Fadd	chr7	151765572	151768281	-2.06	-0.21
Cyld	chr8	91220956	91226218	-0.51	0.31
Ikbkb	chr8	23770546	23770997	-1.89	-0.25
Bag4	chr8	26877798	26878878	-2.46	0.26
Tradd	chr8	107782474	107783441	-0.33	0.01
Tradd	chr8	107783843	107788494	-1.18	0.34
Smpd3	chr8	108779174	108779539	-0.33	-1.29
Usp2	chr9	43883576	43884236	-2.87	2.50
Usp2	chr9	43902032	43902332	-0.41	-0.10
Usp4	chr9	108293381	108294561	-0.32	0.00
Birc2	chr9	7818584	7818943	-0.91	0.25
Birc2	chr9	7834955	7835255	-3.06	0.75

Birc3	chr9	7848939	7850928	-0.49	0.05
Birc3	chr9	7860311	7872961	-0.93	0.08
Sppl2b	chr10	80329626	80329897	-0.88	0.59
Tab2	chr10	7625983	7626463	-1.95	0.18
Tab2	chr10	7626671	7644544	-2.25	0.07
Tnfaip3	chr10	18721581	18725137	-2.68	-0.19
Ripk1	chr13	34119764	34125274	-2.04	-0.13
Ripk1	chr13	34125393	34126112	-1.53	-0.25
Sharpin	chr15	76180512	76181540	-0.37	-0.02
Chuk	chr19	44181846	44181937	-0.41	-0.11
Ikbkg	chrX	71675074	71678148	-0.70	-0.30
Tab3	chrX	82860462	82860673	-2.95	0.81
Tab3	chrX	82875864	82876014	-3.15	1.28

C-type lectin receptors

gene_name	chr	chromStart	chromEnd	diff.lg.p	diff.log2.fc
Casp8	chr1	58890568	58903481	-0.47	0.03
Psmd1	chr1	87961193	87961313	-0.67	0.21
Psmc3	chr2	90894439	90895182	-1.38	-0.10
Traf6	chr2	101531587	101537929	-3.55	-0.22
Src	chr2	157295226	157296471	-0.85	0.32
Card9	chr2	26212789	26214357	-0.38	0.02
Psmd5	chr2	34707993	34709794	-1.37	-0.17
Psmd5	chr2	34722532	34726482	-1.60	-0.40
Psmb7	chr2	38498946	38499426	-1.35	-0.19
Il1b	chr2	129195984	129196856	-1.18	3.00
Psmf1	chr2	151543111	151543232	-0.83	-0.47
Psmf1	chr2	151543649	151544068	-0.81	-0.19
Nfatc2	chr2	168301939	168303199	-2.11	-0.21
Nfatc2	chr2	168304128	168330396	-0.75	0.06
Nfatc2	chr2	168396287	168427005	-2.25	-0.19
Nras	chr3	102868375	102868495	-0.73	-0.10
Bcl10	chr3	145587232	145587592	-0.60	0.07
Bcl10	chr3	145588099	145593615	-2.28	0.24
Psmd4	chr3	94836626	94836924	-1.79	-0.12
Psmd4	chr3	94839816	94846467	-2.16	-0.13
Nfkb1	chr3	135248217	135252299	-2.46	-0.20
Nfkb1	chr3	135354240	135354361	-0.32	0.21
Prkacb	chr3	146394723	146394993	-1.39	-0.21
Lyn	chr4	3605267	3605506	-3.28	-0.20
Map3k7	chr4	32051081	32051232	-2.61	-1.03
Map3k7	chr4	32109961	32110261	-0.43	0.08
Psmd9	chr5	123678228	123684616	-1.07	0.18
Card11	chr5	141348952	141350390	-3.03	-0.50

Raf1	chr6	115568620	115569280	-2.59	-0.22
Kras	chr6	145168783	145169023	-0.29	0.00
Calm3	chr7	17502039	17502309	-1.49	0.09
Relb	chr7	20191570	20192229	-0.55	-0.06
Psmd8	chr7	29965393	29965573	-1.95	-0.13
Hras	chr7	148376639	148378177	-1.50	-0.13
Hras	chr7	148378835	148379754	-0.27	-0.01
Hras	chr7	148379812	148379903	-0.43	0.54
Nfatc3	chr8	108602571	108603618	-0.98	0.07
Nfatc3	chr8	108632435	108652166	-2.54	0.17
Plcg2	chr8	120022190	120028105	-0.40	0.04
Ikbkb	chr8	23770546	23770997	-1.89	-0.25
Psmb10	chr8	108461631	108461997	-1.25	-0.38
Psma4	chr9	54805658	54805748	-1.39	0.21
Fyn	chr10	39089604	39089964	-1.17	-0.23
Fyn	chr10	39271197	39284162	-3.65	-0.24
Tab2	chr10	7625983	7626463	-1.95	0.18
Tab2	chr10	7626671	7644544	-2.25	0.07
Ube2d1	chr10	70718055	70718592	-2.86	0.34
Psme4	chr11	30671774	30672135	-0.53	-0.17
Fbxw11	chr11	32644536	32644986	-0.65	0.06
Psmd11	chr11	80285574	80285635	-1.26	-0.32
Psmd3	chr11	98556993	98557203	-2.02	-0.21
Psme3	chr11	101177564	101178336	-0.77	0.09
Psme3	chr11	101182100	101183258	-2.86	-0.26
Map3k14	chr11	103081077	103082247	-0.72	-0.12
Map3k14	chr11	103100286	103101085	-0.44	0.06
Map3k14	chr11	103103466	103128715	-0.61	-0.18
Psma6	chr12	56499811	56499900	-1.08	-0.18
Psmc1	chr12	101348377	101351316	-0.70	-0.11
Rps6ka5	chr12	101789211	101791305	-1.35	-0.27
Psmc6	chr14	45949498	45950514	-0.58	0.08
Ppp3cb	chr14	21365614	21365735	-1.14	-0.58
Prkcd	chr14	31408808	31411699	-0.55	0.03
Prkcd	chr14	31411787	31411937	-0.29	-0.01
Ep300	chr15	81416643	81438767	-1.05	0.06
Ep300	chr15	81479273	81481819	-0.51	0.02
Psmd2	chr16	20651724	20652324	-3.25	-0.17
Crebbp	chr16	4154961	4213404	-1.70	-0.10
Pak2	chr16	32019862	32021909	-1.43	0.09
Psmb8	chr17	34335288	34336214	-1.01	-0.16
Pdpk1	chr17	24215583	24216033	-2.93	0.32
Calm2	chr17	87835011	87846216	-3.10	-0.20

Ube2d2a	chr18	35931242	35931392	-1.92	-0.45
Ube2d2a	chr18	35965360	35965570	-1.78	0.47
Nfatc1	chr18	80803872	80832687	-3.26	-0.24
Nfatc1	chr18	80846262	80846653	-1.93	0.30
Nfatc1	chr18	80879052	80904894	-0.33	-0.01
Rela	chr19	5637578	5637639	-0.26	0.03
Btrc	chr19	45604207	45604268	-0.27	-0.42
Nfkb2	chr19	46379346	46380320	-1.80	0.46
Chuk	chr19	44181846	44181937	-0.41	-0.11
Ikbkg	chrX	71675074	71678148	-0.70	-0.30
Tab3	chrX	82860462	82860673	-2.95	0.81
Tab3	chrX	82875864	82876014	-3.15	1.28

Differentially methylated genes following 4 h *P. aeruginosa* infection

Gene expression (transcription)

gene_name	chr	chromStart	chromEnd	diff.lg.p	diff.log2.fc
Cnot9	chr1	74575438	74575798	-0.32	0.01
Psmd1	chr1	87961193	87961254	-0.37	-0.11
Ing5	chr1	95714871	95715891	-2.55	0.30
Ccnt2	chr1	129698762	129700561	-1.19	0.07
Rbbp5	chr1	134394590	134400982	-3.46	0.33
Rbbp5	chr1	134401131	134401282	-0.55	-0.11
Kdm5b	chr1	136514466	136514617	-1.35	0.58
Kdm5b	chr1	136521340	136521461	-0.57	-0.18
Kdm5b	chr1	136521520	136521700	-0.79	-0.26
Kdm5b	chr1	136525318	136527448	-0.82	-0.14
Kdm5b	chr1	136527777	136528227	-0.93	-0.18
Nr1i3	chr1	173148035	173148515	-0.64	0.16
Nr1i3	chr1	173148874	173149204	-1.38	-0.45
Exo1	chr1	177829710	177831466	-3.37	-0.85
Exo1	chr1	177839222	177839403	-0.90	-1.64
Parp1	chr1	182499105	182503813	-3.02	-0.53
Trp53bp2	chr1	184376701	184379234	-3.08	-0.39
Trp53bp2	chr1	184389061	184392298	-0.35	-0.03
Ints7	chr1	193443646	193445390	-0.49	-0.07
Nabp1	chr1	51523330	51523540	-1.18	1.15
Gls	chr1	52223048	52223109	-3.29	0.64
Gls	chr1	52276864	52290076	-0.98	-0.10
Sf3b1	chr1	55084231	55084322	-0.60	-0.19
Sumo1	chr1	59696425	59696959	-2.60	0.18
Bard1	chr1	71121361	71122020	-3.24	-0.57
Hdac4	chr1	93829331	93829930	-2.32	-0.46
Mdm4	chr1	134888236	134893148	-1.31	0.10
Zc3h11a	chr1	135516447	135517433	-0.86	0.06

Zc3h11a	chr1	135517641	135518703	-1.96	0.10
Zc3h11a	chr1	135521340	135523811	-0.72	0.04
Rnf2	chr1	153318408	153318469	-0.48	0.17
Pou2f1	chr1	167805604	167806174	-0.37	0.04
Tfb2m	chr1	181476099	181476398	-1.92	-0.43
Smyd2	chr1	191704608	191704728	-0.52	-0.07
Bmi1	chr2	18605637	18606468	-2.42	0.17
Tsc1	chr2	28531258	28532030	-1.43	-0.24
Tsc1	chr2	28541343	28542943	-2.03	-0.23
Ttf1	chr2	28915782	28922626	-1.94	-0.17
Ttf1	chr2	28941735	28942005	-0.65	0.21
Abl1	chr2	31544075	31551320	-1.18	-0.13
Abl1	chr2	31653102	31658129	-1.05	-0.08
Nup214	chr2	31888785	31893623	-1.12	-0.08
Nup214	chr2	31907789	31907850	-0.40	-0.11
Mapkap1	chr2	34479153	34479423	-2.45	-0.44
Ssrp1	chr2	84885611	84887208	-3.11	-0.12
Psmc3	chr2	90894439	90895182	-2.45	-0.16
Rtf1	chr2	119558206	119559166	-1.97	-0.19
Rtf1	chr2	119560273	119560484	-0.78	-0.27
Polr1b	chr2	128950958	128952063	-1.78	0.14
Polr3f	chr2	144362909	144365002	-0.63	0.07
Tpx2	chr2	152719325	152719804	-0.61	0.07
Itch	chr2	154959216	154959307	-0.30	0.07
Itch	chr2	155050132	155050403	-0.62	-0.11
Phf20	chr2	156092990	156093201	-0.52	0.09
Phf20	chr2	156124429	156134013	-0.50	0.03
Tgif2	chr2	156679307	156679783	-2.73	-0.95
Src	chr2	157295196	157296561	-1.97	0.63
Rprd1b	chr2	157900751	157901171	-0.47	-0.06
Srsf6	chr2	162757243	162757817	-0.60	-0.04
Trp53rkb	chr2	166620945	166621725	-1.43	-0.33
Cstf1	chr2	172203213	172206586	-1.68	-0.11
Gata3	chr2	9779686	9779776	-0.33	0.26
Gata3	chr2	9779865	9779925	-0.37	0.52
Gata3	chr2	9779984	9780252	-0.85	0.39
Gata3	chr2	9790440	9796131	-0.26	0.02
Taf3	chr2	9837377	9862619	-2.08	-0.23
Taf3	chr2	9862678	9874561	-0.84	0.07
Ehmt1	chr2	24646742	24647852	-2.26	-0.23
Ehmt1	chr2	24733063	24775080	-1.70	0.17
Snapc4	chr2	26223896	26225494	-2.87	-0.39
Notch1	chr2	26314049	26314349	-0.81	0.70

Notch1	chr2	26315066	26315157	-0.65	-1.86
Notch1	chr2	26315335	26315486	-0.50	-0.74
Notch1	chr2	26316053	26317703	-0.30	-0.07
Notch1	chr2	26323937	26324027	-0.49	-1.14
Gtf3c5	chr2	28421871	28423253	-0.44	-0.04
Gtf3c5	chr2	28438382	28438531	-1.11	-0.37
Gtf3c4	chr2	28679915	28683050	-0.71	0.06
Gtf3c4	chr2	28690219	28692035	-1.30	0.14
Gtf3c4	chr2	28695550	28695880	-1.08	-0.26
Cdk9	chr2	32562880	32565348	-3.08	-0.10
Psmd5	chr2	34707993	34709823	-0.82	-0.10
Psmd5	chr2	34726274	34726482	-3.28	-0.70
Psmb7	chr2	38498946	38499426	-1.70	-0.24
Nr4a2	chr2	56960145	56961132	-1.31	-0.24
Nr4a2	chr2	56965686	56976354	-1.92	1.13
Clp1	chr2	84563548	84566299	-1.88	0.17
Zfp770	chr2	114021193	114021283	-0.43	0.17
Zfp661	chr2	127402341	127403505	-0.42	0.05
Snrpb	chr2	130002885	130005100	-1.37	-0.14
Psmf1	chr2	151543679	151544068	-0.32	0.02
Zfp334	chr2	165203354	165203685	-0.40	-0.13
Zfp334	chr2	165205181	165206141	-0.43	-0.06
Zfp334	chr2	165207038	165207219	-1.00	-0.50
Trp53rka	chr2	165315878	165318784	-1.13	0.14
Aurka	chr2	172182168	172182653	-0.85	0.13
Skil	chr3	30994042	30997382	-0.39	0.01
Gatad2b	chr3	90145581	90145879	-2.33	-0.23
Gatad2b	chr3	90155817	90155908	-2.67	-0.37
Hist2h2be	chr3	96025043	96025490	-0.87	-0.31
Rbm8a	chr3	96433850	96433910	-0.48	0.21
Trim33	chr3	103148225	103148495	-2.43	0.25
Ints12	chr3	132771859	132772487	-1.86	0.21
Gtf2b	chr3	142428210	142434431	-1.44	-0.33
Gtf2b	chr3	142446182	142446450	-3.59	0.37
Phc3	chr3	30805917	30813151	-0.46	0.06
Phc3	chr3	30864763	30868337	-1.50	-0.73
Maml3	chr3	51491893	51493212	-1.42	0.26
Maml3	chr3	51493600	51496170	-0.52	0.08
Maml3	chr3	51501693	51600368	-0.39	0.34
Maml3	chr3	51659540	51660829	-2.03	0.23
Maml3	chr3	51907999	51908149	-1.14	0.58
Maml3	chr3	51908418	51908808	-0.35	0.06
Ints3	chr3	90195399	90196193	-3.05	-0.26

Chtop	chr3	90303623	90304012	-0.38	0.01
Chtop	chr3	90313185	90313365	-0.85	-0.09
Psmb4	chr3	94690551	94690880	-2.31	-0.17
Rprd2	chr3	95567509	95570116	-0.33	0.00
Rprd2	chr3	95576734	95577094	-0.64	0.14
Polr3gl	chr3	96382213	96386015	-0.88	-0.10
Polr3gl	chr3	96398020	96398081	-0.73	-0.63
Hipk1	chr3	103547180	103548199	-0.93	-0.04
Prmt6	chr3	110050616	110051517	-1.73	-0.62
Prmt6	chr3	110052386	110053916	-3.52	-0.20
Nbn	chr4	15906479	15908877	-2.76	0.36
Rngtt	chr4	33587224	33587673	-1.48	0.31
Rngtt	chr4	33588240	33588330	-0.44	0.40
Polr1e	chr4	45044885	45045245	-0.56	-0.14
Polr1e	chr4	45045964	45046084	-0.94	-1.25
Nr4a3	chr4	48080715	48096224	-0.55	0.37
Rraga	chr4	86221576	86222293	-1.17	0.09
Tal1	chr4	114737352	114742061	-0.43	-0.04
Med8	chr4	118087470	118087618	-1.43	0.25
Scmh1	chr4	120201905	120201966	-0.42	-0.19
Rragc	chr4	123597235	123597356	-2.18	0.32
Rragc	chr4	123598193	123598284	-1.16	0.24
Lsm10	chr4	125773896	125775828	-0.48	0.04
Phc2	chr4	128404881	128405061	-0.49	0.29
Nr0b2	chr4	133112156	133112304	-1.32	-1.45
Zfp46	chr4	135846237	135848601	-0.61	-0.06
Mtor	chr4	147930392	147931764	-0.76	0.10
Ssu72	chr4	155078983	155079074	-1.02	0.85
Ssu72	chr4	155079462	155089672	-3.59	-0.27
Mllt3	chr4	87419218	87419789	-0.47	0.14
Mllt3	chr4	87486765	87487216	-0.56	-0.10
Zfp691	chr4	118842660	118845711	-0.33	0.01
Zfp691	chr4	118846309	118846770	-2.39	1.37
Zfp69	chr4	120602801	120603819	-0.86	0.27
Ago1	chr4	126116441	126116592	-2.37	-0.72
Ago4	chr4	126167982	126168462	-0.37	-0.10
Ago4	chr4	126169867	126170047	-0.27	0.12
Tfap2e	chr4	126393574	126393694	-1.54	2.92
Sesn2	chr4	132048721	132050516	-0.58	-0.04
Sfn	chr4	133157754	133158083	-0.27	-0.02
Srrm1	chr4	134878175	134878385	-1.96	-0.19
Srrm1	chr4	134902739	134909069	-0.74	0.09
Eloa	chr4	135561252	135561580	-1.33	0.22

Eloa	chr4	135565701	135577564	-0.37	0.01
Ski	chr4	154529795	154530722	-0.41	-0.02
Ski	chr4	154530781	154533563	-1.24	0.09
Ski	chr4	154533945	154596644	-3.03	-0.18
Cdk6	chr5	3519406	3520884	-1.97	-0.19
Mterf1b	chr5	4196342	4196551	-0.53	-0.37
Mterf1b	chr5	4196758	4197205	-0.91	0.44
Kmt2e	chr5	22940246	22940487	-0.36	0.04
Kmt2e	chr5	23002879	23005273	-2.07	0.16
Kmt2e	chr5	23007381	23008999	-2.35	0.12
Nupl2	chr5	23688056	23689616	-0.80	0.11
Ywhah	chr5	33361494	33361614	-0.36	0.07
Ywhah	chr5	33369299	33370047	-1.92	-0.08
Rbpj	chr5	54044875	54045116	-0.40	-0.92
Rpap2	chr5	108048973	108049573	-2.30	0.34
Mtf2	chr5	108494760	108498406	-0.37	-0.03
Mtf2	chr5	108535653	108536103	-0.55	-0.06
Zfp605	chr5	110556690	110556990	-0.28	0.01
Zfp605	chr5	110557318	110558156	-0.31	-0.02
Triap1	chr5	115791255	115793441	-0.77	0.08
Psmd9	chr5	123678228	123678437	-1.01	0.20
Kmt5a	chr5	124911266	124911327	-0.69	-0.13
Gtf2h3	chr5	125046222	125046283	-0.86	-0.51
Zfp664	chr5	125343785	125366362	-3.24	-0.19
Zfp664	chr5	125366751	125367081	-0.41	-0.05
Crcp	chr5	130505205	130510800	-0.78	-0.28
Crcp	chr5	130535663	130536293	-2.60	-0.28
Rfc2	chr5	135073840	135074198	-2.20	-0.32
Baz1b	chr5	135718322	135721070	-0.60	0.04
Zkscan1	chr5	138526311	138534046	-0.28	-0.01
Zkscan1	chr5	138541944	138542455	-2.08	-0.24
Zkscan1	chr5	138544222	138545932	-1.09	0.11
Zfp12	chr5	144005811	144007821	-2.30	-0.23
Zfp12	chr5	144007940	144008390	-0.87	-0.26
Zfp12	chr5	144008869	144009619	-2.02	-0.39
Cpsf4	chr5	145939775	145942670	-0.46	0.04
Zkscan5	chr5	145979715	145981274	-2.23	0.66
Zkscan5	chr5	145981691	145982380	-0.37	-0.06
Zfp655	chr5	145992583	145994463	-1.50	-0.36
Smarcd3	chr5	24100691	24100811	-0.72	-0.86
Prkag2	chr5	24605860	24606370	-1.09	-0.18
Kmt2c	chr5	24790138	24790949	-1.48	0.18
Kmt2c	chr5	24819830	24820730	-2.10	0.18

Gtf3c2	chr5	31459414	31460596	-2.84	-0.16
Slbp	chr5	33992455	33994955	-2.31	0.29
Nup54	chr5	92845101	92846543	-0.74	0.09
Prkab1	chr5	116463747	116467999	-0.33	-0.01
Prkab1	chr5	116474139	116474378	-0.83	-0.19
Ptpn11	chr5	121581827	121582307	-1.65	0.20
Ptpn11	chr5	121583292	121587519	-2.53	-0.16
Ncor2	chr5	125538304	125548276	-0.66	-0.08
Ncor2	chr5	125659463	125659584	-0.24	-0.12
Ubc	chr5	125867967	125870328	-2.39	0.10
Zfp11	chr5	130162172	130162950	-0.33	-0.05
Zfp113	chr5	138585541	138586141	-1.27	-0.30
Zfp113	chr5	138586470	138586680	-0.76	0.33
Taf6	chr5	138619844	138620202	-3.23	-0.49
Smurf1	chr5	145637663	145637963	-1.05	-0.44
Smurf1	chr5	145638322	145638682	-0.71	-0.20
Smurf1	chr5	145639310	145639521	-0.43	0.15
Smurf1	chr5	145726518	145726669	-0.30	0.31
Zkscan14	chr5	145956021	145962455	-1.48	-0.29
Rfc3	chr5	152445398	152445638	-0.35	0.05
Nup205	chr6	35127615	35133895	-0.46	0.13
Cul1	chr6	47474617	47476108	-2.74	-0.21
Zfp398	chr6	47790290	47790621	-3.35	-1.10
Zfp282	chr6	47854827	47855966	-0.80	-0.11
Zfp282	chr6	47856175	47856805	-0.84	-0.17
Zfp212	chr6	47870655	47876681	-3.35	-1.03
Zfp212	chr6	47880732	47881447	-2.64	-0.48
Zfp775	chr6	48555624	48569787	-2.72	-0.92
Zfp775	chr6	48569965	48570026	-0.29	-0.39
Zfp775	chr6	48570384	48570923	-3.10	-1.25
Polr1a	chr6	71928026	71930131	-0.59	0.05
Gata2	chr6	88154683	88155829	-0.38	-0.05
Nr2c2	chr6	92041411	92042461	-0.44	-0.16
Nr2c2	chr6	92114226	92117902	-0.73	-0.11
Chd4	chr6	125079888	125080369	-0.40	-0.01
Cdkn1b	chr6	134870958	134872360	-0.68	0.10
Gm8994	chr6	136279021	136279082	-0.20	-0.85
Gm8994	chr6	136279763	136279882	-0.28	0.95
H2afj	chr6	136757367	136757517	-1.06	-0.54
Aebp2	chr6	140571543	140572353	-3.40	-0.64
Cnot4	chr6	34973352	34974287	-0.81	0.20
Cnot4	chr6	34996066	35002891	-0.64	-0.06
Hipk2	chr6	38697991	38787107	-0.63	0.02

Hipk2	chr6	38825829	38826040	-1.39	-0.22
Zfp786	chr6	47769651	47770307	-0.93	-0.92
Zfp786	chr6	47770514	47770575	-0.14	0.00
Zfp786	chr6	47770752	47771200	-1.52	-1.37
Zfp786	chr6	47771348	47771646	-0.99	-1.29
Zfp746	chr6	48013500	48016503	-0.85	-0.09
Zfp746	chr6	48033523	48036532	-0.28	0.01
Gadd45a	chr6	66985208	66987341	-0.31	0.00
Nup210	chr6	90963060	90963630	-0.69	-0.12
Rybp	chr6	100182244	100183112	-0.71	0.26
Zfp248	chr6	118378764	118380402	-2.34	0.42
Phc1	chr6	122275560	122275800	-2.40	-0.84
Tigar	chr6	127037468	127038307	-1.14	0.14
Ccnd2	chr6	127079526	127080245	-0.86	0.11
Rhno1	chr6	128307285	128309180	-0.40	-0.02
Kras	chr6	145168813	145168964	-1.25	-0.35
U2af2	chr7	5013783	5013844	-0.45	0.13
U2af2	chr7	5030853	5031123	-0.52	-0.04
Zfp667	chr7	6256343	6256524	-0.69	0.61
Zfp667	chr7	6257749	6257810	-0.29	0.32
Zfp78	chr7	6331550	6332238	-0.37	0.13
Zfp28	chr7	6344996	6347379	-0.53	0.10
Zfp110	chr7	13434054	13434502	-0.47	-0.06
Zfp324	chr7	13555642	13556271	-2.04	-0.62
Zfp324	chr7	13556928	13557616	-2.87	-0.66
Zfp446	chr7	13567286	13568246	-1.90	-0.38
Ercc2	chr7	19979500	19981041	-0.83	-0.08
Zfp180	chr7	24882912	24891516	-1.80	0.13
Zfp112	chr7	24910123	24910184	-1.08	-3.00
Zfp112	chr7	24910243	24910454	-0.57	-0.73
Zfp112	chr7	24910513	24910604	-0.13	-0.17
Zfp112	chr7	24910962	24911473	-0.38	0.21
Zfp112	chr7	24912431	24912551	-0.80	1.66
Zfp235	chr7	24925651	24925742	-0.66	0.68
Zfp235	chr7	24927899	24928200	-0.36	-0.20
Akt2	chr7	28422173	28423428	-0.40	0.03
Zfp30	chr7	30577880	30578810	-1.97	0.80
Zfp790	chr7	30601090	30601151	-0.45	0.29
Zfp420	chr7	30645086	30661279	-2.76	0.27
Zfp382	chr7	30916575	30919814	-1.94	0.18
Polr2i	chr7	31017332	31017529	-0.50	-0.12
Zfp658	chr7	50828078	50828289	-0.99	-0.64
Zfp658	chr7	50828587	50829455	-0.67	-0.16

Zfp658	chr7	50829663	50830293	-0.55	0.15
Nup62	chr7	52071739	52086176	-1.81	-0.05
Zfp710	chr7	87231504	87231625	-1.34	-0.43
Zfp710	chr7	87233516	87236264	-1.90	-0.12
Lamtor1	chr7	109054380	109058241	-0.51	-0.09
Ctr9	chr7	118172674	118177414	-0.59	-0.08
Ctr9	chr7	118198901	118199651	-0.33	0.01
Setd1a	chr7	134927665	134930705	-3.33	-0.13
Setd1a	chr7	134931780	134932028	-0.75	-0.13
Zik1	chr7	11075894	11076075	-0.62	0.50
Zfp551	chr7	13000795	13000946	-0.55	-0.63
Zfp551	chr7	13001544	13001605	-0.37	0.66
Zfp551	chr7	13001783	13001874	-0.23	-0.07
Zfp551	chr7	13002053	13002203	-0.45	-0.51
Pou2f2	chr7	25876133	25878039	-1.00	-0.09
Pou2f2	chr7	25906059	25917479	-0.36	0.12
Zfp940	chr7	30629198	30629379	-2.28	-1.54
Zfp940	chr7	30630307	30630488	-0.67	-0.73
Zfp27	chr7	30678504	30680419	-1.09	0.13
Zfp27	chr7	30680807	30683850	-0.49	0.07
Zfp14	chr7	30823226	30823406	-0.82	1.24
Zfp566	chr7	30862385	30862831	-0.63	0.23
Zfp566	chr7	30863008	30863483	-0.99	0.28
Ccne1	chr7	38883180	38883329	-1.29	-0.73
Zfp473	chr7	51987570	51988080	-0.82	0.69
Zfp473	chr7	51988469	51988860	-0.49	-0.50
Med25	chr7	52132313	52133597	-2.46	-0.25
Bax	chr7	52722059	52722238	-0.71	0.19
E2f8	chr7	56123087	56123387	-2.78	-0.89
Blm	chr7	87647292	87647681	-0.45	0.09
Blm	chr7	87654437	87658340	-2.18	0.36
Eed	chr7	97129276	97129426	-0.44	0.08
Pcf11	chr7	99801691	99808380	-0.63	-0.03
Nup98	chr7	109268003	109269174	-0.67	-0.03
Rrp8	chr7	112881232	112881920	-1.23	0.14
Rrp8	chr7	112882533	112882833	-2.17	-0.27
Rrp8	chr7	112883248	112885000	-0.45	-0.05
Gtf3c1	chr7	132786855	132787843	-1.82	-0.20
Gtf3c1	chr7	132790192	132791099	-0.40	0.03
Gtf3c1	chr7	132851051	132851202	-0.60	-0.15
Zfp747	chr7	134516438	134516647	-0.30	0.04
Zfp747	chr7	134516706	134519504	-2.94	0.29
9130019O22Rik	chr7	134526847	134529144	-0.29	0.00

E430018J23Rik	chr7	134534349	134536523	-1.20	-0.34
E430018J23Rik	chr7	134536927	134537135	-0.26	0.15
Zfp764	chr7	134547867	134549122	-1.58	-0.25
Zfp688	chr7	134562479	134563593	-2.08	-0.41
Zfp689	chr7	134587294	134588642	-2.65	-0.51
Zfp689	chr7	134592222	134592433	-0.67	0.30
Pidd1	chr7	148624413	148624744	-1.68	-0.61
Snapc2	chr8	4255341	4256161	-2.46	-0.36
Tfdp1	chr8	13377196	13377345	-0.76	-0.13
Tfdp1	chr8	13377582	13377702	-1.12	-0.18
Kat6a	chr8	24040759	24043005	-0.79	-0.08
Kat6a	chr8	24046218	24051430	-0.82	0.04
Ppp2cb	chr8	34710092	34710330	-0.63	-0.43
Gtf2e2	chr8	34886513	34887055	-0.33	0.02
Nr2c2ap	chr8	72655231	72655498	-1.07	0.29
Nr2c2ap	chr8	72657115	72657440	-0.91	-0.13
Ell	chr8	73063573	73063694	-2.62	-1.00
Ell	chr8	73107871	73114673	-3.00	-0.34
Sin3b	chr8	75247186	75248032	-1.12	0.19
Sin3b	chr8	75273726	75273966	-0.65	0.20
Sin3b	chr8	75280930	75281383	-2.20	-0.49
Nr3c2	chr8	79433507	79433776	-0.32	-0.42
Cbfb	chr8	107694632	107694812	-1.00	-0.17
Zfp90	chr8	108947880	108948329	-3.26	0.83
Zfp90	chr8	108948657	108949137	-0.47	0.12
Zfp90	chr8	108949196	108949675	-1.18	0.35
Zfp1	chr8	114190002	114194702	-0.53	-0.06
Banp	chr8	124550340	124550731	-0.77	-0.40
Banp	chr8	124550880	124551060	-0.48	-0.38
Banp	chr8	124551419	124551540	-1.16	-1.10
Banp	chr8	124552528	124552798	-1.31	-0.79
Zfpm1	chr8	124806220	124856010	-0.98	0.18
Zfpm1	chr8	124859027	124861147	-1.02	-0.10
Ash2l	chr8	26950536	26958082	-1.35	-0.23
Brf2	chr8	28234303	28236503	-0.90	0.15
Brf2	chr8	28238956	28239104	-0.75	-0.30
Wrn	chr8	34346284	34346854	-0.41	-0.08
Wrn	chr8	34434722	34439590	-2.21	0.55
Cnot7	chr8	41592731	41595397	-1.59	0.21
Ing2	chr8	48753850	48754661	-0.78	0.10
Ing2	chr8	48760302	48760513	-2.46	-1.21
Gatad2a	chr8	72498029	72498150	-0.57	-0.26
Gatad2a	chr8	72519918	72520248	-0.39	0.08

Csnk2a2	chr8	97976954	97977225	-1.72	-0.27
Dhx38	chr8	112071923	112073723	-0.63	0.08
Taf1c	chr8	122121770	122123330	-1.40	-0.15
Maml2	chr9	13424522	13425092	-2.48	0.70
Maml2	chr9	13510203	13511312	-3.34	0.53
Pin1	chr9	20456573	20459888	-1.84	-0.36
Zfp202	chr9	40015019	40015258	-3.36	-0.92
Usp2	chr9	43902061	43902302	-1.62	-0.76
H2afx	chr9	44143446	44144156	-0.58	0.03
Ppp2r1b	chr9	50688020	50688710	-0.53	0.05
Ppp2r1b	chr9	50701016	50701675	-1.83	-0.33
Sin3a	chr9	56958532	56958892	-0.66	0.11
Sin3a	chr9	56973115	56975606	-1.32	0.16
Ints14	chr9	64809611	64809761	-2.80	0.75
Rora	chr9	69226745	69226835	-0.38	-0.37
Ice2	chr9	69263440	69263950	-0.69	0.12
Ice2	chr9	69280073	69280163	-0.60	-0.34
Atr	chr9	95765579	95765699	-0.73	-0.27
Tfdp2	chr9	96218608	96218729	-0.27	0.00
Ctnnb1	chr9	120842577	120859351	-2.42	-0.18
Ctnnb1	chr9	120868936	120868997	-1.38	0.13
Zkscan7	chr9	122797695	122797846	-0.50	-0.44
Zkscan7	chr9	122803837	122804973	-1.16	-0.38
Zfp558	chr9	18260716	18261377	-0.28	0.03
Zfp266	chr9	20304179	20306501	-0.43	0.03
Dnmt1	chr9	20733552	20734608	-0.96	0.27
Kmt2a	chr9	44612666	44613327	-0.91	-0.13
Kmt2a	chr9	44629634	44630384	-2.09	0.19
Kmt2a	chr9	44655514	44656204	-3.18	0.26
Kmt2a	chr9	44656473	44656924	-0.49	0.06
Pml	chr9	58067502	58068341	-3.10	-0.28
Pml	chr9	58080996	58094952	-1.77	-0.21
Pml	chr9	58097382	58097593	-1.39	-0.43
Smad3	chr9	63496369	63496909	-0.75	0.36
Smad3	chr9	63497357	63498046	-0.81	-0.34
Smad3	chr9	63605729	63605891	-0.28	-0.36
Rnf111	chr9	70274555	70277420	-1.37	0.15
Tcf12	chr9	71958831	71958892	-0.31	-0.03
Tcf12	chr9	71959385	71959626	-0.98	-0.22
Atrip	chr9	108960445	108961754	-3.54	-0.18
Atrip	chr9	108967670	108968299	-2.16	0.32
Cnot10	chr9	114541870	114549228	-1.71	-0.34
Sgk1	chr10	21718573	21718873	-1.56	0.74

Sesn1	chr10	41530409	41607263	-0.57	0.15
Sesn1	chr10	41627347	41627735	-0.58	0.32
Ranbp2	chr10	57941534	57941955	-2.61	0.14
Stk11	chr10	79579091	79587616	-1.24	-0.11
Stk11	chr10	79592650	79593215	-1.30	-0.18
Txnrd1	chr10	82296755	82323178	-0.61	0.15
Polr3b	chr10	84188455	84188875	-0.88	-0.17
Smarcc2	chr10	127920231	127920695	-2.24	0.31
Smarcc2	chr10	127926123	127926273	-0.50	0.09
Ddit4	chr10	59412629	59412689	-0.57	0.25
Sirt1	chr10	62801484	62801753	-2.02	-0.87
Nrbf2	chr10	66729823	66730867	-1.76	0.19
Tfam	chr10	70690735	70691424	-1.82	0.15
Ube2d1	chr10	70718174	70718562	-1.17	0.19
Smarcb1	chr10	75359631	75359751	-1.58	-0.36
Polrmt	chr10	79201183	79202890	-3.07	-0.34
Polr2e	chr10	79501286	79502404	-1.51	-0.21
Mbd3	chr10	79855285	79856691	-1.70	-0.10
Tcf3	chr10	79872416	79873225	-0.84	-0.10
Cradd	chr10	94637889	94638400	-1.23	-0.45
Cnot2	chr10	115922246	115922606	-0.97	0.18
Cnot2	chr10	115922874	115923115	-1.67	0.33
Yeats4	chr10	116652465	116652884	-1.13	0.17
Dyrk2	chr10	118296404	118305900	-0.46	0.03
Pip4k2c	chr10	126635286	126635914	-1.06	0.12
Thoc5	chr11	4799846	4799907	-2.53	-0.61
Psme4	chr11	30671804	30671925	-0.83	-0.91
Psme4	chr11	30672014	30672135	-0.38	0.12
Slu7	chr11	43247232	43250977	-0.69	-0.10
Med7	chr11	46250448	46250688	-1.24	-0.38
Ppp2ca	chr11	51935449	51935836	-1.90	0.10
Aff4	chr11	53164268	53186578	-0.79	-0.04
Aff4	chr11	53211860	53217858	-0.54	0.02
Cnot8	chr11	57930988	57931258	-1.37	0.34
Zkscan6	chr11	65636412	65642412	-0.89	-0.14
Trp53	chr11	69393860	69394608	-0.68	0.14
Ywhae	chr11	75546388	75546567	-2.12	-0.15
Suz12	chr11	79843530	79845978	-2.27	0.14
Psmd11	chr11	80285544	80285635	-1.72	-0.32
Cdk5r1	chr11	80293403	80293969	-3.03	0.92
Cdk5r1	chr11	80293392	80293962	-2.75	0.87
Taf15	chr11	83286609	83286700	-0.71	-0.14
Ppm1d	chr11	85140408	85140529	-0.31	-0.04

Ppm1d	chr11	85153180	85159827	-0.35	0.02
Supt4a	chr11	87557029	87557119	-0.77	0.39
Srsf1	chr11	87861172	87861352	-2.81	-0.34
Cdk12	chr11	98071677	98072546	-0.31	0.00
Thra	chr11	98603186	98617447	-0.55	0.14
Thra	chr11	98625630	98626259	-2.85	-0.46
Casc3	chr11	98692883	98694014	-1.36	-0.17
Rara	chr11	98799009	98808938	-1.27	-0.44
Psme3	chr11	101177564	101178336	-0.63	-0.07
Psme3	chr11	101182070	101183288	-1.70	-0.15
Tnrc6c	chr11	117515962	117562114	-0.45	-0.17
Tnrc6c	chr11	117623163	117623674	-2.09	0.60
Cbx2	chr11	118885531	118887831	-0.67	1.03
Rptor	chr11	119465086	119517298	-0.84	0.12
Rptor	chr11	119759767	119760755	-0.49	-0.04
Hus1	chr11	8896741	8896861	-0.52	0.16
Lsm11	chr11	45747117	45747446	-1.53	-0.39
Maml1	chr11	50069972	50070390	-0.48	0.08
Maml1	chr11	50076862	50105838	-3.04	-0.21
Rad50	chr11	53463680	53465769	-1.04	-0.17
Zkscan17	chr11	59299022	59299352	-0.46	0.07
Zkscan17	chr11	59300338	59305712	-0.44	0.02
Top3a	chr11	60555827	60557475	-1.90	-0.31
Top3a	chr11	60590716	60590867	-1.91	0.89
Ncor1	chr11	62130910	62131481	-1.03	0.10
Ncor1	chr11	62147216	62147367	-0.98	0.19
Ncor1	chr11	62182894	62186997	-2.47	-0.16
Ncor1	chr11	62251972	62270948	-0.37	-0.06
Zfp287	chr11	62527143	62527774	-0.60	0.18
Zfp287	chr11	62528133	62528313	-0.31	0.07
Zfp287	chr11	62528582	62528703	-0.25	0.02
Zfp287	chr11	62541813	62542444	-0.65	-0.22
Chd3	chr11	69174956	69175626	-3.21	-0.43
Nup88	chr11	70783414	70783475	-0.56	0.19
Rpa1	chr11	75154547	75161885	-2.86	-0.51
Supt6	chr11	78047100	78059205	-2.14	-0.27
Med13	chr11	86100091	86100242	-0.90	-0.15
Med13	chr11	86132926	86141950	-2.79	0.16
Pip4k2b	chr11	97578204	97583775	-1.02	-0.05
Med1	chr11	98014158	98015024	-0.76	0.11
Med1	chr11	98015143	98015684	-0.34	0.01
Med1	chr11	98016102	98017333	-0.90	0.06
Med1	chr11	98017932	98018173	-0.41	-0.03

Med1	chr11	98018352	98019732	-1.77	0.10
Med1	chr11	98054124	98054607	-1.22	-0.20
Nr1d1	chr11	98629338	98629726	-1.00	0.26
Nr1d1	chr11	98631783	98632540	-3.18	-0.44
Nr1d1	chr11	98633136	98636556	-0.49	-0.06
Cavin1	chr11	100819819	100819880	-0.37	-2.17
Cavin1	chr11	100820119	100820270	-0.37	-0.94
Cavin1	chr11	100820629	100824135	-0.27	-1.32
Brca1	chr11	101384814	101385204	-1.75	-0.62
Brca1	chr11	101385473	101386581	-0.92	-0.20
Brca1	chr11	101386909	101387659	-3.36	0.64
Ubtf	chr11	102167282	102168062	-3.20	-0.19
Ubtf	chr11	102180259	102180320	-0.49	-0.34
Smurf2	chr11	106683743	106684358	-0.92	-0.20
Cbx8	chr11	118899867	118901442	-1.43	0.25
Cbx4	chr11	118940531	118940592	-1.14	1.49
Cbx4	chr11	118941070	118941400	-3.20	1.23
Cbx4	chr11	118941759	118947461	-0.50	0.02
Dnmt3a	chr12	3806582	3807208	-0.30	0.08
Dnmt3a	chr12	3835069	3866168	-3.48	-0.38
Dnmt3a	chr12	3906804	3908036	-0.47	-0.02
E2f6	chr12	16832231	16832859	-1.35	0.19
Twistnb	chr12	34121850	34123143	-1.92	0.15
Psma6	chr12	56499811	56499871	-1.67	-0.32
Ppm1a	chr12	73862227	73885470	-0.74	-0.04
Ppm1a	chr12	73894973	73895153	-0.48	0.08
Snapc1	chr12	75065516	75068947	-1.32	-0.31
Psmc1	chr12	101348377	101351286	-1.53	-0.29
Papola	chr12	107072945	107075506	-1.04	0.06
Ccnk	chr12	109418007	109424590	-0.70	-0.17
Zfp839	chr12	112106479	112107344	-0.76	-0.14
Mta1	chr12	114374970	114375239	-3.26	-0.32
Ywhaq	chr12	21422717	21423076	-1.30	-0.20
Max	chr12	78038822	78039718	-1.25	0.06
Max	chr12	78063025	78063235	-0.95	-0.16
Dicer1	chr12	105936045	105942279	-1.69	0.15
Dicer1	chr12	105960317	105960378	-1.42	0.41
Ppp1r13b	chr12	113067028	113067478	-1.41	-0.33
Akt1	chr12	113912217	113912487	-0.40	0.08
Brf1	chr12	114198252	114200794	-1.00	-0.12
Zkscan4	chr13	21576264	21576955	-0.44	-0.43
Hist1h3h	chr13	21809496	21809698	-0.75	-0.19
Hist1h3f	chr13	23636303	23636393	-1.63	-0.30

Hist1h2bg	chr13	23663268	23663474	-0.87	-0.07
Hist1h3d	chr13	23667608	23667786	-0.59	-0.11
Hist1h2bc	chr13	23776067	23776213	-1.76	0.26
Hist1h2ab	chr13	23842956	23843224	-1.74	-0.27
Rmi1	chr13	58503698	58503988	-0.71	-0.12
Med10	chr13	69954496	69954853	-0.30	0.00
Ell2	chr13	75844931	75845141	-0.97	-0.42
Taf9	chr13	101435858	101436098	-2.24	0.28
Plk2	chr13	111190024	111190991	-3.23	0.37
Slc38a9	chr13	113528239	113528690	-0.79	0.50
Cdk13	chr13	17858388	17896931	-0.44	-0.02
Zkscan3	chr13	21488092	21494295	-2.58	-0.45
Zkscan8	chr13	21611911	21613722	-1.26	-0.18
Zkscan8	chr13	21617156	21618818	-1.82	0.41
Hist1h3i	chr13	21875208	21875266	-0.36	-0.05
Hist1h3c	chr13	23837329	23837390	-0.45	0.12
Tfap2a	chr13	40812539	40812690	-0.17	0.59
Nup153	chr13	46776228	46776529	-1.65	0.22
Dek	chr13	47201073	47201589	-0.67	-0.13
Thoc3	chr13	54561210	54561569	-0.37	0.03
Ctsl	chr13	64470393	64471614	-2.04	-0.09
Zfp729a	chr13	67720451	67721021	-0.67	0.12
Zfp729a	chr13	67721529	67722158	-0.70	0.18
Zfp729a	chr13	67722397	67722547	-0.30	0.02
Zfp738	chr13	67768497	67769604	-3.07	0.55
Zfp738	chr13	67770112	67770352	-0.31	0.04
Zfp738	chr13	67771338	67771758	-1.04	0.44
Ice1	chr13	70741154	70747297	-2.33	-0.08
Ice1	chr13	70747386	70748030	-0.41	-0.05
Nr2f1	chr13	78328745	78328956	-1.15	-3.17
Polr3g	chr13	81814362	81815052	-1.54	0.31
Jmy	chr13	94234651	94269644	-2.87	-0.26
Rad17	chr13	101387477	101389058	-2.53	-0.36
Ercc6	chr14	33326706	33332187	-3.22	-0.42
Psmc6	chr14	45949498	45950514	-0.68	0.11
Ints9	chr14	65658332	65658483	-0.78	-0.25
Med4	chr14	73909855	73909974	-2.48	-0.83
Med4	chr14	73917729	73918293	-0.71	-0.10
Elf1	chr14	79881389	79936053	-0.38	0.05
Elf1	chr14	79973144	79980817	-0.52	-0.03
Rarb	chr14	17407447	17451237	-0.24	0.12
Nr1d2	chr14	19044433	19047065	-1.52	1.13
Polr3a	chr14	25267945	25269957	-2.74	-0.39

Pip4p1	chr14	51545984	51546434	-1.90	0.16
Supt16	chr14	52803669	52816914	-0.75	-0.13
Prmt5	chr14	55126018	55126553	-0.37	0.02
Rabggta	chr14	56340688	56340807	-0.30	0.04
Cenpj	chr14	57145687	57148592	-0.68	-0.24
Cenpj	chr14	57170817	57171506	-0.75	-0.17
Cenpj	chr14	57171774	57172282	-0.44	0.08
Ints6	chr14	63296571	63296812	-1.23	1.58
Polr3d	chr14	70842813	70842874	-0.73	-0.23
Gtf2f2	chr14	76297012	76306621	-0.38	0.02
Prkaa1	chr15	5093860	5110647	-3.53	-0.59
Prkaa1	chr15	5126670	5126850	-0.53	-0.06
Prkaa1	chr15	5128706	5129184	-0.87	0.12
Rictor	chr15	6736486	6737266	-3.47	-0.21
Rictor	chr15	6745928	6747254	-1.18	-0.11
Zfp707	chr15	75804010	75804159	-1.43	-0.84
Tnrc6b	chr15	80709443	80711723	-3.34	0.20
Tnrc6b	chr15	80759640	80760240	-0.73	-0.19
Ep300	chr15	81416643	81438767	-1.28	-0.07
Ep300	chr15	81479273	81481819	-1.93	-0.07
L3mbtl2	chr15	81516741	81518556	-1.99	-0.18
Nup50	chr15	84757805	84765852	-2.65	-0.15
Nup50	chr15	84770131	84771000	-1.89	-0.14
Arid2	chr15	96199968	96201115	-0.78	-0.06
Arid2	chr15	96223066	96232775	-1.58	0.29
Zfp740	chr15	102034168	102035505	-1.63	0.18
Zfp740	chr15	102039660	102043880	-1.11	-0.09
Sp1	chr15	102238241	102256144	-0.99	-0.04
Sp1	chr15	102261170	102261651	-0.65	-0.06
Skp2	chr15	9043329	9043510	-0.80	-0.31
Skp2	chr15	9069207	9070137	-0.35	-0.03
Zfp706	chr15	36937037	36937157	-3.06	-1.12
Taf2	chr15	54847403	54848154	-0.55	-0.07
Atad2	chr15	57966487	57966637	-2.14	-0.86
Ago2	chr15	72936518	72937656	-0.42	0.01
Ago2	chr15	73015286	73015377	-3.20	-1.28
Polr3h	chr15	81719674	81745484	-0.31	-0.02
Polr3h	chr15	81746470	81746920	-0.35	0.02
Polr3h	chr15	81755745	81756045	-0.77	0.26
Brd1	chr15	88543169	88543779	-0.31	0.01
Yaf2	chr15	93115848	93167246	-2.54	0.27
Vdr	chr15	97687183	97687273	-0.18	0.38
Prkag1	chr15	98643704	98643794	-0.63	-0.14

Zfp263	chr16	3744098	3745086	-1.38	-0.17
Zfp263	chr16	3748158	3750040	-0.38	0.01
Rmi2	chr16	10886343	10886404	-0.50	3.17
Rrn3	chr16	13813284	13813525	-0.59	-0.08
Ncbp2	chr16	31957240	31957450	-1.88	-0.32
Fyttd1	chr16	32877869	32878080	-0.83	-0.18
Fyttd1	chr16	32902586	32905812	-1.29	-0.23
Gsk3b	chr16	38089086	38090434	-2.53	-0.16
Zfp597	chr16	3865645	3871203	-2.90	0.39
Crebbp	chr16	4084047	4087743	-1.51	-0.07
Crebbp	chr16	4154961	4213404	-1.65	-0.09
Usp7	chr16	8738344	8757028	-1.22	-0.49
Gtf2e1	chr16	37510570	37510991	-0.85	-0.15
Gtf2e1	chr16	37511589	37516029	-1.29	0.20
Gtf2e1	chr16	37522998	37539795	-0.45	0.05
Nr1i2	chr16	38249474	38251285	-1.74	3.10
Runx1	chr16	92605638	92606179	-2.19	-0.21
Runx1	chr16	92697371	92697492	-0.37	-0.10
Arid1b	chr17	4995073	4995882	-1.14	-0.42
Arid1b	chr17	4996061	5040748	-2.27	-0.39
Arid1b	chr17	5332607	5337057	-0.44	0.03
Arid1b	chr17	5337146	5339263	-0.95	-0.21
Arid1b	chr17	5341548	5343285	-1.16	0.08
Zfp160	chr17	21162569	21164422	-2.96	0.26
Zfp160	chr17	21178570	21179347	-0.32	0.07
Rnps1	chr17	24551619	24552103	-0.56	-0.11
Ppard	chr17	28369728	28432393	-2.21	-0.47
Mapk14	chr17	28884007	28885350	-0.47	0.02
Brpf3	chr17	28938160	28938221	-0.31	-0.68
Brpf3	chr17	28954078	28960704	-1.02	0.17
Brpf3	chr17	28973668	28973878	-0.31	-0.05
Rxrb	chr17	34174187	34175165	-0.69	0.06
Psmb8	chr17	34335288	34335468	-2.26	-0.35
Ehmt2	chr17	35049712	35050992	-2.85	-0.23
Ddx39b	chr17	35378720	35378917	-2.01	-0.34
Ddx39b	chr17	35566011	35566699	-0.29	-0.01
Zfp213	chr17	23694297	23694714	-0.78	-0.43
Zfp213	chr17	23694951	23696378	-0.51	0.41
Thoc6	chr17	23810678	23810831	-0.28	-0.01
Mlst8	chr17	24612351	24613015	-1.70	-0.31
Ube2i	chr17	25398025	25398746	-3.13	-0.38
Taf11	chr17	28038342	28038672	-0.66	-0.10
Taf11	chr17	28042348	28045128	-2.46	-0.34

Ring1	chr17	34158095	34160251	-0.37	-0.02
Runx2	chr17	44745018	44745079	-0.41	0.19
Taf8	chr17	47625387	47626945	-3.39	-0.27
Taf8	chr17	47638265	47639236	-0.75	-0.21
Srsf7	chr17	80606178	80606645	-0.44	-0.07
Thoc1	chr18	9993386	9993537	-0.43	0.05
Rbbp8	chr18	11880308	11881165	-1.62	0.19
Taf4b	chr18	14965941	14971957	-1.84	0.42
Polr2d	chr18	31955847	31956355	-3.53	0.44
Wdr33	chr18	32049204	32067143	-0.39	0.01
Iws1	chr18	32227387	32242970	-1.95	-0.09
Ercc3	chr18	32399984	32400342	-0.44	-0.08
Ercc3	chr18	32429532	32429682	-0.45	0.08
Phax	chr18	56732606	56746980	-0.52	0.02
Prelid3a	chr18	67639310	67639460	-0.26	0.03
Rnmt	chr18	68460038	68465632	-0.35	0.01
Smad7	chr18	75527018	75527704	-1.68	0.45
Smad2	chr18	76422217	76422308	-0.97	-0.22
Smad2	chr18	76462139	76464727	-1.91	0.24
Epc1	chr18	6490659	6515906	-1.32	-0.37
Kctd1	chr18	15127940	15128061	-0.21	-0.06
Kctd1	chr18	15220279	15220489	-0.21	0.00
Kctd1	chr18	15220578	15220788	-0.20	0.00
Kctd1	chr18	15221534	15221834	-0.22	0.17
Nr3c1	chr18	39588392	39649457	-2.22	0.16
Smad4	chr18	73799858	73800038	-0.58	0.10
Smad4	chr18	73837362	73837780	-3.05	0.33
Smad4	chr18	73863156	73863395	-2.18	-1.27
Ctdp1	chr18	80642798	80647906	-0.48	0.03
Men1	chr19	6334978	6336811	-2.76	-0.24
Men1	chr19	6338843	6340864	-1.39	-0.08
Ints5	chr19	8969266	8971691	-0.31	0.00
Ints5	chr19	8971840	8972380	-0.43	0.04
Cpsf7	chr19	10614163	10620757	-1.15	-0.06
Smarca2	chr19	26705324	26705475	-0.54	-0.24
Pcgf5	chr19	36519525	36530396	-3.45	-0.52
Taf5	chr19	47145518	47149532	-0.62	-0.16
Taf5	chr19	47156922	47157312	-0.74	-0.19
Rad9a	chr19	4195437	4196226	-0.51	-0.05
Rbm14	chr19	4803491	4811634	-3.01	-0.15
Kat5	chr19	5603043	5603692	-2.39	-0.21
Kat5	chr19	5609321	5609592	-0.63	-0.14
Kat5	chr19	5609884	5610034	-1.24	-0.66

Polr2g	chr19	8872986	8873047	-0.42	-0.12
Ldb1	chr19	46107090	46108545	-1.10	-0.08
Pcgf6	chr19	47108886	47109036	-0.32	0.03
Med12	chrX	98488099	98488190	-0.95	0.27
Taf1	chrX	98794675	98794885	-0.74	-0.16
Cstf2	chrX	130609104	130609225	-0.88	0.22
Rragb	chrX	149574707	149575911	-1.22	0.28
Suv39h1	chrX	7647888	7649500	-0.85	0.14
Upf3b	chrX	34632248	34635529	-1.00	-0.19
Upf3b	chrX	34635618	34635799	-3.11	0.64
G6pdx	chrX	71655359	71655479	-1.22	-0.12
Chm	chrX	110156917	110157037	-1.01	0.43

RNA polymerase II transcription

gene_name	chr	chromStart	chromEnd	diff.lg.p	diff.log2.fc
Cnot9	chr1	74575438	74575798	-0.32	0.01
Psmd1	chr1	87961193	87961254	-0.37	-0.11
Ing5	chr1	95714871	95715891	-2.55	0.30
Ccnt2	chr1	129698762	129700561	-1.19	0.07
Rbbp5	chr1	134394590	134400982	-3.46	0.33
Rbbp5	chr1	134401131	134401282	-0.55	-0.11
Kdm5b	chr1	136514466	136514617	-1.35	0.58
Kdm5b	chr1	136521340	136521461	-0.57	-0.18
Kdm5b	chr1	136521520	136521700	-0.79	-0.26
Kdm5b	chr1	136525318	136527448	-0.82	-0.14
Kdm5b	chr1	136527777	136528227	-0.93	-0.18
Nr1i3	chr1	173148035	173148515	-0.64	0.16
Nr1i3	chr1	173148874	173149204	-1.38	-0.45
Exo1	chr1	177829710	177831466	-3.37	-0.85
Exo1	chr1	177839222	177839403	-0.90	-1.64
Parp1	chr1	182499105	182503813	-3.02	-0.53
Trp53bp2	chr1	184376701	184379234	-3.08	-0.39
Trp53bp2	chr1	184389061	184392298	-0.35	-0.03
Ints7	chr1	193443646	193445390	-0.49	-0.07
Nabp1	chr1	51523330	51523540	-1.18	1.15
Gls	chr1	52223048	52223109	-3.29	0.64
Gls	chr1	52276864	52290076	-0.98	-0.10
Sumo1	chr1	59696425	59696959	-2.60	0.18
Bard1	chr1	71121361	71122020	-3.24	-0.57
Hdac4	chr1	93829331	93829930	-2.32	-0.46
Mdm4	chr1	134888236	134893148	-1.31	0.10
Zc3h11a	chr1	135516447	135517433	-0.86	0.06
Zc3h11a	chr1	135517641	135518703	-1.96	0.10
Zc3h11a	chr1	135521340	135523811	-0.72	0.04

Rnf2	chr1	153318408	153318469	-0.48	0.17
Pou2f1	chr1	167805604	167806174	-0.37	0.04
Smyd2	chr1	191704608	191704728	-0.52	-0.07
Bmi1	chr2	18605637	18606468	-2.42	0.17
Tsc1	chr2	28531258	28532030	-1.43	-0.24
Tsc1	chr2	28541343	28542943	-2.03	-0.23
Abl1	chr2	31544075	31551320	-1.18	-0.13
Abl1	chr2	31653102	31658129	-1.05	-0.08
Mapkap1	chr2	34479153	34479423	-2.45	-0.44
Ssrp1	chr2	84885611	84887208	-3.11	-0.12
Psmc3	chr2	90894439	90895182	-2.45	-0.16
Rtf1	chr2	119558206	119559166	-1.97	-0.19
Rtf1	chr2	119560273	119560484	-0.78	-0.27
Tpx2	chr2	152719325	152719804	-0.61	0.07
Itch	chr2	154959216	154959307	-0.30	0.07
Itch	chr2	155050132	155050403	-0.62	-0.11
Phf20	chr2	156092990	156093201	-0.52	0.09
Phf20	chr2	156124429	156134013	-0.50	0.03
Tgif2	chr2	156679307	156679783	-2.73	-0.95
Src	chr2	157295196	157296561	-1.97	0.63
Rprd1b	chr2	157900751	157901171	-0.47	-0.06
Srsf6	chr2	162757243	162757817	-0.60	-0.04
Trp53rkb	chr2	166620945	166621725	-1.43	-0.33
Cstf1	chr2	172203213	172206586	-1.68	-0.11
Gata3	chr2	9779686	9779776	-0.33	0.26
Gata3	chr2	9779865	9779925	-0.37	0.52
Gata3	chr2	9779984	9780252	-0.85	0.39
Gata3	chr2	9790440	9796131	-0.26	0.02
Taf3	chr2	9837377	9862619	-2.08	-0.23
Taf3	chr2	9862678	9874561	-0.84	0.07
Ehmt1	chr2	24646742	24647852	-2.26	-0.23
Ehmt1	chr2	24733063	24775080	-1.70	0.17
Snapc4	chr2	26223896	26225494	-2.87	-0.39
Notch1	chr2	26314049	26314349	-0.81	0.70
Notch1	chr2	26315066	26315157	-0.65	-1.86
Notch1	chr2	26315335	26315486	-0.50	-0.74
Notch1	chr2	26316053	26317703	-0.30	-0.07
Notch1	chr2	26323937	26324027	-0.49	-1.14
Cdk9	chr2	32562880	32565348	-3.08	-0.10
Psmd5	chr2	34707993	34709823	-0.82	-0.10
Psmd5	chr2	34726274	34726482	-3.28	-0.70
Psmb7	chr2	38498946	38499426	-1.70	-0.24
Nr4a2	chr2	56960145	56961132	-1.31	-0.24

Nr4a2	chr2	56965686	56976354	-1.92	1.13
Clp1	chr2	84563548	84566299	-1.88	0.17
Zfp770	chr2	114021193	114021283	-0.43	0.17
Zfp661	chr2	127402341	127403505	-0.42	0.05
Snrpb	chr2	130002885	130005100	-1.37	-0.14
Psmf1	chr2	151543679	151544068	-0.32	0.02
Zfp334	chr2	165203354	165203685	-0.40	-0.13
Zfp334	chr2	165205181	165206141	-0.43	-0.06
Zfp334	chr2	165207038	165207219	-1.00	-0.50
Trp53rka	chr2	165315878	165318784	-1.13	0.14
Aurka	chr2	172182168	172182653	-0.85	0.13
Skil	chr3	30994042	30997382	-0.39	0.01
Gatad2b	chr3	90145581	90145879	-2.33	-0.23
Gatad2b	chr3	90155817	90155908	-2.67	-0.37
Hist2h2be	chr3	96025043	96025490	-0.87	-0.31
Rbm8a	chr3	96433850	96433910	-0.48	0.21
Trim33	chr3	103148225	103148495	-2.43	0.25
Ints12	chr3	132771859	132772487	-1.86	0.21
Gtf2b	chr3	142428210	142434431	-1.44	-0.33
Gtf2b	chr3	142446182	142446450	-3.59	0.37
Phc3	chr3	30805917	30813151	-0.46	0.06
Phc3	chr3	30864763	30868337	-1.50	-0.73
Maml3	chr3	51491893	51493212	-1.42	0.26
Maml3	chr3	51493600	51496170	-0.52	0.08
Maml3	chr3	51501693	51600368	-0.39	0.34
Maml3	chr3	51659540	51660829	-2.03	0.23
Maml3	chr3	51907999	51908149	-1.14	0.58
Maml3	chr3	51908418	51908808	-0.35	0.06
Ints3	chr3	90195399	90196193	-3.05	-0.26
Chtop	chr3	90303623	90304012	-0.38	0.01
Chtop	chr3	90313185	90313365	-0.85	-0.09
Psmb4	chr3	94690551	94690880	-2.31	-0.17
Rprd2	chr3	95567509	95570116	-0.33	0.00
Rprd2	chr3	95576734	95577094	-0.64	0.14
Hipk1	chr3	103547180	103548199	-0.93	-0.04
Prmt6	chr3	110050616	110051517	-1.73	-0.62
Prmt6	chr3	110052386	110053916	-3.52	-0.20
Nbn	chr4	15906479	15908877	-2.76	0.36
Rngtt	chr4	33587224	33587673	-1.48	0.31
Rngtt	chr4	33588240	33588330	-0.44	0.40
Nr4a3	chr4	48080715	48096224	-0.55	0.37
Rraga	chr4	86221576	86222293	-1.17	0.09
Tal1	chr4	114737352	114742061	-0.43	-0.04

Med8	chr4	118087470	118087618	-1.43	0.25
Scmh1	chr4	120201905	120201966	-0.42	-0.19
Rragc	chr4	123597235	123597356	-2.18	0.32
Rragc	chr4	123598193	123598284	-1.16	0.24
Lsm10	chr4	125773896	125775828	-0.48	0.04
Phc2	chr4	128404881	128405061	-0.49	0.29
Nr0b2	chr4	133112156	133112304	-1.32	-1.45
Zfp46	chr4	135846237	135848601	-0.61	-0.06
Mtor	chr4	147930392	147931764	-0.76	0.10
Ssu72	chr4	155078983	155079074	-1.02	0.85
Ssu72	chr4	155079462	155089672	-3.59	-0.27
Mllt3	chr4	87419218	87419789	-0.47	0.14
Mllt3	chr4	87486765	87487216	-0.56	-0.10
Zfp691	chr4	118842660	118845711	-0.33	0.01
Zfp691	chr4	118846309	118846770	-2.39	1.37
Zfp69	chr4	120602801	120603819	-0.86	0.27
Tfap2e	chr4	126393574	126393694	-1.54	2.92
Sesn2	chr4	132048721	132050516	-0.58	-0.04
Sfn	chr4	133157754	133158083	-0.27	-0.02
Srrm1	chr4	134878175	134878385	-1.96	-0.19
Srrm1	chr4	134902739	134909069	-0.74	0.09
Eloa	chr4	135561252	135561580	-1.33	0.22
Eloa	chr4	135565701	135577564	-0.37	0.01
Ski	chr4	154529795	154530722	-0.41	-0.02
Ski	chr4	154530781	154533563	-1.24	0.09
Ski	chr4	154533945	154596644	-3.03	-0.18
Cdk6	chr5	3519406	3520884	-1.97	-0.19
Kmt2e	chr5	22940246	22940487	-0.36	0.04
Kmt2e	chr5	23002879	23005273	-2.07	0.16
Kmt2e	chr5	23007381	23008999	-2.35	0.12
Ywhah	chr5	33361494	33361614	-0.36	0.07
Ywhah	chr5	33369299	33370047	-1.92	-0.08
Rbpj	chr5	54044875	54045116	-0.40	-0.92
Rpap2	chr5	108048973	108049573	-2.30	0.34
Zfp605	chr5	110556690	110556990	-0.28	0.01
Zfp605	chr5	110557318	110558156	-0.31	-0.02
Triap1	chr5	115791255	115793441	-0.77	0.08
Psmd9	chr5	123678228	123678437	-1.01	0.20
Kmt5a	chr5	124911266	124911327	-0.69	-0.13
Gtf2h3	chr5	125046222	125046283	-0.86	-0.51
Zfp664	chr5	125343785	125366362	-3.24	-0.19
Zfp664	chr5	125366751	125367081	-0.41	-0.05
Rfc2	chr5	135073840	135074198	-2.20	-0.32

Zkscan1	chr5	138526311	138534046	-0.28	-0.01
Zkscan1	chr5	138541944	138542455	-2.08	-0.24
Zkscan1	chr5	138544222	138545932	-1.09	0.11
Zfp12	chr5	144005811	144007821	-2.30	-0.23
Zfp12	chr5	144007940	144008390	-0.87	-0.26
Zfp12	chr5	144008869	144009619	-2.02	-0.39
Cpsf4	chr5	145939775	145942670	-0.46	0.04
Zkscan5	chr5	145979715	145981274	-2.23	0.66
Zkscan5	chr5	145981691	145982380	-0.37	-0.06
Zfp655	chr5	145992583	145994463	-1.50	-0.36
Smarcd3	chr5	24100691	24100811	-0.72	-0.86
Prkag2	chr5	24605860	24606370	-1.09	-0.18
Kmt2c	chr5	24790138	24790949	-1.48	0.18
Kmt2c	chr5	24819830	24820730	-2.10	0.18
Slbp	chr5	33992455	33994955	-2.31	0.29
Prkab1	chr5	116463747	116467999	-0.33	-0.01
Prkab1	chr5	116474139	116474378	-0.83	-0.19
Ptpn11	chr5	121581827	121582307	-1.65	0.20
Ptpn11	chr5	121583292	121587519	-2.53	-0.16
Ncor2	chr5	125538304	125548276	-0.66	-0.08
Ncor2	chr5	125659463	125659584	-0.24	-0.12
Ubc	chr5	125867967	125870328	-2.39	0.10
Zfp11	chr5	130162172	130162950	-0.33	-0.05
Zfp113	chr5	138585541	138586141	-1.27	-0.30
Zfp113	chr5	138586470	138586680	-0.76	0.33
Taf6	chr5	138619844	138620202	-3.23	-0.49
Smurf1	chr5	145637663	145637963	-1.05	-0.44
Smurf1	chr5	145638322	145638682	-0.71	-0.20
Smurf1	chr5	145639310	145639521	-0.43	0.15
Smurf1	chr5	145726518	145726669	-0.30	0.31
Zkscan14	chr5	145956021	145962455	-1.48	-0.29
Rfc3	chr5	152445398	152445638	-0.35	0.05
Cul1	chr6	47474617	47476108	-2.74	-0.21
Zfp398	chr6	47790290	47790621	-3.35	-1.10
Zfp282	chr6	47854827	47855966	-0.80	-0.11
Zfp282	chr6	47856175	47856805	-0.84	-0.17
Zfp212	chr6	47870655	47876681	-3.35	-1.03
Zfp212	chr6	47880732	47881447	-2.64	-0.48
Zfp775	chr6	48555624	48569787	-2.72	-0.92
Zfp775	chr6	48569965	48570026	-0.29	-0.39
Zfp775	chr6	48570384	48570923	-3.10	-1.25
Gata2	chr6	88154683	88155829	-0.38	-0.05
Nr2c2	chr6	92041411	92042461	-0.44	-0.16

Nr2c2	chr6	92114226	92117902	-0.73	-0.11
Chd4	chr6	125079888	125080369	-0.40	-0.01
Cdkn1b	chr6	134870958	134872360	-0.68	0.10
Gm8994	chr6	136279021	136279082	-0.20	-0.85
Gm8994	chr6	136279763	136279882	-0.28	0.95
H2afj	chr6	136757367	136757517	-1.06	-0.54
Cnot4	chr6	34973352	34974287	-0.81	0.20
Cnot4	chr6	34996066	35002891	-0.64	-0.06
Hipk2	chr6	38697991	38787107	-0.63	0.02
Hipk2	chr6	38825829	38826040	-1.39	-0.22
Zfp786	chr6	47769651	47770307	-0.93	-0.92
Zfp786	chr6	47770514	47770575	-0.14	0.00
Zfp786	chr6	47770752	47771200	-1.52	-1.37
Zfp786	chr6	47771348	47771646	-0.99	-1.29
Zfp746	chr6	48013500	48016503	-0.85	-0.09
Zfp746	chr6	48033523	48036532	-0.28	0.01
Gadd45a	chr6	66985208	66987341	-0.31	0.00
Rybp	chr6	100182244	100183112	-0.71	0.26
Zfp248	chr6	118378764	118380402	-2.34	0.42
Phc1	chr6	122275560	122275800	-2.40	-0.84
Tigar	chr6	127037468	127038307	-1.14	0.14
Ccnd2	chr6	127079526	127080245	-0.86	0.11
Rhno1	chr6	128307285	128309180	-0.40	-0.02
Kras	chr6	145168813	145168964	-1.25	-0.35
U2af2	chr7	5013783	5013844	-0.45	0.13
U2af2	chr7	5030853	5031123	-0.52	-0.04
Zfp667	chr7	6256343	6256524	-0.69	0.61
Zfp667	chr7	6257749	6257810	-0.29	0.32
Zfp78	chr7	6331550	6332238	-0.37	0.13
Zfp28	chr7	6344996	6347379	-0.53	0.10
Zfp110	chr7	13434054	13434502	-0.47	-0.06
Zfp324	chr7	13555642	13556271	-2.04	-0.62
Zfp324	chr7	13556928	13557616	-2.87	-0.66
Zfp446	chr7	13567286	13568246	-1.90	-0.38
Ercc2	chr7	19979500	19981041	-0.83	-0.08
Zfp180	chr7	24882912	24891516	-1.80	0.13
Zfp112	chr7	24910123	24910184	-1.08	-3.00
Zfp112	chr7	24910243	24910454	-0.57	-0.73
Zfp112	chr7	24910513	24910604	-0.13	-0.17
Zfp112	chr7	24910962	24911473	-0.38	0.21
Zfp112	chr7	24912431	24912551	-0.80	1.66
Zfp235	chr7	24925651	24925742	-0.66	0.68
Zfp235	chr7	24927899	24928200	-0.36	-0.20

Akt2	chr7	28422173	28423428	-0.40	0.03
Zfp30	chr7	30577880	30578810	-1.97	0.80
Zfp790	chr7	30601090	30601151	-0.45	0.29
Zfp420	chr7	30645086	30661279	-2.76	0.27
Zfp382	chr7	30916575	30919814	-1.94	0.18
Polr2i	chr7	31017332	31017529	-0.50	-0.12
Zfp658	chr7	50828078	50828289	-0.99	-0.64
Zfp658	chr7	50828587	50829455	-0.67	-0.16
Zfp658	chr7	50829663	50830293	-0.55	0.15
Zfp710	chr7	87231504	87231625	-1.34	-0.43
Zfp710	chr7	87233516	87236264	-1.90	-0.12
Lamtor1	chr7	109054380	109058241	-0.51	-0.09
Ctr9	chr7	118172674	118177414	-0.59	-0.08
Ctr9	chr7	118198901	118199651	-0.33	0.01
Setd1a	chr7	134927665	134930705	-3.33	-0.13
Setd1a	chr7	134931780	134932028	-0.75	-0.13
Zik1	chr7	11075894	11076075	-0.62	0.50
Zfp551	chr7	13000795	13000946	-0.55	-0.63
Zfp551	chr7	13001544	13001605	-0.37	0.66
Zfp551	chr7	13001783	13001874	-0.23	-0.07
Zfp551	chr7	13002053	13002203	-0.45	-0.51
Pou2f2	chr7	25876133	25878039	-1.00	-0.09
Pou2f2	chr7	25906059	25917479	-0.36	0.12
Zfp940	chr7	30629198	30629379	-2.28	-1.54
Zfp940	chr7	30630307	30630488	-0.67	-0.73
Zfp27	chr7	30678504	30680419	-1.09	0.13
Zfp27	chr7	30680807	30683850	-0.49	0.07
Zfp14	chr7	30823226	30823406	-0.82	1.24
Zfp566	chr7	30862385	30862831	-0.63	0.23
Zfp566	chr7	30863008	30863483	-0.99	0.28
Ccne1	chr7	38883180	38883329	-1.29	-0.73
Zfp473	chr7	51987570	51988080	-0.82	0.69
Zfp473	chr7	51988469	51988860	-0.49	-0.50
Med25	chr7	52132313	52133597	-2.46	-0.25
Bax	chr7	52722059	52722238	-0.71	0.19
E2f8	chr7	56123087	56123387	-2.78	-0.89
Blm	chr7	87647292	87647681	-0.45	0.09
Blm	chr7	87654437	87658340	-2.18	0.36
Eed	chr7	97129276	97129426	-0.44	0.08
Pcf11	chr7	99801691	99808380	-0.63	-0.03
Zfp747	chr7	134516438	134516647	-0.30	0.04
Zfp747	chr7	134516706	134519504	-2.94	0.29
9130019O22Rik	chr7	134526847	134529144	-0.29	0.00

E430018J23Rik	chr7	134534349	134536523	-1.20	-0.34
E430018J23Rik	chr7	134536927	134537135	-0.26	0.15
Zfp764	chr7	134547867	134549122	-1.58	-0.25
Zfp688	chr7	134562479	134563593	-2.08	-0.41
Zfp689	chr7	134587294	134588642	-2.65	-0.51
Zfp689	chr7	134592222	134592433	-0.67	0.30
Pidd1	chr7	148624413	148624744	-1.68	-0.61
Snapc2	chr8	4255341	4256161	-2.46	-0.36
Tfdp1	chr8	13377196	13377345	-0.76	-0.13
Tfdp1	chr8	13377582	13377702	-1.12	-0.18
Kat6a	chr8	24040759	24043005	-0.79	-0.08
Kat6a	chr8	24046218	24051430	-0.82	0.04
Ppp2cb	chr8	34710092	34710330	-0.63	-0.43
Gtf2e2	chr8	34886513	34887055	-0.33	0.02
Nr2c2ap	chr8	72655231	72655498	-1.07	0.29
Nr2c2ap	chr8	72657115	72657440	-0.91	-0.13
Ell	chr8	73063573	73063694	-2.62	-1.00
Ell	chr8	73107871	73114673	-3.00	-0.34
Sin3b	chr8	75247186	75248032	-1.12	0.19
Sin3b	chr8	75273726	75273966	-0.65	0.20
Sin3b	chr8	75280930	75281383	-2.20	-0.49
Nr3c2	chr8	79433507	79433776	-0.32	-0.42
Cbfb	chr8	107694632	107694812	-1.00	-0.17
Zfp90	chr8	108947880	108948329	-3.26	0.83
Zfp90	chr8	108948657	108949137	-0.47	0.12
Zfp90	chr8	108949196	108949675	-1.18	0.35
Zfp1	chr8	114190002	114194702	-0.53	-0.06
Banp	chr8	124550340	124550731	-0.77	-0.40
Banp	chr8	124550880	124551060	-0.48	-0.38
Banp	chr8	124551419	124551540	-1.16	-1.10
Banp	chr8	124552528	124552798	-1.31	-0.79
Zfpm1	chr8	124806220	124856010	-0.98	0.18
Zfpm1	chr8	124859027	124861147	-1.02	-0.10
Ash2l	chr8	26950536	26958082	-1.35	-0.23
Wrn	chr8	34346284	34346854	-0.41	-0.08
Wrn	chr8	34434722	34439590	-2.21	0.55
Cnot7	chr8	41592731	41595397	-1.59	0.21
Ing2	chr8	48753850	48754661	-0.78	0.10
Ing2	chr8	48760302	48760513	-2.46	-1.21
Gatad2a	chr8	72498029	72498150	-0.57	-0.26
Gatad2a	chr8	72519918	72520248	-0.39	0.08
Csnk2a2	chr8	97976954	97977225	-1.72	-0.27
Dhx38	chr8	112071923	112073723	-0.63	0.08

Maml2	chr9	13424522	13425092	-2.48	0.70
Maml2	chr9	13510203	13511312	-3.34	0.53
Pin1	chr9	20456573	20459888	-1.84	-0.36
Zfp202	chr9	40015019	40015258	-3.36	-0.92
Usp2	chr9	43902061	43902302	-1.62	-0.76
H2afx	chr9	44143446	44144156	-0.58	0.03
Ppp2r1b	chr9	50688020	50688710	-0.53	0.05
Ppp2r1b	chr9	50701016	50701675	-1.83	-0.33
Sin3a	chr9	56958532	56958892	-0.66	0.11
Sin3a	chr9	56973115	56975606	-1.32	0.16
Ints14	chr9	64809611	64809761	-2.80	0.75
Rora	chr9	69226745	69226835	-0.38	-0.37
Ice2	chr9	69263440	69263950	-0.69	0.12
Ice2	chr9	69280073	69280163	-0.60	-0.34
Atr	chr9	95765579	95765699	-0.73	-0.27
Tfdp2	chr9	96218608	96218729	-0.27	0.00
Ctnnb1	chr9	120842577	120859351	-2.42	-0.18
Ctnnb1	chr9	120868936	120868997	-1.38	0.13
Zkscan7	chr9	122797695	122797846	-0.50	-0.44
Zkscan7	chr9	122803837	122804973	-1.16	-0.38
Zfp558	chr9	18260716	18261377	-0.28	0.03
Zfp266	chr9	20304179	20306501	-0.43	0.03
Kmt2a	chr9	44612666	44613327	-0.91	-0.13
Kmt2a	chr9	44629634	44630384	-2.09	0.19
Kmt2a	chr9	44655514	44656204	-3.18	0.26
Kmt2a	chr9	44656473	44656924	-0.49	0.06
Pml	chr9	58067502	58068341	-3.10	-0.28
Pml	chr9	58080996	58094952	-1.77	-0.21
Pml	chr9	58097382	58097593	-1.39	-0.43
Smad3	chr9	63496369	63496909	-0.75	0.36
Smad3	chr9	63497357	63498046	-0.81	-0.34
Smad3	chr9	63605729	63605891	-0.28	-0.36
Rnf111	chr9	70274555	70277420	-1.37	0.15
Tcf12	chr9	71958831	71958892	-0.31	-0.03
Tcf12	chr9	71959385	71959626	-0.98	-0.22
Atrip	chr9	108960445	108961754	-3.54	-0.18
Atrip	chr9	108967670	108968299	-2.16	0.32
Cnot10	chr9	114541870	114549228	-1.71	-0.34
Sgk1	chr10	21718573	21718873	-1.56	0.74
Sesn1	chr10	41530409	41607263	-0.57	0.15
Sesn1	chr10	41627347	41627735	-0.58	0.32
Stk11	chr10	79579091	79587616	-1.24	-0.11
Stk11	chr10	79592650	79593215	-1.30	-0.18

Txnrd1	chr10	82296755	82323178	-0.61	0.15
Smarcc2	chr10	127920231	127920695	-2.24	0.31
Smarcc2	chr10	127926123	127926273	-0.50	0.09
Ddit4	chr10	59412629	59412689	-0.57	0.25
Nrbf2	chr10	66729823	66730867	-1.76	0.19
Ube2d1	chr10	70718174	70718562	-1.17	0.19
Smarcb1	chr10	75359631	75359751	-1.58	-0.36
Polr2e	chr10	79501286	79502404	-1.51	-0.21
Mbd3	chr10	79855285	79856691	-1.70	-0.10
Tcf3	chr10	79872416	79873225	-0.84	-0.10
Cradd	chr10	94637889	94638400	-1.23	-0.45
Cnot2	chr10	115922246	115922606	-0.97	0.18
Cnot2	chr10	115922874	115923115	-1.67	0.33
Yeats4	chr10	116652465	116652884	-1.13	0.17
Dyrk2	chr10	118296404	118305900	-0.46	0.03
Pip4k2c	chr10	126635286	126635914	-1.06	0.12
Thoc5	chr11	4799846	4799907	-2.53	-0.61
Psme4	chr11	30671804	30671925	-0.83	-0.91
Psme4	chr11	30672014	30672135	-0.38	0.12
Slu7	chr11	43247232	43250977	-0.69	-0.10
Med7	chr11	46250448	46250688	-1.24	-0.38
Ppp2ca	chr11	51935449	51935836	-1.90	0.10
Aff4	chr11	53164268	53186578	-0.79	-0.04
Aff4	chr11	53211860	53217858	-0.54	0.02
Cnot8	chr11	57930988	57931258	-1.37	0.34
Zkscan6	chr11	65636412	65642412	-0.89	-0.14
Trp53	chr11	69393860	69394608	-0.68	0.14
Ywhae	chr11	75546388	75546567	-2.12	-0.15
Suz12	chr11	79843530	79845978	-2.27	0.14
Psmd11	chr11	80285544	80285635	-1.72	-0.32
Cdk5r1	chr11	80293403	80293969	-3.03	0.92
Cdk5r1	chr11	80293392	80293962	-2.75	0.87
Taf15	chr11	83286609	83286700	-0.71	-0.14
Ppm1d	chr11	85140408	85140529	-0.31	-0.04
Ppm1d	chr11	85153180	85159827	-0.35	0.02
Supt4a	chr11	87557029	87557119	-0.77	0.39
Srsf1	chr11	87861172	87861352	-2.81	-0.34
Cdk12	chr11	98071677	98072546	-0.31	0.00
Thra	chr11	98603186	98617447	-0.55	0.14
Thra	chr11	98625630	98626259	-2.85	-0.46
Casc3	chr11	98692883	98694014	-1.36	-0.17
Rara	chr11	98799009	98808938	-1.27	-0.44
Psme3	chr11	101177564	101178336	-0.63	-0.07

Psme3	chr11	101182070	101183288	-1.70	-0.15
Cbx2	chr11	118885531	118887831	-0.67	1.03
Rptor	chr11	119465086	119517298	-0.84	0.12
Rptor	chr11	119759767	119760755	-0.49	-0.04
Hus1	chr11	8896741	8896861	-0.52	0.16
Lsm11	chr11	45747117	45747446	-1.53	-0.39
Maml1	chr11	50069972	50070390	-0.48	0.08
Maml1	chr11	50076862	50105838	-3.04	-0.21
Rad50	chr11	53463680	53465769	-1.04	-0.17
Zkscan17	chr11	59299022	59299352	-0.46	0.07
Zkscan17	chr11	59300338	59305712	-0.44	0.02
Top3a	chr11	60555827	60557475	-1.90	-0.31
Top3a	chr11	60590716	60590867	-1.91	0.89
Ncor1	chr11	62130910	62131481	-1.03	0.10
Ncor1	chr11	62147216	62147367	-0.98	0.19
Ncor1	chr11	62182894	62186997	-2.47	-0.16
Ncor1	chr11	62251972	62270948	-0.37	-0.06
Zfp287	chr11	62527143	62527774	-0.60	0.18
Zfp287	chr11	62528133	62528313	-0.31	0.07
Zfp287	chr11	62528582	62528703	-0.25	0.02
Zfp287	chr11	62541813	62542444	-0.65	-0.22
Chd3	chr11	69174956	69175626	-3.21	-0.43
Rpa1	chr11	75154547	75161885	-2.86	-0.51
Supt6	chr11	78047100	78059205	-2.14	-0.27
Med13	chr11	86100091	86100242	-0.90	-0.15
Med13	chr11	86132926	86141950	-2.79	0.16
Pip4k2b	chr11	97578204	97583775	-1.02	-0.05
Med1	chr11	98014158	98015024	-0.76	0.11
Med1	chr11	98015143	98015684	-0.34	0.01
Med1	chr11	98016102	98017333	-0.90	0.06
Med1	chr11	98017932	98018173	-0.41	-0.03
Med1	chr11	98018352	98019732	-1.77	0.10
Med1	chr11	98054124	98054607	-1.22	-0.20
Nr1d1	chr11	98629338	98629726	-1.00	0.26
Nr1d1	chr11	98631783	98632540	-3.18	-0.44
Nr1d1	chr11	98633136	98636556	-0.49	-0.06
Brca1	chr11	101384814	101385204	-1.75	-0.62
Brca1	chr11	101385473	101386581	-0.92	-0.20
Brca1	chr11	101386909	101387659	-3.36	0.64
Smurf2	chr11	106683743	106684358	-0.92	-0.20
Cbx8	chr11	118899867	118901442	-1.43	0.25
Cbx4	chr11	118940531	118940592	-1.14	1.49
Cbx4	chr11	118941070	118941400	-3.20	1.23

Cbx4	chr11	118941759	118947461	-0.50	0.02
E2f6	chr12	16832231	16832859	-1.35	0.19
Psma6	chr12	56499811	56499871	-1.67	-0.32
Ppm1a	chr12	73862227	73885470	-0.74	-0.04
Ppm1a	chr12	73894973	73895153	-0.48	0.08
Snapc1	chr12	75065516	75068947	-1.32	-0.31
Psmc1	chr12	101348377	101351286	-1.53	-0.29
Papola	chr12	107072945	107075506	-1.04	0.06
Ccnk	chr12	109418007	109424590	-0.70	-0.17
Zfp839	chr12	112106479	112107344	-0.76	-0.14
Ywhaq	chr12	21422717	21423076	-1.30	-0.20
Max	chr12	78038822	78039718	-1.25	0.06
Max	chr12	78063025	78063235	-0.95	-0.16
Ppp1r13b	chr12	113067028	113067478	-1.41	-0.33
Akt1	chr12	113912217	113912487	-0.40	0.08
Zkscan4	chr13	21576264	21576955	-0.44	-0.43
Hist1h3h	chr13	21809496	21809698	-0.75	-0.19
Hist1h3f	chr13	23636303	23636393	-1.63	-0.30
Hist1h2bg	chr13	23663268	23663474	-0.87	-0.07
Hist1h3d	chr13	23667608	23667786	-0.59	-0.11
Hist1h2bc	chr13	23776067	23776213	-1.76	0.26
Hist1h2ab	chr13	23842956	23843224	-1.74	-0.27
Rmi1	chr13	58503698	58503988	-0.71	-0.12
Med10	chr13	69954496	69954853	-0.30	0.00
Ell2	chr13	75844931	75845141	-0.97	-0.42
Taf9	chr13	101435858	101436098	-2.24	0.28
Plk2	chr13	111190024	111190991	-3.23	0.37
Slc38a9	chr13	113528239	113528690	-0.79	0.50
Cdk13	chr13	17858388	17896931	-0.44	-0.02
Zkscan3	chr13	21488092	21494295	-2.58	-0.45
Zkscan8	chr13	21611911	21613722	-1.26	-0.18
Zkscan8	chr13	21617156	21618818	-1.82	0.41
Hist1h3i	chr13	21875208	21875266	-0.36	-0.05
Hist1h3c	chr13	23837329	23837390	-0.45	0.12
Tfap2a	chr13	40812539	40812690	-0.17	0.59
Dek	chr13	47201073	47201589	-0.67	-0.13
Thoc3	chr13	54561210	54561569	-0.37	0.03
Ctsl	chr13	64470393	64471614	-2.04	-0.09
Zfp729a	chr13	67720451	67721021	-0.67	0.12
Zfp729a	chr13	67721529	67722158	-0.70	0.18
Zfp729a	chr13	67722397	67722547	-0.30	0.02
Zfp738	chr13	67768497	67769604	-3.07	0.55
Zfp738	chr13	67770112	67770352	-0.31	0.04

Zfp738	chr13	67771338	67771758	-1.04	0.44
Ice1	chr13	70741154	70747297	-2.33	-0.08
Ice1	chr13	70747386	70748030	-0.41	-0.05
Nr2f1	chr13	78328745	78328956	-1.15	-3.17
Jmy	chr13	94234651	94269644	-2.87	-0.26
Rad17	chr13	101387477	101389058	-2.53	-0.36
Psmc6	chr14	45949498	45950514	-0.68	0.11
Ints9	chr14	65658332	65658483	-0.78	-0.25
Med4	chr14	73909855	73909974	-2.48	-0.83
Med4	chr14	73917729	73918293	-0.71	-0.10
Elf1	chr14	79881389	79936053	-0.38	0.05
Elf1	chr14	79973144	79980817	-0.52	-0.03
Rarb	chr14	17407447	17451237	-0.24	0.12
Nr1d2	chr14	19044433	19047065	-1.52	1.13
Pip4p1	chr14	51545984	51546434	-1.90	0.16
Supt16	chr14	52803669	52816914	-0.75	-0.13
Prmt5	chr14	55126018	55126553	-0.37	0.02
Rabggta	chr14	56340688	56340807	-0.30	0.04
Cenpj	chr14	57145687	57148592	-0.68	-0.24
Cenpj	chr14	57170817	57171506	-0.75	-0.17
Cenpj	chr14	57171774	57172282	-0.44	0.08
Ints6	chr14	63296571	63296812	-1.23	1.58
Gtf2f2	chr14	76297012	76306621	-0.38	0.02
Prkaa1	chr15	5093860	5110647	-3.53	-0.59
Prkaa1	chr15	5126670	5126850	-0.53	-0.06
Prkaa1	chr15	5128706	5129184	-0.87	0.12
Rictor	chr15	6736486	6737266	-3.47	-0.21
Rictor	chr15	6745928	6747254	-1.18	-0.11
Zfp707	chr15	75804010	75804159	-1.43	-0.84
Ep300	chr15	81416643	81438767	-1.28	-0.07
Ep300	chr15	81479273	81481819	-1.93	-0.07
L3mbtl2	chr15	81516741	81518556	-1.99	-0.18
Arid2	chr15	96199968	96201115	-0.78	-0.06
Arid2	chr15	96223066	96232775	-1.58	0.29
Zfp740	chr15	102034168	102035505	-1.63	0.18
Zfp740	chr15	102039660	102043880	-1.11	-0.09
Sp1	chr15	102238241	102256144	-0.99	-0.04
Sp1	chr15	102261170	102261651	-0.65	-0.06
Skp2	chr15	9043329	9043510	-0.80	-0.31
Skp2	chr15	9069207	9070137	-0.35	-0.03
Zfp706	chr15	36937037	36937157	-3.06	-1.12
Taf2	chr15	54847403	54848154	-0.55	-0.07
Atad2	chr15	57966487	57966637	-2.14	-0.86

Brd1	chr15	88543169	88543779	-0.31	0.01
Yaf2	chr15	93115848	93167246	-2.54	0.27
Vdr	chr15	97687183	97687273	-0.18	0.38
Prkag1	chr15	98643704	98643794	-0.63	-0.14
Zfp263	chr16	3744098	3745086	-1.38	-0.17
Zfp263	chr16	3748158	3750040	-0.38	0.01
Rmi2	chr16	10886343	10886404	-0.50	3.17
Ncbp2	chr16	31957240	31957450	-1.88	-0.32
Fyttd1	chr16	32877869	32878080	-0.83	-0.18
Fyttd1	chr16	32902586	32905812	-1.29	-0.23
Zfp597	chr16	3865645	3871203	-2.90	0.39
Crebbp	chr16	4084047	4087743	-1.51	-0.07
Crebbp	chr16	4154961	4213404	-1.65	-0.09
Usp7	chr16	8738344	8757028	-1.22	-0.49
Gtf2e1	chr16	37510570	37510991	-0.85	-0.15
Gtf2e1	chr16	37511589	37516029	-1.29	0.20
Gtf2e1	chr16	37522998	37539795	-0.45	0.05
Nr1i2	chr16	38249474	38251285	-1.74	3.10
Runx1	chr16	92605638	92606179	-2.19	-0.21
Runx1	chr16	92697371	92697492	-0.37	-0.10
Arid1b	chr17	4995073	4995882	-1.14	-0.42
Arid1b	chr17	4996061	5040748	-2.27	-0.39
Arid1b	chr17	5332607	5337057	-0.44	0.03
Arid1b	chr17	5337146	5339263	-0.95	-0.21
Arid1b	chr17	5341548	5343285	-1.16	0.08
Zfp160	chr17	21162569	21164422	-2.96	0.26
Zfp160	chr17	21178570	21179347	-0.32	0.07
Rnps1	chr17	24551619	24552103	-0.56	-0.11
Ppard	chr17	28369728	28432393	-2.21	-0.47
Mapk14	chr17	28884007	28885350	-0.47	0.02
Brpf3	chr17	28938160	28938221	-0.31	-0.68
Brpf3	chr17	28954078	28960704	-1.02	0.17
Brpf3	chr17	28973668	28973878	-0.31	-0.05
Rxrb	chr17	34174187	34175165	-0.69	0.06
Psmb8	chr17	34335288	34335468	-2.26	-0.35
Ehmt2	chr17	35049712	35050992	-2.85	-0.23
Ddx39b	chr17	35378720	35378917	-2.01	-0.34
Ddx39b	chr17	35566011	35566699	-0.29	-0.01
Zfp213	chr17	23694297	23694714	-0.78	-0.43
Zfp213	chr17	23694951	23696378	-0.51	0.41
Thoc6	chr17	23810678	23810831	-0.28	-0.01
Mlst8	chr17	24612351	24613015	-1.70	-0.31
Ube2i	chr17	25398025	25398746	-3.13	-0.38

Taf11	chr17	28038342	28038672	-0.66	-0.10
Taf11	chr17	28042348	28045128	-2.46	-0.34
Ring1	chr17	34158095	34160251	-0.37	-0.02
Runx2	chr17	44745018	44745079	-0.41	0.19
Taf8	chr17	47625387	47626945	-3.39	-0.27
Taf8	chr17	47638265	47639236	-0.75	-0.21
Srsf7	chr17	80606178	80606645	-0.44	-0.07
Thoc1	chr18	9993386	9993537	-0.43	0.05
Rbbp8	chr18	11880308	11881165	-1.62	0.19
Taf4b	chr18	14965941	14971957	-1.84	0.42
Polr2d	chr18	31955847	31956355	-3.53	0.44
Wdr33	chr18	32049204	32067143	-0.39	0.01
Iws1	chr18	32227387	32242970	-1.95	-0.09
Ercc3	chr18	32399984	32400342	-0.44	-0.08
Ercc3	chr18	32429532	32429682	-0.45	0.08
Phax	chr18	56732606	56746980	-0.52	0.02
Prelid3a	chr18	67639310	67639460	-0.26	0.03
Rnmt	chr18	68460038	68465632	-0.35	0.01
Smad7	chr18	75527018	75527704	-1.68	0.45
Smad2	chr18	76422217	76422308	-0.97	-0.22
Smad2	chr18	76462139	76464727	-1.91	0.24
Epc1	chr18	6490659	6515906	-1.32	-0.37
Kctd1	chr18	15127940	15128061	-0.21	-0.06
Kctd1	chr18	15220279	15220489	-0.21	0.00
Kctd1	chr18	15220578	15220788	-0.20	0.00
Kctd1	chr18	15221534	15221834	-0.22	0.17
Nr3c1	chr18	39588392	39649457	-2.22	0.16
Smad4	chr18	73799858	73800038	-0.58	0.10
Smad4	chr18	73837362	73837780	-3.05	0.33
Smad4	chr18	73863156	73863395	-2.18	-1.27
Ctdp1	chr18	80642798	80647906	-0.48	0.03
Men1	chr19	6334978	6336811	-2.76	-0.24
Men1	chr19	6338843	6340864	-1.39	-0.08
Ints5	chr19	8969266	8971691	-0.31	0.00
Ints5	chr19	8971840	8972380	-0.43	0.04
Cpsf7	chr19	10614163	10620757	-1.15	-0.06
Smarca2	chr19	26705324	26705475	-0.54	-0.24
Pcgf5	chr19	36519525	36530396	-3.45	-0.52
Taf5	chr19	47145518	47149532	-0.62	-0.16
Taf5	chr19	47156922	47157312	-0.74	-0.19
Rad9a	chr19	4195437	4196226	-0.51	-0.05
Rbm14	chr19	4803491	4811634	-3.01	-0.15
Kat5	chr19	5603043	5603692	-2.39	-0.21

Kat5	chr19	5609321	5609592	-0.63	-0.14
Kat5	chr19	5609884	5610034	-1.24	-0.66
Polr2g	chr19	8872986	8873047	-0.42	-0.12
Ldb1	chr19	46107090	46108545	-1.10	-0.08
Pcgf6	chr19	47108886	47109036	-0.32	0.03
Med12	chrX	98488099	98488190	-0.95	0.27
Taf1	chrX	98794675	98794885	-0.74	-0.16
Cstf2	chrX	130609104	130609225	-0.88	0.22
Rragb	chrX	149574707	149575911	-1.22	0.28
Upf3b	chrX	34632248	34635529	-1.00	-0.19
Upf3b	chrX	34635618	34635799	-3.11	0.64
G6pdx	chrX	71655359	71655479	-1.22	-0.12
Chm	chrX	110156917	110157037	-1.01	0.43

Immune system

gene_name	chr	chromStart	chromEnd	diff.lg.p	diff.log2.fc
Il1rl1	chr1	40518643	40519152	-1.17	-0.31
Il18rap	chr1	40605514	40606142	-1.93	0.43
Stat1	chr1	52216218	52216729	-2.88	-0.31
Casp8	chr1	58890568	58901676	-0.30	0.00
Abi2	chr1	60527596	60534244	-1.45	0.18
Cd28	chr1	60826604	60826815	-0.22	-0.36
Cd28	chr1	60827743	60827984	-0.70	-1.34
Slc11a1	chr1	74421776	74422382	-3.48	-0.58
Slc11a1	chr1	74432056	74432625	-0.70	0.06
Cab39	chr1	87690021	87715038	-1.14	-0.12
Cab39	chr1	87745789	87746418	-1.09	-0.07
Psmd1	chr1	87961193	87961254	-0.37	-0.11
Eif4e2	chr1	89110518	89110878	-0.75	-0.16
Inpp5d	chr1	89516916	89517096	-1.01	-0.27
Inpp5d	chr1	89614465	89616815	-0.79	-0.06
Ube2f	chr1	93182033	93182602	-1.08	-0.09
Asb1	chr1	93440810	93443711	-1.60	-0.49
Asb1	chr1	93451373	93451734	-0.52	0.13
Tnfrsf11a	chr1	107677299	107704793	-1.56	0.68
Arl8a	chr1	137043410	137043590	-2.14	-0.35
Ncf2	chr1	154654984	154655314	-0.34	0.01
Rnasel	chr1	155600751	155602337	-1.96	-0.29
Rnasel	chr1	155610213	155610334	-0.42	-0.24
Creg1	chr1	167693910	167694001	-0.45	-0.10
Tmem63a	chr1	182904339	182904638	-1.16	-0.26
Hecw2	chr1	53869614	53883756	-1.23	1.77
Hecw2	chr1	53982375	53983924	-0.31	-0.08
Sumo1	chr1	59696425	59696959	-2.60	0.18

Tmbim1	chr1	74335180	74335241	-2.07	-0.51
Tuba4a	chr1	75211607	75211936	-0.33	0.05
Tuba4a	chr1	75212113	75215828	-2.13	-0.09
Ap1s3	chr1	79668486	79668547	-0.31	-0.11
Cul3	chr1	80336855	80337005	-0.41	-0.05
Trip12	chr1	84782695	84836815	-0.84	-0.03
Pdcd1	chr1	95937874	95949130	-2.07	-1.60
Bcl2	chr1	108438384	108439674	-0.90	0.22
Bcl2	chr1	108439763	108608408	-0.87	0.54
Bcl2	chr1	108608737	108610507	-1.27	-0.20
Bcl2	chr1	108610626	108610867	-0.58	0.39
Ptpn4	chr1	121733557	121733648	-0.97	-0.74
Ikbke	chr1	133151178	133153534	-2.21	-0.20
Dhx9	chr1	155303276	155303397	-3.17	0.68
Qsox1	chr1	157626448	157628411	-2.42	-0.35
Klhl20	chr1	163019047	163019258	-2.02	-0.55
Klhl20	chr1	163020426	163023835	-3.23	0.59
Klhl20	chr1	163039327	163039897	-0.46	0.06
Fcgr2b	chr1	172906081	172906200	-0.31	0.02
Fcgr3	chr1	172981537	172981597	-1.79	-0.50
Degs1	chr1	184206857	184208760	-3.23	0.22
Ppp2r5a	chr1	193176577	193176847	-1.42	0.15
Ppp2r5a	chr1	193196363	193220920	-1.78	-0.16
Il15ra	chr2	11654900	11655469	-1.69	0.44
Commd3	chr2	18596461	18597638	-0.32	-0.01
Il1rn	chr2	24205157	24205248	-0.60	-0.54
Anapc2	chr2	25128463	25128941	-1.02	-0.11
Fbxw5	chr2	25358984	25359776	-1.40	-0.18
Fbxw5	chr2	25360013	25360422	-1.11	-0.22
Rapgef1	chr2	29475329	29475390	-0.74	-0.73
Rapgef1	chr2	29557821	29558001	-0.64	-0.09
Rapgef1	chr2	29592963	29594536	-3.14	-0.17
Abl1	chr2	31544075	31551320	-1.18	-0.13
Abl1	chr2	31653102	31658129	-1.05	-0.08
Ptges2	chr2	32257786	32257964	-3.07	-0.78
Mapkap1	chr2	34479153	34479423	-2.45	-0.44
Hspa5	chr2	34629598	34629837	-1.43	0.04
Hspa5	chr2	34630750	34631960	-2.77	-0.03
Tank	chr2	61416642	61431255	-0.74	-0.21
Tank	chr2	61482442	61484290	-0.30	-0.01
Tank	chr2	61484409	61488467	-1.76	0.12
Tank	chr2	61491449	61491629	-0.53	0.07
Klhl41	chr2	69508654	69508893	-1.20	0.67

Dync1i2	chr2	71049792	71050012	-1.03	0.40
Itgav	chr2	83643777	83643958	-1.72	-0.33
Psmc3	chr2	90894439	90895182	-2.45	-0.16
Traf6	chr2	101531587	101537959	-2.12	0.17
Kif18a	chr2	109173521	109173672	-0.98	-0.57
Trim69	chr2	122004226	122004674	-0.56	-1.46
Sirpa	chr2	129418574	129418993	-2.78	0.40
Sirpa	chr2	129419320	129457247	-0.96	-0.02
Pygb	chr2	150654268	150656555	-0.44	0.05
Hck	chr2	152934263	152934443	-1.07	0.34
Hck	chr2	152976907	152977087	-0.51	-0.07
Kif3b	chr2	153141983	153146011	-2.34	0.20
Kif3b	chr2	153155890	153156639	-1.20	0.17
Kif3b	chr2	153158285	153158915	-0.67	0.12
Itch	chr2	154959216	154959307	-0.30	0.07
Itch	chr2	155050132	155050403	-0.62	-0.11
Src	chr2	157295196	157296561	-1.97	0.63
Mmp9	chr2	164780329	164780659	-0.85	-0.58
Cd40	chr2	164896238	164896475	-0.37	-0.13
Cd40	chr2	164896681	164896977	-0.48	0.36
Rnf114	chr2	167318622	167318832	-0.88	-1.10
Ptpn1	chr2	167800939	167802433	-1.33	-0.16
Ptpn1	chr2	167802821	167803599	-3.44	-0.45
Tubb1	chr2	174282844	174283054	-0.17	0.13
Abi1	chr2	22895609	22895730	-1.85	-0.40
Tubb4b	chr2	25077855	25080192	-0.38	-0.01
Dpp7	chr2	25211792	25211852	-1.75	0.43
Traf2	chr2	25374427	25375145	-1.67	-0.19
Card9	chr2	26212789	26213529	-0.79	0.09
Agpat2	chr2	26449106	26450082	-0.51	-0.12
Vav2	chr2	27282074	27282345	-1.98	-1.07
Lrsam1	chr2	32781993	32782383	-0.52	-0.14
Fbxw2	chr2	34661048	34661869	-2.35	-0.23
Fbxw2	chr2	34662794	34664040	-0.82	-0.21
Fbxw2	chr2	34668436	34678672	-0.41	-0.02
Fbxw2	chr2	34681468	34681725	-0.52	0.16
Psmd5	chr2	34707993	34709823	-0.82	-0.10
Psmd5	chr2	34726274	34726482	-3.28	-0.70
Psmb7	chr2	38498946	38499426	-1.70	-0.24
Wipf1	chr2	73270330	73270480	-0.29	0.00
Wipf1	chr2	73275357	73278120	-3.10	-0.24
Wipf1	chr2	73282586	73291856	-0.45	0.07
Atf2	chr2	73656977	73657038	-1.83	0.77

Ptprj	chr2	90420444	90420774	-0.85	-0.18
Atp8b4	chr2	126148354	126148594	-0.41	0.05
Anapc1	chr2	128436537	128438637	-0.86	0.07
Il1a	chr2	129126416	129126507	-0.23	0.11
Il1b	chr2	129195984	129196856	-1.15	2.95
Ubox5	chr2	130416996	130417596	-0.43	0.07
Ubox5	chr2	130425355	130426375	-3.53	-0.43
Siglec1	chr2	130895224	130895524	-0.41	-0.15
Siglec1	chr2	130900602	130901900	-0.28	0.00
Siglec1	chr2	130903954	130904467	-0.53	0.19
Siglec1	chr2	130907034	130909110	-0.62	-0.22
Siglec1	chr2	130912201	130912381	-0.76	0.65
Cd93	chr2	148263313	148263433	-0.69	4.00
Cd93	chr2	148266721	148267597	-0.52	1.75
Cd93	chr2	148267925	148268195	-0.71	3.50
Cd93	chr2	148268851	148269062	-0.38	1.48
Psmf1	chr2	151543679	151544068	-0.32	0.02
Trib3	chr2	152163160	152164409	-0.93	0.07
Trib3	chr2	152169617	152169796	-0.42	-0.11
Bcl2l1	chr2	152606912	152607242	-0.45	0.04
Bcl2l1	chr2	152656237	152657418	-2.87	-0.78
Cpne1	chr2	155922158	155937671	-0.31	0.00
Dsn1	chr2	156821512	156821930	-1.54	-0.43
Elmo2	chr2	165115177	165116717	-2.78	-0.28
Elmo2	chr2	165117584	165117675	-2.59	-1.26
Nfatc2	chr2	168301999	168303229	-1.92	-0.20
Fabp5	chr3	10015061	10015506	-2.68	-0.28
Pld1	chr3	28029315	28031326	-1.86	-0.40
Pld1	chr3	28031774	28031835	-0.53	-0.67
Pik3ca	chr3	32335072	32335372	-0.45	-0.08
Pik3ca	chr3	32360681	32362022	-3.50	0.35
Rap2b	chr3	61171125	61171306	-0.74	-0.36
Ctso	chr3	81756174	81758885	-1.40	0.23
Fbxw7	chr3	84619498	84694070	-0.58	0.12
Fbxw7	chr3	84694129	84756226	-2.67	0.26
Fbxw7	chr3	84780140	84781955	-0.67	0.08
Shc1	chr3	89222472	89225594	-0.41	-0.06
Shc1	chr3	89232244	89232484	-0.39	0.03
Ube2q1	chr3	89577560	89577890	-1.31	-0.19
Ube2q1	chr3	89585705	89587919	-2.90	0.11
Ilf2	chr3	90291521	90291822	-1.69	-0.21
Them4	chr3	94135497	94135946	-0.95	0.23
Ctss	chr3	95330707	95333452	-1.96	-0.10

Rnf115	chr3	96593799	96594008	-1.03	-0.13
Vav3	chr3	109143660	109143900	-1.03	-0.68
Cenpe	chr3	134902767	134903916	-0.98	0.27
Cenpe	chr3	134905051	134905504	-0.46	0.08
Cenpe	chr3	134910323	134910534	-0.46	0.09
Cenpe	chr3	134936264	134936504	-0.62	0.21
Manba	chr3	135230676	135233888	-0.32	0.01
Bcl10	chr3	145587262	145587592	-1.35	-0.17
Bcl10	chr3	145588099	145593586	-0.30	0.00
Il7	chr3	7573784	7573845	-0.14	-0.32
Pag1	chr3	9691860	9692940	-2.76	-0.45
Nhlrc3	chr3	53255917	53257521	-3.38	-0.42
Siah2	chr3	58479649	58495411	-0.38	-0.02
Siah2	chr3	58495680	58495891	-0.39	-0.11
Dhx36	chr3	62274511	62275295	-0.82	-0.16
Tlr2	chr3	83640193	83645500	-1.21	0.04
Il6ra	chr3	89673305	89675305	-2.83	-0.24
Psmb4	chr3	94690551	94690880	-2.31	-0.17
Cd101	chr3	100797451	100797632	-1.28	-1.85
Csf1	chr3	107544324	107544444	-0.74	0.65
Csf1	chr3	107544802	107548597	-0.55	0.11
Csf1	chr3	107548745	107550765	-0.94	-1.24
Csf1	chr3	107551063	107552051	-0.42	0.07
Sec24b	chr3	129686102	129687101	-1.10	-0.09
Dapp1	chr3	137595557	137598550	-1.00	-0.29
Prkacb	chr3	146394723	146394993	-0.71	0.12
Map3k7	chr4	32051081	32051232	-1.86	-0.83
Map3k7	chr4	32110141	32110231	-1.49	-0.76
Ube2r2	chr4	41137807	41138136	-0.82	-0.10
Atp6v1g1	chr4	63205828	63209679	-0.73	0.11
Trim32	chr4	65274556	65274646	-0.66	-0.27
Pgm2	chr4	99602085	99602234	-1.37	-0.46
Lrrc41	chr4	115768961	115769201	-3.42	-0.29
Csf3r	chr4	125720790	125721238	-1.32	-0.36
Ptafr	chr4	132119981	132136781	-0.79	0.06
Fgr	chr4	132530039	132542068	-0.98	0.34
Fgr	chr4	132557289	132557469	-2.85	0.87
Fuca1	chr4	135495027	135495325	-1.89	0.27
Eif4g3	chr4	137676266	137682738	-0.87	-0.04
Eif4g3	chr4	137762176	137762386	-3.36	0.46
Ddost	chr4	137868046	137868406	-0.32	-0.01
Capzb	chr4	138748845	138794826	-1.20	-0.15
Casp9	chr4	141352424	141352694	-2.96	-0.57

Casp9	chr4	141368153	141369672	-0.74	0.33
Casp9	chr4	141369761	141369941	-0.56	1.17
Mtor	chr4	147930392	147931764	-0.76	0.10
Slc2a5	chr4	149494002	149494152	-0.29	1.46
Klhl21	chr4	151383838	151389959	-1.59	-0.17
Klhl21	chr4	151390257	151390408	-2.24	0.94
Ufl1	chr4	25177588	25177679	-0.29	-0.02
Ddx58	chr4	40152603	40155517	-1.09	-0.14
Ddx58	chr4	40186678	40186858	-0.63	0.23
B4galt1	chr4	40753786	40754830	-2.74	0.20
B4galt1	chr4	40770597	40800583	-1.25	0.19
B4galt1	chr4	40800821	40800971	-2.38	-0.70
Vcp	chr4	43013259	43013349	-0.47	-0.15
Fbxo10	chr4	45048105	45048374	-0.54	-0.64
Fbxo10	chr4	45048672	45048852	-0.34	0.51
Fbxo10	chr4	45071353	45071981	-0.56	-0.37
Cdc26	chr4	62055609	62055968	-0.83	0.27
Alad	chr4	62170203	62171204	-0.29	0.00
Ifnb1	chr4	88167987	88168698	-0.97	2.98
Jun	chr4	94716292	94718913	-1.94	0.04
Jak1	chr4	100825566	100826046	-1.09	0.11
Kif2c	chr4	116832539	116832928	-2.15	-0.69
Rnf220	chr4	116961136	116968749	-1.18	-0.20
Lck	chr4	129225826	129225977	-1.80	-0.75
Txlna	chr4	129305539	129309456	-2.39	-0.11
Txlna	chr4	129316526	129317724	-1.08	-0.08
Rps6ka1	chr4	133403354	133404010	-1.09	-0.10
Tnfrsf8	chr4	144858878	144864193	-0.32	-0.12
Fbxo6	chr4	147520122	147520183	-0.29	0.01
Pik3cd	chr4	149023545	149025880	-1.73	-0.13
Spsb1	chr4	149271976	149281277	-0.37	-0.15
Kcnab2	chr4	151766373	151767149	-2.14	-0.15
Mib2	chr4	155028700	155028761	-1.11	-1.14
Atad3a	chr4	155114748	155115286	-0.82	-0.07
Ube3c	chr5	29895781	29895962	-1.11	-0.74
Ube3c	chr5	30001057	30001298	-0.35	0.03
Bst1	chr5	44210161	44210281	-0.88	-0.46
Anapc4	chr5	53257704	53257973	-1.19	-0.20
Klhl5	chr5	65558241	65558691	-1.07	-0.19
Cxcl3	chr5	91215158	91215370	-0.42	-0.69
Stbd1	chr5	93034080	93034200	-0.12	0.00
Ube3b	chr5	114869623	114869922	-2.59	-0.34
Fbxo21	chr5	118452093	118458648	-2.38	-0.37

Hvcn1	chr5	122690237	122692276	-1.11	-0.20
Anapc7	chr5	122891328	122891508	-1.48	-0.33
Psmd9	chr5	123678228	123678437	-1.01	0.20
Atp6v0a2	chr5	125200361	125202517	-1.22	0.17
Kctd7	chr5	130628217	130628755	-1.29	-0.61
Arpc1b	chr5	145875124	145883438	-3.09	-0.16
Tnip2	chr5	34839311	34839491	-0.39	0.07
Tnip2	chr5	34842145	34842355	-1.03	0.23
Fbxl5	chr5	44142150	44149098	-0.30	0.01
Fbxl5	chr5	44149396	44151063	-1.01	0.09
Tlr1	chr5	65316072	65316462	-1.84	0.58
Tlr1	chr5	65317358	65317538	-0.27	0.02
Tlr1	chr5	65318106	65318495	-0.63	0.23
Atp8a1	chr5	68013273	68013783	-1.25	0.26
Tec	chr5	73147081	73148633	-1.81	-0.24
Rchy1	chr5	92391795	92392094	-2.32	0.64
Sec31a	chr5	100790907	100791058	-1.68	-0.19
Mlec	chr5	115597834	115600228	-0.81	-0.07
Fbxw8	chr5	118515497	118515767	-0.43	-0.12
Ptpn11	chr5	121581827	121582307	-1.65	0.20
Ptpn11	chr5	121583292	121587519	-2.53	-0.16
Anapc5	chr5	123271138	123271348	-1.70	-0.25
Rhof	chr5	123567437	123567766	-1.20	-0.45
Ubc	chr5	125867967	125870328	-2.39	0.10
Slc15a4	chr5	128076540	128080872	-0.31	0.00
Gusb	chr5	130464890	130465519	-2.75	0.10
Trim56	chr5	137587784	137588144	-0.80	-0.16
Trim56	chr5	137588275	137588336	-0.64	0.56
Pilra	chr5	138276667	138276728	-0.74	1.17
Card11	chr5	141348952	141350301	-2.19	-0.58
Fbxl18	chr5	143640471	143644134	-2.38	-0.99
Rac1	chr5	144288651	144288861	-1.33	0.22
Smurf1	chr5	145637663	145637963	-1.05	-0.44
Smurf1	chr5	145638322	145638682	-0.71	-0.20
Smurf1	chr5	145639310	145639521	-0.43	0.15
Smurf1	chr5	145726518	145726669	-0.30	0.31
Rnf6	chr5	147021822	147022092	-0.81	0.13
Rnf6	chr5	147023104	147032058	-0.38	-0.01
Atp6v1f	chr6	29417782	29417841	-1.46	-0.23
Cul1	chr6	47474617	47476108	-2.74	-0.21
Znrf2	chr6	54838538	54838599	-0.34	-0.02
Herc6	chr6	57531001	57531152	-0.51	-0.34
Dctn1	chr6	83140226	83141007	-1.23	-0.14

Arpc4	chr6	113328136	113330743	-0.46	0.06
Vhl	chr6	113578147	113579978	-1.22	0.20
Fbxl14	chr6	119429685	119431421	-0.82	0.06
Il17ra	chr6	120413244	120413425	-1.58	-0.49
Il17ra	chr6	120433025	120433146	-1.60	-0.40
Usp18	chr6	121195923	121196013	-1.05	-1.68
Clec4d	chr6	123212154	123212273	-2.02	0.39
C1ra	chr6	124472382	124472951	-1.99	-0.43
C1rb	chr6	124530824	124530943	-0.36	0.74
Gm8994	chr6	136279021	136279082	-0.20	-0.85
Gm8994	chr6	136279763	136279882	-0.28	0.95
Lrmp	chr6	145086263	145086444	-0.85	-0.32
Klhl42	chr6	147039984	147056940	-0.79	-0.10
Impdh1	chr6	29162071	29162252	-2.44	-0.43
Ube2h	chr6	30163827	30164395	-0.70	-0.09
Ube2h	chr6	30164783	30164963	-0.64	0.31
Clec5a	chr6	40527283	40529441	-0.30	0.00
Nod1	chr6	54893317	54893527	-0.62	1.79
Nod1	chr6	54894659	54894899	-0.40	-0.75
Vamp8	chr6	72335214	72335477	-0.41	-0.03
Vamp8	chr6	72338403	72340632	-1.83	-0.34
Kcmf1	chr6	72849172	72849233	-0.31	-0.52
Kcmf1	chr6	72849822	72849973	-0.27	0.15
Rbsn	chr6	92139135	92140425	-0.38	0.02
Raf1	chr6	115568590	115569280	-0.54	-0.05
Alox5	chr6	116410986	116411166	-1.01	-0.30
Slc2a3	chr6	122679681	122680131	-0.97	-0.19
C1s1	chr6	124481017	124481078	-0.14	-0.42
C1s1	chr6	124481405	124481466	-0.11	0.00
C1s2	chr6	124575330	124575809	-0.33	1.51
Ptpn6	chr6	124683045	124688519	-0.44	0.09
Ltbr	chr6	125256618	125258152	-0.36	-0.01
Olr1	chr6	129436974	129437604	-0.52	1.98
Kras	chr6	145168813	145168964	-1.25	-0.35
Itpr2	chr6	146060001	146060981	-1.32	0.19
Ap2s1	chr7	17323792	17328621	-1.80	-0.22
Akt2	chr7	28422173	28423428	-0.40	0.03
Tyrobp	chr7	31198806	31198866	-0.60	-0.05
Irf3	chr7	52253029	52253209	-0.42	-0.09
Irf3	chr7	52257067	52257801	-2.15	-0.33
Cyfip1	chr7	63109393	63127258	-0.43	-0.06
Ube3a	chr7	66484119	66496353	-3.29	0.33
Klhl25	chr7	82993313	83012167	-0.53	-0.05

Klhl25	chr7	83018746	83018926	-0.98	-0.56
Ctsc	chr7	95437274	95437455	-0.26	0.00
Ctsc	chr7	95458037	95458487	-2.08	0.25
Prcp	chr7	100081684	100082313	-0.64	-0.10
Ndufc2	chr7	104548571	104548690	-0.85	0.14
Lamtor1	chr7	109054380	109058241	-0.51	-0.09
Ampd3	chr7	117954502	117954682	-3.55	0.65
Dctn5	chr7	129276554	129277689	-0.62	0.13
Dctn5	chr7	129291850	129292499	-2.82	0.17
Rbbp6	chr7	130114077	130114468	-1.85	-0.17
Rbbp6	chr7	130140585	130140736	-1.81	-0.24
Il4ra	chr7	132719062	132720501	-3.11	-0.43
Il4ra	chr7	132720800	132721370	-2.64	0.59
Il21r	chr7	132776155	132776515	-0.40	-0.35
Fbxl19	chr7	134911014	134911817	-1.61	-0.24
Fbxl19	chr7	134911935	134912442	-2.06	-0.46
Stx4a	chr7	134985380	134985840	-3.36	-0.34
Itgax	chr7	135292687	135294081	-3.59	1.65
Lair1	chr7	3961115	3961265	-1.04	-0.76
Il11	chr7	4724965	4727794	-3.33	0.80
Il11	chr7	4727991	4729743	-1.71	-0.76
Ube2m	chr7	13623108	13623619	-2.32	-0.37
C5ar2	chr7	16822087	16822178	-0.28	-0.26
C5ar2	chr7	16822715	16822896	-1.32	-1.43
C5ar2	chr7	16823104	16823255	-0.32	0.53
Calm3	chr7	17502039	17502309	-2.51	-0.14
Klc3	chr7	19979788	19981184	-1.39	-0.21
Relb	chr7	20191570	20192258	-0.92	-0.11
Nectin2	chr7	20302531	20302921	-0.73	-0.39
Nectin2	chr7	20306812	20310098	-0.79	-0.21
Nectin2	chr7	20334682	20334892	-0.51	-0.23
Pvr	chr7	20490268	20494550	-0.53	0.08
Ceacam1	chr7	26247769	26248608	-3.38	0.29
Ceacam1	chr7	26256984	26258859	-1.12	-0.38
Ceacam1	chr7	26259008	26274241	-1.35	0.18
Cd22	chr7	31662583	31662644	-0.18	-0.74
Siglece	chr7	50915321	50915471	-0.80	0.91
Flt3l	chr7	52386558	52386888	-2.06	0.77
Asb7	chr7	73792504	73823980	-1.38	-0.19
Det1	chr7	85972688	85972809	-1.30	-0.59
Det1	chr7	85988116	85988896	-0.39	0.03
Anpep	chr7	86966748	86966927	-0.81	-0.13
Anpep	chr7	86986085	86993080	-3.53	-0.12

Iqgap1	chr7	87858478	87858659	-1.28	-0.06
Inppl1	chr7	108985423	108986317	-1.03	-0.23
Inppl1	chr7	108986589	108986740	-2.25	1.44
Trim21	chr7	109707151	109708560	-3.22	0.40
Sh2b1	chr7	133610925	133611549	-2.04	-0.35
Sh2b1	chr7	133618136	133618435	-2.21	-0.61
Kif22	chr7	134179351	134185934	-1.83	-0.72
Ifitm2	chr7	148141799	148141860	-1.53	-0.15
Hras	chr7	148376758	148378177	-0.33	-0.01
Hras	chr7	148378805	148379903	-1.48	0.51
Fadd	chr7	151765572	151768221	-1.92	-0.22
Map2k7	chr8	4245576	4246251	-2.64	-0.23
Tnfsf13b	chr8	10035462	10035731	-1.56	0.69
Atp11a	chr8	12757015	12757255	-0.36	0.24
Atp11a	chr8	12864987	12866454	-1.34	0.14
Lamp1	chr8	13159223	13159402	-1.74	-0.18
Cdc16	chr8	13779263	13781763	-1.50	-0.16
Plpp5	chr8	26834581	26835060	-0.38	-0.03
Ppp2cb	chr8	34710092	34710330	-0.63	-0.43
Casp3	chr8	47702790	47715112	-2.57	1.25
Sh3rf1	chr8	63703117	63808042	-0.87	0.26
Sh3rf1	chr8	63863646	63863797	-0.27	0.10
Myo9b	chr8	73796612	73839062	-0.95	0.06
Myo9b	chr8	73882022	73884611	-1.43	0.07
Prkaca	chr8	86496876	86500855	-3.14	-0.78
Man2b1	chr8	87607167	87607377	-1.30	-0.24
Cyld	chr8	91220956	91221047	-0.33	-0.20
Fbxl8	chr8	107791952	107793137	-2.37	-0.45
Nfatc3	chr8	108602601	108603618	-1.85	0.13
Nfatc3	chr8	108632465	108652166	-1.29	0.10
Ap1g1	chr8	112382745	112384686	-0.52	0.03
Znrf1	chr8	114060539	114062246	-1.56	-0.12
Znrf1	chr8	114143157	114145711	-0.85	-0.11
Znrf1	chr8	114145770	114146010	-0.96	0.20
Znrf1	chr8	114146219	114147028	-1.23	-0.08
Plcg2	chr8	120022190	120028105	-0.37	-0.04
Tubb3	chr8	125944632	125944960	-0.21	-0.02
Itgb1	chr8	131209643	131209853	-0.47	-0.04
Ikbkb	chr8	23770546	23770997	-0.43	-0.04
Golga7	chr8	24352876	24353057	-1.98	-0.24
Hgsnat	chr8	27083350	27087156	-0.46	0.09
Dctn6	chr8	35169406	35171536	-0.90	0.26
Lonrf1	chr8	37280167	37282962	-0.49	0.09

Tlr3	chr8	46482427	46484485	-1.03	0.28
Klhl2	chr8	67219421	67219870	-1.56	-0.30
Pik3r2	chr8	73292079	73292528	-3.00	-0.41
Pik3r2	chr8	73298580	73300551	-1.20	-0.17
Siah1a	chr8	89248786	89269816	-0.55	0.06
Dync1li2	chr8	106966676	106966737	-0.30	-0.05
Hp	chr8	112099087	112099805	-2.48	-0.74
Il34	chr8	113266113	113266292	-0.28	1.28
Cotl1	chr8	122364299	122364479	-1.03	0.15
Fbxo31	chr8	124075878	124076177	-0.56	0.18
Galns	chr8	125102315	125103030	-1.46	-0.23
Casp1	chr9	5298664	5299458	-1.72	0.32
Pin1	chr9	20456573	20459888	-1.84	-0.36
Icam1	chr9	20820403	20823609	-1.32	-0.17
Icam1	chr9	20824057	20824596	-2.71	0.54
Icam1	chr9	20831648	20833180	-0.80	0.03
Icam5	chr9	20839000	20839269	-0.89	0.59
Icam5	chr9	20840869	20842061	-0.40	0.09
Dnm2	chr9	21310781	21311349	-0.34	0.01
Dnm2	chr9	21311898	21312198	-3.13	0.36
Ppp2r1b	chr9	50688020	50688710	-0.53	0.05
Ppp2r1b	chr9	50701016	50701675	-1.83	-0.33
Fbxo22	chr9	55066270	55071642	-1.39	0.12
Fbxo22	chr9	55071791	55072031	-0.79	0.18
Pstpip1	chr9	55937782	55937902	-1.36	-0.40
Adam10	chr9	70526807	70526958	-0.76	0.19
Adam10	chr9	70626086	70626267	-1.49	0.28
Rab27a	chr9	72892731	72908786	-0.70	-1.27
Rab27a	chr9	72943119	72943840	-0.62	-0.22
Myo5a	chr9	74919042	74919403	-0.47	0.08
Anapc13	chr9	102528625	102536370	-0.39	-0.04
Pik3r4	chr9	105546522	105551238	-3.59	0.32
Pik3r4	chr9	105552647	105556359	-0.68	0.10
Pik3r4	chr9	105589445	105589895	-1.34	0.26
Dusp7	chr9	106270962	106271201	-1.93	-1.11
Dusp7	chr9	106271586	106276865	-0.38	-0.01
Dcaf1	chr9	106739021	106740882	-1.41	0.32
Dcaf1	chr9	106760607	106761940	-2.76	0.33
Dcaf1	chr9	106776529	106782364	-0.77	0.23
Nckipsd	chr9	108714765	108714915	-1.56	-0.69
Glb1	chr9	114310242	114326084	-1.04	0.28
Glb1	chr9	114368770	114373539	-0.71	0.08
Dync1li1	chr9	114632686	114632895	-0.48	0.07

Ctnnb1	chr9	120842577	120859351	-2.42	-0.18
Ctnnb1	chr9	120868936	120868997	-1.38	0.13
Ccr2	chr9	124021097	124021367	-1.86	-0.98
Birc2	chr9	7818494	7818943	-1.37	0.33
Birc2	chr9	7834955	7835225	-0.88	0.29
Birc3	chr9	7848939	7850928	-0.65	0.09
Birc3	chr9	7860281	7872961	-1.11	0.10
Panx1	chr9	14810228	14812439	-0.40	-0.04
Fbxl12	chr9	20448880	20449151	-1.08	-0.58
Keap1	chr9	21034503	21042276	-2.14	0.12
Keap1	chr9	21043235	21043776	-0.68	-0.28
Tirap	chr9	34994185	34994305	-0.27	0.01
Tirap	chr9	34994483	34996852	-1.16	0.12
Crtam	chr9	40781146	40781531	-0.39	-0.40
Cbl	chr9	44041829	44042099	-1.98	-0.42
Nlrx1	chr9	44060795	44061574	-0.93	-0.29
Nlrx1	chr9	44064954	44068731	-0.40	-0.37
Nlrx1	chr9	44070466	44072299	-0.30	-0.01
Ube4a	chr9	44731538	44732048	-2.54	0.32
Il10ra	chr9	45062190	45062579	-1.86	0.43
Il10ra	chr9	45063175	45068489	-2.21	0.18
Chrnb4	chr9	54883115	54883236	-0.29	1.46
Arih1	chr9	59302235	59325571	-1.19	0.37
Pias1	chr9	62729583	62729943	-1.17	0.27
Pias1	chr9	62799649	62828686	-1.72	0.23
Rnf111	chr9	70274555	70277420	-1.37	0.15
Fbxo9	chr9	77943550	77943611	-0.20	0.01
Tmem30a	chr9	79617076	79617405	-1.31	0.16
Armc8	chr9	99380819	99381090	-0.59	0.18
Armc8	chr9	99420671	99420882	-2.06	0.72
Il20rb	chr9	100358674	100359660	-0.51	-0.25
Apeh	chr9	107987744	107987894	-0.37	-0.08
Arih2	chr9	108522434	108547287	-1.40	0.26
Trim71	chr9	114422150	114422300	-0.59	3.58
Myd88	chr9	119248949	119249158	-0.83	-0.18
Fuca2	chr10	13222977	13225667	-1.46	0.28
Ifngr1	chr10	19329041	19329761	-0.36	0.02
Frk	chr10	34203714	34203864	-0.26	0.90
Frk	chr10	34327775	34328373	-0.21	-0.81
Fyn	chr10	39089604	39089964	-2.08	-0.38
Fyn	chr10	39271197	39284162	-0.31	0.00
Psap	chr10	59740405	59754400	-0.37	-0.02
Itgb2	chr10	76993092	77005145	-2.27	-0.27

Ube2g2	chr10	77107280	77107637	-1.28	-0.14
Ube2g2	chr10	77108022	77108082	-0.37	-0.06
Icosl	chr10	77536549	77536670	-0.36	-0.19
Arhgap45	chr10	79485025	79488219	-1.43	-0.13
Arhgap45	chr10	79493111	79494216	-2.35	-0.14
Eef2	chr10	80644273	80645254	-1.14	-0.02
Eea1	chr10	95499325	95499386	-0.41	-0.05
Eea1	chr10	95502273	95504101	-1.18	-0.12
Eea1	chr10	95504160	95504884	-0.30	0.01
Cep290	chr10	100034774	100035631	-1.17	0.53
Gns	chr10	120802145	120802355	-0.34	0.03
Gns	chr10	120832206	120832746	-2.71	0.13
Arhgap9	chr10	126759908	126761407	-1.07	0.40
Stat6	chr10	127080041	127080222	-2.27	-0.32
Stat6	chr10	127096737	127097564	-1.15	-0.06
Stat2	chr10	127727880	127728797	-0.81	-0.11
Stat2	chr10	127729335	127729635	-1.70	-0.31
Rnf41	chr10	127874437	127875799	-0.76	-0.07
Tab2	chr10	7625983	7626463	-0.30	0.00
Tab2	chr10	7626701	7629919	-0.81	-0.05
Tab2	chr10	7630969	7644574	-2.87	-0.09
Tnfaip3	chr10	18721581	18725108	-0.69	-0.06
Ube2d1	chr10	70718174	70718562	-1.17	0.19
Rnf126	chr10	79229508	79229686	-1.66	-0.45
Fzr1	chr10	80829742	80830217	-2.52	0.24
Ckap4	chr10	83990001	83991399	-0.32	0.00
Ckap4	chr10	83996275	83996633	-1.47	-0.37
Irak3	chr10	119579488	119580238	-0.33	0.01
Kif5a	chr10	126700029	126700180	-1.47	-0.63
Ap1b1	chr11	4941834	4942313	-0.40	-0.03
Dbnl	chr11	5688485	5691845	-3.37	-0.49
Peli1	chr11	20991326	20991536	-0.55	-0.13
Psme4	chr11	30671804	30671925	-0.83	-0.91
Psme4	chr11	30672014	30672135	-0.38	0.12
Stk10	chr11	32433265	32433386	-0.63	0.22
Fbxw11	chr11	32644536	32645016	-2.21	0.20
Mapk9	chr11	49696697	49697026	-1.50	0.25
Ppp2ca	chr11	51935449	51935836	-1.90	0.10
Gm2a	chr11	54923031	54923210	-0.87	-0.15
Trim11	chr11	58791617	58791916	-3.37	-0.52
Trim11	chr11	58802336	58802515	-0.53	0.13
Trim11	chr11	58802842	58804483	-2.09	-0.17
Trim11	chr11	58804542	58804960	-2.11	0.29

2310033P09Rik	chr11	59022495	59023911	-0.43	0.04
Nlrp3	chr11	59379889	59380129	-0.80	-0.15
Map2k3	chr11	60765438	60766245	-3.57	-0.18
Vamp2	chr11	68902059	68902686	-1.04	-0.39
Ube2g1	chr11	72493612	72497325	-2.70	0.54
Crk	chr11	75492811	75517946	-0.68	-0.03
Crk	chr11	75518934	75519324	-1.20	0.15
Nos2	chr11	78734360	78734481	-0.28	0.42
Psmd11	chr11	80285544	80285635	-1.72	-0.32
Mpo	chr11	87611071	87613383	-0.49	2.58
Wipf2	chr11	98725061	98752118	-1.36	-0.28
Stat5a	chr11	100737679	100737740	-1.24	2.22
Stat5a	chr11	100737889	100738040	-2.39	2.12
Psme3	chr11	101177564	101178336	-0.63	-0.07
Psme3	chr11	101182070	101183288	-1.70	-0.15
Map3k3	chr11	106013617	106016298	-1.58	0.15
Baiap2	chr11	119867827	119868096	-0.54	0.08
Nf2	chr11	4748990	4749260	-0.77	-0.15
Dock2	chr11	34127102	34128632	-0.54	-0.03
Dock2	chr11	34546949	34597325	-2.61	0.24
Cyfip2	chr11	46050820	46050881	-0.14	-0.26
Cyfip2	chr11	46051389	46051569	-0.67	1.95
Trim41	chr11	48629367	48630893	-1.35	-0.11
Sec24a	chr11	51569959	51570170	-2.57	-1.70
Csf2	chr11	54060889	54061185	-0.66	-1.67
Atox1	chr11	55264107	55274640	-1.13	-0.14
Mapk7	chr11	61306993	61307412	-1.70	-0.35
Map2k4	chr11	65503265	65507000	-2.15	-0.17
Nlrp1b	chr11	70966603	70969541	-0.48	-0.10
Nlrp1b	chr11	70995251	70995342	-0.44	-0.27
Nlrp1b	chr11	70996028	70996238	-1.60	-0.87
Wsb1	chr11	79053599	79053898	-1.62	-0.11
Ube2z	chr11	95911494	95911704	-3.10	-0.34
Kpnb1	chr11	97049025	97049206	-3.31	-0.43
Fbxl20	chr11	97950510	97953462	-0.36	0.03
Ormdl3	chr11	98443291	98443381	-1.34	0.67
Ormdl3	chr11	98443826	98444154	-1.62	-0.36
Klhl11	chr11	100333798	100334096	-1.09	0.31
Rab5c	chr11	100581475	100599444	-1.38	-0.37
Ghdc	chr11	100630466	100630763	-0.63	0.22
Dusp3	chr11	101834513	101835259	-2.71	0.14
Dusp3	chr11	101835527	101835855	-2.15	-0.15
Dusp3	chr11	101835944	101836213	-0.54	-0.05

Map3k14	chr11	103081077	103082247	-2.79	-0.40
Map3k14	chr11	103100406	103100995	-3.03	-0.56
Map3k14	chr11	103103496	103128715	-0.28	0.01
Cd79b	chr11	106172654	106173044	-1.64	3.90
Smurf2	chr11	106683743	106684358	-0.92	-0.20
Cd300e	chr11	114914519	114914670	-0.30	0.16
Grb2	chr11	115505517	115505876	-0.46	0.05
Grb2	chr11	115506621	115508033	-1.62	0.09
Ube2o	chr11	116399663	116399932	-0.71	0.17
Ube2o	chr11	116400140	116404120	-1.25	-0.09
Ube2o	chr11	116405905	116405995	-0.32	0.03
Cant1	chr11	118268408	118271504	-0.49	-0.03
Kif3c	chr12	3403323	3403414	-2.27	-0.91
Kif3c	chr12	3405476	3405866	-0.46	0.05
Sdc1	chr12	8778201	8778382	-1.50	-0.70
Sdc1	chr12	8798545	8799295	-1.00	-0.19
Psma6	chr12	56499811	56499871	-1.67	-0.32
Lrr1	chr12	70275442	70276002	-0.27	0.05
Psen1	chr12	85072709	85075730	-1.54	-0.08
Psmc1	chr12	101348377	101351286	-1.53	-0.29
Calm1	chr12	101445209	101445479	-1.73	0.08
Dync1h1	chr12	111839634	111839815	-0.77	-0.17
Dync1h1	chr12	111855964	111857506	-0.91	0.03
Dync1h1	chr12	111904592	111905154	-0.63	0.04
Traf3	chr12	112499736	112500694	-0.44	0.03
Klc1	chr12	112997059	113010574	-1.27	-0.23
Pld4	chr12	113998865	113999998	-0.41	0.04
Pld4	chr12	114000237	114001663	-0.54	0.07
Btbd6	chr12	114215550	114217061	-3.22	-0.34
Sos2	chr12	70685554	70689696	-0.63	-0.04
Sos2	chr12	70782599	70782839	-0.62	-0.54
Ppp2r5e	chr12	76616497	76694287	-1.19	0.16
Ppp2r5e	chr12	76696737	76697187	-3.02	-0.33
Atp6v1d	chr12	79962444	79962595	-0.40	-0.06
Arel1	chr12	86259755	86261519	-1.15	-0.07
Rps6ka5	chr12	101789241	101791305	-3.25	-0.84
Akt1	chr12	113912217	113912487	-0.40	0.08
Elmo1	chr13	20692056	20697377	-0.39	0.02
Hist1h3h	chr13	21809496	21809698	-0.75	-0.19
Hist1h3f	chr13	23636303	23636393	-1.63	-0.30
Hist1h3d	chr13	23667608	23667786	-0.59	-0.11
Ripk1	chr13	34119794	34126112	-1.97	0.13
Dsp	chr13	38287748	38287928	-0.38	0.81

Rnf182	chr13	43763512	43763603	-0.50	3.17
Rnf144b	chr13	47338305	47340700	-1.37	-0.32
Rnf144b	chr13	47342881	47342942	-0.24	0.19
Rnf144b	chr13	47343000	47343181	-1.76	-1.16
Syk	chr13	52737300	52738605	-0.48	-0.06
Syk	chr13	52738964	52742902	-1.56	-0.08
Syk	chr13	52743830	52743951	-1.80	-0.43
Faf2	chr13	54723144	54723235	-0.40	0.17
Faf2	chr13	54757723	54763688	-0.38	-0.01
Lpcat1	chr13	73651356	73651986	-0.47	0.09
Erap1	chr13	74777319	74783985	-2.44	0.26
Il6st	chr13	113254277	113255963	-0.68	-0.20
Il6st	chr13	113296618	113296739	-0.43	0.09
Cdk13	chr13	17858388	17896931	-0.44	-0.02
Hist1h3i	chr13	21875208	21875266	-0.36	-0.05
Hist1h3c	chr13	23837329	23837390	-0.45	0.12
Tubb2b	chr13	34219290	34219557	-1.15	1.09
Tubb2b	chr13	34219999	34221135	-0.49	0.22
Txndc5	chr13	38592171	38592559	-0.55	0.04
Txndc5	chr13	38620243	38620304	-0.32	0.03
Hk3	chr13	55107346	55107674	-3.38	-0.24
Hk3	chr13	55122245	55122665	-0.55	0.15
Rab24	chr13	55421293	55421501	-1.79	-0.26
Rab24	chr13	55422955	55423341	-2.38	-0.32
Dok3	chr13	55624595	55625593	-1.57	-0.09
Dok3	chr13	55628788	55629086	-1.40	-0.17
Ddx41	chr13	55637396	55637930	-2.55	-0.37
Ctsl	chr13	64470393	64471614	-2.04	-0.09
Scamp1	chr13	94973830	94978130	-2.09	-0.34
Pik3r1	chr13	102454016	102456264	-1.08	0.13
Pik3r1	chr13	102478932	102525656	-0.51	0.13
Pik3r1	chr13	102527214	102538172	-1.57	0.16
Map3k1	chr13	112538703	112540665	-1.60	0.22
Map3k1	chr13	112545624	112546521	-0.86	-0.09
Map3k1	chr13	112566138	112599191	-2.43	-0.66
Flnb	chr14	8650500	8650591	-0.44	0.13
Flnb	chr14	8737140	8739810	-0.50	0.02
Flnb	chr14	8782901	8784101	-0.35	0.01
Kctd6	chr14	9052713	9055546	-1.19	-0.22
Nkiras1	chr14	19111014	19112745	-0.34	0.03
Plau	chr14	21661560	21662161	-0.63	-0.04
Vcl	chr14	21748654	21748715	-1.30	-0.59
Psmc6	chr14	45949498	45950514	-0.68	0.11

Pnp	chr14	51564155	51567563	-2.20	0.60
Pnp	chr14	51571126	51571721	-2.41	0.19
Irf9	chr14	56222821	56223460	-0.61	0.20
Irf9	chr14	56223519	56224000	-1.33	-0.43
Kbtbd7	chr14	79827126	79828716	-0.67	0.13
Ppp3cb	chr14	21320302	21327131	-1.02	-0.09
Ppp3cb	chr14	21365614	21365735	-0.60	-0.29
Camk2g	chr14	21554783	21555022	-1.32	0.47
Camk2g	chr14	21555081	21555948	-0.48	-0.05
Prkcd	chr14	31408778	31411699	-1.32	-0.09
Prkcd	chr14	31411787	31411937	-0.35	-0.04
Prkcd	chr14	31439303	31439394	-1.67	-0.55
Tspan14	chr14	41720841	41721322	-0.90	-0.19
Fbxl3	chr14	103482030	103488661	-0.97	0.07
Fbxl3	chr14	103491640	103498726	-2.05	0.22
Rictor	chr15	6736486	6737266	-3.47	-0.21
Rictor	chr15	6745928	6747254	-1.18	-0.11
Ncf4	chr15	78075240	78075447	-1.52	-0.21
Ncf4	chr15	78091438	78093010	-2.87	-0.16
Tab1	chr15	79989200	79992132	-3.29	-0.26
Ep300	chr15	81416643	81438767	-1.28	-0.07
Ep300	chr15	81479273	81481819	-1.93	-0.07
Xrcc6	chr15	81870214	81870424	-0.78	-0.17
Ano6	chr15	95621303	95661131	-1.66	-0.31
Mettl7a1	chr15	100143493	100143673	-0.64	0.57
Skp2	chr15	9043329	9043510	-0.80	-0.31
Skp2	chr15	9069207	9070137	-0.35	-0.03
Rnf19a	chr15	36170916	36173866	-0.57	0.06
Fbxo32	chr15	58011932	58012112	-0.37	-0.51
Fbxo32	chr15	58012410	58013069	-1.76	-1.01
Fbxo32	chr15	58046282	58046433	-1.44	-1.83
Ptk2	chr15	73035624	73039772	-0.52	-0.04
Csf2rb2	chr15	78112969	78113060	-1.96	0.70
Csf2rb2	chr15	78114648	78115039	-2.56	-0.44
Csf2rb2	chr15	78115098	78116449	-0.69	-0.10
Il2rb	chr15	78311757	78312087	-1.25	4.00
Il2rb	chr15	78312236	78312356	-0.40	2.32
Pla2g6	chr15	79116836	79117224	-0.65	-0.14
Nfam1	chr15	82828225	82828824	-1.06	-0.14
Arsa	chr15	89303025	89303862	-1.00	-0.17
Arsa	chr15	89305988	89306615	-2.33	-0.41
Col2a1	chr15	97806181	97807085	-2.85	2.87
Asb8	chr15	97965969	97971774	-0.40	-0.02

Racgap1	chr15	99470224	99473919	-3.35	-0.72
Itgb7	chr15	102046603	102046957	-1.86	-0.16
Itgb7	chr15	102057968	102062366	-0.36	-0.03
Gpr84	chr15	103138665	103140869	-0.78	-0.04
Hmox2	chr16	4764613	4765867	-0.51	0.03
Mapk1	chr16	17038671	17038882	-0.44	0.05
Snap29	chr16	17427202	17428502	-2.91	0.37
Crkl	chr16	17452109	17484552	-0.65	0.04
Ap2m1	chr16	20535576	20537801	-0.67	-0.19
Eif4g1	chr16	20679306	20680085	-2.29	0.06
Il1rap	chr16	26714958	26715289	-1.33	0.48
Itgb5	chr16	33829750	33830079	-1.89	-0.82
Itgb5	chr16	33919922	33920281	-0.73	-0.11
Itgb5	chr16	33947843	33949245	-0.62	-0.07
Cd80	chr16	38486386	38486895	-0.99	-0.15
Cd80	chr16	38487013	38494806	-3.34	0.87
Cd200r1	chr16	44794312	44794493	-1.45	-0.86
Cd200r4	chr16	44811897	44820870	-2.46	-1.49
Cd200r2	chr16	44915270	44915805	-0.75	-0.67
Cblb	chr16	52204595	52205075	-0.66	-0.17
Ifnar2	chr16	91399551	91405652	-1.71	0.14
Il10rb	chr16	91425363	91425632	-0.46	-0.04
Ifnar1	chr16	91503665	91506361	-3.27	-0.21
Ifnar1	chr16	91506810	91507680	-0.37	0.02
Ifngr2	chr16	91563142	91563652	-2.12	-0.13
Crebbp	chr16	4084047	4087743	-1.51	-0.07
Crebbp	chr16	4154961	4213404	-1.65	-0.09
Socs1	chr16	10783990	10785629	-0.29	0.03
Cpped1	chr16	11804530	11827393	-3.40	0.27
Cpped1	chr16	11828497	11828677	-0.40	-0.08
Cpped1	chr16	11887837	11909516	-3.47	-0.88
Pak2	chr16	32019832	32021909	-3.04	0.15
Dtx3l	chr16	35928994	35932046	-0.87	0.09
Dtx3l	chr16	35932315	35933572	-1.20	0.10
Cd86	chr16	36604193	36604492	-0.58	-0.63
Cd86	chr16	36620826	36620887	-0.49	0.89
Cct8	chr16	87496053	87496114	-1.39	0.33
Brwd1	chr16	96223575	96225611	-1.11	-0.08
Brwd1	chr16	96229450	96229631	-0.85	0.39
Rps6ka2	chr17	7374493	7374644	-1.21	0.96
Rps6ka2	chr17	7506914	7507424	-0.39	-0.14
Itpr3	chr17	27258568	27258749	-1.05	-0.28
Mapk14	chr17	28884007	28885350	-0.47	0.02

H2-DMa	chr17	34272232	34272650	-2.02	0.70
Tap1	chr17	34333659	34333780	-0.87	-0.27
Psmb8	chr17	34335288	34335468	-2.26	-0.35
Neu1	chr17	35071556	35072840	-0.43	0.02
H2-D1	chr17	35400807	35401046	-2.46	-0.23
H2-Q2	chr17	35479307	35479659	-0.13	0.17
H2-Q6	chr17	35565949	35566691	-0.29	0.01
Nfkbie	chr17	45697418	45700117	-2.30	-0.19
Cul7	chr17	46788096	46788277	-3.52	5.23
Cul7	chr17	46788546	46788937	-0.63	-0.57
Cul7	chr17	46790989	46791170	-0.24	0.13
Trem1	chr17	48376164	48376373	-0.47	-1.34
Trem1	chr17	48383770	48384842	-0.66	0.48
Treml2	chr17	48439323	48439534	-0.18	0.36
Treml2	chr17	48449130	48449580	-0.30	-0.03
Ebi3	chr17	56095665	56096445	-2.71	-0.27
Tnfsf9	chr17	57244807	57244898	-1.14	-0.35
Tnfsf9	chr17	57245676	57247180	-0.42	0.01
Vav1	chr17	57418522	57418613	-1.00	-0.44
Vav1	chr17	57467106	57467554	-0.56	0.05
Ypel5	chr17	73198557	73198888	-0.59	0.14
Rasgrp3	chr17	75924352	75926570	-0.37	0.01
Rasgrp3	chr17	75928063	75928184	-1.00	-0.25
Ppm1b	chr17	85357671	85357732	-0.37	-0.15
Ppm1b	chr17	85373436	85402617	-3.08	0.18
Prkce	chr17	86567124	86568262	-0.44	-0.06
Prkce	chr17	87053997	87055374	-0.59	-0.12
Socs5	chr17	87520136	87534893	-3.18	0.20
Dynlt1f	chr17	6306339	6306549	-0.36	0.20
Igf2r	chr17	12876409	12876980	-2.88	-0.19
Lnpep	chr17	17715497	17746283	-1.25	0.05
Fpr1	chr17	18013552	18020874	-0.21	-0.18
Mmp25	chr17	23782026	23782176	-0.64	3.81
Atp6v0c	chr17	24306196	24306374	-0.89	-0.16
Ccnf	chr17	24360684	24360924	-0.81	0.23
Mlst8	chr17	24612351	24613015	-1.70	-0.31
Hspa1a	chr17	35107196	35107464	-1.02	3.56
Hspa1a	chr17	35108505	35108744	-1.36	5.33
Lta	chr17	35340462	35340551	-0.59	-0.80
Trim39	chr17	36397225	36398550	-1.71	0.22
Enpp4	chr17	44234667	44235237	-0.30	0.05
Klc4	chr17	46781254	46782971	-0.90	-0.29
Ppp2r5d	chr17	46820058	46820802	-0.99	-0.12

Ppp2r5d	chr17	46837029	46841951	-0.82	-0.21
Ubr2	chr17	47147240	47147481	-2.49	-0.41
Tubb4a	chr17	57220052	57220528	-0.75	-0.24
Tubb4a	chr17	57220705	57221152	-0.66	-0.22
Tubb4a	chr17	57227085	57227175	-0.30	0.20
Tnfsf14	chr17	57329363	57329424	-0.27	-1.58
Tnfsf14	chr17	57329482	57330022	-0.26	0.40
Tnfsf14	chr17	57330230	57330291	-0.42	-2.81
Pja2	chr17	64658065	64658455	-0.40	-0.03
Pja2	chr17	64658514	64660528	-0.38	0.03
Vapa	chr17	65962685	65962895	-2.37	-0.25
Rab31	chr17	66001065	66001632	-0.94	0.07
Rab31	chr17	66002078	66002287	-3.48	-0.28
Rab31	chr17	66122031	66122092	-0.23	0.08
Xdh	chr17	74233606	74233756	-0.51	-0.15
Eif2ak2	chr17	79251889	79252337	-0.63	0.09
Eif2ak2	chr17	79277606	79281912	-1.21	-0.27
Sos1	chr17	80797100	80799097	-0.65	-0.07
Sos1	chr17	80833039	80834290	-0.49	-0.07
Sos1	chr17	80879343	80879554	-0.37	-0.28
Calm2	chr17	87835011	87846216	-1.79	-0.13
Fbxo11	chr17	88390945	88391656	-2.00	0.16
Fbxo11	chr17	88464415	88464625	-2.23	-0.69
Colec12	chr18	9707645	9840283	-0.41	-0.02
Colec12	chr18	9848135	9859890	-2.84	0.07
Ube2d2a	chr18	35931212	35931392	-0.74	-0.18
Ube2d2a	chr18	35965390	35965540	-0.95	0.36
Rnf14	chr18	38467690	38467990	-1.19	0.25
Dctn4	chr18	60686409	60686560	-0.23	-0.01
Dctn4	chr18	60716345	60716496	-0.32	-0.02
Camk2a	chr18	61144517	61145265	-0.99	-0.43
Csf1r	chr18	61265471	61269328	-1.21	0.08
Tubb6	chr18	67550384	67552330	-2.55	-0.26
Mex3c	chr18	73732718	73749293	-3.27	-0.38
Mex3c	chr18	73749352	73750612	-0.69	-0.04
Map3k8	chr18	4348929	4352951	-0.72	-0.15
Rock1	chr18	10181251	10181790	-0.29	0.00
Cdc23	chr18	34793500	34793621	-0.34	-0.05
Tmem173	chr18	35893749	35894258	-1.04	-0.15
Trim36	chr18	46326899	46328816	-2.13	0.74
Ticam2	chr18	46718782	46719052	-1.30	-0.50
Ticam2	chr18	46719441	46720699	-0.89	-0.13
Nfatc1	chr18	80803003	80803394	-3.39	-0.87

Nfatc1	chr18	80803872	80832717	-0.36	0.02
Nfatc1	chr18	80846232	80846653	-2.89	0.45
Nfatc1	chr18	80879052	80904865	-0.59	-0.05
Unc93b1	chr19	3935215	3935774	-0.37	0.02
Unc93b1	chr19	3936437	3936737	-0.41	0.03
Sptbn2	chr19	4744144	4744488	-3.16	-1.29
Jak2	chr19	29385463	29386732	-1.91	0.26
Pdcd1lg2	chr19	29545180	29545271	-0.76	-2.35
Il33	chr19	30033410	30033650	-0.36	1.61
Btrc	chr19	45604177	45604358	-1.03	2.52
Nfkb2	chr19	46379346	46380320	-0.71	0.19
Fbxl15	chr19	46403649	46404936	-1.75	-0.27
Tcirg1	chr19	3906859	3907103	-2.15	-0.38
Tbc1d10c	chr19	4184415	4184595	-0.49	-0.45
Peli3	chr19	4931973	4932510	-0.93	-0.92
Sipa1	chr19	5651184	5651544	-1.35	0.18
Sipa1	chr19	5653976	5655118	-2.76	-0.25
Sipa1	chr19	5659601	5662646	-2.78	-0.18
Stx3	chr19	11893652	11893893	-0.43	0.15
Dtx4	chr19	12543910	12544151	-2.93	-0.29
Fbxw4	chr19	45652836	45653256	-0.43	0.04
Actr1a	chr19	46452376	46452913	-1.36	-0.09
Ddx3x	chrX	12858147	12861347	-2.40	0.16
Pgrmc1	chrX	34138515	34138605	-3.07	0.49
Ube2a	chrX	34414360	34414509	-1.29	0.25
Tab3	chrX	82860492	82860673	-1.77	0.62
Kif4	chrX	97921953	97922371	-0.51	0.14
Was	chrX	7667556	7667617	-0.56	-0.25
Irak1	chrX	71260386	71261911	-1.24	0.14
Dnase1l1	chrX	71526935	71526996	-0.63	-0.37
Eda2r	chrX	94533994	94534294	-0.47	0.34
Pja1	chrX	96661699	96661909	-2.14	0.49
Pja1	chrX	96662087	96663013	-1.28	0.13
Pja1	chrX	96663132	96663849	-0.65	-0.09
Magt1	chrX	103207027	103207148	-2.06	0.47
Gla	chrX	131135394	131135514	-1.45	0.53
Siah1b	chrX	160509608	160510052	-0.28	-0.01
Tlr8	chrX	163680663	163681230	-0.68	0.77
Tlr8	chrX	163681497	163681617	-0.19	0.13
Tlr8	chrX	163681973	163682213	-0.46	-0.65
Tlr8	chrX	163683254	163683344	-0.41	1.28
Tlr8	chrX	163683522	163683672	-0.98	2.22
Tlr7	chrX	163745757	163746354	-1.39	0.09

Stim1	chr7	109416755	109416875	-0.27	-0.02
Dynll2	chr11	87794645	87799923	-0.33	-0.01
Dynll2	chr11	87800072	87801030	-2.21	-0.32
Diaph1	chr18	38094885	38095065	-1.72	-0.48

Generic transcription pathway

gene_name	chr	chromStart	chromEnd	diff.lg.p	diff.log2.fc
Cnot9	chr1	74575438	74575798	-0.32	0.01
Psmd1	chr1	87961193	87961254	-0.37	-0.11
Ing5	chr1	95714871	95715891	-2.55	0.30
Ccnt2	chr1	129698762	129700561	-1.19	0.07
Rbbp5	chr1	134394590	134400982	-3.46	0.33
Rbbp5	chr1	134401131	134401282	-0.55	-0.11
Kdm5b	chr1	136514466	136514617	-1.35	0.58
Kdm5b	chr1	136521340	136521461	-0.57	-0.18
Kdm5b	chr1	136521520	136521700	-0.79	-0.26
Kdm5b	chr1	136525318	136527448	-0.82	-0.14
Kdm5b	chr1	136527777	136528227	-0.93	-0.18
Nr1i3	chr1	173148035	173148515	-0.64	0.16
Nr1i3	chr1	173148874	173149204	-1.38	-0.45
Exo1	chr1	177829710	177831466	-3.37	-0.85
Exo1	chr1	177839222	177839403	-0.90	-1.64
Parp1	chr1	182499105	182503813	-3.02	-0.53
Trp53bp2	chr1	184376701	184379234	-3.08	-0.39
Trp53bp2	chr1	184389061	184392298	-0.35	-0.03
Gls	chr1	52223048	52223109	-3.29	0.64
Gls	chr1	52276864	52290076	-0.98	-0.10
Sumo1	chr1	59696425	59696959	-2.60	0.18
Bard1	chr1	71121361	71122020	-3.24	-0.57
Hdac4	chr1	93829331	93829930	-2.32	-0.46
Mdm4	chr1	134888236	134893148	-1.31	0.10
Rnf2	chr1	153318408	153318469	-0.48	0.17
Smyd2	chr1	191704608	191704728	-0.52	-0.07
Bmi1	chr2	18605637	18606468	-2.42	0.17
Tsc1	chr2	28531258	28532030	-1.43	-0.24
Tsc1	chr2	28541343	28542943	-2.03	-0.23
Abl1	chr2	31544075	31551320	-1.18	-0.13
Abl1	chr2	31653102	31658129	-1.05	-0.08
Mapkap1	chr2	34479153	34479423	-2.45	-0.44
Ssrp1	chr2	84885611	84887208	-3.11	-0.12
Psmc3	chr2	90894439	90895182	-2.45	-0.16
Tpx2	chr2	152719325	152719804	-0.61	0.07
Itch	chr2	154959216	154959307	-0.30	0.07
Itch	chr2	155050132	155050403	-0.62	-0.11

Phf20	chr2	156092990	156093201	-0.52	0.09
Phf20	chr2	156124429	156134013	-0.50	0.03
Tgif2	chr2	156679307	156679783	-2.73	-0.95
Src	chr2	157295196	157296561	-1.97	0.63
Trp53rkb	chr2	166620945	166621725	-1.43	-0.33
Gata3	chr2	9779686	9779776	-0.33	0.26
Gata3	chr2	9779865	9779925	-0.37	0.52
Gata3	chr2	9779984	9780252	-0.85	0.39
Gata3	chr2	9790440	9796131	-0.26	0.02
Taf3	chr2	9837377	9862619	-2.08	-0.23
Taf3	chr2	9862678	9874561	-0.84	0.07
Ehmt1	chr2	24646742	24647852	-2.26	-0.23
Ehmt1	chr2	24733063	24775080	-1.70	0.17
Notch1	chr2	26314049	26314349	-0.81	0.70
Notch1	chr2	26315066	26315157	-0.65	-1.86
Notch1	chr2	26315335	26315486	-0.50	-0.74
Notch1	chr2	26316053	26317703	-0.30	-0.07
Notch1	chr2	26323937	26324027	-0.49	-1.14
Cdk9	chr2	32562880	32565348	-3.08	-0.10
Psmd5	chr2	34707993	34709823	-0.82	-0.10
Psmd5	chr2	34726274	34726482	-3.28	-0.70
Psmb7	chr2	38498946	38499426	-1.70	-0.24
Nr4a2	chr2	56960145	56961132	-1.31	-0.24
Nr4a2	chr2	56965686	56976354	-1.92	1.13
Zfp770	chr2	114021193	114021283	-0.43	0.17
Zfp661	chr2	127402341	127403505	-0.42	0.05
Psmf1	chr2	151543679	151544068	-0.32	0.02
Zfp334	chr2	165203354	165203685	-0.40	-0.13
Zfp334	chr2	165205181	165206141	-0.43	-0.06
Zfp334	chr2	165207038	165207219	-1.00	-0.50
Trp53rka	chr2	165315878	165318784	-1.13	0.14
Aurka	chr2	172182168	172182653	-0.85	0.13
Skil	chr3	30994042	30997382	-0.39	0.01
Gatad2b	chr3	90145581	90145879	-2.33	-0.23
Gatad2b	chr3	90155817	90155908	-2.67	-0.37
Hist2h2be	chr3	96025043	96025490	-0.87	-0.31
Trim33	chr3	103148225	103148495	-2.43	0.25
Phc3	chr3	30805917	30813151	-0.46	0.06
Phc3	chr3	30864763	30868337	-1.50	-0.73
Maml3	chr3	51491893	51493212	-1.42	0.26
Maml3	chr3	51493600	51496170	-0.52	0.08
Maml3	chr3	51501693	51600368	-0.39	0.34
Maml3	chr3	51659540	51660829	-2.03	0.23

Maml3	chr3	51907999	51908149	-1.14	0.58
Maml3	chr3	51908418	51908808	-0.35	0.06
Psmb4	chr3	94690551	94690880	-2.31	-0.17
Hipk1	chr3	103547180	103548199	-0.93	-0.04
Prmt6	chr3	110050616	110051517	-1.73	-0.62
Prmt6	chr3	110052386	110053916	-3.52	-0.20
Nbn	chr4	15906479	15908877	-2.76	0.36
Nr4a3	chr4	48080715	48096224	-0.55	0.37
Rraga	chr4	86221576	86222293	-1.17	0.09
Tal1	chr4	114737352	114742061	-0.43	-0.04
Med8	chr4	118087470	118087618	-1.43	0.25
Scmh1	chr4	120201905	120201966	-0.42	-0.19
Rragc	chr4	123597235	123597356	-2.18	0.32
Rragc	chr4	123598193	123598284	-1.16	0.24
Phc2	chr4	128404881	128405061	-0.49	0.29
Nr0b2	chr4	133112156	133112304	-1.32	-1.45
Zfp46	chr4	135846237	135848601	-0.61	-0.06
Mtor	chr4	147930392	147931764	-0.76	0.10
Zfp691	chr4	118842660	118845711	-0.33	0.01
Zfp691	chr4	118846309	118846770	-2.39	1.37
Zfp69	chr4	120602801	120603819	-0.86	0.27
Tfap2e	chr4	126393574	126393694	-1.54	2.92
Sesn2	chr4	132048721	132050516	-0.58	-0.04
Sfn	chr4	133157754	133158083	-0.27	-0.02
Eloa	chr4	135561252	135561580	-1.33	0.22
Eloa	chr4	135565701	135577564	-0.37	0.01
Ski	chr4	154529795	154530722	-0.41	-0.02
Ski	chr4	154530781	154533563	-1.24	0.09
Ski	chr4	154533945	154596644	-3.03	-0.18
Cdk6	chr5	3519406	3520884	-1.97	-0.19
Kmt2e	chr5	22940246	22940487	-0.36	0.04
Kmt2e	chr5	23002879	23005273	-2.07	0.16
Kmt2e	chr5	23007381	23008999	-2.35	0.12
Ywhah	chr5	33361494	33361614	-0.36	0.07
Ywhah	chr5	33369299	33370047	-1.92	-0.08
Rbpj	chr5	54044875	54045116	-0.40	-0.92
Zfp605	chr5	110556690	110556990	-0.28	0.01
Zfp605	chr5	110557318	110558156	-0.31	-0.02
Triap1	chr5	115791255	115793441	-0.77	0.08
Psmd9	chr5	123678228	123678437	-1.01	0.20
Kmt5a	chr5	124911266	124911327	-0.69	-0.13
Gtf2h3	chr5	125046222	125046283	-0.86	-0.51
Zfp664	chr5	125343785	125366362	-3.24	-0.19

Zfp664	chr5	125366751	125367081	-0.41	-0.05
Rfc2	chr5	135073840	135074198	-2.20	-0.32
Zkscan1	chr5	138526311	138534046	-0.28	-0.01
Zkscan1	chr5	138541944	138542455	-2.08	-0.24
Zkscan1	chr5	138544222	138545932	-1.09	0.11
Zfp12	chr5	144005811	144007821	-2.30	-0.23
Zfp12	chr5	144007940	144008390	-0.87	-0.26
Zfp12	chr5	144008869	144009619	-2.02	-0.39
Zkscan5	chr5	145979715	145981274	-2.23	0.66
Zkscan5	chr5	145981691	145982380	-0.37	-0.06
Zfp655	chr5	145992583	145994463	-1.50	-0.36
Smarcd3	chr5	24100691	24100811	-0.72	-0.86
Prkag2	chr5	24605860	24606370	-1.09	-0.18
Kmt2c	chr5	24790138	24790949	-1.48	0.18
Kmt2c	chr5	24819830	24820730	-2.10	0.18
Prkab1	chr5	116463747	116467999	-0.33	-0.01
Prkab1	chr5	116474139	116474378	-0.83	-0.19
Ptpn11	chr5	121581827	121582307	-1.65	0.20
Ptpn11	chr5	121583292	121587519	-2.53	-0.16
Ncor2	chr5	125538304	125548276	-0.66	-0.08
Ncor2	chr5	125659463	125659584	-0.24	-0.12
Ubc	chr5	125867967	125870328	-2.39	0.10
Zfp11	chr5	130162172	130162950	-0.33	-0.05
Zfp113	chr5	138585541	138586141	-1.27	-0.30
Zfp113	chr5	138586470	138586680	-0.76	0.33
Taf6	chr5	138619844	138620202	-3.23	-0.49
Smurf1	chr5	145637663	145637963	-1.05	-0.44
Smurf1	chr5	145638322	145638682	-0.71	-0.20
Smurf1	chr5	145639310	145639521	-0.43	0.15
Smurf1	chr5	145726518	145726669	-0.30	0.31
Zkscan14	chr5	145956021	145962455	-1.48	-0.29
Rfc3	chr5	152445398	152445638	-0.35	0.05
Cul1	chr6	47474617	47476108	-2.74	-0.21
Zfp398	chr6	47790290	47790621	-3.35	-1.10
Zfp282	chr6	47854827	47855966	-0.80	-0.11
Zfp282	chr6	47856175	47856805	-0.84	-0.17
Zfp212	chr6	47870655	47876681	-3.35	-1.03
Zfp212	chr6	47880732	47881447	-2.64	-0.48
Zfp775	chr6	48555624	48569787	-2.72	-0.92
Zfp775	chr6	48569965	48570026	-0.29	-0.39
Zfp775	chr6	48570384	48570923	-3.10	-1.25
Gata2	chr6	88154683	88155829	-0.38	-0.05
Nr2c2	chr6	92041411	92042461	-0.44	-0.16

Nr2c2	chr6	92114226	92117902	-0.73	-0.11
Chd4	chr6	125079888	125080369	-0.40	-0.01
Cdkn1b	chr6	134870958	134872360	-0.68	0.10
H2afj	chr6	136757367	136757517	-1.06	-0.54
Cnot4	chr6	34973352	34974287	-0.81	0.20
Cnot4	chr6	34996066	35002891	-0.64	-0.06
Hipk2	chr6	38697991	38787107	-0.63	0.02
Hipk2	chr6	38825829	38826040	-1.39	-0.22
Zfp786	chr6	47769651	47770307	-0.93	-0.92
Zfp786	chr6	47770514	47770575	-0.14	0.00
Zfp786	chr6	47770752	47771200	-1.52	-1.37
Zfp786	chr6	47771348	47771646	-0.99	-1.29
Zfp746	chr6	48013500	48016503	-0.85	-0.09
Zfp746	chr6	48033523	48036532	-0.28	0.01
Gadd45a	chr6	66985208	66987341	-0.31	0.00
Rybp	chr6	100182244	100183112	-0.71	0.26
Zfp248	chr6	118378764	118380402	-2.34	0.42
Phc1	chr6	122275560	122275800	-2.40	-0.84
Tigar	chr6	127037468	127038307	-1.14	0.14
Ccnd2	chr6	127079526	127080245	-0.86	0.11
Rhno1	chr6	128307285	128309180	-0.40	-0.02
Kras	chr6	145168813	145168964	-1.25	-0.35
Zfp667	chr7	6256343	6256524	-0.69	0.61
Zfp667	chr7	6257749	6257810	-0.29	0.32
Zfp78	chr7	6331550	6332238	-0.37	0.13
Zfp28	chr7	6344996	6347379	-0.53	0.10
Zfp110	chr7	13434054	13434502	-0.47	-0.06
Zfp324	chr7	13555642	13556271	-2.04	-0.62
Zfp324	chr7	13556928	13557616	-2.87	-0.66
Zfp446	chr7	13567286	13568246	-1.90	-0.38
Ercc2	chr7	19979500	19981041	-0.83	-0.08
Zfp180	chr7	24882912	24891516	-1.80	0.13
Zfp112	chr7	24910123	24910184	-1.08	-3.00
Zfp112	chr7	24910243	24910454	-0.57	-0.73
Zfp112	chr7	24910513	24910604	-0.13	-0.17
Zfp112	chr7	24910962	24911473	-0.38	0.21
Zfp112	chr7	24912431	24912551	-0.80	1.66
Zfp235	chr7	24925651	24925742	-0.66	0.68
Zfp235	chr7	24927899	24928200	-0.36	-0.20
Akt2	chr7	28422173	28423428	-0.40	0.03
Zfp30	chr7	30577880	30578810	-1.97	0.80
Zfp790	chr7	30601090	30601151	-0.45	0.29
Zfp420	chr7	30645086	30661279	-2.76	0.27

Zfp382	chr7	30916575	30919814	-1.94	0.18
Polr2i	chr7	31017332	31017529	-0.50	-0.12
Zfp658	chr7	50828078	50828289	-0.99	-0.64
Zfp658	chr7	50828587	50829455	-0.67	-0.16
Zfp658	chr7	50829663	50830293	-0.55	0.15
Zfp710	chr7	87231504	87231625	-1.34	-0.43
Zfp710	chr7	87233516	87236264	-1.90	-0.12
Lamtor1	chr7	109054380	109058241	-0.51	-0.09
Setd1a	chr7	134927665	134930705	-3.33	-0.13
Setd1a	chr7	134931780	134932028	-0.75	-0.13
Zik1	chr7	11075894	11076075	-0.62	0.50
Zfp551	chr7	13000795	13000946	-0.55	-0.63
Zfp551	chr7	13001544	13001605	-0.37	0.66
Zfp551	chr7	13001783	13001874	-0.23	-0.07
Zfp551	chr7	13002053	13002203	-0.45	-0.51
Zfp940	chr7	30629198	30629379	-2.28	-1.54
Zfp940	chr7	30630307	30630488	-0.67	-0.73
Zfp27	chr7	30678504	30680419	-1.09	0.13
Zfp27	chr7	30680807	30683850	-0.49	0.07
Zfp14	chr7	30823226	30823406	-0.82	1.24
Zfp566	chr7	30862385	30862831	-0.63	0.23
Zfp566	chr7	30863008	30863483	-0.99	0.28
Ccne1	chr7	38883180	38883329	-1.29	-0.73
Zfp473	chr7	51987570	51988080	-0.82	0.69
Zfp473	chr7	51988469	51988860	-0.49	-0.50
Med25	chr7	52132313	52133597	-2.46	-0.25
Bax	chr7	52722059	52722238	-0.71	0.19
E2f8	chr7	56123087	56123387	-2.78	-0.89
Blm	chr7	87647292	87647681	-0.45	0.09
Blm	chr7	87654437	87658340	-2.18	0.36
Eed	chr7	97129276	97129426	-0.44	0.08
Zfp747	chr7	134516438	134516647	-0.30	0.04
Zfp747	chr7	134516706	134519504	-2.94	0.29
9130019O22Rik	chr7	134526847	134529144	-0.29	0.00
E430018J23Rik	chr7	134534349	134536523	-1.20	-0.34
E430018J23Rik	chr7	134536927	134537135	-0.26	0.15
Zfp764	chr7	134547867	134549122	-1.58	-0.25
Zfp688	chr7	134562479	134563593	-2.08	-0.41
Zfp689	chr7	134587294	134588642	-2.65	-0.51
Zfp689	chr7	134592222	134592433	-0.67	0.30
Pidd1	chr7	148624413	148624744	-1.68	-0.61
Tfdp1	chr8	13377196	13377345	-0.76	-0.13
Tfdp1	chr8	13377582	13377702	-1.12	-0.18

Kat6a	chr8	24040759	24043005	-0.79	-0.08
Kat6a	chr8	24046218	24051430	-0.82	0.04
Ppp2cb	chr8	34710092	34710330	-0.63	-0.43
Nr2c2ap	chr8	72655231	72655498	-1.07	0.29
Nr2c2ap	chr8	72657115	72657440	-0.91	-0.13
Ell	chr8	73063573	73063694	-2.62	-1.00
Ell	chr8	73107871	73114673	-3.00	-0.34
Sin3b	chr8	75247186	75248032	-1.12	0.19
Sin3b	chr8	75273726	75273966	-0.65	0.20
Sin3b	chr8	75280930	75281383	-2.20	-0.49
Nr3c2	chr8	79433507	79433776	-0.32	-0.42
Cbfb	chr8	107694632	107694812	-1.00	-0.17
Zfp90	chr8	108947880	108948329	-3.26	0.83
Zfp90	chr8	108948657	108949137	-0.47	0.12
Zfp90	chr8	108949196	108949675	-1.18	0.35
Zfp1	chr8	114190002	114194702	-0.53	-0.06
Banp	chr8	124550340	124550731	-0.77	-0.40
Banp	chr8	124550880	124551060	-0.48	-0.38
Banp	chr8	124551419	124551540	-1.16	-1.10
Banp	chr8	124552528	124552798	-1.31	-0.79
Zfpm1	chr8	124806220	124856010	-0.98	0.18
Zfpm1	chr8	124859027	124861147	-1.02	-0.10
Ash2l	chr8	26950536	26958082	-1.35	-0.23
Wrn	chr8	34346284	34346854	-0.41	-0.08
Wrn	chr8	34434722	34439590	-2.21	0.55
Cnot7	chr8	41592731	41595397	-1.59	0.21
Ing2	chr8	48753850	48754661	-0.78	0.10
Ing2	chr8	48760302	48760513	-2.46	-1.21
Gatad2a	chr8	72498029	72498150	-0.57	-0.26
Gatad2a	chr8	72519918	72520248	-0.39	0.08
Csnk2a2	chr8	97976954	97977225	-1.72	-0.27
Maml2	chr9	13424522	13425092	-2.48	0.70
Maml2	chr9	13510203	13511312	-3.34	0.53
Pin1	chr9	20456573	20459888	-1.84	-0.36
Zfp202	chr9	40015019	40015258	-3.36	-0.92
Usp2	chr9	43902061	43902302	-1.62	-0.76
H2afx	chr9	44143446	44144156	-0.58	0.03
Ppp2r1b	chr9	50688020	50688710	-0.53	0.05
Ppp2r1b	chr9	50701016	50701675	-1.83	-0.33
Sin3a	chr9	56958532	56958892	-0.66	0.11
Sin3a	chr9	56973115	56975606	-1.32	0.16
Rora	chr9	69226745	69226835	-0.38	-0.37
Atr	chr9	95765579	95765699	-0.73	-0.27

Tfdp2	chr9	96218608	96218729	-0.27	0.00
Ctnnb1	chr9	120842577	120859351	-2.42	-0.18
Ctnnb1	chr9	120868936	120868997	-1.38	0.13
Zkscan7	chr9	122797695	122797846	-0.50	-0.44
Zkscan7	chr9	122803837	122804973	-1.16	-0.38
Zfp558	chr9	18260716	18261377	-0.28	0.03
Zfp266	chr9	20304179	20306501	-0.43	0.03
Kmt2a	chr9	44612666	44613327	-0.91	-0.13
Kmt2a	chr9	44629634	44630384	-2.09	0.19
Kmt2a	chr9	44655514	44656204	-3.18	0.26
Kmt2a	chr9	44656473	44656924	-0.49	0.06
Pml	chr9	58067502	58068341	-3.10	-0.28
Pml	chr9	58080996	58094952	-1.77	-0.21
Pml	chr9	58097382	58097593	-1.39	-0.43
Smad3	chr9	63496369	63496909	-0.75	0.36
Smad3	chr9	63497357	63498046	-0.81	-0.34
Smad3	chr9	63605729	63605891	-0.28	-0.36
Rnf111	chr9	70274555	70277420	-1.37	0.15
Tcf12	chr9	71958831	71958892	-0.31	-0.03
Tcf12	chr9	71959385	71959626	-0.98	-0.22
Atrip	chr9	108960445	108961754	-3.54	-0.18
Atrip	chr9	108967670	108968299	-2.16	0.32
Cnot10	chr9	114541870	114549228	-1.71	-0.34
Sgk1	chr10	21718573	21718873	-1.56	0.74
Sesn1	chr10	41530409	41607263	-0.57	0.15
Sesn1	chr10	41627347	41627735	-0.58	0.32
Stk11	chr10	79579091	79587616	-1.24	-0.11
Stk11	chr10	79592650	79593215	-1.30	-0.18
Txnrd1	chr10	82296755	82323178	-0.61	0.15
Smarcc2	chr10	127920231	127920695	-2.24	0.31
Smarcc2	chr10	127926123	127926273	-0.50	0.09
Ddit4	chr10	59412629	59412689	-0.57	0.25
Nrbf2	chr10	66729823	66730867	-1.76	0.19
Ube2d1	chr10	70718174	70718562	-1.17	0.19
Smarcb1	chr10	75359631	75359751	-1.58	-0.36
Polr2e	chr10	79501286	79502404	-1.51	-0.21
Mbd3	chr10	79855285	79856691	-1.70	-0.10
Tcf3	chr10	79872416	79873225	-0.84	-0.10
Cradd	chr10	94637889	94638400	-1.23	-0.45
Cnot2	chr10	115922246	115922606	-0.97	0.18
Cnot2	chr10	115922874	115923115	-1.67	0.33
Yeats4	chr10	116652465	116652884	-1.13	0.17
Dyrk2	chr10	118296404	118305900	-0.46	0.03

Pip4k2c	chr10	126635286	126635914	-1.06	0.12
Psme4	chr11	30671804	30671925	-0.83	-0.91
Psme4	chr11	30672014	30672135	-0.38	0.12
Med7	chr11	46250448	46250688	-1.24	-0.38
Ppp2ca	chr11	51935449	51935836	-1.90	0.10
Cnot8	chr11	57930988	57931258	-1.37	0.34
Zkscan6	chr11	65636412	65642412	-0.89	-0.14
Trp53	chr11	69393860	69394608	-0.68	0.14
Ywhae	chr11	75546388	75546567	-2.12	-0.15
Suz12	chr11	79843530	79845978	-2.27	0.14
Psmd11	chr11	80285544	80285635	-1.72	-0.32
Cdk5r1	chr11	80293403	80293969	-3.03	0.92
Cdk5r1	chr11	80293392	80293962	-2.75	0.87
Taf15	chr11	83286609	83286700	-0.71	-0.14
Ppm1d	chr11	85140408	85140529	-0.31	-0.04
Ppm1d	chr11	85153180	85159827	-0.35	0.02
Supt4a	chr11	87557029	87557119	-0.77	0.39
Cdk12	chr11	98071677	98072546	-0.31	0.00
Thra	chr11	98603186	98617447	-0.55	0.14
Thra	chr11	98625630	98626259	-2.85	-0.46
Rara	chr11	98799009	98808938	-1.27	-0.44
Psme3	chr11	101177564	101178336	-0.63	-0.07
Psme3	chr11	101182070	101183288	-1.70	-0.15
Cbx2	chr11	118885531	118887831	-0.67	1.03
Rptor	chr11	119465086	119517298	-0.84	0.12
Rptor	chr11	119759767	119760755	-0.49	-0.04
Hus1	chr11	8896741	8896861	-0.52	0.16
Maml1	chr11	50069972	50070390	-0.48	0.08
Maml1	chr11	50076862	50105838	-3.04	-0.21
Rad50	chr11	53463680	53465769	-1.04	-0.17
Zkscan17	chr11	59299022	59299352	-0.46	0.07
Zkscan17	chr11	59300338	59305712	-0.44	0.02
Top3a	chr11	60555827	60557475	-1.90	-0.31
Top3a	chr11	60590716	60590867	-1.91	0.89
Ncor1	chr11	62130910	62131481	-1.03	0.10
Ncor1	chr11	62147216	62147367	-0.98	0.19
Ncor1	chr11	62182894	62186997	-2.47	-0.16
Ncor1	chr11	62251972	62270948	-0.37	-0.06
Zfp287	chr11	62527143	62527774	-0.60	0.18
Zfp287	chr11	62528133	62528313	-0.31	0.07
Zfp287	chr11	62528582	62528703	-0.25	0.02
Zfp287	chr11	62541813	62542444	-0.65	-0.22
Chd3	chr11	69174956	69175626	-3.21	-0.43

Rpa1	chr11	75154547	75161885	-2.86	-0.51
Med13	chr11	86100091	86100242	-0.90	-0.15
Med13	chr11	86132926	86141950	-2.79	0.16
Pip4k2b	chr11	97578204	97583775	-1.02	-0.05
Med1	chr11	98014158	98015024	-0.76	0.11
Med1	chr11	98015143	98015684	-0.34	0.01
Med1	chr11	98016102	98017333	-0.90	0.06
Med1	chr11	98017932	98018173	-0.41	-0.03
Med1	chr11	98018352	98019732	-1.77	0.10
Med1	chr11	98054124	98054607	-1.22	-0.20
Nr1d1	chr11	98629338	98629726	-1.00	0.26
Nr1d1	chr11	98631783	98632540	-3.18	-0.44
Nr1d1	chr11	98633136	98636556	-0.49	-0.06
Brca1	chr11	101384814	101385204	-1.75	-0.62
Brca1	chr11	101385473	101386581	-0.92	-0.20
Brca1	chr11	101386909	101387659	-3.36	0.64
Smurf2	chr11	106683743	106684358	-0.92	-0.20
Cbx8	chr11	118899867	118901442	-1.43	0.25
Cbx4	chr11	118940531	118940592	-1.14	1.49
Cbx4	chr11	118941070	118941400	-3.20	1.23
Cbx4	chr11	118941759	118947461	-0.50	0.02
E2f6	chr12	16832231	16832859	-1.35	0.19
Psma6	chr12	56499811	56499871	-1.67	-0.32
Ppm1a	chr12	73862227	73885470	-0.74	-0.04
Ppm1a	chr12	73894973	73895153	-0.48	0.08
Psmc1	chr12	101348377	101351286	-1.53	-0.29
Ccnk	chr12	109418007	109424590	-0.70	-0.17
Zfp839	chr12	112106479	112107344	-0.76	-0.14
Ywhaq	chr12	21422717	21423076	-1.30	-0.20
Max	chr12	78038822	78039718	-1.25	0.06
Max	chr12	78063025	78063235	-0.95	-0.16
Ppp1r13b	chr12	113067028	113067478	-1.41	-0.33
Akt1	chr12	113912217	113912487	-0.40	0.08
Zkscan4	chr13	21576264	21576955	-0.44	-0.43
Hist1h3h	chr13	21809496	21809698	-0.75	-0.19
Hist1h3f	chr13	23636303	23636393	-1.63	-0.30
Hist1h2bg	chr13	23663268	23663474	-0.87	-0.07
Hist1h3d	chr13	23667608	23667786	-0.59	-0.11
Hist1h2bc	chr13	23776067	23776213	-1.76	0.26
Hist1h2ab	chr13	23842956	23843224	-1.74	-0.27
Rmi1	chr13	58503698	58503988	-0.71	-0.12
Med10	chr13	69954496	69954853	-0.30	0.00
Taf9	chr13	101435858	101436098	-2.24	0.28

Plk2	chr13	111190024	111190991	-3.23	0.37
Slc38a9	chr13	113528239	113528690	-0.79	0.50
Cdk13	chr13	17858388	17896931	-0.44	-0.02
Zkscan3	chr13	21488092	21494295	-2.58	-0.45
Zkscan8	chr13	21611911	21613722	-1.26	-0.18
Zkscan8	chr13	21617156	21618818	-1.82	0.41
Hist1h3i	chr13	21875208	21875266	-0.36	-0.05
Hist1h3c	chr13	23837329	23837390	-0.45	0.12
Tfap2a	chr13	40812539	40812690	-0.17	0.59
Dek	chr13	47201073	47201589	-0.67	-0.13
Ctsl	chr13	64470393	64471614	-2.04	-0.09
Zfp729a	chr13	67720451	67721021	-0.67	0.12
Zfp729a	chr13	67721529	67722158	-0.70	0.18
Zfp729a	chr13	67722397	67722547	-0.30	0.02
Zfp738	chr13	67768497	67769604	-3.07	0.55
Zfp738	chr13	67770112	67770352	-0.31	0.04
Zfp738	chr13	67771338	67771758	-1.04	0.44
Nr2f1	chr13	78328745	78328956	-1.15	-3.17
Jmy	chr13	94234651	94269644	-2.87	-0.26
Rad17	chr13	101387477	101389058	-2.53	-0.36
Psmc6	chr14	45949498	45950514	-0.68	0.11
Med4	chr14	73909855	73909974	-2.48	-0.83
Med4	chr14	73917729	73918293	-0.71	-0.10
Elf1	chr14	79881389	79936053	-0.38	0.05
Elf1	chr14	79973144	79980817	-0.52	-0.03
Rarb	chr14	17407447	17451237	-0.24	0.12
Nr1d2	chr14	19044433	19047065	-1.52	1.13
Pip4p1	chr14	51545984	51546434	-1.90	0.16
Supt16	chr14	52803669	52816914	-0.75	-0.13
Prmt5	chr14	55126018	55126553	-0.37	0.02
Rabggta	chr14	56340688	56340807	-0.30	0.04
Cenpj	chr14	57145687	57148592	-0.68	-0.24
Cenpj	chr14	57170817	57171506	-0.75	-0.17
Cenpj	chr14	57171774	57172282	-0.44	0.08
Gtf2f2	chr14	76297012	76306621	-0.38	0.02
Prkaa1	chr15	5093860	5110647	-3.53	-0.59
Prkaa1	chr15	5126670	5126850	-0.53	-0.06
Prkaa1	chr15	5128706	5129184	-0.87	0.12
Rictor	chr15	6736486	6737266	-3.47	-0.21
Rictor	chr15	6745928	6747254	-1.18	-0.11
Zfp707	chr15	75804010	75804159	-1.43	-0.84
Ep300	chr15	81416643	81438767	-1.28	-0.07
Ep300	chr15	81479273	81481819	-1.93	-0.07

L3mbtl2	chr15	81516741	81518556	-1.99	-0.18
Arid2	chr15	96199968	96201115	-0.78	-0.06
Arid2	chr15	96223066	96232775	-1.58	0.29
Zfp740	chr15	102034168	102035505	-1.63	0.18
Zfp740	chr15	102039660	102043880	-1.11	-0.09
Sp1	chr15	102238241	102256144	-0.99	-0.04
Sp1	chr15	102261170	102261651	-0.65	-0.06
Skp2	chr15	9043329	9043510	-0.80	-0.31
Skp2	chr15	9069207	9070137	-0.35	-0.03
Zfp706	chr15	36937037	36937157	-3.06	-1.12
Taf2	chr15	54847403	54848154	-0.55	-0.07
Atad2	chr15	57966487	57966637	-2.14	-0.86
Brd1	chr15	88543169	88543779	-0.31	0.01
Yaf2	chr15	93115848	93167246	-2.54	0.27
Vdr	chr15	97687183	97687273	-0.18	0.38
Prkag1	chr15	98643704	98643794	-0.63	-0.14
Zfp263	chr16	3744098	3745086	-1.38	-0.17
Zfp263	chr16	3748158	3750040	-0.38	0.01
Rmi2	chr16	10886343	10886404	-0.50	3.17
Zfp597	chr16	3865645	3871203	-2.90	0.39
Crebbp	chr16	4084047	4087743	-1.51	-0.07
Crebbp	chr16	4154961	4213404	-1.65	-0.09
Usp7	chr16	8738344	8757028	-1.22	-0.49
Nr1i2	chr16	38249474	38251285	-1.74	3.10
Runx1	chr16	92605638	92606179	-2.19	-0.21
Runx1	chr16	92697371	92697492	-0.37	-0.10
Arid1b	chr17	4995073	4995882	-1.14	-0.42
Arid1b	chr17	4996061	5040748	-2.27	-0.39
Arid1b	chr17	5332607	5337057	-0.44	0.03
Arid1b	chr17	5337146	5339263	-0.95	-0.21
Arid1b	chr17	5341548	5343285	-1.16	0.08
Zfp160	chr17	21162569	21164422	-2.96	0.26
Zfp160	chr17	21178570	21179347	-0.32	0.07
Ppard	chr17	28369728	28432393	-2.21	-0.47
Mapk14	chr17	28884007	28885350	-0.47	0.02
Brpf3	chr17	28938160	28938221	-0.31	-0.68
Brpf3	chr17	28954078	28960704	-1.02	0.17
Brpf3	chr17	28973668	28973878	-0.31	-0.05
Rxrb	chr17	34174187	34175165	-0.69	0.06
Psmb8	chr17	34335288	34335468	-2.26	-0.35
Ehmt2	chr17	35049712	35050992	-2.85	-0.23
Zfp213	chr17	23694297	23694714	-0.78	-0.43
Zfp213	chr17	23694951	23696378	-0.51	0.41

Mlst8	chr17	24612351	24613015	-1.70	-0.31
Ube2i	chr17	25398025	25398746	-3.13	-0.38
Taf11	chr17	28038342	28038672	-0.66	-0.10
Taf11	chr17	28042348	28045128	-2.46	-0.34
Ring1	chr17	34158095	34160251	-0.37	-0.02
Runx2	chr17	44745018	44745079	-0.41	0.19
Rbbp8	chr18	11880308	11881165	-1.62	0.19
Taf4b	chr18	14965941	14971957	-1.84	0.42
Polr2d	chr18	31955847	31956355	-3.53	0.44
Ercc3	chr18	32399984	32400342	-0.44	-0.08
Ercc3	chr18	32429532	32429682	-0.45	0.08
Prelid3a	chr18	67639310	67639460	-0.26	0.03
Smad7	chr18	75527018	75527704	-1.68	0.45
Smad2	chr18	76422217	76422308	-0.97	-0.22
Smad2	chr18	76462139	76464727	-1.91	0.24
Epc1	chr18	6490659	6515906	-1.32	-0.37
Kctd1	chr18	15127940	15128061	-0.21	-0.06
Kctd1	chr18	15220279	15220489	-0.21	0.00
Kctd1	chr18	15220578	15220788	-0.20	0.00
Kctd1	chr18	15221534	15221834	-0.22	0.17
Nr3c1	chr18	39588392	39649457	-2.22	0.16
Smad4	chr18	73799858	73800038	-0.58	0.10
Smad4	chr18	73837362	73837780	-3.05	0.33
Smad4	chr18	73863156	73863395	-2.18	-1.27
Ctdp1	chr18	80642798	80647906	-0.48	0.03
Men1	chr19	6334978	6336811	-2.76	-0.24
Men1	chr19	6338843	6340864	-1.39	-0.08
Smarca2	chr19	26705324	26705475	-0.54	-0.24
Pcgf5	chr19	36519525	36530396	-3.45	-0.52
Taf5	chr19	47145518	47149532	-0.62	-0.16
Taf5	chr19	47156922	47157312	-0.74	-0.19
Rad9a	chr19	4195437	4196226	-0.51	-0.05
Rbm14	chr19	4803491	4811634	-3.01	-0.15
Kat5	chr19	5603043	5603692	-2.39	-0.21
Kat5	chr19	5609321	5609592	-0.63	-0.14
Kat5	chr19	5609884	5610034	-1.24	-0.66
Polr2g	chr19	8872986	8873047	-0.42	-0.12
Ldb1	chr19	46107090	46108545	-1.10	-0.08
Pcgf6	chr19	47108886	47109036	-0.32	0.03
Med12	chrX	98488099	98488190	-0.95	0.27
Taf1	chrX	98794675	98794885	-0.74	-0.16
Rragb	chrX	149574707	149575911	-1.22	0.28
G6pdx	chrX	71655359	71655479	-1.22	-0.12

Chm	chrX	110156917	110157037	-1.01	0.43

Metabolism of proteins

gene_name	chr	chromStart	chromEnd	diff.lg.p	diff.log2.fc
Rab23	chr1	33795210	33796952	-0.42	-0.04
Mrpl44	chr1	79772741	79774975	-2.39	0.25
Mrpl44	chr1	79776125	79777602	-0.95	0.21
Sp100	chr1	87605784	87606023	-1.37	0.49
Psmd1	chr1	87961193	87961254	-0.37	-0.11
Cops8	chr1	92508845	92509678	-0.50	0.03
Ube2f	chr1	93182033	93182602	-1.08	-0.09
Asb1	chr1	93440810	93443711	-1.60	-0.49
Asb1	chr1	93451373	93451734	-0.52	0.13
Mgat5	chr1	129203315	129203466	-1.16	-0.41
Mgat5	chr1	129376047	129379549	-1.02	-0.16
Yod1	chr1	132613903	132614024	-0.24	0.29
Yod1	chr1	132614351	132616572	-2.82	0.26
Rab7b	chr1	133585271	133593615	-0.56	-0.20
Rab7b	chr1	133602280	133610067	-2.45	-0.25
Rbbp5	chr1	134394590	134400982	-3.46	0.33
Rbbp5	chr1	134401131	134401282	-0.55	-0.11
Ube2t	chr1	136859153	136864304	-2.08	-0.99
Edem3	chr1	153658769	153666557	-0.36	0.01
Pigc	chr1	163899348	163901752	-0.98	-0.09
F5	chr1	166122292	166122503	-0.98	2.87
F5	chr1	166122922	166123223	-2.19	4.15
F5	chr1	166124151	166124452	-2.66	4.37
F5	chr1	166124661	166124722	-0.14	-0.15
Adamts4	chr1	173189066	173189276	-0.94	-2.16
B4galt3	chr1	173206311	173206788	-3.02	-0.30
Dcaf8	chr1	174078175	174078505	-1.42	-0.39
Dcaf8	chr1	174079403	174103118	-3.22	-0.21
Dcaf8	chr1	174124786	174125087	-1.07	0.21
Pigm	chr1	174306661	174308397	-1.98	0.12
Parp1	chr1	182499105	182503813	-3.02	-0.53
Mrpl15	chr1	4766407	4766796	-1.18	0.15
Cops5	chr1	10027829	10027979	-0.46	-0.04
Uggt1	chr1	36200615	36203523	-1.07	0.08
Uggt1	chr1	36300966	36301147	-2.77	-0.51
Lman2l	chr1	36480210	36481290	-2.06	-0.23
Chst10	chr1	38920717	38922965	-1.47	0.09
Satb2	chr1	56852645	56854020	-1.68	0.15
Satb2	chr1	56928445	56928625	-2.86	-0.44
Satb2	chr1	57026707	57028178	-2.44	-0.49

Sumo1	chr1	59696425	59696959	-2.60	0.18
Bard1	chr1	71121361	71122020	-3.24	-0.57
Usp37	chr1	74485767	74485918	-0.75	0.27
Tuba4a	chr1	75211607	75211936	-0.33	0.05
Tuba4a	chr1	75212113	75215828	-2.13	-0.09
Cul3	chr1	80336855	80337005	-0.41	-0.05
Hdac4	chr1	93829331	93829930	-2.32	-0.46
Pign	chr1	107560192	107560253	-0.60	0.26
Ikbke	chr1	133151178	133153534	-2.21	-0.20
Mdm4	chr1	134888236	134893148	-1.31	0.10
Rnf2	chr1	153318408	153318469	-0.48	0.17
Lamc1	chr1	155066798	155066859	-0.32	0.03
Lamc1	chr1	155179317	155179826	-2.31	-0.22
Qsox1	chr1	157626448	157628411	-2.42	-0.35
Klhl20	chr1	163019047	163019258	-2.02	-0.55
Klhl20	chr1	163020426	163023835	-3.23	0.59
Klhl20	chr1	163039327	163039897	-0.46	0.06
Dcaf6	chr1	167281092	167281331	-0.55	0.15
Uap1	chr1	172072432	172072642	-2.20	0.58
Dtl	chr1	193363068	193363969	-3.46	1.70
Dtl	chr1	193364536	193364986	-1.04	-0.31
Stam	chr2	14067864	14068433	-2.00	0.29
Commd3	chr2	18596461	18597638	-0.32	-0.01
Bmi1	chr2	18605637	18606468	-2.42	0.17
Fbxw5	chr2	25358984	25359776	-1.40	-0.18
Fbxw5	chr2	25360013	25360422	-1.11	-0.22
Mrps2	chr2	28323600	28323661	-0.37	0.21
Ttf1	chr2	28915782	28922626	-1.94	-0.17
Ttf1	chr2	28941735	28942005	-0.65	0.21
Dolpp1	chr2	30252653	30255005	-0.66	-0.14
Usp20	chr2	30865689	30865870	-0.98	-0.37
Nup214	chr2	31888785	31893623	-1.12	-0.08
Nup214	chr2	31907789	31907850	-0.40	-0.11
Pomt1	chr2	32109957	32110226	-2.95	-0.65
Golga2	chr2	32158692	32159742	-1.00	-0.15
Golga2	chr2	32161974	32162215	-0.31	-0.01
Dpm2	chr2	32427908	32429091	-2.78	-0.21
St6galnac4	chr2	32451265	32451356	-0.49	0.18
St6galnac4	chr2	32452492	32453780	-1.27	-0.12
Mrrf	chr2	36044752	36045143	-0.30	0.01
Mbd5	chr2	49101163	49101343	-0.39	0.12
Mbd5	chr2	49111717	49113842	-0.74	-0.09
Mbd5	chr2	49134446	49169227	-0.47	0.17

Klhl41	chr2	69508654	69508893	-1.20	0.67
Dync1i2	chr2	71049792	71050012	-1.03	0.40
Psmc3	chr2	90894439	90895182	-2.45	-0.16
Traf6	chr2	101531587	101537959	-2.12	0.17
Rtf1	chr2	119558206	119559166	-1.97	-0.19
Rtf1	chr2	119560273	119560484	-0.78	-0.27
Usp8	chr2	126563807	126568357	-3.01	-0.21
Prnd	chr2	131735723	131776618	-0.50	0.21
Tbc1d20	chr2	152119607	152127898	-0.30	0.01
Tbc1d20	chr2	152137106	152137759	-0.37	0.03
Asxl1	chr2	153171874	153177942	-2.57	-0.61
Actr5	chr2	158462488	158464947	-0.87	-0.18
Top1	chr2	160471632	160471932	-2.93	-0.34
Top1	chr2	160547273	160547394	-0.59	0.10
Tubb1	chr2	174282844	174283054	-0.17	0.13
Adrm1	chr2	179906292	179906906	-0.32	0.02
Gata3	chr2	9779686	9779776	-0.33	0.26
Gata3	chr2	9779865	9779925	-0.37	0.52
Gata3	chr2	9779984	9780252	-0.85	0.39
Gata3	chr2	9790440	9796131	-0.26	0.02
Mrpl41	chr2	24829604	24830588	-2.76	0.25
Tubb4b	chr2	25077855	25080192	-0.38	-0.01
Traf2	chr2	25374427	25375145	-1.67	-0.19
Sec16a	chr2	26277717	26279943	-0.64	0.09
Sec16a	chr2	26294102	26294703	-0.51	0.04
Sec16a	chr2	26295032	26300736	-1.17	0.06
Ntng2	chr2	29063096	29103560	-1.80	-0.66
Dolk	chr2	30139748	30140467	-1.14	0.19
Dolk	chr2	30140616	30141814	-0.56	-0.08
Fbxw2	chr2	34661048	34661869	-2.35	-0.23
Fbxw2	chr2	34662794	34664040	-0.82	-0.21
Fbxw2	chr2	34668436	34678672	-0.41	-0.02
Fbxw2	chr2	34681468	34681725	-0.52	0.16
Psmd5	chr2	34707993	34709823	-0.82	-0.10
Psmd5	chr2	34726274	34726482	-3.28	-0.70
Psmb7	chr2	38498946	38499426	-1.70	-0.24
Galnt3	chr2	65945079	65962610	-1.45	0.54
Slc35c1	chr2	92293995	92294146	-1.70	-0.44
Slc35c1	chr2	92294234	92294832	-0.73	-0.10
Slc35c1	chr2	92298932	92299589	-0.72	-0.08
Rcn1	chr2	105239178	105239446	-0.85	-0.22
Ino80	chr2	119200037	119200278	-1.91	0.39
Trp53bp1	chr2	121030727	121033735	-0.29	0.00

Trp53bp1	chr2	121034169	121046975	-0.79	-0.09
Trp53bp1	chr2	121061343	121062603	-0.86	0.09
Cops2	chr2	125657568	125660555	-1.21	-0.08
Psmf1	chr2	151543679	151544068	-0.32	0.02
Edem2	chr2	155527855	155531394	-0.83	-0.09
Edem2	chr2	155555061	155555181	-0.35	-0.07
Neurl2	chr2	164656229	164658385	-1.59	0.26
B4galt5	chr2	167174439	167174678	-3.17	-0.64
Sec62	chr3	30692244	30692305	-0.50	0.19
Sec62	chr3	30711269	30718098	-0.87	0.06
Usp13	chr3	32832209	32832389	-0.70	3.46
Fbxw7	chr3	84619498	84694070	-0.58	0.12
Fbxw7	chr3	84694129	84756226	-2.67	0.26
Fbxw7	chr3	84780140	84781955	-0.67	0.08
Rab13	chr3	90028667	90029856	-1.43	0.42
Mrpl9	chr3	94251792	94252630	-1.18	-0.10
Otud7b	chr3	95908449	95908540	-1.61	-0.69
Otud7b	chr3	95956546	95960193	-1.03	-0.07
Hist2h2be	chr3	96025043	96025490	-0.87	-0.31
Dph5	chr3	115631581	115632001	-0.77	0.15
Bcl10	chr3	145587262	145587592	-1.35	-0.17
Bcl10	chr3	145588099	145593586	-0.30	0.00
Usp33	chr3	152031258	152033363	-1.23	-0.12
Pigk	chr3	152448420	152449109	-1.33	-0.21
Pigk	chr3	152449825	152450483	-1.30	0.25
Pigk	chr3	152451617	152451828	-0.79	0.29
Pex2	chr3	5561142	5563166	-0.32	0.01
Mrps28	chr3	8802318	8802406	-0.53	0.14
Phc3	chr3	30805917	30813151	-0.46	0.06
Phc3	chr3	30864763	30868337	-1.50	-0.73
Cog6	chr3	52798099	52798220	-0.40	-1.58
Commd2	chr3	57450273	57450603	-0.62	-0.13
Siah2	chr3	58479649	58495411	-0.38	-0.02
Siah2	chr3	58495680	58495891	-0.39	-0.11
Dap3	chr3	88727172	88727531	-0.35	-0.07
Psmb4	chr3	94690551	94690880	-2.31	-0.17
Man1a2	chr3	100371011	100382434	-0.85	0.08
Man1a2	chr3	100460030	100489396	-2.89	0.15
Csf1	chr3	107544324	107544444	-0.74	0.65
Csf1	chr3	107544802	107548597	-0.55	0.11
Csf1	chr3	107548745	107550765	-0.94	-1.24
Csf1	chr3	107551063	107552051	-0.42	0.07
Gnai3	chr3	107911702	107912208	-0.87	0.06

Gnai3	chr3	107948823	107949032	-1.51	-0.18
Rwdd3	chr3	120859364	120862089	-1.12	-0.32
Ank2	chr3	126625873	126626114	-0.36	1.15
Ank2	chr3	126626353	126626834	-1.70	-1.10
Sec24b	chr3	129686102	129687101	-1.10	-0.09
Rab2a	chr4	8462790	8462851	-1.78	-0.59
Map3k7	chr4	32051081	32051232	-1.86	-0.83
Map3k7	chr4	32110141	32110231	-1.49	-0.76
Ube2r2	chr4	41137807	41138136	-0.82	-0.10
Creb3	chr4	43575505	43575685	-1.36	-0.32
Creb3	chr4	43579692	43579932	-0.66	-0.15
Dcaf10	chr4	45385889	45387268	-0.55	-0.08
Tgfbr1	chr4	47419877	47423747	-3.57	0.68
Rad23b	chr4	55398385	55402936	-1.44	0.10
Rraga	chr4	86221576	86222293	-1.17	0.09
Usp24	chr4	105988817	105989117	-1.39	-0.42
Usp24	chr4	106111387	106111837	-3.22	-0.26
Lrrc41	chr4	115768961	115769201	-3.42	-0.29
Scmh1	chr4	120201905	120201966	-0.42	-0.19
Trappc3	chr4	125939648	125949968	-0.91	-0.16
Trappc3	chr4	125951220	125952918	-1.33	0.11
Clspn	chr4	126237254	126239019	-2.37	-0.64
Clspn	chr4	126243253	126249549	-1.57	-0.49
Clspn	chr4	126270339	126270758	-1.05	-0.48
Phc2	chr4	128404881	128405061	-0.49	0.29
Fuca1	chr4	135495027	135495325	-1.89	0.27
Usp48	chr4	137212056	137213524	-1.00	0.10
Mul1	chr4	137996388	137996837	-0.57	-0.15
Capzb	chr4	138748845	138794826	-1.20	-0.15
Klhl21	chr4	151383838	151389959	-1.59	-0.17
Klhl21	chr4	151390257	151390408	-2.24	0.94
Icmt	chr4	151674797	151677845	-1.15	-0.14
Manea	chr4	26254667	26256281	-2.07	0.28
Ddx58	chr4	40152603	40155517	-1.09	-0.14
Ddx58	chr4	40186678	40186858	-0.63	0.23
B4galt1	chr4	40753786	40754830	-2.74	0.20
B4galt1	chr4	40770597	40800583	-1.25	0.19
B4galt1	chr4	40800821	40800971	-2.38	-0.70
Vcp	chr4	43013259	43013349	-0.47	-0.15
Gne	chr4	44047905	44048266	-3.23	-0.97
Gne	chr4	44068129	44079645	-1.35	-0.23
Fbxo10	chr4	45048105	45048374	-0.54	-0.64
Fbxo10	chr4	45048672	45048852	-0.34	0.51

Fbxo10	chr4	45071353	45071981	-0.56	-0.37
Alg2	chr4	47482973	47483543	-0.65	0.08
Mrpl50	chr4	49526415	49527334	-1.38	-0.13
Mysm1	chr4	94631863	94632432	-1.52	0.19
B4galt2	chr4	117546388	117546538	-2.37	2.64
Dph2	chr4	117562463	117563268	-3.28	-0.40
Dph2	chr4	117564387	117564628	-0.53	-0.14
St3gal3	chr4	117604757	117612678	-1.50	-0.18
Pigv	chr4	133218200	133220631	-0.38	-0.05
Dhdds	chr4	133526767	133536084	-3.00	-0.27
Man1c1	chr4	134119221	134119671	-1.30	0.19
Otud3	chr4	138451293	138453499	-1.99	-0.27
Fbxo6	chr4	147520122	147520183	-0.29	0.01
Pex14	chr4	148358178	148473921	-0.30	0.00
Spsb1	chr4	149271976	149281277	-0.37	-0.15
Nupl2	chr5	23688056	23689616	-0.80	0.11
Nub1	chr5	24191751	24195229	-0.32	-0.04
Nub1	chr5	24216073	24216194	-1.10	-0.40
Galnt11	chr5	24771142	24771321	-1.45	-0.43
Bst1	chr5	44210161	44210281	-0.88	-0.46
Klhl5	chr5	65558241	65558691	-1.07	-0.19
Dcun1d4	chr5	73950683	73951373	-0.84	0.26
Exoc1	chr5	76998801	76999310	-0.75	0.14
Uso1	chr5	92630205	92631402	-0.84	-0.06
Pigg	chr5	108776959	108777289	-0.56	-0.20
Usp30	chr5	114572332	114572933	-2.34	-0.40
Wsb2	chr5	117827434	117828091	-2.97	-0.26
Fbxo21	chr5	118452093	118458648	-2.38	-0.37
Psmd9	chr5	123678228	123678437	-1.01	0.20
Sumf2	chr5	130338841	130338932	-0.89	-1.00
Kctd7	chr5	130628217	130628755	-1.29	-0.61
Vgf	chr5	137506871	137509013	-0.62	-0.14
Ache	chr5	137731508	137731867	-0.71	-0.85
Cops6	chr5	138605129	138605308	-1.66	0.48
Fam20c	chr5	139284018	139286017	-3.49	-0.20
Foxk1	chr5	142931509	142934770	-1.58	0.09
Kdelr2	chr5	144165498	144165618	-0.81	-0.20
Preb	chr5	31254099	31254430	-3.13	0.83
Preb	chr5	31254579	31254819	-1.65	0.61
Preb	chr5	31255148	31255718	-1.53	0.29
Preb	chr5	31256197	31256528	-1.13	0.29
Preb	chr5	31257126	31257247	-0.79	-0.36
Preb	chr5	31257366	31258308	-0.98	-0.07

Tnip2	chr5	34839311	34839491	-0.39	0.07
Tnip2	chr5	34842145	34842355	-1.03	0.23
Tada2b	chr5	36818258	36826725	-1.77	0.18
Wfs1	chr5	37358240	37358331	-1.75	-1.37
Wfs1	chr5	37358929	37359708	-1.86	-0.46
Fbxl5	chr5	44142150	44149098	-0.30	0.01
Fbxl5	chr5	44149396	44151063	-1.01	0.09
Nup54	chr5	92845101	92846543	-0.74	0.09
Sec31a	chr5	100790907	100791058	-1.68	-0.19
Mlec	chr5	115597834	115600228	-0.81	-0.07
Suds3	chr5	117542584	117542943	-0.42	-0.03
Suds3	chr5	117558427	117565972	-3.36	-0.34
Fbxw8	chr5	118515497	118515767	-0.43	-0.12
Eif2b1	chr5	125020669	125021791	-1.66	0.15
Ubc	chr5	125867967	125870328	-2.39	0.10
Fbxl18	chr5	143640471	143644134	-2.38	-0.99
Usp42	chr5	144471642	144472211	-0.41	-0.06
Usp42	chr5	144477517	144477978	-2.04	-0.45
Usp12	chr5	147548927	147550725	-0.54	0.07
Calu	chr6	29298148	29306471	-0.77	-0.32
Calu	chr6	29322584	29324851	-1.99	0.11
Exoc4	chr6	33199149	33199210	-0.30	0.03
Nup205	chr6	35127615	35133895	-0.46	0.13
Rab19	chr6	39339614	39340231	-1.34	-0.45
Cul1	chr6	47474617	47476108	-2.74	-0.21
Tnip3	chr6	65564764	65582063	-2.52	0.23
Eif2ak3	chr6	70794580	70794730	-1.47	1.87
Eif2ak3	chr6	70840206	70843273	-0.84	-0.13
Eif2ak3	chr6	70843421	70850228	-0.60	0.22
Eif2ak3	chr6	70854128	70854726	-0.40	-0.04
St3gal5	chr6	72092201	72097116	-0.54	-0.05
St3gal5	chr6	72099116	72104206	-1.29	0.11
Dctn1	chr6	83140226	83141007	-1.23	-0.14
Cct7	chr6	85402007	85402187	-0.79	-0.15
Mitf	chr6	97960393	97968956	-2.29	-0.13
Mitf	chr6	97969614	97970094	-1.49	-0.18
Edem1	chr6	108805488	108805698	-0.64	-0.04
Ttll3	chr6	113357235	113359288	-2.43	0.39
Ttll3	chr6	113362895	113364057	-1.63	-0.42
Vhl	chr6	113578147	113579978	-1.22	0.20
Fbxl14	chr6	119429685	119431421	-0.82	0.06
Rad52	chr6	119865354	119869071	-0.36	0.02
Rad52	chr6	119871318	119872841	-0.33	-0.02

Usp18	chr6	121195923	121196013	-1.05	-1.68
Mrpl51	chr6	125143277	125143516	-0.40	0.02
Cmas	chr6	142705235	142705504	-3.50	-0.70
Mrps35	chr6	147019127	147019424	-0.64	-0.10
Klhl42	chr6	147039984	147056940	-0.79	-0.10
Etfbkmt	chr6	149092586	149092885	-3.29	-1.16
Etfbkmt	chr6	149095803	149099215	-3.09	-1.29
Ube2h	chr6	30163827	30164395	-0.70	-0.09
Ube2h	chr6	30164783	30164963	-0.64	0.31
Hipk2	chr6	38697991	38787107	-0.63	0.02
Hipk2	chr6	38825829	38826040	-1.39	-0.22
Nod1	chr6	54893317	54893527	-0.62	1.79
Nod1	chr6	54894659	54894899	-0.40	-0.75
Ptcd3	chr6	71830841	71831536	-3.58	-0.34
Rnf181	chr6	72310209	72310535	-1.67	-0.25
Tgoln1	chr6	72566903	72566994	-0.12	0.00
Mrpl19	chr6	81911638	81912330	-1.58	0.13
Rab43	chr6	87761473	87761773	-0.67	-0.19
Nup210	chr6	90963060	90963630	-0.69	-0.12
Xpc	chr6	91439837	91440135	-1.44	-0.58
Mrps25	chr6	92124773	92125193	-1.59	-0.19
Sumf1	chr6	108058060	108058479	-1.21	0.20
Sumf1	chr6	108135367	108135577	-1.99	-0.39
Rad18	chr6	112577091	112577691	-0.33	0.02
Tada3	chr6	113316922	113317131	-1.20	0.33
Tada3	chr6	113322460	113324488	-0.48	0.11
Tada3	chr6	113324725	113325142	-2.83	0.54
Phc1	chr6	122275560	122275800	-2.40	-0.84
Usp5	chr6	124776424	124779465	-0.32	0.01
Ccdc8	chr7	17580174	17580295	-0.16	0.42
B3gnt8	chr7	26415160	26415521	-1.98	-0.29
Josd2	chr7	51723425	51723545	-0.38	0.08
Napsa	chr7	51827934	51827995	-1.32	-0.53
Nup62	chr7	52071739	52086176	-1.81	-0.05
Hrc	chr7	52590728	52591119	-0.83	-0.95
Hrc	chr7	52591508	52591689	-0.33	0.42
Prmt3	chr7	57112858	57113008	-0.98	-0.21
Klhl25	chr7	82993313	83012167	-0.53	-0.05
Klhl25	chr7	83018746	83018926	-0.98	-0.56
Ctsc	chr7	95437274	95437455	-0.26	0.00
Ctsc	chr7	95458037	95458487	-2.08	0.25
Rab30	chr7	99984220	99984700	-0.22	-0.11
Alg8	chr7	104540130	104540578	-0.40	0.05

Arrb1	chr7	106748328	106749813	-0.38	-0.04
Arrb1	chr7	106752471	106752711	-0.95	-0.19
Ctr9	chr7	118172674	118177414	-0.59	-0.08
Ctr9	chr7	118198901	118199651	-0.33	0.01
Usp47	chr7	119167079	119167170	-0.67	0.25
Usp47	chr7	119234949	119245400	-0.57	0.03
Usp47	chr7	119252192	119254032	-1.45	0.15
Spon1	chr7	121183532	121183832	-1.33	-0.98
Ccp110	chr7	125865000	125865449	-3.00	-0.57
Ccp110	chr7	125865897	125867971	-0.41	0.05
Dctn5	chr7	129276554	129277689	-0.62	0.13
Dctn5	chr7	129291850	129292499	-2.82	0.17
Fbxl19	chr7	134911014	134911817	-1.61	-0.24
Fbxl19	chr7	134911935	134912442	-2.06	-0.46
Abraxas2	chr7	140072975	140075508	-1.01	0.09
Zranb1	chr7	140122884	140158338	-0.66	0.06
Zranb1	chr7	140174515	140175634	-1.19	-0.23
Zranb1	chr7	140175516	140175874	-0.37	-0.03
Pnpla2	chr7	148641086	148641236	-0.41	0.07
Pnpla2	chr7	148645626	148646045	-0.65	-0.08
Tfpt	chr7	3581426	3581486	-1.18	-0.65
Ube2m	chr7	13623108	13623619	-2.32	-0.37
Sae1	chr7	16912865	16913105	-0.52	-0.05
Calm3	chr7	17502039	17502309	-2.51	-0.14
Apoe	chr7	20282157	20282891	-2.78	-0.52
Ceacam1	chr7	26247769	26248608	-3.38	0.29
Ceacam1	chr7	26256984	26258859	-1.12	-0.38
Ceacam1	chr7	26259008	26274241	-1.35	0.18
Sptbn4	chr7	28189491	28189582	-0.15	0.17
Mrps12	chr7	29524778	29525105	-1.17	-0.21
Nucb1	chr7	52758551	52762906	-2.24	-0.25
Spaca4	chr7	52980801	52981068	-0.84	0.89
Spaca4	chr7	52981126	52981186	-0.15	-0.15
Nsmce3	chr7	72016624	72017896	-1.52	-0.13
Asb7	chr7	73792504	73823980	-1.38	-0.19
Mrpl46	chr7	85920226	85925350	-1.42	0.21
Man2a2	chr7	87496117	87497882	-1.79	-0.10
Blm	chr7	87647292	87647681	-0.45	0.09
Blm	chr7	87654437	87658340	-2.18	0.36
Neu3	chr7	106961618	106962752	-2.05	0.46
Nup98	chr7	109268003	109269174	-0.67	-0.03
Usp17ld	chr7	110398629	110398808	-0.25	-0.42
Mrpl17	chr7	112958202	112958622	-2.02	0.12

Dcun1d3	chr7	127000917	127038861	-3.18	-0.26
Cog7	chr7	129066679	129066829	-3.22	-0.98
Cog7	chr7	129121227	129125207	-0.83	-0.17
Nsmce4a	chr7	137690685	137690865	-1.34	-0.83
Trappc5	chr8	3676506	3679785	-2.29	-0.19
Trappc5	chr8	3680083	3680622	-2.14	-0.39
F7	chr8	13034819	13035449	-1.19	-0.75
Cul4a	chr8	13144401	13146773	-2.17	0.30
Cul4a	chr8	13146892	13147072	-1.09	-0.25
Thsd1	chr8	23353890	23354367	-0.39	1.22
Thsd1	chr8	23368989	23369377	-0.36	-1.64
Ank1	chr8	24242672	24243002	-0.59	-0.63
Cope	chr8	72826683	72826743	-0.97	-0.26
Fkbp8	chr8	73051641	73053542	-2.28	-0.16
Babam1	chr8	73920753	73921677	-0.98	-0.13
Mrpl34	chr8	73988824	73989652	-1.48	-0.14
Dda1	chr8	73998687	73999436	-1.57	-0.10
Dda1	chr8	73999764	73999855	-0.77	0.18
Rab8a	chr8	74704051	74704703	-1.19	-0.09
Nr3c2	chr8	79433507	79433776	-0.32	-0.42
Cyld	chr8	91220956	91221047	-0.33	-0.20
Mmp2	chr8	95374128	95376723	-0.68	0.53
Mmp2	chr8	95377050	95377319	-0.32	0.09
Fbxl8	chr8	107791952	107793137	-2.37	-0.45
Cog4	chr8	113405654	113406134	-0.74	-0.12
St3gal2	chr8	113444003	113491381	-0.85	-0.10
St3gal2	chr8	113493520	113493610	-0.45	0.29
St3gal2	chr8	113493699	113494575	-1.50	-0.33
St3gal2	chr8	113494724	113495173	-3.09	-0.60
Usp10	chr8	122480587	122481247	-1.81	-0.16
Trappc2l	chr8	125135525	125136985	-0.52	-0.21
Tubb3	chr8	125944632	125944960	-0.21	-0.02
Galnt2	chr8	126864828	126868362	-1.82	0.19
Dcun1d2	chr8	13256291	13257907	-0.80	-0.08
Gas6	chr8	13465641	13465821	-3.11	-0.71
Vdac3	chr8	23687783	23688050	-2.48	0.31
Pomk	chr8	27092830	27098060	-0.32	0.01
Dctn6	chr8	35169406	35171536	-0.90	0.26
Ing2	chr8	48753850	48754661	-0.78	0.10
Ing2	chr8	48760302	48760513	-2.46	-1.21
Klhl2	chr8	67219421	67219870	-1.56	-0.30
Ano8	chr8	74000275	74000456	-0.96	-0.91
Ano8	chr8	74000634	74002643	-0.60	0.70

B3gnt3	chr8	74217467	74227314	-0.27	0.00
Rad23a	chr8	87358550	87358761	-2.05	-0.60
Amfr	chr8	96496474	96497102	-1.31	-0.11
Csnk2a2	chr8	97976954	97977225	-1.72	-0.27
Dync1li2	chr8	106966676	106966737	-0.30	-0.05
Cog8	chr8	109574194	109580248	-1.23	0.11
Mbtps1	chr8	122032081	122032770	-0.70	0.06
Mbtps1	chr8	122070114	122082661	-1.48	0.13
Fbxo31	chr8	124075878	124076177	-0.56	0.18
Mvd	chr8	124957554	124958775	-0.56	-0.04
Exoc8	chr8	127418756	127418906	-0.61	-0.27
Exoc8	chr8	127419205	127421575	-0.64	0.06
Mrpl4	chr9	20812982	20813281	-1.52	-0.15
Pigyl	chr9	21961289	21962741	-2.33	-0.36
Opcml	chr9	28210755	28211235	-0.28	-0.82
Opcml	chr9	28211624	28211685	-0.29	-1.22
Opcml	chr9	28729664	28729844	-0.95	-1.68
Opcml	chr9	28730203	28730384	-0.20	0.00
Opcml	chr9	28730533	28730714	-0.53	0.75
Nfrkb	chr9	31218903	31226648	-2.78	0.27
Usp2	chr9	43902061	43902302	-1.62	-0.76
Dpagt1	chr9	44141235	44141504	-0.58	0.09
Apoa1	chr9	46037922	46038460	-0.32	0.95
Usp28	chr9	48849662	48849753	-0.68	0.33
Alg9	chr9	50651063	50651633	-1.34	-0.24
Fbxo22	chr9	55066270	55071642	-1.39	0.12
Fbxo22	chr9	55071791	55072031	-0.79	0.18
Sin3a	chr9	56958532	56958892	-0.66	0.11
Sin3a	chr9	56973115	56975606	-1.32	0.16
Rora	chr9	69226745	69226835	-0.38	-0.37
Adam10	chr9	70526807	70526958	-0.76	0.19
Adam10	chr9	70626086	70626267	-1.49	0.28
Rab27a	chr9	72892731	72908786	-0.70	-1.27
Rab27a	chr9	72943119	72943840	-0.62	-0.22
Tmem115	chr9	107436394	107440987	-1.57	0.09
Usp4	chr9	108293381	108294591	-0.82	0.07
Usp19	chr9	108393096	108395032	-3.28	-0.27
Cdc25a	chr9	109778082	109778888	-0.80	-0.16
Cdc25a	chr9	109794596	109795525	-1.21	0.17
Glb1	chr9	114310242	114326084	-1.04	0.28
Glb1	chr9	114368770	114373539	-0.71	0.08
Dync1li1	chr9	114632686	114632895	-0.48	0.07
Wdr48	chr9	119833344	119835040	-3.23	0.21

Birc2	chr9	7818494	7818943	-1.37	0.33
Birc2	chr9	7834955	7835225	-0.88	0.29
Birc3	chr9	7848939	7850928	-0.65	0.09
Birc3	chr9	7860281	7872961	-1.11	0.10
Fbxl12	chr9	20448880	20449151	-1.08	-0.58
Dnmt1	chr9	20733552	20734608	-0.96	0.27
Keap1	chr9	21034503	21042276	-2.14	0.12
Keap1	chr9	21043235	21043776	-0.68	-0.28
Aplp2	chr9	30957469	30957679	-1.95	-0.19
Aplp2	chr9	30985646	31019400	-1.25	0.28
Arcn1	chr9	44551783	44551994	-2.37	0.20
Arcn1	chr9	44568207	44575891	-1.28	0.17
Rab39	chr9	53494560	53494740	-0.32	0.63
Pml	chr9	58067502	58068341	-3.10	-0.28
Pml	chr9	58080996	58094952	-1.77	-0.21
Pml	chr9	58097382	58097593	-1.39	-0.43
Senp8	chr9	59584151	59585492	-1.58	0.27
Pias1	chr9	62729583	62729943	-1.17	0.27
Pias1	chr9	62799649	62828686	-1.72	0.23
Smad3	chr9	63496369	63496909	-0.75	0.36
Smad3	chr9	63497357	63498046	-0.81	-0.34
Smad3	chr9	63605729	63605891	-0.28	-0.36
Rab11a	chr9	64585502	64585563	-1.25	0.56
Usp3	chr9	66366133	66366372	-0.54	-0.11
Usp3	chr9	66441201	66441261	-0.27	-0.29
Rab8b	chr9	66715068	66767482	-0.50	0.07
Pigb	chr9	72863505	72865261	-2.04	0.25
Fbxo9	chr9	77943550	77943611	-0.20	0.01
Pgm3	chr9	86447697	86448057	-3.55	-0.68
Mrps22	chr9	98498714	98502098	-0.52	0.10
Apeh	chr9	107987744	107987894	-0.37	-0.08
Gorasp1	chr9	119834790	119835029	-0.52	0.12
Sec22c	chr9	121593506	121593866	-1.05	-0.53
Mtrf1l	chr10	4532471	4533463	-0.37	0.07
Shprh	chr10	10869257	10871973	-1.04	-0.18
Shprh	chr10	10882742	10884564	-2.81	-0.36
Fuca2	chr10	13222977	13225667	-1.46	0.28
Sec63	chr10	42548789	42548969	-3.08	-0.29
Nus1	chr10	52153241	52156613	-2.27	-0.23
Nus1	chr10	52158200	52158410	-1.09	-0.22
Ranbp2	chr10	57941534	57941955	-2.61	0.14
Sumo3	chr10	77068978	77069482	-0.67	-0.17
Ube2g2	chr10	77107280	77107637	-1.28	-0.14

Ube2g2	chr10	77108022	77108082	-0.37	-0.06
Eef2	chr10	80644273	80645254	-1.14	-0.02
Eid3	chr10	82329548	82330082	-0.66	-0.10
Galnt4	chr10	98571396	98572084	-0.88	0.20
Galnt4	chr10	98572412	98573220	-1.11	0.24
Ccdc59	chr10	105278534	105284446	-0.79	0.06
Pcmt1	chr10	7350508	7350747	-1.21	-0.26
Tnfaip3	chr10	18721581	18725108	-0.69	-0.06
Rnf146	chr10	29064221	29064521	-3.45	0.40
Rnf146	chr10	29066497	29067546	-0.61	0.05
Man1a	chr10	53795361	53795512	-0.40	-0.49
Ube2d1	chr10	70718174	70718562	-1.17	0.19
Trappc10	chr10	77650129	77651570	-0.54	-0.05
Pias4	chr10	80616715	80617300	-1.40	-0.19
Mrpl54	chr10	80727466	80729671	-1.32	0.11
Ckap4	chr10	83990001	83991399	-0.32	0.00
Ckap4	chr10	83996275	83996633	-1.47	-0.37
Rab21	chr10	114727694	114731953	-1.61	0.13
Os9	chr10	126531494	126532125	-0.76	-0.17
Os9	chr10	126535916	126536493	-1.03	-0.18
Os9	chr10	126557594	126558216	-1.54	-0.23
Mbd6	chr10	126719101	126719461	-1.32	0.15
Mbd6	chr10	126719550	126720682	-2.45	-0.19
Ykt6	chr11	5855760	5856000	-1.50	-0.31
Ykt6	chr11	5866082	5866381	-2.00	0.22
Usp34	chr11	23206894	23233439	-0.33	0.02
Usp34	chr11	23233527	23233678	-2.09	0.31
Usp34	chr11	23263863	23264511	-0.40	0.01
Usp34	chr11	23388308	23389213	-1.84	0.16
Psme4	chr11	30671804	30671925	-0.83	-0.91
Psme4	chr11	30672014	30672135	-0.38	0.12
Fbxw11	chr11	32644536	32645016	-2.21	0.20
Mgat4b	chr11	50039046	50039137	-1.58	-0.54
Galnt10	chr11	57598076	57598614	-0.49	-0.14
Nlrp3	chr11	59379889	59380129	-0.80	-0.15
Adora2b	chr11	62062485	62062754	-0.32	-0.11
Pigl	chr11	62271961	62272052	-2.04	-1.11
Trp53	chr11	69393860	69394608	-0.68	0.14
Asgr1	chr11	69870833	69871366	-0.46	1.72
Ube2g1	chr11	72493612	72497325	-2.70	0.54
Rtn4rl1	chr11	75079893	75079983	-0.50	3.17
Pigs	chr11	78141953	78142237	-1.31	-0.70
Pigs	chr11	78155437	78156068	-0.60	-0.05

Suz12	chr11	79843530	79845978	-2.27	0.14
Psmd11	chr11	80285544	80285635	-1.72	-0.32
Ptrh2	chr11	86497514	86503722	-0.63	0.05
Mrps23	chr11	88024206	88024950	-1.49	0.16
Gngt2	chr11	95703995	95704054	-0.73	-0.59
Mrpl10	chr11	96909612	96910055	-1.05	0.12
Thra	chr11	98603186	98617447	-0.55	0.14
Thra	chr11	98625630	98626259	-2.85	-0.46
Rara	chr11	98799009	98808938	-1.27	-0.44
Psme3	chr11	101177564	101178336	-0.63	-0.07
Psme3	chr11	101182070	101183288	-1.70	-0.15
Dcaf7	chr11	105898185	105898485	-0.69	-0.09
Dcaf7	chr11	105916210	105916330	-0.89	-0.17
Dcaf7	chr11	105919829	105920099	-0.59	-0.07
Arsg	chr11	109346860	109378636	-0.37	-0.02
Arsg	chr11	109433779	109434107	-1.59	0.43
Sphk1	chr11	116393254	116393851	-0.68	3.20
Birc5	chr11	117716547	117716877	-3.30	-0.42
Engase	chr11	118348531	118349162	-0.37	-0.08
Cbx2	chr11	118885531	118887831	-0.67	1.03
Gps1	chr11	120648708	120649402	-0.62	0.20
Gps1	chr11	120649969	120650416	-1.12	-0.20
Foxk2	chr11	121161028	121169022	-2.30	0.14
Fn3k	chr11	121303142	121303232	-0.53	-3.32
Fn3k	chr11	121303589	121303799	-0.67	-3.37
Mrps24	chr11	5604429	5604637	-1.33	0.18
B3gnt2	chr11	22735900	22759719	-0.66	0.02
Commd1	chr11	22799727	22800056	-1.19	0.13
Commd1	chr11	22856647	22878367	-0.86	0.20
Commd1	chr11	22878962	22882284	-0.58	-0.15
Pex13	chr11	23548927	23549226	-0.93	0.25
Pex13	chr11	23551042	23555901	-0.65	-0.10
Sptbn1	chr11	30017714	30018324	-2.28	0.32
Sptbn1	chr11	30036124	30038367	-0.64	0.05
Sptbn1	chr11	30038816	30040543	-1.03	-0.10
Sptbn1	chr11	30119730	30168025	-0.45	0.12
Stc2	chr11	31260009	31260460	-0.39	-0.10
0610009B22Rik	chr11	51499121	51499534	-0.41	-0.04
Sec24a	chr11	51569959	51570170	-2.57	-1.70
Tnip1	chr11	54769581	54776442	-0.41	0.13
Nup88	chr11	70783414	70783475	-0.56	0.19
Derl2	chr11	70823390	70826702	-1.07	-0.09
Derl2	chr11	70832716	70832777	-0.54	0.26

Hic1	chr11	74979054	74979175	-0.19	-0.46
Hic1	chr11	74979353	74979534	-0.28	-0.54
Hic1	chr11	74980731	74980792	-0.14	-0.42
Rpa1	chr11	75154547	75161885	-2.86	-0.51
Gosr1	chr11	76543085	76548136	-1.36	0.15
Eral1	chr11	77887232	77887988	-0.99	-0.14
Wsb1	chr11	79053599	79053898	-1.62	-0.11
Pex12	chr11	83109905	83111913	-1.20	0.24
Pigw	chr11	84691211	84691984	-0.64	-0.09
Ube2z	chr11	95911494	95911704	-3.10	-0.34
Fbxl20	chr11	97950510	97953462	-0.36	0.03
Klhl11	chr11	100333798	100334096	-1.09	0.31
Rab5c	chr11	100581475	100599444	-1.38	-0.37
Becn1	chr11	101163065	101163581	-2.50	-0.31
Brca1	chr11	101384814	101385204	-1.75	-0.62
Brca1	chr11	101385473	101386581	-0.92	-0.20
Brca1	chr11	101386909	101387659	-3.36	0.64
Gosr2	chr11	103540134	103540702	-0.78	-0.07
Nsf	chr11	103684382	103685071	-0.57	-0.08
Nsf	chr11	103792113	103815310	-0.41	0.18
Ern1	chr11	106259082	106261558	-1.71	0.18
Smurf2	chr11	106683743	106684358	-0.92	-0.20
Mrpl38	chr11	115993130	115993341	-1.02	0.18
Mrpl38	chr11	115999521	115999702	-0.89	0.42
Exoc7	chr11	116149998	116150478	-0.48	-0.10
Cbx8	chr11	118899867	118901442	-1.43	0.25
Cbx4	chr11	118940531	118940592	-1.14	1.49
Cbx4	chr11	118941070	118941400	-3.20	1.23
Cbx4	chr11	118941759	118947461	-0.50	0.02
Csnk1d	chr11	120819778	120820048	-1.21	-0.15
Csnk1d	chr11	120823779	120824080	-1.10	-0.14
Csnk1d	chr11	120852347	120852438	-1.28	-0.33
B3gntl1	chr11	121478331	121479112	-0.57	-0.07
Asxl2	chr12	3426883	3442534	-0.98	-0.12
Asxl2	chr12	3496396	3496996	-0.33	0.01
Smc6	chr12	11292342	11292522	-1.73	0.67
Smc6	chr12	11324583	11324974	-0.83	0.12
Psma6	chr12	56499811	56499871	-1.67	-0.32
Lrr1	chr12	70275442	70276002	-0.27	0.05
Fut8	chr12	78432957	78466016	-1.24	0.35
Fut8	chr12	78576193	78576884	-0.99	0.24
Eif2b2	chr12	86567102	86567459	-0.31	-0.01
Ttll5	chr12	87165622	87165743	-0.32	-0.11

Psmc1	chr12	101348377	101351286	-1.53	-0.29
Calm1	chr12	101445209	101445479	-1.73	0.08
Otub2	chr12	104643783	104643844	-0.48	0.32
Dync1h1	chr12	111839634	111839815	-0.77	-0.17
Dync1h1	chr12	111855964	111857506	-0.91	0.03
Dync1h1	chr12	111904592	111905154	-0.63	0.04
Wdr20	chr12	112017684	112032696	-0.32	-0.01
Traf3	chr12	112499736	112500694	-0.44	0.03
Eif5	chr12	112776401	112777093	-0.85	0.08
Btbd6	chr12	114215550	114217061	-3.22	-0.34
Mta1	chr12	114374970	114375239	-3.26	-0.32
Ncoa1	chr12	4247630	4248379	-1.43	-0.26
Ncoa1	chr12	4248916	4249007	-0.66	-0.36
Ncoa1	chr12	4249155	4252804	-0.79	-0.16
Ncoa1	chr12	4267739	4267919	-0.46	-0.11
Ncoa1	chr12	4294980	4296087	-2.92	0.28
Trappc6b	chr12	60162329	60162448	-2.54	-0.76
Sptb	chr12	77714030	77714151	-0.25	-1.14
Rab15	chr12	77899366	77899605	-0.77	2.23
Rab15	chr12	77900616	77900676	-0.21	-1.00
Pigh	chr12	80183182	80183482	-0.71	-0.23
Dcaf5	chr12	81437908	81438268	-1.65	-0.32
Dcaf5	chr12	81439342	81449328	-0.57	0.04
Pomt2	chr12	88449791	88451254	-0.31	-0.01
Sel1l	chr12	93080291	93087597	-3.35	-0.59
Atxn3	chr12	103157140	103157589	-0.55	0.09
Ankrd9	chr12	112214580	112215269	-1.06	-0.47
Ero1lb	chr13	12658149	12658389	-0.65	0.17
Ero1lb	chr13	12699110	12699291	-0.39	0.11
Inhba	chr13	16106343	16119044	-1.11	1.74
Trim27	chr13	21283975	21284426	-1.81	0.13
Hist1h2bg	chr13	23663268	23663474	-0.87	-0.07
Hist1h2bc	chr13	23776067	23776213	-1.76	0.26
Hist1h2ab	chr13	23842956	23843224	-1.74	-0.27
Ripk1	chr13	34119794	34126112	-1.97	0.13
2010111I01Rik	chr13	63116375	63134452	-2.12	0.31
2010111I01Rik	chr13	63400033	63400274	-1.72	-0.16
2010111I01Rik	chr13	63400901	63403592	-1.03	-0.08
Mrpl36	chr13	73468456	73468515	-0.39	0.16
Gfm2	chr13	97945566	97945897	-0.42	-0.06
Mrps27	chr13	100184740	100185516	-0.32	-0.01
Ercc8	chr13	108948931	108950875	-0.50	-0.19
Gmds	chr13	31911574	31911785	-0.51	-0.13

Tubb2b	chr13	34219290	34219557	-1.15	1.09
Tubb2b	chr13	34219999	34221135	-0.49	0.22
Nup153	chr13	46776228	46776529	-1.65	0.22
Uimc1	chr13	55129866	55132327	-1.14	0.25
Uimc1	chr13	55176900	55177348	-2.31	-0.33
Rab24	chr13	55421293	55421501	-1.79	-0.26
Rab24	chr13	55422955	55423341	-2.38	-0.32
Lman2	chr13	55447467	55447708	-0.64	-0.09
Lman2	chr13	55448036	55448306	-2.19	-0.17
Hnrnpk	chr13	58501349	58504674	-0.47	-0.03
Golm1	chr13	59738440	59741498	-0.33	0.02
Exoc3	chr13	74327576	74344379	-2.42	-0.18
Slc30a5	chr13	101572752	101573383	-1.55	0.14
Kctd6	chr14	9052713	9055546	-1.19	-0.22
Atxn7	chr14	14932503	14936225	-1.34	-0.13
Atxn7	chr14	14936403	14936644	-1.24	-0.33
Actr8	chr14	30804041	30804784	-2.92	-0.88
Bap1	chr14	32071949	32072607	-1.80	-0.20
Psmc6	chr14	45949498	45950514	-0.68	0.11
Ktn1	chr14	48283451	48283932	-0.75	0.08
Oxa1l	chr14	54988035	54988365	-0.95	0.14
Mrpl52	chr14	55045745	55045901	-1.68	0.33
Dcaf11	chr14	56178865	56179786	-0.52	0.29
Dcaf11	chr14	56188485	56188902	-0.36	0.03
Mrpl57	chr14	58445075	58445797	-1.44	-0.11
Kbtbd7	chr14	79827126	79828716	-0.67	0.13
Gng2	chr14	20694858	20796232	-0.41	-0.04
Spcs1	chr14	31813159	31813397	-2.40	0.19
Ankrd28	chr14	32514699	32518527	-0.54	-0.04
Dph3	chr14	32893956	32894255	-0.43	0.13
Dph3	chr14	32896016	32898124	-3.51	0.32
Rabggta	chr14	56340688	56340807	-0.30	0.04
Cog3	chr14	76103115	76106953	-1.26	-0.15
Commd6	chr14	102033281	102039330	-3.04	0.28
Fbxl3	chr14	103482030	103488661	-0.97	0.07
Fbxl3	chr14	103491640	103498726	-2.05	0.22
Sema5a	chr15	32616573	32620160	-0.80	-0.13
Dcaf13	chr15	38944419	38944568	-0.30	-0.01
Rnf139	chr15	58729987	58731846	-2.60	0.15
Commd5	chr15	76730370	76731727	-1.87	-0.20
Apol9b	chr15	77566250	77566664	-0.63	0.63
Tab1	chr15	79989200	79992132	-3.29	-0.26
Ep300	chr15	81416643	81438767	-1.28	-0.07

Ep300	chr15	81479273	81481819	-1.93	-0.07
L3mbtl2	chr15	81516741	81518556	-1.99	-0.18
Nup50	chr15	84757805	84765852	-2.65	-0.15
Nup50	chr15	84770131	84771000	-1.89	-0.14
Eif4b	chr15	101925104	101925897	-3.00	-0.10
Skp2	chr15	9043329	9043510	-0.80	-0.31
Skp2	chr15	9069207	9070137	-0.35	-0.03
Otulin	chr15	27535675	27538550	-1.58	-0.11
Otulin	chr15	27553110	27560448	-2.84	-0.65
6-Mar	chr15	31388351	31388771	-0.48	-0.05
Rad21	chr15	51794834	51794984	-1.95	0.28
Rad21	chr15	51798216	51799422	-1.38	0.19
Mrpl13	chr15	55386902	55388867	-0.39	0.08
Derl1	chr15	57701565	57701686	-0.33	0.03
Derl1	chr15	57702583	57703393	-2.02	-0.09
Fbxo32	chr15	58011932	58012112	-0.37	-0.51
Fbxo32	chr15	58012410	58013069	-1.76	-1.01
Fbxo32	chr15	58046282	58046433	-1.44	-1.83
St3gal1	chr15	66944067	67005237	-2.70	-0.20
Apol9a	chr15	77234337	77235198	-0.69	-0.40
Apol10b	chr15	77415950	77416129	-0.15	-0.07
Csf2rb2	chr15	78112969	78113060	-1.96	0.70
Csf2rb2	chr15	78114648	78115039	-2.56	-0.44
Csf2rb2	chr15	78115098	78116449	-0.69	-0.10
Ddx17	chr15	79376930	79377021	-2.48	-0.43
Josd1	chr15	79506286	79506525	-1.88	-0.45
Josd1	chr15	79517170	79517588	-0.55	-0.09
Josd1	chr15	79518003	79518153	-1.50	-0.62
Rangap1	chr15	81535546	81535636	-0.36	0.04
Rangap1	chr15	81556518	81556579	-0.84	0.35
Rangap1	chr15	81560168	81560349	-0.56	-0.15
Pmm1	chr15	81781535	81781891	-0.64	-0.13
Ttll12	chr15	83406959	83407199	-2.44	-0.49
Alg12	chr15	88635911	88636421	-1.79	-0.29
Arsa	chr15	89303025	89303862	-1.00	-0.17
Arsa	chr15	89305988	89306615	-2.33	-0.41
Vdr	chr15	97687183	97687273	-0.18	0.38
Senp1	chr15	97872854	97873333	-1.81	-0.22
Asb8	chr15	97965969	97971774	-0.40	-0.02
Galnt6	chr15	100522228	100522617	-0.68	0.17
Galnt6	chr15	100544605	100550892	-0.66	0.07
Alg1	chr16	5244821	5245001	-2.03	-0.46
Eif4g1	chr16	20679306	20680085	-2.29	0.06

Senp2	chr16	22035885	22036066	-0.89	-0.68
Senp2	chr16	22046889	22047129	-1.44	-0.24
Rnf168	chr16	32277666	32278386	-0.70	-0.12
Rnf168	chr16	32298678	32299428	-1.44	0.24
Golgb1	chr16	36898590	36898921	-0.57	0.08
Golgb1	chr16	36911968	36914157	-0.85	0.05
Golgb1	chr16	36914935	36915176	-1.27	0.23
Golgb1	chr16	36915445	36916525	-2.10	0.18
Golgb1	chr16	36932061	36932212	-1.22	0.38
Golgb1	chr16	36932271	36933171	-1.55	-0.18
Tomm70a	chr16	57152724	57153084	-0.86	0.07
Usp25	chr16	77115246	77116156	-0.36	-0.01
Usp16	chr16	87477080	87480005	-0.31	0.00
Usp7	chr16	8738344	8757028	-1.22	-0.49
Gspt1	chr16	11218069	11218249	-1.35	0.45
Mrpl40	chr16	18872286	18872638	-2.95	-0.32
Senp5	chr16	31963382	31965869	-1.09	-0.20
Senp5	chr16	31989077	31990517	-2.36	0.14
Sec22a	chr16	35313658	35318950	-0.52	0.07
Nr1i2	chr16	38249474	38251285	-1.74	3.10
Nrip1	chr16	76291226	76295153	-0.33	-0.01
Nrip1	chr16	76352759	76374042	-1.73	-0.89
Cct8	chr16	87496053	87496114	-1.39	0.33
Tulp4	chr17	6232220	6232851	-0.86	-0.11
Tulp4	chr17	6232910	6233480	-0.35	0.03
Tulp4	chr17	6234109	6236560	-1.49	0.35
Tcp1	chr17	13117452	13117603	-1.11	-0.28
Abca3	chr17	24546305	24547146	-0.75	-0.09
Spsb3	chr17	25023648	25024470	-2.71	-0.65
Spsb3	chr17	25027107	25029092	-0.56	0.04
Mrps34	chr17	25032064	25033497	-2.86	0.20
Axin1	chr17	26330968	26332096	-2.64	-0.40
Adamts10	chr17	33665942	33666153	-2.67	-0.99
Adamts10	chr17	33667199	33667439	-1.77	-0.90
Psmb8	chr17	34335288	34335468	-2.26	-0.35
Neu1	chr17	35071556	35072840	-0.43	0.02
Mdc1	chr17	35983321	35985582	-0.49	0.04
Mdc1	chr17	35987884	35987945	-0.39	0.10
Mdc1	chr17	35989459	35989938	-0.32	0.01
Mdc1	chr17	35994492	35994583	-1.40	0.43
Mrpl14	chr17	45835027	45835444	-0.94	-0.11
Mrps18a	chr17	46247952	46262241	-1.07	-0.13
Cul7	chr17	46788096	46788277	-3.52	5.23

Cul7	chr17	46788546	46788937	-0.63	-0.57
Cul7	chr17	46790989	46791170	-0.24	0.13
Mrps10	chr17	47505835	47506167	-2.06	-0.94
Rab5a	chr17	53618797	53623279	-1.97	-0.24
Fem1a	chr17	56400758	56401238	-0.31	0.01
Fem1a	chr17	56401745	56402672	-0.64	-0.09
Safb	chr17	56733278	56735128	-0.91	0.09
Safb	chr17	56738105	56738646	-2.60	-0.23
Man2a1	chr17	64950988	64974652	-0.54	-0.07
Slc30a6	chr17	74822435	74823092	-0.96	0.14
Socs5	chr17	87520136	87534893	-3.18	0.20
Mrpl18	chr17	13104220	13104550	-2.87	0.32
Ccnf	chr17	24360684	24360924	-0.81	0.23
Ube2i	chr17	25398025	25398746	-3.13	-0.38
Rab40c	chr17	26020195	26020794	-2.34	-0.34
Pigq	chr17	26073805	26078817	-0.55	-0.05
Rab11b	chr17	33883969	33884119	-3.52	-0.42
Rab11b	chr17	33884327	33884537	-0.51	-0.10
Ring1	chr17	34158095	34160251	-0.37	-0.02
Ly6g6d	chr17	35208321	35208494	-0.16	0.23
Tubb4a	chr17	57220052	57220528	-0.75	-0.24
Tubb4a	chr17	57220705	57221152	-0.66	-0.22
Tubb4a	chr17	57227085	57227175	-0.30	0.20
Rab31	chr17	66001065	66001632	-0.94	0.07
Rab31	chr17	66002078	66002287	-3.48	-0.28
Rab31	chr17	66122031	66122092	-0.23	0.08
Rab12	chr17	66844211	66844961	-0.70	-0.06
Mcfd2	chr17	87654739	87656518	-0.67	0.05
Calm2	chr17	87835011	87846216	-1.79	-0.13
Fbxo11	chr17	88390945	88391656	-2.00	0.16
Fbxo11	chr17	88464415	88464625	-2.23	-0.69
Wac	chr18	7869378	7869558	-0.93	0.30
Wac	chr18	7905960	7910820	-2.90	0.18
Galnt1	chr18	24438485	24443794	-2.39	-0.20
Apc	chr18	34458082	34458232	-2.06	-0.61
Apc	chr18	34470478	34472504	-0.64	0.07
Apc	chr18	34472563	34472923	-0.96	0.19
Apc	chr18	34473880	34475977	-0.83	0.06
Ube2d2a	chr18	35931212	35931392	-0.74	-0.18
Ube2d2a	chr18	35965390	35965540	-0.95	0.36
Commd10	chr18	47246724	47246904	-2.66	0.43
Dctn4	chr18	60686409	60686560	-0.23	-0.01
Dctn4	chr18	60716345	60716496	-0.32	-0.02

Napg	chr18	63154672	63156813	-2.33	-0.27
Sec11c	chr18	65960231	65960321	-0.47	-0.08
Tubb6	chr18	67550384	67552330	-2.55	-0.26
Mbd1	chr18	74437185	74438824	-2.30	-0.33
Mbd1	chr18	74439959	74440678	-0.86	-0.13
Mbd1	chr18	74441560	74442159	-3.42	-0.34
Smad7	chr18	75527018	75527704	-1.68	0.45
Smad2	chr18	76422217	76422308	-0.97	-0.22
Smad2	chr18	76462139	76464727	-1.91	0.24
Pias2	chr18	77304625	77336416	-0.55	0.05
Pias2	chr18	77388818	77391933	-2.14	0.29
Usp14	chr18	10030026	10030117	-0.98	-0.24
B4galt6	chr18	20846332	20847761	-1.10	-0.18
Ino80c	chr18	24263918	24264068	-1.17	-0.32
Ino80c	chr18	24264515	24265143	-0.98	0.15
Etf1	chr18	35064380	35064649	-2.18	0.11
Nr3c1	chr18	39588392	39649457	-2.22	0.16
Tmed7	chr18	46747793	46748242	-2.21	0.16
Lman1	chr18	66140602	66140992	-1.93	-0.18
Lman1	chr18	66162108	66162289	-2.29	-0.31
Smad4	chr18	73799858	73800038	-0.58	0.10
Smad4	chr18	73837362	73837780	-3.05	0.33
Smad4	chr18	73863156	73863395	-2.18	-1.27
Socs6	chr18	89038427	89056418	-2.00	0.18
Sptbn2	chr19	4744144	4744488	-3.16	-1.29
B4gat1	chr19	5039213	5040921	-0.88	-0.09
B4gat1	chr19	5041307	5046422	-3.29	-0.28
B4gat1	chr19	5049005	5049917	-1.92	-0.19
Men1	chr19	6334978	6336811	-2.76	-0.24
Men1	chr19	6338843	6340864	-1.39	-0.08
Trmt112	chr19	6984277	6984947	-1.57	-0.13
Stx5a	chr19	8829416	8830132	-2.12	0.19
Ubxn1	chr19	8945985	8946196	-1.74	-0.30
Mrpl16	chr19	11848726	11849170	-0.77	0.09
Il33	chr19	30033410	30033650	-0.36	1.61
Stambpl1	chr19	34266759	34267030	-1.24	-0.21
Stambpl1	chr19	34307271	34308637	-0.51	-0.08
Exoc6	chr19	37757471	37757650	-1.37	-0.29
Btrc	chr19	45604177	45604358	-1.03	2.52
Gbf1	chr19	46227047	46227228	-0.33	0.03
Nfkb2	chr19	46379346	46380320	-0.71	0.19
Fbxl15	chr19	46403649	46404936	-1.75	-0.27
Adra2a	chr19	54121733	54121794	-0.14	0.26

Adra2a	chr19	54121706	54121796	-0.17	-0.42
Rce1	chr19	4622787	4622995	-2.78	-0.50
Incenp	chr19	9947175	9947265	-0.87	0.15
Incenp	chr19	9949324	9951121	-1.33	-0.18
Incenp	chr19	9958237	9968481	-2.40	0.10
Incenp	chr19	9973932	9974023	-0.33	-0.09
Gcnt1	chr19	17403142	17407678	-0.84	0.08
Smc5	chr19	23348276	23348337	-1.02	-0.47
Mrpl43	chr19	45079562	45080843	-1.75	-0.08
Fbxw4	chr19	45652836	45653256	-0.43	0.04
Actr1a	chr19	46452376	46452913	-1.36	-0.09
Ube2a	chrX	34414360	34414509	-1.29	0.25
Foxo4	chrX	98449866	98450612	-2.10	-0.37
Ogt	chrX	98874610	98878285	-3.11	0.13
Piga	chrX	160857718	160861236	-3.59	0.49
Piga	chrX	160866911	160869851	-2.05	0.57
Piga	chrX	160870327	160870388	-0.37	0.22
Cul4b	chrX	35916890	35916951	-2.43	0.70
Hcfc1	chrX	71189302	71189693	-1.73	-0.14
Hcfc1	chrX	71194650	71196000	-0.95	0.04
Hcfc1	chrX	71211533	71211654	-1.28	0.19
Chm	chrX	110156917	110157037	-1.01	0.43
Mbtps2	chrX	153988740	153989011	-2.22	0.41
Mbtps2	chrX	154006232	154006563	-0.90	-0.32
Yy2	chrX	154005662	154006559	-1.40	0.26
Yy2	chrX	154005166	154005227	-1.85	1.67
Yy2	chrX	154005615	154006545	-1.57	0.28
Vcpip1	chr1	9714443	9738463	-1.88	0.05
Napb	chr2	148538465	148558156	-1.00	0.56
Tnks	chr8	36028534	36028744	-1.19	-0.36
Dynll2	chr11	87794645	87799923	-0.33	-0.01
Dynll2	chr11	87800072	87801030	-2.21	-0.32

Post translational protein modification

gene_name	chr	chromStart	chromEnd	diff.lg.p	diff.log2.fc
Rab23	chr1	33795210	33796952	-0.42	-0.04
Sp100	chr1	87605784	87606023	-1.37	0.49
Psmd1	chr1	87961193	87961254	-0.37	-0.11
Cops8	chr1	92508845	92509678	-0.50	0.03
Ube2f	chr1	93182033	93182602	-1.08	-0.09
Asb1	chr1	93440810	93443711	-1.60	-0.49
Asb1	chr1	93451373	93451734	-0.52	0.13
Mgat5	chr1	129203315	129203466	-1.16	-0.41
Mgat5	chr1	129376047	129379549	-1.02	-0.16

Yod1	chr1	132613903	132614024	-0.24	0.29
Yod1	chr1	132614351	132616572	-2.82	0.26
Rab7b	chr1	133585271	133593615	-0.56	-0.20
Rab7b	chr1	133602280	133610067	-2.45	-0.25
Rbbp5	chr1	134394590	134400982	-3.46	0.33
Rbbp5	chr1	134401131	134401282	-0.55	-0.11
Ube2t	chr1	136859153	136864304	-2.08	-0.99
Edem3	chr1	153658769	153666557	-0.36	0.01
Pigc	chr1	163899348	163901752	-0.98	-0.09
F5	chr1	166122292	166122503	-0.98	2.87
F5	chr1	166122922	166123223	-2.19	4.15
F5	chr1	166124151	166124452	-2.66	4.37
F5	chr1	166124661	166124722	-0.14	-0.15
Adamts4	chr1	173189066	173189276	-0.94	-2.16
B4galt3	chr1	173206311	173206788	-3.02	-0.30
Dcaf8	chr1	174078175	174078505	-1.42	-0.39
Dcaf8	chr1	174079403	174103118	-3.22	-0.21
Dcaf8	chr1	174124786	174125087	-1.07	0.21
Pigm	chr1	174306661	174308397	-1.98	0.12
Parp1	chr1	182499105	182503813	-3.02	-0.53
Cops5	chr1	10027829	10027979	-0.46	-0.04
Uggt1	chr1	36200615	36203523	-1.07	0.08
Uggt1	chr1	36300966	36301147	-2.77	-0.51
Lman2l	chr1	36480210	36481290	-2.06	-0.23
Chst10	chr1	38920717	38922965	-1.47	0.09
Satb2	chr1	56852645	56854020	-1.68	0.15
Satb2	chr1	56928445	56928625	-2.86	-0.44
Satb2	chr1	57026707	57028178	-2.44	-0.49
Sumo1	chr1	59696425	59696959	-2.60	0.18
Bard1	chr1	71121361	71122020	-3.24	-0.57
Usp37	chr1	74485767	74485918	-0.75	0.27
Tuba4a	chr1	75211607	75211936	-0.33	0.05
Tuba4a	chr1	75212113	75215828	-2.13	-0.09
Cul3	chr1	80336855	80337005	-0.41	-0.05
Hdac4	chr1	93829331	93829930	-2.32	-0.46
Pign	chr1	107560192	107560253	-0.60	0.26
Ikbke	chr1	133151178	133153534	-2.21	-0.20
Mdm4	chr1	134888236	134893148	-1.31	0.10
Rnf2	chr1	153318408	153318469	-0.48	0.17
Lamc1	chr1	155066798	155066859	-0.32	0.03
Lamc1	chr1	155179317	155179826	-2.31	-0.22
Qsox1	chr1	157626448	157628411	-2.42	-0.35
Klhl20	chr1	163019047	163019258	-2.02	-0.55

Klhl20	chr1	163020426	163023835	-3.23	0.59
Klhl20	chr1	163039327	163039897	-0.46	0.06
Dcaf6	chr1	167281092	167281331	-0.55	0.15
Uap1	chr1	172072432	172072642	-2.20	0.58
Dtl	chr1	193363068	193363969	-3.46	1.70
Dtl	chr1	193364536	193364986	-1.04	-0.31
Stam	chr2	14067864	14068433	-2.00	0.29
Commd3	chr2	18596461	18597638	-0.32	-0.01
Bmi1	chr2	18605637	18606468	-2.42	0.17
Fbxw5	chr2	25358984	25359776	-1.40	-0.18
Fbxw5	chr2	25360013	25360422	-1.11	-0.22
Dolpp1	chr2	30252653	30255005	-0.66	-0.14
Usp20	chr2	30865689	30865870	-0.98	-0.37
Nup214	chr2	31888785	31893623	-1.12	-0.08
Nup214	chr2	31907789	31907850	-0.40	-0.11
Pomt1	chr2	32109957	32110226	-2.95	-0.65
Golga2	chr2	32158692	32159742	-1.00	-0.15
Golga2	chr2	32161974	32162215	-0.31	-0.01
Dpm2	chr2	32427908	32429091	-2.78	-0.21
St6galnac4	chr2	32451265	32451356	-0.49	0.18
St6galnac4	chr2	32452492	32453780	-1.27	-0.12
Mbd5	chr2	49101163	49101343	-0.39	0.12
Mbd5	chr2	49111717	49113842	-0.74	-0.09
Mbd5	chr2	49134446	49169227	-0.47	0.17
Klhl41	chr2	69508654	69508893	-1.20	0.67
Dync1i2	chr2	71049792	71050012	-1.03	0.40
Psmc3	chr2	90894439	90895182	-2.45	-0.16
Traf6	chr2	101531587	101537959	-2.12	0.17
Rtf1	chr2	119558206	119559166	-1.97	-0.19
Rtf1	chr2	119560273	119560484	-0.78	-0.27
Usp8	chr2	126563807	126568357	-3.01	-0.21
Prnd	chr2	131735723	131776618	-0.50	0.21
Tbc1d20	chr2	152119607	152127898	-0.30	0.01
Tbc1d20	chr2	152137106	152137759	-0.37	0.03
Asxl1	chr2	153171874	153177942	-2.57	-0.61
Actr5	chr2	158462488	158464947	-0.87	-0.18
Top1	chr2	160471632	160471932	-2.93	-0.34
Top1	chr2	160547273	160547394	-0.59	0.10
Tubb1	chr2	174282844	174283054	-0.17	0.13
Adrm1	chr2	179906292	179906906	-0.32	0.02
Gata3	chr2	9779686	9779776	-0.33	0.26
Gata3	chr2	9779865	9779925	-0.37	0.52
Gata3	chr2	9779984	9780252	-0.85	0.39

Gata3	chr2	9790440	9796131	-0.26	0.02
Tubb4b	chr2	25077855	25080192	-0.38	-0.01
Traf2	chr2	25374427	25375145	-1.67	-0.19
Sec16a	chr2	26277717	26279943	-0.64	0.09
Sec16a	chr2	26294102	26294703	-0.51	0.04
Sec16a	chr2	26295032	26300736	-1.17	0.06
Ntng2	chr2	29063096	29103560	-1.80	-0.66
Dolk	chr2	30139748	30140467	-1.14	0.19
Dolk	chr2	30140616	30141814	-0.56	-0.08
Fbxw2	chr2	34661048	34661869	-2.35	-0.23
Fbxw2	chr2	34662794	34664040	-0.82	-0.21
Fbxw2	chr2	34668436	34678672	-0.41	-0.02
Fbxw2	chr2	34681468	34681725	-0.52	0.16
Psmd5	chr2	34707993	34709823	-0.82	-0.10
Psmd5	chr2	34726274	34726482	-3.28	-0.70
Psmb7	chr2	38498946	38499426	-1.70	-0.24
Galnt3	chr2	65945079	65962610	-1.45	0.54
Slc35c1	chr2	92293995	92294146	-1.70	-0.44
Slc35c1	chr2	92294234	92294832	-0.73	-0.10
Slc35c1	chr2	92298932	92299589	-0.72	-0.08
Rcn1	chr2	105239178	105239446	-0.85	-0.22
Ino80	chr2	119200037	119200278	-1.91	0.39
Trp53bp1	chr2	121030727	121033735	-0.29	0.00
Trp53bp1	chr2	121034169	121046975	-0.79	-0.09
Trp53bp1	chr2	121061343	121062603	-0.86	0.09
Cops2	chr2	125657568	125660555	-1.21	-0.08
Psmf1	chr2	151543679	151544068	-0.32	0.02
Edem2	chr2	155527855	155531394	-0.83	-0.09
Edem2	chr2	155555061	155555181	-0.35	-0.07
Neurl2	chr2	164656229	164658385	-1.59	0.26
B4galt5	chr2	167174439	167174678	-3.17	-0.64
Usp13	chr3	32832209	32832389	-0.70	3.46
Fbxw7	chr3	84619498	84694070	-0.58	0.12
Fbxw7	chr3	84694129	84756226	-2.67	0.26
Fbxw7	chr3	84780140	84781955	-0.67	0.08
Rab13	chr3	90028667	90029856	-1.43	0.42
Otud7b	chr3	95908449	95908540	-1.61	-0.69
Otud7b	chr3	95956546	95960193	-1.03	-0.07
Hist2h2be	chr3	96025043	96025490	-0.87	-0.31
Dph5	chr3	115631581	115632001	-0.77	0.15
Bcl10	chr3	145587262	145587592	-1.35	-0.17
Bcl10	chr3	145588099	145593586	-0.30	0.00
Usp33	chr3	152031258	152033363	-1.23	-0.12

Pigk	chr3	152448420	152449109	-1.33	-0.21
Pigk	chr3	152449825	152450483	-1.30	0.25
Pigk	chr3	152451617	152451828	-0.79	0.29
Pex2	chr3	5561142	5563166	-0.32	0.01
Phc3	chr3	30805917	30813151	-0.46	0.06
Phc3	chr3	30864763	30868337	-1.50	-0.73
Cog6	chr3	52798099	52798220	-0.40	-1.58
Commd2	chr3	57450273	57450603	-0.62	-0.13
Siah2	chr3	58479649	58495411	-0.38	-0.02
Siah2	chr3	58495680	58495891	-0.39	-0.11
Psmb4	chr3	94690551	94690880	-2.31	-0.17
Man1a2	chr3	100371011	100382434	-0.85	0.08
Man1a2	chr3	100460030	100489396	-2.89	0.15
Csf1	chr3	107544324	107544444	-0.74	0.65
Csf1	chr3	107544802	107548597	-0.55	0.11
Csf1	chr3	107548745	107550765	-0.94	-1.24
Csf1	chr3	107551063	107552051	-0.42	0.07
Rwdd3	chr3	120859364	120862089	-1.12	-0.32
Ank2	chr3	126625873	126626114	-0.36	1.15
Ank2	chr3	126626353	126626834	-1.70	-1.10
Sec24b	chr3	129686102	129687101	-1.10	-0.09
Rab2a	chr4	8462790	8462851	-1.78	-0.59
Map3k7	chr4	32051081	32051232	-1.86	-0.83
Map3k7	chr4	32110141	32110231	-1.49	-0.76
Ube2r2	chr4	41137807	41138136	-0.82	-0.10
Dcaf10	chr4	45385889	45387268	-0.55	-0.08
Tgfbr1	chr4	47419877	47423747	-3.57	0.68
Rad23b	chr4	55398385	55402936	-1.44	0.10
Rraga	chr4	86221576	86222293	-1.17	0.09
Usp24	chr4	105988817	105989117	-1.39	-0.42
Usp24	chr4	106111387	106111837	-3.22	-0.26
Lrrc41	chr4	115768961	115769201	-3.42	-0.29
Scmh1	chr4	120201905	120201966	-0.42	-0.19
Trappc3	chr4	125939648	125949968	-0.91	-0.16
Trappc3	chr4	125951220	125952918	-1.33	0.11
Clspn	chr4	126237254	126239019	-2.37	-0.64
Clspn	chr4	126243253	126249549	-1.57	-0.49
Clspn	chr4	126270339	126270758	-1.05	-0.48
Phc2	chr4	128404881	128405061	-0.49	0.29
Fuca1	chr4	135495027	135495325	-1.89	0.27
Usp48	chr4	137212056	137213524	-1.00	0.10
Mul1	chr4	137996388	137996837	-0.57	-0.15
Capzb	chr4	138748845	138794826	-1.20	-0.15

Klhl21	chr4	151383838	151389959	-1.59	-0.17
Klhl21	chr4	151390257	151390408	-2.24	0.94
Icmt	chr4	151674797	151677845	-1.15	-0.14
Manea	chr4	26254667	26256281	-2.07	0.28
Ddx58	chr4	40152603	40155517	-1.09	-0.14
Ddx58	chr4	40186678	40186858	-0.63	0.23
B4galt1	chr4	40753786	40754830	-2.74	0.20
B4galt1	chr4	40770597	40800583	-1.25	0.19
B4galt1	chr4	40800821	40800971	-2.38	-0.70
Vcp	chr4	43013259	43013349	-0.47	-0.15
Gne	chr4	44047905	44048266	-3.23	-0.97
Gne	chr4	44068129	44079645	-1.35	-0.23
Fbxo10	chr4	45048105	45048374	-0.54	-0.64
Fbxo10	chr4	45048672	45048852	-0.34	0.51
Fbxo10	chr4	45071353	45071981	-0.56	-0.37
Alg2	chr4	47482973	47483543	-0.65	0.08
Mysm1	chr4	94631863	94632432	-1.52	0.19
B4galt2	chr4	117546388	117546538	-2.37	2.64
Dph2	chr4	117562463	117563268	-3.28	-0.40
Dph2	chr4	117564387	117564628	-0.53	-0.14
St3gal3	chr4	117604757	117612678	-1.50	-0.18
Pigv	chr4	133218200	133220631	-0.38	-0.05
Dhdds	chr4	133526767	133536084	-3.00	-0.27
Man1c1	chr4	134119221	134119671	-1.30	0.19
Otud3	chr4	138451293	138453499	-1.99	-0.27
Fbxo6	chr4	147520122	147520183	-0.29	0.01
Pex14	chr4	148358178	148473921	-0.30	0.00
Spsb1	chr4	149271976	149281277	-0.37	-0.15
Nupl2	chr5	23688056	23689616	-0.80	0.11
Nub1	chr5	24191751	24195229	-0.32	-0.04
Nub1	chr5	24216073	24216194	-1.10	-0.40
Galnt11	chr5	24771142	24771321	-1.45	-0.43
Bst1	chr5	44210161	44210281	-0.88	-0.46
Klhl5	chr5	65558241	65558691	-1.07	-0.19
Dcun1d4	chr5	73950683	73951373	-0.84	0.26
Uso1	chr5	92630205	92631402	-0.84	-0.06
Pigg	chr5	108776959	108777289	-0.56	-0.20
Usp30	chr5	114572332	114572933	-2.34	-0.40
Wsb2	chr5	117827434	117828091	-2.97	-0.26
Fbxo21	chr5	118452093	118458648	-2.38	-0.37
Psmd9	chr5	123678228	123678437	-1.01	0.20
Sumf2	chr5	130338841	130338932	-0.89	-1.00
Kctd7	chr5	130628217	130628755	-1.29	-0.61

Vgf	chr5	137506871	137509013	-0.62	-0.14
Cops6	chr5	138605129	138605308	-1.66	0.48
Fam20c	chr5	139284018	139286017	-3.49	-0.20
Foxk1	chr5	142931509	142934770	-1.58	0.09
Kdelr2	chr5	144165498	144165618	-0.81	-0.20
Preb	chr5	31254099	31254430	-3.13	0.83
Preb	chr5	31254579	31254819	-1.65	0.61
Preb	chr5	31255148	31255718	-1.53	0.29
Preb	chr5	31256197	31256528	-1.13	0.29
Preb	chr5	31257126	31257247	-0.79	-0.36
Preb	chr5	31257366	31258308	-0.98	-0.07
Tnip2	chr5	34839311	34839491	-0.39	0.07
Tnip2	chr5	34842145	34842355	-1.03	0.23
Tada2b	chr5	36818258	36826725	-1.77	0.18
Wfs1	chr5	37358240	37358331	-1.75	-1.37
Wfs1	chr5	37358929	37359708	-1.86	-0.46
Fbxl5	chr5	44142150	44149098	-0.30	0.01
Fbxl5	chr5	44149396	44151063	-1.01	0.09
Nup54	chr5	92845101	92846543	-0.74	0.09
Sec31a	chr5	100790907	100791058	-1.68	-0.19
Mlec	chr5	115597834	115600228	-0.81	-0.07
Suds3	chr5	117542584	117542943	-0.42	-0.03
Suds3	chr5	117558427	117565972	-3.36	-0.34
Fbxw8	chr5	118515497	118515767	-0.43	-0.12
Ubc	chr5	125867967	125870328	-2.39	0.10
Fbxl18	chr5	143640471	143644134	-2.38	-0.99
Usp42	chr5	144471642	144472211	-0.41	-0.06
Usp42	chr5	144477517	144477978	-2.04	-0.45
Usp12	chr5	147548927	147550725	-0.54	0.07
Calu	chr6	29298148	29306471	-0.77	-0.32
Calu	chr6	29322584	29324851	-1.99	0.11
Nup205	chr6	35127615	35133895	-0.46	0.13
Rab19	chr6	39339614	39340231	-1.34	-0.45
Cul1	chr6	47474617	47476108	-2.74	-0.21
Tnip3	chr6	65564764	65582063	-2.52	0.23
St3gal5	chr6	72092201	72097116	-0.54	-0.05
St3gal5	chr6	72099116	72104206	-1.29	0.11
Dctn1	chr6	83140226	83141007	-1.23	-0.14
Mitf	chr6	97960393	97968956	-2.29	-0.13
Mitf	chr6	97969614	97970094	-1.49	-0.18
Edem1	chr6	108805488	108805698	-0.64	-0.04
Ttll3	chr6	113357235	113359288	-2.43	0.39
Ttll3	chr6	113362895	113364057	-1.63	-0.42

Vhl	chr6	113578147	113579978	-1.22	0.20
Fbxl14	chr6	119429685	119431421	-0.82	0.06
Rad52	chr6	119865354	119869071	-0.36	0.02
Rad52	chr6	119871318	119872841	-0.33	-0.02
Usp18	chr6	121195923	121196013	-1.05	-1.68
Cmas	chr6	142705235	142705504	-3.50	-0.70
Klhl42	chr6	147039984	147056940	-0.79	-0.10
Etfbkmt	chr6	149092586	149092885	-3.29	-1.16
Etfbkmt	chr6	149095803	149099215	-3.09	-1.29
Ube2h	chr6	30163827	30164395	-0.70	-0.09
Ube2h	chr6	30164783	30164963	-0.64	0.31
Hipk2	chr6	38697991	38787107	-0.63	0.02
Hipk2	chr6	38825829	38826040	-1.39	-0.22
Nod1	chr6	54893317	54893527	-0.62	1.79
Nod1	chr6	54894659	54894899	-0.40	-0.75
Rnf181	chr6	72310209	72310535	-1.67	-0.25
Tgoln1	chr6	72566903	72566994	-0.12	0.00
Rab43	chr6	87761473	87761773	-0.67	-0.19
Nup210	chr6	90963060	90963630	-0.69	-0.12
Xpc	chr6	91439837	91440135	-1.44	-0.58
Sumf1	chr6	108058060	108058479	-1.21	0.20
Sumf1	chr6	108135367	108135577	-1.99	-0.39
Rad18	chr6	112577091	112577691	-0.33	0.02
Tada3	chr6	113316922	113317131	-1.20	0.33
Tada3	chr6	113322460	113324488	-0.48	0.11
Tada3	chr6	113324725	113325142	-2.83	0.54
Phc1	chr6	122275560	122275800	-2.40	-0.84
Usp5	chr6	124776424	124779465	-0.32	0.01
Ccdc8	chr7	17580174	17580295	-0.16	0.42
B3gnt8	chr7	26415160	26415521	-1.98	-0.29
Josd2	chr7	51723425	51723545	-0.38	0.08
Nup62	chr7	52071739	52086176	-1.81	-0.05
Hrc	chr7	52590728	52591119	-0.83	-0.95
Hrc	chr7	52591508	52591689	-0.33	0.42
Prmt3	chr7	57112858	57113008	-0.98	-0.21
Klhl25	chr7	82993313	83012167	-0.53	-0.05
Klhl25	chr7	83018746	83018926	-0.98	-0.56
Ctsc	chr7	95437274	95437455	-0.26	0.00
Ctsc	chr7	95458037	95458487	-2.08	0.25
Rab30	chr7	99984220	99984700	-0.22	-0.11
Alg8	chr7	104540130	104540578	-0.40	0.05
Arrb1	chr7	106748328	106749813	-0.38	-0.04
Arrb1	chr7	106752471	106752711	-0.95	-0.19

Ctr9	chr7	118172674	118177414	-0.59	-0.08
Ctr9	chr7	118198901	118199651	-0.33	0.01
Usp47	chr7	119167079	119167170	-0.67	0.25
Usp47	chr7	119234949	119245400	-0.57	0.03
Usp47	chr7	119252192	119254032	-1.45	0.15
Spon1	chr7	121183532	121183832	-1.33	-0.98
Ccp110	chr7	125865000	125865449	-3.00	-0.57
Ccp110	chr7	125865897	125867971	-0.41	0.05
Dctn5	chr7	129276554	129277689	-0.62	0.13
Dctn5	chr7	129291850	129292499	-2.82	0.17
Fbxl19	chr7	134911014	134911817	-1.61	-0.24
Fbxl19	chr7	134911935	134912442	-2.06	-0.46
Abraxas2	chr7	140072975	140075508	-1.01	0.09
Zranb1	chr7	140122884	140158338	-0.66	0.06
Zranb1	chr7	140174515	140175634	-1.19	-0.23
Zranb1	chr7	140175516	140175874	-0.37	-0.03
Pnpla2	chr7	148641086	148641236	-0.41	0.07
Pnpla2	chr7	148645626	148646045	-0.65	-0.08
Tfpt	chr7	3581426	3581486	-1.18	-0.65
Ube2m	chr7	13623108	13623619	-2.32	-0.37
Sae1	chr7	16912865	16913105	-0.52	-0.05
Calm3	chr7	17502039	17502309	-2.51	-0.14
Apoe	chr7	20282157	20282891	-2.78	-0.52
Ceacam1	chr7	26247769	26248608	-3.38	0.29
Ceacam1	chr7	26256984	26258859	-1.12	-0.38
Ceacam1	chr7	26259008	26274241	-1.35	0.18
Sptbn4	chr7	28189491	28189582	-0.15	0.17
Nucb1	chr7	52758551	52762906	-2.24	-0.25
Spaca4	chr7	52980801	52981068	-0.84	0.89
Spaca4	chr7	52981126	52981186	-0.15	-0.15
Nsmce3	chr7	72016624	72017896	-1.52	-0.13
Asb7	chr7	73792504	73823980	-1.38	-0.19
Man2a2	chr7	87496117	87497882	-1.79	-0.10
Blm	chr7	87647292	87647681	-0.45	0.09
Blm	chr7	87654437	87658340	-2.18	0.36
Neu3	chr7	106961618	106962752	-2.05	0.46
Nup98	chr7	109268003	109269174	-0.67	-0.03
Usp17ld	chr7	110398629	110398808	-0.25	-0.42
Dcun1d3	chr7	127000917	127038861	-3.18	-0.26
Cog7	chr7	129066679	129066829	-3.22	-0.98
Cog7	chr7	129121227	129125207	-0.83	-0.17
Nsmce4a	chr7	137690685	137690865	-1.34	-0.83
Trappc5	chr8	3676506	3679785	-2.29	-0.19

Trappc5	chr8	3680083	3680622	-2.14	-0.39
F7	chr8	13034819	13035449	-1.19	-0.75
Cul4a	chr8	13144401	13146773	-2.17	0.30
Cul4a	chr8	13146892	13147072	-1.09	-0.25
Thsd1	chr8	23353890	23354367	-0.39	1.22
Thsd1	chr8	23368989	23369377	-0.36	-1.64
Ank1	chr8	24242672	24243002	-0.59	-0.63
Cope	chr8	72826683	72826743	-0.97	-0.26
Fkbp8	chr8	73051641	73053542	-2.28	-0.16
Babam1	chr8	73920753	73921677	-0.98	-0.13
Dda1	chr8	73998687	73999436	-1.57	-0.10
Dda1	chr8	73999764	73999855	-0.77	0.18
Rab8a	chr8	74704051	74704703	-1.19	-0.09
Nr3c2	chr8	79433507	79433776	-0.32	-0.42
Cyld	chr8	91220956	91221047	-0.33	-0.20
Fbxl8	chr8	107791952	107793137	-2.37	-0.45
Cog4	chr8	113405654	113406134	-0.74	-0.12
St3gal2	chr8	113444003	113491381	-0.85	-0.10
St3gal2	chr8	113493520	113493610	-0.45	0.29
St3gal2	chr8	113493699	113494575	-1.50	-0.33
St3gal2	chr8	113494724	113495173	-3.09	-0.60
Usp10	chr8	122480587	122481247	-1.81	-0.16
Trappc2l	chr8	125135525	125136985	-0.52	-0.21
Tubb3	chr8	125944632	125944960	-0.21	-0.02
Galnt2	chr8	126864828	126868362	-1.82	0.19
Dcun1d2	chr8	13256291	13257907	-0.80	-0.08
Gas6	chr8	13465641	13465821	-3.11	-0.71
Vdac3	chr8	23687783	23688050	-2.48	0.31
Pomk	chr8	27092830	27098060	-0.32	0.01
Dctn6	chr8	35169406	35171536	-0.90	0.26
Ing2	chr8	48753850	48754661	-0.78	0.10
Ing2	chr8	48760302	48760513	-2.46	-1.21
Klhl2	chr8	67219421	67219870	-1.56	-0.30
Ano8	chr8	74000275	74000456	-0.96	-0.91
Ano8	chr8	74000634	74002643	-0.60	0.70
B3gnt3	chr8	74217467	74227314	-0.27	0.00
Rad23a	chr8	87358550	87358761	-2.05	-0.60
Amfr	chr8	96496474	96497102	-1.31	-0.11
Dync1li2	chr8	106966676	106966737	-0.30	-0.05
Cog8	chr8	109574194	109580248	-1.23	0.11
Mbtps1	chr8	122032081	122032770	-0.70	0.06
Mbtps1	chr8	122070114	122082661	-1.48	0.13
Fbxo31	chr8	124075878	124076177	-0.56	0.18

Mvd	chr8	124957554	124958775	-0.56	-0.04
Pigyl	chr9	21961289	21962741	-2.33	-0.36
Opcml	chr9	28210755	28211235	-0.28	-0.82
Opcml	chr9	28211624	28211685	-0.29	-1.22
Opcml	chr9	28729664	28729844	-0.95	-1.68
Opcml	chr9	28730203	28730384	-0.20	0.00
Opcml	chr9	28730533	28730714	-0.53	0.75
Nfrkb	chr9	31218903	31226648	-2.78	0.27
Usp2	chr9	43902061	43902302	-1.62	-0.76
Dpagt1	chr9	44141235	44141504	-0.58	0.09
Apoa1	chr9	46037922	46038460	-0.32	0.95
Usp28	chr9	48849662	48849753	-0.68	0.33
Alg9	chr9	50651063	50651633	-1.34	-0.24
Fbxo22	chr9	55066270	55071642	-1.39	0.12
Fbxo22	chr9	55071791	55072031	-0.79	0.18
Sin3a	chr9	56958532	56958892	-0.66	0.11
Sin3a	chr9	56973115	56975606	-1.32	0.16
Rora	chr9	69226745	69226835	-0.38	-0.37
Adam10	chr9	70526807	70526958	-0.76	0.19
Adam10	chr9	70626086	70626267	-1.49	0.28
Rab27a	chr9	72892731	72908786	-0.70	-1.27
Rab27a	chr9	72943119	72943840	-0.62	-0.22
Tmem115	chr9	107436394	107440987	-1.57	0.09
Usp4	chr9	108293381	108294591	-0.82	0.07
Usp19	chr9	108393096	108395032	-3.28	-0.27
Cdc25a	chr9	109778082	109778888	-0.80	-0.16
Cdc25a	chr9	109794596	109795525	-1.21	0.17
Glb1	chr9	114310242	114326084	-1.04	0.28
Glb1	chr9	114368770	114373539	-0.71	0.08
Dync1li1	chr9	114632686	114632895	-0.48	0.07
Wdr48	chr9	119833344	119835040	-3.23	0.21
Birc2	chr9	7818494	7818943	-1.37	0.33
Birc2	chr9	7834955	7835225	-0.88	0.29
Birc3	chr9	7848939	7850928	-0.65	0.09
Birc3	chr9	7860281	7872961	-1.11	0.10
Fbxl12	chr9	20448880	20449151	-1.08	-0.58
Dnmt1	chr9	20733552	20734608	-0.96	0.27
Keap1	chr9	21034503	21042276	-2.14	0.12
Keap1	chr9	21043235	21043776	-0.68	-0.28
Aplp2	chr9	30957469	30957679	-1.95	-0.19
Aplp2	chr9	30985646	31019400	-1.25	0.28
Arcn1	chr9	44551783	44551994	-2.37	0.20
Arcn1	chr9	44568207	44575891	-1.28	0.17

Rab39	chr9	53494560	53494740	-0.32	0.63
Pml	chr9	58067502	58068341	-3.10	-0.28
Pml	chr9	58080996	58094952	-1.77	-0.21
Pml	chr9	58097382	58097593	-1.39	-0.43
Senp8	chr9	59584151	59585492	-1.58	0.27
Pias1	chr9	62729583	62729943	-1.17	0.27
Pias1	chr9	62799649	62828686	-1.72	0.23
Smad3	chr9	63496369	63496909	-0.75	0.36
Smad3	chr9	63497357	63498046	-0.81	-0.34
Smad3	chr9	63605729	63605891	-0.28	-0.36
Rab11a	chr9	64585502	64585563	-1.25	0.56
Usp3	chr9	66366133	66366372	-0.54	-0.11
Usp3	chr9	66441201	66441261	-0.27	-0.29
Rab8b	chr9	66715068	66767482	-0.50	0.07
Pigb	chr9	72863505	72865261	-2.04	0.25
Fbxo9	chr9	77943550	77943611	-0.20	0.01
Pgm3	chr9	86447697	86448057	-3.55	-0.68
Gorasp1	chr9	119834790	119835029	-0.52	0.12
Sec22c	chr9	121593506	121593866	-1.05	-0.53
Shprh	chr10	10869257	10871973	-1.04	-0.18
Shprh	chr10	10882742	10884564	-2.81	-0.36
Fuca2	chr10	13222977	13225667	-1.46	0.28
Nus1	chr10	52153241	52156613	-2.27	-0.23
Nus1	chr10	52158200	52158410	-1.09	-0.22
Ranbp2	chr10	57941534	57941955	-2.61	0.14
Sumo3	chr10	77068978	77069482	-0.67	-0.17
Ube2g2	chr10	77107280	77107637	-1.28	-0.14
Ube2g2	chr10	77108022	77108082	-0.37	-0.06
Eef2	chr10	80644273	80645254	-1.14	-0.02
Eid3	chr10	82329548	82330082	-0.66	-0.10
Galnt4	chr10	98571396	98572084	-0.88	0.20
Galnt4	chr10	98572412	98573220	-1.11	0.24
Tnfaip3	chr10	18721581	18725108	-0.69	-0.06
Rnf146	chr10	29064221	29064521	-3.45	0.40
Rnf146	chr10	29066497	29067546	-0.61	0.05
Man1a	chr10	53795361	53795512	-0.40	-0.49
Ube2d1	chr10	70718174	70718562	-1.17	0.19
Trappc10	chr10	77650129	77651570	-0.54	-0.05
Pias4	chr10	80616715	80617300	-1.40	-0.19
Ckap4	chr10	83990001	83991399	-0.32	0.00
Ckap4	chr10	83996275	83996633	-1.47	-0.37
Rab21	chr10	114727694	114731953	-1.61	0.13
Os9	chr10	126531494	126532125	-0.76	-0.17

Os9	chr10	126535916	126536493	-1.03	-0.18
Os9	chr10	126557594	126558216	-1.54	-0.23
Mbd6	chr10	126719101	126719461	-1.32	0.15
Mbd6	chr10	126719550	126720682	-2.45	-0.19
Ykt6	chr11	5855760	5856000	-1.50	-0.31
Ykt6	chr11	5866082	5866381	-2.00	0.22
Usp34	chr11	23206894	23233439	-0.33	0.02
Usp34	chr11	23233527	23233678	-2.09	0.31
Usp34	chr11	23263863	23264511	-0.40	0.01
Usp34	chr11	23388308	23389213	-1.84	0.16
Psme4	chr11	30671804	30671925	-0.83	-0.91
Psme4	chr11	30672014	30672135	-0.38	0.12
Fbxw11	chr11	32644536	32645016	-2.21	0.20
Mgat4b	chr11	50039046	50039137	-1.58	-0.54
Galnt10	chr11	57598076	57598614	-0.49	-0.14
Nlrp3	chr11	59379889	59380129	-0.80	-0.15
Pigl	chr11	62271961	62272052	-2.04	-1.11
Trp53	chr11	69393860	69394608	-0.68	0.14
Asgr1	chr11	69870833	69871366	-0.46	1.72
Ube2g1	chr11	72493612	72497325	-2.70	0.54
Rtn4rl1	chr11	75079893	75079983	-0.50	3.17
Pigs	chr11	78141953	78142237	-1.31	-0.70
Pigs	chr11	78155437	78156068	-0.60	-0.05
Suz12	chr11	79843530	79845978	-2.27	0.14
Psmd11	chr11	80285544	80285635	-1.72	-0.32
Ptrh2	chr11	86497514	86503722	-0.63	0.05
Thra	chr11	98603186	98617447	-0.55	0.14
Thra	chr11	98625630	98626259	-2.85	-0.46
Rara	chr11	98799009	98808938	-1.27	-0.44
Psme3	chr11	101177564	101178336	-0.63	-0.07
Psme3	chr11	101182070	101183288	-1.70	-0.15
Dcaf7	chr11	105898185	105898485	-0.69	-0.09
Dcaf7	chr11	105916210	105916330	-0.89	-0.17
Dcaf7	chr11	105919829	105920099	-0.59	-0.07
Arsg	chr11	109346860	109378636	-0.37	-0.02
Arsg	chr11	109433779	109434107	-1.59	0.43
Birc5	chr11	117716547	117716877	-3.30	-0.42
Engase	chr11	118348531	118349162	-0.37	-0.08
Cbx2	chr11	118885531	118887831	-0.67	1.03
Gps1	chr11	120648708	120649402	-0.62	0.20
Gps1	chr11	120649969	120650416	-1.12	-0.20
Foxk2	chr11	121161028	121169022	-2.30	0.14
Fn3k	chr11	121303142	121303232	-0.53	-3.32

Fn3k	chr11	121303589	121303799	-0.67	-3.37
B3gnt2	chr11	22735900	22759719	-0.66	0.02
Commd1	chr11	22799727	22800056	-1.19	0.13
Commd1	chr11	22856647	22878367	-0.86	0.20
Commd1	chr11	22878962	22882284	-0.58	-0.15
Pex13	chr11	23548927	23549226	-0.93	0.25
Pex13	chr11	23551042	23555901	-0.65	-0.10
Sptbn1	chr11	30017714	30018324	-2.28	0.32
Sptbn1	chr11	30036124	30038367	-0.64	0.05
Sptbn1	chr11	30038816	30040543	-1.03	-0.10
Sptbn1	chr11	30119730	30168025	-0.45	0.12
Stc2	chr11	31260009	31260460	-0.39	-0.10
0610009B22Rik	chr11	51499121	51499534	-0.41	-0.04
Sec24a	chr11	51569959	51570170	-2.57	-1.70
Tnip1	chr11	54769581	54776442	-0.41	0.13
Nup88	chr11	70783414	70783475	-0.56	0.19
Derl2	chr11	70823390	70826702	-1.07	-0.09
Derl2	chr11	70832716	70832777	-0.54	0.26
Hic1	chr11	74979054	74979175	-0.19	-0.46
Hic1	chr11	74979353	74979534	-0.28	-0.54
Hic1	chr11	74980731	74980792	-0.14	-0.42
Rpa1	chr11	75154547	75161885	-2.86	-0.51
Gosr1	chr11	76543085	76548136	-1.36	0.15
Wsb1	chr11	79053599	79053898	-1.62	-0.11
Pex12	chr11	83109905	83111913	-1.20	0.24
Pigw	chr11	84691211	84691984	-0.64	-0.09
Ube2z	chr11	95911494	95911704	-3.10	-0.34
Fbxl20	chr11	97950510	97953462	-0.36	0.03
Klhl11	chr11	100333798	100334096	-1.09	0.31
Rab5c	chr11	100581475	100599444	-1.38	-0.37
Becn1	chr11	101163065	101163581	-2.50	-0.31
Brca1	chr11	101384814	101385204	-1.75	-0.62
Brca1	chr11	101385473	101386581	-0.92	-0.20
Brca1	chr11	101386909	101387659	-3.36	0.64
Gosr2	chr11	103540134	103540702	-0.78	-0.07
Nsf	chr11	103684382	103685071	-0.57	-0.08
Nsf	chr11	103792113	103815310	-0.41	0.18
Smurf2	chr11	106683743	106684358	-0.92	-0.20
Cbx8	chr11	118899867	118901442	-1.43	0.25
Cbx4	chr11	118940531	118940592	-1.14	1.49
Cbx4	chr11	118941070	118941400	-3.20	1.23
Cbx4	chr11	118941759	118947461	-0.50	0.02
Csnk1d	chr11	120819778	120820048	-1.21	-0.15

Csnk1d	chr11	120823779	120824080	-1.10	-0.14
Csnk1d	chr11	120852347	120852438	-1.28	-0.33
B3gntl1	chr11	121478331	121479112	-0.57	-0.07
Asxl2	chr12	3426883	3442534	-0.98	-0.12
Asxl2	chr12	3496396	3496996	-0.33	0.01
Smc6	chr12	11292342	11292522	-1.73	0.67
Smc6	chr12	11324583	11324974	-0.83	0.12
Psma6	chr12	56499811	56499871	-1.67	-0.32
Lrr1	chr12	70275442	70276002	-0.27	0.05
Fut8	chr12	78432957	78466016	-1.24	0.35
Fut8	chr12	78576193	78576884	-0.99	0.24
Ttll5	chr12	87165622	87165743	-0.32	-0.11
Psmc1	chr12	101348377	101351286	-1.53	-0.29
Calm1	chr12	101445209	101445479	-1.73	0.08
Otub2	chr12	104643783	104643844	-0.48	0.32
Dync1h1	chr12	111839634	111839815	-0.77	-0.17
Dync1h1	chr12	111855964	111857506	-0.91	0.03
Dync1h1	chr12	111904592	111905154	-0.63	0.04
Wdr20	chr12	112017684	112032696	-0.32	-0.01
Traf3	chr12	112499736	112500694	-0.44	0.03
Btbd6	chr12	114215550	114217061	-3.22	-0.34
Mta1	chr12	114374970	114375239	-3.26	-0.32
Ncoa1	chr12	4247630	4248379	-1.43	-0.26
Ncoa1	chr12	4248916	4249007	-0.66	-0.36
Ncoa1	chr12	4249155	4252804	-0.79	-0.16
Ncoa1	chr12	4267739	4267919	-0.46	-0.11
Ncoa1	chr12	4294980	4296087	-2.92	0.28
Trappc6b	chr12	60162329	60162448	-2.54	-0.76
Sptb	chr12	77714030	77714151	-0.25	-1.14
Rab15	chr12	77899366	77899605	-0.77	2.23
Rab15	chr12	77900616	77900676	-0.21	-1.00
Pigh	chr12	80183182	80183482	-0.71	-0.23
Dcaf5	chr12	81437908	81438268	-1.65	-0.32
Dcaf5	chr12	81439342	81449328	-0.57	0.04
Pomt2	chr12	88449791	88451254	-0.31	-0.01
Sel1l	chr12	93080291	93087597	-3.35	-0.59
Atxn3	chr12	103157140	103157589	-0.55	0.09
Ankrd9	chr12	112214580	112215269	-1.06	-0.47
Trim27	chr13	21283975	21284426	-1.81	0.13
Hist1h2bg	chr13	23663268	23663474	-0.87	-0.07
Hist1h2bc	chr13	23776067	23776213	-1.76	0.26
Hist1h2ab	chr13	23842956	23843224	-1.74	-0.27
Ripk1	chr13	34119794	34126112	-1.97	0.13

Ercc8	chr13	108948931	108950875	-0.50	-0.19
Gmds	chr13	31911574	31911785	-0.51	-0.13
Tubb2b	chr13	34219290	34219557	-1.15	1.09
Tubb2b	chr13	34219999	34221135	-0.49	0.22
Nup153	chr13	46776228	46776529	-1.65	0.22
Uimc1	chr13	55129866	55132327	-1.14	0.25
Uimc1	chr13	55176900	55177348	-2.31	-0.33
Rab24	chr13	55421293	55421501	-1.79	-0.26
Rab24	chr13	55422955	55423341	-2.38	-0.32
Lman2	chr13	55447467	55447708	-0.64	-0.09
Lman2	chr13	55448036	55448306	-2.19	-0.17
Hnrnpk	chr13	58501349	58504674	-0.47	-0.03
Golm1	chr13	59738440	59741498	-0.33	0.02
Kctd6	chr14	9052713	9055546	-1.19	-0.22
Atxn7	chr14	14932503	14936225	-1.34	-0.13
Atxn7	chr14	14936403	14936644	-1.24	-0.33
Actr8	chr14	30804041	30804784	-2.92	-0.88
Bap1	chr14	32071949	32072607	-1.80	-0.20
Psmc6	chr14	45949498	45950514	-0.68	0.11
Ktn1	chr14	48283451	48283932	-0.75	0.08
Dcaf11	chr14	56178865	56179786	-0.52	0.29
Dcaf11	chr14	56188485	56188902	-0.36	0.03
Kbtbd7	chr14	79827126	79828716	-0.67	0.13
Ankrd28	chr14	32514699	32518527	-0.54	-0.04
Dph3	chr14	32893956	32894255	-0.43	0.13
Dph3	chr14	32896016	32898124	-3.51	0.32
Rabggta	chr14	56340688	56340807	-0.30	0.04
Cog3	chr14	76103115	76106953	-1.26	-0.15
Commd6	chr14	102033281	102039330	-3.04	0.28
Fbxl3	chr14	103482030	103488661	-0.97	0.07
Fbxl3	chr14	103491640	103498726	-2.05	0.22
Sema5a	chr15	32616573	32620160	-0.80	-0.13
Dcaf13	chr15	38944419	38944568	-0.30	-0.01
Rnf139	chr15	58729987	58731846	-2.60	0.15
Commd5	chr15	76730370	76731727	-1.87	-0.20
Apol9b	chr15	77566250	77566664	-0.63	0.63
Tab1	chr15	79989200	79992132	-3.29	-0.26
Ep300	chr15	81416643	81438767	-1.28	-0.07
Ep300	chr15	81479273	81481819	-1.93	-0.07
L3mbtl2	chr15	81516741	81518556	-1.99	-0.18
Nup50	chr15	84757805	84765852	-2.65	-0.15
Nup50	chr15	84770131	84771000	-1.89	-0.14
Skp2	chr15	9043329	9043510	-0.80	-0.31

Skp2	chr15	9069207	9070137	-0.35	-0.03
Otulin	chr15	27535675	27538550	-1.58	-0.11
Otulin	chr15	27553110	27560448	-2.84	-0.65
6-Mar	chr15	31388351	31388771	-0.48	-0.05
Rad21	chr15	51794834	51794984	-1.95	0.28
Rad21	chr15	51798216	51799422	-1.38	0.19
Derl1	chr15	57701565	57701686	-0.33	0.03
Derl1	chr15	57702583	57703393	-2.02	-0.09
Fbxo32	chr15	58011932	58012112	-0.37	-0.51
Fbxo32	chr15	58012410	58013069	-1.76	-1.01
Fbxo32	chr15	58046282	58046433	-1.44	-1.83
St3gal1	chr15	66944067	67005237	-2.70	-0.20
Apol9a	chr15	77234337	77235198	-0.69	-0.40
Apol10b	chr15	77415950	77416129	-0.15	-0.07
Ddx17	chr15	79376930	79377021	-2.48	-0.43
Josd1	chr15	79506286	79506525	-1.88	-0.45
Josd1	chr15	79517170	79517588	-0.55	-0.09
Josd1	chr15	79518003	79518153	-1.50	-0.62
Rangap1	chr15	81535546	81535636	-0.36	0.04
Rangap1	chr15	81556518	81556579	-0.84	0.35
Rangap1	chr15	81560168	81560349	-0.56	-0.15
Pmm1	chr15	81781535	81781891	-0.64	-0.13
Ttll12	chr15	83406959	83407199	-2.44	-0.49
Alg12	chr15	88635911	88636421	-1.79	-0.29
Arsa	chr15	89303025	89303862	-1.00	-0.17
Arsa	chr15	89305988	89306615	-2.33	-0.41
Vdr	chr15	97687183	97687273	-0.18	0.38
Senp1	chr15	97872854	97873333	-1.81	-0.22
Asb8	chr15	97965969	97971774	-0.40	-0.02
Galnt6	chr15	100522228	100522617	-0.68	0.17
Galnt6	chr15	100544605	100550892	-0.66	0.07
Alg1	chr16	5244821	5245001	-2.03	-0.46
Senp2	chr16	22035885	22036066	-0.89	-0.68
Senp2	chr16	22046889	22047129	-1.44	-0.24
Rnf168	chr16	32277666	32278386	-0.70	-0.12
Rnf168	chr16	32298678	32299428	-1.44	0.24
Golgb1	chr16	36898590	36898921	-0.57	0.08
Golgb1	chr16	36911968	36914157	-0.85	0.05
Golgb1	chr16	36914935	36915176	-1.27	0.23
Golgb1	chr16	36915445	36916525	-2.10	0.18
Golgb1	chr16	36932061	36932212	-1.22	0.38
Golgb1	chr16	36932271	36933171	-1.55	-0.18
Tomm70a	chr16	57152724	57153084	-0.86	0.07

Usp25	chr16	77115246	77116156	-0.36	-0.01
Usp16	chr16	87477080	87480005	-0.31	0.00
Usp7	chr16	8738344	8757028	-1.22	-0.49
Senp5	chr16	31963382	31965869	-1.09	-0.20
Senp5	chr16	31989077	31990517	-2.36	0.14
Sec22a	chr16	35313658	35318950	-0.52	0.07
Nr1i2	chr16	38249474	38251285	-1.74	3.10
Nrip1	chr16	76291226	76295153	-0.33	-0.01
Nrip1	chr16	76352759	76374042	-1.73	-0.89
Tulp4	chr17	6232220	6232851	-0.86	-0.11
Tulp4	chr17	6232910	6233480	-0.35	0.03
Tulp4	chr17	6234109	6236560	-1.49	0.35
Spsb3	chr17	25023648	25024470	-2.71	-0.65
Spsb3	chr17	25027107	25029092	-0.56	0.04
Axin1	chr17	26330968	26332096	-2.64	-0.40
Adamts10	chr17	33665942	33666153	-2.67	-0.99
Adamts10	chr17	33667199	33667439	-1.77	-0.90
Psmb8	chr17	34335288	34335468	-2.26	-0.35
Neu1	chr17	35071556	35072840	-0.43	0.02
Mdc1	chr17	35983321	35985582	-0.49	0.04
Mdc1	chr17	35987884	35987945	-0.39	0.10
Mdc1	chr17	35989459	35989938	-0.32	0.01
Mdc1	chr17	35994492	35994583	-1.40	0.43
Cul7	chr17	46788096	46788277	-3.52	5.23
Cul7	chr17	46788546	46788937	-0.63	-0.57
Cul7	chr17	46790989	46791170	-0.24	0.13
Rab5a	chr17	53618797	53623279	-1.97	-0.24
Fem1a	chr17	56400758	56401238	-0.31	0.01
Fem1a	chr17	56401745	56402672	-0.64	-0.09
Safb	chr17	56733278	56735128	-0.91	0.09
Safb	chr17	56738105	56738646	-2.60	-0.23
Man2a1	chr17	64950988	64974652	-0.54	-0.07
Socs5	chr17	87520136	87534893	-3.18	0.20
Ccnf	chr17	24360684	24360924	-0.81	0.23
Ube2i	chr17	25398025	25398746	-3.13	-0.38
Rab40c	chr17	26020195	26020794	-2.34	-0.34
Pigq	chr17	26073805	26078817	-0.55	-0.05
Rab11b	chr17	33883969	33884119	-3.52	-0.42
Rab11b	chr17	33884327	33884537	-0.51	-0.10
Ring1	chr17	34158095	34160251	-0.37	-0.02
Ly6g6d	chr17	35208321	35208494	-0.16	0.23
Tubb4a	chr17	57220052	57220528	-0.75	-0.24
Tubb4a	chr17	57220705	57221152	-0.66	-0.22

Tubb4a	chr17	57227085	57227175	-0.30	0.20
Rab31	chr17	66001065	66001632	-0.94	0.07
Rab31	chr17	66002078	66002287	-3.48	-0.28
Rab31	chr17	66122031	66122092	-0.23	0.08
Rab12	chr17	66844211	66844961	-0.70	-0.06
Mcfd2	chr17	87654739	87656518	-0.67	0.05
Calm2	chr17	87835011	87846216	-1.79	-0.13
Fbxo11	chr17	88390945	88391656	-2.00	0.16
Fbxo11	chr17	88464415	88464625	-2.23	-0.69
Wac	chr18	7869378	7869558	-0.93	0.30
Wac	chr18	7905960	7910820	-2.90	0.18
Galnt1	chr18	24438485	24443794	-2.39	-0.20
Apc	chr18	34458082	34458232	-2.06	-0.61
Apc	chr18	34470478	34472504	-0.64	0.07
Apc	chr18	34472563	34472923	-0.96	0.19
Apc	chr18	34473880	34475977	-0.83	0.06
Ube2d2a	chr18	35931212	35931392	-0.74	-0.18
Ube2d2a	chr18	35965390	35965540	-0.95	0.36
Commd10	chr18	47246724	47246904	-2.66	0.43
Dctn4	chr18	60686409	60686560	-0.23	-0.01
Dctn4	chr18	60716345	60716496	-0.32	-0.02
Napg	chr18	63154672	63156813	-2.33	-0.27
Tubb6	chr18	67550384	67552330	-2.55	-0.26
Mbd1	chr18	74437185	74438824	-2.30	-0.33
Mbd1	chr18	74439959	74440678	-0.86	-0.13
Mbd1	chr18	74441560	74442159	-3.42	-0.34
Smad7	chr18	75527018	75527704	-1.68	0.45
Smad2	chr18	76422217	76422308	-0.97	-0.22
Smad2	chr18	76462139	76464727	-1.91	0.24
Pias2	chr18	77304625	77336416	-0.55	0.05
Pias2	chr18	77388818	77391933	-2.14	0.29
Usp14	chr18	10030026	10030117	-0.98	-0.24
B4galt6	chr18	20846332	20847761	-1.10	-0.18
Ino80c	chr18	24263918	24264068	-1.17	-0.32
Ino80c	chr18	24264515	24265143	-0.98	0.15
Nr3c1	chr18	39588392	39649457	-2.22	0.16
Tmed7	chr18	46747793	46748242	-2.21	0.16
Lman1	chr18	66140602	66140992	-1.93	-0.18
Lman1	chr18	66162108	66162289	-2.29	-0.31
Smad4	chr18	73799858	73800038	-0.58	0.10
Smad4	chr18	73837362	73837780	-3.05	0.33
Smad4	chr18	73863156	73863395	-2.18	-1.27
Socs6	chr18	89038427	89056418	-2.00	0.18

Sptbn2	chr19	4744144	4744488	-3.16	-1.29
B4gat1	chr19	5039213	5040921	-0.88	-0.09
B4gat1	chr19	5041307	5046422	-3.29	-0.28
B4gat1	chr19	5049005	5049917	-1.92	-0.19
Men1	chr19	6334978	6336811	-2.76	-0.24
Men1	chr19	6338843	6340864	-1.39	-0.08
Stx5a	chr19	8829416	8830132	-2.12	0.19
Ubxn1	chr19	8945985	8946196	-1.74	-0.30
Il33	chr19	30033410	30033650	-0.36	1.61
Stambpl1	chr19	34266759	34267030	-1.24	-0.21
Stambpl1	chr19	34307271	34308637	-0.51	-0.08
Btrc	chr19	45604177	45604358	-1.03	2.52
Gbf1	chr19	46227047	46227228	-0.33	0.03
Nfkb2	chr19	46379346	46380320	-0.71	0.19
Fbxl15	chr19	46403649	46404936	-1.75	-0.27
Rce1	chr19	4622787	4622995	-2.78	-0.50
Incenp	chr19	9947175	9947265	-0.87	0.15
Incenp	chr19	9949324	9951121	-1.33	-0.18
Incenp	chr19	9958237	9968481	-2.40	0.10
Incenp	chr19	9973932	9974023	-0.33	-0.09
Gcnt1	chr19	17403142	17407678	-0.84	0.08
Smc5	chr19	23348276	23348337	-1.02	-0.47
Fbxw4	chr19	45652836	45653256	-0.43	0.04
Actr1a	chr19	46452376	46452913	-1.36	-0.09
Ube2a	chrX	34414360	34414509	-1.29	0.25
Foxo4	chrX	98449866	98450612	-2.10	-0.37
Ogt	chrX	98874610	98878285	-3.11	0.13
Piga	chrX	160857718	160861236	-3.59	0.49
Piga	chrX	160866911	160869851	-2.05	0.57
Piga	chrX	160870327	160870388	-0.37	0.22
Cul4b	chrX	35916890	35916951	-2.43	0.70
Hcfc1	chrX	71189302	71189693	-1.73	-0.14
Hcfc1	chrX	71194650	71196000	-0.95	0.04
Hcfc1	chrX	71211533	71211654	-1.28	0.19
Chm	chrX	110156917	110157037	-1.01	0.43
Vcpip1	chr1	9714443	9738463	-1.88	0.05
Napb	chr2	148538465	148558156	-1.00	0.56
Tnks	chr8	36028534	36028744	-1.19	-0.36
Dynll2	chr11	87794645	87799923	-0.33	-0.01
Dynll2	chr11	87800072	87801030	-2.21	-0.32

Adaptive immune system

gene_name	chr	chromStart	chromEnd	diff.lg.p	diff.log2.fc
Cd28	chr1	60826604	60826815	-0.22	-0.36

Cd28	chr1	60827743	60827984	-0.70	-1.34
Psmd1	chr1	87961193	87961254	-0.37	-0.11
Inpp5d	chr1	89516916	89517096	-1.01	-0.27
Inpp5d	chr1	89614465	89616815	-0.79	-0.06
Ube2f	chr1	93182033	93182602	-1.08	-0.09
Asb1	chr1	93440810	93443711	-1.60	-0.49
Asb1	chr1	93451373	93451734	-0.52	0.13
Ncf2	chr1	154654984	154655314	-0.34	0.01
Hecw2	chr1	53869614	53883756	-1.23	1.77
Hecw2	chr1	53982375	53983924	-0.31	-0.08
Tuba4a	chr1	75211607	75211936	-0.33	0.05
Tuba4a	chr1	75212113	75215828	-2.13	-0.09
Ap1s3	chr1	79668486	79668547	-0.31	-0.11
Cul3	chr1	80336855	80337005	-0.41	-0.05
Trip12	chr1	84782695	84836815	-0.84	-0.03
Pdcd1	chr1	95937874	95949130	-2.07	-1.60
Klhl20	chr1	163019047	163019258	-2.02	-0.55
Klhl20	chr1	163020426	163023835	-3.23	0.59
Klhl20	chr1	163039327	163039897	-0.46	0.06
Ppp2r5a	chr1	193176577	193176847	-1.42	0.15
Ppp2r5a	chr1	193196363	193220920	-1.78	-0.16
Anapc2	chr2	25128463	25128941	-1.02	-0.11
Fbxw5	chr2	25358984	25359776	-1.40	-0.18
Fbxw5	chr2	25360013	25360422	-1.11	-0.22
Mapkap1	chr2	34479153	34479423	-2.45	-0.44
Hspa5	chr2	34629598	34629837	-1.43	0.04
Hspa5	chr2	34630750	34631960	-2.77	-0.03
Klhl41	chr2	69508654	69508893	-1.20	0.67
Dync1i2	chr2	71049792	71050012	-1.03	0.40
Itgav	chr2	83643777	83643958	-1.72	-0.33
Psmc3	chr2	90894439	90895182	-2.45	-0.16
Traf6	chr2	101531587	101537959	-2.12	0.17
Kif18a	chr2	109173521	109173672	-0.98	-0.57
Trim69	chr2	122004226	122004674	-0.56	-1.46
Kif3b	chr2	153141983	153146011	-2.34	0.20
Kif3b	chr2	153155890	153156639	-1.20	0.17
Kif3b	chr2	153158285	153158915	-0.67	0.12
Itch	chr2	154959216	154959307	-0.30	0.07
Itch	chr2	155050132	155050403	-0.62	-0.11
Src	chr2	157295196	157296561	-1.97	0.63
Cd40	chr2	164896238	164896475	-0.37	-0.13
Cd40	chr2	164896681	164896977	-0.48	0.36
Rnf114	chr2	167318622	167318832	-0.88	-1.10

Tubb1	chr2	174282844	174283054	-0.17	0.13
Tubb4b	chr2	25077855	25080192	-0.38	-0.01
Lrsam1	chr2	32781993	32782383	-0.52	-0.14
Fbxw2	chr2	34661048	34661869	-2.35	-0.23
Fbxw2	chr2	34662794	34664040	-0.82	-0.21
Fbxw2	chr2	34668436	34678672	-0.41	-0.02
Fbxw2	chr2	34681468	34681725	-0.52	0.16
Psmd5	chr2	34707993	34709823	-0.82	-0.10
Psmd5	chr2	34726274	34726482	-3.28	-0.70
Psmb7	chr2	38498946	38499426	-1.70	-0.24
Ptprj	chr2	90420444	90420774	-0.85	-0.18
Anapc1	chr2	128436537	128438637	-0.86	0.07
Ubox5	chr2	130416996	130417596	-0.43	0.07
Ubox5	chr2	130425355	130426375	-3.53	-0.43
Siglec1	chr2	130895224	130895524	-0.41	-0.15
Siglec1	chr2	130900602	130901900	-0.28	0.00
Siglec1	chr2	130903954	130904467	-0.53	0.19
Siglec1	chr2	130907034	130909110	-0.62	-0.22
Siglec1	chr2	130912201	130912381	-0.76	0.65
Psmf1	chr2	151543679	151544068	-0.32	0.02
Trib3	chr2	152163160	152164409	-0.93	0.07
Trib3	chr2	152169617	152169796	-0.42	-0.11
Nfatc2	chr2	168301999	168303229	-1.92	-0.20
Pik3ca	chr3	32335072	32335372	-0.45	-0.08
Pik3ca	chr3	32360681	32362022	-3.50	0.35
Ctso	chr3	81756174	81758885	-1.40	0.23
Fbxw7	chr3	84619498	84694070	-0.58	0.12
Fbxw7	chr3	84694129	84756226	-2.67	0.26
Fbxw7	chr3	84780140	84781955	-0.67	0.08
Ube2q1	chr3	89577560	89577890	-1.31	-0.19
Ube2q1	chr3	89585705	89587919	-2.90	0.11
Them4	chr3	94135497	94135946	-0.95	0.23
Ctss	chr3	95330707	95333452	-1.96	-0.10
Rnf115	chr3	96593799	96594008	-1.03	-0.13
Cenpe	chr3	134902767	134903916	-0.98	0.27
Cenpe	chr3	134905051	134905504	-0.46	0.08
Cenpe	chr3	134910323	134910534	-0.46	0.09
Cenpe	chr3	134936264	134936504	-0.62	0.21
Bcl10	chr3	145587262	145587592	-1.35	-0.17
Bcl10	chr3	145588099	145593586	-0.30	0.00
Pag1	chr3	9691860	9692940	-2.76	-0.45
Siah2	chr3	58479649	58495411	-0.38	-0.02
Siah2	chr3	58495680	58495891	-0.39	-0.11

Psmb4	chr3	94690551	94690880	-2.31	-0.17
Cd101	chr3	100797451	100797632	-1.28	-1.85
Sec24b	chr3	129686102	129687101	-1.10	-0.09
Dapp1	chr3	137595557	137598550	-1.00	-0.29
Prkacb	chr3	146394723	146394993	-0.71	0.12
Map3k7	chr4	32051081	32051232	-1.86	-0.83
Map3k7	chr4	32110141	32110231	-1.49	-0.76
Ube2r2	chr4	41137807	41138136	-0.82	-0.10
Trim32	chr4	65274556	65274646	-0.66	-0.27
Lrrc41	chr4	115768961	115769201	-3.42	-0.29
Capzb	chr4	138748845	138794826	-1.20	-0.15
Mtor	chr4	147930392	147931764	-0.76	0.10
Klhl21	chr4	151383838	151389959	-1.59	-0.17
Klhl21	chr4	151390257	151390408	-2.24	0.94
Ufl1	chr4	25177588	25177679	-0.29	-0.02
Fbxo10	chr4	45048105	45048374	-0.54	-0.64
Fbxo10	chr4	45048672	45048852	-0.34	0.51
Fbxo10	chr4	45071353	45071981	-0.56	-0.37
Cdc26	chr4	62055609	62055968	-0.83	0.27
Kif2c	chr4	116832539	116832928	-2.15	-0.69
Rnf220	chr4	116961136	116968749	-1.18	-0.20
Lck	chr4	129225826	129225977	-1.80	-0.75
Fbxo6	chr4	147520122	147520183	-0.29	0.01
Pik3cd	chr4	149023545	149025880	-1.73	-0.13
Spsb1	chr4	149271976	149281277	-0.37	-0.15
Mib2	chr4	155028700	155028761	-1.11	-1.14
Ube3c	chr5	29895781	29895962	-1.11	-0.74
Ube3c	chr5	30001057	30001298	-0.35	0.03
Anapc4	chr5	53257704	53257973	-1.19	-0.20
Klhl5	chr5	65558241	65558691	-1.07	-0.19
Ube3b	chr5	114869623	114869922	-2.59	-0.34
Fbxo21	chr5	118452093	118458648	-2.38	-0.37
Anapc7	chr5	122891328	122891508	-1.48	-0.33
Psmd9	chr5	123678228	123678437	-1.01	0.20
Kctd7	chr5	130628217	130628755	-1.29	-0.61
Fbxl5	chr5	44142150	44149098	-0.30	0.01
Fbxl5	chr5	44149396	44151063	-1.01	0.09
Rchy1	chr5	92391795	92392094	-2.32	0.64
Sec31a	chr5	100790907	100791058	-1.68	-0.19
Fbxw8	chr5	118515497	118515767	-0.43	-0.12
Ptpn11	chr5	121581827	121582307	-1.65	0.20
Ptpn11	chr5	121583292	121587519	-2.53	-0.16
Anapc5	chr5	123271138	123271348	-1.70	-0.25

Ubc	chr5	125867967	125870328	-2.39	0.10
Pilra	chr5	138276667	138276728	-0.74	1.17
Card11	chr5	141348952	141350301	-2.19	-0.58
Fbxl18	chr5	143640471	143644134	-2.38	-0.99
Rac1	chr5	144288651	144288861	-1.33	0.22
Smurf1	chr5	145637663	145637963	-1.05	-0.44
Smurf1	chr5	145638322	145638682	-0.71	-0.20
Smurf1	chr5	145639310	145639521	-0.43	0.15
Smurf1	chr5	145726518	145726669	-0.30	0.31
Rnf6	chr5	147021822	147022092	-0.81	0.13
Rnf6	chr5	147023104	147032058	-0.38	-0.01
Cul1	chr6	47474617	47476108	-2.74	-0.21
Znrf2	chr6	54838538	54838599	-0.34	-0.02
Herc6	chr6	57531001	57531152	-0.51	-0.34
Dctn1	chr6	83140226	83141007	-1.23	-0.14
Vhl	chr6	113578147	113579978	-1.22	0.20
Fbxl14	chr6	119429685	119431421	-0.82	0.06
Klhl42	chr6	147039984	147056940	-0.79	-0.10
Ube2h	chr6	30163827	30164395	-0.70	-0.09
Ube2h	chr6	30164783	30164963	-0.64	0.31
Vamp8	chr6	72335214	72335477	-0.41	-0.03
Vamp8	chr6	72338403	72340632	-1.83	-0.34
Raf1	chr6	115568590	115569280	-0.54	-0.05
Ptpn6	chr6	124683045	124688519	-0.44	0.09
Kras	chr6	145168813	145168964	-1.25	-0.35
Itpr2	chr6	146060001	146060981	-1.32	0.19
Ap2s1	chr7	17323792	17328621	-1.80	-0.22
Akt2	chr7	28422173	28423428	-0.40	0.03
Ube3a	chr7	66484119	66496353	-3.29	0.33
Klhl25	chr7	82993313	83012167	-0.53	-0.05
Klhl25	chr7	83018746	83018926	-0.98	-0.56
Ctsc	chr7	95437274	95437455	-0.26	0.00
Ctsc	chr7	95458037	95458487	-2.08	0.25
Dctn5	chr7	129276554	129277689	-0.62	0.13
Dctn5	chr7	129291850	129292499	-2.82	0.17
Rbbp6	chr7	130114077	130114468	-1.85	-0.17
Rbbp6	chr7	130140585	130140736	-1.81	-0.24
Fbxl19	chr7	134911014	134911817	-1.61	-0.24
Fbxl19	chr7	134911935	134912442	-2.06	-0.46
Stx4a	chr7	134985380	134985840	-3.36	-0.34
Lair1	chr7	3961115	3961265	-1.04	-0.76
Ube2m	chr7	13623108	13623619	-2.32	-0.37
Calm3	chr7	17502039	17502309	-2.51	-0.14

Klc3	chr7	19979788	19981184	-1.39	-0.21
Nectin2	chr7	20302531	20302921	-0.73	-0.39
Nectin2	chr7	20306812	20310098	-0.79	-0.21
Nectin2	chr7	20334682	20334892	-0.51	-0.23
Pvr	chr7	20490268	20494550	-0.53	0.08
Cd22	chr7	31662583	31662644	-0.18	-0.74
Siglece	chr7	50915321	50915471	-0.80	0.91
Asb7	chr7	73792504	73823980	-1.38	-0.19
Det1	chr7	85972688	85972809	-1.30	-0.59
Det1	chr7	85988116	85988896	-0.39	0.03
Trim21	chr7	109707151	109708560	-3.22	0.40
Kif22	chr7	134179351	134185934	-1.83	-0.72
Ifitm2	chr7	148141799	148141860	-1.53	-0.15
Hras	chr7	148376758	148378177	-0.33	-0.01
Hras	chr7	148378805	148379903	-1.48	0.51
Cdc16	chr8	13779263	13781763	-1.50	-0.16
Ppp2cb	chr8	34710092	34710330	-0.63	-0.43
Sh3rf1	chr8	63703117	63808042	-0.87	0.26
Sh3rf1	chr8	63863646	63863797	-0.27	0.10
Prkaca	chr8	86496876	86500855	-3.14	-0.78
Fbxl8	chr8	107791952	107793137	-2.37	-0.45
Nfatc3	chr8	108602601	108603618	-1.85	0.13
Nfatc3	chr8	108632465	108652166	-1.29	0.10
Ap1g1	chr8	112382745	112384686	-0.52	0.03
Znrf1	chr8	114060539	114062246	-1.56	-0.12
Znrf1	chr8	114143157	114145711	-0.85	-0.11
Znrf1	chr8	114145770	114146010	-0.96	0.20
Znrf1	chr8	114146219	114147028	-1.23	-0.08
Plcg2	chr8	120022190	120028105	-0.37	-0.04
Tubb3	chr8	125944632	125944960	-0.21	-0.02
Itgb1	chr8	131209643	131209853	-0.47	-0.04
Ikbkb	chr8	23770546	23770997	-0.43	-0.04
Dctn6	chr8	35169406	35171536	-0.90	0.26
Lonrf1	chr8	37280167	37282962	-0.49	0.09
Klhl2	chr8	67219421	67219870	-1.56	-0.30
Pik3r2	chr8	73292079	73292528	-3.00	-0.41
Pik3r2	chr8	73298580	73300551	-1.20	-0.17
Siah1a	chr8	89248786	89269816	-0.55	0.06
Dync1li2	chr8	106966676	106966737	-0.30	-0.05
Fbxo31	chr8	124075878	124076177	-0.56	0.18
Icam1	chr9	20820403	20823609	-1.32	-0.17
Icam1	chr9	20824057	20824596	-2.71	0.54
Icam1	chr9	20831648	20833180	-0.80	0.03

Icam5	chr9	20839000	20839269	-0.89	0.59
Icam5	chr9	20840869	20842061	-0.40	0.09
Dnm2	chr9	21310781	21311349	-0.34	0.01
Dnm2	chr9	21311898	21312198	-3.13	0.36
Ppp2r1b	chr9	50688020	50688710	-0.53	0.05
Ppp2r1b	chr9	50701016	50701675	-1.83	-0.33
Fbxo22	chr9	55066270	55071642	-1.39	0.12
Fbxo22	chr9	55071791	55072031	-0.79	0.18
Anapc13	chr9	102528625	102536370	-0.39	-0.04
Dcaf1	chr9	106739021	106740882	-1.41	0.32
Dcaf1	chr9	106760607	106761940	-2.76	0.33
Dcaf1	chr9	106776529	106782364	-0.77	0.23
Dync1li1	chr9	114632686	114632895	-0.48	0.07
Fbxl12	chr9	20448880	20449151	-1.08	-0.58
Keap1	chr9	21034503	21042276	-2.14	0.12
Keap1	chr9	21043235	21043776	-0.68	-0.28
Crtam	chr9	40781146	40781531	-0.39	-0.40
Ube4a	chr9	44731538	44732048	-2.54	0.32
Rnf111	chr9	70274555	70277420	-1.37	0.15
Fbxo9	chr9	77943550	77943611	-0.20	0.01
Arih2	chr9	108522434	108547287	-1.40	0.26
Trim71	chr9	114422150	114422300	-0.59	3.58
Fyn	chr10	39089604	39089964	-2.08	-0.38
Fyn	chr10	39271197	39284162	-0.31	0.00
Itgb2	chr10	76993092	77005145	-2.27	-0.27
Ube2g2	chr10	77107280	77107637	-1.28	-0.14
Ube2g2	chr10	77108022	77108082	-0.37	-0.06
Icosl	chr10	77536549	77536670	-0.36	-0.19
Rnf41	chr10	127874437	127875799	-0.76	-0.07
Tab2	chr10	7625983	7626463	-0.30	0.00
Tab2	chr10	7626701	7629919	-0.81	-0.05
Tab2	chr10	7630969	7644574	-2.87	-0.09
Ube2d1	chr10	70718174	70718562	-1.17	0.19
Rnf126	chr10	79229508	79229686	-1.66	-0.45
Fzr1	chr10	80829742	80830217	-2.52	0.24
Kif5a	chr10	126700029	126700180	-1.47	-0.63
Ap1b1	chr11	4941834	4942313	-0.40	-0.03
Psme4	chr11	30671804	30671925	-0.83	-0.91
Psme4	chr11	30672014	30672135	-0.38	0.12
Fbxw11	chr11	32644536	32645016	-2.21	0.20
Ppp2ca	chr11	51935449	51935836	-1.90	0.10
Trim11	chr11	58791617	58791916	-3.37	-0.52
Trim11	chr11	58802336	58802515	-0.53	0.13

Trim11	chr11	58802842	58804483	-2.09	-0.17
Trim11	chr11	58804542	58804960	-2.11	0.29
Ube2g1	chr11	72493612	72497325	-2.70	0.54
Psmd11	chr11	80285544	80285635	-1.72	-0.32
Psme3	chr11	101177564	101178336	-0.63	-0.07
Psme3	chr11	101182070	101183288	-1.70	-0.15
Trim41	chr11	48629367	48630893	-1.35	-0.11
Sec24a	chr11	51569959	51570170	-2.57	-1.70
Wsb1	chr11	79053599	79053898	-1.62	-0.11
Ube2z	chr11	95911494	95911704	-3.10	-0.34
Fbxl20	chr11	97950510	97953462	-0.36	0.03
Klhl11	chr11	100333798	100334096	-1.09	0.31
Map3k14	chr11	103081077	103082247	-2.79	-0.40
Map3k14	chr11	103100406	103100995	-3.03	-0.56
Map3k14	chr11	103103496	103128715	-0.28	0.01
Cd79b	chr11	106172654	106173044	-1.64	3.90
Smurf2	chr11	106683743	106684358	-0.92	-0.20
Cd300e	chr11	114914519	114914670	-0.30	0.16
Grb2	chr11	115505517	115505876	-0.46	0.05
Grb2	chr11	115506621	115508033	-1.62	0.09
Ube2o	chr11	116399663	116399932	-0.71	0.17
Ube2o	chr11	116400140	116404120	-1.25	-0.09
Ube2o	chr11	116405905	116405995	-0.32	0.03
Kif3c	chr12	3403323	3403414	-2.27	-0.91
Kif3c	chr12	3405476	3405866	-0.46	0.05
Psma6	chr12	56499811	56499871	-1.67	-0.32
Lrr1	chr12	70275442	70276002	-0.27	0.05
Psmc1	chr12	101348377	101351286	-1.53	-0.29
Calm1	chr12	101445209	101445479	-1.73	0.08
Dync1h1	chr12	111839634	111839815	-0.77	-0.17
Dync1h1	chr12	111855964	111857506	-0.91	0.03
Dync1h1	chr12	111904592	111905154	-0.63	0.04
Klc1	chr12	112997059	113010574	-1.27	-0.23
Btbd6	chr12	114215550	114217061	-3.22	-0.34
Ppp2r5e	chr12	76616497	76694287	-1.19	0.16
Ppp2r5e	chr12	76696737	76697187	-3.02	-0.33
Arel1	chr12	86259755	86261519	-1.15	-0.07
Akt1	chr12	113912217	113912487	-0.40	0.08
Rnf182	chr13	43763512	43763603	-0.50	3.17
Rnf144b	chr13	47338305	47340700	-1.37	-0.32
Rnf144b	chr13	47342881	47342942	-0.24	0.19
Rnf144b	chr13	47343000	47343181	-1.76	-1.16
Syk	chr13	52737300	52738605	-0.48	-0.06

Syk	chr13	52738964	52742902	-1.56	-0.08
Syk	chr13	52743830	52743951	-1.80	-0.43
Erap1	chr13	74777319	74783985	-2.44	0.26
Tubb2b	chr13	34219290	34219557	-1.15	1.09
Tubb2b	chr13	34219999	34221135	-0.49	0.22
Ctsl	chr13	64470393	64471614	-2.04	-0.09
Pik3r1	chr13	102454016	102456264	-1.08	0.13
Pik3r1	chr13	102478932	102525656	-0.51	0.13
Pik3r1	chr13	102527214	102538172	-1.57	0.16
Kctd6	chr14	9052713	9055546	-1.19	-0.22
Psmc6	chr14	45949498	45950514	-0.68	0.11
Kbtbd7	chr14	79827126	79828716	-0.67	0.13
Ppp3cb	chr14	21320302	21327131	-1.02	-0.09
Ppp3cb	chr14	21365614	21365735	-0.60	-0.29
Fbxl3	chr14	103482030	103488661	-0.97	0.07
Fbxl3	chr14	103491640	103498726	-2.05	0.22
Rictor	chr15	6736486	6737266	-3.47	-0.21
Rictor	chr15	6745928	6747254	-1.18	-0.11
Ncf4	chr15	78075240	78075447	-1.52	-0.21
Ncf4	chr15	78091438	78093010	-2.87	-0.16
Skp2	chr15	9043329	9043510	-0.80	-0.31
Skp2	chr15	9069207	9070137	-0.35	-0.03
Rnf19a	chr15	36170916	36173866	-0.57	0.06
Fbxo32	chr15	58011932	58012112	-0.37	-0.51
Fbxo32	chr15	58012410	58013069	-1.76	-1.01
Fbxo32	chr15	58046282	58046433	-1.44	-1.83
Col2a1	chr15	97806181	97807085	-2.85	2.87
Asb8	chr15	97965969	97971774	-0.40	-0.02
Racgap1	chr15	99470224	99473919	-3.35	-0.72
Itgb7	chr15	102046603	102046957	-1.86	-0.16
Itgb7	chr15	102057968	102062366	-0.36	-0.03
Ap2m1	chr16	20535576	20537801	-0.67	-0.19
Itgb5	chr16	33829750	33830079	-1.89	-0.82
Itgb5	chr16	33919922	33920281	-0.73	-0.11
Itgb5	chr16	33947843	33949245	-0.62	-0.07
Cd80	chr16	38486386	38486895	-0.99	-0.15
Cd80	chr16	38487013	38494806	-3.34	0.87
Cd200r1	chr16	44794312	44794493	-1.45	-0.86
Cd200r4	chr16	44811897	44820870	-2.46	-1.49
Cd200r2	chr16	44915270	44915805	-0.75	-0.67
Cblb	chr16	52204595	52205075	-0.66	-0.17
Socs1	chr16	10783990	10785629	-0.29	0.03
Pak2	chr16	32019832	32021909	-3.04	0.15

Dtx3l	chr16	35928994	35932046	-0.87	0.09
Dtx3l	chr16	35932315	35933572	-1.20	0.10
Cd86	chr16	36604193	36604492	-0.58	-0.63
Cd86	chr16	36620826	36620887	-0.49	0.89
Itpr3	chr17	27258568	27258749	-1.05	-0.28
H2-DMa	chr17	34272232	34272650	-2.02	0.70
Tap1	chr17	34333659	34333780	-0.87	-0.27
Psmb8	chr17	34335288	34335468	-2.26	-0.35
H2-D1	chr17	35400807	35401046	-2.46	-0.23
H2-Q2	chr17	35479307	35479659	-0.13	0.17
H2-Q6	chr17	35565949	35566691	-0.29	0.01
Nfkbie	chr17	45697418	45700117	-2.30	-0.19
Cul7	chr17	46788096	46788277	-3.52	5.23
Cul7	chr17	46788546	46788937	-0.63	-0.57
Cul7	chr17	46790989	46791170	-0.24	0.13
Trem1	chr17	48376164	48376373	-0.47	-1.34
Trem1	chr17	48383770	48384842	-0.66	0.48
Treml2	chr17	48439323	48439534	-0.18	0.36
Treml2	chr17	48449130	48449580	-0.30	-0.03
Vav1	chr17	57418522	57418613	-1.00	-0.44
Vav1	chr17	57467106	57467554	-0.56	0.05
Rasgrp3	chr17	75924352	75926570	-0.37	0.01
Rasgrp3	chr17	75928063	75928184	-1.00	-0.25
Lnpep	chr17	17715497	17746283	-1.25	0.05
Ccnf	chr17	24360684	24360924	-0.81	0.23
Mlst8	chr17	24612351	24613015	-1.70	-0.31
Trim39	chr17	36397225	36398550	-1.71	0.22
Klc4	chr17	46781254	46782971	-0.90	-0.29
Ppp2r5d	chr17	46820058	46820802	-0.99	-0.12
Ppp2r5d	chr17	46837029	46841951	-0.82	-0.21
Ubr2	chr17	47147240	47147481	-2.49	-0.41
Tubb4a	chr17	57220052	57220528	-0.75	-0.24
Tubb4a	chr17	57220705	57221152	-0.66	-0.22
Tubb4a	chr17	57227085	57227175	-0.30	0.20
Pja2	chr17	64658065	64658455	-0.40	-0.03
Pja2	chr17	64658514	64660528	-0.38	0.03
Xdh	chr17	74233606	74233756	-0.51	-0.15
Sos1	chr17	80797100	80799097	-0.65	-0.07
Sos1	chr17	80833039	80834290	-0.49	-0.07
Sos1	chr17	80879343	80879554	-0.37	-0.28
Calm2	chr17	87835011	87846216	-1.79	-0.13
Fbxo11	chr17	88390945	88391656	-2.00	0.16
Fbxo11	chr17	88464415	88464625	-2.23	-0.69

Colec12	chr18	9707645	9840283	-0.41	-0.02
Colec12	chr18	9848135	9859890	-2.84	0.07
Ube2d2a	chr18	35931212	35931392	-0.74	-0.18
Ube2d2a	chr18	35965390	35965540	-0.95	0.36
Rnf14	chr18	38467690	38467990	-1.19	0.25
Dctn4	chr18	60686409	60686560	-0.23	-0.01
Dctn4	chr18	60716345	60716496	-0.32	-0.02
Tubb6	chr18	67550384	67552330	-2.55	-0.26
Mex3c	chr18	73732718	73749293	-3.27	-0.38
Mex3c	chr18	73749352	73750612	-0.69	-0.04
Map3k8	chr18	4348929	4352951	-0.72	-0.15
Cdc23	chr18	34793500	34793621	-0.34	-0.05
Trim36	chr18	46326899	46328816	-2.13	0.74
Nfatc1	chr18	80803003	80803394	-3.39	-0.87
Nfatc1	chr18	80803872	80832717	-0.36	0.02
Nfatc1	chr18	80846232	80846653	-2.89	0.45
Nfatc1	chr18	80879052	80904865	-0.59	-0.05
Sptbn2	chr19	4744144	4744488	-3.16	-1.29
Pdcd1lg2	chr19	29545180	29545271	-0.76	-2.35
Btrc	chr19	45604177	45604358	-1.03	2.52
Fbxl15	chr19	46403649	46404936	-1.75	-0.27
Sipa1	chr19	5651184	5651544	-1.35	0.18
Sipa1	chr19	5653976	5655118	-2.76	-0.25
Sipa1	chr19	5659601	5662646	-2.78	-0.18
Fbxw4	chr19	45652836	45653256	-0.43	0.04
Actr1a	chr19	46452376	46452913	-1.36	-0.09
Ube2a	chrX	34414360	34414509	-1.29	0.25
Kif4	chrX	97921953	97922371	-0.51	0.14
Was	chrX	7667556	7667617	-0.56	-0.25
Pja1	chrX	96661699	96661909	-2.14	0.49
Pja1	chrX	96662087	96663013	-1.28	0.13
Pja1	chrX	96663132	96663849	-0.65	-0.09
Siah1b	chrX	160509608	160510052	-0.28	-0.01
Stim1	chr7	109416755	109416875	-0.27	-0.02
Dynll2	chr11	87794645	87799923	-0.33	-0.01
Dynll2	chr11	87800072	87801030	-2.21	-0.32

Death receptor signaling

gene_name	chr	chromStart	chromEnd	diff.lg.p	diff.log2.fc
Arhgef4	chr1	34868671	34868911	-1.94	-0.94
Cflar	chr1	58779883	58783419	-1.48	0.39
Casp8	chr1	58890568	58901676	-0.30	0.00
Psen2	chr1	182157552	182159055	-1.26	0.19
Traf6	chr2	101531587	101537959	-2.12	0.17

Bcl2l11	chr2	127953514	127954562	-2.45	-0.34
Traf2	chr2	25374427	25375145	-1.67	-0.19
Vav2	chr2	27282074	27282345	-1.98	-1.07
Madd	chr2	90977576	90977726	-1.53	-0.64
Madd	chr2	91018210	91018420	-1.66	-0.48
Prex1	chr2	166392563	166394859	-0.46	0.03
Tnfsf10	chr3	27234510	27234750	-0.88	4.75
Prkci	chr3	30949204	30949475	-1.58	0.40
Arhgef11	chr3	87541536	87541835	-0.65	0.12
Otud7b	chr3	95908449	95908540	-1.61	-0.69
Otud7b	chr3	95956546	95960193	-1.03	-0.07
Vav3	chr3	109143660	109143900	-1.03	-0.68
Map3k7	chr4	32051081	32051232	-1.86	-0.83
Map3k7	chr4	32110141	32110231	-1.49	-0.76
Plekhg5	chr4	151487948	151488609	-1.41	-0.63
Plekhg5	chr4	151489058	151489179	-1.08	-1.11
Ubc	chr5	125867967	125870328	-2.39	0.10
Rac1	chr5	144288651	144288861	-1.33	0.22
Akap13	chr7	82748167	82754850	-1.77	0.09
Plekhg2	chr7	29155131	29155328	-1.21	1.12
Plekhg2	chr7	29155536	29156236	-2.24	1.13
Plekhg2	chr7	29156874	29157024	-3.37	-1.59
Arhgef17	chr7	108021528	108021589	-0.81	-1.22
Arhgef17	chr7	108021798	108022069	-0.88	-0.83
Arhgef17	chr7	108077855	108078066	-0.22	-0.30
Arhgef17	chr7	108078725	108078935	-0.18	0.59
Arhgef17	chr7	108079114	108079235	-0.23	-1.00
Fadd	chr7	151765572	151768221	-1.92	-0.22
Arhgef18	chr8	3452259	3453370	-2.83	-0.80
Arhgef18	chr8	3454924	3455254	-2.67	-0.76
Arhgef7	chr8	11831528	11833540	-2.52	-0.13
Mcf2l	chr8	13019010	13019580	-1.10	-0.25
Casp3	chr8	47702790	47715112	-2.57	1.25
Cyld	chr8	91220956	91221047	-0.33	-0.20
Ikbkb	chr8	23770546	23770997	-0.43	-0.04
Bag4	chr8	26877828	26878848	-1.70	0.20
Tradd	chr8	107782474	107783499	-0.91	0.12
Tradd	chr8	107783873	107788494	-1.84	-0.49
Usp2	chr9	43902061	43902302	-1.62	-0.76
Usp4	chr9	108293381	108294591	-0.82	0.07
Birc2	chr9	7818494	7818943	-1.37	0.33
Birc2	chr9	7834955	7835225	-0.88	0.29
Birc3	chr9	7848939	7850928	-0.65	0.09

Birc3	chr9	7860281	7872961	-1.11	0.10
Arhgef12	chr9	42780101	42782733	-1.98	0.35
Aph1b	chr9	66626699	66627088	-0.39	-0.12
Aph1c	chr9	66666666	66667266	-0.43	-0.05
Myd88	chr9	119248949	119249158	-0.83	-0.18
Sppl2b	chr10	80329686	80329837	-1.06	-0.56
Tab2	chr10	7625983	7626463	-0.30	0.00
Tab2	chr10	7626701	7629919	-0.81	-0.05
Tab2	chr10	7630969	7644574	-2.87	-0.09
Tnfaip3	chr10	18721581	18725108	-0.69	-0.06
Rtn4	chr11	29606743	29607552	-1.68	0.11
Ywhae	chr11	75546388	75546567	-2.12	-0.15
Gna13	chr11	109226954	109241616	-3.14	-0.32
Abr	chr11	76308107	76308378	-1.66	0.52
Psen1	chr12	85072709	85075730	-1.54	-0.08
Adam17	chr12	21330026	21333946	-1.67	0.06
Sos2	chr12	70685554	70689696	-0.63	-0.04
Sos2	chr12	70782599	70782839	-0.62	-0.54
Ripk1	chr13	34119794	34126112	-1.97	0.13
Net1	chr13	3883211	3883659	-0.31	-0.01
Net1	chr13	3892692	3892782	-0.37	-0.35
Fgd3	chr13	49358478	49360098	-0.37	0.01
Fgd3	chr13	49391745	49393309	-2.16	-0.24
Arhgef3	chr14	28213491	28215536	-1.55	0.20
Tnfrsf10b	chr14	70182066	70182455	-1.70	0.70
Tab1	chr15	79989200	79992132	-3.29	-0.26
Otulin	chr15	27535675	27538550	-1.58	-0.11
Otulin	chr15	27553110	27560448	-2.84	-0.65
Trio	chr15	27661511	27663334	-3.28	0.24
Trio	chr15	27673777	27673868	-0.30	-0.02
Trio	chr15	27677706	27678186	-0.55	-0.07
Trio	chr15	27830982	27834917	-1.73	-0.20
Sharpin	chr15	76180541	76181540	-0.51	-0.06
Fgd4	chr16	16422549	16422879	-0.77	-0.22
Fgd4	chr16	16475223	16482703	-0.73	-0.21
Tiam1	chr16	89789181	89791963	-1.20	0.31
Tiam1	chr16	89884989	89898536	-1.55	0.32
Tiam2	chr17	3518287	3518737	-0.28	0.02
Tiam2	chr17	3519186	3519397	-1.04	-0.52
Vav1	chr17	57418522	57418613	-1.00	-0.44
Vav1	chr17	57467106	57467554	-0.56	0.05
Sos1	chr17	80797100	80799097	-0.65	-0.07
Sos1	chr17	80833039	80834290	-0.49	-0.07

Sos1	chr17	80879343	80879554	-0.37	-0.28
Bad	chr19	7017092	7025164	-0.77	-0.18
Xiap	chrX	39455267	39458162	-2.29	0.32
Tab3	chrX	82860492	82860673	-1.77	0.62
Bex3	chrX	132804820	132805873	-0.58	0.08
Irak1	chrX	71260386	71261911	-1.24	0.14

Metabolism of RNA

gene_name	chr	chromStart	chromEnd	diff.lg.p	diff.log2.fc
Imp4	chr1	34496703	34496907	-0.50	-0.12
Cnot9	chr1	74575438	74575798	-0.32	0.01
Utp14b	chr1	78654429	78655125	-0.60	-0.10
Psmd1	chr1	87961193	87961254	-0.37	-0.11
Sf3b1	chr1	55084231	55084322	-0.60	-0.19
Ncl	chr1	88254748	88256030	-1.99	0.08
Zc3h11a	chr1	135516447	135517433	-0.86	0.06
Zc3h11a	chr1	135517641	135518703	-1.96	0.10
Zc3h11a	chr1	135521340	135523811	-0.72	0.04
Smg7	chr1	154685051	154685410	-0.36	-0.03
Dhx9	chr1	155303276	155303397	-3.17	0.68
Hnrnpu	chr1	180259373	180260299	-2.65	-0.09
Diexf	chr1	194933444	194933955	-0.31	-0.01
Diexf	chr1	194944575	194948207	-1.70	-0.17
Exosc2	chr2	31536119	31536296	-0.62	-0.13
Nup214	chr2	31888785	31893623	-1.12	-0.08
Nup214	chr2	31907789	31907850	-0.40	-0.11
Psmc3	chr2	90894439	90895182	-2.45	-0.16
Nop56	chr2	130100147	130100394	-0.55	0.15
Nxt1	chr2	148498350	148501762	-0.48	-0.03
Ctnnbl1	chr2	157563136	157600285	-2.55	-0.24
Srsf6	chr2	162757243	162757817	-0.60	-0.04
Cstf1	chr2	172203213	172206586	-1.68	-0.11
Rpp38	chr2	3246220	3249900	-1.27	0.14
Rbm17	chr2	11522350	11524826	-0.61	-0.12
Psmd5	chr2	34707993	34709823	-0.82	-0.10
Psmd5	chr2	34726274	34726482	-3.28	-0.70
Psmb7	chr2	38498946	38499426	-1.70	-0.24
Prpf40a	chr2	53049040	53050262	-1.20	0.19
Cwc22	chr2	77733949	77741916	-1.28	-0.10
Clp1	chr2	84563548	84566299	-1.88	0.17
Snrpb	chr2	130002885	130005100	-1.37	-0.14
Psmf1	chr2	151543679	151544068	-0.32	0.02
Smg5	chr3	88140181	88145781	-1.90	-0.42
Smg5	chr3	88154740	88155513	-3.12	-0.17

Smg5	chr3	88165183	88165423	-1.03	0.18
Rbm8a	chr3	96433850	96433910	-0.48	0.21
Bcas2	chr3	102982305	102982484	-1.47	0.15
Prcc	chr3	87662854	87664130	-1.39	-0.16
Chtop	chr3	90303623	90304012	-0.38	0.01
Chtop	chr3	90313185	90313365	-0.85	-0.09
Psmb4	chr3	94690551	94690880	-2.31	-0.17
Wdr3	chr3	99942341	99942760	-3.24	0.23
Ddx20	chr3	105481379	105482396	-0.40	-0.02
Tgs1	chr4	3542039	3542248	-1.77	-0.35
Rngtt	chr4	33587224	33587673	-1.48	0.31
Rngtt	chr4	33588240	33588330	-0.44	0.40
Prpf4	chr4	62083697	62085481	-2.54	0.23
Tut4	chr4	108151647	108159167	-0.67	0.06
Tut4	chr4	108185594	108190009	-2.37	0.34
Ebna1bp2	chr4	118293433	118293524	-2.73	-0.62
Lsm10	chr4	125773896	125775828	-0.48	0.04
Dnajc8	chr4	132108975	132109154	-0.70	-0.07
Syf2	chr4	134493303	134493452	-1.58	-0.40
Hnrnpr	chr4	135895398	135895996	-0.45	0.04
Exosc10	chr4	147932535	147933683	-0.62	0.12
Nol9	chr4	151431914	151434091	-3.59	-0.15
Nol6	chr4	41070633	41071312	-0.31	-0.01
Tex10	chr4	48481183	48482144	-1.03	0.09
Ppih	chr4	118979838	118980467	-0.40	0.06
Srrm1	chr4	134878175	134878385	-1.96	-0.19
Srrm1	chr4	134902739	134909069	-0.74	0.09
Nupl2	chr5	23688056	23689616	-0.80	0.11
Utp3	chr5	88983507	88985078	-1.24	0.05
Psmd9	chr5	123678228	123678437	-1.01	0.20
Snrnp35	chr5	124933163	124940954	-0.80	-0.10
Gtf2h3	chr5	125046222	125046283	-0.86	-0.51
Bud31	chr5	145908765	145908943	-0.74	-0.11
Cpsf4	chr5	145939775	145942670	-0.46	0.04
Pan3	chr5	148242155	148242515	-0.41	-0.10
Pan3	chr5	148357203	148359090	-1.21	0.09
Slbp	chr5	33992455	33994955	-2.31	0.29
Nop14	chr5	35000629	35002797	-2.47	-0.24
Nup54	chr5	92845101	92846543	-0.74	0.09
Ubc	chr5	125867967	125870328	-2.39	0.10
Dhx37	chr5	125894892	125895712	-0.73	0.09
Nup205	chr6	35127615	35133895	-0.46	0.13
Hnrnpf	chr6	117850506	117856962	-1.09	-0.18

Hnrnpf	chr6	117873756	117874265	-2.10	-0.15
Ddx47	chr6	134971525	134973794	-2.24	-0.15
Gm8994	chr6	136279021	136279082	-0.20	-0.85
Gm8994	chr6	136279763	136279882	-0.28	0.95
Cnot4	chr6	34973352	34974287	-0.81	0.20
Cnot4	chr6	34996066	35002891	-0.64	-0.06
Hnrnpa2b1	chr6	51419713	51419893	-0.88	-0.08
Usp39	chr6	72268788	72269207	-3.44	0.38
Isy1	chr6	87769040	87769634	-1.43	-0.23
Nup210	chr6	90963060	90963630	-0.69	-0.12
Bms1	chr6	118333488	118334272	-1.79	-0.15
Apobec1	chr6	122528673	122530724	-0.93	0.08
Apobec1	chr6	122530782	122531627	-0.81	-0.07
Prpf31	chr7	3581586	3582328	-3.35	-0.27
U2af2	chr7	5013783	5013844	-0.45	0.13
U2af2	chr7	5030853	5031123	-0.52	-0.04
Snrpd2	chr7	19735186	19736653	-0.98	-0.16
Ercc2	chr7	19979500	19981041	-0.83	-0.08
Polr2i	chr7	31017332	31017529	-0.50	-0.12
Nup62	chr7	52071739	52086176	-1.81	-0.05
Gemin7	chr7	20150297	20158692	-1.99	0.12
Zfp473	chr7	51987570	51988080	-0.82	0.69
Zfp473	chr7	51988469	51988860	-0.49	-0.50
Mphosph10	chr7	71534463	71537122	-0.51	0.03
Pcf11	chr7	99801691	99808380	-0.63	-0.03
Nup98	chr7	109268003	109269174	-0.67	-0.03
Sugp1	chr8	72566920	72572439	-0.31	-0.03
Exosc6	chr8	113580238	113581499	-0.45	0.02
Elavl1	chr8	4289159	4289430	-2.72	-0.30
Cnot7	chr8	41592731	41595397	-1.59	0.21
Ddx49	chr8	72817002	72817479	-2.75	-0.34
Nob1	chr8	109936418	109936954	-0.43	0.03
Dhx38	chr8	112071923	112073723	-0.63	0.08
Sf3b3	chr8	113334571	113334842	-0.99	0.06
Mphosph6	chr8	120316041	120316188	-0.39	-0.05
Imp3	chr9	56785306	56786205	-2.85	0.13
Snupn	chr9	56830617	56830946	-0.82	-0.21
Edc3	chr9	57563979	57575316	-0.29	0.00
Edc3	chr9	57596059	57596567	-0.45	0.05
Xrn1	chr9	95953088	95953208	-0.30	0.01
Rrp9	chr9	106379639	106379729	-0.79	0.39
Cnot10	chr9	114541870	114549228	-1.71	-0.34
Ppil4	chr10	7534516	7541105	-2.25	-0.26

Hbs1l	chr10	21087565	21088546	-0.87	-0.11
Ranbp2	chr10	57941534	57941955	-2.61	0.14
Wdr18	chr10	79422896	79423107	-2.05	-0.33
Sf3a2	chr10	80261479	80263960	-1.32	-0.33
Pan2	chr10	127740480	127741343	-1.63	-0.62
Pan2	chr10	127747269	127747819	-0.53	-0.12
Ccar1	chr10	62208074	62209806	-0.97	0.09
Adarb1	chr10	76757182	76758458	-1.66	-0.57
Adarb1	chr10	76784665	76785205	-0.71	0.23
Pwp2	chr10	77633893	77636365	-0.75	0.06
Polr2e	chr10	79501286	79502404	-1.51	-0.21
Utp20	chr10	88209351	88209801	-0.55	0.06
Cnot2	chr10	115922246	115922606	-0.97	0.18
Cnot2	chr10	115922874	115923115	-1.67	0.33
Pes1	chr11	3878878	3878968	-0.51	0.06
Sf3a1	chr11	4080296	4080657	-1.02	-0.15
Zmat5	chr11	4604673	4628589	-1.66	-0.52
Thoc5	chr11	4799846	4799907	-2.53	-0.61
Psme4	chr11	30671804	30671925	-0.83	-0.91
Psme4	chr11	30672014	30672135	-0.38	0.12
Snrnp25	chr11	32105414	32105652	-1.18	-0.36
Slu7	chr11	43247232	43250977	-0.69	-0.10
Hnrnph1	chr11	50191247	50191914	-2.25	0.25
Ppp2ca	chr11	51935449	51935836	-1.90	0.10
Cnot8	chr11	57930988	57931258	-1.37	0.34
Tsr1	chr11	74711581	74711923	-2.23	-0.39
Tsr1	chr11	74713687	74715018	-3.53	-0.25
Smg6	chr11	74739373	74744156	-0.45	0.03
Psmd11	chr11	80285544	80285635	-1.72	-0.32
Ddx52	chr11	83773028	83775812	-1.48	0.19
Ddx52	chr11	83776110	83776319	-0.39	0.07
Srsf1	chr11	87861172	87861352	-2.81	-0.34
Casc3	chr11	98692883	98694014	-1.36	-0.17
Psme3	chr11	101177564	101178336	-0.63	-0.07
Psme3	chr11	101182070	101183288	-1.70	-0.15
Pno1	chr11	17111355	17111592	-1.86	-0.23
Lsm11	chr11	45747117	45747446	-1.53	-0.39
Gemin5	chr11	57934607	57938883	-0.32	0.01
Pelp1	chr11	70206472	70208529	-1.91	-0.12
Nup88	chr11	70783414	70783475	-0.56	0.19
Cwc25	chr11	97608042	97614061	-2.33	0.21
Eftud2	chr11	102699785	102700145	-2.70	0.23
Nol11	chr11	107028583	107029031	-0.64	0.09

Nol11	chr11	107048232	107050606	-0.96	-0.22
Csnk1d	chr11	120819778	120820048	-1.21	-0.15
Csnk1d	chr11	120823779	120824080	-1.10	-0.14
Csnk1d	chr11	120852347	120852438	-1.28	-0.33
Psma6	chr12	56499811	56499871	-1.67	-0.32
Psmc1	chr12	101348377	101351286	-1.53	-0.29
Papola	chr12	107072945	107075506	-1.04	0.06
Zfp36l1	chr12	81210446	81214000	-2.03	-0.17
Akt1	chr12	113912217	113912487	-0.40	0.08
Riok1	chr13	38152704	38153063	-0.29	0.00
Ddx46	chr13	55736387	55736448	-1.12	-0.40
Smn1	chr13	100894809	100896580	-2.61	-0.55
Nup153	chr13	46776228	46776529	-1.65	0.22
Thoc3	chr13	54561210	54561569	-0.37	0.03
Hnrnpk	chr13	58501349	58504674	-0.47	-0.03
Tut7	chr13	59873508	59883679	-1.92	0.17
Tut7	chr13	59900461	59902000	-1.87	0.14
Tut7	chr13	59918167	59921566	-0.48	0.10
Utp15	chr13	99018475	99020783	-1.22	0.09
Ppwd1	chr13	105018773	105018924	-0.38	0.08
Rpp14	chr14	8922458	8922934	-1.05	0.14
Dcp1a	chr14	31336008	31337397	-1.47	-0.14
Dcp1a	chr14	31339492	31339853	-0.61	-0.12
Psmc6	chr14	45949498	45950514	-0.68	0.11
Prkcd	chr14	31408778	31411699	-1.32	-0.09
Prkcd	chr14	31411787	31411937	-0.35	-0.04
Prkcd	chr14	31439303	31439394	-1.67	-0.55
Gnl3	chr14	31830581	31832275	-0.48	-0.03
Gtf2f2	chr14	76297012	76306621	-0.38	0.02
Dis3	chr14	99476449	99476599	-1.45	0.55
Dcaf13	chr15	38944419	38944568	-0.30	-0.01
Exosc4	chr15	76159579	76160266	-2.99	-0.34
Nup50	chr15	84757805	84765852	-2.65	-0.15
Nup50	chr15	84770131	84771000	-1.89	-0.14
Eif4b	chr15	101925104	101925897	-3.00	-0.10
Puf60	chr15	75900611	75900909	-0.74	-0.07
Bop1	chr15	76305282	76307699	-2.55	-0.31
Csnk1e	chr15	79272227	79272487	-0.29	-0.14
Eif4g1	chr16	20679306	20680085	-2.29	0.06
Ncbp2	chr16	31957240	31957450	-1.88	-0.32
Fyttd1	chr16	32877869	32878080	-0.83	-0.18
Fyttd1	chr16	32902586	32905812	-1.29	-0.23
Tra2b	chr16	22265822	22266002	-1.45	-0.29

Srrm2	chr17	23957516	23958056	-0.44	-0.01
Rnps1	chr17	24551619	24552103	-0.56	-0.11
Wdr46	chr17	34077667	34077865	-2.62	-0.51
Psmb8	chr17	34335288	34335468	-2.26	-0.35
Ddx39b	chr17	35378720	35378917	-2.01	-0.34
Ddx39b	chr17	35566011	35566699	-0.29	-0.01
Wdr43	chr17	71965588	71965738	-0.45	-0.07
Wdr43	chr17	72008040	72008371	-0.41	-0.04
Gemin6	chr17	80625068	80627484	-0.34	0.03
Thoc6	chr17	23810678	23810831	-0.28	-0.01
Tbl3	chr17	24843061	24844598	-1.28	-0.31
Ppil1	chr17	29387927	29388668	-2.42	-0.27
Hnrnpm	chr17	33786662	33789227	-3.10	-0.13
Hnrnpm	chr17	33814217	33822403	-0.43	0.02
Hspa1a	chr17	35107196	35107464	-1.02	3.56
Hspa1a	chr17	35108505	35108744	-1.36	5.33
Cdc5l	chr17	45528987	45530093	-0.78	-0.11
Cdc5l	chr17	45564979	45570477	-1.28	-0.56
Rrp36	chr17	46804404	46807115	-1.90	-0.20
Rrp36	chr17	46809705	46811204	-0.37	-0.05
Khsrp	chr17	57170601	57170870	-2.51	-0.35
Srsf7	chr17	80606178	80606645	-0.44	-0.07
Thoc1	chr18	9993386	9993537	-0.43	0.05
Snrpd1	chr18	10617823	10623647	-0.37	0.04
Riok3	chr18	12313911	12313972	-0.43	-0.08
Polr2d	chr18	31955847	31956355	-3.53	0.44
Wdr33	chr18	32049204	32067143	-0.39	0.01
Ercc3	chr18	32399984	32400342	-0.44	-0.08
Ercc3	chr18	32429532	32429682	-0.45	0.08
Dcp2	chr18	44572199	44578164	-2.58	0.22
Phax	chr18	56732606	56746980	-0.52	0.02
Rnmt	chr18	68460038	68465632	-0.35	0.01
Txnl4a	chr18	80403957	80409422	-1.96	0.06
Txnl4a	chr18	80415452	80419332	-0.42	-0.02
Txnl4a	chr18	80421035	80421873	-1.14	-0.40
Tmem223	chr19	8846725	8846845	-2.14	1.40
Cpsf7	chr19	10614163	10620757	-1.15	-0.06
Prpf19	chr19	10969781	10969872	-1.39	-0.35
Rcl1	chr19	29208971	29218299	-1.54	0.14
Pdcd11	chr19	47165255	47167320	-1.83	-0.43
Pdcd11	chr19	47186632	47191796	-3.08	-0.17
Pdcd11	chr19	47203460	47203640	-1.48	-0.58
Sart1	chr19	5381212	5381698	-0.52	-0.07

Polr2g	chr19	8872986	8873047	-0.42	-0.12
Smndc1	chr19	53454959	53455080	-0.47	0.05
Gpkow	chrX	7285271	7285480	-3.00	0.59
Cstf2	chrX	130609104	130609225	-0.88	0.22
Gemin8	chrX	162618344	162627493	-2.26	-0.77
Upf3b	chrX	34632248	34635529	-1.00	-0.19
Upf3b	chrX	34635618	34635799	-3.11	0.64
Zrsr2	chrX	160374663	160374874	-0.55	-0.14
Zrsr2	chrX	160396297	160396568	-0.44	-0.07

Innate immune system

gene_name	chr	chromStart	chromEnd	diff.lg.p	diff.log2.fc
Casp8	chr1	58890568	58901676	-0.30	0.00
Abi2	chr1	60527596	60534244	-1.45	0.18
Creb1	chr1	64622786	64644320	-12.70	0.59
Slc11a1	chr1	74421776	74422382	-3.48	-0.58
Slc11a1	chr1	74432056	74432625	-0.70	0.06
Cab39	chr1	87690021	87715038	-1.14	-0.12
Cab39	chr1	87745789	87746418	-1.09	-0.07
Psmd1	chr1	87961193	87961254	-0.37	-0.11
Arl8a	chr1	137043410	137043590	-2.14	-0.35
Arpc5	chr1	154613671	154613968	-7.60	-0.29
Ncf2	chr1	154654984	154655314	-0.34	0.01
Creg1	chr1	167693910	167694001	-0.45	-0.10
Aim2	chr1	175389828	175395719	-6.98	0.20
Tmem63a	chr1	182904339	182904638	-1.16	-0.26
Tmbim1	chr1	74335180	74335241	-2.07	-0.51
Tmbim1	chr1	74341896	74350760	-11.80	-0.96
Bcl2	chr1	108438384	108439674	-0.90	0.22
Bcl2	chr1	108439763	108608408	-0.87	0.54
Bcl2	chr1	108608737	108610507	-1.27	-0.20
Bcl2	chr1	108610626	108610867	-0.58	0.39
Ptpn4	chr1	121555598	121572143	-7.36	0.52
Ptpn4	chr1	121733557	121733648	-0.97	-0.74
Actr3	chr1	127331982	127332265	-4.20	-0.39
Ikbke	chr1	133151178	133153534	-2.21	-0.20
Ikbke	chr1	133172403	133173518	-4.46	-0.66
Dhx9	chr1	155303276	155303397	-3.17	0.68
Qsox1	chr1	157626448	157628411	-2.42	-0.35
Fcgr2b	chr1	172890688	172891165	-4.70	0.21
Fcgr2b	chr1	172906081	172906200	-0.31	0.02
Fcgr3	chr1	172981537	172981597	-1.79	-0.50
Ncstn	chr1	173996440	173996917	-6.01	-0.35
Degs1	chr1	184206857	184208760	-3.23	0.22

Degs1	chr1	184209059	184209748	-12.40	0.28
Commd3	chr2	18596461	18597638	-0.32	-0.01
Abl1	chr2	31544075	31551320	-1.18	-0.13
Abl1	chr2	31653102	31658129	-1.05	-0.08
Ptges2	chr2	32257786	32257964	-3.07	-0.78
Tank	chr2	61416642	61431255	-0.74	-0.21
Tank	chr2	61482442	61484290	-0.30	-0.01
Tank	chr2	61484409	61488467	-1.76	0.12
Tank	chr2	61491449	61491629	-0.53	0.07
Itgav	chr2	83564583	83565003	-11.70	-0.93
Itgav	chr2	83643447	83643718	-4.06	-0.49
Itgav	chr2	83643777	83643958	-1.72	-0.33
Psmc3	chr2	90894439	90895182	-2.45	-0.16
Traf6	chr2	101531587	101537959	-2.12	0.17
B2m	chr2	121973422	121973632	-3.80	-0.13
Sirpa	chr2	129418574	129418993	-2.78	0.40
Sirpa	chr2	129419320	129457247	-0.96	-0.02
Mavs	chr2	131068979	131072532	-4.44	-0.40
Pygb	chr2	150654268	150656555	-0.44	0.05
Hck	chr2	152934263	152934443	-1.07	0.34
Hck	chr2	152976907	152977087	-0.51	-0.07
Itch	chr2	154959216	154959307	-0.30	0.07
Itch	chr2	155050132	155050403	-0.62	-0.11
Src	chr2	157295196	157296561	-1.97	0.63
Plcg1	chr2	160587522	160588203	-4.39	-0.75
Ctsa	chr2	164659287	164659646	-8.35	-0.27
Mmp9	chr2	164780329	164780659	-0.85	-0.58
Abi1	chr2	22895609	22895730	-1.85	-0.40
Tubb4b	chr2	25077855	25080192	-0.38	-0.01
Dpp7	chr2	25207809	25207987	-3.64	0.45
Dpp7	chr2	25211792	25211852	-1.75	0.43
Card9	chr2	26212789	26213529	-0.79	0.09
Agpat2	chr2	26449106	26450082	-0.51	-0.12
Surf4	chr2	26777259	26777678	-5.01	-0.17
Surf4	chr2	26782443	26788971	-5.66	-0.47
Vav2	chr2	27282074	27282345	-1.98	-1.07
Psmd5	chr2	34707993	34709823	-0.82	-0.10
Psmd5	chr2	34726274	34726482	-3.28	-0.70
Rab14	chr2	35056341	35056550	-13.00	-0.63
Psmb7	chr2	38498946	38499426	-1.70	-0.24
Wipf1	chr2	73270330	73270480	-0.29	0.00
Wipf1	chr2	73273054	73275208	-7.06	-0.48
Wipf1	chr2	73275357	73278120	-3.10	-0.24

Wipf1	chr2	73282586	73291856	-0.45	0.07
Atf2	chr2	73656977	73657038	-1.83	0.77
Ptprj	chr2	90273053	90274369	-19.20	-0.53
Ptprj	chr2	90420444	90420774	-0.85	-0.18
Cd44	chr2	102654086	102662407	-79.70	-0.56
Atp8b4	chr2	126148354	126148594	-0.41	0.05
Il1b	chr2	129190434	129191002	-4.06	2.30
Il1b	chr2	129195984	129196856	-1.15	2.95
Cd93	chr2	148263313	148263433	-0.69	4.00
Cd93	chr2	148266721	148267597	-0.52	1.75
Cd93	chr2	148267925	148268195	-0.71	3.50
Cd93	chr2	148268851	148269062	-0.38	1.48
Psmf1	chr2	151543679	151544068	-0.32	0.02
Bcl2l1	chr2	152606912	152607242	-0.45	0.04
Bcl2l1	chr2	152607660	152653984	-27.50	-0.71
Bcl2l1	chr2	152655121	152656044	-111.00	-0.82
Bcl2l1	chr2	152656237	152657418	-2.87	-0.78
Cpne1	chr2	155922158	155937671	-0.31	0.00
Dsn1	chr2	156821512	156821930	-1.54	-0.43
Slpi	chr2	164181290	164182042	-96.20	-1.36
Elmo2	chr2	165115177	165116717	-2.78	-0.28
Elmo2	chr2	165117584	165117675	-2.59	-1.26
Nfatc2	chr2	168301999	168303229	-1.92	-0.20
Nfatc2	chr2	168304128	168330367	-7.02	-0.30
Nfatc2	chr2	168396317	168427005	-7.34	-0.40
Ctsz	chr2	174252994	174254182	-52.90	-0.22
Ctsz	chr2	174264374	174264434	-4.27	-0.36
Fabp5	chr3	10015061	10015506	-2.68	-0.28
Pld1	chr3	28029315	28031326	-1.86	-0.40
Pld1	chr3	28031774	28031835	-0.53	-0.67
Pik3ca	chr3	32335072	32335372	-0.45	-0.08
Pik3ca	chr3	32360681	32362022	-3.50	0.35
Rap2b	chr3	61168428	61170347	-12.00	0.31
Rap2b	chr3	61171125	61171306	-0.74	-0.36
Shc1	chr3	89222472	89225594	-0.41	-0.06
Shc1	chr3	89225712	89226222	-14.10	-1.02
Shc1	chr3	89232244	89232484	-0.39	0.03
Ilf2	chr3	90291521	90291822	-1.69	-0.21
Ctss	chr3	95330707	95333452	-1.96	-0.10
Txnip	chr3	96362296	96362849	-42.00	0.57
Txnip	chr3	96363879	96364976	-345.00	0.76
Txnip	chr3	96365273	96365601	-111.00	1.13
Vav3	chr3	109143660	109143900	-1.03	-0.68

Ube2d3	chr3	135101656	135102228	-6.45	-0.41
Manba	chr3	135230676	135233888	-0.32	0.01
Bcl10	chr3	145587262	145587592	-1.35	-0.17
Bcl10	chr3	145588099	145593586	-0.30	0.00
Nhlrc3	chr3	53255917	53257521	-3.38	-0.42
Dhx36	chr3	62274511	62275295	-0.82	-0.16
Tlr2	chr3	83640193	83645500	-1.21	0.04
Lamtor2	chr3	88353884	88356849	-9.91	-0.33
Psmb4	chr3	94690551	94690880	-2.31	-0.17
Psmd4	chr3	94836626	94836894	-5.68	-0.27
Psmd4	chr3	94839816	94846467	-4.83	-0.24
Fcgr1	chr3	96087757	96088506	-3.70	-0.43
Cd53	chr3	106563447	106563716	-7.16	0.21
Nfkb1	chr3	135248217	135252299	-9.16	-0.42
Nfkb1	chr3	135354210	135354481	-4.75	1.80
Prkacb	chr3	146394723	146394993	-0.71	0.12
Lyn	chr4	3605267	3605506	-17.80	-0.52
Lyn	chr4	3716911	3718014	-9.19	-0.18
Map3k7	chr4	32051081	32051232	-1.86	-0.83
Map3k7	chr4	32104109	32107267	-4.75	0.31
Map3k7	chr4	32110141	32110231	-1.49	-0.76
Atp6v1g1	chr4	63205828	63209679	-0.73	0.11
Trim32	chr4	65274556	65274646	-0.66	-0.27
Trim32	chr4	65274824	65276139	-4.40	0.35
Tlr4	chr4	66494995	66502966	-7.48	0.33
Pgm2	chr4	99602085	99602234	-1.37	-0.46
Pgm2	chr4	99659304	99659542	-4.78	-0.71
Ptafr	chr4	132119981	132136781	-0.79	0.06
Fgr	chr4	132530039	132542068	-0.98	0.34
Fgr	chr4	132556096	132556514	-7.15	-0.57
Fgr	chr4	132557289	132557469	-2.85	0.87
Wasf2	chr4	132751642	132752809	-3.91	-0.14
Fuca1	chr4	135476640	135476908	-9.09	-0.79
Fuca1	chr4	135495027	135495325	-1.89	0.27
Ddost	chr4	137868046	137868406	-0.32	-0.01
Casp9	chr4	141352424	141352694	-2.96	-0.57
Casp9	chr4	141368153	141369672	-0.74	0.33
Casp9	chr4	141369761	141369941	-0.56	1.17
Slc2a5	chr4	149494002	149494152	-0.29	1.46
Ripk2	chr4	16050700	16051621	-9.33	-0.60
Ripk2	chr4	16090465	16090645	-12.00	-1.26
Ddx58	chr4	40152603	40155517	-1.09	-0.14
Ddx58	chr4	40186678	40186858	-0.63	0.23

B4galt1	chr4	40753786	40754830	-2.74	0.20
B4galt1	chr4	40770597	40800583	-1.25	0.19
B4galt1	chr4	40800821	40800971	-2.38	-0.70
Vcp	chr4	43013259	43013349	-0.47	-0.15
Alad	chr4	62170203	62171204	-0.29	0.00
Jun	chr4	94716292	94718913	-1.94	0.04
Cap1	chr4	122536500	122536680	-41.90	-0.54
Lck	chr4	129225826	129225977	-1.80	-0.75
Rps6ka1	chr4	133403354	133404010	-1.09	-0.10
Tnfrsf1b	chr4	144803977	144809673	-187.00	-0.87
Tnfrsf1b	chr4	144818945	144836773	-24.30	-1.26
Tnfrsf1b	chr4	144836594	144836774	-3.62	-0.69
Kcnab2	chr4	151766373	151767149	-2.14	-0.15
Atad3a	chr4	155114748	155115286	-0.82	-0.07
Bst1	chr5	44210161	44210281	-0.88	-0.46
Cxcl3	chr5	91215158	91215370	-0.42	-0.69
Cxcl2	chr5	91332924	91333518	-434.00	-2.15
Stbd1	chr5	93034080	93034200	-0.12	0.00
Hvcn1	chr5	122690237	122692276	-1.11	-0.20
P2rx7	chr5	123093919	123094010	-4.98	1.77
P2rx7	chr5	123130997	123132855	-10.40	0.31
P2rx7	chr5	123133124	123133994	-8.79	0.52
Psmd9	chr5	123678228	123678437	-1.01	0.20
Atp6v0a2	chr5	125200361	125202517	-1.22	0.17
Arpc1b	chr5	145875124	145883438	-3.09	-0.16
Tnip2	chr5	34839311	34839491	-0.39	0.07
Tnip2	chr5	34842145	34842355	-1.03	0.23
Tlr1	chr5	65316072	65316462	-1.84	0.58
Tlr1	chr5	65317358	65317538	-0.27	0.02
Tlr1	chr5	65318106	65318495	-0.63	0.23
Tlr6	chr5	65344333	65351228	-28.20	0.94
Atp8a1	chr5	68013273	68013783	-1.25	0.26
Tec	chr5	73147081	73148633	-1.81	-0.24
Tec	chr5	73214842	73259687	-5.06	0.82
Mlec	chr5	115597834	115600228	-0.81	-0.07
Mlec	chr5	115600927	115608185	-3.72	-0.22
Ptpn11	chr5	121581827	121582307	-1.65	0.20
Ptpn11	chr5	121583292	121587519	-2.53	-0.16
Rhof	chr5	123553529	123554067	-7.00	2.05
Rhof	chr5	123567437	123567766	-1.20	-0.45
Rhof	chr5	123568123	123582399	-23.20	-0.42
Ubc	chr5	125866482	125866751	-7.45	-0.16
Ubc	chr5	125867492	125867701	-3.77	1.98

Ubc	chr5	125867967	125870328	-2.39	0.10
Slc15a4	chr5	128076540	128080872	-0.31	0.00
Gusb	chr5	130464890	130465519	-2.75	0.10
Gusb	chr5	130478517	130478698	-4.28	-0.24
Ncf1	chr5	134697332	134697663	-8.46	-0.28
Lat2	chr5	135076464	135078531	-5.64	-0.19
Trim56	chr5	137587155	137587605	-10.60	-0.53
Trim56	chr5	137587784	137588144	-0.80	-0.16
Trim56	chr5	137588275	137588336	-0.64	0.56
Trim56	chr5	137588635	137592077	-28.50	-0.43
Card11	chr5	141348952	141350301	-2.19	-0.58
Rac1	chr5	144288651	144288861	-1.33	0.22
Pdap1	chr5	145892323	145900958	-18.10	-0.36
Atp6v1f	chr6	29417782	29417841	-1.46	-0.23
Cul1	chr6	47404345	47445165	-12.40	0.63
Cul1	chr6	47474617	47476108	-2.74	-0.21
Arpc4	chr6	113328136	113330743	-0.46	0.06
Irak2	chr6	113640953	113644510	-13.00	-0.41
Clec4d	chr6	123212154	123212273	-2.02	0.39
C1ra	chr6	124472382	124472951	-1.99	-0.43
C1rb	chr6	124530824	124530943	-0.36	0.74
Lrmp	chr6	145086263	145086444	-0.85	-0.32
Impdh1	chr6	29162071	29162252	-2.44	-0.43
Clec5a	chr6	40527283	40529441	-0.30	0.00
Nod1	chr6	54893317	54893527	-0.62	1.79
Nod1	chr6	54894659	54894899	-0.40	-0.75
Vamp8	chr6	72335214	72335477	-0.41	-0.03
Vamp8	chr6	72338403	72340632	-1.83	-0.34
Kcmf1	chr6	72849172	72849233	-0.31	-0.52
Kcmf1	chr6	72849822	72849973	-0.27	0.15
Rbsn	chr6	92139135	92140425	-0.38	0.02
Raf1	chr6	115568590	115569280	-0.54	-0.05
Alox5	chr6	116410986	116411166	-1.01	-0.30
Atp6v1e1	chr6	120768397	120772703	-4.00	-0.38
Slc2a3	chr6	122679681	122680131	-0.97	-0.19
C3ar1	chr6	122799485	122806145	-5.99	-0.19
C1s1	chr6	124481017	124481078	-0.14	-0.42
C1s1	chr6	124481405	124481466	-0.11	0.00
C1s2	chr6	124575330	124575809	-0.33	1.51
Ptpn6	chr6	124683045	124688519	-0.44	0.09
Olr1	chr6	129436974	129437604	-0.52	1.98
Kras	chr6	145168813	145168964	-1.25	-0.35
Plaur	chr7	25247518	25251724	-91.60	-1.63

Plaur	chr7	25260294	25260713	-133.00	-1.32
Tyrobp	chr7	31198806	31198866	-0.60	-0.05
Irf3	chr7	52253029	52253209	-0.42	-0.09
Irf3	chr7	52257067	52257801	-2.15	-0.33
Cyfip1	chr7	63109393	63127258	-0.43	-0.06
Ctsc	chr7	95437274	95437455	-0.26	0.00
Ctsc	chr7	95458037	95458487	-2.08	0.25
Prcp	chr7	100081684	100082313	-0.64	-0.10
Ndufc2	chr7	104548571	104548690	-0.85	0.14
Lamtor1	chr7	109054380	109058241	-0.51	-0.09
Lamtor1	chr7	109059912	109060091	-7.00	-0.38
Ampd3	chr7	117954502	117954682	-3.55	0.65
Itgam	chr7	135270356	135270776	-10.60	1.52
Itgax	chr7	135292687	135294081	-3.59	1.65
Lair1	chr7	3961115	3961265	-1.04	-0.76
Ube2m	chr7	13623108	13623619	-2.32	-0.37
C5ar2	chr7	16822087	16822178	-0.28	-0.26
C5ar2	chr7	16822715	16822896	-1.32	-1.43
C5ar2	chr7	16823104	16823255	-0.32	0.53
C5ar1	chr7	16833166	16844566	-34.20	-0.54
Calm3	chr7	17502039	17502309	-2.51	-0.14
Relb	chr7	20191570	20192258	-0.92	-0.11
Ceacam1	chr7	26247769	26248608	-3.38	0.29
Ceacam1	chr7	26256984	26258859	-1.12	-0.38
Ceacam1	chr7	26259008	26274241	-1.35	0.18
Nfkbib	chr7	29546684	29546893	-27.50	-0.92
Nfkbib	chr7	29547132	29551543	-9.72	-0.61
Psmd8	chr7	29965393	29965573	-6.44	-0.28
Gpi1	chr7	34987212	34987689	-18.10	-0.27
Siglece	chr7	50915321	50915471	-0.80	0.91
Ftl1	chr7	52715051	52715256	-402.00	-0.43
Anpep	chr7	86966748	86966927	-0.81	-0.13
Anpep	chr7	86986085	86993080	-3.53	-0.12
Iqgap1	chr7	87857938	87858329	-7.34	-0.18
Iqgap1	chr7	87858478	87858659	-1.28	-0.06
Iqgap1	chr7	87944725	87948187	-32.40	-0.56
Rhog	chr7	109387636	109398632	-17.50	-0.20
Trim21	chr7	109707151	109708560	-3.22	0.40
Adam8	chr7	147164899	147165378	-38.70	-0.54
Hras	chr7	148376758	148378177	-0.33	-0.01
Hras	chr7	148378805	148379903	-1.48	0.51
Tollip	chr7	149069998	149075492	-4.83	-0.28
Ctsd	chr7	149561820	149562294	-47.90	-0.19

Fadd	chr7	151765572	151768221	-1.92	-0.22
Map2k7	chr8	4245576	4246251	-2.64	-0.23
Atp11a	chr8	12757015	12757255	-0.36	0.24
Atp11a	chr8	12864987	12866454	-1.34	0.14
Lamp1	chr8	13159223	13159402	-1.74	-0.18
Plpp5	chr8	26834581	26835060	-0.38	-0.03
Ppp2cb	chr8	34710092	34710330	-0.63	-0.43
Dusp4	chr8	35870663	35882259	-110.00	-0.60
Myo9b	chr8	73796612	73839062	-0.95	0.06
Myo9b	chr8	73871734	73876118	-11.40	-0.25
Myo9b	chr8	73882022	73884611	-1.43	0.07
Prkaca	chr8	86496876	86500855	-3.14	-0.78
Man2b1	chr8	87607167	87607377	-1.30	-0.24
Cyld	chr8	91220956	91221047	-0.33	-0.20
Cyld	chr8	91226307	91233793	-8.51	0.40
Nfatc3	chr8	108602601	108603618	-1.85	0.13
Nfatc3	chr8	108632465	108652166	-1.29	0.10
Plcg2	chr8	120022190	120028105	-0.37	-0.04
Ikbkb	chr8	23770546	23770997	-0.43	-0.04
Golga7	chr8	24352876	24353057	-1.98	-0.24
Hgsnat	chr8	27054960	27055586	-11.20	0.64
Hgsnat	chr8	27083350	27087156	-0.46	0.09
Tlr3	chr8	46482427	46484485	-1.03	0.28
Pik3r2	chr8	73292079	73292528	-3.00	-0.41
Pik3r2	chr8	73298580	73300551	-1.20	-0.17
Adgre5	chr8	86247268	86247657	-11.70	-1.33
Psmd7	chr8	110104309	110108107	-19.20	0.30
Hp	chr8	112099087	112099805	-2.48	-0.74
Ist1	chr8	112195759	112196600	-7.45	0.33
Cotl1	chr8	122364299	122364479	-1.03	0.15
Galns	chr8	125102315	125103030	-1.46	-0.23
Casp1	chr9	5298664	5299458	-1.72	0.32
Pin1	chr9	20456573	20459888	-1.84	-0.36
Slc44a2	chr9	21156935	21159322	-7.12	0.27
Dnm2	chr9	21310781	21311349	-0.34	0.01
Dnm2	chr9	21311898	21312198	-3.13	0.36
Ppp2r1b	chr9	50688020	50688710	-0.53	0.05
Ppp2r1b	chr9	50701016	50701675	-1.83	-0.33
Pstpip1	chr9	55937782	55937902	-1.36	-0.40
Adam10	chr9	70526807	70526958	-0.76	0.19
Adam10	chr9	70626086	70626267	-1.49	0.28
Rab27a	chr9	72892731	72908786	-0.70	-1.27
Rab27a	chr9	72943119	72943840	-0.62	-0.22

Myo5a	chr9	74919042	74919403	-0.47	0.08
Myo5a	chr9	75065709	75066579	-14.50	0.35
Pik3r4	chr9	105546522	105551238	-3.59	0.32
Pik3r4	chr9	105552647	105556359	-0.68	0.10
Pik3r4	chr9	105589445	105589895	-1.34	0.26
Tlr9	chr9	106124928	106129177	-57.50	1.05
Dusp7	chr9	106270962	106271201	-1.93	-1.11
Dusp7	chr9	106271586	106276865	-0.38	-0.01
Nckipsd	chr9	108714765	108714915	-1.56	-0.69
Glb1	chr9	114310242	114326084	-1.04	0.28
Glb1	chr9	114368770	114373539	-0.71	0.08
Glb1	chr9	114374377	114383495	-16.40	0.68
Dync1li1	chr9	114632686	114632895	-0.48	0.07
Dync1li1	chr9	114632812	114633189	-3.66	0.29
Ctnnb1	chr9	120842577	120859351	-2.42	-0.18
Ctnnb1	chr9	120866971	120868877	-19.00	0.26
Ctnnb1	chr9	120868936	120868997	-1.38	0.13
Ccr2	chr9	124021097	124021367	-1.86	-0.98
Birc2	chr9	7818494	7818943	-1.37	0.33
Birc2	chr9	7833550	7834657	-8.57	0.55
Birc2	chr9	7834955	7835225	-0.88	0.29
Birc3	chr9	7848939	7850928	-0.65	0.09
Birc3	chr9	7860281	7872961	-1.11	0.10
Panx1	chr9	14810228	14812439	-0.40	-0.04
Rab3d	chr9	21714741	21715012	-4.69	-1.31
Tirap	chr9	34994185	34994305	-0.27	0.01
Tirap	chr9	34994483	34996852	-1.16	0.12
Nlrx1	chr9	44060795	44061574	-0.93	-0.29
Nlrx1	chr9	44064954	44068731	-0.40	-0.37
Nlrx1	chr9	44070466	44072299	-0.30	-0.01
Pafah1b2	chr9	45776623	45777013	-5.68	0.31
Chrnb4	chr9	54883115	54883236	-0.29	1.46
Tmem30a	chr9	79617076	79617405	-1.31	0.16
Pik3cb	chr9	98939598	98941447	-3.91	0.26
Armc8	chr9	99380819	99381090	-0.59	0.18
Armc8	chr9	99420671	99420882	-2.06	0.72
Nck1	chr9	100395689	100407191	-3.91	0.25
Dnajc13	chr9	104054165	104054614	-3.68	0.31
Apeh	chr9	107987744	107987894	-0.37	-0.08
Myd88	chr9	119248949	119249158	-0.83	-0.18
Fuca2	chr10	13222977	13225667	-1.46	0.28
Fuca2	chr10	13234515	13235470	-8.82	0.38
Frk	chr10	34203714	34203864	-0.26	0.90

Frk	chr10	34327775	34328373	-0.21	-0.81
Fyn	chr10	39089604	39089964	-2.08	-0.38
Fyn	chr10	39271197	39284162	-0.31	0.00
Lilr4b	chr10	51200478	51200715	-7.27	-0.46
Lilr4b	chr10	51205082	51205501	-207.00	-1.21
Psap	chr10	59740405	59754400	-0.37	-0.02
Itgb2	chr10	76993092	77005145	-2.27	-0.27
Cdc34	chr10	79144939	79145322	-7.32	-0.60
Cdc34	chr10	79148043	79151143	-8.59	-0.24
Arhgap45	chr10	79479416	79480812	-10.80	-0.44
Arhgap45	chr10	79485025	79488219	-1.43	-0.13
Arhgap45	chr10	79493111	79494216	-2.35	-0.14
Eef2	chr10	80644273	80645254	-1.14	-0.02
Eea1	chr10	95473536	95474748	-15.20	-0.62
Eea1	chr10	95488768	95489708	-4.51	-0.29
Eea1	chr10	95499325	95499386	-0.41	-0.05
Eea1	chr10	95502273	95504101	-1.18	-0.12
Eea1	chr10	95504160	95504884	-0.30	0.01
Dusp6	chr10	98725864	98729379	-7.49	0.29
Cep290	chr10	100034774	100035631	-1.17	0.53
Gns	chr10	120802145	120802355	-0.34	0.03
Gns	chr10	120832206	120832746	-2.71	0.13
Gns	chr10	120833971	120834301	-7.42	0.38
Arhgap9	chr10	126759908	126761407	-1.07	0.40
Stat6	chr10	127080041	127080222	-2.27	-0.32
Stat6	chr10	127096737	127097564	-1.15	-0.06
Tab2	chr10	7625983	7626463	-0.30	0.00
Tab2	chr10	7626701	7629919	-0.81	-0.05
Tab2	chr10	7630969	7644574	-2.87	-0.09
Tnfaip3	chr10	18721581	18725108	-0.69	-0.06
Ube2d1	chr10	70718174	70718562	-1.17	0.19
Ckap4	chr10	83990001	83991399	-0.32	0.00
Ckap4	chr10	83996275	83996633	-1.47	-0.37
Hsp90b1	chr10	86168098	86168159	-17.40	-0.72
Glipr1	chr10	111434115	111434291	-3.73	0.43
Cand1	chr10	118647870	118648919	-5.77	0.23
Dbnl	chr11	5688485	5691845	-3.37	-0.49
Dbnl	chr11	5699963	5700503	-8.51	-0.43
Peli1	chr11	20991326	20991536	-0.55	-0.13
Peli1	chr11	21047425	21048774	-5.67	-0.32
Psme4	chr11	30671804	30671925	-0.83	-0.91
Psme4	chr11	30672014	30672135	-0.38	0.12
Stk10	chr11	32433265	32433386	-0.63	0.22

Stk10	chr11	32489401	32503691	-28.60	-0.48
Stk10	chr11	32504289	32512777	-6.57	-0.53
Stk10	chr11	32517862	32522921	-14.90	-0.45
Fbxw11	chr11	32644536	32645016	-2.21	0.20
Mapk9	chr11	49696697	49697026	-1.50	0.25
Ppp2ca	chr11	51912325	51912474	-3.96	-0.95
Ppp2ca	chr11	51935449	51935836	-1.90	0.10
Gm2a	chr11	54923031	54923210	-0.87	-0.15
2310033P09Rik	chr11	59022495	59023911	-0.43	0.04
Nlrp3	chr11	59355086	59365364	-47.90	-0.41
Nlrp3	chr11	59379889	59380129	-0.80	-0.15
Map2k3	chr11	60755823	60757022	-4.47	-0.42
Map2k3	chr11	60765438	60766245	-3.57	-0.18
Pld2	chr11	70371043	70371612	-5.37	-0.72
Crk	chr11	75492811	75517946	-0.68	-0.03
Crk	chr11	75518934	75519324	-1.20	0.15
Nos2	chr11	78734360	78734481	-0.28	0.42
Psmd11	chr11	80285544	80285635	-1.72	-0.32
Mpo	chr11	87611071	87613383	-0.49	2.58
Psmd3	chr11	98543867	98546829	-17.20	-0.58
Psmd3	chr11	98557023	98557113	-5.43	-0.52
Wipf2	chr11	98725061	98752118	-1.36	-0.28
Wipf2	chr11	98752840	98761613	-8.56	-0.36
Atp6v0a1	chr11	100923774	100924043	-4.60	-0.50
Psme3	chr11	101177564	101178336	-0.63	-0.07
Psme3	chr11	101182070	101183288	-1.70	-0.15
Grn	chr11	102291784	102294419	-6.26	-0.23
Grn	chr11	102297674	102297824	-15.10	-0.22
Gaa	chr11	119129310	119131951	-12.60	-0.42
Baiap2	chr11	119867827	119868096	-0.54	0.08
Nf2	chr11	4748990	4749260	-0.77	-0.15
Dock2	chr11	34127102	34128632	-0.54	-0.03
Dock2	chr11	34546949	34597325	-2.61	0.24
Cyfip2	chr11	46050820	46050881	-0.14	-0.26
Cyfip2	chr11	46051389	46051569	-0.67	1.95
Atox1	chr11	55264107	55274640	-1.13	-0.14
Mapk7	chr11	61303447	61306665	-9.04	-0.38
Mapk7	chr11	61306993	61307412	-1.70	-0.35
Map2k4	chr11	65503265	65507000	-2.15	-0.17
Nlrp1b	chr11	70966603	70969541	-0.48	-0.10
Nlrp1b	chr11	70995251	70995342	-0.44	-0.27
Nlrp1b	chr11	70996028	70996238	-1.60	-0.87
Kpnb1	chr11	97023572	97023813	-3.72	-0.21

Kpnb1	chr11	97049025	97049206	-3.31	-0.43
Ormdl3	chr11	98443291	98443381	-1.34	0.67
Ormdl3	chr11	98443826	98444154	-1.62	-0.36
Rab5c	chr11	100581475	100599444	-1.38	-0.37
Ghdc	chr11	100630466	100630763	-0.63	0.22
Dusp3	chr11	101834513	101835259	-2.71	0.14
Dusp3	chr11	101835527	101835855	-2.15	-0.15
Dusp3	chr11	101835944	101836213	-0.54	-0.05
Map3k14	chr11	103081077	103082247	-2.79	-0.40
Map3k14	chr11	103100406	103100995	-3.03	-0.56
Map3k14	chr11	103103496	103128715	-0.28	0.01
Cd300lb	chr11	114784364	114795700	-3.76	-0.10
Cd300e	chr11	114914519	114914670	-0.30	0.16
Grb2	chr11	115505517	115505876	-0.46	0.05
Grb2	chr11	115506621	115508033	-1.62	0.09
Tmc6	chr11	117627298	117628154	-3.89	-0.62
Cant1	chr11	118268408	118271504	-0.49	-0.03
Cant1	chr11	118272131	118279086	-6.00	-0.42
Psma6	chr12	56499811	56499871	-1.67	-0.32
Psen1	chr12	85072709	85075730	-1.54	-0.08
Fos	chr12	86814850	86815030	-8.25	-0.74
Fos	chr12	86815089	86817741	-45.40	-0.57
Psmc1	chr12	101348377	101351286	-1.53	-0.29
Calm1	chr12	101437750	101440606	-7.62	-0.30
Calm1	chr12	101445209	101445479	-1.73	0.08
Dync1h1	chr12	111839634	111839815	-0.77	-0.17
Dync1h1	chr12	111855964	111857506	-0.91	0.03
Dync1h1	chr12	111904592	111905154	-0.63	0.04
Traf3	chr12	112499736	112500694	-0.44	0.03
Pld4	chr12	113998865	113999998	-0.41	0.04
Pld4	chr12	114000237	114001663	-0.54	0.07
Pld4	chr12	114006322	114007047	-8.58	-0.25
Nfkbia	chr12	56593425	56593604	-76.60	-1.29
Atp6v1d	chr12	79962444	79962595	-0.40	-0.06
Npc2	chr12	86097931	86101679	-9.24	-0.21
Rps6ka5	chr12	101789241	101791305	-3.25	-0.84
Lgmn	chr12	103651753	103677907	-10.20	-0.26
Elmo1	chr13	20692056	20697377	-0.39	0.02
Ripk1	chr13	34094265	34094762	-5.04	-0.74
Ripk1	chr13	34119794	34126112	-1.97	0.13
Dsp	chr13	38287748	38287928	-0.38	0.81
Syk	chr13	52678805	52706632	-3.71	-0.23
Syk	chr13	52727069	52733425	-5.32	-0.47

Syk	chr13	52737300	52738605	-0.48	-0.06
Syk	chr13	52738964	52742902	-1.56	-0.08
Syk	chr13	52743830	52743951	-1.80	-0.43
Faf2	chr13	54723144	54723235	-0.40	0.17
Faf2	chr13	54757723	54763688	-0.38	-0.01
Lpcat1	chr13	73651356	73651986	-0.47	0.09
Cd180	chr13	103494924	103496711	-6.64	0.16
Cdk13	chr13	17809683	17811597	-9.02	0.30
Cdk13	chr13	17858388	17896931	-0.44	-0.02
Txndc5	chr13	38592171	38592559	-0.55	0.04
Txndc5	chr13	38593006	38595355	-3.77	-0.15
Txndc5	chr13	38620243	38620304	-0.32	0.03
Hk3	chr13	55107346	55107674	-3.38	-0.24
Hk3	chr13	55122245	55122665	-0.55	0.15
Rab24	chr13	55421293	55421501	-1.79	-0.26
Rab24	chr13	55422955	55423341	-2.38	-0.32
Dok3	chr13	55624595	55625593	-1.57	-0.09
Dok3	chr13	55628788	55629086	-1.40	-0.17
Dok3	chr13	55629708	55629917	-6.73	-1.05
Ddx41	chr13	55637396	55637930	-2.55	-0.37
Ctsl	chr13	64470393	64471614	-2.04	-0.09
Scamp1	chr13	94973830	94978130	-2.09	-0.34
Pik3r1	chr13	102454016	102456264	-1.08	0.13
Pik3r1	chr13	102478932	102525656	-0.51	0.13
Pik3r1	chr13	102527214	102538172	-1.57	0.16
Map3k1	chr13	112538703	112540665	-1.60	0.22
Map3k1	chr13	112545624	112546521	-0.86	-0.09
Map3k1	chr13	112566138	112599191	-2.43	-0.66
Nkiras1	chr14	19111014	19112745	-0.34	0.03
Plau	chr14	21661560	21662161	-0.63	-0.04
Vcl	chr14	21748654	21748715	-1.30	-0.59
Vcl	chr14	21850830	21851280	-5.31	0.38
Psmc6	chr14	45949498	45950514	-0.68	0.11
Pnp	chr14	51564155	51567563	-2.20	0.60
Pnp	chr14	51571126	51571721	-2.41	0.19
Ctsb	chr14	63761159	63761760	-116.00	-0.21
Clu	chr14	66587437	66590689	-7.01	-0.74
Ppp3cb	chr14	21320302	21327131	-1.02	-0.09
Ppp3cb	chr14	21365614	21365735	-0.60	-0.29
Prkcd	chr14	31408778	31411699	-1.32	-0.09
Prkcd	chr14	31411787	31411937	-0.35	-0.04
Prkcd	chr14	31439303	31439394	-1.67	-0.55
Tspan14	chr14	41720841	41721322	-0.90	-0.19

Ripk3	chr14	56403831	56404011	-4.18	1.03
Myo10	chr15	25552304	25552754	-96.80	-1.21
Myo10	chr15	25709521	25712766	-120.00	-0.72
Myo10	chr15	25736823	25740300	-157.00	-1.07
Myo10	chr15	25741959	25742409	-19.50	-0.50
Myo10	chr15	25743096	25743426	-3.95	-0.37
Ncf4	chr15	78075240	78075447	-1.52	-0.21
Ncf4	chr15	78091438	78093010	-2.87	-0.16
Tab1	chr15	79989200	79992132	-3.29	-0.26
Ep300	chr15	81416643	81438767	-1.28	-0.07
Ep300	chr15	81479273	81481819	-1.93	-0.07
Xrcc6	chr15	81870214	81870424	-0.78	-0.17
Ano6	chr15	95621303	95661131	-1.66	-0.31
Ano6	chr15	95802935	95804972	-5.92	0.19
Atf1	chr15	100084825	100090754	-5.11	0.31
Mettl7a1	chr15	100143493	100143673	-0.64	0.57
Nckap1l	chr15	103328061	103328301	-5.25	-0.34
Ptk2	chr15	73035624	73039772	-0.52	-0.04
Myh9	chr15	77592006	77593481	-52.20	-0.46
Myh9	chr15	77594368	77606283	-414.00	-0.50
Myh9	chr15	77643639	77672545	-3.85	-0.23
Pla2g6	chr15	79116836	79117224	-0.65	-0.14
Nfam1	chr15	82828225	82828824	-1.06	-0.14
Nfam1	chr15	82829452	82831880	-6.49	-0.34
Arsa	chr15	89303025	89303862	-1.00	-0.17
Arsa	chr15	89305988	89306615	-2.33	-0.41
Gpr84	chr15	103138665	103140869	-0.78	-0.04
Hmox2	chr16	4764613	4765867	-0.51	0.03
Mapk1	chr16	17038671	17038882	-0.44	0.05
Snap29	chr16	17427202	17428502	-2.91	0.37
Psmd2	chr16	20651724	20652324	-8.74	-0.32
Cd47	chr16	49867877	49868177	-13.10	0.61
Crebbp	chr16	4084047	4087743	-1.51	-0.07
Crebbp	chr16	4154961	4213404	-1.65	-0.09
Cpped1	chr16	11804530	11827393	-3.40	0.27
Cpped1	chr16	11828497	11828677	-0.40	-0.08
Cpped1	chr16	11887837	11909516	-3.47	-0.88
Pak2	chr16	32019832	32021909	-3.04	0.15
App	chr16	85025115	85043833	-35.70	-0.45
App	chr16	85120877	85173892	-5.24	-0.38
Cct8	chr16	87496053	87496114	-1.39	0.33
Rps6ka2	chr17	7374493	7374644	-1.21	0.96
Rps6ka2	chr17	7506914	7507424	-0.39	-0.14

1600012H06Rik	chr17	15080188	15081447	-11.60	-0.50
Mapk14	chr17	28828286	28828645	-6.50	-0.79
Mapk14	chr17	28884007	28885350	-0.47	0.02
Psmb8	chr17	34335288	34335468	-2.26	-0.35
Neu1	chr17	35071556	35072840	-0.43	0.02
H2-D1	chr17	35400038	35400558	-40.70	-0.54
H2-D1	chr17	35400807	35401046	-2.46	-0.23
H2-D1	chr17	35403816	35404204	-67.80	-0.36
H2-Q2	chr17	35479307	35479659	-0.13	0.17
H2-Q6	chr17	35561821	35564910	-7.70	-0.41
H2-Q6	chr17	35565949	35566691	-0.29	0.01
H2-Q10	chr17	35607880	35610039	-7.41	3.75
Trem1	chr17	48376164	48376373	-0.47	-1.34
Trem1	chr17	48383770	48384842	-0.66	0.48
Vav1	chr17	57418522	57418613	-1.00	-0.44
Vav1	chr17	57467106	57467554	-0.56	0.05
Ypel5	chr17	73198557	73198888	-0.59	0.14
Prkce	chr17	86567124	86568262	-0.44	-0.06
Prkce	chr17	87053997	87055374	-0.59	-0.12
Dynlt1f	chr17	6306339	6306549	-0.36	0.20
Rnaset2b	chr17	8335409	8340667	-8.21	-0.65
Igf2r	chr17	12876409	12876980	-2.88	-0.19
Fpr1	chr17	18013552	18020874	-0.21	-0.18
Mmp25	chr17	23782026	23782176	-0.64	3.81
Pdpk1	chr17	24215553	24216063	-6.49	0.47
Atp6v0c	chr17	24306196	24306374	-0.89	-0.16
H2-K1	chr17	34133105	34133495	-29.30	-0.60
H2-K1	chr17	34136698	34137218	-41.80	-0.78
Hspa1b	chr17	35093821	35096183	-7.51	0.43
Hspa1a	chr17	35107196	35107464	-1.02	3.56
Hspa1a	chr17	35108505	35108744	-1.36	5.33
Tubb5	chr17	35972171	35972588	-27.00	-0.24
H2-T23	chr17	36169129	36248130	-4.86	-0.40
Enpp4	chr17	44234667	44235237	-0.30	0.05
Enpp4	chr17	44236165	44242667	-6.56	1.02
Ppp2r5d	chr17	46820058	46820802	-0.99	-0.12
Ppp2r5d	chr17	46837029	46841951	-0.82	-0.21
Cnpy3	chr17	46872809	46874755	-3.98	-0.22
Ticam1	chr17	56408884	56416190	-14.90	-0.37
Vapa	chr17	65962685	65962895	-2.37	-0.25
Rab31	chr17	66001065	66001632	-0.94	0.07
Rab31	chr17	66002078	66002287	-3.48	-0.28
Rab31	chr17	66003448	66017287	-5.03	0.23

Rab31	chr17	66122031	66122092	-0.23	0.08
Sos1	chr17	80797100	80799097	-0.65	-0.07
Sos1	chr17	80833039	80834290	-0.49	-0.07
Sos1	chr17	80879343	80879554	-0.37	-0.28
Calm2	chr17	87835011	87846216	-1.79	-0.13
Ube2d2a	chr18	35931212	35931392	-0.74	-0.18
Ube2d2a	chr18	35965390	35965540	-0.95	0.36
Malt1	chr18	65635688	65635988	-3.80	-0.39
Map3k8	chr18	4348929	4352951	-0.72	-0.15
Rock1	chr18	10181251	10181790	-0.29	0.00
Tmem173	chr18	35893749	35894258	-1.04	-0.15
Cd14	chr18	36884720	36886308	-12.60	-0.07
Ticam2	chr18	46718782	46719052	-1.30	-0.50
Ticam2	chr18	46719441	46720699	-0.89	-0.13
Nfatc1	chr18	80803003	80803394	-3.39	-0.87
Nfatc1	chr18	80803872	80832717	-0.36	0.02
Nfatc1	chr18	80846232	80846653	-2.89	0.45
Nfatc1	chr18	80879052	80904865	-0.59	-0.05
Unc93b1	chr19	3935215	3935774	-0.37	0.02
Unc93b1	chr19	3936437	3936737	-0.41	0.03
Unc93b1	chr19	3948771	3948951	-21.20	-0.51
Rela	chr19	5646968	5647773	-3.73	-0.21
Pgam1	chr19	41986508	41990163	-5.12	-0.95
Btrc	chr19	45604177	45604358	-1.03	2.52
Nfkb2	chr19	46379346	46380320	-0.71	0.19
Tcirg1	chr19	3906859	3907103	-2.15	-0.38
Aldh3b1	chr19	3913756	3913816	-3.78	-0.66
Tbc1d10c	chr19	4184415	4184595	-0.49	-0.45
Peli3	chr19	4931973	4932510	-0.93	-0.92
Dtx4	chr19	12543910	12544151	-2.93	-0.29
Dtx4	chr19	12560929	12576605	-6.88	-0.19
Ddx3x	chrX	12858147	12861347	-2.40	0.16
Ddx3x	chrX	12868539	12868809	-39.40	0.65
Pgrmc1	chrX	34138515	34138605	-3.07	0.49
Tab3	chrX	82859477	82860195	-3.99	0.50
Tab3	chrX	82860492	82860673	-1.77	0.62
Tab3	chrX	82875804	82876044	-8.19	1.78
Atp7a	chrX	103265124	103280870	-18.90	0.76
Atp7a	chrX	103319725	103320025	-27.70	1.65
Huwe1	chrX	148353134	148354606	-16.40	0.30
Was	chrX	7667556	7667617	-0.56	-0.25
Rap2c	chrX	48359709	48371195	-4.90	0.26
Irak1	chrX	71260386	71261911	-1.24	0.14

Dnase1l1	chrX	71526935	71526996	-0.63	-0.37
Magt1	chrX	103207027	103207148	-2.06	0.47
Gla	chrX	131124401	131124580	-74.80	1.69
Gla	chrX	131135394	131135514	-1.45	0.53
Tlr8	chrX	163680663	163681230	-0.68	0.77
Tlr8	chrX	163681497	163681617	-0.19	0.13
Tlr8	chrX	163681973	163682213	-0.46	-0.65
Tlr8	chrX	163683254	163683344	-0.41	1.28
Tlr8	chrX	163683522	163683672	-0.98	2.22
Tlr7	chrX	163742862	163745489	-33.30	0.39
Tlr7	chrX	163745757	163746354	-1.39	0.09
Diaph1	chr18	38004807	38005077	-9.38	-0.60
Diaph1	chr18	38094885	38095065	-1.72	-0.48

Cytokine signaling in immune system

gene_name	chr	chromStart	chromEnd	diff.lg.p	diff.log2.fc
Casp8	chr1	58890568	58901676	-0.30	0.00
Abi2	chr1	60527596	60534244	-1.45	0.18
Creb1	chr1	64622786	64644320	-12.70	0.59
Slc11a1	chr1	74421776	74422382	-3.48	-0.58
Slc11a1	chr1	74432056	74432625	-0.70	0.06
Cab39	chr1	87690021	87715038	-1.14	-0.12
Cab39	chr1	87745789	87746418	-1.09	-0.07
Psmd1	chr1	87961193	87961254	-0.37	-0.11
Arl8a	chr1	137043410	137043590	-2.14	-0.35
Arpc5	chr1	154613671	154613968	-7.60	-0.29
Ncf2	chr1	154654984	154655314	-0.34	0.01
Creg1	chr1	167693910	167694001	-0.45	-0.10
Aim2	chr1	175389828	175395719	-6.98	0.20
Tmem63a	chr1	182904339	182904638	-1.16	-0.26
Tmbim1	chr1	74335180	74335241	-2.07	-0.51
Tmbim1	chr1	74341896	74350760	-11.80	-0.96
Bcl2	chr1	108438384	108439674	-0.90	0.22
Bcl2	chr1	108439763	108608408	-0.87	0.54
Bcl2	chr1	108608737	108610507	-1.27	-0.20
Bcl2	chr1	108610626	108610867	-0.58	0.39
Ptpn4	chr1	121555598	121572143	-7.36	0.52
Ptpn4	chr1	121733557	121733648	-0.97	-0.74
Actr3	chr1	127331982	127332265	-4.20	-0.39
Ikbke	chr1	133151178	133153534	-2.21	-0.20
Ikbke	chr1	133172403	133173518	-4.46	-0.66
Dhx9	chr1	155303276	155303397	-3.17	0.68
Qsox1	chr1	157626448	157628411	-2.42	-0.35
Fcgr2b	chr1	172890688	172891165	-4.70	0.21

Fcgr2b	chr1	172906081	172906200	-0.31	0.02
Fcgr3	chr1	172981537	172981597	-1.79	-0.50
Ncstn	chr1	173996440	173996917	-6.01	-0.35
Degs1	chr1	184206857	184208760	-3.23	0.22
Degs1	chr1	184209059	184209748	-12.40	0.28
Commd3	chr2	18596461	18597638	-0.32	-0.01
Abl1	chr2	31544075	31551320	-1.18	-0.13
Abl1	chr2	31653102	31658129	-1.05	-0.08
Ptges2	chr2	32257786	32257964	-3.07	-0.78
Tank	chr2	61416642	61431255	-0.74	-0.21
Tank	chr2	61482442	61484290	-0.30	-0.01
Tank	chr2	61484409	61488467	-1.76	0.12
Tank	chr2	61491449	61491629	-0.53	0.07
Itgav	chr2	83564583	83565003	-11.70	-0.93
Itgav	chr2	83643447	83643718	-4.06	-0.49
Itgav	chr2	83643777	83643958	-1.72	-0.33
Psmc3	chr2	90894439	90895182	-2.45	-0.16
Traf6	chr2	101531587	101537959	-2.12	0.17
B2m	chr2	121973422	121973632	-3.80	-0.13
Sirpa	chr2	129418574	129418993	-2.78	0.40
Sirpa	chr2	129419320	129457247	-0.96	-0.02
Mavs	chr2	131068979	131072532	-4.44	-0.40
Pygb	chr2	150654268	150656555	-0.44	0.05
Hck	chr2	152934263	152934443	-1.07	0.34
Hck	chr2	152976907	152977087	-0.51	-0.07
Itch	chr2	154959216	154959307	-0.30	0.07
Itch	chr2	155050132	155050403	-0.62	-0.11
Src	chr2	157295196	157296561	-1.97	0.63
Plcg1	chr2	160587522	160588203	-4.39	-0.75
Ctsa	chr2	164659287	164659646	-8.35	-0.27
Mmp9	chr2	164780329	164780659	-0.85	-0.58
Abi1	chr2	22895609	22895730	-1.85	-0.40
Tubb4b	chr2	25077855	25080192	-0.38	-0.01
Dpp7	chr2	25207809	25207987	-3.64	0.45
Dpp7	chr2	25211792	25211852	-1.75	0.43
Card9	chr2	26212789	26213529	-0.79	0.09
Agpat2	chr2	26449106	26450082	-0.51	-0.12
Surf4	chr2	26777259	26777678	-5.01	-0.17
Surf4	chr2	26782443	26788971	-5.66	-0.47
Vav2	chr2	27282074	27282345	-1.98	-1.07
Psmd5	chr2	34707993	34709823	-0.82	-0.10
Psmd5	chr2	34726274	34726482	-3.28	-0.70
Rab14	chr2	35056341	35056550	-13.00	-0.63

Psmb7	chr2	38498946	38499426	-1.70	-0.24
Wipf1	chr2	73270330	73270480	-0.29	0.00
Wipf1	chr2	73273054	73275208	-7.06	-0.48
Wipf1	chr2	73275357	73278120	-3.10	-0.24
Wipf1	chr2	73282586	73291856	-0.45	0.07
Atf2	chr2	73656977	73657038	-1.83	0.77
Ptprj	chr2	90273053	90274369	-19.20	-0.53
Ptprj	chr2	90420444	90420774	-0.85	-0.18
Cd44	chr2	102654086	102662407	-79.70	-0.56
Atp8b4	chr2	126148354	126148594	-0.41	0.05
Il1b	chr2	129190434	129191002	-4.06	2.30
Il1b	chr2	129195984	129196856	-1.15	2.95
Cd93	chr2	148263313	148263433	-0.69	4.00
Cd93	chr2	148266721	148267597	-0.52	1.75
Cd93	chr2	148267925	148268195	-0.71	3.50
Cd93	chr2	148268851	148269062	-0.38	1.48
Psmf1	chr2	151543679	151544068	-0.32	0.02
Bcl2l1	chr2	152606912	152607242	-0.45	0.04
Bcl2l1	chr2	152607660	152653984	-27.50	-0.71
Bcl2l1	chr2	152655121	152656044	-111.00	-0.82
Bcl2l1	chr2	152656237	152657418	-2.87	-0.78
Cpne1	chr2	155922158	155937671	-0.31	0.00
Dsn1	chr2	156821512	156821930	-1.54	-0.43
Slpi	chr2	164181290	164182042	-96.20	-1.36
Elmo2	chr2	165115177	165116717	-2.78	-0.28
Elmo2	chr2	165117584	165117675	-2.59	-1.26
Nfatc2	chr2	168301999	168303229	-1.92	-0.20
Nfatc2	chr2	168304128	168330367	-7.02	-0.30
Nfatc2	chr2	168396317	168427005	-7.34	-0.40
Ctsz	chr2	174252994	174254182	-52.90	-0.22
Ctsz	chr2	174264374	174264434	-4.27	-0.36
Fabp5	chr3	10015061	10015506	-2.68	-0.28
Pld1	chr3	28029315	28031326	-1.86	-0.40
Pld1	chr3	28031774	28031835	-0.53	-0.67
Pik3ca	chr3	32335072	32335372	-0.45	-0.08
Pik3ca	chr3	32360681	32362022	-3.50	0.35
Rap2b	chr3	61168428	61170347	-12.00	0.31
Rap2b	chr3	61171125	61171306	-0.74	-0.36
Shc1	chr3	89222472	89225594	-0.41	-0.06
Shc1	chr3	89225712	89226222	-14.10	-1.02
Shc1	chr3	89232244	89232484	-0.39	0.03
Ilf2	chr3	90291521	90291822	-1.69	-0.21
Ctss	chr3	95330707	95333452	-1.96	-0.10

Txnip	chr3	96362296	96362849	-42.00	0.57
Txnip	chr3	96363879	96364976	-345.00	0.76
Txnip	chr3	96365273	96365601	-111.00	1.13
Vav3	chr3	109143660	109143900	-1.03	-0.68
Ube2d3	chr3	135101656	135102228	-6.45	-0.41
Manba	chr3	135230676	135233888	-0.32	0.01
Bcl10	chr3	145587262	145587592	-1.35	-0.17
Bcl10	chr3	145588099	145593586	-0.30	0.00
Nhlrc3	chr3	53255917	53257521	-3.38	-0.42
Dhx36	chr3	62274511	62275295	-0.82	-0.16
Tlr2	chr3	83640193	83645500	-1.21	0.04
Lamtor2	chr3	88353884	88356849	-9.91	-0.33
Psmb4	chr3	94690551	94690880	-2.31	-0.17
Psmd4	chr3	94836626	94836894	-5.68	-0.27
Psmd4	chr3	94839816	94846467	-4.83	-0.24
Fcgr1	chr3	96087757	96088506	-3.70	-0.43
Cd53	chr3	106563447	106563716	-7.16	0.21
Nfkb1	chr3	135248217	135252299	-9.16	-0.42
Nfkb1	chr3	135354210	135354481	-4.75	1.80
Prkacb	chr3	146394723	146394993	-0.71	0.12
Lyn	chr4	3605267	3605506	-17.80	-0.52
Lyn	chr4	3716911	3718014	-9.19	-0.18
Map3k7	chr4	32051081	32051232	-1.86	-0.83
Map3k7	chr4	32104109	32107267	-4.75	0.31
Map3k7	chr4	32110141	32110231	-1.49	-0.76
Atp6v1g1	chr4	63205828	63209679	-0.73	0.11
Trim32	chr4	65274556	65274646	-0.66	-0.27
Trim32	chr4	65274824	65276139	-4.40	0.35
Tlr4	chr4	66494995	66502966	-7.48	0.33
Pgm2	chr4	99602085	99602234	-1.37	-0.46
Pgm2	chr4	99659304	99659542	-4.78	-0.71
Ptafr	chr4	132119981	132136781	-0.79	0.06
Fgr	chr4	132530039	132542068	-0.98	0.34
Fgr	chr4	132556096	132556514	-7.15	-0.57
Fgr	chr4	132557289	132557469	-2.85	0.87
Wasf2	chr4	132751642	132752809	-3.91	-0.14
Fuca1	chr4	135476640	135476908	-9.09	-0.79
Fuca1	chr4	135495027	135495325	-1.89	0.27
Ddost	chr4	137868046	137868406	-0.32	-0.01
Casp9	chr4	141352424	141352694	-2.96	-0.57
Casp9	chr4	141368153	141369672	-0.74	0.33
Casp9	chr4	141369761	141369941	-0.56	1.17
Slc2a5	chr4	149494002	149494152	-0.29	1.46

Ripk2	chr4	16050700	16051621	-9.33	-0.60
Ripk2	chr4	16090465	16090645	-12.00	-1.26
Ddx58	chr4	40152603	40155517	-1.09	-0.14
Ddx58	chr4	40186678	40186858	-0.63	0.23
B4galt1	chr4	40753786	40754830	-2.74	0.20
B4galt1	chr4	40770597	40800583	-1.25	0.19
B4galt1	chr4	40800821	40800971	-2.38	-0.70
Vcp	chr4	43013259	43013349	-0.47	-0.15
Alad	chr4	62170203	62171204	-0.29	0.00
Jun	chr4	94716292	94718913	-1.94	0.04
Cap1	chr4	122536500	122536680	-41.90	-0.54
Lck	chr4	129225826	129225977	-1.80	-0.75
Rps6ka1	chr4	133403354	133404010	-1.09	-0.10
Tnfrsf1b	chr4	144803977	144809673	-187.00	-0.87
Tnfrsf1b	chr4	144818945	144836773	-24.30	-1.26
Tnfrsf1b	chr4	144836594	144836774	-3.62	-0.69
Kcnab2	chr4	151766373	151767149	-2.14	-0.15
Atad3a	chr4	155114748	155115286	-0.82	-0.07
Bst1	chr5	44210161	44210281	-0.88	-0.46
Cxcl3	chr5	91215158	91215370	-0.42	-0.69
Cxcl2	chr5	91332924	91333518	-434.00	-2.15
Stbd1	chr5	93034080	93034200	-0.12	0.00
Hvcn1	chr5	122690237	122692276	-1.11	-0.20
P2rx7	chr5	123093919	123094010	-4.98	1.77
P2rx7	chr5	123130997	123132855	-10.40	0.31
P2rx7	chr5	123133124	123133994	-8.79	0.52
Psmd9	chr5	123678228	123678437	-1.01	0.20
Atp6v0a2	chr5	125200361	125202517	-1.22	0.17
Arpc1b	chr5	145875124	145883438	-3.09	-0.16
Tnip2	chr5	34839311	34839491	-0.39	0.07
Tnip2	chr5	34842145	34842355	-1.03	0.23
Tlr1	chr5	65316072	65316462	-1.84	0.58
Tlr1	chr5	65317358	65317538	-0.27	0.02
Tlr1	chr5	65318106	65318495	-0.63	0.23
Tlr6	chr5	65344333	65351228	-28.20	0.94
Atp8a1	chr5	68013273	68013783	-1.25	0.26
Tec	chr5	73147081	73148633	-1.81	-0.24
Tec	chr5	73214842	73259687	-5.06	0.82
Mlec	chr5	115597834	115600228	-0.81	-0.07
Mlec	chr5	115600927	115608185	-3.72	-0.22
Ptpn11	chr5	121581827	121582307	-1.65	0.20
Ptpn11	chr5	121583292	121587519	-2.53	-0.16
Rhof	chr5	123553529	123554067	-7.00	2.05

Rhof	chr5	123567437	123567766	-1.20	-0.45
Rhof	chr5	123568123	123582399	-23.20	-0.42
Ubc	chr5	125866482	125866751	-7.45	-0.16
Ubc	chr5	125867492	125867701	-3.77	1.98
Ubc	chr5	125867967	125870328	-2.39	0.10
Slc15a4	chr5	128076540	128080872	-0.31	0.00
Gusb	chr5	130464890	130465519	-2.75	0.10
Gusb	chr5	130478517	130478698	-4.28	-0.24
Ncf1	chr5	134697332	134697663	-8.46	-0.28
Lat2	chr5	135076464	135078531	-5.64	-0.19
Trim56	chr5	137587155	137587605	-10.60	-0.53
Trim56	chr5	137587784	137588144	-0.80	-0.16
Trim56	chr5	137588275	137588336	-0.64	0.56
Trim56	chr5	137588635	137592077	-28.50	-0.43
Card11	chr5	141348952	141350301	-2.19	-0.58
Rac1	chr5	144288651	144288861	-1.33	0.22
Pdap1	chr5	145892323	145900958	-18.10	-0.36
Atp6v1f	chr6	29417782	29417841	-1.46	-0.23
Cul1	chr6	47404345	47445165	-12.40	0.63
Cul1	chr6	47474617	47476108	-2.74	-0.21
Arpc4	chr6	113328136	113330743	-0.46	0.06
Irak2	chr6	113640953	113644510	-13.00	-0.41
Clec4d	chr6	123212154	123212273	-2.02	0.39
C1ra	chr6	124472382	124472951	-1.99	-0.43
C1rb	chr6	124530824	124530943	-0.36	0.74
Lrmp	chr6	145086263	145086444	-0.85	-0.32
Impdh1	chr6	29162071	29162252	-2.44	-0.43
Clec5a	chr6	40527283	40529441	-0.30	0.00
Nod1	chr6	54893317	54893527	-0.62	1.79
Nod1	chr6	54894659	54894899	-0.40	-0.75
Vamp8	chr6	72335214	72335477	-0.41	-0.03
Vamp8	chr6	72338403	72340632	-1.83	-0.34
Kcmf1	chr6	72849172	72849233	-0.31	-0.52
Kcmf1	chr6	72849822	72849973	-0.27	0.15
Rbsn	chr6	92139135	92140425	-0.38	0.02
Raf1	chr6	115568590	115569280	-0.54	-0.05
Alox5	chr6	116410986	116411166	-1.01	-0.30
Atp6v1e1	chr6	120768397	120772703	-4.00	-0.38
Slc2a3	chr6	122679681	122680131	-0.97	-0.19
C3ar1	chr6	122799485	122806145	-5.99	-0.19
C1s1	chr6	124481017	124481078	-0.14	-0.42
C1s1	chr6	124481405	124481466	-0.11	0.00
C1s2	chr6	124575330	124575809	-0.33	1.51

Ptpn6	chr6	124683045	124688519	-0.44	0.09
Olr1	chr6	129436974	129437604	-0.52	1.98
Kras	chr6	145168813	145168964	-1.25	-0.35
Plaur	chr7	25247518	25251724	-91.60	-1.63
Plaur	chr7	25260294	25260713	-133.00	-1.32
Tyrobp	chr7	31198806	31198866	-0.60	-0.05
Irf3	chr7	52253029	52253209	-0.42	-0.09
Irf3	chr7	52257067	52257801	-2.15	-0.33
Cyfip1	chr7	63109393	63127258	-0.43	-0.06
Ctsc	chr7	95437274	95437455	-0.26	0.00
Ctsc	chr7	95458037	95458487	-2.08	0.25
Prcp	chr7	100081684	100082313	-0.64	-0.10
Ndufc2	chr7	104548571	104548690	-0.85	0.14
Lamtor1	chr7	109054380	109058241	-0.51	-0.09
Lamtor1	chr7	109059912	109060091	-7.00	-0.38
Ampd3	chr7	117954502	117954682	-3.55	0.65
Itgam	chr7	135270356	135270776	-10.60	1.52
Itgax	chr7	135292687	135294081	-3.59	1.65
Lair1	chr7	3961115	3961265	-1.04	-0.76
Ube2m	chr7	13623108	13623619	-2.32	-0.37
C5ar2	chr7	16822087	16822178	-0.28	-0.26
C5ar2	chr7	16822715	16822896	-1.32	-1.43
C5ar2	chr7	16823104	16823255	-0.32	0.53
C5ar1	chr7	16833166	16844566	-34.20	-0.54
Calm3	chr7	17502039	17502309	-2.51	-0.14
Relb	chr7	20191570	20192258	-0.92	-0.11
Ceacam1	chr7	26247769	26248608	-3.38	0.29
Ceacam1	chr7	26256984	26258859	-1.12	-0.38
Ceacam1	chr7	26259008	26274241	-1.35	0.18
Nfkbib	chr7	29546684	29546893	-27.50	-0.92
Nfkbib	chr7	29547132	29551543	-9.72	-0.61
Psmd8	chr7	29965393	29965573	-6.44	-0.28
Gpi1	chr7	34987212	34987689	-18.10	-0.27
Siglece	chr7	50915321	50915471	-0.80	0.91
Ftl1	chr7	52715051	52715256	-402.00	-0.43
Anpep	chr7	86966748	86966927	-0.81	-0.13
Anpep	chr7	86986085	86993080	-3.53	-0.12
Iqgap1	chr7	87857938	87858329	-7.34	-0.18
Iqgap1	chr7	87858478	87858659	-1.28	-0.06
Iqgap1	chr7	87944725	87948187	-32.40	-0.56
Rhog	chr7	109387636	109398632	-17.50	-0.20
Trim21	chr7	109707151	109708560	-3.22	0.40
Adam8	chr7	147164899	147165378	-38.70	-0.54

Hras	chr7	148376758	148378177	-0.33	-0.01
Hras	chr7	148378805	148379903	-1.48	0.51
Tollip	chr7	149069998	149075492	-4.83	-0.28
Ctsd	chr7	149561820	149562294	-47.90	-0.19
Fadd	chr7	151765572	151768221	-1.92	-0.22
Map2k7	chr8	4245576	4246251	-2.64	-0.23
Atp11a	chr8	12757015	12757255	-0.36	0.24
Atp11a	chr8	12864987	12866454	-1.34	0.14
Lamp1	chr8	13159223	13159402	-1.74	-0.18
Plpp5	chr8	26834581	26835060	-0.38	-0.03
Ppp2cb	chr8	34710092	34710330	-0.63	-0.43
Dusp4	chr8	35870663	35882259	-110.00	-0.60
Myo9b	chr8	73796612	73839062	-0.95	0.06
Myo9b	chr8	73871734	73876118	-11.40	-0.25
Myo9b	chr8	73882022	73884611	-1.43	0.07
Prkaca	chr8	86496876	86500855	-3.14	-0.78
Man2b1	chr8	87607167	87607377	-1.30	-0.24
Cyld	chr8	91220956	91221047	-0.33	-0.20
Cyld	chr8	91226307	91233793	-8.51	0.40
Nfatc3	chr8	108602601	108603618	-1.85	0.13
Nfatc3	chr8	108632465	108652166	-1.29	0.10
Plcg2	chr8	120022190	120028105	-0.37	-0.04
Ikbkb	chr8	23770546	23770997	-0.43	-0.04
Golga7	chr8	24352876	24353057	-1.98	-0.24
Hgsnat	chr8	27054960	27055586	-11.20	0.64
Hgsnat	chr8	27083350	27087156	-0.46	0.09
Tlr3	chr8	46482427	46484485	-1.03	0.28
Pik3r2	chr8	73292079	73292528	-3.00	-0.41
Pik3r2	chr8	73298580	73300551	-1.20	-0.17
Adgre5	chr8	86247268	86247657	-11.70	-1.33
Psmd7	chr8	110104309	110108107	-19.20	0.30
Hp	chr8	112099087	112099805	-2.48	-0.74
Ist1	chr8	112195759	112196600	-7.45	0.33
Cotl1	chr8	122364299	122364479	-1.03	0.15
Galns	chr8	125102315	125103030	-1.46	-0.23
Casp1	chr9	5298664	5299458	-1.72	0.32
Pin1	chr9	20456573	20459888	-1.84	-0.36
Slc44a2	chr9	21156935	21159322	-7.12	0.27
Dnm2	chr9	21310781	21311349	-0.34	0.01
Dnm2	chr9	21311898	21312198	-3.13	0.36
Ppp2r1b	chr9	50688020	50688710	-0.53	0.05
Ppp2r1b	chr9	50701016	50701675	-1.83	-0.33
Pstpip1	chr9	55937782	55937902	-1.36	-0.40

Adam10	chr9	70526807	70526958	-0.76	0.19
Adam10	chr9	70626086	70626267	-1.49	0.28
Rab27a	chr9	72892731	72908786	-0.70	-1.27
Rab27a	chr9	72943119	72943840	-0.62	-0.22
Myo5a	chr9	74919042	74919403	-0.47	0.08
Myo5a	chr9	75065709	75066579	-14.50	0.35
Pik3r4	chr9	105546522	105551238	-3.59	0.32
Pik3r4	chr9	105552647	105556359	-0.68	0.10
Pik3r4	chr9	105589445	105589895	-1.34	0.26
Tlr9	chr9	106124928	106129177	-57.50	1.05
Dusp7	chr9	106270962	106271201	-1.93	-1.11
Dusp7	chr9	106271586	106276865	-0.38	-0.01
Nckipsd	chr9	108714765	108714915	-1.56	-0.69
Glb1	chr9	114310242	114326084	-1.04	0.28
Glb1	chr9	114368770	114373539	-0.71	0.08
Glb1	chr9	114374377	114383495	-16.40	0.68
Dync1li1	chr9	114632686	114632895	-0.48	0.07
Dync1li1	chr9	114632812	114633189	-3.66	0.29
Ctnnb1	chr9	120842577	120859351	-2.42	-0.18
Ctnnb1	chr9	120866971	120868877	-19.00	0.26
Ctnnb1	chr9	120868936	120868997	-1.38	0.13
Ccr2	chr9	124021097	124021367	-1.86	-0.98
Birc2	chr9	7818494	7818943	-1.37	0.33
Birc2	chr9	7833550	7834657	-8.57	0.55
Birc2	chr9	7834955	7835225	-0.88	0.29
Birc3	chr9	7848939	7850928	-0.65	0.09
Birc3	chr9	7860281	7872961	-1.11	0.10
Panx1	chr9	14810228	14812439	-0.40	-0.04
Rab3d	chr9	21714741	21715012	-4.69	-1.31
Tirap	chr9	34994185	34994305	-0.27	0.01
Tirap	chr9	34994483	34996852	-1.16	0.12
Nlrx1	chr9	44060795	44061574	-0.93	-0.29
Nlrx1	chr9	44064954	44068731	-0.40	-0.37
Nlrx1	chr9	44070466	44072299	-0.30	-0.01
Pafah1b2	chr9	45776623	45777013	-5.68	0.31
Chrnb4	chr9	54883115	54883236	-0.29	1.46
Tmem30a	chr9	79617076	79617405	-1.31	0.16
Pik3cb	chr9	98939598	98941447	-3.91	0.26
Armc8	chr9	99380819	99381090	-0.59	0.18
Armc8	chr9	99420671	99420882	-2.06	0.72
Nck1	chr9	100395689	100407191	-3.91	0.25
Dnajc13	chr9	104054165	104054614	-3.68	0.31
Apeh	chr9	107987744	107987894	-0.37	-0.08

Myd88	chr9	119248949	119249158	-0.83	-0.18
Fuca2	chr10	13222977	13225667	-1.46	0.28
Fuca2	chr10	13234515	13235470	-8.82	0.38
Frk	chr10	34203714	34203864	-0.26	0.90
Frk	chr10	34327775	34328373	-0.21	-0.81
Fyn	chr10	39089604	39089964	-2.08	-0.38
Fyn	chr10	39271197	39284162	-0.31	0.00
Lilr4b	chr10	51200478	51200715	-7.27	-0.46
Lilr4b	chr10	51205082	51205501	-207.00	-1.21
Psap	chr10	59740405	59754400	-0.37	-0.02
Itgb2	chr10	76993092	77005145	-2.27	-0.27
Cdc34	chr10	79144939	79145322	-7.32	-0.60
Cdc34	chr10	79148043	79151143	-8.59	-0.24
Arhgap45	chr10	79479416	79480812	-10.80	-0.44
Arhgap45	chr10	79485025	79488219	-1.43	-0.13
Arhgap45	chr10	79493111	79494216	-2.35	-0.14
Eef2	chr10	80644273	80645254	-1.14	-0.02
Eea1	chr10	95473536	95474748	-15.20	-0.62
Eea1	chr10	95488768	95489708	-4.51	-0.29
Eea1	chr10	95499325	95499386	-0.41	-0.05
Eea1	chr10	95502273	95504101	-1.18	-0.12
Eea1	chr10	95504160	95504884	-0.30	0.01
Dusp6	chr10	98725864	98729379	-7.49	0.29
Cep290	chr10	100034774	100035631	-1.17	0.53
Gns	chr10	120802145	120802355	-0.34	0.03
Gns	chr10	120832206	120832746	-2.71	0.13
Gns	chr10	120833971	120834301	-7.42	0.38
Arhgap9	chr10	126759908	126761407	-1.07	0.40
Stat6	chr10	127080041	127080222	-2.27	-0.32
Stat6	chr10	127096737	127097564	-1.15	-0.06
Tab2	chr10	7625983	7626463	-0.30	0.00
Tab2	chr10	7626701	7629919	-0.81	-0.05
Tab2	chr10	7630969	7644574	-2.87	-0.09
Tnfaip3	chr10	18721581	18725108	-0.69	-0.06
Ube2d1	chr10	70718174	70718562	-1.17	0.19
Ckap4	chr10	83990001	83991399	-0.32	0.00
Ckap4	chr10	83996275	83996633	-1.47	-0.37
Hsp90b1	chr10	86168098	86168159	-17.40	-0.72
Glipr1	chr10	111434115	111434291	-3.73	0.43
Cand1	chr10	118647870	118648919	-5.77	0.23
Dbnl	chr11	5688485	5691845	-3.37	-0.49
Dbnl	chr11	5699963	5700503	-8.51	-0.43
Peli1	chr11	20991326	20991536	-0.55	-0.13

Peli1	chr11	21047425	21048774	-5.67	-0.32
Psme4	chr11	30671804	30671925	-0.83	-0.91
Psme4	chr11	30672014	30672135	-0.38	0.12
Stk10	chr11	32433265	32433386	-0.63	0.22
Stk10	chr11	32489401	32503691	-28.60	-0.48
Stk10	chr11	32504289	32512777	-6.57	-0.53
Stk10	chr11	32517862	32522921	-14.90	-0.45
Fbxw11	chr11	32644536	32645016	-2.21	0.20
Mapk9	chr11	49696697	49697026	-1.50	0.25
Ppp2ca	chr11	51912325	51912474	-3.96	-0.95
Ppp2ca	chr11	51935449	51935836	-1.90	0.10
Gm2a	chr11	54923031	54923210	-0.87	-0.15
2310033P09Rik	chr11	59022495	59023911	-0.43	0.04
Nlrp3	chr11	59355086	59365364	-47.90	-0.41
Nlrp3	chr11	59379889	59380129	-0.80	-0.15
Map2k3	chr11	60755823	60757022	-4.47	-0.42
Map2k3	chr11	60765438	60766245	-3.57	-0.18
Pld2	chr11	70371043	70371612	-5.37	-0.72
Crk	chr11	75492811	75517946	-0.68	-0.03
Crk	chr11	75518934	75519324	-1.20	0.15
Nos2	chr11	78734360	78734481	-0.28	0.42
Psmd11	chr11	80285544	80285635	-1.72	-0.32
Mpo	chr11	87611071	87613383	-0.49	2.58
Psmd3	chr11	98543867	98546829	-17.20	-0.58
Psmd3	chr11	98557023	98557113	-5.43	-0.52
Wipf2	chr11	98725061	98752118	-1.36	-0.28
Wipf2	chr11	98752840	98761613	-8.56	-0.36
Atp6v0a1	chr11	100923774	100924043	-4.60	-0.50
Psme3	chr11	101177564	101178336	-0.63	-0.07
Psme3	chr11	101182070	101183288	-1.70	-0.15
Grn	chr11	102291784	102294419	-6.26	-0.23
Grn	chr11	102297674	102297824	-15.10	-0.22
Gaa	chr11	119129310	119131951	-12.60	-0.42
Baiap2	chr11	119867827	119868096	-0.54	0.08
Nf2	chr11	4748990	4749260	-0.77	-0.15
Dock2	chr11	34127102	34128632	-0.54	-0.03
Dock2	chr11	34546949	34597325	-2.61	0.24
Cyfip2	chr11	46050820	46050881	-0.14	-0.26
Cyfip2	chr11	46051389	46051569	-0.67	1.95
Atox1	chr11	55264107	55274640	-1.13	-0.14
Mapk7	chr11	61303447	61306665	-9.04	-0.38
Mapk7	chr11	61306993	61307412	-1.70	-0.35
Map2k4	chr11	65503265	65507000	-2.15	-0.17

Nlrp1b	chr11	70966603	70969541	-0.48	-0.10
Nlrp1b	chr11	70995251	70995342	-0.44	-0.27
Nlrp1b	chr11	70996028	70996238	-1.60	-0.87
Kpnb1	chr11	97023572	97023813	-3.72	-0.21
Kpnb1	chr11	97049025	97049206	-3.31	-0.43
Ormdl3	chr11	98443291	98443381	-1.34	0.67
Ormdl3	chr11	98443826	98444154	-1.62	-0.36
Rab5c	chr11	100581475	100599444	-1.38	-0.37
Ghdc	chr11	100630466	100630763	-0.63	0.22
Dusp3	chr11	101834513	101835259	-2.71	0.14
Dusp3	chr11	101835527	101835855	-2.15	-0.15
Dusp3	chr11	101835944	101836213	-0.54	-0.05
Map3k14	chr11	103081077	103082247	-2.79	-0.40
Map3k14	chr11	103100406	103100995	-3.03	-0.56
Map3k14	chr11	103103496	103128715	-0.28	0.01
Cd300lb	chr11	114784364	114795700	-3.76	-0.10
Cd300e	chr11	114914519	114914670	-0.30	0.16
Grb2	chr11	115505517	115505876	-0.46	0.05
Grb2	chr11	115506621	115508033	-1.62	0.09
Tmc6	chr11	117627298	117628154	-3.89	-0.62
Cant1	chr11	118268408	118271504	-0.49	-0.03
Cant1	chr11	118272131	118279086	-6.00	-0.42
Psma6	chr12	56499811	56499871	-1.67	-0.32
Psen1	chr12	85072709	85075730	-1.54	-0.08
Fos	chr12	86814850	86815030	-8.25	-0.74
Fos	chr12	86815089	86817741	-45.40	-0.57
Psmc1	chr12	101348377	101351286	-1.53	-0.29
Calm1	chr12	101437750	101440606	-7.62	-0.30
Calm1	chr12	101445209	101445479	-1.73	0.08
Dync1h1	chr12	111839634	111839815	-0.77	-0.17
Dync1h1	chr12	111855964	111857506	-0.91	0.03
Dync1h1	chr12	111904592	111905154	-0.63	0.04
Traf3	chr12	112499736	112500694	-0.44	0.03
Pld4	chr12	113998865	113999998	-0.41	0.04
Pld4	chr12	114000237	114001663	-0.54	0.07
Pld4	chr12	114006322	114007047	-8.58	-0.25
Nfkbia	chr12	56593425	56593604	-76.60	-1.29
Atp6v1d	chr12	79962444	79962595	-0.40	-0.06
Npc2	chr12	86097931	86101679	-9.24	-0.21
Rps6ka5	chr12	101789241	101791305	-3.25	-0.84
Lgmn	chr12	103651753	103677907	-10.20	-0.26
Elmo1	chr13	20692056	20697377	-0.39	0.02
Ripk1	chr13	34094265	34094762	-5.04	-0.74

Ripk1	chr13	34119794	34126112	-1.97	0.13
Dsp	chr13	38287748	38287928	-0.38	0.81
Syk	chr13	52678805	52706632	-3.71	-0.23
Syk	chr13	52727069	52733425	-5.32	-0.47
Syk	chr13	52737300	52738605	-0.48	-0.06
Syk	chr13	52738964	52742902	-1.56	-0.08
Syk	chr13	52743830	52743951	-1.80	-0.43
Faf2	chr13	54723144	54723235	-0.40	0.17
Faf2	chr13	54757723	54763688	-0.38	-0.01
Lpcat1	chr13	73651356	73651986	-0.47	0.09
Cd180	chr13	103494924	103496711	-6.64	0.16
Cdk13	chr13	17809683	17811597	-9.02	0.30
Cdk13	chr13	17858388	17896931	-0.44	-0.02
Txndc5	chr13	38592171	38592559	-0.55	0.04
Txndc5	chr13	38593006	38595355	-3.77	-0.15
Txndc5	chr13	38620243	38620304	-0.32	0.03
Hk3	chr13	55107346	55107674	-3.38	-0.24
Hk3	chr13	55122245	55122665	-0.55	0.15
Rab24	chr13	55421293	55421501	-1.79	-0.26
Rab24	chr13	55422955	55423341	-2.38	-0.32
Dok3	chr13	55624595	55625593	-1.57	-0.09
Dok3	chr13	55628788	55629086	-1.40	-0.17
Dok3	chr13	55629708	55629917	-6.73	-1.05
Ddx41	chr13	55637396	55637930	-2.55	-0.37
Ctsl	chr13	64470393	64471614	-2.04	-0.09
Scamp1	chr13	94973830	94978130	-2.09	-0.34
Pik3r1	chr13	102454016	102456264	-1.08	0.13
Pik3r1	chr13	102478932	102525656	-0.51	0.13
Pik3r1	chr13	102527214	102538172	-1.57	0.16
Map3k1	chr13	112538703	112540665	-1.60	0.22
Map3k1	chr13	112545624	112546521	-0.86	-0.09
Map3k1	chr13	112566138	112599191	-2.43	-0.66
Nkiras1	chr14	19111014	19112745	-0.34	0.03
Plau	chr14	21661560	21662161	-0.63	-0.04
Vcl	chr14	21748654	21748715	-1.30	-0.59
Vcl	chr14	21850830	21851280	-5.31	0.38
Psmc6	chr14	45949498	45950514	-0.68	0.11
Pnp	chr14	51564155	51567563	-2.20	0.60
Pnp	chr14	51571126	51571721	-2.41	0.19
Ctsb	chr14	63761159	63761760	-116.00	-0.21
Clu	chr14	66587437	66590689	-7.01	-0.74
Ppp3cb	chr14	21320302	21327131	-1.02	-0.09
Ppp3cb	chr14	21365614	21365735	-0.60	-0.29

Prkcd	chr14	31408778	31411699	-1.32	-0.09
Prkcd	chr14	31411787	31411937	-0.35	-0.04
Prkcd	chr14	31439303	31439394	-1.67	-0.55
Tspan14	chr14	41720841	41721322	-0.90	-0.19
Ripk3	chr14	56403831	56404011	-4.18	1.03
Myo10	chr15	25552304	25552754	-96.80	-1.21
Myo10	chr15	25709521	25712766	-120.00	-0.72
Myo10	chr15	25736823	25740300	-157.00	-1.07
Myo10	chr15	25741959	25742409	-19.50	-0.50
Myo10	chr15	25743096	25743426	-3.95	-0.37
Ncf4	chr15	78075240	78075447	-1.52	-0.21
Ncf4	chr15	78091438	78093010	-2.87	-0.16
Tab1	chr15	79989200	79992132	-3.29	-0.26
Ep300	chr15	81416643	81438767	-1.28	-0.07
Ep300	chr15	81479273	81481819	-1.93	-0.07
Xrcc6	chr15	81870214	81870424	-0.78	-0.17
Ano6	chr15	95621303	95661131	-1.66	-0.31
Ano6	chr15	95802935	95804972	-5.92	0.19
Atf1	chr15	100084825	100090754	-5.11	0.31
Mettl7a1	chr15	100143493	100143673	-0.64	0.57
Nckap1l	chr15	103328061	103328301	-5.25	-0.34
Ptk2	chr15	73035624	73039772	-0.52	-0.04
Myh9	chr15	77592006	77593481	-52.20	-0.46
Myh9	chr15	77594368	77606283	-414.00	-0.50
Myh9	chr15	77643639	77672545	-3.85	-0.23
Pla2g6	chr15	79116836	79117224	-0.65	-0.14
Nfam1	chr15	82828225	82828824	-1.06	-0.14
Nfam1	chr15	82829452	82831880	-6.49	-0.34
Arsa	chr15	89303025	89303862	-1.00	-0.17
Arsa	chr15	89305988	89306615	-2.33	-0.41
Gpr84	chr15	103138665	103140869	-0.78	-0.04
Hmox2	chr16	4764613	4765867	-0.51	0.03
Mapk1	chr16	17038671	17038882	-0.44	0.05
Snap29	chr16	17427202	17428502	-2.91	0.37
Psmd2	chr16	20651724	20652324	-8.74	-0.32
Cd47	chr16	49867877	49868177	-13.10	0.61
Crebbp	chr16	4084047	4087743	-1.51	-0.07
Crebbp	chr16	4154961	4213404	-1.65	-0.09
Cpped1	chr16	11804530	11827393	-3.40	0.27
Cpped1	chr16	11828497	11828677	-0.40	-0.08
Cpped1	chr16	11887837	11909516	-3.47	-0.88
Pak2	chr16	32019832	32021909	-3.04	0.15
App	chr16	85025115	85043833	-35.70	-0.45

App	chr16	85120877	85173892	-5.24	-0.38
Cct8	chr16	87496053	87496114	-1.39	0.33
Rps6ka2	chr17	7374493	7374644	-1.21	0.96
Rps6ka2	chr17	7506914	7507424	-0.39	-0.14
1600012H06Rik	chr17	15080188	15081447	-11.60	-0.50
Mapk14	chr17	28828286	28828645	-6.50	-0.79
Mapk14	chr17	28884007	28885350	-0.47	0.02
Psmb8	chr17	34335288	34335468	-2.26	-0.35
Neu1	chr17	35071556	35072840	-0.43	0.02
H2-D1	chr17	35400038	35400558	-40.70	-0.54
H2-D1	chr17	35400807	35401046	-2.46	-0.23
H2-D1	chr17	35403816	35404204	-67.80	-0.36
H2-Q2	chr17	35479307	35479659	-0.13	0.17
H2-Q6	chr17	35561821	35564910	-7.70	-0.41
H2-Q6	chr17	35565949	35566691	-0.29	0.01
H2-Q10	chr17	35607880	35610039	-7.41	3.75
Trem1	chr17	48376164	48376373	-0.47	-1.34
Trem1	chr17	48383770	48384842	-0.66	0.48
Vav1	chr17	57418522	57418613	-1.00	-0.44
Vav1	chr17	57467106	57467554	-0.56	0.05
Ypel5	chr17	73198557	73198888	-0.59	0.14
Prkce	chr17	86567124	86568262	-0.44	-0.06
Prkce	chr17	87053997	87055374	-0.59	-0.12
Dynlt1f	chr17	6306339	6306549	-0.36	0.20
Rnaset2b	chr17	8335409	8340667	-8.21	-0.65
Igf2r	chr17	12876409	12876980	-2.88	-0.19
Fpr1	chr17	18013552	18020874	-0.21	-0.18
Mmp25	chr17	23782026	23782176	-0.64	3.81
Pdpk1	chr17	24215553	24216063	-6.49	0.47
Atp6v0c	chr17	24306196	24306374	-0.89	-0.16
H2-K1	chr17	34133105	34133495	-29.30	-0.60
H2-K1	chr17	34136698	34137218	-41.80	-0.78
Hspa1b	chr17	35093821	35096183	-7.51	0.43
Hspa1a	chr17	35107196	35107464	-1.02	3.56
Hspa1a	chr17	35108505	35108744	-1.36	5.33
Tubb5	chr17	35972171	35972588	-27.00	-0.24
H2-T23	chr17	36169129	36248130	-4.86	-0.40
Enpp4	chr17	44234667	44235237	-0.30	0.05
Enpp4	chr17	44236165	44242667	-6.56	1.02
Ppp2r5d	chr17	46820058	46820802	-0.99	-0.12
Ppp2r5d	chr17	46837029	46841951	-0.82	-0.21
Cnpy3	chr17	46872809	46874755	-3.98	-0.22
Ticam1	chr17	56408884	56416190	-14.90	-0.37

Vapa	chr17	65962685	65962895	-2.37	-0.25
Rab31	chr17	66001065	66001632	-0.94	0.07
Rab31	chr17	66002078	66002287	-3.48	-0.28
Rab31	chr17	66003448	66017287	-5.03	0.23
Rab31	chr17	66122031	66122092	-0.23	0.08
Sos1	chr17	80797100	80799097	-0.65	-0.07
Sos1	chr17	80833039	80834290	-0.49	-0.07
Sos1	chr17	80879343	80879554	-0.37	-0.28
Calm2	chr17	87835011	87846216	-1.79	-0.13
Ube2d2a	chr18	35931212	35931392	-0.74	-0.18
Ube2d2a	chr18	35965390	35965540	-0.95	0.36
Malt1	chr18	65635688	65635988	-3.80	-0.39
Map3k8	chr18	4348929	4352951	-0.72	-0.15
Rock1	chr18	10181251	10181790	-0.29	0.00
Tmem173	chr18	35893749	35894258	-1.04	-0.15
Cd14	chr18	36884720	36886308	-12.60	-0.07
Ticam2	chr18	46718782	46719052	-1.30	-0.50
Ticam2	chr18	46719441	46720699	-0.89	-0.13
Nfatc1	chr18	80803003	80803394	-3.39	-0.87
Nfatc1	chr18	80803872	80832717	-0.36	0.02
Nfatc1	chr18	80846232	80846653	-2.89	0.45
Nfatc1	chr18	80879052	80904865	-0.59	-0.05
Unc93b1	chr19	3935215	3935774	-0.37	0.02
Unc93b1	chr19	3936437	3936737	-0.41	0.03
Unc93b1	chr19	3948771	3948951	-21.20	-0.51
Rela	chr19	5646968	5647773	-3.73	-0.21
Pgam1	chr19	41986508	41990163	-5.12	-0.95
Btrc	chr19	45604177	45604358	-1.03	2.52
Nfkb2	chr19	46379346	46380320	-0.71	0.19
Tcirg1	chr19	3906859	3907103	-2.15	-0.38
Aldh3b1	chr19	3913756	3913816	-3.78	-0.66
Tbc1d10c	chr19	4184415	4184595	-0.49	-0.45
Peli3	chr19	4931973	4932510	-0.93	-0.92
Dtx4	chr19	12543910	12544151	-2.93	-0.29
Dtx4	chr19	12560929	12576605	-6.88	-0.19
Ddx3x	chrX	12858147	12861347	-2.40	0.16
Ddx3x	chrX	12868539	12868809	-39.40	0.65
Pgrmc1	chrX	34138515	34138605	-3.07	0.49
Tab3	chrX	82859477	82860195	-3.99	0.50
Tab3	chrX	82860492	82860673	-1.77	0.62
Tab3	chrX	82875804	82876044	-8.19	1.78
Atp7a	chrX	103265124	103280870	-18.90	0.76
Atp7a	chrX	103319725	103320025	-27.70	1.65

Huwe1	chrX	148353134	148354606	-16.40	0.30
Was	chrX	7667556	7667617	-0.56	-0.25
Rap2c	chrX	48359709	48371195	-4.90	0.26
Irak1	chrX	71260386	71261911	-1.24	0.14
Dnase1l1	chrX	71526935	71526996	-0.63	-0.37
Magt1	chrX	103207027	103207148	-2.06	0.47
Gla	chrX	131124401	131124580	-74.80	1.69
Gla	chrX	131135394	131135514	-1.45	0.53
Tlr8	chrX	163680663	163681230	-0.68	0.77
Tlr8	chrX	163681497	163681617	-0.19	0.13
Tlr8	chrX	163681973	163682213	-0.46	-0.65
Tlr8	chrX	163683254	163683344	-0.41	1.28
Tlr8	chrX	163683522	163683672	-0.98	2.22
Tlr7	chrX	163742862	163745489	-33.30	0.39
Tlr7	chrX	163745757	163746354	-1.39	0.09
Diaph1	chr18	38004807	38005077	-9.38	-0.60
Diaph1	chr18	38094885	38095065	-1.72	-0.48

mRNA splicing

gene_name	chr	chromStart	chromEnd	diff.lg.p	diff.log2.fc
Sf3b1	chr1	55084231	55084322	-0.60	-0.19
Dhx9	chr1	155303276	155303397	-3.17	0.68
Hnrnpu	chr1	180259373	180260299	-2.65	-0.09
Hnrnpu	chr1	180267465	180267915	-23.00	-0.29
Cstf3	chr2	104504891	104505372	-10.50	0.78
Ctnnbl1	chr2	157563136	157600285	-2.55	-0.24
Srsf6	chr2	162757243	162757817	-0.60	-0.04
Cstf1	chr2	172203213	172206586	-1.68	-0.11
Rbm17	chr2	11522350	11524826	-0.61	-0.12
Prpf40a	chr2	53049040	53050262	-1.20	0.19
Cwc22	chr2	77733949	77741916	-1.28	-0.10
Clp1	chr2	84563548	84566299	-1.88	0.17
Snrpb	chr2	130002885	130005100	-1.37	-0.14
Sf3b4	chr3	95976502	95978290	-10.70	-0.33
Rbm8a	chr3	96433850	96433910	-0.48	0.21
Bcas2	chr3	102982305	102982484	-1.47	0.15
Prcc	chr3	87662854	87664130	-1.39	-0.16
Prcc	chr3	87673527	87689484	-9.87	-0.31
Srsf11	chr3	157674772	157675808	-4.18	-0.15
Prpf4	chr4	62083697	62085481	-2.54	0.23
Srsf4	chr4	131453781	131457610	-4.89	-0.19
Dnajc8	chr4	132108975	132109154	-0.70	-0.07
Syf2	chr4	134486894	134490903	-18.00	-0.50
Syf2	chr4	134493303	134493452	-1.58	-0.40

Hnrnpr	chr4	135895398	135895996	-0.45	0.04
Ybx1	chr4	118957701	118967118	-53.20	-0.42
Ppih	chr4	118979838	118980467	-0.40	0.06
Srrm1	chr4	134878175	134878385	-1.96	-0.19
Srrm1	chr4	134880747	134881048	-27.70	-0.57
Srrm1	chr4	134902739	134909069	-0.74	0.09
Snrnp35	chr5	124933163	124940954	-0.80	-0.10
Bud31	chr5	145908765	145908943	-0.74	-0.11
Cpsf4	chr5	145939775	145942670	-0.46	0.04
Hnrnpf	chr6	117850506	117856962	-1.09	-0.18
Hnrnpf	chr6	117873756	117874265	-2.10	-0.15
Hnrnpf	chr6	117874623	117874893	-13.30	-0.40
Gm8994	chr6	136279021	136279082	-0.20	-0.85
Gm8994	chr6	136279763	136279882	-0.28	0.95
Hnrnpa2b1	chr6	51419713	51419893	-0.88	-0.08
Usp39	chr6	72268788	72269207	-3.44	0.38
Usp39	chr6	72294959	72295169	-8.67	-0.94
Pcbp1	chr6	86474877	86476159	-6.49	0.07
Isy1	chr6	87769040	87769634	-1.43	-0.23
Wbp11	chr6	136762473	136764721	-13.60	-0.37
Prpf31	chr7	3581586	3582328	-3.35	-0.27
Prpf31	chr7	3591293	3593127	-5.65	-0.22
U2af2	chr7	5013783	5013844	-0.45	0.13
U2af2	chr7	5030853	5031123	-0.52	-0.04
Snrpd2	chr7	19735186	19736653	-0.98	-0.16
Polr2i	chr7	31017332	31017529	-0.50	-0.12
Snrpa1	chr7	73205399	73205519	-3.94	-0.73
Hnrnpul1	chr7	26533405	26539769	-24.20	-0.34
Snrnp70	chr7	52631823	52636129	-42.80	-0.34
Snrnp70	chr7	52647692	52651017	-6.22	-0.19
Pcf11	chr7	99793088	99795438	-30.30	1.25
Pcf11	chr7	99801691	99808380	-0.63	-0.03
Pcf11	chr7	99809597	99810434	-7.78	0.23
Pcf11	chr7	99818003	99818422	-5.65	-0.42
Cd2bp2	chr7	134337180	134338617	-12.70	-0.29
Sugp1	chr8	72566920	72572439	-0.31	-0.03
Polr2c	chr8	97387514	97387634	-3.83	-0.48
Elavl1	chr8	4289159	4289430	-2.72	-0.30
Dhx38	chr8	112071923	112073723	-0.63	0.08
Dhx38	chr8	112080084	112080835	-5.29	0.46
Dhx38	chr8	112084583	112089501	-5.62	0.27
Sf3b3	chr8	113334571	113334842	-0.99	0.06
Ppil4	chr10	7534516	7541105	-2.25	-0.26

Sf3b5	chr10	12728255	12728989	-12.30	-0.33
Sf3a2	chr10	80261479	80263960	-1.32	-0.33
Ccar1	chr10	62208074	62209806	-0.97	0.09
Ccar1	chr10	62214563	62223000	-7.87	0.33
Polr2e	chr10	79501286	79502404	-1.51	-0.21
Sf3a1	chr11	4080296	4080657	-1.02	-0.15
Zmat5	chr11	4604673	4628589	-1.66	-0.52
Snrnp25	chr11	32105414	32105652	-1.18	-0.36
Hnrnph1	chr11	50191247	50191914	-2.25	0.25
Srsf1	chr11	87861172	87861352	-2.81	-0.34
Ddx42	chr11	106078239	106085433	-7.43	-0.79
Ddx42	chr11	106108808	106109766	-11.90	-0.37
Cwc25	chr11	97608042	97614061	-2.33	0.21
Eftud2	chr11	102699785	102700145	-2.70	0.23
Ddx5	chr11	106650118	106650179	-4.48	1.68
Srsf2	chr11	116714018	116714408	-55.20	-0.59
Srsf5	chr12	82046490	82046729	-9.58	-0.34
Srsf5	chr12	82050810	82051078	-60.20	-0.75
Cpsf2	chr12	103240910	103241994	-4.02	0.21
Papola	chr12	107022971	107023122	-4.63	-0.32
Papola	chr12	107072945	107075506	-1.04	0.06
Snw1	chr12	88791126	88791810	-6.17	-0.30
Snw1	chr12	88794816	88796541	-6.59	-0.48
Ddx46	chr13	55736387	55736448	-1.12	-0.40
Ddx46	chr13	55739545	55746827	-9.45	-0.38
Hnrnpa0	chr13	58228280	58229917	-53.20	-0.32
Hnrnpk	chr13	58501349	58504674	-0.47	-0.03
Ppwd1	chr13	105007352	105010559	-4.01	0.41
Ppwd1	chr13	105018773	105018924	-0.38	0.08
Gtf2f2	chr14	76297012	76306621	-0.38	0.02
Wbp4	chr14	79861569	79863753	-10.60	-0.73
Puf60	chr15	75900611	75900909	-0.74	-0.07
Ddx23	chr15	98475937	98476325	-7.92	-0.39
Ddx23	chr15	98488768	98493320	-3.79	-0.37
Ncbp2	chr16	31957240	31957450	-1.88	-0.32
Tra2b	chr16	22265822	22266002	-1.45	-0.29
Srrm2	chr17	23952780	23953141	-27.50	-0.34
Srrm2	chr17	23953680	23955569	-73.20	0.27
Srrm2	chr17	23957516	23958056	-0.44	-0.01
Rnps1	chr17	24551619	24552103	-0.56	-0.11
Dhx16	chr17	36019018	36019620	-7.24	-0.56
Ppil1	chr17	29387927	29388668	-2.42	-0.27
Hnrnpm	chr17	33786662	33789227	-3.10	-0.13

Hnrnpm	chr17	33814217	33822403	-0.43	0.02
Cdc5l	chr17	45528987	45530093	-0.78	-0.11
Cdc5l	chr17	45564979	45570477	-1.28	-0.56
Srsf7	chr17	80606178	80606645	-0.44	-0.07
Snrpd1	chr18	10617823	10623647	-0.37	0.04
Polr2d	chr18	31955847	31956355	-3.53	0.44
Wdr33	chr18	31963710	31986988	-16.20	-0.82
Wdr33	chr18	32047456	32048936	-6.56	-0.33
Wdr33	chr18	32049204	32067143	-0.39	0.01
Txnl4a	chr18	80403957	80409422	-1.96	0.06
Txnl4a	chr18	80415452	80419332	-0.42	-0.02
Txnl4a	chr18	80421035	80421873	-1.14	-0.40
Cpsf7	chr19	10614163	10620757	-1.15	-0.06
Prpf19	chr19	10969781	10969872	-1.39	-0.35
Cstf2t	chr19	31157360	31159177	-3.62	0.14
Sart1	chr19	5381212	5381698	-0.52	-0.07
Sart1	chr19	5384882	5388703	-4.07	-0.28
Polr2g	chr19	8872986	8873047	-0.42	-0.12
Smndc1	chr19	53454959	53455080	-0.47	0.05
Gpkow	chrX	7285271	7285480	-3.00	0.59
Cstf2	chrX	130609104	130609225	-0.88	0.22
Hnrnph2	chrX	131137272	131140187	-32.60	0.52
Upf3b	chrX	34632248	34635529	-1.00	-0.19
Upf3b	chrX	34635618	34635799	-3.11	0.64
Zrsr2	chrX	160374663	160374874	-0.55	-0.14
Zrsr2	chrX	160396297	160396568	-0.44	-0.07

Processing of capped intro-containing pre-mRNA

gene_name	chr	chromStart	chromEnd	diff.lg.p	diff.log2.fc
Sf3b1	chr1	55084231	55084322	-0.60	-0.19
Zc3h11a	chr1	135516447	135517433	-0.86	0.06
Zc3h11a	chr1	135517641	135518703	-1.96	0.10
Zc3h11a	chr1	135521340	135523811	-0.72	0.04
Dhx9	chr1	155303276	155303397	-3.17	0.68
Hnrnpu	chr1	180259373	180260299	-2.65	-0.09
Hnrnpu	chr1	180267465	180267915	-23.00	-0.29
Nup214	chr2	31888785	31893623	-1.12	-0.08
Nup214	chr2	31907789	31907850	-0.40	-0.11
Cstf3	chr2	104504891	104505372	-10.50	0.78
Nxt1	chr2	148498350	148501762	-0.48	-0.03
Ctnnbl1	chr2	157563136	157600285	-2.55	-0.24
Srsf6	chr2	162757243	162757817	-0.60	-0.04
Cstf1	chr2	172203213	172206586	-1.68	-0.11
Rae1	chr2	172839488	172841203	-3.81	-0.39

Rbm17	chr2	11522350	11524826	-0.61	-0.12
Prpf40a	chr2	53049040	53050262	-1.20	0.19
Cwc22	chr2	77733949	77741916	-1.28	-0.10
Clp1	chr2	84563548	84566299	-1.88	0.17
Snrpb	chr2	130002885	130005100	-1.37	-0.14
Sf3b4	chr3	95976502	95978290	-10.70	-0.33
Rbm8a	chr3	96433850	96433910	-0.48	0.21
Bcas2	chr3	102982305	102982484	-1.47	0.15
Prcc	chr3	87662854	87664130	-1.39	-0.16
Prcc	chr3	87673527	87689484	-9.87	-0.31
Chtop	chr3	90303623	90304012	-0.38	0.01
Chtop	chr3	90313185	90313365	-0.85	-0.09
Srsf11	chr3	157674772	157675808	-4.18	-0.15
Prpf4	chr4	62083697	62085481	-2.54	0.23
Ndc1	chr4	107083754	107086943	-5.63	0.52
Srsf4	chr4	131453781	131457610	-4.89	-0.19
Dnajc8	chr4	132108975	132109154	-0.70	-0.07
Syf2	chr4	134486894	134490903	-18.00	-0.50
Syf2	chr4	134493303	134493452	-1.58	-0.40
Hnrnpr	chr4	135895398	135895996	-0.45	0.04
Ybx1	chr4	118957701	118967118	-53.20	-0.42
Ppih	chr4	118979838	118980467	-0.40	0.06
Srrm1	chr4	134878175	134878385	-1.96	-0.19
Srrm1	chr4	134880747	134881048	-27.70	-0.57
Srrm1	chr4	134902739	134909069	-0.74	0.09
Nupl2	chr5	23688056	23689616	-0.80	0.11
Snrnp35	chr5	124933163	124940954	-0.80	-0.10
Bud31	chr5	145908765	145908943	-0.74	-0.11
Cpsf4	chr5	145939775	145942670	-0.46	0.04
Nup54	chr5	92845101	92846543	-0.74	0.09
Pom121	chr5	135853620	135857168	-10.60	-0.41
Pom121	chr5	135857555	135857765	-9.09	-0.71
Nup205	chr6	35127615	35133895	-0.46	0.13
Hnrnpf	chr6	117850506	117856962	-1.09	-0.18
Hnrnpf	chr6	117873756	117874265	-2.10	-0.15
Hnrnpf	chr6	117874623	117874893	-13.30	-0.40
Gm8994	chr6	136279021	136279082	-0.20	-0.85
Gm8994	chr6	136279763	136279882	-0.28	0.95
Hnrnpa2b1	chr6	51419713	51419893	-0.88	-0.08
Usp39	chr6	72268788	72269207	-3.44	0.38
Usp39	chr6	72294959	72295169	-8.67	-0.94
Pcbp1	chr6	86474877	86476159	-6.49	0.07
Isy1	chr6	87769040	87769634	-1.43	-0.23

Nup210	chr6	90963060	90963630	-0.69	-0.12
Wbp11	chr6	136762473	136764721	-13.60	-0.37
Prpf31	chr7	3581586	3582328	-3.35	-0.27
Prpf31	chr7	3591293	3593127	-5.65	-0.22
U2af2	chr7	5013783	5013844	-0.45	0.13
U2af2	chr7	5030853	5031123	-0.52	-0.04
Snrpd2	chr7	19735186	19736653	-0.98	-0.16
Polr2i	chr7	31017332	31017529	-0.50	-0.12
Nup62	chr7	52071739	52086176	-1.81	-0.05
Snrpa1	chr7	73205399	73205519	-3.94	-0.73
Hnrnpul1	chr7	26533405	26539769	-24.20	-0.34
Snrnp70	chr7	52631823	52636129	-42.80	-0.34
Snrnp70	chr7	52647692	52651017	-6.22	-0.19
Pcf11	chr7	99793088	99795438	-30.30	1.25
Pcf11	chr7	99801691	99808380	-0.63	-0.03
Pcf11	chr7	99809597	99810434	-7.78	0.23
Pcf11	chr7	99818003	99818422	-5.65	-0.42
Nup98	chr7	109268003	109269174	-0.67	-0.03
Nup98	chr7	109283070	109283401	-11.60	-0.60
Cd2bp2	chr7	134337180	134338617	-12.70	-0.29
Sugp1	chr8	72566920	72572439	-0.31	-0.03
Polr2c	chr8	97387514	97387634	-3.83	-0.48
Elavl1	chr8	4289159	4289430	-2.72	-0.30
Dhx38	chr8	112071923	112073723	-0.63	0.08
Dhx38	chr8	112080084	112080835	-5.29	0.46
Dhx38	chr8	112084583	112089501	-5.62	0.27
Sf3b3	chr8	113334571	113334842	-0.99	0.06
Ppil4	chr10	7534516	7541105	-2.25	-0.26
Sf3b5	chr10	12728255	12728989	-12.30	-0.33
Ranbp2	chr10	57939345	57940486	-28.40	0.48
Ranbp2	chr10	57940754	57941085	-10.30	0.60
Ranbp2	chr10	57941534	57941955	-2.61	0.14
Ranbp2	chr10	57942164	57945874	-47.50	0.47
Ranbp2	chr10	57955388	57956782	-14.50	0.43
Sf3a2	chr10	80261479	80263960	-1.32	-0.33
Ccar1	chr10	62208074	62209806	-0.97	0.09
Ccar1	chr10	62214563	62223000	-7.87	0.33
Polr2e	chr10	79501286	79502404	-1.51	-0.21
Sf3a1	chr11	4080296	4080657	-1.02	-0.15
Zmat5	chr11	4604673	4628589	-1.66	-0.52
Thoc5	chr11	4799846	4799907	-2.53	-0.61
Snrnp25	chr11	32105414	32105652	-1.18	-0.36
Slu7	chr11	43247232	43250977	-0.69	-0.10

Slu7	chr11	43258883	43259812	-6.75	-0.39
Hnrnph1	chr11	50191247	50191914	-2.25	0.25
Srsf1	chr11	87861172	87861352	-2.81	-0.34
Casc3	chr11	98683799	98689940	-7.62	-0.37
Casc3	chr11	98692883	98694014	-1.36	-0.17
Ddx42	chr11	106078239	106085433	-7.43	-0.79
Ddx42	chr11	106108808	106109766	-11.90	-0.37
Nup88	chr11	70783414	70783475	-0.56	0.19
Cwc25	chr11	97608042	97614061	-2.33	0.21
Eftud2	chr11	102699785	102700145	-2.70	0.23
Ddx5	chr11	106650118	106650179	-4.48	1.68
Srsf2	chr11	116714018	116714408	-55.20	-0.59
Srsf5	chr12	82046490	82046729	-9.58	-0.34
Srsf5	chr12	82050810	82051078	-60.20	-0.75
Cpsf2	chr12	103240910	103241994	-4.02	0.21
Papola	chr12	107022971	107023122	-4.63	-0.32
Papola	chr12	107072945	107075506	-1.04	0.06
Snw1	chr12	88791126	88791810	-6.17	-0.30
Snw1	chr12	88794816	88796541	-6.59	-0.48
Ddx46	chr13	55736387	55736448	-1.12	-0.40
Ddx46	chr13	55739545	55746827	-9.45	-0.38
Nup153	chr13	46776228	46776529	-1.65	0.22
Nup153	chr13	46782010	46792068	-8.48	0.21
Nup153	chr13	46822738	46823218	-8.20	-0.51
Thoc3	chr13	54561210	54561569	-0.37	0.03
Hnrnpa0	chr13	58228280	58229917	-53.20	-0.32
Hnrnpk	chr13	58501349	58504674	-0.47	-0.03
Ppwd1	chr13	105007352	105010559	-4.01	0.41
Ppwd1	chr13	105018773	105018924	-0.38	0.08
Gtf2f2	chr14	76297012	76306621	-0.38	0.02
Wbp4	chr14	79861569	79863753	-10.60	-0.73
Nup50	chr15	84757805	84765852	-2.65	-0.15
Nup50	chr15	84770131	84771000	-1.89	-0.14
Nup50	chr15	84773003	84773303	-4.39	0.58
Puf60	chr15	75900611	75900909	-0.74	-0.07
Poldip3	chr15	82958053	82959641	-13.20	-0.35
Ddx23	chr15	98475937	98476325	-7.92	-0.39
Ddx23	chr15	98488768	98493320	-3.79	-0.37
Ncbp2	chr16	31957240	31957450	-1.88	-0.32
Fyttd1	chr16	32877869	32878080	-0.83	-0.18
Fyttd1	chr16	32902586	32905812	-1.29	-0.23
Tra2b	chr16	22265822	22266002	-1.45	-0.29
Srrm2	chr17	23952780	23953141	-27.50	-0.34

Srrm2	chr17	23953680	23955569	-73.20	0.27
Srrm2	chr17	23957516	23958056	-0.44	-0.01
Rnps1	chr17	24551619	24552103	-0.56	-0.11
Ddx39b	chr17	35378720	35378917	-2.01	-0.34
Ddx39b	chr17	35386162	35386790	-5.14	-0.25
Ddx39b	chr17	35566011	35566699	-0.29	-0.01
Dhx16	chr17	36019018	36019620	-7.24	-0.56
Wtap	chr17	13160263	13163915	-3.74	-0.20
Wtap	chr17	13167327	13168316	-4.95	0.37
Thoc6	chr17	23810678	23810831	-0.28	-0.01
Ppil1	chr17	29387927	29388668	-2.42	-0.27
Hnrnpm	chr17	33786662	33789227	-3.10	-0.13
Hnrnpm	chr17	33814217	33822403	-0.43	0.02
Cdc5l	chr17	45528987	45530093	-0.78	-0.11
Cdc5l	chr17	45564979	45570477	-1.28	-0.56
Srsf7	chr17	80606178	80606645	-0.44	-0.07
Thoc1	chr18	9993386	9993537	-0.43	0.05
Snrpd1	chr18	10617823	10623647	-0.37	0.04
Polr2d	chr18	31955847	31956355	-3.53	0.44
Wdr33	chr18	31963710	31986988	-16.20	-0.82
Wdr33	chr18	32047456	32048936	-6.56	-0.33
Wdr33	chr18	32049204	32067143	-0.39	0.01
Txnl4a	chr18	80403957	80409422	-1.96	0.06
Txnl4a	chr18	80415452	80419332	-0.42	-0.02
Txnl4a	chr18	80421035	80421873	-1.14	-0.40
Tmem223	chr19	8846725	8846845	-2.14	1.40
Cpsf7	chr19	10614163	10620757	-1.15	-0.06
Prpf19	chr19	10969781	10969872	-1.39	-0.35
Cstf2t	chr19	31157360	31159177	-3.62	0.14
Sart1	chr19	5381212	5381698	-0.52	-0.07
Sart1	chr19	5384882	5388703	-4.07	-0.28
Polr2g	chr19	8872986	8873047	-0.42	-0.12
Smndc1	chr19	53454959	53455080	-0.47	0.05
Gpkow	chrX	7285271	7285480	-3.00	0.59
Cstf2	chrX	130609104	130609225	-0.88	0.22
Hnrnph2	chrX	131137272	131140187	-32.60	0.52
Upf3b	chrX	34632248	34635529	-1.00	-0.19
Upf3b	chrX	34635618	34635799	-3.11	0.64
Thoc2	chrX	39150565	39150955	-10.10	0.73
Zrsr2	chrX	160374663	160374874	-0.55	-0.14
Zrsr2	chrX	160396297	160396568	-0.44	-0.07

mRNA splicing-major pathway

gene_name	chr	chromStart	chromEnd	diff.lg.p	diff.log2.fc

Sf3b1	chr1	55084231	55084322	-0.60	-0.19
Dhx9	chr1	155303276	155303397	-3.17	0.68
Hnrnpu	chr1	180259373	180260299	-2.65	-0.09
Hnrnpu	chr1	180267465	180267915	-23.00	-0.29
Cstf3	chr2	104504891	104505372	-10.50	0.78
Ctnnbl1	chr2	157563136	157600285	-2.55	-0.24
Srsf6	chr2	162757243	162757817	-0.60	-0.04
Cstf1	chr2	172203213	172206586	-1.68	-0.11
Rbm17	chr2	11522350	11524826	-0.61	-0.12
Prpf40a	chr2	53049040	53050262	-1.20	0.19
Cwc22	chr2	77733949	77741916	-1.28	-0.10
Clp1	chr2	84563548	84566299	-1.88	0.17
Snrpb	chr2	130002885	130005100	-1.37	-0.14
Sf3b4	chr3	95976502	95978290	-10.70	-0.33
Rbm8a	chr3	96433850	96433910	-0.48	0.21
Bcas2	chr3	102982305	102982484	-1.47	0.15
Prcc	chr3	87662854	87664130	-1.39	-0.16
Prcc	chr3	87673527	87689484	-9.87	-0.31
Srsf11	chr3	157674772	157675808	-4.18	-0.15
Prpf4	chr4	62083697	62085481	-2.54	0.23
Srsf4	chr4	131453781	131457610	-4.89	-0.19
Dnajc8	chr4	132108975	132109154	-0.70	-0.07
Syf2	chr4	134486894	134490903	-18.00	-0.50
Syf2	chr4	134493303	134493452	-1.58	-0.40
Hnrnpr	chr4	135895398	135895996	-0.45	0.04
Ybx1	chr4	118957701	118967118	-53.20	-0.42
Ppih	chr4	118979838	118980467	-0.40	0.06
Srrm1	chr4	134878175	134878385	-1.96	-0.19
Srrm1	chr4	134880747	134881048	-27.70	-0.57
Srrm1	chr4	134902739	134909069	-0.74	0.09
Bud31	chr5	145908765	145908943	-0.74	-0.11
Cpsf4	chr5	145939775	145942670	-0.46	0.04
Hnrnpf	chr6	117850506	117856962	-1.09	-0.18
Hnrnpf	chr6	117873756	117874265	-2.10	-0.15
Hnrnpf	chr6	117874623	117874893	-13.30	-0.40
Gm8994	chr6	136279021	136279082	-0.20	-0.85
Gm8994	chr6	136279763	136279882	-0.28	0.95
Hnrnpa2b1	chr6	51419713	51419893	-0.88	-0.08
Usp39	chr6	72268788	72269207	-3.44	0.38
Usp39	chr6	72294959	72295169	-8.67	-0.94
Pcbp1	chr6	86474877	86476159	-6.49	0.07
Isy1	chr6	87769040	87769634	-1.43	-0.23
Wbp11	chr6	136762473	136764721	-13.60	-0.37

Prpf31	chr7	3581586	3582328	-3.35	-0.27
Prpf31	chr7	3591293	3593127	-5.65	-0.22
U2af2	chr7	5013783	5013844	-0.45	0.13
U2af2	chr7	5030853	5031123	-0.52	-0.04
Snrpd2	chr7	19735186	19736653	-0.98	-0.16
Polr2i	chr7	31017332	31017529	-0.50	-0.12
Snrpa1	chr7	73205399	73205519	-3.94	-0.73
Hnrnpul1	chr7	26533405	26539769	-24.20	-0.34
Snrnp70	chr7	52631823	52636129	-42.80	-0.34
Snrnp70	chr7	52647692	52651017	-6.22	-0.19
Pcf11	chr7	99793088	99795438	-30.30	1.25
Pcf11	chr7	99801691	99808380	-0.63	-0.03
Pcf11	chr7	99809597	99810434	-7.78	0.23
Pcf11	chr7	99818003	99818422	-5.65	-0.42
Cd2bp2	chr7	134337180	134338617	-12.70	-0.29
Sugp1	chr8	72566920	72572439	-0.31	-0.03
Polr2c	chr8	97387514	97387634	-3.83	-0.48
Elavl1	chr8	4289159	4289430	-2.72	-0.30
Dhx38	chr8	112071923	112073723	-0.63	0.08
Dhx38	chr8	112080084	112080835	-5.29	0.46
Dhx38	chr8	112084583	112089501	-5.62	0.27
Sf3b3	chr8	113334571	113334842	-0.99	0.06
Ppil4	chr10	7534516	7541105	-2.25	-0.26
Sf3b5	chr10	12728255	12728989	-12.30	-0.33
Sf3a2	chr10	80261479	80263960	-1.32	-0.33
Ccar1	chr10	62208074	62209806	-0.97	0.09
Ccar1	chr10	62214563	62223000	-7.87	0.33
Polr2e	chr10	79501286	79502404	-1.51	-0.21
Sf3a1	chr11	4080296	4080657	-1.02	-0.15
Hnrnph1	chr11	50191247	50191914	-2.25	0.25
Srsf1	chr11	87861172	87861352	-2.81	-0.34
Ddx42	chr11	106078239	106085433	-7.43	-0.79
Ddx42	chr11	106108808	106109766	-11.90	-0.37
Cwc25	chr11	97608042	97614061	-2.33	0.21
Eftud2	chr11	102699785	102700145	-2.70	0.23
Ddx5	chr11	106650118	106650179	-4.48	1.68
Srsf2	chr11	116714018	116714408	-55.20	-0.59
Srsf5	chr12	82046490	82046729	-9.58	-0.34
Srsf5	chr12	82050810	82051078	-60.20	-0.75
Cpsf2	chr12	103240910	103241994	-4.02	0.21
Papola	chr12	107022971	107023122	-4.63	-0.32
Papola	chr12	107072945	107075506	-1.04	0.06
Snw1	chr12	88791126	88791810	-6.17	-0.30

Snw1	chr12	88794816	88796541	-6.59	-0.48
Ddx46	chr13	55736387	55736448	-1.12	-0.40
Ddx46	chr13	55739545	55746827	-9.45	-0.38
Hnrnpa0	chr13	58228280	58229917	-53.20	-0.32
Hnrnpk	chr13	58501349	58504674	-0.47	-0.03
Ppwd1	chr13	105007352	105010559	-4.01	0.41
Ppwd1	chr13	105018773	105018924	-0.38	0.08
Gtf2f2	chr14	76297012	76306621	-0.38	0.02
Wbp4	chr14	79861569	79863753	-10.60	-0.73
Puf60	chr15	75900611	75900909	-0.74	-0.07
Ddx23	chr15	98475937	98476325	-7.92	-0.39
Ddx23	chr15	98488768	98493320	-3.79	-0.37
Ncbp2	chr16	31957240	31957450	-1.88	-0.32
Tra2b	chr16	22265822	22266002	-1.45	-0.29
Srrm2	chr17	23952780	23953141	-27.50	-0.34
Srrm2	chr17	23953680	23955569	-73.20	0.27
Srrm2	chr17	23957516	23958056	-0.44	-0.01
Rnps1	chr17	24551619	24552103	-0.56	-0.11
Dhx16	chr17	36019018	36019620	-7.24	-0.56
Ppil1	chr17	29387927	29388668	-2.42	-0.27
Hnrnpm	chr17	33786662	33789227	-3.10	-0.13
Hnrnpm	chr17	33814217	33822403	-0.43	0.02
Cdc5l	chr17	45528987	45530093	-0.78	-0.11
Cdc5l	chr17	45564979	45570477	-1.28	-0.56
Srsf7	chr17	80606178	80606645	-0.44	-0.07
Snrpd1	chr18	10617823	10623647	-0.37	0.04
Polr2d	chr18	31955847	31956355	-3.53	0.44
Wdr33	chr18	31963710	31986988	-16.20	-0.82
Wdr33	chr18	32047456	32048936	-6.56	-0.33
Wdr33	chr18	32049204	32067143	-0.39	0.01
Txnl4a	chr18	80403957	80409422	-1.96	0.06
Txnl4a	chr18	80415452	80419332	-0.42	-0.02
Txnl4a	chr18	80421035	80421873	-1.14	-0.40
Cpsf7	chr19	10614163	10620757	-1.15	-0.06
Prpf19	chr19	10969781	10969872	-1.39	-0.35
Cstf2t	chr19	31157360	31159177	-3.62	0.14
Sart1	chr19	5381212	5381698	-0.52	-0.07
Sart1	chr19	5384882	5388703	-4.07	-0.28
Polr2g	chr19	8872986	8873047	-0.42	-0.12
Smndc1	chr19	53454959	53455080	-0.47	0.05
Gpkow	chrX	7285271	7285480	-3.00	0.59
Cstf2	chrX	130609104	130609225	-0.88	0.22
Hnrnph2	chrX	131137272	131140187	-32.60	0.52

Upf3b	chrX	34632248	34635529	-1.00	-0.19
Upf3b	chrX	34635618	34635799	-3.11	0.64

Class I MHC mediated antigen processing & presentation

gene_name	chr	chromStart	chromEnd	diff.lg.p	diff.log2.fc
Psmd1	chr1	87961193	87961254	-0.37	-0.11
Ube2f	chr1	93182033	93182602	-1.08	-0.09
Asb1	chr1	93440810	93443711	-1.60	-0.49
Asb1	chr1	93451373	93451734	-0.52	0.13
Ncf2	chr1	154654984	154655314	-0.34	0.01
Hecw2	chr1	53869614	53883756	-1.23	1.77
Hecw2	chr1	53982375	53983924	-0.31	-0.08
Rnf25	chr1	74640325	74640975	-3.60	-0.28
Cul3	chr1	80263601	80265851	-7.81	0.36
Cul3	chr1	80336855	80337005	-0.41	-0.05
Trip12	chr1	84721027	84722028	-8.53	0.37
Trip12	chr1	84782695	84836815	-0.84	-0.03
Klhl20	chr1	163019047	163019258	-2.02	-0.55
Klhl20	chr1	163020426	163023835	-3.23	0.59
Klhl20	chr1	163039327	163039897	-0.46	0.06
Anapc2	chr2	25128463	25128941	-1.02	-0.11
Fbxw5	chr2	25358984	25359776	-1.40	-0.18
Fbxw5	chr2	25360013	25360422	-1.11	-0.22
Hspa5	chr2	34629598	34629837	-1.43	0.04
Hspa5	chr2	34630750	34631960	-2.77	-0.03
Klhl41	chr2	69508654	69508893	-1.20	0.67
Itgav	chr2	83564583	83565003	-11.70	-0.93
Itgav	chr2	83643447	83643718	-4.06	-0.49
Itgav	chr2	83643777	83643958	-1.72	-0.33
Psmc3	chr2	90894439	90895182	-2.45	-0.16
Pdia3	chr2	121263353	121263680	-7.81	0.20
B2m	chr2	121973422	121973632	-3.80	-0.13
Trim69	chr2	122004226	122004674	-0.56	-1.46
Itch	chr2	154959216	154959307	-0.30	0.07
Itch	chr2	155050132	155050403	-0.62	-0.11
Rnf114	chr2	167318622	167318832	-0.88	-1.10
Asb6	chr2	30678617	30679897	-9.32	-0.39
Lrsam1	chr2	32781993	32782383	-0.52	-0.14
Fbxw2	chr2	34661048	34661869	-2.35	-0.23
Fbxw2	chr2	34662794	34664040	-0.82	-0.21
Fbxw2	chr2	34668436	34678672	-0.41	-0.02
Fbxw2	chr2	34681468	34681725	-0.52	0.16
Psmd5	chr2	34707993	34709823	-0.82	-0.10
Psmd5	chr2	34726274	34726482	-3.28	-0.70

Psmb7	chr2	38498946	38499426	-1.70	-0.24
Anapc1	chr2	128436537	128438637	-0.86	0.07
Ubox5	chr2	130416996	130417596	-0.43	0.07
Ubox5	chr2	130425355	130426375	-3.53	-0.43
Psmf1	chr2	151543679	151544068	-0.32	0.02
Fbxw7	chr3	84619498	84694070	-0.58	0.12
Fbxw7	chr3	84694129	84756226	-2.67	0.26
Fbxw7	chr3	84780140	84781955	-0.67	0.08
Ube2q1	chr3	89577560	89577890	-1.31	-0.19
Ube2q1	chr3	89585705	89587919	-2.90	0.11
Rnf115	chr3	96593799	96594008	-1.03	-0.13
Sec22b	chr3	97705149	97705328	-5.43	-0.64
Sec22b	chr3	97725140	97725438	-4.07	-0.31
Ube2d3	chr3	135101656	135102228	-6.45	-0.41
Siah2	chr3	58479649	58495411	-0.38	-0.02
Siah2	chr3	58495680	58495891	-0.39	-0.11
Psmb4	chr3	94690551	94690880	-2.31	-0.17
Psmd4	chr3	94836626	94836894	-5.68	-0.27
Psmd4	chr3	94839816	94846467	-4.83	-0.24
Fcgr1	chr3	96087757	96088506	-3.70	-0.43
Sec24b	chr3	129686102	129687101	-1.10	-0.09
Sec24b	chr3	129743865	129763675	-17.10	-0.55
Fbxl4	chr4	22303743	22313289	-6.40	-0.70
Ube2r2	chr4	41137807	41138136	-0.82	-0.10
Trim32	chr4	65274556	65274646	-0.66	-0.27
Trim32	chr4	65274824	65276139	-4.40	0.35
Lrrc41	chr4	115761466	115762183	-9.56	-0.32
Lrrc41	chr4	115768961	115769201	-3.42	-0.29
Rnf19b	chr4	128735514	128735993	-14.30	-0.99
Rnf19b	chr4	128760028	128761535	-86.40	-1.29
Klhl21	chr4	151383179	151383749	-6.64	-1.09
Klhl21	chr4	151383838	151389959	-1.59	-0.17
Klhl21	chr4	151390257	151390408	-2.24	0.94
Ube2j2	chr4	155330537	155331768	-5.83	-0.33
Ufl1	chr4	25177588	25177679	-0.29	-0.02
Fbxo10	chr4	45048105	45048374	-0.54	-0.64
Fbxo10	chr4	45048672	45048852	-0.34	0.51
Fbxo10	chr4	45071353	45071981	-0.56	-0.37
Cdc26	chr4	62055609	62055968	-0.83	0.27
Klhl9	chr4	88365989	88367814	-20.30	0.34
Rnf220	chr4	116961136	116968749	-1.18	-0.20
Fbxo6	chr4	147520122	147520183	-0.29	0.01
Spsb1	chr4	149271976	149281277	-0.37	-0.15

Mib2	chr4	155028700	155028761	-1.11	-1.14
Ube3c	chr5	29895781	29895962	-1.11	-0.74
Ube3c	chr5	30001057	30001298	-0.35	0.03
Rnf4	chr5	34694045	34694166	-4.54	-0.45
Anapc4	chr5	53257704	53257973	-1.19	-0.20
Klhl5	chr5	65558241	65558691	-1.07	-0.19
Ube3b	chr5	114869623	114869922	-2.59	-0.34
Fbxo21	chr5	118452093	118458648	-2.38	-0.37
Anapc7	chr5	122891328	122891508	-1.48	-0.33
Anapc7	chr5	122893875	122894502	-3.76	-0.41
Rnf34	chr5	123273869	123314504	-4.17	0.34
Psmd9	chr5	123678228	123678437	-1.01	0.20
Kctd7	chr5	130628217	130628755	-1.29	-0.61
Fbxl5	chr5	44142150	44149098	-0.30	0.01
Fbxl5	chr5	44149396	44151063	-1.01	0.09
Rchy1	chr5	92391795	92392094	-2.32	0.64
Sec31a	chr5	100790907	100791058	-1.68	-0.19
Fbxw8	chr5	118515497	118515767	-0.43	-0.12
Fbxw8	chr5	118605137	118605467	-8.06	-0.80
Anapc5	chr5	123271138	123271348	-1.70	-0.25
Ubc	chr5	125866482	125866751	-7.45	-0.16
Ubc	chr5	125867492	125867701	-3.77	1.98
Ubc	chr5	125867967	125870328	-2.39	0.10
Ncf1	chr5	134697332	134697663	-8.46	-0.28
Fbxl18	chr5	143633766	143634036	-4.20	-0.80
Fbxl18	chr5	143640471	143644134	-2.38	-0.99
Fbxl18	chr5	143647064	143648978	-54.80	-1.28
Smurf1	chr5	145637663	145637963	-1.05	-0.44
Smurf1	chr5	145638322	145638682	-0.71	-0.20
Smurf1	chr5	145639310	145639521	-0.43	0.15
Smurf1	chr5	145726518	145726669	-0.30	0.31
Rnf6	chr5	147021822	147022092	-0.81	0.13
Rnf6	chr5	147023104	147032058	-0.38	-0.01
Cul1	chr6	47404345	47445165	-12.40	0.63
Cul1	chr6	47474617	47476108	-2.74	-0.21
Znrf2	chr6	54838538	54838599	-0.34	-0.02
Herc6	chr6	57531001	57531152	-0.51	-0.34
Kbtbd8	chr6	95071961	95077327	-8.27	0.74
Vhl	chr6	113578147	113579978	-1.22	0.20
Fbxl14	chr6	119429685	119431421	-0.82	0.06
Spsb2	chr6	124759016	124760485	-34.00	-1.18
Klhl42	chr6	147039984	147056940	-0.79	-0.10
Ube2h	chr6	30163827	30164395	-0.70	-0.09

Ube2h	chr6	30164783	30164963	-0.64	0.31
Vamp8	chr6	72335214	72335477	-0.41	-0.03
Vamp8	chr6	72338403	72340632	-1.83	-0.34
Herc2	chr7	63485072	63486898	-5.28	0.30
Ube3a	chr7	66484119	66496353	-3.29	0.33
Ube3a	chr7	66531169	66540150	-6.21	0.19
Klhl25	chr7	82993313	83012167	-0.53	-0.05
Klhl25	chr7	83017611	83018269	-5.57	-0.87
Klhl25	chr7	83018746	83018926	-0.98	-0.56
Rbbp6	chr7	130114077	130114468	-1.85	-0.17
Rbbp6	chr7	130140585	130140736	-1.81	-0.24
Rbbp6	chr7	130142091	130145876	-9.55	0.14
Fbxl19	chr7	134890288	134891685	-4.78	-1.09
Fbxl19	chr7	134893330	134904820	-7.37	-0.55
Fbxl19	chr7	134911014	134911817	-1.61	-0.24
Fbxl19	chr7	134911935	134912442	-2.06	-0.46
Stx4a	chr7	134985380	134985840	-3.36	-0.34
Ube2m	chr7	13623108	13623619	-2.32	-0.37
Psmd8	chr7	29965393	29965573	-6.44	-0.28
Asb7	chr7	73792504	73823980	-1.38	-0.19
Det1	chr7	85972688	85972809	-1.30	-0.59
Det1	chr7	85988116	85988896	-0.39	0.03
Trim21	chr7	109707151	109708560	-3.22	0.40
Cdc16	chr8	13779263	13781763	-1.50	-0.16
Sh3rf1	chr8	63703117	63808042	-0.87	0.26
Sh3rf1	chr8	63863646	63863797	-0.27	0.10
Sh3rf1	chr8	63872501	63873011	-3.70	-1.02
Fbxl8	chr8	107791952	107793137	-2.37	-0.45
Znrf1	chr8	114060539	114062246	-1.56	-0.12
Znrf1	chr8	114143157	114145711	-0.85	-0.11
Znrf1	chr8	114145770	114146010	-0.96	0.20
Znrf1	chr8	114146219	114147028	-1.23	-0.08
Gan	chr8	119728191	119729086	-3.75	0.43
Lonrf1	chr8	37280167	37282962	-0.49	0.09
Lonrf1	chr8	37311669	37312030	-4.41	-1.72
Klhl2	chr8	67219421	67219870	-1.56	-0.30
Calr	chr8	87366315	87366923	-44.80	-0.26
Calr	chr8	87370204	87370800	-3.99	-0.13
Siah1a	chr8	89248786	89269816	-0.55	0.06
Psmd7	chr8	110104309	110108107	-19.20	0.30
Fbxo31	chr8	124075878	124076177	-0.56	0.18
Fbxo22	chr9	55066270	55071642	-1.39	0.12
Fbxo22	chr9	55071791	55072031	-0.79	0.18

Herc1	chr9	66198256	66220531	-15.00	0.57
Herc1	chr9	66293942	66296296	-11.40	0.70
Herc1	chr9	66298605	66298965	-7.49	0.58
Anapc13	chr9	102528625	102536370	-0.39	-0.04
Dcaf1	chr9	106739021	106740882	-1.41	0.32
Dcaf1	chr9	106760607	106761940	-2.76	0.33
Dcaf1	chr9	106776529	106782364	-0.77	0.23
Fbxl12	chr9	20448880	20449151	-1.08	-0.58
Keap1	chr9	21034503	21042276	-2.14	0.12
Keap1	chr9	21043235	21043776	-0.68	-0.28
Ube4a	chr9	44731538	44732048	-2.54	0.32
Cul5	chr9	53425530	53426100	-4.80	0.34
Rnf111	chr9	70274555	70277420	-1.37	0.15
Rnf111	chr9	70323620	70350792	-32.50	0.60
Fbxo9	chr9	77943550	77943611	-0.20	0.01
Uba5	chr9	103949213	103951621	-20.60	0.65
Rnf123	chr9	107954002	107954273	-11.20	0.85
Arih2	chr9	108522434	108547287	-1.40	0.26
Trim71	chr9	114422150	114422300	-0.59	3.58
Fbxo30	chr10	11001127	11015285	-31.60	0.77
Herc4	chr10	62706723	62726819	-8.30	-0.39
Ube2g2	chr10	77107280	77107637	-1.28	-0.14
Ube2g2	chr10	77108022	77108082	-0.37	-0.06
Cdc34	chr10	79144939	79145322	-7.32	-0.60
Cdc34	chr10	79148043	79151143	-8.59	-0.24
Rnf41	chr10	127874437	127875799	-0.76	-0.07
Rnf217	chr10	31328190	31329531	-4.21	-0.65
Ube2d1	chr10	70718174	70718562	-1.17	0.19
Rnf126	chr10	79221259	79223613	-6.52	-0.24
Rnf126	chr10	79229508	79229686	-1.66	-0.45
Fzr1	chr10	80829742	80830217	-2.52	0.24
Psme4	chr11	30671804	30671925	-0.83	-0.91
Psme4	chr11	30672014	30672135	-0.38	0.12
Fbxw11	chr11	32644536	32645016	-2.21	0.20
Trim11	chr11	58791617	58791916	-3.37	-0.52
Trim11	chr11	58802336	58802515	-0.53	0.13
Trim11	chr11	58802842	58804483	-2.09	-0.17
Trim11	chr11	58804542	58804960	-2.11	0.29
Ube2g1	chr11	72420822	72421002	-6.10	-1.15
Ube2g1	chr11	72493612	72497325	-2.70	0.54
Blmh	chr11	76800026	76800265	-6.38	-0.57
Psmd11	chr11	80285544	80285635	-1.72	-0.32
Psmd3	chr11	98543867	98546829	-17.20	-0.58

Psmd3	chr11	98557023	98557113	-5.43	-0.52
Psme3	chr11	101177564	101178336	-0.63	-0.07
Psme3	chr11	101182070	101183288	-1.70	-0.15
Trim41	chr11	48620503	48622573	-15.80	-0.49
Trim41	chr11	48629367	48630893	-1.35	-0.11
Canx	chr11	50109707	50110776	-20.90	-0.31
Sec24a	chr11	51556985	51569811	-10.80	-0.41
Sec24a	chr11	51569959	51570170	-2.57	-1.70
Wsb1	chr11	79053599	79053898	-1.62	-0.11
Ube2z	chr11	95911494	95911704	-3.10	-0.34
Fbxl20	chr11	97950510	97953462	-0.36	0.03
Klhl11	chr11	100323925	100325737	-8.62	0.35
Klhl11	chr11	100333798	100334096	-1.09	0.31
Cdc27	chr11	104366175	104368590	-17.50	0.51
Smurf2	chr11	106683743	106684358	-0.92	-0.20
Smurf2	chr11	106781639	106781760	-4.20	-1.91
Ube2o	chr11	116399663	116399932	-0.71	0.17
Ube2o	chr11	116400140	116404120	-1.25	-0.09
Ube2o	chr11	116405905	116405995	-0.32	0.03
Socs3	chr11	117827699	117830231	-155.00	-0.84
Psma6	chr12	56499811	56499871	-1.67	-0.32
Lrr1	chr12	70275442	70276002	-0.27	0.05
Psmc1	chr12	101348377	101351286	-1.53	-0.29
Btbd6	chr12	114215550	114217061	-3.22	-0.34
Arel1	chr12	86259755	86261519	-1.15	-0.07
Rnf182	chr13	43763512	43763603	-0.50	3.17
Mylip	chr13	45485110	45499843	-9.87	0.82
Mylip	chr13	45503785	45506534	-34.30	1.21
Rnf144b	chr13	47338305	47340700	-1.37	-0.32
Rnf144b	chr13	47342881	47342942	-0.24	0.19
Rnf144b	chr13	47343000	47343181	-1.76	-1.16
Erap1	chr13	74777319	74783985	-2.44	0.26
Kctd6	chr14	9052713	9055546	-1.19	-0.22
Sec24c	chr14	21512428	21513175	-8.53	-0.43
Sec24c	chr14	21513563	21514072	-3.70	-0.25
Psmc6	chr14	45949498	45950514	-0.68	0.11
Kbtbd7	chr14	79827126	79828716	-0.67	0.13
Fbxl3	chr14	103482030	103488661	-0.97	0.07
Fbxl3	chr14	103491640	103498726	-2.05	0.22
Ncf4	chr15	78075240	78075447	-1.52	-0.21
Ncf4	chr15	78091438	78093010	-2.87	-0.16
Skp2	chr15	9043329	9043510	-0.80	-0.31
Skp2	chr15	9069207	9070137	-0.35	-0.03

Rnf19a	chr15	36170916	36173866	-0.57	0.06
Rnf19a	chr15	36195033	36212872	-7.79	0.66
Fbxo32	chr15	58011932	58012112	-0.37	-0.51
Fbxo32	chr15	58012410	58013069	-1.76	-1.01
Fbxo32	chr15	58046282	58046433	-1.44	-1.83
Asb8	chr15	97965969	97971774	-0.40	-0.02
Mgrn1	chr16	4936953	4937312	-4.30	-0.39
Klhl22	chr16	17776518	17777328	-4.43	-0.44
Psmd2	chr16	20651724	20652324	-8.74	-0.32
Itgb5	chr16	33829750	33830079	-1.89	-0.82
Itgb5	chr16	33919922	33920281	-0.73	-0.11
Itgb5	chr16	33947843	33949245	-0.62	-0.07
Cblb	chr16	52204595	52205075	-0.66	-0.17
Socs1	chr16	10783990	10785629	-0.29	0.03
Dtx3l	chr16	35928994	35932046	-0.87	0.09
Dtx3l	chr16	35932315	35933572	-1.20	0.10
Ltn1	chr16	87415422	87416587	-4.95	0.34
Tapbp	chr17	34056512	34056928	-15.30	-0.62
Tapbp	chr17	34062443	34063061	-73.10	-0.79
Tap1	chr17	34324500	34325221	-8.82	-0.65
Tap1	chr17	34333659	34333780	-0.87	-0.27
Psmb8	chr17	34335288	34335468	-2.26	-0.35
Tap2	chr17	34341453	34342537	-7.03	-0.67
Tap2	chr17	34352372	34353206	-4.39	-0.46
H2-D1	chr17	35400038	35400558	-40.70	-0.54
H2-D1	chr17	35400807	35401046	-2.46	-0.23
H2-D1	chr17	35403816	35404204	-67.80	-0.36
H2-Q2	chr17	35479307	35479659	-0.13	0.17
H2-Q6	chr17	35561821	35564910	-7.70	-0.41
H2-Q6	chr17	35565949	35566691	-0.29	0.01
H2-Q10	chr17	35607880	35610039	-7.41	3.75
Cul7	chr17	46788096	46788277	-3.52	5.23
Cul7	chr17	46788546	46788937	-0.63	-0.57
Cul7	chr17	46790989	46791170	-0.24	0.13
Lnpep	chr17	17715497	17746283	-1.25	0.05
Ccnf	chr17	24360684	24360924	-0.81	0.23
Ccnf	chr17	24361102	24361903	-7.06	0.99
Stub1	chr17	25967580	25967909	-3.74	-0.25
H2-K1	chr17	34133105	34133495	-29.30	-0.60
H2-K1	chr17	34136698	34137218	-41.80	-0.78
H2-T23	chr17	36169129	36248130	-4.86	-0.40
Trim39	chr17	36397225	36398550	-1.71	0.22
Ubr2	chr17	47065899	47067369	-7.14	-0.35

Ubr2	chr17	47147240	47147481	-2.49	-0.41
Pja2	chr17	64658065	64658455	-0.40	-0.03
Pja2	chr17	64658514	64660528	-0.38	0.03
Fbxo11	chr17	88390945	88391656	-2.00	0.16
Fbxo11	chr17	88464415	88464625	-2.23	-0.69
Ube2d2a	chr18	35931212	35931392	-0.74	-0.18
Ube2d2a	chr18	35965390	35965540	-0.95	0.36
Rnf14	chr18	38467690	38467990	-1.19	0.25
Mex3c	chr18	73732718	73749293	-3.27	-0.38
Mex3c	chr18	73749352	73750612	-0.69	-0.04
Cdc23	chr18	34792931	34793262	-3.67	-0.70
Cdc23	chr18	34793500	34793621	-0.34	-0.05
Trim36	chr18	46326899	46328816	-2.13	0.74
Btrc	chr19	45604177	45604358	-1.03	2.52
Fbxl15	chr19	46403649	46404936	-1.75	-0.27
Fbxw4	chr19	45652836	45653256	-0.43	0.04
Ube2a	chrX	34414360	34414509	-1.29	0.25
Huwe1	chrX	148353134	148354606	-16.40	0.30
Pja1	chrX	96661699	96661909	-2.14	0.49
Pja1	chrX	96662087	96663013	-1.28	0.13
Pja1	chrX	96663132	96663849	-0.65	-0.09
Rlim	chrX	101156612	101157152	-18.30	0.53
Rlim	chrX	101157421	101160637	-130.00	0.61
Siah1b	chrX	160509608	160510052	-0.28	-0.01
Uba6	chr5	86541249	86547113	-4.93	0.37

Chromatin modifying enzymes

gene_name	chr	chromStart	chromEnd	diff.lg.p	diff.log2.fc
Ing5	chr1	95714871	95715891	-2.55	0.30
Rbbp5	chr1	134394590	134400982	-3.46	0.33
Rbbp5	chr1	134401131	134401282	-0.55	-0.11
Kdm5b	chr1	136514466	136514617	-1.35	0.58
Kdm5b	chr1	136521340	136521461	-0.57	-0.18
Kdm5b	chr1	136521520	136521700	-0.79	-0.26
Kdm5b	chr1	136525318	136527448	-0.82	-0.14
Kdm5b	chr1	136527777	136528227	-0.93	-0.18
Ncoa2	chr1	13132685	13132776	-0.67	0.29
Ncoa2	chr1	13157086	13165408	-1.09	0.10
Kansl3	chr1	36392574	36393263	-0.30	0.00
Smyd3	chr1	181325708	181325888	-0.42	-0.24
Smyd2	chr1	191704608	191704728	-0.52	-0.07
Phf21a	chr2	92199286	92200964	-2.83	-0.45
Phf20	chr2	156092990	156093201	-0.52	0.09
Phf20	chr2	156124429	156134013	-0.50	0.03

Suv39h2	chr2	3379880	3382149	-1.70	-0.32
Suv39h2	chr2	3392077	3392258	-0.42	-0.26
Ehmt1	chr2	24646742	24647852	-2.26	-0.23
Ehmt1	chr2	24733063	24775080	-1.70	0.17
Atf2	chr2	73656977	73657038	-1.83	0.77
Tbl1xr1	chr3	22108479	22109805	-3.67	0.30
Jade1	chr3	41408675	41418244	-1.19	-0.19
Ash1l	chr3	88754285	88786231	-1.65	0.08
Ash1l	chr3	88786769	88786890	-2.91	0.50
Ash1l	chr3	88787428	88787759	-0.30	0.00
Ash1l	chr3	88787967	88788567	-2.16	0.15
Ash1l	chr3	88789046	88811794	-1.47	0.07
Ash1l	chr3	88878647	88881260	-0.59	0.05
Gatad2b	chr3	90145581	90145879	-2.33	-0.23
Gatad2b	chr3	90155817	90155908	-2.67	-0.37
Hist2h2be	chr3	96025043	96025490	-0.87	-0.31
Hist2h2be	chr3	96026738	96027215	-4.41	-0.94
Hist2h2be	chr3	96027422	96027542	-16.90	4.03
Hist2h2bb	chr3	96073622	96073710	-3.44	0.29
Wdr77	chr3	105770196	105770257	-2.12	0.32
Setd7	chr3	51324920	51325369	-1.48	-0.26
Setd7	chr3	51354232	51364745	-0.74	-0.20
Setdb1	chr3	95127865	95128046	-0.44	0.06
Setdb1	chr3	95130188	95132524	-1.57	0.13
Setdb1	chr3	95141164	95142843	-0.78	0.08
Prmt6	chr3	110050616	110051517	-1.73	-0.62
Prmt6	chr3	110052386	110053916	-3.52	-0.20
Nfkb1	chr3	135248217	135252299	-9.16	-0.42
Nfkb1	chr3	135354210	135354481	-4.75	1.80
Kdm4a	chr4	117809936	117810176	-3.42	-0.49
Kdm4a	chr4	117810444	117811478	-1.02	-0.08
Kdm4a	chr4	117834353	117835073	-1.06	0.18
Arid1a	chr4	133235910	133241652	-6.81	-0.11
Arid1a	chr4	133276907	133312385	-15.30	-0.39
Kdm1a	chr4	136106507	136106746	-1.16	0.25
Kmt2e	chr5	22940246	22940487	-0.36	0.04
Kmt2e	chr5	22998122	22999368	-9.91	0.64
Kmt2e	chr5	23002879	23005273	-2.07	0.16
Kmt2e	chr5	23007381	23008999	-2.35	0.12
Nsd2	chr5	34164323	34188823	-0.43	0.03
Nsd2	chr5	34197344	34203748	-1.97	-0.16
Nsd2	chr5	34236231	34238037	-1.81	-0.33
Rest	chr5	77694578	77697550	-1.62	0.16

Rest	chr5	77709852	77712154	-0.68	0.03
Kmt5a	chr5	124909965	124910788	-20.00	-0.59
Kmt5a	chr5	124911266	124911327	-0.69	-0.13
Smarcd3	chr5	24100691	24100811	-0.72	-0.86
Kmt2c	chr5	24790138	24790949	-1.48	0.18
Kmt2c	chr5	24804711	24805401	-10.20	0.57
Kmt2c	chr5	24805460	24806151	-4.91	0.39
Kmt2c	chr5	24815734	24816754	-8.16	0.42
Kmt2c	chr5	24819830	24820730	-2.10	0.18
Kmt2c	chr5	24820879	24824835	-8.46	0.44
Kmt2c	chr5	24878616	24880356	-8.87	0.54
Suds3	chr5	117542584	117542943	-0.42	-0.03
Suds3	chr5	117558427	117565972	-3.36	-0.34
Kdm2b	chr5	123321403	123321823	-2.44	-0.54
Kdm2b	chr5	123330581	123330821	-5.02	-0.78
Kdm2b	chr5	123382481	123382872	-3.77	-0.96
Ncor2	chr5	125497524	125500195	-10.60	-0.33
Ncor2	chr5	125504014	125509353	-19.10	-0.49
Ncor2	chr5	125517648	125517709	-3.60	-0.95
Ncor2	chr5	125518339	125531298	-9.68	-0.27
Ncor2	chr5	125538304	125548276	-0.66	-0.08
Ncor2	chr5	125659463	125659584	-0.24	-0.12
Brpf1	chr6	113257160	113267069	-6.78	-0.19
Brpf1	chr6	113267573	113269353	-12.70	-0.45
Brpf1	chr6	113272447	113274519	-6.02	-0.40
Kdm5a	chr6	120314116	120314686	-2.13	-0.21
Kdm5a	chr6	120369521	120377827	-1.36	0.14
Kdm5a	chr6	120380152	120389527	-1.63	0.12
Chd4	chr6	125046180	125050907	-31.50	-0.53
Chd4	chr6	125079888	125080369	-0.40	-0.01
Atf7ip	chr6	136508513	136509922	-2.19	0.19
Atf7ip	chr6	136535654	136536194	-1.87	0.24
Atf7ip	chr6	136555060	136555630	-0.73	-0.11
H2afj	chr6	136757367	136757517	-1.06	-0.54
Aebp2	chr6	140571543	140572353	-3.40	-0.64
Aebp2	chr6	140572682	140573517	-7.20	-0.57
Aebp2	chr6	140602621	140602921	-5.18	0.60
Kdm7a	chr6	39092756	39096881	-0.84	-0.08
Kdm3a	chr6	71539383	71540465	-0.87	0.20
Kdm3a	chr6	71553954	71554045	-0.59	-0.21
Kdm3a	chr6	71557595	71562002	-0.49	-0.05
Kmt5c	chr7	4697833	4698936	-7.60	-0.71
Prmt3	chr7	57112858	57113008	-0.98	-0.21

Setd1a	chr7	134927665	134930705	-3.33	-0.13
Setd1a	chr7	134931780	134932028	-0.75	-0.13
Setd1a	chr7	134934941	134940915	-5.78	-0.19
Kat8	chr7	135056030	135056181	-0.75	-0.39
Kmt2b	chr7	31359347	31361734	-15.80	-0.41
Kmt2b	chr7	31368742	31373685	-39.70	-0.49
Eed	chr7	97125526	97129068	-8.65	-0.51
Eed	chr7	97129276	97129426	-0.44	0.08
Ccnd1	chr7	152118471	152118592	-8.79	-0.35
Ccnd1	chr7	152125559	152125830	-6.46	-0.36
Kat6a	chr8	23970010	24013653	-6.85	0.25
Kat6a	chr8	24040759	24043005	-0.79	-0.08
Kat6a	chr8	24046218	24051430	-0.82	0.04
Nsd3	chr8	26751014	26760027	-0.61	-0.03
Nsd3	chr8	26760146	26773719	-1.77	0.12
Nsd3	chr8	26787194	26787644	-1.98	-0.39
Setd6	chr8	98242397	98242815	-2.76	0.34
Ash2l	chr8	26950536	26958082	-1.35	-0.23
Sap30	chr8	59966124	59966657	-73.20	-0.80
Gatad2a	chr8	72433213	72435724	-18.30	-0.41
Gatad2a	chr8	72498029	72498150	-0.57	-0.26
Gatad2a	chr8	72519918	72520248	-0.39	0.08
Carm1	chr9	21392499	21393190	-5.85	-0.25
H2afx	chr9	44143446	44144156	-0.58	0.03
Msl2	chr9	100978450	101004495	-102.00	0.64
Setd2	chr9	110449720	110450379	-17.00	0.63
Setd2	chr9	110450737	110452085	-39.00	0.67
Setd2	chr9	110452354	110452475	-10.20	1.08
Setd2	chr9	110452893	110453971	-27.20	0.59
Setd2	chr9	110474472	110497130	-25.90	0.46
Kmt2a	chr9	44612666	44613327	-0.91	-0.13
Kmt2a	chr9	44613506	44614137	-3.87	0.35
Kmt2a	chr9	44615605	44617182	-4.05	0.41
Kmt2a	chr9	44625809	44629185	-22.40	0.38
Kmt2a	chr9	44629634	44630384	-2.09	0.19
Kmt2a	chr9	44655514	44656204	-3.18	0.26
Kmt2a	chr9	44656473	44656924	-0.49	0.06
Kmt2a	chr9	44657433	44658123	-17.30	0.68
Dot1l	chr10	80217950	80218161	-0.37	-0.06
Dot1l	chr10	80248280	80248979	-17.90	-0.60
Dot1l	chr10	80251442	80254428	-43.00	-0.48
Dot1l	chr10	80254516	80254847	-2.72	-0.65
Dot1l	chr10	80256224	80257034	-4.74	-0.27

Smarcc2	chr10	127920231	127920695	-2.24	0.31
Smarcc2	chr10	127926123	127926273	-0.50	0.09
Arid5b	chr10	67558669	67561394	-0.32	0.00
Smarcb1	chr10	75359631	75359751	-1.58	-0.36
Mbd3	chr10	79855285	79856691	-1.70	-0.10
Hmg20b	chr10	80808790	80809677	-15.90	-0.72
Hmg20b	chr10	80811711	80811861	-0.76	-0.14
Hmg20b	chr10	80812188	80812248	-0.39	-0.10
Hmg20b	chr10	80812903	80813172	-5.09	-1.02
Sap30l	chr11	57615138	57615734	-2.81	-0.55
Sap30l	chr11	57621583	57623790	-1.30	-0.24
Hist3h2a	chr11	58768661	58768840	-3.94	0.83
Suz12	chr11	79843530	79845978	-2.27	0.14
Msl1	chr11	98657082	98662223	-3.04	-0.13
Jade2	chr11	51627943	51629289	-0.36	0.01
Jade2	chr11	51629557	51641772	-14.40	-0.32
Ncor1	chr11	62130910	62131481	-1.03	0.10
Ncor1	chr11	62147216	62147367	-0.98	0.19
Ncor1	chr11	62182894	62186997	-2.47	-0.16
Ncor1	chr11	62251972	62270948	-0.37	-0.06
Chd3	chr11	69157463	69157943	-4.16	-0.37
Chd3	chr11	69174956	69175626	-3.21	-0.43
Chd3	chr11	69177569	69182868	-4.43	-0.47
Kdm6b	chr11	69215671	69216507	-8.07	-1.15
Kdm6b	chr11	69217076	69219995	-161.00	-1.08
Kdm6b	chr11	69220054	69220322	-3.77	-0.70
Kdm6b	chr11	69221831	69227145	-25.90	-1.31
Kat7	chr11	95171379	95171530	-6.36	-0.76
Kansl1	chr11	104196011	104196131	-0.45	-0.10
Kansl1	chr11	104196220	104196882	-7.15	0.50
Kansl1	chr11	104240123	104282447	-3.36	0.66
Kansl1	chr11	104285114	104303284	-21.50	0.39
Kansl1	chr11	104303433	104303554	-0.49	-0.23
Smarcd2	chr11	106133926	106134286	-3.65	-0.78
Jmjd6	chr11	116698745	116700329	-6.01	-0.38
Jmjd6	chr11	116702283	116702432	-0.41	-0.08
Jmjd6	chr11	116703734	116704704	-11.90	-0.46
Dnmt3a	chr12	3806582	3807208	-0.30	0.08
Dnmt3a	chr12	3835069	3866168	-3.48	-0.38
Dnmt3a	chr12	3906804	3908036	-0.47	-0.02
Arid4a	chr12	72116983	72117769	-0.62	0.20
Arid4a	chr12	72173660	72187581	-75.50	1.08
Rcor1	chr12	112348059	112350521	-0.56	0.04

Mta1	chr12	114374970	114375239	-3.26	-0.32
Ncoa1	chr12	4247630	4248379	-1.43	-0.26
Ncoa1	chr12	4248916	4249007	-0.66	-0.36
Ncoa1	chr12	4249155	4252804	-0.79	-0.16
Ncoa1	chr12	4267739	4267919	-0.46	-0.11
Ncoa1	chr12	4294980	4296087	-2.92	0.28
Setd3	chr12	109345444	109346800	-0.48	0.04
Arid4b	chr13	14156058	14156209	-0.55	0.17
Arid4b	chr13	14262331	14270255	-4.99	1.00
Arid4b	chr13	14276698	14283557	-33.70	0.75
Arid4b	chr13	14287645	14290373	-6.79	0.72
Hist1h3h	chr13	21809496	21809698	-0.75	-0.19
Hist1h3f	chr13	23636303	23636393	-1.63	-0.30
Hist1h2bg	chr13	23663268	23663474	-0.87	-0.07
Hist1h3d	chr13	23667608	23667786	-0.59	-0.11
Hist1h2bc	chr13	23776067	23776213	-1.76	0.26
Hist1h2ab	chr13	23842956	23843224	-1.74	-0.27
Hist1h3i	chr13	21875208	21875266	-0.36	-0.05
Hist1h3c	chr13	23837329	23837390	-0.45	0.12
Phf2	chr13	48898225	48899711	-3.11	-0.39
Phf2	chr13	48912894	48913224	-2.05	-0.36
Kat6b	chr14	22320299	22336775	-0.61	0.10
Kat6b	chr14	22481439	22481680	-2.25	0.90
Kat6b	chr14	22483878	22490307	-21.30	0.75
Sap18	chr14	58423518	58423608	-5.12	2.40
Prmt5	chr14	55126018	55126553	-0.37	0.02
Ep300	chr15	81416643	81438767	-1.28	-0.07
Ep300	chr15	81479273	81481819	-1.93	-0.07
Arid2	chr15	96199968	96201115	-0.78	-0.06
Arid2	chr15	96201714	96209326	-6.05	0.24
Arid2	chr15	96223066	96232775	-1.58	0.29
Brd1	chr15	88543169	88543779	-0.31	0.01
Brd1	chr15	88547485	88564529	-11.30	0.33
Kansl2	chr15	98349531	98349829	-0.44	-0.08
Riox2	chr16	59471749	59472260	-0.78	0.28
Riox2	chr16	59475394	59475634	-0.92	0.21
Brwd1	chr16	96223575	96225611	-1.11	-0.08
Brwd1	chr16	96229450	96229631	-0.85	0.39
Arid1b	chr17	4995073	4995882	-1.14	-0.42
Arid1b	chr17	4996061	5040748	-2.27	-0.39
Arid1b	chr17	5332607	5337057	-0.44	0.03
Arid1b	chr17	5337146	5339263	-0.95	-0.21
Arid1b	chr17	5341548	5343285	-1.16	0.08

Brpf3	chr17	28938160	28938221	-0.31	-0.68
Brpf3	chr17	28943116	28944254	-4.03	0.47
Brpf3	chr17	28954078	28960704	-1.02	0.17
Brpf3	chr17	28973668	28973878	-0.31	-0.05
Ehmt2	chr17	35042149	35042557	-6.40	-0.61
Ehmt2	chr17	35049712	35050992	-2.85	-0.23
Kdm4b	chr17	56540981	56542236	-2.85	-0.33
Kdm3b	chr18	34963125	34963784	-0.53	0.04
Kdm3b	chr18	34964262	34970049	-4.14	0.21
Kdm3b	chr18	34997317	34998156	-0.35	0.01
Hdac3	chr18	38096953	38097193	-2.88	-0.41
Kmt5b	chr19	3807383	3807473	-0.32	0.04
Kmt5b	chr19	3808821	3816385	-0.31	0.00
Brms1	chr19	5040306	5040901	-1.70	-0.29
Brms1	chr19	5041315	5046427	-3.40	-0.29
Brms1	chr19	5049008	5049917	-1.85	-0.18
Rela	chr19	5646968	5647773	-3.73	-0.21
Smarca2	chr19	26705324	26705475	-0.54	-0.24
Nfkb2	chr19	46379346	46380320	-0.71	0.19
Kdm2a	chr19	4316804	4316954	-0.36	-0.03
Kdm2a	chr19	4318748	4320217	-1.20	-0.08
Kdm2a	chr19	4322495	4326821	-3.96	-0.21
Kdm2a	chr19	4342850	4345636	-3.25	-0.22
Kdm2a	chr19	4396362	4396752	-9.60	-0.67
Kdm6a	chrX	17827982	17831812	-2.06	0.22
Kdm6a	chrX	17855854	17855975	-1.60	0.69
Jade3	chrX	20088240	20088510	-10.90	1.67
Jade3	chrX	20088599	20094813	-36.50	1.80
Tbl1x	chrX	74905095	74905514	-6.62	0.41
Ogt	chrX	98874610	98878285	-3.11	0.13
Ogt	chrX	98878822	98878973	-4.04	-0.63
Kdm5c	chrX	148667772	148667922	-1.79	0.45
Kdm5c	chrX	148705250	148708120	-1.06	-0.06
Suv39h1	chrX	7647888	7649500	-0.85	0.14
Hcfc1	chrX	71189302	71189693	-1.73	-0.14
Hcfc1	chrX	71194650	71196000	-0.95	0.04
Hcfc1	chrX	71211533	71211654	-1.28	0.19
Kdm5d	chrY	277702	278241	-4.97	0.56
Uty	chrY	494530	494860	-6.05	0.72

Chromatin organization

gene_name	chr	chromStart	chromEnd	diff.lg.p	diff.log2.fc
Ing5	chr1	95714871	95715891	-2.55	0.30
Rbbp5	chr1	134394590	134400982	-3.46	0.33

Rbbp5	chr1	134401131	134401282	-0.55	-0.11
Kdm5b	chr1	136514466	136514617	-1.35	0.58
Kdm5b	chr1	136521340	136521461	-0.57	-0.18
Kdm5b	chr1	136521520	136521700	-0.79	-0.26
Kdm5b	chr1	136525318	136527448	-0.82	-0.14
Kdm5b	chr1	136527777	136528227	-0.93	-0.18
Ncoa2	chr1	13132685	13132776	-0.67	0.29
Ncoa2	chr1	13157086	13165408	-1.09	0.10
Kansl3	chr1	36392574	36393263	-0.30	0.00
Smyd3	chr1	181325708	181325888	-0.42	-0.24
Smyd2	chr1	191704608	191704728	-0.52	-0.07
Phf21a	chr2	92199286	92200964	-2.83	-0.45
Phf20	chr2	156092990	156093201	-0.52	0.09
Phf20	chr2	156124429	156134013	-0.50	0.03
Suv39h2	chr2	3379880	3382149	-1.70	-0.32
Suv39h2	chr2	3392077	3392258	-0.42	-0.26
Ehmt1	chr2	24646742	24647852	-2.26	-0.23
Ehmt1	chr2	24733063	24775080	-1.70	0.17
Atf2	chr2	73656977	73657038	-1.83	0.77
Tbl1xr1	chr3	22108479	22109805	-3.67	0.30
Jade1	chr3	41408675	41418244	-1.19	-0.19
Ash1l	chr3	88754285	88786231	-1.65	0.08
Ash1l	chr3	88786769	88786890	-2.91	0.50
Ash1l	chr3	88787428	88787759	-0.30	0.00
Ash1l	chr3	88787967	88788567	-2.16	0.15
Ash1l	chr3	88789046	88811794	-1.47	0.07
Ash1l	chr3	88878647	88881260	-0.59	0.05
Gatad2b	chr3	90145581	90145879	-2.33	-0.23
Gatad2b	chr3	90155817	90155908	-2.67	-0.37
Hist2h2be	chr3	96025043	96025490	-0.87	-0.31
Hist2h2be	chr3	96026738	96027215	-4.41	-0.94
Hist2h2be	chr3	96027422	96027542	-16.90	4.03
Hist2h2bb	chr3	96073622	96073710	-3.44	0.29
Wdr77	chr3	105770196	105770257	-2.12	0.32
Setd7	chr3	51324920	51325369	-1.48	-0.26
Setd7	chr3	51354232	51364745	-0.74	-0.20
Setdb1	chr3	95127865	95128046	-0.44	0.06
Setdb1	chr3	95130188	95132524	-1.57	0.13
Setdb1	chr3	95141164	95142843	-0.78	0.08
Prmt6	chr3	110050616	110051517	-1.73	-0.62
Prmt6	chr3	110052386	110053916	-3.52	-0.20
Nfkb1	chr3	135248217	135252299	-9.16	-0.42
Nfkb1	chr3	135354210	135354481	-4.75	1.80

Kdm4a	chr4	117809936	117810176	-3.42	-0.49
Kdm4a	chr4	117810444	117811478	-1.02	-0.08
Kdm4a	chr4	117834353	117835073	-1.06	0.18
Arid1a	chr4	133235910	133241652	-6.81	-0.11
Arid1a	chr4	133276907	133312385	-15.30	-0.39
Kdm1a	chr4	136106507	136106746	-1.16	0.25
Kmt2e	chr5	22940246	22940487	-0.36	0.04
Kmt2e	chr5	22998122	22999368	-9.91	0.64
Kmt2e	chr5	23002879	23005273	-2.07	0.16
Kmt2e	chr5	23007381	23008999	-2.35	0.12
Nsd2	chr5	34164323	34188823	-0.43	0.03
Nsd2	chr5	34197344	34203748	-1.97	-0.16
Nsd2	chr5	34236231	34238037	-1.81	-0.33
Rest	chr5	77694578	77697550	-1.62	0.16
Rest	chr5	77709852	77712154	-0.68	0.03
Kmt5a	chr5	124909965	124910788	-20.00	-0.59
Kmt5a	chr5	124911266	124911327	-0.69	-0.13
Smarcd3	chr5	24100691	24100811	-0.72	-0.86
Kmt2c	chr5	24790138	24790949	-1.48	0.18
Kmt2c	chr5	24804711	24805401	-10.20	0.57
Kmt2c	chr5	24805460	24806151	-4.91	0.39
Kmt2c	chr5	24815734	24816754	-8.16	0.42
Kmt2c	chr5	24819830	24820730	-2.10	0.18
Kmt2c	chr5	24820879	24824835	-8.46	0.44
Kmt2c	chr5	24878616	24880356	-8.87	0.54
Suds3	chr5	117542584	117542943	-0.42	-0.03
Suds3	chr5	117558427	117565972	-3.36	-0.34
Kdm2b	chr5	123321403	123321823	-2.44	-0.54
Kdm2b	chr5	123330581	123330821	-5.02	-0.78
Kdm2b	chr5	123382481	123382872	-3.77	-0.96
Ncor2	chr5	125497524	125500195	-10.60	-0.33
Ncor2	chr5	125504014	125509353	-19.10	-0.49
Ncor2	chr5	125517648	125517709	-3.60	-0.95
Ncor2	chr5	125518339	125531298	-9.68	-0.27
Ncor2	chr5	125538304	125548276	-0.66	-0.08
Ncor2	chr5	125659463	125659584	-0.24	-0.12
Brpf1	chr6	113257160	113267069	-6.78	-0.19
Brpf1	chr6	113267573	113269353	-12.70	-0.45
Brpf1	chr6	113272447	113274519	-6.02	-0.40
Kdm5a	chr6	120314116	120314686	-2.13	-0.21
Kdm5a	chr6	120369521	120377827	-1.36	0.14
Kdm5a	chr6	120380152	120389527	-1.63	0.12
Chd4	chr6	125046180	125050907	-31.50	-0.53

Chd4	chr6	125079888	125080369	-0.40	-0.01
Atf7ip	chr6	136508513	136509922	-2.19	0.19
Atf7ip	chr6	136535654	136536194	-1.87	0.24
Atf7ip	chr6	136555060	136555630	-0.73	-0.11
H2afj	chr6	136757367	136757517	-1.06	-0.54
Aebp2	chr6	140571543	140572353	-3.40	-0.64
Aebp2	chr6	140572682	140573517	-7.20	-0.57
Aebp2	chr6	140602621	140602921	-5.18	0.60
Kdm7a	chr6	39092756	39096881	-0.84	-0.08
Kdm3a	chr6	71539383	71540465	-0.87	0.20
Kdm3a	chr6	71553954	71554045	-0.59	-0.21
Kdm3a	chr6	71557595	71562002	-0.49	-0.05
Kmt5c	chr7	4697833	4698936	-7.60	-0.71
Prmt3	chr7	57112858	57113008	-0.98	-0.21
Setd1a	chr7	134927665	134930705	-3.33	-0.13
Setd1a	chr7	134931780	134932028	-0.75	-0.13
Setd1a	chr7	134934941	134940915	-5.78	-0.19
Kat8	chr7	135056030	135056181	-0.75	-0.39
Kmt2b	chr7	31359347	31361734	-15.80	-0.41
Kmt2b	chr7	31368742	31373685	-39.70	-0.49
Eed	chr7	97125526	97129068	-8.65	-0.51
Eed	chr7	97129276	97129426	-0.44	0.08
Ccnd1	chr7	152118471	152118592	-8.79	-0.35
Ccnd1	chr7	152125559	152125830	-6.46	-0.36
Kat6a	chr8	23970010	24013653	-6.85	0.25
Kat6a	chr8	24040759	24043005	-0.79	-0.08
Kat6a	chr8	24046218	24051430	-0.82	0.04
Nsd3	chr8	26751014	26760027	-0.61	-0.03
Nsd3	chr8	26760146	26773719	-1.77	0.12
Nsd3	chr8	26787194	26787644	-1.98	-0.39
Setd6	chr8	98242397	98242815	-2.76	0.34
Ash2l	chr8	26950536	26958082	-1.35	-0.23
Sap30	chr8	59966124	59966657	-73.20	-0.80
Gatad2a	chr8	72433213	72435724	-18.30	-0.41
Gatad2a	chr8	72498029	72498150	-0.57	-0.26
Gatad2a	chr8	72519918	72520248	-0.39	0.08
Carm1	chr9	21392499	21393190	-5.85	-0.25
H2afx	chr9	44143446	44144156	-0.58	0.03
Msl2	chr9	100978450	101004495	-102.00	0.64
Setd2	chr9	110449720	110450379	-17.00	0.63
Setd2	chr9	110450737	110452085	-39.00	0.67
Setd2	chr9	110452354	110452475	-10.20	1.08
Setd2	chr9	110452893	110453971	-27.20	0.59

Setd2	chr9	110474472	110497130	-25.90	0.46
Kmt2a	chr9	44612666	44613327	-0.91	-0.13
Kmt2a	chr9	44613506	44614137	-3.87	0.35
Kmt2a	chr9	44615605	44617182	-4.05	0.41
Kmt2a	chr9	44625809	44629185	-22.40	0.38
Kmt2a	chr9	44629634	44630384	-2.09	0.19
Kmt2a	chr9	44655514	44656204	-3.18	0.26
Kmt2a	chr9	44656473	44656924	-0.49	0.06
Kmt2a	chr9	44657433	44658123	-17.30	0.68
Dot1l	chr10	80217950	80218161	-0.37	-0.06
Dot1l	chr10	80248280	80248979	-17.90	-0.60
Dot1l	chr10	80251442	80254428	-43.00	-0.48
Dot1l	chr10	80254516	80254847	-2.72	-0.65
Dot1l	chr10	80256224	80257034	-4.74	-0.27
Smarcc2	chr10	127920231	127920695	-2.24	0.31
Smarcc2	chr10	127926123	127926273	-0.50	0.09
Arid5b	chr10	67558669	67561394	-0.32	0.00
Smarcb1	chr10	75359631	75359751	-1.58	-0.36
Mbd3	chr10	79855285	79856691	-1.70	-0.10
Hmg20b	chr10	80808790	80809677	-15.90	-0.72
Hmg20b	chr10	80811711	80811861	-0.76	-0.14
Hmg20b	chr10	80812188	80812248	-0.39	-0.10
Hmg20b	chr10	80812903	80813172	-5.09	-1.02
Sap30l	chr11	57615138	57615734	-2.81	-0.55
Sap30l	chr11	57621583	57623790	-1.30	-0.24
Hist3h2a	chr11	58768661	58768840	-3.94	0.83
Suz12	chr11	79843530	79845978	-2.27	0.14
Msl1	chr11	98657082	98662223	-3.04	-0.13
Jade2	chr11	51627943	51629289	-0.36	0.01
Jade2	chr11	51629557	51641772	-14.40	-0.32
Ncor1	chr11	62130910	62131481	-1.03	0.10
Ncor1	chr11	62147216	62147367	-0.98	0.19
Ncor1	chr11	62182894	62186997	-2.47	-0.16
Ncor1	chr11	62251972	62270948	-0.37	-0.06
Chd3	chr11	69157463	69157943	-4.16	-0.37
Chd3	chr11	69174956	69175626	-3.21	-0.43
Chd3	chr11	69177569	69182868	-4.43	-0.47
Kdm6b	chr11	69215671	69216507	-8.07	-1.15
Kdm6b	chr11	69217076	69219995	-161.00	-1.08
Kdm6b	chr11	69220054	69220322	-3.77	-0.70
Kdm6b	chr11	69221831	69227145	-25.90	-1.31
Kat7	chr11	95171379	95171530	-6.36	-0.76
Kansl1	chr11	104196011	104196131	-0.45	-0.10

Kansl1	chr11	104196220	104196882	-7.15	0.50
Kansl1	chr11	104240123	104282447	-3.36	0.66
Kansl1	chr11	104285114	104303284	-21.50	0.39
Kansl1	chr11	104303433	104303554	-0.49	-0.23
Smarcd2	chr11	106133926	106134286	-3.65	-0.78
Jmjd6	chr11	116698745	116700329	-6.01	-0.38
Jmjd6	chr11	116702283	116702432	-0.41	-0.08
Jmjd6	chr11	116703734	116704704	-11.90	-0.46
Dnmt3a	chr12	3806582	3807208	-0.30	0.08
Dnmt3a	chr12	3835069	3866168	-3.48	-0.38
Dnmt3a	chr12	3906804	3908036	-0.47	-0.02
Arid4a	chr12	72116983	72117769	-0.62	0.20
Arid4a	chr12	72173660	72187581	-75.50	1.08
Rcor1	chr12	112348059	112350521	-0.56	0.04
Mta1	chr12	114374970	114375239	-3.26	-0.32
Ncoa1	chr12	4247630	4248379	-1.43	-0.26
Ncoa1	chr12	4248916	4249007	-0.66	-0.36
Ncoa1	chr12	4249155	4252804	-0.79	-0.16
Ncoa1	chr12	4267739	4267919	-0.46	-0.11
Ncoa1	chr12	4294980	4296087	-2.92	0.28
Setd3	chr12	109345444	109346800	-0.48	0.04
Arid4b	chr13	14156058	14156209	-0.55	0.17
Arid4b	chr13	14262331	14270255	-4.99	1.00
Arid4b	chr13	14276698	14283557	-33.70	0.75
Arid4b	chr13	14287645	14290373	-6.79	0.72
Hist1h3h	chr13	21809496	21809698	-0.75	-0.19
Hist1h3f	chr13	23636303	23636393	-1.63	-0.30
Hist1h2bg	chr13	23663268	23663474	-0.87	-0.07
Hist1h3d	chr13	23667608	23667786	-0.59	-0.11
Hist1h2bc	chr13	23776067	23776213	-1.76	0.26
Hist1h2ab	chr13	23842956	23843224	-1.74	-0.27
Hist1h3i	chr13	21875208	21875266	-0.36	-0.05
Hist1h3c	chr13	23837329	23837390	-0.45	0.12
Phf2	chr13	48898225	48899711	-3.11	-0.39
Phf2	chr13	48912894	48913224	-2.05	-0.36
Kat6b	chr14	22320299	22336775	-0.61	0.10
Kat6b	chr14	22481439	22481680	-2.25	0.90
Kat6b	chr14	22483878	22490307	-21.30	0.75
Sap18	chr14	58423518	58423608	-5.12	2.40
Prmt5	chr14	55126018	55126553	-0.37	0.02
Ep300	chr15	81416643	81438767	-1.28	-0.07
Ep300	chr15	81479273	81481819	-1.93	-0.07
Arid2	chr15	96199968	96201115	-0.78	-0.06

Arid2	chr15	96201714	96209326	-6.05	0.24
Arid2	chr15	96223066	96232775	-1.58	0.29
Brd1	chr15	88543169	88543779	-0.31	0.01
Brd1	chr15	88547485	88564529	-11.30	0.33
Kansl2	chr15	98349531	98349829	-0.44	-0.08
Riox2	chr16	59471749	59472260	-0.78	0.28
Riox2	chr16	59475394	59475634	-0.92	0.21
Brwd1	chr16	96223575	96225611	-1.11	-0.08
Brwd1	chr16	96229450	96229631	-0.85	0.39
Arid1b	chr17	4995073	4995882	-1.14	-0.42
Arid1b	chr17	4996061	5040748	-2.27	-0.39
Arid1b	chr17	5332607	5337057	-0.44	0.03
Arid1b	chr17	5337146	5339263	-0.95	-0.21
Arid1b	chr17	5341548	5343285	-1.16	0.08
Brpf3	chr17	28938160	28938221	-0.31	-0.68
Brpf3	chr17	28943116	28944254	-4.03	0.47
Brpf3	chr17	28954078	28960704	-1.02	0.17
Brpf3	chr17	28973668	28973878	-0.31	-0.05
Ehmt2	chr17	35042149	35042557	-6.40	-0.61
Ehmt2	chr17	35049712	35050992	-2.85	-0.23
Kdm4b	chr17	56540981	56542236	-2.85	-0.33
Kdm3b	chr18	34963125	34963784	-0.53	0.04
Kdm3b	chr18	34964262	34970049	-4.14	0.21
Kdm3b	chr18	34997317	34998156	-0.35	0.01
Hdac3	chr18	38096953	38097193	-2.88	-0.41
Kmt5b	chr19	3807383	3807473	-0.32	0.04
Kmt5b	chr19	3808821	3816385	-0.31	0.00
Brms1	chr19	5040306	5040901	-1.70	-0.29
Brms1	chr19	5041315	5046427	-3.40	-0.29
Brms1	chr19	5049008	5049917	-1.85	-0.18
Rela	chr19	5646968	5647773	-3.73	-0.21
Smarca2	chr19	26705324	26705475	-0.54	-0.24
Nfkb2	chr19	46379346	46380320	-0.71	0.19
Kdm2a	chr19	4316804	4316954	-0.36	-0.03
Kdm2a	chr19	4318748	4320217	-1.20	-0.08
Kdm2a	chr19	4322495	4326821	-3.96	-0.21
Kdm2a	chr19	4342850	4345636	-3.25	-0.22
Kdm2a	chr19	4396362	4396752	-9.60	-0.67
Kdm6a	chrX	17827982	17831812	-2.06	0.22
Kdm6a	chrX	17855854	17855975	-1.60	0.69
Jade3	chrX	20088240	20088510	-10.90	1.67
Jade3	chrX	20088599	20094813	-36.50	1.80
Tbl1x	chrX	74905095	74905514	-6.62	0.41

Ogt	chrX	98874610	98878285	-3.11	0.13
Ogt	chrX	98878822	98878973	-4.04	-0.63
Kdm5c	chrX	148667772	148667922	-1.79	0.45
Kdm5c	chrX	148705250	148708120	-1.06	-0.06
Suv39h1	chrX	7647888	7649500	-0.85	0.14
Hcfc1	chrX	71189302	71189693	-1.73	-0.14
Hcfc1	chrX	71194650	71196000	-0.95	0.04
Hcfc1	chrX	71211533	71211654	-1.28	0.19
Kdm5d	chrY	277702	278241	-4.97	0.56
Uty	chrY	494530	494860	-6.05	0.72

TNF-signaling

gene_name	chr	chromStart	chromEnd	diff.lg.p	diff.log2.fc
Casp8	chr1	58890568	58901676	-0.30	0.00
Usp21	chr1	173216526	173218003	-13.40	-1.17
Traf2	chr2	25374427	25375145	-1.67	-0.19
Traf1	chr2	34798777	34802204	-38.90	-0.89
Traf1	chr2	34803387	34803955	-5.86	-0.60
Traf1	chr2	34813509	34814038	-10.80	-1.05
Madd	chr2	90977576	90977726	-1.53	-0.64
Madd	chr2	91018210	91018420	-1.66	-0.48
Otud7b	chr3	95908449	95908540	-1.61	-0.69
Otud7b	chr3	95956546	95960193	-1.03	-0.07
Map3k7	chr4	32051081	32051232	-1.86	-0.83
Map3k7	chr4	32104109	32107267	-4.75	0.31
Map3k7	chr4	32110141	32110231	-1.49	-0.76
Ubc	chr5	125866482	125866751	-7.45	-0.16
Ubc	chr5	125867492	125867701	-3.77	1.98
Ubc	chr5	125867967	125870328	-2.39	0.10
Tnfrsf1a	chr6	125311590	125312501	-7.16	-0.29
Fadd	chr7	151765572	151768221	-1.92	-0.22
Cyld	chr8	91220956	91221047	-0.33	-0.20
Cyld	chr8	91226307	91233793	-8.51	0.40
Ikbkb	chr8	23770546	23770997	-0.43	-0.04
Bag4	chr8	26877828	26878848	-1.70	0.20
Tradd	chr8	107782474	107783499	-0.91	0.12
Tradd	chr8	107783873	107788494	-1.84	-0.49
Usp2	chr9	43902061	43902302	-1.62	-0.76
Usp4	chr9	108293381	108294591	-0.82	0.07
Birc2	chr9	7818494	7818943	-1.37	0.33
Birc2	chr9	7833550	7834657	-8.57	0.55
Birc2	chr9	7834955	7835225	-0.88	0.29
Birc3	chr9	7848939	7850928	-0.65	0.09
Birc3	chr9	7860281	7872961	-1.11	0.10

Sppl2b	chr10	80329686	80329837	-1.06	-0.56
Sppl2b	chr10	80330135	80331453	-9.93	-0.64
Tab2	chr10	7625983	7626463	-0.30	0.00
Tab2	chr10	7626701	7629919	-0.81	-0.05
Tab2	chr10	7630969	7644574	-2.87	-0.09
Tnfaip3	chr10	18721581	18725108	-0.69	-0.06
Adam17	chr12	21330026	21333946	-1.67	0.06
Ripk1	chr13	34094265	34094762	-5.04	-0.74
Ripk1	chr13	34119794	34126112	-1.97	0.13
Tab1	chr15	79989200	79992132	-3.29	-0.26
Otulin	chr15	27535675	27538550	-1.58	-0.11
Otulin	chr15	27553110	27560448	-2.84	-0.65
Sharpin	chr15	76180541	76181540	-0.51	-0.06
Tnf	chr17	35336634	35337444	-297.00	-0.91
Tnf	chr17	35338760	35338941	-96.20	-1.00
Xiap	chrX	39447566	39448347	-7.00	0.37
Xiap	chrX	39455267	39458162	-2.29	0.32
Tab3	chrX	82859477	82860195	-3.99	0.50
Tab3	chrX	82860492	82860673	-1.77	0.62
Tab3	chrX	82875804	82876044	-8.19	1.78

Differentially methylated genes following 6 h *P. aeruginosa* infection

Gene expression (transcription)

gene_name	chr	chromStart	chromEnd	diff.lg.p	diff.log2.fc
Cnot11	chr1	39603302	39603632	-9.27	-1.03
Cnot9	chr1	74575438	74575798	-1.13	-0.18
Ing5	chr1	95713260	95715951	-3.10	0.35
Ccnt2	chr1	129698247	129700591	-5.04	0.20
Rbbp5	chr1	134394620	134400982	-0.63	-0.07
Rbbp5	chr1	134401101	134401282	-1.08	0.24
Kdm5b	chr1	136514466	136514617	-0.28	0.01
Kdm5b	chr1	136521370	136521700	-0.83	-0.21
Kdm5b	chr1	136525318	136527418	-2.33	-0.37
Kdm5b	chr1	136527777	136528227	-0.52	0.09
Nr1i3	chr1	173148035	173148455	-0.57	-0.14
Nr1i3	chr1	173148844	173149204	-1.85	-0.52
Exo1	chr1	177829710	177831466	-2.00	-0.72
Exo1	chr1	177839222	177839403	-0.50	-2.10
Parp1	chr1	182499105	182503813	-1.96	-0.45
Trp53bp2	chr1	184371133	184371894	-0.32	-0.03
Trp53bp2	chr1	184376642	184379234	-2.02	-0.28
Trp53bp2	chr1	184391818	184392328	-2.00	-0.40
Ints7	chr1	193443646	193445360	-2.16	-0.35
Nabp1	chr1	51523300	51523510	-1.13	1.14

Nabp1	chr1	51528047	51529205	-6.86	1.58
Nabp1	chr1	51535022	51535263	-1.91	0.77
Gls	chr1	52244535	52244686	-10.30	0.83
Gls	chr1	52276864	52290076	-11.90	-0.48
Sf3b1	chr1	55084201	55084322	-0.60	-0.13
Sumo1	chr1	59696455	59697048	-0.33	-0.01
Bard1	chr1	71121361	71122020	-1.25	-0.38
Hdac4	chr1	93829331	93829960	-0.46	0.09
Steap3	chr1	122123232	122123862	-3.78	-0.81
Steap3	chr1	122140355	122166591	-6.06	-1.12
Mdm4	chr1	134888236	134893148	-1.07	-0.08
Zc3h11a	chr1	135516447	135517433	-9.17	-0.31
Zc3h11a	chr1	135517641	135518733	-2.10	-0.10
Zc3h11a	chr1	135521340	135523781	-17.40	-0.37
Btg2	chr1	135973565	135975732	-1.48	0.08
Rnf2	chr1	153318438	153320426	-0.40	0.03
Pou2f1	chr1	167805604	167806174	-0.89	-0.19
Tfb2m	chr1	181476129	181476398	-1.12	-0.33
Smyd2	chr1	191704578	191704728	-0.49	0.06
Bmi1	chr2	18605667	18606468	-0.37	-0.01
Rxra	chr2	27615158	27615398	-3.09	-0.87
Rxra	chr2	27615487	27616386	-13.50	-1.14
Rxra	chr2	27617342	27618510	-7.46	-0.74
Tsc1	chr2	28531519	28532030	-4.79	-0.58
Tsc1	chr2	28541313	28542943	-0.95	-0.12
Ttf1	chr2	28915782	28922656	-2.19	-0.19
Ttf1	chr2	28941735	28942005	-1.37	-0.42
Abl1	chr2	31544075	31551290	-7.77	-0.43
Abl1	chr2	31653102	31658189	-1.51	-0.10
Abl1	chr2	31658367	31658428	-0.69	-0.40
Nup214	chr2	31888785	31893623	-1.70	-0.12
Mapkap1	chr2	34479093	34479423	-0.34	0.02
Ssrp1	chr2	84885611	84887238	-5.04	-0.18
Tnks1bp1	chr2	84891036	84904146	-4.25	-0.23
Psmc3	chr2	90894439	90895182	-3.33	-0.20
Lmo2	chr2	103809861	103821531	-3.10	-0.19
Cstf3	chr2	104504891	104505372	-2.93	0.31
Rtf1	chr2	119558176	119559166	-0.81	0.09
Rtf1	chr2	119560243	119560484	-0.48	0.12
Mga	chr2	119698286	119698347	-0.21	0.26
Mga	chr2	119722990	119743194	-1.74	0.09
Mga	chr2	119749246	119752139	-1.06	0.11
Mga	chr2	119767083	119767563	-7.58	-0.49

Mga	chr2	119772610	119774079	-2.10	0.12
Mga	chr2	119787692	119790793	-0.32	0.01
Polr1b	chr2	128950958	128952033	-0.34	0.01
Polr3f	chr2	144362104	144365002	-3.29	0.32
Csnk2a1	chr2	152107019	152107169	-10.00	-0.77
Tpx2	chr2	152717983	152719804	-0.61	0.08
Itch	chr2	154959216	154959307	-0.27	-0.02
Itch	chr2	155050102	155050403	-0.31	-0.01
Phf20	chr2	156093020	156093171	-0.35	-0.03
Phf20	chr2	156124429	156134043	-2.43	-0.18
Tgif2	chr2	156679337	156679783	-0.33	-0.07
Src	chr2	157295136	157296531	-2.35	0.70
Rprd1b	chr2	157900721	157901171	-1.37	0.22
Srsf6	chr2	162757243	162757817	-0.30	0.00
L3mbtl1	chr2	162773851	162773971	-0.35	1.56
Ywhab	chr2	163820932	163837497	-10.90	-0.39
Trp53rkb	chr2	166619352	166619593	-0.34	0.09
Trp53rkb	chr2	166620885	166621755	-0.75	-0.18
Cstf1	chr2	172203242	172206586	-1.17	-0.10
Rae1	chr2	172840787	172841203	-9.79	-0.73
Polr3k	chr2	181599124	181600709	-1.00	0.27
Gata3	chr2	9779597	9784722	-2.88	0.70
Gata3	chr2	9790440	9796250	-0.57	-0.22
Taf3	chr2	9837317	9874531	-2.60	-0.16
Pip4k2a	chr2	18765527	18769115	-4.26	0.31
Ehmt1	chr2	24646742	24647852	-3.96	-0.36
Ehmt1	chr2	24733063	24775080	-0.58	-0.06
Snapc4	chr2	26218284	26222737	-9.30	-0.42
Snapc4	chr2	26223926	26225494	-4.22	-0.53
Notch1	chr2	26314079	26314319	-0.24	0.00
Notch1	chr2	26315305	26315396	-0.28	0.42
Notch1	chr2	26315993	26316443	-0.58	-0.38
Gtf3c5	chr2	28421871	28423253	-0.61	-0.08
Gtf3c4	chr2	28679915	28680574	-1.17	-0.20
Gtf3c4	chr2	28680723	28683050	-0.74	0.08
Gtf3c4	chr2	28686074	28692035	-2.43	-0.13
Gtf3c4	chr2	28695460	28695880	-1.50	-0.31
Cdk9	chr2	32562910	32565131	-22.70	-0.34
Psmd5	chr2	34707993	34709823	-1.65	-0.19
Psmd5	chr2	34726274	34726482	-4.41	-0.82
Psmb7	chr2	38498946	38499426	-3.13	-0.36
Nr6a1	chr2	38740753	38781783	-1.41	1.01
Nr4a2	chr2	56960145	56961485	-4.91	-0.57

Nr4a2	chr2	56964219	56965507	-14.90	-0.92
Nr4a2	chr2	56967144	56976354	-1.04	0.68
Clp1	chr2	84563518	84566269	-0.31	0.00
Pin1rt1	chr2	104554491	104554756	-2.79	3.80
Zfp770	chr2	114021670	114027138	-1.72	0.21
Zfp661	chr2	127402341	127403416	-0.33	0.03
Snrpb	chr2	130004981	130005100	-1.98	-0.21
Pcna	chr2	132077710	132078916	-7.08	0.28
Psmf1	chr2	151543111	151543202	-1.31	-0.87
Psmf1	chr2	151543649	151544038	-0.30	0.01
Zfp334	chr2	165203384	165203715	-0.29	0.04
Zfp334	chr2	165205241	165206231	-1.16	0.28
Zfp334	chr2	165207038	165207279	-0.43	0.16
Trp53rka	chr2	165315878	165318814	-1.44	-0.19
Aurka	chr2	172182168	172182683	-0.29	0.00
Taf4	chr2	179647388	179656678	-2.00	-0.23
Taf4	chr2	179710752	179710813	-0.34	0.87
Skil	chr3	30994042	30997412	-0.91	-0.07
Skil	chr3	31015852	31017641	-3.54	-0.30
Gatad2b	chr3	90127990	90145879	-1.31	-0.15
Gatad2b	chr3	90155787	90155908	-1.99	-0.28
Hist2h2be	chr3	96025043	96025490	-2.37	-0.78
Hist2h2be	chr3	96026708	96027215	-0.64	-0.19
Hist2h2be	chr3	96027422	96027483	-10.00	3.73
Notch2	chr3	97817460	97817641	-2.65	-0.57
Notch2	chr3	97938527	97941254	-16.60	-0.46
Notch2	chr3	97950009	97954290	-14.00	-0.14
Trim33	chr3	103148225	103148495	-2.77	0.27
Trim33	chr3	103156043	103158233	-4.71	0.28
Trim33	chr3	103158830	103159400	-0.41	-0.03
Psma5	chr3	108059873	108060022	-0.64	3.39
Ints12	chr3	132771889	132772517	-1.00	-0.12
Ube2d3	chr3	135101656	135102228	-19.60	-0.75
Gtf2b	chr3	142428210	142434402	-2.43	-0.52
Gtf2b	chr3	142444579	142446421	-0.99	0.12
Phc3	chr3	30805887	30813151	-0.72	-0.12
Phc3	chr3	30864733	30868337	-1.81	-0.73
Ccna2	chr3	36470827	36470918	-0.54	-0.57
Elf2	chr3	51059675	51060839	-1.69	0.13
Elf2	chr3	51061545	51064994	-11.20	0.72
Maml3	chr3	51491863	51493302	-3.62	0.65
Maml3	chr3	51493600	51496200	-1.82	0.41
Maml3	chr3	51501693	51600338	-0.20	0.07

Maml3	chr3	51659510	51660829	-9.25	0.71
Maml3	chr3	51907999	51908179	-1.49	0.85
Maml3	chr3	51908418	51908808	-2.19	-0.81
Lamtor2	chr3	88353913	88356849	-9.31	-0.34
Ints3	chr3	90195369	90196223	-2.53	-0.21
Ints3	chr3	90237258	90237498	-4.48	-0.71
Chtop	chr3	90303653	90306049	-0.50	-0.02
Chtop	chr3	90313185	90313365	-3.07	-0.25
Psmb4	chr3	94690701	94690880	-8.50	-0.41
Psmd4	chr3	94836626	94836894	-2.54	-0.16
Psmd4	chr3	94840526	94846467	-8.21	-0.35
Rprd2	chr3	95567509	95570116	-4.11	-0.13
Rprd2	chr3	95576763	95577094	-0.41	-0.07
Hist2h3c2	chr3	96042365	96042604	-1.05	0.63
Polr3gl	chr3	96382213	96386015	-1.46	-0.18
Polr3gl	chr3	96398020	96398081	-0.65	-0.57
Hipk1	chr3	103547180	103548289	-2.19	-0.08
Hipk1	chr3	103581236	103595486	-33.60	-0.28
Prmt6	chr3	110050616	110051517	-0.57	-0.23
Prmt6	chr3	110052356	110053916	-1.37	-0.11
Srsf11	chr3	157674772	157675808	-16.50	-0.32
Trp53inp1	chr4	11083587	11092710	-11.10	0.96
Trp53inp1	chr4	11095044	11097120	-9.82	1.10
Nbn	chr4	15906479	15911111	-1.10	0.17
Rngtt	chr4	33586031	33587733	-1.66	0.32
Rngtt	chr4	33588270	33588360	-0.29	0.12
Polr1e	chr4	45044885	45045215	-2.89	-0.68
Polr1e	chr4	45045964	45046114	-0.41	-0.71
Nr4a3	chr4	48058221	48058400	-1.45	-1.08
Nr4a3	chr4	48064136	48065027	-8.33	-1.10
Nr4a3	chr4	48080626	48096224	-0.76	0.49
Zfp189	chr4	49535279	49543489	-4.61	0.49
Rraga	chr4	86221606	86222293	-1.18	-0.09
Ndc1	chr4	107083754	107086943	-1.16	0.18
Tal1	chr4	114737293	114741792	-0.36	0.03
Tal1	chr4	114741970	114742091	-0.38	-0.13
Med8	chr4	118087440	118087589	-1.21	0.21
Rragc	chr4	123598193	123598284	-0.30	-0.01
Rragc	chr4	123612802	123613851	-0.52	0.02
Lsm10	chr4	125773896	125775828	-0.39	0.02
Phc2	chr4	128404910	128405061	-0.82	-0.46
Phc2	chr4	128424470	128429198	-18.30	-0.40
Phc2	chr4	128429466	128429976	-16.20	-0.47

Srsf4	chr4	131453751	131457610	-9.11	-0.28
Rpa2	chr4	132333826	132334035	-2.59	-0.74
Nr0b2	chr4	133112127	133112392	-0.35	0.24
Zfp46	chr4	135846237	135848601	-1.27	0.14
Mtor	chr4	147822690	147822781	-3.13	-1.30
Mtor	chr4	147930392	147931764	-3.53	-0.34
Ssu72	chr4	155079133	155079193	-1.21	1.30
Ssu72	chr4	155079432	155089762	-0.99	-0.09
Mllt3	chr4	87419218	87419819	-1.24	-0.35
Mllt3	chr4	87486765	87487246	-0.67	0.14
Zfp691	chr4	118842631	118845741	-0.75	0.13
Zfp691	chr4	118845980	118846800	-9.76	2.33
Zfp69	chr4	120602801	120603819	-1.72	0.55
Ago1	chr4	126116441	126116622	-1.67	-0.56
Ago4	chr4	126167982	126168462	-0.68	-0.38
Ago4	chr4	126169837	126170017	-0.34	0.37
Tfap2e	chr4	126393574	126393783	-1.59	2.55
Sesn2	chr4	132048721	132052822	-3.34	-0.16
Sesn2	chr4	132066221	132066371	-3.98	-0.48
Sesn2	chr4	132066234	132066414	-3.65	-0.47
Sfn	chr4	133156530	133157635	-3.71	-1.00
Sfn	chr4	133157694	133158083	-0.48	0.30
Arid1a	chr4	133235940	133241652	-2.63	-0.08
Arid1a	chr4	133276907	133312385	-11.90	-0.45
Srrm1	chr4	134878175	134878385	-1.65	-0.18
Srrm1	chr4	134880008	134881048	-18.30	-0.47
Srrm1	chr4	134902739	134909069	-0.66	-0.08
Eloa	chr4	135561281	135561551	-1.02	-0.17
Eloa	chr4	135565701	135577564	-4.34	-0.14
Ski	chr4	154529795	154533593	-10.40	0.28
Ski	chr4	154533945	154596644	-10.70	-0.37
Cdk6	chr5	3519406	3520884	-1.33	-0.15
Mterf1b	chr5	4196342	4196641	-0.39	-0.20
Mterf1b	chr5	4196818	4197027	-0.65	0.74
Mterf1b	chr5	4197175	4197324	-0.70	0.63
Kmt2e	chr5	22940246	22940516	-2.58	0.46
Kmt2e	chr5	22998122	22999338	-0.91	0.13
Kmt2e	chr5	23002790	23005303	-1.02	0.08
Kmt2e	chr5	23007352	23008999	-1.84	0.10
Nupl2	chr5	23687726	23687877	-0.80	0.38
Nupl2	chr5	23687966	23689616	-1.38	-0.19
Ywhah	chr5	33361494	33361614	-1.23	0.51
Ywhah	chr5	33369299	33370077	-1.30	-0.06

Rbpj	chr5	53981538	53981866	-29.80	-1.15
Rbpj	chr5	54044845	54045026	-0.27	-0.52
Rpap2	chr5	108048973	108049513	-1.52	0.29
Mtf2	chr5	108494760	108498466	-1.61	0.29
Mtf2	chr5	108535623	108536133	-0.32	0.01
Zfp605	chr5	110539457	110539517	-0.19	1.00
Zfp605	chr5	110556601	110558156	-4.58	0.65
Triap1	chr5	115791255	115793441	-0.39	-0.02
Rnf34	chr5	123273869	123314504	-0.81	0.09
Psmd9	chr5	123678228	123678437	-0.34	0.03
Kmt5a	chr5	124909965	124910818	-10.10	-0.42
Kmt5a	chr5	124911146	124911447	-1.43	0.14
Gtf2h3	chr5	125046192	125046313	-0.64	-0.25
Zfp664	chr5	125343755	125366362	-1.00	-0.09
Zfp664	chr5	125366751	125367081	-0.42	-0.06
Crcp	chr5	130505175	130513731	-0.51	-0.10
Crcp	chr5	130535663	130536293	-1.25	-0.16
Rfc2	chr5	135073840	135074198	-2.30	-0.39
Baz1b	chr5	135663192	135663582	-1.11	-0.22
Baz1b	chr5	135692585	135694113	-7.96	0.20
Baz1b	chr5	135718322	135721070	-1.43	-0.09
Zkscan1	chr5	138526311	138534016	-1.05	-0.35
Zkscan1	chr5	138534105	138534645	-16.30	-1.03
Zkscan1	chr5	138541914	138542485	-0.49	-0.05
Zkscan1	chr5	138542723	138543984	-3.46	0.27
Zkscan1	chr5	138544222	138545962	-1.19	-0.12
Zfp12	chr5	144005811	144008390	-3.66	0.31
Zfp12	chr5	144008839	144009619	-0.78	-0.19
Cpsf4	chr5	145939775	145942700	-0.65	0.07
Zkscan5	chr5	145979715	145981274	-1.73	0.54
Zkscan5	chr5	145981632	145981812	-0.41	-0.19
Zkscan5	chr5	145981871	145982380	-1.47	0.57
Zfp655	chr5	145992583	145994463	-2.98	-0.51
Zfp655	chr5	146004460	146006138	-2.46	0.12
Zscan25	chr5	146044360	146052189	-9.62	-0.48
Cdk8	chr5	147042885	147071089	-9.50	-1.16
Mterf1a	chr5	3890610	3891441	-4.06	0.85
Smarcd3	chr5	24100691	24100989	-0.59	-0.63
Prkag2	chr5	24369540	24369661	-0.30	-0.04
Prkag2	chr5	24527783	24527844	-5.60	1.58
Prkag2	chr5	24534296	24606370	-0.56	0.08
Kmt2c	chr5	24790138	24790949	-2.35	-0.28
Kmt2c	chr5	24804711	24805371	-6.01	0.48

Kmt2c	chr5	24805490	24806151	-3.45	0.35
Kmt2c	chr5	24815764	24816754	-4.22	0.34
Kmt2c	chr5	24819800	24820700	-2.61	0.23
Kmt2c	chr5	24820849	24821690	-2.45	0.22
Kmt2c	chr5	24878616	24880356	-8.08	0.57
Gtf3c2	chr5	31459355	31460596	-7.73	-0.30
Slbp	chr5	33992455	33994955	-3.22	0.32
Nup54	chr5	92845130	92846543	-1.18	-0.16
Prkab1	chr5	116463747	116467999	-0.85	-0.08
Prkab1	chr5	116474139	116474407	-0.44	-0.06
Ptpn11	chr5	121581827	121582307	-1.21	0.16
Ptpn11	chr5	121583292	121587519	-0.40	0.02
Ncor2	chr5	125497524	125500255	-17.90	-0.42
Ncor2	chr5	125504014	125509323	-4.66	-0.22
Ncor2	chr5	125517648	125517739	-5.60	-1.05
Ncor2	chr5	125518279	125531358	-10.10	-0.27
Ncor2	chr5	125538304	125548306	-0.58	0.07
Ncor2	chr5	125659493	125659584	-0.45	-0.84
Ubc	chr5	125866453	125867671	-172.00	0.73
Ubc	chr5	125867938	125868147	-3.15	0.20
Ubc	chr5	125868235	125870298	-22.80	-0.40
Zfp11	chr5	130162143	130162980	-1.61	0.64
Pom121	chr5	135853620	135857139	-7.02	-0.36
Pom121	chr5	135857525	135857795	-2.51	-0.32
Ywhag	chr5	136384965	136385384	-2.91	-0.09
Ywhag	chr5	136386428	136406621	-96.80	-0.35
Ywhag	chr5	136410241	136410481	-13.00	-0.40
Zfp113	chr5	138585481	138586141	-3.89	-0.70
Zfp113	chr5	138586529	138586680	-0.41	0.19
Taf6	chr5	138619844	138620232	-4.14	-0.57
Smurf1	chr5	145637663	145638682	-0.50	0.08
Smurf1	chr5	145639310	145639521	-0.66	0.35
Zkscan14	chr5	145955991	145962485	-2.97	-0.45
Rfc3	chr5	152445398	152445638	-0.43	0.10
Nup205	chr6	35127615	35133895	-0.30	0.02
Cul1	chr6	47404345	47445165	-3.56	0.31
Cul1	chr6	47474617	47476108	-1.64	-0.15
Zfp398	chr6	47785899	47790681	-0.56	-0.29
Zfp398	chr6	47815976	47817057	-5.16	-0.83
Zfp282	chr6	47827763	47830376	-1.26	-0.29
Zfp282	chr6	47854827	47855966	-11.40	-0.77
Zfp282	chr6	47856175	47856835	-0.42	-0.06
Zfp212	chr6	47870625	47876740	-0.95	-0.37

Zfp212	chr6	47879063	47881476	-0.63	-0.12
Zfp775	chr6	48555624	48569787	-0.41	-0.16
Zfp775	chr6	48569935	48570026	-0.24	0.38
Zfp775	chr6	48570414	48570923	-1.15	-0.76
Polr1a	chr6	71928026	71930161	-4.10	-0.25
Polr1a	chr6	71934568	71934749	-0.56	0.44
Gata2	chr6	88154654	88155829	-0.38	-0.05
Nr2c2	chr6	92041411	92042461	-2.10	-0.74
Nr2c2	chr6	92114256	92117961	-0.62	0.09
Brpf1	chr6	113257130	113267099	-6.11	-0.18
Brpf1	chr6	113267573	113269353	-20.40	-0.58
Brpf1	chr6	113272417	113274519	-13.10	-0.60
Chd4	chr6	125046180	125050907	-20.10	-0.46
Chd4	chr6	125079888	125080399	-1.95	-0.09
Cdkn1b	chr6	134870958	134872360	-3.01	0.32
Gm8994	chr6	136279021	136279141	-0.30	-0.81
Gm8994	chr6	136279763	136279853	-0.25	0.86
H2afj	chr6	136757307	136757517	-2.15	-0.48
Aebp2	chr6	140571543	140572383	-0.56	-0.13
Aebp2	chr6	140572622	140573517	-1.76	0.25
Aebp2	chr6	140602621	140602891	-4.77	0.69
Cnot4	chr6	34973322	34992713	-1.58	0.32
Cnot4	chr6	34996096	35002891	-0.80	0.08
Hipk2	chr6	38647898	38665995	-46.30	-0.52
Hipk2	chr6	38767930	38787077	-4.22	-0.11
Hipk2	chr6	38825800	38826040	-0.40	-0.04
Zfp786	chr6	47769651	47770337	-0.58	-0.74
Zfp786	chr6	47770782	47771140	-0.50	-0.90
Zfp786	chr6	47771318	47771676	-0.29	0.81
Zfp777	chr6	47974246	47998850	-8.38	-0.33
Zfp746	chr6	48013500	48016503	-1.30	-0.16
Zfp746	chr6	48033523	48036532	-0.67	0.27
Gadd45a	chr6	66985208	66987341	-8.35	-0.44
Nup210	chr6	90963060	90963660	-3.36	-0.50
Rybp	chr6	100182064	100183112	-0.57	0.17
Zfp248	chr6	118378705	118382923	-2.80	0.51
Phc1	chr6	122273438	122273618	-0.97	0.44
Phc1	chr6	122275560	122275830	-0.61	-0.25
Tigar	chr6	127037498	127038307	-0.89	-0.11
Ccnd2	chr6	127079556	127080275	-3.38	0.32
Ccnd2	chr6	127100856	127101036	-0.93	0.38
Rhno1	chr6	128307315	128309210	-1.30	-0.13
Kras	chr6	145168813	145168964	-1.16	-0.35

Ints13	chr6	146502576	146503064	-2.74	0.36
U2af2	chr7	5030883	5031212	-1.10	-0.10
Zfp667	chr7	6256343	6256524	-1.03	1.18
Zfp667	chr7	6256792	6256942	-0.55	0.66
Zfp667	chr7	6257689	6257840	-1.67	2.29
Zfp78	chr7	6331550	6332268	-2.40	1.42
Zfp28	chr7	6344996	6347498	-0.41	0.06
Zfp110	chr7	13434024	13434472	-0.91	0.17
Zfp324	chr7	13555672	13556361	-3.85	-1.08
Zfp324	chr7	13556928	13557646	-0.89	-0.35
Zfp446	chr7	13564366	13564486	-0.27	-0.05
Zfp446	chr7	13567286	13568276	-1.47	-0.29
Zfp446	chr7	13568455	13569056	-0.40	-0.07
Ppp1r13l	chr7	19956897	19960606	-7.13	-0.66
Ercc2	chr7	19979470	19981041	-4.45	-0.33
Zfp180	chr7	24882912	24891546	-4.68	0.24
Zfp112	chr7	24910093	24910364	-0.73	-1.09
Zfp112	chr7	24910573	24910664	-0.48	-1.72
Zfp112	chr7	24911022	24911533	-0.31	-0.12
Zfp235	chr7	24925651	24925772	-1.05	0.94
Zfp235	chr7	24926760	24927181	-1.25	-0.51
Zfp235	chr7	24927869	24928170	-0.36	-0.17
Tgfb1	chr7	26472020	26477430	-36.70	-0.26
Akt2	chr7	28422173	28423398	-0.50	-0.06
Paf1	chr7	29178014	29178133	-0.41	-0.07
Paf1	chr7	29181953	29184402	-0.48	0.02
Zfp30	chr7	30577820	30578780	-4.53	1.80
Zfp790	chr7	30613435	30616012	-3.35	0.25
Zfp420	chr7	30645086	30661309	-4.28	0.31
Zfp382	chr7	30916575	30919814	-0.57	-0.05
Polr2i	chr7	31017332	31017772	-0.55	0.13
Zfp658	chr7	50828048	50828348	-0.67	0.44
Zfp658	chr7	50828646	50829515	-0.33	-0.03
Zfp658	chr7	50829663	50830323	-0.90	-0.28
Zfp719	chr7	50839645	50846689	-6.02	0.33
Zfp819	chr7	50873231	50873322	-0.50	3.17
Nup62	chr7	52071739	52086176	-19.00	-0.23
Gtf2h1	chr7	54051493	54051644	-1.33	-0.22
Gtf2h1	chr7	54078270	54078601	-5.40	0.33
Zfp710	chr7	87169699	87227267	-11.50	-0.28
Zfp710	chr7	87231444	87231625	-0.36	0.04
Zfp710	chr7	87233516	87236204	-0.61	0.04
Lamtor1	chr7	109054380	109058241	-0.87	-0.19

Lamtor1	chr7	109059942	109060091	-16.60	-0.68
Zfp143	chr7	117236697	117238428	-1.89	0.27
Ctr9	chr7	118173736	118177414	-3.03	-0.38
Ctr9	chr7	118198901	118199621	-1.34	-0.16
Polr3e	chr7	128082784	128089396	-8.55	-0.38
Zfp771	chr7	134388039	134398315	-3.36	-0.36
Setd1a	chr7	134920902	134921023	-1.37	0.88
Setd1a	chr7	134927635	134930735	-0.69	-0.03
Setd1a	chr7	134931750	134932028	-1.01	-0.16
Setd1a	chr7	134934941	134940915	-7.31	-0.23
Zik1	chr7	11075804	11076075	-2.76	1.74
Zfp551	chr7	13000735	13000886	-0.20	-0.08
Zfp551	chr7	13001514	13002233	-2.46	1.45
Cd3eap	chr7	19941357	19942974	-31.70	-0.76
Zfp94	chr7	25087140	25088844	-2.13	-0.44
Pou2f2	chr7	25876133	25878098	-1.03	0.11
Psmd8	chr7	29965393	29965573	-13.80	-0.43
Zfp940	chr7	30629198	30629439	-0.25	-0.03
Zfp940	chr7	30629588	30629649	-0.64	1.82
Zfp940	chr7	30630307	30630548	-0.95	1.24
Zfp940	chr7	30630817	30630998	-0.73	0.68
Zfp27	chr7	30678594	30680419	-0.52	-0.05
Zfp27	chr7	30680747	30683850	-0.91	0.18
Zfp14	chr7	30823256	30823436	-0.57	1.58
Zfp14	chr7	30823733	30823854	-0.28	0.22
Zfp566	chr7	30862385	30862860	-0.44	0.13
Zfp566	chr7	30862978	30863483	-3.46	0.75
Kmt2b	chr7	31359377	31361794	-20.90	-0.55
Kmt2b	chr7	31368445	31373745	-14.90	-0.34
Gpi1	chr7	34987182	34987689	-15.30	-0.25
Ccne1	chr7	38883150	38883358	-1.08	-0.54
Zfp715	chr7	50552997	50556994	-8.01	0.30
Zfp473	chr7	51987480	51987751	-0.36	-0.29
Zfp473	chr7	51987810	51988051	-0.70	-0.61
Zfp473	chr7	51988379	51988830	-1.38	1.24
Zfp473	chr7	51988979	51989760	-8.39	2.40
Med25	chr7	52132313	52133597	-6.95	-0.47
Bax	chr7	52721428	52721703	-3.69	-0.20
Bax	chr7	52721999	52722238	-1.35	0.28
E2f8	chr7	56123087	56123387	-0.98	-0.44
E2f8	chr7	56126142	56126233	-0.71	-0.66
E2f8	chr7	56126292	56127247	-0.85	-0.32
Blm	chr7	87647292	87647681	-3.04	-0.71

Blm	chr7	87654437	87658369	-0.32	-0.01
Eed	chr7	97125556	97129068	-6.88	-0.52
Eed	chr7	97129276	97129396	-0.46	-0.10
Pcf11	chr7	99793118	99795438	-8.06	0.60
Pcf11	chr7	99801721	99808380	-11.00	-0.25
Pcf11	chr7	99809567	99810434	-2.11	-0.10
Pcf11	chr7	99818003	99818422	-5.53	-0.40
Nup98	chr7	109268003	109269291	-7.87	-0.22
Nup98	chr7	109283070	109283401	-23.60	-0.89
Rrp8	chr7	112881232	112881920	-1.78	-0.20
Rrp8	chr7	112882533	112882833	-3.86	-0.41
Rrp8	chr7	112883248	112885029	-1.08	-0.16
Gtf3c1	chr7	132784557	132785247	-1.18	-0.15
Gtf3c1	chr7	132786109	132787843	-6.33	-0.43
Gtf3c1	chr7	132790222	132791099	-4.80	-0.44
Gtf3c1	chr7	132851021	132851202	-0.57	-0.12
Zfp747	chr7	134516438	134516707	-0.85	-0.40
Zfp747	chr7	134516765	134519504	-0.42	0.03
9130019O22Rik	chr7	134526131	134526281	-0.56	1.23
9130019O22Rik	chr7	134526847	134529144	-0.95	-0.14
E430018J23Rik	chr7	134534467	134534528	-0.84	-2.04
E430018J23Rik	chr7	134534616	134536839	-0.37	-0.06
Zfp764	chr7	134547897	134549152	-1.39	-0.24
Zfp764	chr7	134550126	134550336	-0.39	-0.19
Zfp688	chr7	134562479	134563593	-1.17	-0.28
Zfp688	chr7	134565487	134565548	-0.77	-1.45
Zfp689	chr7	134587294	134588642	-1.79	-0.40
Zfp689	chr7	134592252	134592433	-0.39	0.12
Zfp668	chr7	135009351	135009442	-0.32	-0.14
Zfp668	chr7	135009620	135020337	-10.40	-0.40
Pidd1	chr7	148624413	148624744	-3.35	-0.92
Pidd1	chr7	148628991	148629907	-3.04	-1.22
Ccnd1	chr7	152118471	152118592	-5.59	-0.32
Ccnd1	chr7	152125559	152125830	-6.58	-0.44
Snapc2	chr8	4255311	4256190	-6.55	-0.73
Tfdp1	chr8	13377166	13377375	-0.39	0.04
Tfdp1	chr8	13377582	13377702	-0.58	-0.09
Kat6a	chr8	23970010	24013683	-5.12	0.22
Kat6a	chr8	24040759	24043005	-0.60	-0.06
Kat6a	chr8	24046218	24051490	-0.77	-0.03
Zfp703	chr8	28087837	28090862	-45.70	-0.65
Ppp2cb	chr8	34710092	34710359	-0.73	-0.37
Gtf2e2	chr8	34886513	34887055	-0.32	-0.01

Nr2c2ap	chr8	72655231	72655498	-1.93	0.47
Nr2c2ap	chr8	72657115	72657440	-1.00	-0.16
Ell	chr8	73063573	73063724	-2.49	-0.72
Ell	chr8	73109061	73115709	-19.10	-0.74
Sin3b	chr8	75247186	75248002	-1.72	-0.30
Sin3b	chr8	75273755	75273906	-1.47	-0.52
Sin3b	chr8	75280901	75281383	-3.95	-0.67
Nr3c2	chr8	79432403	79432554	-0.61	1.74
Nr3c2	chr8	79433567	79433776	-0.74	-1.52
Cbfb	chr8	107694632	107694812	-1.65	-0.29
Zfp90	chr8	108947910	108948329	-2.59	0.80
Zfp90	chr8	108948657	108949616	-1.45	0.33
Zfp1	chr8	114190002	114193957	-0.62	0.14
Zfp1	chr8	114194046	114194762	-2.59	-0.49
Banp	chr8	124550400	124550731	-0.86	-0.47
Banp	chr8	124551389	124551899	-1.05	-0.63
Banp	chr8	124552078	124552768	-1.73	-0.70
Zfpm1	chr8	124806190	124856010	-4.04	0.63
Zfpm1	chr8	124859027	124861147	-0.69	-0.07
Ash2l	chr8	26950536	26958082	-1.11	-0.21
Brf2	chr8	28234303	28236503	-0.63	-0.10
Brf2	chr8	28238956	28239104	-0.66	-0.28
Wrn	chr8	34346314	34346884	-1.70	0.55
Wrn	chr8	34434722	34439590	-0.78	0.26
Cnot7	chr8	41592761	41593001	-0.56	0.08
Ing2	chr8	48753850	48758943	-1.32	0.15
Ing2	chr8	48760272	48760513	-0.64	-0.35
Gatad2a	chr8	72433183	72435724	-28.20	-0.49
Gatad2a	chr8	72459749	72459840	-2.26	-0.26
Gatad2a	chr8	72498029	72498150	-0.37	-0.11
Gatad2a	chr8	72519918	72520248	-1.28	-0.35
Nr2f6	chr8	73898225	73905851	-12.70	-0.57
Med26	chr8	75019202	75072090	-10.00	-0.44
Smarca5	chr8	83263088	83263358	-1.04	-0.21
Csnk2a2	chr8	97976954	97977225	-0.35	-0.03
Psmb10	chr8	108461631	108461967	-2.80	-0.63
Psmd7	chr8	110104338	110108077	-4.03	0.12
Dhx38	chr8	112071923	112073753	-0.53	0.05
Dhx38	chr8	112080084	112080835	-6.56	0.50
Dhx38	chr8	112084553	112089501	-0.31	0.00
Taf1c	chr8	122121770	122123330	-0.82	-0.10
Maml2	chr9	13424582	13425122	-2.71	0.92
Maml2	chr9	13510293	13511342	-0.77	0.17

Taf1d	chr9	15112220	15113253	-6.13	0.25
Zfp317	chr9	19451183	19452859	-22.30	0.60
Pin1	chr9	20456573	20459918	-1.39	-0.27
Smarca4	chr9	21505589	21507505	-3.30	0.23
Zfp202	chr9	40014989	40015898	-2.39	-0.75
Zfp202	chr9	40018534	40019996	-1.21	0.18
Usp2	chr9	43883786	43884026	-0.81	2.12
Usp2	chr9	43902032	43902302	-0.96	-0.35
H2afx	chr9	44143446	44144156	-0.77	-0.05
Ppp2r1b	chr9	50687990	50688710	-0.71	-0.09
Ppp2r1b	chr9	50701016	50701675	-4.03	-0.60
Sin3a	chr9	56958532	56958892	-0.32	-0.02
Sin3a	chr9	56973115	56975576	-0.84	0.11
Snapc5	chr9	64027103	64027192	-0.58	-0.24
Snapc5	chr9	64030056	64030232	-0.39	-0.07
Ints14	chr9	64809670	64809731	-0.94	0.46
Ints14	chr9	64833706	64834453	-0.78	0.08
Rora	chr9	69226715	69226835	-0.32	0.16
Ice2	chr9	69263470	69263950	-0.82	0.16
Ice2	chr9	69280013	69280223	-0.86	0.36
Leo1	chr9	75289330	75294926	-0.87	-0.07
Atr	chr9	95765579	95765669	-0.85	-0.40
Tfdp2	chr9	96218548	96218699	-1.00	0.56
Ctnnb1	chr9	120842577	120859669	-0.34	-0.01
Ctnnb1	chr9	120867712	120868847	-5.68	0.15
Zkscan7	chr9	122797636	122797876	-0.42	-0.24
Zkscan7	chr9	122803837	122804973	-0.30	0.02
Med17	chr9	15066594	15066715	-0.33	0.04
Zfp558	chr9	18260656	18261436	-1.57	0.79
Zfp426	chr9	20274940	20276201	-1.87	0.28
Zfp266	chr9	20304179	20306501	-0.78	-0.10
Kmt2a	chr9	44612636	44614167	-3.81	0.25
Kmt2a	chr9	44615605	44617182	-1.06	0.16
Kmt2a	chr9	44625809	44629185	-31.10	0.48
Kmt2a	chr9	44629634	44630414	-7.20	0.44
Kmt2a	chr9	44655111	44656174	-7.56	0.48
Kmt2a	chr9	44656473	44656924	-1.40	0.24
Kmt2a	chr9	44657403	44658123	-21.00	0.79
Atm	chr9	53247503	53248264	-1.55	0.23
Pml	chr9	58066214	58066305	-1.33	0.78
Pml	chr9	58067472	58068881	-10.10	-0.47
Pml	chr9	58078383	58097563	-4.44	-0.27
Smad3	chr9	63496369	63496969	-0.55	0.19

Smad3	chr9	63497327	63498046	-0.81	0.32
Smad3	chr9	63605729	63605864	-0.51	-1.42
Rnf111	chr9	70274555	70277390	-0.83	-0.10
Rnf111	chr9	70323650	70350792	-12.70	0.37
Tcf12	chr9	71693887	71694367	-8.81	0.54
Tcf12	chr9	71958621	71958922	-1.18	0.36
Tcf12	chr9	71959385	71959626	-0.68	-0.16
Atrip	chr9	108960445	108961754	-16.60	-0.43
Atrip	chr9	108967670	108968299	-1.36	-0.26
Cnot10	chr9	114541870	114549228	-1.75	-0.36
Zfp445	chr9	122761016	122763080	-42.10	0.58
Zfp445	chr9	122770871	122775124	-1.55	0.17
Cited2	chr10	17443033	17444853	-33.40	-0.20
Sgk1	chr10	21718544	21718843	-0.78	0.37
Sgk1	chr10	21719081	21719290	-4.21	1.23
Sesn1	chr10	41530379	41607293	-0.28	0.00
Sesn1	chr10	41627347	41627735	-0.40	-0.14
Ranbp2	chr10	57939345	57940486	-6.91	0.22
Ranbp2	chr10	57940784	57941055	-0.33	-0.01
Ranbp2	chr10	57941564	57941955	-1.34	-0.09
Ranbp2	chr10	57942134	57945844	-28.50	0.35
Ranbp2	chr10	57955388	57956782	-8.30	0.31
Stk11	chr10	79579091	79588239	-0.80	0.07
Stk11	chr10	79592650	79593215	-1.40	-0.20
Txnrd1	chr10	82296755	82323149	-0.39	-0.07
Txnrd1	chr10	82323297	82335784	-5.40	-0.55
Polr3b	chr10	84188455	84188935	-1.93	-0.31
E2f7	chr10	110216189	110216249	-0.35	-0.75
Smarcc2	chr10	127920261	127920695	-0.29	0.00
Smarcc2	chr10	127924543	127924604	-1.40	-0.59
Smarcc2	chr10	127926123	127926244	-0.38	0.05
Gtf3c6	chr10	39969066	39969593	-5.05	0.57
Ddit4	chr10	59412924	59414518	-67.50	-0.91
Ddx21	chr10	62044843	62047448	-7.21	-0.17
Ddx21	chr10	62057824	62061631	-34.70	-0.29
Sirt1	chr10	62783154	62785044	-44.20	1.03
Sirt1	chr10	62801424	62801783	-0.98	0.38
Nrbf2	chr10	66729823	66730867	-2.28	-0.23
Tfam	chr10	70690765	70691424	-0.60	-0.05
Tfam	chr10	70700740	70701021	-0.37	-0.14
Ube2d1	chr10	70718144	70718592	-2.63	0.38
Smarcb1	chr10	75359602	75359780	-1.07	-0.22
Polrmt	chr10	79201183	79202890	-3.98	-0.40

Polrmt	chr10	79206001	79209296	-4.02	-0.45
Polr2e	chr10	79501316	79502404	-2.85	-0.43
Mbd3	chr10	79855285	79856691	-7.71	-0.28
Tcf3	chr10	79872416	79873314	-1.26	-0.16
Cradd	chr10	94637889	94638400	-0.78	0.33
Cradd	chr10	94785366	94785547	-0.28	0.04
Cnot2	chr10	115922246	115922606	-0.99	-0.19
Cnot2	chr10	115922874	115923144	-4.87	0.64
Yeats4	chr10	116652465	116652884	-0.70	0.11
Mdm2	chr10	117126170	117126441	-1.00	0.08
Mdm2	chr10	117126799	117129718	-14.10	0.17
Dyrk2	chr10	118296404	118305900	-0.44	-0.03
Pip4k2c	chr10	126635286	126635944	-0.36	-0.01
Pip4k2c	chr10	126636730	126637327	-0.37	0.03
Cdk2	chr10	128135921	128136041	-0.48	-0.19
Psme4	chr11	30671774	30672135	-0.54	-0.18
Slu7	chr11	43247232	43251659	-1.97	0.22
Slu7	chr11	43258751	43259812	-3.05	-0.23
Slu7	chr11	43260795	43260976	-0.25	0.11
Med7	chr11	46250448	46253332	-0.31	0.02
Med7	chr11	46254128	46254877	-4.70	0.38
Ppp2ca	chr11	51912325	51912474	-4.51	-0.88
Ppp2ca	chr11	51935449	51935836	-0.34	0.00
Aff4	chr11	53164268	53186578	-4.92	-0.19
Aff4	chr11	53211860	53216039	-0.32	0.00
Aff4	chr11	53225470	53229192	-24.70	0.81
Cnot8	chr11	57930958	57931258	-0.74	0.18
Zkscan6	chr11	65636352	65642442	-3.43	-0.39
Aurkb	chr11	68861818	68863941	-3.92	-0.59
Trp53	chr11	69393860	69394608	-0.43	-0.06
Mybbp1a	chr11	72264572	72265052	-13.50	-0.33
Ywhae	chr11	75546388	75546567	-5.18	-0.26
Suz12	chr11	79843530	79845978	-0.38	0.01
Cdk5r1	chr11	80291441	80292571	-1.52	0.38
Cdk5r1	chr11	80293343	80293493	-0.43	0.26
Cdk5r1	chr11	80291445	80292555	-1.61	0.40
Cdk5r1	chr11	80293392	80293483	-0.29	0.10
Taf15	chr11	83286609	83286670	-0.74	-0.19
Ppm1d	chr11	85124755	85125383	-7.40	-0.93
Ppm1d	chr11	85140378	85140529	-1.24	0.48
Ppm1d	chr11	85153180	85159827	-0.39	-0.03
Supt4a	chr11	87557059	87557119	-0.31	-0.09
Srsf1	chr11	87861172	87861322	-1.85	-0.33

Cdk12	chr11	98064618	98065548	-5.37	-0.20
Cdk12	chr11	98071707	98072546	-3.00	-0.14
Cdk12	chr11	98104793	98107067	-2.29	0.20
Cdk12	chr11	98111003	98111783	-7.97	-0.40
Psmd3	chr11	98543867	98546829	-30.30	-0.74
Thra	chr11	98603186	98614709	-0.74	0.42
Thra	chr11	98614768	98617477	-2.58	-0.78
Thra	chr11	98625600	98626259	-4.68	-0.69
Thra	chr11	98629482	98629782	-10.30	1.38
Casc3	chr11	98683710	98689970	-6.76	-0.34
Casc3	chr11	98692721	98694074	-0.45	0.04
Rara	chr11	98799009	98808908	-1.61	-0.58
Rara	chr11	98808997	98822329	-5.35	-0.55
Rara	chr11	98827782	98829451	-0.74	-0.25
Rara	chr11	98831600	98835508	-19.40	-0.66
Psme3	chr11	101177564	101178336	-0.62	0.07
Psme3	chr11	101182070	101183288	-0.64	-0.06
Tnrc6c	chr11	117515962	117562114	-0.37	0.09
Tnrc6c	chr11	117575512	117593595	-17.40	-0.46
Tnrc6c	chr11	117621843	117622474	-1.79	-0.38
Tnrc6c	chr11	117623163	117623733	-1.00	0.30
Cbx2	chr11	118889293	118890939	-23.30	-1.92
Rptor	chr11	119465086	119517298	-1.88	-0.26
Rptor	chr11	119759737	119760755	-0.53	0.05
Hus1	chr11	8896711	8896861	-0.56	0.17
Lsm11	chr11	45747027	45747446	-3.16	-0.71
Maml1	chr11	50069912	50070420	-0.98	0.21
Maml1	chr11	50071136	50074538	-0.86	-0.10
Maml1	chr11	50076892	50105838	-2.71	-0.20
Rad50	chr11	53463650	53465769	-2.93	-0.38
Zkscan17	chr11	59299022	59299382	-2.54	0.44
Zkscan17	chr11	59300338	59306167	-1.66	-0.14
Zkscan17	chr11	59319782	59319933	-1.97	-1.06
Top3a	chr11	60553560	60555499	-1.34	0.26
Top3a	chr11	60555857	60557535	-1.26	0.23
Top3a	chr11	60590477	60590867	-9.76	1.46
Ncor1	chr11	62130880	62131511	-1.35	0.12
Ncor1	chr11	62147246	62147337	-0.41	-0.06
Ncor1	chr11	62182924	62186997	-26.60	-0.65
Ncor1	chr11	62196845	62198278	-2.94	-0.40
Ncor1	chr11	62252002	62270948	-5.64	-1.11
Zfp287	chr11	62527053	62527804	-2.03	0.58
Zfp287	chr11	62527953	62528373	-1.76	0.62

Zfp287	chr11	62528582	62528703	-0.25	-0.02
Zfp287	chr11	62541753	62542444	-0.32	0.04
Zfp287	chr11	62545196	62545317	-1.08	1.22
Chd3	chr11	69157463	69157973	-5.22	-0.48
Chd3	chr11	69174956	69175626	-0.72	-0.14
Chd3	chr11	69177569	69182868	-4.44	-0.54
Rpa1	chr11	75114477	75116219	-1.52	-0.18
Rpa1	chr11	75154547	75161885	-1.59	0.39
Supt6	chr11	78020490	78021581	-6.20	-0.26
Supt6	chr11	78044521	78044672	-9.16	-0.50
Supt6	chr11	78047130	78059175	-4.29	-0.45
Rffl	chr11	82619321	82632040	-24.80	-0.71
Brip1	chr11	85874715	85878344	-2.90	0.50
Med13	chr11	86084344	86084585	-0.84	0.15
Med13	chr11	86100121	86100212	-2.56	-0.43
Med13	chr11	86104608	86114217	-1.33	0.09
Med13	chr11	86132926	86141980	-0.71	0.05
Pcgf2	chr11	97551180	97551450	-9.46	-1.52
Pcgf2	chr11	97553219	97553423	-0.65	0.22
Pip4k2b	chr11	97578204	97584269	-3.42	-0.14
Med1	chr11	98014158	98015024	-2.02	0.27
Med1	chr11	98015173	98015684	-0.74	-0.09
Med1	chr11	98016102	98017333	-0.59	0.04
Med1	chr11	98017662	98017873	-2.97	-0.38
Med1	chr11	98017962	98018233	-0.58	0.07
Med1	chr11	98018352	98019732	-1.59	0.09
Med1	chr11	98050576	98054607	-1.81	-0.28
Med24	chr11	98590218	98590689	-0.90	0.32
Nr1d1	chr11	98629338	98631545	-20.50	1.35
Nr1d1	chr11	98631693	98632599	-4.38	0.49
Nr1d1	chr11	98633166	98636556	-0.67	0.09
Kat2a	chr11	100566059	100566924	-11.10	-0.42
Kat2a	chr11	100573600	100573691	-0.37	-0.10
Cavin1	chr11	100819819	100820270	-0.70	1.83
Brca1	chr11	101384784	101385234	-1.07	-0.42
Brca1	chr11	101385503	101386581	-0.66	0.14
Brca1	chr11	101386909	101387659	-0.94	0.23
Ubtf	chr11	102167282	102168062	-1.03	-0.09
Smarcd2	chr11	106133897	106134286	-1.29	-0.39
Smurf2	chr11	106683743	106684388	-0.37	-0.04
Smurf2	chr11	106781609	106781789	-3.23	-1.30
Srsf2	chr11	116714108	116714408	-50.00	-0.61
Cbx8	chr11	118899838	118901471	-0.31	0.01

Cbx4	chr11	118941130	118941400	-2.21	0.97
Cbx4	chr11	118941729	118947491	-3.35	-0.13
Dnmt3a	chr12	3806989	3807238	-0.61	0.39
Dnmt3a	chr12	3835069	3866198	-0.42	-0.04
Dnmt3a	chr12	3906804	3908006	-0.38	0.01
E2f6	chr12	16817830	16817920	-1.61	-0.89
E2f6	chr12	16832231	16832859	-2.23	-0.27
Twistnb	chr12	34121850	34123203	-0.59	0.04
Ppm1a	chr12	73862227	73885470	-1.34	-0.08
Ppm1a	chr12	73894914	73895183	-2.86	0.44
Snapc1	chr12	75065516	75068917	-5.03	-0.85
Srsf5	chr12	82046490	82046729	-16.60	-0.48
Srsf5	chr12	82050780	82051078	-44.00	-0.66
Psmc1	chr12	101348377	101351316	-1.50	-0.23
Cpsf2	chr12	103214183	103214244	-0.29	-0.03
Cpsf2	chr12	103240910	103241994	-0.60	-0.04
Papola	chr12	107022971	107023092	-4.18	-0.34
Papola	chr12	107072945	107075506	-2.32	-0.11
Ccnk	chr12	109418007	109424620	-0.44	0.06
Zfp839	chr12	112088488	112099099	-6.28	-0.75
Zfp839	chr12	112106449	112107344	-2.89	-0.47
Mta1	chr12	114374970	114375239	-4.02	-0.39
Ywhaq	chr12	21422717	21423076	-5.19	-0.53
Ywhaq	chr12	21423381	21423442	-0.93	0.75
Max	chr12	78038822	78039718	-3.26	-0.11
Max	chr12	78063025	78063205	-5.46	-0.52
Med6	chr12	82674550	82674905	-2.82	0.39
Snw1	chr12	88791126	88791810	-8.21	-0.35
Gtf2a1	chr12	92797890	92806058	-4.85	0.27
Dicer1	chr12	105929669	105930338	-1.00	0.20
Dicer1	chr12	105932749	105935476	-13.40	-0.48
Dicer1	chr12	105936045	105941366	-1.29	0.14
Ppp1r13b	chr12	113066998	113068529	-0.52	-0.10
Ppp1r13b	chr12	113070821	113073439	-7.32	-0.51
Ppp1r13b	chr12	113146175	113146266	-0.46	1.29
Akt1	chr12	113912217	113912487	-1.97	-0.56
Brf1	chr12	114198252	114199483	-4.74	-0.45
Brf1	chr12	114200643	114200794	-0.31	-0.02
Zkscan4	chr13	21576264	21576865	-1.13	-1.76
Hist1h3h	chr13	21809496	21809698	-1.42	0.30
Hist1h3f	chr13	23636303	23636393	-2.07	-0.34
Hist1h2bg	chr13	23663268	23663474	-1.13	-0.09
Hist1h3d	chr13	23667608	23667786	-1.12	-0.25

Hist1h2bc	chr13	23776067	23776213	-2.98	0.39
Hist1h2ab	chr13	23842956	23843254	-0.48	0.06
Jarid2	chr13	44980362	45002001	-14.00	-0.39
Prelid1	chr13	55423415	55423591	-1.89	-0.18
Rmi1	chr13	58503698	58503988	-0.54	-0.08
Rmi1	chr13	58508365	58511252	-1.36	0.13
Zfp369	chr13	65397670	65398417	-5.12	-0.73
Med10	chr13	69954526	69954824	-5.28	-0.32
Ell2	chr13	75844961	75845111	-1.04	-0.52
Ell2	chr13	75899664	75901521	-116.00	-1.03
Taf9	chr13	101435858	101436098	-1.55	0.21
Plk2	chr13	111185251	111185700	-14.20	-0.89
Plk2	chr13	111190024	111191021	-3.14	-0.32
Slc38a9	chr13	113528209	113528690	-2.86	1.40
Cdk13	chr13	17809653	17811567	-1.53	0.10
Cdk13	chr13	17858358	17896931	-3.76	-0.17
Zkscan3	chr13	21479260	21480454	-4.71	0.32
Zkscan3	chr13	21488062	21494295	-3.85	-0.57
Zkscan8	chr13	21611941	21613722	-2.34	-0.34
Zkscan8	chr13	21617156	21618788	-0.43	0.10
Hist1h3i	chr13	21875180	21875266	-0.41	0.06
Hist1h3c	chr13	23837329	23837390	-0.31	-0.03
Tfap2a	chr13	40812539	40812719	-0.15	0.29
Nup153	chr13	46776258	46776559	-2.23	0.27
Nup153	chr13	46782040	46792068	-1.16	0.05
Nup153	chr13	46822738	46823218	-7.57	-0.49
Dek	chr13	47201073	47201589	-1.54	0.34
Thoc3	chr13	54561210	54561569	-0.42	-0.04
Ctsl	chr13	64470393	64471614	-17.40	-0.32
Zfp729a	chr13	67719104	67720063	-10.30	0.99
Zfp729a	chr13	67720451	67721051	-2.35	0.38
Zfp729a	chr13	67721469	67722248	-0.63	0.15
Zfp738	chr13	67768467	67769574	-3.21	0.63
Zfp738	chr13	67770082	67770382	-0.89	0.38
Zfp738	chr13	67771278	67771698	-0.34	-0.07
Ice1	chr13	70741154	70747297	-1.12	0.05
Ice1	chr13	70747386	70748030	-1.07	-0.20
Nr2f1	chr13	78328745	78328956	-0.58	-2.91
Polr3g	chr13	81814392	81815052	-0.31	0.02
Jmy	chr13	94204723	94212614	-10.00	0.49
Jmy	chr13	94242583	94269644	-4.59	-0.33
Bdp1	chr13	100793718	100793779	-3.61	0.91
Bdp1	chr13	100830496	100834368	-1.24	-0.08

Bdp1	chr13	100869178	100873965	-4.70	-0.47
Rad17	chr13	101387447	101389058	-1.23	-0.21
Eaf1	chr14	32308264	32308534	-4.02	-0.47
Eaf1	chr14	32313335	32319971	-45.70	-0.71
Ercc6	chr14	33326706	33332157	-4.43	-0.52
Ercc6	chr14	33339398	33354435	-17.70	-0.69
Ercc6	chr14	33382960	33390463	-25.10	-0.63
Psmc6	chr14	45949498	45950514	-0.60	-0.09
Ints9	chr14	65658302	65658483	-0.69	-0.21
Med4	chr14	73909855	73910973	-1.63	-0.58
Med4	chr14	73917759	73918293	-3.57	-0.40
Elf1	chr14	79881419	79936023	-1.87	-0.46
Elf1	chr14	79973114	79980877	-2.36	0.14
Rarb	chr14	17407447	17451237	-0.51	-0.82
Nr1d2	chr14	19038938	19040485	-19.30	1.58
Nr1d2	chr14	19047334	19049180	-7.51	0.70
Polr3a	chr14	25267945	25269957	-3.13	-0.42
Pip4p1	chr14	51546074	51546434	-0.32	-0.01
Supt16	chr14	52780871	52782395	-6.16	-0.21
Supt16	chr14	52803669	52816914	-2.64	-0.39
Prmt5	chr14	55126018	55126553	-0.91	-0.11
Rabggta	chr14	56340520	56340807	-0.58	0.19
Cenpj	chr14	57145687	57148622	-0.29	-0.01
Cenpj	chr14	57170788	57171655	-0.31	-0.01
Cenpj	chr14	57171744	57172312	-0.62	0.14
Cenpj	chr14	57183992	57190683	-0.52	0.50
Ints6	chr14	63296661	63296842	-0.41	0.66
Ints6	chr14	63315432	63319557	-52.60	1.62
Polr3d	chr14	70838853	70840064	-7.86	-0.39
Polr3d	chr14	70841182	70842169	-3.93	-0.62
Gtf2f2	chr14	76297012	76306651	-1.19	-0.13
Prkaa1	chr15	5093860	5110647	-7.59	-0.91
Prkaa1	chr15	5126670	5126820	-1.22	-0.18
Prkaa1	chr15	5127194	5129214	-5.38	0.43
Rictor	chr15	6736456	6737266	-1.44	-0.13
Rictor	chr15	6745958	6747284	-0.93	0.10
Polr2k	chr15	36103800	36104751	-0.34	0.08
Zfp707	chr15	75804010	75804159	-0.65	-0.41
Zfp707	chr15	75804871	75806149	-11.00	-0.97
Tnrc6b	chr15	80709413	80711723	-2.49	0.18
Tnrc6b	chr15	80759640	80760240	-1.22	-0.35
Rbx1	chr15	81305971	81306061	-1.28	-0.57
Ep300	chr15	81416643	81438767	-12.50	-0.29

Ep300	chr15	81479273	81481819	-5.01	-0.13
L3mbtl2	chr15	81516741	81518556	-3.63	-0.29
Nup50	chr15	84757805	84765822	-13.80	-0.39
Nup50	chr15	84770131	84771000	-9.31	-0.39
Nup50	chr15	84773033	84773303	-0.67	0.14
Arid2	chr15	96117952	96118043	-0.28	-0.02
Arid2	chr15	96199968	96201175	-7.90	0.36
Arid2	chr15	96201654	96209326	-6.30	0.26
Arid2	chr15	96223066	96232715	-1.11	-0.22
Nr4a1	chr15	101097276	101102664	-424.00	-1.45
Nr4a1	chr15	101103587	101105046	-137.00	-1.52
Zfp740	chr15	102034168	102035505	-2.24	-0.24
Zfp740	chr15	102039660	102043940	-1.65	-0.13
Sp1	chr15	102238241	102260424	-0.48	0.02
Sp1	chr15	102260572	102261651	-2.06	0.20
Skp2	chr15	9043329	9043510	-0.95	0.41
Skp2	chr15	9069207	9070137	-7.83	-1.04
Ywhaz	chr15	36723285	36723376	-0.55	0.11
Zfp706	chr15	36936977	36937157	-1.44	-0.45
Taf2	chr15	54847403	54848124	-0.49	-0.06
Taf2	chr15	54903436	54903647	-0.61	-0.15
Atad2	chr15	57928856	57930279	-4.77	0.52
Atad2	chr15	57966457	57966637	-0.74	-0.28
Ago2	chr15	72935769	72936310	-4.95	-0.27
Ago2	chr15	72936518	72937686	-1.98	0.08
Ago2	chr15	73015256	73015377	-5.30	-1.25
Cbx6	chr15	79658129	79664703	-7.13	-0.57
Polr3h	chr15	81746470	81746890	-1.47	-0.26
Polr3h	chr15	81755745	81756015	-0.27	0.01
Poldip3	chr15	82958023	82959641	-1.64	-0.10
Brd1	chr15	88518753	88518814	-4.89	0.89
Brd1	chr15	88543169	88543809	-0.49	-0.05
Brd1	chr15	88547455	88564529	-12.40	0.36
Yaf2	chr15	93115848	93167246	-0.88	0.11
Vdr	chr15	97687063	97687362	-0.53	1.58
Ccnt1	chr15	98373641	98375016	-10.90	0.30
Ccnt1	chr15	98398086	98398654	-9.19	1.21
Prkag1	chr15	98643704	98643794	-0.45	-0.08
Rarg	chr15	102066030	102069302	-12.80	-0.78
Zfp385a	chr15	103144952	103145191	-1.58	-0.37
Zfp385a	chr15	103150806	103164738	-0.29	0.01
Zfp263	chr16	3744098	3745056	-3.71	-0.36
Zfp263	chr16	3748158	3750040	-1.24	0.09

Rrn3	chr16	13813284	13813585	-0.75	-0.11
Psmd2	chr16	20651724	20652324	-20.60	-0.49
Ncbp2	chr16	31957240	31957480	-1.22	-0.23
Fyttd1	chr16	32877869	32884216	-2.17	-0.38
Fyttd1	chr16	32902616	32905812	-4.94	-0.62
Gsk3b	chr16	38089086	38090434	-0.68	-0.05
Zfp597	chr16	3865585	3871203	-2.18	0.33
Crebbp	chr16	4084047	4087773	-0.48	-0.02
Crebbp	chr16	4154961	4213404	-3.27	-0.16
Usp7	chr16	8738344	8757028	-0.78	-0.28
Gtf2e1	chr16	37510570	37511021	-1.83	-0.31
Gtf2e1	chr16	37511589	37515969	-0.39	0.04
Gtf2e1	chr16	37522998	37539825	-0.52	-0.07
Runx1	chr16	92605608	92606269	-0.70	0.06
Runx1	chr16	92695902	92697552	-25.50	0.56
Arid1b	chr17	4995073	4995882	-1.66	-0.67
Arid1b	chr17	4996061	5097577	-3.74	-0.55
Arid1b	chr17	5332607	5337057	-2.55	0.24
Arid1b	chr17	5337116	5339263	-0.52	0.10
Arid1b	chr17	5341518	5343345	-1.18	0.09
Zfp160	chr17	21162599	21164033	-0.57	0.06
Zfp160	chr17	21164271	21164332	-0.31	0.21
Zfp160	chr17	21178510	21179347	-0.31	0.05
Rnps1	chr17	24551619	24552074	-1.31	-0.33
Ppard	chr17	28369758	28432393	-6.75	-0.70
Ppard	chr17	28435930	28437576	-21.80	-0.79
Mapk14	chr17	28828316	28828704	-3.84	-0.61
Mapk14	chr17	28884007	28885350	-1.27	0.08
Brpf3	chr17	28938100	28938251	-0.36	0.50
Brpf3	chr17	28943116	28944224	-7.70	0.75
Brpf3	chr17	28954048	28960704	-0.96	0.16
Brpf3	chr17	28973668	28973878	-0.46	0.17
Cdkn1a	chr17	29228020	29235694	-168.00	-0.80
Daxx	chr17	34047504	34050602	-124.00	-0.92
Rxrb	chr17	34174187	34175165	-2.09	-0.18
Psmb8	chr17	34335288	34335468	-2.22	-0.33
Ehmt2	chr17	35042119	35042557	-5.05	-0.58
Ehmt2	chr17	35049712	35050992	-0.63	0.07
Ddx39b	chr17	35378720	35378917	-1.75	-0.31
Ddx39b	chr17	35386162	35386790	-0.58	-0.04
Ddx39b	chr17	35566041	35566729	-1.02	-0.30
Med20	chr17	47759792	47760513	-3.12	-0.33
Zfp213	chr17	23694178	23694744	-0.80	0.45

Zfp213	chr17	23694951	23696378	-0.41	-0.22
Thoc6	chr17	23810648	23810831	-0.28	0.01
Pdpk1	chr17	24215553	24216093	-11.80	0.63
Mlst8	chr17	24612351	24613045	-0.59	-0.10
Ube2i	chr17	25398055	25398776	-6.49	-0.64
Stub1	chr17	25967580	25967909	-3.51	-0.27
Stub1	chr17	25969947	25970008	-0.31	-0.35
Stub1	chr17	25970096	25970306	-1.82	-0.63
Taf11	chr17	28038342	28038672	-4.35	-0.52
Taf11	chr17	28042348	28045188	-1.94	-0.31
Notch3	chr17	32278193	32278314	-0.50	2.81
Ring1	chr17	34158095	34160251	-0.84	-0.10
Brd2	chr17	34249292	34249918	-19.30	0.29
Brd2	chr17	34251401	34252141	-0.53	0.02
Brd2	chr17	34254036	34257250	-434.00	-1.12
Runx2	chr17	44744958	44745199	-11.00	1.69
Runx2	chr17	44872639	44873298	-1.80	-0.39
Taf8	chr17	47625387	47626945	-7.66	-0.45
Taf8	chr17	47638265	47639236	-2.89	-0.65
Mllt1	chr17	57035733	57045221	-14.60	-0.53
Gtf2f1	chr17	57143372	57143559	-0.74	-0.13
Tgif1	chr17	71193544	71195840	-57.00	-0.42
Tgif1	chr17	71200227	71202842	-14.10	-0.47
Srsf7	chr17	80606178	80606645	-0.35	-0.03
Rbbp8	chr18	11880308	11881195	-1.41	0.17
Taf4b	chr18	14971627	14971957	-0.31	0.02
Polr2d	chr18	31955877	31956355	-0.34	-0.02
Wdr33	chr18	31963710	31986988	-10.60	-0.69
Wdr33	chr18	32047426	32048936	-16.50	-0.52
Wdr33	chr18	32049204	32067113	-1.24	-0.09
Iws1	chr18	32227387	32243000	-5.29	-0.18
Ercc3	chr18	32399984	32400372	-0.60	-0.12
Ercc3	chr18	32429502	32429682	-1.02	0.23
Phax	chr18	56732636	56746951	-2.10	-0.09
Prelid3a	chr18	67639251	67639460	-1.99	1.09
Rnmt	chr18	68460038	68465572	-0.29	0.00
Smad7	chr18	75527018	75527675	-0.56	0.13
Smad7	chr18	75528299	75554128	-4.14	0.55
Smad2	chr18	76462139	76464727	-1.34	-0.19
Epc1	chr18	6436931	6440144	-10.10	0.66
Epc1	chr18	6441163	6450203	-18.10	1.12
Epc1	chr18	6490689	6515876	-1.67	-0.51
Kctd1	chr18	15127880	15128090	-0.86	2.17

Kctd1	chr18	15128179	15132643	-0.22	0.12
Kctd1	chr18	15166356	15220489	-0.39	-0.45
Kctd1	chr18	15220578	15220848	-0.65	1.31
Kctd1	chr18	15221623	15221804	-0.65	-1.71
Taf7	chr18	37801809	37803149	-6.84	0.58
Nr3c1	chr18	39588392	39650238	-0.66	-0.04
Smad4	chr18	73799858	73800008	-0.35	-0.03
Smad4	chr18	73837391	73837750	-0.38	-0.03
Smad4	chr18	73863156	73863395	-1.93	-1.18
Ctdp1	chr18	80605326	80630972	-2.69	0.27
Ctdp1	chr18	80642798	80647936	-2.79	-0.16
Men1	chr19	6334978	6336781	-0.48	-0.04
Men1	chr19	6338873	6340864	-2.26	-0.13
Ints5	chr19	8967596	8971721	-3.22	-0.17
Ints5	chr19	8971870	8972350	-0.36	-0.03
Cpsf7	chr19	10614133	10620757	-1.51	0.08
Cpsf7	chr19	10621625	10621806	-12.40	0.88
Smarca2	chr19	26705324	26705445	-2.19	-1.03
Cstf2t	chr19	31157390	31159177	-2.75	0.13
Pcgf5	chr19	36484296	36486641	-0.57	0.12
Pcgf5	chr19	36519525	36530396	-3.63	-0.53
Taf5	chr19	47145518	47149502	-0.56	-0.15
Taf5	chr19	47156922	47157401	-0.43	-0.08
Rad9a	chr19	4195437	4196375	-0.34	-0.01
Rbm14	chr19	4743957	4744585	-0.74	-0.21
Rbm14	chr19	4803462	4811634	-0.83	-0.05
Kat5	chr19	5603043	5603836	-2.52	-0.22
Kat5	chr19	5609321	5609592	-0.57	-0.12
Kat5	chr19	5609854	5610034	-0.61	-0.31
Prdx5	chr19	6984015	6984135	-10.60	-0.65
Esrra	chr19	6996237	6996298	-0.43	-0.27
Polr2g	chr19	8872986	8873047	-0.44	-0.14
Ldb1	chr19	46107090	46108545	-0.46	-0.03
Pcgf6	chr19	47108856	47109007	-0.32	0.04
Pcgf6	chr19	47125005	47125335	-10.30	-1.25
Usp9x	chrX	12733152	12733872	-13.20	0.35
Med12	chrX	98488099	98488220	-1.26	0.32
Taf1	chrX	98794675	98794885	-1.77	-0.35
Rragb	chrX	149574678	149575911	-1.22	0.33
Suv39h1	chrX	7647858	7649470	-1.89	-0.30
Upf3b	chrX	34632248	34632639	-0.43	-0.05
Thoc2	chrX	39150625	39150955	-4.71	0.47
G6pdx	chrX	71655299	71655479	-0.33	-0.01

Chm	chrX	110156917	110157037	-0.85	0.35
Rad9b	chr5	122803670	122803730	-0.23	0.81
Rad9b	chr5	122803907	122804085	-0.68	0.80

RNA polymerase II transcription

gene_name	chr	chromStart	chromEnd	diff.lg.p	diff.log2.fc
Cnot11	chr1	39603302	39603632	-9.27	-1.03
Cnot9	chr1	74575438	74575798	-1.13	-0.18
Ing5	chr1	95713260	95715951	-3.10	0.35
Ccnt2	chr1	129698247	129700591	-5.04	0.20
Rbbp5	chr1	134394620	134400982	-0.63	-0.07
Rbbp5	chr1	134401101	134401282	-1.08	0.24
Kdm5b	chr1	136514466	136514617	-0.28	0.01
Kdm5b	chr1	136521370	136521700	-0.83	-0.21
Kdm5b	chr1	136525318	136527418	-2.33	-0.37
Kdm5b	chr1	136527777	136528227	-0.52	0.09
Nr1i3	chr1	173148035	173148455	-0.57	-0.14
Nr1i3	chr1	173148844	173149204	-1.85	-0.52
Exo1	chr1	177829710	177831466	-2.00	-0.72
Exo1	chr1	177839222	177839403	-0.50	-2.10
Parp1	chr1	182499105	182503813	-1.96	-0.45
Trp53bp2	chr1	184371133	184371894	-0.32	-0.03
Trp53bp2	chr1	184376642	184379234	-2.02	-0.28
Trp53bp2	chr1	184391818	184392328	-2.00	-0.40
Ints7	chr1	193443646	193445360	-2.16	-0.35
Nabp1	chr1	51523300	51523510	-1.13	1.14
Nabp1	chr1	51528047	51529205	-6.86	1.58
Nabp1	chr1	51535022	51535263	-1.91	0.77
Gls	chr1	52244535	52244686	-10.30	0.83
Gls	chr1	52276864	52290076	-11.90	-0.48
Sumo1	chr1	59696455	59697048	-0.33	-0.01
Bard1	chr1	71121361	71122020	-1.25	-0.38
Hdac4	chr1	93829331	93829960	-0.46	0.09
Steap3	chr1	122123232	122123862	-3.78	-0.81
Steap3	chr1	122140355	122166591	-6.06	-1.12
Mdm4	chr1	134888236	134893148	-1.07	-0.08
Zc3h11a	chr1	135516447	135517433	-9.17	-0.31
Zc3h11a	chr1	135517641	135518733	-2.10	-0.10
Zc3h11a	chr1	135521340	135523781	-17.40	-0.37
Btg2	chr1	135973565	135975732	-1.48	0.08
Rnf2	chr1	153318438	153320426	-0.40	0.03
Pou2f1	chr1	167805604	167806174	-0.89	-0.19
Smyd2	chr1	191704578	191704728	-0.49	0.06
Bmi1	chr2	18605667	18606468	-0.37	-0.01

Rxra	chr2	27615158	27615398	-3.09	-0.87
Rxra	chr2	27615487	27616386	-13.50	-1.14
Rxra	chr2	27617342	27618510	-7.46	-0.74
Tsc1	chr2	28531519	28532030	-4.79	-0.58
Tsc1	chr2	28541313	28542943	-0.95	-0.12
Abl1	chr2	31544075	31551290	-7.77	-0.43
Abl1	chr2	31653102	31658189	-1.51	-0.10
Abl1	chr2	31658367	31658428	-0.69	-0.40
Mapkap1	chr2	34479093	34479423	-0.34	0.02
Ssrp1	chr2	84885611	84887238	-5.04	-0.18
Tnks1bp1	chr2	84891036	84904146	-4.25	-0.23
Psmc3	chr2	90894439	90895182	-3.33	-0.20
Lmo2	chr2	103809861	103821531	-3.10	-0.19
Cstf3	chr2	104504891	104505372	-2.93	0.31
Rtf1	chr2	119558176	119559166	-0.81	0.09
Rtf1	chr2	119560243	119560484	-0.48	0.12
Mga	chr2	119698286	119698347	-0.21	0.26
Mga	chr2	119722990	119743194	-1.74	0.09
Mga	chr2	119749246	119752139	-1.06	0.11
Mga	chr2	119767083	119767563	-7.58	-0.49
Mga	chr2	119772610	119774079	-2.10	0.12
Mga	chr2	119787692	119790793	-0.32	0.01
Csnk2a1	chr2	152107019	152107169	-10.00	-0.77
Tpx2	chr2	152717983	152719804	-0.61	0.08
Itch	chr2	154959216	154959307	-0.27	-0.02
Itch	chr2	155050102	155050403	-0.31	-0.01
Phf20	chr2	156093020	156093171	-0.35	-0.03
Phf20	chr2	156124429	156134043	-2.43	-0.18
Tgif2	chr2	156679337	156679783	-0.33	-0.07
Src	chr2	157295136	157296531	-2.35	0.70
Rprd1b	chr2	157900721	157901171	-1.37	0.22
Srsf6	chr2	162757243	162757817	-0.30	0.00
L3mbtl1	chr2	162773851	162773971	-0.35	1.56
Ywhab	chr2	163820932	163837497	-10.90	-0.39
Trp53rkb	chr2	166619352	166619593	-0.34	0.09
Trp53rkb	chr2	166620885	166621755	-0.75	-0.18
Cstf1	chr2	172203242	172206586	-1.17	-0.10
Gata3	chr2	9779597	9784722	-2.88	0.70
Gata3	chr2	9790440	9796250	-0.57	-0.22
Taf3	chr2	9837317	9874531	-2.60	-0.16
Pip4k2a	chr2	18765527	18769115	-4.26	0.31
Ehmt1	chr2	24646742	24647852	-3.96	-0.36
Ehmt1	chr2	24733063	24775080	-0.58	-0.06

Snapc4	chr2	26218284	26222737	-9.30	-0.42
Snapc4	chr2	26223926	26225494	-4.22	-0.53
Notch1	chr2	26314079	26314319	-0.24	0.00
Notch1	chr2	26315305	26315396	-0.28	0.42
Notch1	chr2	26315993	26316443	-0.58	-0.38
Cdk9	chr2	32562910	32565131	-22.70	-0.34
Psmd5	chr2	34707993	34709823	-1.65	-0.19
Psmd5	chr2	34726274	34726482	-4.41	-0.82
Psmb7	chr2	38498946	38499426	-3.13	-0.36
Nr6a1	chr2	38740753	38781783	-1.41	1.01
Nr4a2	chr2	56960145	56961485	-4.91	-0.57
Nr4a2	chr2	56964219	56965507	-14.90	-0.92
Nr4a2	chr2	56967144	56976354	-1.04	0.68
Clp1	chr2	84563518	84566269	-0.31	0.00
Pin1rt1	chr2	104554491	104554756	-2.79	3.80
Zfp770	chr2	114021670	114027138	-1.72	0.21
Zfp661	chr2	127402341	127403416	-0.33	0.03
Snrpb	chr2	130004981	130005100	-1.98	-0.21
Pcna	chr2	132077710	132078916	-7.08	0.28
Psmf1	chr2	151543111	151543202	-1.31	-0.87
Psmf1	chr2	151543649	151544038	-0.30	0.01
Zfp334	chr2	165203384	165203715	-0.29	0.04
Zfp334	chr2	165205241	165206231	-1.16	0.28
Zfp334	chr2	165207038	165207279	-0.43	0.16
Trp53rka	chr2	165315878	165318814	-1.44	-0.19
Aurka	chr2	172182168	172182683	-0.29	0.00
Taf4	chr2	179647388	179656678	-2.00	-0.23
Taf4	chr2	179710752	179710813	-0.34	0.87
Skil	chr3	30994042	30997412	-0.91	-0.07
Skil	chr3	31015852	31017641	-3.54	-0.30
Gatad2b	chr3	90127990	90145879	-1.31	-0.15
Gatad2b	chr3	90155787	90155908	-1.99	-0.28
Hist2h2be	chr3	96025043	96025490	-2.37	-0.78
Hist2h2be	chr3	96026708	96027215	-0.64	-0.19
Hist2h2be	chr3	96027422	96027483	-10.00	3.73
Notch2	chr3	97817460	97817641	-2.65	-0.57
Notch2	chr3	97938527	97941254	-16.60	-0.46
Notch2	chr3	97950009	97954290	-14.00	-0.14
Trim33	chr3	103148225	103148495	-2.77	0.27
Trim33	chr3	103156043	103158233	-4.71	0.28
Trim33	chr3	103158830	103159400	-0.41	-0.03
Psma5	chr3	108059873	108060022	-0.64	3.39
Ints12	chr3	132771889	132772517	-1.00	-0.12

Ube2d3	chr3	135101656	135102228	-19.60	-0.75
Gtf2b	chr3	142428210	142434402	-2.43	-0.52
Gtf2b	chr3	142444579	142446421	-0.99	0.12
Phc3	chr3	30805887	30813151	-0.72	-0.12
Phc3	chr3	30864733	30868337	-1.81	-0.73
Ccna2	chr3	36470827	36470918	-0.54	-0.57
Elf2	chr3	51059675	51060839	-1.69	0.13
Elf2	chr3	51061545	51064994	-11.20	0.72
Maml3	chr3	51491863	51493302	-3.62	0.65
Maml3	chr3	51493600	51496200	-1.82	0.41
Maml3	chr3	51501693	51600338	-0.20	0.07
Maml3	chr3	51659510	51660829	-9.25	0.71
Maml3	chr3	51907999	51908179	-1.49	0.85
Maml3	chr3	51908418	51908808	-2.19	-0.81
Lamtor2	chr3	88353913	88356849	-9.31	-0.34
Ints3	chr3	90195369	90196223	-2.53	-0.21
Ints3	chr3	90237258	90237498	-4.48	-0.71
Chtop	chr3	90303653	90306049	-0.50	-0.02
Chtop	chr3	90313185	90313365	-3.07	-0.25
Psmb4	chr3	94690701	94690880	-8.50	-0.41
Psmd4	chr3	94836626	94836894	-2.54	-0.16
Psmd4	chr3	94840526	94846467	-8.21	-0.35
Rprd2	chr3	95567509	95570116	-4.11	-0.13
Rprd2	chr3	95576763	95577094	-0.41	-0.07
Hist2h3c2	chr3	96042365	96042604	-1.05	0.63
Hipk1	chr3	103547180	103548289	-2.19	-0.08
Hipk1	chr3	103581236	103595486	-33.60	-0.28
Prmt6	chr3	110050616	110051517	-0.57	-0.23
Prmt6	chr3	110052356	110053916	-1.37	-0.11
Srsf11	chr3	157674772	157675808	-16.50	-0.32
Trp53inp1	chr4	11083587	11092710	-11.10	0.96
Trp53inp1	chr4	11095044	11097120	-9.82	1.10
Nbn	chr4	15906479	15911111	-1.10	0.17
Rngtt	chr4	33586031	33587733	-1.66	0.32
Rngtt	chr4	33588270	33588360	-0.29	0.12
Nr4a3	chr4	48058221	48058400	-1.45	-1.08
Nr4a3	chr4	48064136	48065027	-8.33	-1.10
Nr4a3	chr4	48080626	48096224	-0.76	0.49
Zfp189	chr4	49535279	49543489	-4.61	0.49
Rraga	chr4	86221606	86222293	-1.18	-0.09
Tal1	chr4	114737293	114741792	-0.36	0.03
Tal1	chr4	114741970	114742091	-0.38	-0.13
Med8	chr4	118087440	118087589	-1.21	0.21

Rragc	chr4	123598193	123598284	-0.30	-0.01
Rragc	chr4	123612802	123613851	-0.52	0.02
Lsm10	chr4	125773896	125775828	-0.39	0.02
Phc2	chr4	128404910	128405061	-0.82	-0.46
Phc2	chr4	128424470	128429198	-18.30	-0.40
Phc2	chr4	128429466	128429976	-16.20	-0.47
Srsf4	chr4	131453751	131457610	-9.11	-0.28
Rpa2	chr4	132333826	132334035	-2.59	-0.74
Nr0b2	chr4	133112127	133112392	-0.35	0.24
Zfp46	chr4	135846237	135848601	-1.27	0.14
Mtor	chr4	147822690	147822781	-3.13	-1.30
Mtor	chr4	147930392	147931764	-3.53	-0.34
Ssu72	chr4	155079133	155079193	-1.21	1.30
Ssu72	chr4	155079432	155089762	-0.99	-0.09
Mllt3	chr4	87419218	87419819	-1.24	-0.35
Mllt3	chr4	87486765	87487246	-0.67	0.14
Zfp691	chr4	118842631	118845741	-0.75	0.13
Zfp691	chr4	118845980	118846800	-9.76	2.33
Zfp69	chr4	120602801	120603819	-1.72	0.55
Tfap2e	chr4	126393574	126393783	-1.59	2.55
Sesn2	chr4	132048721	132052822	-3.34	-0.16
Sesn2	chr4	132066221	132066371	-3.98	-0.48
Sesn2	chr4	132066234	132066414	-3.65	-0.47
Sfn	chr4	133156530	133157635	-3.71	-1.00
Sfn	chr4	133157694	133158083	-0.48	0.30
Arid1a	chr4	133235940	133241652	-2.63	-0.08
Arid1a	chr4	133276907	133312385	-11.90	-0.45
Srrm1	chr4	134878175	134878385	-1.65	-0.18
Srrm1	chr4	134880008	134881048	-18.30	-0.47
Srrm1	chr4	134902739	134909069	-0.66	-0.08
Eloa	chr4	135561281	135561551	-1.02	-0.17
Eloa	chr4	135565701	135577564	-4.34	-0.14
Ski	chr4	154529795	154533593	-10.40	0.28
Ski	chr4	154533945	154596644	-10.70	-0.37
Cdk6	chr5	3519406	3520884	-1.33	-0.15
Kmt2e	chr5	22940246	22940516	-2.58	0.46
Kmt2e	chr5	22998122	22999338	-0.91	0.13
Kmt2e	chr5	23002790	23005303	-1.02	0.08
Kmt2e	chr5	23007352	23008999	-1.84	0.10
Ywhah	chr5	33361494	33361614	-1.23	0.51
Ywhah	chr5	33369299	33370077	-1.30	-0.06
Rbpj	chr5	53981538	53981866	-29.80	-1.15
Rbpj	chr5	54044845	54045026	-0.27	-0.52

Rpap2	chr5	108048973	108049513	-1.52	0.29
Zfp605	chr5	110539457	110539517	-0.19	1.00
Zfp605	chr5	110556601	110558156	-4.58	0.65
Triap1	chr5	115791255	115793441	-0.39	-0.02
Rnf34	chr5	123273869	123314504	-0.81	0.09
Psmd9	chr5	123678228	123678437	-0.34	0.03
Kmt5a	chr5	124909965	124910818	-10.10	-0.42
Kmt5a	chr5	124911146	124911447	-1.43	0.14
Gtf2h3	chr5	125046192	125046313	-0.64	-0.25
Zfp664	chr5	125343755	125366362	-1.00	-0.09
Zfp664	chr5	125366751	125367081	-0.42	-0.06
Rfc2	chr5	135073840	135074198	-2.30	-0.39
Zkscan1	chr5	138526311	138534016	-1.05	-0.35
Zkscan1	chr5	138534105	138534645	-16.30	-1.03
Zkscan1	chr5	138541914	138542485	-0.49	-0.05
Zkscan1	chr5	138542723	138543984	-3.46	0.27
Zkscan1	chr5	138544222	138545962	-1.19	-0.12
Zfp12	chr5	144005811	144008390	-3.66	0.31
Zfp12	chr5	144008839	144009619	-0.78	-0.19
Cpsf4	chr5	145939775	145942700	-0.65	0.07
Zkscan5	chr5	145979715	145981274	-1.73	0.54
Zkscan5	chr5	145981632	145981812	-0.41	-0.19
Zkscan5	chr5	145981871	145982380	-1.47	0.57
Zfp655	chr5	145992583	145994463	-2.98	-0.51
Zfp655	chr5	146004460	146006138	-2.46	0.12
Zscan25	chr5	146044360	146052189	-9.62	-0.48
Cdk8	chr5	147042885	147071089	-9.50	-1.16
Smarcd3	chr5	24100691	24100989	-0.59	-0.63
Prkag2	chr5	24369540	24369661	-0.30	-0.04
Prkag2	chr5	24527783	24527844	-5.60	1.58
Prkag2	chr5	24534296	24606370	-0.56	0.08
Kmt2c	chr5	24790138	24790949	-2.35	-0.28
Kmt2c	chr5	24804711	24805371	-6.01	0.48
Kmt2c	chr5	24805490	24806151	-3.45	0.35
Kmt2c	chr5	24815764	24816754	-4.22	0.34
Kmt2c	chr5	24819800	24820700	-2.61	0.23
Kmt2c	chr5	24820849	24821690	-2.45	0.22
Kmt2c	chr5	24878616	24880356	-8.08	0.57
Slbp	chr5	33992455	33994955	-3.22	0.32
Prkab1	chr5	116463747	116467999	-0.85	-0.08
Prkab1	chr5	116474139	116474407	-0.44	-0.06
Ptpn11	chr5	121581827	121582307	-1.21	0.16
Ptpn11	chr5	121583292	121587519	-0.40	0.02

Ncor2	chr5	125497524	125500255	-17.90	-0.42
Ncor2	chr5	125504014	125509323	-4.66	-0.22
Ncor2	chr5	125517648	125517739	-5.60	-1.05
Ncor2	chr5	125518279	125531358	-10.10	-0.27
Ncor2	chr5	125538304	125548306	-0.58	0.07
Ncor2	chr5	125659493	125659584	-0.45	-0.84
Ubc	chr5	125866453	125867671	-172.00	0.73
Ubc	chr5	125867938	125868147	-3.15	0.20
Ubc	chr5	125868235	125870298	-22.80	-0.40
Zfp11	chr5	130162143	130162980	-1.61	0.64
Ywhag	chr5	136384965	136385384	-2.91	-0.09
Ywhag	chr5	136386428	136406621	-96.80	-0.35
Ywhag	chr5	136410241	136410481	-13.00	-0.40
Zfp113	chr5	138585481	138586141	-3.89	-0.70
Zfp113	chr5	138586529	138586680	-0.41	0.19
Taf6	chr5	138619844	138620232	-4.14	-0.57
Smurf1	chr5	145637663	145638682	-0.50	0.08
Smurf1	chr5	145639310	145639521	-0.66	0.35
Zkscan14	chr5	145955991	145962485	-2.97	-0.45
Rfc3	chr5	152445398	152445638	-0.43	0.10
Cul1	chr6	47404345	47445165	-3.56	0.31
Cul1	chr6	47474617	47476108	-1.64	-0.15
Zfp398	chr6	47785899	47790681	-0.56	-0.29
Zfp398	chr6	47815976	47817057	-5.16	-0.83
Zfp282	chr6	47827763	47830376	-1.26	-0.29
Zfp282	chr6	47854827	47855966	-11.40	-0.77
Zfp282	chr6	47856175	47856835	-0.42	-0.06
Zfp212	chr6	47870625	47876740	-0.95	-0.37
Zfp212	chr6	47879063	47881476	-0.63	-0.12
Zfp775	chr6	48555624	48569787	-0.41	-0.16
Zfp775	chr6	48569935	48570026	-0.24	0.38
Zfp775	chr6	48570414	48570923	-1.15	-0.76
Gata2	chr6	88154654	88155829	-0.38	-0.05
Nr2c2	chr6	92041411	92042461	-2.10	-0.74
Nr2c2	chr6	92114256	92117961	-0.62	0.09
Brpf1	chr6	113257130	113267099	-6.11	-0.18
Brpf1	chr6	113267573	113269353	-20.40	-0.58
Brpf1	chr6	113272417	113274519	-13.10	-0.60
Chd4	chr6	125046180	125050907	-20.10	-0.46
Chd4	chr6	125079888	125080399	-1.95	-0.09
Cdkn1b	chr6	134870958	134872360	-3.01	0.32
Gm8994	chr6	136279021	136279141	-0.30	-0.81
Gm8994	chr6	136279763	136279853	-0.25	0.86

H2afj	chr6	136757307	136757517	-2.15	-0.48
Cnot4	chr6	34973322	34992713	-1.58	0.32
Cnot4	chr6	34996096	35002891	-0.80	0.08
Hipk2	chr6	38647898	38665995	-46.30	-0.52
Hipk2	chr6	38767930	38787077	-4.22	-0.11
Hipk2	chr6	38825800	38826040	-0.40	-0.04
Zfp786	chr6	47769651	47770337	-0.58	-0.74
Zfp786	chr6	47770782	47771140	-0.50	-0.90
Zfp786	chr6	47771318	47771676	-0.29	0.81
Zfp777	chr6	47974246	47998850	-8.38	-0.33
Zfp746	chr6	48013500	48016503	-1.30	-0.16
Zfp746	chr6	48033523	48036532	-0.67	0.27
Gadd45a	chr6	66985208	66987341	-8.35	-0.44
Rybp	chr6	100182064	100183112	-0.57	0.17
Zfp248	chr6	118378705	118382923	-2.80	0.51
Phc1	chr6	122273438	122273618	-0.97	0.44
Phc1	chr6	122275560	122275830	-0.61	-0.25
Tigar	chr6	127037498	127038307	-0.89	-0.11
Ccnd2	chr6	127079556	127080275	-3.38	0.32
Ccnd2	chr6	127100856	127101036	-0.93	0.38
Rhno1	chr6	128307315	128309210	-1.30	-0.13
Kras	chr6	145168813	145168964	-1.16	-0.35
Ints13	chr6	146502576	146503064	-2.74	0.36
U2af2	chr7	5030883	5031212	-1.10	-0.10
Zfp667	chr7	6256343	6256524	-1.03	1.18
Zfp667	chr7	6256792	6256942	-0.55	0.66
Zfp667	chr7	6257689	6257840	-1.67	2.29
Zfp78	chr7	6331550	6332268	-2.40	1.42
Zfp28	chr7	6344996	6347498	-0.41	0.06
Zfp110	chr7	13434024	13434472	-0.91	0.17
Zfp324	chr7	13555672	13556361	-3.85	-1.08
Zfp324	chr7	13556928	13557646	-0.89	-0.35
Zfp446	chr7	13564366	13564486	-0.27	-0.05
Zfp446	chr7	13567286	13568276	-1.47	-0.29
Zfp446	chr7	13568455	13569056	-0.40	-0.07
Ppp1r13l	chr7	19956897	19960606	-7.13	-0.66
Ercc2	chr7	19979470	19981041	-4.45	-0.33
Zfp180	chr7	24882912	24891546	-4.68	0.24
Zfp112	chr7	24910093	24910364	-0.73	-1.09
Zfp112	chr7	24910573	24910664	-0.48	-1.72
Zfp112	chr7	24911022	24911533	-0.31	-0.12
Zfp235	chr7	24925651	24925772	-1.05	0.94
Zfp235	chr7	24926760	24927181	-1.25	-0.51

Zfp235	chr7	24927869	24928170	-0.36	-0.17
Tgfb1	chr7	26472020	26477430	-36.70	-0.26
Akt2	chr7	28422173	28423398	-0.50	-0.06
Paf1	chr7	29178014	29178133	-0.41	-0.07
Paf1	chr7	29181953	29184402	-0.48	0.02
Zfp30	chr7	30577820	30578780	-4.53	1.80
Zfp790	chr7	30613435	30616012	-3.35	0.25
Zfp420	chr7	30645086	30661309	-4.28	0.31
Zfp382	chr7	30916575	30919814	-0.57	-0.05
Polr2i	chr7	31017332	31017772	-0.55	0.13
Zfp658	chr7	50828048	50828348	-0.67	0.44
Zfp658	chr7	50828646	50829515	-0.33	-0.03
Zfp658	chr7	50829663	50830323	-0.90	-0.28
Zfp719	chr7	50839645	50846689	-6.02	0.33
Zfp819	chr7	50873231	50873322	-0.50	3.17
Gtf2h1	chr7	54051493	54051644	-1.33	-0.22
Gtf2h1	chr7	54078270	54078601	-5.40	0.33
Zfp710	chr7	87169699	87227267	-11.50	-0.28
Zfp710	chr7	87231444	87231625	-0.36	0.04
Zfp710	chr7	87233516	87236204	-0.61	0.04
Lamtor1	chr7	109054380	109058241	-0.87	-0.19
Lamtor1	chr7	109059942	109060091	-16.60	-0.68
Zfp143	chr7	117236697	117238428	-1.89	0.27
Ctr9	chr7	118173736	118177414	-3.03	-0.38
Ctr9	chr7	118198901	118199621	-1.34	-0.16
Zfp771	chr7	134388039	134398315	-3.36	-0.36
Setd1a	chr7	134920902	134921023	-1.37	0.88
Setd1a	chr7	134927635	134930735	-0.69	-0.03
Setd1a	chr7	134931750	134932028	-1.01	-0.16
Setd1a	chr7	134934941	134940915	-7.31	-0.23
Zik1	chr7	11075804	11076075	-2.76	1.74
Zfp551	chr7	13000735	13000886	-0.20	-0.08
Zfp551	chr7	13001514	13002233	-2.46	1.45
Zfp94	chr7	25087140	25088844	-2.13	-0.44
Pou2f2	chr7	25876133	25878098	-1.03	0.11
Psmd8	chr7	29965393	29965573	-13.80	-0.43
Zfp940	chr7	30629198	30629439	-0.25	-0.03
Zfp940	chr7	30629588	30629649	-0.64	1.82
Zfp940	chr7	30630307	30630548	-0.95	1.24
Zfp940	chr7	30630817	30630998	-0.73	0.68
Zfp27	chr7	30678594	30680419	-0.52	-0.05
Zfp27	chr7	30680747	30683850	-0.91	0.18
Zfp14	chr7	30823256	30823436	-0.57	1.58

Zfp14	chr7	30823733	30823854	-0.28	0.22
Zfp566	chr7	30862385	30862860	-0.44	0.13
Zfp566	chr7	30862978	30863483	-3.46	0.75
Kmt2b	chr7	31359377	31361794	-20.90	-0.55
Kmt2b	chr7	31368445	31373745	-14.90	-0.34
Gpi1	chr7	34987182	34987689	-15.30	-0.25
Ccne1	chr7	38883150	38883358	-1.08	-0.54
Zfp715	chr7	50552997	50556994	-8.01	0.30
Zfp473	chr7	51987480	51987751	-0.36	-0.29
Zfp473	chr7	51987810	51988051	-0.70	-0.61
Zfp473	chr7	51988379	51988830	-1.38	1.24
Zfp473	chr7	51988979	51989760	-8.39	2.40
Med25	chr7	52132313	52133597	-6.95	-0.47
Bax	chr7	52721428	52721703	-3.69	-0.20
Bax	chr7	52721999	52722238	-1.35	0.28
E2f8	chr7	56123087	56123387	-0.98	-0.44
E2f8	chr7	56126142	56126233	-0.71	-0.66
E2f8	chr7	56126292	56127247	-0.85	-0.32
Blm	chr7	87647292	87647681	-3.04	-0.71
Blm	chr7	87654437	87658369	-0.32	-0.01
Eed	chr7	97125556	97129068	-6.88	-0.52
Eed	chr7	97129276	97129396	-0.46	-0.10
Pcf11	chr7	99793118	99795438	-8.06	0.60
Pcf11	chr7	99801721	99808380	-11.00	-0.25
Pcf11	chr7	99809567	99810434	-2.11	-0.10
Pcf11	chr7	99818003	99818422	-5.53	-0.40
Zfp747	chr7	134516438	134516707	-0.85	-0.40
Zfp747	chr7	134516765	134519504	-0.42	0.03
9130019O22Rik	chr7	134526131	134526281	-0.56	1.23
9130019O22Rik	chr7	134526847	134529144	-0.95	-0.14
E430018J23Rik	chr7	134534467	134534528	-0.84	-2.04
E430018J23Rik	chr7	134534616	134536839	-0.37	-0.06
Zfp764	chr7	134547897	134549152	-1.39	-0.24
Zfp764	chr7	134550126	134550336	-0.39	-0.19
Zfp688	chr7	134562479	134563593	-1.17	-0.28
Zfp688	chr7	134565487	134565548	-0.77	-1.45
Zfp689	chr7	134587294	134588642	-1.79	-0.40
Zfp689	chr7	134592252	134592433	-0.39	0.12
Zfp668	chr7	135009351	135009442	-0.32	-0.14
Zfp668	chr7	135009620	135020337	-10.40	-0.40
Pidd1	chr7	148624413	148624744	-3.35	-0.92
Pidd1	chr7	148628991	148629907	-3.04	-1.22
Ccnd1	chr7	152118471	152118592	-5.59	-0.32

Ccnd1	chr7	152125559	152125830	-6.58	-0.44
Snapc2	chr8	4255311	4256190	-6.55	-0.73
Tfdp1	chr8	13377166	13377375	-0.39	0.04
Tfdp1	chr8	13377582	13377702	-0.58	-0.09
Kat6a	chr8	23970010	24013683	-5.12	0.22
Kat6a	chr8	24040759	24043005	-0.60	-0.06
Kat6a	chr8	24046218	24051490	-0.77	-0.03
Zfp703	chr8	28087837	28090862	-45.70	-0.65
Ppp2cb	chr8	34710092	34710359	-0.73	-0.37
Gtf2e2	chr8	34886513	34887055	-0.32	-0.01
Nr2c2ap	chr8	72655231	72655498	-1.93	0.47
Nr2c2ap	chr8	72657115	72657440	-1.00	-0.16
Ell	chr8	73063573	73063724	-2.49	-0.72
Ell	chr8	73109061	73115709	-19.10	-0.74
Sin3b	chr8	75247186	75248002	-1.72	-0.30
Sin3b	chr8	75273755	75273906	-1.47	-0.52
Sin3b	chr8	75280901	75281383	-3.95	-0.67
Nr3c2	chr8	79432403	79432554	-0.61	1.74
Nr3c2	chr8	79433567	79433776	-0.74	-1.52
Cbfb	chr8	107694632	107694812	-1.65	-0.29
Zfp90	chr8	108947910	108948329	-2.59	0.80
Zfp90	chr8	108948657	108949616	-1.45	0.33
Zfp1	chr8	114190002	114193957	-0.62	0.14
Zfp1	chr8	114194046	114194762	-2.59	-0.49
Banp	chr8	124550400	124550731	-0.86	-0.47
Banp	chr8	124551389	124551899	-1.05	-0.63
Banp	chr8	124552078	124552768	-1.73	-0.70
Zfpm1	chr8	124806190	124856010	-4.04	0.63
Zfpm1	chr8	124859027	124861147	-0.69	-0.07
Ash2l	chr8	26950536	26958082	-1.11	-0.21
Wrn	chr8	34346314	34346884	-1.70	0.55
Wrn	chr8	34434722	34439590	-0.78	0.26
Cnot7	chr8	41592761	41593001	-0.56	0.08
Ing2	chr8	48753850	48758943	-1.32	0.15
Ing2	chr8	48760272	48760513	-0.64	-0.35
Gatad2a	chr8	72433183	72435724	-28.20	-0.49
Gatad2a	chr8	72459749	72459840	-2.26	-0.26
Gatad2a	chr8	72498029	72498150	-0.37	-0.11
Gatad2a	chr8	72519918	72520248	-1.28	-0.35
Nr2f6	chr8	73898225	73905851	-12.70	-0.57
Med26	chr8	75019202	75072090	-10.00	-0.44
Csnk2a2	chr8	97976954	97977225	-0.35	-0.03
Psmb10	chr8	108461631	108461967	-2.80	-0.63

Psmd7	chr8	110104338	110108077	-4.03	0.12
Dhx38	chr8	112071923	112073753	-0.53	0.05
Dhx38	chr8	112080084	112080835	-6.56	0.50
Dhx38	chr8	112084553	112089501	-0.31	0.00
Maml2	chr9	13424582	13425122	-2.71	0.92
Maml2	chr9	13510293	13511342	-0.77	0.17
Zfp317	chr9	19451183	19452859	-22.30	0.60
Pin1	chr9	20456573	20459918	-1.39	-0.27
Smarca4	chr9	21505589	21507505	-3.30	0.23
Zfp202	chr9	40014989	40015898	-2.39	-0.75
Zfp202	chr9	40018534	40019996	-1.21	0.18
Usp2	chr9	43883786	43884026	-0.81	2.12
Usp2	chr9	43902032	43902302	-0.96	-0.35
H2afx	chr9	44143446	44144156	-0.77	-0.05
Ppp2r1b	chr9	50687990	50688710	-0.71	-0.09
Ppp2r1b	chr9	50701016	50701675	-4.03	-0.60
Sin3a	chr9	56958532	56958892	-0.32	-0.02
Sin3a	chr9	56973115	56975576	-0.84	0.11
Snapc5	chr9	64027103	64027192	-0.58	-0.24
Snapc5	chr9	64030056	64030232	-0.39	-0.07
Ints14	chr9	64809670	64809731	-0.94	0.46
Ints14	chr9	64833706	64834453	-0.78	0.08
Rora	chr9	69226715	69226835	-0.32	0.16
Ice2	chr9	69263470	69263950	-0.82	0.16
Ice2	chr9	69280013	69280223	-0.86	0.36
Leo1	chr9	75289330	75294926	-0.87	-0.07
Atr	chr9	95765579	95765669	-0.85	-0.40
Tfdp2	chr9	96218548	96218699	-1.00	0.56
Ctnnb1	chr9	120842577	120859669	-0.34	-0.01
Ctnnb1	chr9	120867712	120868847	-5.68	0.15
Zkscan7	chr9	122797636	122797876	-0.42	-0.24
Zkscan7	chr9	122803837	122804973	-0.30	0.02
Med17	chr9	15066594	15066715	-0.33	0.04
Zfp558	chr9	18260656	18261436	-1.57	0.79
Zfp426	chr9	20274940	20276201	-1.87	0.28
Zfp266	chr9	20304179	20306501	-0.78	-0.10
Kmt2a	chr9	44612636	44614167	-3.81	0.25
Kmt2a	chr9	44615605	44617182	-1.06	0.16
Kmt2a	chr9	44625809	44629185	-31.10	0.48
Kmt2a	chr9	44629634	44630414	-7.20	0.44
Kmt2a	chr9	44655111	44656174	-7.56	0.48
Kmt2a	chr9	44656473	44656924	-1.40	0.24
Kmt2a	chr9	44657403	44658123	-21.00	0.79

Atm	chr9	53247503	53248264	-1.55	0.23
Pml	chr9	58066214	58066305	-1.33	0.78
Pml	chr9	58067472	58068881	-10.10	-0.47
Pml	chr9	58078383	58097563	-4.44	-0.27
Smad3	chr9	63496369	63496969	-0.55	0.19
Smad3	chr9	63497327	63498046	-0.81	0.32
Smad3	chr9	63605729	63605864	-0.51	-1.42
Rnf111	chr9	70274555	70277390	-0.83	-0.10
Rnf111	chr9	70323650	70350792	-12.70	0.37
Tcf12	chr9	71693887	71694367	-8.81	0.54
Tcf12	chr9	71958621	71958922	-1.18	0.36
Tcf12	chr9	71959385	71959626	-0.68	-0.16
Atrip	chr9	108960445	108961754	-16.60	-0.43
Atrip	chr9	108967670	108968299	-1.36	-0.26
Cnot10	chr9	114541870	114549228	-1.75	-0.36
Zfp445	chr9	122761016	122763080	-42.10	0.58
Zfp445	chr9	122770871	122775124	-1.55	0.17
Cited2	chr10	17443033	17444853	-33.40	-0.20
Sgk1	chr10	21718544	21718843	-0.78	0.37
Sgk1	chr10	21719081	21719290	-4.21	1.23
Sesn1	chr10	41530379	41607293	-0.28	0.00
Sesn1	chr10	41627347	41627735	-0.40	-0.14
Stk11	chr10	79579091	79588239	-0.80	0.07
Stk11	chr10	79592650	79593215	-1.40	-0.20
Txnrd1	chr10	82296755	82323149	-0.39	-0.07
Txnrd1	chr10	82323297	82335784	-5.40	-0.55
E2f7	chr10	110216189	110216249	-0.35	-0.75
Smarcc2	chr10	127920261	127920695	-0.29	0.00
Smarcc2	chr10	127924543	127924604	-1.40	-0.59
Smarcc2	chr10	127926123	127926244	-0.38	0.05
Ddit4	chr10	59412924	59414518	-67.50	-0.91
Nrbf2	chr10	66729823	66730867	-2.28	-0.23
Ube2d1	chr10	70718144	70718592	-2.63	0.38
Smarcb1	chr10	75359602	75359780	-1.07	-0.22
Polr2e	chr10	79501316	79502404	-2.85	-0.43
Mbd3	chr10	79855285	79856691	-7.71	-0.28
Tcf3	chr10	79872416	79873314	-1.26	-0.16
Cradd	chr10	94637889	94638400	-0.78	0.33
Cradd	chr10	94785366	94785547	-0.28	0.04
Cnot2	chr10	115922246	115922606	-0.99	-0.19
Cnot2	chr10	115922874	115923144	-4.87	0.64
Yeats4	chr10	116652465	116652884	-0.70	0.11
Mdm2	chr10	117126170	117126441	-1.00	0.08

Mdm2	chr10	117126799	117129718	-14.10	0.17
Dyrk2	chr10	118296404	118305900	-0.44	-0.03
Pip4k2c	chr10	126635286	126635944	-0.36	-0.01
Pip4k2c	chr10	126636730	126637327	-0.37	0.03
Cdk2	chr10	128135921	128136041	-0.48	-0.19
Psme4	chr11	30671774	30672135	-0.54	-0.18
Slu7	chr11	43247232	43251659	-1.97	0.22
Slu7	chr11	43258751	43259812	-3.05	-0.23
Slu7	chr11	43260795	43260976	-0.25	0.11
Med7	chr11	46250448	46253332	-0.31	0.02
Med7	chr11	46254128	46254877	-4.70	0.38
Ppp2ca	chr11	51912325	51912474	-4.51	-0.88
Ppp2ca	chr11	51935449	51935836	-0.34	0.00
Aff4	chr11	53164268	53186578	-4.92	-0.19
Aff4	chr11	53211860	53216039	-0.32	0.00
Aff4	chr11	53225470	53229192	-24.70	0.81
Cnot8	chr11	57930958	57931258	-0.74	0.18
Zkscan6	chr11	65636352	65642442	-3.43	-0.39
Aurkb	chr11	68861818	68863941	-3.92	-0.59
Trp53	chr11	69393860	69394608	-0.43	-0.06
Ywhae	chr11	75546388	75546567	-5.18	-0.26
Suz12	chr11	79843530	79845978	-0.38	0.01
Cdk5r1	chr11	80291441	80292571	-1.52	0.38
Cdk5r1	chr11	80293343	80293493	-0.43	0.26
Cdk5r1	chr11	80291445	80292555	-1.61	0.40
Cdk5r1	chr11	80293392	80293483	-0.29	0.10
Taf15	chr11	83286609	83286670	-0.74	-0.19
Ppm1d	chr11	85124755	85125383	-7.40	-0.93
Ppm1d	chr11	85140378	85140529	-1.24	0.48
Ppm1d	chr11	85153180	85159827	-0.39	-0.03
Supt4a	chr11	87557059	87557119	-0.31	-0.09
Srsf1	chr11	87861172	87861322	-1.85	-0.33
Cdk12	chr11	98064618	98065548	-5.37	-0.20
Cdk12	chr11	98071707	98072546	-3.00	-0.14
Cdk12	chr11	98104793	98107067	-2.29	0.20
Cdk12	chr11	98111003	98111783	-7.97	-0.40
Psmd3	chr11	98543867	98546829	-30.30	-0.74
Thra	chr11	98603186	98614709	-0.74	0.42
Thra	chr11	98614768	98617477	-2.58	-0.78
Thra	chr11	98625600	98626259	-4.68	-0.69
Thra	chr11	98629482	98629782	-10.30	1.38
Casc3	chr11	98683710	98689970	-6.76	-0.34
Casc3	chr11	98692721	98694074	-0.45	0.04

Rara	chr11	98799009	98808908	-1.61	-0.58
Rara	chr11	98808997	98822329	-5.35	-0.55
Rara	chr11	98827782	98829451	-0.74	-0.25
Rara	chr11	98831600	98835508	-19.40	-0.66
Psme3	chr11	101177564	101178336	-0.62	0.07
Psme3	chr11	101182070	101183288	-0.64	-0.06
Cbx2	chr11	118889293	118890939	-23.30	-1.92
Rptor	chr11	119465086	119517298	-1.88	-0.26
Rptor	chr11	119759737	119760755	-0.53	0.05
Hus1	chr11	8896711	8896861	-0.56	0.17
Lsm11	chr11	45747027	45747446	-3.16	-0.71
Maml1	chr11	50069912	50070420	-0.98	0.21
Maml1	chr11	50071136	50074538	-0.86	-0.10
Maml1	chr11	50076892	50105838	-2.71	-0.20
Rad50	chr11	53463650	53465769	-2.93	-0.38
Zkscan17	chr11	59299022	59299382	-2.54	0.44
Zkscan17	chr11	59300338	59306167	-1.66	-0.14
Zkscan17	chr11	59319782	59319933	-1.97	-1.06
Top3a	chr11	60553560	60555499	-1.34	0.26
Top3a	chr11	60555857	60557535	-1.26	0.23
Top3a	chr11	60590477	60590867	-9.76	1.46
Ncor1	chr11	62130880	62131511	-1.35	0.12
Ncor1	chr11	62147246	62147337	-0.41	-0.06
Ncor1	chr11	62182924	62186997	-26.60	-0.65
Ncor1	chr11	62196845	62198278	-2.94	-0.40
Ncor1	chr11	62252002	62270948	-5.64	-1.11
Zfp287	chr11	62527053	62527804	-2.03	0.58
Zfp287	chr11	62527953	62528373	-1.76	0.62
Zfp287	chr11	62528582	62528703	-0.25	-0.02
Zfp287	chr11	62541753	62542444	-0.32	0.04
Zfp287	chr11	62545196	62545317	-1.08	1.22
Chd3	chr11	69157463	69157973	-5.22	-0.48
Chd3	chr11	69174956	69175626	-0.72	-0.14
Chd3	chr11	69177569	69182868	-4.44	-0.54
Rpa1	chr11	75114477	75116219	-1.52	-0.18
Rpa1	chr11	75154547	75161885	-1.59	0.39
Supt6	chr11	78020490	78021581	-6.20	-0.26
Supt6	chr11	78044521	78044672	-9.16	-0.50
Supt6	chr11	78047130	78059175	-4.29	-0.45
Rffl	chr11	82619321	82632040	-24.80	-0.71
Brip1	chr11	85874715	85878344	-2.90	0.50
Med13	chr11	86084344	86084585	-0.84	0.15
Med13	chr11	86100121	86100212	-2.56	-0.43

Med13	chr11	86104608	86114217	-1.33	0.09
Med13	chr11	86132926	86141980	-0.71	0.05
Pcgf2	chr11	97551180	97551450	-9.46	-1.52
Pcgf2	chr11	97553219	97553423	-0.65	0.22
Pip4k2b	chr11	97578204	97584269	-3.42	-0.14
Med1	chr11	98014158	98015024	-2.02	0.27
Med1	chr11	98015173	98015684	-0.74	-0.09
Med1	chr11	98016102	98017333	-0.59	0.04
Med1	chr11	98017662	98017873	-2.97	-0.38
Med1	chr11	98017962	98018233	-0.58	0.07
Med1	chr11	98018352	98019732	-1.59	0.09
Med1	chr11	98050576	98054607	-1.81	-0.28
Med24	chr11	98590218	98590689	-0.90	0.32
Nr1d1	chr11	98629338	98631545	-20.50	1.35
Nr1d1	chr11	98631693	98632599	-4.38	0.49
Nr1d1	chr11	98633166	98636556	-0.67	0.09
Kat2a	chr11	100566059	100566924	-11.10	-0.42
Kat2a	chr11	100573600	100573691	-0.37	-0.10
Brca1	chr11	101384784	101385234	-1.07	-0.42
Brca1	chr11	101385503	101386581	-0.66	0.14
Brca1	chr11	101386909	101387659	-0.94	0.23
Smarcd2	chr11	106133897	106134286	-1.29	-0.39
Smurf2	chr11	106683743	106684388	-0.37	-0.04
Smurf2	chr11	106781609	106781789	-3.23	-1.30
Srsf2	chr11	116714108	116714408	-50.00	-0.61
Cbx8	chr11	118899838	118901471	-0.31	0.01
Cbx4	chr11	118941130	118941400	-2.21	0.97
Cbx4	chr11	118941729	118947491	-3.35	-0.13
E2f6	chr12	16817830	16817920	-1.61	-0.89
E2f6	chr12	16832231	16832859	-2.23	-0.27
Ppm1a	chr12	73862227	73885470	-1.34	-0.08
Ppm1a	chr12	73894914	73895183	-2.86	0.44
Snapc1	chr12	75065516	75068917	-5.03	-0.85
Srsf5	chr12	82046490	82046729	-16.60	-0.48
Srsf5	chr12	82050780	82051078	-44.00	-0.66
Psmc1	chr12	101348377	101351316	-1.50	-0.23
Cpsf2	chr12	103214183	103214244	-0.29	-0.03
Cpsf2	chr12	103240910	103241994	-0.60	-0.04
Papola	chr12	107022971	107023092	-4.18	-0.34
Papola	chr12	107072945	107075506	-2.32	-0.11
Ccnk	chr12	109418007	109424620	-0.44	0.06
Zfp839	chr12	112088488	112099099	-6.28	-0.75
Zfp839	chr12	112106449	112107344	-2.89	-0.47

Ywhaq	chr12	21422717	21423076	-5.19	-0.53
Ywhaq	chr12	21423381	21423442	-0.93	0.75
Max	chr12	78038822	78039718	-3.26	-0.11
Max	chr12	78063025	78063205	-5.46	-0.52
Med6	chr12	82674550	82674905	-2.82	0.39
Snw1	chr12	88791126	88791810	-8.21	-0.35
Gtf2a1	chr12	92797890	92806058	-4.85	0.27
Ppp1r13b	chr12	113066998	113068529	-0.52	-0.10
Ppp1r13b	chr12	113070821	113073439	-7.32	-0.51
Ppp1r13b	chr12	113146175	113146266	-0.46	1.29
Akt1	chr12	113912217	113912487	-1.97	-0.56
Zkscan4	chr13	21576264	21576865	-1.13	-1.76
Hist1h3h	chr13	21809496	21809698	-1.42	0.30
Hist1h3f	chr13	23636303	23636393	-2.07	-0.34
Hist1h2bg	chr13	23663268	23663474	-1.13	-0.09
Hist1h3d	chr13	23667608	23667786	-1.12	-0.25
Hist1h2bc	chr13	23776067	23776213	-2.98	0.39
Hist1h2ab	chr13	23842956	23843254	-0.48	0.06
Prelid1	chr13	55423415	55423591	-1.89	-0.18
Rmi1	chr13	58503698	58503988	-0.54	-0.08
Rmi1	chr13	58508365	58511252	-1.36	0.13
Zfp369	chr13	65397670	65398417	-5.12	-0.73
Med10	chr13	69954526	69954824	-5.28	-0.32
Ell2	chr13	75844961	75845111	-1.04	-0.52
Ell2	chr13	75899664	75901521	-116.00	-1.03
Taf9	chr13	101435858	101436098	-1.55	0.21
Plk2	chr13	111185251	111185700	-14.20	-0.89
Plk2	chr13	111190024	111191021	-3.14	-0.32
Slc38a9	chr13	113528209	113528690	-2.86	1.40
Cdk13	chr13	17809653	17811567	-1.53	0.10
Cdk13	chr13	17858358	17896931	-3.76	-0.17
Zkscan3	chr13	21479260	21480454	-4.71	0.32
Zkscan3	chr13	21488062	21494295	-3.85	-0.57
Zkscan8	chr13	21611941	21613722	-2.34	-0.34
Zkscan8	chr13	21617156	21618788	-0.43	0.10
Hist1h3i	chr13	21875180	21875266	-0.41	0.06
Hist1h3c	chr13	23837329	23837390	-0.31	-0.03
Tfap2a	chr13	40812539	40812719	-0.15	0.29
Dek	chr13	47201073	47201589	-1.54	0.34
Thoc3	chr13	54561210	54561569	-0.42	-0.04
Ctsl	chr13	64470393	64471614	-17.40	-0.32
Zfp729a	chr13	67719104	67720063	-10.30	0.99
Zfp729a	chr13	67720451	67721051	-2.35	0.38

Zfp729a	chr13	67721469	67722248	-0.63	0.15
Zfp738	chr13	67768467	67769574	-3.21	0.63
Zfp738	chr13	67770082	67770382	-0.89	0.38
Zfp738	chr13	67771278	67771698	-0.34	-0.07
Ice1	chr13	70741154	70747297	-1.12	0.05
Ice1	chr13	70747386	70748030	-1.07	-0.20
Nr2f1	chr13	78328745	78328956	-0.58	-2.91
Jmy	chr13	94204723	94212614	-10.00	0.49
Jmy	chr13	94242583	94269644	-4.59	-0.33
Rad17	chr13	101387447	101389058	-1.23	-0.21
Eaf1	chr14	32308264	32308534	-4.02	-0.47
Eaf1	chr14	32313335	32319971	-45.70	-0.71
Psmc6	chr14	45949498	45950514	-0.60	-0.09
Ints9	chr14	65658302	65658483	-0.69	-0.21
Med4	chr14	73909855	73910973	-1.63	-0.58
Med4	chr14	73917759	73918293	-3.57	-0.40
Elf1	chr14	79881419	79936023	-1.87	-0.46
Elf1	chr14	79973114	79980877	-2.36	0.14
Rarb	chr14	17407447	17451237	-0.51	-0.82
Nr1d2	chr14	19038938	19040485	-19.30	1.58
Nr1d2	chr14	19047334	19049180	-7.51	0.70
Pip4p1	chr14	51546074	51546434	-0.32	-0.01
Supt16	chr14	52780871	52782395	-6.16	-0.21
Supt16	chr14	52803669	52816914	-2.64	-0.39
Prmt5	chr14	55126018	55126553	-0.91	-0.11
Rabggta	chr14	56340520	56340807	-0.58	0.19
Cenpj	chr14	57145687	57148622	-0.29	-0.01
Cenpj	chr14	57170788	57171655	-0.31	-0.01
Cenpj	chr14	57171744	57172312	-0.62	0.14
Cenpj	chr14	57183992	57190683	-0.52	0.50
Ints6	chr14	63296661	63296842	-0.41	0.66
Ints6	chr14	63315432	63319557	-52.60	1.62
Gtf2f2	chr14	76297012	76306651	-1.19	-0.13
Prkaa1	chr15	5093860	5110647	-7.59	-0.91
Prkaa1	chr15	5126670	5126820	-1.22	-0.18
Prkaa1	chr15	5127194	5129214	-5.38	0.43
Rictor	chr15	6736456	6737266	-1.44	-0.13
Rictor	chr15	6745958	6747284	-0.93	0.10
Polr2k	chr15	36103800	36104751	-0.34	0.08
Zfp707	chr15	75804010	75804159	-0.65	-0.41
Zfp707	chr15	75804871	75806149	-11.00	-0.97
Rbx1	chr15	81305971	81306061	-1.28	-0.57
Ep300	chr15	81416643	81438767	-12.50	-0.29

Ep300	chr15	81479273	81481819	-5.01	-0.13
L3mbtl2	chr15	81516741	81518556	-3.63	-0.29
Arid2	chr15	96117952	96118043	-0.28	-0.02
Arid2	chr15	96199968	96201175	-7.90	0.36
Arid2	chr15	96201654	96209326	-6.30	0.26
Arid2	chr15	96223066	96232715	-1.11	-0.22
Nr4a1	chr15	101097276	101102664	-424.00	-1.45
Nr4a1	chr15	101103587	101105046	-137.00	-1.52
Zfp740	chr15	102034168	102035505	-2.24	-0.24
Zfp740	chr15	102039660	102043940	-1.65	-0.13
Sp1	chr15	102238241	102260424	-0.48	0.02
Sp1	chr15	102260572	102261651	-2.06	0.20
Skp2	chr15	9043329	9043510	-0.95	0.41
Skp2	chr15	9069207	9070137	-7.83	-1.04
Ywhaz	chr15	36723285	36723376	-0.55	0.11
Zfp706	chr15	36936977	36937157	-1.44	-0.45
Taf2	chr15	54847403	54848124	-0.49	-0.06
Taf2	chr15	54903436	54903647	-0.61	-0.15
Atad2	chr15	57928856	57930279	-4.77	0.52
Atad2	chr15	57966457	57966637	-0.74	-0.28
Cbx6	chr15	79658129	79664703	-7.13	-0.57
Poldip3	chr15	82958023	82959641	-1.64	-0.10
Brd1	chr15	88518753	88518814	-4.89	0.89
Brd1	chr15	88543169	88543809	-0.49	-0.05
Brd1	chr15	88547455	88564529	-12.40	0.36
Yaf2	chr15	93115848	93167246	-0.88	0.11
Vdr	chr15	97687063	97687362	-0.53	1.58
Ccnt1	chr15	98373641	98375016	-10.90	0.30
Ccnt1	chr15	98398086	98398654	-9.19	1.21
Prkag1	chr15	98643704	98643794	-0.45	-0.08
Rarg	chr15	102066030	102069302	-12.80	-0.78
Zfp385a	chr15	103144952	103145191	-1.58	-0.37
Zfp385a	chr15	103150806	103164738	-0.29	0.01
Zfp263	chr16	3744098	3745056	-3.71	-0.36
Zfp263	chr16	3748158	3750040	-1.24	0.09
Psmd2	chr16	20651724	20652324	-20.60	-0.49
Ncbp2	chr16	31957240	31957480	-1.22	-0.23
Fyttd1	chr16	32877869	32884216	-2.17	-0.38
Fyttd1	chr16	32902616	32905812	-4.94	-0.62
Zfp597	chr16	3865585	3871203	-2.18	0.33
Crebbp	chr16	4084047	4087773	-0.48	-0.02
Crebbp	chr16	4154961	4213404	-3.27	-0.16
Usp7	chr16	8738344	8757028	-0.78	-0.28

Gtf2e1	chr16	37510570	37511021	-1.83	-0.31
Gtf2e1	chr16	37511589	37515969	-0.39	0.04
Gtf2e1	chr16	37522998	37539825	-0.52	-0.07
Runx1	chr16	92605608	92606269	-0.70	0.06
Runx1	chr16	92695902	92697552	-25.50	0.56
Arid1b	chr17	4995073	4995882	-1.66	-0.67
Arid1b	chr17	4996061	5097577	-3.74	-0.55
Arid1b	chr17	5332607	5337057	-2.55	0.24
Arid1b	chr17	5337116	5339263	-0.52	0.10
Arid1b	chr17	5341518	5343345	-1.18	0.09
Zfp160	chr17	21162599	21164033	-0.57	0.06
Zfp160	chr17	21164271	21164332	-0.31	0.21
Zfp160	chr17	21178510	21179347	-0.31	0.05
Rnps1	chr17	24551619	24552074	-1.31	-0.33
Ppard	chr17	28369758	28432393	-6.75	-0.70
Ppard	chr17	28435930	28437576	-21.80	-0.79
Mapk14	chr17	28828316	28828704	-3.84	-0.61
Mapk14	chr17	28884007	28885350	-1.27	0.08
Brpf3	chr17	28938100	28938251	-0.36	0.50
Brpf3	chr17	28943116	28944224	-7.70	0.75
Brpf3	chr17	28954048	28960704	-0.96	0.16
Brpf3	chr17	28973668	28973878	-0.46	0.17
Cdkn1a	chr17	29228020	29235694	-168.00	-0.80
Daxx	chr17	34047504	34050602	-124.00	-0.92
Rxrb	chr17	34174187	34175165	-2.09	-0.18
Psmb8	chr17	34335288	34335468	-2.22	-0.33
Ehmt2	chr17	35042119	35042557	-5.05	-0.58
Ehmt2	chr17	35049712	35050992	-0.63	0.07
Ddx39b	chr17	35378720	35378917	-1.75	-0.31
Ddx39b	chr17	35386162	35386790	-0.58	-0.04
Ddx39b	chr17	35566041	35566729	-1.02	-0.30
Med20	chr17	47759792	47760513	-3.12	-0.33
Zfp213	chr17	23694178	23694744	-0.80	0.45
Zfp213	chr17	23694951	23696378	-0.41	-0.22
Thoc6	chr17	23810648	23810831	-0.28	0.01
Pdpk1	chr17	24215553	24216093	-11.80	0.63
Mlst8	chr17	24612351	24613045	-0.59	-0.10
Ube2i	chr17	25398055	25398776	-6.49	-0.64
Stub1	chr17	25967580	25967909	-3.51	-0.27
Stub1	chr17	25969947	25970008	-0.31	-0.35
Stub1	chr17	25970096	25970306	-1.82	-0.63
Taf11	chr17	28038342	28038672	-4.35	-0.52
Taf11	chr17	28042348	28045188	-1.94	-0.31

Notch3	chr17	32278193	32278314	-0.50	2.81
Ring1	chr17	34158095	34160251	-0.84	-0.10
Brd2	chr17	34249292	34249918	-19.30	0.29
Brd2	chr17	34251401	34252141	-0.53	0.02
Brd2	chr17	34254036	34257250	-434.00	-1.12
Runx2	chr17	44744958	44745199	-11.00	1.69
Runx2	chr17	44872639	44873298	-1.80	-0.39
Taf8	chr17	47625387	47626945	-7.66	-0.45
Taf8	chr17	47638265	47639236	-2.89	-0.65
Mllt1	chr17	57035733	57045221	-14.60	-0.53
Gtf2f1	chr17	57143372	57143559	-0.74	-0.13
Tgif1	chr17	71193544	71195840	-57.00	-0.42
Tgif1	chr17	71200227	71202842	-14.10	-0.47
Srsf7	chr17	80606178	80606645	-0.35	-0.03
Rbbp8	chr18	11880308	11881195	-1.41	0.17
Taf4b	chr18	14971627	14971957	-0.31	0.02
Polr2d	chr18	31955877	31956355	-0.34	-0.02
Wdr33	chr18	31963710	31986988	-10.60	-0.69
Wdr33	chr18	32047426	32048936	-16.50	-0.52
Wdr33	chr18	32049204	32067113	-1.24	-0.09
Iws1	chr18	32227387	32243000	-5.29	-0.18
Ercc3	chr18	32399984	32400372	-0.60	-0.12
Ercc3	chr18	32429502	32429682	-1.02	0.23
Phax	chr18	56732636	56746951	-2.10	-0.09
Prelid3a	chr18	67639251	67639460	-1.99	1.09
Rnmt	chr18	68460038	68465572	-0.29	0.00
Smad7	chr18	75527018	75527675	-0.56	0.13
Smad7	chr18	75528299	75554128	-4.14	0.55
Smad2	chr18	76462139	76464727	-1.34	-0.19
Epc1	chr18	6436931	6440144	-10.10	0.66
Epc1	chr18	6441163	6450203	-18.10	1.12
Epc1	chr18	6490689	6515876	-1.67	-0.51
Kctd1	chr18	15127880	15128090	-0.86	2.17
Kctd1	chr18	15128179	15132643	-0.22	0.12
Kctd1	chr18	15166356	15220489	-0.39	-0.45
Kctd1	chr18	15220578	15220848	-0.65	1.31
Kctd1	chr18	15221623	15221804	-0.65	-1.71
Taf7	chr18	37801809	37803149	-6.84	0.58
Nr3c1	chr18	39588392	39650238	-0.66	-0.04
Smad4	chr18	73799858	73800008	-0.35	-0.03
Smad4	chr18	73837391	73837750	-0.38	-0.03
Smad4	chr18	73863156	73863395	-1.93	-1.18
Ctdp1	chr18	80605326	80630972	-2.69	0.27

Ctdp1	chr18	80642798	80647936	-2.79	-0.16
Men1	chr19	6334978	6336781	-0.48	-0.04
Men1	chr19	6338873	6340864	-2.26	-0.13
Ints5	chr19	8967596	8971721	-3.22	-0.17
Ints5	chr19	8971870	8972350	-0.36	-0.03
Cpsf7	chr19	10614133	10620757	-1.51	0.08
Cpsf7	chr19	10621625	10621806	-12.40	0.88
Smarca2	chr19	26705324	26705445	-2.19	-1.03
Cstf2t	chr19	31157390	31159177	-2.75	0.13
Pcgf5	chr19	36484296	36486641	-0.57	0.12
Pcgf5	chr19	36519525	36530396	-3.63	-0.53
Taf5	chr19	47145518	47149502	-0.56	-0.15
Taf5	chr19	47156922	47157401	-0.43	-0.08
Rad9a	chr19	4195437	4196375	-0.34	-0.01
Rbm14	chr19	4743957	4744585	-0.74	-0.21
Rbm14	chr19	4803462	4811634	-0.83	-0.05
Kat5	chr19	5603043	5603836	-2.52	-0.22
Kat5	chr19	5609321	5609592	-0.57	-0.12
Kat5	chr19	5609854	5610034	-0.61	-0.31
Prdx5	chr19	6984015	6984135	-10.60	-0.65
Esrra	chr19	6996237	6996298	-0.43	-0.27
Polr2g	chr19	8872986	8873047	-0.44	-0.14
Ldb1	chr19	46107090	46108545	-0.46	-0.03
Pcgf6	chr19	47108856	47109007	-0.32	0.04
Pcgf6	chr19	47125005	47125335	-10.30	-1.25
Usp9x	chrX	12733152	12733872	-13.20	0.35
Med12	chrX	98488099	98488220	-1.26	0.32
Taf1	chrX	98794675	98794885	-1.77	-0.35
Rragb	chrX	149574678	149575911	-1.22	0.33
Upf3b	chrX	34632248	34632639	-0.43	-0.05
Thoc2	chrX	39150625	39150955	-4.71	0.47
G6pdx	chrX	71655299	71655479	-0.33	-0.01
Chm	chrX	110156917	110157037	-0.85	0.35
Rad9b	chr5	122803670	122803730	-0.23	0.81
Rad9b	chr5	122803907	122804085	-0.68	0.80

Immune system

gene_name	chr	chromStart	chromEnd	diff.lg.p	diff.log2.fc
Il1r1	chr1	40370414	40370475	-0.14	0.42
Il1rl1	chr1	40518613	40519152	-1.16	-0.29
Il1rl1	chr1	40521541	40521602	-0.61	0.36
Il18rap	chr1	40605544	40606142	-0.97	-0.28
Stat1	chr1	52216188	52216729	-5.25	-0.42

Casp8	chr1	58890568	58901646	-12.80	-0.51
Abi2	chr1	60527626	60534244	-0.85	-0.13
Cd28	chr1	60826604	60826815	-0.22	-0.36
Cd28	chr1	60827743	60827984	-0.68	-1.75
Creb1	chr1	64622786	64644320	-11.60	0.58
Slc11a1	chr1	74421776	74422382	-2.22	-0.41
Slc11a1	chr1	74432026	74432625	-1.17	-0.10
Cab39	chr1	87690021	87714978	-6.91	-0.42
Cab39	chr1	87745760	87746448	-1.80	-0.10
Eif4e2	chr1	89110488	89110878	-2.76	-0.47
Eif4e2	chr1	89134456	89135775	-1.77	0.21
Inpp5d	chr1	89516916	89517096	-1.75	-0.39
Inpp5d	chr1	89614406	89616844	-1.50	-0.10
Ube2f	chr1	93182063	93182602	-2.18	-0.17
Asb1	chr1	93437321	93437716	-0.36	-0.19
Asb1	chr1	93440810	93443681	-2.13	-0.63
Asb1	chr1	93451344	93451764	-1.52	0.40
Tnfrsf11a	chr1	107723580	107741623	-11.00	-0.52
Arl8a	chr1	137043410	137043590	-5.51	-0.63
Arpc5	chr1	154613671	154613968	-8.47	-0.32
Ncf2	chr1	154654984	154658055	-3.20	-0.20
Rnasel	chr1	155600751	155602307	-0.89	-0.19
Rnasel	chr1	155610184	155610334	-1.19	1.11
Creg1	chr1	167693910	167693971	-1.14	-0.51
Fcgr4	chr1	172959296	172959476	-0.48	-0.13
Aim2	chr1	175389858	175395719	-0.61	0.03
Tmem63a	chr1	182904369	182904638	-2.93	-0.56
Hecw2	chr1	53869614	53883756	-0.93	1.33
Hecw2	chr1	53982405	53983513	-0.56	-0.34
Sumo1	chr1	59696455	59697048	-0.33	-0.01
Tmbim1	chr1	74335180	74335271	-1.94	-0.39
Tmbim1	chr1	74341896	74350790	-7.20	-0.72
Rnf25	chr1	74640325	74640975	-9.13	-0.51
Tuba4a	chr1	75211607	75212025	-1.98	0.61
Tuba4a	chr1	75212113	75215828	-13.90	-0.27
Ap1s3	chr1	79668486	79668547	-0.38	-0.21
Cul3	chr1	80263601	80265851	-6.24	0.34
Cul3	chr1	80336915	80337005	-0.92	-0.29
Trip12	chr1	84721087	84721998	-0.90	-0.07
Trip12	chr1	84782695	84836815	-20.50	-0.26
Pdcd1	chr1	95935089	95937353	-2.58	0.66
Pdcd1	chr1	95938082	95949130	-1.40	-2.60
Bcl2	chr1	108438384	108439704	-0.91	0.22

Bcl2	chr1	108439763	108608408	-0.53	0.24
Bcl2	chr1	108608737	108610537	-2.43	-0.30
Bcl2	chr1	108610626	108610807	-0.63	-0.42
Ptpn4	chr1	121555568	121572143	-4.13	0.38
Ptpn4	chr1	121733557	121733648	-1.41	-1.02
Ikbke	chr1	133151178	133153534	-4.45	-0.31
Ikbke	chr1	133172403	133173518	-2.95	-0.52
Dhx9	chr1	155303276	155303397	-3.58	0.71
Qsox1	chr1	157626418	157628411	-6.98	-0.66
Klhl20	chr1	163019017	163019228	-1.31	-0.43
Klhl20	chr1	163020426	163023835	-2.61	0.52
Klhl20	chr1	163039357	163039897	-3.74	-0.46
Fcgr2b	chr1	172890688	172891194	-2.80	0.13
Fcgr2b	chr1	172906140	172906200	-1.10	-0.38
Fcgr3	chr1	172981477	172981627	-0.50	-0.12
Slamf7	chr1	173562982	173563102	-0.59	3.58
Slamf7	chr1	173563191	173563341	-0.79	4.39
Slamf7	chr1	173563669	173563999	-0.98	5.13
Slamf7	chr1	173564746	173565046	-0.31	0.94
Ncstn	chr1	173996440	173996917	-4.50	-0.29
Degs1	chr1	184206857	184208760	-0.35	-0.01
Degs1	chr1	184209059	184209598	-0.40	0.01
Ppp2r5a	chr1	193176577	193176847	-2.61	0.23
Ppp2r5a	chr1	193196363	193220920	-4.26	-0.27
Il15ra	chr2	11654960	11655499	-0.57	-0.15
Commd3	chr2	18596490	18597638	-0.74	-0.08
Il1rn	chr2	24205097	24205277	-0.69	-0.43
Anapc2	chr2	25128463	25128911	-3.00	-0.30
Fbxw5	chr2	25358984	25359776	-3.57	-0.40
Fbxw5	chr2	25360013	25360422	-1.07	-0.24
Rapgef1	chr2	29475329	29475390	-0.80	-0.67
Rapgef1	chr2	29557850	29558001	-1.86	-0.29
Rapgef1	chr2	29592963	29594536	-4.59	-0.22
Abl1	chr2	31544075	31551290	-7.77	-0.43
Abl1	chr2	31653102	31658189	-1.51	-0.10
Abl1	chr2	31658367	31658428	-0.69	-0.40
Ptges2	chr2	32257786	32257964	-3.05	-0.79
Mapkap1	chr2	34479093	34479423	-0.34	0.02
Hspa5	chr2	34629658	34629748	-39.30	-0.48
Hspa5	chr2	34630750	34631960	-27.90	-0.12
Tank	chr2	61416642	61431195	-2.03	-0.57
Tank	chr2	61482412	61484290	-1.09	0.22
Tank	chr2	61484409	61488467	-4.44	-0.20

Tank	chr2	61491479	61491599	-1.20	-0.21
Klhl41	chr2	69508683	69508923	-1.52	0.86
Dync1i2	chr2	71049792	71050012	-0.90	0.33
Rapgef4	chr2	72091018	72094782	-0.34	-0.96
Itga4	chr2	79165818	79168203	-23.70	0.20
Itgav	chr2	83564583	83564973	-10.50	-1.02
Itgav	chr2	83643447	83643718	-3.67	-0.49
Itgav	chr2	83643807	83643987	-0.74	-0.16
Psmc3	chr2	90894439	90895182	-3.33	-0.20
Traf6	chr2	101530344	101537929	-2.95	-0.21
Kif18a	chr2	109173491	109173672	-0.51	0.32
Pdia3	chr2	121263353	121263680	-0.63	0.03
B2m	chr2	121973422	121973632	-11.70	-0.24
Trim69	chr2	122004226	122004644	-0.30	-0.80
Sirpa	chr2	129418574	129418993	-0.37	-0.03
Sirpa	chr2	129419320	129457247	-2.42	-0.04
Mavs	chr2	131070939	131072532	-2.10	-0.25
Pygb	chr2	150654298	150656585	-0.41	-0.05
Hck	chr2	152934203	152934473	-1.97	0.48
Hck	chr2	152976907	152977028	-1.50	-0.25
Kif3b	chr2	153141983	153143391	-0.42	0.03
Kif3b	chr2	153155890	153156669	-4.11	0.44
Kif3b	chr2	153158285	153158915	-2.59	0.43
Itch	chr2	154959216	154959307	-0.27	-0.02
Itch	chr2	155050102	155050403	-0.31	-0.01
Src	chr2	157295136	157296531	-2.35	0.70
Plcg1	chr2	160587552	160588233	-9.17	-1.31
Ywhab	chr2	163820932	163837497	-10.90	-0.39
Ctsa	chr2	164659257	164659646	-12.00	-0.34
Mmp9	chr2	164780329	164780718	-0.35	-0.13
Cd40	chr2	164896268	164897006	-0.30	-0.03
Rnf114	chr2	167318622	167318832	-1.07	-1.19
Ptpn1	chr2	167757946	167758006	-0.38	-0.08
Ptpn1	chr2	167800939	167802433	-5.76	-0.45
Ptpn1	chr2	167802851	167803629	-1.63	-0.30
Tubb1	chr2	174282485	174283323	-0.40	0.53
Abi1	chr2	22895639	22895730	-1.77	-0.49
Tubb4b	chr2	25077825	25079655	-27.50	-0.32
Tubb4b	chr2	25079713	25080192	-0.32	0.02
Dpp7	chr2	25207809	25207987	-1.48	0.23
Traf2	chr2	25374427	25375145	-2.44	-0.26
Card9	chr2	26212789	26213529	-2.20	-0.22
Agpat2	chr2	26449106	26450082	-0.80	-0.23

Surf4	chr2	26777259	26777678	-29.60	-0.44
Surf4	chr2	26782443	26788971	-5.03	-0.42
Vav2	chr2	27282104	27282345	-2.65	-1.29
Asb6	chr2	30678647	30680212	-9.57	-0.39
Lrsam1	chr2	32781993	32782383	-0.31	-0.02
Fbxw2	chr2	34661048	34661869	-4.78	-0.39
Fbxw2	chr2	34662764	34664040	-0.64	-0.16
Fbxw2	chr2	34668436	34678672	-6.10	-0.35
Fbxw2	chr2	34681468	34681725	-0.46	0.13
Psmd5	chr2	34707993	34709823	-1.65	-0.19
Psmd5	chr2	34726274	34726482	-4.41	-0.82
Rab14	chr2	35056311	35056550	-6.69	-0.39
Psmb7	chr2	38498946	38499426	-3.13	-0.36
Wipf1	chr2	73270300	73270450	-1.18	-0.22
Wipf1	chr2	73273054	73275208	-4.10	-0.31
Wipf1	chr2	73275357	73278120	-11.00	-0.46
Wipf1	chr2	73282616	73291856	-0.37	-0.03
Atf2	chr2	73656947	73657068	-4.98	1.16
Serping1	chr2	84613462	84613523	-0.33	2.32
Ptprj	chr2	90273053	90274190	-33.00	-0.77
Ptprj	chr2	90420444	90420774	-1.91	-0.36
Cd44	chr2	102654086	102662407	-105.00	-0.64
Pin1rt1	chr2	104554491	104554756	-2.79	3.80
Atp8b4	chr2	126148384	126148594	-0.74	-0.12
Anapc1	chr2	128436507	128438637	-1.24	-0.11
Il1b	chr2	129190464	129191002	-3.00	1.94
Il1b	chr2	129195984	129196856	-1.12	2.89
Ubox5	chr2	130416996	130417566	-1.55	-0.37
Ubox5	chr2	130425355	130426405	-1.35	-0.24
Siglec1	chr2	130895015	130895584	-2.61	0.78
Siglec1	chr2	130900602	130901900	-1.04	-0.31
Siglec1	chr2	130903954	130904467	-0.76	-0.28
Siglec1	chr2	130907034	130909080	-1.77	-0.56
Siglec1	chr2	130911312	130911373	-0.89	-1.09
Siglec1	chr2	130911872	130912501	-2.29	0.94
Cd93	chr2	148263313	148263403	-0.46	2.58
Cd93	chr2	148266721	148267417	-0.45	1.49
Cd93	chr2	148268044	148268583	-0.86	3.61
Cd93	chr2	148268792	148269092	-0.67	2.88
Psmf1	chr2	151543111	151543202	-1.31	-0.87
Psmf1	chr2	151543649	151544038	-0.30	0.01
Trib3	chr2	152163160	152164409	-25.70	-0.34
Trib3	chr2	152169058	152169796	-0.86	-0.16

Bcl2l1	chr2	152606912	152607272	-0.96	0.13
Bcl2l1	chr2	152607660	152653984	-20.10	-0.61
Bcl2l1	chr2	152655121	152657418	-135.00	-0.91
Cpne1	chr2	155922188	155937671	-1.89	0.11
Dsn1	chr2	156820797	156820977	-0.82	0.77
Dsn1	chr2	156821482	156822471	-0.69	-0.19
Slpi	chr2	164181923	164182042	-77.90	-1.59
Elmo2	chr2	165115177	165116717	-4.35	-0.36
Elmo2	chr2	165117584	165119793	-0.30	0.03
Nfatc2	chr2	168301969	168303229	-1.59	-0.26
Nfatc2	chr2	168304128	168330396	-9.73	-0.53
Nfatc2	chr2	168396287	168427005	-6.54	-0.50
Ctsz	chr2	174252994	174254182	-128.00	-0.34
Fabp5	chr3	10014973	10015506	-2.36	-0.25
Pld1	chr3	28029285	28031356	-0.61	-0.13
Pld1	chr3	28031505	28031596	-0.48	-0.38
Pld1	chr3	28031774	28031985	-0.32	-0.11
Pik3ca	chr3	32335072	32335342	-3.17	-0.61
Pik3ca	chr3	32360651	32362022	-2.40	0.26
Rap2b	chr3	61168488	61170347	-6.56	0.26
Rap2b	chr3	61171125	61171336	-0.48	0.24
Ctso	chr3	81756174	81758885	-0.59	0.09
Fbxw7	chr3	84619498	84694070	-0.58	-0.12
Fbxw7	chr3	84694129	84756226	-0.51	0.05
Fbxw7	chr3	84780110	84781955	-0.35	0.02
Shc1	chr3	89222472	89225623	-0.50	-0.10
Shc1	chr3	89225682	89226222	-15.40	-1.10
Shc1	chr3	89232244	89232484	-0.52	0.07
Ube2q1	chr3	89577530	89577890	-0.54	-0.06
Ube2q1	chr3	89585705	89587919	-0.80	-0.04
Ilf2	chr3	90291521	90291852	-2.02	-0.23
Them4	chr3	94135467	94135946	-0.33	0.03
Ctss	chr3	95330707	95333423	-10.90	-0.35
Txnip	chr3	96362296	96362849	-20.70	0.38
Txnip	chr3	96363757	96364946	-72.50	0.32
Txnip	chr3	96365302	96365542	-18.10	0.47
Rnf115	chr3	96592580	96594008	-0.38	-0.02
Sec22b	chr3	97705149	97711158	-2.16	-0.33
Sec22b	chr3	97725110	97725468	-2.27	-0.20
Ptpn22	chr3	103664257	103664347	-5.38	-0.77
Ptpn22	chr3	103685999	103690208	-24.30	-0.48
Psma5	chr3	108059873	108060022	-0.64	3.39
Vav3	chr3	109143660	109143930	-1.24	-0.66

Cenpe	chr3	134902767	134902887	-0.61	0.20
Cenpe	chr3	134905081	134905534	-0.42	-0.07
Cenpe	chr3	134910353	134910534	-1.68	-0.55
Cenpe	chr3	134936264	134936504	-0.50	-0.15
Ube2d3	chr3	135101656	135102228	-19.60	-0.75
Manba	chr3	135230676	135233888	-1.56	-0.17
Bcl10	chr3	145587232	145587592	-4.99	-0.42
Bcl10	chr3	145588099	145593556	-3.89	-0.41
Il7	chr3	7573665	7573875	-0.26	-0.89
Pag1	chr3	9690420	9691051	-2.03	-0.38
Pag1	chr3	9691830	9704750	-16.60	-0.50
Nhlrc3	chr3	53255917	53257551	-1.05	-0.16
Siah2	chr3	58479649	58495381	-3.84	-0.27
Siah2	chr3	58495620	58496010	-0.94	0.28
Dhx36	chr3	62274511	62275295	-0.71	-0.15
Tlr2	chr3	83640193	83642691	-5.43	-0.11
Lamtor2	chr3	88353913	88356849	-9.31	-0.34
Il6ra	chr3	89673305	89680757	-0.99	0.10
Psmb4	chr3	94690701	94690880	-8.50	-0.41
Psmd4	chr3	94836626	94836894	-2.54	-0.16
Psmd4	chr3	94840526	94846467	-8.21	-0.35
Hist2h3c2	chr3	96042365	96042604	-1.05	0.63
Fcgr1	chr3	96087130	96087191	-0.26	0.03
Fcgr1	chr3	96087728	96088506	-8.72	-0.57
Cd101	chr3	100797451	100797662	-0.59	-2.75
Cd53	chr3	106563447	106563626	-3.51	-0.17
Csf1	chr3	107544832	107548597	-0.68	0.16
Csf1	chr3	107548745	107550736	-0.68	-0.96
Csf1	chr3	107551093	107553341	-0.96	0.24
Sec24b	chr3	129686102	129687101	-1.82	-0.14
Sec24b	chr3	129743865	129763735	-15.50	-0.56
Nfkb1	chr3	135248247	135252299	-9.58	-0.45
Nfkb1	chr3	135354240	135354451	-1.35	0.77
Dapp1	chr3	137595557	137598550	-2.97	-0.66
Prkacb	chr3	146394694	146395023	-0.79	0.13
Lyn	chr4	3605267	3605506	-12.00	-0.40
Lyn	chr4	3716941	3718014	-40.00	-0.36
Fbxl4	chr4	22303713	22313289	-1.60	-0.28
Map3k7	chr4	32051081	32051232	-3.02	-1.01
Map3k7	chr4	32104109	32107267	-5.97	0.38
Map3k7	chr4	32110051	32110231	-0.27	0.00
Ube2r2	chr4	41137807	41138136	-0.41	-0.03
Atp6v1g1	chr4	63205828	63209679	-2.22	-0.30

Trim32	chr4	65274645	65274706	-0.48	-0.20
Trim32	chr4	65274854	65276169	-0.55	-0.05
Tlr4	chr4	66494965	66502995	-6.26	0.21
Pgm2	chr4	99602055	99602264	-0.67	-0.17
Pgm2	chr4	99656703	99659542	-3.27	-0.43
Lrrc41	chr4	115761466	115762183	-23.20	-0.55
Lrrc41	chr4	115768961	115769201	-3.73	-0.33
Pik3r3	chr4	115945443	115972695	-28.90	3.76
Pik3r3	chr4	115975001	115975152	-1.61	1.80
Csf3r	chr4	125720761	125721238	-1.10	-0.26
Rnf19b	chr4	128735514	128735993	-17.90	-1.08
Rnf19b	chr4	128759998	128761505	-99.00	-1.37
Ptafr	chr4	132119981	132136781	-1.25	0.09
Fgr	chr4	132530069	132540100	-0.30	0.03
Fgr	chr4	132556096	132556544	-10.20	-0.69
Fgr	chr4	132557289	132557558	-7.56	1.29
Wasf2	chr4	132751672	132752809	-0.40	0.01
Fuca1	chr4	135476640	135476908	-6.65	-0.76
Fuca1	chr4	135495027	135495325	-0.87	0.15
Eif4g3	chr4	137676266	137682738	-1.73	-0.08
Eif4g3	chr4	137762235	137762386	-0.44	0.08
Ddost	chr4	137868076	137868376	-2.25	-0.24
Capzb	chr4	138748845	138794826	-3.32	-0.32
Casp9	chr4	141352424	141352724	-6.06	-0.88
Casp9	chr4	141368153	141369672	-1.63	-0.61
Casp9	chr4	141369791	141369941	-0.24	-0.15
Mtor	chr4	147822690	147822781	-3.13	-1.30
Mtor	chr4	147930392	147931764	-3.53	-0.34
Masp2	chr4	147988211	147988362	-7.34	1.67
Slc2a5	chr4	149493913	149494152	-0.24	0.47
Klhl21	chr4	151383209	151383749	-20.10	-1.89
Klhl21	chr4	151383868	151389959	-10.20	-0.52
Klhl21	chr4	151390257	151390438	-3.61	1.07
Ube2j2	chr4	155330537	155331768	-9.18	-0.43
Ripk2	chr4	16050670	16051621	-4.17	-0.41
Ripk2	chr4	16090436	16090645	-16.00	-1.44
Ufl1	chr4	25177588	25177709	-0.30	-0.02
Ddx58	chr4	40152603	40155547	-1.90	-0.20
Ddx58	chr4	40186678	40186858	-0.44	0.12
B4galt1	chr4	40753786	40754830	-1.36	0.12
B4galt1	chr4	40770597	40800613	-1.85	0.25
B4galt1	chr4	40800671	40801001	-2.65	-0.48
Fbxo10	chr4	45048045	45048374	-1.12	1.75

Fbxo10	chr4	45048642	45048793	-0.22	0.14
Fbxo10	chr4	45071323	45071981	-0.27	-0.03
Cdc26	chr4	62055639	62055998	-0.37	-0.07
Alad	chr4	62170203	62171204	-0.56	-0.14
Ifnb1	chr4	88167987	88168314	-0.43	1.96
Klhl9	chr4	88366049	88367814	-9.57	0.24
Jun	chr4	94716292	94718913	-60.00	-0.27
Jak1	chr4	100825566	100826076	-0.60	0.05
Kif2c	chr4	116832539	116832928	-0.28	0.02
Rnf220	chr4	116961136	116968779	-2.43	-0.37
Cdc20	chr4	118109887	118109948	-0.76	-0.68
Cap1	chr4	122536500	122536651	-58.00	-0.70
Lck	chr4	129225797	129226157	-0.39	0.06
Lck	chr4	129234249	129234661	-0.99	2.21
Txlna	chr4	129305539	129309516	-6.31	-0.20
Txlna	chr4	129316496	129317784	-1.42	-0.10
Rps6ka1	chr4	133403354	133404010	-3.84	-0.27
Cda	chr4	137894622	137894742	-1.22	1.10
Tnfrsf1b	chr4	144803977	144809673	-299.00	-1.07
Tnfrsf1b	chr4	144818975	144836773	-24.20	-1.34
Tnfrsf1b	chr4	144836594	144836774	-2.87	-0.59
Tnfrsf8	chr4	144858908	144859116	-0.56	-0.47
Pik3cd	chr4	149023575	149025880	-7.02	-0.36
Spsb1	chr4	149271916	149281307	-0.79	-0.50
Kcnab2	chr4	151766373	151767149	-1.53	0.12
Mib2	chr4	155028580	155028821	-0.32	-0.05
Atad3a	chr4	155114748	155115286	-0.74	-0.07
Isg15	chr4	155573619	155574927	-38.70	-1.36
Ube3c	chr5	29895781	29895962	-0.89	-0.61
Ube3c	chr5	30001057	30001238	-1.45	-0.31
Rnf4	chr5	34694045	34694196	-2.72	-0.31
Bst1	chr5	44210131	44210281	-0.40	-0.14
Anapc4	chr5	53257704	53257973	-1.23	-0.22
Klhl5	chr5	65558211	65558721	-0.75	-0.12
Cxcl3	chr5	91215188	91215370	-0.57	-0.91
Cxcl2	chr5	91332924	91333224	-434.00	-2.46
Stbd1	chr5	93034080	93034170	-0.19	-0.81
Ube3b	chr5	114830704	114830795	-0.26	0.26
Ube3b	chr5	114869593	114869952	-0.90	0.14
Fbxo21	chr5	118452063	118458618	-6.36	-0.76
Hvcn1	chr5	122690237	122692276	-4.38	-0.49
Anapc7	chr5	122891328	122891538	-0.73	0.15
Anapc7	chr5	122893905	122894532	-19.20	-0.92

P2rx7	chr5	123093919	123093980	-1.91	1.26
P2rx7	chr5	123130997	123132855	-5.21	0.20
P2rx7	chr5	123133124	123134024	-5.90	0.37
Rnf34	chr5	123273869	123314504	-0.81	0.09
Psmd9	chr5	123678228	123678437	-0.34	0.03
Atp6v0a2	chr5	125200361	125202517	-2.97	0.32
Kctd7	chr5	130628217	130628785	-0.53	-0.24
Arpc1b	chr5	145875124	145883438	-11.60	-0.35
Tnip2	chr5	34842175	34842385	-0.32	-0.02
Fbxl5	chr5	44142150	44149098	-2.88	-0.56
Fbxl5	chr5	44149396	44151973	-0.84	0.08
Tlr1	chr5	65316072	65316492	-1.44	0.54
Tlr1	chr5	65318106	65318495	-0.55	-0.20
Tlr6	chr5	65344333	65351228	-7.16	0.44
Atp8a1	chr5	68013273	68013813	-0.94	-0.18
Tec	chr5	73147051	73148633	-0.92	-0.13
Tec	chr5	73214871	73259687	-0.34	0.03
Rchy1	chr5	92391825	92392094	-0.33	0.04
Sec31a	chr5	100790907	100791058	-0.72	-0.09
Mlec	chr5	115597834	115600228	-0.61	-0.05
Mlec	chr5	115600898	115608185	-12.50	-0.43
Fbxw8	chr5	118515497	118515797	-0.28	0.02
Fbxw8	chr5	118605107	118605467	-13.80	-1.01
Ptpn11	chr5	121581827	121582307	-1.21	0.16
Ptpn11	chr5	121583292	121587519	-0.40	0.02
Anapc5	chr5	123271138	123271348	-2.63	-0.39
Rhof	chr5	123553559	123554127	-8.81	2.11
Rhof	chr5	123554185	123554246	-2.05	2.49
Rhof	chr5	123567437	123567796	-0.54	-0.19
Rhof	chr5	123568153	123582399	-35.90	-0.59
Ubc	chr5	125866453	125867671	-172.00	0.73
Ubc	chr5	125867938	125868147	-3.15	0.20
Ubc	chr5	125868235	125870298	-22.80	-0.40
Slc15a4	chr5	128076540	128080872	-0.95	-0.12
Gusb	chr5	130464890	130465519	-1.85	-0.08
Gusb	chr5	130478517	130478698	-10.40	-0.43
Ncf1	chr5	134697302	134697663	-2.36	-0.12
Lat2	chr5	135076494	135078531	-7.71	-0.25
Trim56	chr5	137587155	137588276	-8.63	-0.39
Trim56	chr5	137588605	137592077	-46.30	-0.54
Pilra	chr5	138263299	138263719	-6.34	2.30
Pilra	chr5	138264706	138265015	-0.36	0.18
Pilra	chr5	138276577	138276877	-2.44	1.44

Card11	chr5	141348952	141352360	-0.69	0.26
Fbxl18	chr5	143633766	143634006	-6.05	-1.06
Fbxl18	chr5	143647153	143648949	-184.00	-2.23
Rac1	chr5	144288681	144288861	-1.21	-0.22
Smurf1	chr5	145637663	145638682	-0.50	0.08
Smurf1	chr5	145639310	145639521	-0.66	0.35
Pdap1	chr5	145892293	145900958	-14.40	-0.32
Rnf6	chr5	147021793	147022181	-6.60	0.51
Rnf6	chr5	147023075	147031998	-2.47	0.16
Cul1	chr6	47404345	47445165	-3.56	0.31
Cul1	chr6	47474617	47476108	-1.64	-0.15
Herc6	chr6	57445639	57531152	-0.66	-0.46
Dctn1	chr6	83140226	83141067	-0.45	0.03
Kbtbd8	chr6	95071991	95077327	-2.49	0.37
Vhl	chr6	113578147	113579978	-0.46	-0.06
Irak2	chr6	113640953	113644480	-29.40	-0.65
Fbxl14	chr6	119429685	119431451	-0.36	-0.01
Il17ra	chr6	120413214	120413425	-0.34	0.04
Il17ra	chr6	120431139	120433146	-3.70	-0.15
Clec4d	chr6	123212154	123212214	-0.48	0.14
C1ra	chr6	124472382	124472951	-1.63	-0.40
Spsb2	chr6	124758812	124760455	-90.80	-1.86
Pianp	chr6	124949362	124950749	-5.78	-0.31
Tnfrsf1a	chr6	125311560	125312501	-18.50	-0.45
Gm8994	chr6	136279021	136279141	-0.30	-0.81
Gm8994	chr6	136279763	136279853	-0.25	0.86
Lrmp	chr6	145086263	145086444	-0.29	-0.02
Klhl42	chr6	147040014	147050109	-0.97	-0.28
Klhl42	chr6	147050168	147056940	-3.38	-0.59
Impdh1	chr6	29159242	29166081	-0.86	-0.16
Ube2h	chr6	30163827	30164395	-1.91	0.24
Ube2h	chr6	30164783	30164933	-0.40	-0.15
Clec5a	chr6	40527283	40529441	-4.88	-0.13
Nod1	chr6	54874531	54874682	-0.75	1.04
Nod1	chr6	54893168	54893616	-0.68	0.56
Nod1	chr6	54893675	54894034	-0.75	0.80
Nod1	chr6	54894242	54894451	-0.24	-0.71
Nod1	chr6	54894600	54897981	-1.54	1.88
Vamp8	chr6	72335214	72335448	-3.83	-0.35
Vamp8	chr6	72338374	72340603	-0.85	-0.14
Kcmf1	chr6	72849792	72849973	-0.26	-0.02
Rbsn	chr6	92139135	92140425	-0.94	0.10
Uba3	chr6	97134556	97134676	-1.20	0.47

Raf1	chr6	115568620	115569280	-0.58	0.06
Alox5	chr6	116411016	116411196	-1.91	-0.74
Atp6v1e1	chr6	120768397	120772703	-5.89	-0.47
Slc2a3	chr6	122679711	122680131	-3.19	-0.53
C3ar1	chr6	122799485	122806115	-3.47	-0.14
C1s2	chr6	124575599	124575809	-0.29	1.36
Ptpn6	chr6	124683045	124688519	-0.41	-0.07
Ltbr	chr6	125256588	125259229	-3.92	-0.23
Olr1	chr6	129437034	129437784	-0.56	2.18
Kras	chr6	145168813	145168964	-1.16	-0.35
Itpr2	chr6	146060001	146061041	-8.07	0.54
Ap2s1	chr7	17323792	17328591	-2.88	-0.37
Plaur	chr7	25247518	25251694	-110.00	-1.93
Plaur	chr7	25260294	25260713	-183.00	-1.49
Akt2	chr7	28422173	28423398	-0.50	-0.06
Irf3	chr7	52253029	52253179	-1.59	-0.48
Irf3	chr7	52257097	52257801	-2.81	-0.37
Irf3	chr7	52257979	52258099	-1.43	-1.19
Cyfip1	chr7	63097610	63127258	-0.50	0.06
Herc2	chr7	63485072	63486928	-1.61	0.14
Ube3a	chr7	66484418	66484628	-2.76	-0.49
Ube3a	chr7	66531169	66540150	-0.51	-0.02
Klhl25	chr7	82993313	83012167	-0.30	0.00
Klhl25	chr7	83012375	83018269	-2.48	-0.60
Klhl25	chr7	83018657	83018926	-0.33	0.09
Ctsc	chr7	95437245	95437485	-0.96	0.45
Ctsc	chr7	95458037	95458547	-0.94	0.12
Prcp	chr7	100081624	100082283	-0.57	-0.08
Ndufc2	chr7	104548571	104548690	-0.43	0.05
Lamtor1	chr7	109054380	109058241	-0.87	-0.19
Lamtor1	chr7	109059942	109060091	-16.60	-0.68
Ampd3	chr7	117954502	117954712	-3.65	0.61
Dctn5	chr7	129276554	129277689	-0.48	-0.08
Dctn5	chr7	129291791	129292440	-0.33	-0.01
Rbbp6	chr7	130114077	130114468	-5.64	-0.37
Rbbp6	chr7	130133635	130133876	-1.64	0.20
Rbbp6	chr7	130140555	130140795	-1.26	0.15
Rbbp6	chr7	130142061	130145876	-2.57	0.06
Il4ra	chr7	132719062	132720501	-2.64	-0.43
Il4ra	chr7	132720770	132721340	-2.72	0.67
Il21r	chr7	132776035	132776545	-0.83	1.25
Fbxl19	chr7	134890288	134891744	-4.33	-0.90
Fbxl19	chr7	134891803	134891893	-0.66	-0.35

Fbxl19	chr7	134893270	134904820	-11.30	-0.67
Fbxl19	chr7	134906111	134911817	-1.15	-0.18
Fbxl19	chr7	134911876	134912442	-2.21	-0.45
Stx4a	chr7	134985321	134985978	-1.03	-0.12
Itgam	chr7	135270386	135270776	-5.15	0.99
Itgax	chr7	135292687	135294081	-2.59	1.26
Lair1	chr7	3960550	3960699	-0.30	0.16
Lair1	chr7	3961056	3961295	-0.66	-0.33
Il11	chr7	4724009	4724369	-3.93	1.73
Il11	chr7	4724995	4729743	-1.13	0.28
Ube2m	chr7	13623018	13623619	-6.02	-0.45
C5ar2	chr7	16822655	16823045	-0.48	0.50
C5ar2	chr7	16823134	16823315	-0.25	-0.26
C5ar1	chr7	16833166	16844566	-69.90	-0.77
Calm3	chr7	17502039	17502309	-1.46	-0.09
Klc3	chr7	19979788	19981213	-2.01	-0.31
Relb	chr7	20191690	20192229	-0.83	-0.11
Nectin2	chr7	20302501	20302950	-0.50	0.24
Nectin2	chr7	20306842	20310098	-3.95	-0.76
Nectin2	chr7	20334652	20334862	-0.34	-0.09
Pvr	chr7	20490268	20494550	-0.33	0.02
Pvr	chr7	20502401	20506492	-38.60	-0.97
Ceacam1	chr7	26247739	26248638	-4.67	0.32
Ceacam1	chr7	26257014	26258919	-1.16	-0.37
Ceacam1	chr7	26259008	26274241	-0.36	-0.02
Nfkbib	chr7	29546684	29546864	-25.70	-0.94
Nfkbib	chr7	29547132	29551543	-9.87	-0.61
Psmd8	chr7	29965393	29965573	-13.80	-0.43
Cd22	chr7	31662494	31662794	-1.10	2.82
Gpi1	chr7	34987182	34987689	-15.30	-0.25
Siglece	chr7	50906826	50906947	-0.53	0.45
Siglece	chr7	50914669	50914730	-0.37	-0.51
Flt3l	chr7	52386558	52386918	-0.67	0.27
Ftl1	chr7	52713604	52713914	-25.90	-0.25
Ftl1	chr7	52715051	52715227	-434.00	-0.45
Asb7	chr7	73792534	73823980	-3.67	-0.42
Asb7	chr7	73834207	73834387	-0.58	0.31
Det1	chr7	85972688	85972809	-1.10	-0.51
Det1	chr7	85988146	85988866	-0.56	-0.07
Anpep	chr7	86966748	86966897	-1.17	-0.22
Anpep	chr7	86986085	86993080	-7.52	-0.20
Iqgap1	chr7	87857938	87858329	-21.50	-0.33
Iqgap1	chr7	87944725	87948217	-28.40	-0.56

Il16	chr7	90792230	90794430	-1.06	-0.23
Il16	chr7	90800555	90809792	-1.37	-0.12
Il16	chr7	90870990	90871201	-1.10	0.43
Inppl1	chr7	108971385	108974549	-40.10	-0.55
Inppl1	chr7	108985423	108986317	-1.81	-0.40
Inppl1	chr7	108986619	108986740	-1.05	1.07
Rhog	chr7	109387636	109398632	-32.90	-0.30
Trim21	chr7	109707151	109710434	-1.56	0.22
Sh2b1	chr7	133610925	133611549	-2.80	-0.45
Sh2b1	chr7	133615254	133616302	-10.60	-0.55
Sh2b1	chr7	133618136	133618465	-0.50	-0.15
Aldoa	chr7	133943900	133943961	-0.20	0.10
Kif22	chr7	134179351	134185934	-2.04	-0.85
Adam8	chr7	147164929	147165378	-47.80	-0.66
Ifitm2	chr7	148141799	148141860	-2.49	-0.23
Ifitm3	chr7	148196466	148196643	-44.70	-0.36
Hras	chr7	148376788	148378177	-0.34	-0.01
Hras	chr7	148378775	148379903	-0.71	0.21
Tollip	chr7	149069998	149076626	-5.15	-0.28
Ctsd	chr7	149561820	149562294	-118.00	-0.34
Fadd	chr7	151765572	151768192	-4.39	-0.39
Map2k7	chr8	4245546	4246251	-0.79	0.09
Tnfsf13b	chr8	10035492	10035790	-2.07	0.90
Atp11a	chr8	12757045	12757285	-0.28	0.04
Atp11a	chr8	12864987	12866484	-1.11	0.12
Lamp1	chr8	13159223	13159402	-12.50	-0.58
Cdc16	chr8	13779263	13781763	-4.99	-0.38
Plpp5	chr8	26834551	26835000	-0.42	0.03
Ppp2cb	chr8	34710092	34710359	-0.73	-0.37
Dusp4	chr8	35870663	35882259	-130.00	-0.63
Sh3rf1	chr8	63704632	63808042	-0.38	0.08
Sh3rf1	chr8	63863616	63863767	-0.29	-0.10
Sh3rf1	chr8	63872471	63873071	-0.74	-0.31
Rab3a	chr8	73282404	73282465	-0.44	0.34
Il12rb1	chr8	73343643	73344933	-13.80	-1.50
Myo9b	chr8	73796612	73839032	-3.45	-0.17
Myo9b	chr8	73871704	73876118	-7.70	-0.21
Myo9b	chr8	73880532	73884611	-0.34	0.00
Prkaca	chr8	86496876	86500855	-1.59	-0.45
Man2b1	chr8	87607167	87607377	-1.33	0.29
Cyld	chr8	91220956	91226218	-0.50	0.37
Cyld	chr8	91226307	91233793	-2.31	0.21
Fbxl8	chr8	107790925	107791573	-0.65	0.30

Fbxl8	chr8	107791922	107793108	-0.55	0.10
Nfatc3	chr8	108602601	108603618	-3.27	0.21
Nfatc3	chr8	108632435	108652196	-8.48	0.41
Ap1g1	chr8	112382745	112384686	-3.66	-0.19
Znrf1	chr8	114060539	114062275	-0.69	0.05
Znrf1	chr8	114143127	114147028	-1.41	-0.07
Gan	chr8	119728191	119729086	-7.70	0.65
Plcg2	chr8	120022190	120028105	-0.35	-0.03
Tubb3	chr8	125942898	125945049	-0.53	-0.56
Itgb1	chr8	131209643	131209853	-0.68	-0.08
Itgb1	chr8	131256163	131256313	-1.27	0.17
Ikbkb	chr8	23770606	23771027	-1.32	-0.20
Golga7	chr8	24352876	24353027	-5.60	-0.49
Hgsnat	chr8	27054960	27055615	-2.49	0.23
Hgsnat	chr8	27083350	27087156	-0.93	-0.21
Dctn6	chr8	35169435	35171565	-0.29	-0.02
Lonrf1	chr8	37280167	37282962	-0.30	-0.01
Lonrf1	chr8	37311699	37312090	-2.20	-1.14
Tlr3	chr8	46482187	46484515	-6.15	0.91
Tlr3	chr8	46488157	46488217	-1.04	1.33
Klhl2	chr8	67219391	67219870	-0.73	-0.15
Pik3r2	chr8	73292079	73292528	-7.07	-0.81
Pik3r2	chr8	73296811	73300581	-13.40	-0.87
Adgre5	chr8	86247268	86247657	-11.60	-1.46
Calr	chr8	87366315	87366863	-41.40	-0.26
Calr	chr8	87370234	87370800	-11.20	-0.26
Siah1a	chr8	89248786	89269816	-0.36	-0.02
Dync1li2	chr8	106944120	106944421	-2.39	-0.28
Dync1li2	chr8	106966646	106966737	-0.40	0.11
Psmb10	chr8	108461631	108461967	-2.80	-0.63
Psmd7	chr8	110104338	110108077	-4.03	0.12
Hp	chr8	112099087	112099805	-0.32	-0.05
Ist1	chr8	112195789	112196630	-0.63	0.04
Cotl1	chr8	122364299	122364449	-0.56	-0.07
Fbxo31	chr8	124075878	124076147	-0.29	0.02
Galns	chr8	125102404	125103030	-5.07	-0.53
Casp1	chr9	5298635	5299458	-1.15	-0.21
Pin1	chr9	20456573	20459918	-1.39	-0.27
Icam1	chr9	20820403	20823430	-2.14	-0.45
Icam1	chr9	20823638	20823729	-0.38	-0.07
Icam1	chr9	20824146	20824566	-0.50	0.12
Icam1	chr9	20831708	20833091	-2.44	-0.10
Icam5	chr9	20841007	20841187	-0.45	-0.34

Slc44a2	chr9	21156935	21159322	-1.03	0.07
Dnm2	chr9	21310134	21311379	-2.43	0.16
Dnm2	chr9	21311898	21312168	-2.03	0.27
Smarca4	chr9	21505589	21507505	-3.30	0.23
Ppp2r1b	chr9	50687990	50688710	-0.71	-0.09
Ppp2r1b	chr9	50701016	50701675	-4.03	-0.60
Fbxo22	chr9	55066270	55071672	-1.24	0.12
Fbxo22	chr9	55071791	55072031	-0.53	0.10
Herc1	chr9	66198256	66220501	-4.31	0.30
Herc1	chr9	66293942	66296296	-2.94	0.33
Herc1	chr9	66298665	66298935	-1.74	0.27
Adam10	chr9	70526807	70526958	-1.07	0.28
Adam10	chr9	70626086	70626297	-2.80	0.46
Rab27a	chr9	72892731	72908786	-0.39	-0.66
Rab27a	chr9	72943089	72943780	-0.59	0.27
Myo5a	chr9	74919012	74919403	-0.86	0.18
Myo5a	chr9	75065709	75066549	-8.29	0.29
Anapc13	chr9	102528625	102532216	-1.16	-0.23
Pik3r4	chr9	105545624	105545744	-0.46	-0.22
Pik3r4	chr9	105546492	105551238	-0.65	0.07
Pik3r4	chr9	105552647	105556359	-1.62	-0.23
Pik3r4	chr9	105589445	105589955	-0.34	-0.02
Tlr9	chr9	106124928	106129177	-69.30	1.15
Dusp7	chr9	106270962	106271201	-3.92	-1.70
Dusp7	chr9	106271527	106276865	-1.31	-0.10
Dcaf1	chr9	106738991	106740882	-3.19	0.55
Dcaf1	chr9	106760577	106761417	-1.12	0.15
Dcaf1	chr9	106776469	106782364	-1.52	0.38
Glb1	chr9	114310242	114326114	-1.32	0.33
Glb1	chr9	114368800	114373509	-0.47	-0.05
Glb1	chr9	114374377	114383495	-4.81	0.37
Dync1li1	chr9	114632686	114632895	-0.93	0.16
Dync1li1	chr9	114632812	114633189	-1.24	0.13
Ctnnb1	chr9	120842577	120859669	-0.34	-0.01
Ctnnb1	chr9	120867712	120868847	-5.68	0.15
Ccr2	chr9	124021097	124021367	-0.45	-0.18
Birc2	chr9	7818614	7818734	-0.55	-0.29
Birc2	chr9	7833610	7834657	-0.90	0.13
Birc2	chr9	7834955	7835195	-1.05	0.42
Birc3	chr9	7848969	7850928	-1.17	-0.17
Birc3	chr9	7860311	7872961	-0.30	0.00
Panx1	chr9	14810228	14812409	-2.17	-0.38
Fbxl12	chr9	20448880	20449151	-0.85	-0.48

Keap1	chr9	21034473	21038380	-0.55	0.03
Keap1	chr9	21041615	21042276	-1.55	-0.22
Keap1	chr9	21043205	21043686	-0.40	0.13
Rab3d	chr9	21714741	21715042	-1.16	-0.47
Tirap	chr9	34994065	34994215	-0.38	-0.10
Tirap	chr9	34994423	34996823	-0.38	0.02
Crtam	chr9	40781146	40781620	-0.44	0.47
Cbl	chr9	43959287	43960877	-0.32	0.00
Cbl	chr9	44041859	44042099	-4.62	-0.81
Nlrx1	chr9	44060795	44061574	-1.60	-0.43
Nlrx1	chr9	44070526	44072299	-3.29	-0.49
Nlrx1	chr9	44076107	44076168	-0.85	1.38
Ube4a	chr9	44731508	44732078	-3.85	0.39
Il10ra	chr9	45062219	45062638	-0.85	0.20
Il10ra	chr9	45063175	45068489	-0.47	0.03
Pafah1b2	chr9	45776653	45777013	-2.67	0.20
Cul5	chr9	53425530	53429279	-5.02	0.34
Chrnb4	chr9	54882486	54883206	-4.11	2.77
Csk	chr9	57474661	57475560	-6.84	-0.21
Arih1	chr9	59302235	59325631	-2.11	0.44
Arih1	chr9	59329494	59334181	-4.64	0.32
Pias1	chr9	62729583	62729943	-0.50	0.10
Pias1	chr9	62799649	62828686	-0.80	0.12
Rnf111	chr9	70274555	70277390	-0.83	-0.10
Rnf111	chr9	70323650	70350792	-12.70	0.37
Tmem30a	chr9	79617046	79617405	-0.64	-0.07
Pik3cb	chr9	98939628	98941447	-5.88	0.37
Armc8	chr9	99380819	99381090	-0.78	-0.26
Armc8	chr9	99420641	99420852	-1.07	0.54
Il20rb	chr9	100358436	100359660	-0.56	-0.27
Nck1	chr9	100395659	100407191	-0.86	0.08
Uba5	chr9	103949273	103951621	-4.43	0.27
Dnajc13	chr9	104054165	104054644	-6.55	0.41
Rnf123	chr9	107954002	107954243	-6.46	0.71
Apeh	chr9	107987744	107987894	-0.71	-0.29
Arih2	chr9	108506380	108506471	-9.95	5.42
Arih2	chr9	108546363	108547287	-1.88	-0.31
Trim71	chr9	114422031	114422092	-0.38	2.58
Trim71	chr9	114422299	114422658	-0.97	5.09
Myd88	chr9	119248978	119249158	-3.52	-0.60
Fbxo30	chr10	11001127	11015345	-10.80	0.40
Fuca2	chr10	13223007	13225667	-1.77	-0.36
Fuca2	chr10	13234515	13235470	-1.15	0.10

Ifngr1	chr10	19329011	19329761	-0.30	0.00
Frk	chr10	34203714	34203924	-0.23	-0.42
Fyn	chr10	39089604	39089994	-0.68	-0.12
Fyn	chr10	39271197	39284102	-0.31	0.00
Lilr4b	chr10	51200478	51200715	-8.29	-0.49
Lilr4b	chr10	51205082	51205501	-195.00	-1.21
Psap	chr10	59740405	59754400	-4.91	-0.38
Herc4	chr10	62706723	62726819	-16.00	-0.60
Itgb2	chr10	76993092	77004744	-8.05	-0.66
Ube2g2	chr10	77107280	77107637	-1.33	-0.14
Ube2g2	chr10	77107992	77108082	-0.37	-0.04
Icosl	chr10	77539904	77541941	-1.63	0.31
Cdc34	chr10	79144939	79145322	-5.03	-0.46
Cdc34	chr10	79148043	79151143	-18.90	-0.38
Arhgap45	chr10	79479416	79480842	-6.48	-0.36
Arhgap45	chr10	79485025	79488219	-0.85	-0.08
Arhgap45	chr10	79493111	79494216	-1.79	-0.12
Eef2	chr10	80644273	80645254	-24.30	-0.11
Eea1	chr10	95473536	95474748	-15.40	-0.60
Eea1	chr10	95488798	95489514	-17.80	-0.66
Eea1	chr10	95499355	95500064	-0.36	0.03
Eea1	chr10	95502273	95503004	-2.73	-0.24
Eea1	chr10	95504160	95504914	-0.43	0.04
Dusp6	chr10	98725864	98729379	-0.51	-0.02
Gns	chr10	120802145	120802325	-0.30	-0.01
Gns	chr10	120832206	120832776	-1.28	0.07
Gns	chr10	120833971	120834301	-5.31	0.31
Stat6	chr10	127080041	127080222	-2.32	-0.33
Stat6	chr10	127096737	127097564	-4.04	-0.16
Stat2	chr10	127727850	127728797	-3.58	-0.35
Stat2	chr10	127729305	127729665	-1.61	-0.28
Rnf41	chr10	127874437	127875829	-14.90	-0.48
Tab2	chr10	7625983	7626463	-1.79	0.18
Tab2	chr10	7626701	7630910	-3.35	-0.17
Tab2	chr10	7631204	7644574	-1.97	-0.07
Tnfaip3	chr10	18721581	18725137	-0.67	-0.06
Rnf217	chr10	31328190	31329531	-5.20	-0.76
Ube2d1	chr10	70718144	70718592	-2.63	0.38
Trpm2	chr10	77372533	77372804	-0.19	0.13
Pfkl	chr10	77450802	77450893	-0.32	-0.01
Rnf126	chr10	79221259	79223613	-5.73	-0.22
Rnf126	chr10	79229508	79229686	-0.71	-0.18
Fzr1	chr10	80829682	80830217	-4.88	0.37

Ckap4	chr10	83990030	83991369	-1.40	-0.09
Ckap4	chr10	83996275	83996633	-3.93	-0.77
Glipr1	chr10	111434202	111434291	-1.57	0.44
Cand1	chr10	118647900	118648919	-0.49	0.03
Irak3	chr10	119579458	119580238	-1.04	-0.13
Kif5a	chr10	126700029	126700180	-1.42	-0.60
Il23a	chr10	127733667	127733845	-0.28	-0.14
Osm	chr11	4136787	4140791	-321.00	-1.80
Lif	chr11	4166528	4170243	-65.90	-1.66
Lif	chr11	4171319	4172158	-36.30	-1.91
Ap1b1	chr11	4941834	4942343	-0.86	-0.12
Dbnl	chr11	5688485	5691845	-3.17	-0.48
Dbnl	chr11	5699963	5700503	-8.04	-0.43
Peli1	chr11	20991326	20991536	-0.58	0.15
Peli1	chr11	21047425	21048774	-11.20	-0.48
Psme4	chr11	30671774	30672135	-0.54	-0.18
Stk10	chr11	32433265	32433356	-0.44	0.16
Stk10	chr11	32489371	32503751	-45.30	-0.58
Stk10	chr11	32504289	32512777	-5.35	-0.46
Stk10	chr11	32517862	32522951	-27.20	-0.58
Fbxw11	chr11	32644536	32644986	-0.78	-0.08
Mapk9	chr11	49696697	49697026	-1.80	0.29
Rnf130	chr11	49933551	49933881	-0.38	0.41
Ppp2ca	chr11	51912325	51912474	-4.51	-0.88
Ppp2ca	chr11	51935449	51935836	-0.34	0.00
Kif3a	chr11	53380880	53380941	-0.99	-0.79
Gm2a	chr11	54923031	54923240	-0.86	0.16
Trim11	chr11	58791617	58791916	-2.16	-0.42
Trim11	chr11	58802336	58802515	-0.47	-0.11
Trim11	chr11	58802902	58804960	-1.42	-0.11
2310033P09Rik	chr11	59021980	59022040	-1.19	0.50
2310033P09Rik	chr11	59022524	59023941	-1.13	0.17
Nlrp3	chr11	59355086	59365364	-68.60	-0.49
Nlrp3	chr11	59379889	59380219	-0.60	0.09
Map2k3	chr11	60745558	60745738	-0.87	-0.13
Map2k3	chr11	60755435	60757081	-0.31	0.00
Map2k3	chr11	60765468	60766215	-11.10	-0.38
Vamp2	chr11	68902029	68902686	-0.49	-0.14
Pld2	chr11	70371013	70371612	-0.52	-0.09
Ube2g1	chr11	72420792	72421032	-3.90	-0.86
Ube2g1	chr11	72493612	72497295	-0.33	0.03
Crk	chr11	75492811	75517946	-16.70	-0.28
Crk	chr11	75518934	75519414	-1.35	-0.15

Blmh	chr11	76799996	76800265	-6.75	-0.58
Trim25	chr11	88860866	88861137	-34.20	-0.75
Trim25	chr11	88877093	88878370	-50.00	-0.45
Col1a1	chr11	94812810	94813020	-0.54	2.56
Psmd3	chr11	98543867	98546829	-30.30	-0.74
Csf3	chr11	98562626	98562686	-18.20	-2.81
Wipf2	chr11	98725061	98761613	-12.30	-0.40
Stat5a	chr11	100737560	100737770	-3.89	2.58
Stat5a	chr11	100737859	100738070	-4.30	2.42
Atp6v0a1	chr11	100923714	100924043	-0.91	-0.13
Psme3	chr11	101177564	101178336	-0.62	0.07
Psme3	chr11	101182070	101183288	-0.64	-0.06
Grn	chr11	102291725	102294419	-31.60	-0.59
Grn	chr11	102297674	102297824	-14.10	-0.23
Map3k3	chr11	106013617	106016298	-2.85	-0.22
Gaa	chr11	119129310	119131951	-7.63	-0.33
Baiap2	chr11	119867856	119868096	-3.26	-0.42
Nf2	chr11	4749020	4749260	-1.15	-0.24
Rel	chr11	23642262	23644451	-4.65	-0.27
Rel	chr11	23661118	23670970	-10.60	-0.81
Dock2	chr11	34127072	34128632	-0.68	0.05
Dock2	chr11	34546949	34597325	-2.05	-0.22
Cyfip2	chr11	46050850	46051031	-0.34	1.17
Cyfip2	chr11	46051479	46051569	-0.17	-0.26
Trim41	chr11	48620443	48622633	-2.33	-0.15
Trim41	chr11	48629367	48630893	-3.55	-0.21
Canx	chr11	50109707	50110776	-30.20	-0.37
Sec24a	chr11	51556985	51570140	-6.97	-0.33
Csf2	chr11	54060859	54061986	-0.80	-1.87
Atox1	chr11	55264107	55274640	-3.02	-0.32
Mapk7	chr11	61303417	61306635	-16.60	-0.51
Mapk7	chr11	61306963	61307441	-3.11	-0.52
Map2k4	chr11	65503265	65506970	-4.86	-0.31
Nlrp1b	chr11	70966603	70969541	-2.25	-0.41
Nlrp1b	chr11	70995132	70995372	-0.40	-0.12
Nlrp1b	chr11	70996028	70996268	-1.04	-0.49
Wsb1	chr11	79053599	79053839	-11.70	-0.39
Ube2z	chr11	95911494	95911704	-1.74	-0.23
Kpnb1	chr11	97023572	97023813	-3.10	-0.19
Kpnb1	chr11	97048995	97049206	-2.34	-0.30
Fbxl20	chr11	97950510	97953462	-0.74	0.16
Ormdl3	chr11	98443440	98443619	-1.16	0.44
Ormdl3	chr11	98443767	98444154	-2.37	-0.45

Klhl11	chr11	100323925	100325737	-6.64	0.31
Klhl11	chr11	100333798	100334096	-2.00	0.50
Ghdc	chr11	100630436	100630733	-0.74	-0.25
Dusp3	chr11	101834513	101835259	-0.43	0.02
Dusp3	chr11	101835527	101835885	-0.41	0.02
Dusp3	chr11	101835944	101836213	-0.85	-0.10
Map3k14	chr11	103081077	103082247	-3.53	-0.47
Map3k14	chr11	103100346	103101055	-0.68	-0.14
Map3k14	chr11	103103466	103128715	-0.39	-0.09
Cdc27	chr11	104366175	104368590	-6.73	0.30
Cd79b	chr11	106172684	106173355	-0.96	2.28
Icam2	chr11	106239145	106239263	-0.38	0.20
Smurf2	chr11	106683743	106684388	-0.37	-0.04
Smurf2	chr11	106781609	106781789	-3.23	-1.30
Cd300lb	chr11	114784364	114784935	-3.81	-0.24
Cd300lb	chr11	114784994	114795700	-23.20	-0.42
Cd300e	chr11	114914339	114914460	-0.23	0.05
Grb2	chr11	115505517	115505906	-1.13	0.17
Grb2	chr11	115506591	115507248	-1.12	-0.07
Ube2o	chr11	116399633	116399872	-0.36	0.05
Ube2o	chr11	116400110	116404120	-7.85	-0.32
Ube2o	chr11	116405875	116406055	-1.26	0.30
Ube2o	chr11	116442641	116442761	-0.21	0.26
Tmc6	chr11	117627298	117628154	-2.06	-0.44
Socs3	chr11	117827669	117830291	-196.00	-0.95
Cant1	chr11	118268378	118271504	-1.27	-0.10
Cant1	chr11	118272131	118279176	-4.34	-0.34
P4hb	chr11	120421617	120422006	-31.00	-0.33
P4hb	chr11	120422453	120422573	-21.50	-0.34
Kif3c	chr12	3365310	3367524	-6.08	-0.42
Kif3c	chr12	3403293	3403444	-1.82	-0.55
Kif3c	chr12	3404400	3404580	-3.67	0.79
Kif3c	chr12	3405446	3405956	-3.69	0.36
Sdc1	chr12	8778201	8778442	-0.29	-0.04
Sdc1	chr12	8798515	8799355	-3.14	-0.55
Lrr1	chr12	70275442	70275972	-0.48	-0.36
Psen1	chr12	85072679	85075730	-7.32	-0.20
Fos	chr12	86814850	86817711	-87.60	-0.74
Psmc1	chr12	101348377	101351316	-1.50	-0.23
Calm1	chr12	101437750	101440606	-16.30	-0.45
Calm1	chr12	101445239	101445449	-3.24	-0.14
Dync1h1	chr12	111839634	111839815	-0.41	-0.05
Dync1h1	chr12	111855934	111857506	-0.43	-0.01

Dync1h1	chr12	111904592	111905154	-1.97	-0.13
Traf3	chr12	112498880	112500724	-1.28	0.14
Klc1	chr12	112997059	113010574	-0.30	-0.01
Pld4	chr12	113998865	113999998	-0.67	0.14
Pld4	chr12	114000237	114001663	-1.26	-0.24
Pld4	chr12	114006322	114007047	-12.40	-0.38
Btbd6	chr12	114215520	114217031	-10.90	-0.80
Colec11	chr12	29279478	29279597	-0.39	1.91
Nfkbia	chr12	56593395	56593604	-103.00	-1.36
Sos2	chr12	70685554	70689696	-1.22	0.08
Sos2	chr12	70717850	70719054	-1.36	0.14
Sos2	chr12	70782599	70782839	-0.78	-0.61
Ppp2r5e	chr12	76554410	76554710	-5.69	0.49
Ppp2r5e	chr12	76594419	76616258	-1.73	0.31
Ppp2r5e	chr12	76616527	76694287	-1.42	-0.18
Ppp2r5e	chr12	76696767	76697187	-1.88	-0.25
Atp6v1d	chr12	79962444	79962625	-1.13	0.27
Npc2	chr12	86097961	86098142	-28.50	-0.41
Arel1	chr12	86259755	86261648	-4.64	-0.19
Arel1	chr12	86311686	86311836	-0.86	0.32
Rps6ka5	chr12	101789211	101791305	-0.71	0.25
Lgmn	chr12	103651753	103677907	-27.90	-0.55
Hsp90aa1	chr12	111933947	111934546	-6.23	2.48
Akt1	chr12	113912217	113912487	-1.97	-0.56
Elmo1	chr13	20684291	20697406	-1.26	0.14
Hist1h3h	chr13	21809496	21809698	-1.42	0.30
Hist1h3f	chr13	23636303	23636393	-2.07	-0.34
Hist1h3d	chr13	23667608	23667786	-1.12	-0.25
Ripk1	chr13	34094265	34094762	-9.02	-0.95
Ripk1	chr13	34119794	34125274	-0.32	0.00
Ripk1	chr13	34125393	34126082	-9.17	-0.86
Dsp	chr13	38283950	38285120	-2.85	3.39
Dsp	chr13	38285209	38285510	-0.30	-0.82
Dsp	chr13	38286818	38287269	-5.14	3.79
Dsp	chr13	38287568	38288078	-1.88	2.14
Dsp	chr13	38288227	38288738	-0.92	2.25
Dsp	chr13	38288946	38289187	-0.63	3.09
Rnf182	chr13	43763214	43763781	-1.24	5.46
Mylip	chr13	45485140	45499872	-7.91	0.67
Mylip	chr13	45503785	45506564	-28.20	0.99
Rnf144b	chr13	47338305	47340700	-1.08	-0.22
Rnf144b	chr13	47342881	47343181	-0.33	-0.08
Syk	chr13	52678805	52706632	-13.30	-0.48

Syk	chr13	52727069	52733575	-2.37	-0.22
Syk	chr13	52737300	52738605	-0.64	0.10
Syk	chr13	52738964	52742902	-5.19	-0.18
Syk	chr13	52743770	52743981	-0.44	0.06
Faf2	chr13	54723144	54730781	-0.85	0.35
Faf2	chr13	54757693	54763748	-1.55	-0.12
Lpcat1	chr13	73651326	73651986	-0.31	-0.02
Erap1	chr13	74777319	74784015	-3.77	0.34
Arsb	chr13	94710665	94710756	-0.36	-0.06
Cd180	chr13	103494954	103496711	-2.85	0.12
Il6st	chr13	113254307	113255963	-0.83	-0.24
Il6st	chr13	113294105	113296021	-3.87	0.19
Il6st	chr13	113296618	113296739	-0.70	-0.20
Cdk13	chr13	17809653	17811567	-1.53	0.10
Cdk13	chr13	17858358	17896931	-3.76	-0.17
Hist1h3i	chr13	21875180	21875266	-0.41	0.06
Hist1h3c	chr13	23837329	23837390	-0.31	-0.03
Tubb2a	chr13	34166146	34168454	-10.90	0.67
Tubb2b	chr13	34219290	34219586	-1.55	1.29
Tubb2b	chr13	34219940	34221135	-0.88	0.43
Txndc5	chr13	38592141	38592649	-2.68	0.19
Txndc5	chr13	38593006	38595355	-10.40	-0.27
Txndc5	chr13	38620124	38620304	-1.02	0.19
Hk3	chr13	55107346	55107674	-8.50	-0.40
Rab24	chr13	55421264	55421501	-3.81	-0.46
Rab24	chr13	55422955	55423341	-4.54	-0.47
Dok3	chr13	55624595	55625457	-4.45	-0.17
Dok3	chr13	55628729	55629086	-0.69	0.07
Dok3	chr13	55629708	55629917	-4.20	-0.81
Ddx41	chr13	55637366	55637930	-0.75	0.12
Ctsl	chr13	64470393	64471614	-17.40	-0.32
Scamp1	chr13	94973800	94978130	-0.65	0.12
Pik3r1	chr13	102453986	102456264	-3.37	0.30
Pik3r1	chr13	102478932	102525656	-0.37	-0.05
Pik3r1	chr13	102527244	102538172	-0.72	0.07
Map3k1	chr13	112538703	112540665	-0.67	0.09
Map3k1	chr13	112545624	112546521	-2.16	-0.18
Map3k1	chr13	112566138	112599191	-3.70	-0.70
Flnb	chr14	8650500	8650621	-0.79	0.25
Flnb	chr14	8737110	8739780	-5.32	-0.21
Flnb	chr14	8782901	8784101	-1.39	-0.09
Kctd6	chr14	9052713	9055575	-1.94	-0.30
Nkiras1	chr14	19111014	19112745	-0.47	-0.11

Sec24c	chr14	21512428	21513175	-11.10	-0.51
Sec24c	chr14	21513593	21514072	-11.40	-0.48
Plau	chr14	21661560	21662161	-4.06	-0.22
Vcl	chr14	21850890	21851310	-6.08	0.44
Psmc6	chr14	45949498	45950514	-0.60	-0.09
Pnp	chr14	51564155	51567533	-1.16	0.41
Pnp	chr14	51571126	51571721	-7.21	-0.33
Irf9	chr14	56222821	56223460	-0.30	0.02
Irf9	chr14	56223519	56224000	-1.25	-0.42
Irf9	chr14	56227759	56228328	-0.79	0.10
Ctsb	chr14	63761159	63761760	-164.00	-0.25
Clu	chr14	66587437	66590689	-4.00	-0.56
Kbtbd7	chr14	79827156	79828746	-2.42	0.38
Ppp3cb	chr14	21320302	21327131	-0.50	-0.04
Ppp3cb	chr14	21365614	21365735	-0.90	-0.41
Camk2g	chr14	21554783	21555022	-0.76	0.27
Camk2g	chr14	21555081	21555948	-2.05	-0.26
Prkcd	chr14	31408808	31411699	-1.85	-0.13
Prkcd	chr14	31411787	31411937	-0.73	0.22
Prkcd	chr14	31439273	31439394	-1.07	-0.33
Tspan14	chr14	41720841	41721322	-8.13	-0.95
Ripk3	chr14	56403831	56404011	-3.56	0.89
Ptk2b	chr14	66772153	66773020	-20.90	-0.52
Fbxl3	chr14	103482090	103488631	-0.87	-0.07
Fbxl3	chr14	103491640	103498113	-0.65	0.09
Fbxl3	chr14	103498172	103498726	-0.67	-0.17
Fyb	chr15	6529870	6569639	-15.30	-0.34
Fyb	chr15	6584885	6590459	-12.30	-0.75
Rictor	chr15	6736456	6737266	-1.44	-0.13
Rictor	chr15	6745958	6747284	-0.93	0.10
Myo10	chr15	25552304	25552784	-84.80	-1.10
Myo10	chr15	25709521	25712766	-107.00	-0.68
Myo10	chr15	25736823	25740210	-170.00	-1.17
Myo10	chr15	25740299	25742438	-33.40	-0.63
Myo10	chr15	25743096	25743426	-6.82	-0.51
Ncf4	chr15	78092626	78092951	-7.16	-0.30
Csf2rb	chr15	78156456	78166828	-4.16	-0.44
Csf2rb	chr15	78177155	78179733	-19.50	-0.49
Tab1	chr15	79989230	79992132	-13.10	-0.63
Rbx1	chr15	81305971	81306061	-1.28	-0.57
Ep300	chr15	81416643	81438767	-12.50	-0.29
Ep300	chr15	81479273	81481819	-5.01	-0.13
Xrcc6	chr15	81870214	81870424	-0.38	-0.05

Ano6	chr15	95621303	95661131	-0.52	-0.10
Ano6	chr15	95802935	95805002	-3.51	0.15
Tuba1c	chr15	98868116	98868326	-32.90	-0.97
Atf1	chr15	100084825	100090754	-1.03	0.09
Mettl7a1	chr15	100143523	100143732	-0.46	0.33
Nckap1l	chr15	103328091	103328272	-7.79	-0.50
Skp2	chr15	9043329	9043510	-0.95	0.41
Skp2	chr15	9069207	9070137	-7.83	-1.04
Il7r	chr15	9436363	9437293	-4.34	0.79
Il7r	chr15	9437531	9439923	-4.04	0.79
Rnf19a	chr15	36170946	36173866	-1.80	-0.21
Rnf19a	chr15	36195033	36212902	-6.05	0.56
Ywhaz	chr15	36723285	36723376	-0.55	0.11
Fbxo32	chr15	58011902	58012112	-0.40	-0.84
Fbxo32	chr15	58012410	58013039	-1.36	-1.07
Fbxo32	chr15	58046223	58046433	-0.22	-0.12
Ptk2	chr15	73035594	73039802	-1.03	-0.09
Dgat1	chr15	76342128	76342248	-1.28	-0.41
Myh9	chr15	77592006	77593541	-15.60	-0.23
Myh9	chr15	77594338	77606283	-281.00	-0.43
Myh9	chr15	77643609	77672545	-2.53	-0.16
Csf2rb2	chr15	78112999	78113060	-1.58	0.68
Csf2rb2	chr15	78114558	78116509	-0.74	0.08
Csf2rb2	chr15	78127494	78136033	-1.82	-0.32
Pla2g6	chr15	79116836	79117254	-2.27	-0.40
Nfam1	chr15	82828225	82828854	-0.48	-0.04
Nfam1	chr15	82829422	82831880	-7.67	-0.36
Arsa	chr15	89303025	89303862	-0.98	-0.16
Arsa	chr15	89305988	89306615	-6.67	-0.79
Col2a1	chr15	97806241	97806600	-2.18	2.47
Asb8	chr15	97965969	97971744	-1.49	-0.17
Racgap1	chr15	99470224	99481962	-0.78	0.26
Itgb7	chr15	102046603	102046986	-2.10	-0.17
Itgb7	chr15	102057997	102062366	-0.74	-0.15
Gpr84	chr15	103138665	103140869	-8.09	-0.21
Hmox2	chr16	4764613	4765838	-8.41	-0.34
Mgrn1	chr16	4936923	4937342	-0.32	-0.01
Mapk1	chr16	17038671	17038882	-0.49	0.07
Snap29	chr16	17427202	17428532	-2.76	0.33
Crkl	chr16	17452169	17484582	-4.45	-0.19
Klhl22	chr16	17771956	17777358	-0.41	-0.03
Ap2m1	chr16	20535576	20537801	-1.17	-0.35
Psmd2	chr16	20651724	20652324	-20.60	-0.49

Eif4g1	chr16	20672821	20673965	-1.18	-0.11
Eif4g1	chr16	20674380	20675449	-1.23	-0.13
Eif4g1	chr16	20679335	20680055	-13.00	-0.18
Eif4g1	chr16	20689353	20692866	-16.70	-0.31
Il1rap	chr16	26714898	26715319	-4.11	0.91
Itgb5	chr16	33829750	33830079	-4.10	-1.27
Itgb5	chr16	33919922	33920221	-1.38	-0.29
Itgb5	chr16	33947843	33949245	-1.75	-0.23
Cd80	chr16	38473975	38479185	-8.20	-0.52
Cd80	chr16	38486386	38486895	-4.13	-0.46
Cd80	chr16	38487013	38494806	-2.19	0.67
Cd200r1	chr16	44794312	44794552	-1.93	-0.75
Cd200r4	chr16	44811897	44820900	-2.09	-1.18
Cd200r2	chr16	44915300	44915864	-0.30	-0.12
Cd47	chr16	49855915	49868147	-3.21	0.29
Cblb	chr16	52204625	52205075	-5.73	-0.89
Ifnar2	chr16	91399551	91405652	-2.13	-0.16
Il10rb	chr16	91425363	91425632	-3.68	-0.33
Ifnar1	chr16	91503665	91506361	-3.40	-0.24
Ifnar1	chr16	91506810	91506901	-0.62	0.22
Ifnar1	chr16	91507170	91507680	-1.40	-0.22
Ifngr2	chr16	91563142	91563682	-0.51	-0.03
Crebbp	chr16	4084047	4087773	-0.48	-0.02
Crebbp	chr16	4154961	4213404	-3.27	-0.16
Socs1	chr16	10783990	10784912	-0.46	-0.26
Socs1	chr16	10784970	10785629	-0.59	-1.60
Cpped1	chr16	11804560	11827393	-0.86	0.10
Cpped1	chr16	11887837	11909516	-2.45	-0.79
Pak2	chr16	32019862	32021909	-0.84	0.05
Dtx3l	chr16	35928994	35932076	-0.66	-0.06
Dtx3l	chr16	35932345	35933542	-0.72	-0.06
Cd86	chr16	36604253	36604522	-0.59	-0.66
App	chr16	85025115	85043833	-16.90	-0.31
App	chr16	85120907	85173892	-5.77	-0.46
Ltn1	chr16	87415807	87416587	-1.51	0.15
Brwd1	chr16	96214898	96214959	-2.85	1.53
Brwd1	chr16	96223575	96225521	-4.61	-0.24
Brwd1	chr16	96226300	96226615	-0.56	-1.29
Rps6ka2	chr17	7374493	7374614	-0.54	0.42
Rps6ka2	chr17	7506884	7507544	-0.43	0.14
1600012H06Rik	chr17	15080218	15081387	-18.40	-0.69
Itpr3	chr17	27258568	27258749	-1.96	-0.56
Mapk14	chr17	28828316	28828704	-3.84	-0.61

Mapk14	chr17	28884007	28885350	-1.27	0.08
Tapbp	chr17	34056422	34056928	-18.20	-0.66
Tapbp	chr17	34062443	34063061	-65.40	-0.74
H2-DMa	chr17	34272232	34272620	-0.59	0.23
Tap1	chr17	34324500	34325221	-16.90	-0.87
Tap1	chr17	34333599	34333840	-0.55	-0.11
Psmb8	chr17	34335288	34335468	-2.22	-0.33
Tap2	chr17	34341453	34342537	-9.79	-0.80
Tap2	chr17	34352342	34353206	-4.14	-0.42
Neu1	chr17	35071556	35072840	-6.58	-0.28
Ltb	chr17	35331600	35332385	-11.70	-0.51
Ltb	chr17	35332444	35333220	-19.10	-0.52
H2-D1	chr17	35400038	35400558	-64.10	-0.68
H2-D1	chr17	35400926	35402744	-4.71	-0.33
H2-D1	chr17	35403816	35404204	-71.80	-0.36
H2-Q2	chr17	35479307	35479570	-0.14	0.42
H2-Q6	chr17	35561821	35562745	-5.87	-0.37
H2-Q6	chr17	35565949	35566720	-1.19	-0.34
H2-Q7	chr17	35576925	35577015	-0.36	-0.83
H2-Q10	chr17	35607880	35610039	-0.46	-0.18
Nfkbie	chr17	45692664	45693263	-1.50	-0.23
Nfkbie	chr17	45697537	45700027	-7.33	-0.49
Cul7	chr17	46788096	46788277	-2.58	3.86
Cul7	chr17	46788516	46788967	-0.83	-0.64
Cul7	chr17	46791019	46791200	-0.63	1.18
Trem1	chr17	48371921	48385080	-1.75	0.78
Treml2	chr17	48439293	48439684	-0.27	0.22
Treml2	chr17	48449070	48449580	-0.73	-0.24
Ebi3	chr17	56095665	56096445	-4.37	-0.35
Tnfsf9	chr17	57244807	57244958	-0.73	-0.14
Tnfsf9	chr17	57245676	57247180	-1.89	-0.10
Vav1	chr17	57418522	57418613	-0.31	0.06
Vav1	chr17	57467106	57467554	-1.28	0.13
Ypel5	chr17	73198557	73198888	-0.87	-0.22
Rasgrp3	chr17	75924352	75926570	-0.88	0.08
Rasgrp3	chr17	75928033	75928214	-0.97	-0.23
Ppm1b	chr17	85357671	85364400	-0.33	0.09
Ppm1b	chr17	85373436	85402617	-0.40	-0.02
Ppm1b	chr17	85405048	85413253	-7.19	-0.91
Prkce	chr17	86567124	86568262	-0.54	0.09
Prkce	chr17	87053997	87055374	-0.74	-0.16
Socs5	chr17	87507227	87507318	-0.46	-0.23
Socs5	chr17	87520106	87534863	-0.79	0.07

Dynlt1f	chr17	6306339	6306549	-0.61	0.48
Rnaset2b	chr17	8338409	8340697	-14.10	-0.91
Igf2r	chr17	12876409	12876980	-0.50	-0.04
Igf2r	chr17	12962451	12962572	-0.53	0.18
Lnpep	chr17	17715497	17746283	-1.10	-0.05
Fpr1	chr17	18013552	18013876	-0.16	-0.22
Fpr1	chr17	18014139	18020903	-0.38	1.24
Mmp25	chr17	23781787	23781878	-0.56	3.46
Mmp25	chr17	23782026	23782236	-0.61	2.96
Tnfrsf12a	chr17	23814327	23814416	-0.36	0.14
Pdpk1	chr17	24215553	24216093	-11.80	0.63
Atp6v0c	chr17	24306225	24306374	-1.17	-0.23
Ccnf	chr17	24360594	24360954	-5.87	0.78
Ccnf	chr17	24361042	24361933	-12.00	1.09
Mlst8	chr17	24612351	24613045	-0.59	-0.10
Stub1	chr17	25967580	25967909	-3.51	-0.27
Stub1	chr17	25969947	25970008	-0.31	-0.35
Stub1	chr17	25970096	25970306	-1.82	-0.63
H2-K1	chr17	34133105	34133495	-21.60	-0.51
H2-K1	chr17	34136728	34137188	-34.60	-0.71
Hspa1b	chr17	35093821	35096183	-6.05	0.37
Hspa1a	chr17	35106868	35108327	-2.67	3.71
Hspa1a	chr17	35108505	35108744	-1.59	6.01
Tnf	chr17	35336694	35337384	-434.00	-1.28
Tnf	chr17	35338760	35338941	-117.00	-1.09
Lta	chr17	35340403	35340580	-0.52	-0.46
Tubb5	chr17	35972171	35972588	-48.90	-0.35
H2-T23	chr17	36169189	36248130	-6.88	-0.55
Trim39	chr17	36397225	36398520	-0.49	0.05
Trim39	chr17	36400794	36404080	-0.93	-0.33
Enpp4	chr17	44234607	44235267	-0.53	0.24
Enpp4	chr17	44236165	44242757	-6.51	1.00
Klc4	chr17	46781254	46782971	-0.81	-0.28
Ppp2r5d	chr17	46820058	46820862	-0.90	-0.11
Ppp2r5d	chr17	46837029	46841951	-0.53	-0.11
Cnpy3	chr17	46872839	46874785	-3.02	-0.19
Ubr2	chr17	47065869	47067369	-3.79	-0.22
Ubr2	chr17	47147270	47147481	-5.21	-0.65
Ticam1	chr17	56408914	56416190	-37.60	-0.63
Tubb4a	chr17	57220052	57220528	-1.24	-0.37
Tubb4a	chr17	57220616	57221122	-1.23	-0.35
Tnfsf14	chr17	57329542	57330291	-0.18	0.06
Pja2	chr17	64658065	64658365	-1.01	-0.18

Pja2	chr17	64658514	64660528	-2.68	-0.35
Vapa	chr17	65962685	65962865	-2.72	-0.27
Rab31	chr17	66001065	66001632	-0.68	-0.05
Rab31	chr17	66002078	66002287	-2.81	-0.26
Rab31	chr17	66003418	66017257	-2.20	0.14
Rab31	chr17	66122031	66122092	-0.25	-0.17
Xdh	chr17	74233606	74233727	-0.41	-0.14
Eif2ak2	chr17	79251889	79252308	-0.49	0.06
Eif2ak2	chr17	79252515	79254306	-29.10	-0.53
Eif2ak2	chr17	79277606	79281912	-2.74	-0.48
Sos1	chr17	80797100	80799127	-0.43	-0.03
Sos1	chr17	80833039	80834290	-0.90	-0.18
Sos1	chr17	80879343	80879554	-1.15	-0.89
Calm2	chr17	87835011	87846216	-2.03	-0.14
Fbxo11	chr17	88390945	88391686	-0.95	0.09
Fbxo11	chr17	88464385	88464625	-1.02	-0.30
Colec12	chr18	9707645	9840283	-0.36	-0.01
Colec12	chr18	9848284	9859890	-0.96	-0.03
Colec12	chr18	9874892	9877425	-4.78	0.23
Ube2d2a	chr18	35931242	35931392	-3.18	-0.65
Ube2d2a	chr18	35965479	35965570	-0.43	0.27
Rnf14	chr18	38467690	38467990	-0.56	0.10
Dctn4	chr18	60686499	60686560	-0.25	-0.32
Dctn4	chr18	60716315	60716526	-1.59	0.32
Camk2a	chr18	61144547	61145295	-1.50	-0.78
Csf1r	chr18	61265501	61269298	-2.66	-0.20
Csf1r	chr18	61290043	61290284	-12.60	-0.40
Csf1r	chr18	61290463	61290793	-14.80	-0.34
Malt1	chr18	65635658	65636018	-2.72	-0.30
Tubb6	chr18	67550384	67552360	-1.76	-0.20
Tubb6	chr18	67552568	67561747	-29.40	-0.45
Mex3c	chr18	73732538	73732629	-1.20	1.10
Mex3c	chr18	73732718	73732899	-1.18	-2.10
Mex3c	chr18	73732988	73750582	-0.64	0.04
Map3k8	chr18	4348899	4352951	-3.59	-0.54
Kif5b	chr18	6241222	6241522	-8.22	-0.56
Rock1	chr18	10181251	10181790	-9.40	-0.56
Cdc23	chr18	34792901	34793291	-0.68	-0.16
Cdc23	chr18	34793470	34793621	-0.76	-0.25
Tmem173	chr18	35893809	35894258	-4.25	-0.40
Cd14	chr18	36884720	36886308	-168.00	-0.28
Trim36	chr18	46326839	46328996	-8.68	1.58
Ticam2	chr18	46718782	46719052	-0.87	-0.33

Ticam2	chr18	46719411	46732457	-0.64	0.08
Nfatc1	chr18	80802973	80803394	-0.79	-0.30
Nfatc1	chr18	80803872	80832717	-0.41	0.03
Nfatc1	chr18	80846232	80846653	-2.71	0.51
Nfatc1	chr18	80879052	80904894	-2.97	0.23
Unc93b1	chr19	3935215	3935306	-3.82	-0.42
Unc93b1	chr19	3936467	3936707	-9.44	-0.66
Unc93b1	chr19	3948801	3948951	-25.80	-0.61
Sptbn2	chr19	4744054	4745130	-1.41	-0.53
Rela	chr19	5646968	5647773	-3.54	-0.20
Jak2	chr19	29385493	29386732	-0.30	0.00
Jak2	chr19	29386881	29387450	-3.92	0.54
Cd274	chr19	29441927	29455003	-40.60	-1.29
Cd274	chr19	29456978	29460318	-29.60	-1.05
Pdcd1lg2	chr19	29545061	29545301	-0.32	0.93
Pten	chr19	32832126	32832995	-2.07	-0.13
Pten	chr19	32872591	32874607	-0.82	0.07
Pten	chr19	32892519	32894752	-15.20	0.55
Kif11	chr19	37494852	37495032	-4.82	-1.09
Pgam1	chr19	41986508	41990163	-6.96	-1.07
Btrc	chr19	45604117	45604298	-0.65	1.17
Nfkb2	chr19	46379436	46380290	-0.39	0.13
Fbxl15	chr19	46403649	46404936	-3.45	-0.46
Tcirg1	chr19	3906889	3906949	-2.38	-0.62
Peli3	chr19	4932033	4932629	-0.24	0.04
Klc2	chr19	5117426	5118318	-2.71	-0.53
Sipa1	chr19	5651184	5651544	-0.61	-0.07
Sipa1	chr19	5653976	5655148	-7.20	-0.43
Sipa1	chr19	5659601	5662646	-15.60	-0.49
Ppp2r5b	chr19	6227944	6228243	-1.38	-0.37
Stx3	chr19	11893652	11893893	-0.37	0.09
Dtx4	chr19	12543910	12544151	-8.93	-0.57
Dtx4	chr19	12560929	12576605	-3.81	-0.14
Pik3ap1	chr19	41348707	41357626	-14.30	-0.57
Pik3ap1	chr19	41406769	41459560	-89.20	-1.22
Chuk	chr19	44181817	44181937	-0.55	-0.16
Fbxw4	chr19	45652866	45653256	-0.75	-0.11
Actr1a	chr19	46452346	46452913	-2.60	-0.15
Csf2ra	chr19	61300453	61302211	-14.30	-0.30
Csf2ra	chr19	61302992	61304321	-7.97	-0.39
Ddx3x	chrX	12858147	12861347	-0.41	0.02
Ddx3x	chrX	12868399	12868809	-48.00	0.69
Il13ra1	chrX	33695553	33709728	-1.69	0.25

Pgrmc1	chrX	34138219	34144830	-27.20	0.84
Pgrmc1	chrX	34145392	34145600	-79.00	2.46
Ube2a	chrX	34414360	34414420	-1.18	-0.44
Tab3	chrX	82859477	82860195	-4.67	0.57
Tab3	chrX	82860522	82860673	-1.01	0.46
Tab3	chrX	82873570	82876044	-16.40	2.20
Kif4	chrX	97921893	97922371	-1.53	-0.53
Atp7a	chrX	103222674	103280870	-3.97	0.27
Atp7a	chrX	103319725	103319995	-12.90	0.99
Huwe1	chrX	148353194	148354606	-2.14	0.09
Was	chrX	7667496	7667617	-0.70	0.20
Rap2c	chrX	48359709	48370808	-2.49	0.22
Rap2c	chrX	48370867	48371195	-0.35	-0.02
Irak1	chrX	71260386	71260835	-0.46	0.04
Eda2r	chrX	94533994	94534384	-1.02	0.70
Pja1	chrX	96661699	96661909	-2.76	0.63
Pja1	chrX	96662087	96663013	-1.08	0.12
Pja1	chrX	96663072	96663789	-0.87	-0.14
Rlim	chrX	101156612	101157212	-16.70	0.50
Rlim	chrX	101157331	101160637	-88.80	0.50
Gla	chrX	131124431	131124610	-68.60	1.61
Gla	chrX	131135394	131135514	-0.61	0.21
Siah1b	chrX	160509638	160510081	-0.33	0.04
Tlr8	chrX	163680663	163681230	-0.44	0.52
Tlr8	chrX	163681497	163681647	-0.61	1.67
Tlr8	chrX	163681944	163682362	-0.65	0.76
Tlr8	chrX	163683254	163683672	-3.07	3.59
Tlr7	chrX	163742862	163745310	-7.00	0.16
Tlr7	chrX	163745428	163745549	-4.15	0.44
Tlr7	chrX	163745727	163746354	-0.94	0.06
Uba6	chr5	86541279	86547113	-0.45	0.04
Stim1	chr7	109416725	109416845	-0.32	0.12
Dynll2	chr11	87794645	87799923	-0.59	0.05
Dynll2	chr11	87800072	87801030	-0.39	-0.04
Diaph1	chr18	38004807	38005077	-2.74	-0.30
Diaph1	chr18	38094885	38095065	-2.39	-0.61

Generic transcription pathway

gene_name	chr	chromStart	chromEnd	diff.lg.p	diff.log2.fc
Cnot11	chr1	39603302	39603632	-9.27	-1.03
Cnot9	chr1	74575438	74575798	-1.13	-0.18
Ing5	chr1	95713260	95715951	-3.10	0.35
Ccnt2	chr1	129698247	129700591	-5.04	0.20
Rbbp5	chr1	134394620	134400982	-0.63	-0.07

Rbbp5	chr1	134401101	134401282	-1.08	0.24
Kdm5b	chr1	136514466	136514617	-0.28	0.01
Kdm5b	chr1	136521370	136521700	-0.83	-0.21
Kdm5b	chr1	136525318	136527418	-2.33	-0.37
Kdm5b	chr1	136527777	136528227	-0.52	0.09
Nr1i3	chr1	173148035	173148455	-0.57	-0.14
Nr1i3	chr1	173148844	173149204	-1.85	-0.52
Exo1	chr1	177829710	177831466	-2.00	-0.72
Exo1	chr1	177839222	177839403	-0.50	-2.10
Parp1	chr1	182499105	182503813	-1.96	-0.45
Trp53bp2	chr1	184371133	184371894	-0.32	-0.03
Trp53bp2	chr1	184376642	184379234	-2.02	-0.28
Trp53bp2	chr1	184391818	184392328	-2.00	-0.40
Gls	chr1	52244535	52244686	-10.30	0.83
Gls	chr1	52276864	52290076	-11.90	-0.48
Sumo1	chr1	59696455	59697048	-0.33	-0.01
Bard1	chr1	71121361	71122020	-1.25	-0.38
Hdac4	chr1	93829331	93829960	-0.46	0.09
Steap3	chr1	122123232	122123862	-3.78	-0.81
Steap3	chr1	122140355	122166591	-6.06	-1.12
Mdm4	chr1	134888236	134893148	-1.07	-0.08
Btg2	chr1	135973565	135975732	-1.48	0.08
Rnf2	chr1	153318438	153320426	-0.40	0.03
Smyd2	chr1	191704578	191704728	-0.49	0.06
Bmi1	chr2	18605667	18606468	-0.37	-0.01
Rxra	chr2	27615158	27615398	-3.09	-0.87
Rxra	chr2	27615487	27616386	-13.50	-1.14
Rxra	chr2	27617342	27618510	-7.46	-0.74
Tsc1	chr2	28531519	28532030	-4.79	-0.58
Tsc1	chr2	28541313	28542943	-0.95	-0.12
Abl1	chr2	31544075	31551290	-7.77	-0.43
Abl1	chr2	31653102	31658189	-1.51	-0.10
Abl1	chr2	31658367	31658428	-0.69	-0.40
Mapkap1	chr2	34479093	34479423	-0.34	0.02
Ssrp1	chr2	84885611	84887238	-5.04	-0.18
Tnks1bp1	chr2	84891036	84904146	-4.25	-0.23
Psmc3	chr2	90894439	90895182	-3.33	-0.20
Lmo2	chr2	103809861	103821531	-3.10	-0.19
Mga	chr2	119698286	119698347	-0.21	0.26
Mga	chr2	119722990	119743194	-1.74	0.09
Mga	chr2	119749246	119752139	-1.06	0.11
Mga	chr2	119767083	119767563	-7.58	-0.49
Mga	chr2	119772610	119774079	-2.10	0.12

Mga	chr2	119787692	119790793	-0.32	0.01
Csnk2a1	chr2	152107019	152107169	-10.00	-0.77
Tpx2	chr2	152717983	152719804	-0.61	0.08
Itch	chr2	154959216	154959307	-0.27	-0.02
Itch	chr2	155050102	155050403	-0.31	-0.01
Phf20	chr2	156093020	156093171	-0.35	-0.03
Phf20	chr2	156124429	156134043	-2.43	-0.18
Tgif2	chr2	156679337	156679783	-0.33	-0.07
Src	chr2	157295136	157296531	-2.35	0.70
L3mbtl1	chr2	162773851	162773971	-0.35	1.56
Ywhab	chr2	163820932	163837497	-10.90	-0.39
Trp53rkb	chr2	166619352	166619593	-0.34	0.09
Trp53rkb	chr2	166620885	166621755	-0.75	-0.18
Gata3	chr2	9779597	9784722	-2.88	0.70
Gata3	chr2	9790440	9796250	-0.57	-0.22
Taf3	chr2	9837317	9874531	-2.60	-0.16
Pip4k2a	chr2	18765527	18769115	-4.26	0.31
Ehmt1	chr2	24646742	24647852	-3.96	-0.36
Ehmt1	chr2	24733063	24775080	-0.58	-0.06
Notch1	chr2	26314079	26314319	-0.24	0.00
Notch1	chr2	26315305	26315396	-0.28	0.42
Notch1	chr2	26315993	26316443	-0.58	-0.38
Cdk9	chr2	32562910	32565131	-22.70	-0.34
Psmd5	chr2	34707993	34709823	-1.65	-0.19
Psmd5	chr2	34726274	34726482	-4.41	-0.82
Psmb7	chr2	38498946	38499426	-3.13	-0.36
Nr6a1	chr2	38740753	38781783	-1.41	1.01
Nr4a2	chr2	56960145	56961485	-4.91	-0.57
Nr4a2	chr2	56964219	56965507	-14.90	-0.92
Nr4a2	chr2	56967144	56976354	-1.04	0.68
Pin1rt1	chr2	104554491	104554756	-2.79	3.80
Zfp770	chr2	114021670	114027138	-1.72	0.21
Zfp661	chr2	127402341	127403416	-0.33	0.03
Pcna	chr2	132077710	132078916	-7.08	0.28
Psmf1	chr2	151543111	151543202	-1.31	-0.87
Psmf1	chr2	151543649	151544038	-0.30	0.01
Zfp334	chr2	165203384	165203715	-0.29	0.04
Zfp334	chr2	165205241	165206231	-1.16	0.28
Zfp334	chr2	165207038	165207279	-0.43	0.16
Trp53rka	chr2	165315878	165318814	-1.44	-0.19
Aurka	chr2	172182168	172182683	-0.29	0.00
Taf4	chr2	179647388	179656678	-2.00	-0.23
Taf4	chr2	179710752	179710813	-0.34	0.87

Skil	chr3	30994042	30997412	-0.91	-0.07
Skil	chr3	31015852	31017641	-3.54	-0.30
Gatad2b	chr3	90127990	90145879	-1.31	-0.15
Gatad2b	chr3	90155787	90155908	-1.99	-0.28
Hist2h2be	chr3	96025043	96025490	-2.37	-0.78
Hist2h2be	chr3	96026708	96027215	-0.64	-0.19
Hist2h2be	chr3	96027422	96027483	-10.00	3.73
Notch2	chr3	97817460	97817641	-2.65	-0.57
Notch2	chr3	97938527	97941254	-16.60	-0.46
Notch2	chr3	97950009	97954290	-14.00	-0.14
Trim33	chr3	103148225	103148495	-2.77	0.27
Trim33	chr3	103156043	103158233	-4.71	0.28
Trim33	chr3	103158830	103159400	-0.41	-0.03
Psma5	chr3	108059873	108060022	-0.64	3.39
Ube2d3	chr3	135101656	135102228	-19.60	-0.75
Phc3	chr3	30805887	30813151	-0.72	-0.12
Phc3	chr3	30864733	30868337	-1.81	-0.73
Ccna2	chr3	36470827	36470918	-0.54	-0.57
Elf2	chr3	51059675	51060839	-1.69	0.13
Elf2	chr3	51061545	51064994	-11.20	0.72
Maml3	chr3	51491863	51493302	-3.62	0.65
Maml3	chr3	51493600	51496200	-1.82	0.41
Maml3	chr3	51501693	51600338	-0.20	0.07
Maml3	chr3	51659510	51660829	-9.25	0.71
Maml3	chr3	51907999	51908179	-1.49	0.85
Maml3	chr3	51908418	51908808	-2.19	-0.81
Lamtor2	chr3	88353913	88356849	-9.31	-0.34
Psmb4	chr3	94690701	94690880	-8.50	-0.41
Psmd4	chr3	94836626	94836894	-2.54	-0.16
Psmd4	chr3	94840526	94846467	-8.21	-0.35
Hist2h3c2	chr3	96042365	96042604	-1.05	0.63
Hipk1	chr3	103547180	103548289	-2.19	-0.08
Hipk1	chr3	103581236	103595486	-33.60	-0.28
Prmt6	chr3	110050616	110051517	-0.57	-0.23
Prmt6	chr3	110052356	110053916	-1.37	-0.11
Trp53inp1	chr4	11083587	11092710	-11.10	0.96
Trp53inp1	chr4	11095044	11097120	-9.82	1.10
Nbn	chr4	15906479	15911111	-1.10	0.17
Nr4a3	chr4	48058221	48058400	-1.45	-1.08
Nr4a3	chr4	48064136	48065027	-8.33	-1.10
Nr4a3	chr4	48080626	48096224	-0.76	0.49
Zfp189	chr4	49535279	49543489	-4.61	0.49
Rraga	chr4	86221606	86222293	-1.18	-0.09

Tal1	chr4	114737293	114741792	-0.36	0.03
Tal1	chr4	114741970	114742091	-0.38	-0.13
Med8	chr4	118087440	118087589	-1.21	0.21
Rragc	chr4	123598193	123598284	-0.30	-0.01
Rragc	chr4	123612802	123613851	-0.52	0.02
Phc2	chr4	128404910	128405061	-0.82	-0.46
Phc2	chr4	128424470	128429198	-18.30	-0.40
Phc2	chr4	128429466	128429976	-16.20	-0.47
Rpa2	chr4	132333826	132334035	-2.59	-0.74
Nr0b2	chr4	133112127	133112392	-0.35	0.24
Zfp46	chr4	135846237	135848601	-1.27	0.14
Mtor	chr4	147822690	147822781	-3.13	-1.30
Mtor	chr4	147930392	147931764	-3.53	-0.34
Zfp691	chr4	118842631	118845741	-0.75	0.13
Zfp691	chr4	118845980	118846800	-9.76	2.33
Zfp69	chr4	120602801	120603819	-1.72	0.55
Tfap2e	chr4	126393574	126393783	-1.59	2.55
Sesn2	chr4	132048721	132052822	-3.34	-0.16
Sesn2	chr4	132066221	132066371	-3.98	-0.48
Sesn2	chr4	132066234	132066414	-3.65	-0.47
Sfn	chr4	133156530	133157635	-3.71	-1.00
Sfn	chr4	133157694	133158083	-0.48	0.30
Arid1a	chr4	133235940	133241652	-2.63	-0.08
Arid1a	chr4	133276907	133312385	-11.90	-0.45
Eloa	chr4	135561281	135561551	-1.02	-0.17
Eloa	chr4	135565701	135577564	-4.34	-0.14
Ski	chr4	154529795	154533593	-10.40	0.28
Ski	chr4	154533945	154596644	-10.70	-0.37
Cdk6	chr5	3519406	3520884	-1.33	-0.15
Kmt2e	chr5	22940246	22940516	-2.58	0.46
Kmt2e	chr5	22998122	22999338	-0.91	0.13
Kmt2e	chr5	23002790	23005303	-1.02	0.08
Kmt2e	chr5	23007352	23008999	-1.84	0.10
Ywhah	chr5	33361494	33361614	-1.23	0.51
Ywhah	chr5	33369299	33370077	-1.30	-0.06
Rbpj	chr5	53981538	53981866	-29.80	-1.15
Rbpj	chr5	54044845	54045026	-0.27	-0.52
Zfp605	chr5	110539457	110539517	-0.19	1.00
Zfp605	chr5	110556601	110558156	-4.58	0.65
Triap1	chr5	115791255	115793441	-0.39	-0.02
Rnf34	chr5	123273869	123314504	-0.81	0.09
Psmd9	chr5	123678228	123678437	-0.34	0.03
Kmt5a	chr5	124909965	124910818	-10.10	-0.42

Kmt5a	chr5	124911146	124911447	-1.43	0.14
Gtf2h3	chr5	125046192	125046313	-0.64	-0.25
Zfp664	chr5	125343755	125366362	-1.00	-0.09
Zfp664	chr5	125366751	125367081	-0.42	-0.06
Rfc2	chr5	135073840	135074198	-2.30	-0.39
Zkscan1	chr5	138526311	138534016	-1.05	-0.35
Zkscan1	chr5	138534105	138534645	-16.30	-1.03
Zkscan1	chr5	138541914	138542485	-0.49	-0.05
Zkscan1	chr5	138542723	138543984	-3.46	0.27
Zkscan1	chr5	138544222	138545962	-1.19	-0.12
Zfp12	chr5	144005811	144008390	-3.66	0.31
Zfp12	chr5	144008839	144009619	-0.78	-0.19
Zkscan5	chr5	145979715	145981274	-1.73	0.54
Zkscan5	chr5	145981632	145981812	-0.41	-0.19
Zkscan5	chr5	145981871	145982380	-1.47	0.57
Zfp655	chr5	145992583	145994463	-2.98	-0.51
Zfp655	chr5	146004460	146006138	-2.46	0.12
Zscan25	chr5	146044360	146052189	-9.62	-0.48
Cdk8	chr5	147042885	147071089	-9.50	-1.16
Smarcd3	chr5	24100691	24100989	-0.59	-0.63
Prkag2	chr5	24369540	24369661	-0.30	-0.04
Prkag2	chr5	24527783	24527844	-5.60	1.58
Prkag2	chr5	24534296	24606370	-0.56	0.08
Kmt2c	chr5	24790138	24790949	-2.35	-0.28
Kmt2c	chr5	24804711	24805371	-6.01	0.48
Kmt2c	chr5	24805490	24806151	-3.45	0.35
Kmt2c	chr5	24815764	24816754	-4.22	0.34
Kmt2c	chr5	24819800	24820700	-2.61	0.23
Kmt2c	chr5	24820849	24821690	-2.45	0.22
Kmt2c	chr5	24878616	24880356	-8.08	0.57
Prkab1	chr5	116463747	116467999	-0.85	-0.08
Prkab1	chr5	116474139	116474407	-0.44	-0.06
Ptpn11	chr5	121581827	121582307	-1.21	0.16
Ptpn11	chr5	121583292	121587519	-0.40	0.02
Ncor2	chr5	125497524	125500255	-17.90	-0.42
Ncor2	chr5	125504014	125509323	-4.66	-0.22
Ncor2	chr5	125517648	125517739	-5.60	-1.05
Ncor2	chr5	125518279	125531358	-10.10	-0.27
Ncor2	chr5	125538304	125548306	-0.58	0.07
Ncor2	chr5	125659493	125659584	-0.45	-0.84
Ubc	chr5	125866453	125867671	-172.00	0.73
Ubc	chr5	125867938	125868147	-3.15	0.20
Ubc	chr5	125868235	125870298	-22.80	-0.40

Zfp11	chr5	130162143	130162980	-1.61	0.64
Ywhag	chr5	136384965	136385384	-2.91	-0.09
Ywhag	chr5	136386428	136406621	-96.80	-0.35
Ywhag	chr5	136410241	136410481	-13.00	-0.40
Zfp113	chr5	138585481	138586141	-3.89	-0.70
Zfp113	chr5	138586529	138586680	-0.41	0.19
Taf6	chr5	138619844	138620232	-4.14	-0.57
Smurf1	chr5	145637663	145638682	-0.50	0.08
Smurf1	chr5	145639310	145639521	-0.66	0.35
Zkscan14	chr5	145955991	145962485	-2.97	-0.45
Rfc3	chr5	152445398	152445638	-0.43	0.10
Cul1	chr6	47404345	47445165	-3.56	0.31
Cul1	chr6	47474617	47476108	-1.64	-0.15
Zfp398	chr6	47785899	47790681	-0.56	-0.29
Zfp398	chr6	47815976	47817057	-5.16	-0.83
Zfp282	chr6	47827763	47830376	-1.26	-0.29
Zfp282	chr6	47854827	47855966	-11.40	-0.77
Zfp282	chr6	47856175	47856835	-0.42	-0.06
Zfp212	chr6	47870625	47876740	-0.95	-0.37
Zfp212	chr6	47879063	47881476	-0.63	-0.12
Zfp775	chr6	48555624	48569787	-0.41	-0.16
Zfp775	chr6	48569935	48570026	-0.24	0.38
Zfp775	chr6	48570414	48570923	-1.15	-0.76
Gata2	chr6	88154654	88155829	-0.38	-0.05
Nr2c2	chr6	92041411	92042461	-2.10	-0.74
Nr2c2	chr6	92114256	92117961	-0.62	0.09
Brpf1	chr6	113257130	113267099	-6.11	-0.18
Brpf1	chr6	113267573	113269353	-20.40	-0.58
Brpf1	chr6	113272417	113274519	-13.10	-0.60
Chd4	chr6	125046180	125050907	-20.10	-0.46
Chd4	chr6	125079888	125080399	-1.95	-0.09
Cdkn1b	chr6	134870958	134872360	-3.01	0.32
H2afj	chr6	136757307	136757517	-2.15	-0.48
Cnot4	chr6	34973322	34992713	-1.58	0.32
Cnot4	chr6	34996096	35002891	-0.80	0.08
Hipk2	chr6	38647898	38665995	-46.30	-0.52
Hipk2	chr6	38767930	38787077	-4.22	-0.11
Hipk2	chr6	38825800	38826040	-0.40	-0.04
Zfp786	chr6	47769651	47770337	-0.58	-0.74
Zfp786	chr6	47770782	47771140	-0.50	-0.90
Zfp786	chr6	47771318	47771676	-0.29	0.81
Zfp777	chr6	47974246	47998850	-8.38	-0.33
Zfp746	chr6	48013500	48016503	-1.30	-0.16

Zfp746	chr6	48033523	48036532	-0.67	0.27
Gadd45a	chr6	66985208	66987341	-8.35	-0.44
Rybp	chr6	100182064	100183112	-0.57	0.17
Zfp248	chr6	118378705	118382923	-2.80	0.51
Phc1	chr6	122273438	122273618	-0.97	0.44
Phc1	chr6	122275560	122275830	-0.61	-0.25
Tigar	chr6	127037498	127038307	-0.89	-0.11
Ccnd2	chr6	127079556	127080275	-3.38	0.32
Ccnd2	chr6	127100856	127101036	-0.93	0.38
Rhno1	chr6	128307315	128309210	-1.30	-0.13
Kras	chr6	145168813	145168964	-1.16	-0.35
Zfp667	chr7	6256343	6256524	-1.03	1.18
Zfp667	chr7	6256792	6256942	-0.55	0.66
Zfp667	chr7	6257689	6257840	-1.67	2.29
Zfp78	chr7	6331550	6332268	-2.40	1.42
Zfp28	chr7	6344996	6347498	-0.41	0.06
Zfp110	chr7	13434024	13434472	-0.91	0.17
Zfp324	chr7	13555672	13556361	-3.85	-1.08
Zfp324	chr7	13556928	13557646	-0.89	-0.35
Zfp446	chr7	13564366	13564486	-0.27	-0.05
Zfp446	chr7	13567286	13568276	-1.47	-0.29
Zfp446	chr7	13568455	13569056	-0.40	-0.07
Ppp1r13l	chr7	19956897	19960606	-7.13	-0.66
Ercc2	chr7	19979470	19981041	-4.45	-0.33
Zfp180	chr7	24882912	24891546	-4.68	0.24
Zfp112	chr7	24910093	24910364	-0.73	-1.09
Zfp112	chr7	24910573	24910664	-0.48	-1.72
Zfp112	chr7	24911022	24911533	-0.31	-0.12
Zfp235	chr7	24925651	24925772	-1.05	0.94
Zfp235	chr7	24926760	24927181	-1.25	-0.51
Zfp235	chr7	24927869	24928170	-0.36	-0.17
Tgfb1	chr7	26472020	26477430	-36.70	-0.26
Akt2	chr7	28422173	28423398	-0.50	-0.06
Zfp30	chr7	30577820	30578780	-4.53	1.80
Zfp790	chr7	30613435	30616012	-3.35	0.25
Zfp420	chr7	30645086	30661309	-4.28	0.31
Zfp382	chr7	30916575	30919814	-0.57	-0.05
Polr2i	chr7	31017332	31017772	-0.55	0.13
Zfp658	chr7	50828048	50828348	-0.67	0.44
Zfp658	chr7	50828646	50829515	-0.33	-0.03
Zfp658	chr7	50829663	50830323	-0.90	-0.28
Zfp719	chr7	50839645	50846689	-6.02	0.33
Zfp819	chr7	50873231	50873322	-0.50	3.17

Gtf2h1	chr7	54051493	54051644	-1.33	-0.22
Gtf2h1	chr7	54078270	54078601	-5.40	0.33
Zfp710	chr7	87169699	87227267	-11.50	-0.28
Zfp710	chr7	87231444	87231625	-0.36	0.04
Zfp710	chr7	87233516	87236204	-0.61	0.04
Lamtor1	chr7	109054380	109058241	-0.87	-0.19
Lamtor1	chr7	109059942	109060091	-16.60	-0.68
Zfp771	chr7	134388039	134398315	-3.36	-0.36
Setd1a	chr7	134920902	134921023	-1.37	0.88
Setd1a	chr7	134927635	134930735	-0.69	-0.03
Setd1a	chr7	134931750	134932028	-1.01	-0.16
Setd1a	chr7	134934941	134940915	-7.31	-0.23
Zik1	chr7	11075804	11076075	-2.76	1.74
Zfp551	chr7	13000735	13000886	-0.20	-0.08
Zfp551	chr7	13001514	13002233	-2.46	1.45
Zfp94	chr7	25087140	25088844	-2.13	-0.44
Psmd8	chr7	29965393	29965573	-13.80	-0.43
Zfp940	chr7	30629198	30629439	-0.25	-0.03
Zfp940	chr7	30629588	30629649	-0.64	1.82
Zfp940	chr7	30630307	30630548	-0.95	1.24
Zfp940	chr7	30630817	30630998	-0.73	0.68
Zfp27	chr7	30678594	30680419	-0.52	-0.05
Zfp27	chr7	30680747	30683850	-0.91	0.18
Zfp14	chr7	30823256	30823436	-0.57	1.58
Zfp14	chr7	30823733	30823854	-0.28	0.22
Zfp566	chr7	30862385	30862860	-0.44	0.13
Zfp566	chr7	30862978	30863483	-3.46	0.75
Kmt2b	chr7	31359377	31361794	-20.90	-0.55
Kmt2b	chr7	31368445	31373745	-14.90	-0.34
Gpi1	chr7	34987182	34987689	-15.30	-0.25
Ccne1	chr7	38883150	38883358	-1.08	-0.54
Zfp715	chr7	50552997	50556994	-8.01	0.30
Zfp473	chr7	51987480	51987751	-0.36	-0.29
Zfp473	chr7	51987810	51988051	-0.70	-0.61
Zfp473	chr7	51988379	51988830	-1.38	1.24
Zfp473	chr7	51988979	51989760	-8.39	2.40
Med25	chr7	52132313	52133597	-6.95	-0.47
Bax	chr7	52721428	52721703	-3.69	-0.20
Bax	chr7	52721999	52722238	-1.35	0.28
E2f8	chr7	56123087	56123387	-0.98	-0.44
E2f8	chr7	56126142	56126233	-0.71	-0.66
E2f8	chr7	56126292	56127247	-0.85	-0.32
Blm	chr7	87647292	87647681	-3.04	-0.71

Blm	chr7	87654437	87658369	-0.32	-0.01
Eed	chr7	97125556	97129068	-6.88	-0.52
Eed	chr7	97129276	97129396	-0.46	-0.10
Zfp747	chr7	134516438	134516707	-0.85	-0.40
Zfp747	chr7	134516765	134519504	-0.42	0.03
9130019O22Rik	chr7	134526131	134526281	-0.56	1.23
9130019O22Rik	chr7	134526847	134529144	-0.95	-0.14
E430018J23Rik	chr7	134534467	134534528	-0.84	-2.04
E430018J23Rik	chr7	134534616	134536839	-0.37	-0.06
Zfp764	chr7	134547897	134549152	-1.39	-0.24
Zfp764	chr7	134550126	134550336	-0.39	-0.19
Zfp688	chr7	134562479	134563593	-1.17	-0.28
Zfp688	chr7	134565487	134565548	-0.77	-1.45
Zfp689	chr7	134587294	134588642	-1.79	-0.40
Zfp689	chr7	134592252	134592433	-0.39	0.12
Zfp668	chr7	135009351	135009442	-0.32	-0.14
Zfp668	chr7	135009620	135020337	-10.40	-0.40
Pidd1	chr7	148624413	148624744	-3.35	-0.92
Pidd1	chr7	148628991	148629907	-3.04	-1.22
Ccnd1	chr7	152118471	152118592	-5.59	-0.32
Ccnd1	chr7	152125559	152125830	-6.58	-0.44
Tfdp1	chr8	13377166	13377375	-0.39	0.04
Tfdp1	chr8	13377582	13377702	-0.58	-0.09
Kat6a	chr8	23970010	24013683	-5.12	0.22
Kat6a	chr8	24040759	24043005	-0.60	-0.06
Kat6a	chr8	24046218	24051490	-0.77	-0.03
Zfp703	chr8	28087837	28090862	-45.70	-0.65
Ppp2cb	chr8	34710092	34710359	-0.73	-0.37
Nr2c2ap	chr8	72655231	72655498	-1.93	0.47
Nr2c2ap	chr8	72657115	72657440	-1.00	-0.16
Ell	chr8	73063573	73063724	-2.49	-0.72
Ell	chr8	73109061	73115709	-19.10	-0.74
Sin3b	chr8	75247186	75248002	-1.72	-0.30
Sin3b	chr8	75273755	75273906	-1.47	-0.52
Sin3b	chr8	75280901	75281383	-3.95	-0.67
Nr3c2	chr8	79432403	79432554	-0.61	1.74
Nr3c2	chr8	79433567	79433776	-0.74	-1.52
Cbfb	chr8	107694632	107694812	-1.65	-0.29
Zfp90	chr8	108947910	108948329	-2.59	0.80
Zfp90	chr8	108948657	108949616	-1.45	0.33
Zfp1	chr8	114190002	114193957	-0.62	0.14
Zfp1	chr8	114194046	114194762	-2.59	-0.49
Banp	chr8	124550400	124550731	-0.86	-0.47

Banp	chr8	124551389	124551899	-1.05	-0.63
Banp	chr8	124552078	124552768	-1.73	-0.70
Zfpm1	chr8	124806190	124856010	-4.04	0.63
Zfpm1	chr8	124859027	124861147	-0.69	-0.07
Ash2l	chr8	26950536	26958082	-1.11	-0.21
Wrn	chr8	34346314	34346884	-1.70	0.55
Wrn	chr8	34434722	34439590	-0.78	0.26
Cnot7	chr8	41592761	41593001	-0.56	0.08
Ing2	chr8	48753850	48758943	-1.32	0.15
Ing2	chr8	48760272	48760513	-0.64	-0.35
Gatad2a	chr8	72433183	72435724	-28.20	-0.49
Gatad2a	chr8	72459749	72459840	-2.26	-0.26
Gatad2a	chr8	72498029	72498150	-0.37	-0.11
Gatad2a	chr8	72519918	72520248	-1.28	-0.35
Nr2f6	chr8	73898225	73905851	-12.70	-0.57
Med26	chr8	75019202	75072090	-10.00	-0.44
Csnk2a2	chr8	97976954	97977225	-0.35	-0.03
Psmb10	chr8	108461631	108461967	-2.80	-0.63
Psmd7	chr8	110104338	110108077	-4.03	0.12
Maml2	chr9	13424582	13425122	-2.71	0.92
Maml2	chr9	13510293	13511342	-0.77	0.17
Zfp317	chr9	19451183	19452859	-22.30	0.60
Pin1	chr9	20456573	20459918	-1.39	-0.27
Smarca4	chr9	21505589	21507505	-3.30	0.23
Zfp202	chr9	40014989	40015898	-2.39	-0.75
Zfp202	chr9	40018534	40019996	-1.21	0.18
Usp2	chr9	43883786	43884026	-0.81	2.12
Usp2	chr9	43902032	43902302	-0.96	-0.35
H2afx	chr9	44143446	44144156	-0.77	-0.05
Ppp2r1b	chr9	50687990	50688710	-0.71	-0.09
Ppp2r1b	chr9	50701016	50701675	-4.03	-0.60
Sin3a	chr9	56958532	56958892	-0.32	-0.02
Sin3a	chr9	56973115	56975576	-0.84	0.11
Rora	chr9	69226715	69226835	-0.32	0.16
Atr	chr9	95765579	95765669	-0.85	-0.40
Tfdp2	chr9	96218548	96218699	-1.00	0.56
Ctnnb1	chr9	120842577	120859669	-0.34	-0.01
Ctnnb1	chr9	120867712	120868847	-5.68	0.15
Zkscan7	chr9	122797636	122797876	-0.42	-0.24
Zkscan7	chr9	122803837	122804973	-0.30	0.02
Med17	chr9	15066594	15066715	-0.33	0.04
Zfp558	chr9	18260656	18261436	-1.57	0.79
Zfp426	chr9	20274940	20276201	-1.87	0.28

Zfp266	chr9	20304179	20306501	-0.78	-0.10
Kmt2a	chr9	44612636	44614167	-3.81	0.25
Kmt2a	chr9	44615605	44617182	-1.06	0.16
Kmt2a	chr9	44625809	44629185	-31.10	0.48
Kmt2a	chr9	44629634	44630414	-7.20	0.44
Kmt2a	chr9	44655111	44656174	-7.56	0.48
Kmt2a	chr9	44656473	44656924	-1.40	0.24
Kmt2a	chr9	44657403	44658123	-21.00	0.79
Atm	chr9	53247503	53248264	-1.55	0.23
Pml	chr9	58066214	58066305	-1.33	0.78
Pml	chr9	58067472	58068881	-10.10	-0.47
Pml	chr9	58078383	58097563	-4.44	-0.27
Smad3	chr9	63496369	63496969	-0.55	0.19
Smad3	chr9	63497327	63498046	-0.81	0.32
Smad3	chr9	63605729	63605864	-0.51	-1.42
Rnf111	chr9	70274555	70277390	-0.83	-0.10
Rnf111	chr9	70323650	70350792	-12.70	0.37
Tcf12	chr9	71693887	71694367	-8.81	0.54
Tcf12	chr9	71958621	71958922	-1.18	0.36
Tcf12	chr9	71959385	71959626	-0.68	-0.16
Atrip	chr9	108960445	108961754	-16.60	-0.43
Atrip	chr9	108967670	108968299	-1.36	-0.26
Cnot10	chr9	114541870	114549228	-1.75	-0.36
Zfp445	chr9	122761016	122763080	-42.10	0.58
Zfp445	chr9	122770871	122775124	-1.55	0.17
Cited2	chr10	17443033	17444853	-33.40	-0.20
Sgk1	chr10	21718544	21718843	-0.78	0.37
Sgk1	chr10	21719081	21719290	-4.21	1.23
Sesn1	chr10	41530379	41607293	-0.28	0.00
Sesn1	chr10	41627347	41627735	-0.40	-0.14
Stk11	chr10	79579091	79588239	-0.80	0.07
Stk11	chr10	79592650	79593215	-1.40	-0.20
Txnrd1	chr10	82296755	82323149	-0.39	-0.07
Txnrd1	chr10	82323297	82335784	-5.40	-0.55
E2f7	chr10	110216189	110216249	-0.35	-0.75
Smarcc2	chr10	127920261	127920695	-0.29	0.00
Smarcc2	chr10	127924543	127924604	-1.40	-0.59
Smarcc2	chr10	127926123	127926244	-0.38	0.05
Ddit4	chr10	59412924	59414518	-67.50	-0.91
Nrbf2	chr10	66729823	66730867	-2.28	-0.23
Ube2d1	chr10	70718144	70718592	-2.63	0.38
Smarcb1	chr10	75359602	75359780	-1.07	-0.22
Polr2e	chr10	79501316	79502404	-2.85	-0.43

Mbd3	chr10	79855285	79856691	-7.71	-0.28
Tcf3	chr10	79872416	79873314	-1.26	-0.16
Cradd	chr10	94637889	94638400	-0.78	0.33
Cradd	chr10	94785366	94785547	-0.28	0.04
Cnot2	chr10	115922246	115922606	-0.99	-0.19
Cnot2	chr10	115922874	115923144	-4.87	0.64
Yeats4	chr10	116652465	116652884	-0.70	0.11
Mdm2	chr10	117126170	117126441	-1.00	0.08
Mdm2	chr10	117126799	117129718	-14.10	0.17
Dyrk2	chr10	118296404	118305900	-0.44	-0.03
Pip4k2c	chr10	126635286	126635944	-0.36	-0.01
Pip4k2c	chr10	126636730	126637327	-0.37	0.03
Cdk2	chr10	128135921	128136041	-0.48	-0.19
Psme4	chr11	30671774	30672135	-0.54	-0.18
Med7	chr11	46250448	46253332	-0.31	0.02
Med7	chr11	46254128	46254877	-4.70	0.38
Ppp2ca	chr11	51912325	51912474	-4.51	-0.88
Ppp2ca	chr11	51935449	51935836	-0.34	0.00
Cnot8	chr11	57930958	57931258	-0.74	0.18
Zkscan6	chr11	65636352	65642442	-3.43	-0.39
Aurkb	chr11	68861818	68863941	-3.92	-0.59
Trp53	chr11	69393860	69394608	-0.43	-0.06
Ywhae	chr11	75546388	75546567	-5.18	-0.26
Suz12	chr11	79843530	79845978	-0.38	0.01
Cdk5r1	chr11	80291441	80292571	-1.52	0.38
Cdk5r1	chr11	80293343	80293493	-0.43	0.26
Cdk5r1	chr11	80291445	80292555	-1.61	0.40
Cdk5r1	chr11	80293392	80293483	-0.29	0.10
Taf15	chr11	83286609	83286670	-0.74	-0.19
Ppm1d	chr11	85124755	85125383	-7.40	-0.93
Ppm1d	chr11	85140378	85140529	-1.24	0.48
Ppm1d	chr11	85153180	85159827	-0.39	-0.03
Supt4a	chr11	87557059	87557119	-0.31	-0.09
Cdk12	chr11	98064618	98065548	-5.37	-0.20
Cdk12	chr11	98071707	98072546	-3.00	-0.14
Cdk12	chr11	98104793	98107067	-2.29	0.20
Cdk12	chr11	98111003	98111783	-7.97	-0.40
Psmd3	chr11	98543867	98546829	-30.30	-0.74
Thra	chr11	98603186	98614709	-0.74	0.42
Thra	chr11	98614768	98617477	-2.58	-0.78
Thra	chr11	98625600	98626259	-4.68	-0.69
Thra	chr11	98629482	98629782	-10.30	1.38
Rara	chr11	98799009	98808908	-1.61	-0.58

Rara	chr11	98808997	98822329	-5.35	-0.55
Rara	chr11	98827782	98829451	-0.74	-0.25
Rara	chr11	98831600	98835508	-19.40	-0.66
Psme3	chr11	101177564	101178336	-0.62	0.07
Psme3	chr11	101182070	101183288	-0.64	-0.06
Cbx2	chr11	118889293	118890939	-23.30	-1.92
Rptor	chr11	119465086	119517298	-1.88	-0.26
Rptor	chr11	119759737	119760755	-0.53	0.05
Hus1	chr11	8896711	8896861	-0.56	0.17
Maml1	chr11	50069912	50070420	-0.98	0.21
Maml1	chr11	50071136	50074538	-0.86	-0.10
Maml1	chr11	50076892	50105838	-2.71	-0.20
Rad50	chr11	53463650	53465769	-2.93	-0.38
Zkscan17	chr11	59299022	59299382	-2.54	0.44
Zkscan17	chr11	59300338	59306167	-1.66	-0.14
Zkscan17	chr11	59319782	59319933	-1.97	-1.06
Top3a	chr11	60553560	60555499	-1.34	0.26
Top3a	chr11	60555857	60557535	-1.26	0.23
Top3a	chr11	60590477	60590867	-9.76	1.46
Ncor1	chr11	62130880	62131511	-1.35	0.12
Ncor1	chr11	62147246	62147337	-0.41	-0.06
Ncor1	chr11	62182924	62186997	-26.60	-0.65
Ncor1	chr11	62196845	62198278	-2.94	-0.40
Ncor1	chr11	62252002	62270948	-5.64	-1.11
Zfp287	chr11	62527053	62527804	-2.03	0.58
Zfp287	chr11	62527953	62528373	-1.76	0.62
Zfp287	chr11	62528582	62528703	-0.25	-0.02
Zfp287	chr11	62541753	62542444	-0.32	0.04
Zfp287	chr11	62545196	62545317	-1.08	1.22
Chd3	chr11	69157463	69157973	-5.22	-0.48
Chd3	chr11	69174956	69175626	-0.72	-0.14
Chd3	chr11	69177569	69182868	-4.44	-0.54
Rpa1	chr11	75114477	75116219	-1.52	-0.18
Rpa1	chr11	75154547	75161885	-1.59	0.39
Rffl	chr11	82619321	82632040	-24.80	-0.71
Brip1	chr11	85874715	85878344	-2.90	0.50
Med13	chr11	86084344	86084585	-0.84	0.15
Med13	chr11	86100121	86100212	-2.56	-0.43
Med13	chr11	86104608	86114217	-1.33	0.09
Med13	chr11	86132926	86141980	-0.71	0.05
Pcgf2	chr11	97551180	97551450	-9.46	-1.52
Pcgf2	chr11	97553219	97553423	-0.65	0.22
Pip4k2b	chr11	97578204	97584269	-3.42	-0.14

Med1	chr11	98014158	98015024	-2.02	0.27
Med1	chr11	98015173	98015684	-0.74	-0.09
Med1	chr11	98016102	98017333	-0.59	0.04
Med1	chr11	98017662	98017873	-2.97	-0.38
Med1	chr11	98017962	98018233	-0.58	0.07
Med1	chr11	98018352	98019732	-1.59	0.09
Med1	chr11	98050576	98054607	-1.81	-0.28
Med24	chr11	98590218	98590689	-0.90	0.32
Nr1d1	chr11	98629338	98631545	-20.50	1.35
Nr1d1	chr11	98631693	98632599	-4.38	0.49
Nr1d1	chr11	98633166	98636556	-0.67	0.09
Kat2a	chr11	100566059	100566924	-11.10	-0.42
Kat2a	chr11	100573600	100573691	-0.37	-0.10
Brca1	chr11	101384784	101385234	-1.07	-0.42
Brca1	chr11	101385503	101386581	-0.66	0.14
Brca1	chr11	101386909	101387659	-0.94	0.23
Smarcd2	chr11	106133897	106134286	-1.29	-0.39
Smurf2	chr11	106683743	106684388	-0.37	-0.04
Smurf2	chr11	106781609	106781789	-3.23	-1.30
Cbx8	chr11	118899838	118901471	-0.31	0.01
Cbx4	chr11	118941130	118941400	-2.21	0.97
Cbx4	chr11	118941729	118947491	-3.35	-0.13
E2f6	chr12	16817830	16817920	-1.61	-0.89
E2f6	chr12	16832231	16832859	-2.23	-0.27
Ppm1a	chr12	73862227	73885470	-1.34	-0.08
Ppm1a	chr12	73894914	73895183	-2.86	0.44
Psmc1	chr12	101348377	101351316	-1.50	-0.23
Ccnk	chr12	109418007	109424620	-0.44	0.06
Zfp839	chr12	112088488	112099099	-6.28	-0.75
Zfp839	chr12	112106449	112107344	-2.89	-0.47
Ywhaq	chr12	21422717	21423076	-5.19	-0.53
Ywhaq	chr12	21423381	21423442	-0.93	0.75
Max	chr12	78038822	78039718	-3.26	-0.11
Max	chr12	78063025	78063205	-5.46	-0.52
Med6	chr12	82674550	82674905	-2.82	0.39
Snw1	chr12	88791126	88791810	-8.21	-0.35
Ppp1r13b	chr12	113066998	113068529	-0.52	-0.10
Ppp1r13b	chr12	113070821	113073439	-7.32	-0.51
Ppp1r13b	chr12	113146175	113146266	-0.46	1.29
Akt1	chr12	113912217	113912487	-1.97	-0.56
Zkscan4	chr13	21576264	21576865	-1.13	-1.76
Hist1h3h	chr13	21809496	21809698	-1.42	0.30
Hist1h3f	chr13	23636303	23636393	-2.07	-0.34

Hist1h2bg	chr13	23663268	23663474	-1.13	-0.09
Hist1h3d	chr13	23667608	23667786	-1.12	-0.25
Hist1h2bc	chr13	23776067	23776213	-2.98	0.39
Hist1h2ab	chr13	23842956	23843254	-0.48	0.06
Prelid1	chr13	55423415	55423591	-1.89	-0.18
Rmi1	chr13	58503698	58503988	-0.54	-0.08
Rmi1	chr13	58508365	58511252	-1.36	0.13
Zfp369	chr13	65397670	65398417	-5.12	-0.73
Med10	chr13	69954526	69954824	-5.28	-0.32
Taf9	chr13	101435858	101436098	-1.55	0.21
Plk2	chr13	111185251	111185700	-14.20	-0.89
Plk2	chr13	111190024	111191021	-3.14	-0.32
Slc38a9	chr13	113528209	113528690	-2.86	1.40
Cdk13	chr13	17809653	17811567	-1.53	0.10
Cdk13	chr13	17858358	17896931	-3.76	-0.17
Zkscan3	chr13	21479260	21480454	-4.71	0.32
Zkscan3	chr13	21488062	21494295	-3.85	-0.57
Zkscan8	chr13	21611941	21613722	-2.34	-0.34
Zkscan8	chr13	21617156	21618788	-0.43	0.10
Hist1h3i	chr13	21875180	21875266	-0.41	0.06
Hist1h3c	chr13	23837329	23837390	-0.31	-0.03
Tfap2a	chr13	40812539	40812719	-0.15	0.29
Dek	chr13	47201073	47201589	-1.54	0.34
Ctsl	chr13	64470393	64471614	-17.40	-0.32
Zfp729a	chr13	67719104	67720063	-10.30	0.99
Zfp729a	chr13	67720451	67721051	-2.35	0.38
Zfp729a	chr13	67721469	67722248	-0.63	0.15
Zfp738	chr13	67768467	67769574	-3.21	0.63
Zfp738	chr13	67770082	67770382	-0.89	0.38
Zfp738	chr13	67771278	67771698	-0.34	-0.07
Nr2f1	chr13	78328745	78328956	-0.58	-2.91
Jmy	chr13	94204723	94212614	-10.00	0.49
Jmy	chr13	94242583	94269644	-4.59	-0.33
Rad17	chr13	101387447	101389058	-1.23	-0.21
Psmc6	chr14	45949498	45950514	-0.60	-0.09
Med4	chr14	73909855	73910973	-1.63	-0.58
Med4	chr14	73917759	73918293	-3.57	-0.40
Elf1	chr14	79881419	79936023	-1.87	-0.46
Elf1	chr14	79973114	79980877	-2.36	0.14
Rarb	chr14	17407447	17451237	-0.51	-0.82
Nr1d2	chr14	19038938	19040485	-19.30	1.58
Nr1d2	chr14	19047334	19049180	-7.51	0.70
Pip4p1	chr14	51546074	51546434	-0.32	-0.01

Supt16	chr14	52780871	52782395	-6.16	-0.21
Supt16	chr14	52803669	52816914	-2.64	-0.39
Prmt5	chr14	55126018	55126553	-0.91	-0.11
Rabggta	chr14	56340520	56340807	-0.58	0.19
Cenpj	chr14	57145687	57148622	-0.29	-0.01
Cenpj	chr14	57170788	57171655	-0.31	-0.01
Cenpj	chr14	57171744	57172312	-0.62	0.14
Cenpj	chr14	57183992	57190683	-0.52	0.50
Gtf2f2	chr14	76297012	76306651	-1.19	-0.13
Prkaa1	chr15	5093860	5110647	-7.59	-0.91
Prkaa1	chr15	5126670	5126820	-1.22	-0.18
Prkaa1	chr15	5127194	5129214	-5.38	0.43
Rictor	chr15	6736456	6737266	-1.44	-0.13
Rictor	chr15	6745958	6747284	-0.93	0.10
Polr2k	chr15	36103800	36104751	-0.34	0.08
Zfp707	chr15	75804010	75804159	-0.65	-0.41
Zfp707	chr15	75804871	75806149	-11.00	-0.97
Rbx1	chr15	81305971	81306061	-1.28	-0.57
Ep300	chr15	81416643	81438767	-12.50	-0.29
Ep300	chr15	81479273	81481819	-5.01	-0.13
L3mbtl2	chr15	81516741	81518556	-3.63	-0.29
Arid2	chr15	96117952	96118043	-0.28	-0.02
Arid2	chr15	96199968	96201175	-7.90	0.36
Arid2	chr15	96201654	96209326	-6.30	0.26
Arid2	chr15	96223066	96232715	-1.11	-0.22
Nr4a1	chr15	101097276	101102664	-424.00	-1.45
Nr4a1	chr15	101103587	101105046	-137.00	-1.52
Zfp740	chr15	102034168	102035505	-2.24	-0.24
Zfp740	chr15	102039660	102043940	-1.65	-0.13
Sp1	chr15	102238241	102260424	-0.48	0.02
Sp1	chr15	102260572	102261651	-2.06	0.20
Skp2	chr15	9043329	9043510	-0.95	0.41
Skp2	chr15	9069207	9070137	-7.83	-1.04
Ywhaz	chr15	36723285	36723376	-0.55	0.11
Zfp706	chr15	36936977	36937157	-1.44	-0.45
Taf2	chr15	54847403	54848124	-0.49	-0.06
Taf2	chr15	54903436	54903647	-0.61	-0.15
Atad2	chr15	57928856	57930279	-4.77	0.52
Atad2	chr15	57966457	57966637	-0.74	-0.28
Cbx6	chr15	79658129	79664703	-7.13	-0.57
Brd1	chr15	88518753	88518814	-4.89	0.89
Brd1	chr15	88543169	88543809	-0.49	-0.05
Brd1	chr15	88547455	88564529	-12.40	0.36

Yaf2	chr15	93115848	93167246	-0.88	0.11
Vdr	chr15	97687063	97687362	-0.53	1.58
Ccnt1	chr15	98373641	98375016	-10.90	0.30
Ccnt1	chr15	98398086	98398654	-9.19	1.21
Prkag1	chr15	98643704	98643794	-0.45	-0.08
Rarg	chr15	102066030	102069302	-12.80	-0.78
Zfp385a	chr15	103144952	103145191	-1.58	-0.37
Zfp385a	chr15	103150806	103164738	-0.29	0.01
Zfp263	chr16	3744098	3745056	-3.71	-0.36
Zfp263	chr16	3748158	3750040	-1.24	0.09
Psmd2	chr16	20651724	20652324	-20.60	-0.49
Zfp597	chr16	3865585	3871203	-2.18	0.33
Crebbp	chr16	4084047	4087773	-0.48	-0.02
Crebbp	chr16	4154961	4213404	-3.27	-0.16
Usp7	chr16	8738344	8757028	-0.78	-0.28
Runx1	chr16	92605608	92606269	-0.70	0.06
Runx1	chr16	92695902	92697552	-25.50	0.56
Arid1b	chr17	4995073	4995882	-1.66	-0.67
Arid1b	chr17	4996061	5097577	-3.74	-0.55
Arid1b	chr17	5332607	5337057	-2.55	0.24
Arid1b	chr17	5337116	5339263	-0.52	0.10
Arid1b	chr17	5341518	5343345	-1.18	0.09
Zfp160	chr17	21162599	21164033	-0.57	0.06
Zfp160	chr17	21164271	21164332	-0.31	0.21
Zfp160	chr17	21178510	21179347	-0.31	0.05
Ppard	chr17	28369758	28432393	-6.75	-0.70
Ppard	chr17	28435930	28437576	-21.80	-0.79
Mapk14	chr17	28828316	28828704	-3.84	-0.61
Mapk14	chr17	28884007	28885350	-1.27	0.08
Brpf3	chr17	28938100	28938251	-0.36	0.50
Brpf3	chr17	28943116	28944224	-7.70	0.75
Brpf3	chr17	28954048	28960704	-0.96	0.16
Brpf3	chr17	28973668	28973878	-0.46	0.17
Cdkn1a	chr17	29228020	29235694	-168.00	-0.80
Daxx	chr17	34047504	34050602	-124.00	-0.92
Rxrb	chr17	34174187	34175165	-2.09	-0.18
Psmb8	chr17	34335288	34335468	-2.22	-0.33
Ehmt2	chr17	35042119	35042557	-5.05	-0.58
Ehmt2	chr17	35049712	35050992	-0.63	0.07
Med20	chr17	47759792	47760513	-3.12	-0.33
Zfp213	chr17	23694178	23694744	-0.80	0.45
Zfp213	chr17	23694951	23696378	-0.41	-0.22
Pdpk1	chr17	24215553	24216093	-11.80	0.63

Mlst8	chr17	24612351	24613045	-0.59	-0.10
Ube2i	chr17	25398055	25398776	-6.49	-0.64
Stub1	chr17	25967580	25967909	-3.51	-0.27
Stub1	chr17	25969947	25970008	-0.31	-0.35
Stub1	chr17	25970096	25970306	-1.82	-0.63
Taf11	chr17	28038342	28038672	-4.35	-0.52
Taf11	chr17	28042348	28045188	-1.94	-0.31
Notch3	chr17	32278193	32278314	-0.50	2.81
Ring1	chr17	34158095	34160251	-0.84	-0.10
Brd2	chr17	34249292	34249918	-19.30	0.29
Brd2	chr17	34251401	34252141	-0.53	0.02
Brd2	chr17	34254036	34257250	-434.00	-1.12
Runx2	chr17	44744958	44745199	-11.00	1.69
Runx2	chr17	44872639	44873298	-1.80	-0.39
Gtf2f1	chr17	57143372	57143559	-0.74	-0.13
Tgif1	chr17	71193544	71195840	-57.00	-0.42
Tgif1	chr17	71200227	71202842	-14.10	-0.47
Rbbp8	chr18	11880308	11881195	-1.41	0.17
Taf4b	chr18	14971627	14971957	-0.31	0.02
Polr2d	chr18	31955877	31956355	-0.34	-0.02
Ercc3	chr18	32399984	32400372	-0.60	-0.12
Ercc3	chr18	32429502	32429682	-1.02	0.23
Prelid3a	chr18	67639251	67639460	-1.99	1.09
Smad7	chr18	75527018	75527675	-0.56	0.13
Smad7	chr18	75528299	75554128	-4.14	0.55
Smad2	chr18	76462139	76464727	-1.34	-0.19
Epc1	chr18	6436931	6440144	-10.10	0.66
Epc1	chr18	6441163	6450203	-18.10	1.12
Epc1	chr18	6490689	6515876	-1.67	-0.51
Kctd1	chr18	15127880	15128090	-0.86	2.17
Kctd1	chr18	15128179	15132643	-0.22	0.12
Kctd1	chr18	15166356	15220489	-0.39	-0.45
Kctd1	chr18	15220578	15220848	-0.65	1.31
Kctd1	chr18	15221623	15221804	-0.65	-1.71
Taf7	chr18	37801809	37803149	-6.84	0.58
Nr3c1	chr18	39588392	39650238	-0.66	-0.04
Smad4	chr18	73799858	73800008	-0.35	-0.03
Smad4	chr18	73837391	73837750	-0.38	-0.03
Smad4	chr18	73863156	73863395	-1.93	-1.18
Ctdp1	chr18	80605326	80630972	-2.69	0.27
Ctdp1	chr18	80642798	80647936	-2.79	-0.16
Men1	chr19	6334978	6336781	-0.48	-0.04
Men1	chr19	6338873	6340864	-2.26	-0.13

Smarca2	chr19	26705324	26705445	-2.19	-1.03
Pcgf5	chr19	36484296	36486641	-0.57	0.12
Pcgf5	chr19	36519525	36530396	-3.63	-0.53
Taf5	chr19	47145518	47149502	-0.56	-0.15
Taf5	chr19	47156922	47157401	-0.43	-0.08
Rad9a	chr19	4195437	4196375	-0.34	-0.01
Rbm14	chr19	4743957	4744585	-0.74	-0.21
Rbm14	chr19	4803462	4811634	-0.83	-0.05
Kat5	chr19	5603043	5603836	-2.52	-0.22
Kat5	chr19	5609321	5609592	-0.57	-0.12
Kat5	chr19	5609854	5610034	-0.61	-0.31
Prdx5	chr19	6984015	6984135	-10.60	-0.65
Esrra	chr19	6996237	6996298	-0.43	-0.27
Polr2g	chr19	8872986	8873047	-0.44	-0.14
Ldb1	chr19	46107090	46108545	-0.46	-0.03
Pcgf6	chr19	47108856	47109007	-0.32	0.04
Pcgf6	chr19	47125005	47125335	-10.30	-1.25
Usp9x	chrX	12733152	12733872	-13.20	0.35
Med12	chrX	98488099	98488220	-1.26	0.32
Taf1	chrX	98794675	98794885	-1.77	-0.35
Rragb	chrX	149574678	149575911	-1.22	0.33
G6pdx	chrX	71655299	71655479	-0.33	-0.01
Chm	chrX	110156917	110157037	-0.85	0.35
Rad9b	chr5	122803670	122803730	-0.23	0.81
Rad9b	chr5	122803907	122804085	-0.68	0.80

Metabolism of proteins

gene_name	chr	chromStart	chromEnd	diff.lg.p	diff.log2.fc
Rab23	chr1	33795210	33796982	-0.80	0.16
Ttll4	chr1	74725556	74727916	-10.70	-0.35
Ttll4	chr1	74744098	74744368	-1.67	-0.40
Mrpl44	chr1	79772801	79774975	-2.18	-0.24
Mrpl44	chr1	79776154	79777602	-0.35	0.03
Sp100	chr1	87605724	87606023	-1.74	0.47
Cops7b	chr1	88502539	88502837	-3.51	-0.57
Cops8	chr1	92508875	92509678	-1.17	-0.09
Ube2f	chr1	93182063	93182602	-2.18	-0.17
Asb1	chr1	93437321	93437716	-0.36	-0.19
Asb1	chr1	93440810	93443681	-2.13	-0.63
Asb1	chr1	93451344	93451764	-1.52	0.40
Mgat5	chr1	129203345	129203496	-1.16	-0.43
Mgat5	chr1	129376047	129379549	-1.14	-0.18
Yod1	chr1	132614351	132616572	-0.52	0.05
Rab7b	chr1	133585271	133593615	-0.74	-0.38

Rab7b	chr1	133602280	133610067	-11.80	-0.70
Rab29	chr1	133763853	133763913	-0.39	-0.39
Rbbp5	chr1	134394620	134400982	-0.63	-0.07
Rbbp5	chr1	134401101	134401282	-1.08	0.24
Ube2t	chr1	136859153	136864304	-0.88	-0.47
Edem3	chr1	153658740	153666587	-2.13	-0.17
Pigc	chr1	163899318	163901752	-3.01	-0.22
F5	chr1	166122352	166122563	-1.98	4.32
F5	chr1	166123252	166123343	-0.35	1.70
F5	chr1	166123612	166123702	-0.24	1.22
F5	chr1	166124001	166124272	-1.70	2.22
Fcgr4	chr1	172959296	172959476	-0.48	-0.13
Adamts4	chr1	173188947	173189485	-1.30	-1.86
B4galt3	chr1	173206252	173206788	-5.31	-0.40
Dcaf8	chr1	174078175	174078475	-2.57	-0.67
Dcaf8	chr1	174079403	174103118	-5.58	-0.32
Dcaf8	chr1	174124786	174125087	-0.67	-0.13
Pigm	chr1	174306661	174308487	-3.05	0.17
Parp1	chr1	182499105	182503813	-1.96	-0.45
Mrpl15	chr1	4766437	4766796	-0.89	-0.11
Cops5	chr1	10027829	10027979	-1.20	-0.16
Uggt1	chr1	36200615	36203553	-2.05	0.15
Uggt1	chr1	36300966	36301147	-1.89	-0.42
Lman2l	chr1	36480210	36481260	-8.25	-0.53
Mgat4a	chr1	37501090	37501720	-2.18	0.31
Chst10	chr1	38920747	38922995	-1.47	-0.11
Satb2	chr1	56852645	56854050	-1.14	0.10
Satb2	chr1	56928445	56928625	-1.56	-0.30
Satb2	chr1	57027968	57028178	-2.65	-0.59
Sumo1	chr1	59696455	59697048	-0.33	-0.01
Ino80d	chr1	63104148	63108707	-0.37	-0.01
Ino80d	chr1	63121010	63140028	-2.38	-0.17
Bard1	chr1	71121361	71122020	-1.25	-0.38
Usp37	chr1	74485767	74485858	-0.67	-0.28
Tuba4a	chr1	75211607	75212025	-1.98	0.61
Tuba4a	chr1	75212113	75215828	-13.90	-0.27
Scg2	chr1	79432859	79433100	-0.81	4.46
Cul3	chr1	80263601	80265851	-6.24	0.34
Cul3	chr1	80336915	80337005	-0.92	-0.29
Hdac4	chr1	93829331	93829960	-0.46	0.09
St8sia4	chr1	97487698	97523934	-12.00	0.49
St8sia4	chr1	97523993	97550125	-0.30	0.00
St8sia4	chr1	97563781	97564171	-0.35	0.02

Ikbke	chr1	133151178	133153534	-4.45	-0.31
Ikbke	chr1	133172403	133173518	-2.95	-0.52
Mdm4	chr1	134888236	134893148	-1.07	-0.08
Rnf2	chr1	153318438	153320426	-0.40	0.03
Lamc1	chr1	155068083	155074682	-60.80	-0.72
Lamc1	chr1	155080624	155081598	-3.02	-0.51
Lamc1	chr1	155179317	155179737	-8.10	-0.55
Qsox1	chr1	157626418	157628411	-6.98	-0.66
Klhl20	chr1	163019017	163019228	-1.31	-0.43
Klhl20	chr1	163020426	163023835	-2.61	0.52
Klhl20	chr1	163039357	163039897	-3.74	-0.46
Dcaf6	chr1	167281092	167281331	-1.18	-0.39
Uap1	chr1	172072402	172072642	-3.37	0.76
Uap1	chr1	172096797	172105047	-0.80	-0.15
Atf6	chr1	172639771	172677270	-5.29	0.28
Usp21	chr1	173216496	173218062	-11.50	-1.09
Chml	chr1	177616085	177617585	-4.84	0.48
Chml	chr1	177617764	177618484	-4.49	0.64
Dtl	chr1	193363098	193363969	-1.56	0.77
Dtl	chr1	193364536	193365016	-0.36	0.06
Kin	chr2	10013317	10014328	-0.32	-0.01
Stam	chr2	14067864	14068522	-0.94	0.13
Commd3	chr2	18596490	18597638	-0.74	-0.08
Bmi1	chr2	18605667	18606468	-0.37	-0.01
Man1b1	chr2	25188382	25188990	-22.80	3.20
Fbxw5	chr2	25358984	25359776	-3.57	-0.40
Fbxw5	chr2	25360013	25360422	-1.07	-0.24
Rxra	chr2	27615158	27615398	-3.09	-0.87
Rxra	chr2	27615487	27616386	-13.50	-1.14
Rxra	chr2	27617342	27618510	-7.46	-0.74
Mrps2	chr2	28324898	28325708	-3.10	-0.19
Ttf1	chr2	28915782	28922656	-2.19	-0.19
Ttf1	chr2	28941735	28942005	-1.37	-0.42
Dolpp1	chr2	30252653	30254975	-1.91	-0.41
Usp20	chr2	30865719	30865840	-3.26	-1.16
Nup214	chr2	31888785	31893623	-1.70	-0.12
Pomt1	chr2	32109927	32110226	-0.88	-0.25
Golga2	chr2	32158521	32160180	-1.33	-0.18
Golga2	chr2	32161974	32162244	-0.31	-0.01
Dpm2	chr2	32427908	32429091	-7.50	-0.39
St6galnac4	chr2	32451235	32451416	-2.40	0.71
St6galnac4	chr2	32452522	32453780	-6.35	-0.35
Mrrf	chr2	36044722	36045143	-0.33	-0.02

Mbd5	chr2	49101193	49101313	-0.37	-0.10
Mbd5	chr2	49111746	49113872	-0.35	-0.01
Mbd5	chr2	49134386	49169197	-1.26	0.48
Mbd5	chr2	49171947	49172008	-0.60	1.30
Klhl41	chr2	69508683	69508923	-1.52	0.86
Dcaf17	chr2	70928077	70928138	-0.87	0.71
Dync1i2	chr2	71049792	71050012	-0.90	0.33
Psmc3	chr2	90894439	90895182	-3.33	-0.20
Traf6	chr2	101530344	101537929	-2.95	-0.21
Rtf1	chr2	119558176	119559166	-0.81	0.09
Rtf1	chr2	119560243	119560484	-0.48	0.12
Pdia3	chr2	121263353	121263680	-0.63	0.03
Usp8	chr2	126567877	126568387	-5.37	-0.31
Mrps26	chr2	130389638	130391130	-7.95	-0.31
Prnd	chr2	131735723	131776618	-0.32	0.05
Csnk2a1	chr2	152107019	152107169	-10.00	-0.77
Tbc1d20	chr2	152119607	152127898	-2.40	-0.40
Tbc1d20	chr2	152137106	152137759	-2.58	-0.27
Asxl1	chr2	153171874	153177942	-0.65	-0.21
Asxl1	chr2	153222520	153228307	-3.63	-0.12
Actr5	chr2	158450648	158451037	-2.64	-0.85
Actr5	chr2	158462488	158464947	-0.38	0.05
Top1	chr2	160471632	160471932	-2.62	-0.31
Top1	chr2	160547243	160547394	-2.75	0.37
Ctsa	chr2	164659257	164659646	-12.00	-0.34
Rae1	chr2	172840787	172841203	-9.79	-0.73
Rab22a	chr2	173521497	173526997	-5.09	-0.36
Tubb1	chr2	174282485	174283323	-0.40	0.53
Adrm1	chr2	179906292	179906906	-2.50	-0.41
Arfgap1	chr2	180714403	180717020	-14.60	-0.46
Gata3	chr2	9779597	9784722	-2.88	0.70
Gata3	chr2	9790440	9796250	-0.57	-0.22
Mrpl41	chr2	24829604	24830588	-0.36	0.02
Tubb4b	chr2	25077825	25079655	-27.50	-0.32
Tubb4b	chr2	25079713	25080192	-0.32	0.02
Traf2	chr2	25374427	25375145	-2.44	-0.26
Sec16a	chr2	26265370	26265551	-1.04	-0.25
Sec16a	chr2	26265610	26266241	-5.32	-0.43
Sec16a	chr2	26277717	26279943	-0.47	-0.05
Sec16a	chr2	26294102	26294703	-2.30	0.19
Sec16a	chr2	26295032	26300736	-0.84	0.04
Ntng2	chr2	29062977	29103560	-0.46	-0.14
Dolk	chr2	30139748	30140407	-0.70	-0.13

Dolk	chr2	30140646	30141874	-2.09	-0.30
Asb6	chr2	30678647	30680212	-9.57	-0.39
Fbxw2	chr2	34661048	34661869	-4.78	-0.39
Fbxw2	chr2	34662764	34664040	-0.64	-0.16
Fbxw2	chr2	34668436	34678672	-6.10	-0.35
Fbxw2	chr2	34681468	34681725	-0.46	0.13
Psmd5	chr2	34707993	34709823	-1.65	-0.19
Psmd5	chr2	34726274	34726482	-4.41	-0.82
Rab14	chr2	35056311	35056550	-6.69	-0.39
Pdcl	chr2	37207111	37212852	-9.76	0.42
Pdcl	chr2	37214702	37214852	-6.52	1.97
Psmb7	chr2	38498946	38499426	-3.13	-0.36
Ifih1	chr2	62472979	62473100	-1.19	0.92
Ifih1	chr2	62477495	62484252	-1.48	0.23
Galnt3	chr2	65945049	65962670	-1.01	0.32
Sp3	chr2	72775988	72779227	-8.58	0.42
Sp3	chr2	72784602	72809399	-22.20	0.39
Slc35c1	chr2	92293995	92294175	-2.01	-0.48
Slc35c1	chr2	92294234	92294832	-3.36	-0.37
Slc35c1	chr2	92298872	92299589	-0.36	0.02
Rcn1	chr2	105227977	105229255	-5.40	-0.46
Rcn1	chr2	105239207	105239446	-1.42	-0.39
Ino80	chr2	119200037	119200278	-1.42	0.29
Trp53bp1	chr2	121030697	121033675	-0.56	-0.10
Trp53bp1	chr2	121034169	121046975	-2.75	-0.26
Trp53bp1	chr2	121061313	121069657	-0.65	0.06
Cops2	chr2	125657568	125660555	-1.53	-0.10
Pcna	chr2	132077710	132078916	-7.08	0.28
Psmf1	chr2	151543111	151543202	-1.31	-0.87
Psmf1	chr2	151543649	151544038	-0.30	0.01
Edem2	chr2	155527795	155528302	-2.18	-0.22
Neurl2	chr2	164656229	164658385	-0.74	0.13
B4galt5	chr2	167124332	167124601	-0.54	-0.04
B4galt5	chr2	167126508	167130515	-27.30	-0.43
B4galt5	chr2	167174439	167174678	-8.54	-1.08
Ctsz	chr2	174252994	174254182	-128.00	-0.34
Sec62	chr3	30692244	30692305	-0.33	-0.06
Sec62	chr3	30713176	30718068	-5.06	-0.24
Usp13	chr3	32832089	32832389	-0.75	3.43
Rab33b	chr3	51288366	51298069	-4.15	0.43
Gfm1	chr3	67278519	67278960	-2.63	0.33
Fbxw7	chr3	84619498	84694070	-0.58	-0.12
Fbxw7	chr3	84694129	84756226	-0.51	0.05

Fbxw7	chr3	84780110	84781955	-0.35	0.02
Muc1	chr3	89033855	89035018	-25.00	2.71
Rab13	chr3	90028667	90029826	-0.48	0.13
Mrpl9	chr3	94251762	94252630	-0.63	-0.05
Otud7b	chr3	95908449	95908540	-1.47	-0.65
Otud7b	chr3	95956546	95960193	-6.78	-0.26
Hist2h2be	chr3	96025043	96025490	-2.37	-0.78
Hist2h2be	chr3	96026708	96027215	-0.64	-0.19
Hist2h2be	chr3	96027422	96027483	-10.00	3.73
Sec22b	chr3	97705149	97711158	-2.16	-0.33
Sec22b	chr3	97725110	97725468	-2.27	-0.20
Psma5	chr3	108059873	108060022	-0.64	3.39
Dph5	chr3	115591100	115591191	-0.73	0.31
Dph5	chr3	115631611	115632001	-0.66	-0.12
Ube2d3	chr3	135101656	135102228	-19.60	-0.75
Bcl10	chr3	145587232	145587592	-4.99	-0.42
Bcl10	chr3	145588099	145593556	-3.89	-0.41
Usp33	chr3	152031228	152033363	-11.50	-0.53
Pigk	chr3	152448420	152449109	-0.30	-0.01
Pigk	chr3	152449855	152450483	-0.83	0.17
Pigk	chr3	152451617	152451828	-0.44	0.13
Pex2	chr3	5561142	5563166	-2.98	-0.30
Phc3	chr3	30805887	30813151	-0.72	-0.12
Phc3	chr3	30864733	30868337	-1.81	-0.73
Ccna2	chr3	36470827	36470918	-0.54	-0.57
Commd2	chr3	57450273	57450573	-1.50	-0.35
Serp1	chr3	58329387	58329687	-14.40	-0.47
Siah2	chr3	58479649	58495381	-3.84	-0.27
Siah2	chr3	58495620	58496010	-0.94	0.28
Dap3	chr3	88726545	88726725	-0.26	0.00
Dap3	chr3	88727202	88727531	-1.42	-0.51
Psmb4	chr3	94690701	94690880	-8.50	-0.41
Psmd4	chr3	94836626	94836894	-2.54	-0.16
Psmd4	chr3	94840526	94846467	-8.21	-0.35
Man1a2	chr3	100371041	100382434	-0.59	0.05
Man1a2	chr3	100460000	100489396	-0.70	-0.04
Csf1	chr3	107544832	107548597	-0.68	0.16
Csf1	chr3	107548745	107550736	-0.68	-0.96
Csf1	chr3	107551093	107553341	-0.96	0.24
Gnai3	chr3	107911672	107912208	-1.72	0.11
Gnai3	chr3	107948823	107949032	-5.04	-0.38
Rwdd3	chr3	120859364	120862119	-0.35	-0.04
Ank2	chr3	126625933	126626054	-0.57	-1.42

Ank2	chr3	126626293	126626744	-0.78	0.77
Sec24b	chr3	129686102	129687101	-1.82	-0.14
Sec24b	chr3	129743865	129763735	-15.50	-0.56
Fpgt	chr3	154749523	154753147	-2.83	0.28
Rab2a	chr4	8462790	8462910	-1.92	-0.42
Fbxl4	chr4	22303713	22313289	-1.60	-0.28
Map3k7	chr4	32051081	32051232	-3.02	-1.01
Map3k7	chr4	32104109	32107267	-5.97	0.38
Map3k7	chr4	32110051	32110231	-0.27	0.00
Ube2r2	chr4	41137807	41138136	-0.41	-0.03
Creb3	chr4	43575505	43575715	-2.17	-0.41
Creb3	chr4	43579692	43579932	-0.44	0.08
Dcaf10	chr4	45355032	45355482	-3.02	-0.62
Dcaf10	chr4	45385889	45387298	-0.47	-0.06
Nans	chr4	46515563	46516280	-1.13	-0.18
Tgfbr1	chr4	47419877	47423777	-9.55	1.12
Stx17	chr4	48197791	48198211	-1.94	-0.55
Rad23b	chr4	55398385	55402936	-0.38	-0.01
Rraga	chr4	86221606	86222293	-1.18	-0.09
Usp24	chr4	105988817	105989117	-2.50	-0.67
Usp24	chr4	106111387	106111867	-5.72	-0.38
Usp24	chr4	106112884	106113842	-1.36	0.15
Ndc1	chr4	107083754	107086943	-1.16	0.18
Lrrc41	chr4	115761466	115762183	-23.20	-0.55
Lrrc41	chr4	115768961	115769201	-3.73	-0.33
Trappc3	chr4	125939648	125949998	-0.36	-0.03
Trappc3	chr4	125951220	125952918	-0.98	0.08
Clspn	chr4	126237254	126239019	-2.42	-0.81
Clspn	chr4	126243253	126249609	-1.43	-0.48
Clspn	chr4	126270339	126270758	-2.44	-0.82
Phc2	chr4	128404910	128405061	-0.82	-0.46
Phc2	chr4	128424470	128429198	-18.30	-0.40
Phc2	chr4	128429466	128429976	-16.20	-0.47
Ptp4a2	chr4	129497464	129516421	-0.57	0.05
Fuca1	chr4	135476640	135476908	-6.65	-0.76
Fuca1	chr4	135495027	135495325	-0.87	0.15
Usp48	chr4	137212056	137213554	-0.56	0.05
Mul1	chr4	137990586	137996001	-8.75	-0.48
Mul1	chr4	137996358	137996837	-1.10	-0.33
Capzb	chr4	138748845	138794826	-3.32	-0.32
Klhl21	chr4	151383209	151383749	-20.10	-1.89
Klhl21	chr4	151383868	151389959	-10.20	-0.52
Klhl21	chr4	151390257	151390438	-3.61	1.07

Icmt	chr4	151674797	151677875	-6.72	-0.48
Mrpl20	chr4	155182648	155182938	-2.44	-0.26
Ripk2	chr4	16050670	16051621	-4.17	-0.41
Ripk2	chr4	16090436	16090645	-16.00	-1.44
Manea	chr4	26254667	26273799	-1.17	0.13
Ddx58	chr4	40152603	40155547	-1.90	-0.20
Ddx58	chr4	40186678	40186858	-0.44	0.12
Topors	chr4	40206906	40216844	-74.50	0.55
B4galt1	chr4	40753786	40754830	-1.36	0.12
B4galt1	chr4	40770597	40800613	-1.85	0.25
B4galt1	chr4	40800671	40801001	-2.65	-0.48
Gne	chr4	44047905	44048266	-2.53	-0.83
Gne	chr4	44049704	44051897	-7.07	-0.91
Gne	chr4	44068129	44079645	-3.41	-0.45
Fbxo10	chr4	45048045	45048374	-1.12	1.75
Fbxo10	chr4	45048642	45048793	-0.22	0.14
Fbxo10	chr4	45071323	45071981	-0.27	-0.03
Alg2	chr4	47482973	47483543	-1.56	0.21
Mrpl50	chr4	49526445	49527334	-2.11	-0.20
Klhl9	chr4	88366049	88367814	-9.57	0.24
Mysm1	chr4	94631863	94632462	-1.59	0.20
Mrpl37	chr4	106737372	106739471	-25.00	-0.87
Dph2	chr4	117562115	117562176	-0.32	0.08
Dph2	chr4	117562463	117563268	-5.52	-0.58
Dph2	chr4	117564417	117564628	-0.41	-0.10
St3gal3	chr4	117604757	117612678	-1.17	-0.15
Cdc20	chr4	118109887	118109948	-0.76	-0.68
Wdtc1	chr4	132849782	132850290	-1.99	-0.39
Pigv	chr4	133218171	133220780	-1.21	0.27
Dhdds	chr4	133526737	133536084	-0.70	-0.07
Man1c1	chr4	134119221	134119671	-0.57	0.09
Man1c1	chr4	134196206	134260205	-5.72	-0.33
Otud3	chr4	138451293	138453499	-0.36	0.02
Pex14	chr4	148335151	148335868	-1.28	-0.14
Pex14	chr4	148358178	148473921	-0.44	-0.05
Spsb1	chr4	149271916	149281307	-0.79	-0.50
Park7	chr4	150271241	150271562	-12.30	-0.42
Vwa1	chr4	155147147	155147208	-0.18	0.74
Nupl2	chr5	23687726	23687877	-0.80	0.38
Nupl2	chr5	23687966	23689616	-1.38	-0.19
Nub1	chr5	24191781	24195199	-0.68	-0.33
Nub1	chr5	24216043	24216194	-0.77	-0.21
Galnt11	chr5	24771112	24771321	-1.25	-0.40

Bst1	chr5	44210131	44210281	-0.40	-0.14
Klhl5	chr5	65558211	65558721	-0.75	-0.12
Dcun1d4	chr5	73950743	73951343	-0.46	0.13
Exoc1	chr5	76998801	76999310	-1.61	-0.30
Uso1	chr5	92630205	92631431	-0.46	-0.02
Art3	chr5	92821553	92821820	-0.86	2.58
Spp1	chr5	104864129	104865280	-280.00	-0.71
Pigg	chr5	108776899	108777230	-2.37	-0.73
Usp30	chr5	114572362	114572933	-6.41	-0.79
Rab35	chr5	116093492	116096664	-1.72	-0.10
Wsb2	chr5	117827463	117828091	-9.87	-0.54
Fbxo21	chr5	118452063	118458618	-6.36	-0.76
Psmd9	chr5	123678228	123678437	-0.34	0.03
Mrps17	chr5	130224057	130224357	-1.58	0.26
Sumf2	chr5	130338841	130339081	-1.81	-0.86
Kctd7	chr5	130628217	130628785	-0.53	-0.24
Vgf	chr5	137506930	137509043	-3.19	-0.55
Ache	chr5	137731538	137731808	-1.21	-1.77
Ache	chr5	137732750	137733199	-0.89	-0.55
Cops6	chr5	138605188	138605278	-0.36	-0.10
Fam20c	chr5	139231034	139232081	-22.20	-0.41
Fam20c	chr5	139284018	139286017	-18.70	-0.53
Eif3b	chr5	140895258	140895738	-21.50	-0.43
Foxk1	chr5	142932437	142934770	-0.93	-0.07
Kdelr2	chr5	144165498	144165618	-1.27	-0.30
Trrap	chr5	145550403	145551932	-3.37	-0.52
Trrap	chr5	145618437	145620522	-11.00	-0.41
Preb	chr5	31254099	31254460	-1.14	0.42
Preb	chr5	31254638	31254759	-0.75	0.43
Preb	chr5	31255148	31255718	-1.31	0.28
Preb	chr5	31256197	31256558	-0.80	0.21
Preb	chr5	31257096	31257217	-0.42	-0.16
Preb	chr5	31257366	31258308	-13.30	-0.41
Preb	chr5	31262403	31262704	-7.96	-0.81
Ctbp1	chr5	33590909	33591269	-25.10	-0.66
Tnip2	chr5	34842175	34842385	-0.32	-0.02
Tada2b	chr5	36818228	36826725	-1.61	-0.17
Wfs1	chr5	37358091	37358361	-1.79	-0.98
Wfs1	chr5	37358629	37359738	-0.65	-0.17
Fbxl5	chr5	44142150	44149098	-2.88	-0.56
Fbxl5	chr5	44149396	44151973	-0.84	0.08
Commd8	chr5	72550577	72551087	-5.42	0.48
Nup54	chr5	92845130	92846543	-1.18	-0.16

Sec31a	chr5	100790907	100791058	-0.72	-0.09
Abraxas1	chr5	101234923	101235430	-2.15	-0.39
Mlec	chr5	115597834	115600228	-0.61	-0.05
Mlec	chr5	115600898	115608185	-12.50	-0.43
Suds3	chr5	117542584	117542973	-0.58	-0.07
Suds3	chr5	117556655	117565972	-3.08	0.27
Fbxw8	chr5	118515497	118515797	-0.28	0.02
Fbxw8	chr5	118605107	118605467	-13.80	-1.01
Eif2b1	chr5	125020639	125021791	-0.64	0.06
Ubc	chr5	125866453	125867671	-172.00	0.73
Ubc	chr5	125867938	125868147	-3.15	0.20
Ubc	chr5	125868235	125870298	-22.80	-0.40
Pom121	chr5	135853620	135857139	-7.02	-0.36
Pom121	chr5	135857525	135857795	-2.51	-0.32
Gnb2	chr5	137972009	137974457	-9.62	-0.57
Fbxl18	chr5	143633766	143634006	-6.05	-1.06
Fbxl18	chr5	143647153	143648949	-184.00	-2.23
Usp42	chr5	144471582	144472847	-0.29	-0.01
Usp42	chr5	144474632	144476980	-6.09	-0.41
Usp42	chr5	144477517	144477978	-4.07	-0.78
Usp12	chr5	147548897	147550725	-1.72	-0.27
C1galt1	chr6	7816416	7821773	-6.24	0.33
Calu	chr6	29298148	29306501	-0.80	-0.24
Calu	chr6	29322584	29324851	-2.79	-0.14
Exoc4	chr6	33921942	33922332	-2.74	0.36
Nup205	chr6	35127615	35133895	-0.30	0.02
Rab19	chr6	39339614	39340231	-1.34	-0.57
Cul1	chr6	47404345	47445165	-3.56	0.31
Cul1	chr6	47474617	47476108	-1.64	-0.15
Tnip3	chr6	65564734	65582063	-0.35	0.01
Eif2ak3	chr6	70840206	70843273	-1.46	-0.24
Eif2ak3	chr6	70843421	70850228	-0.78	0.31
Eif2ak3	chr6	70854128	70854726	-2.04	-0.36
Rnf103	chr6	71458868	71460366	-1.54	0.19
St3gal5	chr6	72092231	72097086	-1.87	-0.24
St3gal5	chr6	72099116	72104266	-2.73	0.22
Mogs	chr6	83067105	83068743	-16.50	-0.34
Dctn1	chr6	83140226	83141067	-0.45	0.03
Cct7	chr6	85402007	85402187	-1.02	-0.19
Kbtbd8	chr6	95071991	95077327	-2.49	0.37
Mitf	chr6	97960423	97968956	-1.11	-0.08
Mitf	chr6	97969584	97970094	-0.70	-0.09
Edem1	chr6	108778634	108779024	-6.01	-0.33

Edem1	chr6	108805488	108805668	-3.63	-0.20
Ttll3	chr6	113357264	113357355	-1.22	0.45
Ttll3	chr6	113358988	113359318	-0.96	-0.26
Ttll3	chr6	113362865	113364057	-1.14	-0.28
Ttll3	chr6	113364206	113364505	-4.11	-0.73
Vhl	chr6	113578147	113579978	-0.46	-0.06
Fbxl14	chr6	119429685	119431451	-0.36	-0.01
Rad52	chr6	119865354	119869071	-3.47	-0.44
Rad52	chr6	119870988	119871109	-0.75	0.26
Rad52	chr6	119871288	119872841	-0.35	0.03
Spsb2	chr6	124758812	124760455	-90.80	-1.86
Mrpl51	chr6	125143307	125143546	-2.74	-0.26
Cmas	chr6	142705235	142705504	-2.29	-0.47
Mrps35	chr6	147019097	147019424	-1.38	-0.25
Klhl42	chr6	147040014	147050109	-0.97	-0.28
Klhl42	chr6	147050168	147056940	-3.38	-0.59
Etfbkmt	chr6	149092586	149092945	-0.59	-0.44
Etfbkmt	chr6	149095803	149099215	-2.26	-1.34
Ube2h	chr6	30163827	30164395	-1.91	0.24
Ube2h	chr6	30164783	30164933	-0.40	-0.15
Hipk2	chr6	38647898	38665995	-46.30	-0.52
Hipk2	chr6	38767930	38787077	-4.22	-0.11
Hipk2	chr6	38825800	38826040	-0.40	-0.04
Nod1	chr6	54874531	54874682	-0.75	1.04
Nod1	chr6	54893168	54893616	-0.68	0.56
Nod1	chr6	54893675	54894034	-0.75	0.80
Nod1	chr6	54894242	54894451	-0.24	-0.71
Nod1	chr6	54894600	54897981	-1.54	1.88
Ptcd3	chr6	71830841	71831536	-2.10	-0.25
Rnf181	chr6	72310239	72310565	-2.88	-0.36
Tgoln1	chr6	72561643	72566785	-5.10	-0.11
Mrpl19	chr6	81911638	81912147	-0.38	-0.02
Ino80b	chr6	83074730	83075023	-0.55	-0.20
Rab43	chr6	87742107	87744044	-5.29	-0.48
Rab43	chr6	87761473	87761773	-1.00	-0.35
Sec61a1	chr6	88454913	88455213	-5.26	-0.23
Nup210	chr6	90963060	90963660	-3.36	-0.50
Xpc	chr6	91439866	91440195	-0.47	-0.16
Xpc	chr6	91448159	91450008	-8.98	-0.94
Mrps25	chr6	92124773	92125723	-2.58	-0.28
Uba3	chr6	97134556	97134676	-1.20	0.47
Sumf1	chr6	108058030	108058509	-1.48	0.21
Sumf1	chr6	108135337	108135577	-0.45	-0.07

Rad18	chr6	112577061	112577661	-0.87	-0.24
Tada3	chr6	113316892	113320261	-0.78	0.17
Tada3	chr6	113322347	113324488	-2.13	0.54
Tada3	chr6	113324725	113325142	-2.16	0.45
Phc1	chr6	122273438	122273618	-0.97	0.44
Phc1	chr6	122275560	122275830	-0.61	-0.25
Pex5	chr6	124347220	124347399	-0.29	-0.01
Usp5	chr6	124765306	124767020	-15.00	-0.58
Usp5	chr6	124776424	124779465	-1.09	-0.24
Napa	chr7	16702038	16703324	-5.35	-0.18
Ccdc8	chr7	17580174	17580295	-0.16	0.42
Trappc6a	chr7	20094077	20099776	-2.15	-0.28
Plaur	chr7	25247518	25251694	-110.00	-1.93
Plaur	chr7	25260294	25260713	-183.00	-1.49
B3gnt8	chr7	26415220	26415521	-5.92	-0.75
Paf1	chr7	29178014	29178133	-0.41	-0.07
Paf1	chr7	29181953	29184402	-0.48	0.02
Josd2	chr7	51723425	51723545	-0.29	-0.02
Napsa	chr7	51827934	51827995	-0.89	-0.42
Nup62	chr7	52071739	52086176	-19.00	-0.23
Hrc	chr7	52590638	52591029	-0.75	-0.98
Hrc	chr7	52591478	52591689	-0.64	0.91
Prmt3	chr7	57112858	57113008	-1.15	-0.24
Herc2	chr7	63485072	63486928	-1.61	0.14
Klhl25	chr7	82993313	83012167	-0.30	0.00
Klhl25	chr7	83012375	83018269	-2.48	-0.60
Klhl25	chr7	83018657	83018926	-0.33	0.09
Mrps11	chr7	85936812	85937724	-2.34	-0.39
Ctsc	chr7	95437245	95437485	-0.96	0.45
Ctsc	chr7	95458037	95458547	-0.94	0.12
Rab30	chr7	99984250	99984670	-0.49	-0.72
Alg8	chr7	104540130	104540578	-2.19	-0.39
Arrb1	chr7	106748328	106749813	-0.89	-0.21
Arrb1	chr7	106752471	106752711	-1.70	-0.44
Usp17lc	chr7	110567241	110567301	-0.11	0.00
Ctr9	chr7	118173736	118177414	-3.03	-0.38
Ctr9	chr7	118198901	118199621	-1.34	-0.16
Usp47	chr7	119167079	119167140	-0.41	0.13
Usp47	chr7	119234919	119245370	-0.77	-0.05
Usp47	chr7	119252192	119254002	-1.50	-0.16
Spon1	chr7	121183502	121183922	-0.45	0.48
Ccp110	chr7	125864970	125865449	-1.59	-0.38
Ccp110	chr7	125865867	125867971	-0.97	-0.19

Dctn5	chr7	129276554	129277689	-0.48	-0.08
Dctn5	chr7	129291791	129292440	-0.33	-0.01
Rnf40	chr7	134739594	134740336	-9.26	-0.41
Rnf40	chr7	134744053	134746671	-2.53	-0.16
Fbxl19	chr7	134890288	134891744	-4.33	-0.90
Fbxl19	chr7	134891803	134891893	-0.66	-0.35
Fbxl19	chr7	134893270	134904820	-11.30	-0.67
Fbxl19	chr7	134906111	134911817	-1.15	-0.18
Fbxl19	chr7	134911876	134912442	-2.21	-0.45
Sec23ip	chr7	135888443	135893982	-17.50	-0.53
Abraxas2	chr7	140072616	140072677	-0.76	0.42
Abraxas2	chr7	140072975	140075538	-1.28	0.12
Zranb1	chr7	140122884	140158368	-4.10	-0.27
Zranb1	chr7	140174515	140175634	-0.78	-0.16
Zranb1	chr7	140175516	140175963	-0.36	-0.03
Pnpla2	chr7	148641086	148641236	-0.31	0.02
Pnpla2	chr7	148645626	148645985	-2.36	-0.30
Tfpt	chr7	3581426	3581486	-1.90	-0.92
Ppp6r1	chr7	4610133	4610552	-20.20	-0.92
Ube2m	chr7	13623018	13623619	-6.02	-0.45
Sae1	chr7	16912835	16913105	-0.93	-0.11
Calm3	chr7	17502039	17502309	-1.46	-0.09
Apoe	chr7	20282098	20282861	-0.83	-0.19
Ceacam1	chr7	26247739	26248638	-4.67	0.32
Ceacam1	chr7	26257014	26258919	-1.16	-0.37
Ceacam1	chr7	26259008	26274241	-0.36	-0.02
Sptbn4	chr7	28149267	28149508	-0.42	1.58
Mrps12	chr7	29524778	29525105	-0.68	-0.12
Psmd8	chr7	29965393	29965573	-13.80	-0.43
Nucb1	chr7	52758551	52762906	-4.53	-0.38
Nucb1	chr7	52765032	52765778	-4.41	-0.49
Nsmce3	chr7	72016624	72017749	-1.96	-0.18
Asb7	chr7	73792534	73823980	-3.67	-0.42
Asb7	chr7	73834207	73834387	-0.58	0.31
Mrpl46	chr7	85920226	85920609	-2.05	-0.29
Mfge8	chr7	86278653	86279192	-3.32	-0.24
Man2a2	chr7	87494918	87495788	-3.80	0.21
Man2a2	chr7	87496117	87497911	-1.12	0.09
Man2a2	chr7	87515749	87516012	-0.66	-0.32
Furin	chr7	87534797	87536644	-178.00	-0.71
Furin	chr7	87538324	87540367	-66.60	-0.82
Furin	chr7	87542790	87550317	-43.80	-0.68
Blm	chr7	87647292	87647681	-3.04	-0.71

Blm	chr7	87654437	87658369	-0.32	-0.01
Neu3	chr7	106961469	106962782	-1.93	0.47
P2ry2	chr7	108145858	108160527	-13.60	-0.44
Nup98	chr7	109268003	109269291	-7.87	-0.22
Nup98	chr7	109283070	109283401	-23.60	-0.89
Usp17ld	chr7	110398718	110398868	-0.21	-0.17
Usp17ld	chr7	110399373	110399493	-0.18	0.68
Usp17lb	chr7	111989694	111989755	-0.14	0.42
Mrpl17	chr7	112958202	112958592	-6.25	-0.27
Dcun1d3	chr7	127000827	127038861	-10.40	-0.47
Cog7	chr7	129066679	129066829	-1.49	-0.61
Cog7	chr7	129121227	129125207	-0.94	-0.20
Nsmce4a	chr7	137690685	137690865	-3.11	-1.21
Fuom	chr7	147285428	147285765	-5.94	-1.24
Bet1l	chr7	148039342	148042252	-24.40	-0.67
Ctsd	chr7	149561820	149562294	-118.00	-0.34
Trappc5	chr8	3676506	3679785	-6.86	-0.39
Trappc5	chr8	3680083	3680622	-2.35	-0.40
F7	chr8	13034729	13035419	-0.25	-0.01
Cul4a	chr8	13144401	13146773	-0.97	0.16
Cul4a	chr8	13146832	13147072	-0.47	0.07
Thsd1	chr8	23353920	23354367	-0.35	-0.59
Thsd1	chr8	23369018	23369287	-2.72	-4.39
Ank1	chr8	24242642	24242942	-0.36	-0.31
Ank1	chr8	24259141	24259292	-0.17	-0.12
Fkbp8	chr8	73051641	73051859	-1.76	-0.42
Fkbp8	chr8	73051918	73053571	-4.53	-0.26
Fkbp8	chr8	73058839	73058959	-6.16	-0.46
Rab3a	chr8	73282404	73282465	-0.44	0.34
Babam1	chr8	73920753	73921648	-6.08	-0.54
Mrpl34	chr8	73988824	73989652	-0.57	0.05
Dda1	chr8	73998657	73999436	-3.49	-0.18
Dda1	chr8	73999764	73999855	-0.88	0.22
Rab8a	chr8	74704051	74704674	-6.42	-0.29
Nr3c2	chr8	79432403	79432554	-0.61	1.74
Nr3c2	chr8	79433567	79433776	-0.74	-1.52
Gadd45gip1	chr8	87356270	87358724	-39.80	-0.82
Dhps	chr8	87595655	87595834	-0.47	0.12
Cyld	chr8	91220956	91226218	-0.50	0.37
Cyld	chr8	91226307	91233793	-2.31	0.21
Mmp2	chr8	95376573	95376783	-0.84	-0.51
Mmp2	chr8	95377050	95377319	-0.44	-0.17
Fbxl8	chr8	107790925	107791573	-0.65	0.30

Fbxl8	chr8	107791922	107793108	-0.55	0.10
Cog4	chr8	113370709	113371100	-5.20	-1.02
Cog4	chr8	113405654	113406134	-2.02	-0.31
St3gal2	chr8	113444003	113491411	-1.25	-0.16
St3gal2	chr8	113493699	113495173	-5.29	-0.56
Gan	chr8	119728191	119729086	-7.70	0.65
Usp10	chr8	122460483	122471049	-1.73	0.10
Usp10	chr8	122480587	122481247	-0.46	-0.03
Trappc2l	chr8	125135525	125136985	-0.45	-0.17
Tubb3	chr8	125942898	125945049	-0.53	-0.56
Galnt2	chr8	126867222	126868362	-0.87	-0.10
Rab20	chr8	11453976	11478499	-58.40	-1.28
Dcun1d2	chr8	13256291	13257907	-1.09	-0.11
Gas6	chr8	13465611	13465821	-0.49	-0.15
Vdac3	chr8	23687812	23688050	-0.92	0.16
Pomk	chr8	27092801	27100129	-0.41	-0.02
Dctn6	chr8	35169435	35171565	-0.29	-0.02
Ing2	chr8	48753850	48758943	-1.32	0.15
Ing2	chr8	48760272	48760513	-0.64	-0.35
Galnt7	chr8	60004507	60010487	-11.50	0.30
Galnt7	chr8	60062627	60131852	-20.80	-0.39
Klhl2	chr8	67219391	67219870	-0.73	-0.15
Ano8	chr8	74000246	74000426	-1.28	-1.09
Ano8	chr8	74000574	74000814	-0.40	0.31
Ano8	chr8	74003128	74005058	-7.55	-0.71
B3gnt3	chr8	74216002	74217291	-1.83	-0.34
B3gnt3	chr8	74217349	74227314	-0.35	0.05
Rad23a	chr8	87358550	87358701	-2.33	-0.85
Calr	chr8	87366315	87366863	-41.40	-0.26
Calr	chr8	87370234	87370800	-11.20	-0.26
Amfr	chr8	96496474	96497043	-0.34	-0.01
Csnk2a2	chr8	97976954	97977225	-0.35	-0.03
Dync1li2	chr8	106944120	106944421	-2.39	-0.28
Dync1li2	chr8	106966646	106966737	-0.40	0.11
B3gnt9	chr8	107777431	107779051	-4.70	0.62
Psmb10	chr8	108461631	108461967	-2.80	-0.63
Cog8	chr8	109570548	109572735	-0.43	0.03
Cog8	chr8	109572794	109572975	-4.23	-0.66
Cog8	chr8	109576156	109578097	-1.38	-0.12
Psmd7	chr8	110104338	110108077	-4.03	0.12
Mbtps1	chr8	122032081	122032770	-0.81	0.07
Mbtps1	chr8	122070114	122082661	-0.67	0.06
Fbxo31	chr8	124075878	124076147	-0.29	0.02

Mvd	chr8	124957554	124958775	-4.80	-0.29
Exoc8	chr8	127418726	127418906	-0.33	0.08
Exoc8	chr8	127419175	127421575	-0.62	0.07
Mrpl4	chr9	20812982	20813281	-1.14	-0.13
Pigyl	chr9	21961289	21962769	-0.41	0.06
Opcml	chr9	28210785	28210966	-0.62	-3.17
Opcml	chr9	28211054	28211415	-0.32	-1.17
Opcml	chr9	28729364	28729455	-0.20	-0.85
Opcml	chr9	28729664	28729784	-1.29	-3.17
Opcml	chr9	28730173	28730744	-1.82	1.53
Nfrkb	chr9	31218903	31227409	-0.77	0.09
Usp2	chr9	43883786	43884026	-0.81	2.12
Usp2	chr9	43902032	43902302	-0.96	-0.35
Dpagt1	chr9	44141235	44141504	-0.33	0.02
Apoa1	chr9	46038131	46038430	-0.42	1.51
Alg9	chr9	50651034	50651633	-0.34	0.02
Fbxo22	chr9	55066270	55071672	-1.24	0.12
Fbxo22	chr9	55071791	55072031	-0.53	0.10
Sin3a	chr9	56958532	56958892	-0.32	-0.02
Sin3a	chr9	56973115	56975576	-0.84	0.11
Rora	chr9	69226715	69226835	-0.32	0.16
Adam10	chr9	70526807	70526958	-1.07	0.28
Adam10	chr9	70626086	70626297	-2.80	0.46
Rab27a	chr9	72892731	72908786	-0.39	-0.66
Rab27a	chr9	72943089	72943780	-0.59	0.27
Leo1	chr9	75289330	75294926	-0.87	-0.07
Cish	chr9	107202782	107203759	-21.00	3.35
Tmem115	chr9	107436364	107440987	-2.51	0.14
Usp4	chr9	108293381	108294591	-3.30	-0.22
Usp19	chr9	108393096	108395299	-1.59	-0.15
Cdc25a	chr9	109778082	109778918	-5.19	-0.75
Cdc25a	chr9	109794596	109795525	-0.60	-0.08
Glb1	chr9	114310242	114326114	-1.32	0.33
Glb1	chr9	114368800	114373509	-0.47	-0.05
Glb1	chr9	114374377	114383495	-4.81	0.37
Dync1li1	chr9	114632686	114632895	-0.93	0.16
Dync1li1	chr9	114632812	114633189	-1.24	0.13
Wdr48	chr9	119833314	119835040	-0.42	0.02
Birc2	chr9	7818614	7818734	-0.55	-0.29
Birc2	chr9	7833610	7834657	-0.90	0.13
Birc2	chr9	7834955	7835195	-1.05	0.42
Birc3	chr9	7848969	7850928	-1.17	-0.17
Birc3	chr9	7860311	7872961	-0.30	0.00

Fbxl12	chr9	20448880	20449151	-0.85	-0.48
Keap1	chr9	21034473	21038380	-0.55	0.03
Keap1	chr9	21041615	21042276	-1.55	-0.22
Keap1	chr9	21043205	21043686	-0.40	0.13
Rab3d	chr9	21714741	21715042	-1.16	-0.47
Aplp2	chr9	30957469	30957679	-1.25	-0.13
Aplp2	chr9	30985646	31019400	-0.58	0.12
Arcn1	chr9	44551783	44551994	-1.44	0.13
Arcn1	chr9	44568266	44575891	-2.60	-0.34
Cul5	chr9	53425530	53429279	-5.02	0.34
Rab39	chr9	53494590	53494829	-0.25	-0.22
Pml	chr9	58066214	58066305	-1.33	0.78
Pml	chr9	58067472	58068881	-10.10	-0.47
Pml	chr9	58078383	58097563	-4.44	-0.27
Senp8	chr9	59584180	59587831	-0.78	0.14
Thsd4	chr9	59819164	59819314	-0.27	1.26
Fem1b	chr9	62643314	62659036	-48.90	0.46
Pias1	chr9	62729583	62729943	-0.50	0.10
Pias1	chr9	62799649	62828686	-0.80	0.12
Smad3	chr9	63496369	63496969	-0.55	0.19
Smad3	chr9	63497327	63498046	-0.81	0.32
Smad3	chr9	63605729	63605864	-0.51	-1.42
Usp3	chr9	66366163	66367920	-0.69	0.16
Rab8b	chr9	66695066	66696453	-10.50	0.31
Rab8b	chr9	66715068	66767482	-1.83	0.32
Pigb	chr9	72863505	72865261	-0.48	0.05
Pgm3	chr9	86447697	86448087	-3.55	-0.65
Mrps22	chr9	98498714	98502098	-0.83	-0.20
Gnai2	chr9	107516826	107517065	-17.60	0.37
Rnf123	chr9	107954002	107954243	-6.46	0.71
Apeh	chr9	107987744	107987894	-0.71	-0.29
Dag1	chr9	108109094	108155448	-8.64	0.23
Gorasp1	chr9	119834879	119835029	-0.52	-0.15
Gorasp1	chr9	119836695	119837112	-5.71	-0.94
Sec22c	chr9	121593476	121593866	-0.35	0.10
Mtrf1l	chr10	4532471	4533463	-1.43	-0.44
Shprh	chr10	10869257	10871943	-2.23	-0.37
Shprh	chr10	10882772	10884564	-4.44	-0.54
Fbxo30	chr10	11001127	11015345	-10.80	0.40
Fuca2	chr10	13223007	13225667	-1.77	-0.36
Fuca2	chr10	13234515	13235470	-1.15	0.10
Sec63	chr10	42548789	42548969	-4.11	-0.35
Nus1	chr10	52137562	52137682	-7.69	-0.70

Nus1	chr10	52156402	52156613	-4.96	-0.41
Nus1	chr10	52158170	52158350	-1.58	-0.34
Ranbp2	chr10	57939345	57940486	-6.91	0.22
Ranbp2	chr10	57940784	57941055	-0.33	-0.01
Ranbp2	chr10	57941564	57941955	-1.34	-0.09
Ranbp2	chr10	57942134	57945844	-28.50	0.35
Ranbp2	chr10	57955388	57956782	-8.30	0.31
Adora2a	chr10	74795982	74796103	-0.57	2.94
Adora2a	chr10	74796310	74796699	-0.49	1.14
Pofut2	chr10	76731353	76732331	-3.21	-0.17
Sumo3	chr10	77068978	77069512	-0.42	-0.07
Ube2g2	chr10	77107280	77107637	-1.33	-0.14
Ube2g2	chr10	77107992	77108082	-0.37	-0.04
Cdc34	chr10	79144939	79145322	-5.03	-0.46
Cdc34	chr10	79148043	79151143	-18.90	-0.38
Eef2	chr10	80644273	80645254	-24.30	-0.11
Dohh	chr10	80853555	80854150	-3.43	-0.73
Eid3	chr10	82329518	82330082	-0.37	-0.03
Eid3	chr10	82330466	82330644	-3.01	1.11
Galnt4	chr10	98570977	98571038	-0.40	0.27
Galnt4	chr10	98571426	98572144	-0.49	-0.09
Galnt4	chr10	98572382	98573280	-1.98	0.41
Ccdc59	chr10	105278534	105284446	-1.03	0.09
Cpm	chr10	117121110	117121651	-1.22	0.46
Pcmt1	chr10	7350508	7350747	-0.66	-0.14
Pcmt1	chr10	7383084	7383144	-0.25	0.06
Rab32	chr10	10265729	10270492	-5.49	-0.40
Tnfaip3	chr10	18721581	18725137	-0.67	-0.06
Rnf146	chr10	29064251	29064491	-0.35	0.02
Rnf146	chr10	29066497	29067546	-0.70	-0.06
Man1a	chr10	53750579	53795542	-4.76	-1.15
Ube2d1	chr10	70718144	70718592	-2.63	0.38
Trappc10	chr10	77650129	77651540	-1.80	-0.19
Pias4	chr10	80616715	80617300	-0.74	-0.10
Pias4	chr10	80619974	80625923	-1.46	-0.22
Pias4	chr10	80626491	80630465	-4.33	-0.46
Mrpl54	chr10	80727466	80728472	-0.87	-0.09
Mrpl54	chr10	80728529	80729671	-0.46	-0.07
Ckap4	chr10	83990030	83991369	-1.40	-0.09
Ckap4	chr10	83996275	83996633	-3.93	-0.77
Rab21	chr10	114727724	114727964	-0.37	0.01
Mdm2	chr10	117126170	117126441	-1.00	0.08
Mdm2	chr10	117126799	117129718	-14.10	0.17

Cand1	chr10	118647900	118648919	-0.49	0.03
Usp15	chr10	122550299	122558549	-0.60	0.05
Os9	chr10	126531344	126532125	-2.77	-0.49
Os9	chr10	126532363	126535514	-5.24	-0.16
Os9	chr10	126557594	126558216	-2.38	-0.34
Mbd6	chr10	126719071	126720682	-0.32	0.00
Ykt6	chr11	5855760	5856000	-4.46	-0.65
Ykt6	chr11	5866082	5866352	-0.37	-0.02
Usp34	chr11	23206894	23233439	-0.42	0.06
Usp34	chr11	23233527	23233678	-2.02	0.32
Usp34	chr11	23263863	23264511	-1.71	-0.12
Usp34	chr11	23364346	23364676	-0.48	0.05
Usp34	chr11	23388278	23389213	-1.07	-0.10
Psme4	chr11	30671774	30672135	-0.54	-0.18
Fbxw11	chr11	32644536	32644986	-0.78	-0.08
Mgat1	chr11	49057722	49075721	-5.05	-0.26
Mgat4b	chr11	50039046	50039137	-4.28	-1.00
Mgat4b	chr11	50048124	50048515	-6.26	-0.32
Galnt10	chr11	57597256	57598674	-0.70	0.22
Hist3h2a	chr11	58768661	58768929	-4.41	0.77
Nlrp3	chr11	59355086	59365364	-68.60	-0.49
Nlrp3	chr11	59379889	59380219	-0.60	0.09
Adora2b	chr11	62062485	62062664	-0.34	0.18
Adora2b	chr11	62062842	62079805	-4.54	0.57
Pigl	chr11	62271961	62272082	-2.00	-0.93
Aurkb	chr11	68861818	68863941	-3.92	-0.59
Trp53	chr11	69393860	69394608	-0.43	-0.06
Asgr1	chr11	69870863	69871366	-0.36	1.28
Ube2g1	chr11	72420792	72421032	-3.90	-0.86
Ube2g1	chr11	72493612	72497295	-0.33	0.03
Rtn4rl1	chr11	75079118	75079476	-0.61	2.96
Pigs	chr11	78155467	78156068	-7.36	-0.45
Suz12	chr11	79843530	79845978	-0.38	0.01
Ptrh2	chr11	86497514	86503722	-9.69	-0.43
Mrps23	chr11	88024206	88024890	-1.70	-0.18
Mrpl10	chr11	96909612	96910055	-0.43	-0.03
Psmd3	chr11	98543867	98546829	-30.30	-0.74
Thra	chr11	98603186	98614709	-0.74	0.42
Thra	chr11	98614768	98617477	-2.58	-0.78
Thra	chr11	98625600	98626259	-4.68	-0.69
Thra	chr11	98629482	98629782	-10.30	1.38
Rara	chr11	98799009	98808908	-1.61	-0.58
Rara	chr11	98808997	98822329	-5.35	-0.55

Rara	chr11	98827782	98829451	-0.74	-0.25
Rara	chr11	98831600	98835508	-19.40	-0.66
Psme3	chr11	101177564	101178336	-0.62	0.07
Psme3	chr11	101182070	101183288	-0.64	-0.06
Dcaf7	chr11	105898185	105898485	-1.07	-0.15
Dcaf7	chr11	105916210	105916360	-1.24	0.22
Dcaf7	chr11	105919829	105920129	-0.82	-0.12
Arsg	chr11	109346860	109378636	-0.38	-0.02
Arsg	chr11	109433808	109434107	-0.35	0.06
Cog1	chr11	113517332	113518437	-0.57	-0.07
Mrpl58	chr11	115271633	115272227	-8.48	-0.50
Mrps7	chr11	115467375	115468938	-14.00	-0.46
Sphk1	chr11	116393314	116393761	-0.47	2.50
Birc5	chr11	117716547	117716877	-2.95	-0.53
Engase	chr11	118348561	118349312	-2.33	-0.74
Cbx2	chr11	118889293	118890939	-23.30	-1.92
Hgs	chr11	120345028	120345208	-9.55	-0.93
Mrpl12	chr11	120345982	120350068	-18.00	-0.36
Gps1	chr11	120648738	120649402	-0.35	-0.06
Gps1	chr11	120649969	120650386	-3.03	-0.46
Foxk2	chr11	121160998	121169022	-0.31	0.00
Fn3k	chr11	121303142	121303262	-0.62	-3.70
Fn3k	chr11	121303589	121303799	-0.67	-3.37
Mrps24	chr11	5604429	5604637	-0.82	0.13
B3gnt2	chr11	22735930	22759719	-2.61	-0.09
Commd1	chr11	22799727	22800026	-0.83	-0.10
Commd1	chr11	22856676	22878367	-0.64	0.15
Commd1	chr11	22878962	22882284	-2.22	-0.60
Pex13	chr11	23548957	23549346	-1.39	0.33
Pex13	chr11	23551042	23555901	-2.58	-0.39
Sptbn1	chr11	30017714	30018324	-0.64	0.10
Sptbn1	chr11	30036124	30037605	-1.75	0.16
Sptbn1	chr11	30038786	30040543	-0.57	-0.05
Stc2	chr11	31259890	31265353	-2.88	0.65
Canx	chr11	50109707	50110776	-30.20	-0.37
0610009B22Rik	chr11	51499121	51499563	-2.06	0.33
Sec24a	chr11	51556985	51570140	-6.97	-0.33
Tnip1	chr11	54724288	54731425	-26.80	-0.37
Tnip1	chr11	54740251	54744279	-11.00	-0.69
Tnip1	chr11	54753132	54754378	-8.03	-0.57
Tnip1	chr11	54769581	54776442	-0.49	0.19
Derl2	chr11	70823390	70826702	-4.56	-0.26
Hic1	chr11	74979024	74979444	-1.07	-1.34

Rpa1	chr11	75114477	75116219	-1.52	-0.18
Rpa1	chr11	75154547	75161885	-1.59	0.39
Gosr1	chr11	76543115	76548136	-0.51	0.05
Eral1	chr11	77887262	77887988	-2.22	-0.30
Wsb1	chr11	79053599	79053839	-11.70	-0.39
Pex12	chr11	83109875	83111883	-1.19	0.24
Pigw	chr11	84691181	84691925	-0.65	-0.09
Ube2z	chr11	95911494	95911704	-1.74	-0.23
Pcgf2	chr11	97551180	97551450	-9.46	-1.52
Pcgf2	chr11	97553219	97553423	-0.65	0.22
Fbxl20	chr11	97950510	97953462	-0.74	0.16
Klhl11	chr11	100323925	100325737	-6.64	0.31
Klhl11	chr11	100333798	100334096	-2.00	0.50
Kat2a	chr11	100566059	100566924	-11.10	-0.42
Kat2a	chr11	100573600	100573691	-0.37	-0.10
Becn1	chr11	101163065	101163581	-1.84	-0.26
Brca1	chr11	101384784	101385234	-1.07	-0.42
Brca1	chr11	101385503	101386581	-0.66	0.14
Brca1	chr11	101386909	101387659	-0.94	0.23
Gosr2	chr11	103540134	103540702	-4.90	-0.28
Nsf	chr11	103684412	103685071	-1.08	-0.20
Nsf	chr11	103792113	103815310	-0.40	0.17
Ern1	chr11	106259052	106261558	-0.55	-0.04
Ddx5	chr11	106643371	106643551	-60.70	0.67
Ddx5	chr11	106650118	106650179	-4.34	1.55
Smurf2	chr11	106683743	106684388	-0.37	-0.04
Smurf2	chr11	106781609	106781789	-3.23	-1.30
Mrpl38	chr11	115993160	115993341	-0.73	-0.12
Mrpl38	chr11	115999581	115999702	-0.45	-0.23
Exoc7	chr11	116149939	116150478	-4.87	-0.80
Socs3	chr11	117827669	117830291	-196.00	-0.95
Cbx8	chr11	118899838	118901471	-0.31	0.01
Cbx4	chr11	118941130	118941400	-2.21	0.97
Cbx4	chr11	118941729	118947491	-3.35	-0.13
P4hb	chr11	120421617	120422006	-31.00	-0.33
P4hb	chr11	120422453	120422573	-21.50	-0.34
Csnk1d	chr11	120819778	120820048	-1.86	-0.20
Csnk1d	chr11	120823809	120824109	-0.87	-0.12
Csnk1d	chr11	120824767	120826288	-4.93	-0.25
Csnk1d	chr11	120844636	120852467	-0.33	-0.01
B3gntl1	chr11	121478331	121479112	-4.34	-0.46
Asxl2	chr12	3426883	3442534	-2.32	-0.27
Asxl2	chr12	3496396	3496756	-2.31	0.18

Asxl2	chr12	3496815	3497026	-0.44	0.05
Asxl2	chr12	3497115	3502505	-2.19	0.08
Pomc	chr12	3959970	3960089	-0.43	1.11
Smc6	chr12	11292342	11292462	-0.56	0.29
Smc6	chr12	11324583	11325004	-0.86	0.12
Mia2	chr12	60208874	60209969	-4.23	1.41
Lrr1	chr12	70275442	70275972	-0.48	-0.36
Mgat2	chr12	70285182	70287153	-2.50	0.08
Mgat2	chr12	70285144	70287166	-2.79	0.08
Hif1a	chr12	75008969	75009300	-10.10	-0.56
Hif1a	chr12	75043097	75043308	-29.20	-0.61
Fut8	chr12	78432957	78466016	-0.35	0.04
Fut8	chr12	78576163	78576974	-3.78	0.62
Eif2b2	chr12	86560462	86560886	-5.50	-0.34
Eif2b2	chr12	86567073	86567489	-0.32	0.01
Ttll5	chr12	87165622	87165743	-0.28	-0.08
Ttll5	chr12	87259935	87260176	-8.05	-1.20
Psmc1	chr12	101348377	101351316	-1.50	-0.23
Calm1	chr12	101437750	101440606	-16.30	-0.45
Calm1	chr12	101445239	101445449	-3.24	-0.14
Otub2	chr12	104641553	104643038	-2.18	-0.30
Yy1	chr12	110031520	110044742	-20.20	-0.57
Yy1	chr12	110052912	110054425	-13.00	0.43
Dync1h1	chr12	111839634	111839815	-0.41	-0.05
Dync1h1	chr12	111855934	111857506	-0.43	-0.01
Dync1h1	chr12	111904592	111905154	-1.97	-0.13
Wdr20	chr12	112031349	112032696	-1.57	-0.15
Traf3	chr12	112498880	112500724	-1.28	0.14
Eif5	chr12	112776401	112777093	-0.78	0.07
Eif5	chr12	112783135	112783316	-9.79	0.51
Btbd6	chr12	114215520	114217031	-10.90	-0.80
Mta1	chr12	114374970	114375239	-4.02	-0.39
Ncoa1	chr12	4247600	4248409	-2.05	-0.34
Ncoa1	chr12	4248946	4249007	-0.88	-0.53
Ncoa1	chr12	4249095	4252804	-0.98	-0.20
Ncoa1	chr12	4267739	4267919	-1.13	-0.35
Ncoa1	chr12	4295010	4295788	-1.68	-0.24
Ncoa1	chr12	4295907	4296027	-0.69	-0.27
Nfkbia	chr12	56593395	56593604	-103.00	-1.36
Trappc6b	chr12	60162299	60162448	-2.15	-0.55
Sptb	chr12	77714000	77714211	-0.38	1.30
Rab15	chr12	77899425	77899575	-0.18	0.47
Pigh	chr12	80183182	80183512	-0.39	-0.08

Dcaf5	chr12	81437908	81438298	-0.31	-0.01
Dcaf5	chr12	81439342	81449328	-1.21	-0.09
Tmed10	chr12	86683962	86685208	-9.54	-0.24
Pomt2	chr12	88449791	88451284	-3.44	-0.46
Sel1l	chr12	93046846	93048492	-1.21	0.05
Sel1l	chr12	93080261	93087597	-2.29	-0.43
Atxn3	chr12	103157200	103157589	-0.54	-0.09
Ankrd9	chr12	112214580	112215239	-4.52	-1.58
Ero1lb	chr13	12658149	12658419	-0.59	0.14
Ero1lb	chr13	12699110	12699321	-0.36	0.08
B3galnt2	chr13	14090832	14091101	-6.32	-0.86
Inhba	chr13	16106283	16119044	-0.73	1.11
Trim27	chr13	21283975	21284396	-0.44	-0.02
Hist1h2bg	chr13	23663268	23663474	-1.13	-0.09
Hist1h2bc	chr13	23776067	23776213	-2.98	0.39
Hist1h2ab	chr13	23842956	23843254	-0.48	0.06
Gpld1	chr13	25078887	25082222	-4.74	-0.74
Ripk1	chr13	34094265	34094762	-9.02	-0.95
Ripk1	chr13	34119794	34125274	-0.32	0.00
Ripk1	chr13	34125393	34126082	-9.17	-0.86
Tmed9	chr13	55696944	55698672	-34.70	-0.59
2010111I01Rik	chr13	63116375	63134422	-0.48	0.07
2010111I01Rik	chr13	63400063	63400274	-4.23	-0.34
2010111I01Rik	chr13	63400931	63403592	-3.62	-0.21
Arsb	chr13	94710665	94710756	-0.36	-0.06
Gfm2	chr13	97945566	97945897	-0.33	-0.02
Gfm2	chr13	97949530	97949591	-0.77	0.76
Mrps27	chr13	100182308	100185516	-0.61	0.07
Ercc8	chr13	108948931	108950875	-0.31	-0.05
Gmds	chr13	31911574	31911785	-2.65	-0.65
Tubb2a	chr13	34166146	34168454	-10.90	0.67
Tubb2b	chr13	34219290	34219586	-1.55	1.29
Tubb2b	chr13	34219940	34221135	-0.88	0.43
Nup153	chr13	46776258	46776559	-2.23	0.27
Nup153	chr13	46782040	46792068	-1.16	0.05
Nup153	chr13	46822738	46823218	-7.57	-0.49
Uimc1	chr13	55129866	55132327	-0.91	0.20
Uimc1	chr13	55176930	55177378	-3.37	-0.43
Rab24	chr13	55421264	55421501	-3.81	-0.46
Rab24	chr13	55422955	55423341	-4.54	-0.47
Lman2	chr13	55447437	55447738	-0.36	0.02
Lman2	chr13	55448006	55448306	-6.45	-0.33
Hnrnpk	chr13	58501349	58504674	-3.06	-0.25

Golm1	chr13	59736356	59736893	-2.58	-0.39
Golm1	chr13	59738410	59741528	-2.06	-0.35
Exoc3	chr13	74327547	74344379	-6.09	-0.31
Vcan	chr13	89829747	89829807	-0.19	1.00
Vcan	chr13	89830646	89830706	-0.19	1.00
Slc30a5	chr13	101572662	101573383	-0.95	0.09
Mrps30	chr13	119169246	119169337	-2.14	0.40
Kctd6	chr14	9052713	9055575	-1.94	-0.30
Atxn7	chr14	14928824	14936674	-0.35	0.01
Sec24c	chr14	21512428	21513175	-11.10	-0.51
Sec24c	chr14	21513593	21514072	-11.40	-0.48
Actr8	chr14	30804041	30804131	-3.81	-1.37
Bap1	chr14	32072009	32072607	-7.27	-0.52
Psmc6	chr14	45949498	45950514	-0.60	-0.09
Ktn1	chr14	48283451	48283962	-3.75	0.33
Oxa1l	chr14	54988005	54988365	-0.95	-0.14
Mrpl52	chr14	55048471	55048587	-8.67	-0.61
Dcaf11	chr14	56178865	56178955	-0.45	-0.26
Dcaf11	chr14	56188485	56188902	-0.70	-0.12
Mrpl57	chr14	58445075	58445797	-9.14	-0.38
Trim13	chr14	62223590	62224752	-5.87	0.61
Kbtbd7	chr14	79827156	79828746	-2.42	0.38
Gng2	chr14	20694858	20796232	-0.84	-0.13
Spcs1	chr14	31813189	31813397	-2.85	-0.22
Ankrd28	chr14	32514699	32518527	-2.62	-0.26
Dph3	chr14	32893956	32894136	-0.34	-0.09
Dph3	chr14	32896016	32898095	-0.33	0.01
Exoc5	chr14	49633588	49634009	-10.50	0.57
Nedd8	chr14	56290685	56290743	-0.59	-0.14
Rabggta	chr14	56340520	56340807	-0.58	0.19
Cog3	chr14	76103115	76106953	-7.11	-0.50
Commd6	chr14	102033341	102039330	-1.33	-0.15
Fbxl3	chr14	103482090	103488631	-0.87	-0.07
Fbxl3	chr14	103491640	103498113	-0.65	0.09
Fbxl3	chr14	103498172	103498726	-0.67	-0.17
Sema5a	chr15	32616573	32620190	-0.36	0.02
Dcaf13	chr15	38944419	38944568	-1.48	-0.33
Rnf139	chr15	58730017	58731816	-1.36	-0.10
Commd5	chr15	76730370	76731727	-6.12	-0.48
Apol9b	chr15	77565748	77566812	-1.14	0.81
Csf2rb	chr15	78156456	78166828	-4.16	-0.44
Csf2rb	chr15	78177155	78179733	-19.50	-0.49
Tab1	chr15	79989230	79992132	-13.10	-0.63

Mgat3	chr15	80042031	80042242	-0.25	1.12
Rbx1	chr15	81305971	81306061	-1.28	-0.57
Ep300	chr15	81416643	81438767	-12.50	-0.29
Ep300	chr15	81479273	81481819	-5.01	-0.13
L3mbtl2	chr15	81516741	81518556	-3.63	-0.29
Nup50	chr15	84757805	84765822	-13.80	-0.39
Nup50	chr15	84770131	84771000	-9.31	-0.39
Nup50	chr15	84773033	84773303	-0.67	0.14
Tuba1c	chr15	98868116	98868326	-32.90	-0.97
Eif4b	chr15	101925104	101925897	-0.89	-0.04
Skp2	chr15	9043329	9043510	-0.95	0.41
Skp2	chr15	9069207	9070137	-7.83	-1.04
Otulin	chr15	27535675	27538550	-1.81	-0.14
Otulin	chr15	27553050	27560448	-1.52	-0.31
6-Mar	chr15	31388351	31388771	-2.84	-0.29
Rad21	chr15	51794834	51794954	-0.74	0.12
Rad21	chr15	51798216	51799452	-7.34	0.55
Mrpl13	chr15	55386872	55388867	-1.12	0.33
Derl1	chr15	57701535	57701686	-0.52	0.09
Derl1	chr15	57702583	57703422	-5.82	-0.18
Fbxo32	chr15	58011902	58012112	-0.40	-0.84
Fbxo32	chr15	58012410	58013039	-1.36	-1.07
Fbxo32	chr15	58046223	58046433	-0.22	-0.12
St3gal1	chr15	66936984	66938364	-4.76	-0.34
St3gal1	chr15	66944067	67005237	-3.10	-0.24
Apol9a	chr15	77234218	77235287	-0.50	-0.25
Apol10b	chr15	77415861	77416010	-0.29	1.46
Apol8	chr15	77579401	77583243	-8.81	2.69
Csf2rb2	chr15	78112999	78113060	-1.58	0.68
Csf2rb2	chr15	78114558	78116509	-0.74	0.08
Csf2rb2	chr15	78127494	78136033	-1.82	-0.32
Ddx17	chr15	79359532	79359623	-1.79	-0.30
Ddx17	chr15	79376930	79376991	-1.99	-0.41
Josd1	chr15	79506286	79506525	-1.24	-0.34
Josd1	chr15	79517170	79517647	-1.09	0.21
Josd1	chr15	79517914	79518183	-1.04	-0.32
Mkl1	chr15	80843459	80848650	-8.27	-0.28
Rangap1	chr15	81535516	81535666	-0.95	0.18
Rangap1	chr15	81560168	81560349	-4.41	-0.86
Pmm1	chr15	81781535	81781862	-2.41	-0.51
Ttll12	chr15	83406959	83407199	-3.52	-0.70
Alg12	chr15	88635941	88636391	-1.81	-0.30
Arsa	chr15	89303025	89303862	-0.98	-0.16

Arsa	chr15	89305988	89306615	-6.67	-0.79
Vdr	chr15	97687063	97687362	-0.53	1.58
Senp1	chr15	97872854	97873333	-1.22	-0.16
Asb8	chr15	97965969	97971744	-1.49	-0.17
Galnt6	chr15	100522228	100522617	-0.89	-0.21
Galnt6	chr15	100544605	100550892	-2.41	-0.23
Alg1	chr16	5244821	5245001	-1.38	-0.34
Klhl22	chr16	17771956	17777358	-0.41	-0.03
Psmd2	chr16	20651724	20652324	-20.60	-0.49
Eif4g1	chr16	20672821	20673965	-1.18	-0.11
Eif4g1	chr16	20674380	20675449	-1.23	-0.13
Eif4g1	chr16	20679335	20680055	-13.00	-0.18
Eif4g1	chr16	20689353	20692866	-16.70	-0.31
Senp2	chr16	22035915	22036036	-1.16	-0.87
Senp2	chr16	22046859	22047159	-0.36	0.02
St6gal1	chr16	23321224	23321703	-0.42	-0.35
Rnf168	chr16	32277606	32278356	-0.49	-0.08
Rnf168	chr16	32298678	32299428	-0.38	0.03
Golgb1	chr16	36898620	36898980	-0.86	-0.15
Golgb1	chr16	36907962	36914816	-3.50	0.16
Golgb1	chr16	36914905	36915146	-0.37	0.03
Golgb1	chr16	36915415	36916525	-5.29	0.32
Golgb1	chr16	36916673	36919800	-0.64	0.04
Golgb1	chr16	36932301	36933171	-5.73	-0.44
Tomm70a	chr16	57152724	57153054	-5.15	-0.28
Usp25	chr16	77115246	77116186	-1.62	-0.12
Usp16	chr16	87477080	87480005	-7.48	-0.34
Mrps6	chr16	92112001	92112296	-11.70	0.52
Eef2kmt	chr16	5244426	5244486	-0.43	-0.18
Eef2kmt	chr16	5244813	5245351	-3.03	-0.38
Usp7	chr16	8738344	8757028	-0.78	-0.28
Gspt1	chr16	11218069	11218249	-1.19	0.43
Gspt1	chr16	11253908	11254418	-13.90	-0.53
Mrpl40	chr16	18872286	18872697	-1.94	-0.23
Senp5	chr16	31963352	31965869	-0.38	0.03
Senp5	chr16	31989077	31990517	-1.86	-0.12
Sec22a	chr16	35313658	35318950	-0.30	0.00
Nrip1	chr16	76291226	76295183	-5.84	0.27
Nrip1	chr16	76352759	76374042	-0.85	-0.46
App	chr16	85025115	85043833	-16.90	-0.31
App	chr16	85120907	85173892	-5.77	-0.46
Tulp4	chr17	6106833	6122253	-14.10	-0.52
Tulp4	chr17	6124111	6138054	-19.70	0.47

Tulp4	chr17	6138143	6139103	-10.10	0.29
Tulp4	chr17	6232220	6232851	-0.61	0.07
Tulp4	chr17	6232910	6233480	-0.97	0.22
Tulp4	chr17	6234109	6236560	-0.44	0.07
Tcp1	chr17	13117452	13117603	-0.51	-0.11
Abca3	chr17	24546305	24547146	-3.17	-0.34
Spsb3	chr17	25023618	25029092	-0.66	-0.05
Mrps34	chr17	25032064	25033409	-1.86	-0.15
Axin1	chr17	26275810	26310709	-1.53	0.13
Axin1	chr17	26321127	26325117	-8.61	-0.59
Axin1	chr17	26330938	26332096	-0.71	0.13
Crebrf	chr17	26873877	26879828	-3.05	0.25
Adamts10	chr17	33665942	33666123	-1.41	-0.95
Adamts10	chr17	33667169	33667439	-2.37	-1.68
Adamts10	chr17	33690397	33690697	-2.53	-1.03
Daxx	chr17	34047504	34050602	-124.00	-0.92
Psmb8	chr17	34335288	34335468	-2.22	-0.33
Neu1	chr17	35071556	35072840	-6.58	-0.28
Mdc1	chr17	35983321	35985735	-0.37	0.02
Mdc1	chr17	35987855	35988082	-1.14	0.38
Mdc1	chr17	35989429	35989938	-3.36	0.34
Mdc1	chr17	35994492	35994583	-1.22	0.38
Mrpl14	chr17	45834998	45835444	-0.40	-0.03
Mrps18a	chr17	46247952	46259753	-4.55	-0.41
Mrpl2	chr17	46783196	46783346	-0.70	-0.18
Cul7	chr17	46788096	46788277	-2.58	3.86
Cul7	chr17	46788516	46788967	-0.83	-0.64
Cul7	chr17	46791019	46791200	-0.63	1.18
Mrps10	chr17	47505835	47506196	-1.59	-0.68
Rab5a	chr17	53618797	53623279	-2.09	-0.24
Rab5a	chr17	53639752	53646106	-0.93	0.08
Fem1a	chr17	56396215	56399474	-1.33	-0.07
Fem1a	chr17	56400758	56401238	-0.56	-0.12
Fem1a	chr17	56401745	56402642	-3.47	-0.51
Safb	chr17	56733308	56735158	-0.34	-0.01
Safb	chr17	56738105	56738706	-1.43	-0.14
Man2a1	chr17	64950988	64974682	-0.45	0.05
Slc30a6	chr17	74822465	74823122	-0.78	-0.11
Socs5	chr17	87507227	87507318	-0.46	-0.23
Socs5	chr17	87520106	87534863	-0.79	0.07
Mrpl18	chr17	13104220	13104550	-0.93	0.14
Ccnf	chr17	24360594	24360954	-5.87	0.78
Ccnf	chr17	24361042	24361933	-12.00	1.09

Ube2i	chr17	25398055	25398776	-6.49	-0.64
Rab40c	chr17	26020195	26020794	-2.20	-0.30
Pigq	chr17	26064506	26064597	-0.37	-0.03
Pigq	chr17	26071113	26071234	-3.26	1.37
Pigq	chr17	26073535	26073656	-2.01	1.11
Pigq	chr17	26073775	26078847	-1.69	0.17
Rab11b	chr17	33883909	33884089	-4.70	-0.48
Rab11b	chr17	33884327	33884477	-0.75	-0.25
Ring1	chr17	34158095	34160251	-0.84	-0.10
Ly6g6d	chr17	35208436	35208609	-0.20	-0.07
Cul9	chr17	46680214	46680695	-2.00	-0.64
Tubb4a	chr17	57220052	57220528	-1.24	-0.37
Tubb4a	chr17	57220616	57221122	-1.23	-0.35
Rab31	chr17	66001065	66001632	-0.68	-0.05
Rab31	chr17	66002078	66002287	-2.81	-0.26
Rab31	chr17	66003418	66017257	-2.20	0.14
Rab31	chr17	66122031	66122092	-0.25	-0.17
Rab12	chr17	66844241	66844961	-2.21	-0.20
Pigf	chr17	87396598	87396896	-1.18	0.11
Mcfd2	chr17	87654470	87654591	-0.51	0.10
Mcfd2	chr17	87654769	87656548	-2.71	-0.18
Calm2	chr17	87835011	87846216	-2.03	-0.14
Fbxo11	chr17	88390945	88391686	-0.95	0.09
Fbxo11	chr17	88464385	88464625	-1.02	-0.30
Wac	chr18	7869438	7869558	-1.86	-0.56
Wac	chr18	7905960	7910820	-1.20	0.09
Wac	chr18	7921952	7926308	-5.51	0.73
Galnt1	chr18	24438515	24443824	-0.96	-0.11
Apc	chr18	34458082	34458262	-0.62	-0.18
Apc	chr18	34470478	34472953	-2.03	0.18
Apc	chr18	34473850	34476007	-2.30	0.15
Apc	chr18	34476395	34478342	-2.44	-0.16
Ube2d2a	chr18	35931242	35931392	-3.18	-0.65
Ube2d2a	chr18	35965479	35965570	-0.43	0.27
Commd10	chr18	47246724	47246874	-0.45	0.07
Dctn4	chr18	60686499	60686560	-0.25	-0.32
Dctn4	chr18	60716315	60716526	-1.59	0.32
Napg	chr18	63154672	63156813	-3.03	-0.31
Sec11c	chr18	65960231	65960321	-0.63	-0.13
Tubb6	chr18	67550384	67552360	-1.76	-0.20
Tubb6	chr18	67552568	67561747	-29.40	-0.45
Mbd1	chr18	74437069	74438854	-3.83	-0.42
Mbd1	chr18	74439959	74440737	-1.71	-0.23

Mbd1	chr18	74440886	74442159	-0.82	-0.10
Smad7	chr18	75527018	75527675	-0.56	0.13
Smad7	chr18	75528299	75554128	-4.14	0.55
Smad2	chr18	76462139	76464727	-1.34	-0.19
Pias2	chr18	77304126	77304476	-0.83	0.35
Pias2	chr18	77304625	77336416	-0.31	0.00
Pias2	chr18	77388818	77391933	-0.88	0.13
Usp14	chr18	10030056	10030117	-0.99	-0.34
B4galt6	chr18	20846332	20847761	-0.59	-0.09
Ino80c	chr18	24263948	24264068	-1.28	-0.40
Ino80c	chr18	24264545	24265143	-0.55	0.07
Etf1	chr18	35064380	35064620	-4.80	-0.19
Etf1	chr18	35073803	35091657	-6.76	-0.31
Nr3c1	chr18	39588392	39650238	-0.66	-0.04
Fem1c	chr18	46664557	46685565	-0.38	0.01
Tmed7	chr18	46747763	46748242	-1.09	-0.08
Adrb2	chr18	62337558	62339256	-44.30	0.73
Lman1	chr18	66140572	66140992	-1.11	-0.12
Lman1	chr18	66162108	66162289	-2.28	-0.31
Smad4	chr18	73799858	73800008	-0.35	-0.03
Smad4	chr18	73837391	73837750	-0.38	-0.03
Smad4	chr18	73863156	73863395	-1.93	-1.18
Socs6	chr18	89038427	89056448	-0.73	0.07
Sptbn2	chr19	4744054	4745130	-1.41	-0.53
B4gat1	chr19	5039183	5040891	-7.61	-0.49
B4gat1	chr19	5041278	5046482	-6.38	-0.41
B4gat1	chr19	5049005	5049917	-6.80	-0.44
Rela	chr19	5646968	5647773	-3.54	-0.20
Syvn1	chr19	6052640	6053718	-8.86	-0.27
Men1	chr19	6334978	6336781	-0.48	-0.04
Men1	chr19	6338873	6340864	-2.26	-0.13
Trmt112	chr19	6984277	6984947	-10.30	-0.42
Stx5a	chr19	8829416	8830132	-0.89	-0.09
Ubxn1	chr19	8946015	8946196	-2.26	-0.40
Mrpl16	chr19	11848755	11849170	-2.33	-0.25
Uhrf2	chr19	30105092	30105153	-1.10	-0.51
Uhrf2	chr19	30113733	30131000	-0.49	-0.06
Pten	chr19	32832126	32832995	-2.07	-0.13
Pten	chr19	32872591	32874607	-0.82	0.07
Pten	chr19	32892519	32894752	-15.20	0.55
Stambpl1	chr19	34266759	34267030	-0.53	0.08
Stambpl1	chr19	34307271	34308607	-1.79	-0.39
Exoc6	chr19	37757471	37757650	-1.13	-0.26

Btrc	chr19	45604117	45604298	-0.65	1.17
Gbf1	chr19	46227047	46227228	-1.43	-0.39
Nfkb2	chr19	46379436	46380290	-0.39	0.13
Fbxl15	chr19	46403649	46404936	-3.45	-0.46
Adra2a	chr19	54121524	54121674	-1.08	3.17
Adra2a	chr19	54122092	54122183	-0.67	3.91
Adra2a	chr19	54121526	54121677	-1.08	3.17
Adra2a	chr19	54122095	54122155	-0.56	3.46
Ppp6r3	chr19	3454927	3455647	-21.70	-0.68
Ppp6r3	chr19	3456876	3459395	-1.96	-0.25
Rce1	chr19	4622816	4623024	-4.13	-0.70
1700020D05Rik	chr19	5502858	5503787	-6.87	0.99
Mrpl49	chr19	6057691	6057751	-0.27	-0.01
Incenp	chr19	9947175	9947235	-0.82	-0.16
Incenp	chr19	9949354	9951151	-0.99	-0.15
Incenp	chr19	9957682	9968481	-0.67	-0.03
Incenp	chr19	9973932	9974023	-0.51	0.26
Tmem132a	chr19	10932318	10936113	-125.00	-1.30
Gcnt1	chr19	17403142	17407678	-1.38	0.13
Mrpl43	chr19	45079592	45080932	-23.50	-0.42
Fbxw4	chr19	45652866	45653256	-0.75	-0.11
Actr1a	chr19	46452346	46452913	-2.60	-0.15
Eif3a	chr19	60837737	60844665	-77.00	-0.21
Eif3a	chr19	60845877	60846963	-8.46	-0.29
Csf2ra	chr19	61300453	61302211	-14.30	-0.30
Csf2ra	chr19	61302992	61304321	-7.97	-0.39
Usp9x	chrX	12733152	12733872	-13.20	0.35
Ube2a	chrX	34414360	34414420	-1.18	-0.44
Foxo4	chrX	98449866	98450612	-10.40	-0.99
Ogt	chrX	98874610	98878315	-1.22	-0.06
Ogt	chrX	98878792	98878973	-1.73	-0.32
Piga	chrX	160857718	160861207	-2.61	0.43
Piga	chrX	160866881	160869822	-1.27	0.39
Piga	chrX	160870327	160870477	-1.00	0.56
C1galt1c1	chrX	35984195	35988290	-0.70	0.07
Hcfc1	chrX	71189242	71189693	-2.65	0.19
Hcfc1	chrX	71194710	71196030	-1.82	-0.08
Hcfc1	chrX	71211533	71211654	-0.42	0.04
Chm	chrX	110156917	110157037	-0.85	0.35
Mbtps2	chrX	153988770	153989011	-1.09	0.24
Mbtps2	chrX	154006202	154006623	-0.87	0.27
Mbtps2	chrX	154010302	154010483	-0.64	0.30
Yy2	chrX	154005005	154006618	-13.20	0.86

Yy2	chrX	154005016	154006605	-13.60	0.88
Rab9	chrX	162895660	162900249	-8.56	0.50
Vcpip1	chr1	9714443	9738463	-10.40	-0.15
Napb	chr2	148538465	148558156	-0.50	-0.19
Uba6	chr5	86541279	86547113	-0.45	0.04
Tnks	chr8	36028534	36028744	-3.00	-0.65
Dynll2	chr11	87794645	87799923	-0.59	0.05
Dynll2	chr11	87800072	87801030	-0.39	-0.04

Post translational protein modification

gene_name	chr	chromStart	chromEnd	diff.lg.p	diff.log2.fc
Rab23	chr1	33795210	33796982	-0.80	0.16
Ttll4	chr1	74725556	74727916	-10.70	-0.35
Ttll4	chr1	74744098	74744368	-1.67	-0.40
Sp100	chr1	87605724	87606023	-1.74	0.47
Cops7b	chr1	88502539	88502837	-3.51	-0.57
Cops8	chr1	92508875	92509678	-1.17	-0.09
Ube2f	chr1	93182063	93182602	-2.18	-0.17
Asb1	chr1	93437321	93437716	-0.36	-0.19
Asb1	chr1	93440810	93443681	-2.13	-0.63
Asb1	chr1	93451344	93451764	-1.52	0.40
Mgat5	chr1	129203345	129203496	-1.16	-0.43
Mgat5	chr1	129376047	129379549	-1.14	-0.18
Yod1	chr1	132614351	132616572	-0.52	0.05
Rab7b	chr1	133585271	133593615	-0.74	-0.38
Rab7b	chr1	133602280	133610067	-11.80	-0.70
Rab29	chr1	133763853	133763913	-0.39	-0.39
Rbbp5	chr1	134394620	134400982	-0.63	-0.07
Rbbp5	chr1	134401101	134401282	-1.08	0.24
Ube2t	chr1	136859153	136864304	-0.88	-0.47
Edem3	chr1	153658740	153666587	-2.13	-0.17
Pigc	chr1	163899318	163901752	-3.01	-0.22
F5	chr1	166122352	166122563	-1.98	4.32
F5	chr1	166123252	166123343	-0.35	1.70
F5	chr1	166123612	166123702	-0.24	1.22
F5	chr1	166124001	166124272	-1.70	2.22
Fcgr4	chr1	172959296	172959476	-0.48	-0.13
Adamts4	chr1	173188947	173189485	-1.30	-1.86
B4galt3	chr1	173206252	173206788	-5.31	-0.40
Dcaf8	chr1	174078175	174078475	-2.57	-0.67
Dcaf8	chr1	174079403	174103118	-5.58	-0.32
Dcaf8	chr1	174124786	174125087	-0.67	-0.13
Pigm	chr1	174306661	174308487	-3.05	0.17
Parp1	chr1	182499105	182503813	-1.96	-0.45

Cops5	chr1	10027829	10027979	-1.20	-0.16
Uggt1	chr1	36200615	36203553	-2.05	0.15
Uggt1	chr1	36300966	36301147	-1.89	-0.42
Lman2l	chr1	36480210	36481260	-8.25	-0.53
Mgat4a	chr1	37501090	37501720	-2.18	0.31
Chst10	chr1	38920747	38922995	-1.47	-0.11
Satb2	chr1	56852645	56854050	-1.14	0.10
Satb2	chr1	56928445	56928625	-1.56	-0.30
Satb2	chr1	57027968	57028178	-2.65	-0.59
Sumo1	chr1	59696455	59697048	-0.33	-0.01
Ino80d	chr1	63104148	63108707	-0.37	-0.01
Ino80d	chr1	63121010	63140028	-2.38	-0.17
Bard1	chr1	71121361	71122020	-1.25	-0.38
Usp37	chr1	74485767	74485858	-0.67	-0.28
Tuba4a	chr1	75211607	75212025	-1.98	0.61
Tuba4a	chr1	75212113	75215828	-13.90	-0.27
Scg2	chr1	79432859	79433100	-0.81	4.46
Cul3	chr1	80263601	80265851	-6.24	0.34
Cul3	chr1	80336915	80337005	-0.92	-0.29
Hdac4	chr1	93829331	93829960	-0.46	0.09
St8sia4	chr1	97487698	97523934	-12.00	0.49
St8sia4	chr1	97523993	97550125	-0.30	0.00
St8sia4	chr1	97563781	97564171	-0.35	0.02
Ikbke	chr1	133151178	133153534	-4.45	-0.31
Ikbke	chr1	133172403	133173518	-2.95	-0.52
Mdm4	chr1	134888236	134893148	-1.07	-0.08
Rnf2	chr1	153318438	153320426	-0.40	0.03
Lamc1	chr1	155068083	155074682	-60.80	-0.72
Lamc1	chr1	155080624	155081598	-3.02	-0.51
Lamc1	chr1	155179317	155179737	-8.10	-0.55
Qsox1	chr1	157626418	157628411	-6.98	-0.66
Klhl20	chr1	163019017	163019228	-1.31	-0.43
Klhl20	chr1	163020426	163023835	-2.61	0.52
Klhl20	chr1	163039357	163039897	-3.74	-0.46
Dcaf6	chr1	167281092	167281331	-1.18	-0.39
Uap1	chr1	172072402	172072642	-3.37	0.76
Uap1	chr1	172096797	172105047	-0.80	-0.15
Usp21	chr1	173216496	173218062	-11.50	-1.09
Chml	chr1	177616085	177617585	-4.84	0.48
Chml	chr1	177617764	177618484	-4.49	0.64
Dtl	chr1	193363098	193363969	-1.56	0.77
Dtl	chr1	193364536	193365016	-0.36	0.06
Kin	chr2	10013317	10014328	-0.32	-0.01

Stam	chr2	14067864	14068522	-0.94	0.13
Commd3	chr2	18596490	18597638	-0.74	-0.08
Bmi1	chr2	18605667	18606468	-0.37	-0.01
Man1b1	chr2	25188382	25188990	-22.80	3.20
Fbxw5	chr2	25358984	25359776	-3.57	-0.40
Fbxw5	chr2	25360013	25360422	-1.07	-0.24
Rxra	chr2	27615158	27615398	-3.09	-0.87
Rxra	chr2	27615487	27616386	-13.50	-1.14
Rxra	chr2	27617342	27618510	-7.46	-0.74
Dolpp1	chr2	30252653	30254975	-1.91	-0.41
Usp20	chr2	30865719	30865840	-3.26	-1.16
Nup214	chr2	31888785	31893623	-1.70	-0.12
Pomt1	chr2	32109927	32110226	-0.88	-0.25
Golga2	chr2	32158521	32160180	-1.33	-0.18
Golga2	chr2	32161974	32162244	-0.31	-0.01
Dpm2	chr2	32427908	32429091	-7.50	-0.39
St6galnac4	chr2	32451235	32451416	-2.40	0.71
St6galnac4	chr2	32452522	32453780	-6.35	-0.35
Mbd5	chr2	49101193	49101313	-0.37	-0.10
Mbd5	chr2	49111746	49113872	-0.35	-0.01
Mbd5	chr2	49134386	49169197	-1.26	0.48
Mbd5	chr2	49171947	49172008	-0.60	1.30
Klhl41	chr2	69508683	69508923	-1.52	0.86
Dcaf17	chr2	70928077	70928138	-0.87	0.71
Dync1i2	chr2	71049792	71050012	-0.90	0.33
Psmc3	chr2	90894439	90895182	-3.33	-0.20
Traf6	chr2	101530344	101537929	-2.95	-0.21
Rtf1	chr2	119558176	119559166	-0.81	0.09
Rtf1	chr2	119560243	119560484	-0.48	0.12
Pdia3	chr2	121263353	121263680	-0.63	0.03
Usp8	chr2	126567877	126568387	-5.37	-0.31
Prnd	chr2	131735723	131776618	-0.32	0.05
Tbc1d20	chr2	152119607	152127898	-2.40	-0.40
Tbc1d20	chr2	152137106	152137759	-2.58	-0.27
Asxl1	chr2	153171874	153177942	-0.65	-0.21
Asxl1	chr2	153222520	153228307	-3.63	-0.12
Actr5	chr2	158450648	158451037	-2.64	-0.85
Actr5	chr2	158462488	158464947	-0.38	0.05
Top1	chr2	160471632	160471932	-2.62	-0.31
Top1	chr2	160547243	160547394	-2.75	0.37
Ctsa	chr2	164659257	164659646	-12.00	-0.34
Rae1	chr2	172840787	172841203	-9.79	-0.73
Rab22a	chr2	173521497	173526997	-5.09	-0.36

Tubb1	chr2	174282485	174283323	-0.40	0.53
Adrm1	chr2	179906292	179906906	-2.50	-0.41
Arfgap1	chr2	180714403	180717020	-14.60	-0.46
Gata3	chr2	9779597	9784722	-2.88	0.70
Gata3	chr2	9790440	9796250	-0.57	-0.22
Tubb4b	chr2	25077825	25079655	-27.50	-0.32
Tubb4b	chr2	25079713	25080192	-0.32	0.02
Traf2	chr2	25374427	25375145	-2.44	-0.26
Sec16a	chr2	26265370	26265551	-1.04	-0.25
Sec16a	chr2	26265610	26266241	-5.32	-0.43
Sec16a	chr2	26277717	26279943	-0.47	-0.05
Sec16a	chr2	26294102	26294703	-2.30	0.19
Sec16a	chr2	26295032	26300736	-0.84	0.04
Ntng2	chr2	29062977	29103560	-0.46	-0.14
Dolk	chr2	30139748	30140407	-0.70	-0.13
Dolk	chr2	30140646	30141874	-2.09	-0.30
Asb6	chr2	30678647	30680212	-9.57	-0.39
Fbxw2	chr2	34661048	34661869	-4.78	-0.39
Fbxw2	chr2	34662764	34664040	-0.64	-0.16
Fbxw2	chr2	34668436	34678672	-6.10	-0.35
Fbxw2	chr2	34681468	34681725	-0.46	0.13
Psmd5	chr2	34707993	34709823	-1.65	-0.19
Psmd5	chr2	34726274	34726482	-4.41	-0.82
Rab14	chr2	35056311	35056550	-6.69	-0.39
Psmb7	chr2	38498946	38499426	-3.13	-0.36
Ifih1	chr2	62472979	62473100	-1.19	0.92
Ifih1	chr2	62477495	62484252	-1.48	0.23
Galnt3	chr2	65945049	65962670	-1.01	0.32
Sp3	chr2	72775988	72779227	-8.58	0.42
Sp3	chr2	72784602	72809399	-22.20	0.39
Slc35c1	chr2	92293995	92294175	-2.01	-0.48
Slc35c1	chr2	92294234	92294832	-3.36	-0.37
Slc35c1	chr2	92298872	92299589	-0.36	0.02
Rcn1	chr2	105227977	105229255	-5.40	-0.46
Rcn1	chr2	105239207	105239446	-1.42	-0.39
Ino80	chr2	119200037	119200278	-1.42	0.29
Trp53bp1	chr2	121030697	121033675	-0.56	-0.10
Trp53bp1	chr2	121034169	121046975	-2.75	-0.26
Trp53bp1	chr2	121061313	121069657	-0.65	0.06
Cops2	chr2	125657568	125660555	-1.53	-0.10
Pcna	chr2	132077710	132078916	-7.08	0.28
Psmf1	chr2	151543111	151543202	-1.31	-0.87
Psmf1	chr2	151543649	151544038	-0.30	0.01

Edem2	chr2	155527795	155528302	-2.18	-0.22
Neurl2	chr2	164656229	164658385	-0.74	0.13
B4galt5	chr2	167124332	167124601	-0.54	-0.04
B4galt5	chr2	167126508	167130515	-27.30	-0.43
B4galt5	chr2	167174439	167174678	-8.54	-1.08
Ctsz	chr2	174252994	174254182	-128.00	-0.34
Usp13	chr3	32832089	32832389	-0.75	3.43
Rab33b	chr3	51288366	51298069	-4.15	0.43
Fbxw7	chr3	84619498	84694070	-0.58	-0.12
Fbxw7	chr3	84694129	84756226	-0.51	0.05
Fbxw7	chr3	84780110	84781955	-0.35	0.02
Muc1	chr3	89033855	89035018	-25.00	2.71
Rab13	chr3	90028667	90029826	-0.48	0.13
Otud7b	chr3	95908449	95908540	-1.47	-0.65
Otud7b	chr3	95956546	95960193	-6.78	-0.26
Hist2h2be	chr3	96025043	96025490	-2.37	-0.78
Hist2h2be	chr3	96026708	96027215	-0.64	-0.19
Hist2h2be	chr3	96027422	96027483	-10.00	3.73
Sec22b	chr3	97705149	97711158	-2.16	-0.33
Sec22b	chr3	97725110	97725468	-2.27	-0.20
Psma5	chr3	108059873	108060022	-0.64	3.39
Dph5	chr3	115591100	115591191	-0.73	0.31
Dph5	chr3	115631611	115632001	-0.66	-0.12
Ube2d3	chr3	135101656	135102228	-19.60	-0.75
Bcl10	chr3	145587232	145587592	-4.99	-0.42
Bcl10	chr3	145588099	145593556	-3.89	-0.41
Usp33	chr3	152031228	152033363	-11.50	-0.53
Pigk	chr3	152448420	152449109	-0.30	-0.01
Pigk	chr3	152449855	152450483	-0.83	0.17
Pigk	chr3	152451617	152451828	-0.44	0.13
Pex2	chr3	5561142	5563166	-2.98	-0.30
Phc3	chr3	30805887	30813151	-0.72	-0.12
Phc3	chr3	30864733	30868337	-1.81	-0.73
Ccna2	chr3	36470827	36470918	-0.54	-0.57
Commd2	chr3	57450273	57450573	-1.50	-0.35
Siah2	chr3	58479649	58495381	-3.84	-0.27
Siah2	chr3	58495620	58496010	-0.94	0.28
Psmb4	chr3	94690701	94690880	-8.50	-0.41
Psmd4	chr3	94836626	94836894	-2.54	-0.16
Psmd4	chr3	94840526	94846467	-8.21	-0.35
Man1a2	chr3	100371041	100382434	-0.59	0.05
Man1a2	chr3	100460000	100489396	-0.70	-0.04
Csf1	chr3	107544832	107548597	-0.68	0.16

Csf1	chr3	107548745	107550736	-0.68	-0.96
Csf1	chr3	107551093	107553341	-0.96	0.24
Rwdd3	chr3	120859364	120862119	-0.35	-0.04
Ank2	chr3	126625933	126626054	-0.57	-1.42
Ank2	chr3	126626293	126626744	-0.78	0.77
Sec24b	chr3	129686102	129687101	-1.82	-0.14
Sec24b	chr3	129743865	129763735	-15.50	-0.56
Fpgt	chr3	154749523	154753147	-2.83	0.28
Rab2a	chr4	8462790	8462910	-1.92	-0.42
Fbxl4	chr4	22303713	22313289	-1.60	-0.28
Map3k7	chr4	32051081	32051232	-3.02	-1.01
Map3k7	chr4	32104109	32107267	-5.97	0.38
Map3k7	chr4	32110051	32110231	-0.27	0.00
Ube2r2	chr4	41137807	41138136	-0.41	-0.03
Dcaf10	chr4	45355032	45355482	-3.02	-0.62
Dcaf10	chr4	45385889	45387298	-0.47	-0.06
Nans	chr4	46515563	46516280	-1.13	-0.18
Tgfbr1	chr4	47419877	47423777	-9.55	1.12
Stx17	chr4	48197791	48198211	-1.94	-0.55
Rad23b	chr4	55398385	55402936	-0.38	-0.01
Rraga	chr4	86221606	86222293	-1.18	-0.09
Usp24	chr4	105988817	105989117	-2.50	-0.67
Usp24	chr4	106111387	106111867	-5.72	-0.38
Usp24	chr4	106112884	106113842	-1.36	0.15
Ndc1	chr4	107083754	107086943	-1.16	0.18
Lrrc41	chr4	115761466	115762183	-23.20	-0.55
Lrrc41	chr4	115768961	115769201	-3.73	-0.33
Trappc3	chr4	125939648	125949998	-0.36	-0.03
Trappc3	chr4	125951220	125952918	-0.98	0.08
Clspn	chr4	126237254	126239019	-2.42	-0.81
Clspn	chr4	126243253	126249609	-1.43	-0.48
Clspn	chr4	126270339	126270758	-2.44	-0.82
Phc2	chr4	128404910	128405061	-0.82	-0.46
Phc2	chr4	128424470	128429198	-18.30	-0.40
Phc2	chr4	128429466	128429976	-16.20	-0.47
Ptp4a2	chr4	129497464	129516421	-0.57	0.05
Fuca1	chr4	135476640	135476908	-6.65	-0.76
Fuca1	chr4	135495027	135495325	-0.87	0.15
Usp48	chr4	137212056	137213554	-0.56	0.05
Mul1	chr4	137990586	137996001	-8.75	-0.48
Mul1	chr4	137996358	137996837	-1.10	-0.33
Capzb	chr4	138748845	138794826	-3.32	-0.32
Klhl21	chr4	151383209	151383749	-20.10	-1.89

Klhl21	chr4	151383868	151389959	-10.20	-0.52
Klhl21	chr4	151390257	151390438	-3.61	1.07
Icmt	chr4	151674797	151677875	-6.72	-0.48
Ripk2	chr4	16050670	16051621	-4.17	-0.41
Ripk2	chr4	16090436	16090645	-16.00	-1.44
Manea	chr4	26254667	26273799	-1.17	0.13
Ddx58	chr4	40152603	40155547	-1.90	-0.20
Ddx58	chr4	40186678	40186858	-0.44	0.12
Topors	chr4	40206906	40216844	-74.50	0.55
B4galt1	chr4	40753786	40754830	-1.36	0.12
B4galt1	chr4	40770597	40800613	-1.85	0.25
B4galt1	chr4	40800671	40801001	-2.65	-0.48
Gne	chr4	44047905	44048266	-2.53	-0.83
Gne	chr4	44049704	44051897	-7.07	-0.91
Gne	chr4	44068129	44079645	-3.41	-0.45
Fbxo10	chr4	45048045	45048374	-1.12	1.75
Fbxo10	chr4	45048642	45048793	-0.22	0.14
Fbxo10	chr4	45071323	45071981	-0.27	-0.03
Alg2	chr4	47482973	47483543	-1.56	0.21
Klhl9	chr4	88366049	88367814	-9.57	0.24
Mysm1	chr4	94631863	94632462	-1.59	0.20
Dph2	chr4	117562115	117562176	-0.32	0.08
Dph2	chr4	117562463	117563268	-5.52	-0.58
Dph2	chr4	117564417	117564628	-0.41	-0.10
St3gal3	chr4	117604757	117612678	-1.17	-0.15
Cdc20	chr4	118109887	118109948	-0.76	-0.68
Wdtc1	chr4	132849782	132850290	-1.99	-0.39
Pigv	chr4	133218171	133220780	-1.21	0.27
Dhdds	chr4	133526737	133536084	-0.70	-0.07
Man1c1	chr4	134119221	134119671	-0.57	0.09
Man1c1	chr4	134196206	134260205	-5.72	-0.33
Otud3	chr4	138451293	138453499	-0.36	0.02
Pex14	chr4	148335151	148335868	-1.28	-0.14
Pex14	chr4	148358178	148473921	-0.44	-0.05
Spsb1	chr4	149271916	149281307	-0.79	-0.50
Park7	chr4	150271241	150271562	-12.30	-0.42
Vwa1	chr4	155147147	155147208	-0.18	0.74
Nupl2	chr5	23687726	23687877	-0.80	0.38
Nupl2	chr5	23687966	23689616	-1.38	-0.19
Nub1	chr5	24191781	24195199	-0.68	-0.33
Nub1	chr5	24216043	24216194	-0.77	-0.21
Galnt11	chr5	24771112	24771321	-1.25	-0.40
Bst1	chr5	44210131	44210281	-0.40	-0.14

Klhl5	chr5	65558211	65558721	-0.75	-0.12
Dcun1d4	chr5	73950743	73951343	-0.46	0.13
Uso1	chr5	92630205	92631431	-0.46	-0.02
Art3	chr5	92821553	92821820	-0.86	2.58
Spp1	chr5	104864129	104865280	-280.00	-0.71
Pigg	chr5	108776899	108777230	-2.37	-0.73
Usp30	chr5	114572362	114572933	-6.41	-0.79
Rab35	chr5	116093492	116096664	-1.72	-0.10
Wsb2	chr5	117827463	117828091	-9.87	-0.54
Fbxo21	chr5	118452063	118458618	-6.36	-0.76
Psmd9	chr5	123678228	123678437	-0.34	0.03
Sumf2	chr5	130338841	130339081	-1.81	-0.86
Kctd7	chr5	130628217	130628785	-0.53	-0.24
Vgf	chr5	137506930	137509043	-3.19	-0.55
Cops6	chr5	138605188	138605278	-0.36	-0.10
Fam20c	chr5	139231034	139232081	-22.20	-0.41
Fam20c	chr5	139284018	139286017	-18.70	-0.53
Foxk1	chr5	142932437	142934770	-0.93	-0.07
Kdelr2	chr5	144165498	144165618	-1.27	-0.30
Trrap	chr5	145550403	145551932	-3.37	-0.52
Trrap	chr5	145618437	145620522	-11.00	-0.41
Preb	chr5	31254099	31254460	-1.14	0.42
Preb	chr5	31254638	31254759	-0.75	0.43
Preb	chr5	31255148	31255718	-1.31	0.28
Preb	chr5	31256197	31256558	-0.80	0.21
Preb	chr5	31257096	31257217	-0.42	-0.16
Preb	chr5	31257366	31258308	-13.30	-0.41
Preb	chr5	31262403	31262704	-7.96	-0.81
Ctbp1	chr5	33590909	33591269	-25.10	-0.66
Tnip2	chr5	34842175	34842385	-0.32	-0.02
Tada2b	chr5	36818228	36826725	-1.61	-0.17
Wfs1	chr5	37358091	37358361	-1.79	-0.98
Wfs1	chr5	37358629	37359738	-0.65	-0.17
Fbxl5	chr5	44142150	44149098	-2.88	-0.56
Fbxl5	chr5	44149396	44151973	-0.84	0.08
Commd8	chr5	72550577	72551087	-5.42	0.48
Nup54	chr5	92845130	92846543	-1.18	-0.16
Sec31a	chr5	100790907	100791058	-0.72	-0.09
Abraxas1	chr5	101234923	101235430	-2.15	-0.39
Mlec	chr5	115597834	115600228	-0.61	-0.05
Mlec	chr5	115600898	115608185	-12.50	-0.43
Suds3	chr5	117542584	117542973	-0.58	-0.07
Suds3	chr5	117556655	117565972	-3.08	0.27

Fbxw8	chr5	118515497	118515797	-0.28	0.02
Fbxw8	chr5	118605107	118605467	-13.80	-1.01
Ubc	chr5	125866453	125867671	-172.00	0.73
Ubc	chr5	125867938	125868147	-3.15	0.20
Ubc	chr5	125868235	125870298	-22.80	-0.40
Pom121	chr5	135853620	135857139	-7.02	-0.36
Pom121	chr5	135857525	135857795	-2.51	-0.32
Fbxl18	chr5	143633766	143634006	-6.05	-1.06
Fbxl18	chr5	143647153	143648949	-184.00	-2.23
Usp42	chr5	144471582	144472847	-0.29	-0.01
Usp42	chr5	144474632	144476980	-6.09	-0.41
Usp42	chr5	144477517	144477978	-4.07	-0.78
Usp12	chr5	147548897	147550725	-1.72	-0.27
C1galt1	chr6	7816416	7821773	-6.24	0.33
Calu	chr6	29298148	29306501	-0.80	-0.24
Calu	chr6	29322584	29324851	-2.79	-0.14
Nup205	chr6	35127615	35133895	-0.30	0.02
Rab19	chr6	39339614	39340231	-1.34	-0.57
Cul1	chr6	47404345	47445165	-3.56	0.31
Cul1	chr6	47474617	47476108	-1.64	-0.15
Tnip3	chr6	65564734	65582063	-0.35	0.01
Rnf103	chr6	71458868	71460366	-1.54	0.19
St3gal5	chr6	72092231	72097086	-1.87	-0.24
St3gal5	chr6	72099116	72104266	-2.73	0.22
Mogs	chr6	83067105	83068743	-16.50	-0.34
Dctn1	chr6	83140226	83141067	-0.45	0.03
Kbtbd8	chr6	95071991	95077327	-2.49	0.37
Mitf	chr6	97960423	97968956	-1.11	-0.08
Mitf	chr6	97969584	97970094	-0.70	-0.09
Edem1	chr6	108778634	108779024	-6.01	-0.33
Edem1	chr6	108805488	108805668	-3.63	-0.20
Ttll3	chr6	113357264	113357355	-1.22	0.45
Ttll3	chr6	113358988	113359318	-0.96	-0.26
Ttll3	chr6	113362865	113364057	-1.14	-0.28
Ttll3	chr6	113364206	113364505	-4.11	-0.73
Vhl	chr6	113578147	113579978	-0.46	-0.06
Fbxl14	chr6	119429685	119431451	-0.36	-0.01
Rad52	chr6	119865354	119869071	-3.47	-0.44
Rad52	chr6	119870988	119871109	-0.75	0.26
Rad52	chr6	119871288	119872841	-0.35	0.03
Spsb2	chr6	124758812	124760455	-90.80	-1.86
Cmas	chr6	142705235	142705504	-2.29	-0.47
Klhl42	chr6	147040014	147050109	-0.97	-0.28

Klhl42	chr6	147050168	147056940	-3.38	-0.59
Etfbkmt	chr6	149092586	149092945	-0.59	-0.44
Etfbkmt	chr6	149095803	149099215	-2.26	-1.34
Ube2h	chr6	30163827	30164395	-1.91	0.24
Ube2h	chr6	30164783	30164933	-0.40	-0.15
Hipk2	chr6	38647898	38665995	-46.30	-0.52
Hipk2	chr6	38767930	38787077	-4.22	-0.11
Hipk2	chr6	38825800	38826040	-0.40	-0.04
Nod1	chr6	54874531	54874682	-0.75	1.04
Nod1	chr6	54893168	54893616	-0.68	0.56
Nod1	chr6	54893675	54894034	-0.75	0.80
Nod1	chr6	54894242	54894451	-0.24	-0.71
Nod1	chr6	54894600	54897981	-1.54	1.88
Rnf181	chr6	72310239	72310565	-2.88	-0.36
Tgoln1	chr6	72561643	72566785	-5.10	-0.11
Ino80b	chr6	83074730	83075023	-0.55	-0.20
Rab43	chr6	87742107	87744044	-5.29	-0.48
Rab43	chr6	87761473	87761773	-1.00	-0.35
Nup210	chr6	90963060	90963660	-3.36	-0.50
Xpc	chr6	91439866	91440195	-0.47	-0.16
Xpc	chr6	91448159	91450008	-8.98	-0.94
Uba3	chr6	97134556	97134676	-1.20	0.47
Sumf1	chr6	108058030	108058509	-1.48	0.21
Sumf1	chr6	108135337	108135577	-0.45	-0.07
Rad18	chr6	112577061	112577661	-0.87	-0.24
Tada3	chr6	113316892	113320261	-0.78	0.17
Tada3	chr6	113322347	113324488	-2.13	0.54
Tada3	chr6	113324725	113325142	-2.16	0.45
Phc1	chr6	122273438	122273618	-0.97	0.44
Phc1	chr6	122275560	122275830	-0.61	-0.25
Pex5	chr6	124347220	124347399	-0.29	-0.01
Usp5	chr6	124765306	124767020	-15.00	-0.58
Usp5	chr6	124776424	124779465	-1.09	-0.24
Napa	chr7	16702038	16703324	-5.35	-0.18
Ccdc8	chr7	17580174	17580295	-0.16	0.42
Trappc6a	chr7	20094077	20099776	-2.15	-0.28
Plaur	chr7	25247518	25251694	-110.00	-1.93
Plaur	chr7	25260294	25260713	-183.00	-1.49
B3gnt8	chr7	26415220	26415521	-5.92	-0.75
Paf1	chr7	29178014	29178133	-0.41	-0.07
Paf1	chr7	29181953	29184402	-0.48	0.02
Josd2	chr7	51723425	51723545	-0.29	-0.02
Nup62	chr7	52071739	52086176	-19.00	-0.23

Hrc	chr7	52590638	52591029	-0.75	-0.98
Hrc	chr7	52591478	52591689	-0.64	0.91
Prmt3	chr7	57112858	57113008	-1.15	-0.24
Herc2	chr7	63485072	63486928	-1.61	0.14
Klhl25	chr7	82993313	83012167	-0.30	0.00
Klhl25	chr7	83012375	83018269	-2.48	-0.60
Klhl25	chr7	83018657	83018926	-0.33	0.09
Ctsc	chr7	95437245	95437485	-0.96	0.45
Ctsc	chr7	95458037	95458547	-0.94	0.12
Rab30	chr7	99984250	99984670	-0.49	-0.72
Alg8	chr7	104540130	104540578	-2.19	-0.39
Arrb1	chr7	106748328	106749813	-0.89	-0.21
Arrb1	chr7	106752471	106752711	-1.70	-0.44
Usp17lc	chr7	110567241	110567301	-0.11	0.00
Ctr9	chr7	118173736	118177414	-3.03	-0.38
Ctr9	chr7	118198901	118199621	-1.34	-0.16
Usp47	chr7	119167079	119167140	-0.41	0.13
Usp47	chr7	119234919	119245370	-0.77	-0.05
Usp47	chr7	119252192	119254002	-1.50	-0.16
Spon1	chr7	121183502	121183922	-0.45	0.48
Ccp110	chr7	125864970	125865449	-1.59	-0.38
Ccp110	chr7	125865867	125867971	-0.97	-0.19
Dctn5	chr7	129276554	129277689	-0.48	-0.08
Dctn5	chr7	129291791	129292440	-0.33	-0.01
Rnf40	chr7	134739594	134740336	-9.26	-0.41
Rnf40	chr7	134744053	134746671	-2.53	-0.16
Fbxl19	chr7	134890288	134891744	-4.33	-0.90
Fbxl19	chr7	134891803	134891893	-0.66	-0.35
Fbxl19	chr7	134893270	134904820	-11.30	-0.67
Fbxl19	chr7	134906111	134911817	-1.15	-0.18
Fbxl19	chr7	134911876	134912442	-2.21	-0.45
Sec23ip	chr7	135888443	135893982	-17.50	-0.53
Abraxas2	chr7	140072616	140072677	-0.76	0.42
Abraxas2	chr7	140072975	140075538	-1.28	0.12
Zranb1	chr7	140122884	140158368	-4.10	-0.27
Zranb1	chr7	140174515	140175634	-0.78	-0.16
Zranb1	chr7	140175516	140175963	-0.36	-0.03
Pnpla2	chr7	148641086	148641236	-0.31	0.02
Pnpla2	chr7	148645626	148645985	-2.36	-0.30
Tfpt	chr7	3581426	3581486	-1.90	-0.92
Ppp6r1	chr7	4610133	4610552	-20.20	-0.92
Ube2m	chr7	13623018	13623619	-6.02	-0.45
Sae1	chr7	16912835	16913105	-0.93	-0.11

Calm3	chr7	17502039	17502309	-1.46	-0.09
Apoe	chr7	20282098	20282861	-0.83	-0.19
Ceacam1	chr7	26247739	26248638	-4.67	0.32
Ceacam1	chr7	26257014	26258919	-1.16	-0.37
Ceacam1	chr7	26259008	26274241	-0.36	-0.02
Sptbn4	chr7	28149267	28149508	-0.42	1.58
Psmd8	chr7	29965393	29965573	-13.80	-0.43
Nucb1	chr7	52758551	52762906	-4.53	-0.38
Nucb1	chr7	52765032	52765778	-4.41	-0.49
Nsmce3	chr7	72016624	72017749	-1.96	-0.18
Asb7	chr7	73792534	73823980	-3.67	-0.42
Asb7	chr7	73834207	73834387	-0.58	0.31
Mfge8	chr7	86278653	86279192	-3.32	-0.24
Man2a2	chr7	87494918	87495788	-3.80	0.21
Man2a2	chr7	87496117	87497911	-1.12	0.09
Man2a2	chr7	87515749	87516012	-0.66	-0.32
Furin	chr7	87534797	87536644	-178.00	-0.71
Furin	chr7	87538324	87540367	-66.60	-0.82
Furin	chr7	87542790	87550317	-43.80	-0.68
Blm	chr7	87647292	87647681	-3.04	-0.71
Blm	chr7	87654437	87658369	-0.32	-0.01
Neu3	chr7	106961469	106962782	-1.93	0.47
Nup98	chr7	109268003	109269291	-7.87	-0.22
Nup98	chr7	109283070	109283401	-23.60	-0.89
Usp17ld	chr7	110398718	110398868	-0.21	-0.17
Usp17ld	chr7	110399373	110399493	-0.18	0.68
Usp17lb	chr7	111989694	111989755	-0.14	0.42
Dcun1d3	chr7	127000827	127038861	-10.40	-0.47
Cog7	chr7	129066679	129066829	-1.49	-0.61
Cog7	chr7	129121227	129125207	-0.94	-0.20
Nsmce4a	chr7	137690685	137690865	-3.11	-1.21
Fuom	chr7	147285428	147285765	-5.94	-1.24
Bet1l	chr7	148039342	148042252	-24.40	-0.67
Trappc5	chr8	3676506	3679785	-6.86	-0.39
Trappc5	chr8	3680083	3680622	-2.35	-0.40
F7	chr8	13034729	13035419	-0.25	-0.01
Cul4a	chr8	13144401	13146773	-0.97	0.16
Cul4a	chr8	13146832	13147072	-0.47	0.07
Thsd1	chr8	23353920	23354367	-0.35	-0.59
Thsd1	chr8	23369018	23369287	-2.72	-4.39
Ank1	chr8	24242642	24242942	-0.36	-0.31
Ank1	chr8	24259141	24259292	-0.17	-0.12
Fkbp8	chr8	73051641	73051859	-1.76	-0.42

Fkbp8	chr8	73051918	73053571	-4.53	-0.26
Fkbp8	chr8	73058839	73058959	-6.16	-0.46
Rab3a	chr8	73282404	73282465	-0.44	0.34
Babam1	chr8	73920753	73921648	-6.08	-0.54
Dda1	chr8	73998657	73999436	-3.49	-0.18
Dda1	chr8	73999764	73999855	-0.88	0.22
Rab8a	chr8	74704051	74704674	-6.42	-0.29
Nr3c2	chr8	79432403	79432554	-0.61	1.74
Nr3c2	chr8	79433567	79433776	-0.74	-1.52
Dhps	chr8	87595655	87595834	-0.47	0.12
Cyld	chr8	91220956	91226218	-0.50	0.37
Cyld	chr8	91226307	91233793	-2.31	0.21
Fbxl8	chr8	107790925	107791573	-0.65	0.30
Fbxl8	chr8	107791922	107793108	-0.55	0.10
Cog4	chr8	113370709	113371100	-5.20	-1.02
Cog4	chr8	113405654	113406134	-2.02	-0.31
St3gal2	chr8	113444003	113491411	-1.25	-0.16
St3gal2	chr8	113493699	113495173	-5.29	-0.56
Gan	chr8	119728191	119729086	-7.70	0.65
Usp10	chr8	122460483	122471049	-1.73	0.10
Usp10	chr8	122480587	122481247	-0.46	-0.03
Trappc2l	chr8	125135525	125136985	-0.45	-0.17
Tubb3	chr8	125942898	125945049	-0.53	-0.56
Galnt2	chr8	126867222	126868362	-0.87	-0.10
Rab20	chr8	11453976	11478499	-58.40	-1.28
Dcun1d2	chr8	13256291	13257907	-1.09	-0.11
Gas6	chr8	13465611	13465821	-0.49	-0.15
Vdac3	chr8	23687812	23688050	-0.92	0.16
Pomk	chr8	27092801	27100129	-0.41	-0.02
Dctn6	chr8	35169435	35171565	-0.29	-0.02
Ing2	chr8	48753850	48758943	-1.32	0.15
Ing2	chr8	48760272	48760513	-0.64	-0.35
Galnt7	chr8	60004507	60010487	-11.50	0.30
Galnt7	chr8	60062627	60131852	-20.80	-0.39
Klhl2	chr8	67219391	67219870	-0.73	-0.15
Ano8	chr8	74000246	74000426	-1.28	-1.09
Ano8	chr8	74000574	74000814	-0.40	0.31
Ano8	chr8	74003128	74005058	-7.55	-0.71
B3gnt3	chr8	74216002	74217291	-1.83	-0.34
B3gnt3	chr8	74217349	74227314	-0.35	0.05
Rad23a	chr8	87358550	87358701	-2.33	-0.85
Calr	chr8	87366315	87366863	-41.40	-0.26
Calr	chr8	87370234	87370800	-11.20	-0.26

Amfr	chr8	96496474	96497043	-0.34	-0.01
Dync1li2	chr8	106944120	106944421	-2.39	-0.28
Dync1li2	chr8	106966646	106966737	-0.40	0.11
B3gnt9	chr8	107777431	107779051	-4.70	0.62
Psmb10	chr8	108461631	108461967	-2.80	-0.63
Cog8	chr8	109570548	109572735	-0.43	0.03
Cog8	chr8	109572794	109572975	-4.23	-0.66
Cog8	chr8	109576156	109578097	-1.38	-0.12
Psmd7	chr8	110104338	110108077	-4.03	0.12
Mbtps1	chr8	122032081	122032770	-0.81	0.07
Mbtps1	chr8	122070114	122082661	-0.67	0.06
Fbxo31	chr8	124075878	124076147	-0.29	0.02
Mvd	chr8	124957554	124958775	-4.80	-0.29
Pigyl	chr9	21961289	21962769	-0.41	0.06
Opcml	chr9	28210785	28210966	-0.62	-3.17
Opcml	chr9	28211054	28211415	-0.32	-1.17
Opcml	chr9	28729364	28729455	-0.20	-0.85
Opcml	chr9	28729664	28729784	-1.29	-3.17
Opcml	chr9	28730173	28730744	-1.82	1.53
Nfrkb	chr9	31218903	31227409	-0.77	0.09
Usp2	chr9	43883786	43884026	-0.81	2.12
Usp2	chr9	43902032	43902302	-0.96	-0.35
Dpagt1	chr9	44141235	44141504	-0.33	0.02
Apoa1	chr9	46038131	46038430	-0.42	1.51
Alg9	chr9	50651034	50651633	-0.34	0.02
Fbxo22	chr9	55066270	55071672	-1.24	0.12
Fbxo22	chr9	55071791	55072031	-0.53	0.10
Sin3a	chr9	56958532	56958892	-0.32	-0.02
Sin3a	chr9	56973115	56975576	-0.84	0.11
Rora	chr9	69226715	69226835	-0.32	0.16
Adam10	chr9	70526807	70526958	-1.07	0.28
Adam10	chr9	70626086	70626297	-2.80	0.46
Rab27a	chr9	72892731	72908786	-0.39	-0.66
Rab27a	chr9	72943089	72943780	-0.59	0.27
Leo1	chr9	75289330	75294926	-0.87	-0.07
Cish	chr9	107202782	107203759	-21.00	3.35
Tmem115	chr9	107436364	107440987	-2.51	0.14
Usp4	chr9	108293381	108294591	-3.30	-0.22
Usp19	chr9	108393096	108395299	-1.59	-0.15
Cdc25a	chr9	109778082	109778918	-5.19	-0.75
Cdc25a	chr9	109794596	109795525	-0.60	-0.08
Glb1	chr9	114310242	114326114	-1.32	0.33
Glb1	chr9	114368800	114373509	-0.47	-0.05

Glb1	chr9	114374377	114383495	-4.81	0.37
Dync1li1	chr9	114632686	114632895	-0.93	0.16
Dync1li1	chr9	114632812	114633189	-1.24	0.13
Wdr48	chr9	119833314	119835040	-0.42	0.02
Birc2	chr9	7818614	7818734	-0.55	-0.29
Birc2	chr9	7833610	7834657	-0.90	0.13
Birc2	chr9	7834955	7835195	-1.05	0.42
Birc3	chr9	7848969	7850928	-1.17	-0.17
Birc3	chr9	7860311	7872961	-0.30	0.00
Fbxl12	chr9	20448880	20449151	-0.85	-0.48
Keap1	chr9	21034473	21038380	-0.55	0.03
Keap1	chr9	21041615	21042276	-1.55	-0.22
Keap1	chr9	21043205	21043686	-0.40	0.13
Rab3d	chr9	21714741	21715042	-1.16	-0.47
Aplp2	chr9	30957469	30957679	-1.25	-0.13
Aplp2	chr9	30985646	31019400	-0.58	0.12
Arcn1	chr9	44551783	44551994	-1.44	0.13
Arcn1	chr9	44568266	44575891	-2.60	-0.34
Cul5	chr9	53425530	53429279	-5.02	0.34
Rab39	chr9	53494590	53494829	-0.25	-0.22
Pml	chr9	58066214	58066305	-1.33	0.78
Pml	chr9	58067472	58068881	-10.10	-0.47
Pml	chr9	58078383	58097563	-4.44	-0.27
Senp8	chr9	59584180	59587831	-0.78	0.14
Thsd4	chr9	59819164	59819314	-0.27	1.26
Fem1b	chr9	62643314	62659036	-48.90	0.46
Pias1	chr9	62729583	62729943	-0.50	0.10
Pias1	chr9	62799649	62828686	-0.80	0.12
Smad3	chr9	63496369	63496969	-0.55	0.19
Smad3	chr9	63497327	63498046	-0.81	0.32
Smad3	chr9	63605729	63605864	-0.51	-1.42
Usp3	chr9	66366163	66367920	-0.69	0.16
Rab8b	chr9	66695066	66696453	-10.50	0.31
Rab8b	chr9	66715068	66767482	-1.83	0.32
Pigb	chr9	72863505	72865261	-0.48	0.05
Pgm3	chr9	86447697	86448087	-3.55	-0.65
Rnf123	chr9	107954002	107954243	-6.46	0.71
Dag1	chr9	108109094	108155448	-8.64	0.23
Gorasp1	chr9	119834879	119835029	-0.52	-0.15
Gorasp1	chr9	119836695	119837112	-5.71	-0.94
Sec22c	chr9	121593476	121593866	-0.35	0.10
Shprh	chr10	10869257	10871943	-2.23	-0.37
Shprh	chr10	10882772	10884564	-4.44	-0.54

Fbxo30	chr10	11001127	11015345	-10.80	0.40
Fuca2	chr10	13223007	13225667	-1.77	-0.36
Fuca2	chr10	13234515	13235470	-1.15	0.10
Nus1	chr10	52137562	52137682	-7.69	-0.70
Nus1	chr10	52156402	52156613	-4.96	-0.41
Nus1	chr10	52158170	52158350	-1.58	-0.34
Ranbp2	chr10	57939345	57940486	-6.91	0.22
Ranbp2	chr10	57940784	57941055	-0.33	-0.01
Ranbp2	chr10	57941564	57941955	-1.34	-0.09
Ranbp2	chr10	57942134	57945844	-28.50	0.35
Ranbp2	chr10	57955388	57956782	-8.30	0.31
Pofut2	chr10	76731353	76732331	-3.21	-0.17
Sumo3	chr10	77068978	77069512	-0.42	-0.07
Ube2g2	chr10	77107280	77107637	-1.33	-0.14
Ube2g2	chr10	77107992	77108082	-0.37	-0.04
Cdc34	chr10	79144939	79145322	-5.03	-0.46
Cdc34	chr10	79148043	79151143	-18.90	-0.38
Eef2	chr10	80644273	80645254	-24.30	-0.11
Dohh	chr10	80853555	80854150	-3.43	-0.73
Eid3	chr10	82329518	82330082	-0.37	-0.03
Eid3	chr10	82330466	82330644	-3.01	1.11
Galnt4	chr10	98570977	98571038	-0.40	0.27
Galnt4	chr10	98571426	98572144	-0.49	-0.09
Galnt4	chr10	98572382	98573280	-1.98	0.41
Cpm	chr10	117121110	117121651	-1.22	0.46
Rab32	chr10	10265729	10270492	-5.49	-0.40
Tnfaip3	chr10	18721581	18725137	-0.67	-0.06
Rnf146	chr10	29064251	29064491	-0.35	0.02
Rnf146	chr10	29066497	29067546	-0.70	-0.06
Man1a	chr10	53750579	53795542	-4.76	-1.15
Ube2d1	chr10	70718144	70718592	-2.63	0.38
Trappc10	chr10	77650129	77651540	-1.80	-0.19
Pias4	chr10	80616715	80617300	-0.74	-0.10
Pias4	chr10	80619974	80625923	-1.46	-0.22
Pias4	chr10	80626491	80630465	-4.33	-0.46
Ckap4	chr10	83990030	83991369	-1.40	-0.09
Ckap4	chr10	83996275	83996633	-3.93	-0.77
Rab21	chr10	114727724	114727964	-0.37	0.01
Mdm2	chr10	117126170	117126441	-1.00	0.08
Mdm2	chr10	117126799	117129718	-14.10	0.17
Cand1	chr10	118647900	118648919	-0.49	0.03
Usp15	chr10	122550299	122558549	-0.60	0.05
Os9	chr10	126531344	126532125	-2.77	-0.49

Os9	chr10	126532363	126535514	-5.24	-0.16
Os9	chr10	126557594	126558216	-2.38	-0.34
Mbd6	chr10	126719071	126720682	-0.32	0.00
Ykt6	chr11	5855760	5856000	-4.46	-0.65
Ykt6	chr11	5866082	5866352	-0.37	-0.02
Usp34	chr11	23206894	23233439	-0.42	0.06
Usp34	chr11	23233527	23233678	-2.02	0.32
Usp34	chr11	23263863	23264511	-1.71	-0.12
Usp34	chr11	23364346	23364676	-0.48	0.05
Usp34	chr11	23388278	23389213	-1.07	-0.10
Psme4	chr11	30671774	30672135	-0.54	-0.18
Fbxw11	chr11	32644536	32644986	-0.78	-0.08
Mgat1	chr11	49057722	49075721	-5.05	-0.26
Mgat4b	chr11	50039046	50039137	-4.28	-1.00
Mgat4b	chr11	50048124	50048515	-6.26	-0.32
Galnt10	chr11	57597256	57598674	-0.70	0.22
Hist3h2a	chr11	58768661	58768929	-4.41	0.77
Nlrp3	chr11	59355086	59365364	-68.60	-0.49
Nlrp3	chr11	59379889	59380219	-0.60	0.09
Pigl	chr11	62271961	62272082	-2.00	-0.93
Aurkb	chr11	68861818	68863941	-3.92	-0.59
Trp53	chr11	69393860	69394608	-0.43	-0.06
Asgr1	chr11	69870863	69871366	-0.36	1.28
Ube2g1	chr11	72420792	72421032	-3.90	-0.86
Ube2g1	chr11	72493612	72497295	-0.33	0.03
Rtn4rl1	chr11	75079118	75079476	-0.61	2.96
Pigs	chr11	78155467	78156068	-7.36	-0.45
Suz12	chr11	79843530	79845978	-0.38	0.01
Ptrh2	chr11	86497514	86503722	-9.69	-0.43
Psmd3	chr11	98543867	98546829	-30.30	-0.74
Thra	chr11	98603186	98614709	-0.74	0.42
Thra	chr11	98614768	98617477	-2.58	-0.78
Thra	chr11	98625600	98626259	-4.68	-0.69
Thra	chr11	98629482	98629782	-10.30	1.38
Rara	chr11	98799009	98808908	-1.61	-0.58
Rara	chr11	98808997	98822329	-5.35	-0.55
Rara	chr11	98827782	98829451	-0.74	-0.25
Rara	chr11	98831600	98835508	-19.40	-0.66
Psme3	chr11	101177564	101178336	-0.62	0.07
Psme3	chr11	101182070	101183288	-0.64	-0.06
Dcaf7	chr11	105898185	105898485	-1.07	-0.15
Dcaf7	chr11	105916210	105916360	-1.24	0.22
Dcaf7	chr11	105919829	105920129	-0.82	-0.12

Arsg	chr11	109346860	109378636	-0.38	-0.02
Arsg	chr11	109433808	109434107	-0.35	0.06
Cog1	chr11	113517332	113518437	-0.57	-0.07
Birc5	chr11	117716547	117716877	-2.95	-0.53
Engase	chr11	118348561	118349312	-2.33	-0.74
Cbx2	chr11	118889293	118890939	-23.30	-1.92
Hgs	chr11	120345028	120345208	-9.55	-0.93
Gps1	chr11	120648738	120649402	-0.35	-0.06
Gps1	chr11	120649969	120650386	-3.03	-0.46
Foxk2	chr11	121160998	121169022	-0.31	0.00
Fn3k	chr11	121303142	121303262	-0.62	-3.70
Fn3k	chr11	121303589	121303799	-0.67	-3.37
B3gnt2	chr11	22735930	22759719	-2.61	-0.09
Commd1	chr11	22799727	22800026	-0.83	-0.10
Commd1	chr11	22856676	22878367	-0.64	0.15
Commd1	chr11	22878962	22882284	-2.22	-0.60
Pex13	chr11	23548957	23549346	-1.39	0.33
Pex13	chr11	23551042	23555901	-2.58	-0.39
Sptbn1	chr11	30017714	30018324	-0.64	0.10
Sptbn1	chr11	30036124	30037605	-1.75	0.16
Sptbn1	chr11	30038786	30040543	-0.57	-0.05
Stc2	chr11	31259890	31265353	-2.88	0.65
Canx	chr11	50109707	50110776	-30.20	-0.37
0610009B22Rik	chr11	51499121	51499563	-2.06	0.33
Sec24a	chr11	51556985	51570140	-6.97	-0.33
Tnip1	chr11	54724288	54731425	-26.80	-0.37
Tnip1	chr11	54740251	54744279	-11.00	-0.69
Tnip1	chr11	54753132	54754378	-8.03	-0.57
Tnip1	chr11	54769581	54776442	-0.49	0.19
Derl2	chr11	70823390	70826702	-4.56	-0.26
Hic1	chr11	74979024	74979444	-1.07	-1.34
Rpa1	chr11	75114477	75116219	-1.52	-0.18
Rpa1	chr11	75154547	75161885	-1.59	0.39
Gosr1	chr11	76543115	76548136	-0.51	0.05
Wsb1	chr11	79053599	79053839	-11.70	-0.39
Pex12	chr11	83109875	83111883	-1.19	0.24
Pigw	chr11	84691181	84691925	-0.65	-0.09
Ube2z	chr11	95911494	95911704	-1.74	-0.23
Pcgf2	chr11	97551180	97551450	-9.46	-1.52
Pcgf2	chr11	97553219	97553423	-0.65	0.22
Fbxl20	chr11	97950510	97953462	-0.74	0.16
Klhl11	chr11	100323925	100325737	-6.64	0.31
Klhl11	chr11	100333798	100334096	-2.00	0.50

Kat2a	chr11	100566059	100566924	-11.10	-0.42
Kat2a	chr11	100573600	100573691	-0.37	-0.10
Becn1	chr11	101163065	101163581	-1.84	-0.26
Brca1	chr11	101384784	101385234	-1.07	-0.42
Brca1	chr11	101385503	101386581	-0.66	0.14
Brca1	chr11	101386909	101387659	-0.94	0.23
Gosr2	chr11	103540134	103540702	-4.90	-0.28
Nsf	chr11	103684412	103685071	-1.08	-0.20
Nsf	chr11	103792113	103815310	-0.40	0.17
Ddx5	chr11	106643371	106643551	-60.70	0.67
Ddx5	chr11	106650118	106650179	-4.34	1.55
Smurf2	chr11	106683743	106684388	-0.37	-0.04
Smurf2	chr11	106781609	106781789	-3.23	-1.30
Socs3	chr11	117827669	117830291	-196.00	-0.95
Cbx8	chr11	118899838	118901471	-0.31	0.01
Cbx4	chr11	118941130	118941400	-2.21	0.97
Cbx4	chr11	118941729	118947491	-3.35	-0.13
P4hb	chr11	120421617	120422006	-31.00	-0.33
P4hb	chr11	120422453	120422573	-21.50	-0.34
Csnk1d	chr11	120819778	120820048	-1.86	-0.20
Csnk1d	chr11	120823809	120824109	-0.87	-0.12
Csnk1d	chr11	120824767	120826288	-4.93	-0.25
Csnk1d	chr11	120844636	120852467	-0.33	-0.01
B3gntl1	chr11	121478331	121479112	-4.34	-0.46
Asxl2	chr12	3426883	3442534	-2.32	-0.27
Asxl2	chr12	3496396	3496756	-2.31	0.18
Asxl2	chr12	3496815	3497026	-0.44	0.05
Asxl2	chr12	3497115	3502505	-2.19	0.08
Smc6	chr12	11292342	11292462	-0.56	0.29
Smc6	chr12	11324583	11325004	-0.86	0.12
Mia2	chr12	60208874	60209969	-4.23	1.41
Lrr1	chr12	70275442	70275972	-0.48	-0.36
Mgat2	chr12	70285182	70287153	-2.50	0.08
Mgat2	chr12	70285144	70287166	-2.79	0.08
Hif1a	chr12	75008969	75009300	-10.10	-0.56
Hif1a	chr12	75043097	75043308	-29.20	-0.61
Fut8	chr12	78432957	78466016	-0.35	0.04
Fut8	chr12	78576163	78576974	-3.78	0.62
Ttll5	chr12	87165622	87165743	-0.28	-0.08
Ttll5	chr12	87259935	87260176	-8.05	-1.20
Psmc1	chr12	101348377	101351316	-1.50	-0.23
Calm1	chr12	101437750	101440606	-16.30	-0.45
Calm1	chr12	101445239	101445449	-3.24	-0.14

Otub2	chr12	104641553	104643038	-2.18	-0.30
Yy1	chr12	110031520	110044742	-20.20	-0.57
Yy1	chr12	110052912	110054425	-13.00	0.43
Dync1h1	chr12	111839634	111839815	-0.41	-0.05
Dync1h1	chr12	111855934	111857506	-0.43	-0.01
Dync1h1	chr12	111904592	111905154	-1.97	-0.13
Wdr20	chr12	112031349	112032696	-1.57	-0.15
Traf3	chr12	112498880	112500724	-1.28	0.14
Btbd6	chr12	114215520	114217031	-10.90	-0.80
Mta1	chr12	114374970	114375239	-4.02	-0.39
Ncoa1	chr12	4247600	4248409	-2.05	-0.34
Ncoa1	chr12	4248946	4249007	-0.88	-0.53
Ncoa1	chr12	4249095	4252804	-0.98	-0.20
Ncoa1	chr12	4267739	4267919	-1.13	-0.35
Ncoa1	chr12	4295010	4295788	-1.68	-0.24
Ncoa1	chr12	4295907	4296027	-0.69	-0.27
Nfkbia	chr12	56593395	56593604	-103.00	-1.36
Trappc6b	chr12	60162299	60162448	-2.15	-0.55
Sptb	chr12	77714000	77714211	-0.38	1.30
Rab15	chr12	77899425	77899575	-0.18	0.47
Pigh	chr12	80183182	80183512	-0.39	-0.08
Dcaf5	chr12	81437908	81438298	-0.31	-0.01
Dcaf5	chr12	81439342	81449328	-1.21	-0.09
Tmed10	chr12	86683962	86685208	-9.54	-0.24
Pomt2	chr12	88449791	88451284	-3.44	-0.46
Sel1l	chr12	93046846	93048492	-1.21	0.05
Sel1l	chr12	93080261	93087597	-2.29	-0.43
Atxn3	chr12	103157200	103157589	-0.54	-0.09
Ankrd9	chr12	112214580	112215239	-4.52	-1.58
B3galnt2	chr13	14090832	14091101	-6.32	-0.86
Trim27	chr13	21283975	21284396	-0.44	-0.02
Hist1h2bg	chr13	23663268	23663474	-1.13	-0.09
Hist1h2bc	chr13	23776067	23776213	-2.98	0.39
Hist1h2ab	chr13	23842956	23843254	-0.48	0.06
Gpld1	chr13	25078887	25082222	-4.74	-0.74
Ripk1	chr13	34094265	34094762	-9.02	-0.95
Ripk1	chr13	34119794	34125274	-0.32	0.00
Ripk1	chr13	34125393	34126082	-9.17	-0.86
Tmed9	chr13	55696944	55698672	-34.70	-0.59
Arsb	chr13	94710665	94710756	-0.36	-0.06
Ercc8	chr13	108948931	108950875	-0.31	-0.05
Gmds	chr13	31911574	31911785	-2.65	-0.65
Tubb2a	chr13	34166146	34168454	-10.90	0.67

Tubb2b	chr13	34219290	34219586	-1.55	1.29
Tubb2b	chr13	34219940	34221135	-0.88	0.43
Nup153	chr13	46776258	46776559	-2.23	0.27
Nup153	chr13	46782040	46792068	-1.16	0.05
Nup153	chr13	46822738	46823218	-7.57	-0.49
Uimc1	chr13	55129866	55132327	-0.91	0.20
Uimc1	chr13	55176930	55177378	-3.37	-0.43
Rab24	chr13	55421264	55421501	-3.81	-0.46
Rab24	chr13	55422955	55423341	-4.54	-0.47
Lman2	chr13	55447437	55447738	-0.36	0.02
Lman2	chr13	55448006	55448306	-6.45	-0.33
Hnrnpk	chr13	58501349	58504674	-3.06	-0.25
Golm1	chr13	59736356	59736893	-2.58	-0.39
Golm1	chr13	59738410	59741528	-2.06	-0.35
Vcan	chr13	89829747	89829807	-0.19	1.00
Vcan	chr13	89830646	89830706	-0.19	1.00
Kctd6	chr14	9052713	9055575	-1.94	-0.30
Atxn7	chr14	14928824	14936674	-0.35	0.01
Sec24c	chr14	21512428	21513175	-11.10	-0.51
Sec24c	chr14	21513593	21514072	-11.40	-0.48
Actr8	chr14	30804041	30804131	-3.81	-1.37
Bap1	chr14	32072009	32072607	-7.27	-0.52
Psmc6	chr14	45949498	45950514	-0.60	-0.09
Ktn1	chr14	48283451	48283962	-3.75	0.33
Dcaf11	chr14	56178865	56178955	-0.45	-0.26
Dcaf11	chr14	56188485	56188902	-0.70	-0.12
Trim13	chr14	62223590	62224752	-5.87	0.61
Kbtbd7	chr14	79827156	79828746	-2.42	0.38
Ankrd28	chr14	32514699	32518527	-2.62	-0.26
Dph3	chr14	32893956	32894136	-0.34	-0.09
Dph3	chr14	32896016	32898095	-0.33	0.01
Nedd8	chr14	56290685	56290743	-0.59	-0.14
Rabggta	chr14	56340520	56340807	-0.58	0.19
Cog3	chr14	76103115	76106953	-7.11	-0.50
Commd6	chr14	102033341	102039330	-1.33	-0.15
Fbxl3	chr14	103482090	103488631	-0.87	-0.07
Fbxl3	chr14	103491640	103498113	-0.65	0.09
Fbxl3	chr14	103498172	103498726	-0.67	-0.17
Sema5a	chr15	32616573	32620190	-0.36	0.02
Dcaf13	chr15	38944419	38944568	-1.48	-0.33
Rnf139	chr15	58730017	58731816	-1.36	-0.10
Commd5	chr15	76730370	76731727	-6.12	-0.48
Apol9b	chr15	77565748	77566812	-1.14	0.81

Tab1	chr15	79989230	79992132	-13.10	-0.63
Mgat3	chr15	80042031	80042242	-0.25	1.12
Rbx1	chr15	81305971	81306061	-1.28	-0.57
Ep300	chr15	81416643	81438767	-12.50	-0.29
Ep300	chr15	81479273	81481819	-5.01	-0.13
L3mbtl2	chr15	81516741	81518556	-3.63	-0.29
Nup50	chr15	84757805	84765822	-13.80	-0.39
Nup50	chr15	84770131	84771000	-9.31	-0.39
Nup50	chr15	84773033	84773303	-0.67	0.14
Tuba1c	chr15	98868116	98868326	-32.90	-0.97
Skp2	chr15	9043329	9043510	-0.95	0.41
Skp2	chr15	9069207	9070137	-7.83	-1.04
Otulin	chr15	27535675	27538550	-1.81	-0.14
Otulin	chr15	27553050	27560448	-1.52	-0.31
6-Mar	chr15	31388351	31388771	-2.84	-0.29
Rad21	chr15	51794834	51794954	-0.74	0.12
Rad21	chr15	51798216	51799452	-7.34	0.55
Derl1	chr15	57701535	57701686	-0.52	0.09
Derl1	chr15	57702583	57703422	-5.82	-0.18
Fbxo32	chr15	58011902	58012112	-0.40	-0.84
Fbxo32	chr15	58012410	58013039	-1.36	-1.07
Fbxo32	chr15	58046223	58046433	-0.22	-0.12
St3gal1	chr15	66936984	66938364	-4.76	-0.34
St3gal1	chr15	66944067	67005237	-3.10	-0.24
Apol9a	chr15	77234218	77235287	-0.50	-0.25
Apol10b	chr15	77415861	77416010	-0.29	1.46
Apol8	chr15	77579401	77583243	-8.81	2.69
Ddx17	chr15	79359532	79359623	-1.79	-0.30
Ddx17	chr15	79376930	79376991	-1.99	-0.41
Josd1	chr15	79506286	79506525	-1.24	-0.34
Josd1	chr15	79517170	79517647	-1.09	0.21
Josd1	chr15	79517914	79518183	-1.04	-0.32
Mkl1	chr15	80843459	80848650	-8.27	-0.28
Rangap1	chr15	81535516	81535666	-0.95	0.18
Rangap1	chr15	81560168	81560349	-4.41	-0.86
Pmm1	chr15	81781535	81781862	-2.41	-0.51
Ttll12	chr15	83406959	83407199	-3.52	-0.70
Alg12	chr15	88635941	88636391	-1.81	-0.30
Arsa	chr15	89303025	89303862	-0.98	-0.16
Arsa	chr15	89305988	89306615	-6.67	-0.79
Vdr	chr15	97687063	97687362	-0.53	1.58
Senp1	chr15	97872854	97873333	-1.22	-0.16
Asb8	chr15	97965969	97971744	-1.49	-0.17

Galnt6	chr15	100522228	100522617	-0.89	-0.21
Galnt6	chr15	100544605	100550892	-2.41	-0.23
Alg1	chr16	5244821	5245001	-1.38	-0.34
Klhl22	chr16	17771956	17777358	-0.41	-0.03
Psmd2	chr16	20651724	20652324	-20.60	-0.49
Senp2	chr16	22035915	22036036	-1.16	-0.87
Senp2	chr16	22046859	22047159	-0.36	0.02
St6gal1	chr16	23321224	23321703	-0.42	-0.35
Rnf168	chr16	32277606	32278356	-0.49	-0.08
Rnf168	chr16	32298678	32299428	-0.38	0.03
Golgb1	chr16	36898620	36898980	-0.86	-0.15
Golgb1	chr16	36907962	36914816	-3.50	0.16
Golgb1	chr16	36914905	36915146	-0.37	0.03
Golgb1	chr16	36915415	36916525	-5.29	0.32
Golgb1	chr16	36916673	36919800	-0.64	0.04
Golgb1	chr16	36932301	36933171	-5.73	-0.44
Tomm70a	chr16	57152724	57153054	-5.15	-0.28
Usp25	chr16	77115246	77116186	-1.62	-0.12
Usp16	chr16	87477080	87480005	-7.48	-0.34
Eef2kmt	chr16	5244426	5244486	-0.43	-0.18
Eef2kmt	chr16	5244813	5245351	-3.03	-0.38
Usp7	chr16	8738344	8757028	-0.78	-0.28
Senp5	chr16	31963352	31965869	-0.38	0.03
Senp5	chr16	31989077	31990517	-1.86	-0.12
Sec22a	chr16	35313658	35318950	-0.30	0.00
Nrip1	chr16	76291226	76295183	-5.84	0.27
Nrip1	chr16	76352759	76374042	-0.85	-0.46
App	chr16	85025115	85043833	-16.90	-0.31
App	chr16	85120907	85173892	-5.77	-0.46
Tulp4	chr17	6106833	6122253	-14.10	-0.52
Tulp4	chr17	6124111	6138054	-19.70	0.47
Tulp4	chr17	6138143	6139103	-10.10	0.29
Tulp4	chr17	6232220	6232851	-0.61	0.07
Tulp4	chr17	6232910	6233480	-0.97	0.22
Tulp4	chr17	6234109	6236560	-0.44	0.07
Spsb3	chr17	25023618	25029092	-0.66	-0.05
Axin1	chr17	26275810	26310709	-1.53	0.13
Axin1	chr17	26321127	26325117	-8.61	-0.59
Axin1	chr17	26330938	26332096	-0.71	0.13
Adamts10	chr17	33665942	33666123	-1.41	-0.95
Adamts10	chr17	33667169	33667439	-2.37	-1.68
Adamts10	chr17	33690397	33690697	-2.53	-1.03
Daxx	chr17	34047504	34050602	-124.00	-0.92

Psmb8	chr17	34335288	34335468	-2.22	-0.33
Neu1	chr17	35071556	35072840	-6.58	-0.28
Mdc1	chr17	35983321	35985735	-0.37	0.02
Mdc1	chr17	35987855	35988082	-1.14	0.38
Mdc1	chr17	35989429	35989938	-3.36	0.34
Mdc1	chr17	35994492	35994583	-1.22	0.38
Cul7	chr17	46788096	46788277	-2.58	3.86
Cul7	chr17	46788516	46788967	-0.83	-0.64
Cul7	chr17	46791019	46791200	-0.63	1.18
Rab5a	chr17	53618797	53623279	-2.09	-0.24
Rab5a	chr17	53639752	53646106	-0.93	0.08
Fem1a	chr17	56396215	56399474	-1.33	-0.07
Fem1a	chr17	56400758	56401238	-0.56	-0.12
Fem1a	chr17	56401745	56402642	-3.47	-0.51
Safb	chr17	56733308	56735158	-0.34	-0.01
Safb	chr17	56738105	56738706	-1.43	-0.14
Man2a1	chr17	64950988	64974682	-0.45	0.05
Socs5	chr17	87507227	87507318	-0.46	-0.23
Socs5	chr17	87520106	87534863	-0.79	0.07
Ccnf	chr17	24360594	24360954	-5.87	0.78
Ccnf	chr17	24361042	24361933	-12.00	1.09
Ube2i	chr17	25398055	25398776	-6.49	-0.64
Rab40c	chr17	26020195	26020794	-2.20	-0.30
Pigq	chr17	26064506	26064597	-0.37	-0.03
Pigq	chr17	26071113	26071234	-3.26	1.37
Pigq	chr17	26073535	26073656	-2.01	1.11
Pigq	chr17	26073775	26078847	-1.69	0.17
Rab11b	chr17	33883909	33884089	-4.70	-0.48
Rab11b	chr17	33884327	33884477	-0.75	-0.25
Ring1	chr17	34158095	34160251	-0.84	-0.10
Ly6g6d	chr17	35208436	35208609	-0.20	-0.07
Cul9	chr17	46680214	46680695	-2.00	-0.64
Tubb4a	chr17	57220052	57220528	-1.24	-0.37
Tubb4a	chr17	57220616	57221122	-1.23	-0.35
Rab31	chr17	66001065	66001632	-0.68	-0.05
Rab31	chr17	66002078	66002287	-2.81	-0.26
Rab31	chr17	66003418	66017257	-2.20	0.14
Rab31	chr17	66122031	66122092	-0.25	-0.17
Rab12	chr17	66844241	66844961	-2.21	-0.20
Pigf	chr17	87396598	87396896	-1.18	0.11
Mcfd2	chr17	87654470	87654591	-0.51	0.10
Mcfd2	chr17	87654769	87656548	-2.71	-0.18
Calm2	chr17	87835011	87846216	-2.03	-0.14

Fbxo11	chr17	88390945	88391686	-0.95	0.09
Fbxo11	chr17	88464385	88464625	-1.02	-0.30
Wac	chr18	7869438	7869558	-1.86	-0.56
Wac	chr18	7905960	7910820	-1.20	0.09
Wac	chr18	7921952	7926308	-5.51	0.73
Galnt1	chr18	24438515	24443824	-0.96	-0.11
Apc	chr18	34458082	34458262	-0.62	-0.18
Apc	chr18	34470478	34472953	-2.03	0.18
Apc	chr18	34473850	34476007	-2.30	0.15
Apc	chr18	34476395	34478342	-2.44	-0.16
Ube2d2a	chr18	35931242	35931392	-3.18	-0.65
Ube2d2a	chr18	35965479	35965570	-0.43	0.27
Commd10	chr18	47246724	47246874	-0.45	0.07
Dctn4	chr18	60686499	60686560	-0.25	-0.32
Dctn4	chr18	60716315	60716526	-1.59	0.32
Napg	chr18	63154672	63156813	-3.03	-0.31
Tubb6	chr18	67550384	67552360	-1.76	-0.20
Tubb6	chr18	67552568	67561747	-29.40	-0.45
Mbd1	chr18	74437069	74438854	-3.83	-0.42
Mbd1	chr18	74439959	74440737	-1.71	-0.23
Mbd1	chr18	74440886	74442159	-0.82	-0.10
Smad7	chr18	75527018	75527675	-0.56	0.13
Smad7	chr18	75528299	75554128	-4.14	0.55
Smad2	chr18	76462139	76464727	-1.34	-0.19
Pias2	chr18	77304126	77304476	-0.83	0.35
Pias2	chr18	77304625	77336416	-0.31	0.00
Pias2	chr18	77388818	77391933	-0.88	0.13
Usp14	chr18	10030056	10030117	-0.99	-0.34
B4galt6	chr18	20846332	20847761	-0.59	-0.09
Ino80c	chr18	24263948	24264068	-1.28	-0.40
Ino80c	chr18	24264545	24265143	-0.55	0.07
Nr3c1	chr18	39588392	39650238	-0.66	-0.04
Fem1c	chr18	46664557	46685565	-0.38	0.01
Tmed7	chr18	46747763	46748242	-1.09	-0.08
Adrb2	chr18	62337558	62339256	-44.30	0.73
Lman1	chr18	66140572	66140992	-1.11	-0.12
Lman1	chr18	66162108	66162289	-2.28	-0.31
Smad4	chr18	73799858	73800008	-0.35	-0.03
Smad4	chr18	73837391	73837750	-0.38	-0.03
Smad4	chr18	73863156	73863395	-1.93	-1.18
Socs6	chr18	89038427	89056448	-0.73	0.07
Sptbn2	chr19	4744054	4745130	-1.41	-0.53
B4gat1	chr19	5039183	5040891	-7.61	-0.49

B4gat1	chr19	5041278	5046482	-6.38	-0.41
B4gat1	chr19	5049005	5049917	-6.80	-0.44
Rela	chr19	5646968	5647773	-3.54	-0.20
Syvn1	chr19	6052640	6053718	-8.86	-0.27
Men1	chr19	6334978	6336781	-0.48	-0.04
Men1	chr19	6338873	6340864	-2.26	-0.13
Stx5a	chr19	8829416	8830132	-0.89	-0.09
Ubxn1	chr19	8946015	8946196	-2.26	-0.40
Uhrf2	chr19	30105092	30105153	-1.10	-0.51
Uhrf2	chr19	30113733	30131000	-0.49	-0.06
Pten	chr19	32832126	32832995	-2.07	-0.13
Pten	chr19	32872591	32874607	-0.82	0.07
Pten	chr19	32892519	32894752	-15.20	0.55
Stambpl1	chr19	34266759	34267030	-0.53	0.08
Stambpl1	chr19	34307271	34308607	-1.79	-0.39
Btrc	chr19	45604117	45604298	-0.65	1.17
Gbf1	chr19	46227047	46227228	-1.43	-0.39
Nfkb2	chr19	46379436	46380290	-0.39	0.13
Fbxl15	chr19	46403649	46404936	-3.45	-0.46
Ppp6r3	chr19	3454927	3455647	-21.70	-0.68
Ppp6r3	chr19	3456876	3459395	-1.96	-0.25
Rce1	chr19	4622816	4623024	-4.13	-0.70
1700020D05Rik	chr19	5502858	5503787	-6.87	0.99
Incenp	chr19	9947175	9947235	-0.82	-0.16
Incenp	chr19	9949354	9951151	-0.99	-0.15
Incenp	chr19	9957682	9968481	-0.67	-0.03
Incenp	chr19	9973932	9974023	-0.51	0.26
Tmem132a	chr19	10932318	10936113	-125.00	-1.30
Gcnt1	chr19	17403142	17407678	-1.38	0.13
Fbxw4	chr19	45652866	45653256	-0.75	-0.11
Actr1a	chr19	46452346	46452913	-2.60	-0.15
Usp9x	chrX	12733152	12733872	-13.20	0.35
Ube2a	chrX	34414360	34414420	-1.18	-0.44
Foxo4	chrX	98449866	98450612	-10.40	-0.99
Ogt	chrX	98874610	98878315	-1.22	-0.06
Ogt	chrX	98878792	98878973	-1.73	-0.32
Piga	chrX	160857718	160861207	-2.61	0.43
Piga	chrX	160866881	160869822	-1.27	0.39
Piga	chrX	160870327	160870477	-1.00	0.56
C1galt1c1	chrX	35984195	35988290	-0.70	0.07
Hcfc1	chrX	71189242	71189693	-2.65	0.19
Hcfc1	chrX	71194710	71196030	-1.82	-0.08
Hcfc1	chrX	71211533	71211654	-0.42	0.04

Chm	chrX	110156917	110157037	-0.85	0.35
Rab9	chrX	162895660	162900249	-8.56	0.50
Vcpip1	chr1	9714443	9738463	-10.40	-0.15
Napb	chr2	148538465	148558156	-0.50	-0.19
Uba6	chr5	86541279	86547113	-0.45	0.04
Tnks	chr8	36028534	36028744	-3.00	-0.65
Dynll2	chr11	87794645	87799923	-0.59	0.05
Dynll2	chr11	87800072	87801030	-0.39	-0.04

Adaptive immune system

gene_name	chr	chromStart	chromEnd	diff.lg.p	diff.log2.fc
Cd28	chr1	60826604	60826815	-0.22	-0.36
Cd28	chr1	60827743	60827984	-0.68	-1.75
Inpp5d	chr1	89516916	89517096	-1.75	-0.39
Inpp5d	chr1	89614406	89616844	-1.50	-0.10
Ube2f	chr1	93182063	93182602	-2.18	-0.17
Asb1	chr1	93437321	93437716	-0.36	-0.19
Asb1	chr1	93440810	93443681	-2.13	-0.63
Asb1	chr1	93451344	93451764	-1.52	0.40
Ncf2	chr1	154654984	154658055	-3.20	-0.20
Hecw2	chr1	53869614	53883756	-0.93	1.33
Hecw2	chr1	53982405	53983513	-0.56	-0.34
Rnf25	chr1	74640325	74640975	-9.13	-0.51
Tuba4a	chr1	75211607	75212025	-1.98	0.61
Tuba4a	chr1	75212113	75215828	-13.90	-0.27
Ap1s3	chr1	79668486	79668547	-0.38	-0.21
Cul3	chr1	80263601	80265851	-6.24	0.34
Cul3	chr1	80336915	80337005	-0.92	-0.29
Trip12	chr1	84721087	84721998	-0.90	-0.07
Trip12	chr1	84782695	84836815	-20.50	-0.26
Pdcd1	chr1	95935089	95937353	-2.58	0.66
Pdcd1	chr1	95938082	95949130	-1.40	-2.60
Klhl20	chr1	163019017	163019228	-1.31	-0.43
Klhl20	chr1	163020426	163023835	-2.61	0.52
Klhl20	chr1	163039357	163039897	-3.74	-0.46
Slamf7	chr1	173562982	173563102	-0.59	3.58
Slamf7	chr1	173563191	173563341	-0.79	4.39
Slamf7	chr1	173563669	173563999	-0.98	5.13
Slamf7	chr1	173564746	173565046	-0.31	0.94
Ppp2r5a	chr1	193176577	193176847	-2.61	0.23
Ppp2r5a	chr1	193196363	193220920	-4.26	-0.27
Anapc2	chr2	25128463	25128911	-3.00	-0.30
Fbxw5	chr2	25358984	25359776	-3.57	-0.40
Fbxw5	chr2	25360013	25360422	-1.07	-0.24

Mapkap1	chr2	34479093	34479423	-0.34	0.02
Hspa5	chr2	34629658	34629748	-39.30	-0.48
Hspa5	chr2	34630750	34631960	-27.90	-0.12
Klhl41	chr2	69508683	69508923	-1.52	0.86
Dync1i2	chr2	71049792	71050012	-0.90	0.33
Rapgef4	chr2	72091018	72094782	-0.34	-0.96
Itga4	chr2	79165818	79168203	-23.70	0.20
Itgav	chr2	83564583	83564973	-10.50	-1.02
Itgav	chr2	83643447	83643718	-3.67	-0.49
Itgav	chr2	83643807	83643987	-0.74	-0.16
Psmc3	chr2	90894439	90895182	-3.33	-0.20
Traf6	chr2	101530344	101537929	-2.95	-0.21
Kif18a	chr2	109173491	109173672	-0.51	0.32
Pdia3	chr2	121263353	121263680	-0.63	0.03
B2m	chr2	121973422	121973632	-11.70	-0.24
Trim69	chr2	122004226	122004644	-0.30	-0.80
Kif3b	chr2	153141983	153143391	-0.42	0.03
Kif3b	chr2	153155890	153156669	-4.11	0.44
Kif3b	chr2	153158285	153158915	-2.59	0.43
Itch	chr2	154959216	154959307	-0.27	-0.02
Itch	chr2	155050102	155050403	-0.31	-0.01
Src	chr2	157295136	157296531	-2.35	0.70
Plcg1	chr2	160587552	160588233	-9.17	-1.31
Ywhab	chr2	163820932	163837497	-10.90	-0.39
Ctsa	chr2	164659257	164659646	-12.00	-0.34
Cd40	chr2	164896268	164897006	-0.30	-0.03
Rnf114	chr2	167318622	167318832	-1.07	-1.19
Tubb1	chr2	174282485	174283323	-0.40	0.53
Tubb4b	chr2	25077825	25079655	-27.50	-0.32
Tubb4b	chr2	25079713	25080192	-0.32	0.02
Asb6	chr2	30678647	30680212	-9.57	-0.39
Lrsam1	chr2	32781993	32782383	-0.31	-0.02
Fbxw2	chr2	34661048	34661869	-4.78	-0.39
Fbxw2	chr2	34662764	34664040	-0.64	-0.16
Fbxw2	chr2	34668436	34678672	-6.10	-0.35
Fbxw2	chr2	34681468	34681725	-0.46	0.13
Psmd5	chr2	34707993	34709823	-1.65	-0.19
Psmd5	chr2	34726274	34726482	-4.41	-0.82
Psmb7	chr2	38498946	38499426	-3.13	-0.36
Ptprj	chr2	90273053	90274190	-33.00	-0.77
Ptprj	chr2	90420444	90420774	-1.91	-0.36
Anapc1	chr2	128436507	128438637	-1.24	-0.11
Ubox5	chr2	130416996	130417566	-1.55	-0.37

Ubox5	chr2	130425355	130426405	-1.35	-0.24
Siglec1	chr2	130895015	130895584	-2.61	0.78
Siglec1	chr2	130900602	130901900	-1.04	-0.31
Siglec1	chr2	130903954	130904467	-0.76	-0.28
Siglec1	chr2	130907034	130909080	-1.77	-0.56
Siglec1	chr2	130911312	130911373	-0.89	-1.09
Siglec1	chr2	130911872	130912501	-2.29	0.94
Psmf1	chr2	151543111	151543202	-1.31	-0.87
Psmf1	chr2	151543649	151544038	-0.30	0.01
Trib3	chr2	152163160	152164409	-25.70	-0.34
Trib3	chr2	152169058	152169796	-0.86	-0.16
Nfatc2	chr2	168301969	168303229	-1.59	-0.26
Nfatc2	chr2	168304128	168330396	-9.73	-0.53
Nfatc2	chr2	168396287	168427005	-6.54	-0.50
Pik3ca	chr3	32335072	32335342	-3.17	-0.61
Pik3ca	chr3	32360651	32362022	-2.40	0.26
Ctso	chr3	81756174	81758885	-0.59	0.09
Fbxw7	chr3	84619498	84694070	-0.58	-0.12
Fbxw7	chr3	84694129	84756226	-0.51	0.05
Fbxw7	chr3	84780110	84781955	-0.35	0.02
Ube2q1	chr3	89577530	89577890	-0.54	-0.06
Ube2q1	chr3	89585705	89587919	-0.80	-0.04
Them4	chr3	94135467	94135946	-0.33	0.03
Ctss	chr3	95330707	95333423	-10.90	-0.35
Rnf115	chr3	96592580	96594008	-0.38	-0.02
Sec22b	chr3	97705149	97711158	-2.16	-0.33
Sec22b	chr3	97725110	97725468	-2.27	-0.20
Ptpn22	chr3	103664257	103664347	-5.38	-0.77
Ptpn22	chr3	103685999	103690208	-24.30	-0.48
Psma5	chr3	108059873	108060022	-0.64	3.39
Cenpe	chr3	134902767	134902887	-0.61	0.20
Cenpe	chr3	134905081	134905534	-0.42	-0.07
Cenpe	chr3	134910353	134910534	-1.68	-0.55
Cenpe	chr3	134936264	134936504	-0.50	-0.15
Ube2d3	chr3	135101656	135102228	-19.60	-0.75
Bcl10	chr3	145587232	145587592	-4.99	-0.42
Bcl10	chr3	145588099	145593556	-3.89	-0.41
Pag1	chr3	9690420	9691051	-2.03	-0.38
Pag1	chr3	9691830	9704750	-16.60	-0.50
Siah2	chr3	58479649	58495381	-3.84	-0.27
Siah2	chr3	58495620	58496010	-0.94	0.28
Psmb4	chr3	94690701	94690880	-8.50	-0.41
Psmd4	chr3	94836626	94836894	-2.54	-0.16

Psmd4	chr3	94840526	94846467	-8.21	-0.35
Fcgr1	chr3	96087130	96087191	-0.26	0.03
Fcgr1	chr3	96087728	96088506	-8.72	-0.57
Cd101	chr3	100797451	100797662	-0.59	-2.75
Sec24b	chr3	129686102	129687101	-1.82	-0.14
Sec24b	chr3	129743865	129763735	-15.50	-0.56
Nfkb1	chr3	135248247	135252299	-9.58	-0.45
Nfkb1	chr3	135354240	135354451	-1.35	0.77
Dapp1	chr3	137595557	137598550	-2.97	-0.66
Prkacb	chr3	146394694	146395023	-0.79	0.13
Lyn	chr4	3605267	3605506	-12.00	-0.40
Lyn	chr4	3716941	3718014	-40.00	-0.36
Fbxl4	chr4	22303713	22313289	-1.60	-0.28
Map3k7	chr4	32051081	32051232	-3.02	-1.01
Map3k7	chr4	32104109	32107267	-5.97	0.38
Map3k7	chr4	32110051	32110231	-0.27	0.00
Ube2r2	chr4	41137807	41138136	-0.41	-0.03
Trim32	chr4	65274645	65274706	-0.48	-0.20
Trim32	chr4	65274854	65276169	-0.55	-0.05
Lrrc41	chr4	115761466	115762183	-23.20	-0.55
Lrrc41	chr4	115768961	115769201	-3.73	-0.33
Pik3r3	chr4	115945443	115972695	-28.90	3.76
Pik3r3	chr4	115975001	115975152	-1.61	1.80
Rnf19b	chr4	128735514	128735993	-17.90	-1.08
Rnf19b	chr4	128759998	128761505	-99.00	-1.37
Capzb	chr4	138748845	138794826	-3.32	-0.32
Mtor	chr4	147822690	147822781	-3.13	-1.30
Mtor	chr4	147930392	147931764	-3.53	-0.34
Klhl21	chr4	151383209	151383749	-20.10	-1.89
Klhl21	chr4	151383868	151389959	-10.20	-0.52
Klhl21	chr4	151390257	151390438	-3.61	1.07
Ube2j2	chr4	155330537	155331768	-9.18	-0.43
Ripk2	chr4	16050670	16051621	-4.17	-0.41
Ripk2	chr4	16090436	16090645	-16.00	-1.44
Ufl1	chr4	25177588	25177709	-0.30	-0.02
Fbxo10	chr4	45048045	45048374	-1.12	1.75
Fbxo10	chr4	45048642	45048793	-0.22	0.14
Fbxo10	chr4	45071323	45071981	-0.27	-0.03
Cdc26	chr4	62055639	62055998	-0.37	-0.07
Klhl9	chr4	88366049	88367814	-9.57	0.24
Kif2c	chr4	116832539	116832928	-0.28	0.02
Rnf220	chr4	116961136	116968779	-2.43	-0.37
Cdc20	chr4	118109887	118109948	-0.76	-0.68

Lck	chr4	129225797	129226157	-0.39	0.06
Lck	chr4	129234249	129234661	-0.99	2.21
Pik3cd	chr4	149023575	149025880	-7.02	-0.36
Spsb1	chr4	149271916	149281307	-0.79	-0.50
Mib2	chr4	155028580	155028821	-0.32	-0.05
Ube3c	chr5	29895781	29895962	-0.89	-0.61
Ube3c	chr5	30001057	30001238	-1.45	-0.31
Rnf4	chr5	34694045	34694196	-2.72	-0.31
Anapc4	chr5	53257704	53257973	-1.23	-0.22
Klhl5	chr5	65558211	65558721	-0.75	-0.12
Ube3b	chr5	114830704	114830795	-0.26	0.26
Ube3b	chr5	114869593	114869952	-0.90	0.14
Fbxo21	chr5	118452063	118458618	-6.36	-0.76
Anapc7	chr5	122891328	122891538	-0.73	0.15
Anapc7	chr5	122893905	122894532	-19.20	-0.92
Rnf34	chr5	123273869	123314504	-0.81	0.09
Psmd9	chr5	123678228	123678437	-0.34	0.03
Kctd7	chr5	130628217	130628785	-0.53	-0.24
Fbxl5	chr5	44142150	44149098	-2.88	-0.56
Fbxl5	chr5	44149396	44151973	-0.84	0.08
Rchy1	chr5	92391825	92392094	-0.33	0.04
Sec31a	chr5	100790907	100791058	-0.72	-0.09
Fbxw8	chr5	118515497	118515797	-0.28	0.02
Fbxw8	chr5	118605107	118605467	-13.80	-1.01
Ptpn11	chr5	121581827	121582307	-1.21	0.16
Ptpn11	chr5	121583292	121587519	-0.40	0.02
Anapc5	chr5	123271138	123271348	-2.63	-0.39
Ubc	chr5	125866453	125867671	-172.00	0.73
Ubc	chr5	125867938	125868147	-3.15	0.20
Ubc	chr5	125868235	125870298	-22.80	-0.40
Ncf1	chr5	134697302	134697663	-2.36	-0.12
Pilra	chr5	138263299	138263719	-6.34	2.30
Pilra	chr5	138264706	138265015	-0.36	0.18
Pilra	chr5	138276577	138276877	-2.44	1.44
Card11	chr5	141348952	141352360	-0.69	0.26
Fbxl18	chr5	143633766	143634006	-6.05	-1.06
Fbxl18	chr5	143647153	143648949	-184.00	-2.23
Rac1	chr5	144288681	144288861	-1.21	-0.22
Smurf1	chr5	145637663	145638682	-0.50	0.08
Smurf1	chr5	145639310	145639521	-0.66	0.35
Rnf6	chr5	147021793	147022181	-6.60	0.51
Rnf6	chr5	147023075	147031998	-2.47	0.16
Cul1	chr6	47404345	47445165	-3.56	0.31

Cul1	chr6	47474617	47476108	-1.64	-0.15
Herc6	chr6	57445639	57531152	-0.66	-0.46
Dctn1	chr6	83140226	83141067	-0.45	0.03
Kbtbd8	chr6	95071991	95077327	-2.49	0.37
Vhl	chr6	113578147	113579978	-0.46	-0.06
Fbxl14	chr6	119429685	119431451	-0.36	-0.01
Spsb2	chr6	124758812	124760455	-90.80	-1.86
Pianp	chr6	124949362	124950749	-5.78	-0.31
Klhl42	chr6	147040014	147050109	-0.97	-0.28
Klhl42	chr6	147050168	147056940	-3.38	-0.59
Ube2h	chr6	30163827	30164395	-1.91	0.24
Ube2h	chr6	30164783	30164933	-0.40	-0.15
Vamp8	chr6	72335214	72335448	-3.83	-0.35
Vamp8	chr6	72338374	72340603	-0.85	-0.14
Uba3	chr6	97134556	97134676	-1.20	0.47
Raf1	chr6	115568620	115569280	-0.58	0.06
Ptpn6	chr6	124683045	124688519	-0.41	-0.07
Kras	chr6	145168813	145168964	-1.16	-0.35
Itpr2	chr6	146060001	146061041	-8.07	0.54
Ap2s1	chr7	17323792	17328591	-2.88	-0.37
Akt2	chr7	28422173	28423398	-0.50	-0.06
Herc2	chr7	63485072	63486928	-1.61	0.14
Ube3a	chr7	66484418	66484628	-2.76	-0.49
Ube3a	chr7	66531169	66540150	-0.51	-0.02
Klhl25	chr7	82993313	83012167	-0.30	0.00
Klhl25	chr7	83012375	83018269	-2.48	-0.60
Klhl25	chr7	83018657	83018926	-0.33	0.09
Ctsc	chr7	95437245	95437485	-0.96	0.45
Ctsc	chr7	95458037	95458547	-0.94	0.12
Dctn5	chr7	129276554	129277689	-0.48	-0.08
Dctn5	chr7	129291791	129292440	-0.33	-0.01
Rbbp6	chr7	130114077	130114468	-5.64	-0.37
Rbbp6	chr7	130133635	130133876	-1.64	0.20
Rbbp6	chr7	130140555	130140795	-1.26	0.15
Rbbp6	chr7	130142061	130145876	-2.57	0.06
Fbxl19	chr7	134890288	134891744	-4.33	-0.90
Fbxl19	chr7	134891803	134891893	-0.66	-0.35
Fbxl19	chr7	134893270	134904820	-11.30	-0.67
Fbxl19	chr7	134906111	134911817	-1.15	-0.18
Fbxl19	chr7	134911876	134912442	-2.21	-0.45
Stx4a	chr7	134985321	134985978	-1.03	-0.12
Lair1	chr7	3960550	3960699	-0.30	0.16
Lair1	chr7	3961056	3961295	-0.66	-0.33

Ube2m	chr7	13623018	13623619	-6.02	-0.45
Calm3	chr7	17502039	17502309	-1.46	-0.09
Klc3	chr7	19979788	19981213	-2.01	-0.31
Nectin2	chr7	20302501	20302950	-0.50	0.24
Nectin2	chr7	20306842	20310098	-3.95	-0.76
Nectin2	chr7	20334652	20334862	-0.34	-0.09
Pvr	chr7	20490268	20494550	-0.33	0.02
Pvr	chr7	20502401	20506492	-38.60	-0.97
Nfkbib	chr7	29546684	29546864	-25.70	-0.94
Nfkbib	chr7	29547132	29551543	-9.87	-0.61
Psmd8	chr7	29965393	29965573	-13.80	-0.43
Cd22	chr7	31662494	31662794	-1.10	2.82
Siglece	chr7	50906826	50906947	-0.53	0.45
Siglece	chr7	50914669	50914730	-0.37	-0.51
Asb7	chr7	73792534	73823980	-3.67	-0.42
Asb7	chr7	73834207	73834387	-0.58	0.31
Det1	chr7	85972688	85972809	-1.10	-0.51
Det1	chr7	85988146	85988866	-0.56	-0.07
Trim21	chr7	109707151	109710434	-1.56	0.22
Kif22	chr7	134179351	134185934	-2.04	-0.85
Ifitm2	chr7	148141799	148141860	-2.49	-0.23
Ifitm3	chr7	148196466	148196643	-44.70	-0.36
Hras	chr7	148376788	148378177	-0.34	-0.01
Hras	chr7	148378775	148379903	-0.71	0.21
Ctsd	chr7	149561820	149562294	-118.00	-0.34
Cdc16	chr8	13779263	13781763	-4.99	-0.38
Ppp2cb	chr8	34710092	34710359	-0.73	-0.37
Sh3rf1	chr8	63704632	63808042	-0.38	0.08
Sh3rf1	chr8	63863616	63863767	-0.29	-0.10
Sh3rf1	chr8	63872471	63873071	-0.74	-0.31
Prkaca	chr8	86496876	86500855	-1.59	-0.45
Fbxl8	chr8	107790925	107791573	-0.65	0.30
Fbxl8	chr8	107791922	107793108	-0.55	0.10
Nfatc3	chr8	108602601	108603618	-3.27	0.21
Nfatc3	chr8	108632435	108652196	-8.48	0.41
Ap1g1	chr8	112382745	112384686	-3.66	-0.19
Znrf1	chr8	114060539	114062275	-0.69	0.05
Znrf1	chr8	114143127	114147028	-1.41	-0.07
Gan	chr8	119728191	119729086	-7.70	0.65
Plcg2	chr8	120022190	120028105	-0.35	-0.03
Tubb3	chr8	125942898	125945049	-0.53	-0.56
Itgb1	chr8	131209643	131209853	-0.68	-0.08
Itgb1	chr8	131256163	131256313	-1.27	0.17

Ikbkb	chr8	23770606	23771027	-1.32	-0.20
Dctn6	chr8	35169435	35171565	-0.29	-0.02
Lonrf1	chr8	37280167	37282962	-0.30	-0.01
Lonrf1	chr8	37311699	37312090	-2.20	-1.14
Klhl2	chr8	67219391	67219870	-0.73	-0.15
Pik3r2	chr8	73292079	73292528	-7.07	-0.81
Pik3r2	chr8	73296811	73300581	-13.40	-0.87
Calr	chr8	87366315	87366863	-41.40	-0.26
Calr	chr8	87370234	87370800	-11.20	-0.26
Siah1a	chr8	89248786	89269816	-0.36	-0.02
Dync1li2	chr8	106944120	106944421	-2.39	-0.28
Dync1li2	chr8	106966646	106966737	-0.40	0.11
Psmb10	chr8	108461631	108461967	-2.80	-0.63
Psmd7	chr8	110104338	110108077	-4.03	0.12
Fbxo31	chr8	124075878	124076147	-0.29	0.02
Icam1	chr9	20820403	20823430	-2.14	-0.45
Icam1	chr9	20823638	20823729	-0.38	-0.07
Icam1	chr9	20824146	20824566	-0.50	0.12
Icam1	chr9	20831708	20833091	-2.44	-0.10
Icam5	chr9	20841007	20841187	-0.45	-0.34
Dnm2	chr9	21310134	21311379	-2.43	0.16
Dnm2	chr9	21311898	21312168	-2.03	0.27
Ppp2r1b	chr9	50687990	50688710	-0.71	-0.09
Ppp2r1b	chr9	50701016	50701675	-4.03	-0.60
Fbxo22	chr9	55066270	55071672	-1.24	0.12
Fbxo22	chr9	55071791	55072031	-0.53	0.10
Herc1	chr9	66198256	66220501	-4.31	0.30
Herc1	chr9	66293942	66296296	-2.94	0.33
Herc1	chr9	66298665	66298935	-1.74	0.27
Anapc13	chr9	102528625	102532216	-1.16	-0.23
Dcaf1	chr9	106738991	106740882	-3.19	0.55
Dcaf1	chr9	106760577	106761417	-1.12	0.15
Dcaf1	chr9	106776469	106782364	-1.52	0.38
Dync1li1	chr9	114632686	114632895	-0.93	0.16
Dync1li1	chr9	114632812	114633189	-1.24	0.13
Fbxl12	chr9	20448880	20449151	-0.85	-0.48
Keap1	chr9	21034473	21038380	-0.55	0.03
Keap1	chr9	21041615	21042276	-1.55	-0.22
Keap1	chr9	21043205	21043686	-0.40	0.13
Crtam	chr9	40781146	40781620	-0.44	0.47
Ube4a	chr9	44731508	44732078	-3.85	0.39
Cul5	chr9	53425530	53429279	-5.02	0.34
Csk	chr9	57474661	57475560	-6.84	-0.21

Rnf111	chr9	70274555	70277390	-0.83	-0.10
Rnf111	chr9	70323650	70350792	-12.70	0.37
Pik3cb	chr9	98939628	98941447	-5.88	0.37
Nck1	chr9	100395659	100407191	-0.86	0.08
Uba5	chr9	103949273	103951621	-4.43	0.27
Rnf123	chr9	107954002	107954243	-6.46	0.71
Arih2	chr9	108506380	108506471	-9.95	5.42
Arih2	chr9	108546363	108547287	-1.88	-0.31
Trim71	chr9	114422031	114422092	-0.38	2.58
Trim71	chr9	114422299	114422658	-0.97	5.09
Fbxo30	chr10	11001127	11015345	-10.80	0.40
Fyn	chr10	39089604	39089994	-0.68	-0.12
Fyn	chr10	39271197	39284102	-0.31	0.00
Herc4	chr10	62706723	62726819	-16.00	-0.60
Itgb2	chr10	76993092	77004744	-8.05	-0.66
Ube2g2	chr10	77107280	77107637	-1.33	-0.14
Ube2g2	chr10	77107992	77108082	-0.37	-0.04
Icosl	chr10	77539904	77541941	-1.63	0.31
Cdc34	chr10	79144939	79145322	-5.03	-0.46
Cdc34	chr10	79148043	79151143	-18.90	-0.38
Rnf41	chr10	127874437	127875829	-14.90	-0.48
Tab2	chr10	7625983	7626463	-1.79	0.18
Tab2	chr10	7626701	7630910	-3.35	-0.17
Tab2	chr10	7631204	7644574	-1.97	-0.07
Rnf217	chr10	31328190	31329531	-5.20	-0.76
Ube2d1	chr10	70718144	70718592	-2.63	0.38
Rnf126	chr10	79221259	79223613	-5.73	-0.22
Rnf126	chr10	79229508	79229686	-0.71	-0.18
Fzr1	chr10	80829682	80830217	-4.88	0.37
Kif5a	chr10	126700029	126700180	-1.42	-0.60
Ap1b1	chr11	4941834	4942343	-0.86	-0.12
Psme4	chr11	30671774	30672135	-0.54	-0.18
Fbxw11	chr11	32644536	32644986	-0.78	-0.08
Rnf130	chr11	49933551	49933881	-0.38	0.41
Ppp2ca	chr11	51912325	51912474	-4.51	-0.88
Ppp2ca	chr11	51935449	51935836	-0.34	0.00
Kif3a	chr11	53380880	53380941	-0.99	-0.79
Trim11	chr11	58791617	58791916	-2.16	-0.42
Trim11	chr11	58802336	58802515	-0.47	-0.11
Trim11	chr11	58802902	58804960	-1.42	-0.11
Ube2g1	chr11	72420792	72421032	-3.90	-0.86
Ube2g1	chr11	72493612	72497295	-0.33	0.03
Blmh	chr11	76799996	76800265	-6.75	-0.58

Col1a1	chr11	94812810	94813020	-0.54	2.56
Psmd3	chr11	98543867	98546829	-30.30	-0.74
Psme3	chr11	101177564	101178336	-0.62	0.07
Psme3	chr11	101182070	101183288	-0.64	-0.06
Rel	chr11	23642262	23644451	-4.65	-0.27
Rel	chr11	23661118	23670970	-10.60	-0.81
Trim41	chr11	48620443	48622633	-2.33	-0.15
Trim41	chr11	48629367	48630893	-3.55	-0.21
Canx	chr11	50109707	50110776	-30.20	-0.37
Sec24a	chr11	51556985	51570140	-6.97	-0.33
Wsb1	chr11	79053599	79053839	-11.70	-0.39
Ube2z	chr11	95911494	95911704	-1.74	-0.23
Fbxl20	chr11	97950510	97953462	-0.74	0.16
Klhl11	chr11	100323925	100325737	-6.64	0.31
Klhl11	chr11	100333798	100334096	-2.00	0.50
Map3k14	chr11	103081077	103082247	-3.53	-0.47
Map3k14	chr11	103100346	103101055	-0.68	-0.14
Map3k14	chr11	103103466	103128715	-0.39	-0.09
Cdc27	chr11	104366175	104368590	-6.73	0.30
Cd79b	chr11	106172684	106173355	-0.96	2.28
Icam2	chr11	106239145	106239263	-0.38	0.20
Smurf2	chr11	106683743	106684388	-0.37	-0.04
Smurf2	chr11	106781609	106781789	-3.23	-1.30
Cd300lb	chr11	114784364	114784935	-3.81	-0.24
Cd300lb	chr11	114784994	114795700	-23.20	-0.42
Cd300e	chr11	114914339	114914460	-0.23	0.05
Grb2	chr11	115505517	115505906	-1.13	0.17
Grb2	chr11	115506591	115507248	-1.12	-0.07
Ube2o	chr11	116399633	116399872	-0.36	0.05
Ube2o	chr11	116400110	116404120	-7.85	-0.32
Ube2o	chr11	116405875	116406055	-1.26	0.30
Ube2o	chr11	116442641	116442761	-0.21	0.26
Socs3	chr11	117827669	117830291	-196.00	-0.95
Kif3c	chr12	3365310	3367524	-6.08	-0.42
Kif3c	chr12	3403293	3403444	-1.82	-0.55
Kif3c	chr12	3404400	3404580	-3.67	0.79
Kif3c	chr12	3405446	3405956	-3.69	0.36
Lrr1	chr12	70275442	70275972	-0.48	-0.36
Psmc1	chr12	101348377	101351316	-1.50	-0.23
Calm1	chr12	101437750	101440606	-16.30	-0.45
Calm1	chr12	101445239	101445449	-3.24	-0.14
Dync1h1	chr12	111839634	111839815	-0.41	-0.05
Dync1h1	chr12	111855934	111857506	-0.43	-0.01

Dync1h1	chr12	111904592	111905154	-1.97	-0.13
Klc1	chr12	112997059	113010574	-0.30	-0.01
Btbd6	chr12	114215520	114217031	-10.90	-0.80
Nfkbia	chr12	56593395	56593604	-103.00	-1.36
Ppp2r5e	chr12	76554410	76554710	-5.69	0.49
Ppp2r5e	chr12	76594419	76616258	-1.73	0.31
Ppp2r5e	chr12	76616527	76694287	-1.42	-0.18
Ppp2r5e	chr12	76696767	76697187	-1.88	-0.25
Arel1	chr12	86259755	86261648	-4.64	-0.19
Arel1	chr12	86311686	86311836	-0.86	0.32
Lgmn	chr12	103651753	103677907	-27.90	-0.55
Akt1	chr12	113912217	113912487	-1.97	-0.56
Rnf182	chr13	43763214	43763781	-1.24	5.46
Mylip	chr13	45485140	45499872	-7.91	0.67
Mylip	chr13	45503785	45506564	-28.20	0.99
Rnf144b	chr13	47338305	47340700	-1.08	-0.22
Rnf144b	chr13	47342881	47343181	-0.33	-0.08
Syk	chr13	52678805	52706632	-13.30	-0.48
Syk	chr13	52727069	52733575	-2.37	-0.22
Syk	chr13	52737300	52738605	-0.64	0.10
Syk	chr13	52738964	52742902	-5.19	-0.18
Syk	chr13	52743770	52743981	-0.44	0.06
Erap1	chr13	74777319	74784015	-3.77	0.34
Tubb2a	chr13	34166146	34168454	-10.90	0.67
Tubb2b	chr13	34219290	34219586	-1.55	1.29
Tubb2b	chr13	34219940	34221135	-0.88	0.43
Ctsl	chr13	64470393	64471614	-17.40	-0.32
Pik3r1	chr13	102453986	102456264	-3.37	0.30
Pik3r1	chr13	102478932	102525656	-0.37	-0.05
Pik3r1	chr13	102527244	102538172	-0.72	0.07
Kctd6	chr14	9052713	9055575	-1.94	-0.30
Sec24c	chr14	21512428	21513175	-11.10	-0.51
Sec24c	chr14	21513593	21514072	-11.40	-0.48
Psmc6	chr14	45949498	45950514	-0.60	-0.09
Ctsb	chr14	63761159	63761760	-164.00	-0.25
Kbtbd7	chr14	79827156	79828746	-2.42	0.38
Ppp3cb	chr14	21320302	21327131	-0.50	-0.04
Ppp3cb	chr14	21365614	21365735	-0.90	-0.41
Fbxl3	chr14	103482090	103488631	-0.87	-0.07
Fbxl3	chr14	103491640	103498113	-0.65	0.09
Fbxl3	chr14	103498172	103498726	-0.67	-0.17
Fyb	chr15	6529870	6569639	-15.30	-0.34
Fyb	chr15	6584885	6590459	-12.30	-0.75

Rictor	chr15	6736456	6737266	-1.44	-0.13
Rictor	chr15	6745958	6747284	-0.93	0.10
Ncf4	chr15	78092626	78092951	-7.16	-0.30
Rbx1	chr15	81305971	81306061	-1.28	-0.57
Tuba1c	chr15	98868116	98868326	-32.90	-0.97
Skp2	chr15	9043329	9043510	-0.95	0.41
Skp2	chr15	9069207	9070137	-7.83	-1.04
Rnf19a	chr15	36170946	36173866	-1.80	-0.21
Rnf19a	chr15	36195033	36212902	-6.05	0.56
Ywhaz	chr15	36723285	36723376	-0.55	0.11
Fbxo32	chr15	58011902	58012112	-0.40	-0.84
Fbxo32	chr15	58012410	58013039	-1.36	-1.07
Fbxo32	chr15	58046223	58046433	-0.22	-0.12
Col2a1	chr15	97806241	97806600	-2.18	2.47
Asb8	chr15	97965969	97971744	-1.49	-0.17
Racgap1	chr15	99470224	99481962	-0.78	0.26
Itgb7	chr15	102046603	102046986	-2.10	-0.17
Itgb7	chr15	102057997	102062366	-0.74	-0.15
Mgrn1	chr16	4936923	4937342	-0.32	-0.01
Klhl22	chr16	17771956	17777358	-0.41	-0.03
Ap2m1	chr16	20535576	20537801	-1.17	-0.35
Psmd2	chr16	20651724	20652324	-20.60	-0.49
Itgb5	chr16	33829750	33830079	-4.10	-1.27
Itgb5	chr16	33919922	33920221	-1.38	-0.29
Itgb5	chr16	33947843	33949245	-1.75	-0.23
Cd80	chr16	38473975	38479185	-8.20	-0.52
Cd80	chr16	38486386	38486895	-4.13	-0.46
Cd80	chr16	38487013	38494806	-2.19	0.67
Cd200r1	chr16	44794312	44794552	-1.93	-0.75
Cd200r4	chr16	44811897	44820900	-2.09	-1.18
Cd200r2	chr16	44915300	44915864	-0.30	-0.12
Cblb	chr16	52204625	52205075	-5.73	-0.89
Socs1	chr16	10783990	10784912	-0.46	-0.26
Socs1	chr16	10784970	10785629	-0.59	-1.60
Pak2	chr16	32019862	32021909	-0.84	0.05
Dtx3l	chr16	35928994	35932076	-0.66	-0.06
Dtx3l	chr16	35932345	35933542	-0.72	-0.06
Cd86	chr16	36604253	36604522	-0.59	-0.66
Ltn1	chr16	87415807	87416587	-1.51	0.15
Itpr3	chr17	27258568	27258749	-1.96	-0.56
Tapbp	chr17	34056422	34056928	-18.20	-0.66
Tapbp	chr17	34062443	34063061	-65.40	-0.74
H2-DMa	chr17	34272232	34272620	-0.59	0.23

Tap1	chr17	34324500	34325221	-16.90	-0.87
Tap1	chr17	34333599	34333840	-0.55	-0.11
Psmb8	chr17	34335288	34335468	-2.22	-0.33
Tap2	chr17	34341453	34342537	-9.79	-0.80
Tap2	chr17	34352342	34353206	-4.14	-0.42
H2-D1	chr17	35400038	35400558	-64.10	-0.68
H2-D1	chr17	35400926	35402744	-4.71	-0.33
H2-D1	chr17	35403816	35404204	-71.80	-0.36
H2-Q2	chr17	35479307	35479570	-0.14	0.42
H2-Q6	chr17	35561821	35562745	-5.87	-0.37
H2-Q6	chr17	35565949	35566720	-1.19	-0.34
H2-Q7	chr17	35576925	35577015	-0.36	-0.83
H2-Q10	chr17	35607880	35610039	-0.46	-0.18
Nfkbie	chr17	45692664	45693263	-1.50	-0.23
Nfkbie	chr17	45697537	45700027	-7.33	-0.49
Cul7	chr17	46788096	46788277	-2.58	3.86
Cul7	chr17	46788516	46788967	-0.83	-0.64
Cul7	chr17	46791019	46791200	-0.63	1.18
Trem1	chr17	48371921	48385080	-1.75	0.78
Treml2	chr17	48439293	48439684	-0.27	0.22
Treml2	chr17	48449070	48449580	-0.73	-0.24
Vav1	chr17	57418522	57418613	-0.31	0.06
Vav1	chr17	57467106	57467554	-1.28	0.13
Rasgrp3	chr17	75924352	75926570	-0.88	0.08
Rasgrp3	chr17	75928033	75928214	-0.97	-0.23
Lnpep	chr17	17715497	17746283	-1.10	-0.05
Pdpk1	chr17	24215553	24216093	-11.80	0.63
Ccnf	chr17	24360594	24360954	-5.87	0.78
Ccnf	chr17	24361042	24361933	-12.00	1.09
Mlst8	chr17	24612351	24613045	-0.59	-0.10
Stub1	chr17	25967580	25967909	-3.51	-0.27
Stub1	chr17	25969947	25970008	-0.31	-0.35
Stub1	chr17	25970096	25970306	-1.82	-0.63
H2-K1	chr17	34133105	34133495	-21.60	-0.51
H2-K1	chr17	34136728	34137188	-34.60	-0.71
H2-T23	chr17	36169189	36248130	-6.88	-0.55
Trim39	chr17	36397225	36398520	-0.49	0.05
Trim39	chr17	36400794	36404080	-0.93	-0.33
Klc4	chr17	46781254	46782971	-0.81	-0.28
Ppp2r5d	chr17	46820058	46820862	-0.90	-0.11
Ppp2r5d	chr17	46837029	46841951	-0.53	-0.11
Ubr2	chr17	47065869	47067369	-3.79	-0.22
Ubr2	chr17	47147270	47147481	-5.21	-0.65

Tubb4a	chr17	57220052	57220528	-1.24	-0.37
Tubb4a	chr17	57220616	57221122	-1.23	-0.35
Pja2	chr17	64658065	64658365	-1.01	-0.18
Pja2	chr17	64658514	64660528	-2.68	-0.35
Xdh	chr17	74233606	74233727	-0.41	-0.14
Sos1	chr17	80797100	80799127	-0.43	-0.03
Sos1	chr17	80833039	80834290	-0.90	-0.18
Sos1	chr17	80879343	80879554	-1.15	-0.89
Calm2	chr17	87835011	87846216	-2.03	-0.14
Fbxo11	chr17	88390945	88391686	-0.95	0.09
Fbxo11	chr17	88464385	88464625	-1.02	-0.30
Colec12	chr18	9707645	9840283	-0.36	-0.01
Colec12	chr18	9848284	9859890	-0.96	-0.03
Colec12	chr18	9874892	9877425	-4.78	0.23
Ube2d2a	chr18	35931242	35931392	-3.18	-0.65
Ube2d2a	chr18	35965479	35965570	-0.43	0.27
Rnf14	chr18	38467690	38467990	-0.56	0.10
Dctn4	chr18	60686499	60686560	-0.25	-0.32
Dctn4	chr18	60716315	60716526	-1.59	0.32
Malt1	chr18	65635658	65636018	-2.72	-0.30
Tubb6	chr18	67550384	67552360	-1.76	-0.20
Tubb6	chr18	67552568	67561747	-29.40	-0.45
Mex3c	chr18	73732538	73732629	-1.20	1.10
Mex3c	chr18	73732718	73732899	-1.18	-2.10
Mex3c	chr18	73732988	73750582	-0.64	0.04
Map3k8	chr18	4348899	4352951	-3.59	-0.54
Kif5b	chr18	6241222	6241522	-8.22	-0.56
Cdc23	chr18	34792901	34793291	-0.68	-0.16
Cdc23	chr18	34793470	34793621	-0.76	-0.25
Trim36	chr18	46326839	46328996	-8.68	1.58
Nfatc1	chr18	80802973	80803394	-0.79	-0.30
Nfatc1	chr18	80803872	80832717	-0.41	0.03
Nfatc1	chr18	80846232	80846653	-2.71	0.51
Nfatc1	chr18	80879052	80904894	-2.97	0.23
Sptbn2	chr19	4744054	4745130	-1.41	-0.53
Rela	chr19	5646968	5647773	-3.54	-0.20
Cd274	chr19	29441927	29455003	-40.60	-1.29
Cd274	chr19	29456978	29460318	-29.60	-1.05
Pdcd1lg2	chr19	29545061	29545301	-0.32	0.93
Pten	chr19	32832126	32832995	-2.07	-0.13
Pten	chr19	32872591	32874607	-0.82	0.07
Pten	chr19	32892519	32894752	-15.20	0.55
Kif11	chr19	37494852	37495032	-4.82	-1.09

Btrc	chr19	45604117	45604298	-0.65	1.17
Fbxl15	chr19	46403649	46404936	-3.45	-0.46
Klc2	chr19	5117426	5118318	-2.71	-0.53
Sipa1	chr19	5651184	5651544	-0.61	-0.07
Sipa1	chr19	5653976	5655148	-7.20	-0.43
Sipa1	chr19	5659601	5662646	-15.60	-0.49
Ppp2r5b	chr19	6227944	6228243	-1.38	-0.37
Pik3ap1	chr19	41348707	41357626	-14.30	-0.57
Pik3ap1	chr19	41406769	41459560	-89.20	-1.22
Chuk	chr19	44181817	44181937	-0.55	-0.16
Fbxw4	chr19	45652866	45653256	-0.75	-0.11
Actr1a	chr19	46452346	46452913	-2.60	-0.15
Ube2a	chrX	34414360	34414420	-1.18	-0.44
Kif4	chrX	97921893	97922371	-1.53	-0.53
Huwe1	chrX	148353194	148354606	-2.14	0.09
Was	chrX	7667496	7667617	-0.70	0.20
Pja1	chrX	96661699	96661909	-2.76	0.63
Pja1	chrX	96662087	96663013	-1.08	0.12
Pja1	chrX	96663072	96663789	-0.87	-0.14
Rlim	chrX	101156612	101157212	-16.70	0.50
Rlim	chrX	101157331	101160637	-88.80	0.50
Siah1b	chrX	160509638	160510081	-0.33	0.04
Uba6	chr5	86541279	86547113	-0.45	0.04
Stim1	chr7	109416725	109416845	-0.32	0.12
Dynll2	chr11	87794645	87799923	-0.59	0.05
Dynll2	chr11	87800072	87801030	-0.39	-0.04

Death receptor signaling

gene_name	chr	chromStart	chromEnd	diff.lg.p	diff.log2.fc
Arhgef4	chr1	34868671	34868941	-2.97	-1.43
Cflar	chr1	58779883	58783449	-1.33	0.34
Cflar	chr1	58809209	58810837	-23.80	-0.48
Casp8	chr1	58890568	58901646	-12.80	-0.51
Usp21	chr1	173216496	173218062	-11.50	-1.09
Ncstn	chr1	173996440	173996917	-4.50	-0.29
Psen2	chr1	182157552	182159055	-0.95	-0.16
Traf6	chr2	101530344	101537929	-2.95	-0.21
Bcl2l11	chr2	127951773	127953425	-15.00	-0.75
Bcl2l11	chr2	127953514	127954592	-1.66	-0.24
Bcl2l11	chr2	127954771	127954832	-1.61	0.69
Bcl2l11	chr2	127957197	127984389	-7.23	0.50
Bcl2l11	chr2	127985436	127985737	-30.60	2.16
Bcl2l11	chr2	127987413	127987774	-17.40	1.64
Traf2	chr2	25374427	25375145	-2.44	-0.26

Vav2	chr2	27282104	27282345	-2.65	-1.29
Traf1	chr2	34798777	34802204	-45.80	-0.96
Traf1	chr2	34803536	34803955	-12.30	-0.99
Traf1	chr2	34813569	34814068	-7.79	-1.05
Madd	chr2	90977546	90977726	-1.15	-0.52
Madd	chr2	91018210	91018420	-1.85	-0.53
Prex1	chr2	166392563	166394859	-1.63	-0.17
Tnfsf10	chr3	27234390	27234869	-0.65	3.00
Prkci	chr3	30949234	30949445	-1.06	-0.32
Arhgef11	chr3	87536744	87538447	-8.91	-0.65
Arhgef11	chr3	87541566	87541835	-0.57	0.10
Arhgef2	chr3	88425102	88425163	-2.57	-0.61
Arhgef2	chr3	88448103	88451105	-20.20	-0.40
Aph1a	chr3	95700181	95700660	-12.80	-0.63
Otud7b	chr3	95908449	95908540	-1.47	-0.65
Otud7b	chr3	95956546	95960193	-6.78	-0.26
Vav3	chr3	109143660	109143930	-1.24	-0.66
Nfkb1	chr3	135248247	135252299	-9.58	-0.45
Nfkb1	chr3	135354240	135354451	-1.35	0.77
Map3k7	chr4	32051081	32051232	-3.02	-1.01
Map3k7	chr4	32104109	32107267	-5.97	0.38
Map3k7	chr4	32110051	32110231	-0.27	0.00
Plekhg5	chr4	151487978	151488579	-2.17	-0.88
Plekhg5	chr4	151488878	151488999	-0.20	0.17
Plekhg5	chr4	151489118	151489179	-1.40	-2.24
Ripk2	chr4	16050670	16051621	-4.17	-0.41
Ripk2	chr4	16090436	16090645	-16.00	-1.44
Arhgef10l	chr4	140070399	140072692	-7.33	-0.37
Ubc	chr5	125866453	125867671	-172.00	0.73
Ubc	chr5	125867938	125868147	-3.15	0.20
Ubc	chr5	125868235	125870298	-22.80	-0.40
Rac1	chr5	144288681	144288861	-1.21	-0.22
Tnfrsf1a	chr6	125311560	125312501	-18.50	-0.45
Arhgef1	chr7	25688004	25693331	-1.99	-0.19
Akap13	chr7	82748167	82754790	-0.38	-0.01
Akap13	chr7	82754999	82756802	-18.60	-0.39
Akap13	chr7	82892510	82896016	-22.20	-0.39
Plekhg2	chr7	29144712	29147558	-0.33	0.01
Plekhg2	chr7	29155536	29156206	-1.52	0.87
Arhgef17	chr7	108021498	108022099	-0.52	0.31
Arhgef17	chr7	108078095	108078156	-0.12	0.00
Arhgef17	chr7	108079054	108079265	-0.17	-0.50
Fadd	chr7	151765572	151768192	-4.39	-0.39

Arhgef18	chr8	3452229	3453340	-2.38	-0.75
Arhgef18	chr8	3454835	3455284	-0.47	-0.12
Arhgef7	chr8	11831528	11833540	-13.80	-0.29
Mcf2l	chr8	12963213	12984656	-5.57	-1.12
Mcf2l	chr8	13019010	13019580	-1.13	-0.29
Arhgef10	chr8	14997603	15000037	-6.47	-0.70
Cyld	chr8	91220956	91226218	-0.50	0.37
Cyld	chr8	91226307	91233793	-2.31	0.21
Ikbkb	chr8	23770606	23771027	-1.32	-0.20
Bag4	chr8	26877798	26878878	-1.37	-0.17
Tradd	chr8	107782474	107783499	-1.93	-0.23
Tradd	chr8	107783873	107788494	-0.92	-0.28
Smpd3	chr8	108789066	108789216	-0.20	0.24
Usp2	chr9	43883786	43884026	-0.81	2.12
Usp2	chr9	43902032	43902302	-0.96	-0.35
Usp4	chr9	108293381	108294591	-3.30	-0.22
Birc2	chr9	7818614	7818734	-0.55	-0.29
Birc2	chr9	7833610	7834657	-0.90	0.13
Birc2	chr9	7834955	7835195	-1.05	0.42
Birc3	chr9	7848969	7850928	-1.17	-0.17
Birc3	chr9	7860311	7872961	-0.30	0.00
Arhgef12	chr9	42780101	42782733	-0.29	0.00
Arhgef12	chr9	42850752	42913771	-0.50	-0.08
Aph1b	chr9	66626699	66627088	-0.85	0.47
Aph1c	chr9	66666666	66667266	-0.57	-0.14
Myd88	chr9	119248978	119249158	-3.52	-0.60
Sppl2b	chr10	80329626	80329807	-1.32	-0.66
Sppl2b	chr10	80330135	80331453	-14.20	-0.76
Tab2	chr10	7625983	7626463	-1.79	0.18
Tab2	chr10	7626701	7630910	-3.35	-0.17
Tab2	chr10	7631204	7644574	-1.97	-0.07
Tnfaip3	chr10	18721581	18725137	-0.67	-0.06
Smpd2	chr10	41209967	41210146	-1.95	1.58
Rtn4	chr11	29592927	29593816	-51.00	-0.53
Rtn4	chr11	29606773	29607134	-5.20	-0.35
Rtn4	chr11	29607432	29607523	-1.42	-0.29
Rtn4	chr11	29607911	29608181	-6.14	-0.46
Ywhae	chr11	75546388	75546567	-5.18	-0.26
Gna13	chr11	109224197	109241616	-6.09	-0.29
Gna13	chr11	109241765	109257871	-5.85	-0.25
Abr	chr11	76232302	76233353	-25.70	-0.38
Abr	chr11	76308077	76308138	-0.36	0.21
Abr	chr11	76308197	76308378	-1.63	0.63

Abr	chr11	76322437	76322617	-6.70	-0.41
Abr	chr11	76384647	76384857	-5.06	-0.38
Arhgdia	chr11	120440341	120440731	-101.00	-0.45
Arhgdia	chr11	120441525	120442361	-23.00	-0.29
Psen1	chr12	85072679	85075730	-7.32	-0.20
Adam17	chr12	21330026	21333946	-5.54	-0.14
Nfkbia	chr12	56593395	56593604	-103.00	-1.36
Sos2	chr12	70685554	70689696	-1.22	0.08
Sos2	chr12	70717850	70719054	-1.36	0.14
Sos2	chr12	70782599	70782839	-0.78	-0.61
Ripk1	chr13	34094265	34094762	-9.02	-0.95
Ripk1	chr13	34119794	34125274	-0.32	0.00
Ripk1	chr13	34125393	34126082	-9.17	-0.86
Net1	chr13	3883241	3883659	-0.91	0.17
Net1	chr13	3892692	3892752	-0.24	-0.08
Fgd3	chr13	49358508	49360098	-1.29	-0.14
Fgd3	chr13	49391745	49393279	-4.23	-0.50
Arhgef3	chr14	28213461	28215507	-1.09	0.15
Arhgef3	chr14	28215804	28216373	-2.44	0.41
Arhgef40	chr14	52604567	52604628	-0.33	-0.38
Arhgef40	chr14	52608387	52609787	-5.89	-0.72
Arhgef40	chr14	52616039	52617414	-6.29	-1.03
Tnfrsf10b	chr14	70182066	70182455	-0.75	0.31
Tab1	chr15	79989230	79992132	-13.10	-0.63
Otulin	chr15	27535675	27538550	-1.81	-0.14
Otulin	chr15	27553050	27560448	-1.52	-0.31
Trio	chr15	27661541	27663334	-0.78	-0.07
Trio	chr15	27673777	27673868	-0.37	-0.07
Trio	chr15	27677706	27678186	-5.50	-0.55
Trio	chr15	27830982	27832759	-1.60	-0.21
Sharpin	chr15	76180541	76181540	-3.28	-0.38
Fgd4	chr16	16422549	16422879	-1.42	0.27
Fgd4	chr16	16475223	16482703	-0.55	0.11
Fgd4	chr16	16484139	16487285	-1.97	0.25
Tiam1	chr16	89789181	89791933	-0.49	0.11
Tiam1	chr16	89885019	89898566	-2.35	0.47
Tiam2	chr17	3518257	3518737	-0.63	0.27
Tiam2	chr17	3518976	3519397	-1.05	0.40
Vav1	chr17	57418522	57418613	-0.31	0.06
Vav1	chr17	57467106	57467554	-1.28	0.13
Tnf	chr17	35336694	35337384	-434.00	-1.28
Tnf	chr17	35338760	35338941	-117.00	-1.09
Sos1	chr17	80797100	80799127	-0.43	-0.03

Sos1	chr17	80833039	80834290	-0.90	-0.18
Sos1	chr17	80879343	80879554	-1.15	-0.89
Rela	chr19	5646968	5647773	-3.54	-0.20
Bad	chr19	7017092	7025164	-1.35	-0.34
Fas	chr19	34401551	34402260	-3.03	0.67
Chuk	chr19	44181817	44181937	-0.55	-0.16
Xiap	chrX	39447566	39448377	-1.27	0.11
Xiap	chrX	39453031	39458192	-4.92	0.44
Tab3	chrX	82859477	82860195	-4.67	0.57
Tab3	chrX	82860522	82860673	-1.01	0.46
Tab3	chrX	82873570	82876044	-16.40	2.20
Bex3	chrX	132804820	132805902	-4.60	0.52
Irak1	chrX	71260386	71260835	-0.46	0.04

Metabolism of RNA

gene_name	chr	chromStart	chromEnd	diff.lg.p	diff.log2.fc
Cnot11	chr1	39603302	39603632	-9.27	-1.03
Cnot9	chr1	74575438	74575798	-1.13	-0.18
Utp14b	chr1	78654429	78655125	-1.94	0.30
Utp14b	chr1	78656939	78664176	-185.00	1.38
Sf3b1	chr1	55084201	55084322	-0.60	-0.13
Ncl	chr1	88246797	88247451	-0.39	0.01
Ncl	chr1	88253281	88253706	-90.30	-0.41
Ncl	chr1	88254778	88256030	-0.67	0.03
Zc3h11a	chr1	135516447	135517433	-9.17	-0.31
Zc3h11a	chr1	135517641	135518733	-2.10	-0.10
Zc3h11a	chr1	135521340	135523781	-17.40	-0.37
Smg7	chr1	154685021	154685380	-1.89	-0.30
Smg7	chr1	154686037	154687255	-2.82	-0.26
Smg7	chr1	154695761	154696002	-16.00	-0.86
Dhx9	chr1	155303276	155303397	-3.58	0.71
Hnrnpu	chr1	180259403	180260044	-6.87	-0.19
Hnrnpu	chr1	180267465	180267915	-20.60	-0.28
Diexf	chr1	194933444	194939955	-1.58	-0.22
Diexf	chr1	194944545	194948207	-3.72	-0.29
Exosc2	chr2	31536089	31536414	-1.79	0.28
Nup214	chr2	31888785	31893623	-1.70	-0.12
Tnks1bp1	chr2	84891036	84904146	-4.25	-0.23
Psmc3	chr2	90894439	90895182	-3.33	-0.20
Cstf3	chr2	104504891	104505372	-2.93	0.31
Nop56	chr2	130100147	130100207	-0.37	-0.09
Nop56	chr2	130104634	130105019	-8.24	-0.34
Nxt1	chr2	148498350	148501762	-3.02	-0.22
Ctnnbl1	chr2	157563136	157600315	-0.47	-0.04

Srsf6	chr2	162757243	162757817	-0.30	0.00
Ywhab	chr2	163820932	163837497	-10.90	-0.39
Cstf1	chr2	172203242	172206586	-1.17	-0.10
Rae1	chr2	172840787	172841203	-9.79	-0.73
Rpp38	chr2	3246220	3249900	-0.56	0.06
Rbm17	chr2	11522379	11524826	-2.08	-0.47
Psmd5	chr2	34707993	34709823	-1.65	-0.19
Psmd5	chr2	34726274	34726482	-4.41	-0.82
Psmb7	chr2	38498946	38499426	-3.13	-0.36
Prpf40a	chr2	53049040	53050262	-0.95	-0.15
Cwc22	chr2	77733949	77741916	-2.10	-0.15
Clp1	chr2	84563518	84566269	-0.31	0.00
Snrpb	chr2	130004981	130005100	-1.98	-0.21
Psmf1	chr2	151543111	151543202	-1.31	-0.87
Psmf1	chr2	151543649	151544038	-0.30	0.01
Smg5	chr3	88140181	88140391	-2.26	-0.53
Smg5	chr3	88154740	88155513	-6.71	-0.28
Smg5	chr3	88165123	88165423	-0.78	0.12
Adar	chr3	89518943	89540188	-49.20	-0.49
Adar	chr3	89554632	89555344	-14.70	-0.54
Adar	chr3	89555553	89557018	-8.75	-0.29
Sf3b4	chr3	95976984	95978290	-33.20	-0.63
Bcas2	chr3	102981340	102982484	-12.40	0.55
Psma5	chr3	108059873	108060022	-0.64	3.39
Prcc	chr3	87662883	87664130	-1.84	-0.22
Prcc	chr3	87673527	87689484	-20.90	-0.46
Chtop	chr3	90303653	90306049	-0.50	-0.02
Chtop	chr3	90313185	90313365	-3.07	-0.25
Psmb4	chr3	94690701	94690880	-8.50	-0.41
Psmd4	chr3	94836626	94836894	-2.54	-0.16
Psmd4	chr3	94840526	94846467	-8.21	-0.35
Wdr3	chr3	99942341	99942760	-0.77	0.07
Ddx20	chr3	105481379	105482396	-2.03	-0.19
Srsf11	chr3	157674772	157675808	-16.50	-0.32
Tgs1	chr4	3512631	3516319	-4.05	0.25
Tgs1	chr4	3542039	3542248	-0.89	-0.20
Rngtt	chr4	33586031	33587733	-1.66	0.32
Rngtt	chr4	33588270	33588360	-0.29	0.12
Prpf4	chr4	62083727	62085481	-2.02	-0.20
Ndc1	chr4	107083754	107086943	-1.16	0.18
Tut4	chr4	108151617	108159137	-2.09	0.18
Tut4	chr4	108185594	108190009	-0.40	-0.04
Ebna1bp2	chr4	118293433	118293494	-3.14	-0.87

Lsm10	chr4	125773896	125775828	-0.39	0.02
Srsf4	chr4	131453751	131457610	-9.11	-0.28
Dnajc8	chr4	132108975	132109154	-0.85	-0.09
Syf2	chr4	134486894	134490903	-15.80	-0.46
Syf2	chr4	134493303	134493452	-1.95	-0.45
Hnrnpr	chr4	135895398	135895548	-0.47	-0.07
Hnrnpr	chr4	135895666	135895966	-2.48	-0.47
Exosc10	chr4	147932535	147933683	-1.79	-0.34
Nol9	chr4	151431914	151434061	-7.78	-0.27
Nol6	chr4	41062057	41062930	-3.18	-0.23
Nol6	chr4	41070603	41071312	-0.94	0.22
Exosc3	chr4	45333265	45333475	-2.99	-0.49
Tex10	chr4	48481183	48482144	-0.81	-0.07
Ybx1	chr4	118957671	118967118	-120.00	-0.62
Ppih	chr4	118979868	118980437	-3.08	-0.75
Srrm1	chr4	134878175	134878385	-1.65	-0.18
Srrm1	chr4	134880008	134881048	-18.30	-0.47
Srrm1	chr4	134902739	134909069	-0.66	-0.08
Nupl2	chr5	23687726	23687877	-0.80	0.38
Nupl2	chr5	23687966	23689616	-1.38	-0.19
Utp3	chr5	88983507	88985078	-0.40	-0.01
Psmd9	chr5	123678228	123678437	-0.34	0.03
Snrnp35	chr5	124933163	124940925	-3.86	-0.36
Gtf2h3	chr5	125046192	125046313	-0.64	-0.25
Bud31	chr5	145908765	145908943	-1.40	-0.20
Cpsf4	chr5	145939775	145942700	-0.65	0.07
Pan3	chr5	148242155	148242515	-1.13	-0.38
Pan3	chr5	148357203	148359120	-0.42	-0.02
Slbp	chr5	33992455	33994955	-3.22	0.32
Nop14	chr5	35002499	35002797	-5.80	-0.43
Nup54	chr5	92845130	92846543	-1.18	-0.16
Hnrnpd	chr5	100407566	100407657	-0.58	0.43
Ubc	chr5	125866453	125867671	-172.00	0.73
Ubc	chr5	125867938	125868147	-3.15	0.20
Ubc	chr5	125868235	125870298	-22.80	-0.40
Dhx37	chr5	125894833	125895712	-1.52	-0.17
Dhx37	chr5	125910274	125914418	-7.00	-0.47
Pom121	chr5	135853620	135857139	-7.02	-0.36
Pom121	chr5	135857525	135857795	-2.51	-0.32
Nup205	chr6	35127615	35133895	-0.30	0.02
Hnrnpf	chr6	117856811	117856962	-2.52	-0.35
Hnrnpf	chr6	117873875	117874265	-7.79	-0.46
Hnrnpf	chr6	117874653	117874893	-29.00	-0.62

Ddx47	chr6	134971495	134973794	-1.67	-0.12
Gm8994	chr6	136279021	136279141	-0.30	-0.81
Gm8994	chr6	136279763	136279853	-0.25	0.86
Cnot4	chr6	34973322	34992713	-1.58	0.32
Cnot4	chr6	34996096	35002891	-0.80	0.08
Hnrnpa2b1	chr6	51419743	51419893	-2.17	-0.20
Usp39	chr6	72268758	72269207	-0.81	0.11
Usp39	chr6	72294989	72295169	-8.82	-1.02
Pcbp1	chr6	86474877	86476159	-0.55	-0.01
Isy1	chr6	87769040	87771528	-1.21	-0.18
Nup210	chr6	90963060	90963660	-3.36	-0.50
Bms1	chr6	118333488	118334272	-2.75	-0.21
Bms1	chr6	118348132	118355257	-6.54	-0.25
Apobec1	chr6	122528673	122531597	-2.92	-0.14
Wbp11	chr6	136762473	136765520	-10.20	-0.30
Prpf31	chr7	3581586	3582328	-1.76	-0.17
Prpf31	chr7	3591264	3593127	-11.30	-0.32
U2af2	chr7	5030883	5031212	-1.10	-0.10
Snrpd2	chr7	19735186	19736596	-0.85	-0.20
Ercc2	chr7	19979470	19981041	-4.45	-0.33
Polr2i	chr7	31017332	31017772	-0.55	0.13
Nup62	chr7	52071739	52086176	-19.00	-0.23
Gtf2h1	chr7	54051493	54051644	-1.33	-0.22
Gtf2h1	chr7	54078270	54078601	-5.40	0.33
Snrpa1	chr7	73205399	73205549	-2.12	-0.41
Gemin7	chr7	20150297	20158692	-0.85	0.07
Hnrnpul1	chr7	26533405	26539769	-33.90	-0.44
Psmd8	chr7	29965393	29965573	-13.80	-0.43
Zfp473	chr7	51987480	51987751	-0.36	-0.29
Zfp473	chr7	51987810	51988051	-0.70	-0.61
Zfp473	chr7	51988379	51988830	-1.38	1.24
Zfp473	chr7	51988979	51989760	-8.39	2.40
Snrnp70	chr7	52631823	52636159	-50.50	-0.38
Snrnp70	chr7	52647692	52651017	-13.70	-0.31
Mphosph10	chr7	71534493	71537122	-7.76	-0.33
Pcf11	chr7	99793118	99795438	-8.06	0.60
Pcf11	chr7	99801721	99808380	-11.00	-0.25
Pcf11	chr7	99809567	99810434	-2.11	-0.10
Pcf11	chr7	99818003	99818422	-5.53	-0.40
Nup98	chr7	109268003	109269291	-7.87	-0.22
Nup98	chr7	109283070	109283401	-23.60	-0.89
Cd2bp2	chr7	134337180	134338647	-23.50	-0.40
Exosc6	chr8	113580238	113581499	-1.47	-0.13

Elavl1	chr8	4289099	4289460	-0.68	0.07
Cnot7	chr8	41592761	41593001	-0.56	0.08
Ddx49	chr8	72816972	72817479	-1.42	-0.20
Upf1	chr8	72855420	72856610	-3.27	-0.17
Upf1	chr8	72868160	72877112	-12.40	-0.52
Psmb10	chr8	108461631	108461967	-2.80	-0.63
Nob1	chr8	109936418	109936925	-1.11	-0.11
Psmd7	chr8	110104338	110108077	-4.03	0.12
Dhx38	chr8	112071923	112073753	-0.53	0.05
Dhx38	chr8	112080084	112080835	-6.56	0.50
Dhx38	chr8	112084553	112089501	-0.31	0.00
Sf3b3	chr8	113334571	113334842	-1.18	-0.08
Mphosph6	chr8	120316041	120316188	-0.73	-0.16
Ddx6	chr9	44412974	44415652	-14.10	0.42
Ddx6	chr9	44443935	44445638	-31.20	0.44
Imp3	chr9	56785306	56786205	-2.20	-0.11
Snupn	chr9	56830587	56830946	-0.56	-0.13
Edc3	chr9	57564009	57575286	-3.38	-0.43
Edc3	chr9	57596029	57596567	-0.40	0.04
Xrn1	chr9	95953058	95953238	-0.45	0.06
Rrp9	chr9	106379639	106380024	-1.05	0.37
Cnot10	chr9	114541870	114549228	-1.75	-0.36
Ppil4	chr10	7534486	7541105	-2.05	-0.23
Sf3b5	chr10	12728255	12728989	-8.10	-0.27
Hbs1l	chr10	21087565	21088516	-3.49	-0.34
Ranbp2	chr10	57939345	57940486	-6.91	0.22
Ranbp2	chr10	57940784	57941055	-0.33	-0.01
Ranbp2	chr10	57941564	57941955	-1.34	-0.09
Ranbp2	chr10	57942134	57945844	-28.50	0.35
Ranbp2	chr10	57955388	57956782	-8.30	0.31
Wdr18	chr10	79422896	79423107	-1.13	0.21
Sf3a2	chr10	80261479	80263960	-0.95	-0.26
Sf3a2	chr10	80266830	80266980	-0.93	-0.18
Pan2	chr10	127740450	127741373	-0.96	-0.37
Pan2	chr10	127747269	127747789	-1.16	-0.31
Ddx21	chr10	62044843	62047448	-7.21	-0.17
Ddx21	chr10	62057824	62061631	-34.70	-0.29
Ccar1	chr10	62208074	62209806	-0.86	0.08
Ccar1	chr10	62214563	62223000	-0.51	-0.03
Adarb1	chr10	76757182	76758518	-0.39	-0.10
Adarb1	chr10	76784665	76785295	-4.91	1.42
Pwp2	chr10	77633953	77636365	-1.10	-0.10
Polr2e	chr10	79501316	79502404	-2.85	-0.43

Utp20	chr10	88209351	88209771	-0.81	-0.10
Cnot2	chr10	115922246	115922606	-0.99	-0.19
Cnot2	chr10	115922874	115923144	-4.87	0.64
Pes1	chr11	3878848	3878938	-0.29	0.00
Sf3a1	chr11	4080296	4080657	-2.08	-0.28
Zmat5	chr11	4604673	4622487	-2.64	-0.83
Psme4	chr11	30671774	30672135	-0.54	-0.18
Snrnp25	chr11	32105414	32105652	-1.78	-0.52
Slu7	chr11	43247232	43251659	-1.97	0.22
Slu7	chr11	43258751	43259812	-3.05	-0.23
Slu7	chr11	43260795	43260976	-0.25	0.11
Hnrnph1	chr11	50191824	50191914	-0.98	0.13
Ppp2ca	chr11	51912325	51912474	-4.51	-0.88
Ppp2ca	chr11	51935449	51935836	-0.34	0.00
Cnot8	chr11	57930958	57931258	-0.74	0.18
Tsr1	chr11	74711581	74711923	-5.50	-0.71
Tsr1	chr11	74713717	74715018	-7.32	-0.41
Smg6	chr11	74739373	74744156	-1.79	-0.16
Ddx52	chr11	83773028	83775782	-0.43	0.04
Ddx52	chr11	83776110	83776290	-0.43	-0.10
Srsf1	chr11	87861172	87861322	-1.85	-0.33
Psmd3	chr11	98543867	98546829	-30.30	-0.74
Casc3	chr11	98683710	98689970	-6.76	-0.34
Casc3	chr11	98692721	98694074	-0.45	0.04
Psme3	chr11	101177564	101178336	-0.62	0.07
Psme3	chr11	101182070	101183288	-0.64	-0.06
Ddx42	chr11	106078239	106085433	-6.07	-0.73
Ddx42	chr11	106108808	106109826	-12.90	-0.40
Pno1	chr11	17111355	17111474	-3.09	-0.34
Lsm11	chr11	45747027	45747446	-3.16	-0.71
Gemin5	chr11	57933831	57934100	-1.45	0.73
Gemin5	chr11	57934607	57938883	-0.67	0.07
Senp3	chr11	69492540	69495389	-16.70	-0.32
Pelp1	chr11	70206472	70208499	-3.73	-0.21
Gemin4	chr11	76024102	76025507	-2.87	-0.32
Gemin4	chr11	76025895	76027390	-0.59	0.06
Utp6	chr11	79748406	79749421	-11.40	-0.43
Utp18	chr11	93746691	93747080	-8.52	-0.47
Cwc25	chr11	97608012	97614001	-1.06	-0.11
Eftud2	chr11	102699785	102700145	-3.75	-0.28
Ddx5	chr11	106643371	106643551	-60.70	0.67
Ddx5	chr11	106650118	106650179	-4.34	1.55
Nol11	chr11	107028583	107029061	-4.76	-0.46

Nol11	chr11	107050456	107050606	-0.91	-0.25
Prkca	chr11	107797639	107797700	-0.40	1.45
Prkca	chr11	107797789	107797850	-1.20	2.17
Srsf2	chr11	116714108	116714408	-50.00	-0.61
Csnk1d	chr11	120819778	120820048	-1.86	-0.20
Csnk1d	chr11	120823809	120824109	-0.87	-0.12
Csnk1d	chr11	120824767	120826288	-4.93	-0.25
Csnk1d	chr11	120844636	120852467	-0.33	-0.01
Srsf5	chr12	82046490	82046729	-16.60	-0.48
Srsf5	chr12	82050780	82051078	-44.00	-0.66
Psmc1	chr12	101348377	101351316	-1.50	-0.23
Cpsf2	chr12	103214183	103214244	-0.29	-0.03
Cpsf2	chr12	103240910	103241994	-0.60	-0.04
Papola	chr12	107022971	107023092	-4.18	-0.34
Papola	chr12	107072945	107075506	-2.32	-0.11
Zfp36l1	chr12	81209701	81210089	-4.76	0.64
Zfp36l1	chr12	81210446	81214000	-4.94	-0.27
Snw1	chr12	88791126	88791810	-8.21	-0.35
Akt1	chr12	113912217	113912487	-1.97	-0.56
Riok1	chr13	38128887	38129854	-38.40	2.21
Riok1	chr13	38152674	38153063	-1.09	0.22
Ddx46	chr13	55741151	55749105	-10.30	-0.37
Smn1	chr13	100894809	100896580	-0.66	-0.18
Nup153	chr13	46776258	46776559	-2.23	0.27
Nup153	chr13	46782040	46792068	-1.16	0.05
Nup153	chr13	46822738	46823218	-7.57	-0.49
Thoc3	chr13	54561210	54561569	-0.42	-0.04
Hnrnpa0	chr13	58228250	58229917	-61.50	-0.36
Hnrnpk	chr13	58501349	58504674	-3.06	-0.25
Tut7	chr13	59873418	59883679	-0.97	-0.08
Tut7	chr13	59900491	59902000	-0.99	-0.08
Tut7	chr13	59918167	59921566	-0.97	0.24
Tut7	chr13	59922850	59924388	-3.41	0.20
Utp15	chr13	99018475	99020812	-1.49	-0.11
Ppwd1	chr13	105010109	105010529	-1.01	0.16
Ppwd1	chr13	105018773	105018924	-0.32	-0.04
Rpp14	chr14	8922458	8922934	-0.56	-0.07
Dcp1a	chr14	31332515	31332666	-0.36	0.03
Dcp1a	chr14	31336008	31337397	-1.85	-0.18
Dcp1a	chr14	31339492	31339853	-0.38	-0.04
Psmc6	chr14	45949498	45950514	-0.60	-0.09
Prkcd	chr14	31408808	31411699	-1.85	-0.13
Prkcd	chr14	31411787	31411937	-0.73	0.22

Prkcd	chr14	31439273	31439394	-1.07	-0.33
Gnl3	chr14	31830581	31832216	-1.21	-0.12
Ppp2r2a	chr14	67634507	67635434	-1.32	-0.08
Gtf2f2	chr14	76297012	76306651	-1.19	-0.13
Wbp4	chr14	79861599	79863753	-7.89	-0.67
Dis3	chr14	99476389	99476599	-1.95	0.65
Polr2k	chr15	36103800	36104751	-0.34	0.08
Dcaf13	chr15	38944419	38944568	-1.48	-0.33
Exosc4	chr15	76157856	76159461	-2.29	-0.52
Exosc4	chr15	76159579	76160295	-13.70	-0.86
Nup50	chr15	84757805	84765822	-13.80	-0.39
Nup50	chr15	84770131	84771000	-9.31	-0.39
Nup50	chr15	84773033	84773303	-0.67	0.14
Eif4b	chr15	101925104	101925897	-0.89	-0.04
Ywhaz	chr15	36723285	36723376	-0.55	0.11
Puf60	chr15	75900611	75900909	-0.30	0.00
Bop1	chr15	76305282	76307699	-2.05	-0.27
Csnk1e	chr15	79248821	79248941	-1.61	-0.71
Csnk1e	chr15	79249180	79251297	-1.90	-0.22
Csnk1e	chr15	79252946	79254631	-6.43	-0.54
Csnk1e	chr15	79272227	79272487	-1.60	-1.22
Rrp7a	chr15	82946306	82948173	-33.50	-0.45
Poldip3	chr15	82958023	82959641	-1.64	-0.10
Ddx23	chr15	98475937	98476385	-4.86	-0.28
Ddx23	chr15	98488768	98493320	-11.90	-0.70
Psmd2	chr16	20651724	20652324	-20.60	-0.49
Eif4g1	chr16	20672821	20673965	-1.18	-0.11
Eif4g1	chr16	20674380	20675449	-1.23	-0.13
Eif4g1	chr16	20679335	20680055	-13.00	-0.18
Eif4g1	chr16	20689353	20692866	-16.70	-0.31
Ncbp2	chr16	31957240	31957480	-1.22	-0.23
Fyttd1	chr16	32877869	32884216	-2.17	-0.38
Fyttd1	chr16	32902616	32905812	-4.94	-0.62
Tra2b	chr16	22265852	22266002	-2.23	-0.43
Srrm2	chr17	23952780	23953201	-31.90	-0.37
Srrm2	chr17	23953650	23955539	-13.20	0.12
Srrm2	chr17	23957486	23958086	-12.30	0.23
Rnps1	chr17	24551619	24552074	-1.31	-0.33
Wdr46	chr17	34077667	34077865	-3.15	-0.57
Psmb8	chr17	34335288	34335468	-2.22	-0.33
Ddx39b	chr17	35378720	35378917	-1.75	-0.31
Ddx39b	chr17	35386162	35386790	-0.58	-0.04
Ddx39b	chr17	35566041	35566729	-1.02	-0.30

Dhx16	chr17	36019018	36019798	-2.95	-0.31
Wdr43	chr17	71965558	71965738	-0.68	-0.14
Wdr43	chr17	72006902	72007322	-2.27	-0.14
Wdr43	chr17	72008010	72008371	-0.96	-0.14
Gemin6	chr17	80625009	80627484	-1.24	-0.28
Wtap	chr17	13160233	13163945	-7.53	-0.30
Wtap	chr17	13167357	13168316	-0.54	-0.06
Thoc6	chr17	23810648	23810831	-0.28	0.01
Tbl3	chr17	24843091	24844598	-3.54	-0.72
Ppil1	chr17	29387927	29388668	-0.82	-0.12
Hnrnpm	chr17	33786662	33789227	-12.90	-0.29
Hnrnpm	chr17	33814217	33822403	-1.55	-0.10
Hspa1b	chr17	35093821	35096183	-6.05	0.37
Hspa1a	chr17	35106868	35108327	-2.67	3.71
Hspa1a	chr17	35108505	35108744	-1.59	6.01
Rpp21	chr17	36394428	36394791	-3.61	-0.75
Cdc5l	chr17	45528957	45530123	-2.27	0.27
Cdc5l	chr17	45564949	45570477	-1.04	-0.44
Rrp36	chr17	46804404	46807115	-5.59	-0.41
Rrp36	chr17	46809705	46811204	-0.28	0.00
Bysl	chr17	47738101	47738819	-4.50	-0.32
Bysl	chr17	47743878	47747963	-6.54	-0.51
Gtf2f1	chr17	57143372	57143559	-0.74	-0.13
Khsrp	chr17	57161754	57162725	-8.52	-0.27
Khsrp	chr17	57170601	57170840	-5.56	-0.55
Srsf7	chr17	80606178	80606645	-0.35	-0.03
Polr2d	chr18	31955877	31956355	-0.34	-0.02
Wdr33	chr18	31963710	31986988	-10.60	-0.69
Wdr33	chr18	32047426	32048936	-16.50	-0.52
Wdr33	chr18	32049204	32067113	-1.24	-0.09
Ercc3	chr18	32399984	32400372	-0.60	-0.12
Ercc3	chr18	32429502	32429682	-1.02	0.23
Dcp2	chr18	44572199	44578194	-0.47	0.03
Phax	chr18	56732636	56746951	-2.10	-0.09
Rnmt	chr18	68460038	68465572	-0.29	0.00
Txnl4a	chr18	80403957	80409392	-5.60	-0.13
Txnl4a	chr18	80415452	80419302	-1.93	-0.17
Txnl4a	chr18	80421035	80421843	-0.77	-0.27
Tmem223	chr19	8846695	8846965	-4.06	1.60
Cpsf7	chr19	10614133	10620757	-1.51	0.08
Cpsf7	chr19	10621625	10621806	-12.40	0.88
Prpf19	chr19	10969781	10969902	-0.74	-0.17
Rcl1	chr19	29217639	29218299	-1.62	-0.15

Cstf2t	chr19	31157390	31159177	-2.75	0.13
A1cf	chr19	32021886	32022036	-0.71	4.09
Pdcd11	chr19	47165255	47167320	-4.19	-0.76
Pdcd11	chr19	47186632	47191796	-23.00	-0.54
Pdcd11	chr19	47201789	47202487	-7.50	-0.72
Pdcd11	chr19	47202848	47202968	-6.18	-0.65
Pdcd11	chr19	47203460	47203610	-1.99	-0.97
Pdcd11	chr19	47204511	47205635	-4.75	-0.27
Sart1	chr19	5381212	5381273	-0.82	-0.17
Sart1	chr19	5382852	5383166	-1.19	-0.14
Sart1	chr19	5384663	5388703	-2.03	-0.15
Polr2g	chr19	8872986	8873047	-0.44	-0.14
Smndc1	chr19	53454959	53455080	-1.53	0.23
Gpkow	chrX	7285241	7285480	-4.10	0.65
Utp14a	chrX	45616799	45620147	-0.50	0.07
Utp14a	chrX	45626928	45630764	-1.77	-0.10
Hnrnph2	chrX	131137272	131140187	-17.40	0.37
Gemin8	chrX	162618314	162627493	-2.10	-0.70
Upf3b	chrX	34632248	34632639	-0.43	-0.05
Thoc2	chrX	39150625	39150955	-4.71	0.47
Las1l	chrX	93135774	93136221	-2.07	0.28
Zrsr2	chrX	160374663	160377244	-0.42	0.08
Zrsr2	chrX	160396267	160396568	-0.53	0.09

Innate immune system

gene_name	chr	chromStart	chromEnd	diff.lg.p	diff.log2.fc
Casp8	chr1	58890568	58901646	-12.80	-0.51
Abi2	chr1	60527626	60534244	-0.85	-0.13
Creb1	chr1	64622786	64644320	-11.60	0.58
Slc11a1	chr1	74421776	74422382	-2.22	-0.41
Slc11a1	chr1	74432026	74432625	-1.17	-0.10
Cab39	chr1	87690021	87714978	-6.91	-0.42
Cab39	chr1	87745760	87746448	-1.80	-0.10
Arl8a	chr1	137043410	137043590	-5.51	-0.63
Arpc5	chr1	154613671	154613968	-8.47	-0.32
Ncf2	chr1	154654984	154658055	-3.20	-0.20
Creg1	chr1	167693910	167693971	-1.14	-0.51
Fcgr4	chr1	172959296	172959476	-0.48	-0.13
Aim2	chr1	175389858	175395719	-0.61	0.03
Tmem63a	chr1	182904369	182904638	-2.93	-0.56
Tmbim1	chr1	74335180	74335271	-1.94	-0.39
Tmbim1	chr1	74341896	74350790	-7.20	-0.72
Bcl2	chr1	108438384	108439704	-0.91	0.22
Bcl2	chr1	108439763	108608408	-0.53	0.24

Bcl2	chr1	108608737	108610537	-2.43	-0.30
Bcl2	chr1	108610626	108610807	-0.63	-0.42
Ptpn4	chr1	121555568	121572143	-4.13	0.38
Ptpn4	chr1	121733557	121733648	-1.41	-1.02
Ikbke	chr1	133151178	133153534	-4.45	-0.31
Ikbke	chr1	133172403	133173518	-2.95	-0.52
Dhx9	chr1	155303276	155303397	-3.58	0.71
Qsox1	chr1	157626418	157628411	-6.98	-0.66
Fcgr2b	chr1	172890688	172891194	-2.80	0.13
Fcgr2b	chr1	172906140	172906200	-1.10	-0.38
Fcgr3	chr1	172981477	172981627	-0.50	-0.12
Ncstn	chr1	173996440	173996917	-4.50	-0.29
Degs1	chr1	184206857	184208760	-0.35	-0.01
Degs1	chr1	184209059	184209598	-0.40	0.01
Commd3	chr2	18596490	18597638	-0.74	-0.08
Abl1	chr2	31544075	31551290	-7.77	-0.43
Abl1	chr2	31653102	31658189	-1.51	-0.10
Abl1	chr2	31658367	31658428	-0.69	-0.40
Ptges2	chr2	32257786	32257964	-3.05	-0.79
Tank	chr2	61416642	61431195	-2.03	-0.57
Tank	chr2	61482412	61484290	-1.09	0.22
Tank	chr2	61484409	61488467	-4.44	-0.20
Tank	chr2	61491479	61491599	-1.20	-0.21
Itgav	chr2	83564583	83564973	-10.50	-1.02
Itgav	chr2	83643447	83643718	-3.67	-0.49
Itgav	chr2	83643807	83643987	-0.74	-0.16
Psmc3	chr2	90894439	90895182	-3.33	-0.20
Traf6	chr2	101530344	101537929	-2.95	-0.21
B2m	chr2	121973422	121973632	-11.70	-0.24
Sirpa	chr2	129418574	129418993	-0.37	-0.03
Sirpa	chr2	129419320	129457247	-2.42	-0.04
Mavs	chr2	131070939	131072532	-2.10	-0.25
Pygb	chr2	150654298	150656585	-0.41	-0.05
Hck	chr2	152934203	152934473	-1.97	0.48
Hck	chr2	152976907	152977028	-1.50	-0.25
Itch	chr2	154959216	154959307	-0.27	-0.02
Itch	chr2	155050102	155050403	-0.31	-0.01
Src	chr2	157295136	157296531	-2.35	0.70
Plcg1	chr2	160587552	160588233	-9.17	-1.31
Ctsa	chr2	164659257	164659646	-12.00	-0.34
Mmp9	chr2	164780329	164780718	-0.35	-0.13
Abi1	chr2	22895639	22895730	-1.77	-0.49
Tubb4b	chr2	25077825	25079655	-27.50	-0.32

Tubb4b	chr2	25079713	25080192	-0.32	0.02
Dpp7	chr2	25207809	25207987	-1.48	0.23
Card9	chr2	26212789	26213529	-2.20	-0.22
Agpat2	chr2	26449106	26450082	-0.80	-0.23
Surf4	chr2	26777259	26777678	-29.60	-0.44
Surf4	chr2	26782443	26788971	-5.03	-0.42
Vav2	chr2	27282104	27282345	-2.65	-1.29
Psmd5	chr2	34707993	34709823	-1.65	-0.19
Psmd5	chr2	34726274	34726482	-4.41	-0.82
Rab14	chr2	35056311	35056550	-6.69	-0.39
Psmb7	chr2	38498946	38499426	-3.13	-0.36
Wipf1	chr2	73270300	73270450	-1.18	-0.22
Wipf1	chr2	73273054	73275208	-4.10	-0.31
Wipf1	chr2	73275357	73278120	-11.00	-0.46
Wipf1	chr2	73282616	73291856	-0.37	-0.03
Atf2	chr2	73656947	73657068	-4.98	1.16
Serping1	chr2	84613462	84613523	-0.33	2.32
Ptprj	chr2	90273053	90274190	-33.00	-0.77
Ptprj	chr2	90420444	90420774	-1.91	-0.36
Cd44	chr2	102654086	102662407	-105.00	-0.64
Pin1rt1	chr2	104554491	104554756	-2.79	3.80
Atp8b4	chr2	126148384	126148594	-0.74	-0.12
Il1b	chr2	129190464	129191002	-3.00	1.94
Il1b	chr2	129195984	129196856	-1.12	2.89
Cd93	chr2	148263313	148263403	-0.46	2.58
Cd93	chr2	148266721	148267417	-0.45	1.49
Cd93	chr2	148268044	148268583	-0.86	3.61
Cd93	chr2	148268792	148269092	-0.67	2.88
Psmf1	chr2	151543111	151543202	-1.31	-0.87
Psmf1	chr2	151543649	151544038	-0.30	0.01
Bcl2l1	chr2	152606912	152607272	-0.96	0.13
Bcl2l1	chr2	152607660	152653984	-20.10	-0.61
Bcl2l1	chr2	152655121	152657418	-135.00	-0.91
Cpne1	chr2	155922188	155937671	-1.89	0.11
Dsn1	chr2	156820797	156820977	-0.82	0.77
Dsn1	chr2	156821482	156822471	-0.69	-0.19
Slpi	chr2	164181923	164182042	-77.90	-1.59
Elmo2	chr2	165115177	165116717	-4.35	-0.36
Elmo2	chr2	165117584	165119793	-0.30	0.03
Nfatc2	chr2	168301969	168303229	-1.59	-0.26
Nfatc2	chr2	168304128	168330396	-9.73	-0.53
Nfatc2	chr2	168396287	168427005	-6.54	-0.50
Ctsz	chr2	174252994	174254182	-128.00	-0.34

Fabp5	chr3	10014973	10015506	-2.36	-0.25
Pld1	chr3	28029285	28031356	-0.61	-0.13
Pld1	chr3	28031505	28031596	-0.48	-0.38
Pld1	chr3	28031774	28031985	-0.32	-0.11
Pik3ca	chr3	32335072	32335342	-3.17	-0.61
Pik3ca	chr3	32360651	32362022	-2.40	0.26
Rap2b	chr3	61168488	61170347	-6.56	0.26
Rap2b	chr3	61171125	61171336	-0.48	0.24
Shc1	chr3	89222472	89225623	-0.50	-0.10
Shc1	chr3	89225682	89226222	-15.40	-1.10
Shc1	chr3	89232244	89232484	-0.52	0.07
Ilf2	chr3	90291521	90291852	-2.02	-0.23
Ctss	chr3	95330707	95333423	-10.90	-0.35
Txnip	chr3	96362296	96362849	-20.70	0.38
Txnip	chr3	96363757	96364946	-72.50	0.32
Txnip	chr3	96365302	96365542	-18.10	0.47
Psma5	chr3	108059873	108060022	-0.64	3.39
Vav3	chr3	109143660	109143930	-1.24	-0.66
Ube2d3	chr3	135101656	135102228	-19.60	-0.75
Manba	chr3	135230676	135233888	-1.56	-0.17
Bcl10	chr3	145587232	145587592	-4.99	-0.42
Bcl10	chr3	145588099	145593556	-3.89	-0.41
Nhlrc3	chr3	53255917	53257551	-1.05	-0.16
Dhx36	chr3	62274511	62275295	-0.71	-0.15
Tlr2	chr3	83640193	83642691	-5.43	-0.11
Lamtor2	chr3	88353913	88356849	-9.31	-0.34
Psmb4	chr3	94690701	94690880	-8.50	-0.41
Psmd4	chr3	94836626	94836894	-2.54	-0.16
Psmd4	chr3	94840526	94846467	-8.21	-0.35
Fcgr1	chr3	96087130	96087191	-0.26	0.03
Fcgr1	chr3	96087728	96088506	-8.72	-0.57
Cd53	chr3	106563447	106563626	-3.51	-0.17
Nfkb1	chr3	135248247	135252299	-9.58	-0.45
Nfkb1	chr3	135354240	135354451	-1.35	0.77
Prkacb	chr3	146394694	146395023	-0.79	0.13
Lyn	chr4	3605267	3605506	-12.00	-0.40
Lyn	chr4	3716941	3718014	-40.00	-0.36
Map3k7	chr4	32051081	32051232	-3.02	-1.01
Map3k7	chr4	32104109	32107267	-5.97	0.38
Map3k7	chr4	32110051	32110231	-0.27	0.00
Atp6v1g1	chr4	63205828	63209679	-2.22	-0.30
Trim32	chr4	65274645	65274706	-0.48	-0.20
Trim32	chr4	65274854	65276169	-0.55	-0.05

Tlr4	chr4	66494965	66502995	-6.26	0.21
Pgm2	chr4	99602055	99602264	-0.67	-0.17
Pgm2	chr4	99656703	99659542	-3.27	-0.43
Ptafr	chr4	132119981	132136781	-1.25	0.09
Fgr	chr4	132530069	132540100	-0.30	0.03
Fgr	chr4	132556096	132556544	-10.20	-0.69
Fgr	chr4	132557289	132557558	-7.56	1.29
Wasf2	chr4	132751672	132752809	-0.40	0.01
Fuca1	chr4	135476640	135476908	-6.65	-0.76
Fuca1	chr4	135495027	135495325	-0.87	0.15
Ddost	chr4	137868076	137868376	-2.25	-0.24
Casp9	chr4	141352424	141352724	-6.06	-0.88
Casp9	chr4	141368153	141369672	-1.63	-0.61
Casp9	chr4	141369791	141369941	-0.24	-0.15
Masp2	chr4	147988211	147988362	-7.34	1.67
Slc2a5	chr4	149493913	149494152	-0.24	0.47
Ripk2	chr4	16050670	16051621	-4.17	-0.41
Ripk2	chr4	16090436	16090645	-16.00	-1.44
Ddx58	chr4	40152603	40155547	-1.90	-0.20
Ddx58	chr4	40186678	40186858	-0.44	0.12
B4galt1	chr4	40753786	40754830	-1.36	0.12
B4galt1	chr4	40770597	40800613	-1.85	0.25
B4galt1	chr4	40800671	40801001	-2.65	-0.48
Alad	chr4	62170203	62171204	-0.56	-0.14
Jun	chr4	94716292	94718913	-60.00	-0.27
Cap1	chr4	122536500	122536651	-58.00	-0.70
Lck	chr4	129225797	129226157	-0.39	0.06
Lck	chr4	129234249	129234661	-0.99	2.21
Rps6ka1	chr4	133403354	133404010	-3.84	-0.27
Cda	chr4	137894622	137894742	-1.22	1.10
Tnfrsf1b	chr4	144803977	144809673	-299.00	-1.07
Tnfrsf1b	chr4	144818975	144836773	-24.20	-1.34
Tnfrsf1b	chr4	144836594	144836774	-2.87	-0.59
Kcnab2	chr4	151766373	151767149	-1.53	0.12
Atad3a	chr4	155114748	155115286	-0.74	-0.07
Bst1	chr5	44210131	44210281	-0.40	-0.14
Cxcl3	chr5	91215188	91215370	-0.57	-0.91
Cxcl2	chr5	91332924	91333224	-434.00	-2.46
Stbd1	chr5	93034080	93034170	-0.19	-0.81
Hvcn1	chr5	122690237	122692276	-4.38	-0.49
P2rx7	chr5	123093919	123093980	-1.91	1.26
P2rx7	chr5	123130997	123132855	-5.21	0.20
P2rx7	chr5	123133124	123134024	-5.90	0.37

Psmd9	chr5	123678228	123678437	-0.34	0.03
Atp6v0a2	chr5	125200361	125202517	-2.97	0.32
Arpc1b	chr5	145875124	145883438	-11.60	-0.35
Tnip2	chr5	34842175	34842385	-0.32	-0.02
Tlr1	chr5	65316072	65316492	-1.44	0.54
Tlr1	chr5	65318106	65318495	-0.55	-0.20
Tlr6	chr5	65344333	65351228	-7.16	0.44
Atp8a1	chr5	68013273	68013813	-0.94	-0.18
Tec	chr5	73147051	73148633	-0.92	-0.13
Tec	chr5	73214871	73259687	-0.34	0.03
Mlec	chr5	115597834	115600228	-0.61	-0.05
Mlec	chr5	115600898	115608185	-12.50	-0.43
Ptpn11	chr5	121581827	121582307	-1.21	0.16
Ptpn11	chr5	121583292	121587519	-0.40	0.02
Rhof	chr5	123553559	123554127	-8.81	2.11
Rhof	chr5	123554185	123554246	-2.05	2.49
Rhof	chr5	123567437	123567796	-0.54	-0.19
Rhof	chr5	123568153	123582399	-35.90	-0.59
Ubc	chr5	125866453	125867671	-172.00	0.73
Ubc	chr5	125867938	125868147	-3.15	0.20
Ubc	chr5	125868235	125870298	-22.80	-0.40
Slc15a4	chr5	128076540	128080872	-0.95	-0.12
Gusb	chr5	130464890	130465519	-1.85	-0.08
Gusb	chr5	130478517	130478698	-10.40	-0.43
Ncf1	chr5	134697302	134697663	-2.36	-0.12
Lat2	chr5	135076494	135078531	-7.71	-0.25
Trim56	chr5	137587155	137588276	-8.63	-0.39
Trim56	chr5	137588605	137592077	-46.30	-0.54
Card11	chr5	141348952	141352360	-0.69	0.26
Rac1	chr5	144288681	144288861	-1.21	-0.22
Pdap1	chr5	145892293	145900958	-14.40	-0.32
Cul1	chr6	47404345	47445165	-3.56	0.31
Cul1	chr6	47474617	47476108	-1.64	-0.15
Irak2	chr6	113640953	113644480	-29.40	-0.65
Clec4d	chr6	123212154	123212214	-0.48	0.14
C1ra	chr6	124472382	124472951	-1.63	-0.40
Lrmp	chr6	145086263	145086444	-0.29	-0.02
Impdh1	chr6	29159242	29166081	-0.86	-0.16
Clec5a	chr6	40527283	40529441	-4.88	-0.13
Nod1	chr6	54874531	54874682	-0.75	1.04
Nod1	chr6	54893168	54893616	-0.68	0.56
Nod1	chr6	54893675	54894034	-0.75	0.80
Nod1	chr6	54894242	54894451	-0.24	-0.71

Nod1	chr6	54894600	54897981	-1.54	1.88
Vamp8	chr6	72335214	72335448	-3.83	-0.35
Vamp8	chr6	72338374	72340603	-0.85	-0.14
Kcmf1	chr6	72849792	72849973	-0.26	-0.02
Rbsn	chr6	92139135	92140425	-0.94	0.10
Uba3	chr6	97134556	97134676	-1.20	0.47
Raf1	chr6	115568620	115569280	-0.58	0.06
Alox5	chr6	116411016	116411196	-1.91	-0.74
Atp6v1e1	chr6	120768397	120772703	-5.89	-0.47
Slc2a3	chr6	122679711	122680131	-3.19	-0.53
C3ar1	chr6	122799485	122806115	-3.47	-0.14
C1s2	chr6	124575599	124575809	-0.29	1.36
Ptpn6	chr6	124683045	124688519	-0.41	-0.07
Olr1	chr6	129437034	129437784	-0.56	2.18
Kras	chr6	145168813	145168964	-1.16	-0.35
Plaur	chr7	25247518	25251694	-110.00	-1.93
Plaur	chr7	25260294	25260713	-183.00	-1.49
Irf3	chr7	52253029	52253179	-1.59	-0.48
Irf3	chr7	52257097	52257801	-2.81	-0.37
Irf3	chr7	52257979	52258099	-1.43	-1.19
Cyfip1	chr7	63097610	63127258	-0.50	0.06
Ctsc	chr7	95437245	95437485	-0.96	0.45
Ctsc	chr7	95458037	95458547	-0.94	0.12
Prcp	chr7	100081624	100082283	-0.57	-0.08
Ndufc2	chr7	104548571	104548690	-0.43	0.05
Lamtor1	chr7	109054380	109058241	-0.87	-0.19
Lamtor1	chr7	109059942	109060091	-16.60	-0.68
Ampd3	chr7	117954502	117954712	-3.65	0.61
Itgam	chr7	135270386	135270776	-5.15	0.99
Itgax	chr7	135292687	135294081	-2.59	1.26
Lair1	chr7	3960550	3960699	-0.30	0.16
Lair1	chr7	3961056	3961295	-0.66	-0.33
Ube2m	chr7	13623018	13623619	-6.02	-0.45
C5ar2	chr7	16822655	16823045	-0.48	0.50
C5ar2	chr7	16823134	16823315	-0.25	-0.26
C5ar1	chr7	16833166	16844566	-69.90	-0.77
Calm3	chr7	17502039	17502309	-1.46	-0.09
Relb	chr7	20191690	20192229	-0.83	-0.11
Ceacam1	chr7	26247739	26248638	-4.67	0.32
Ceacam1	chr7	26257014	26258919	-1.16	-0.37
Ceacam1	chr7	26259008	26274241	-0.36	-0.02
Nfkbib	chr7	29546684	29546864	-25.70	-0.94
Nfkbib	chr7	29547132	29551543	-9.87	-0.61

Psmd8	chr7	29965393	29965573	-13.80	-0.43
Gpi1	chr7	34987182	34987689	-15.30	-0.25
Siglece	chr7	50906826	50906947	-0.53	0.45
Siglece	chr7	50914669	50914730	-0.37	-0.51
Ftl1	chr7	52713604	52713914	-25.90	-0.25
Ftl1	chr7	52715051	52715227	-434.00	-0.45
Anpep	chr7	86966748	86966897	-1.17	-0.22
Anpep	chr7	86986085	86993080	-7.52	-0.20
Iqgap1	chr7	87857938	87858329	-21.50	-0.33
Iqgap1	chr7	87944725	87948217	-28.40	-0.56
Rhog	chr7	109387636	109398632	-32.90	-0.30
Trim21	chr7	109707151	109710434	-1.56	0.22
Aldoa	chr7	133943900	133943961	-0.20	0.10
Adam8	chr7	147164929	147165378	-47.80	-0.66
Hras	chr7	148376788	148378177	-0.34	-0.01
Hras	chr7	148378775	148379903	-0.71	0.21
Tollip	chr7	149069998	149076626	-5.15	-0.28
Ctsd	chr7	149561820	149562294	-118.00	-0.34
Fadd	chr7	151765572	151768192	-4.39	-0.39
Map2k7	chr8	4245546	4246251	-0.79	0.09
Atp11a	chr8	12757045	12757285	-0.28	0.04
Atp11a	chr8	12864987	12866484	-1.11	0.12
Lamp1	chr8	13159223	13159402	-12.50	-0.58
Plpp5	chr8	26834551	26835000	-0.42	0.03
Ppp2cb	chr8	34710092	34710359	-0.73	-0.37
Dusp4	chr8	35870663	35882259	-130.00	-0.63
Rab3a	chr8	73282404	73282465	-0.44	0.34
Myo9b	chr8	73796612	73839032	-3.45	-0.17
Myo9b	chr8	73871704	73876118	-7.70	-0.21
Myo9b	chr8	73880532	73884611	-0.34	0.00
Prkaca	chr8	86496876	86500855	-1.59	-0.45
Man2b1	chr8	87607167	87607377	-1.33	0.29
Cyld	chr8	91220956	91226218	-0.50	0.37
Cyld	chr8	91226307	91233793	-2.31	0.21
Nfatc3	chr8	108602601	108603618	-3.27	0.21
Nfatc3	chr8	108632435	108652196	-8.48	0.41
Plcg2	chr8	120022190	120028105	-0.35	-0.03
Ikbkb	chr8	23770606	23771027	-1.32	-0.20
Golga7	chr8	24352876	24353027	-5.60	-0.49
Hgsnat	chr8	27054960	27055615	-2.49	0.23
Hgsnat	chr8	27083350	27087156	-0.93	-0.21
Tlr3	chr8	46482187	46484515	-6.15	0.91
Tlr3	chr8	46488157	46488217	-1.04	1.33

Pik3r2	chr8	73292079	73292528	-7.07	-0.81
Pik3r2	chr8	73296811	73300581	-13.40	-0.87
Adgre5	chr8	86247268	86247657	-11.60	-1.46
Psmb10	chr8	108461631	108461967	-2.80	-0.63
Psmd7	chr8	110104338	110108077	-4.03	0.12
Hp	chr8	112099087	112099805	-0.32	-0.05
Ist1	chr8	112195789	112196630	-0.63	0.04
Cotl1	chr8	122364299	122364449	-0.56	-0.07
Galns	chr8	125102404	125103030	-5.07	-0.53
Casp1	chr9	5298635	5299458	-1.15	-0.21
Pin1	chr9	20456573	20459918	-1.39	-0.27
Slc44a2	chr9	21156935	21159322	-1.03	0.07
Dnm2	chr9	21310134	21311379	-2.43	0.16
Dnm2	chr9	21311898	21312168	-2.03	0.27
Ppp2r1b	chr9	50687990	50688710	-0.71	-0.09
Ppp2r1b	chr9	50701016	50701675	-4.03	-0.60
Adam10	chr9	70526807	70526958	-1.07	0.28
Adam10	chr9	70626086	70626297	-2.80	0.46
Rab27a	chr9	72892731	72908786	-0.39	-0.66
Rab27a	chr9	72943089	72943780	-0.59	0.27
Myo5a	chr9	74919012	74919403	-0.86	0.18
Myo5a	chr9	75065709	75066549	-8.29	0.29
Pik3r4	chr9	105545624	105545744	-0.46	-0.22
Pik3r4	chr9	105546492	105551238	-0.65	0.07
Pik3r4	chr9	105552647	105556359	-1.62	-0.23
Pik3r4	chr9	105589445	105589955	-0.34	-0.02
Tlr9	chr9	106124928	106129177	-69.30	1.15
Dusp7	chr9	106270962	106271201	-3.92	-1.70
Dusp7	chr9	106271527	106276865	-1.31	-0.10
Glb1	chr9	114310242	114326114	-1.32	0.33
Glb1	chr9	114368800	114373509	-0.47	-0.05
Glb1	chr9	114374377	114383495	-4.81	0.37
Dync1li1	chr9	114632686	114632895	-0.93	0.16
Dync1li1	chr9	114632812	114633189	-1.24	0.13
Ctnnb1	chr9	120842577	120859669	-0.34	-0.01
Ctnnb1	chr9	120867712	120868847	-5.68	0.15
Ccr2	chr9	124021097	124021367	-0.45	-0.18
Birc2	chr9	7818614	7818734	-0.55	-0.29
Birc2	chr9	7833610	7834657	-0.90	0.13
Birc2	chr9	7834955	7835195	-1.05	0.42
Birc3	chr9	7848969	7850928	-1.17	-0.17
Birc3	chr9	7860311	7872961	-0.30	0.00
Panx1	chr9	14810228	14812409	-2.17	-0.38

Rab3d	chr9	21714741	21715042	-1.16	-0.47
Tirap	chr9	34994065	34994215	-0.38	-0.10
Tirap	chr9	34994423	34996823	-0.38	0.02
Nlrx1	chr9	44060795	44061574	-1.60	-0.43
Nlrx1	chr9	44070526	44072299	-3.29	-0.49
Nlrx1	chr9	44076107	44076168	-0.85	1.38
Pafah1b2	chr9	45776653	45777013	-2.67	0.20
Chrnb4	chr9	54882486	54883206	-4.11	2.77
Tmem30a	chr9	79617046	79617405	-0.64	-0.07
Pik3cb	chr9	98939628	98941447	-5.88	0.37
Armc8	chr9	99380819	99381090	-0.78	-0.26
Armc8	chr9	99420641	99420852	-1.07	0.54
Nck1	chr9	100395659	100407191	-0.86	0.08
Dnajc13	chr9	104054165	104054644	-6.55	0.41
Apeh	chr9	107987744	107987894	-0.71	-0.29
Myd88	chr9	119248978	119249158	-3.52	-0.60
Fuca2	chr10	13223007	13225667	-1.77	-0.36
Fuca2	chr10	13234515	13235470	-1.15	0.10
Frk	chr10	34203714	34203924	-0.23	-0.42
Fyn	chr10	39089604	39089994	-0.68	-0.12
Fyn	chr10	39271197	39284102	-0.31	0.00
Lilr4b	chr10	51200478	51200715	-8.29	-0.49
Lilr4b	chr10	51205082	51205501	-195.00	-1.21
Psap	chr10	59740405	59754400	-4.91	-0.38
Itgb2	chr10	76993092	77004744	-8.05	-0.66
Cdc34	chr10	79144939	79145322	-5.03	-0.46
Cdc34	chr10	79148043	79151143	-18.90	-0.38
Arhgap45	chr10	79479416	79480842	-6.48	-0.36
Arhgap45	chr10	79485025	79488219	-0.85	-0.08
Arhgap45	chr10	79493111	79494216	-1.79	-0.12
Eef2	chr10	80644273	80645254	-24.30	-0.11
Eea1	chr10	95473536	95474748	-15.40	-0.60
Eea1	chr10	95488798	95489514	-17.80	-0.66
Eea1	chr10	95499355	95500064	-0.36	0.03
Eea1	chr10	95502273	95503004	-2.73	-0.24
Eea1	chr10	95504160	95504914	-0.43	0.04
Dusp6	chr10	98725864	98729379	-0.51	-0.02
Gns	chr10	120802145	120802325	-0.30	-0.01
Gns	chr10	120832206	120832776	-1.28	0.07
Gns	chr10	120833971	120834301	-5.31	0.31
Stat6	chr10	127080041	127080222	-2.32	-0.33
Stat6	chr10	127096737	127097564	-4.04	-0.16
Tab2	chr10	7625983	7626463	-1.79	0.18

Tab2	chr10	7626701	7630910	-3.35	-0.17
Tab2	chr10	7631204	7644574	-1.97	-0.07
Tnfaip3	chr10	18721581	18725137	-0.67	-0.06
Ube2d1	chr10	70718144	70718592	-2.63	0.38
Trpm2	chr10	77372533	77372804	-0.19	0.13
Pfkl	chr10	77450802	77450893	-0.32	-0.01
Ckap4	chr10	83990030	83991369	-1.40	-0.09
Ckap4	chr10	83996275	83996633	-3.93	-0.77
Glipr1	chr10	111434202	111434291	-1.57	0.44
Cand1	chr10	118647900	118648919	-0.49	0.03
Dbnl	chr11	5688485	5691845	-3.17	-0.48
Dbnl	chr11	5699963	5700503	-8.04	-0.43
Peli1	chr11	20991326	20991536	-0.58	0.15
Peli1	chr11	21047425	21048774	-11.20	-0.48
Psme4	chr11	30671774	30672135	-0.54	-0.18
Stk10	chr11	32433265	32433356	-0.44	0.16
Stk10	chr11	32489371	32503751	-45.30	-0.58
Stk10	chr11	32504289	32512777	-5.35	-0.46
Stk10	chr11	32517862	32522951	-27.20	-0.58
Fbxw11	chr11	32644536	32644986	-0.78	-0.08
Mapk9	chr11	49696697	49697026	-1.80	0.29
Ppp2ca	chr11	51912325	51912474	-4.51	-0.88
Ppp2ca	chr11	51935449	51935836	-0.34	0.00
Gm2a	chr11	54923031	54923240	-0.86	0.16
2310033P09Rik	chr11	59021980	59022040	-1.19	0.50
2310033P09Rik	chr11	59022524	59023941	-1.13	0.17
Nlrp3	chr11	59355086	59365364	-68.60	-0.49
Nlrp3	chr11	59379889	59380219	-0.60	0.09
Map2k3	chr11	60745558	60745738	-0.87	-0.13
Map2k3	chr11	60755435	60757081	-0.31	0.00
Map2k3	chr11	60765468	60766215	-11.10	-0.38
Pld2	chr11	70371013	70371612	-0.52	-0.09
Crk	chr11	75492811	75517946	-16.70	-0.28
Crk	chr11	75518934	75519414	-1.35	-0.15
Psmd3	chr11	98543867	98546829	-30.30	-0.74
Wipf2	chr11	98725061	98761613	-12.30	-0.40
Atp6v0a1	chr11	100923714	100924043	-0.91	-0.13
Psme3	chr11	101177564	101178336	-0.62	0.07
Psme3	chr11	101182070	101183288	-0.64	-0.06
Grn	chr11	102291725	102294419	-31.60	-0.59
Grn	chr11	102297674	102297824	-14.10	-0.23
Gaa	chr11	119129310	119131951	-7.63	-0.33
Baiap2	chr11	119867856	119868096	-3.26	-0.42

Nf2	chr11	4749020	4749260	-1.15	-0.24
Dock2	chr11	34127072	34128632	-0.68	0.05
Dock2	chr11	34546949	34597325	-2.05	-0.22
Cyfip2	chr11	46050850	46051031	-0.34	1.17
Cyfip2	chr11	46051479	46051569	-0.17	-0.26
Atox1	chr11	55264107	55274640	-3.02	-0.32
Mapk7	chr11	61303417	61306635	-16.60	-0.51
Mapk7	chr11	61306963	61307441	-3.11	-0.52
Map2k4	chr11	65503265	65506970	-4.86	-0.31
Nlrp1b	chr11	70966603	70969541	-2.25	-0.41
Nlrp1b	chr11	70995132	70995372	-0.40	-0.12
Nlrp1b	chr11	70996028	70996268	-1.04	-0.49
Kpnb1	chr11	97023572	97023813	-3.10	-0.19
Kpnb1	chr11	97048995	97049206	-2.34	-0.30
Ormdl3	chr11	98443440	98443619	-1.16	0.44
Ormdl3	chr11	98443767	98444154	-2.37	-0.45
Ghdc	chr11	100630436	100630733	-0.74	-0.25
Dusp3	chr11	101834513	101835259	-0.43	0.02
Dusp3	chr11	101835527	101835885	-0.41	0.02
Dusp3	chr11	101835944	101836213	-0.85	-0.10
Map3k14	chr11	103081077	103082247	-3.53	-0.47
Map3k14	chr11	103100346	103101055	-0.68	-0.14
Map3k14	chr11	103103466	103128715	-0.39	-0.09
Icam2	chr11	106239145	106239263	-0.38	0.20
Cd300lb	chr11	114784364	114784935	-3.81	-0.24
Cd300lb	chr11	114784994	114795700	-23.20	-0.42
Cd300e	chr11	114914339	114914460	-0.23	0.05
Grb2	chr11	115505517	115505906	-1.13	0.17
Grb2	chr11	115506591	115507248	-1.12	-0.07
Tmc6	chr11	117627298	117628154	-2.06	-0.44
Cant1	chr11	118268378	118271504	-1.27	-0.10
Cant1	chr11	118272131	118279176	-4.34	-0.34
Psen1	chr12	85072679	85075730	-7.32	-0.20
Fos	chr12	86814850	86817711	-87.60	-0.74
Psmc1	chr12	101348377	101351316	-1.50	-0.23
Calm1	chr12	101437750	101440606	-16.30	-0.45
Calm1	chr12	101445239	101445449	-3.24	-0.14
Dync1h1	chr12	111839634	111839815	-0.41	-0.05
Dync1h1	chr12	111855934	111857506	-0.43	-0.01
Dync1h1	chr12	111904592	111905154	-1.97	-0.13
Traf3	chr12	112498880	112500724	-1.28	0.14
Pld4	chr12	113998865	113999998	-0.67	0.14
Pld4	chr12	114000237	114001663	-1.26	-0.24

Pld4	chr12	114006322	114007047	-12.40	-0.38
Colec11	chr12	29279478	29279597	-0.39	1.91
Nfkbia	chr12	56593395	56593604	-103.00	-1.36
Atp6v1d	chr12	79962444	79962625	-1.13	0.27
Npc2	chr12	86097961	86098142	-28.50	-0.41
Rps6ka5	chr12	101789211	101791305	-0.71	0.25
Lgmn	chr12	103651753	103677907	-27.90	-0.55
Hsp90aa1	chr12	111933947	111934546	-6.23	2.48
Elmo1	chr13	20684291	20697406	-1.26	0.14
Ripk1	chr13	34094265	34094762	-9.02	-0.95
Ripk1	chr13	34119794	34125274	-0.32	0.00
Ripk1	chr13	34125393	34126082	-9.17	-0.86
Dsp	chr13	38283950	38285120	-2.85	3.39
Dsp	chr13	38285209	38285510	-0.30	-0.82
Dsp	chr13	38286818	38287269	-5.14	3.79
Dsp	chr13	38287568	38288078	-1.88	2.14
Dsp	chr13	38288227	38288738	-0.92	2.25
Dsp	chr13	38288946	38289187	-0.63	3.09
Syk	chr13	52678805	52706632	-13.30	-0.48
Syk	chr13	52727069	52733575	-2.37	-0.22
Syk	chr13	52737300	52738605	-0.64	0.10
Syk	chr13	52738964	52742902	-5.19	-0.18
Syk	chr13	52743770	52743981	-0.44	0.06
Faf2	chr13	54723144	54730781	-0.85	0.35
Faf2	chr13	54757693	54763748	-1.55	-0.12
Lpcat1	chr13	73651326	73651986	-0.31	-0.02
Arsb	chr13	94710665	94710756	-0.36	-0.06
Cd180	chr13	103494954	103496711	-2.85	0.12
Cdk13	chr13	17809653	17811567	-1.53	0.10
Cdk13	chr13	17858358	17896931	-3.76	-0.17
Txndc5	chr13	38592141	38592649	-2.68	0.19
Txndc5	chr13	38593006	38595355	-10.40	-0.27
Txndc5	chr13	38620124	38620304	-1.02	0.19
Hk3	chr13	55107346	55107674	-8.50	-0.40
Rab24	chr13	55421264	55421501	-3.81	-0.46
Rab24	chr13	55422955	55423341	-4.54	-0.47
Dok3	chr13	55624595	55625457	-4.45	-0.17
Dok3	chr13	55628729	55629086	-0.69	0.07
Dok3	chr13	55629708	55629917	-4.20	-0.81
Ddx41	chr13	55637366	55637930	-0.75	0.12
Ctsl	chr13	64470393	64471614	-17.40	-0.32
Scamp1	chr13	94973800	94978130	-0.65	0.12
Pik3r1	chr13	102453986	102456264	-3.37	0.30

Pik3r1	chr13	102478932	102525656	-0.37	-0.05
Pik3r1	chr13	102527244	102538172	-0.72	0.07
Map3k1	chr13	112538703	112540665	-0.67	0.09
Map3k1	chr13	112545624	112546521	-2.16	-0.18
Map3k1	chr13	112566138	112599191	-3.70	-0.70
Nkiras1	chr14	19111014	19112745	-0.47	-0.11
Plau	chr14	21661560	21662161	-4.06	-0.22
Vcl	chr14	21850890	21851310	-6.08	0.44
Psmc6	chr14	45949498	45950514	-0.60	-0.09
Pnp	chr14	51564155	51567533	-1.16	0.41
Pnp	chr14	51571126	51571721	-7.21	-0.33
Ctsb	chr14	63761159	63761760	-164.00	-0.25
Clu	chr14	66587437	66590689	-4.00	-0.56
Ppp3cb	chr14	21320302	21327131	-0.50	-0.04
Ppp3cb	chr14	21365614	21365735	-0.90	-0.41
Prkcd	chr14	31408808	31411699	-1.85	-0.13
Prkcd	chr14	31411787	31411937	-0.73	0.22
Prkcd	chr14	31439273	31439394	-1.07	-0.33
Tspan14	chr14	41720841	41721322	-8.13	-0.95
Ripk3	chr14	56403831	56404011	-3.56	0.89
Myo10	chr15	25552304	25552784	-84.80	-1.10
Myo10	chr15	25709521	25712766	-107.00	-0.68
Myo10	chr15	25736823	25740210	-170.00	-1.17
Myo10	chr15	25740299	25742438	-33.40	-0.63
Myo10	chr15	25743096	25743426	-6.82	-0.51
Ncf4	chr15	78092626	78092951	-7.16	-0.30
Tab1	chr15	79989230	79992132	-13.10	-0.63
Ep300	chr15	81416643	81438767	-12.50	-0.29
Ep300	chr15	81479273	81481819	-5.01	-0.13
Xrcc6	chr15	81870214	81870424	-0.38	-0.05
Ano6	chr15	95621303	95661131	-0.52	-0.10
Ano6	chr15	95802935	95805002	-3.51	0.15
Atf1	chr15	100084825	100090754	-1.03	0.09
Mettl7a1	chr15	100143523	100143732	-0.46	0.33
Nckap1l	chr15	103328091	103328272	-7.79	-0.50
Ptk2	chr15	73035594	73039802	-1.03	-0.09
Dgat1	chr15	76342128	76342248	-1.28	-0.41
Myh9	chr15	77592006	77593541	-15.60	-0.23
Myh9	chr15	77594338	77606283	-281.00	-0.43
Myh9	chr15	77643609	77672545	-2.53	-0.16
Pla2g6	chr15	79116836	79117254	-2.27	-0.40
Nfam1	chr15	82828225	82828854	-0.48	-0.04
Nfam1	chr15	82829422	82831880	-7.67	-0.36

Arsa	chr15	89303025	89303862	-0.98	-0.16
Arsa	chr15	89305988	89306615	-6.67	-0.79
Gpr84	chr15	103138665	103140869	-8.09	-0.21
Hmox2	chr16	4764613	4765838	-8.41	-0.34
Mapk1	chr16	17038671	17038882	-0.49	0.07
Snap29	chr16	17427202	17428532	-2.76	0.33
Psmd2	chr16	20651724	20652324	-20.60	-0.49
Cd47	chr16	49855915	49868147	-3.21	0.29
Crebbp	chr16	4084047	4087773	-0.48	-0.02
Crebbp	chr16	4154961	4213404	-3.27	-0.16
Cpped1	chr16	11804560	11827393	-0.86	0.10
Cpped1	chr16	11887837	11909516	-2.45	-0.79
Pak2	chr16	32019862	32021909	-0.84	0.05
App	chr16	85025115	85043833	-16.90	-0.31
App	chr16	85120907	85173892	-5.77	-0.46
Rps6ka2	chr17	7374493	7374614	-0.54	0.42
Rps6ka2	chr17	7506884	7507544	-0.43	0.14
1600012H06Rik	chr17	15080218	15081387	-18.40	-0.69
Mapk14	chr17	28828316	28828704	-3.84	-0.61
Mapk14	chr17	28884007	28885350	-1.27	0.08
Psmb8	chr17	34335288	34335468	-2.22	-0.33
Neu1	chr17	35071556	35072840	-6.58	-0.28
H2-D1	chr17	35400038	35400558	-64.10	-0.68
H2-D1	chr17	35400926	35402744	-4.71	-0.33
H2-D1	chr17	35403816	35404204	-71.80	-0.36
H2-Q2	chr17	35479307	35479570	-0.14	0.42
H2-Q6	chr17	35561821	35562745	-5.87	-0.37
H2-Q6	chr17	35565949	35566720	-1.19	-0.34
H2-Q7	chr17	35576925	35577015	-0.36	-0.83
H2-Q10	chr17	35607880	35610039	-0.46	-0.18
Trem1	chr17	48371921	48385080	-1.75	0.78
Vav1	chr17	57418522	57418613	-0.31	0.06
Vav1	chr17	57467106	57467554	-1.28	0.13
Ypel5	chr17	73198557	73198888	-0.87	-0.22
Prkce	chr17	86567124	86568262	-0.54	0.09
Prkce	chr17	87053997	87055374	-0.74	-0.16
Dynlt1f	chr17	6306339	6306549	-0.61	0.48
Rnaset2b	chr17	8338409	8340697	-14.10	-0.91
Igf2r	chr17	12876409	12876980	-0.50	-0.04
Igf2r	chr17	12962451	12962572	-0.53	0.18
Fpr1	chr17	18013552	18013876	-0.16	-0.22
Fpr1	chr17	18014139	18020903	-0.38	1.24
Mmp25	chr17	23781787	23781878	-0.56	3.46

Mmp25	chr17	23782026	23782236	-0.61	2.96
Pdpk1	chr17	24215553	24216093	-11.80	0.63
Atp6v0c	chr17	24306225	24306374	-1.17	-0.23
H2-K1	chr17	34133105	34133495	-21.60	-0.51
H2-K1	chr17	34136728	34137188	-34.60	-0.71
Hspa1b	chr17	35093821	35096183	-6.05	0.37
Hspa1a	chr17	35106868	35108327	-2.67	3.71
Hspa1a	chr17	35108505	35108744	-1.59	6.01
Tubb5	chr17	35972171	35972588	-48.90	-0.35
H2-T23	chr17	36169189	36248130	-6.88	-0.55
Enpp4	chr17	44234607	44235267	-0.53	0.24
Enpp4	chr17	44236165	44242757	-6.51	1.00
Ppp2r5d	chr17	46820058	46820862	-0.90	-0.11
Ppp2r5d	chr17	46837029	46841951	-0.53	-0.11
Cnpy3	chr17	46872839	46874785	-3.02	-0.19
Ticam1	chr17	56408914	56416190	-37.60	-0.63
Vapa	chr17	65962685	65962865	-2.72	-0.27
Rab31	chr17	66001065	66001632	-0.68	-0.05
Rab31	chr17	66002078	66002287	-2.81	-0.26
Rab31	chr17	66003418	66017257	-2.20	0.14
Rab31	chr17	66122031	66122092	-0.25	-0.17
Sos1	chr17	80797100	80799127	-0.43	-0.03
Sos1	chr17	80833039	80834290	-0.90	-0.18
Sos1	chr17	80879343	80879554	-1.15	-0.89
Calm2	chr17	87835011	87846216	-2.03	-0.14
Ube2d2a	chr18	35931242	35931392	-3.18	-0.65
Ube2d2a	chr18	35965479	35965570	-0.43	0.27
Malt1	chr18	65635658	65636018	-2.72	-0.30
Map3k8	chr18	4348899	4352951	-3.59	-0.54
Rock1	chr18	10181251	10181790	-9.40	-0.56
Tmem173	chr18	35893809	35894258	-4.25	-0.40
Cd14	chr18	36884720	36886308	-168.00	-0.28
Ticam2	chr18	46718782	46719052	-0.87	-0.33
Ticam2	chr18	46719411	46732457	-0.64	0.08
Nfatc1	chr18	80802973	80803394	-0.79	-0.30
Nfatc1	chr18	80803872	80832717	-0.41	0.03
Nfatc1	chr18	80846232	80846653	-2.71	0.51
Nfatc1	chr18	80879052	80904894	-2.97	0.23
Unc93b1	chr19	3935215	3935306	-3.82	-0.42
Unc93b1	chr19	3936467	3936707	-9.44	-0.66
Unc93b1	chr19	3948801	3948951	-25.80	-0.61
Rela	chr19	5646968	5647773	-3.54	-0.20
Pgam1	chr19	41986508	41990163	-6.96	-1.07

Btrc	chr19	45604117	45604298	-0.65	1.17
Nfkb2	chr19	46379436	46380290	-0.39	0.13
Tcirg1	chr19	3906889	3906949	-2.38	-0.62
Peli3	chr19	4932033	4932629	-0.24	0.04
Dtx4	chr19	12543910	12544151	-8.93	-0.57
Dtx4	chr19	12560929	12576605	-3.81	-0.14
Chuk	chr19	44181817	44181937	-0.55	-0.16
Ddx3x	chrX	12858147	12861347	-0.41	0.02
Ddx3x	chrX	12868399	12868809	-48.00	0.69
Pgrmc1	chrX	34138219	34144830	-27.20	0.84
Pgrmc1	chrX	34145392	34145600	-79.00	2.46
Tab3	chrX	82859477	82860195	-4.67	0.57
Tab3	chrX	82860522	82860673	-1.01	0.46
Tab3	chrX	82873570	82876044	-16.40	2.20
Atp7a	chrX	103222674	103280870	-3.97	0.27
Atp7a	chrX	103319725	103319995	-12.90	0.99
Huwe1	chrX	148353194	148354606	-2.14	0.09
Was	chrX	7667496	7667617	-0.70	0.20
Rap2c	chrX	48359709	48370808	-2.49	0.22
Rap2c	chrX	48370867	48371195	-0.35	-0.02
Irak1	chrX	71260386	71260835	-0.46	0.04
Gla	chrX	131124431	131124610	-68.60	1.61
Gla	chrX	131135394	131135514	-0.61	0.21
Tlr8	chrX	163680663	163681230	-0.44	0.52
Tlr8	chrX	163681497	163681647	-0.61	1.67
Tlr8	chrX	163681944	163682362	-0.65	0.76
Tlr8	chrX	163683254	163683672	-3.07	3.59
Tlr7	chrX	163742862	163745310	-7.00	0.16
Tlr7	chrX	163745428	163745549	-4.15	0.44
Tlr7	chrX	163745727	163746354	-0.94	0.06
Diaph1	chr18	38004807	38005077	-2.74	-0.30
Diaph1	chr18	38094885	38095065	-2.39	-0.61

Cytokine signaling in immune system

gene_name	chr	chromStart	chromEnd	diff.lg.p	diff.log2.fc
Il1r1	chr1	40370414	40370475	-0.14	0.42
Il1rl1	chr1	40518613	40519152	-1.16	-0.29
Il1rl1	chr1	40521541	40521602	-0.61	0.36
Il18rap	chr1	40605544	40606142	-0.97	-0.28
Stat1	chr1	52216188	52216729	-5.25	-0.42
Creb1	chr1	64622786	64644320	-11.60	0.58
Eif4e2	chr1	89110488	89110878	-2.76	-0.47

Eif4e2	chr1	89134456	89135775	-1.77	0.21
Inpp5d	chr1	89516916	89517096	-1.75	-0.39
Inpp5d	chr1	89614406	89616844	-1.50	-0.10
Tnfrsf11a	chr1	107723580	107741623	-11.00	-0.52
Rnasel	chr1	155600751	155602307	-0.89	-0.19
Rnasel	chr1	155610184	155610334	-1.19	1.11
Sumo1	chr1	59696455	59697048	-0.33	-0.01
Dhx9	chr1	155303276	155303397	-3.58	0.71
Il15ra	chr2	11654960	11655499	-0.57	-0.15
Il1rn	chr2	24205097	24205277	-0.69	-0.43
Rapgef1	chr2	29475329	29475390	-0.80	-0.67
Rapgef1	chr2	29557850	29558001	-1.86	-0.29
Rapgef1	chr2	29592963	29594536	-4.59	-0.22
Psmc3	chr2	90894439	90895182	-3.33	-0.20
Traf6	chr2	101530344	101537929	-2.95	-0.21
Hck	chr2	152934203	152934473	-1.97	0.48
Hck	chr2	152976907	152977028	-1.50	-0.25
Plcg1	chr2	160587552	160588233	-9.17	-1.31
Cd40	chr2	164896268	164897006	-0.30	-0.03
Ptpn1	chr2	167757946	167758006	-0.38	-0.08
Ptpn1	chr2	167800939	167802433	-5.76	-0.45
Ptpn1	chr2	167802851	167803629	-1.63	-0.30
Traf2	chr2	25374427	25375145	-2.44	-0.26
Psmd5	chr2	34707993	34709823	-1.65	-0.19
Psmd5	chr2	34726274	34726482	-4.41	-0.82
Psmb7	chr2	38498946	38499426	-3.13	-0.36
Atf2	chr2	73656947	73657068	-4.98	1.16
Il1b	chr2	129190464	129191002	-3.00	1.94
Il1b	chr2	129195984	129196856	-1.12	2.89
Psmf1	chr2	151543111	151543202	-1.31	-0.87
Psmf1	chr2	151543649	151544038	-0.30	0.01
Pik3ca	chr3	32335072	32335342	-3.17	-0.61
Pik3ca	chr3	32360651	32362022	-2.40	0.26
Shc1	chr3	89222472	89225623	-0.50	-0.10
Shc1	chr3	89225682	89226222	-15.40	-1.10
Shc1	chr3	89232244	89232484	-0.52	0.07
Psma5	chr3	108059873	108060022	-0.64	3.39
Il7	chr3	7573665	7573875	-0.26	-0.89
Il6ra	chr3	89673305	89680757	-0.99	0.10
Psmb4	chr3	94690701	94690880	-8.50	-0.41
Psmd4	chr3	94836626	94836894	-2.54	-0.16
Psmd4	chr3	94840526	94846467	-8.21	-0.35
Hist2h3c2	chr3	96042365	96042604	-1.05	0.63

Csf1	chr3	107544832	107548597	-0.68	0.16
Csf1	chr3	107548745	107550736	-0.68	-0.96
Csf1	chr3	107551093	107553341	-0.96	0.24
Nfkb1	chr3	135248247	135252299	-9.58	-0.45
Nfkb1	chr3	135354240	135354451	-1.35	0.77
Lyn	chr4	3605267	3605506	-12.00	-0.40
Lyn	chr4	3716941	3718014	-40.00	-0.36
Map3k7	chr4	32051081	32051232	-3.02	-1.01
Map3k7	chr4	32104109	32107267	-5.97	0.38
Map3k7	chr4	32110051	32110231	-0.27	0.00
Pik3r3	chr4	115945443	115972695	-28.90	3.76
Pik3r3	chr4	115975001	115975152	-1.61	1.80
Csf3r	chr4	125720761	125721238	-1.10	-0.26
Eif4g3	chr4	137676266	137682738	-1.73	-0.08
Eif4g3	chr4	137762235	137762386	-0.44	0.08
Ripk2	chr4	16050670	16051621	-4.17	-0.41
Ripk2	chr4	16090436	16090645	-16.00	-1.44
Ddx58	chr4	40152603	40155547	-1.90	-0.20
Ddx58	chr4	40186678	40186858	-0.44	0.12
Ifnb1	chr4	88167987	88168314	-0.43	1.96
Jun	chr4	94716292	94718913	-60.00	-0.27
Jak1	chr4	100825566	100826076	-0.60	0.05
Lck	chr4	129225797	129226157	-0.39	0.06
Lck	chr4	129234249	129234661	-0.99	2.21
Txlna	chr4	129305539	129309516	-6.31	-0.20
Txlna	chr4	129316496	129317784	-1.42	-0.10
Rps6ka1	chr4	133403354	133404010	-3.84	-0.27
Tnfrsf1b	chr4	144803977	144809673	-299.00	-1.07
Tnfrsf1b	chr4	144818975	144836773	-24.20	-1.34
Tnfrsf1b	chr4	144836594	144836774	-2.87	-0.59
Tnfrsf8	chr4	144858908	144859116	-0.56	-0.47
Pik3cd	chr4	149023575	149025880	-7.02	-0.36
Isg15	chr4	155573619	155574927	-38.70	-1.36
Psmd9	chr5	123678228	123678437	-0.34	0.03
Tnip2	chr5	34842175	34842385	-0.32	-0.02
Tec	chr5	73147051	73148633	-0.92	-0.13
Tec	chr5	73214871	73259687	-0.34	0.03
Ptpn11	chr5	121581827	121582307	-1.21	0.16
Ptpn11	chr5	121583292	121587519	-0.40	0.02
Ubc	chr5	125866453	125867671	-172.00	0.73
Ubc	chr5	125867938	125868147	-3.15	0.20
Ubc	chr5	125868235	125870298	-22.80	-0.40
Cul1	chr6	47404345	47445165	-3.56	0.31

Cul1	chr6	47474617	47476108	-1.64	-0.15
Irak2	chr6	113640953	113644480	-29.40	-0.65
Il17ra	chr6	120413214	120413425	-0.34	0.04
Il17ra	chr6	120431139	120433146	-3.70	-0.15
Tnfrsf1a	chr6	125311560	125312501	-18.50	-0.45
Gm8994	chr6	136279021	136279141	-0.30	-0.81
Gm8994	chr6	136279763	136279853	-0.25	0.86
Nod1	chr6	54874531	54874682	-0.75	1.04
Nod1	chr6	54893168	54893616	-0.68	0.56
Nod1	chr6	54893675	54894034	-0.75	0.80
Nod1	chr6	54894242	54894451	-0.24	-0.71
Nod1	chr6	54894600	54897981	-1.54	1.88
Uba3	chr6	97134556	97134676	-1.20	0.47
Ptpn6	chr6	124683045	124688519	-0.41	-0.07
Ltbr	chr6	125256588	125259229	-3.92	-0.23
Irf3	chr7	52253029	52253179	-1.59	-0.48
Irf3	chr7	52257097	52257801	-2.81	-0.37
Irf3	chr7	52257979	52258099	-1.43	-1.19
Il4ra	chr7	132719062	132720501	-2.64	-0.43
Il4ra	chr7	132720770	132721340	-2.72	0.67
Il21r	chr7	132776035	132776545	-0.83	1.25
Stx4a	chr7	134985321	134985978	-1.03	-0.12
Il11	chr7	4724009	4724369	-3.93	1.73
Il11	chr7	4724995	4729743	-1.13	0.28
Ube2m	chr7	13623018	13623619	-6.02	-0.45
Relb	chr7	20191690	20192229	-0.83	-0.11
Nfkbib	chr7	29546684	29546864	-25.70	-0.94
Nfkbib	chr7	29547132	29551543	-9.87	-0.61
Psmd8	chr7	29965393	29965573	-13.80	-0.43
Flt3l	chr7	52386558	52386918	-0.67	0.27
Il16	chr7	90792230	90794430	-1.06	-0.23
Il16	chr7	90800555	90809792	-1.37	-0.12
Il16	chr7	90870990	90871201	-1.10	0.43
Inppl1	chr7	108971385	108974549	-40.10	-0.55
Inppl1	chr7	108985423	108986317	-1.81	-0.40
Inppl1	chr7	108986619	108986740	-1.05	1.07
Sh2b1	chr7	133610925	133611549	-2.80	-0.45
Sh2b1	chr7	133615254	133616302	-10.60	-0.55
Sh2b1	chr7	133618136	133618465	-0.50	-0.15
Tollip	chr7	149069998	149076626	-5.15	-0.28
Map2k7	chr8	4245546	4246251	-0.79	0.09
Tnfsf13b	chr8	10035492	10035790	-2.07	0.90
Ppp2cb	chr8	34710092	34710359	-0.73	-0.37

Dusp4	chr8	35870663	35882259	-130.00	-0.63
Il12rb1	chr8	73343643	73344933	-13.80	-1.50
Prkaca	chr8	86496876	86500855	-1.59	-0.45
Ikbkb	chr8	23770606	23771027	-1.32	-0.20
Pik3r2	chr8	73292079	73292528	-7.07	-0.81
Pik3r2	chr8	73296811	73300581	-13.40	-0.87
Psmb10	chr8	108461631	108461967	-2.80	-0.63
Psmd7	chr8	110104338	110108077	-4.03	0.12
Casp1	chr9	5298635	5299458	-1.15	-0.21
Smarca4	chr9	21505589	21507505	-3.30	0.23
Ppp2r1b	chr9	50687990	50688710	-0.71	-0.09
Ppp2r1b	chr9	50701016	50701675	-4.03	-0.60
Dusp7	chr9	106270962	106271201	-3.92	-1.70
Dusp7	chr9	106271527	106276865	-1.31	-0.10
Birc2	chr9	7818614	7818734	-0.55	-0.29
Birc2	chr9	7833610	7834657	-0.90	0.13
Birc2	chr9	7834955	7835195	-1.05	0.42
Birc3	chr9	7848969	7850928	-1.17	-0.17
Birc3	chr9	7860311	7872961	-0.30	0.00
Cbl	chr9	43959287	43960877	-0.32	0.00
Cbl	chr9	44041859	44042099	-4.62	-0.81
Il10ra	chr9	45062219	45062638	-0.85	0.20
Il10ra	chr9	45063175	45068489	-0.47	0.03
Arih1	chr9	59302235	59325631	-2.11	0.44
Arih1	chr9	59329494	59334181	-4.64	0.32
Pias1	chr9	62729583	62729943	-0.50	0.10
Pias1	chr9	62799649	62828686	-0.80	0.12
Pik3cb	chr9	98939628	98941447	-5.88	0.37
Il20rb	chr9	100358436	100359660	-0.56	-0.27
Myd88	chr9	119248978	119249158	-3.52	-0.60
Ifngr1	chr10	19329011	19329761	-0.30	0.00
Fyn	chr10	39089604	39089994	-0.68	-0.12
Fyn	chr10	39271197	39284102	-0.31	0.00
Dusp6	chr10	98725864	98729379	-0.51	-0.02
Stat6	chr10	127080041	127080222	-2.32	-0.33
Stat6	chr10	127096737	127097564	-4.04	-0.16
Stat2	chr10	127727850	127728797	-3.58	-0.35
Stat2	chr10	127729305	127729665	-1.61	-0.28
Tab2	chr10	7625983	7626463	-1.79	0.18
Tab2	chr10	7626701	7630910	-3.35	-0.17
Tab2	chr10	7631204	7644574	-1.97	-0.07
Irak3	chr10	119579458	119580238	-1.04	-0.13
Il23a	chr10	127733667	127733845	-0.28	-0.14

Osm	chr11	4136787	4140791	-321.00	-1.80
Lif	chr11	4166528	4170243	-65.90	-1.66
Lif	chr11	4171319	4172158	-36.30	-1.91
Peli1	chr11	20991326	20991536	-0.58	0.15
Peli1	chr11	21047425	21048774	-11.20	-0.48
Psme4	chr11	30671774	30672135	-0.54	-0.18
Fbxw11	chr11	32644536	32644986	-0.78	-0.08
Mapk9	chr11	49696697	49697026	-1.80	0.29
Ppp2ca	chr11	51912325	51912474	-4.51	-0.88
Ppp2ca	chr11	51935449	51935836	-0.34	0.00
Map2k3	chr11	60745558	60745738	-0.87	-0.13
Map2k3	chr11	60755435	60757081	-0.31	0.00
Map2k3	chr11	60765468	60766215	-11.10	-0.38
Vamp2	chr11	68902029	68902686	-0.49	-0.14
Crk	chr11	75492811	75517946	-16.70	-0.28
Crk	chr11	75518934	75519414	-1.35	-0.15
Trim25	chr11	88860866	88861137	-34.20	-0.75
Trim25	chr11	88877093	88878370	-50.00	-0.45
Psmd3	chr11	98543867	98546829	-30.30	-0.74
Csf3	chr11	98562626	98562686	-18.20	-2.81
Stat5a	chr11	100737560	100737770	-3.89	2.58
Stat5a	chr11	100737859	100738070	-4.30	2.42
Psme3	chr11	101177564	101178336	-0.62	0.07
Psme3	chr11	101182070	101183288	-0.64	-0.06
Map3k3	chr11	106013617	106016298	-2.85	-0.22
Canx	chr11	50109707	50110776	-30.20	-0.37
Csf2	chr11	54060859	54061986	-0.80	-1.87
Mapk7	chr11	61303417	61306635	-16.60	-0.51
Mapk7	chr11	61306963	61307441	-3.11	-0.52
Map2k4	chr11	65503265	65506970	-4.86	-0.31
Dusp3	chr11	101834513	101835259	-0.43	0.02
Dusp3	chr11	101835527	101835885	-0.41	0.02
Dusp3	chr11	101835944	101836213	-0.85	-0.10
Map3k14	chr11	103081077	103082247	-3.53	-0.47
Map3k14	chr11	103100346	103101055	-0.68	-0.14
Map3k14	chr11	103103466	103128715	-0.39	-0.09
Grb2	chr11	115505517	115505906	-1.13	0.17
Grb2	chr11	115506591	115507248	-1.12	-0.07
Socs3	chr11	117827669	117830291	-196.00	-0.95
P4hb	chr11	120421617	120422006	-31.00	-0.33
P4hb	chr11	120422453	120422573	-21.50	-0.34
Sdc1	chr12	8778201	8778442	-0.29	-0.04
Sdc1	chr12	8798515	8799355	-3.14	-0.55

Fos	chr12	86814850	86817711	-87.60	-0.74
Psmc1	chr12	101348377	101351316	-1.50	-0.23
Traf3	chr12	112498880	112500724	-1.28	0.14
Nfkbia	chr12	56593395	56593604	-103.00	-1.36
Sos2	chr12	70685554	70689696	-1.22	0.08
Sos2	chr12	70717850	70719054	-1.36	0.14
Sos2	chr12	70782599	70782839	-0.78	-0.61
Rps6ka5	chr12	101789211	101791305	-0.71	0.25
Hist1h3h	chr13	21809496	21809698	-1.42	0.30
Hist1h3f	chr13	23636303	23636393	-2.07	-0.34
Hist1h3d	chr13	23667608	23667786	-1.12	-0.25
Syk	chr13	52678805	52706632	-13.30	-0.48
Syk	chr13	52727069	52733575	-2.37	-0.22
Syk	chr13	52737300	52738605	-0.64	0.10
Syk	chr13	52738964	52742902	-5.19	-0.18
Syk	chr13	52743770	52743981	-0.44	0.06
Il6st	chr13	113254307	113255963	-0.83	-0.24
Il6st	chr13	113294105	113296021	-3.87	0.19
Il6st	chr13	113296618	113296739	-0.70	-0.20
Hist1h3i	chr13	21875180	21875266	-0.41	0.06
Hist1h3c	chr13	23837329	23837390	-0.31	-0.03
Pik3r1	chr13	102453986	102456264	-3.37	0.30
Pik3r1	chr13	102478932	102525656	-0.37	-0.05
Pik3r1	chr13	102527244	102538172	-0.72	0.07
Flnb	chr14	8650500	8650621	-0.79	0.25
Flnb	chr14	8737110	8739780	-5.32	-0.21
Flnb	chr14	8782901	8784101	-1.39	-0.09
Nkiras1	chr14	19111014	19112745	-0.47	-0.11
Psmc6	chr14	45949498	45950514	-0.60	-0.09
Irf9	chr14	56222821	56223460	-0.30	0.02
Irf9	chr14	56223519	56224000	-1.25	-0.42
Irf9	chr14	56227759	56228328	-0.79	0.10
Camk2g	chr14	21554783	21555022	-0.76	0.27
Camk2g	chr14	21555081	21555948	-2.05	-0.26
Prkcd	chr14	31408808	31411699	-1.85	-0.13
Prkcd	chr14	31411787	31411937	-0.73	0.22
Prkcd	chr14	31439273	31439394	-1.07	-0.33
Ptk2b	chr14	66772153	66773020	-20.90	-0.52
Csf2rb	chr15	78156456	78166828	-4.16	-0.44
Csf2rb	chr15	78177155	78179733	-19.50	-0.49
Tab1	chr15	79989230	79992132	-13.10	-0.63
Rbx1	chr15	81305971	81306061	-1.28	-0.57
Atf1	chr15	100084825	100090754	-1.03	0.09

Il7r	chr15	9436363	9437293	-4.34	0.79
Il7r	chr15	9437531	9439923	-4.04	0.79
Ywhaz	chr15	36723285	36723376	-0.55	0.11
Csf2rb2	chr15	78112999	78113060	-1.58	0.68
Csf2rb2	chr15	78114558	78116509	-0.74	0.08
Csf2rb2	chr15	78127494	78136033	-1.82	-0.32
Mapk1	chr16	17038671	17038882	-0.49	0.07
Crkl	chr16	17452169	17484582	-4.45	-0.19
Psmd2	chr16	20651724	20652324	-20.60	-0.49
Eif4g1	chr16	20672821	20673965	-1.18	-0.11
Eif4g1	chr16	20674380	20675449	-1.23	-0.13
Eif4g1	chr16	20679335	20680055	-13.00	-0.18
Eif4g1	chr16	20689353	20692866	-16.70	-0.31
Il1rap	chr16	26714898	26715319	-4.11	0.91
Ifnar2	chr16	91399551	91405652	-2.13	-0.16
Il10rb	chr16	91425363	91425632	-3.68	-0.33
Ifnar1	chr16	91503665	91506361	-3.40	-0.24
Ifnar1	chr16	91506810	91506901	-0.62	0.22
Ifnar1	chr16	91507170	91507680	-1.40	-0.22
Ifngr2	chr16	91563142	91563682	-0.51	-0.03
Socs1	chr16	10783990	10784912	-0.46	-0.26
Socs1	chr16	10784970	10785629	-0.59	-1.60
App	chr16	85025115	85043833	-16.90	-0.31
App	chr16	85120907	85173892	-5.77	-0.46
Brwd1	chr16	96214898	96214959	-2.85	1.53
Brwd1	chr16	96223575	96225521	-4.61	-0.24
Brwd1	chr16	96226300	96226615	-0.56	-1.29
Rps6ka2	chr17	7374493	7374614	-0.54	0.42
Rps6ka2	chr17	7506884	7507544	-0.43	0.14
Mapk14	chr17	28828316	28828704	-3.84	-0.61
Mapk14	chr17	28884007	28885350	-1.27	0.08
Psmb8	chr17	34335288	34335468	-2.22	-0.33
Ltb	chr17	35331600	35332385	-11.70	-0.51
Ltb	chr17	35332444	35333220	-19.10	-0.52
Ebi3	chr17	56095665	56096445	-4.37	-0.35
Tnfsf9	chr17	57244807	57244958	-0.73	-0.14
Tnfsf9	chr17	57245676	57247180	-1.89	-0.10
Vav1	chr17	57418522	57418613	-0.31	0.06
Vav1	chr17	57467106	57467554	-1.28	0.13
Ppm1b	chr17	85357671	85364400	-0.33	0.09
Ppm1b	chr17	85373436	85402617	-0.40	-0.02
Ppm1b	chr17	85405048	85413253	-7.19	-0.91
Socs5	chr17	87507227	87507318	-0.46	-0.23

Socs5	chr17	87520106	87534863	-0.79	0.07
Tnfrsf12a	chr17	23814327	23814416	-0.36	0.14
Tnf	chr17	35336694	35337384	-434.00	-1.28
Tnf	chr17	35338760	35338941	-117.00	-1.09
Lta	chr17	35340403	35340580	-0.52	-0.46
Ppp2r5d	chr17	46820058	46820862	-0.90	-0.11
Ppp2r5d	chr17	46837029	46841951	-0.53	-0.11
Tnfsf14	chr17	57329542	57330291	-0.18	0.06
Eif2ak2	chr17	79251889	79252308	-0.49	0.06
Eif2ak2	chr17	79252515	79254306	-29.10	-0.53
Eif2ak2	chr17	79277606	79281912	-2.74	-0.48
Sos1	chr17	80797100	80799127	-0.43	-0.03
Sos1	chr17	80833039	80834290	-0.90	-0.18
Sos1	chr17	80879343	80879554	-1.15	-0.89
Camk2a	chr18	61144547	61145295	-1.50	-0.78
Csf1r	chr18	61265501	61269298	-2.66	-0.20
Csf1r	chr18	61290043	61290284	-12.60	-0.40
Csf1r	chr18	61290463	61290793	-14.80	-0.34
Map3k8	chr18	4348899	4352951	-3.59	-0.54
Rela	chr19	5646968	5647773	-3.54	-0.20
Jak2	chr19	29385493	29386732	-0.30	0.00
Jak2	chr19	29386881	29387450	-3.92	0.54
Btrc	chr19	45604117	45604298	-0.65	1.17
Nfkb2	chr19	46379436	46380290	-0.39	0.13
Peli3	chr19	4932033	4932629	-0.24	0.04
Stx3	chr19	11893652	11893893	-0.37	0.09
Chuk	chr19	44181817	44181937	-0.55	-0.16
Csf2ra	chr19	61300453	61302211	-14.30	-0.30
Csf2ra	chr19	61302992	61304321	-7.97	-0.39
Il13ra1	chrX	33695553	33709728	-1.69	0.25
Tab3	chrX	82859477	82860195	-4.67	0.57
Tab3	chrX	82860522	82860673	-1.01	0.46
Tab3	chrX	82873570	82876044	-16.40	2.20
Irak1	chrX	71260386	71260835	-0.46	0.04
Eda2r	chrX	94533994	94534384	-1.02	0.70

mRNA splicing

gene_name	chr	chromStart	chromEnd	diff.lg.p	diff.log2.fc
Sf3b1	chr1	55084201	55084322	-0.60	-0.13
Dhx9	chr1	155303276	155303397	-3.58	0.71
Hnrnpu	chr1	180259403	180260044	-6.87	-0.19
Hnrnpu	chr1	180267465	180267915	-20.60	-0.28
Cstf3	chr2	104504891	104505372	-2.93	0.31
Ctnnbl1	chr2	157563136	157600315	-0.47	-0.04

Srsf6	chr2	162757243	162757817	-0.30	0.00
Cstf1	chr2	172203242	172206586	-1.17	-0.10
Rbm17	chr2	11522379	11524826	-2.08	-0.47
Prpf40a	chr2	53049040	53050262	-0.95	-0.15
Cwc22	chr2	77733949	77741916	-2.10	-0.15
Clp1	chr2	84563518	84566269	-0.31	0.00
Snrpb	chr2	130004981	130005100	-1.98	-0.21
Sf3b4	chr3	95976984	95978290	-33.20	-0.63
Bcas2	chr3	102981340	102982484	-12.40	0.55
Prcc	chr3	87662883	87664130	-1.84	-0.22
Prcc	chr3	87673527	87689484	-20.90	-0.46
Srsf11	chr3	157674772	157675808	-16.50	-0.32
Prpf4	chr4	62083727	62085481	-2.02	-0.20
Srsf4	chr4	131453751	131457610	-9.11	-0.28
Dnajc8	chr4	132108975	132109154	-0.85	-0.09
Syf2	chr4	134486894	134490903	-15.80	-0.46
Syf2	chr4	134493303	134493452	-1.95	-0.45
Hnrnpr	chr4	135895398	135895548	-0.47	-0.07
Hnrnpr	chr4	135895666	135895966	-2.48	-0.47
Ybx1	chr4	118957671	118967118	-120.00	-0.62
Ppih	chr4	118979868	118980437	-3.08	-0.75
Srrm1	chr4	134878175	134878385	-1.65	-0.18
Srrm1	chr4	134880008	134881048	-18.30	-0.47
Srrm1	chr4	134902739	134909069	-0.66	-0.08
Snrnp35	chr5	124933163	124940925	-3.86	-0.36
Bud31	chr5	145908765	145908943	-1.40	-0.20
Cpsf4	chr5	145939775	145942700	-0.65	0.07
Hnrnpd	chr5	100407566	100407657	-0.58	0.43
Hnrnpf	chr6	117856811	117856962	-2.52	-0.35
Hnrnpf	chr6	117873875	117874265	-7.79	-0.46
Hnrnpf	chr6	117874653	117874893	-29.00	-0.62
Gm8994	chr6	136279021	136279141	-0.30	-0.81
Gm8994	chr6	136279763	136279853	-0.25	0.86
Hnrnpa2b1	chr6	51419743	51419893	-2.17	-0.20
Usp39	chr6	72268758	72269207	-0.81	0.11
Usp39	chr6	72294989	72295169	-8.82	-1.02
Pcbp1	chr6	86474877	86476159	-0.55	-0.01
Isy1	chr6	87769040	87771528	-1.21	-0.18
Wbp11	chr6	136762473	136765520	-10.20	-0.30
Prpf31	chr7	3581586	3582328	-1.76	-0.17
Prpf31	chr7	3591264	3593127	-11.30	-0.32
U2af2	chr7	5030883	5031212	-1.10	-0.10
Snrpd2	chr7	19735186	19736596	-0.85	-0.20

Polr2i	chr7	31017332	31017772	-0.55	0.13
Snrpa1	chr7	73205399	73205549	-2.12	-0.41
Hnrnpul1	chr7	26533405	26539769	-33.90	-0.44
Snrnp70	chr7	52631823	52636159	-50.50	-0.38
Snrnp70	chr7	52647692	52651017	-13.70	-0.31
Pcf11	chr7	99793118	99795438	-8.06	0.60
Pcf11	chr7	99801721	99808380	-11.00	-0.25
Pcf11	chr7	99809567	99810434	-2.11	-0.10
Pcf11	chr7	99818003	99818422	-5.53	-0.40
Cd2bp2	chr7	134337180	134338647	-23.50	-0.40
Elavl1	chr8	4289099	4289460	-0.68	0.07
Dhx38	chr8	112071923	112073753	-0.53	0.05
Dhx38	chr8	112080084	112080835	-6.56	0.50
Dhx38	chr8	112084553	112089501	-0.31	0.00
Sf3b3	chr8	113334571	113334842	-1.18	-0.08
Ppil4	chr10	7534486	7541105	-2.05	-0.23
Sf3b5	chr10	12728255	12728989	-8.10	-0.27
Sf3a2	chr10	80261479	80263960	-0.95	-0.26
Sf3a2	chr10	80266830	80266980	-0.93	-0.18
Ccar1	chr10	62208074	62209806	-0.86	0.08
Ccar1	chr10	62214563	62223000	-0.51	-0.03
Polr2e	chr10	79501316	79502404	-2.85	-0.43
Sf3a1	chr11	4080296	4080657	-2.08	-0.28
Zmat5	chr11	4604673	4622487	-2.64	-0.83
Snrnp25	chr11	32105414	32105652	-1.78	-0.52
Hnrnph1	chr11	50191824	50191914	-0.98	0.13
Srsf1	chr11	87861172	87861322	-1.85	-0.33
Ddx42	chr11	106078239	106085433	-6.07	-0.73
Ddx42	chr11	106108808	106109826	-12.90	-0.40
Cwc25	chr11	97608012	97614001	-1.06	-0.11
Eftud2	chr11	102699785	102700145	-3.75	-0.28
Ddx5	chr11	106643371	106643551	-60.70	0.67
Ddx5	chr11	106650118	106650179	-4.34	1.55
Srsf2	chr11	116714108	116714408	-50.00	-0.61
Srsf5	chr12	82046490	82046729	-16.60	-0.48
Srsf5	chr12	82050780	82051078	-44.00	-0.66
Cpsf2	chr12	103214183	103214244	-0.29	-0.03
Cpsf2	chr12	103240910	103241994	-0.60	-0.04
Papola	chr12	107022971	107023092	-4.18	-0.34
Papola	chr12	107072945	107075506	-2.32	-0.11
Snw1	chr12	88791126	88791810	-8.21	-0.35
Ddx46	chr13	55741151	55749105	-10.30	-0.37
Hnrnpa0	chr13	58228250	58229917	-61.50	-0.36

Hnrnpk	chr13	58501349	58504674	-3.06	-0.25
Ppwd1	chr13	105010109	105010529	-1.01	0.16
Ppwd1	chr13	105018773	105018924	-0.32	-0.04
Gtf2f2	chr14	76297012	76306651	-1.19	-0.13
Wbp4	chr14	79861599	79863753	-7.89	-0.67
Polr2k	chr15	36103800	36104751	-0.34	0.08
Puf60	chr15	75900611	75900909	-0.30	0.00
Ddx23	chr15	98475937	98476385	-4.86	-0.28
Ddx23	chr15	98488768	98493320	-11.90	-0.70
Ncbp2	chr16	31957240	31957480	-1.22	-0.23
Tra2b	chr16	22265852	22266002	-2.23	-0.43
Srrm2	chr17	23952780	23953201	-31.90	-0.37
Srrm2	chr17	23953650	23955539	-13.20	0.12
Srrm2	chr17	23957486	23958086	-12.30	0.23
Rnps1	chr17	24551619	24552074	-1.31	-0.33
Dhx16	chr17	36019018	36019798	-2.95	-0.31
Ppil1	chr17	29387927	29388668	-0.82	-0.12
Hnrnpm	chr17	33786662	33789227	-12.90	-0.29
Hnrnpm	chr17	33814217	33822403	-1.55	-0.10
Cdc5l	chr17	45528957	45530123	-2.27	0.27
Cdc5l	chr17	45564949	45570477	-1.04	-0.44
Gtf2f1	chr17	57143372	57143559	-0.74	-0.13
Srsf7	chr17	80606178	80606645	-0.35	-0.03
Polr2d	chr18	31955877	31956355	-0.34	-0.02
Wdr33	chr18	31963710	31986988	-10.60	-0.69
Wdr33	chr18	32047426	32048936	-16.50	-0.52
Wdr33	chr18	32049204	32067113	-1.24	-0.09
Txnl4a	chr18	80403957	80409392	-5.60	-0.13
Txnl4a	chr18	80415452	80419302	-1.93	-0.17
Txnl4a	chr18	80421035	80421843	-0.77	-0.27
Cpsf7	chr19	10614133	10620757	-1.51	0.08
Cpsf7	chr19	10621625	10621806	-12.40	0.88
Prpf19	chr19	10969781	10969902	-0.74	-0.17
Cstf2t	chr19	31157390	31159177	-2.75	0.13
Sart1	chr19	5381212	5381273	-0.82	-0.17
Sart1	chr19	5382852	5383166	-1.19	-0.14
Sart1	chr19	5384663	5388703	-2.03	-0.15
Polr2g	chr19	8872986	8873047	-0.44	-0.14
Smndc1	chr19	53454959	53455080	-1.53	0.23
Gpkow	chrX	7285241	7285480	-4.10	0.65
Hnrnph2	chrX	131137272	131140187	-17.40	0.37
Upf3b	chrX	34632248	34632639	-0.43	-0.05
Zrsr2	chrX	160374663	160377244	-0.42	0.08

Zrsr2	chrX	160396267	160396568	-0.53	0.09

Processing of capped intro-containing pre-mRNA

gene_name	chr	chromStart	chromEnd	diff.lg.p	diff.log2.fc
Sf3b1	chr1	55084201	55084322	-0.60	-0.13
Zc3h11a	chr1	135516447	135517433	-9.17	-0.31
Zc3h11a	chr1	135517641	135518733	-2.10	-0.10
Zc3h11a	chr1	135521340	135523781	-17.40	-0.37
Dhx9	chr1	155303276	155303397	-3.58	0.71
Hnrnpu	chr1	180259403	180260044	-6.87	-0.19
Hnrnpu	chr1	180267465	180267915	-20.60	-0.28
Nup214	chr2	31888785	31893623	-1.70	-0.12
Cstf3	chr2	104504891	104505372	-2.93	0.31
Nxt1	chr2	148498350	148501762	-3.02	-0.22
Ctnnbl1	chr2	157563136	157600315	-0.47	-0.04
Srsf6	chr2	162757243	162757817	-0.30	0.00
Cstf1	chr2	172203242	172206586	-1.17	-0.10
Rae1	chr2	172840787	172841203	-9.79	-0.73
Rbm17	chr2	11522379	11524826	-2.08	-0.47
Prpf40a	chr2	53049040	53050262	-0.95	-0.15
Cwc22	chr2	77733949	77741916	-2.10	-0.15
Clp1	chr2	84563518	84566269	-0.31	0.00
Snrpb	chr2	130004981	130005100	-1.98	-0.21
Sf3b4	chr3	95976984	95978290	-33.20	-0.63
Bcas2	chr3	102981340	102982484	-12.40	0.55
Prcc	chr3	87662883	87664130	-1.84	-0.22
Prcc	chr3	87673527	87689484	-20.90	-0.46
Chtop	chr3	90303653	90306049	-0.50	-0.02
Chtop	chr3	90313185	90313365	-3.07	-0.25
Srsf11	chr3	157674772	157675808	-16.50	-0.32
Prpf4	chr4	62083727	62085481	-2.02	-0.20
Ndc1	chr4	107083754	107086943	-1.16	0.18
Srsf4	chr4	131453751	131457610	-9.11	-0.28
Dnajc8	chr4	132108975	132109154	-0.85	-0.09
Syf2	chr4	134486894	134490903	-15.80	-0.46
Syf2	chr4	134493303	134493452	-1.95	-0.45
Hnrnpr	chr4	135895398	135895548	-0.47	-0.07
Hnrnpr	chr4	135895666	135895966	-2.48	-0.47
Ybx1	chr4	118957671	118967118	-120.00	-0.62
Ppih	chr4	118979868	118980437	-3.08	-0.75
Srrm1	chr4	134878175	134878385	-1.65	-0.18
Srrm1	chr4	134880008	134881048	-18.30	-0.47
Srrm1	chr4	134902739	134909069	-0.66	-0.08
Nupl2	chr5	23687726	23687877	-0.80	0.38

Nupl2	chr5	23687966	23689616	-1.38	-0.19
Snrnp35	chr5	124933163	124940925	-3.86	-0.36
Bud31	chr5	145908765	145908943	-1.40	-0.20
Cpsf4	chr5	145939775	145942700	-0.65	0.07
Nup54	chr5	92845130	92846543	-1.18	-0.16
Hnrnpd	chr5	100407566	100407657	-0.58	0.43
Pom121	chr5	135853620	135857139	-7.02	-0.36
Pom121	chr5	135857525	135857795	-2.51	-0.32
Nup205	chr6	35127615	35133895	-0.30	0.02
Hnrnpf	chr6	117856811	117856962	-2.52	-0.35
Hnrnpf	chr6	117873875	117874265	-7.79	-0.46
Hnrnpf	chr6	117874653	117874893	-29.00	-0.62
Gm8994	chr6	136279021	136279141	-0.30	-0.81
Gm8994	chr6	136279763	136279853	-0.25	0.86
Hnrnpa2b1	chr6	51419743	51419893	-2.17	-0.20
Usp39	chr6	72268758	72269207	-0.81	0.11
Usp39	chr6	72294989	72295169	-8.82	-1.02
Pcbp1	chr6	86474877	86476159	-0.55	-0.01
Isy1	chr6	87769040	87771528	-1.21	-0.18
Nup210	chr6	90963060	90963660	-3.36	-0.50
Wbp11	chr6	136762473	136765520	-10.20	-0.30
Prpf31	chr7	3581586	3582328	-1.76	-0.17
Prpf31	chr7	3591264	3593127	-11.30	-0.32
U2af2	chr7	5030883	5031212	-1.10	-0.10
Snrpd2	chr7	19735186	19736596	-0.85	-0.20
Polr2i	chr7	31017332	31017772	-0.55	0.13
Nup62	chr7	52071739	52086176	-19.00	-0.23
Snrpa1	chr7	73205399	73205549	-2.12	-0.41
Hnrnpul1	chr7	26533405	26539769	-33.90	-0.44
Snrnp70	chr7	52631823	52636159	-50.50	-0.38
Snrnp70	chr7	52647692	52651017	-13.70	-0.31
Pcf11	chr7	99793118	99795438	-8.06	0.60
Pcf11	chr7	99801721	99808380	-11.00	-0.25
Pcf11	chr7	99809567	99810434	-2.11	-0.10
Pcf11	chr7	99818003	99818422	-5.53	-0.40
Nup98	chr7	109268003	109269291	-7.87	-0.22
Nup98	chr7	109283070	109283401	-23.60	-0.89
Cd2bp2	chr7	134337180	134338647	-23.50	-0.40
Elavl1	chr8	4289099	4289460	-0.68	0.07
Dhx38	chr8	112071923	112073753	-0.53	0.05
Dhx38	chr8	112080084	112080835	-6.56	0.50
Dhx38	chr8	112084553	112089501	-0.31	0.00
Sf3b3	chr8	113334571	113334842	-1.18	-0.08

Ppil4	chr10	7534486	7541105	-2.05	-0.23
Sf3b5	chr10	12728255	12728989	-8.10	-0.27
Ranbp2	chr10	57939345	57940486	-6.91	0.22
Ranbp2	chr10	57940784	57941055	-0.33	-0.01
Ranbp2	chr10	57941564	57941955	-1.34	-0.09
Ranbp2	chr10	57942134	57945844	-28.50	0.35
Ranbp2	chr10	57955388	57956782	-8.30	0.31
Sf3a2	chr10	80261479	80263960	-0.95	-0.26
Sf3a2	chr10	80266830	80266980	-0.93	-0.18
Ccar1	chr10	62208074	62209806	-0.86	0.08
Ccar1	chr10	62214563	62223000	-0.51	-0.03
Polr2e	chr10	79501316	79502404	-2.85	-0.43
Sf3a1	chr11	4080296	4080657	-2.08	-0.28
Zmat5	chr11	4604673	4622487	-2.64	-0.83
Snrnp25	chr11	32105414	32105652	-1.78	-0.52
Slu7	chr11	43247232	43251659	-1.97	0.22
Slu7	chr11	43258751	43259812	-3.05	-0.23
Slu7	chr11	43260795	43260976	-0.25	0.11
Hnrnph1	chr11	50191824	50191914	-0.98	0.13
Srsf1	chr11	87861172	87861322	-1.85	-0.33
Casc3	chr11	98683710	98689970	-6.76	-0.34
Casc3	chr11	98692721	98694074	-0.45	0.04
Ddx42	chr11	106078239	106085433	-6.07	-0.73
Ddx42	chr11	106108808	106109826	-12.90	-0.40
Cwc25	chr11	97608012	97614001	-1.06	-0.11
Eftud2	chr11	102699785	102700145	-3.75	-0.28
Ddx5	chr11	106643371	106643551	-60.70	0.67
Ddx5	chr11	106650118	106650179	-4.34	1.55
Srsf2	chr11	116714108	116714408	-50.00	-0.61
Srsf5	chr12	82046490	82046729	-16.60	-0.48
Srsf5	chr12	82050780	82051078	-44.00	-0.66
Cpsf2	chr12	103214183	103214244	-0.29	-0.03
Cpsf2	chr12	103240910	103241994	-0.60	-0.04
Papola	chr12	107022971	107023092	-4.18	-0.34
Papola	chr12	107072945	107075506	-2.32	-0.11
Snw1	chr12	88791126	88791810	-8.21	-0.35
Ddx46	chr13	55741151	55749105	-10.30	-0.37
Nup153	chr13	46776258	46776559	-2.23	0.27
Nup153	chr13	46782040	46792068	-1.16	0.05
Nup153	chr13	46822738	46823218	-7.57	-0.49
Thoc3	chr13	54561210	54561569	-0.42	-0.04
Hnrnpa0	chr13	58228250	58229917	-61.50	-0.36
Hnrnpk	chr13	58501349	58504674	-3.06	-0.25

Ppwd1	chr13	105010109	105010529	-1.01	0.16
Ppwd1	chr13	105018773	105018924	-0.32	-0.04
Gtf2f2	chr14	76297012	76306651	-1.19	-0.13
Wbp4	chr14	79861599	79863753	-7.89	-0.67
Polr2k	chr15	36103800	36104751	-0.34	0.08
Nup50	chr15	84757805	84765822	-13.80	-0.39
Nup50	chr15	84770131	84771000	-9.31	-0.39
Nup50	chr15	84773033	84773303	-0.67	0.14
Puf60	chr15	75900611	75900909	-0.30	0.00
Poldip3	chr15	82958023	82959641	-1.64	-0.10
Ddx23	chr15	98475937	98476385	-4.86	-0.28
Ddx23	chr15	98488768	98493320	-11.90	-0.70
Ncbp2	chr16	31957240	31957480	-1.22	-0.23
Fyttd1	chr16	32877869	32884216	-2.17	-0.38
Fyttd1	chr16	32902616	32905812	-4.94	-0.62
Tra2b	chr16	22265852	22266002	-2.23	-0.43
Srrm2	chr17	23952780	23953201	-31.90	-0.37
Srrm2	chr17	23953650	23955539	-13.20	0.12
Srrm2	chr17	23957486	23958086	-12.30	0.23
Rnps1	chr17	24551619	24552074	-1.31	-0.33
Ddx39b	chr17	35378720	35378917	-1.75	-0.31
Ddx39b	chr17	35386162	35386790	-0.58	-0.04
Ddx39b	chr17	35566041	35566729	-1.02	-0.30
Dhx16	chr17	36019018	36019798	-2.95	-0.31
Wtap	chr17	13160233	13163945	-7.53	-0.30
Wtap	chr17	13167357	13168316	-0.54	-0.06
Thoc6	chr17	23810648	23810831	-0.28	0.01
Ppil1	chr17	29387927	29388668	-0.82	-0.12
Hnrnpm	chr17	33786662	33789227	-12.90	-0.29
Hnrnpm	chr17	33814217	33822403	-1.55	-0.10
Cdc5l	chr17	45528957	45530123	-2.27	0.27
Cdc5l	chr17	45564949	45570477	-1.04	-0.44
Gtf2f1	chr17	57143372	57143559	-0.74	-0.13
Srsf7	chr17	80606178	80606645	-0.35	-0.03
Polr2d	chr18	31955877	31956355	-0.34	-0.02
Wdr33	chr18	31963710	31986988	-10.60	-0.69
Wdr33	chr18	32047426	32048936	-16.50	-0.52
Wdr33	chr18	32049204	32067113	-1.24	-0.09
Txnl4a	chr18	80403957	80409392	-5.60	-0.13
Txnl4a	chr18	80415452	80419302	-1.93	-0.17
Txnl4a	chr18	80421035	80421843	-0.77	-0.27
Tmem223	chr19	8846695	8846965	-4.06	1.60
Cpsf7	chr19	10614133	10620757	-1.51	0.08

Cpsf7	chr19	10621625	10621806	-12.40	0.88
Prpf19	chr19	10969781	10969902	-0.74	-0.17
Cstf2t	chr19	31157390	31159177	-2.75	0.13
Sart1	chr19	5381212	5381273	-0.82	-0.17
Sart1	chr19	5382852	5383166	-1.19	-0.14
Sart1	chr19	5384663	5388703	-2.03	-0.15
Polr2g	chr19	8872986	8873047	-0.44	-0.14
Smndc1	chr19	53454959	53455080	-1.53	0.23
Gpkow	chrX	7285241	7285480	-4.10	0.65
Hnrnph2	chrX	131137272	131140187	-17.40	0.37
Upf3b	chrX	34632248	34632639	-0.43	-0.05
Thoc2	chrX	39150625	39150955	-4.71	0.47
Zrsr2	chrX	160374663	160377244	-0.42	0.08
Zrsr2	chrX	160396267	160396568	-0.53	0.09

mRNA splicing-major pathway

gene_name	chr	chromStart	chromEnd	diff.lg.p	diff.log2.fc
Sf3b1	chr1	55084201	55084322	-0.60	-0.13
Dhx9	chr1	155303276	155303397	-3.58	0.71
Hnrnpu	chr1	180259403	180260044	-6.87	-0.19
Hnrnpu	chr1	180267465	180267915	-20.60	-0.28
Cstf3	chr2	104504891	104505372	-2.93	0.31
Ctnnbl1	chr2	157563136	157600315	-0.47	-0.04
Srsf6	chr2	162757243	162757817	-0.30	0.00
Cstf1	chr2	172203242	172206586	-1.17	-0.10
Rbm17	chr2	11522379	11524826	-2.08	-0.47
Prpf40a	chr2	53049040	53050262	-0.95	-0.15
Cwc22	chr2	77733949	77741916	-2.10	-0.15
Clp1	chr2	84563518	84566269	-0.31	0.00
Snrpb	chr2	130004981	130005100	-1.98	-0.21
Sf3b4	chr3	95976984	95978290	-33.20	-0.63
Bcas2	chr3	102981340	102982484	-12.40	0.55
Prcc	chr3	87662883	87664130	-1.84	-0.22
Prcc	chr3	87673527	87689484	-20.90	-0.46
Srsf11	chr3	157674772	157675808	-16.50	-0.32
Prpf4	chr4	62083727	62085481	-2.02	-0.20
Srsf4	chr4	131453751	131457610	-9.11	-0.28
Dnajc8	chr4	132108975	132109154	-0.85	-0.09
Syf2	chr4	134486894	134490903	-15.80	-0.46
Syf2	chr4	134493303	134493452	-1.95	-0.45
Hnrnpr	chr4	135895398	135895548	-0.47	-0.07
Hnrnpr	chr4	135895666	135895966	-2.48	-0.47
Ybx1	chr4	118957671	118967118	-120.00	-0.62
Ppih	chr4	118979868	118980437	-3.08	-0.75

Srrm1	chr4	134878175	134878385	-1.65	-0.18
Srrm1	chr4	134880008	134881048	-18.30	-0.47
Srrm1	chr4	134902739	134909069	-0.66	-0.08
Bud31	chr5	145908765	145908943	-1.40	-0.20
Cpsf4	chr5	145939775	145942700	-0.65	0.07
Hnrnpd	chr5	100407566	100407657	-0.58	0.43
Hnrnpf	chr6	117856811	117856962	-2.52	-0.35
Hnrnpf	chr6	117873875	117874265	-7.79	-0.46
Hnrnpf	chr6	117874653	117874893	-29.00	-0.62
Gm8994	chr6	136279021	136279141	-0.30	-0.81
Gm8994	chr6	136279763	136279853	-0.25	0.86
Hnrnpa2b1	chr6	51419743	51419893	-2.17	-0.20
Usp39	chr6	72268758	72269207	-0.81	0.11
Usp39	chr6	72294989	72295169	-8.82	-1.02
Pcbp1	chr6	86474877	86476159	-0.55	-0.01
Isy1	chr6	87769040	87771528	-1.21	-0.18
Wbp11	chr6	136762473	136765520	-10.20	-0.30
Prpf31	chr7	3581586	3582328	-1.76	-0.17
Prpf31	chr7	3591264	3593127	-11.30	-0.32
U2af2	chr7	5030883	5031212	-1.10	-0.10
Snrpd2	chr7	19735186	19736596	-0.85	-0.20
Polr2i	chr7	31017332	31017772	-0.55	0.13
Snrpa1	chr7	73205399	73205549	-2.12	-0.41
Hnrnpul1	chr7	26533405	26539769	-33.90	-0.44
Snrnp70	chr7	52631823	52636159	-50.50	-0.38
Snrnp70	chr7	52647692	52651017	-13.70	-0.31
Pcf11	chr7	99793118	99795438	-8.06	0.60
Pcf11	chr7	99801721	99808380	-11.00	-0.25
Pcf11	chr7	99809567	99810434	-2.11	-0.10
Pcf11	chr7	99818003	99818422	-5.53	-0.40
Cd2bp2	chr7	134337180	134338647	-23.50	-0.40
Elavl1	chr8	4289099	4289460	-0.68	0.07
Dhx38	chr8	112071923	112073753	-0.53	0.05
Dhx38	chr8	112080084	112080835	-6.56	0.50
Dhx38	chr8	112084553	112089501	-0.31	0.00
Sf3b3	chr8	113334571	113334842	-1.18	-0.08
Ppil4	chr10	7534486	7541105	-2.05	-0.23
Sf3b5	chr10	12728255	12728989	-8.10	-0.27
Sf3a2	chr10	80261479	80263960	-0.95	-0.26
Sf3a2	chr10	80266830	80266980	-0.93	-0.18
Ccar1	chr10	62208074	62209806	-0.86	0.08
Ccar1	chr10	62214563	62223000	-0.51	-0.03
Polr2e	chr10	79501316	79502404	-2.85	-0.43

Sf3a1	chr11	4080296	4080657	-2.08	-0.28
Hnrnph1	chr11	50191824	50191914	-0.98	0.13
Srsf1	chr11	87861172	87861322	-1.85	-0.33
Ddx42	chr11	106078239	106085433	-6.07	-0.73
Ddx42	chr11	106108808	106109826	-12.90	-0.40
Cwc25	chr11	97608012	97614001	-1.06	-0.11
Eftud2	chr11	102699785	102700145	-3.75	-0.28
Ddx5	chr11	106643371	106643551	-60.70	0.67
Ddx5	chr11	106650118	106650179	-4.34	1.55
Srsf2	chr11	116714108	116714408	-50.00	-0.61
Srsf5	chr12	82046490	82046729	-16.60	-0.48
Srsf5	chr12	82050780	82051078	-44.00	-0.66
Cpsf2	chr12	103214183	103214244	-0.29	-0.03
Cpsf2	chr12	103240910	103241994	-0.60	-0.04
Papola	chr12	107022971	107023092	-4.18	-0.34
Papola	chr12	107072945	107075506	-2.32	-0.11
Snw1	chr12	88791126	88791810	-8.21	-0.35
Ddx46	chr13	55741151	55749105	-10.30	-0.37
Hnrnpa0	chr13	58228250	58229917	-61.50	-0.36
Hnrnpk	chr13	58501349	58504674	-3.06	-0.25
Ppwd1	chr13	105010109	105010529	-1.01	0.16
Ppwd1	chr13	105018773	105018924	-0.32	-0.04
Gtf2f2	chr14	76297012	76306651	-1.19	-0.13
Wbp4	chr14	79861599	79863753	-7.89	-0.67
Polr2k	chr15	36103800	36104751	-0.34	0.08
Puf60	chr15	75900611	75900909	-0.30	0.00
Ddx23	chr15	98475937	98476385	-4.86	-0.28
Ddx23	chr15	98488768	98493320	-11.90	-0.70
Ncbp2	chr16	31957240	31957480	-1.22	-0.23
Tra2b	chr16	22265852	22266002	-2.23	-0.43
Srrm2	chr17	23952780	23953201	-31.90	-0.37
Srrm2	chr17	23953650	23955539	-13.20	0.12
Srrm2	chr17	23957486	23958086	-12.30	0.23
Rnps1	chr17	24551619	24552074	-1.31	-0.33
Dhx16	chr17	36019018	36019798	-2.95	-0.31
Ppil1	chr17	29387927	29388668	-0.82	-0.12
Hnrnpm	chr17	33786662	33789227	-12.90	-0.29
Hnrnpm	chr17	33814217	33822403	-1.55	-0.10
Cdc5l	chr17	45528957	45530123	-2.27	0.27
Cdc5l	chr17	45564949	45570477	-1.04	-0.44
Gtf2f1	chr17	57143372	57143559	-0.74	-0.13
Srsf7	chr17	80606178	80606645	-0.35	-0.03
Polr2d	chr18	31955877	31956355	-0.34	-0.02

Wdr33	chr18	31963710	31986988	-10.60	-0.69
Wdr33	chr18	32047426	32048936	-16.50	-0.52
Wdr33	chr18	32049204	32067113	-1.24	-0.09
Txnl4a	chr18	80403957	80409392	-5.60	-0.13
Txnl4a	chr18	80415452	80419302	-1.93	-0.17
Txnl4a	chr18	80421035	80421843	-0.77	-0.27
Cpsf7	chr19	10614133	10620757	-1.51	0.08
Cpsf7	chr19	10621625	10621806	-12.40	0.88
Prpf19	chr19	10969781	10969902	-0.74	-0.17
Cstf2t	chr19	31157390	31159177	-2.75	0.13
Sart1	chr19	5381212	5381273	-0.82	-0.17
Sart1	chr19	5382852	5383166	-1.19	-0.14
Sart1	chr19	5384663	5388703	-2.03	-0.15
Polr2g	chr19	8872986	8873047	-0.44	-0.14
Smndc1	chr19	53454959	53455080	-1.53	0.23
Gpkow	chrX	7285241	7285480	-4.10	0.65
Hnrnph2	chrX	131137272	131140187	-17.40	0.37
Upf3b	chrX	34632248	34632639	-0.43	-0.05

Class I MHC mediated antigen processing & presentation

gene_name	chr	chromStart	chromEnd	diff.lg.p	diff.log2.fc
Ube2f	chr1	93182063	93182602	-2.18	-0.17
Asb1	chr1	93437321	93437716	-0.36	-0.19
Asb1	chr1	93440810	93443681	-2.13	-0.63
Asb1	chr1	93451344	93451764	-1.52	0.40
Ncf2	chr1	154654984	154658055	-3.20	-0.20
Hecw2	chr1	53869614	53883756	-0.93	1.33
Hecw2	chr1	53982405	53983513	-0.56	-0.34
Rnf25	chr1	74640325	74640975	-9.13	-0.51
Cul3	chr1	80263601	80265851	-6.24	0.34
Cul3	chr1	80336915	80337005	-0.92	-0.29
Trip12	chr1	84721087	84721998	-0.90	-0.07
Trip12	chr1	84782695	84836815	-20.50	-0.26
Klhl20	chr1	163019017	163019228	-1.31	-0.43
Klhl20	chr1	163020426	163023835	-2.61	0.52
Klhl20	chr1	163039357	163039897	-3.74	-0.46
Anapc2	chr2	25128463	25128911	-3.00	-0.30
Fbxw5	chr2	25358984	25359776	-3.57	-0.40
Fbxw5	chr2	25360013	25360422	-1.07	-0.24
Hspa5	chr2	34629658	34629748	-39.30	-0.48
Hspa5	chr2	34630750	34631960	-27.90	-0.12
Klhl41	chr2	69508683	69508923	-1.52	0.86
Itgav	chr2	83564583	83564973	-10.50	-1.02
Itgav	chr2	83643447	83643718	-3.67	-0.49

Itgav	chr2	83643807	83643987	-0.74	-0.16
Psmc3	chr2	90894439	90895182	-3.33	-0.20
Pdia3	chr2	121263353	121263680	-0.63	0.03
B2m	chr2	121973422	121973632	-11.70	-0.24
Trim69	chr2	122004226	122004644	-0.30	-0.80
Itch	chr2	154959216	154959307	-0.27	-0.02
Itch	chr2	155050102	155050403	-0.31	-0.01
Rnf114	chr2	167318622	167318832	-1.07	-1.19
Asb6	chr2	30678647	30680212	-9.57	-0.39
Lrsam1	chr2	32781993	32782383	-0.31	-0.02
Fbxw2	chr2	34661048	34661869	-4.78	-0.39
Fbxw2	chr2	34662764	34664040	-0.64	-0.16
Fbxw2	chr2	34668436	34678672	-6.10	-0.35
Fbxw2	chr2	34681468	34681725	-0.46	0.13
Psmd5	chr2	34707993	34709823	-1.65	-0.19
Psmd5	chr2	34726274	34726482	-4.41	-0.82
Psmb7	chr2	38498946	38499426	-3.13	-0.36
Anapc1	chr2	128436507	128438637	-1.24	-0.11
Ubox5	chr2	130416996	130417566	-1.55	-0.37
Ubox5	chr2	130425355	130426405	-1.35	-0.24
Psmf1	chr2	151543111	151543202	-1.31	-0.87
Psmf1	chr2	151543649	151544038	-0.30	0.01
Fbxw7	chr3	84619498	84694070	-0.58	-0.12
Fbxw7	chr3	84694129	84756226	-0.51	0.05
Fbxw7	chr3	84780110	84781955	-0.35	0.02
Ube2q1	chr3	89577530	89577890	-0.54	-0.06
Ube2q1	chr3	89585705	89587919	-0.80	-0.04
Rnf115	chr3	96592580	96594008	-0.38	-0.02
Sec22b	chr3	97705149	97711158	-2.16	-0.33
Sec22b	chr3	97725110	97725468	-2.27	-0.20
Psma5	chr3	108059873	108060022	-0.64	3.39
Ube2d3	chr3	135101656	135102228	-19.60	-0.75
Siah2	chr3	58479649	58495381	-3.84	-0.27
Siah2	chr3	58495620	58496010	-0.94	0.28
Psmb4	chr3	94690701	94690880	-8.50	-0.41
Psmd4	chr3	94836626	94836894	-2.54	-0.16
Psmd4	chr3	94840526	94846467	-8.21	-0.35
Fcgr1	chr3	96087130	96087191	-0.26	0.03
Fcgr1	chr3	96087728	96088506	-8.72	-0.57
Sec24b	chr3	129686102	129687101	-1.82	-0.14
Sec24b	chr3	129743865	129763735	-15.50	-0.56
Fbxl4	chr4	22303713	22313289	-1.60	-0.28
Ube2r2	chr4	41137807	41138136	-0.41	-0.03

Trim32	chr4	65274645	65274706	-0.48	-0.20
Trim32	chr4	65274854	65276169	-0.55	-0.05
Lrrc41	chr4	115761466	115762183	-23.20	-0.55
Lrrc41	chr4	115768961	115769201	-3.73	-0.33
Rnf19b	chr4	128735514	128735993	-17.90	-1.08
Rnf19b	chr4	128759998	128761505	-99.00	-1.37
Klhl21	chr4	151383209	151383749	-20.10	-1.89
Klhl21	chr4	151383868	151389959	-10.20	-0.52
Klhl21	chr4	151390257	151390438	-3.61	1.07
Ube2j2	chr4	155330537	155331768	-9.18	-0.43
Ufl1	chr4	25177588	25177709	-0.30	-0.02
Fbxo10	chr4	45048045	45048374	-1.12	1.75
Fbxo10	chr4	45048642	45048793	-0.22	0.14
Fbxo10	chr4	45071323	45071981	-0.27	-0.03
Cdc26	chr4	62055639	62055998	-0.37	-0.07
Klhl9	chr4	88366049	88367814	-9.57	0.24
Rnf220	chr4	116961136	116968779	-2.43	-0.37
Cdc20	chr4	118109887	118109948	-0.76	-0.68
Spsb1	chr4	149271916	149281307	-0.79	-0.50
Mib2	chr4	155028580	155028821	-0.32	-0.05
Ube3c	chr5	29895781	29895962	-0.89	-0.61
Ube3c	chr5	30001057	30001238	-1.45	-0.31
Rnf4	chr5	34694045	34694196	-2.72	-0.31
Anapc4	chr5	53257704	53257973	-1.23	-0.22
Klhl5	chr5	65558211	65558721	-0.75	-0.12
Ube3b	chr5	114830704	114830795	-0.26	0.26
Ube3b	chr5	114869593	114869952	-0.90	0.14
Fbxo21	chr5	118452063	118458618	-6.36	-0.76
Anapc7	chr5	122891328	122891538	-0.73	0.15
Anapc7	chr5	122893905	122894532	-19.20	-0.92
Rnf34	chr5	123273869	123314504	-0.81	0.09
Psmd9	chr5	123678228	123678437	-0.34	0.03
Kctd7	chr5	130628217	130628785	-0.53	-0.24
Fbxl5	chr5	44142150	44149098	-2.88	-0.56
Fbxl5	chr5	44149396	44151973	-0.84	0.08
Rchy1	chr5	92391825	92392094	-0.33	0.04
Sec31a	chr5	100790907	100791058	-0.72	-0.09
Fbxw8	chr5	118515497	118515797	-0.28	0.02
Fbxw8	chr5	118605107	118605467	-13.80	-1.01
Anapc5	chr5	123271138	123271348	-2.63	-0.39
Ubc	chr5	125866453	125867671	-172.00	0.73
Ubc	chr5	125867938	125868147	-3.15	0.20
Ubc	chr5	125868235	125870298	-22.80	-0.40

Ncf1	chr5	134697302	134697663	-2.36	-0.12
Fbxl18	chr5	143633766	143634006	-6.05	-1.06
Fbxl18	chr5	143647153	143648949	-184.00	-2.23
Smurf1	chr5	145637663	145638682	-0.50	0.08
Smurf1	chr5	145639310	145639521	-0.66	0.35
Rnf6	chr5	147021793	147022181	-6.60	0.51
Rnf6	chr5	147023075	147031998	-2.47	0.16
Cul1	chr6	47404345	47445165	-3.56	0.31
Cul1	chr6	47474617	47476108	-1.64	-0.15
Herc6	chr6	57445639	57531152	-0.66	-0.46
Kbtbd8	chr6	95071991	95077327	-2.49	0.37
Vhl	chr6	113578147	113579978	-0.46	-0.06
Fbxl14	chr6	119429685	119431451	-0.36	-0.01
Spsb2	chr6	124758812	124760455	-90.80	-1.86
Klhl42	chr6	147040014	147050109	-0.97	-0.28
Klhl42	chr6	147050168	147056940	-3.38	-0.59
Ube2h	chr6	30163827	30164395	-1.91	0.24
Ube2h	chr6	30164783	30164933	-0.40	-0.15
Vamp8	chr6	72335214	72335448	-3.83	-0.35
Vamp8	chr6	72338374	72340603	-0.85	-0.14
Uba3	chr6	97134556	97134676	-1.20	0.47
Herc2	chr7	63485072	63486928	-1.61	0.14
Ube3a	chr7	66484418	66484628	-2.76	-0.49
Ube3a	chr7	66531169	66540150	-0.51	-0.02
Klhl25	chr7	82993313	83012167	-0.30	0.00
Klhl25	chr7	83012375	83018269	-2.48	-0.60
Klhl25	chr7	83018657	83018926	-0.33	0.09
Rbbp6	chr7	130114077	130114468	-5.64	-0.37
Rbbp6	chr7	130133635	130133876	-1.64	0.20
Rbbp6	chr7	130140555	130140795	-1.26	0.15
Rbbp6	chr7	130142061	130145876	-2.57	0.06
Fbxl19	chr7	134890288	134891744	-4.33	-0.90
Fbxl19	chr7	134891803	134891893	-0.66	-0.35
Fbxl19	chr7	134893270	134904820	-11.30	-0.67
Fbxl19	chr7	134906111	134911817	-1.15	-0.18
Fbxl19	chr7	134911876	134912442	-2.21	-0.45
Stx4a	chr7	134985321	134985978	-1.03	-0.12
Ube2m	chr7	13623018	13623619	-6.02	-0.45
Psmd8	chr7	29965393	29965573	-13.80	-0.43
Asb7	chr7	73792534	73823980	-3.67	-0.42
Asb7	chr7	73834207	73834387	-0.58	0.31
Det1	chr7	85972688	85972809	-1.10	-0.51
Det1	chr7	85988146	85988866	-0.56	-0.07

Trim21	chr7	109707151	109710434	-1.56	0.22
Cdc16	chr8	13779263	13781763	-4.99	-0.38
Sh3rf1	chr8	63704632	63808042	-0.38	0.08
Sh3rf1	chr8	63863616	63863767	-0.29	-0.10
Sh3rf1	chr8	63872471	63873071	-0.74	-0.31
Fbxl8	chr8	107790925	107791573	-0.65	0.30
Fbxl8	chr8	107791922	107793108	-0.55	0.10
Znrf1	chr8	114060539	114062275	-0.69	0.05
Znrf1	chr8	114143127	114147028	-1.41	-0.07
Gan	chr8	119728191	119729086	-7.70	0.65
Lonrf1	chr8	37280167	37282962	-0.30	-0.01
Lonrf1	chr8	37311699	37312090	-2.20	-1.14
Klhl2	chr8	67219391	67219870	-0.73	-0.15
Calr	chr8	87366315	87366863	-41.40	-0.26
Calr	chr8	87370234	87370800	-11.20	-0.26
Siah1a	chr8	89248786	89269816	-0.36	-0.02
Psmb10	chr8	108461631	108461967	-2.80	-0.63
Psmd7	chr8	110104338	110108077	-4.03	0.12
Fbxo31	chr8	124075878	124076147	-0.29	0.02
Fbxo22	chr9	55066270	55071672	-1.24	0.12
Fbxo22	chr9	55071791	55072031	-0.53	0.10
Herc1	chr9	66198256	66220501	-4.31	0.30
Herc1	chr9	66293942	66296296	-2.94	0.33
Herc1	chr9	66298665	66298935	-1.74	0.27
Anapc13	chr9	102528625	102532216	-1.16	-0.23
Dcaf1	chr9	106738991	106740882	-3.19	0.55
Dcaf1	chr9	106760577	106761417	-1.12	0.15
Dcaf1	chr9	106776469	106782364	-1.52	0.38
Fbxl12	chr9	20448880	20449151	-0.85	-0.48
Keap1	chr9	21034473	21038380	-0.55	0.03
Keap1	chr9	21041615	21042276	-1.55	-0.22
Keap1	chr9	21043205	21043686	-0.40	0.13
Ube4a	chr9	44731508	44732078	-3.85	0.39
Cul5	chr9	53425530	53429279	-5.02	0.34
Rnf111	chr9	70274555	70277390	-0.83	-0.10
Rnf111	chr9	70323650	70350792	-12.70	0.37
Uba5	chr9	103949273	103951621	-4.43	0.27
Rnf123	chr9	107954002	107954243	-6.46	0.71
Arih2	chr9	108506380	108506471	-9.95	5.42
Arih2	chr9	108546363	108547287	-1.88	-0.31
Trim71	chr9	114422031	114422092	-0.38	2.58
Trim71	chr9	114422299	114422658	-0.97	5.09
Fbxo30	chr10	11001127	11015345	-10.80	0.40

Herc4	chr10	62706723	62726819	-16.00	-0.60
Ube2g2	chr10	77107280	77107637	-1.33	-0.14
Ube2g2	chr10	77107992	77108082	-0.37	-0.04
Cdc34	chr10	79144939	79145322	-5.03	-0.46
Cdc34	chr10	79148043	79151143	-18.90	-0.38
Rnf41	chr10	127874437	127875829	-14.90	-0.48
Rnf217	chr10	31328190	31329531	-5.20	-0.76
Ube2d1	chr10	70718144	70718592	-2.63	0.38
Rnf126	chr10	79221259	79223613	-5.73	-0.22
Rnf126	chr10	79229508	79229686	-0.71	-0.18
Fzr1	chr10	80829682	80830217	-4.88	0.37
Psme4	chr11	30671774	30672135	-0.54	-0.18
Fbxw11	chr11	32644536	32644986	-0.78	-0.08
Rnf130	chr11	49933551	49933881	-0.38	0.41
Trim11	chr11	58791617	58791916	-2.16	-0.42
Trim11	chr11	58802336	58802515	-0.47	-0.11
Trim11	chr11	58802902	58804960	-1.42	-0.11
Ube2g1	chr11	72420792	72421032	-3.90	-0.86
Ube2g1	chr11	72493612	72497295	-0.33	0.03
Blmh	chr11	76799996	76800265	-6.75	-0.58
Psmd3	chr11	98543867	98546829	-30.30	-0.74
Psme3	chr11	101177564	101178336	-0.62	0.07
Psme3	chr11	101182070	101183288	-0.64	-0.06
Trim41	chr11	48620443	48622633	-2.33	-0.15
Trim41	chr11	48629367	48630893	-3.55	-0.21
Canx	chr11	50109707	50110776	-30.20	-0.37
Sec24a	chr11	51556985	51570140	-6.97	-0.33
Wsb1	chr11	79053599	79053839	-11.70	-0.39
Ube2z	chr11	95911494	95911704	-1.74	-0.23
Fbxl20	chr11	97950510	97953462	-0.74	0.16
Klhl11	chr11	100323925	100325737	-6.64	0.31
Klhl11	chr11	100333798	100334096	-2.00	0.50
Cdc27	chr11	104366175	104368590	-6.73	0.30
Smurf2	chr11	106683743	106684388	-0.37	-0.04
Smurf2	chr11	106781609	106781789	-3.23	-1.30
Ube2o	chr11	116399633	116399872	-0.36	0.05
Ube2o	chr11	116400110	116404120	-7.85	-0.32
Ube2o	chr11	116405875	116406055	-1.26	0.30
Ube2o	chr11	116442641	116442761	-0.21	0.26
Socs3	chr11	117827669	117830291	-196.00	-0.95
Lrr1	chr12	70275442	70275972	-0.48	-0.36
Psmc1	chr12	101348377	101351316	-1.50	-0.23
Btbd6	chr12	114215520	114217031	-10.90	-0.80

Arel1	chr12	86259755	86261648	-4.64	-0.19
Arel1	chr12	86311686	86311836	-0.86	0.32
Rnf182	chr13	43763214	43763781	-1.24	5.46
Mylip	chr13	45485140	45499872	-7.91	0.67
Mylip	chr13	45503785	45506564	-28.20	0.99
Rnf144b	chr13	47338305	47340700	-1.08	-0.22
Rnf144b	chr13	47342881	47343181	-0.33	-0.08
Erap1	chr13	74777319	74784015	-3.77	0.34
Kctd6	chr14	9052713	9055575	-1.94	-0.30
Sec24c	chr14	21512428	21513175	-11.10	-0.51
Sec24c	chr14	21513593	21514072	-11.40	-0.48
Psmc6	chr14	45949498	45950514	-0.60	-0.09
Kbtbd7	chr14	79827156	79828746	-2.42	0.38
Fbxl3	chr14	103482090	103488631	-0.87	-0.07
Fbxl3	chr14	103491640	103498113	-0.65	0.09
Fbxl3	chr14	103498172	103498726	-0.67	-0.17
Ncf4	chr15	78092626	78092951	-7.16	-0.30
Rbx1	chr15	81305971	81306061	-1.28	-0.57
Skp2	chr15	9043329	9043510	-0.95	0.41
Skp2	chr15	9069207	9070137	-7.83	-1.04
Rnf19a	chr15	36170946	36173866	-1.80	-0.21
Rnf19a	chr15	36195033	36212902	-6.05	0.56
Fbxo32	chr15	58011902	58012112	-0.40	-0.84
Fbxo32	chr15	58012410	58013039	-1.36	-1.07
Fbxo32	chr15	58046223	58046433	-0.22	-0.12
Asb8	chr15	97965969	97971744	-1.49	-0.17
Mgrn1	chr16	4936923	4937342	-0.32	-0.01
Klhl22	chr16	17771956	17777358	-0.41	-0.03
Psmd2	chr16	20651724	20652324	-20.60	-0.49
Itgb5	chr16	33829750	33830079	-4.10	-1.27
Itgb5	chr16	33919922	33920221	-1.38	-0.29
Itgb5	chr16	33947843	33949245	-1.75	-0.23
Cblb	chr16	52204625	52205075	-5.73	-0.89
Socs1	chr16	10783990	10784912	-0.46	-0.26
Socs1	chr16	10784970	10785629	-0.59	-1.60
Dtx3l	chr16	35928994	35932076	-0.66	-0.06
Dtx3l	chr16	35932345	35933542	-0.72	-0.06
Ltn1	chr16	87415807	87416587	-1.51	0.15
Tapbp	chr17	34056422	34056928	-18.20	-0.66
Tapbp	chr17	34062443	34063061	-65.40	-0.74
Tap1	chr17	34324500	34325221	-16.90	-0.87
Tap1	chr17	34333599	34333840	-0.55	-0.11
Psmb8	chr17	34335288	34335468	-2.22	-0.33

Tap2	chr17	34341453	34342537	-9.79	-0.80
Tap2	chr17	34352342	34353206	-4.14	-0.42
H2-D1	chr17	35400038	35400558	-64.10	-0.68
H2-D1	chr17	35400926	35402744	-4.71	-0.33
H2-D1	chr17	35403816	35404204	-71.80	-0.36
H2-Q2	chr17	35479307	35479570	-0.14	0.42
H2-Q6	chr17	35561821	35562745	-5.87	-0.37
H2-Q6	chr17	35565949	35566720	-1.19	-0.34
H2-Q7	chr17	35576925	35577015	-0.36	-0.83
H2-Q10	chr17	35607880	35610039	-0.46	-0.18
Cul7	chr17	46788096	46788277	-2.58	3.86
Cul7	chr17	46788516	46788967	-0.83	-0.64
Cul7	chr17	46791019	46791200	-0.63	1.18
Lnpep	chr17	17715497	17746283	-1.10	-0.05
Ccnf	chr17	24360594	24360954	-5.87	0.78
Ccnf	chr17	24361042	24361933	-12.00	1.09
Stub1	chr17	25967580	25967909	-3.51	-0.27
Stub1	chr17	25969947	25970008	-0.31	-0.35
Stub1	chr17	25970096	25970306	-1.82	-0.63
H2-K1	chr17	34133105	34133495	-21.60	-0.51
H2-K1	chr17	34136728	34137188	-34.60	-0.71
H2-T23	chr17	36169189	36248130	-6.88	-0.55
Trim39	chr17	36397225	36398520	-0.49	0.05
Trim39	chr17	36400794	36404080	-0.93	-0.33
Ubr2	chr17	47065869	47067369	-3.79	-0.22
Ubr2	chr17	47147270	47147481	-5.21	-0.65
Pja2	chr17	64658065	64658365	-1.01	-0.18
Pja2	chr17	64658514	64660528	-2.68	-0.35
Fbxo11	chr17	88390945	88391686	-0.95	0.09
Fbxo11	chr17	88464385	88464625	-1.02	-0.30
Ube2d2a	chr18	35931242	35931392	-3.18	-0.65
Ube2d2a	chr18	35965479	35965570	-0.43	0.27
Rnf14	chr18	38467690	38467990	-0.56	0.10
Mex3c	chr18	73732538	73732629	-1.20	1.10
Mex3c	chr18	73732718	73732899	-1.18	-2.10
Mex3c	chr18	73732988	73750582	-0.64	0.04
Cdc23	chr18	34792901	34793291	-0.68	-0.16
Cdc23	chr18	34793470	34793621	-0.76	-0.25
Trim36	chr18	46326839	46328996	-8.68	1.58
Btrc	chr19	45604117	45604298	-0.65	1.17
Fbxl15	chr19	46403649	46404936	-3.45	-0.46
Fbxw4	chr19	45652866	45653256	-0.75	-0.11
Ube2a	chrX	34414360	34414420	-1.18	-0.44

Huwe1	chrX	148353194	148354606	-2.14	0.09
Pja1	chrX	96661699	96661909	-2.76	0.63
Pja1	chrX	96662087	96663013	-1.08	0.12
Pja1	chrX	96663072	96663789	-0.87	-0.14
Rlim	chrX	101156612	101157212	-16.70	0.50
Rlim	chrX	101157331	101160637	-88.80	0.50
Siah1b	chrX	160509638	160510081	-0.33	0.04
Uba6	chr5	86541279	86547113	-0.45	0.04

Chromatin modifying enzymes

gene_name	chr	chromStart	chromEnd	diff.lg.p	diff.log2.fc
Ing5	chr1	95713260	95715951	-3.10	0.35
Rbbp5	chr1	134394620	134400982	-0.63	-0.07
Rbbp5	chr1	134401101	134401282	-1.08	0.24
Kdm5b	chr1	136514466	136514617	-0.28	0.01
Kdm5b	chr1	136521370	136521700	-0.83	-0.21
Kdm5b	chr1	136525318	136527418	-2.33	-0.37
Kdm5b	chr1	136527777	136528227	-0.52	0.09
Ncoa2	chr1	13132595	13132716	-0.72	0.30
Ncoa2	chr1	13157056	13165408	-0.76	-0.08
Kansl3	chr1	36392574	36393263	-1.90	-0.22
Smyd3	chr1	181325708	181325888	-0.91	-0.55
Smyd3	chr1	181447984	181448134	-0.35	0.09
Smyd2	chr1	191704578	191704728	-0.49	0.06
Phf21a	chr2	92199286	92200964	-5.15	-0.63
Phf20	chr2	156093020	156093171	-0.35	-0.03
Phf20	chr2	156124429	156134043	-2.43	-0.18
Suv39h2	chr2	3379880	3382119	-3.13	-0.56
Suv39h2	chr2	3392047	3392228	-0.56	-0.36
Ehmt1	chr2	24646742	24647852	-3.96	-0.36
Ehmt1	chr2	24733063	24775080	-0.58	-0.06
Atf2	chr2	73656947	73657068	-4.98	1.16
Tbl1xr1	chr3	22108479	22109775	-1.17	0.16
Jade1	chr3	41408675	41418304	-0.43	-0.05
Ash1l	chr3	88754285	88786291	-3.01	0.13
Ash1l	chr3	88786679	88786860	-5.29	0.65
Ash1l	chr3	88787428	88787759	-0.78	0.09
Ash1l	chr3	88787997	88788567	-0.40	-0.02
Ash1l	chr3	88789076	88805778	-1.34	0.10
Ash1l	chr3	88811104	88811794	-0.80	0.06
Ash1l	chr3	88876505	88881290	-5.13	0.33
Gatad2b	chr3	90127990	90145879	-1.31	-0.15
Gatad2b	chr3	90155787	90155908	-1.99	-0.28
Hist2h2be	chr3	96025043	96025490	-2.37	-0.78

Hist2h2be	chr3	96026708	96027215	-0.64	-0.19
Hist2h2be	chr3	96027422	96027483	-10.00	3.73
Hist2h2bb	chr3	96073622	96073710	-3.96	0.30
Setd7	chr3	51324890	51325369	-1.63	-0.33
Setd7	chr3	51354202	51364745	-0.38	0.06
Setdb1	chr3	95127865	95128046	-1.98	0.38
Setdb1	chr3	95130188	95132524	-2.29	0.19
Setdb1	chr3	95142274	95142843	-1.02	0.11
Hist2h3c2	chr3	96042365	96042604	-1.05	0.63
Prmt6	chr3	110050616	110051517	-0.57	-0.23
Prmt6	chr3	110052356	110053916	-1.37	-0.11
Nfkb1	chr3	135248247	135252299	-9.58	-0.45
Nfkb1	chr3	135354240	135354451	-1.35	0.77
Kdm4a	chr4	117809906	117810206	-0.58	-0.09
Kdm4a	chr4	117810444	117811478	-2.22	-0.15
Kdm4a	chr4	117834383	117834444	-0.70	-0.16
Arid1a	chr4	133235940	133241652	-2.63	-0.08
Arid1a	chr4	133276907	133312385	-11.90	-0.45
Kdm1a	chr4	136106537	136106717	-0.88	-0.22
Kmt2e	chr5	22940246	22940516	-2.58	0.46
Kmt2e	chr5	22998122	22999338	-0.91	0.13
Kmt2e	chr5	23002790	23005303	-1.02	0.08
Kmt2e	chr5	23007352	23008999	-1.84	0.10
Nsd2	chr5	34163512	34188823	-4.19	-0.35
Nsd2	chr5	34197314	34203748	-2.07	-0.19
Nsd2	chr5	34236231	34238007	-7.34	-0.90
Rest	chr5	77694578	77697550	-2.09	0.21
Rest	chr5	77709792	77712154	-14.20	0.31
Kmt5a	chr5	124909965	124910818	-10.10	-0.42
Kmt5a	chr5	124911146	124911447	-1.43	0.14
Smarcd3	chr5	24100691	24100989	-0.59	-0.63
Kmt2c	chr5	24790138	24790949	-2.35	-0.28
Kmt2c	chr5	24804711	24805371	-6.01	0.48
Kmt2c	chr5	24805490	24806151	-3.45	0.35
Kmt2c	chr5	24815764	24816754	-4.22	0.34
Kmt2c	chr5	24819800	24820700	-2.61	0.23
Kmt2c	chr5	24820849	24821690	-2.45	0.22
Kmt2c	chr5	24878616	24880356	-8.08	0.57
Suds3	chr5	117542584	117542973	-0.58	-0.07
Suds3	chr5	117556655	117565972	-3.08	0.27
Kdm2b	chr5	123321403	123321793	-4.39	-0.89
Kdm2b	chr5	123330581	123330851	-4.56	-0.82
Kdm2b	chr5	123382481	123382872	-1.53	-0.69

Ncor2	chr5	125497524	125500255	-17.90	-0.42
Ncor2	chr5	125504014	125509323	-4.66	-0.22
Ncor2	chr5	125517648	125517739	-5.60	-1.05
Ncor2	chr5	125518279	125531358	-10.10	-0.27
Ncor2	chr5	125538304	125548306	-0.58	0.07
Ncor2	chr5	125659493	125659584	-0.45	-0.84
Brpf1	chr6	113257130	113267099	-6.11	-0.18
Brpf1	chr6	113267573	113269353	-20.40	-0.58
Brpf1	chr6	113272417	113274519	-13.10	-0.60
Kdm5a	chr6	120314116	120314656	-2.00	-0.22
Kdm5a	chr6	120369491	120377857	-0.82	0.08
Kdm5a	chr6	120380152	120389527	-0.45	0.02
Chd4	chr6	125046180	125050907	-20.10	-0.46
Chd4	chr6	125079888	125080399	-1.95	-0.09
Atf7ip	chr6	136508513	136509952	-2.54	0.19
Atf7ip	chr6	136535654	136536194	-2.69	0.26
Atf7ip	chr6	136553991	136555660	-3.72	0.40
H2afj	chr6	136757307	136757517	-2.15	-0.48
Aebp2	chr6	140571543	140572383	-0.56	-0.13
Aebp2	chr6	140572622	140573517	-1.76	0.25
Aebp2	chr6	140602621	140602891	-4.77	0.69
Kdm7a	chr6	39092726	39096881	-9.89	-0.51
Kdm3a	chr6	71539443	71540465	-0.63	-0.13
Kdm3a	chr6	71553984	71554074	-1.00	-0.37
Kdm3a	chr6	71557565	71562002	-1.04	-0.13
Kmt5c	chr7	4697833	4698936	-8.14	-0.81
Prmt3	chr7	57112858	57113008	-1.15	-0.24
Setd1a	chr7	134920902	134921023	-1.37	0.88
Setd1a	chr7	134927635	134930735	-0.69	-0.03
Setd1a	chr7	134931750	134932028	-1.01	-0.16
Setd1a	chr7	134934941	134940915	-7.31	-0.23
Kat8	chr7	135056030	135056241	-0.62	0.21
Kmt2b	chr7	31359377	31361794	-20.90	-0.55
Kmt2b	chr7	31368445	31373745	-14.90	-0.34
Eed	chr7	97125556	97129068	-6.88	-0.52
Eed	chr7	97129276	97129396	-0.46	-0.10
Ccnd1	chr7	152118471	152118592	-5.59	-0.32
Ccnd1	chr7	152125559	152125830	-6.58	-0.44
Kat6a	chr8	23970010	24013683	-5.12	0.22
Kat6a	chr8	24040759	24043005	-0.60	-0.06
Kat6a	chr8	24046218	24051490	-0.77	-0.03
Nsd3	chr8	26751014	26760027	-9.52	-0.28
Nsd3	chr8	26770213	26773659	-12.50	-0.38

Nsd3	chr8	26787194	26787704	-0.82	0.14
Setd6	chr8	98242227	98242844	-7.85	0.69
Ash2l	chr8	26950536	26958082	-1.11	-0.21
Sap30	chr8	59966154	59966657	-121.00	-1.09
Gatad2a	chr8	72433183	72435724	-28.20	-0.49
Gatad2a	chr8	72459749	72459840	-2.26	-0.26
Gatad2a	chr8	72498029	72498150	-0.37	-0.11
Gatad2a	chr8	72519918	72520248	-1.28	-0.35
Carm1	chr9	21392499	21393190	-2.99	-0.18
Smarca4	chr9	21505589	21507505	-3.30	0.23
H2afx	chr9	44143446	44144156	-0.77	-0.05
Msl2	chr9	100978450	101004495	-56.70	0.47
Setd2	chr9	110449720	110450170	-9.17	0.57
Setd2	chr9	110450318	110450409	-7.23	0.92
Setd2	chr9	110450707	110452085	-26.00	0.57
Setd2	chr9	110452863	110453971	-25.90	0.60
Setd2	chr9	110474472	110497130	-14.90	0.36
Kmt2a	chr9	44612636	44614167	-3.81	0.25
Kmt2a	chr9	44615605	44617182	-1.06	0.16
Kmt2a	chr9	44625809	44629185	-31.10	0.48
Kmt2a	chr9	44629634	44630414	-7.20	0.44
Kmt2a	chr9	44655111	44656174	-7.56	0.48
Kmt2a	chr9	44656473	44656924	-1.40	0.24
Kmt2a	chr9	44657403	44658123	-21.00	0.79
Dot1l	chr10	80217950	80218251	-0.81	-0.19
Dot1l	chr10	80248250	80248979	-14.90	-0.56
Dot1l	chr10	80251442	80254428	-43.50	-0.51
Dot1l	chr10	80254516	80254877	-1.29	-0.37
Dot1l	chr10	80255415	80255536	-0.38	-0.11
Dot1l	chr10	80256224	80257034	-1.45	-0.13
Smarcc2	chr10	127920261	127920695	-0.29	0.00
Smarcc2	chr10	127924543	127924604	-1.40	-0.59
Smarcc2	chr10	127926123	127926244	-0.38	0.05
Arid5b	chr10	67558669	67564649	-4.34	0.19
Smarcb1	chr10	75359602	75359780	-1.07	-0.22
Mbd3	chr10	79855285	79856691	-7.71	-0.28
Hmg20b	chr10	80808790	80809707	-2.62	-0.27
Hmg20b	chr10	80812903	80813172	-3.43	-0.88
Sap30l	chr11	57615138	57615764	-2.06	-0.47
Sap30l	chr11	57621583	57623760	-2.35	-0.42
Hist3h2a	chr11	58768661	58768929	-4.41	0.77
Suz12	chr11	79843530	79845978	-0.38	0.01
Msl1	chr11	98657082	98662163	-4.65	-0.18

Jade2	chr11	51627973	51629289	-1.03	-0.09
Jade2	chr11	51629617	51641772	-8.80	-0.29
Ncor1	chr11	62130880	62131511	-1.35	0.12
Ncor1	chr11	62147246	62147337	-0.41	-0.06
Ncor1	chr11	62182924	62186997	-26.60	-0.65
Ncor1	chr11	62196845	62198278	-2.94	-0.40
Ncor1	chr11	62252002	62270948	-5.64	-1.11
Chd3	chr11	69157463	69157973	-5.22	-0.48
Chd3	chr11	69174956	69175626	-0.72	-0.14
Chd3	chr11	69177569	69182868	-4.44	-0.54
Kdm6b	chr11	69215611	69216507	-7.42	-0.90
Kdm6b	chr11	69217076	69220555	-132.00	-0.95
Kdm6b	chr11	69221801	69227145	-14.00	-0.92
Kat7	chr11	95171349	95171530	-2.36	-0.40
Kansl1	chr11	104195981	104196912	-10.80	0.49
Kansl1	chr11	104240093	104282447	-3.35	0.53
Kansl1	chr11	104285084	104303164	-6.67	0.19
Kansl1	chr11	104303433	104303584	-0.37	-0.10
Smarcd2	chr11	106133897	106134286	-1.29	-0.39
Jmjd6	chr11	116698745	116699073	-12.00	-0.56
Jmjd6	chr11	116703794	116704674	-36.40	-0.84
Dnmt3a	chr12	3806989	3807238	-0.61	0.39
Dnmt3a	chr12	3835069	3866198	-0.42	-0.04
Dnmt3a	chr12	3906804	3908006	-0.38	0.01
Arid4a	chr12	72116983	72117799	-0.63	0.17
Arid4a	chr12	72172813	72187641	-63.40	0.85
Rcor1	chr12	112348029	112350550	-3.71	0.25
Mta1	chr12	114374970	114375239	-4.02	-0.39
Ncoa1	chr12	4247600	4248409	-2.05	-0.34
Ncoa1	chr12	4248946	4249007	-0.88	-0.53
Ncoa1	chr12	4249095	4252804	-0.98	-0.20
Ncoa1	chr12	4267739	4267919	-1.13	-0.35
Ncoa1	chr12	4295010	4295788	-1.68	-0.24
Ncoa1	chr12	4295907	4296027	-0.69	-0.27
Setd3	chr12	109345414	109346800	-0.69	0.07
Arid4b	chr13	14156058	14156269	-4.11	0.77
Arid4b	chr13	14256598	14256659	-1.88	0.71
Arid4b	chr13	14262361	14270284	-5.09	1.05
Arid4b	chr13	14276698	14283617	-21.70	0.56
Arid4b	chr13	14285592	14285892	-13.90	1.34
Arid4b	chr13	14287645	14290373	-3.83	0.49
Hist1h3h	chr13	21809496	21809698	-1.42	0.30
Hist1h3f	chr13	23636303	23636393	-2.07	-0.34

Hist1h2bg	chr13	23663268	23663474	-1.13	-0.09
Hist1h3d	chr13	23667608	23667786	-1.12	-0.25
Hist1h2bc	chr13	23776067	23776213	-2.98	0.39
Hist1h2ab	chr13	23842956	23843254	-0.48	0.06
Hist1h3i	chr13	21875180	21875266	-0.41	0.06
Hist1h3c	chr13	23837329	23837390	-0.31	-0.03
Phf2	chr13	48898195	48899711	-2.91	-0.42
Phf2	chr13	48912894	48913224	-0.82	-0.19
Kat6b	chr14	22335875	22336775	-1.32	-0.23
Kat6b	chr14	22481439	22481590	-1.74	0.81
Kat6b	chr14	22483848	22490337	-22.50	0.74
Sap18	chr14	58417025	58420413	-1.79	0.96
Sap18	chr14	58423488	58423817	-16.80	2.55
Prmt5	chr14	55126018	55126553	-0.91	-0.11
Ep300	chr15	81416643	81438767	-12.50	-0.29
Ep300	chr15	81479273	81481819	-5.01	-0.13
Arid2	chr15	96117952	96118043	-0.28	-0.02
Arid2	chr15	96199968	96201175	-7.90	0.36
Arid2	chr15	96201654	96209326	-6.30	0.26
Arid2	chr15	96223066	96232715	-1.11	-0.22
Brd1	chr15	88518753	88518814	-4.89	0.89
Brd1	chr15	88543169	88543809	-0.49	-0.05
Brd1	chr15	88547455	88564529	-12.40	0.36
Kansl2	chr15	98349531	98349829	-0.51	0.11
Riox2	chr16	59471901	59472260	-0.88	0.36
Riox2	chr16	59475394	59475634	-0.29	0.00
Brwd1	chr16	96214898	96214959	-2.85	1.53
Brwd1	chr16	96223575	96225521	-4.61	-0.24
Brwd1	chr16	96226300	96226615	-0.56	-1.29
Arid1b	chr17	4995073	4995882	-1.66	-0.67
Arid1b	chr17	4996061	5097577	-3.74	-0.55
Arid1b	chr17	5332607	5337057	-2.55	0.24
Arid1b	chr17	5337116	5339263	-0.52	0.10
Arid1b	chr17	5341518	5343345	-1.18	0.09
Brpf3	chr17	28938100	28938251	-0.36	0.50
Brpf3	chr17	28943116	28944224	-7.70	0.75
Brpf3	chr17	28954048	28960704	-0.96	0.16
Brpf3	chr17	28973668	28973878	-0.46	0.17
Ehmt2	chr17	35042119	35042557	-5.05	-0.58
Ehmt2	chr17	35049712	35050992	-0.63	0.07
Kdm4b	chr17	56540721	56542296	-3.45	-0.36
Kdm3b	chr18	34963125	34963814	-1.47	-0.15
Kdm3b	chr18	34964262	34970049	-2.22	0.15

Kdm3b	chr18	34997287	34998156	-2.95	-0.28
Hdac3	chr18	38096953	38097223	-2.67	-0.36
Kmt5b	chr19	3767600	3767661	-0.34	-0.27
Kmt5b	chr19	3807323	3807503	-1.22	0.32
Kmt5b	chr19	3808821	3816505	-0.32	0.00
Brms1	chr19	5040306	5040901	-8.72	-0.88
Brms1	chr19	5041286	5046457	-6.09	-0.41
Brms1	chr19	5049008	5049917	-6.88	-0.45
Rela	chr19	5646968	5647773	-3.54	-0.20
Smarca2	chr19	26705324	26705445	-2.19	-1.03
Nfkb2	chr19	46379436	46380290	-0.39	0.13
Kdm2a	chr19	4316774	4316984	-0.68	0.10
Kdm2a	chr19	4318748	4320217	-7.26	-0.27
Kdm2a	chr19	4322495	4326851	-6.01	-0.26
Kdm2a	chr19	4342850	4345606	-11.10	-0.46
Kdm2a	chr19	4396362	4396722	-9.34	-0.65
Kdm6a	chrX	17828012	17831752	-0.52	0.06
Jade3	chrX	20088210	20094873	-65.90	1.93
Tbl1x	chrX	74905095	74905514	-2.36	0.22
Ogt	chrX	98874610	98878315	-1.22	-0.06
Ogt	chrX	98878792	98878973	-1.73	-0.32
Kdm5c	chrX	148667772	148667863	-0.29	-0.02
Kdm5c	chrX	148705250	148708180	-8.68	-0.23
Suv39h1	chrX	7647858	7649470	-1.89	-0.30
Hcfc1	chrX	71189242	71189693	-2.65	0.19
Hcfc1	chrX	71194710	71196030	-1.82	-0.08
Hcfc1	chrX	71211533	71211654	-0.42	0.04
Kdm5d	chrY	277702	278211	-0.89	0.17
Uty	chrY	494590	494860	-3.94	0.62

Chromatin organization

gene_name	chr	chromStart	chromEnd	diff.lg.p	diff.log2.fc
Ing5	chr1	95713260	95715951	-3.10	0.35
Rbbp5	chr1	134394620	134400982	-0.63	-0.07
Rbbp5	chr1	134401101	134401282	-1.08	0.24
Kdm5b	chr1	136514466	136514617	-0.28	0.01
Kdm5b	chr1	136521370	136521700	-0.83	-0.21
Kdm5b	chr1	136525318	136527418	-2.33	-0.37
Kdm5b	chr1	136527777	136528227	-0.52	0.09
Ncoa2	chr1	13132595	13132716	-0.72	0.30
Ncoa2	chr1	13157056	13165408	-0.76	-0.08
Kansl3	chr1	36392574	36393263	-1.90	-0.22
Smyd3	chr1	181325708	181325888	-0.91	-0.55
Smyd3	chr1	181447984	181448134	-0.35	0.09

Smyd2	chr1	191704578	191704728	-0.49	0.06
Phf21a	chr2	92199286	92200964	-5.15	-0.63
Phf20	chr2	156093020	156093171	-0.35	-0.03
Phf20	chr2	156124429	156134043	-2.43	-0.18
Suv39h2	chr2	3379880	3382119	-3.13	-0.56
Suv39h2	chr2	3392047	3392228	-0.56	-0.36
Ehmt1	chr2	24646742	24647852	-3.96	-0.36
Ehmt1	chr2	24733063	24775080	-0.58	-0.06
Atf2	chr2	73656947	73657068	-4.98	1.16
Tbl1xr1	chr3	22108479	22109775	-1.17	0.16
Jade1	chr3	41408675	41418304	-0.43	-0.05
Ash1l	chr3	88754285	88786291	-3.01	0.13
Ash1l	chr3	88786679	88786860	-5.29	0.65
Ash1l	chr3	88787428	88787759	-0.78	0.09
Ash1l	chr3	88787997	88788567	-0.40	-0.02
Ash1l	chr3	88789076	88805778	-1.34	0.10
Ash1l	chr3	88811104	88811794	-0.80	0.06
Ash1l	chr3	88876505	88881290	-5.13	0.33
Gatad2b	chr3	90127990	90145879	-1.31	-0.15
Gatad2b	chr3	90155787	90155908	-1.99	-0.28
Hist2h2be	chr3	96025043	96025490	-2.37	-0.78
Hist2h2be	chr3	96026708	96027215	-0.64	-0.19
Hist2h2be	chr3	96027422	96027483	-10.00	3.73
Hist2h2bb	chr3	96073622	96073710	-3.96	0.30
Setd7	chr3	51324890	51325369	-1.63	-0.33
Setd7	chr3	51354202	51364745	-0.38	0.06
Setdb1	chr3	95127865	95128046	-1.98	0.38
Setdb1	chr3	95130188	95132524	-2.29	0.19
Setdb1	chr3	95142274	95142843	-1.02	0.11
Hist2h3c2	chr3	96042365	96042604	-1.05	0.63
Prmt6	chr3	110050616	110051517	-0.57	-0.23
Prmt6	chr3	110052356	110053916	-1.37	-0.11
Nfkb1	chr3	135248247	135252299	-9.58	-0.45
Nfkb1	chr3	135354240	135354451	-1.35	0.77
Kdm4a	chr4	117809906	117810206	-0.58	-0.09
Kdm4a	chr4	117810444	117811478	-2.22	-0.15
Kdm4a	chr4	117834383	117834444	-0.70	-0.16
Arid1a	chr4	133235940	133241652	-2.63	-0.08
Arid1a	chr4	133276907	133312385	-11.90	-0.45
Kdm1a	chr4	136106537	136106717	-0.88	-0.22
Kmt2e	chr5	22940246	22940516	-2.58	0.46
Kmt2e	chr5	22998122	22999338	-0.91	0.13
Kmt2e	chr5	23002790	23005303	-1.02	0.08

Kmt2e	chr5	23007352	23008999	-1.84	0.10
Nsd2	chr5	34163512	34188823	-4.19	-0.35
Nsd2	chr5	34197314	34203748	-2.07	-0.19
Nsd2	chr5	34236231	34238007	-7.34	-0.90
Rest	chr5	77694578	77697550	-2.09	0.21
Rest	chr5	77709792	77712154	-14.20	0.31
Kmt5a	chr5	124909965	124910818	-10.10	-0.42
Kmt5a	chr5	124911146	124911447	-1.43	0.14
Smarcd3	chr5	24100691	24100989	-0.59	-0.63
Kmt2c	chr5	24790138	24790949	-2.35	-0.28
Kmt2c	chr5	24804711	24805371	-6.01	0.48
Kmt2c	chr5	24805490	24806151	-3.45	0.35
Kmt2c	chr5	24815764	24816754	-4.22	0.34
Kmt2c	chr5	24819800	24820700	-2.61	0.23
Kmt2c	chr5	24820849	24821690	-2.45	0.22
Kmt2c	chr5	24878616	24880356	-8.08	0.57
Suds3	chr5	117542584	117542973	-0.58	-0.07
Suds3	chr5	117556655	117565972	-3.08	0.27
Kdm2b	chr5	123321403	123321793	-4.39	-0.89
Kdm2b	chr5	123330581	123330851	-4.56	-0.82
Kdm2b	chr5	123382481	123382872	-1.53	-0.69
Ncor2	chr5	125497524	125500255	-17.90	-0.42
Ncor2	chr5	125504014	125509323	-4.66	-0.22
Ncor2	chr5	125517648	125517739	-5.60	-1.05
Ncor2	chr5	125518279	125531358	-10.10	-0.27
Ncor2	chr5	125538304	125548306	-0.58	0.07
Ncor2	chr5	125659493	125659584	-0.45	-0.84
Brpf1	chr6	113257130	113267099	-6.11	-0.18
Brpf1	chr6	113267573	113269353	-20.40	-0.58
Brpf1	chr6	113272417	113274519	-13.10	-0.60
Kdm5a	chr6	120314116	120314656	-2.00	-0.22
Kdm5a	chr6	120369491	120377857	-0.82	0.08
Kdm5a	chr6	120380152	120389527	-0.45	0.02
Chd4	chr6	125046180	125050907	-20.10	-0.46
Chd4	chr6	125079888	125080399	-1.95	-0.09
Atf7ip	chr6	136508513	136509952	-2.54	0.19
Atf7ip	chr6	136535654	136536194	-2.69	0.26
Atf7ip	chr6	136553991	136555660	-3.72	0.40
H2afj	chr6	136757307	136757517	-2.15	-0.48
Aebp2	chr6	140571543	140572383	-0.56	-0.13
Aebp2	chr6	140572622	140573517	-1.76	0.25
Aebp2	chr6	140602621	140602891	-4.77	0.69
Kdm7a	chr6	39092726	39096881	-9.89	-0.51

Kdm3a	chr6	71539443	71540465	-0.63	-0.13
Kdm3a	chr6	71553984	71554074	-1.00	-0.37
Kdm3a	chr6	71557565	71562002	-1.04	-0.13
Kmt5c	chr7	4697833	4698936	-8.14	-0.81
Prmt3	chr7	57112858	57113008	-1.15	-0.24
Setd1a	chr7	134920902	134921023	-1.37	0.88
Setd1a	chr7	134927635	134930735	-0.69	-0.03
Setd1a	chr7	134931750	134932028	-1.01	-0.16
Setd1a	chr7	134934941	134940915	-7.31	-0.23
Kat8	chr7	135056030	135056241	-0.62	0.21
Kmt2b	chr7	31359377	31361794	-20.90	-0.55
Kmt2b	chr7	31368445	31373745	-14.90	-0.34
Eed	chr7	97125556	97129068	-6.88	-0.52
Eed	chr7	97129276	97129396	-0.46	-0.10
Ccnd1	chr7	152118471	152118592	-5.59	-0.32
Ccnd1	chr7	152125559	152125830	-6.58	-0.44
Kat6a	chr8	23970010	24013683	-5.12	0.22
Kat6a	chr8	24040759	24043005	-0.60	-0.06
Kat6a	chr8	24046218	24051490	-0.77	-0.03
Nsd3	chr8	26751014	26760027	-9.52	-0.28
Nsd3	chr8	26770213	26773659	-12.50	-0.38
Nsd3	chr8	26787194	26787704	-0.82	0.14
Setd6	chr8	98242227	98242844	-7.85	0.69
Ash2l	chr8	26950536	26958082	-1.11	-0.21
Sap30	chr8	59966154	59966657	-121.00	-1.09
Gatad2a	chr8	72433183	72435724	-28.20	-0.49
Gatad2a	chr8	72459749	72459840	-2.26	-0.26
Gatad2a	chr8	72498029	72498150	-0.37	-0.11
Gatad2a	chr8	72519918	72520248	-1.28	-0.35
Carm1	chr9	21392499	21393190	-2.99	-0.18
Smarca4	chr9	21505589	21507505	-3.30	0.23
H2afx	chr9	44143446	44144156	-0.77	-0.05
Msl2	chr9	100978450	101004495	-56.70	0.47
Setd2	chr9	110449720	110450170	-9.17	0.57
Setd2	chr9	110450318	110450409	-7.23	0.92
Setd2	chr9	110450707	110452085	-26.00	0.57
Setd2	chr9	110452863	110453971	-25.90	0.60
Setd2	chr9	110474472	110497130	-14.90	0.36
Kmt2a	chr9	44612636	44614167	-3.81	0.25
Kmt2a	chr9	44615605	44617182	-1.06	0.16
Kmt2a	chr9	44625809	44629185	-31.10	0.48
Kmt2a	chr9	44629634	44630414	-7.20	0.44
Kmt2a	chr9	44655111	44656174	-7.56	0.48

Kmt2a	chr9	44656473	44656924	-1.40	0.24
Kmt2a	chr9	44657403	44658123	-21.00	0.79
Dot1l	chr10	80217950	80218251	-0.81	-0.19
Dot1l	chr10	80248250	80248979	-14.90	-0.56
Dot1l	chr10	80251442	80254428	-43.50	-0.51
Dot1l	chr10	80254516	80254877	-1.29	-0.37
Dot1l	chr10	80255415	80255536	-0.38	-0.11
Dot1l	chr10	80256224	80257034	-1.45	-0.13
Smarcc2	chr10	127920261	127920695	-0.29	0.00
Smarcc2	chr10	127924543	127924604	-1.40	-0.59
Smarcc2	chr10	127926123	127926244	-0.38	0.05
Arid5b	chr10	67558669	67564649	-4.34	0.19
Smarcb1	chr10	75359602	75359780	-1.07	-0.22
Mbd3	chr10	79855285	79856691	-7.71	-0.28
Hmg20b	chr10	80808790	80809707	-2.62	-0.27
Hmg20b	chr10	80812903	80813172	-3.43	-0.88
Sap30l	chr11	57615138	57615764	-2.06	-0.47
Sap30l	chr11	57621583	57623760	-2.35	-0.42
Hist3h2a	chr11	58768661	58768929	-4.41	0.77
Suz12	chr11	79843530	79845978	-0.38	0.01
Msl1	chr11	98657082	98662163	-4.65	-0.18
Jade2	chr11	51627973	51629289	-1.03	-0.09
Jade2	chr11	51629617	51641772	-8.80	-0.29
Ncor1	chr11	62130880	62131511	-1.35	0.12
Ncor1	chr11	62147246	62147337	-0.41	-0.06
Ncor1	chr11	62182924	62186997	-26.60	-0.65
Ncor1	chr11	62196845	62198278	-2.94	-0.40
Ncor1	chr11	62252002	62270948	-5.64	-1.11
Chd3	chr11	69157463	69157973	-5.22	-0.48
Chd3	chr11	69174956	69175626	-0.72	-0.14
Chd3	chr11	69177569	69182868	-4.44	-0.54
Kdm6b	chr11	69215611	69216507	-7.42	-0.90
Kdm6b	chr11	69217076	69220555	-132.00	-0.95
Kdm6b	chr11	69221801	69227145	-14.00	-0.92
Kat7	chr11	95171349	95171530	-2.36	-0.40
Kansl1	chr11	104195981	104196912	-10.80	0.49
Kansl1	chr11	104240093	104282447	-3.35	0.53
Kansl1	chr11	104285084	104303164	-6.67	0.19
Kansl1	chr11	104303433	104303584	-0.37	-0.10
Smarcd2	chr11	106133897	106134286	-1.29	-0.39
Jmjd6	chr11	116698745	116699073	-12.00	-0.56
Jmjd6	chr11	116703794	116704674	-36.40	-0.84
Dnmt3a	chr12	3806989	3807238	-0.61	0.39

Dnmt3a	chr12	3835069	3866198	-0.42	-0.04
Dnmt3a	chr12	3906804	3908006	-0.38	0.01
Arid4a	chr12	72116983	72117799	-0.63	0.17
Arid4a	chr12	72172813	72187641	-63.40	0.85
Rcor1	chr12	112348029	112350550	-3.71	0.25
Mta1	chr12	114374970	114375239	-4.02	-0.39
Ncoa1	chr12	4247600	4248409	-2.05	-0.34
Ncoa1	chr12	4248946	4249007	-0.88	-0.53
Ncoa1	chr12	4249095	4252804	-0.98	-0.20
Ncoa1	chr12	4267739	4267919	-1.13	-0.35
Ncoa1	chr12	4295010	4295788	-1.68	-0.24
Ncoa1	chr12	4295907	4296027	-0.69	-0.27
Setd3	chr12	109345414	109346800	-0.69	0.07
Arid4b	chr13	14156058	14156269	-4.11	0.77
Arid4b	chr13	14256598	14256659	-1.88	0.71
Arid4b	chr13	14262361	14270284	-5.09	1.05
Arid4b	chr13	14276698	14283617	-21.70	0.56
Arid4b	chr13	14285592	14285892	-13.90	1.34
Arid4b	chr13	14287645	14290373	-3.83	0.49
Hist1h3h	chr13	21809496	21809698	-1.42	0.30
Hist1h3f	chr13	23636303	23636393	-2.07	-0.34
Hist1h2bg	chr13	23663268	23663474	-1.13	-0.09
Hist1h3d	chr13	23667608	23667786	-1.12	-0.25
Hist1h2bc	chr13	23776067	23776213	-2.98	0.39
Hist1h2ab	chr13	23842956	23843254	-0.48	0.06
Hist1h3i	chr13	21875180	21875266	-0.41	0.06
Hist1h3c	chr13	23837329	23837390	-0.31	-0.03
Phf2	chr13	48898195	48899711	-2.91	-0.42
Phf2	chr13	48912894	48913224	-0.82	-0.19
Kat6b	chr14	22335875	22336775	-1.32	-0.23
Kat6b	chr14	22481439	22481590	-1.74	0.81
Kat6b	chr14	22483848	22490337	-22.50	0.74
Sap18	chr14	58417025	58420413	-1.79	0.96
Sap18	chr14	58423488	58423817	-16.80	2.55
Prmt5	chr14	55126018	55126553	-0.91	-0.11
Ep300	chr15	81416643	81438767	-12.50	-0.29
Ep300	chr15	81479273	81481819	-5.01	-0.13
Arid2	chr15	96117952	96118043	-0.28	-0.02
Arid2	chr15	96199968	96201175	-7.90	0.36
Arid2	chr15	96201654	96209326	-6.30	0.26
Arid2	chr15	96223066	96232715	-1.11	-0.22
Brd1	chr15	88518753	88518814	-4.89	0.89
Brd1	chr15	88543169	88543809	-0.49	-0.05

Brd1	chr15	88547455	88564529	-12.40	0.36
Kansl2	chr15	98349531	98349829	-0.51	0.11
Riox2	chr16	59471901	59472260	-0.88	0.36
Riox2	chr16	59475394	59475634	-0.29	0.00
Brwd1	chr16	96214898	96214959	-2.85	1.53
Brwd1	chr16	96223575	96225521	-4.61	-0.24
Brwd1	chr16	96226300	96226615	-0.56	-1.29
Arid1b	chr17	4995073	4995882	-1.66	-0.67
Arid1b	chr17	4996061	5097577	-3.74	-0.55
Arid1b	chr17	5332607	5337057	-2.55	0.24
Arid1b	chr17	5337116	5339263	-0.52	0.10
Arid1b	chr17	5341518	5343345	-1.18	0.09
Brpf3	chr17	28938100	28938251	-0.36	0.50
Brpf3	chr17	28943116	28944224	-7.70	0.75
Brpf3	chr17	28954048	28960704	-0.96	0.16
Brpf3	chr17	28973668	28973878	-0.46	0.17
Ehmt2	chr17	35042119	35042557	-5.05	-0.58
Ehmt2	chr17	35049712	35050992	-0.63	0.07
Kdm4b	chr17	56540721	56542296	-3.45	-0.36
Kdm3b	chr18	34963125	34963814	-1.47	-0.15
Kdm3b	chr18	34964262	34970049	-2.22	0.15
Kdm3b	chr18	34997287	34998156	-2.95	-0.28
Hdac3	chr18	38096953	38097223	-2.67	-0.36
Kmt5b	chr19	3767600	3767661	-0.34	-0.27
Kmt5b	chr19	3807323	3807503	-1.22	0.32
Kmt5b	chr19	3808821	3816505	-0.32	0.00
Brms1	chr19	5040306	5040901	-8.72	-0.88
Brms1	chr19	5041286	5046457	-6.09	-0.41
Brms1	chr19	5049008	5049917	-6.88	-0.45
Rela	chr19	5646968	5647773	-3.54	-0.20
Smarca2	chr19	26705324	26705445	-2.19	-1.03
Nfkb2	chr19	46379436	46380290	-0.39	0.13
Kdm2a	chr19	4316774	4316984	-0.68	0.10
Kdm2a	chr19	4318748	4320217	-7.26	-0.27
Kdm2a	chr19	4322495	4326851	-6.01	-0.26
Kdm2a	chr19	4342850	4345606	-11.10	-0.46
Kdm2a	chr19	4396362	4396722	-9.34	-0.65
Kdm6a	chrX	17828012	17831752	-0.52	0.06
Jade3	chrX	20088210	20094873	-65.90	1.93
Tbl1x	chrX	74905095	74905514	-2.36	0.22
Ogt	chrX	98874610	98878315	-1.22	-0.06
Ogt	chrX	98878792	98878973	-1.73	-0.32
Kdm5c	chrX	148667772	148667863	-0.29	-0.02

Kdm5c	chrX	148705250	148708180	-8.68	-0.23
Suv39h1	chrX	7647858	7649470	-1.89	-0.30
Hcfc1	chrX	71189242	71189693	-2.65	0.19
Hcfc1	chrX	71194710	71196030	-1.82	-0.08
Hcfc1	chrX	71211533	71211654	-0.42	0.04
Kdm5d	chrY	277702	278211	-0.89	0.17
Uty	chrY	494590	494860	-3.94	0.62

TNF-signaling

gene_name	chr	chromStart	chromEnd	diff.lg.p	diff.log2.fc
Casp8	chr1	58890568	58901646	-12.80	-0.51
Usp21	chr1	173216496	173218062	-11.50	-1.09
Traf2	chr2	25374427	25375145	-2.44	-0.26
Traf1	chr2	34798777	34802204	-45.80	-0.96
Traf1	chr2	34803536	34803955	-12.30	-0.99
Traf1	chr2	34813569	34814068	-7.79	-1.05
Madd	chr2	90977546	90977726	-1.15	-0.52
Madd	chr2	91018210	91018420	-1.85	-0.53
Otud7b	chr3	95908449	95908540	-1.47	-0.65
Otud7b	chr3	95956546	95960193	-6.78	-0.26
Map3k7	chr4	32051081	32051232	-3.02	-1.01
Map3k7	chr4	32104109	32107267	-5.97	0.38
Map3k7	chr4	32110051	32110231	-0.27	0.00
Ubc	chr5	125866453	125867671	-172.00	0.73
Ubc	chr5	125867938	125868147	-3.15	0.20
Ubc	chr5	125868235	125870298	-22.80	-0.40
Tnfrsf1a	chr6	125311560	125312501	-18.50	-0.45
Fadd	chr7	151765572	151768192	-4.39	-0.39
Cyld	chr8	91220956	91226218	-0.50	0.37
Cyld	chr8	91226307	91233793	-2.31	0.21
Ikbkb	chr8	23770606	23771027	-1.32	-0.20
Bag4	chr8	26877798	26878878	-1.37	-0.17
Tradd	chr8	107782474	107783499	-1.93	-0.23
Tradd	chr8	107783873	107788494	-0.92	-0.28
Smpd3	chr8	108789066	108789216	-0.20	0.24
Usp2	chr9	43883786	43884026	-0.81	2.12
Usp2	chr9	43902032	43902302	-0.96	-0.35
Usp4	chr9	108293381	108294591	-3.30	-0.22
Birc2	chr9	7818614	7818734	-0.55	-0.29
Birc2	chr9	7833610	7834657	-0.90	0.13
Birc2	chr9	7834955	7835195	-1.05	0.42
Birc3	chr9	7848969	7850928	-1.17	-0.17
Birc3	chr9	7860311	7872961	-0.30	0.00
Sppl2b	chr10	80329626	80329807	-1.32	-0.66

Sppl2b	chr10	80330135	80331453	-14.20	-0.76
Tab2	chr10	7625983	7626463	-1.79	0.18
Tab2	chr10	7626701	7630910	-3.35	-0.17
Tab2	chr10	7631204	7644574	-1.97	-0.07
Tnfaip3	chr10	18721581	18725137	-0.67	-0.06
Smpd2	chr10	41209967	41210146	-1.95	1.58
Adam17	chr12	21330026	21333946	-5.54	-0.14
Ripk1	chr13	34094265	34094762	-9.02	-0.95
Ripk1	chr13	34119794	34125274	-0.32	0.00
Ripk1	chr13	34125393	34126082	-9.17	-0.86
Tab1	chr15	79989230	79992132	-13.10	-0.63
Otulin	chr15	27535675	27538550	-1.81	-0.14
Otulin	chr15	27553050	27560448	-1.52	-0.31
Sharpin	chr15	76180541	76181540	-3.28	-0.38
Tnf	chr17	35336694	35337384	-434.00	-1.28
Tnf	chr17	35338760	35338941	-117.00	-1.09
Chuk	chr19	44181817	44181937	-0.55	-0.16
Xiap	chrX	39447566	39448377	-1.27	0.11
Xiap	chrX	39453031	39458192	-4.92	0.44
Tab3	chrX	82859477	82860195	-4.67	0.57
Tab3	chrX	82860522	82860673	-1.01	0.46
Tab3	chrX	82873570	82876044	-16.40	2.20

C-type lectin receptors

gene_name	chr	chromStart	chromEnd	diff.lg.p	diff.log2.fc
Casp8	chr1	58890568	58901646	-12.80	-0.51
Psmc3	chr2	90894439	90895182	-3.33	-0.20
Traf6	chr2	101530344	101537929	-2.95	-0.21
Src	chr2	157295136	157296531	-2.35	0.70
Card9	chr2	26212789	26213529	-2.20	-0.22
Psmd5	chr2	34707993	34709823	-1.65	-0.19
Psmd5	chr2	34726274	34726482	-4.41	-0.82
Psmb7	chr2	38498946	38499426	-3.13	-0.36
Il1b	chr2	129190464	129191002	-3.00	1.94
Il1b	chr2	129195984	129196856	-1.12	2.89
Psmf1	chr2	151543111	151543202	-1.31	-0.87
Psmf1	chr2	151543649	151544038	-0.30	0.01
Nfatc2	chr2	168301969	168303229	-1.59	-0.26
Nfatc2	chr2	168304128	168330396	-9.73	-0.53
Nfatc2	chr2	168396287	168427005	-6.54	-0.50
Psma5	chr3	108059873	108060022	-0.64	3.39
Bcl10	chr3	145587232	145587592	-4.99	-0.42
Bcl10	chr3	145588099	145593556	-3.89	-0.41
Psmb4	chr3	94690701	94690880	-8.50	-0.41

Psmd4	chr3	94836626	94836894	-2.54	-0.16
Psmd4	chr3	94840526	94846467	-8.21	-0.35
Nfkb1	chr3	135248247	135252299	-9.58	-0.45
Nfkb1	chr3	135354240	135354451	-1.35	0.77
Prkacb	chr3	146394694	146395023	-0.79	0.13
Lyn	chr4	3605267	3605506	-12.00	-0.40
Lyn	chr4	3716941	3718014	-40.00	-0.36
Map3k7	chr4	32051081	32051232	-3.02	-1.01
Map3k7	chr4	32104109	32107267	-5.97	0.38
Map3k7	chr4	32110051	32110231	-0.27	0.00
Psmd9	chr5	123678228	123678437	-0.34	0.03
Ubc	chr5	125866453	125867671	-172.00	0.73
Ubc	chr5	125867938	125868147	-3.15	0.20
Ubc	chr5	125868235	125870298	-22.80	-0.40
Card11	chr5	141348952	141352360	-0.69	0.26
Cul1	chr6	47404345	47445165	-3.56	0.31
Cul1	chr6	47474617	47476108	-1.64	-0.15
Uba3	chr6	97134556	97134676	-1.20	0.47
Raf1	chr6	115568620	115569280	-0.58	0.06
Kras	chr6	145168813	145168964	-1.16	-0.35
Ube2m	chr7	13623018	13623619	-6.02	-0.45
Calm3	chr7	17502039	17502309	-1.46	-0.09
Relb	chr7	20191690	20192229	-0.83	-0.11
Psmd8	chr7	29965393	29965573	-13.80	-0.43
Hras	chr7	148376788	148378177	-0.34	-0.01
Hras	chr7	148378775	148379903	-0.71	0.21
Prkaca	chr8	86496876	86500855	-1.59	-0.45
Nfatc3	chr8	108602601	108603618	-3.27	0.21
Nfatc3	chr8	108632435	108652196	-8.48	0.41
Plcg2	chr8	120022190	120028105	-0.35	-0.03
Ikbkb	chr8	23770606	23771027	-1.32	-0.20
Psmb10	chr8	108461631	108461967	-2.80	-0.63
Psmd7	chr8	110104338	110108077	-4.03	0.12
Fyn	chr10	39089604	39089994	-0.68	-0.12
Fyn	chr10	39271197	39284102	-0.31	0.00
Cdc34	chr10	79144939	79145322	-5.03	-0.46
Cdc34	chr10	79148043	79151143	-18.90	-0.38
Tab2	chr10	7625983	7626463	-1.79	0.18
Tab2	chr10	7626701	7630910	-3.35	-0.17
Tab2	chr10	7631204	7644574	-1.97	-0.07
Ube2d1	chr10	70718144	70718592	-2.63	0.38
Psme4	chr11	30671774	30672135	-0.54	-0.18
Fbxw11	chr11	32644536	32644986	-0.78	-0.08

Psmd3	chr11	98543867	98546829	-30.30	-0.74
Psme3	chr11	101177564	101178336	-0.62	0.07
Psme3	chr11	101182070	101183288	-0.64	-0.06
Map3k14	chr11	103081077	103082247	-3.53	-0.47
Map3k14	chr11	103100346	103101055	-0.68	-0.14
Map3k14	chr11	103103466	103128715	-0.39	-0.09
Icam2	chr11	106239145	106239263	-0.38	0.20
Psmc1	chr12	101348377	101351316	-1.50	-0.23
Calm1	chr12	101437750	101440606	-16.30	-0.45
Calm1	chr12	101445239	101445449	-3.24	-0.14
Nfkbia	chr12	56593395	56593604	-103.00	-1.36
Rps6ka5	chr12	101789211	101791305	-0.71	0.25
Psmc6	chr14	45949498	45950514	-0.60	-0.09
Ppp3cb	chr14	21320302	21327131	-0.50	-0.04
Ppp3cb	chr14	21365614	21365735	-0.90	-0.41
Prkcd	chr14	31408808	31411699	-1.85	-0.13
Prkcd	chr14	31411787	31411937	-0.73	0.22
Prkcd	chr14	31439273	31439394	-1.07	-0.33
Tab1	chr15	79989230	79992132	-13.10	-0.63
Ep300	chr15	81416643	81438767	-12.50	-0.29
Ep300	chr15	81479273	81481819	-5.01	-0.13
Psmd2	chr16	20651724	20652324	-20.60	-0.49
Crebbp	chr16	4084047	4087773	-0.48	-0.02
Crebbp	chr16	4154961	4213404	-3.27	-0.16
Pak2	chr16	32019862	32021909	-0.84	0.05
Psmb8	chr17	34335288	34335468	-2.22	-0.33
Pdpk1	chr17	24215553	24216093	-11.80	0.63
Calm2	chr17	87835011	87846216	-2.03	-0.14
Ube2d2a	chr18	35931242	35931392	-3.18	-0.65
Ube2d2a	chr18	35965479	35965570	-0.43	0.27
Malt1	chr18	65635658	65636018	-2.72	-0.30
Nfatc1	chr18	80802973	80803394	-0.79	-0.30
Nfatc1	chr18	80803872	80832717	-0.41	0.03
Nfatc1	chr18	80846232	80846653	-2.71	0.51
Nfatc1	chr18	80879052	80904894	-2.97	0.23
Rela	chr19	5646968	5647773	-3.54	-0.20
Btrc	chr19	45604117	45604298	-0.65	1.17
Nfkb2	chr19	46379436	46380290	-0.39	0.13
Chuk	chr19	44181817	44181937	-0.55	-0.16
Tab3	chrX	82859477	82860195	-4.67	0.57
Tab3	chrX	82860522	82860673	-1.01	0.46
Tab3	chrX	82873570	82876044	-16.40	2.20

Table S3 Differentially expressed and differentially methylated genes following *P. aeruginosa* infection

Differentially expressed and differentially methylated genes following 2 h *P. aeruginosa* infection

Gene expression (transcription)

gene_name	chr	chromStart	chromEnd	diff.lg.p	diff.log2.fc
Cnot11	chr1	39592697	39593027	-1.55	-0.43
Cnot11	chr1	39603392	39603662	-0.40	-0.08
Cnot9	chr1	74575438	74575798	-0.28	0.00
Psmd1	chr1	87961193	87961284	-0.42	-0.17
Ing5	chr1	95714871	95715801	-0.28	0.00
Ccnt2	chr1	129698792	129700591	-3.01	-0.19
Rbbp5	chr1	134400352	134400982	-0.53	-0.09
Rbbp5	chr1	134401131	134401252	-1.44	-0.61
Kdm5b	chr1	136521430	136521730	-0.84	0.38
Kdm5b	chr1	136527028	136527418	-2.12	-0.56
Kdm5b	chr1	136527777	136528227	-0.93	-0.32
Exo1	chr1	177829740	177831466	-0.36	0.08
Exo1	chr1	177838863	177839044	-0.53	1.66
Exo1	chr1	177839133	177839433	-0.23	0.32
Parp1	chr1	182499105	182503813	-0.82	-0.22
Gls	chr1	52244565	52244686	-2.51	0.56
Gls	chr1	52289446	52290076	-8.87	-0.68
Sf3b1	chr1	55084201	55084322	-0.39	-0.08
Sumo1	chr1	59696425	59697018	-0.64	0.07
Mdm4	chr1	134888236	134893148	-6.34	0.38
Zc3h11a	chr1	135516447	135517433	-0.36	-0.01
Zc3h11a	chr1	135517641	135518358	-1.03	0.08
Zc3h11a	chr1	135521370	135523781	-8.38	0.35
Rnf2	chr1	153318378	153318528	-1.07	0.48
Smyd2	chr1	191704549	191704728	-0.69	-0.12
Bmi1	chr2	18605667	18606468	-1.54	0.19
Rxra	chr2	27615218	27616386	-3.04	-0.43
Rxra	chr2	27617342	27618510	-1.15	-0.20
Tsc1	chr2	28531258	28532030	-0.42	-0.07
Tsc1	chr2	28541537	28542884	-3.54	-0.53
Abl1	chr2	31544075	31551320	-0.81	-0.11
Abl1	chr2	31653102	31658129	-5.37	-0.32
Abl1	chr2	31659237	31659417	-0.55	0.86
Tnks1bp1	chr2	84891036	84905202	-5.10	-0.39
Nup160	chr2	90575704	90575765	-0.94	0.49
Psmc3	chr2	90894439	90895182	-2.65	-0.24
Cstf3	chr2	104504891	104505342	-1.40	0.33
Polr1b	chr2	128926761	128926881	-0.27	0.02

Polr1b	chr2	128950988	128952063	-2.09	-0.21
Polr3f	chr2	144362969	144364972	-2.94	0.39
Itch	chr2	154959216	154959277	-0.88	-1.17
Itch	chr2	155050162	155050343	-1.21	-0.42
Phf20	chr2	156093050	156093141	-0.58	0.23
Phf20	chr2	156124429	156134013	-1.03	-0.14
Tgif2	chr2	156679159	156679219	-0.37	-2.17
Tgif2	chr2	156679337	156679783	-3.32	-1.45
Src	chr2	157295226	157296441	-0.66	0.35
Srsf6	chr2	162757243	162757817	-16.40	-0.71
Ywhab	chr2	163820932	163837497	-5.11	-0.34
Ywhab	chr2	163842532	163842712	-0.32	-0.01
Trp53rkb	chr2	166620945	166621755	-2.25	-0.66
Cstf1	chr2	172201321	172201638	-1.61	0.32
Cstf1	chr2	172203213	172206586	-4.41	-0.29
Gata3	chr2	9779656	9779806	-0.72	1.20
Gata3	chr2	9796219	9796280	-0.19	-0.10
Pip4k2a	chr2	18765497	18769085	-1.88	0.16
Ehmt1	chr2	24646712	24647822	-1.18	-0.21
Ehmt1	chr2	24733063	24775080	-0.36	-0.02
Gtf3c5	chr2	28421871	28423253	-2.17	0.38
Gtf3c4	chr2	28679945	28680454	-1.64	-0.42
Gtf3c4	chr2	28680992	28681502	-1.39	-0.37
Gtf3c4	chr2	28681621	28683020	-1.47	-0.32
Gtf3c4	chr2	28686134	28690130	-2.28	-0.26
Gtf3c4	chr2	28690338	28692035	-1.80	0.29
Gtf3c4	chr2	28695610	28695850	-0.32	-0.04
Cdk9	chr2	32562910	32565101	-2.08	-0.11
Psmb7	chr2	38498946	38499426	-0.68	-0.14
Nr4a2	chr2	56960235	56961013	-0.57	0.24
Nr4a2	chr2	56964339	56964789	-0.86	0.45
Snrpb	chr2	130002885	130005100	-2.11	-0.26
Pcna	chr2	132078675	132078916	-4.27	-0.37
Aurka	chr2	172182198	172182653	-1.29	-0.37
Taf4	chr2	179647358	179656678	-3.26	-0.45
Skil	chr3	30994042	30997442	-7.15	0.42
Skil	chr3	31015942	31017611	-7.07	-0.67
Gatad2b	chr3	90145610	90145850	-0.34	-0.02
Hist2h2be	chr3	96025043	96025490	-1.90	-0.85
Hist2h2be	chr3	96026738	96027185	-0.90	0.58
Notch2	chr3	97817460	97817641	-5.99	-1.08
Notch2	chr3	97938527	97941254	-1.07	-0.09
Notch2	chr3	97950009	97954260	-9.76	-0.14

Ints12	chr3	132771889	132772517	-1.99	0.29
Ube2d3	chr3	135101656	135102228	-4.65	-0.47
Ube2d3	chr3	135128199	135128439	-18.90	0.59
Gtf2b	chr3	142428210	142434431	-1.06	-0.36
Gtf2b	chr3	142446182	142446450	-0.79	-0.15
Elf2	chr3	51059646	51060780	-0.58	0.05
Elf2	chr3	51061575	51064964	-1.46	-0.26
Ints3	chr3	90195399	90196193	-2.42	-0.34
Ints3	chr3	90237228	90237498	-3.56	-0.95
Chtop	chr3	90303594	90304012	-3.30	-0.22
Chtop	chr3	90313215	90313365	-2.06	-0.28
Psmb4	chr3	94690701	94690880	-7.90	-0.58
Rprd2	chr3	95567509	95570056	-0.48	0.03
Rprd2	chr3	95576704	95577124	-1.32	0.38
Hist2h2aa1	chr3	96044042	96044216	-0.53	0.81
Hist2h4	chr3	96067181	96067240	-3.93	-0.68
Polr3gl	chr3	96382184	96398081	-2.41	0.25
Hipk1	chr3	103547210	103547959	-0.55	0.04
Hipk1	chr3	103581206	103595159	-4.73	0.13
Prmt6	chr3	110050706	110051487	-2.53	-1.82
Prmt6	chr3	110052356	110053856	-3.47	-0.24
Srsf11	chr3	157674772	157675357	-4.58	-0.26
Trp53inp1	chr4	11083587	11092680	-0.35	-0.03
Trp53inp1	chr4	11095044	11097090	-1.21	0.33
Nbn	chr4	15906509	15911111	-1.35	0.31
Nr4a3	chr4	48064136	48064938	-0.26	0.15
Rraga	chr4	86221606	86222233	-1.51	0.17
Rraga	chr4	86222828	86222948	-5.17	1.30
Ndc1	chr4	107083754	107086943	-1.13	0.28
Tal1	chr4	114740895	114741732	-0.36	-0.04
Med8	chr4	118087440	118087589	-4.47	0.79
Rragc	chr4	123598193	123598284	-0.48	0.12
Rragc	chr4	123612802	123613821	-5.74	0.23
Rragc	chr4	123614119	123614180	-0.69	0.27
Phc2	chr4	128404910	128405061	-1.54	-1.36
Phc2	chr4	128425295	128429198	-5.36	-0.28
Phc2	chr4	128429795	128429916	-4.38	-0.59
Srsf4	chr4	131456030	131457610	-5.46	-0.26
Rpa2	chr4	132333796	132334035	-1.66	-0.63
Nr0b2	chr4	133112127	133112304	-0.20	0.07
Zfp46	chr4	135846596	135848571	-0.77	0.15
Mtor	chr4	147822690	147826440	-1.52	-0.59
Mtor	chr4	147930422	147931764	-0.33	0.02

Mllt3	chr4	87419188	87419819	-0.28	0.00
Mllt3	chr4	87486765	87487246	-0.56	-0.13
Zfp69	chr4	120602861	120603310	-0.27	0.03
Zfp69	chr4	120603519	120603669	-0.44	-0.32
Ago1	chr4	126116441	126116622	-0.31	0.06
Ago4	chr4	126167863	126168552	-0.35	-0.08
Ago4	chr4	126169807	126170017	-0.91	1.40
Sesn2	chr4	132048721	132050486	-3.16	-0.43
Sesn2	chr4	132066221	132066371	-4.37	-1.49
Sesn2	chr4	132066234	132066414	-4.63	-1.56
Sfn	chr4	133156530	133157337	-0.32	-0.09
Sfn	chr4	133157425	133158083	-2.14	1.70
Arid1a	chr4	133235970	133241652	-0.91	-0.04
Arid1a	chr4	133276907	133312385	-17.00	-0.64
Srrm1	chr4	134878175	134878295	-1.61	-0.33
Srrm1	chr4	134880837	134881018	-4.45	-0.35
Srrm1	chr4	134902769	134909069	-0.67	0.11
Ski	chr4	154529765	154532213	-0.32	0.00
Ski	chr4	154533975	154596644	-2.32	-0.19
Cdk6	chr5	3519435	3520884	-1.56	-0.25
Kmt2e	chr5	22940246	22940457	-0.51	-0.17
Kmt2e	chr5	22998122	22999368	-4.88	0.59
Kmt2e	chr5	23002969	23005243	-0.85	0.12
Kmt2e	chr5	23007441	23008999	-0.73	0.07
Ywhah	chr5	33361494	33361585	-1.08	0.86
Ywhah	chr5	33369269	33370077	-5.50	0.21
Rbpj	chr5	53981598	53981836	-1.39	-0.35
Rbpj	chr5	54044785	54045056	-0.15	0.35
Rpap2	chr5	108049003	108049513	-2.57	0.65
Mtf2	chr5	108535683	108536043	-2.01	-0.42
Triap1	chr5	115791255	115793381	-2.35	-0.35
Kmt5a	chr5	124889998	124895602	-0.24	-0.33
Kmt5a	chr5	124910517	124910788	-4.25	-0.39
Crcp	chr5	130535783	130536233	-3.95	-0.72
Baz1b	chr5	135663192	135663582	-4.41	-0.88
Baz1b	chr5	135692615	135694113	-17.30	0.47
Baz1b	chr5	135718352	135720711	-0.78	-0.10
Baz1b	chr5	135720770	135721040	-0.61	0.14
Zfp12	chr5	144005811	144007911	-2.24	-0.35
Zfp12	chr5	144007970	144008390	-1.50	-0.69
Zfp12	chr5	144008869	144009619	-2.06	-0.69
Cpsf4	chr5	145939775	145942700	-1.59	0.29
Zkscan5	chr5	145979745	145981214	-1.00	-0.64

Zkscan5	chr5	145982110	145982440	-0.70	0.58
Zfp655	chr5	145992583	145994373	-1.48	-0.63
Zfp655	chr5	146004520	146005030	-4.40	0.44
Zfp655	chr5	146005119	146006108	-11.40	0.68
Zscan25	chr5	146044330	146047272	-1.99	-0.47
Zscan25	chr5	146048248	146052219	-5.54	-0.46
Prkag2	chr5	24369540	24371912	-1.91	-0.73
Prkag2	chr5	24605830	24606370	-0.43	-0.05
Gtf3c2	chr5	31459414	31460596	-10.30	-0.47
Nup54	chr5	92845130	92846543	-2.10	0.37
Ubc	chr5	125866572	125866810	-2.56	0.16
Ubc	chr5	125867255	125867493	-1.59	-7.24
Ubc	chr5	125867789	125868176	-3.53	0.25
Ubc	chr5	125868235	125870298	-1.07	0.10
Pom121	chr5	135853650	135857198	-0.40	0.02
Pom121	chr5	135857525	135857795	-0.86	-0.15
Pom121	chr5	135870057	135870386	-1.39	-0.53
Ywhag	chr5	136385024	136385324	-4.97	-0.24
Ywhag	chr5	136386428	136406621	-22.80	-0.25
Ywhag	chr5	136410271	136410481	-5.25	-0.39
Taf6	chr5	138619844	138620172	-2.00	-0.52
Zkscan14	chr5	145956021	145959592	-1.65	-0.73
Zkscan14	chr5	145962189	145962426	-0.66	-0.46
Cul1	chr6	47404345	47445195	-6.27	0.56
Cul1	chr6	47474617	47476108	-1.79	-0.21
Zfp398	chr6	47785899	47790681	-0.75	0.40
Zfp398	chr6	47815976	47817027	-7.69	-1.08
Zfp282	chr6	47827823	47830376	-0.62	-0.17
Zfp282	chr6	47854677	47854738	-0.57	1.09
Zfp282	chr6	47854827	47855936	-4.61	-0.61
Zfp282	chr6	47856235	47856805	-0.70	-0.21
Nr2c2	chr6	92041411	92042461	-6.65	-2.20
Nr2c2	chr6	92114256	92117932	-0.43	0.07
Brpf1	chr6	113257190	113267069	-0.45	-0.02
Brpf1	chr6	113267573	113269263	-6.04	0.44
Brpf1	chr6	113272477	113274549	-3.83	-0.44
Chd4	chr6	125046180	125050907	-20.70	-0.56
Chd4	chr6	125079918	125080429	-2.75	-0.15
Cdkn1b	chr6	134870988	134871169	-0.43	-0.11
Cdkn1b	chr6	134871258	134871349	-0.54	0.22
Cdkn1b	chr6	134872089	134872300	-0.31	0.05
Aebp2	chr6	140571543	140572353	-1.09	-0.34
Aebp2	chr6	140572772	140573517	-1.23	-0.25

Aebp2	chr6	140602621	140602921	-0.84	0.21
Hipk2	chr6	38647898	38666025	-4.07	-0.19
Hipk2	chr6	38767930	38787047	-1.49	-0.06
Hipk2	chr6	38825859	38826040	-5.88	-0.79
Zfp786	chr6	47769651	47770277	-0.22	0.26
Zfp786	chr6	47770782	47770962	-0.16	-0.17
Zfp786	chr6	47771348	47771617	-0.27	0.46
Zfp746	chr6	48013500	48016503	-1.58	-0.22
Zfp746	chr6	48033553	48036532	-0.36	-0.09
Gadd45a	chr6	66985119	66986711	-1.32	0.30
Gadd45a	chr6	66986829	66987371	-0.61	0.22
Nup210	chr6	90963060	90963600	-0.61	-0.14
Zfp248	chr6	118378735	118380402	-1.67	0.60
Kras	chr6	145168753	145169023	-0.82	0.25
Ints13	chr6	146498363	146498424	-0.40	0.25
U2af2	chr7	5030913	5031003	-2.69	-0.46
Zfp667	chr7	6256343	6256524	-1.23	2.11
Zfp667	chr7	6257719	6257900	-0.55	1.12
Zfp324	chr7	13555702	13556301	-1.93	-1.14
Zfp324	chr7	13556928	13557676	-0.46	-0.29
Ppp1r13l	chr7	19956956	19958711	-6.19	-1.04
Zfp235	chr7	24926820	24927181	-2.93	-1.75
Zfp235	chr7	24927899	24928110	-0.36	0.73
Tgfb1	chr7	26472020	26477400	-2.63	-0.09
Akt2	chr7	28423129	28423428	-0.34	-0.03
Paf1	chr7	29178014	29178133	-0.52	0.20
Paf1	chr7	29181953	29184402	-2.28	-0.16
Zfp30	chr7	30577820	30577971	-0.19	0.01
Zfp30	chr7	30578060	30578271	-0.20	0.17
Zfp30	chr7	30578360	30578780	-0.45	0.65
Zfp420	chr7	30645086	30659464	-0.37	0.20
Zfp420	chr7	30659552	30660089	-1.78	1.00
Zfp420	chr7	30660207	30661190	-0.53	-0.20
Zfp658	chr7	50828018	50828318	-0.28	0.03
Zfp658	chr7	50828587	50830412	-2.41	0.37
Zfp719	chr7	50834985	50846808	-4.97	0.35
Zfp710	chr7	87169699	87227267	-5.56	-0.24
Zfp710	chr7	87231474	87231625	-1.43	-0.54
Zfp710	chr7	87233516	87236145	-0.78	0.08
Setd1a	chr7	134920902	134920963	-0.35	0.61
Setd1a	chr7	134927665	134930073	-0.72	0.05
Setd1a	chr7	134935031	134940855	-4.69	-0.24
Zfp94	chr7	25087200	25088844	-2.03	-0.70

Psmd8	chr7	29965423	29965573	-0.49	-0.05
Zfp940	chr7	30629618	30629679	-0.26	0.84
Zfp940	chr7	30630367	30630458	-0.28	-0.59
Zfp14	chr7	30823226	30823287	-0.16	0.30
Zfp14	chr7	30823733	30823824	-0.28	0.85
Zfp14	chr7	30824091	30824152	-0.53	-3.32
Kmt2b	chr7	31359377	31360985	-9.24	-0.43
Kmt2b	chr7	31368742	31373655	-1.84	-0.11
Med25	chr7	52132343	52133478	-0.35	-0.03
E2f8	chr7	56123117	56123387	-1.32	-0.52
E2f8	chr7	56126321	56127461	-0.65	0.20
Blm	chr7	87647292	87647681	-0.29	-0.01
Blm	chr7	87654407	87658369	-1.75	0.38
Pcf11	chr7	99801691	99806557	-3.02	0.30
Pcf11	chr7	99806795	99808380	-0.45	0.04
Pcf11	chr7	99809716	99810434	-14.40	0.62
Pcf11	chr7	99818062	99818422	-3.92	-0.55
Nup98	chr7	109268003	109269144	-0.31	0.00
Nup98	chr7	109283070	109283401	-1.16	-0.19
Gtf3c1	chr7	132784557	132785187	-2.67	-0.38
Gtf3c1	chr7	132786855	132787843	-1.34	-0.20
Gtf3c1	chr7	132790222	132791069	-0.34	-0.02
Gtf3c1	chr7	132851051	132851202	-0.28	0.00
Zfp747	chr7	134516438	134516707	-1.63	0.98
Zfp747	chr7	134516765	134518917	-1.79	0.31
Zfp747	chr7	134518976	134519385	-1.70	0.70
Zfp688	chr7	134562479	134563593	-0.68	-0.19
Zfp688	chr7	134565458	134565548	-0.42	-0.79
Pidd1	chr7	148624413	148625004	-7.46	-1.58
Pidd1	chr7	148628991	148629907	-7.84	-2.18
Snapc2	chr8	4255370	4256190	-1.18	-0.27
Zfp703	chr8	28087837	28090833	-8.85	-0.41
Ppp2cb	chr8	34710092	34710330	-1.15	-1.30
Ell	chr8	73109589	73114440	-0.85	0.23
Ell	chr8	73115469	73115529	-0.40	-0.20
Polr2c	chr8	97387425	97387604	-0.50	0.11
Cbfb	chr8	107694603	107694841	-2.53	-0.45
Banp	chr8	124550490	124550731	-0.55	0.73
Banp	chr8	124551359	124551959	-0.40	0.41
Banp	chr8	124552018	124552768	-0.66	0.57
Zfpm1	chr8	124806220	124856010	-4.41	-0.70
Zfpm1	chr8	124858912	124861147	-5.69	-0.43
Ash2l	chr8	26950507	26958082	-8.59	-0.94

Cnot7	chr8	41592731	41595397	-0.90	-0.17
Gatad2a	chr8	72433213	72435695	-8.00	-0.37
Gatad2a	chr8	72498059	72498150	-1.29	-1.13
Gatad2a	chr8	72519948	72520248	-1.68	-0.77
Smarca5	chr8	83262939	83263358	-1.96	-0.43
Csnk2a2	chr8	97972031	97972092	-0.28	-0.02
Csnk2a2	chr8	97976954	97977225	-2.32	-0.50
Psmb10	chr8	108461631	108461997	-1.29	-0.51
Psmd7	chr8	110104338	110108137	-2.12	0.12
Dhx38	chr8	112071923	112073723	-0.34	-0.02
Dhx38	chr8	112080295	112080776	-2.84	-0.58
Dhx38	chr8	112084553	112089501	-4.56	-0.33
Taf1c	chr8	122121800	122123360	-2.17	-0.28
Maml2	chr9	13424522	13425152	-3.83	1.67
Maml2	chr9	13510293	13511282	-0.36	-0.08
Taf1d	chr9	15112191	15113253	-27.70	0.70
Pin1	chr9	20456573	20459918	-3.27	-0.67
Smarca4	chr9	21505619	21507475	-7.96	-0.62
Usp2	chr9	43883786	43883906	-0.22	-0.70
Usp2	chr9	43902091	43902272	-0.86	0.94
Ppp2r1b	chr9	50665069	50665957	-0.31	-0.06
Ppp2r1b	chr9	50688020	50688710	-0.69	-0.13
Ppp2r1b	chr9	50701016	50701705	-0.88	-0.20
Ppp2r1b	chr9	50702093	50702154	-0.40	-0.32
Psma4	chr9	54805629	54805837	-1.30	0.21
Sin3a	chr9	56958532	56958892	-4.41	0.69
Sin3a	chr9	56973175	56975606	-1.26	-0.23
Ints14	chr9	64809641	64809791	-1.29	0.57
Ints14	chr9	64833706	64834423	-0.61	-0.09
Leo1	chr9	75289330	75294956	-1.86	-0.19
Topbp1	chr9	103252397	103252458	-0.52	0.32
Ctnnb1	chr9	120842577	120859669	-31.20	-0.94
Ctnnb1	chr9	120867712	120868877	-4.71	-0.16
Zkscan7	chr9	122797666	122797876	-0.37	0.42
Zkscan7	chr9	122803837	122804465	-0.47	0.28
Zkscan7	chr9	122804584	122804943	-0.93	1.07
Zfp426	chr9	20273262	20273323	-0.16	0.32
Zfp426	chr9	20275000	20276171	-0.76	-0.21
Zfp266	chr9	20304209	20306501	-2.59	-0.36
Dnmt1	chr9	20733552	20736587	-2.52	-0.52
Kmt2a	chr9	44612666	44614167	-0.53	0.05
Kmt2a	chr9	44615605	44617152	-1.08	-0.22
Kmt2a	chr9	44625839	44629215	-4.98	0.22

Kmt2a	chr9	44629664	44630384	-1.76	0.23
Kmt2a	chr9	44655111	44656204	-0.78	0.11
Kmt2a	chr9	44656443	44656954	-0.87	0.18
Kmt2a	chr9	44657463	44658123	-2.39	0.29
Atm	chr9	53247503	53248264	-1.23	-0.25
Pml	chr9	58067472	58068881	-4.04	-0.65
Pml	chr9	58080996	58094982	-6.71	-1.05
Pml	chr9	58097382	58097563	-1.72	-0.95
Rnf111	chr9	70274555	70277390	-2.37	-0.34
Rnf111	chr9	70323620	70350792	-3.72	0.24
Tcf12	chr9	71693887	71694367	-2.35	0.29
Tcf12	chr9	71958591	71959626	-1.46	-0.31
Atrip	chr9	108960445	108961784	-3.87	-0.23
Atrip	chr9	108967670	108968495	-0.81	-0.18
Zfp445	chr9	122761016	122763050	-15.30	0.42
Zfp445	chr9	122770871	122775124	-5.50	0.48
Cited2	chr10	17443033	17444883	-17.10	-0.15
Sgk1	chr10	21718603	21718783	-0.46	0.40
Sesn1	chr10	41530439	41607293	-1.75	-0.49
Sesn1	chr10	41627317	41627765	-0.60	-0.29
Ranbp2	chr10	57939345	57939706	-5.21	0.45
Ranbp2	chr10	57939885	57940516	-34.30	1.07
Ranbp2	chr10	57940724	57941055	-4.44	0.51
Ranbp2	chr10	57941564	57941925	-3.73	0.28
Ranbp2	chr10	57942194	57945844	-38.80	0.62
Ranbp2	chr10	57955388	57956812	-8.12	0.45
Dna2	chr10	62436065	62436156	-0.64	-0.63
Stk11	chr10	79579091	79587616	-0.87	0.11
Stk11	chr10	79592416	79593215	-2.75	0.41
Txnrd1	chr10	82296755	82323149	-1.14	-0.46
Txnrd1	chr10	82358858	82358978	-5.22	-0.68
E2f7	chr10	110216009	110216249	-0.59	0.50
Smarcc2	chr10	127919969	127921148	-2.11	0.34
Smarcc2	chr10	127924513	127924663	-0.79	-0.31
Smarcc2	chr10	127926123	127926303	-3.50	0.69
Gtf3c6	chr10	39969066	39969535	-0.30	-0.02
Ddit4	chr10	59412924	59414518	-5.40	-0.83
Ddx21	chr10	62044843	62047419	-27.50	-0.47
Ddx21	chr10	62057824	62061601	-17.60	-0.28
Sirt1	chr10	62783214	62784984	-11.40	0.83
Sirt1	chr10	62801394	62801753	-0.41	-0.23
Smarcb1	chr10	75359690	75359780	-2.90	-0.84
Polrmt	chr10	79201183	79202890	-1.17	-0.24

Polrmt	chr10	79206061	79209326	-2.68	-0.51
Polr2e	chr10	79501286	79502404	-4.55	-0.57
Mbd3	chr10	79855285	79856691	-5.75	-0.31
Tcf3	chr10	79872386	79873314	-1.00	0.16
Tcf3	chr10	79876045	79878019	-0.45	-0.12
Cnot2	chr10	115922276	115922606	-0.54	-0.13
Cnot2	chr10	115922814	115923144	-1.17	0.33
Yeats4	chr10	116652435	116652824	-0.52	0.09
Mdm2	chr10	117126230	117126381	-3.39	-0.39
Mdm2	chr10	117126829	117128239	-1.59	-0.06
Dyrk2	chr10	118296404	118305870	-1.58	-0.15
Cdk2	chr10	128135921	128135982	-0.57	0.46
Thoc5	chr11	4795375	4799907	-4.77	-1.07
Med7	chr11	46250478	46253332	-0.93	-0.36
Med7	chr11	46254098	46254907	-11.40	0.92
Ppp2ca	chr11	51912325	51912474	-1.41	-0.58
Ppp2ca	chr11	51935449	51935895	-9.86	0.36
Aff4	chr11	53164268	53186518	-0.95	0.08
Aff4	chr11	53213109	53213978	-1.33	-0.09
Aff4	chr11	53225500	53229192	-0.43	0.04
Cnot8	chr11	57930988	57931228	-0.44	0.14
Zkscan6	chr11	65636382	65642442	-1.52	0.29
Aurkb	chr11	68861788	68863970	-2.20	-0.36
Trp53	chr11	69393860	69394608	-0.85	-0.23
Mybbp1a	chr11	72264662	72265052	-1.41	-0.12
Ywhae	chr11	75546388	75546567	-15.30	-0.67
Suz12	chr11	79843530	79846008	-5.61	0.37
Psmd11	chr11	80285574	80285635	-0.96	-0.36
Cdk5r1	chr11	80290548	80292661	-29.60	2.17
Cdk5r1	chr11	80293284	80294028	-3.53	1.31
Cdk5r1	chr11	80290547	80292674	-31.50	2.18
Cdk5r1	chr11	80293272	80294202	-4.76	1.41
Taf15	chr11	83286609	83286700	-0.89	-0.29
Ppm1d	chr11	85124815	85125293	-5.70	-1.23
Ppm1d	chr11	85153180	85159827	-2.29	-0.40
Srsf1	chr11	87861172	87861352	-2.10	-0.41
Cdk12	chr11	98064618	98065458	-0.67	-0.06
Cdk12	chr11	98071737	98072546	-5.48	0.30
Cdk12	chr11	98104853	98107097	-2.44	0.32
Cdk12	chr11	98111003	98111753	-1.24	-0.17
Psmd3	chr11	98543867	98546829	-9.56	-0.63
Thra	chr11	98603186	98614709	-0.30	0.09
Thra	chr11	98614768	98614918	-0.78	-0.54

Thra	chr11	98625660	98626199	-2.55	-0.66
Casc3	chr11	98683799	98689940	-0.53	0.05
Casc3	chr11	98692913	98694044	-3.83	-0.49
Rara	chr11	98799009	98808908	-0.34	-0.13
Rara	chr11	98808997	98821970	-6.78	-0.99
Rara	chr11	98822029	98822119	-0.30	-0.42
Rara	chr11	98831630	98835508	-11.80	-0.81
Psme3	chr11	101177564	101177745	-2.61	-0.42
Psme3	chr11	101183108	101183258	-2.98	-0.41
Tnrc6c	chr11	117515962	117562084	-1.99	-1.03
Tnrc6c	chr11	117575542	117593595	-5.36	-0.33
Tnrc6c	chr11	117621843	117622474	-1.27	-0.39
Tnrc6c	chr11	117623163	117623704	-0.38	0.10
Cbx2	chr11	118889233	118890909	-2.18	-0.78
Hus1	chr11	8896711	8897375	-0.49	0.23
Ccng1	chr11	40563841	40563901	-1.93	0.58
Rad50	chr11	53463680	53465739	-0.83	-0.20
Zkscan17	chr11	59299022	59299412	-4.28	0.70
Zkscan17	chr11	59300338	59305712	-3.87	-0.30
Zkscan17	chr11	59319782	59319903	-0.55	-0.48
Top3a	chr11	60553560	60555499	-3.15	-0.90
Top3a	chr11	60555827	60556607	-2.62	-0.75
Ncor1	chr11	62130880	62131481	-2.46	0.27
Ncor1	chr11	62147216	62147307	-0.88	0.25
Ncor1	chr11	62182924	62186997	-1.50	-0.15
Ncor1	chr11	62251972	62270948	-8.07	-1.86
Zfp287	chr11	62527173	62527684	-0.37	-0.15
Zfp287	chr11	62541033	62541184	-0.28	0.59
Zfp287	chr11	62541873	62542444	-2.39	1.37
Zfp287	chr11	62545226	62545317	-0.29	0.62
Chd3	chr11	69157463	69157973	-2.59	-0.35
Chd3	chr11	69175016	69175596	-1.19	-0.32
Chd3	chr11	69177569	69182868	-2.61	-0.40
Supt6	chr11	78020430	78021581	-1.46	-0.13
Supt6	chr11	78047130	78059175	-0.50	0.08
Rffl	chr11	82619321	82631980	-1.42	-0.19
Brip1	chr11	85874744	85875522	-0.94	-0.28
Med13	chr11	86084164	86084555	-6.00	0.92
Med13	chr11	86100091	86100242	-2.66	0.54
Med13	chr11	86104608	86114157	-2.32	0.20
Med13	chr11	86132956	86141386	-0.96	0.10
Pcgf2	chr11	97551210	97551390	-6.81	-1.86
Pip4k2b	chr11	97578204	97583745	-0.53	-0.04

Med1	chr11	98014158	98015024	-0.62	-0.12
Med1	chr11	98015143	98015684	-0.72	0.12
Med1	chr11	98016312	98017303	-0.30	0.00
Med1	chr11	98017662	98017843	-1.54	-0.35
Med1	chr11	98018382	98019702	-0.92	0.08
Med1	chr11	98054214	98054607	-2.46	-0.58
Brca1	chr11	101384784	101385144	-0.45	0.17
Brca1	chr11	101385503	101385983	-2.14	0.68
Brca1	chr11	101386071	101386581	-2.01	-0.62
Brca1	chr11	101386909	101387689	-2.04	0.55
Smarcd2	chr11	106133956	106134286	-1.06	-0.47
Srsf2	chr11	116714108	116714408	-47.20	-0.91
Cbx8	chr11	118899897	118901287	-2.56	-0.91
Cbx4	chr11	118941759	118943921	-4.62	-0.42
Cbx4	chr11	118947103	118947461	-1.29	-1.00
E2f6	chr12	16817770	16817950	-1.20	-0.81
E2f6	chr12	16832231	16832859	-2.21	-0.37
Twistnb	chr12	34121879	34123203	-3.76	0.33
Psma6	chr12	56499811	56499871	-0.49	0.12
Ppm1a	chr12	73862227	73885410	-0.92	-0.08
Snapc1	chr12	75065516	75065726	-2.29	-0.72
Srsf5	chr12	82046490	82046729	-20.10	-0.62
Srsf5	chr12	82050810	82051108	-52.80	-0.87
Cpsf2	chr12	103214183	103220142	-0.41	0.11
Cpsf2	chr12	103240910	103242024	-3.50	0.30
Ccnk	chr12	109418007	109424590	-0.57	0.19
Zfp839	chr12	112088488	112099099	-1.68	-0.40
Zfp839	chr12	112106479	112107344	-1.39	-0.40
Mta1	chr12	114374970	114375239	-5.00	-0.55
Ywhaq	chr12	21422717	21423076	-6.14	-0.76
Max	chr12	78038822	78039688	-0.97	0.07
Max	chr12	78063055	78063205	-1.34	-0.40
Med6	chr12	82674550	82674905	-3.56	0.61
Ppp1r13b	chr12	113066968	113067568	-1.38	0.38
Ppp1r13b	chr12	113070821	113073838	-0.63	0.09
Akt1	chr12	113912217	113912487	-9.20	-1.90
Hist1h3h	chr13	21809496	21809783	-3.88	-0.80
Hist1h3g	chr13	23627286	23627403	-1.47	-0.64
Hist1h3f	chr13	23636303	23636510	-10.20	-0.74
Hist1h2bg	chr13	23663268	23663503	-8.24	-0.53
Hist1h3d	chr13	23666339	23666429	-0.36	-0.31
Hist1h3d	chr13	23667608	23667874	-0.43	-0.08
Hist1h2bc	chr13	23776067	23776242	-3.06	-0.52

Hist1h2bb	chr13	23838602	23838778	-0.32	-0.02
Hist1h2ab	chr13	23842956	23843254	-1.60	-0.36
Jarid2	chr13	44991677	45001942	-0.33	-0.01
Rmi1	chr13	58503698	58503988	-0.70	-0.16
Rmi1	chr13	58508424	58509724	-0.62	0.19
Rmi1	chr13	58509903	58511252	-1.10	0.19
Zfp369	chr13	65397670	65398417	-2.03	-0.44
Med10	chr13	69954555	69954853	-3.65	0.44
Ell2	chr13	75844961	75845081	-0.35	-0.23
Ell2	chr13	75901012	75901521	-0.85	0.08
Taf9	chr13	101435888	101436098	-3.19	0.67
Plk2	chr13	111185251	111185491	-0.79	0.33
Plk2	chr13	111190054	111190991	-5.42	0.83
Slc38a9	chr13	113528209	113528600	-0.23	0.05
Zkscan3	chr13	21479290	21480454	-2.86	0.35
Zkscan3	chr13	21488092	21494265	-3.21	-0.74
Zkscan8	chr13	21611911	21613722	-0.86	-0.17
Zkscan8	chr13	21618398	21618788	-1.44	0.47
Hist1h4k	chr13	21842232	21842374	-8.07	-0.57
Hist1h3i	chr13	21875038	21875266	-4.89	-0.55
Hist1h2ae	chr13	23662883	23663060	-3.46	-0.90
Hist1h3c	chr13	23837239	23837390	-0.55	-0.12
Nup153	chr13	46776228	46776499	-0.79	0.16
Nup153	chr13	46782010	46792068	-6.70	0.24
Nup153	chr13	46822768	46823218	-6.18	-0.56
Thoc3	chr13	54561240	54561539	-2.65	-0.51
Ctsl	chr13	64470393	64471614	-2.05	-0.13
Zfp738	chr13	67768437	67769544	-3.93	0.97
Zfp738	chr13	67770112	67770442	-1.35	0.70
Zfp738	chr13	67771308	67771907	-2.22	0.95
Jmy	chr13	94204723	94211561	-0.37	-0.02
Jmy	chr13	94242733	94269644	-3.41	-0.41
Bdp1	chr13	100830496	100831456	-1.77	0.15
Bdp1	chr13	100873605	100873965	-2.82	-0.47
Eaf1	chr14	32308264	32308474	-0.40	-0.09
Eaf1	chr14	32317472	32319941	-0.72	-0.10
Ercc6	chr14	33326706	33330503	-0.39	0.06
Ercc6	chr14	33339398	33354435	-0.35	-0.02
Ercc6	chr14	33382990	33388053	-0.37	0.03
Ercc6	chr14	33388945	33390433	-0.30	0.00
Psmc6	chr14	45949498	45949587	-0.33	-0.04
Elf1	chr14	79915491	79936053	-0.36	-0.08
Elf1	chr14	79973144	79980758	-0.60	-0.07

Rarb	chr14	17264515	17264695	-0.34	-0.17
Rarb	chr14	17407418	17451267	-0.26	0.03
Nr1d2	chr14	19039087	19040455	-1.56	0.46
Nr1d2	chr14	19047304	19049180	-1.22	0.20
Nr1d2	chr14	19071469	19071530	-1.34	-1.22
Pip4p1	chr14	51546044	51546434	-0.80	-0.10
Cenpj	chr14	57145717	57148592	-0.47	0.33
Cenpj	chr14	57170817	57171446	-0.66	0.28
Cenpj	chr14	57171803	57172253	-0.54	0.23
Ints6	chr14	63315522	63315763	-4.74	1.04
Polr3d	chr14	70838883	70840064	-0.82	-0.11
Polr3d	chr14	70842873	70843023	-0.77	-0.35
Gtf2f2	chr14	76297012	76306651	-0.80	-0.12
Rictor	chr15	6736486	6737266	-2.00	0.21
Rictor	chr15	6745928	6747284	-0.42	-0.04
Zfp707	chr15	75804010	75804159	-0.55	-0.54
Zfp707	chr15	75804871	75806120	-1.24	-0.34
Tnrc6b	chr15	80709443	80711723	-0.31	0.00
Tnrc6b	chr15	80759610	80760240	-0.53	0.11
L3mbtl2	chr15	81516741	81518048	-2.41	-0.34
L3mbtl2	chr15	81518136	81518556	-4.95	-0.96
Arid2	chr15	96199968	96201115	-1.65	0.17
Arid2	chr15	96201714	96209296	-5.00	0.29
Arid2	chr15	96223066	96232715	-2.26	0.56
Smarcd1	chr15	99532776	99532867	-1.94	-0.95
Nr4a1	chr15	101097365	101101372	-1.77	-0.39
Nr4a1	chr15	101104449	101105046	-1.94	0.57
Zfp740	chr15	102034168	102035505	-4.14	0.49
Zfp740	chr15	102039690	102043820	-0.64	0.08
Sp1	chr15	102238331	102256084	-12.70	0.29
Sp1	chr15	102261230	102261621	-8.92	-0.60
Skp2	chr15	9043359	9043510	-0.96	-0.67
Skp2	chr15	9069207	9070167	-1.36	-0.47
Ywhaz	chr15	36723405	36723964	-0.78	0.46
Taf2	chr15	54847403	54848124	-1.12	-0.24
Taf2	chr15	54903436	54903677	-1.11	-0.38
Atad2	chr15	57928886	57930308	-0.37	0.03
Atad2	chr15	57966457	57966637	-1.21	-0.55
Ago2	chr15	72935799	72936280	-29.70	-0.80
Ago2	chr15	72936518	72937626	-3.54	0.14
Ago2	chr15	73015316	73015377	-0.41	-0.36
Cbx6	chr15	79658159	79664703	-0.48	-0.04
Poldip3	chr15	82958053	82958443	-6.96	-0.37

Ccnt1	chr15	98373641	98374986	-4.33	0.25
Ccnt1	chr15	98398235	98398595	-4.86	1.69
Zfp385a	chr15	103144892	103145221	-0.95	-0.23
Zfp385a	chr15	103150806	103170487	-1.71	-0.45
Zfp263	chr16	3744098	3745116	-0.62	-0.08
Zfp263	chr16	3745414	3745475	-2.29	1.51
Zfp263	chr16	3748158	3750040	-0.55	-0.04
Rrn3	chr16	13813404	13813465	-0.92	-0.42
Ncbp2	chr16	31957210	31957480	-2.37	-0.47
Crebbp	chr16	4084047	4087773	-1.57	-0.10
Crebbp	chr16	4154991	4213404	-1.18	-0.10
Usp7	chr16	8738374	8757028	-4.03	-1.80
Runx1	chr16	92605608	92606179	-9.09	-0.54
Runx1	chr16	92695962	92825842	-45.30	0.96
Arid1b	chr17	4995073	4995224	-0.43	-0.70
Arid1b	chr17	4995283	4995912	-3.11	-1.01
Arid1b	chr17	4996061	5040748	-5.57	-0.80
Arid1b	chr17	5332577	5339323	-1.08	-0.11
Arid1b	chr17	5341398	5343345	-6.15	-0.30
Arid1b	chr17	5344092	5344273	-2.10	0.72
Arid1b	chr17	5344721	5344782	-0.73	2.29
Ppard	chr17	28374025	28423370	-1.05	0.42
Ppard	chr17	28435960	28437007	-2.02	-0.40
Ppard	chr17	28437186	28437276	-1.27	0.60
Brpf3	chr17	28938160	28938221	-0.11	0.00
Brpf3	chr17	28943116	28944314	-2.09	0.44
Brpf3	chr17	28954078	28960704	-1.40	0.32
Brpf3	chr17	28973668	28973878	-0.63	0.44
Cdkn1a	chr17	29228020	29235664	-1.10	-0.06
Rxrb	chr17	34174328	34175135	-5.15	-0.47
Ehmt2	chr17	35040409	35042557	-5.67	-0.61
Ehmt2	chr17	35049712	35050992	-0.56	-0.06
Ddx39b	chr17	35378720	35378917	-8.80	-1.20
Ddx39b	chr17	35386192	35386790	-2.28	-0.20
Ddx39b	chr17	35566041	35566729	-0.59	0.24
Thoc6	chr17	23810618	23810831	-0.65	0.28
Pdpk1	chr17	24215673	24215973	-2.16	0.53
Ube2i	chr17	25398025	25398806	-0.46	0.06
Brd2	chr17	34249262	34249858	-4.89	-0.22
Brd2	chr17	34251401	34252082	-2.62	0.18
Brd2	chr17	34254096	34257280	-0.91	-0.04
Mllt1	chr17	57036825	57045221	-0.54	-0.06
Tgif1	chr17	71193544	71195513	-0.48	-0.02

Tgif1	chr17	71200227	71202842	-5.14	0.40
Srsf7	chr17	80606178	80606645	-2.08	-0.68
Thoc1	chr18	9993386	9993537	-1.86	0.45
Rbbp8	chr18	11880278	11881165	-3.40	0.45
Taf4b	chr18	14965941	14971957	-5.68	1.12
Polr2d	chr18	31955877	31956355	-4.14	0.77
Wdr33	chr18	31963710	31986988	-7.99	-0.83
Wdr33	chr18	32047486	32048906	-2.43	-0.30
Wdr33	chr18	32049234	32067143	-4.68	-0.40
Iws1	chr18	32227387	32242940	-1.73	-0.11
Prelid3a	chr18	67639310	67639430	-0.29	0.14
Rnmt	chr18	68460038	68465602	-2.18	0.33
Smad7	chr18	75527018	75527734	-0.43	0.11
Smad7	chr18	75528210	75530550	-0.94	0.38
Smad7	chr18	75553501	75554009	-1.87	0.69
Smad2	chr18	76462169	76464727	-0.71	-0.14
Epc1	chr18	6436991	6440084	-3.11	0.52
Epc1	chr18	6449003	6450083	-2.15	0.60
Epc1	chr18	6490629	6515906	-1.03	-0.35
Taf7	chr18	37801780	37802286	-6.38	1.76
Taf7	chr18	37802553	37803149	-7.87	1.03
Smad4	chr18	73837391	73837780	-6.22	0.72
Smad4	chr18	73863185	73863395	-4.31	-2.65
Men1	chr19	6335038	6336751	-5.84	-0.70
Men1	chr19	6338903	6340834	-1.37	-0.16
Ints5	chr19	8967596	8971721	-0.99	0.10
Ints5	chr19	8971870	8972380	-0.47	0.08
Cpsf7	chr19	10614192	10620697	-28.30	-0.67
Smarca2	chr19	26705264	26705505	-1.08	-0.39
Cstf2t	chr19	31157390	31159147	-0.74	0.05
Pcgf5	chr19	36484266	36486641	-2.25	0.73
Pcgf5	chr19	36519525	36530396	-0.30	-0.02
Rad9a	chr19	4195437	4195978	-1.67	-0.43
Kat5	chr19	5603043	5603692	-1.27	-0.18
Kat5	chr19	5609351	5609621	-0.44	0.11
Kat5	chr19	5609680	5610034	-0.76	-0.49
Prdx5	chr19	6984074	6984135	-0.92	-0.30
Ldb1	chr19	46107090	46108515	-0.45	-0.03
Ldb1	chr19	46119373	46119552	-0.95	-0.62
Usp9x	chrX	12733152	12734823	-2.16	0.16
Med12	chrX	98488039	98488220	-0.47	0.10
Taf1	chrX	98794675	98794915	-0.51	-0.12
Cstf2	chrX	130609104	130609285	-0.50	0.13

Upf3b	chrX	34632248	34632639	-3.04	-0.66
Upf3b	chrX	34635648	34636954	-0.80	0.26
Chm	chrX	110229662	110229842	-3.50	1.11

RNA polymerase II transcription

gene_name	chr	chromStart	chromEnd	diff.lg.p	diff.log2.fc
Cnot11	chr1	39592697	39593027	-1.55	-0.43
Cnot11	chr1	39603392	39603662	-0.40	-0.08
Cnot9	chr1	74575438	74575798	-0.28	0.00
Psmd1	chr1	87961193	87961284	-0.42	-0.17
Ing5	chr1	95714871	95715801	-0.28	0.00
Ccnt2	chr1	129698792	129700591	-3.01	-0.19
Rbbp5	chr1	134400352	134400982	-0.53	-0.09
Rbbp5	chr1	134401131	134401252	-1.44	-0.61
Kdm5b	chr1	136521430	136521730	-0.84	0.38
Kdm5b	chr1	136527028	136527418	-2.12	-0.56
Kdm5b	chr1	136527777	136528227	-0.93	-0.32
Exo1	chr1	177829740	177831466	-0.36	0.08
Exo1	chr1	177838863	177839044	-0.53	1.66
Exo1	chr1	177839133	177839433	-0.23	0.32
Parp1	chr1	182499105	182503813	-0.82	-0.22
Gls	chr1	52244565	52244686	-2.51	0.56
Gls	chr1	52289446	52290076	-8.87	-0.68
Sumo1	chr1	59696425	59697018	-0.64	0.07
Mdm4	chr1	134888236	134893148	-6.34	0.38
Zc3h11a	chr1	135516447	135517433	-0.36	-0.01
Zc3h11a	chr1	135517641	135518358	-1.03	0.08
Zc3h11a	chr1	135521370	135523781	-8.38	0.35
Rnf2	chr1	153318378	153318528	-1.07	0.48
Smyd2	chr1	191704549	191704728	-0.69	-0.12
Bmi1	chr2	18605667	18606468	-1.54	0.19
Rxra	chr2	27615218	27616386	-3.04	-0.43
Rxra	chr2	27617342	27618510	-1.15	-0.20
Tsc1	chr2	28531258	28532030	-0.42	-0.07
Tsc1	chr2	28541537	28542884	-3.54	-0.53
Abl1	chr2	31544075	31551320	-0.81	-0.11
Abl1	chr2	31653102	31658129	-5.37	-0.32
Abl1	chr2	31659237	31659417	-0.55	0.86
Tnks1bp1	chr2	84891036	84905202	-5.10	-0.39
Psmc3	chr2	90894439	90895182	-2.65	-0.24
Cstf3	chr2	104504891	104505342	-1.40	0.33
Itch	chr2	154959216	154959277	-0.88	-1.17
Itch	chr2	155050162	155050343	-1.21	-0.42
Phf20	chr2	156093050	156093141	-0.58	0.23

Phf20	chr2	156124429	156134013	-1.03	-0.14
Tgif2	chr2	156679159	156679219	-0.37	-2.17
Tgif2	chr2	156679337	156679783	-3.32	-1.45
Src	chr2	157295226	157296441	-0.66	0.35
Srsf6	chr2	162757243	162757817	-16.40	-0.71
Ywhab	chr2	163820932	163837497	-5.11	-0.34
Ywhab	chr2	163842532	163842712	-0.32	-0.01
Trp53rkb	chr2	166620945	166621755	-2.25	-0.66
Cstf1	chr2	172201321	172201638	-1.61	0.32
Cstf1	chr2	172203213	172206586	-4.41	-0.29
Gata3	chr2	9779656	9779806	-0.72	1.20
Gata3	chr2	9796219	9796280	-0.19	-0.10
Pip4k2a	chr2	18765497	18769085	-1.88	0.16
Ehmt1	chr2	24646712	24647822	-1.18	-0.21
Ehmt1	chr2	24733063	24775080	-0.36	-0.02
Cdk9	chr2	32562910	32565101	-2.08	-0.11
Psmb7	chr2	38498946	38499426	-0.68	-0.14
Nr4a2	chr2	56960235	56961013	-0.57	0.24
Nr4a2	chr2	56964339	56964789	-0.86	0.45
Snrpb	chr2	130002885	130005100	-2.11	-0.26
Pcna	chr2	132078675	132078916	-4.27	-0.37
Aurka	chr2	172182198	172182653	-1.29	-0.37
Taf4	chr2	179647358	179656678	-3.26	-0.45
Skil	chr3	30994042	30997442	-7.15	0.42
Skil	chr3	31015942	31017611	-7.07	-0.67
Gatad2b	chr3	90145610	90145850	-0.34	-0.02
Hist2h2be	chr3	96025043	96025490	-1.90	-0.85
Hist2h2be	chr3	96026738	96027185	-0.90	0.58
Notch2	chr3	97817460	97817641	-5.99	-1.08
Notch2	chr3	97938527	97941254	-1.07	-0.09
Notch2	chr3	97950009	97954260	-9.76	-0.14
Ints12	chr3	132771889	132772517	-1.99	0.29
Ube2d3	chr3	135101656	135102228	-4.65	-0.47
Ube2d3	chr3	135128199	135128439	-18.90	0.59
Gtf2b	chr3	142428210	142434431	-1.06	-0.36
Gtf2b	chr3	142446182	142446450	-0.79	-0.15
Elf2	chr3	51059646	51060780	-0.58	0.05
Elf2	chr3	51061575	51064964	-1.46	-0.26
Ints3	chr3	90195399	90196193	-2.42	-0.34
Ints3	chr3	90237228	90237498	-3.56	-0.95
Chtop	chr3	90303594	90304012	-3.30	-0.22
Chtop	chr3	90313215	90313365	-2.06	-0.28
Psmb4	chr3	94690701	94690880	-7.90	-0.58

Rprd2	chr3	95567509	95570056	-0.48	0.03
Rprd2	chr3	95576704	95577124	-1.32	0.38
Hist2h2aa1	chr3	96044042	96044216	-0.53	0.81
Hist2h4	chr3	96067181	96067240	-3.93	-0.68
Hipk1	chr3	103547210	103547959	-0.55	0.04
Hipk1	chr3	103581206	103595159	-4.73	0.13
Prmt6	chr3	110050706	110051487	-2.53	-1.82
Prmt6	chr3	110052356	110053856	-3.47	-0.24
Srsf11	chr3	157674772	157675357	-4.58	-0.26
Trp53inp1	chr4	11083587	11092680	-0.35	-0.03
Trp53inp1	chr4	11095044	11097090	-1.21	0.33
Nbn	chr4	15906509	15911111	-1.35	0.31
Nr4a3	chr4	48064136	48064938	-0.26	0.15
Rraga	chr4	86221606	86222233	-1.51	0.17
Rraga	chr4	86222828	86222948	-5.17	1.30
Tal1	chr4	114740895	114741732	-0.36	-0.04
Med8	chr4	118087440	118087589	-4.47	0.79
Rragc	chr4	123598193	123598284	-0.48	0.12
Rragc	chr4	123612802	123613821	-5.74	0.23
Rragc	chr4	123614119	123614180	-0.69	0.27
Phc2	chr4	128404910	128405061	-1.54	-1.36
Phc2	chr4	128425295	128429198	-5.36	-0.28
Phc2	chr4	128429795	128429916	-4.38	-0.59
Srsf4	chr4	131456030	131457610	-5.46	-0.26
Rpa2	chr4	132333796	132334035	-1.66	-0.63
Nr0b2	chr4	133112127	133112304	-0.20	0.07
Zfp46	chr4	135846596	135848571	-0.77	0.15
Mtor	chr4	147822690	147826440	-1.52	-0.59
Mtor	chr4	147930422	147931764	-0.33	0.02
Mllt3	chr4	87419188	87419819	-0.28	0.00
Mllt3	chr4	87486765	87487246	-0.56	-0.13
Zfp69	chr4	120602861	120603310	-0.27	0.03
Zfp69	chr4	120603519	120603669	-0.44	-0.32
Sesn2	chr4	132048721	132050486	-3.16	-0.43
Sesn2	chr4	132066221	132066371	-4.37	-1.49
Sesn2	chr4	132066234	132066414	-4.63	-1.56
Sfn	chr4	133156530	133157337	-0.32	-0.09
Sfn	chr4	133157425	133158083	-2.14	1.70
Arid1a	chr4	133235970	133241652	-0.91	-0.04
Arid1a	chr4	133276907	133312385	-17.00	-0.64
Srrm1	chr4	134878175	134878295	-1.61	-0.33
Srrm1	chr4	134880837	134881018	-4.45	-0.35
Srrm1	chr4	134902769	134909069	-0.67	0.11

Ski	chr4	154529765	154532213	-0.32	0.00
Ski	chr4	154533975	154596644	-2.32	-0.19
Cdk6	chr5	3519435	3520884	-1.56	-0.25
Kmt2e	chr5	22940246	22940457	-0.51	-0.17
Kmt2e	chr5	22998122	22999368	-4.88	0.59
Kmt2e	chr5	23002969	23005243	-0.85	0.12
Kmt2e	chr5	23007441	23008999	-0.73	0.07
Ywhah	chr5	33361494	33361585	-1.08	0.86
Ywhah	chr5	33369269	33370077	-5.50	0.21
Rbpj	chr5	53981598	53981836	-1.39	-0.35
Rbpj	chr5	54044785	54045056	-0.15	0.35
Rpap2	chr5	108049003	108049513	-2.57	0.65
Triap1	chr5	115791255	115793381	-2.35	-0.35
Kmt5a	chr5	124889998	124895602	-0.24	-0.33
Kmt5a	chr5	124910517	124910788	-4.25	-0.39
Zfp12	chr5	144005811	144007911	-2.24	-0.35
Zfp12	chr5	144007970	144008390	-1.50	-0.69
Zfp12	chr5	144008869	144009619	-2.06	-0.69
Cpsf4	chr5	145939775	145942700	-1.59	0.29
Zkscan5	chr5	145979745	145981214	-1.00	-0.64
Zkscan5	chr5	145982110	145982440	-0.70	0.58
Zfp655	chr5	145992583	145994373	-1.48	-0.63
Zfp655	chr5	146004520	146005030	-4.40	0.44
Zfp655	chr5	146005119	146006108	-11.40	0.68
Zscan25	chr5	146044330	146047272	-1.99	-0.47
Zscan25	chr5	146048248	146052219	-5.54	-0.46
Prkag2	chr5	24369540	24371912	-1.91	-0.73
Prkag2	chr5	24605830	24606370	-0.43	-0.05
Ubc	chr5	125866572	125866810	-2.56	0.16
Ubc	chr5	125867255	125867493	-1.59	-7.24
Ubc	chr5	125867789	125868176	-3.53	0.25
Ubc	chr5	125868235	125870298	-1.07	0.10
Ywhag	chr5	136385024	136385324	-4.97	-0.24
Ywhag	chr5	136386428	136406621	-22.80	-0.25
Ywhag	chr5	136410271	136410481	-5.25	-0.39
Taf6	chr5	138619844	138620172	-2.00	-0.52
Zkscan14	chr5	145956021	145959592	-1.65	-0.73
Zkscan14	chr5	145962189	145962426	-0.66	-0.46
Cul1	chr6	47404345	47445195	-6.27	0.56
Cul1	chr6	47474617	47476108	-1.79	-0.21
Zfp398	chr6	47785899	47790681	-0.75	0.40
Zfp398	chr6	47815976	47817027	-7.69	-1.08
Zfp282	chr6	47827823	47830376	-0.62	-0.17

Zfp282	chr6	47854677	47854738	-0.57	1.09
Zfp282	chr6	47854827	47855936	-4.61	-0.61
Zfp282	chr6	47856235	47856805	-0.70	-0.21
Nr2c2	chr6	92041411	92042461	-6.65	-2.20
Nr2c2	chr6	92114256	92117932	-0.43	0.07
Brpf1	chr6	113257190	113267069	-0.45	-0.02
Brpf1	chr6	113267573	113269263	-6.04	0.44
Brpf1	chr6	113272477	113274549	-3.83	-0.44
Chd4	chr6	125046180	125050907	-20.70	-0.56
Chd4	chr6	125079918	125080429	-2.75	-0.15
Cdkn1b	chr6	134870988	134871169	-0.43	-0.11
Cdkn1b	chr6	134871258	134871349	-0.54	0.22
Cdkn1b	chr6	134872089	134872300	-0.31	0.05
Hipk2	chr6	38647898	38666025	-4.07	-0.19
Hipk2	chr6	38767930	38787047	-1.49	-0.06
Hipk2	chr6	38825859	38826040	-5.88	-0.79
Zfp786	chr6	47769651	47770277	-0.22	0.26
Zfp786	chr6	47770782	47770962	-0.16	-0.17
Zfp786	chr6	47771348	47771617	-0.27	0.46
Zfp746	chr6	48013500	48016503	-1.58	-0.22
Zfp746	chr6	48033553	48036532	-0.36	-0.09
Gadd45a	chr6	66985119	66986711	-1.32	0.30
Gadd45a	chr6	66986829	66987371	-0.61	0.22
Zfp248	chr6	118378735	118380402	-1.67	0.60
Kras	chr6	145168753	145169023	-0.82	0.25
Ints13	chr6	146498363	146498424	-0.40	0.25
U2af2	chr7	5030913	5031003	-2.69	-0.46
Zfp667	chr7	6256343	6256524	-1.23	2.11
Zfp667	chr7	6257719	6257900	-0.55	1.12
Zfp324	chr7	13555702	13556301	-1.93	-1.14
Zfp324	chr7	13556928	13557676	-0.46	-0.29
Ppp1r13l	chr7	19956956	19958711	-6.19	-1.04
Zfp235	chr7	24926820	24927181	-2.93	-1.75
Zfp235	chr7	24927899	24928110	-0.36	0.73
Tgfb1	chr7	26472020	26477400	-2.63	-0.09
Akt2	chr7	28423129	28423428	-0.34	-0.03
Paf1	chr7	29178014	29178133	-0.52	0.20
Paf1	chr7	29181953	29184402	-2.28	-0.16
Zfp30	chr7	30577820	30577971	-0.19	0.01
Zfp30	chr7	30578060	30578271	-0.20	0.17
Zfp30	chr7	30578360	30578780	-0.45	0.65
Zfp420	chr7	30645086	30659464	-0.37	0.20
Zfp420	chr7	30659552	30660089	-1.78	1.00

Zfp420	chr7	30660207	30661190	-0.53	-0.20
Zfp658	chr7	50828018	50828318	-0.28	0.03
Zfp658	chr7	50828587	50830412	-2.41	0.37
Zfp719	chr7	50834985	50846808	-4.97	0.35
Zfp710	chr7	87169699	87227267	-5.56	-0.24
Zfp710	chr7	87231474	87231625	-1.43	-0.54
Zfp710	chr7	87233516	87236145	-0.78	0.08
Setd1a	chr7	134920902	134920963	-0.35	0.61
Setd1a	chr7	134927665	134930073	-0.72	0.05
Setd1a	chr7	134935031	134940855	-4.69	-0.24
Zfp94	chr7	25087200	25088844	-2.03	-0.70
Psmd8	chr7	29965423	29965573	-0.49	-0.05
Zfp940	chr7	30629618	30629679	-0.26	0.84
Zfp940	chr7	30630367	30630458	-0.28	-0.59
Zfp14	chr7	30823226	30823287	-0.16	0.30
Zfp14	chr7	30823733	30823824	-0.28	0.85
Zfp14	chr7	30824091	30824152	-0.53	-3.32
Kmt2b	chr7	31359377	31360985	-9.24	-0.43
Kmt2b	chr7	31368742	31373655	-1.84	-0.11
Med25	chr7	52132343	52133478	-0.35	-0.03
E2f8	chr7	56123117	56123387	-1.32	-0.52
E2f8	chr7	56126321	56127461	-0.65	0.20
Blm	chr7	87647292	87647681	-0.29	-0.01
Blm	chr7	87654407	87658369	-1.75	0.38
Pcf11	chr7	99801691	99806557	-3.02	0.30
Pcf11	chr7	99806795	99808380	-0.45	0.04
Pcf11	chr7	99809716	99810434	-14.40	0.62
Pcf11	chr7	99818062	99818422	-3.92	-0.55
Zfp747	chr7	134516438	134516707	-1.63	0.98
Zfp747	chr7	134516765	134518917	-1.79	0.31
Zfp747	chr7	134518976	134519385	-1.70	0.70
Zfp688	chr7	134562479	134563593	-0.68	-0.19
Zfp688	chr7	134565458	134565548	-0.42	-0.79
Pidd1	chr7	148624413	148625004	-7.46	-1.58
Pidd1	chr7	148628991	148629907	-7.84	-2.18
Snapc2	chr8	4255370	4256190	-1.18	-0.27
Zfp703	chr8	28087837	28090833	-8.85	-0.41
Ppp2cb	chr8	34710092	34710330	-1.15	-1.30
Ell	chr8	73109589	73114440	-0.85	0.23
Ell	chr8	73115469	73115529	-0.40	-0.20
Polr2c	chr8	97387425	97387604	-0.50	0.11
Cbfb	chr8	107694603	107694841	-2.53	-0.45
Banp	chr8	124550490	124550731	-0.55	0.73

Banp	chr8	124551359	124551959	-0.40	0.41
Banp	chr8	124552018	124552768	-0.66	0.57
Zfpm1	chr8	124806220	124856010	-4.41	-0.70
Zfpm1	chr8	124858912	124861147	-5.69	-0.43
Ash2l	chr8	26950507	26958082	-8.59	-0.94
Cnot7	chr8	41592731	41595397	-0.90	-0.17
Gatad2a	chr8	72433213	72435695	-8.00	-0.37
Gatad2a	chr8	72498059	72498150	-1.29	-1.13
Gatad2a	chr8	72519948	72520248	-1.68	-0.77
Csnk2a2	chr8	97972031	97972092	-0.28	-0.02
Csnk2a2	chr8	97976954	97977225	-2.32	-0.50
Psmb10	chr8	108461631	108461997	-1.29	-0.51
Psmd7	chr8	110104338	110108137	-2.12	0.12
Dhx38	chr8	112071923	112073723	-0.34	-0.02
Dhx38	chr8	112080295	112080776	-2.84	-0.58
Dhx38	chr8	112084553	112089501	-4.56	-0.33
Maml2	chr9	13424522	13425152	-3.83	1.67
Maml2	chr9	13510293	13511282	-0.36	-0.08
Pin1	chr9	20456573	20459918	-3.27	-0.67
Smarca4	chr9	21505619	21507475	-7.96	-0.62
Usp2	chr9	43883786	43883906	-0.22	-0.70
Usp2	chr9	43902091	43902272	-0.86	0.94
Ppp2r1b	chr9	50665069	50665957	-0.31	-0.06
Ppp2r1b	chr9	50688020	50688710	-0.69	-0.13
Ppp2r1b	chr9	50701016	50701705	-0.88	-0.20
Ppp2r1b	chr9	50702093	50702154	-0.40	-0.32
Psma4	chr9	54805629	54805837	-1.30	0.21
Sin3a	chr9	56958532	56958892	-4.41	0.69
Sin3a	chr9	56973175	56975606	-1.26	-0.23
Ints14	chr9	64809641	64809791	-1.29	0.57
Ints14	chr9	64833706	64834423	-0.61	-0.09
Leo1	chr9	75289330	75294956	-1.86	-0.19
Topbp1	chr9	103252397	103252458	-0.52	0.32
Ctnnb1	chr9	120842577	120859669	-31.20	-0.94
Ctnnb1	chr9	120867712	120868877	-4.71	-0.16
Zkscan7	chr9	122797666	122797876	-0.37	0.42
Zkscan7	chr9	122803837	122804465	-0.47	0.28
Zkscan7	chr9	122804584	122804943	-0.93	1.07
Zfp426	chr9	20273262	20273323	-0.16	0.32
Zfp426	chr9	20275000	20276171	-0.76	-0.21
Zfp266	chr9	20304209	20306501	-2.59	-0.36
Kmt2a	chr9	44612666	44614167	-0.53	0.05
Kmt2a	chr9	44615605	44617152	-1.08	-0.22

Kmt2a	chr9	44625839	44629215	-4.98	0.22
Kmt2a	chr9	44629664	44630384	-1.76	0.23
Kmt2a	chr9	44655111	44656204	-0.78	0.11
Kmt2a	chr9	44656443	44656954	-0.87	0.18
Kmt2a	chr9	44657463	44658123	-2.39	0.29
Atm	chr9	53247503	53248264	-1.23	-0.25
Pml	chr9	58067472	58068881	-4.04	-0.65
Pml	chr9	58080996	58094982	-6.71	-1.05
Pml	chr9	58097382	58097563	-1.72	-0.95
Rnf111	chr9	70274555	70277390	-2.37	-0.34
Rnf111	chr9	70323620	70350792	-3.72	0.24
Tcf12	chr9	71693887	71694367	-2.35	0.29
Tcf12	chr9	71958591	71959626	-1.46	-0.31
Atrip	chr9	108960445	108961784	-3.87	-0.23
Atrip	chr9	108967670	108968495	-0.81	-0.18
Zfp445	chr9	122761016	122763050	-15.30	0.42
Zfp445	chr9	122770871	122775124	-5.50	0.48
Cited2	chr10	17443033	17444883	-17.10	-0.15
Sgk1	chr10	21718603	21718783	-0.46	0.40
Sesn1	chr10	41530439	41607293	-1.75	-0.49
Sesn1	chr10	41627317	41627765	-0.60	-0.29
Dna2	chr10	62436065	62436156	-0.64	-0.63
Stk11	chr10	79579091	79587616	-0.87	0.11
Stk11	chr10	79592416	79593215	-2.75	0.41
Txnrd1	chr10	82296755	82323149	-1.14	-0.46
Txnrd1	chr10	82358858	82358978	-5.22	-0.68
E2f7	chr10	110216009	110216249	-0.59	0.50
Smarcc2	chr10	127919969	127921148	-2.11	0.34
Smarcc2	chr10	127924513	127924663	-0.79	-0.31
Smarcc2	chr10	127926123	127926303	-3.50	0.69
Ddit4	chr10	59412924	59414518	-5.40	-0.83
Smarcb1	chr10	75359690	75359780	-2.90	-0.84
Polr2e	chr10	79501286	79502404	-4.55	-0.57
Mbd3	chr10	79855285	79856691	-5.75	-0.31
Tcf3	chr10	79872386	79873314	-1.00	0.16
Tcf3	chr10	79876045	79878019	-0.45	-0.12
Cnot2	chr10	115922276	115922606	-0.54	-0.13
Cnot2	chr10	115922814	115923144	-1.17	0.33
Yeats4	chr10	116652435	116652824	-0.52	0.09
Mdm2	chr10	117126230	117126381	-3.39	-0.39
Mdm2	chr10	117126829	117128239	-1.59	-0.06
Dyrk2	chr10	118296404	118305870	-1.58	-0.15
Cdk2	chr10	128135921	128135982	-0.57	0.46

Thoc5	chr11	4795375	4799907	-4.77	-1.07
Med7	chr11	46250478	46253332	-0.93	-0.36
Med7	chr11	46254098	46254907	-11.40	0.92
Ppp2ca	chr11	51912325	51912474	-1.41	-0.58
Ppp2ca	chr11	51935449	51935895	-9.86	0.36
Aff4	chr11	53164268	53186518	-0.95	0.08
Aff4	chr11	53213109	53213978	-1.33	-0.09
Aff4	chr11	53225500	53229192	-0.43	0.04
Cnot8	chr11	57930988	57931228	-0.44	0.14
Zkscan6	chr11	65636382	65642442	-1.52	0.29
Aurkb	chr11	68861788	68863970	-2.20	-0.36
Trp53	chr11	69393860	69394608	-0.85	-0.23
Ywhae	chr11	75546388	75546567	-15.30	-0.67
Suz12	chr11	79843530	79846008	-5.61	0.37
Psmd11	chr11	80285574	80285635	-0.96	-0.36
Cdk5r1	chr11	80290548	80292661	-29.60	2.17
Cdk5r1	chr11	80293284	80294028	-3.53	1.31
Cdk5r1	chr11	80290547	80292674	-31.50	2.18
Cdk5r1	chr11	80293272	80294202	-4.76	1.41
Taf15	chr11	83286609	83286700	-0.89	-0.29
Ppm1d	chr11	85124815	85125293	-5.70	-1.23
Ppm1d	chr11	85153180	85159827	-2.29	-0.40
Srsf1	chr11	87861172	87861352	-2.10	-0.41
Cdk12	chr11	98064618	98065458	-0.67	-0.06
Cdk12	chr11	98071737	98072546	-5.48	0.30
Cdk12	chr11	98104853	98107097	-2.44	0.32
Cdk12	chr11	98111003	98111753	-1.24	-0.17
Psmd3	chr11	98543867	98546829	-9.56	-0.63
Thra	chr11	98603186	98614709	-0.30	0.09
Thra	chr11	98614768	98614918	-0.78	-0.54
Thra	chr11	98625660	98626199	-2.55	-0.66
Casc3	chr11	98683799	98689940	-0.53	0.05
Casc3	chr11	98692913	98694044	-3.83	-0.49
Rara	chr11	98799009	98808908	-0.34	-0.13
Rara	chr11	98808997	98821970	-6.78	-0.99
Rara	chr11	98822029	98822119	-0.30	-0.42
Rara	chr11	98831630	98835508	-11.80	-0.81
Psme3	chr11	101177564	101177745	-2.61	-0.42
Psme3	chr11	101183108	101183258	-2.98	-0.41
Cbx2	chr11	118889233	118890909	-2.18	-0.78
Hus1	chr11	8896711	8897375	-0.49	0.23
Ccng1	chr11	40563841	40563901	-1.93	0.58
Rad50	chr11	53463680	53465739	-0.83	-0.20

Zkscan17	chr11	59299022	59299412	-4.28	0.70
Zkscan17	chr11	59300338	59305712	-3.87	-0.30
Zkscan17	chr11	59319782	59319903	-0.55	-0.48
Top3a	chr11	60553560	60555499	-3.15	-0.90
Top3a	chr11	60555827	60556607	-2.62	-0.75
Ncor1	chr11	62130880	62131481	-2.46	0.27
Ncor1	chr11	62147216	62147307	-0.88	0.25
Ncor1	chr11	62182924	62186997	-1.50	-0.15
Ncor1	chr11	62251972	62270948	-8.07	-1.86
Zfp287	chr11	62527173	62527684	-0.37	-0.15
Zfp287	chr11	62541033	62541184	-0.28	0.59
Zfp287	chr11	62541873	62542444	-2.39	1.37
Zfp287	chr11	62545226	62545317	-0.29	0.62
Chd3	chr11	69157463	69157973	-2.59	-0.35
Chd3	chr11	69175016	69175596	-1.19	-0.32
Chd3	chr11	69177569	69182868	-2.61	-0.40
Supt6	chr11	78020430	78021581	-1.46	-0.13
Supt6	chr11	78047130	78059175	-0.50	0.08
Rffl	chr11	82619321	82631980	-1.42	-0.19
Brip1	chr11	85874744	85875522	-0.94	-0.28
Med13	chr11	86084164	86084555	-6.00	0.92
Med13	chr11	86100091	86100242	-2.66	0.54
Med13	chr11	86104608	86114157	-2.32	0.20
Med13	chr11	86132956	86141386	-0.96	0.10
Pcgf2	chr11	97551210	97551390	-6.81	-1.86
Pip4k2b	chr11	97578204	97583745	-0.53	-0.04
Med1	chr11	98014158	98015024	-0.62	-0.12
Med1	chr11	98015143	98015684	-0.72	0.12
Med1	chr11	98016312	98017303	-0.30	0.00
Med1	chr11	98017662	98017843	-1.54	-0.35
Med1	chr11	98018382	98019702	-0.92	0.08
Med1	chr11	98054214	98054607	-2.46	-0.58
Brca1	chr11	101384784	101385144	-0.45	0.17
Brca1	chr11	101385503	101385983	-2.14	0.68
Brca1	chr11	101386071	101386581	-2.01	-0.62
Brca1	chr11	101386909	101387689	-2.04	0.55
Smarcd2	chr11	106133956	106134286	-1.06	-0.47
Srsf2	chr11	116714108	116714408	-47.20	-0.91
Cbx8	chr11	118899897	118901287	-2.56	-0.91
Cbx4	chr11	118941759	118943921	-4.62	-0.42
Cbx4	chr11	118947103	118947461	-1.29	-1.00
E2f6	chr12	16817770	16817950	-1.20	-0.81
E2f6	chr12	16832231	16832859	-2.21	-0.37

Psma6	chr12	56499811	56499871	-0.49	0.12
Ppm1a	chr12	73862227	73885410	-0.92	-0.08
Snapc1	chr12	75065516	75065726	-2.29	-0.72
Srsf5	chr12	82046490	82046729	-20.10	-0.62
Srsf5	chr12	82050810	82051108	-52.80	-0.87
Cpsf2	chr12	103214183	103220142	-0.41	0.11
Cpsf2	chr12	103240910	103242024	-3.50	0.30
Ccnk	chr12	109418007	109424590	-0.57	0.19
Zfp839	chr12	112088488	112099099	-1.68	-0.40
Zfp839	chr12	112106479	112107344	-1.39	-0.40
Ywhaq	chr12	21422717	21423076	-6.14	-0.76
Max	chr12	78038822	78039688	-0.97	0.07
Max	chr12	78063055	78063205	-1.34	-0.40
Med6	chr12	82674550	82674905	-3.56	0.61
Ppp1r13b	chr12	113066968	113067568	-1.38	0.38
Ppp1r13b	chr12	113070821	113073838	-0.63	0.09
Akt1	chr12	113912217	113912487	-9.20	-1.90
Hist1h3h	chr13	21809496	21809783	-3.88	-0.80
Hist1h3g	chr13	23627286	23627403	-1.47	-0.64
Hist1h3f	chr13	23636303	23636510	-10.20	-0.74
Hist1h2bg	chr13	23663268	23663503	-8.24	-0.53
Hist1h3d	chr13	23666339	23666429	-0.36	-0.31
Hist1h3d	chr13	23667608	23667874	-0.43	-0.08
Hist1h2bc	chr13	23776067	23776242	-3.06	-0.52
Hist1h2bb	chr13	23838602	23838778	-0.32	-0.02
Hist1h2ab	chr13	23842956	23843254	-1.60	-0.36
Rmi1	chr13	58503698	58503988	-0.70	-0.16
Rmi1	chr13	58508424	58509724	-0.62	0.19
Rmi1	chr13	58509903	58511252	-1.10	0.19
Zfp369	chr13	65397670	65398417	-2.03	-0.44
Med10	chr13	69954555	69954853	-3.65	0.44
Ell2	chr13	75844961	75845081	-0.35	-0.23
Ell2	chr13	75901012	75901521	-0.85	0.08
Taf9	chr13	101435888	101436098	-3.19	0.67
Plk2	chr13	111185251	111185491	-0.79	0.33
Plk2	chr13	111190054	111190991	-5.42	0.83
Slc38a9	chr13	113528209	113528600	-0.23	0.05
Zkscan3	chr13	21479290	21480454	-2.86	0.35
Zkscan3	chr13	21488092	21494265	-3.21	-0.74
Zkscan8	chr13	21611911	21613722	-0.86	-0.17
Zkscan8	chr13	21618398	21618788	-1.44	0.47
Hist1h4k	chr13	21842232	21842374	-8.07	-0.57
Hist1h3i	chr13	21875038	21875266	-4.89	-0.55

Hist1h2ae	chr13	23662883	23663060	-3.46	-0.90
Hist1h3c	chr13	23837239	23837390	-0.55	-0.12
Thoc3	chr13	54561240	54561539	-2.65	-0.51
Ctsl	chr13	64470393	64471614	-2.05	-0.13
Zfp738	chr13	67768437	67769544	-3.93	0.97
Zfp738	chr13	67770112	67770442	-1.35	0.70
Zfp738	chr13	67771308	67771907	-2.22	0.95
Jmy	chr13	94204723	94211561	-0.37	-0.02
Jmy	chr13	94242733	94269644	-3.41	-0.41
Eaf1	chr14	32308264	32308474	-0.40	-0.09
Eaf1	chr14	32317472	32319941	-0.72	-0.10
Psmc6	chr14	45949498	45949587	-0.33	-0.04
Elf1	chr14	79915491	79936053	-0.36	-0.08
Elf1	chr14	79973144	79980758	-0.60	-0.07
Rarb	chr14	17264515	17264695	-0.34	-0.17
Rarb	chr14	17407418	17451267	-0.26	0.03
Nr1d2	chr14	19039087	19040455	-1.56	0.46
Nr1d2	chr14	19047304	19049180	-1.22	0.20
Nr1d2	chr14	19071469	19071530	-1.34	-1.22
Pip4p1	chr14	51546044	51546434	-0.80	-0.10
Cenpj	chr14	57145717	57148592	-0.47	0.33
Cenpj	chr14	57170817	57171446	-0.66	0.28
Cenpj	chr14	57171803	57172253	-0.54	0.23
Ints6	chr14	63315522	63315763	-4.74	1.04
Gtf2f2	chr14	76297012	76306651	-0.80	-0.12
Rictor	chr15	6736486	6737266	-2.00	0.21
Rictor	chr15	6745928	6747284	-0.42	-0.04
Zfp707	chr15	75804010	75804159	-0.55	-0.54
Zfp707	chr15	75804871	75806120	-1.24	-0.34
L3mbtl2	chr15	81516741	81518048	-2.41	-0.34
L3mbtl2	chr15	81518136	81518556	-4.95	-0.96
Arid2	chr15	96199968	96201115	-1.65	0.17
Arid2	chr15	96201714	96209296	-5.00	0.29
Arid2	chr15	96223066	96232715	-2.26	0.56
Smarcd1	chr15	99532776	99532867	-1.94	-0.95
Nr4a1	chr15	101097365	101101372	-1.77	-0.39
Nr4a1	chr15	101104449	101105046	-1.94	0.57
Zfp740	chr15	102034168	102035505	-4.14	0.49
Zfp740	chr15	102039690	102043820	-0.64	0.08
Sp1	chr15	102238331	102256084	-12.70	0.29
Sp1	chr15	102261230	102261621	-8.92	-0.60
Skp2	chr15	9043359	9043510	-0.96	-0.67
Skp2	chr15	9069207	9070167	-1.36	-0.47

Ywhaz	chr15	36723405	36723964	-0.78	0.46
Taf2	chr15	54847403	54848124	-1.12	-0.24
Taf2	chr15	54903436	54903677	-1.11	-0.38
Atad2	chr15	57928886	57930308	-0.37	0.03
Atad2	chr15	57966457	57966637	-1.21	-0.55
Cbx6	chr15	79658159	79664703	-0.48	-0.04
Poldip3	chr15	82958053	82958443	-6.96	-0.37
Ccnt1	chr15	98373641	98374986	-4.33	0.25
Ccnt1	chr15	98398235	98398595	-4.86	1.69
Zfp385a	chr15	103144892	103145221	-0.95	-0.23
Zfp385a	chr15	103150806	103170487	-1.71	-0.45
Zfp263	chr16	3744098	3745116	-0.62	-0.08
Zfp263	chr16	3745414	3745475	-2.29	1.51
Zfp263	chr16	3748158	3750040	-0.55	-0.04
Ncbp2	chr16	31957210	31957480	-2.37	-0.47
Crebbp	chr16	4084047	4087773	-1.57	-0.10
Crebbp	chr16	4154991	4213404	-1.18	-0.10
Usp7	chr16	8738374	8757028	-4.03	-1.80
Runx1	chr16	92605608	92606179	-9.09	-0.54
Runx1	chr16	92695962	92825842	-45.30	0.96
Arid1b	chr17	4995073	4995224	-0.43	-0.70
Arid1b	chr17	4995283	4995912	-3.11	-1.01
Arid1b	chr17	4996061	5040748	-5.57	-0.80
Arid1b	chr17	5332577	5339323	-1.08	-0.11
Arid1b	chr17	5341398	5343345	-6.15	-0.30
Arid1b	chr17	5344092	5344273	-2.10	0.72
Arid1b	chr17	5344721	5344782	-0.73	2.29
Ppard	chr17	28374025	28423370	-1.05	0.42
Ppard	chr17	28435960	28437007	-2.02	-0.40
Ppard	chr17	28437186	28437276	-1.27	0.60
Brpf3	chr17	28938160	28938221	-0.11	0.00
Brpf3	chr17	28943116	28944314	-2.09	0.44
Brpf3	chr17	28954078	28960704	-1.40	0.32
Brpf3	chr17	28973668	28973878	-0.63	0.44
Cdkn1a	chr17	29228020	29235664	-1.10	-0.06
Rxrb	chr17	34174328	34175135	-5.15	-0.47
Ehmt2	chr17	35040409	35042557	-5.67	-0.61
Ehmt2	chr17	35049712	35050992	-0.56	-0.06
Ddx39b	chr17	35378720	35378917	-8.80	-1.20
Ddx39b	chr17	35386192	35386790	-2.28	-0.20
Ddx39b	chr17	35566041	35566729	-0.59	0.24
Thoc6	chr17	23810618	23810831	-0.65	0.28
Pdpk1	chr17	24215673	24215973	-2.16	0.53

Ube2i	chr17	25398025	25398806	-0.46	0.06
Brd2	chr17	34249262	34249858	-4.89	-0.22
Brd2	chr17	34251401	34252082	-2.62	0.18
Brd2	chr17	34254096	34257280	-0.91	-0.04
Mllt1	chr17	57036825	57045221	-0.54	-0.06
Tgif1	chr17	71193544	71195513	-0.48	-0.02
Tgif1	chr17	71200227	71202842	-5.14	0.40
Srsf7	chr17	80606178	80606645	-2.08	-0.68
Thoc1	chr18	9993386	9993537	-1.86	0.45
Rbbp8	chr18	11880278	11881165	-3.40	0.45
Taf4b	chr18	14965941	14971957	-5.68	1.12
Polr2d	chr18	31955877	31956355	-4.14	0.77
Wdr33	chr18	31963710	31986988	-7.99	-0.83
Wdr33	chr18	32047486	32048906	-2.43	-0.30
Wdr33	chr18	32049234	32067143	-4.68	-0.40
Iws1	chr18	32227387	32242940	-1.73	-0.11
Prelid3a	chr18	67639310	67639430	-0.29	0.14
Rnmt	chr18	68460038	68465602	-2.18	0.33
Smad7	chr18	75527018	75527734	-0.43	0.11
Smad7	chr18	75528210	75530550	-0.94	0.38
Smad7	chr18	75553501	75554009	-1.87	0.69
Smad2	chr18	76462169	76464727	-0.71	-0.14
Epc1	chr18	6436991	6440084	-3.11	0.52
Epc1	chr18	6449003	6450083	-2.15	0.60
Epc1	chr18	6490629	6515906	-1.03	-0.35
Taf7	chr18	37801780	37802286	-6.38	1.76
Taf7	chr18	37802553	37803149	-7.87	1.03
Smad4	chr18	73837391	73837780	-6.22	0.72
Smad4	chr18	73863185	73863395	-4.31	-2.65
Men1	chr19	6335038	6336751	-5.84	-0.70
Men1	chr19	6338903	6340834	-1.37	-0.16
Ints5	chr19	8967596	8971721	-0.99	0.10
Ints5	chr19	8971870	8972380	-0.47	0.08
Cpsf7	chr19	10614192	10620697	-28.30	-0.67
Smarca2	chr19	26705264	26705505	-1.08	-0.39
Cstf2t	chr19	31157390	31159147	-0.74	0.05
Pcgf5	chr19	36484266	36486641	-2.25	0.73
Pcgf5	chr19	36519525	36530396	-0.30	-0.02
Rad9a	chr19	4195437	4195978	-1.67	-0.43
Kat5	chr19	5603043	5603692	-1.27	-0.18
Kat5	chr19	5609351	5609621	-0.44	0.11
Kat5	chr19	5609680	5610034	-0.76	-0.49
Prdx5	chr19	6984074	6984135	-0.92	-0.30

Ldb1	chr19	46107090	46108515	-0.45	-0.03
Ldb1	chr19	46119373	46119552	-0.95	-0.62
Usp9x	chrX	12733152	12734823	-2.16	0.16
Med12	chrX	98488039	98488220	-0.47	0.10
Taf1	chrX	98794675	98794915	-0.51	-0.12
Cstf2	chrX	130609104	130609285	-0.50	0.13
Upf3b	chrX	34632248	34632639	-3.04	-0.66
Upf3b	chrX	34635648	34636954	-0.80	0.26
Chm	chrX	110229662	110229842	-3.50	1.11

Generic transcription pathway

gene_name	chr	chromStart	chromEnd	diff.lg.p	diff.log2.fc
Cnot11	chr1	39592697	39593027	-1.55	-0.43
Cnot11	chr1	39603392	39603662	-0.40	-0.08
Cnot9	chr1	74575438	74575798	-0.28	0.00
Psmd1	chr1	87961193	87961284	-0.42	-0.17
Ing5	chr1	95714871	95715801	-0.28	0.00
Ccnt2	chr1	129698792	129700591	-3.01	-0.19
Rbbp5	chr1	134400352	134400982	-0.53	-0.09
Rbbp5	chr1	134401131	134401252	-1.44	-0.61
Kdm5b	chr1	136521430	136521730	-0.84	0.38
Kdm5b	chr1	136527028	136527418	-2.12	-0.56
Kdm5b	chr1	136527777	136528227	-0.93	-0.32
Exo1	chr1	177829740	177831466	-0.36	0.08
Exo1	chr1	177838863	177839044	-0.53	1.66
Exo1	chr1	177839133	177839433	-0.23	0.32
Parp1	chr1	182499105	182503813	-0.82	-0.22
Gls	chr1	52244565	52244686	-2.51	0.56
Gls	chr1	52289446	52290076	-8.87	-0.68
Sumo1	chr1	59696425	59697018	-0.64	0.07
Mdm4	chr1	134888236	134893148	-6.34	0.38
Rnf2	chr1	153318378	153318528	-1.07	0.48
Smyd2	chr1	191704549	191704728	-0.69	-0.12
Bmi1	chr2	18605667	18606468	-1.54	0.19
Rxra	chr2	27615218	27616386	-3.04	-0.43
Rxra	chr2	27617342	27618510	-1.15	-0.20
Tsc1	chr2	28531258	28532030	-0.42	-0.07
Tsc1	chr2	28541537	28542884	-3.54	-0.53
Abl1	chr2	31544075	31551320	-0.81	-0.11
Abl1	chr2	31653102	31658129	-5.37	-0.32
Abl1	chr2	31659237	31659417	-0.55	0.86
Tnks1bp1	chr2	84891036	84905202	-5.10	-0.39
Psmc3	chr2	90894439	90895182	-2.65	-0.24
Itch	chr2	154959216	154959277	-0.88	-1.17

Itch	chr2	155050162	155050343	-1.21	-0.42
Phf20	chr2	156093050	156093141	-0.58	0.23
Phf20	chr2	156124429	156134013	-1.03	-0.14
Tgif2	chr2	156679159	156679219	-0.37	-2.17
Tgif2	chr2	156679337	156679783	-3.32	-1.45
Src	chr2	157295226	157296441	-0.66	0.35
Ywhab	chr2	163820932	163837497	-5.11	-0.34
Ywhab	chr2	163842532	163842712	-0.32	-0.01
Trp53rkb	chr2	166620945	166621755	-2.25	-0.66
Gata3	chr2	9779656	9779806	-0.72	1.20
Gata3	chr2	9796219	9796280	-0.19	-0.10
Pip4k2a	chr2	18765497	18769085	-1.88	0.16
Ehmt1	chr2	24646712	24647822	-1.18	-0.21
Ehmt1	chr2	24733063	24775080	-0.36	-0.02
Cdk9	chr2	32562910	32565101	-2.08	-0.11
Psmb7	chr2	38498946	38499426	-0.68	-0.14
Nr4a2	chr2	56960235	56961013	-0.57	0.24
Nr4a2	chr2	56964339	56964789	-0.86	0.45
Pcna	chr2	132078675	132078916	-4.27	-0.37
Aurka	chr2	172182198	172182653	-1.29	-0.37
Taf4	chr2	179647358	179656678	-3.26	-0.45
Skil	chr3	30994042	30997442	-7.15	0.42
Skil	chr3	31015942	31017611	-7.07	-0.67
Gatad2b	chr3	90145610	90145850	-0.34	-0.02
Hist2h2be	chr3	96025043	96025490	-1.90	-0.85
Hist2h2be	chr3	96026738	96027185	-0.90	0.58
Notch2	chr3	97817460	97817641	-5.99	-1.08
Notch2	chr3	97938527	97941254	-1.07	-0.09
Notch2	chr3	97950009	97954260	-9.76	-0.14
Ube2d3	chr3	135101656	135102228	-4.65	-0.47
Ube2d3	chr3	135128199	135128439	-18.90	0.59
Elf2	chr3	51059646	51060780	-0.58	0.05
Elf2	chr3	51061575	51064964	-1.46	-0.26
Psmb4	chr3	94690701	94690880	-7.90	-0.58
Hist2h2aa1	chr3	96044042	96044216	-0.53	0.81
Hist2h4	chr3	96067181	96067240	-3.93	-0.68
Hipk1	chr3	103547210	103547959	-0.55	0.04
Hipk1	chr3	103581206	103595159	-4.73	0.13
Prmt6	chr3	110050706	110051487	-2.53	-1.82
Prmt6	chr3	110052356	110053856	-3.47	-0.24
Trp53inp1	chr4	11083587	11092680	-0.35	-0.03
Trp53inp1	chr4	11095044	11097090	-1.21	0.33
Nbn	chr4	15906509	15911111	-1.35	0.31

Nr4a3	chr4	48064136	48064938	-0.26	0.15
Rraga	chr4	86221606	86222233	-1.51	0.17
Rraga	chr4	86222828	86222948	-5.17	1.30
Tal1	chr4	114740895	114741732	-0.36	-0.04
Med8	chr4	118087440	118087589	-4.47	0.79
Rragc	chr4	123598193	123598284	-0.48	0.12
Rragc	chr4	123612802	123613821	-5.74	0.23
Rragc	chr4	123614119	123614180	-0.69	0.27
Phc2	chr4	128404910	128405061	-1.54	-1.36
Phc2	chr4	128425295	128429198	-5.36	-0.28
Phc2	chr4	128429795	128429916	-4.38	-0.59
Rpa2	chr4	132333796	132334035	-1.66	-0.63
Nr0b2	chr4	133112127	133112304	-0.20	0.07
Zfp46	chr4	135846596	135848571	-0.77	0.15
Mtor	chr4	147822690	147826440	-1.52	-0.59
Mtor	chr4	147930422	147931764	-0.33	0.02
Zfp69	chr4	120602861	120603310	-0.27	0.03
Zfp69	chr4	120603519	120603669	-0.44	-0.32
Sesn2	chr4	132048721	132050486	-3.16	-0.43
Sesn2	chr4	132066221	132066371	-4.37	-1.49
Sesn2	chr4	132066234	132066414	-4.63	-1.56
Sfn	chr4	133156530	133157337	-0.32	-0.09
Sfn	chr4	133157425	133158083	-2.14	1.70
Arid1a	chr4	133235970	133241652	-0.91	-0.04
Arid1a	chr4	133276907	133312385	-17.00	-0.64
Ski	chr4	154529765	154532213	-0.32	0.00
Ski	chr4	154533975	154596644	-2.32	-0.19
Cdk6	chr5	3519435	3520884	-1.56	-0.25
Kmt2e	chr5	22940246	22940457	-0.51	-0.17
Kmt2e	chr5	22998122	22999368	-4.88	0.59
Kmt2e	chr5	23002969	23005243	-0.85	0.12
Kmt2e	chr5	23007441	23008999	-0.73	0.07
Ywhah	chr5	33361494	33361585	-1.08	0.86
Ywhah	chr5	33369269	33370077	-5.50	0.21
Rbpj	chr5	53981598	53981836	-1.39	-0.35
Rbpj	chr5	54044785	54045056	-0.15	0.35
Triap1	chr5	115791255	115793381	-2.35	-0.35
Kmt5a	chr5	124889998	124895602	-0.24	-0.33
Kmt5a	chr5	124910517	124910788	-4.25	-0.39
Zfp12	chr5	144005811	144007911	-2.24	-0.35
Zfp12	chr5	144007970	144008390	-1.50	-0.69
Zfp12	chr5	144008869	144009619	-2.06	-0.69
Zkscan5	chr5	145979745	145981214	-1.00	-0.64

Zkscan5	chr5	145982110	145982440	-0.70	0.58
Zfp655	chr5	145992583	145994373	-1.48	-0.63
Zfp655	chr5	146004520	146005030	-4.40	0.44
Zfp655	chr5	146005119	146006108	-11.40	0.68
Zscan25	chr5	146044330	146047272	-1.99	-0.47
Zscan25	chr5	146048248	146052219	-5.54	-0.46
Prkag2	chr5	24369540	24371912	-1.91	-0.73
Prkag2	chr5	24605830	24606370	-0.43	-0.05
Ubc	chr5	125866572	125866810	-2.56	0.16
Ubc	chr5	125867255	125867493	-1.59	-7.24
Ubc	chr5	125867789	125868176	-3.53	0.25
Ubc	chr5	125868235	125870298	-1.07	0.10
Ywhag	chr5	136385024	136385324	-4.97	-0.24
Ywhag	chr5	136386428	136406621	-22.80	-0.25
Ywhag	chr5	136410271	136410481	-5.25	-0.39
Taf6	chr5	138619844	138620172	-2.00	-0.52
Zkscan14	chr5	145956021	145959592	-1.65	-0.73
Zkscan14	chr5	145962189	145962426	-0.66	-0.46
Cul1	chr6	47404345	47445195	-6.27	0.56
Cul1	chr6	47474617	47476108	-1.79	-0.21
Zfp398	chr6	47785899	47790681	-0.75	0.40
Zfp398	chr6	47815976	47817027	-7.69	-1.08
Zfp282	chr6	47827823	47830376	-0.62	-0.17
Zfp282	chr6	47854677	47854738	-0.57	1.09
Zfp282	chr6	47854827	47855936	-4.61	-0.61
Zfp282	chr6	47856235	47856805	-0.70	-0.21
Nr2c2	chr6	92041411	92042461	-6.65	-2.20
Nr2c2	chr6	92114256	92117932	-0.43	0.07
Brpf1	chr6	113257190	113267069	-0.45	-0.02
Brpf1	chr6	113267573	113269263	-6.04	0.44
Brpf1	chr6	113272477	113274549	-3.83	-0.44
Chd4	chr6	125046180	125050907	-20.70	-0.56
Chd4	chr6	125079918	125080429	-2.75	-0.15
Cdkn1b	chr6	134870988	134871169	-0.43	-0.11
Cdkn1b	chr6	134871258	134871349	-0.54	0.22
Cdkn1b	chr6	134872089	134872300	-0.31	0.05
Hipk2	chr6	38647898	38666025	-4.07	-0.19
Hipk2	chr6	38767930	38787047	-1.49	-0.06
Hipk2	chr6	38825859	38826040	-5.88	-0.79
Zfp786	chr6	47769651	47770277	-0.22	0.26
Zfp786	chr6	47770782	47770962	-0.16	-0.17
Zfp786	chr6	47771348	47771617	-0.27	0.46
Zfp746	chr6	48013500	48016503	-1.58	-0.22

Zfp746	chr6	48033553	48036532	-0.36	-0.09
Gadd45a	chr6	66985119	66986711	-1.32	0.30
Gadd45a	chr6	66986829	66987371	-0.61	0.22
Zfp248	chr6	118378735	118380402	-1.67	0.60
Kras	chr6	145168753	145169023	-0.82	0.25
Zfp667	chr7	6256343	6256524	-1.23	2.11
Zfp667	chr7	6257719	6257900	-0.55	1.12
Zfp324	chr7	13555702	13556301	-1.93	-1.14
Zfp324	chr7	13556928	13557676	-0.46	-0.29
Ppp1r13l	chr7	19956956	19958711	-6.19	-1.04
Zfp235	chr7	24926820	24927181	-2.93	-1.75
Zfp235	chr7	24927899	24928110	-0.36	0.73
Tgfb1	chr7	26472020	26477400	-2.63	-0.09
Akt2	chr7	28423129	28423428	-0.34	-0.03
Zfp30	chr7	30577820	30577971	-0.19	0.01
Zfp30	chr7	30578060	30578271	-0.20	0.17
Zfp30	chr7	30578360	30578780	-0.45	0.65
Zfp420	chr7	30645086	30659464	-0.37	0.20
Zfp420	chr7	30659552	30660089	-1.78	1.00
Zfp420	chr7	30660207	30661190	-0.53	-0.20
Zfp658	chr7	50828018	50828318	-0.28	0.03
Zfp658	chr7	50828587	50830412	-2.41	0.37
Zfp719	chr7	50834985	50846808	-4.97	0.35
Zfp710	chr7	87169699	87227267	-5.56	-0.24
Zfp710	chr7	87231474	87231625	-1.43	-0.54
Zfp710	chr7	87233516	87236145	-0.78	0.08
Setd1a	chr7	134920902	134920963	-0.35	0.61
Setd1a	chr7	134927665	134930073	-0.72	0.05
Setd1a	chr7	134935031	134940855	-4.69	-0.24
Zfp94	chr7	25087200	25088844	-2.03	-0.70
Psmd8	chr7	29965423	29965573	-0.49	-0.05
Zfp940	chr7	30629618	30629679	-0.26	0.84
Zfp940	chr7	30630367	30630458	-0.28	-0.59
Zfp14	chr7	30823226	30823287	-0.16	0.30
Zfp14	chr7	30823733	30823824	-0.28	0.85
Zfp14	chr7	30824091	30824152	-0.53	-3.32
Kmt2b	chr7	31359377	31360985	-9.24	-0.43
Kmt2b	chr7	31368742	31373655	-1.84	-0.11
Med25	chr7	52132343	52133478	-0.35	-0.03
E2f8	chr7	56123117	56123387	-1.32	-0.52
E2f8	chr7	56126321	56127461	-0.65	0.20
Blm	chr7	87647292	87647681	-0.29	-0.01
Blm	chr7	87654407	87658369	-1.75	0.38

Zfp747	chr7	134516438	134516707	-1.63	0.98
Zfp747	chr7	134516765	134518917	-1.79	0.31
Zfp747	chr7	134518976	134519385	-1.70	0.70
Zfp688	chr7	134562479	134563593	-0.68	-0.19
Zfp688	chr7	134565458	134565548	-0.42	-0.79
Pidd1	chr7	148624413	148625004	-7.46	-1.58
Pidd1	chr7	148628991	148629907	-7.84	-2.18
Zfp703	chr8	28087837	28090833	-8.85	-0.41
Ppp2cb	chr8	34710092	34710330	-1.15	-1.30
Ell	chr8	73109589	73114440	-0.85	0.23
Ell	chr8	73115469	73115529	-0.40	-0.20
Polr2c	chr8	97387425	97387604	-0.50	0.11
Cbfb	chr8	107694603	107694841	-2.53	-0.45
Banp	chr8	124550490	124550731	-0.55	0.73
Banp	chr8	124551359	124551959	-0.40	0.41
Banp	chr8	124552018	124552768	-0.66	0.57
Zfpm1	chr8	124806220	124856010	-4.41	-0.70
Zfpm1	chr8	124858912	124861147	-5.69	-0.43
Ash2l	chr8	26950507	26958082	-8.59	-0.94
Cnot7	chr8	41592731	41595397	-0.90	-0.17
Gatad2a	chr8	72433213	72435695	-8.00	-0.37
Gatad2a	chr8	72498059	72498150	-1.29	-1.13
Gatad2a	chr8	72519948	72520248	-1.68	-0.77
Csnk2a2	chr8	97972031	97972092	-0.28	-0.02
Csnk2a2	chr8	97976954	97977225	-2.32	-0.50
Psmb10	chr8	108461631	108461997	-1.29	-0.51
Psmd7	chr8	110104338	110108137	-2.12	0.12
Maml2	chr9	13424522	13425152	-3.83	1.67
Maml2	chr9	13510293	13511282	-0.36	-0.08
Pin1	chr9	20456573	20459918	-3.27	-0.67
Smarca4	chr9	21505619	21507475	-7.96	-0.62
Usp2	chr9	43883786	43883906	-0.22	-0.70
Usp2	chr9	43902091	43902272	-0.86	0.94
Ppp2r1b	chr9	50665069	50665957	-0.31	-0.06
Ppp2r1b	chr9	50688020	50688710	-0.69	-0.13
Ppp2r1b	chr9	50701016	50701705	-0.88	-0.20
Ppp2r1b	chr9	50702093	50702154	-0.40	-0.32
Psma4	chr9	54805629	54805837	-1.30	0.21
Sin3a	chr9	56958532	56958892	-4.41	0.69
Sin3a	chr9	56973175	56975606	-1.26	-0.23
Topbp1	chr9	103252397	103252458	-0.52	0.32
Ctnnb1	chr9	120842577	120859669	-31.20	-0.94
Ctnnb1	chr9	120867712	120868877	-4.71	-0.16

Zkscan7	chr9	122797666	122797876	-0.37	0.42
Zkscan7	chr9	122803837	122804465	-0.47	0.28
Zkscan7	chr9	122804584	122804943	-0.93	1.07
Zfp426	chr9	20273262	20273323	-0.16	0.32
Zfp426	chr9	20275000	20276171	-0.76	-0.21
Zfp266	chr9	20304209	20306501	-2.59	-0.36
Kmt2a	chr9	44612666	44614167	-0.53	0.05
Kmt2a	chr9	44615605	44617152	-1.08	-0.22
Kmt2a	chr9	44625839	44629215	-4.98	0.22
Kmt2a	chr9	44629664	44630384	-1.76	0.23
Kmt2a	chr9	44655111	44656204	-0.78	0.11
Kmt2a	chr9	44656443	44656954	-0.87	0.18
Kmt2a	chr9	44657463	44658123	-2.39	0.29
Atm	chr9	53247503	53248264	-1.23	-0.25
Pml	chr9	58067472	58068881	-4.04	-0.65
Pml	chr9	58080996	58094982	-6.71	-1.05
Pml	chr9	58097382	58097563	-1.72	-0.95
Rnf111	chr9	70274555	70277390	-2.37	-0.34
Rnf111	chr9	70323620	70350792	-3.72	0.24
Tcf12	chr9	71693887	71694367	-2.35	0.29
Tcf12	chr9	71958591	71959626	-1.46	-0.31
Atrip	chr9	108960445	108961784	-3.87	-0.23
Atrip	chr9	108967670	108968495	-0.81	-0.18
Zfp445	chr9	122761016	122763050	-15.30	0.42
Zfp445	chr9	122770871	122775124	-5.50	0.48
Cited2	chr10	17443033	17444883	-17.10	-0.15
Sgk1	chr10	21718603	21718783	-0.46	0.40
Sesn1	chr10	41530439	41607293	-1.75	-0.49
Sesn1	chr10	41627317	41627765	-0.60	-0.29
Dna2	chr10	62436065	62436156	-0.64	-0.63
Stk11	chr10	79579091	79587616	-0.87	0.11
Stk11	chr10	79592416	79593215	-2.75	0.41
Txnrd1	chr10	82296755	82323149	-1.14	-0.46
Txnrd1	chr10	82358858	82358978	-5.22	-0.68
E2f7	chr10	110216009	110216249	-0.59	0.50
Smarcc2	chr10	127919969	127921148	-2.11	0.34
Smarcc2	chr10	127924513	127924663	-0.79	-0.31
Smarcc2	chr10	127926123	127926303	-3.50	0.69
Ddit4	chr10	59412924	59414518	-5.40	-0.83
Smarcb1	chr10	75359690	75359780	-2.90	-0.84
Polr2e	chr10	79501286	79502404	-4.55	-0.57
Mbd3	chr10	79855285	79856691	-5.75	-0.31
Tcf3	chr10	79872386	79873314	-1.00	0.16

Tcf3	chr10	79876045	79878019	-0.45	-0.12
Cnot2	chr10	115922276	115922606	-0.54	-0.13
Cnot2	chr10	115922814	115923144	-1.17	0.33
Yeats4	chr10	116652435	116652824	-0.52	0.09
Mdm2	chr10	117126230	117126381	-3.39	-0.39
Mdm2	chr10	117126829	117128239	-1.59	-0.06
Dyrk2	chr10	118296404	118305870	-1.58	-0.15
Cdk2	chr10	128135921	128135982	-0.57	0.46
Med7	chr11	46250478	46253332	-0.93	-0.36
Med7	chr11	46254098	46254907	-11.40	0.92
Ppp2ca	chr11	51912325	51912474	-1.41	-0.58
Ppp2ca	chr11	51935449	51935895	-9.86	0.36
Cnot8	chr11	57930988	57931228	-0.44	0.14
Zkscan6	chr11	65636382	65642442	-1.52	0.29
Aurkb	chr11	68861788	68863970	-2.20	-0.36
Trp53	chr11	69393860	69394608	-0.85	-0.23
Ywhae	chr11	75546388	75546567	-15.30	-0.67
Suz12	chr11	79843530	79846008	-5.61	0.37
Psmd11	chr11	80285574	80285635	-0.96	-0.36
Cdk5r1	chr11	80290548	80292661	-29.60	2.17
Cdk5r1	chr11	80293284	80294028	-3.53	1.31
Cdk5r1	chr11	80290547	80292674	-31.50	2.18
Cdk5r1	chr11	80293272	80294202	-4.76	1.41
Taf15	chr11	83286609	83286700	-0.89	-0.29
Ppm1d	chr11	85124815	85125293	-5.70	-1.23
Ppm1d	chr11	85153180	85159827	-2.29	-0.40
Cdk12	chr11	98064618	98065458	-0.67	-0.06
Cdk12	chr11	98071737	98072546	-5.48	0.30
Cdk12	chr11	98104853	98107097	-2.44	0.32
Cdk12	chr11	98111003	98111753	-1.24	-0.17
Psmd3	chr11	98543867	98546829	-9.56	-0.63
Thra	chr11	98603186	98614709	-0.30	0.09
Thra	chr11	98614768	98614918	-0.78	-0.54
Thra	chr11	98625660	98626199	-2.55	-0.66
Rara	chr11	98799009	98808908	-0.34	-0.13
Rara	chr11	98808997	98821970	-6.78	-0.99
Rara	chr11	98822029	98822119	-0.30	-0.42
Rara	chr11	98831630	98835508	-11.80	-0.81
Psme3	chr11	101177564	101177745	-2.61	-0.42
Psme3	chr11	101183108	101183258	-2.98	-0.41
Cbx2	chr11	118889233	118890909	-2.18	-0.78
Hus1	chr11	8896711	8897375	-0.49	0.23
Ccng1	chr11	40563841	40563901	-1.93	0.58

Rad50	chr11	53463680	53465739	-0.83	-0.20
Zkscan17	chr11	59299022	59299412	-4.28	0.70
Zkscan17	chr11	59300338	59305712	-3.87	-0.30
Zkscan17	chr11	59319782	59319903	-0.55	-0.48
Top3a	chr11	60553560	60555499	-3.15	-0.90
Top3a	chr11	60555827	60556607	-2.62	-0.75
Ncor1	chr11	62130880	62131481	-2.46	0.27
Ncor1	chr11	62147216	62147307	-0.88	0.25
Ncor1	chr11	62182924	62186997	-1.50	-0.15
Ncor1	chr11	62251972	62270948	-8.07	-1.86
Zfp287	chr11	62527173	62527684	-0.37	-0.15
Zfp287	chr11	62541033	62541184	-0.28	0.59
Zfp287	chr11	62541873	62542444	-2.39	1.37
Zfp287	chr11	62545226	62545317	-0.29	0.62
Chd3	chr11	69157463	69157973	-2.59	-0.35
Chd3	chr11	69175016	69175596	-1.19	-0.32
Chd3	chr11	69177569	69182868	-2.61	-0.40
Rffl	chr11	82619321	82631980	-1.42	-0.19
Brip1	chr11	85874744	85875522	-0.94	-0.28
Med13	chr11	86084164	86084555	-6.00	0.92
Med13	chr11	86100091	86100242	-2.66	0.54
Med13	chr11	86104608	86114157	-2.32	0.20
Med13	chr11	86132956	86141386	-0.96	0.10
Pcgf2	chr11	97551210	97551390	-6.81	-1.86
Pip4k2b	chr11	97578204	97583745	-0.53	-0.04
Med1	chr11	98014158	98015024	-0.62	-0.12
Med1	chr11	98015143	98015684	-0.72	0.12
Med1	chr11	98016312	98017303	-0.30	0.00
Med1	chr11	98017662	98017843	-1.54	-0.35
Med1	chr11	98018382	98019702	-0.92	0.08
Med1	chr11	98054214	98054607	-2.46	-0.58
Brca1	chr11	101384784	101385144	-0.45	0.17
Brca1	chr11	101385503	101385983	-2.14	0.68
Brca1	chr11	101386071	101386581	-2.01	-0.62
Brca1	chr11	101386909	101387689	-2.04	0.55
Smarcd2	chr11	106133956	106134286	-1.06	-0.47
Cbx8	chr11	118899897	118901287	-2.56	-0.91
Cbx4	chr11	118941759	118943921	-4.62	-0.42
Cbx4	chr11	118947103	118947461	-1.29	-1.00
E2f6	chr12	16817770	16817950	-1.20	-0.81
E2f6	chr12	16832231	16832859	-2.21	-0.37
Psma6	chr12	56499811	56499871	-0.49	0.12
Ppm1a	chr12	73862227	73885410	-0.92	-0.08

Ccnk	chr12	109418007	109424590	-0.57	0.19
Zfp839	chr12	112088488	112099099	-1.68	-0.40
Zfp839	chr12	112106479	112107344	-1.39	-0.40
Ywhaq	chr12	21422717	21423076	-6.14	-0.76
Max	chr12	78038822	78039688	-0.97	0.07
Max	chr12	78063055	78063205	-1.34	-0.40
Med6	chr12	82674550	82674905	-3.56	0.61
Ppp1r13b	chr12	113066968	113067568	-1.38	0.38
Ppp1r13b	chr12	113070821	113073838	-0.63	0.09
Akt1	chr12	113912217	113912487	-9.20	-1.90
Hist1h3h	chr13	21809496	21809783	-3.88	-0.80
Hist1h3g	chr13	23627286	23627403	-1.47	-0.64
Hist1h3f	chr13	23636303	23636510	-10.20	-0.74
Hist1h2bg	chr13	23663268	23663503	-8.24	-0.53
Hist1h3d	chr13	23666339	23666429	-0.36	-0.31
Hist1h3d	chr13	23667608	23667874	-0.43	-0.08
Hist1h2bc	chr13	23776067	23776242	-3.06	-0.52
Hist1h2bb	chr13	23838602	23838778	-0.32	-0.02
Hist1h2ab	chr13	23842956	23843254	-1.60	-0.36
Rmi1	chr13	58503698	58503988	-0.70	-0.16
Rmi1	chr13	58508424	58509724	-0.62	0.19
Rmi1	chr13	58509903	58511252	-1.10	0.19
Zfp369	chr13	65397670	65398417	-2.03	-0.44
Med10	chr13	69954555	69954853	-3.65	0.44
Taf9	chr13	101435888	101436098	-3.19	0.67
Plk2	chr13	111185251	111185491	-0.79	0.33
Plk2	chr13	111190054	111190991	-5.42	0.83
Slc38a9	chr13	113528209	113528600	-0.23	0.05
Zkscan3	chr13	21479290	21480454	-2.86	0.35
Zkscan3	chr13	21488092	21494265	-3.21	-0.74
Zkscan8	chr13	21611911	21613722	-0.86	-0.17
Zkscan8	chr13	21618398	21618788	-1.44	0.47
Hist1h4k	chr13	21842232	21842374	-8.07	-0.57
Hist1h3i	chr13	21875038	21875266	-4.89	-0.55
Hist1h2ae	chr13	23662883	23663060	-3.46	-0.90
Hist1h3c	chr13	23837239	23837390	-0.55	-0.12
Ctsl	chr13	64470393	64471614	-2.05	-0.13
Zfp738	chr13	67768437	67769544	-3.93	0.97
Zfp738	chr13	67770112	67770442	-1.35	0.70
Zfp738	chr13	67771308	67771907	-2.22	0.95
Jmy	chr13	94204723	94211561	-0.37	-0.02
Jmy	chr13	94242733	94269644	-3.41	-0.41
Psmc6	chr14	45949498	45949587	-0.33	-0.04

Elf1	chr14	79915491	79936053	-0.36	-0.08
Elf1	chr14	79973144	79980758	-0.60	-0.07
Rarb	chr14	17264515	17264695	-0.34	-0.17
Rarb	chr14	17407418	17451267	-0.26	0.03
Nr1d2	chr14	19039087	19040455	-1.56	0.46
Nr1d2	chr14	19047304	19049180	-1.22	0.20
Nr1d2	chr14	19071469	19071530	-1.34	-1.22
Pip4p1	chr14	51546044	51546434	-0.80	-0.10
Cenpj	chr14	57145717	57148592	-0.47	0.33
Cenpj	chr14	57170817	57171446	-0.66	0.28
Cenpj	chr14	57171803	57172253	-0.54	0.23
Gtf2f2	chr14	76297012	76306651	-0.80	-0.12
Rictor	chr15	6736486	6737266	-2.00	0.21
Rictor	chr15	6745928	6747284	-0.42	-0.04
Zfp707	chr15	75804010	75804159	-0.55	-0.54
Zfp707	chr15	75804871	75806120	-1.24	-0.34
L3mbtl2	chr15	81516741	81518048	-2.41	-0.34
L3mbtl2	chr15	81518136	81518556	-4.95	-0.96
Arid2	chr15	96199968	96201115	-1.65	0.17
Arid2	chr15	96201714	96209296	-5.00	0.29
Arid2	chr15	96223066	96232715	-2.26	0.56
Smarcd1	chr15	99532776	99532867	-1.94	-0.95
Nr4a1	chr15	101097365	101101372	-1.77	-0.39
Nr4a1	chr15	101104449	101105046	-1.94	0.57
Zfp740	chr15	102034168	102035505	-4.14	0.49
Zfp740	chr15	102039690	102043820	-0.64	0.08
Sp1	chr15	102238331	102256084	-12.70	0.29
Sp1	chr15	102261230	102261621	-8.92	-0.60
Skp2	chr15	9043359	9043510	-0.96	-0.67
Skp2	chr15	9069207	9070167	-1.36	-0.47
Ywhaz	chr15	36723405	36723964	-0.78	0.46
Taf2	chr15	54847403	54848124	-1.12	-0.24
Taf2	chr15	54903436	54903677	-1.11	-0.38
Atad2	chr15	57928886	57930308	-0.37	0.03
Atad2	chr15	57966457	57966637	-1.21	-0.55
Cbx6	chr15	79658159	79664703	-0.48	-0.04
Ccnt1	chr15	98373641	98374986	-4.33	0.25
Ccnt1	chr15	98398235	98398595	-4.86	1.69
Zfp385a	chr15	103144892	103145221	-0.95	-0.23
Zfp385a	chr15	103150806	103170487	-1.71	-0.45
Zfp263	chr16	3744098	3745116	-0.62	-0.08
Zfp263	chr16	3745414	3745475	-2.29	1.51
Zfp263	chr16	3748158	3750040	-0.55	-0.04

Crebbp	chr16	4084047	4087773	-1.57	-0.10
Crebbp	chr16	4154991	4213404	-1.18	-0.10
Usp7	chr16	8738374	8757028	-4.03	-1.80
Runx1	chr16	92605608	92606179	-9.09	-0.54
Runx1	chr16	92695962	92825842	-45.30	0.96
Arid1b	chr17	4995073	4995224	-0.43	-0.70
Arid1b	chr17	4995283	4995912	-3.11	-1.01
Arid1b	chr17	4996061	5040748	-5.57	-0.80
Arid1b	chr17	5332577	5339323	-1.08	-0.11
Arid1b	chr17	5341398	5343345	-6.15	-0.30
Arid1b	chr17	5344092	5344273	-2.10	0.72
Arid1b	chr17	5344721	5344782	-0.73	2.29
Ppard	chr17	28374025	28423370	-1.05	0.42
Ppard	chr17	28435960	28437007	-2.02	-0.40
Ppard	chr17	28437186	28437276	-1.27	0.60
Brpf3	chr17	28938160	28938221	-0.11	0.00
Brpf3	chr17	28943116	28944314	-2.09	0.44
Brpf3	chr17	28954078	28960704	-1.40	0.32
Brpf3	chr17	28973668	28973878	-0.63	0.44
Cdkn1a	chr17	29228020	29235664	-1.10	-0.06
Rxrb	chr17	34174328	34175135	-5.15	-0.47
Ehmt2	chr17	35040409	35042557	-5.67	-0.61
Ehmt2	chr17	35049712	35050992	-0.56	-0.06
Pdpk1	chr17	24215673	24215973	-2.16	0.53
Ube2i	chr17	25398025	25398806	-0.46	0.06
Brd2	chr17	34249262	34249858	-4.89	-0.22
Brd2	chr17	34251401	34252082	-2.62	0.18
Brd2	chr17	34254096	34257280	-0.91	-0.04
Tgif1	chr17	71193544	71195513	-0.48	-0.02
Tgif1	chr17	71200227	71202842	-5.14	0.40
Rbbp8	chr18	11880278	11881165	-3.40	0.45
Taf4b	chr18	14965941	14971957	-5.68	1.12
Polr2d	chr18	31955877	31956355	-4.14	0.77
Prelid3a	chr18	67639310	67639430	-0.29	0.14
Smad7	chr18	75527018	75527734	-0.43	0.11
Smad7	chr18	75528210	75530550	-0.94	0.38
Smad7	chr18	75553501	75554009	-1.87	0.69
Smad2	chr18	76462169	76464727	-0.71	-0.14
Epc1	chr18	6436991	6440084	-3.11	0.52
Epc1	chr18	6449003	6450083	-2.15	0.60
Epc1	chr18	6490629	6515906	-1.03	-0.35
Taf7	chr18	37801780	37802286	-6.38	1.76
Taf7	chr18	37802553	37803149	-7.87	1.03

Smad4	chr18	73837391	73837780	-6.22	0.72
Smad4	chr18	73863185	73863395	-4.31	-2.65
Men1	chr19	6335038	6336751	-5.84	-0.70
Men1	chr19	6338903	6340834	-1.37	-0.16
Smarca2	chr19	26705264	26705505	-1.08	-0.39
Pcgf5	chr19	36484266	36486641	-2.25	0.73
Pcgf5	chr19	36519525	36530396	-0.30	-0.02
Rad9a	chr19	4195437	4195978	-1.67	-0.43
Kat5	chr19	5603043	5603692	-1.27	-0.18
Kat5	chr19	5609351	5609621	-0.44	0.11
Kat5	chr19	5609680	5610034	-0.76	-0.49
Prdx5	chr19	6984074	6984135	-0.92	-0.30
Ldb1	chr19	46107090	46108515	-0.45	-0.03
Ldb1	chr19	46119373	46119552	-0.95	-0.62
Usp9x	chrX	12733152	12734823	-2.16	0.16
Med12	chrX	98488039	98488220	-0.47	0.10
Taf1	chrX	98794675	98794915	-0.51	-0.12
Chm	chrX	110229662	110229842	-3.50	1.11

Transcriptional regulation by TP53

gene_name	chr	chromStart	chromEnd	diff.lg.p	diff.log2.fc
Cnot11	chr1	39592697	39593027	-1.55	-0.43
Cnot11	chr1	39603392	39603662	-0.40	-0.08
Cnot9	chr1	74575438	74575798	-0.28	0.00
Ing5	chr1	95714871	95715801	-0.28	0.00
Ccnt2	chr1	129698792	129700591	-3.01	-0.19
Exo1	chr1	177829740	177831466	-0.36	0.08
Exo1	chr1	177838863	177839044	-0.53	1.66
Exo1	chr1	177839133	177839433	-0.23	0.32
Gls	chr1	52244565	52244686	-2.51	0.56
Gls	chr1	52289446	52290076	-8.87	-0.68
Mdm4	chr1	134888236	134893148	-6.34	0.38
Smyd2	chr1	191704549	191704728	-0.69	-0.12
Tsc1	chr2	28531258	28532030	-0.42	-0.07
Tsc1	chr2	28541537	28542884	-3.54	-0.53
Tnks1bp1	chr2	84891036	84905202	-5.10	-0.39
Phf20	chr2	156093050	156093141	-0.58	0.23
Phf20	chr2	156124429	156134013	-1.03	-0.14
Ywhab	chr2	163820932	163837497	-5.11	-0.34
Ywhab	chr2	163842532	163842712	-0.32	-0.01
Trp53rkb	chr2	166620945	166621755	-2.25	-0.66
Pip4k2a	chr2	18765497	18769085	-1.88	0.16
Ehmt1	chr2	24646712	24647822	-1.18	-0.21
Ehmt1	chr2	24733063	24775080	-0.36	-0.02

Cdk9	chr2	32562910	32565101	-2.08	-0.11
Pcna	chr2	132078675	132078916	-4.27	-0.37
Aurka	chr2	172182198	172182653	-1.29	-0.37
Taf4	chr2	179647358	179656678	-3.26	-0.45
Gatad2b	chr3	90145610	90145850	-0.34	-0.02
Hipk1	chr3	103547210	103547959	-0.55	0.04
Hipk1	chr3	103581206	103595159	-4.73	0.13
Trp53inp1	chr4	11083587	11092680	-0.35	-0.03
Trp53inp1	chr4	11095044	11097090	-1.21	0.33
Nbn	chr4	15906509	15911111	-1.35	0.31
Rraga	chr4	86221606	86222233	-1.51	0.17
Rraga	chr4	86222828	86222948	-5.17	1.30
Rragc	chr4	123598193	123598284	-0.48	0.12
Rragc	chr4	123612802	123613821	-5.74	0.23
Rragc	chr4	123614119	123614180	-0.69	0.27
Rpa2	chr4	132333796	132334035	-1.66	-0.63
Mtor	chr4	147822690	147826440	-1.52	-0.59
Mtor	chr4	147930422	147931764	-0.33	0.02
Sesn2	chr4	132048721	132050486	-3.16	-0.43
Sesn2	chr4	132066221	132066371	-4.37	-1.49
Sesn2	chr4	132066234	132066414	-4.63	-1.56
Sfn	chr4	133156530	133157337	-0.32	-0.09
Sfn	chr4	133157425	133158083	-2.14	1.70
Ywhah	chr5	33361494	33361585	-1.08	0.86
Ywhah	chr5	33369269	33370077	-5.50	0.21
Triap1	chr5	115791255	115793381	-2.35	-0.35
Kmt5a	chr5	124889998	124895602	-0.24	-0.33
Kmt5a	chr5	124910517	124910788	-4.25	-0.39
Prkag2	chr5	24369540	24371912	-1.91	-0.73
Prkag2	chr5	24605830	24606370	-0.43	-0.05
Ubc	chr5	125866572	125866810	-2.56	0.16
Ubc	chr5	125867255	125867493	-1.59	-7.24
Ubc	chr5	125867789	125868176	-3.53	0.25
Ubc	chr5	125868235	125870298	-1.07	0.10
Ywhag	chr5	136385024	136385324	-4.97	-0.24
Ywhag	chr5	136386428	136406621	-22.80	-0.25
Ywhag	chr5	136410271	136410481	-5.25	-0.39
Taf6	chr5	138619844	138620172	-2.00	-0.52
Brpf1	chr6	113257190	113267069	-0.45	-0.02
Brpf1	chr6	113267573	113269263	-6.04	0.44
Brpf1	chr6	113272477	113274549	-3.83	-0.44
Chd4	chr6	125046180	125050907	-20.70	-0.56
Chd4	chr6	125079918	125080429	-2.75	-0.15

Cdkn1b	chr6	134870988	134871169	-0.43	-0.11
Cdkn1b	chr6	134871258	134871349	-0.54	0.22
Cdkn1b	chr6	134872089	134872300	-0.31	0.05
Hipk2	chr6	38647898	38666025	-4.07	-0.19
Hipk2	chr6	38767930	38787047	-1.49	-0.06
Hipk2	chr6	38825859	38826040	-5.88	-0.79
Gadd45a	chr6	66985119	66986711	-1.32	0.30
Gadd45a	chr6	66986829	66987371	-0.61	0.22
Ppp1r13l	chr7	19956956	19958711	-6.19	-1.04
Akt2	chr7	28423129	28423428	-0.34	-0.03
Zfp420	chr7	30645086	30659464	-0.37	0.20
Zfp420	chr7	30659552	30660089	-1.78	1.00
Zfp420	chr7	30660207	30661190	-0.53	-0.20
E2f8	chr7	56123117	56123387	-1.32	-0.52
E2f8	chr7	56126321	56127461	-0.65	0.20
Blm	chr7	87647292	87647681	-0.29	-0.01
Blm	chr7	87654407	87658369	-1.75	0.38
Pidd1	chr7	148624413	148625004	-7.46	-1.58
Pidd1	chr7	148628991	148629907	-7.84	-2.18
Ppp2cb	chr8	34710092	34710330	-1.15	-1.30
Ell	chr8	73109589	73114440	-0.85	0.23
Ell	chr8	73115469	73115529	-0.40	-0.20
Polr2c	chr8	97387425	97387604	-0.50	0.11
Banp	chr8	124550490	124550731	-0.55	0.73
Banp	chr8	124551359	124551959	-0.40	0.41
Banp	chr8	124552018	124552768	-0.66	0.57
Cnot7	chr8	41592731	41595397	-0.90	-0.17
Gatad2a	chr8	72433213	72435695	-8.00	-0.37
Gatad2a	chr8	72498059	72498150	-1.29	-1.13
Gatad2a	chr8	72519948	72520248	-1.68	-0.77
Csnk2a2	chr8	97972031	97972092	-0.28	-0.02
Csnk2a2	chr8	97976954	97977225	-2.32	-0.50
Pin1	chr9	20456573	20459918	-3.27	-0.67
Usp2	chr9	43883786	43883906	-0.22	-0.70
Usp2	chr9	43902091	43902272	-0.86	0.94
Ppp2r1b	chr9	50665069	50665957	-0.31	-0.06
Ppp2r1b	chr9	50688020	50688710	-0.69	-0.13
Ppp2r1b	chr9	50701016	50701705	-0.88	-0.20
Ppp2r1b	chr9	50702093	50702154	-0.40	-0.32
Topbp1	chr9	103252397	103252458	-0.52	0.32
Atm	chr9	53247503	53248264	-1.23	-0.25
Pml	chr9	58067472	58068881	-4.04	-0.65
Pml	chr9	58080996	58094982	-6.71	-1.05

Pml	chr9	58097382	58097563	-1.72	-0.95
Atrip	chr9	108960445	108961784	-3.87	-0.23
Atrip	chr9	108967670	108968495	-0.81	-0.18
Sgk1	chr10	21718603	21718783	-0.46	0.40
Sesn1	chr10	41530439	41607293	-1.75	-0.49
Sesn1	chr10	41627317	41627765	-0.60	-0.29
Dna2	chr10	62436065	62436156	-0.64	-0.63
Stk11	chr10	79579091	79587616	-0.87	0.11
Stk11	chr10	79592416	79593215	-2.75	0.41
Txnrd1	chr10	82296755	82323149	-1.14	-0.46
Txnrd1	chr10	82358858	82358978	-5.22	-0.68
E2f7	chr10	110216009	110216249	-0.59	0.50
Ddit4	chr10	59412924	59414518	-5.40	-0.83
Polr2e	chr10	79501286	79502404	-4.55	-0.57
Mbd3	chr10	79855285	79856691	-5.75	-0.31
Cnot2	chr10	115922276	115922606	-0.54	-0.13
Cnot2	chr10	115922814	115923144	-1.17	0.33
Mdm2	chr10	117126230	117126381	-3.39	-0.39
Mdm2	chr10	117126829	117128239	-1.59	-0.06
Dyrk2	chr10	118296404	118305870	-1.58	-0.15
Cdk2	chr10	128135921	128135982	-0.57	0.46
Ppp2ca	chr11	51912325	51912474	-1.41	-0.58
Ppp2ca	chr11	51935449	51935895	-9.86	0.36
Cnot8	chr11	57930988	57931228	-0.44	0.14
Aurkb	chr11	68861788	68863970	-2.20	-0.36
Trp53	chr11	69393860	69394608	-0.85	-0.23
Ywhae	chr11	75546388	75546567	-15.30	-0.67
Cdk5r1	chr11	80290548	80292661	-29.60	2.17
Cdk5r1	chr11	80293284	80294028	-3.53	1.31
Cdk5r1	chr11	80290547	80292674	-31.50	2.18
Cdk5r1	chr11	80293272	80294202	-4.76	1.41
Taf15	chr11	83286609	83286700	-0.89	-0.29
Cdk12	chr11	98064618	98065458	-0.67	-0.06
Cdk12	chr11	98071737	98072546	-5.48	0.30
Cdk12	chr11	98104853	98107097	-2.44	0.32
Cdk12	chr11	98111003	98111753	-1.24	-0.17
Hus1	chr11	8896711	8897375	-0.49	0.23
Ccng1	chr11	40563841	40563901	-1.93	0.58
Rad50	chr11	53463680	53465739	-0.83	-0.20
Top3a	chr11	60553560	60555499	-3.15	-0.90
Top3a	chr11	60555827	60556607	-2.62	-0.75
Chd3	chr11	69157463	69157973	-2.59	-0.35
Chd3	chr11	69175016	69175596	-1.19	-0.32

Chd3	chr11	69177569	69182868	-2.61	-0.40
Rffl	chr11	82619321	82631980	-1.42	-0.19
Brip1	chr11	85874744	85875522	-0.94	-0.28
Pip4k2b	chr11	97578204	97583745	-0.53	-0.04
Brca1	chr11	101384784	101385144	-0.45	0.17
Brca1	chr11	101385503	101385983	-2.14	0.68
Brca1	chr11	101386071	101386581	-2.01	-0.62
Brca1	chr11	101386909	101387689	-2.04	0.55
Ccnk	chr12	109418007	109424590	-0.57	0.19
Ywhaq	chr12	21422717	21423076	-6.14	-0.76
Ppp1r13b	chr12	113066968	113067568	-1.38	0.38
Ppp1r13b	chr12	113070821	113073838	-0.63	0.09
Akt1	chr12	113912217	113912487	-9.20	-1.90
Rmi1	chr13	58503698	58503988	-0.70	-0.16
Rmi1	chr13	58508424	58509724	-0.62	0.19
Rmi1	chr13	58509903	58511252	-1.10	0.19
Taf9	chr13	101435888	101436098	-3.19	0.67
Plk2	chr13	111185251	111185491	-0.79	0.33
Plk2	chr13	111190054	111190991	-5.42	0.83
Slc38a9	chr13	113528209	113528600	-0.23	0.05
Jmy	chr13	94204723	94211561	-0.37	-0.02
Jmy	chr13	94242733	94269644	-3.41	-0.41
Pip4p1	chr14	51546044	51546434	-0.80	-0.10
Cenpj	chr14	57145717	57148592	-0.47	0.33
Cenpj	chr14	57170817	57171446	-0.66	0.28
Cenpj	chr14	57171803	57172253	-0.54	0.23
Gtf2f2	chr14	76297012	76306651	-0.80	-0.12
Rictor	chr15	6736486	6737266	-2.00	0.21
Rictor	chr15	6745928	6747284	-0.42	-0.04
Ywhaz	chr15	36723405	36723964	-0.78	0.46
Taf2	chr15	54847403	54848124	-1.12	-0.24
Taf2	chr15	54903436	54903677	-1.11	-0.38
Ccnt1	chr15	98373641	98374986	-4.33	0.25
Ccnt1	chr15	98398235	98398595	-4.86	1.69
Zfp385a	chr15	103144892	103145221	-0.95	-0.23
Zfp385a	chr15	103150806	103170487	-1.71	-0.45
Usp7	chr16	8738374	8757028	-4.03	-1.80
Brpf3	chr17	28938160	28938221	-0.11	0.00
Brpf3	chr17	28943116	28944314	-2.09	0.44
Brpf3	chr17	28954078	28960704	-1.40	0.32
Brpf3	chr17	28973668	28973878	-0.63	0.44
Cdkn1a	chr17	29228020	29235664	-1.10	-0.06
Ehmt2	chr17	35040409	35042557	-5.67	-0.61

Ehmt2	chr17	35049712	35050992	-0.56	-0.06
Pdpk1	chr17	24215673	24215973	-2.16	0.53
Rbbp8	chr18	11880278	11881165	-3.40	0.45
Taf4b	chr18	14965941	14971957	-5.68	1.12
Polr2d	chr18	31955877	31956355	-4.14	0.77
Prelid3a	chr18	67639310	67639430	-0.29	0.14
Taf7	chr18	37801780	37802286	-6.38	1.76
Taf7	chr18	37802553	37803149	-7.87	1.03
Rad9a	chr19	4195437	4195978	-1.67	-0.43
Kat5	chr19	5603043	5603692	-1.27	-0.18
Kat5	chr19	5609351	5609621	-0.44	0.11
Kat5	chr19	5609680	5610034	-0.76	-0.49
Prdx5	chr19	6984074	6984135	-0.92	-0.30
Taf1	chrX	98794675	98794915	-0.51	-0.12
Chm	chrX	110229662	110229842	-3.50	1.11

MyD88-independent TLR4 cascade

gene_name	chr	chromStart	chromEnd	diff.lg.p	diff.log2.fc
Creb1	chr1	64622786	64644320	-4.45	0.46
Ikbke	chr1	133151178	133153534	-0.59	-0.07
Ikbke	chr1	133173083	133173518	-0.44	-0.13
Tank	chr2	61416642	61431225	-0.69	0.29
Tank	chr2	61482412	61484290	-2.15	0.57
Tank	chr2	61484409	61488467	-9.81	0.48
Tank	chr2	61489840	61491718	-2.86	0.49
Traf6	chr2	101531587	101537899	-1.26	-0.14
Ube2d3	chr3	135101656	135102228	-4.65	-0.47
Ube2d3	chr3	135128199	135128439	-18.90	0.59
Nfkb1	chr3	135248187	135252299	-1.43	-0.19
Nfkb1	chr3	135354240	135354361	-0.22	-0.02
Map3k7	chr4	32051081	32051232	-2.12	-1.15
Map3k7	chr4	32104109	32107297	-9.56	0.62
Map3k7	chr4	32110051	32110231	-0.40	-0.13
Tlr4	chr4	66494965	66503025	-13.60	0.46
Ripk2	chr4	16050729	16051591	-1.84	-0.38
Ripk2	chr4	16090555	16090645	-1.23	-0.78
Jun	chr4	94716292	94718913	-0.65	-0.03
Rps6ka1	chr4	133403324	133404010	-2.82	-0.30
Ubc	chr5	125866572	125866810	-2.56	0.16
Ubc	chr5	125867255	125867493	-1.59	-7.24
Ubc	chr5	125867789	125868176	-3.53	0.25
Ubc	chr5	125868235	125870298	-1.07	0.10
Cul1	chr6	47404345	47445195	-6.27	0.56
Cul1	chr6	47474617	47476108	-1.79	-0.21

Irak2	chr6	113588490	113588551	-0.76	-1.06
Irak2	chr6	113641013	113644480	-4.46	-0.37
Nod1	chr6	54893436	54893676	-0.15	0.06
Nod1	chr6	54894600	54894899	-0.32	0.98
Irf3	chr7	52253029	52253209	-0.62	-0.25
Irf3	chr7	52256238	52257801	-2.00	-0.33
Nfkbib	chr7	29543299	29543360	-0.35	1.74
Nfkbib	chr7	29551273	29551543	-0.53	-0.09
Fadd	chr7	151765303	151765394	-0.27	-0.26
Fadd	chr7	151765572	151768192	-0.83	-0.16
Map2k7	chr8	4245576	4246251	-1.17	-0.17
Map2k7	chr8	4246579	4246700	-0.65	0.26
Ppp2cb	chr8	34710092	34710330	-1.15	-1.30
Dusp4	chr8	35870663	35882259	-9.10	-0.24
Ikbkb	chr8	23770516	23770997	-2.23	-0.36
Ppp2r1b	chr9	50665069	50665957	-0.31	-0.06
Ppp2r1b	chr9	50688020	50688710	-0.69	-0.13
Ppp2r1b	chr9	50701016	50701705	-0.88	-0.20
Ppp2r1b	chr9	50702093	50702154	-0.40	-0.32
Dusp7	chr9	106270962	106271201	-3.22	-2.66
Dusp7	chr9	106271467	106276865	-1.01	-0.11
Birc2	chr9	7818614	7818943	-0.66	0.25
Birc2	chr9	7833580	7834717	-5.61	0.55
Birc2	chr9	7834955	7835255	-0.35	0.08
Birc3	chr9	7848909	7850958	-0.70	0.12
Birc3	chr9	7860311	7872991	-1.36	0.15
Myd88	chr9	119245819	119245939	-1.44	-0.36
Myd88	chr9	119248919	119249158	-1.59	-0.46
Dusp6	chr10	98725864	98729379	-7.24	0.27
Tab2	chr10	7625983	7626433	-2.18	0.30
Tab2	chr10	7626671	7629889	-2.99	0.23
Tab2	chr10	7631233	7644544	-8.90	0.25
Fbxw11	chr11	32644536	32645016	-0.47	-0.05
Ppp2ca	chr11	51912325	51912474	-1.41	-0.58
Ppp2ca	chr11	51935449	51935895	-9.86	0.36
Map2k3	chr11	60765498	60766275	-0.72	0.06
Map2k4	chr11	65503265	65506940	-1.24	-0.16
Dusp3	chr11	101834543	101835230	-4.42	-0.25
Dusp3	chr11	101835527	101835855	-0.35	0.01
Dusp3	chr11	101835944	101836213	-1.98	-0.25
Traf3	chr12	112499766	112500664	-1.20	-0.20
Nfkbia	chr12	56593425	56593604	-7.73	-0.63
Rps6ka5	chr12	101789211	101791305	-3.01	-0.71

Tab1	chr15	79988217	79992132	-8.64	-0.65
Atf1	chr15	100084915	100090754	-0.82	-0.12
App	chr16	85025175	85030561	-15.10	-0.48
App	chr16	85120907	85173892	-5.29	-0.55
Rps6ka2	chr17	7374463	7374584	-0.23	0.21
Rps6ka2	chr17	7506974	7507424	-0.38	-0.27
Ppp2r5d	chr17	46820177	46820772	-0.45	-0.06
Ppp2r5d	chr17	46837059	46841951	-2.20	-0.75
Ticam1	chr17	56408884	56416190	-0.73	-0.05
Ube2d2a	chr18	35931242	35931392	-0.83	-0.33
Ube2d2a	chr18	35965240	35965570	-4.99	0.88
Map3k8	chr18	4348959	4352951	-0.29	-0.01
Cd14	chr18	36884720	36886308	-26.10	0.15
Ticam2	chr18	46718812	46719023	-0.65	0.53
Ticam2	chr18	46719501	46720669	-0.88	-0.20
Rela	chr19	5637549	5637639	-0.85	-0.86
Rela	chr19	5646998	5647773	-4.34	-0.29
Nfkb2	chr19	46379316	46380320	-0.99	0.32
Chuk	chr19	44181817	44181937	-0.72	-0.36
Ikbkg	chrX	71675045	71678178	-2.09	-1.00
Tab3	chrX	82859447	82860165	-0.50	-0.10
Tab3	chrX	82860462	82860613	-3.04	1.35
Irak1	chrX	71260386	71261911	-3.38	-0.36

TRIF (TICAM1)-mediated TLR4 signaling

gene_name	chr	chromStart	chromEnd	diff.lg.p	diff.log2.fc
Creb1	chr1	64622786	64644320	-4.45	0.46
Ikbke	chr1	133151178	133153534	-0.59	-0.07
Ikbke	chr1	133173083	133173518	-0.44	-0.13
Tank	chr2	61416642	61431225	-0.69	0.29
Tank	chr2	61482412	61484290	-2.15	0.57
Tank	chr2	61484409	61488467	-9.81	0.48
Tank	chr2	61489840	61491718	-2.86	0.49
Traf6	chr2	101531587	101537899	-1.26	-0.14
Ube2d3	chr3	135101656	135102228	-4.65	-0.47
Ube2d3	chr3	135128199	135128439	-18.90	0.59
Nfkb1	chr3	135248187	135252299	-1.43	-0.19
Nfkb1	chr3	135354240	135354361	-0.22	-0.02
Map3k7	chr4	32051081	32051232	-2.12	-1.15
Map3k7	chr4	32104109	32107297	-9.56	0.62
Map3k7	chr4	32110051	32110231	-0.40	-0.13
Tlr4	chr4	66494965	66503025	-13.60	0.46
Ripk2	chr4	16050729	16051591	-1.84	-0.38
Ripk2	chr4	16090555	16090645	-1.23	-0.78

Jun	chr4	94716292	94718913	-0.65	-0.03
Rps6ka1	chr4	133403324	133404010	-2.82	-0.30
Ubc	chr5	125866572	125866810	-2.56	0.16
Ubc	chr5	125867255	125867493	-1.59	-7.24
Ubc	chr5	125867789	125868176	-3.53	0.25
Ubc	chr5	125868235	125870298	-1.07	0.10
Cul1	chr6	47404345	47445195	-6.27	0.56
Cul1	chr6	47474617	47476108	-1.79	-0.21
Irak2	chr6	113588490	113588551	-0.76	-1.06
Irak2	chr6	113641013	113644480	-4.46	-0.37
Nod1	chr6	54893436	54893676	-0.15	0.06
Nod1	chr6	54894600	54894899	-0.32	0.98
Irf3	chr7	52253029	52253209	-0.62	-0.25
Irf3	chr7	52256238	52257801	-2.00	-0.33
Nfkbib	chr7	29543299	29543360	-0.35	1.74
Nfkbib	chr7	29551273	29551543	-0.53	-0.09
Fadd	chr7	151765303	151765394	-0.27	-0.26
Fadd	chr7	151765572	151768192	-0.83	-0.16
Map2k7	chr8	4245576	4246251	-1.17	-0.17
Map2k7	chr8	4246579	4246700	-0.65	0.26
Ppp2cb	chr8	34710092	34710330	-1.15	-1.30
Dusp4	chr8	35870663	35882259	-9.10	-0.24
Ikbkb	chr8	23770516	23770997	-2.23	-0.36
Ppp2r1b	chr9	50665069	50665957	-0.31	-0.06
Ppp2r1b	chr9	50688020	50688710	-0.69	-0.13
Ppp2r1b	chr9	50701016	50701705	-0.88	-0.20
Ppp2r1b	chr9	50702093	50702154	-0.40	-0.32
Dusp7	chr9	106270962	106271201	-3.22	-2.66
Dusp7	chr9	106271467	106276865	-1.01	-0.11
Birc2	chr9	7818614	7818943	-0.66	0.25
Birc2	chr9	7833580	7834717	-5.61	0.55
Birc2	chr9	7834955	7835255	-0.35	0.08
Birc3	chr9	7848909	7850958	-0.70	0.12
Birc3	chr9	7860311	7872991	-1.36	0.15
Myd88	chr9	119245819	119245939	-1.44	-0.36
Myd88	chr9	119248919	119249158	-1.59	-0.46
Dusp6	chr10	98725864	98729379	-7.24	0.27
Tab2	chr10	7625983	7626433	-2.18	0.30
Tab2	chr10	7626671	7629889	-2.99	0.23
Tab2	chr10	7631233	7644544	-8.90	0.25
Fbxw11	chr11	32644536	32645016	-0.47	-0.05
Ppp2ca	chr11	51912325	51912474	-1.41	-0.58
Ppp2ca	chr11	51935449	51935895	-9.86	0.36

Map2k3	chr11	60765498	60766275	-0.72	0.06
Map2k4	chr11	65503265	65506940	-1.24	-0.16
Dusp3	chr11	101834543	101835230	-4.42	-0.25
Dusp3	chr11	101835527	101835855	-0.35	0.01
Dusp3	chr11	101835944	101836213	-1.98	-0.25
Traf3	chr12	112499766	112500664	-1.20	-0.20
Nfkbia	chr12	56593425	56593604	-7.73	-0.63
Rps6ka5	chr12	101789211	101791305	-3.01	-0.71
Tab1	chr15	79988217	79992132	-8.64	-0.65
Atf1	chr15	100084915	100090754	-0.82	-0.12
App	chr16	85025175	85030561	-15.10	-0.48
App	chr16	85120907	85173892	-5.29	-0.55
Rps6ka2	chr17	7374463	7374584	-0.23	0.21
Rps6ka2	chr17	7506974	7507424	-0.38	-0.27
Ppp2r5d	chr17	46820177	46820772	-0.45	-0.06
Ppp2r5d	chr17	46837059	46841951	-2.20	-0.75
Ticam1	chr17	56408884	56416190	-0.73	-0.05
Ube2d2a	chr18	35931242	35931392	-0.83	-0.33
Ube2d2a	chr18	35965240	35965570	-4.99	0.88
Map3k8	chr18	4348959	4352951	-0.29	-0.01
Cd14	chr18	36884720	36886308	-26.10	0.15
Ticam2	chr18	46718812	46719023	-0.65	0.53
Ticam2	chr18	46719501	46720669	-0.88	-0.20
Rela	chr19	5637549	5637639	-0.85	-0.86
Rela	chr19	5646998	5647773	-4.34	-0.29
Nfkb2	chr19	46379316	46380320	-0.99	0.32
Chuk	chr19	44181817	44181937	-0.72	-0.36
Ikbkg	chrX	71675045	71678178	-2.09	-1.00
Tab3	chrX	82859447	82860165	-0.50	-0.10
Tab3	chrX	82860462	82860613	-3.04	1.35
Irak1	chrX	71260386	71261911	-3.38	-0.36

Signaling by Rho GTPase

gene_name	chr	chromStart	chromEnd	diff.lg.p	diff.log2.fc
Arhgef4	chr1	34868611	34868971	-0.64	-0.38
Sgo2a	chr1	58072495	58072676	-1.00	0.72
Sgo2a	chr1	58073063	58073573	-5.77	1.44
Sgo2a	chr1	58073662	58073723	-0.44	1.14
Sgo2a	chr1	58073991	58074800	-3.42	1.02
Abi2	chr1	60527626	60534214	-1.29	0.27
Arpc5	chr1	154613701	154613968	-6.51	-0.36
Arhgap30	chr1	173319090	173319450	-0.38	0.04
Arhgap30	chr1	173335168	173339113	-1.11	-0.06
Nsl1	chr1	192906279	192906490	-0.24	0.03

Ncoa2	chr1	13157086	13167163	-2.70	-0.26
Actr3	chr1	127331982	127332265	-2.00	-0.36
Ahctf1	chr1	181677367	181685668	-1.17	-0.08
Ahctf1	chr1	181692900	181693051	-0.83	-0.26
Cenpf	chr1	191466321	191466561	-2.25	-0.96
Cenpf	chr1	191472789	191474534	-4.18	-1.21
Cenpf	chr1	191476338	191477059	-6.52	1.04
Cenpf	chr1	191477148	191482268	-0.66	0.07
Cenpf	chr1	191483016	191483856	-1.50	0.35
Ppp2r5a	chr1	193176577	193176817	-0.62	0.09
Ppp2r5a	chr1	193196363	193220920	-7.48	-0.55
Abl1	chr2	31544075	31551320	-0.81	-0.11
Abl1	chr2	31653102	31658129	-5.37	-0.32
Abl1	chr2	31659237	31659417	-0.55	0.86
Nup160	chr2	90575704	90575765	-0.94	0.49
Kif18a	chr2	109173461	109173732	-1.67	0.79
Mapre1	chr2	153592829	153593040	-0.76	0.14
Src	chr2	157295226	157296441	-0.66	0.35
Ywhab	chr2	163820932	163837497	-5.11	-0.34
Ywhab	chr2	163842532	163842712	-0.32	-0.01
Abi1	chr2	22895609	22895730	-1.32	-0.41
Tubb4b	chr2	25077914	25079487	-0.90	0.06
Tubb4b	chr2	25080103	25080192	-0.50	-0.30
Wipf1	chr2	73273114	73275208	-1.10	-0.21
Wipf1	chr2	73275357	73278120	-3.12	0.32
Wipf1	chr2	73282616	73291856	-1.06	0.35
Depdc7	chr2	104570464	104570643	-0.43	0.36
Prex1	chr2	166392533	166394859	-0.45	-0.04
Arhgef11	chr3	87536714	87538447	-1.55	-0.25
Arhgef11	chr3	87541566	87541835	-1.65	-0.39
Arhgef2	chr3	88425072	88425193	-2.95	-0.66
Arhgef2	chr3	88448133	88451105	-18.80	-0.50
Hist2h2be	chr3	96025043	96025490	-1.90	-0.85
Hist2h2be	chr3	96026738	96027185	-0.90	0.58
Rhoc	chr3	104591951	104594845	-3.84	-0.85
Cenpe	chr3	134902767	134903916	-1.03	-0.49
Cenpe	chr3	134904065	134905504	-1.08	-0.40
Cenpe	chr3	134910323	134910534	-0.64	-0.28
Cenpe	chr3	134936264	134936504	-1.10	0.77
Hist2h2aa1	chr3	96044042	96044216	-0.53	0.81
Hist2h4	chr3	96067181	96067240	-3.93	-0.68
Pkn2	chr3	142456046	142457385	-3.76	0.39
Pkn2	chr3	142516503	142544699	-4.98	0.80

Plekhg5	chr4	151487918	151488669	-0.95	0.38
Plekhg5	chr4	151489058	151489269	-0.46	-0.34
Kif2c	chr4	116832628	116832838	-0.28	0.06
Sfn	chr4	133156530	133157337	-0.32	-0.09
Sfn	chr4	133157425	133158083	-2.14	1.70
Arhgef10l	chr4	140070399	140072722	-3.50	-0.29
Ywhah	chr5	33361494	33361585	-1.08	0.86
Ywhah	chr5	33369269	33370077	-5.50	0.21
Cit	chr5	116375627	116376509	-0.23	0.01
Arpc1b	chr5	145875124	145883438	-23.40	-0.69
Cenpc1	chr5	86464551	86466811	-0.46	0.05
Cenpc1	chr5	86494488	86494548	-0.32	0.13
Rhof	chr5	123553648	123553918	-1.94	1.94
Rhof	chr5	123567408	123567916	-1.14	0.52
Rhof	chr5	123568094	123582429	-0.92	-0.08
Clip1	chr5	124029078	124029498	-0.51	0.06
Clip1	chr5	124062170	124067553	-0.41	-0.05
Clip1	chr5	124077308	124081141	-0.52	-0.04
Clip1	chr5	124103521	124106024	-3.11	0.25
Ywhag	chr5	136385024	136385324	-4.97	-0.24
Ywhag	chr5	136386428	136406621	-22.80	-0.25
Ywhag	chr5	136410271	136410481	-5.25	-0.39
Mad1l1	chr5	140791001	140797506	-0.48	-0.22
Rac1	chr5	144288681	144288861	-5.59	-0.89
Rtkn	chr6	83087964	83088145	-0.64	1.73
Rtkn	chr6	83102212	83102363	-0.42	0.84
Arhgap25	chr6	87409378	87413310	-2.55	-0.36
Arhgap25	chr6	87482930	87483110	-0.46	0.41
Srgap3	chr6	112668833	112669582	-2.31	-0.67
Srgap3	chr6	112671886	112672875	-0.53	-0.14
Srgap3	chr6	112672994	112673115	-0.34	-0.19
Srgap3	chr6	112896481	112896751	-0.48	0.15
Srgap3	chr6	112897020	112897260	-0.45	-0.18
Cyfip1	chr7	63097490	63127258	-0.29	0.00
Akap13	chr7	82753380	82754790	-12.10	0.47
Akap13	chr7	82754969	82756772	-2.34	0.17
Akap13	chr7	82892540	82895986	-2.97	-0.18
Arap1	chr7	108496612	108526720	-13.10	-0.50
Arap1	chr7	108559930	108560380	-7.90	-0.46
Plk1	chr7	129276554	129303004	-0.59	-0.17
Plk1	chr7	129313000	129313091	-0.76	-0.56
Plekhg2	chr7	29144712	29146146	-0.62	-0.22
Plekhg2	chr7	29146960	29147647	-0.57	0.27

Plekhg2	chr7	29156384	29156696	-0.19	1.00
Plekhg2	chr7	29156784	29157502	-1.08	-0.60
Arhgap33	chr7	31309015	31309465	-1.32	-0.97
Iqgap1	chr7	87857908	87858329	-4.34	-0.18
Iqgap1	chr7	87858448	87858659	-3.33	0.18
Iqgap1	chr7	87944725	87948217	-15.10	-0.50
Nup98	chr7	109268003	109269144	-0.31	0.00
Nup98	chr7	109283070	109283401	-1.16	-0.19
Rhog	chr7	109387636	109398632	-1.04	0.04
Arhgap17	chr7	130422811	130430171	-0.46	0.04
Arhgap17	chr7	130470849	130513369	-2.86	-0.95
Arhgef18	chr8	3452049	3453370	-1.45	-0.65
Arhgef18	chr8	3454894	3455284	-1.19	-0.62
Mcf2l	chr8	12963213	12984716	-1.76	-0.62
Mcf2l	chr8	13019010	13019610	-1.31	-0.41
Arhgef10	chr8	14997663	15000127	-7.73	-1.00
Ppp2cb	chr8	34710092	34710330	-1.15	-1.30
Myo9b	chr8	73796642	73839032	-0.36	-0.01
Myo9b	chr8	73871704	73876088	-3.07	-0.14
Myo9b	chr8	73880532	73884611	-0.65	-0.03
Pik3r2	chr8	73292079	73292558	-0.69	-0.13
Pik3r2	chr8	73296811	73300551	-1.84	-0.26
Arhgap10	chr8	79774383	79774890	-1.23	-0.30
Pkn1	chr8	86193660	86194199	-4.44	-0.40
Pkn1	chr8	86216676	86217939	-6.80	-0.83
Pkn1	chr8	86222898	86223078	-1.14	-0.97
Dync1li2	chr8	106944120	106944421	-2.85	-0.50
Zw10	chr9	48886338	48886728	-1.51	0.32
Ppp2r1b	chr9	50665069	50665957	-0.31	-0.06
Ppp2r1b	chr9	50688020	50688710	-0.69	-0.13
Ppp2r1b	chr9	50701016	50701705	-0.88	-0.20
Ppp2r1b	chr9	50702093	50702154	-0.40	-0.32
Myo9a	chr9	59599100	59628166	-0.62	0.11
Myo9a	chr9	59717536	59718478	-0.51	-0.15
Myo9a	chr9	59719195	59722455	-0.42	0.06
Myo9a	chr9	59771534	59773141	-1.66	-0.39
Ctnnb1	chr9	120842577	120859669	-31.20	-0.94
Ctnnb1	chr9	120867712	120868877	-4.71	-0.16
Arhgef12	chr9	42780101	42782733	-0.32	-0.02
Arhgef12	chr9	42850752	42913801	-0.32	-0.02
Zwilch	chr9	63985369	63985819	-0.67	0.25
Ranbp2	chr10	57939345	57939706	-5.21	0.45
Ranbp2	chr10	57939885	57940516	-34.30	1.07

Ranbp2	chr10	57940724	57941055	-4.44	0.51
Ranbp2	chr10	57941564	57941925	-3.73	0.28
Ranbp2	chr10	57942194	57945844	-38.80	0.62
Ranbp2	chr10	57955388	57956812	-8.12	0.45
Zwint	chr10	72117642	72118991	-1.35	-0.32
Zwint	chr10	72131564	72131923	-1.77	0.41
Arhgap45	chr10	79479416	79480842	-10.70	-0.44
Arhgap45	chr10	79485025	79488189	-9.77	-0.49
Arhgap45	chr10	79493496	79494216	-6.50	-0.29
Ppp1r12a	chr10	107706405	107714631	-0.52	-0.05
Xpo1	chr11	23156040	23156131	-0.32	0.11
Ppp2ca	chr11	51912325	51912474	-1.41	-0.58
Ppp2ca	chr11	51935449	51935895	-9.86	0.36
Aurkb	chr11	68861788	68863970	-2.20	-0.36
Dvl2	chr11	69825412	69825473	-0.29	0.56
Ywhae	chr11	75546388	75546567	-15.30	-0.67
Ska2	chr11	86935514	86935724	-1.12	-0.75
Wipf2	chr11	98725121	98752028	-0.42	-0.11
Wipf2	chr11	98752900	98757438	-0.32	0.01
Wipf2	chr11	98760051	98761553	-4.50	-0.56
Fmnl1	chr11	103032451	103032631	-1.31	-0.19
Gna13	chr11	109241765	109257871	-0.40	0.02
Baiap2	chr11	119862794	119863004	-1.98	1.02
Baiap2	chr11	119863569	119863660	-0.69	0.89
Baiap2	chr11	119867827	119868096	-0.74	0.20
Spdl1	chr11	34622746	34623344	-0.29	-0.01
Spdl1	chr11	34639040	34647113	-1.00	-0.36
Cyfip2	chr11	46050880	46051031	-0.34	-1.32
Cyfip2	chr11	46051389	46051569	-0.36	1.26
Ndel1	chr11	68635873	68636023	-0.49	-0.12
Pfn1	chr11	70467911	70468152	-16.50	-0.36
Pafah1b1	chr11	74537356	74537856	-5.43	-0.53
Abr	chr11	76232332	76233353	-20.20	-0.50
Abr	chr11	76307868	76308378	-0.70	0.20
Taok1	chr11	77351295	77353710	-0.74	-0.08
Arhgap27	chr11	103193622	103194512	-1.97	-0.39
Arhgap27	chr11	103201311	103205926	-3.84	-0.86
Arhgdia	chr11	120440371	120440731	-24.80	-0.30
Arhgdia	chr11	120441525	120442361	-18.60	-0.36
Rock2	chr12	16901783	16901994	-0.59	0.42
Rock2	chr12	16984795	16985036	-1.67	0.36
Rock2	chr12	16987074	16992022	-1.04	0.13
Rhoj	chr12	76501275	76502143	-4.25	1.11

Calm1	chr12	101437750	101440606	-6.75	-0.43
Calm1	chr12	101445269	101445389	-1.47	-0.16
Dync1h1	chr12	111839634	111839755	-5.40	-1.41
Dync1h1	chr12	111855994	111856235	-1.10	0.11
Dync1h1	chr12	111856414	111857476	-4.99	-0.25
Dync1h1	chr12	111904592	111905154	-0.31	0.00
Klc1	chr12	112997059	113010574	-0.34	-0.03
Cenpo	chr12	4214270	4214600	-2.23	-1.07
Cenpo	chr12	4234024	4234115	-1.15	1.14
Ywhaq	chr12	21422717	21423076	-6.14	-0.76
Ppp2r5e	chr12	76554380	76554710	-1.63	0.36
Ppp2r5e	chr12	76594449	76616288	-2.54	0.70
Ppp2r5e	chr12	76616467	76694227	-4.57	0.67
Ppp2r5e	chr12	76697006	76697187	-0.99	-0.36
Hist1h3h	chr13	21809496	21809783	-3.88	-0.80
Hist1h3g	chr13	23627286	23627403	-1.47	-0.64
Hist1h3f	chr13	23636303	23636510	-10.20	-0.74
Hist1h2bg	chr13	23663268	23663503	-8.24	-0.53
Hist1h3d	chr13	23666339	23666429	-0.36	-0.31
Hist1h3d	chr13	23667608	23667874	-0.43	-0.08
Hist1h2bc	chr13	23776067	23776242	-3.06	-0.52
Hist1h2bb	chr13	23838602	23838778	-0.32	-0.02
Hist1h2ab	chr13	23842956	23843254	-1.60	-0.36
Hist1h4k	chr13	21842232	21842374	-8.07	-0.57
Hist1h3i	chr13	21875038	21875266	-4.89	-0.55
Hist1h2ae	chr13	23662883	23663060	-3.46	-0.90
Hist1h3c	chr13	23837239	23837390	-0.55	-0.12
Tubb2a	chr13	34166205	34166682	-0.45	0.13
Tubb2a	chr13	34166979	34168134	-1.13	0.36
Tubb2b	chr13	34219319	34219498	-0.64	2.13
Tubb2b	chr13	34220058	34220384	-0.29	0.20
Fgd3	chr13	49358508	49360128	-2.37	-0.20
Fgd3	chr13	49391715	49393309	-1.20	0.15
Arhgef3	chr14	28213521	28215536	-1.21	0.22
Arhgef3	chr14	28215804	28216463	-4.19	0.84
Arhgap22	chr14	34179810	34183122	-1.20	-0.18
Ktn1	chr14	48283451	48283932	-6.66	0.62
Arhgef40	chr14	52604567	52607099	-0.30	-0.16
Arhgef40	chr14	52608387	52609787	-5.27	-0.62
Arhgef40	chr14	52616069	52617444	-1.86	-0.42
Rhobtb2	chr14	70186895	70187286	-0.38	-0.22
Rhobtb2	chr14	70196136	70196976	-2.06	-0.68
Ncf4	chr15	78075240	78075506	-1.95	-0.28

Ncf4	chr15	78092626	78093010	-2.35	-0.20
Tuba1c	chr15	98867757	98868476	-1.06	-0.06
Nckap1l	chr15	103328091	103328301	-4.40	-0.43
Ywhaz	chr15	36723405	36723964	-0.78	0.46
Arhgap39	chr15	76554862	76555521	-0.80	-0.29
Arhgap39	chr15	76557218	76564008	-1.60	-0.67
Arhgap39	chr15	76565202	76582066	-6.14	-0.54
Arhgap39	chr15	76595930	76595991	-0.39	-0.25
Myh9	chr15	77592066	77593452	-18.50	-0.36
Myh9	chr15	77594368	77600009	-100.00	-0.42
Myh9	chr15	77600374	77605023	-64.90	-0.47
Myh9	chr15	77605521	77605702	-4.99	-0.26
Myh9	chr15	77643639	77672545	-7.52	-0.43
Mkl1	chr15	80843459	80848650	-1.19	-0.09
Rangap1	chr15	81540029	81540903	-4.37	-0.88
Rangap1	chr15	81560168	81560349	-2.77	-1.02
Fmnl3	chr15	99147713	99148374	-2.05	0.37
Fmnl3	chr15	99149660	99150028	-0.28	0.02
Racgap1	chr15	99470224	99481962	-4.78	-1.11
Nde1	chr16	14163367	14163457	-0.58	-0.56
Nde1	chr16	14192301	14192986	-0.70	-0.12
Fgd4	chr16	16422549	16422879	-2.94	-0.90
Fgd4	chr16	16475223	16482703	-1.10	-0.55
Fgd4	chr16	16484139	16487285	-1.00	-0.28
Pak2	chr16	32019862	32021879	-1.05	0.10
Arhgap31	chr16	38598455	38598665	-4.26	1.36
Arhgap31	chr16	38599053	38599981	-2.15	0.39
Arhgap31	chr16	38600129	38600309	-1.20	0.69
Arhgap31	chr16	38601325	38606981	-4.51	0.29
Arhgap31	chr16	38712968	38713148	-0.44	0.37
Tiam1	chr16	89789210	89791963	-0.50	0.15
Tiam1	chr16	89884959	89898625	-4.84	0.86
Tagap	chr17	8125970	8127195	-0.51	-0.09
Vav1	chr17	57418522	57418702	-4.22	-1.05
Vav1	chr17	57467135	57467524	-2.08	-0.27
Tagap1	chr17	7159880	7161313	-0.44	-0.05
Pdpk1	chr17	24215673	24215973	-2.16	0.53
Rhot2	chr17	25976110	25976231	-1.78	-0.93
Srf	chr17	46685579	46686796	-2.18	-0.23
Srf	chr17	46690564	46693111	-7.87	-0.45
Klc4	chr17	46781254	46782971	-2.15	-0.68
Ppp2r5d	chr17	46820177	46820772	-0.45	-0.06
Ppp2r5d	chr17	46837059	46841951	-2.20	-0.75

Sgo1	chr17	53818475	53819013	-1.95	0.90
Tubb4a	chr17	57220052	57220528	-2.01	-0.71
Tubb4a	chr17	57220705	57221122	-1.23	-0.53
Tubb4a	chr17	57225671	57225732	-0.31	0.64
Tubb4a	chr17	57226907	57227205	-0.54	0.40
Ralbp1	chr17	66198157	66198667	-4.53	-0.44
Ralbp1	chr17	66198935	66202084	-6.37	-0.43
Ralbp1	chr17	66213630	66234055	-0.32	0.01
Sos1	chr17	80797100	80797999	-1.14	-0.22
Sos1	chr17	80833039	80833489	-2.39	0.56
Sos1	chr17	80879313	80879584	-0.69	-0.93
Calm2	chr17	87835041	87846216	-6.73	-0.44
Ctnna1	chr18	35278565	35312334	-0.52	-0.12
Arhgap26	chr18	39522813	39526590	-0.39	1.25
Arhgap26	chr18	39530727	39531387	-0.31	-0.08
Arhgap26	chr18	39531686	39532136	-0.59	0.52
Tubb6	chr18	67550384	67552330	-1.73	-0.28
Tubb6	chr18	67552598	67561747	-0.60	-0.04
Arhgap12	chr18	6026328	6026687	-0.95	0.49
Arhgap12	chr18	6069981	6135827	-1.43	-0.24
Kif5b	chr18	6241222	6241522	-4.24	-0.53
Rock1	chr18	10181281	10181790	-0.61	-0.10
Arap3	chr18	38132276	38133234	-0.74	-0.07
Arap3	chr18	38156079	38156992	-1.26	-0.14
Men1	chr19	6335038	6336751	-5.84	-0.70
Men1	chr19	6338903	6340834	-1.37	-0.16
Rhod	chr19	4425696	4426625	-0.61	0.24
Klc2	chr19	5117426	5118318	-2.85	-0.86
Incenp	chr19	9947085	9947325	-0.71	0.10
Incenp	chr19	9949354	9951271	-2.94	0.35
Incenp	chr19	9958237	9958489	-1.41	0.21
Incenp	chr19	9961187	9968481	-0.66	0.05
Incenp	chr19	9969946	9973993	-0.42	0.09
Stard8	chrX	96261273	96261604	-0.40	-0.19
Stard8	chrX	96268478	96268689	-0.63	0.40
Cybb	chrX	9015095	9015186	-1.15	-0.12
Flna	chrX	71469158	71470262	-4.86	-0.28
Flna	chrX	71481183	71482098	-8.75	0.37
Dynll2	chr11	87794645	87799923	-3.30	-0.32
Dynll2	chr11	87800789	87801030	-10.50	-1.20
Diaph1	chr18	38004807	38005047	-6.09	-0.67
Diaph1	chr18	38094885	38095065	-1.10	-0.45

Cytokine signaling in immune system

gene_name	chr	chromStart	chromEnd	diff.lg.p	diff.log2.fc
Il1rl1	chr1	40518643	40519212	-1.23	-0.49
Il1rl1	chr1	40521272	40521661	-3.47	1.20
Il18rap	chr1	40605544	40606142	-0.60	-0.21
Stat1	chr1	52216248	52216669	-1.38	-0.43
Creb1	chr1	64622786	64644320	-4.45	0.46
Psmd1	chr1	87961193	87961284	-0.42	-0.17
Eif4e2	chr1	89110548	89110818	-9.20	-1.45
Eif4e2	chr1	89134456	89135715	-0.57	0.08
Tnfrsf11a	chr1	107677299	107704763	-0.32	0.11
Tnfrsf11a	chr1	107723580	107741653	-7.84	-0.45
Rnasel	chr1	155600002	155602307	-2.22	-0.32
Rnasel	chr1	155610184	155610304	-0.49	0.43
Sumo1	chr1	59696425	59697018	-0.64	0.07
Il15ra	chr2	11654960	11655469	-1.10	0.66
Il1rn	chr2	24205037	24205277	-0.51	-0.57
Rapgef1	chr2	29475329	29475390	-0.21	0.04
Rapgef1	chr2	29557850	29558001	-1.22	-0.28
Rapgef1	chr2	29593487	29594506	-3.84	-0.29
Psmc3	chr2	90894439	90895182	-2.65	-0.24
Traf6	chr2	101531587	101537899	-1.26	-0.14
Hck	chr2	152934293	152934473	-1.32	0.63
Hck	chr2	152976907	152977087	-0.28	0.00
Plcg1	chr2	160587492	160588233	-0.75	-0.25
Psmb7	chr2	38498946	38499426	-0.68	-0.14
Il1a	chr2	129126387	129126566	-1.44	1.77
Il1b	chr2	129190494	129190972	-1.68	1.64
Il1b	chr2	129195984	129196856	-1.06	2.80
Il6ra	chr3	89673275	89675305	-3.28	-0.27
Psmb4	chr3	94690701	94690880	-7.90	-0.58
Csf1	chr3	107544324	107544474	-0.76	1.33
Csf1	chr3	107544712	107548597	-0.78	0.36
Csf1	chr3	107548745	107550706	-0.26	0.33
Csf1	chr3	107551063	107553311	-3.91	1.32
Nfkb1	chr3	135248187	135252299	-1.43	-0.19
Nfkb1	chr3	135354240	135354361	-0.22	-0.02
Lyn	chr4	3605267	3605476	-3.18	-0.30
Lyn	chr4	3716941	3718014	-0.33	0.00
Map3k7	chr4	32051081	32051232	-2.12	-1.15
Map3k7	chr4	32104109	32107297	-9.56	0.62
Map3k7	chr4	32110051	32110231	-0.40	-0.13
Csf3r	chr4	125720820	125721208	-1.96	-0.65
Eif4g3	chr4	137676266	137682738	-0.55	0.03

Eif4g3	chr4	137762205	137762386	-1.04	0.29
Ripk2	chr4	16050729	16051591	-1.84	-0.38
Ripk2	chr4	16090555	16090645	-1.23	-0.78
Ifnb1	chr4	88167987	88168373	-0.39	1.77
Ifnb1	chr4	88168549	88168698	-0.30	1.29
Jun	chr4	94716292	94718913	-0.65	-0.03
Jak1	chr4	100825596	100826046	-0.40	0.03
Txlna	chr4	129305539	129308867	-6.13	-0.30
Txlna	chr4	129316556	129317754	-0.63	-0.06
Rps6ka1	chr4	133403324	133404010	-2.82	-0.30
Tnfrsf1b	chr4	144804067	144809643	-18.40	-0.40
Tnfrsf1b	chr4	144818945	144836773	-6.49	-0.95
Tnfrsf1b	chr4	144836594	144836774	-0.89	-0.35
Pik3cd	chr4	149023575	149025880	-1.92	-0.20
Isg15	chr4	155573561	155574927	-8.94	1.48
Il6	chr5	30346074	30346164	-0.27	1.15
Tec	chr5	73147021	73148603	-0.41	-0.05
Tec	chr5	73214871	73259687	-0.86	-0.30
Ubc	chr5	125866572	125866810	-2.56	0.16
Ubc	chr5	125867255	125867493	-1.59	-7.24
Ubc	chr5	125867789	125868176	-3.53	0.25
Ubc	chr5	125868235	125870298	-1.07	0.10
Cul1	chr6	47404345	47445195	-6.27	0.56
Cul1	chr6	47474617	47476108	-1.79	-0.21
Irak2	chr6	113588490	113588551	-0.76	-1.06
Irak2	chr6	113641013	113644480	-4.46	-0.37
Il17ra	chr6	120413214	120413484	-0.46	-0.09
Il17ra	chr6	120431139	120433176	-18.50	-0.43
Nod1	chr6	54893436	54893676	-0.15	0.06
Nod1	chr6	54894600	54894899	-0.32	0.98
Ptpn6	chr6	124683045	124688519	-0.39	-0.09
Irf3	chr7	52253029	52253209	-0.62	-0.25
Irf3	chr7	52256238	52257801	-2.00	-0.33
Il4ra	chr7	132719092	132720561	-0.49	0.10
Il4ra	chr7	132720890	132721340	-2.16	0.79
Relb	chr7	20191570	20192258	-0.84	-0.14
Nfkbib	chr7	29543299	29543360	-0.35	1.74
Nfkbib	chr7	29551273	29551543	-0.53	-0.09
Psmd8	chr7	29965423	29965573	-0.49	-0.05
Flt3l	chr7	52386588	52386858	-0.89	0.61
Il16	chr7	90792260	90794400	-0.50	-0.19
Il16	chr7	90800585	90809673	-5.43	-0.60
Inppl1	chr7	108971355	108974489	-0.68	0.04

Inppl1	chr7	108985423	108986470	-1.44	-0.44
Inppl1	chr7	108986529	108986740	-0.97	0.87
Sh2b1	chr7	133610925	133611520	-4.55	-0.93
Sh2b1	chr7	133615254	133616332	-1.09	-0.16
Sh2b1	chr7	133618136	133618435	-1.67	-0.74
Tollip	chr7	149070058	149075462	-0.90	-0.13
Map2k7	chr8	4245576	4246251	-1.17	-0.17
Map2k7	chr8	4246579	4246700	-0.65	0.26
Tnfsf13b	chr8	10035462	10035522	-0.54	0.62
Ppp2cb	chr8	34710092	34710330	-1.15	-1.30
Dusp4	chr8	35870663	35882259	-9.10	-0.24
Il12rb1	chr8	73343613	73344903	-2.32	-1.22
Ikbkb	chr8	23770516	23770997	-2.23	-0.36
Pik3r2	chr8	73292079	73292558	-0.69	-0.13
Pik3r2	chr8	73296811	73300551	-1.84	-0.26
Psmb10	chr8	108461631	108461997	-1.29	-0.51
Psmd7	chr8	110104338	110108137	-2.12	0.12
Smarca4	chr9	21505619	21507475	-7.96	-0.62
Ppp2r1b	chr9	50665069	50665957	-0.31	-0.06
Ppp2r1b	chr9	50688020	50688710	-0.69	-0.13
Ppp2r1b	chr9	50701016	50701705	-0.88	-0.20
Ppp2r1b	chr9	50702093	50702154	-0.40	-0.32
Psma4	chr9	54805629	54805837	-1.30	0.21
Dusp7	chr9	106270962	106271201	-3.22	-2.66
Dusp7	chr9	106271467	106276865	-1.01	-0.11
Birc2	chr9	7818614	7818943	-0.66	0.25
Birc2	chr9	7833580	7834717	-5.61	0.55
Birc2	chr9	7834955	7835255	-0.35	0.08
Birc3	chr9	7848909	7850958	-0.70	0.12
Birc3	chr9	7860311	7872991	-1.36	0.15
Tyk2	chr9	20933138	20935719	-1.41	-0.74
Il10ra	chr9	45062190	45062579	-1.30	-0.35
Il10ra	chr9	45063175	45068489	-4.42	-0.31
Arih1	chr9	59329494	59334181	-5.23	-0.49
Pik3cb	chr9	98939568	98941447	-1.42	-0.17
Myd88	chr9	119245819	119245939	-1.44	-0.36
Myd88	chr9	119248919	119249158	-1.59	-0.46
Ifngr1	chr10	19329041	19329761	-1.35	0.19
Dusp6	chr10	98725864	98729379	-7.24	0.27
Stat6	chr10	127080041	127080252	-0.60	-0.11
Stat6	chr10	127096737	127097564	-1.23	-0.09
Stat2	chr10	127727970	127728707	-0.65	-0.22
Stat2	chr10	127729455	127729606	-0.29	-0.05

Tab2	chr10	7625983	7626433	-2.18	0.30
Tab2	chr10	7626671	7629889	-2.99	0.23
Tab2	chr10	7631233	7644544	-8.90	0.25
Irak3	chr10	119579548	119580208	-0.76	-0.16
Il23a	chr10	127733431	127733491	-0.39	1.02
Il23a	chr10	127733549	127733934	-0.80	-1.88
Il23a	chr10	127735050	127735110	-0.16	0.10
Osm	chr11	4136817	4140731	-0.59	0.10
Lif	chr11	4157690	4170243	-13.10	1.91
Lif	chr11	4171170	4172188	-3.27	1.38
Peli1	chr11	20991326	20991566	-0.86	-0.32
Peli1	chr11	21047965	21048744	-2.31	-0.31
Fbxw11	chr11	32644536	32645016	-0.47	-0.05
Ppp2ca	chr11	51912325	51912474	-1.41	-0.58
Ppp2ca	chr11	51935449	51935895	-9.86	0.36
Map2k3	chr11	60765498	60766275	-0.72	0.06
Psmd11	chr11	80285574	80285635	-0.96	-0.36
Trim25	chr11	88860716	88861107	-0.59	-0.07
Trim25	chr11	88877620	88878370	-0.53	0.03
Psmd3	chr11	98543867	98546829	-9.56	-0.63
Stat5a	chr11	100721039	100722689	-0.59	-0.40
Psme3	chr11	101177564	101177745	-2.61	-0.42
Psme3	chr11	101183108	101183258	-2.98	-0.41
Map3k3	chr11	106013617	106016028	-0.47	-0.05
Map3k3	chr11	106016147	106016328	-3.97	-1.07
Canx	chr11	50109677	50110776	-16.20	-0.37
Csf2	chr11	54060859	54061185	-0.36	1.25
Map2k4	chr11	65503265	65506940	-1.24	-0.16
Dusp3	chr11	101834543	101835230	-4.42	-0.25
Dusp3	chr11	101835527	101835855	-0.35	0.01
Dusp3	chr11	101835944	101836213	-1.98	-0.25
Map3k14	chr11	103081077	103082217	-1.19	-0.30
Map3k14	chr11	103100286	103100965	-1.14	-0.34
Map3k14	chr11	103103556	103128715	-1.83	-0.94
Socs3	chr11	117827729	117829585	-5.80	-0.22
Sdc1	chr12	8778201	8778442	-1.91	-0.93
Sdc1	chr12	8798575	8799325	-0.73	-0.17
Psma6	chr12	56499811	56499871	-0.49	0.12
Traf3	chr12	112499766	112500664	-1.20	-0.20
Nfkbia	chr12	56593425	56593604	-7.73	-0.63
Rps6ka5	chr12	101789211	101791305	-3.01	-0.71
Hist1h3h	chr13	21809496	21809783	-3.88	-0.80
Hist1h3g	chr13	23627286	23627403	-1.47	-0.64

Hist1h3f	chr13	23636303	23636510	-10.20	-0.74
Hist1h3d	chr13	23666339	23666429	-0.36	-0.31
Hist1h3d	chr13	23667608	23667874	-0.43	-0.08
Syk	chr13	52678805	52706603	-2.12	0.21
Syk	chr13	52728302	52728393	-0.30	-0.01
Syk	chr13	52737330	52738605	-1.17	-0.26
Syk	chr13	52738964	52742902	-0.87	0.06
Il6st	chr13	113254307	113255963	-4.36	-1.18
Il6st	chr13	113294135	113296021	-1.60	0.12
Il6st	chr13	113296588	113296769	-0.30	-0.02
Hist1h3i	chr13	21875038	21875266	-4.89	-0.55
Hist1h3c	chr13	23837239	23837390	-0.55	-0.12
Pik3r1	chr13	102453986	102456264	-2.42	-0.34
Pik3r1	chr13	102478902	102525656	-0.28	0.01
Pik3r1	chr13	102527244	102538172	-0.58	-0.08
Flnb	chr14	8737170	8739780	-7.02	0.37
Flnb	chr14	8782901	8784071	-0.79	-0.08
Psmc6	chr14	45949498	45949587	-0.33	-0.04
Irf9	chr14	56227878	56228328	-1.26	0.44
Camk2g	chr14	21555111	21555918	-2.12	-0.38
Prkcd	chr14	31408748	31411699	-0.65	-0.06
Ptk2b	chr14	66772153	66772990	-3.53	-0.24
Ptk2b	chr14	66899768	66899889	-0.32	0.09
Csf2rb	chr15	78156427	78165685	-0.53	-0.12
Csf2rb	chr15	78177883	78179614	-7.76	-0.47
Tab1	chr15	79988217	79992132	-8.64	-0.65
Atf1	chr15	100084915	100090754	-0.82	-0.12
Ywhaz	chr15	36723405	36723964	-0.78	0.46
Csf2rb2	chr15	78114648	78116419	-0.48	-0.06
Csf2rb2	chr15	78128593	78136033	-4.52	-0.85
Crkl	chr16	17452109	17484522	-0.51	0.04
Eif4g1	chr16	20672821	20673724	-4.60	-0.43
Eif4g1	chr16	20679306	20680055	-11.50	0.22
Eif4g1	chr16	20689383	20692866	-18.90	-0.46
Ifnar2	chr16	91399551	91405652	-1.16	-0.13
Il10rb	chr16	91425363	91425632	-0.37	-0.03
Ifnar1	chr16	91503724	91506421	-4.59	-0.32
Ifnar1	chr16	91506690	91507680	-1.50	0.20
Ifngr2	chr16	91563142	91563622	-0.78	0.08
Socs1	chr16	10783960	10784764	-0.26	-0.21
Socs1	chr16	10784822	10785540	-0.43	-1.26
App	chr16	85025175	85030561	-15.10	-0.48
App	chr16	85120907	85173892	-5.29	-0.55

Brwd1	chr16	96223575	96225491	-0.68	-0.07
Brwd1	chr16	96226300	96226615	-0.30	-1.00
Brwd1	chr16	96229420	96229571	-0.66	0.49
Rps6ka2	chr17	7374463	7374584	-0.23	0.21
Rps6ka2	chr17	7506974	7507424	-0.38	-0.27
Ltb	chr17	35331540	35333220	-14.10	-0.45
Ebi3	chr17	56095753	56096445	-0.64	-0.12
Tnfsf9	chr17	57244807	57244958	-4.13	-0.66
Tnfsf9	chr17	57245227	57247180	-0.42	0.02
Vav1	chr17	57418522	57418702	-4.22	-1.05
Vav1	chr17	57467135	57467524	-2.08	-0.27
Ppm1b	chr17	85393054	85402617	-0.64	0.06
Ppm1b	chr17	85412924	85413283	-6.95	-0.95
Socs5	chr17	87507287	87507348	-0.62	-0.46
Socs5	chr17	87520106	87534893	-2.88	0.23
Tnf	chr17	35336634	35337444	-2.44	-0.10
Tnf	chr17	35338760	35338941	-9.88	-0.47
Ppp2r5d	chr17	46820177	46820772	-0.45	-0.06
Ppp2r5d	chr17	46837059	46841951	-2.20	-0.75
Eif2ak2	chr17	79251889	79252218	-0.53	0.18
Eif2ak2	chr17	79252545	79253888	-2.79	-0.38
Eif2ak2	chr17	79254037	79254276	-0.74	0.37
Sos1	chr17	80797100	80797999	-1.14	-0.22
Sos1	chr17	80833039	80833489	-2.39	0.56
Sos1	chr17	80879313	80879584	-0.69	-0.93
Csf1r	chr18	61265471	61269358	-0.38	0.01
Csf1r	chr18	61290043	61290793	-7.74	-0.14
Map3k8	chr18	4348959	4352951	-0.29	-0.01
Rela	chr19	5637549	5637639	-0.85	-0.86
Rela	chr19	5646998	5647773	-4.34	-0.29
Jak2	chr19	29385493	29386762	-3.33	0.56
Jak2	chr19	29386881	29387540	-5.55	0.96
Nfkb2	chr19	46379316	46380320	-0.99	0.32
Stx3	chr19	11893622	11893893	-0.37	0.13
Chuk	chr19	44181817	44181937	-0.72	-0.36
Csf2ra	chr19	61300453	61302211	-8.18	-0.30
Csf2ra	chr19	61302992	61304321	-7.98	-0.54
Il13ra1	chrX	33695553	33709519	-0.35	-0.03
Ikbkg	chrX	71675045	71678178	-2.09	-1.00
Tab3	chrX	82859447	82860165	-0.50	-0.10
Tab3	chrX	82860462	82860613	-3.04	1.35
Irak1	chrX	71260386	71261911	-3.38	-0.36
Il2rg	chrX	98463484	98463545	-0.75	0.39

DNA repair

gene_name	chr	chromStart	chromEnd	diff.lg.p	diff.log2.fc
Ercc5	chr1	44223653	44224611	-0.81	-0.14
Ercc5	chr1	44237387	44238015	-0.92	-0.20
Cops8	chr1	92508875	92509708	-0.34	-0.01
Ube2t	chr1	136859153	136864304	-1.71	-1.07
Exo1	chr1	177829740	177831466	-0.36	0.08
Exo1	chr1	177838863	177839044	-0.53	1.66
Exo1	chr1	177839133	177839433	-0.23	0.32
Parp1	chr1	182499105	182503813	-0.82	-0.22
Cops5	chr1	10027829	10027979	-0.30	-0.01
Rev1	chr1	38142448	38148963	-0.31	-0.01
Sumo1	chr1	59696425	59697018	-0.64	0.07
Ino80d	chr1	63104207	63107720	-7.78	-0.39
Ino80d	chr1	63125565	63139998	-0.55	-0.05
Dtl	chr1	193363039	193363969	-0.71	-0.34
Dtl	chr1	193364536	193364986	-1.79	-0.56
Dclre1c	chr2	3370124	3371143	-0.30	0.00
Abl1	chr2	31544075	31551320	-0.81	-0.11
Abl1	chr2	31653102	31658129	-5.37	-0.32
Abl1	chr2	31659237	31659417	-0.55	0.86
Rad51	chr2	118960220	118961209	-1.24	-0.47
Ino80	chr2	119200097	119200218	-0.67	0.29
Ino80	chr2	119303244	119303365	-0.60	-0.39
Cops2	chr2	125657538	125660555	-1.89	-0.17
Pcna	chr2	132078675	132078916	-4.27	-0.37
Hist2h2be	chr3	96025043	96025490	-1.90	-0.85
Hist2h2be	chr3	96026738	96027185	-0.90	0.58
Pias3	chr3	96509040	96509100	-1.04	-1.54
Hist2h4	chr3	96067181	96067240	-3.93	-0.68
Dclre1b	chr3	103606745	103607975	-1.01	-0.10
Dclre1b	chr3	103612889	103613280	-0.68	0.14
Nbn	chr4	15906509	15911111	-1.35	0.31
Usp45	chr4	21751701	21751942	-1.33	-0.43
Rad23b	chr4	55398385	55402936	-2.32	0.22
Clspn	chr4	126237164	126239019	-0.48	-0.11
Clspn	chr4	126243253	126243703	-0.40	0.09
Clspn	chr4	126270339	126270788	-0.75	-0.26
Eya3	chr4	132277213	132277961	-1.45	0.21
Rpa2	chr4	132333796	132334035	-1.66	-0.63
Vcp	chr4	43013288	43013349	-0.70	-0.54
Kdm4a	chr4	117809936	117810146	-0.69	-0.25
Kdm4a	chr4	117810503	117811156	-0.33	0.01

Isg15	chr4	155573561	155574927	-8.94	1.48
Uvssa	chr5	33761952	33762373	-0.75	0.34
Rnf4	chr5	34694045	34694196	-3.17	-0.47
Ung	chr5	114588380	114588766	-2.05	-0.80
Baz1b	chr5	135663192	135663582	-4.41	-0.88
Baz1b	chr5	135692615	135694113	-17.30	0.47
Baz1b	chr5	135718352	135720711	-0.78	-0.10
Baz1b	chr5	135720770	135721040	-0.61	0.14
Cops6	chr5	138605188	138605278	-0.50	-0.32
Pms2	chr5	144686171	144686799	-1.86	-0.50
Brca2	chr5	151338110	151338950	-1.33	0.60
Brca2	chr5	151339099	151339550	-0.91	0.41
Brca2	chr5	151341660	151342110	-2.24	0.89
Brca2	chr5	151342799	151343130	-0.82	0.58
Brca2	chr5	151343608	151344119	-4.33	1.39
Brca2	chr5	151344298	151344449	-0.75	0.56
Brca2	chr5	151345677	151347474	-0.73	0.36
Brca2	chr5	151356819	151359500	-1.43	0.86
Brca2	chr5	151371783	151372054	-0.26	0.00
Xrcc2	chr5	25198222	25204121	-0.31	0.04
Rfc1	chr5	65670770	65671010	-0.77	-0.16
Abraxas1	chr5	101235012	101235311	-1.27	-0.52
Ubc	chr5	125866572	125866810	-2.56	0.16
Ubc	chr5	125867255	125867493	-1.59	-7.24
Ubc	chr5	125867789	125868176	-3.53	0.25
Ubc	chr5	125868235	125870298	-1.07	0.10
Ruvbl1	chr6	88415416	88415565	-0.26	-0.01
Ppp4r2	chr6	100815971	100816831	-8.11	-0.44
Rad52	chr6	119865354	119869042	-0.49	0.08
Rad52	chr6	119871288	119872841	-0.83	0.21
Pole4	chr6	82597655	82597805	-2.63	0.88
Pole4	chr6	82602620	82602859	-5.63	-0.84
Ino80b	chr6	83074641	83075023	-0.31	-0.02
Isy1	chr6	87768980	87769634	-1.24	-0.28
Xpc	chr6	91439837	91440076	-0.68	-0.40
Xpc	chr6	91448159	91449978	-2.73	-0.57
Xpc	chr6	91460626	91465822	-0.38	-0.20
Rad18	chr6	112577091	112577691	-5.63	1.03
Mbd4	chr6	115798826	115799695	-0.36	0.12
Mbd4	chr6	115802903	115803084	-1.43	-2.60
Rad51ap1	chr6	126874096	126874974	-0.53	0.26
Ercc1	chr7	19941783	19941873	-8.22	-1.65
Xrcc1	chr7	25352932	25355282	-0.69	-0.30

Xrcc1	chr7	25355570	25356042	-2.57	-0.66
Pnkp	chr7	52118120	52118269	-1.07	-0.81
Herc2	chr7	63485072	63486928	-0.51	-0.04
Zbtb32	chr7	31374699	31375114	-2.76	0.60
Zbtb32	chr7	31376394	31376720	-3.25	-0.67
Fan1	chr7	71491643	71493745	-0.50	-0.83
Fan1	chr7	71517396	71517546	-1.66	2.97
Fan1	chr7	71517665	71517994	-1.37	0.77
Blm	chr7	87647292	87647681	-0.29	-0.01
Blm	chr7	87654407	87658369	-1.75	0.38
Palb2	chr7	129267188	129269139	-0.66	-0.28
Palb2	chr7	129270543	129270992	-1.63	-0.72
Palb2	chr7	129271141	129271919	-0.35	-0.06
Slx1b	chr7	133838939	133839297	-6.25	-1.37
Ppp4c	chr7	133929470	133929707	-2.31	-0.18
Ppp4c	chr7	133935578	133935985	-9.68	-0.48
Ino80e	chr7	134005440	134005501	-0.33	0.49
Polr2c	chr8	97387425	97387604	-0.50	0.11
Usp10	chr8	122460483	122471049	-2.53	-0.17
Usp10	chr8	122480587	122481367	-0.70	-0.09
Smarca5	chr8	83262939	83263358	-1.96	-0.43
Fanca	chr8	125792143	125792234	-0.48	0.50
Fanca	chr8	125792893	125793224	-0.64	0.42
Topbp1	chr9	103252397	103252458	-0.52	0.32
Wdr48	chr9	119833344	119835040	-0.44	-0.03
Atm	chr9	53247503	53248264	-1.23	-0.25
Rnf111	chr9	70274555	70277390	-2.37	-0.34
Rnf111	chr9	70323620	70350792	-3.72	0.24
Atrip	chr9	108960445	108961784	-3.87	-0.23
Atrip	chr9	108967670	108968495	-0.81	-0.18
Rev3l	chr10	39541327	39542617	-3.02	0.41
Rev3l	chr10	39543484	39543605	-0.77	0.53
Rev3l	chr10	39544383	39548363	-0.63	0.10
Dna2	chr10	62436065	62436156	-0.64	-0.63
Timeless	chr10	127669148	127676960	-0.34	0.17
Timeless	chr10	127689519	127689967	-0.64	0.26
Polr2e	chr10	79501286	79502404	-4.55	-0.57
Cdk2	chr10	128135921	128135982	-0.57	0.46
Alkbh5	chr11	60351424	60352684	-1.36	-0.09
Alkbh5	chr11	60367787	60367998	-0.88	-0.13
Trp53	chr11	69393860	69394608	-0.85	-0.23
Trim25	chr11	88860716	88861107	-0.59	-0.07
Trim25	chr11	88877620	88878370	-0.53	0.03

Gps1	chr11	120648738	120649262	-2.03	-0.91
Gps1	chr11	120649939	120650416	-1.77	-0.36
Polm	chr11	5728280	5729483	-0.31	-0.02
Hus1	chr11	8896711	8897375	-0.49	0.23
Rad50	chr11	53463680	53465739	-0.83	-0.20
Top3a	chr11	60553560	60555499	-3.15	-0.90
Top3a	chr11	60555827	60556607	-2.62	-0.75
Rad51d	chr11	82692394	82692735	-0.91	-0.48
Brip1	chr11	85874744	85875522	-0.94	-0.28
Brca1	chr11	101384784	101385144	-0.45	0.17
Brca1	chr11	101385503	101385983	-2.14	0.68
Brca1	chr11	101386071	101386581	-2.01	-0.62
Brca1	chr11	101386909	101387689	-2.04	0.55
Faap100	chr11	120231055	120231355	-0.50	-0.21
Faap100	chr11	120235214	120237593	-0.92	0.19
Faap100	chr11	120237951	120238641	-0.50	-0.11
Nploc4	chr11	120243498	120244646	-0.63	-0.09
Fancm	chr12	66176682	66176953	-0.41	0.31
Fancm	chr12	66206066	66206516	-2.80	-1.29
Fancm	chr12	66207115	66207865	-1.40	-0.47
Fancm	chr12	66222587	66222948	-3.94	1.37
Tdp1	chr12	101129429	101131842	-3.04	0.39
Gen1	chr12	11247880	11249224	-0.97	0.32
Hist1h2bg	chr13	23663268	23663503	-8.24	-0.53
Hist1h2bc	chr13	23776067	23776242	-3.06	-0.52
Hist1h2bb	chr13	23838602	23838778	-0.32	-0.02
Tdp2	chr13	24933063	24933363	-0.76	-0.31
Rmi1	chr13	58503698	58503988	-0.70	-0.16
Rmi1	chr13	58508424	58509724	-0.62	0.19
Rmi1	chr13	58509903	58511252	-1.10	0.19
Hist1h4k	chr13	21842232	21842374	-8.07	-0.57
Msh3	chr13	92981865	92985448	-0.33	-0.03
Polk	chr13	97251062	97251213	-0.45	0.24
Polk	chr13	97251990	97254241	-0.98	0.25
Actr8	chr14	30804041	30804844	-1.12	-0.49
Ercc6	chr14	33326706	33330503	-0.39	0.06
Ercc6	chr14	33339398	33354435	-0.35	-0.02
Ercc6	chr14	33382990	33388053	-0.37	0.03
Ercc6	chr14	33388945	33390433	-0.30	0.00
Xrcc6	chr15	81870214	81870514	-0.44	-0.08
Prkdc	chr16	15839306	15842272	-0.50	-0.14
Rnf168	chr16	32277696	32278296	-0.90	0.26
Rnf168	chr16	32298708	32299428	-0.74	-0.18

Polq	chr16	37048852	37060389	-0.59	-0.52
Polq	chr16	37060507	37061676	-1.27	0.44
Polq	chr16	37062004	37062484	-0.86	0.52
Polq	chr16	37062663	37062993	-0.40	0.22
Usp7	chr16	8738374	8757028	-4.03	-1.80
Hmgn1	chr16	96349053	96349332	-1.64	-0.40
Nthl1	chr17	24769626	24770939	-2.74	-1.15
Rnf8	chr17	29763521	29765948	-1.92	-0.33
Rnf8	chr17	29772903	29778485	-1.50	-0.46
Mdc1	chr17	35983321	35985582	-2.52	-0.26
Mdc1	chr17	35987855	35989938	-1.01	0.15
Mdc1	chr17	35994492	35994553	-0.29	-0.03
Kdm4b	chr17	56540721	56542236	-4.13	-0.58
Ube2i	chr17	25398025	25398806	-0.46	0.06
Polh	chr17	46308941	46309962	-0.99	-0.19
Rbbp8	chr18	11880278	11881165	-3.40	0.45
Polr2d	chr18	31955877	31956355	-4.14	0.77
Poli	chr18	70668512	70669049	-0.90	-0.38
Rad9a	chr19	4195437	4195978	-1.67	-0.43
Kat5	chr19	5603043	5603692	-1.27	-0.18
Kat5	chr19	5609351	5609621	-0.44	0.11
Kat5	chr19	5609680	5610034	-0.76	-0.49
Poll	chr19	45626765	45628002	-5.09	-0.70
Dclre1a	chr19	56618508	56618987	-2.40	-0.62
Dclre1a	chr19	56619255	56621171	-1.48	-0.36
Dclre1a	chr19	56621409	56622516	-1.30	0.41
Cul4b	chrX	35914892	35917100	-9.25	1.45

Regulation of TP53 activity

gene_name	chr	chromStart	chromEnd	diff.lg.p	diff.log2.fc
Ing5	chr1	95714871	95715801	-0.28	0.00
Exo1	chr1	177829740	177831466	-0.36	0.08
Exo1	chr1	177838863	177839044	-0.53	1.66
Exo1	chr1	177839133	177839433	-0.23	0.32
Mdm4	chr1	134888236	134893148	-6.34	0.38
Smyd2	chr1	191704549	191704728	-0.69	-0.12
Phf20	chr2	156093050	156093141	-0.58	0.23
Phf20	chr2	156124429	156134013	-1.03	-0.14
Trp53rkb	chr2	166620945	166621755	-2.25	-0.66
Pip4k2a	chr2	18765497	18769085	-1.88	0.16
Ehmt1	chr2	24646712	24647822	-1.18	-0.21
Ehmt1	chr2	24733063	24775080	-0.36	-0.02
Aurka	chr2	172182198	172182653	-1.29	-0.37
Taf4	chr2	179647358	179656678	-3.26	-0.45

Gatad2b	chr3	90145610	90145850	-0.34	-0.02
Hipk1	chr3	103547210	103547959	-0.55	0.04
Hipk1	chr3	103581206	103595159	-4.73	0.13
Trp53inp1	chr4	11083587	11092680	-0.35	-0.03
Trp53inp1	chr4	11095044	11097090	-1.21	0.33
Nbn	chr4	15906509	15911111	-1.35	0.31
Rpa2	chr4	132333796	132334035	-1.66	-0.63
Mtor	chr4	147822690	147826440	-1.52	-0.59
Mtor	chr4	147930422	147931764	-0.33	0.02
Kmt5a	chr5	124889998	124895602	-0.24	-0.33
Kmt5a	chr5	124910517	124910788	-4.25	-0.39
Prkag2	chr5	24369540	24371912	-1.91	-0.73
Prkag2	chr5	24605830	24606370	-0.43	-0.05
Ubc	chr5	125866572	125866810	-2.56	0.16
Ubc	chr5	125867255	125867493	-1.59	-7.24
Ubc	chr5	125867789	125868176	-3.53	0.25
Ubc	chr5	125868235	125870298	-1.07	0.10
Taf6	chr5	138619844	138620172	-2.00	-0.52
Brpf1	chr6	113257190	113267069	-0.45	-0.02
Brpf1	chr6	113267573	113269263	-6.04	0.44
Brpf1	chr6	113272477	113274549	-3.83	-0.44
Chd4	chr6	125046180	125050907	-20.70	-0.56
Chd4	chr6	125079918	125080429	-2.75	-0.15
Hipk2	chr6	38647898	38666025	-4.07	-0.19
Hipk2	chr6	38767930	38787047	-1.49	-0.06
Hipk2	chr6	38825859	38826040	-5.88	-0.79
Ppp1r13l	chr7	19956956	19958711	-6.19	-1.04
Akt2	chr7	28423129	28423428	-0.34	-0.03
Blm	chr7	87647292	87647681	-0.29	-0.01
Blm	chr7	87654407	87658369	-1.75	0.38
Ppp2cb	chr8	34710092	34710330	-1.15	-1.30
Banp	chr8	124550490	124550731	-0.55	0.73
Banp	chr8	124551359	124551959	-0.40	0.41
Banp	chr8	124552018	124552768	-0.66	0.57
Gatad2a	chr8	72433213	72435695	-8.00	-0.37
Gatad2a	chr8	72498059	72498150	-1.29	-1.13
Gatad2a	chr8	72519948	72520248	-1.68	-0.77
Csnk2a2	chr8	97972031	97972092	-0.28	-0.02
Csnk2a2	chr8	97976954	97977225	-2.32	-0.50
Pin1	chr9	20456573	20459918	-3.27	-0.67
Usp2	chr9	43883786	43883906	-0.22	-0.70
Usp2	chr9	43902091	43902272	-0.86	0.94
Ppp2r1b	chr9	50665069	50665957	-0.31	-0.06

Ppp2r1b	chr9	50688020	50688710	-0.69	-0.13
Ppp2r1b	chr9	50701016	50701705	-0.88	-0.20
Ppp2r1b	chr9	50702093	50702154	-0.40	-0.32
Topbp1	chr9	103252397	103252458	-0.52	0.32
Atm	chr9	53247503	53248264	-1.23	-0.25
Pml	chr9	58067472	58068881	-4.04	-0.65
Pml	chr9	58080996	58094982	-6.71	-1.05
Pml	chr9	58097382	58097563	-1.72	-0.95
Atrip	chr9	108960445	108961784	-3.87	-0.23
Atrip	chr9	108967670	108968495	-0.81	-0.18
Sgk1	chr10	21718603	21718783	-0.46	0.40
Dna2	chr10	62436065	62436156	-0.64	-0.63
Stk11	chr10	79579091	79587616	-0.87	0.11
Stk11	chr10	79592416	79593215	-2.75	0.41
Mbd3	chr10	79855285	79856691	-5.75	-0.31
Mdm2	chr10	117126230	117126381	-3.39	-0.39
Mdm2	chr10	117126829	117128239	-1.59	-0.06
Dyrk2	chr10	118296404	118305870	-1.58	-0.15
Cdk2	chr10	128135921	128135982	-0.57	0.46
Ppp2ca	chr11	51912325	51912474	-1.41	-0.58
Ppp2ca	chr11	51935449	51935895	-9.86	0.36
Aurkb	chr11	68861788	68863970	-2.20	-0.36
Trp53	chr11	69393860	69394608	-0.85	-0.23
Cdk5r1	chr11	80290548	80292661	-29.60	2.17
Cdk5r1	chr11	80293284	80294028	-3.53	1.31
Cdk5r1	chr11	80290547	80292674	-31.50	2.18
Cdk5r1	chr11	80293272	80294202	-4.76	1.41
Taf15	chr11	83286609	83286700	-0.89	-0.29
Hus1	chr11	8896711	8897375	-0.49	0.23
Ccng1	chr11	40563841	40563901	-1.93	0.58
Rad50	chr11	53463680	53465739	-0.83	-0.20
Top3a	chr11	60553560	60555499	-3.15	-0.90
Top3a	chr11	60555827	60556607	-2.62	-0.75
Chd3	chr11	69157463	69157973	-2.59	-0.35
Chd3	chr11	69175016	69175596	-1.19	-0.32
Chd3	chr11	69177569	69182868	-2.61	-0.40
Rffl	chr11	82619321	82631980	-1.42	-0.19
Brip1	chr11	85874744	85875522	-0.94	-0.28
Pip4k2b	chr11	97578204	97583745	-0.53	-0.04
Brca1	chr11	101384784	101385144	-0.45	0.17
Brca1	chr11	101385503	101385983	-2.14	0.68
Brca1	chr11	101386071	101386581	-2.01	-0.62
Brca1	chr11	101386909	101387689	-2.04	0.55

Ppp1r13b	chr12	113066968	113067568	-1.38	0.38
Ppp1r13b	chr12	113070821	113073838	-0.63	0.09
Akt1	chr12	113912217	113912487	-9.20	-1.90
Rmi1	chr13	58503698	58503988	-0.70	-0.16
Rmi1	chr13	58508424	58509724	-0.62	0.19
Rmi1	chr13	58509903	58511252	-1.10	0.19
Taf9	chr13	101435888	101436098	-3.19	0.67
Jmy	chr13	94204723	94211561	-0.37	-0.02
Jmy	chr13	94242733	94269644	-3.41	-0.41
Pip4p1	chr14	51546044	51546434	-0.80	-0.10
Rictor	chr15	6736486	6737266	-2.00	0.21
Rictor	chr15	6745928	6747284	-0.42	-0.04
Taf2	chr15	54847403	54848124	-1.12	-0.24
Taf2	chr15	54903436	54903677	-1.11	-0.38
Zfp385a	chr15	103144892	103145221	-0.95	-0.23
Zfp385a	chr15	103150806	103170487	-1.71	-0.45
Usp7	chr16	8738374	8757028	-4.03	-1.80
Brpf3	chr17	28938160	28938221	-0.11	0.00
Brpf3	chr17	28943116	28944314	-2.09	0.44
Brpf3	chr17	28954078	28960704	-1.40	0.32
Brpf3	chr17	28973668	28973878	-0.63	0.44
Ehmt2	chr17	35040409	35042557	-5.67	-0.61
Ehmt2	chr17	35049712	35050992	-0.56	-0.06
Pdpk1	chr17	24215673	24215973	-2.16	0.53
Rbbp8	chr18	11880278	11881165	-3.40	0.45
Taf4b	chr18	14965941	14971957	-5.68	1.12
Taf7	chr18	37801780	37802286	-6.38	1.76
Taf7	chr18	37802553	37803149	-7.87	1.03
Rad9a	chr19	4195437	4195978	-1.67	-0.43
Kat5	chr19	5603043	5603692	-1.27	-0.18
Kat5	chr19	5609351	5609621	-0.44	0.11
Kat5	chr19	5609680	5610034	-0.76	-0.49
Taf1	chrX	98794675	98794915	-0.51	-0.12

Cell cycle

gene_name	chr	chromStart	chromEnd	diff.lg.p	diff.log2.fc
Sgo2a	chr1	58072495	58072676	-1.00	0.72
Sgo2a	chr1	58073063	58073573	-5.77	1.44
Sgo2a	chr1	58073662	58073723	-0.44	1.14
Sgo2a	chr1	58073991	58074800	-3.42	1.02
Psmd1	chr1	87961193	87961284	-0.42	-0.17
Exo1	chr1	177829740	177831466	-0.36	0.08
Exo1	chr1	177838863	177839044	-0.53	1.66
Exo1	chr1	177839133	177839433	-0.23	0.32

Nsl1	chr1	192906279	192906490	-0.24	0.03
Mcm3	chr1	20793213	20793571	-6.52	-0.56
Sumo1	chr1	59696425	59697018	-0.64	0.07
Mdm4	chr1	134888236	134893148	-6.34	0.38
Nek7	chr1	140383207	140383298	-1.23	-0.44
Blzf1	chr1	166232420	166232660	-5.04	0.74
Ahctf1	chr1	181677367	181685668	-1.17	-0.08
Ahctf1	chr1	181692900	181693051	-0.83	-0.26
Cenpf	chr1	191466321	191466561	-2.25	-0.96
Cenpf	chr1	191472789	191474534	-4.18	-1.21
Cenpf	chr1	191476338	191477059	-6.52	1.04
Cenpf	chr1	191477148	191482268	-0.66	0.07
Cenpf	chr1	191483016	191483856	-1.50	0.35
Ppp2r5a	chr1	193176577	193176817	-0.62	0.09
Ppp2r5a	chr1	193196363	193220920	-7.48	-0.55
Abl1	chr2	31544075	31551320	-0.81	-0.11
Abl1	chr2	31653102	31658129	-5.37	-0.32
Abl1	chr2	31659237	31659417	-0.55	0.86
Golga2	chr2	32158521	32159712	-4.70	-0.68
Golga2	chr2	32162034	32162185	-0.70	-0.26
Nup160	chr2	90575704	90575765	-0.94	0.49
Psmc3	chr2	90894439	90895182	-2.65	-0.24
Kif18a	chr2	109173461	109173732	-1.67	0.79
Mapre1	chr2	153592829	153593040	-0.76	0.14
Cep250	chr2	155805337	155805487	-0.46	0.13
Cep250	chr2	155811065	155814258	-3.34	0.35
Cep250	chr2	155815214	155824606	-2.06	-0.11
Phf20	chr2	156093050	156093141	-0.58	0.23
Phf20	chr2	156124429	156134013	-1.03	-0.14
Src	chr2	157295226	157296441	-0.66	0.35
Ywhab	chr2	163820932	163837497	-5.11	-0.34
Ywhab	chr2	163842532	163842712	-0.32	-0.01
Mcm10	chr2	4911799	4913167	-4.41	-0.81
Mastl	chr2	22988396	22992248	-1.00	0.41
Tubb4b	chr2	25077914	25079487	-0.90	0.06
Tubb4b	chr2	25080103	25080192	-0.50	-0.30
Ssna1	chr2	25126647	25127005	-0.40	-0.06
Ssna1	chr2	25127589	25127938	-2.32	-0.79
Psmb7	chr2	38498946	38499426	-0.68	-0.14
Pcna	chr2	132078675	132078916	-4.27	-0.37
Ninl	chr2	150770920	150778464	-2.03	-0.57
Ninl	chr2	150834983	150835134	-0.43	0.50
Aurka	chr2	172182198	172182653	-1.29	-0.37

Plk4	chr3	40605894	40609819	-0.92	0.14
Hist2h2be	chr3	96025043	96025490	-1.90	-0.85
Hist2h2be	chr3	96026738	96027185	-0.90	0.58
Cenpe	chr3	134902767	134903916	-1.03	-0.49
Cenpe	chr3	134904065	134905504	-1.08	-0.40
Cenpe	chr3	134910323	134910534	-0.64	-0.28
Cenpe	chr3	134936264	134936504	-1.10	0.77
Lmna	chr3	88306893	88307254	-53.80	-0.65
Psmb4	chr3	94690701	94690880	-7.90	-0.58
Hist2h2aa1	chr3	96044042	96044216	-0.53	0.81
Hist2h4	chr3	96067181	96067240	-3.93	-0.68
Lyn	chr4	3605267	3605476	-3.18	-0.30
Lyn	chr4	3716941	3718014	-0.33	0.00
Rab2a	chr4	8462790	8462940	-3.65	-0.71
Nbn	chr4	15906509	15911111	-1.35	0.31
Ndc1	chr4	107083754	107086943	-1.13	0.28
Clspn	chr4	126237164	126239019	-0.48	-0.11
Clspn	chr4	126243253	126243703	-0.40	0.09
Clspn	chr4	126270339	126270788	-0.75	-0.26
Rpa2	chr4	132333796	132334035	-1.66	-0.63
E2f2	chr4	135728338	135728729	-1.17	1.33
E2f2	chr4	135748672	135749243	-1.37	-0.45
Cdkn2c	chr4	109333929	109337859	-0.80	0.26
Cdkn2c	chr4	109338244	109339202	-0.42	-0.07
Kif2c	chr4	116832628	116832838	-0.28	0.06
Sfn	chr4	133156530	133157337	-0.32	-0.09
Sfn	chr4	133157425	133158083	-2.14	1.70
Cdk6	chr5	3519435	3520884	-1.56	-0.25
Akap9	chr5	3960137	3960918	-0.95	0.10
Akap9	chr5	3961217	3961458	-5.08	0.73
Akap9	chr5	3961937	3962418	-2.03	0.32
Akap9	chr5	4045994	4050528	-0.47	0.03
Akap9	chr5	4064169	4065181	-1.31	0.20
Ywhah	chr5	33361494	33361585	-1.08	0.86
Ywhah	chr5	33369269	33370077	-5.50	0.21
Cdc7	chr5	107408383	107412409	-2.49	1.18
Anapc7	chr5	122891358	122891538	-2.21	-0.73
Anapc7	chr5	122893905	122894502	-3.15	-0.59
Kmt5a	chr5	124889998	124895602	-0.24	-0.33
Kmt5a	chr5	124910517	124910788	-4.25	-0.39
Rfc1	chr5	65670770	65671010	-0.77	-0.16
Cenpc1	chr5	86464551	86466811	-0.46	0.05
Cenpc1	chr5	86494488	86494548	-0.32	0.13

Nup54	chr5	92845130	92846543	-2.10	0.37
Abraxas1	chr5	101235012	101235311	-1.27	-0.52
Clip1	chr5	124029078	124029498	-0.51	0.06
Clip1	chr5	124062170	124067553	-0.41	-0.05
Clip1	chr5	124077308	124081141	-0.52	-0.04
Clip1	chr5	124103521	124106024	-3.11	0.25
Ubc	chr5	125866572	125866810	-2.56	0.16
Ubc	chr5	125867255	125867493	-1.59	-7.24
Ubc	chr5	125867789	125868176	-3.53	0.25
Ubc	chr5	125868235	125870298	-1.07	0.10
Pom121	chr5	135853650	135857198	-0.40	0.02
Pom121	chr5	135857525	135857795	-0.86	-0.15
Pom121	chr5	135870057	135870386	-1.39	-0.53
Ywhag	chr5	136385024	136385324	-4.97	-0.24
Ywhag	chr5	136386428	136406621	-22.80	-0.25
Ywhag	chr5	136410271	136410481	-5.25	-0.39
Mad1l1	chr5	140791001	140797506	-0.48	-0.22
Fbxl18	chr5	143633766	143634126	-0.31	-0.04
Fbxl18	chr5	143640471	143644194	-0.67	-0.50
Fbxl18	chr5	143647064	143648530	-3.61	-0.54
Fbxl18	chr5	143648589	143648978	-1.38	-0.65
Fbxl18	chr5	143650443	143656917	-0.75	0.96
Cul1	chr6	47404345	47445195	-6.27	0.56
Cul1	chr6	47474617	47476108	-1.79	-0.21
Ruvbl1	chr6	88415416	88415565	-0.26	-0.01
Foxm1	chr6	128323004	128324260	-3.90	-0.36
Foxm1	chr6	128324947	128325575	-2.57	-0.47
Cdkn1b	chr6	134870988	134871169	-0.43	-0.11
Cdkn1b	chr6	134871258	134871349	-0.54	0.22
Cdkn1b	chr6	134872089	134872300	-0.31	0.05
Pole4	chr6	82597655	82597805	-2.63	0.88
Pole4	chr6	82602620	82602859	-5.63	-0.84
Nup210	chr6	90963060	90963600	-0.61	-0.14
Akt2	chr7	28423129	28423428	-0.34	-0.03
Herc2	chr7	63485072	63486928	-0.51	-0.04
Numa1	chr7	109146700	109157629	-19.70	-0.29
Numa1	chr7	109162783	109163083	-2.12	-0.31
Wee1	chr7	117265572	117267972	-0.29	0.01
Wee1	chr7	117285493	117285884	-3.51	-0.92
Plk1	chr7	129276554	129303004	-0.59	-0.17
Plk1	chr7	129313000	129313091	-0.76	-0.56
Psmd8	chr7	29965423	29965573	-0.49	-0.05
Blm	chr7	87647292	87647681	-0.29	-0.01

Blm	chr7	87654407	87658369	-1.75	0.38
Nup98	chr7	109268003	109269144	-0.31	0.00
Nup98	chr7	109283070	109283401	-1.16	-0.19
Cdc16	chr8	13779263	13781763	-0.31	-0.01
Mcph1	chr8	18631713	18632643	-0.67	0.11
Ppp2cb	chr8	34710092	34710330	-1.15	-1.30
Pcm1	chr8	42378845	42389405	-8.63	0.96
Rab8a	chr8	74704022	74704703	-1.82	-0.16
Rbl2	chr8	93639707	93646756	-4.20	-0.58
Gins3	chr8	98157666	98168453	-0.76	0.16
Gins3	chr8	98168541	98168751	-0.42	-0.19
Cdt1	chr8	125091914	125092005	-0.91	-0.41
Cdt1	chr8	125096551	125097030	-1.96	0.22
Mau2	chr8	72542534	72543372	-1.84	0.25
Csnk2a2	chr8	97972031	97972092	-0.28	-0.02
Csnk2a2	chr8	97976954	97977225	-2.32	-0.50
Dync1li2	chr8	106944120	106944421	-2.85	-0.50
Psmb10	chr8	108461631	108461997	-1.29	-0.51
Terf2	chr8	109593540	109599454	-0.44	-0.05
Terf2	chr8	109600110	109603406	-0.69	-0.11
Psmd7	chr8	110104338	110108137	-2.12	0.12
Zw10	chr9	48886338	48886728	-1.51	0.32
Ppp2r1b	chr9	50665069	50665957	-0.31	-0.06
Ppp2r1b	chr9	50688020	50688710	-0.69	-0.13
Ppp2r1b	chr9	50701016	50701705	-0.88	-0.20
Ppp2r1b	chr9	50702093	50702154	-0.40	-0.32
Psma4	chr9	54805629	54805837	-1.30	0.21
Topbp1	chr9	103252397	103252458	-0.52	0.32
Cep57	chr9	13612830	13613250	-0.38	-0.05
Cep164	chr9	45575868	45578849	-1.46	-0.23
Cep164	chr9	45583989	45587235	-1.17	-0.24
Cep164	chr9	45587605	45587865	-1.05	-0.40
Atm	chr9	53247503	53248264	-1.23	-0.25
Zwilch	chr9	63985369	63985819	-0.67	0.25
Cep63	chr9	102488967	102489207	-0.36	-0.14
Atrip	chr9	108960445	108961784	-3.87	-0.23
Atrip	chr9	108967670	108968495	-0.81	-0.18
Gorasp1	chr9	119834790	119835059	-0.79	0.29
Gorasp1	chr9	119836754	119837112	-3.21	-1.07
Fbxo5	chr10	4544008	4545375	-0.36	-0.08
Fbxo5	chr10	4546760	4547117	-0.82	-0.48
Ranbp2	chr10	57939345	57939706	-5.21	0.45
Ranbp2	chr10	57939885	57940516	-34.30	1.07

Ranbp2	chr10	57940724	57941055	-4.44	0.51
Ranbp2	chr10	57941564	57941925	-3.73	0.28
Ranbp2	chr10	57942194	57945844	-38.80	0.62
Ranbp2	chr10	57955388	57956812	-8.12	0.45
Dna2	chr10	62436065	62436156	-0.64	-0.63
Zwint	chr10	72117642	72118991	-1.35	-0.32
Zwint	chr10	72131564	72131923	-1.77	0.41
Ppp1r12a	chr10	107706405	107714631	-0.52	-0.05
Phlda1	chr10	110943637	110944495	-1.51	0.60
Phlda1	chr10	110944583	110945646	-5.45	1.78
Fzr1	chr10	80829771	80830217	-10.20	0.84
Tmpo	chr10	90611841	90612321	-7.63	0.74
Tmpo	chr10	90625812	90626173	-0.84	0.24
Tmpo	chr10	90633877	90634327	-1.36	-0.22
Mdm2	chr10	117126230	117126381	-3.39	-0.39
Mdm2	chr10	117126829	117128239	-1.59	-0.06
Lemd3	chr10	120362377	120362857	-0.69	0.25
Lemd3	chr10	120389274	120416386	-0.45	-0.04
Cdk2	chr10	128135921	128135982	-0.57	0.46
Xpo1	chr11	23156040	23156131	-0.32	0.11
Fbxw11	chr11	32644536	32645016	-0.47	-0.05
Ppp2ca	chr11	51912325	51912474	-1.41	-0.58
Ppp2ca	chr11	51935449	51935895	-9.86	0.36
Aurkb	chr11	68861788	68863970	-2.20	-0.36
Trp53	chr11	69393860	69394608	-0.85	-0.23
Ywhae	chr11	75546388	75546567	-15.30	-0.67
Psmd11	chr11	80285574	80285635	-0.96	-0.36
Ska2	chr11	86935514	86935724	-1.12	-0.75
Psmd3	chr11	98543867	98546829	-9.56	-0.63
Cdc6	chr11	98782653	98783192	-0.76	-0.32
Tubg1	chr11	100986641	100987723	-0.55	0.11
Psme3	chr11	101177564	101177745	-2.61	-0.42
Psme3	chr11	101183108	101183258	-2.98	-0.41
Sfi1	chr11	3035067	3035397	-9.30	-1.35
Sfi1	chr11	3049499	3050068	-11.50	-0.51
Hus1	chr11	8896711	8897375	-0.49	0.23
Spdl1	chr11	34622746	34623344	-0.29	-0.01
Spdl1	chr11	34639040	34647113	-1.00	-0.36
Rad50	chr11	53463680	53465739	-0.83	-0.20
Top3a	chr11	60553560	60555499	-3.15	-0.90
Top3a	chr11	60555827	60556607	-2.62	-0.75
Ndel1	chr11	68635873	68636023	-0.49	-0.12
Pafah1b1	chr11	74537356	74537856	-5.43	-0.53

Taok1	chr11	77351295	77353710	-0.74	-0.08
Brip1	chr11	85874744	85875522	-0.94	-0.28
Brca1	chr11	101384784	101385144	-0.45	0.17
Brca1	chr11	101385503	101385983	-2.14	0.68
Brca1	chr11	101386071	101386581	-2.01	-0.62
Brca1	chr11	101386909	101387689	-2.04	0.55
Cdc27	chr11	104366205	104368561	-1.10	0.14
Cep131	chr11	119928075	119928468	-0.56	-0.57
Csnk1d	chr11	120819778	120820048	-1.92	-0.33
Csnk1d	chr11	120823749	120824109	-0.92	0.15
Csnk1d	chr11	120824767	120826288	-0.47	0.03
Psma6	chr12	56499811	56499871	-0.49	0.12
Lin52	chr12	85870666	85871471	-0.76	0.29
Dync1h1	chr12	111839634	111839755	-5.40	-1.41
Dync1h1	chr12	111855994	111856235	-1.10	0.11
Dync1h1	chr12	111856414	111857476	-4.99	-0.25
Dync1h1	chr12	111904592	111905154	-0.31	0.00
Cenpo	chr12	4214270	4214600	-2.23	-1.07
Cenpo	chr12	4234024	4234115	-1.15	1.14
Lpin1	chr12	16545018	16545348	-0.51	0.20
Lpin1	chr12	16583836	16584016	-0.99	-0.40
Ywhaq	chr12	21422717	21423076	-6.14	-0.76
Mis18bp1	chr12	66244376	66253740	-1.88	-0.33
Mis18bp1	chr12	66259768	66262788	-2.84	0.76
Ppp2r5e	chr12	76554380	76554710	-1.63	0.36
Ppp2r5e	chr12	76594449	76616288	-2.54	0.70
Ppp2r5e	chr12	76616467	76694227	-4.57	0.67
Ppp2r5e	chr12	76697006	76697187	-0.99	-0.36
Nek9	chr12	86641959	86644517	-0.76	-0.08
Nek9	chr12	86680042	86680312	-4.28	-0.59
Akt1	chr12	113912217	113912487	-9.20	-1.90
Hist1h3h	chr13	21809496	21809783	-3.88	-0.80
Hist1h3g	chr13	23627286	23627403	-1.47	-0.64
Hist1h3f	chr13	23636303	23636510	-10.20	-0.74
Hist1h2bg	chr13	23663268	23663503	-8.24	-0.53
Hist1h3d	chr13	23666339	23666429	-0.36	-0.31
Hist1h3d	chr13	23667608	23667874	-0.43	-0.08
Hist1h2bc	chr13	23776067	23776242	-3.06	-0.52
Hist1h2bb	chr13	23838602	23838778	-0.32	-0.02
Hist1h2ab	chr13	23842956	23843254	-1.60	-0.36
Rmi1	chr13	58503698	58503988	-0.70	-0.16
Rmi1	chr13	58508424	58509724	-0.62	0.19
Rmi1	chr13	58509903	58511252	-1.10	0.19

Tert	chr13	73765570	73766141	-0.39	0.72
Hist1h4k	chr13	21842232	21842374	-8.07	-0.57
Hist1h3i	chr13	21875038	21875266	-4.89	-0.55
Hist1h2ae	chr13	23662883	23663060	-3.46	-0.90
Hist1h3c	chr13	23837239	23837390	-0.55	-0.12
Tubb2a	chr13	34166205	34166682	-0.45	0.13
Tubb2a	chr13	34166979	34168134	-1.13	0.36
Tubb2b	chr13	34219319	34219498	-0.64	2.13
Tubb2b	chr13	34220058	34220384	-0.29	0.20
Nup153	chr13	46776228	46776499	-0.79	0.16
Nup153	chr13	46782010	46792068	-6.70	0.24
Nup153	chr13	46822768	46823218	-6.18	-0.56
Wapl	chr14	35487203	35490366	-1.09	0.12
Wapl	chr14	35490635	35505861	-57.60	0.72
Wapl	chr14	35558893	35558954	-2.34	0.83
Psmc6	chr14	45949498	45949587	-0.33	-0.04
Bora	chr14	99463678	99463829	-0.28	0.81
Bora	chr14	99467341	99467731	-0.31	0.04
Cenpj	chr14	57145717	57148592	-0.47	0.33
Cenpj	chr14	57170817	57171446	-0.66	0.28
Cenpj	chr14	57171803	57172253	-0.54	0.23
Esco2	chr14	66450039	66450160	-2.07	1.45
Ppp2r2a	chr14	67634567	67635464	-3.36	0.22
Gtse1	chr15	85690436	85693039	-3.16	0.36
Tuba1c	chr15	98867757	98868476	-1.06	-0.06
Espl1	chr15	102127163	102129876	-0.61	-0.11
Espl1	chr15	102144496	102146277	-1.33	-0.32
Espl1	chr15	102146396	102147526	-1.75	-0.40
Skp2	chr15	9043359	9043510	-0.96	-0.67
Skp2	chr15	9069207	9070167	-1.36	-0.47
Ywhaz	chr15	36723405	36723964	-0.78	0.46
Csnk1e	chr15	79249210	79251083	-0.79	-0.13
Csnk1e	chr15	79252976	79254661	-1.80	-0.22
Csnk1e	chr15	79272227	79272487	-0.49	-0.55
Rangap1	chr15	81540029	81540903	-4.37	-0.88
Rangap1	chr15	81560168	81560349	-2.77	-1.02
Tubgcp6	chr15	88930334	88930993	-3.03	-0.49
Tubgcp6	chr15	88933494	88934908	-2.28	-0.34
Tubgcp6	chr15	88951070	88953490	-2.04	-0.38
Zfp385a	chr15	103144892	103145221	-0.95	-0.23
Zfp385a	chr15	103150806	103170487	-1.71	-0.45
Nde1	chr16	14163367	14163457	-0.58	-0.56
Nde1	chr16	14192301	14192986	-0.70	-0.12

Rnf168	chr16	32277696	32278296	-0.90	0.26
Rnf168	chr16	32298708	32299428	-0.74	-0.18
Dyrk1a	chr16	94791812	94792112	-3.68	-1.57
Dyrk1a	chr16	94908201	94914884	-0.30	0.00
Fgfr1op	chr17	8388944	8389095	-0.57	0.28
Pkmyt1	chr17	23863332	23869444	-0.84	0.33
Pkmyt1	chr17	23870753	23871230	-0.35	-0.04
Cdkn1a	chr17	29228020	29235664	-1.10	-0.06
Rnf8	chr17	29763521	29765948	-1.92	-0.33
Rnf8	chr17	29772903	29778485	-1.50	-0.46
Fkbpl	chr17	34781827	34783272	-1.29	-0.17
Mdc1	chr17	35983321	35985582	-2.52	-0.26
Mdc1	chr17	35987855	35989938	-1.01	0.15
Mdc1	chr17	35994492	35994553	-0.29	-0.03
Lpin2	chr17	71531989	71532050	-0.27	-1.58
Lpin2	chr17	71596222	71597780	-0.44	0.04
Lemd2	chr17	27327409	27329879	-0.45	-0.07
Lemd2	chr17	27338596	27341383	-2.10	-0.51
Ppp2r5d	chr17	46820177	46820772	-0.45	-0.06
Ppp2r5d	chr17	46837059	46841951	-2.20	-0.75
Sgo1	chr17	53818475	53819013	-1.95	0.90
Pot1b	chr17	55791561	55791801	-1.23	1.27
Tubb4a	chr17	57220052	57220528	-2.01	-0.71
Tubb4a	chr17	57220705	57221122	-1.23	-0.53
Tubb4a	chr17	57225671	57225732	-0.31	0.64
Tubb4a	chr17	57226907	57227205	-0.54	0.40
Rbbp8	chr18	11880278	11881165	-3.40	0.45
Tubb6	chr18	67550384	67552330	-1.73	-0.28
Tubb6	chr18	67552598	67561747	-0.60	-0.04
Cep192	chr18	67978501	67978681	-1.30	0.53
Cep192	chr18	67994178	67994898	-0.63	-0.11
Cep192	chr18	68000667	68002900	-0.73	0.14
Cdc23	chr18	34792961	34793202	-1.19	-0.58
Cdca5	chr19	6086466	6091000	-2.84	-0.59
Jak2	chr19	29385493	29386762	-3.33	0.56
Jak2	chr19	29386881	29387540	-5.55	0.96
Rad9a	chr19	4195437	4195978	-1.67	-0.43
Kat5	chr19	5603043	5603692	-1.27	-0.18
Kat5	chr19	5609351	5609621	-0.44	0.11
Kat5	chr19	5609680	5610034	-0.76	-0.49
Incenp	chr19	9947085	9947325	-0.71	0.10
Incenp	chr19	9949354	9951271	-2.94	0.35
Incenp	chr19	9958237	9958489	-1.41	0.21

Incenp	chr19	9961187	9968481	-0.66	0.05
Incenp	chr19	9969946	9973993	-0.42	0.09
Cep78	chr19	16030262	16030830	-1.51	-0.37
Cep78	chr19	16059120	16059479	-2.20	-1.01
Actr1a	chr19	46452405	46452883	-0.68	-0.07
Emd	chrX	71506376	71506827	-0.97	0.73
Ofd1	chrX	162844099	162844220	-1.28	1.23
Dynll2	chr11	87794645	87799923	-3.30	-0.32
Dynll2	chr11	87800789	87801030	-10.50	-1.20

Post-transcriptional protein modification

gene_name	chr	chromStart	chromEnd	diff.lg.p	diff.log2.fc
Ttll4	chr1	74708446	74719461	-0.36	-0.14
Ttll4	chr1	74719520	74727916	-3.28	0.22
Ttll4	chr1	74744098	74744368	-1.22	-0.41
Psmd1	chr1	87961193	87961284	-0.42	-0.17
Cops8	chr1	92508875	92509708	-0.34	-0.01
Ube2f	chr1	93182063	93182512	-0.31	0.00
Asb1	chr1	93440750	93443711	-3.03	0.99
Asb1	chr1	93451403	93451794	-0.43	0.12
Yod1	chr1	132615345	132616601	-1.98	0.30
Rab7b	chr1	133585271	133593615	-0.53	-0.19
Rab7b	chr1	133608208	133610067	-0.40	-0.02
Rab29	chr1	133763853	133763943	-0.32	-0.31
Rbbp5	chr1	134400352	134400982	-0.53	-0.09
Rbbp5	chr1	134401131	134401252	-1.44	-0.61
Ube2t	chr1	136859153	136864304	-1.71	-1.07
Edem3	chr1	153658799	153666437	-1.62	-0.23
B4galt3	chr1	173206371	173206788	-1.20	-0.29
Pigm	chr1	174306661	174308397	-2.18	0.17
Parp1	chr1	182499105	182503813	-0.82	-0.22
Cops5	chr1	10027829	10027979	-0.30	-0.01
Lman2l	chr1	36480210	36481290	-6.07	-0.59
Chst10	chr1	38920717	38922995	-0.32	0.00
Sumo1	chr1	59696425	59697018	-0.64	0.07
Ino80d	chr1	63104207	63107720	-7.78	-0.39
Ino80d	chr1	63125565	63139998	-0.55	-0.05
Cul3	chr1	80263571	80265851	-13.30	0.70
St8sia4	chr1	97487668	97550155	-1.77	0.16
St8sia4	chr1	97563751	97564171	-2.16	0.28
Ikbke	chr1	133151178	133153534	-0.59	-0.07
Ikbke	chr1	133173083	133173518	-0.44	-0.13
Mdm4	chr1	134888236	134893148	-6.34	0.38
[truncated: 486,225 more chars]
